# Structure/activity virtual screening and in vitro testing of small molecule inhibitors of 8-hydroxy-5-deazaflavin:NADPH oxidoreductase from gut methanogenic bacteria

**DOI:** 10.1038/s41598-020-70042-w

**Published:** 2020-08-04

**Authors:** Massimiliano Cuccioloni, Laura Bonfili, Valentina Cecarini, Filippo Cocchioni, Dezemona Petrelli, Elena Crotti, Raffaella Zanchi, Anna Maria Eleuteri, Mauro Angeletti

**Affiliations:** 1 School of Biosciences and Veterinary Medicine, University of Camerino, 62032 Camerino, MC Italy; 2 Department of Food, Environmental and Nutritional Sciences, University of Milan, 20133 Milan, Italy

**Keywords:** Protein structure predictions, Virtual drug screening, Oxidoreductases, Bioinformatics, Surface plasmon resonance

## Abstract

Virtual screening techniques and in vitro binding/inhibitory assays were used to search within a set of more than 8,000 naturally occurring small ligands for candidate inhibitors of 8-hydroxy-5-deazaflavin:NADPH oxidoreductase (FNO) from *Methanobrevibacter smithii*, the enzyme that catalyses the bidirectional electron transfer between NADP^+^ and F420H_2_ during the intestinal production of CH_4_ from CO_2_. In silico screening using molecular docking classified the ligand-enzyme complexes in the range between − 4.9 and − 10.5 kcal/mol. Molecular flexibility, the number of H-bond acceptors and donors, the extent of hydrophobic interactions, and the exposure to the solvent were the major discriminants in determining the affinity of the ligands for FNO. In vitro studies on a group of these ligands selected from the most populated/representative clusters provided quantitative kinetic, equilibrium, and structural information on ligands’ behaviour, in optimal agreement with the predictive computational results.

## Introduction

*Methanobrevibacter smithii *is a methanogenic archaeon, and it is one of the bacteria responsible for the intestinal production of CH_4_ from CO_2_^1^, the overall pathway involving a number of methanogen enzymes that catalyse specific reactions in the presence of specific coenzymes^1-3^. Among these, 8-hydroxy-5-deazaflavin:NADPH oxidoreductase (FNO) catalyses the electron transfer key-step between NADP^+^ and F420H_2_^4^. High levels of *M. smithii*, and the consequent overproduction of CH_4_ within the intestinal tract, have been reported in individuals suffering from irritable bowel syndrome (IBS)^5^. These subjects can be treated with statins^6,7^, a class of pharmacological inhibitors of 3-hydroxy-3-methylglutaryl-coenzyme A reductase^8^, the rate-regulating enzyme in the synthesis of mevalonic acid^9^, a precursor of cholesterol and other isoprenoids that constitute cell membranes of methanogenic archaea. Irrespective of the general efficacy of statin-based treatments^7,10^, severe-to-lethal side effects are frequently reported ^11^. In this perspective, polyphenols were demonstrated as potential alternative to statin-based treatments^12-14^, but to date only limited studies focused on their ability to directly inhibit FNO and consequently slow down CH_4_ production in the intestinal tract, although FNO modulation by natural compounds could represent a promising novel approach in the treatment of symptoms associated with IBS with reduced adverse events.

In this context, the purpose of this work is the application of virtual screening and in vitro binding/enzyme activity techniques to identify small inhibitors of FNO. Virtual screening identification of candidate drugs is based on the processing of large libraries of chemicals and searching for a subset of compounds able to adequately bind a biological target. Following experimental tests are restricted to those compounds that are predicted to get better binding performances^15^. Such receptor-based virtual screening has to address a number of fundamental issues, including the identification of the possible conformations of flexible molecules, and the prediction/calculation of the absolute binding energies in a given (aqueous) environment^16^. This approach has recently achieved significant successes, with new ligands having been predicted along with their receptor-bound structures with comparable or higher hit rates (in terms of ligands discovered *per* target molecules tested) with respect to empirical high-throughput screening^17^.

The preliminary in silico study involved the homology modelling of the three-dimensional structure of FNO, the selection of a group of 8,012 candidate ligands/effectors out of more than 120,000 nature-inspired compounds contained in the ZINC Biogenic subset (Zbc) based on molecular weight and octanol:water partition (clogP) properties, and the virtual screening via molecular docking of these compounds onto FNO. The global analysis of the results with DataWarrior led to the identification of scaffolds showing different predicted binding affinities to FNO. Next, to support the results of virtual screening, we experimentally characterized the interaction between FNO and 10 representative ligands selected rationally from the most populated/representative clusters using SPR optical biosensors and fluorometric enzymatic activity assays, with results generally in excellent agreement with the predicted binding affinities.

## Results

### Homology model of FNO from *Methanobrevibacter smithii*

**Figure 1:**
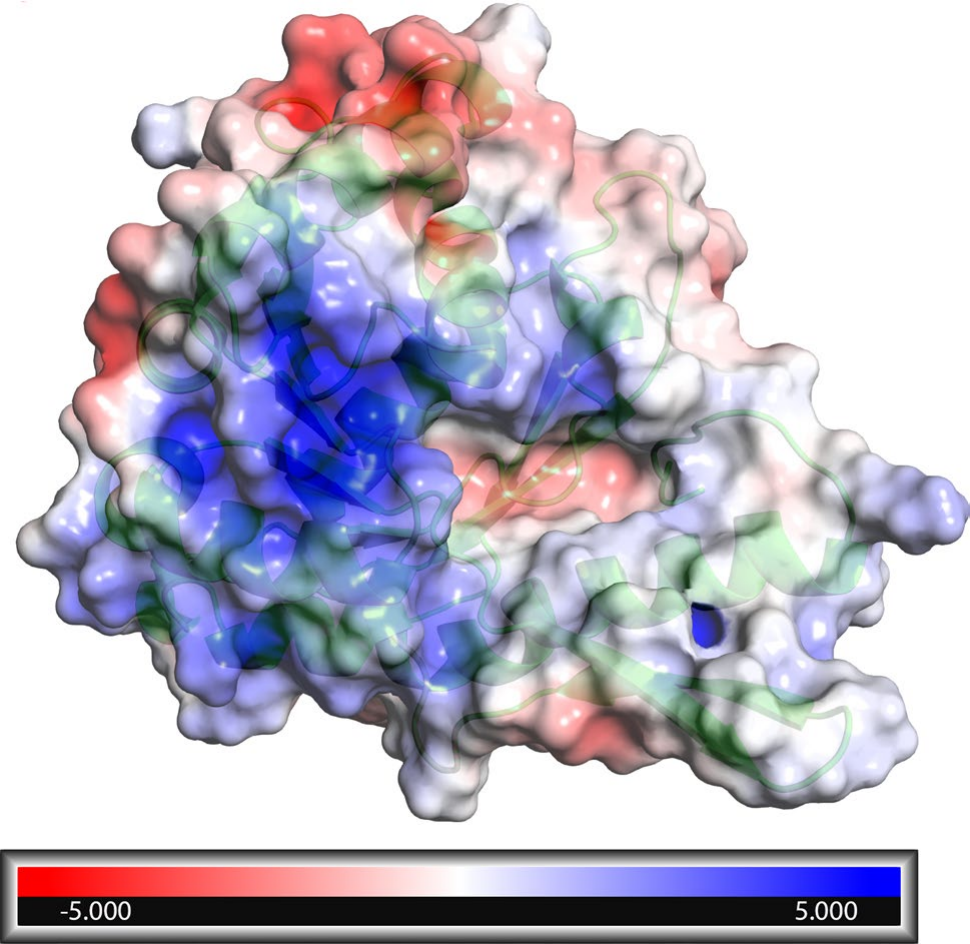
Global electrostatic potential surfaces of the predictive model of FNO calculated with the Adaptive Poisson-Boltzmann Solver Tool—PyMol. Surface was rendered with PyMol 2.3.4.

FNO from *M. smithii* was homology modelled using the 3D structure FNO from *Archaeoglobus fulgidus* as template, as described in the Methods section. The computationally validated predictive model consisted of a major globular core, with 44% helices (41% α-helices, 3% 3(10)-helices), 22% β-sheets content (See Supplementary Information), and extensive polar surfaces. In particular, the exploration of the catalytic site of the reductase revealed a negatively polarized narrow pocket surrounded by positively polarized surfaces, this opposite polarity being among the pivotal factors determining the selectivity for both substrate^18^ and (most likely) site-directed ligands/inhibitors (Fig. 1; the amino acids constituting the catalytic site are shown in Supplemental Information).

### Molecular docking

The 8,012 compounds selected from the Zbc database subset on molecular weight and clogP criteria were individually docked against the homology model of FNO from *M. smithii* using a Perl/Python pipeline on AutoDock Vina. The residues constituting the catalytic site of FNO were retrieved from the available literature^19^ and explicitly defined as the grid centre for all ligands. The quantitative results of docking in terms of ΔG_pred_ of each highest-score pose were collected into a single array (ΔG_pred_ values ranged between − 4.9 and − 10.5 kcal/mol), which was then merged with the other structural descriptors (as PSA, H-donors and acceptors, cLogP, MW, Drug-likeness, Total surface area) available for each compound as summarized in Supplementary Information.

**Figure 2:**
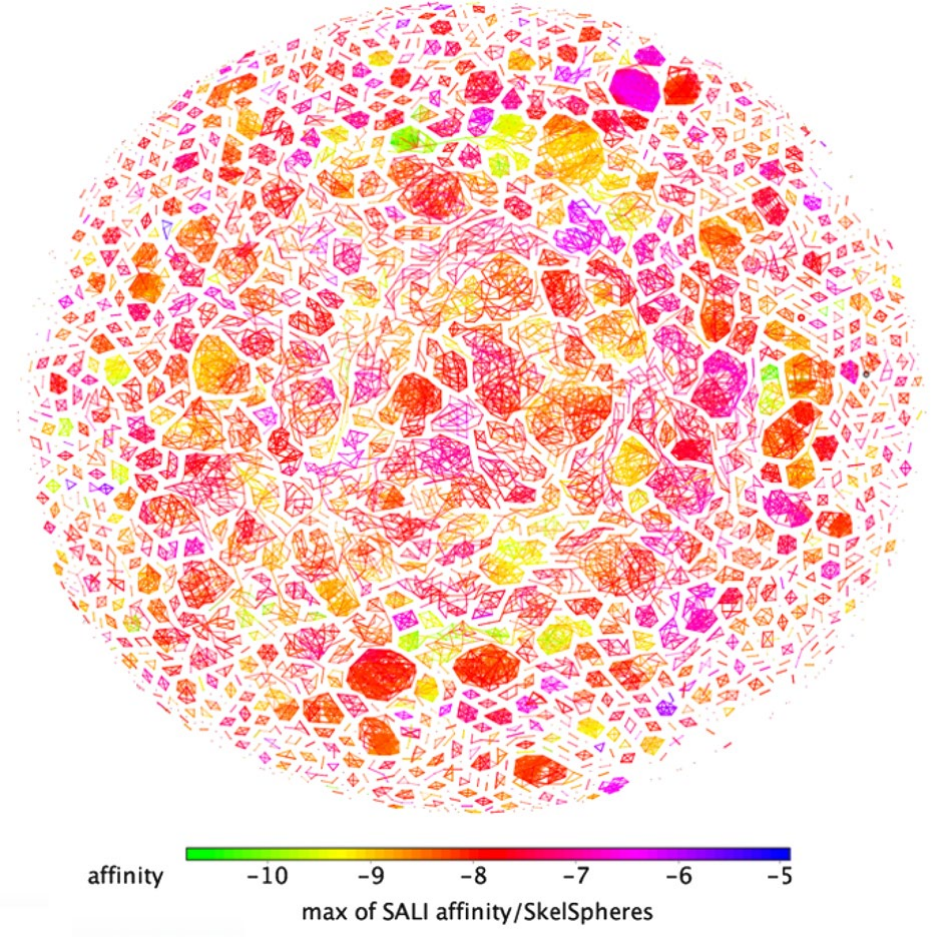
SALI plot clustering of the 8,012 ligands binding to FNO and structural similarity.

The SkelSphere descriptor (a vector of integers representing the occurrence of different substructures in a molecule^20^) was used for the analysis of the dataset, the resulting structure–activity landscape (SALI) heatmap plot^21^ clustering all the 8,012 molecules based on their predicted affinity for FNO and the extent of chemical diversity is shown in Fig. 2.

Resulting clusters can be grouped into three large subsets: blue-to-violet spots, representing clusters of structural analogs with low SALI values (LPA compounds: ΔG_pred_ < -6 kcal/mol; n = 74); pink-to-orange spots, representing clusters of structural analogs with in-between SALI values (MPA compounds: − 9.5 < ΔG_pred_ < − 6.0 kcal/mol; n = 7,614); yellow-to-green spots representing clusters of structural analogs with high SALI values (HPA compounds: − 10.8 < ΔG_pred_ < -9.5 kcal/mol; n = 324). Spot proximity is related to inter-cluster structural similarity.

Ten molecules, each representative of the most populated/representative clusters constituting these three subsets (see Supplemental Information for details), were selected rationally from the most common clustered scaffolds and based on ease-of-retrieval: β-D-glucose pentaacetate (ΔG_pred_ = − 5.9 kcal/mol) for LPA; mangiferin, ononin, 5z-caffeoylquinic acid, ZINC35442308, ZINC100771199, ZINC2120951, ZINC9271779 (ΔG_pred_ = − 8.5, − 9.2, − 8.6, − 8.8, − 7, − 8, − 7.7 kcal/mol, respectively) for MPA; baicalin and frangulin A for HPA (ΔG_pred_ = − 10.0 and − 10.5 kcal/mol, respectively), and successively used for the in vitro validation of the computational results.

**Figure 3:**
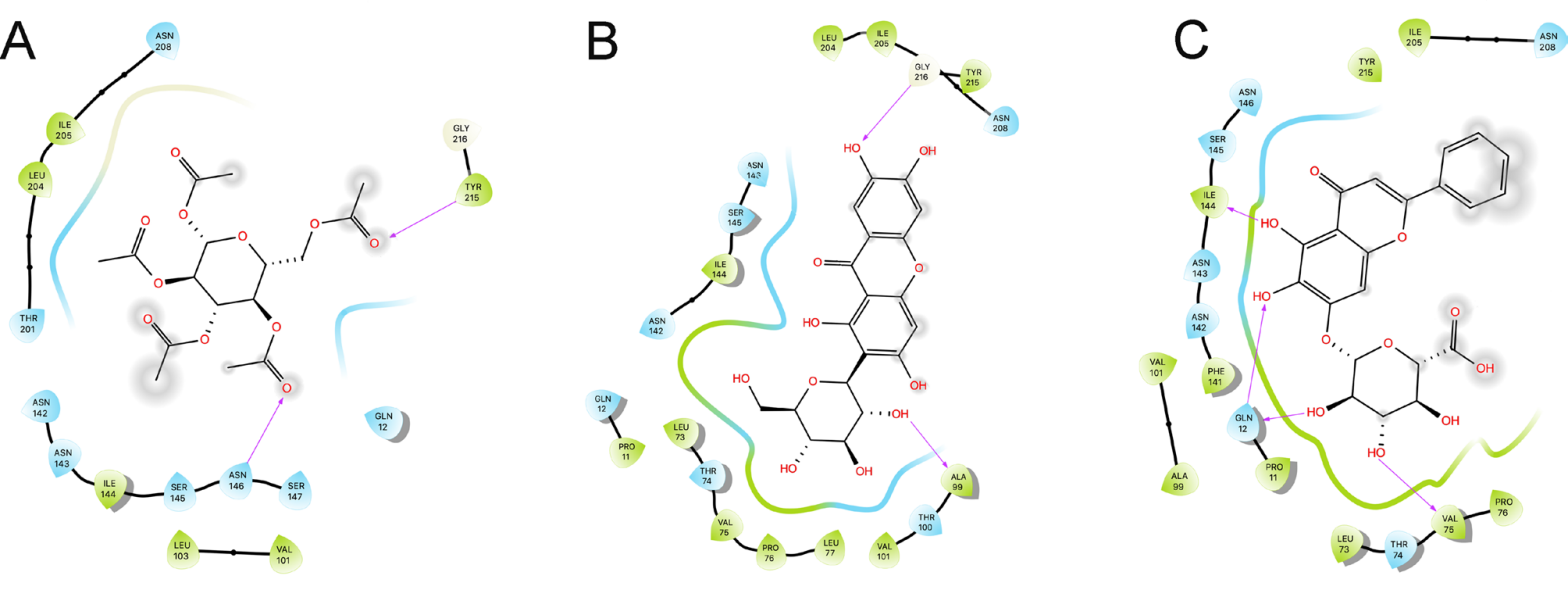
2D visualization of the binding modes of β-D-glucose pentaacetate (inset **A**), mangiferin (inset **B**) and baicalin (inset **C**) to FNO, as representative of LPA, MPA and HPA compounds. Predicted H-bonds are indicated as violet arrows (donor-to-acceptor); polar and hydrophobic interactions, as well as polar and non-polar residues, are indicated in light blue and green ribbons, respectively; functional groups exposed to solvent are highlighted with grey circles.

The complexes formed between these molecules and FNO were generally stabilized by different combination of polar and hydrophobic interactions, and by H-bonds, although in the absence of a linear relationship between individual descriptors and the predicted ΔG_pred_ values (representative binding modes for β-D-glucose pentaacetate, mangiferin and baicalin are shown in Fig. 3; full panel is provided in Supplemental Information).

### SAR analysis

The dependence of the predicted binding affinities for FNO (in terms of ΔG_pred_) from a number of key structural and chemical descriptors conventionally used in the calculation of pharmacokinetic properties of lead compounds, cLogP, molecular weight, polar surface area (PSA), counts of hydrogen bond acceptors and donors, and molecular flexibility (derived from DataWarrior^22^) was evaluated on the whole set of 8,012 molecules.

**Figure 4:**
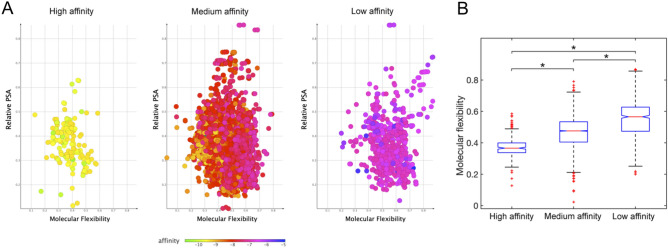
Distribution of relative polar surface area and molecular flexibility for high, medium, and low affinity compounds docked onto FNO (Panel **A**). Summarizing boxplot is reported in panel **B**: red horizontal lines are the median, the bottom and the top of the boxes are the lower and the upper quartiles, and the whiskers are the minimum and maximum values (*P < 0.001).

Complying with the “rule-of-five”^23^, good drug candidates are expected to possess pharmacokinetic parameters with individual scores in the ranges: MW ≤ 500, cLogP ≤ 5, hydrogen bond donors ≤ 5, hydrogen bond acceptors ≤ 10, and Van der Waals bumps polar surface area (PSA) < 120 Å^24^. Here, the molecules showing the highest predicted affinity for FNO were computed to have these descriptors generally scoring within comparable ranges, and specifically: H-donors varying in the interval 1–8 (optimal restricted range: 4–5); H-acceptors varying between 6 and 12 (optimal restricted range: 8–10); PSA varying between 100 and 200 Å; molecular weight between 380 and 450 amu (optimal restricted range: 420–450 amu), and cLogP between − 7.5 and 2. Most interestingly, we recognised the fundamental role of molecular flexibility (Fig. 4), and to lower extent of H-bonding and relative polar surface area in establishing complex stability (see Supplementary Information). In fact, molecular flexibility is a crucial determinant of binding affinity and specificity, with increases in flexibility globally reducing the affinity for FNO.

### Stereoisomerism and binding affinity for FNO

**Figure 5:**
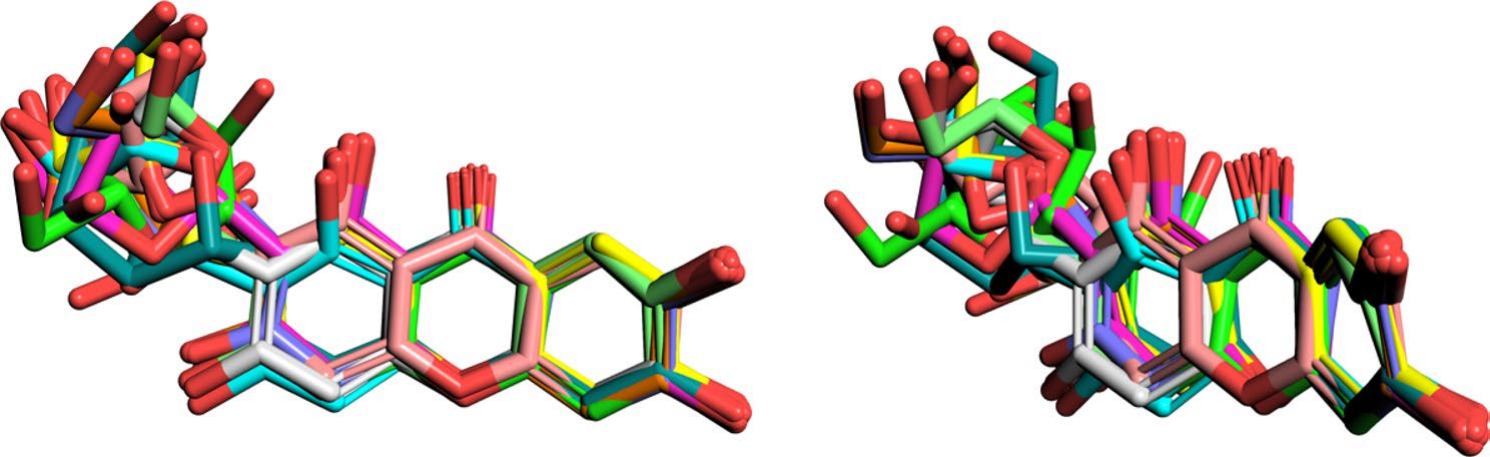
Superimposition of mangiferin stereoisomers (front and side view). Chemical structures were rendered with PyMol 2.3.4.

Focusing on mangiferin, 10 stereoisomers were retrieved in the subset of interest. Consistently with the stereoselective nature of enzymes toward cognate ligands^25^, the SAR studies on mangiferin stereoisomers docked onto FNO provided insight into the role of chiral carbons during ligand accommodation within a stereospecific receptor site. Globally, the configuration of carbon stereocenters had a moderate-to-strong influence on the geometry of the glycosyl group, which in turn partially affected the torsion angle of the xanthone backbone (Fig. 5).

**Figure 6:**
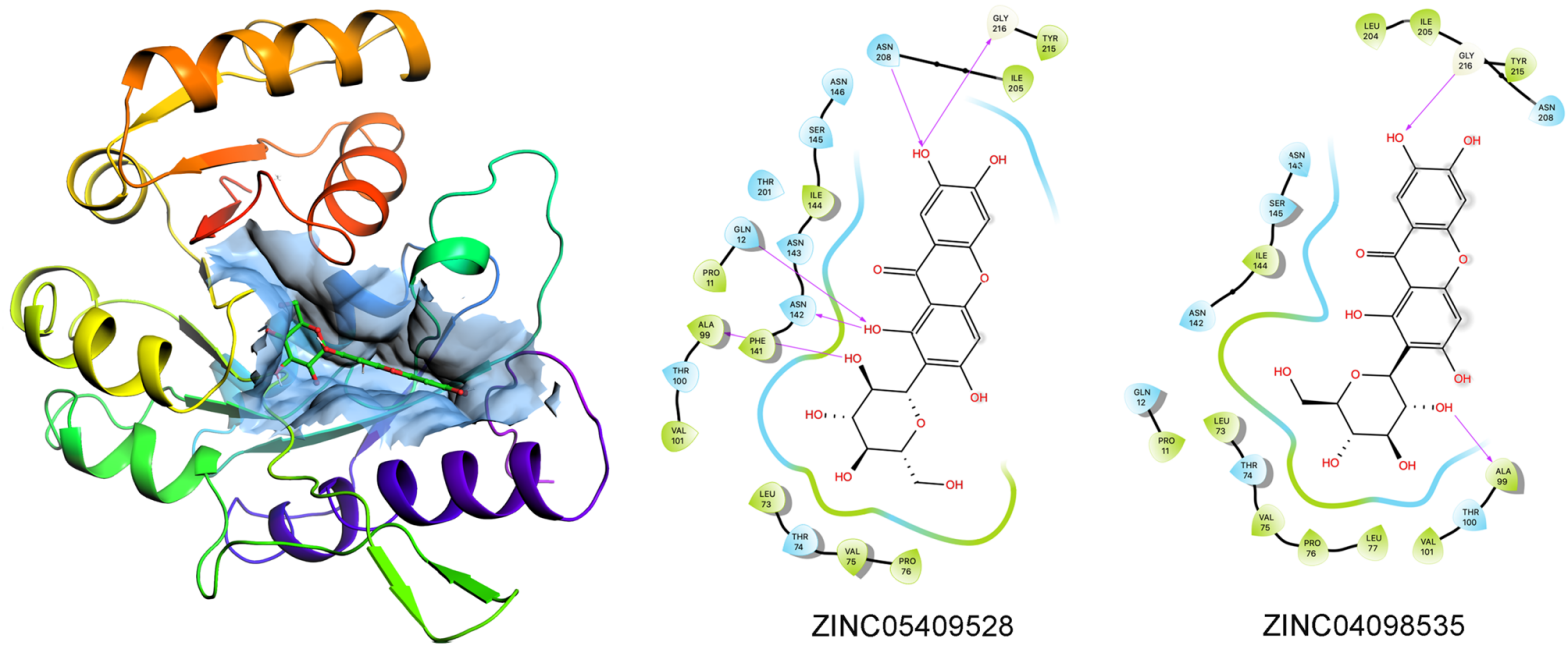
3D representation of predictive complex between FNO and mangiferin stereoisomer ZINC05409528, and comparative 2D visualization of the binding modes of ZINC05409528 (the best scoring stereoisomer of mangiferin) and ZINC04098535 (commercial mangiferin) to FNO. Predicted H-bonds are indicated as violet arrows (donor-to-acceptor); polar and hydrophobic interactions, as well as polar and non-polar residues, are indicated in light blue and green ribbons, respectively; functional groups exposed to solvent are highlighted with grey circles.

Globally, mangiferin stereoisomers were predicted to target FNO in slightly different binding poses but with different affinities in the range 69.6–460 nM. In particular, ZINC04098535 (commercial mangiferin used in the experimental studies), showed nearly sevenfold lower affinity for FNO with respect to ZINC05409528, likely due to an imperfect fitting within the catalytic site that negatively affected the H-bond-stabilizing network (Fig. 6).

**Figure 7:**
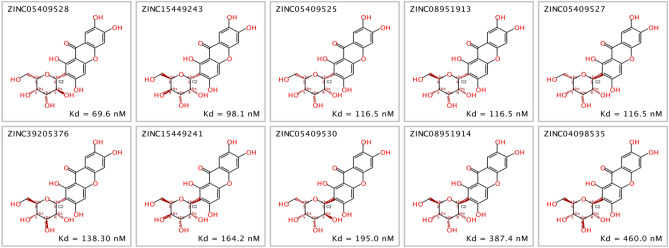
Chemical structures of mangiferin stereoisomers and predicted binding affinities thereof for FNO. The absolute configuration of stereocenters of the glycosyl group is reported.

The structural complementarity for the binding/catalytic site of FNO was the major discriminant in establishing the stability of the complexes, with the configuration of C2 on the glycosyl group likely being a critical factor (specifically, R and S configurations of C2 were predicted to be associated with higher and lower affinity for FNO, respectively—Fig. 7). Moreover, the geometric re-arrangement occurring for stereoisomers ZINC05409525, ZINC08951913 and ZINC05409527 was supposed to be partially “dampened” by the xanthone scaffold, as it resulted in isomers displaying the same binding affinity for FNO.

### Binding studies

The characterization of the interaction between FNO and a representative subset of the molecules screened was performed with a SPR biosensor. Using this analytical approach, we determined the kinetic and equilibrium parameters for the ten different complexes, along with their dependence from pH.

**Figure 8:**
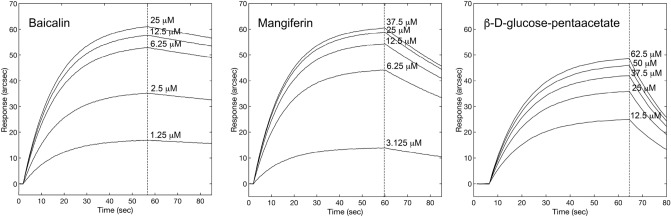
Representative superimposition of sensorgrams obtained at different concentrations of β-D-glucose pentaacetate, mangiferin and baicalin, respectively.

FNO was covalently immobilized on a carboxylate surface according to the protocol described in the Materials and Methods section. The change in the signal related to immobilization of the enzyme (ΔR ≅ 1,000 arcsec) corresponded to the achievement of a partial Langmuir monolayer for a protein of 120 kDa (2 ng/mm^2^). The affinities of FNO for the ten compounds of interest were derived from the fit of raw data (representative sensorgrams are shown in Fig. 8; full panel is provided in Supplemental Information) to a mono-exponential model. Kinetic and equilibrium parameters are summarized in Table 1.

**Table 1:** Kinetic, equilibrium (both experimental (*K*_*D,E*_) and predicted (*K*_*D,P*_)) and inhibition parameters for the complexes formed between FNO and the molecules of interest. Comparison between the experimental equilibrium and predicted constants.

Compound	*k*_*diss*_ (s^−1^)	*k*_*ass*_ (M^−1^ s^−1^)	*K*_*D,E*_ (μM)	*K*_*i *_(μM)	*K*_*D,P *_(μM)
ZINC4262101 (β-D-glucose pentaacetate)	0.013 ± 0.005	2,723 ± 113	4.85 ± 1.85	3.96 ± 0.2	47.40
ZINC04098535 (Mangiferin)	0.002 ± 0.00097	4,825 ± 312	0.40 ± 0.20	0.65 ± 0.25	0.46
ZINC3943903 (Baicalin)	0.00025 ± 0.0001	3,869 ± 578	0.065 ± 0.028	0.045 ± 0.003	0.035
ZINC13544887 (Frangulin A)	0.0045 ± 0.0014	35,232 ± 8,268	0.13 ± 0.05	0.049 ± 0.014	0.015
ZINC1081322 (Ononin)	0.0072 ± 0.0033	29,730 ± 2080	0.24 ± 0.11	0.205 ± 0.069	0.23
ZINC1599733 (5z-caffeoylquinic acid)	0.005 ± 0.003	20,406 ± 2,270	0.23 ± 0.16	0.21 ± 0.14	0.39
ZINC35442308	0.002 ± 0.0009	17,814 ± 5,970	0.13 ± 0.06	1.220 ± 0.202	0.27
ZINC248099510	0.0039 ± 0.0026	2,993 ± 420	1.30 ± 0.89	0.54 ± 0.14	6.04
ZINC2120951	0.004 ± 0.001	2,423 ± 116	1.65 ± 0.41	0.97 ± 0.67	1.08
ZINC9271779	0.0078 ± 0.0008	1,147 ± 267	6.80 ± 1.70	1.07 ± 0.34	1.81

All compounds showed comparable general trend and individual values of binding affinity with respect to predictive studies, with the only exception of β-D-glucose-pentaacetate and frangulin A, which showed nearly tenfold higher and lower binding affinity for FNO, respectively. The exploration of rate parameters helped the identifying two kinetic scenarios. Specifically, β-D-glucose pentaacetate, mangiferin, baicalin, ZINC248099510, ZINC2120951 and ZINC9271779 showed a major contribution of the kinetic stability of the complexes (*k*_*diss*_) to the overall binding affinity, with the recognition phases (*k*_*ass*_) being generally comparable within the experimental error; conversely, the faster recognition process was critical in establishing the affinity of frangulin A, ononin, 5z-caffeoylquinic acid and ZINC35442308 for FNO.

### Competitive binding

**Figure 9:**
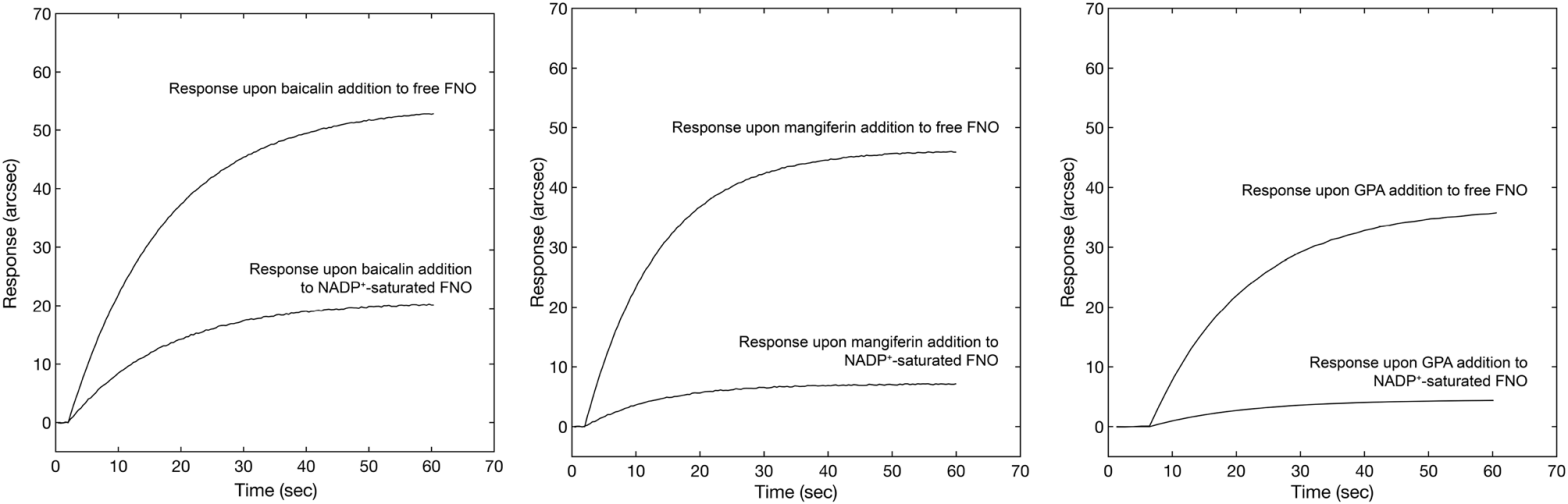
Comparative biosensor responses obtained upon addition of baicalin, mangiferin and GPA to a surface containing free FNO (upper sensor traces) and NADP^+^-saturated FNO (lower sensor traces).

A competitive binding test between NADP^+^ and three out of the ten compounds (with high, moderate and low affinity for FNO, respectively) was performed to identify the binding site on FNO. The protocol involved the addition of a given concentration of baicalin, mangiferin and β-D-glucose pentaacetate to both free and NADP^+^-saturated FNO. The response upon the addition of individual compounds to NADP^+^-saturated FNO was always significantly lower with respect to the signal obtained after addition to free enzyme (Fig. 9) to an extent compatible with individual affinities for FNO (higher affinity was associated with lower decrease in the extent of binding upon pre-saturation), confirming the competitive nature of the compounds of interest with NADP^+^ for the same site, again in excellent agreement with the predictive results of molecular docking.

### Effects of pH and ionic strength on complex affinity

**Figure 10:**
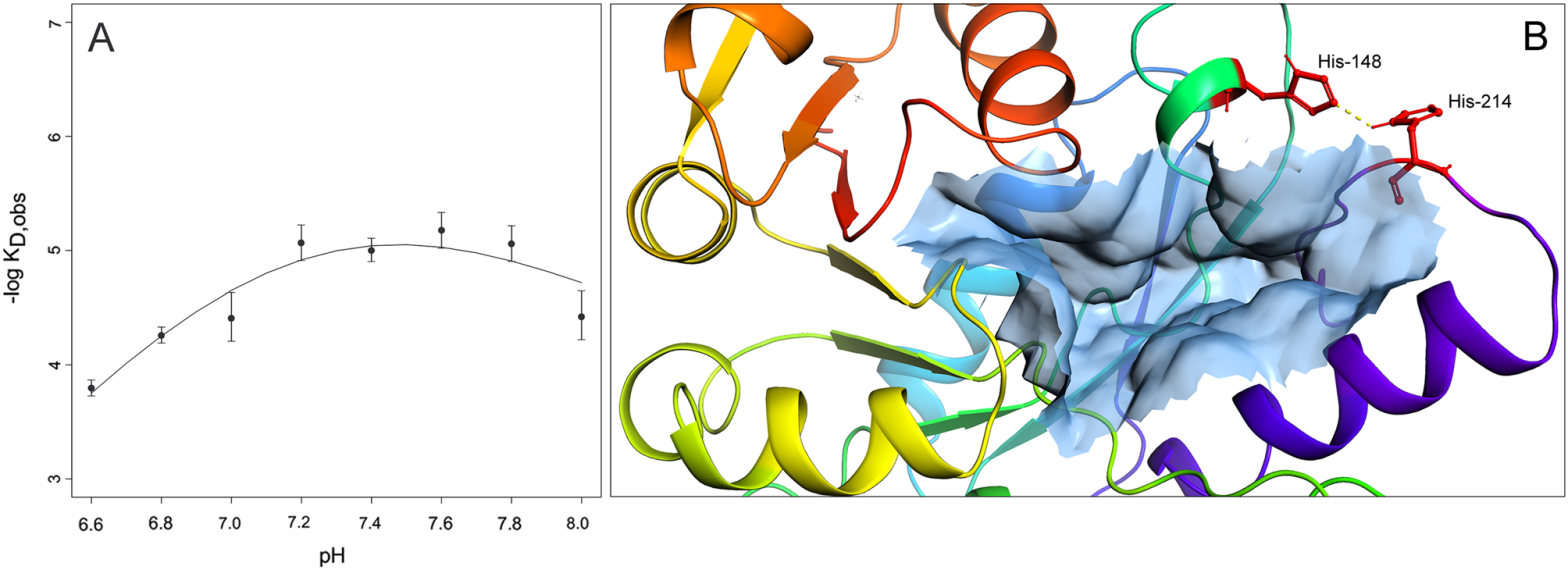
K_D_ vs pH raw data were fit according to Eq. 2 (**A**). Visualization of His-148 and His-214 residues responsible upon protonation for the observed change in binding affinity of FNO for mangiferin; the His-His H-bond is highlighted as solid-dashed yellow line (**B**).

In order to evaluate the effects of pH on the enzyme-ligand interaction, the binding of mangiferin (6.25–62.5 µM) to FNO was studied at different pH values in the range 6.0–8.0. The affinity of mangiferin for FNO (as well as the rate of association) was found to be pH-dependent, with the maximum being observed at pH 7.4–7.6. Importantly, the decreased affinity at basic pH (Fig. 10, Panel A) was exploited to accelerate the surface regeneration phases (in detail, a PBS buffer at pH = 9 was used during baseline recovery).

Furthermore, the results of linkage analysis of *K*_*D*_ dependence on pH suggested the presence of two protonatable groups in the proximity of the binding site. Specifically, the increase in *K*_*D*_ values of FNO-mangiferin complex for pH values ​​between 6.4 and 7.4 was interpreted as a function of the shift in the pK_a_ of a histidine residue from 7.35 ± 0.33 in the free enzyme to 5.26 ± 2.61 in the FNO-mangiferin complex; similarly, the increase in values of *K*_*D*_ for the FNO-mangiferin interaction for pH values between 7.4 and 8.0 was interpreted as a function of the shift in pK_a_ of a histidine residue from 7.35 ± 0.33 in the free enzyme to 8.21 ± 0.12 in the FNO-mangiferin complex. These two histidine residues are likely to correspond to His-148 and His-214 positioned in close proximity to the binding site for NADP^+^ on FNO (Fig. 10, Panel B), which upon inhibitor binding have their ionization pKa values oppositely shifted, resulting in the formation of histidine-histidine H-bond and polar interaction, with the consequent increase in the binding site rigidity^26^.

### In vitro enzyme inhibition

**Figure 11:**
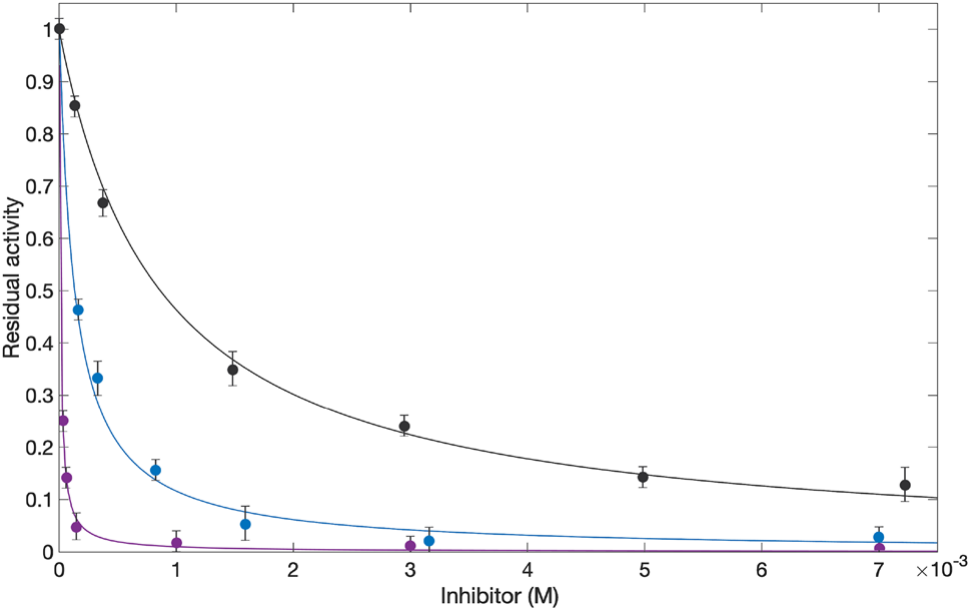
Superimposition of residual activity plots of FNO in the presence of increasing concentrations of β-D-glucose pentaacetate (black curve), mangiferin (blue curve), and baicalin (purple curve).

According to the fluorometric assay of FNO activity, we observed an evident decrease in F420 reduction rates upon 15 min preincubation of FNO with increasing levels of the ten representative compounds of interest (the superimposition of representative residual activity plots obtained with β-D-glucose pentaacetate, mangiferin and baicalin are shown in Fig. 11; the full panel is provided in Supplemental Information).

In detail, at 0.5 mM NADPH, the compounds inhibited (to different extents) the redox activity of FNO from *M. smithii*, the inhibitory potency generally increasing by approximately 100-fold from β-D-glucose pentaacetate to frangulin A (Table 1), with an observed trend consistent with the results of both predictive and binding studies. Only β-D-glucose-pentaacetate and frangulin A showed inhibitory potencies nearly tenfold higher than the experimental binding studies, but in line with the predictive results.

## Discussion

The abnormal production of CH_4_ in the intestine is an established factor in the pathogenesis of severe disorders, including colon cancer, inflammatory bowel disease and irritable bowel syndrome^5^. In the search for efficient anti-methanogenic compounds, we computationally screened Zbc database to identify candidate inhibitors of FNO from *Methanobrevibacter smithii*, the organism responsible for the majority of CH_4_ production in the intestinal tract^27^. Specifically, by integrating in silico and in vitro studies we clustered 8,012 small ligands into specific scaffolds based on their predicted affinity for FNO and structural properties. The results were validated in terms of binding ability and inhibitory properties of ten molecules selected from the most populated/representative clusters as representative of low-, moderate- and high-predicted affinity scaffolds for FNO from *Methanobrevibacter smithii*. In agreement with predictive structural results, these molecules interfered to a different extent with FNO activity by targeting the enzyme at NADP^+^/NADPH site.

Structurally, the stereospecific nature of this cleft strongly discriminated planar/rigid from flexible compounds as an extension of the three-point attachment model^28^ taking into account complex 3D requirements. Rigid species (baicalin, frangulin A and mangiferin) and rigid chiral selectors (the cofactor binding site) better addressed the geometrical conditions for chiral recognition than conformationally flexible structures (β-D-glucose pentaacetate and ZINC9271779). Additionally, the pKa shifts attributable to His-148 and His-214 close to the binding cleft promoted hydrogen bonding between the two residues, this event contributing to further constrain the geometry of the cleft and enhancing the affinity of planar molecules for FNO under neutral physiological conditions, as confirmed by the significant variations in the stability of FNO-mangiferin complex with pH in the range 6.4–8.

Globally, besides confirming the validity of virtual screening techniques, our predictive and experimental results could provide a valuable starting point for the design of stereospecific FNO-inhibitors able to limit the overproduction of intestinal CH_4_ by methanogenic bacteria.

## Methods

### Biologicals and chemicals

*Methanobrevibacter smithii* DSM 861 and *Thermomicrobium roseum* DSM 5159 were purchased from Leibniz-Institut Deutsche Sammlung von Mikroorganismen und Zellkulturen GmbH (Braunschweig, Germany). Tris(hydroxymethyl)amino methane, HCl, NaCl, Na_2_SO_4_, Na_2_HPO_4_, PMSF (phenylmethanesulfonylfluoride), 2-mercaptoethanol, (NH_4_)_2_SO_4_, bovine serum albumin, formic acid, KCl, Tween20, EDC (1-ethyl-3-(3-dimethylaminopropyl)carbodiimide)), NHS (N-hydroxysuccinimide), CH_3_COONa, ethanolamine, DMSO, β-D-glucose pentaacetate, mangiferin, baicalin, NADP^+^, CH_3_COOH and CH_3_OH were purchased from Sigma-Aldrich (Darmstadt, Germany). Frangulin A, ononin, 5z-caffeoylquinic acid were obtained from Extrasynthese (Genay, France). ZINC100771199 and ZINC9271779 were obtained from Interbioscreen (Moscow, Russia). ZINC35442308 and ZINC2120951 were obtained from Vitas-M Laboratory (Bay, Hong Kong). Brilliant Blue R-250 Coomassie were purchased from Bio-Rad (Milan, Italy), acrylamide was obtained from Euroclone S.p.A. (Milan, Italy).

Spectra/Pore dialysis membranes were purchased from Pierce (Milan, Italy). The French Press was obtained from Thermal Electron Corporation (Waltham, MA, USA). Sorvall Discovery M120 SE ultracentrifuge was obtained from Lab Trader (California, USA). Chromatographic separations were carried out on an AKTA Basic HPLC system (GE HealthCare, Milan, Italy) equipped with a fraction collector, using a progel-TSK G2000 SWXL 30 cm × 7.8 mm column (Supelco—Bellefonte, PA, USA). Protein solutions were freeze-dried using a Centrivap centrifugal concentrator (Labconco—Kansas, USA). Spectrophotometric analyses were performed on a Cary 100 Bio UV Visible Spectrophotometer (Varian—Milan, Italy). Binding studies were conducted on IAsys plus optical biosensor (Affinity Sensors—Cambridge, UK), equipped with carboxylate cuvettes (Neosensors—Crewe, UK).

### Homology modelling

In the absence of a crystallographic structure for FNO from *M. smithii*, its three-dimensional structure was obtained by homology modelling, according to a previously reported procedure^29^. Briefly, FNO primary sequence (GenBank entry: ABQ86254.1^30^) was submitted to Swiss Model^31^, using the crystallographic structure of FNO from *Archaeoglobus fulgidus* as template (PDB entry: 1jay^18^), and the output model was refined using Gaia^32^ and eventually validated using PROCHECK^33^. Secondary structures were predicted with Stride Web Tool^34^. Prior to modelling, the signal peptide was predicted using SignalP^35^. and the N-terminus sequence shortened accordingly.

### Molecular docking

8,012 molecules selected from the Zbc subset of ZINC database^36^ on molecular weight (350–450 amu) and hydrophobicity (cLogP < 3.85) criteria were iteratively docked to the homology model of FNO from *M. smithii* using AutoDock Vina^37^. The grid was always configured with the following parameters: size X: 14.63, size Y: 25.50, size Z: 26.63; centre X: 60.70; centre Y: 52.00; centre Z: 49.95; number modes: 3. Structural properties of each molecule and calculated binding affinities to FNO were globally analysed with DataWarrior^38^.

### Binding studies

FNO from *M. smithii* (isolated as described in the Supplementary Information) was covalently coupled to the carboxylate surface of the sensor via EDC/NHS chemistry^39^. Upon successful immobilization of FNO, unreacted carboxyl groups were blocked with ethanolamine 1 M pH 8.5, and the surface was eventually equilibrated with PBS. Independent additions of each molecule at different concentrations (β-D-glucose pentacetate 1.25–62.5 µM; mangiferin 3.125–37.5 µM; baicalin 1.25–25 µM; frangulin A 30–480 nM; ononin 29–465 nM; 5z-caffeoylquinic acid 31.1–497 µM; ZINC35442308 0.30–4.92 µM; ZINC248099510 0.31–4.93 µM; ZINC2120951 0.32–5.1 µM; ZINC9271779 0.32–5.2 µM) to surface-blocked FNO were performed, each time monitoring the association kinetics until equilibrium. The dissociation of complexes was always obtained by a single PBS pH 7.4 wash, whereas surface regeneration was performed with PBS pH 9 washes, as the complex stability was significantly lower at higher pH values. The affinities between surface-blocked FNO and soluble ligands were derived from the fit of raw data to single exponential model.

### Effect of pH on the enzyme-ligand complex stability

To evaluate the effects of pH on the enzyme-ligand interaction, the binding studies between mangiferin and FNO were replicated in a pH range between 6.4 and 8. The observed dependence of experimental *K*_*D*_ from pH was analyzed according to a classic linkage relationship, protons being considered as heterotropic ligands for FNO^40^. This model involves a macromolecule (FNO) having a binding site for a ligand (mangiferin), and heterotropic binding sites able to accommodate a second ligand (H^+^). These binding sites for protons present on the macromolecule can be grouped into two main sets: one with *n* binding sites with an ionization constant λ_1_, and one with *m* binding sites, each having an ionization constant equal to λ_2_. Therefore, the macromolecule can be described in three main ionization conditions: at low and at high pH values the fully protonated and the completely deprotonated forms prevail, respectively, whereas the intermediate form prevails at pH values equal to:

1 $$pH = \frac{{ - log\left( {\lambda_{1} } \right) - log\left( {\lambda_{2} } \right)}}{2}$$

Upon binding, the proton binding sites have their ionization constants shifted to λ_3_ and λ_4_, respectively. The proton heterotropic effect on the macromolecule-ligand complex is thermodynamically linked to the variation of the ionization constants according to the model described in Eq. 2^41^.

2 $$logK_{D,obs} = logK_{D} - log\left( {\left( {\frac{{1 + \lambda_{1} \left[ {H^{ + } } \right]}}{{1 + \lambda_{2} \left[ {H^{ + } } \right]}}} \right)^{n} \times \left( {\frac{{1 + \lambda_{3} \left[ {H^{ + } } \right]}}{{1 + \lambda_{4} \left[ {H^{ + } } \right]}}} \right)^{m} } \right)$$

### Inhibition study

FNO catalyses the reduction of NADP^+^ with F420H_2_ via hydride transfer, and the reverse reaction, i.e. the reduction of F420 with NADPH. Here, the spectrofluorometric assay of FNO activity was based on monitoring the decrease in F420 emission at 470 nm upon excitation at 420 nm. Briefly, FNO (4 nM) was incubated with 0.5 mM F420 (purified from *T. roseum* as described in the Supplementary Information) and 0.5 mM NADPH (this concentration was selected on the basis of *K*_*m*_ values in other methanogenic organisms^42^) at 37 °C in the presence or in the absence of increasing concentrations of the compounds of interest in 50 mM sodium citrate, pH 6.0. The apparent dissociation constant (*K*_*D,app*_) of the preformed FNO-*ligand* complex on the NADPH binding site was determined by measuring the decrease in the catalytic activity upon addition of increasing levels of the ligands (baicalin 0–140 μM; mangiferin 0–3.1 mM; β-D-glucose pentaacetate 0–7.2 mM; frangulin A 0–820 μM; ononin 0–950 μM; 5z-caffeoylquinic acid 0–840 µM; ZINC35442308 0–800 µM; ZINC248099510 0–840 µM; ZINC2120951 0–830 µM; ZINC9271779 0–830 µM). After 15 min preincubation (longer preincubation periods did not further affect FNO activity), residual activities were measured at 470 nm by continuously monitoring the reduction of F420 upon initiation of the reaction. The residual activity ($a$) was expressed as the ratio of the initial velocities of the product formation in the presence ($V_{0,i}$) and in the absence ($V_{0}$) of a given inhibitor concentration [*I*_*i*_]. Raw data were processed as described elsewhere^13^.

### Statistical analysis

The experimental results were expressed as mean values ± standard deviation from (at least) three independent experiments. Statistical analysis was performed with one-way ANOVA, followed by the Tukey multiple comparison test using MATLAB R2019b (MathWorks, Inc., Nattick, MA, USA). *p* values < 0.001 were considered statistically significant.

## Supplementary information

Supplementary information 1

Supplementary information 2
