# Supplementary material for: Structure/activity virtual screening and in vitro testing of small molecule inhibitors of 8-hydroxy-5-deazaflavin:NADPH oxidoreductase from gut methanogenic bacteria
Source: Sci Rep. 2020 Aug 4;10:13150. doi: 10.1038/s41598-020-70042-w (PMC7588429; doi:10.1038/s41598-020-70042-w)
Supplement: Supplementary file 1 — Supplementary information 1 [file 41598_2020_70042_MOESM1_ESM.pdf]

<Supplemental materials>

Structure/activity virtual screening and in vitro testing of small molecule inhibitors of 8-hydroxy-5-deazaflavin:NADPH oxidoreductase from gut methanogenic bacteria

Massimiliano Cuccioloni[1,*], Laura Bonfili[1], Valentina Cecarini[1], Filippo Cocchioni[1], Dezemona Petrelli[1], Elena Sara Crotti[2], Raffaella Zanchi[2], Anna Maria Eleuteri[1], Mauro Angeletti[1].

[1]School of Biosciences and Veterinary Medicine, University of Camerino, 62032, Camerino (MC) - Italy
[2]Department of Food, Environmental and Nutritional Sciences, University of Milan, 20133, Milan - Italy.

*CORRESPONDING AUTHOR
Massimiliano Cuccioloni
School of Biosciences and Veterinary Medicine, University of Camerino
Via Gentile III da Varano – 62032 – Camerino (MC) Italy
Email: massimiliano.cuccioloni@unicam.it

Validation of the 3D model of FNO from *Methanobrevibacter smithii*

The predictive model of FNO was thoroughly validated using ERRAT, PROCHECK, MOLPROBITY and Ramachandran plot scores. A minimal portion of the protein, which was not involved in ligand recognition (residues 49-52), presented structural inconsistencies.

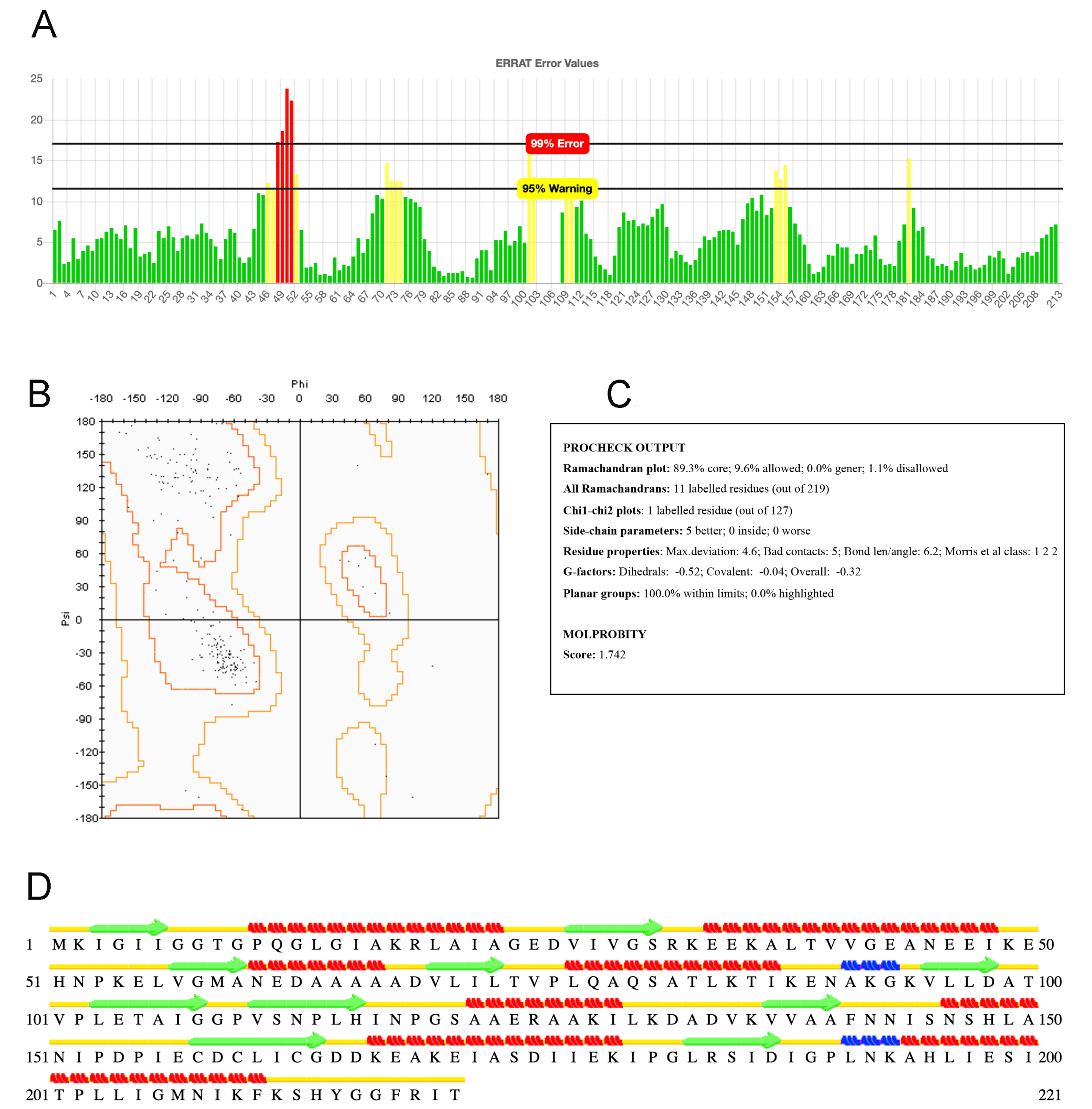

Figure S1. Validation of homology modelled FNO using ERRAT (Panel A) and PROCHECK (Panel C). Ramachandran plot (Panel B). Prediction of secondary structures with Stride Web Tools (Panel D).

Purification of FNO from *Methanobrevibacter smithii*

FNO was purified according to the method described by Berk *et al.*[1] with minor modifications. Briefly, *M. smithii* cells were grown under anaerobic conditions at 80% H_2 and 20% CO_2, then in 65 mM isopropanol. Confluent cells were harvested by centrifugation at 5000 × g for 30 min and stored at -80 °C until use. 0.25 g of bacterial cells were suspended in 10 mL lysis buffer (50 mM Tris HCl, pH 8.0, 300 mM NaCl and 2-mercaptoethanol 20 mM, containing 1 mM PMSF, 1 mM TPCK, and 1 mM aprotinin), and was subjected to three sequential French press cycles at 140 MPa. Cell lysate was ultracentrifuged at 30000 × g for 30 min at 5 °C, and the supernatant was precipitated sequentially with 65% and 75% $(NH_4)_2SO_4$. The ultracentrifugation steps were repeated under the same conditions, always discarding the pellet. Resulting supernatant was further precipitated with 90% of $(NH_4)_2SO_4$, and the *pellet* was collected upon ultracentrifugation at 22000 × g for 30 min at 5 °C. The pellet was resuspended in 0.1 M sodium sulphate and 0.1 M sodium dihydrogen phosphate, pH 6.8 and separated by size-exclusion chromatography with isocratic elution, using a progel-TSK G2000 SWXL column, (mobile phase: 0.1 M sodium sulphate and 0.1 M sodium dihydrogen phosphate, pH 6.8; flow rate: 0.7 mL/min; wavelengths: 218 and 280 nm). The corresponding fractions obtained from different chromatographic runs were pooled, extensively dialyzed, and eventually freeze-dried.

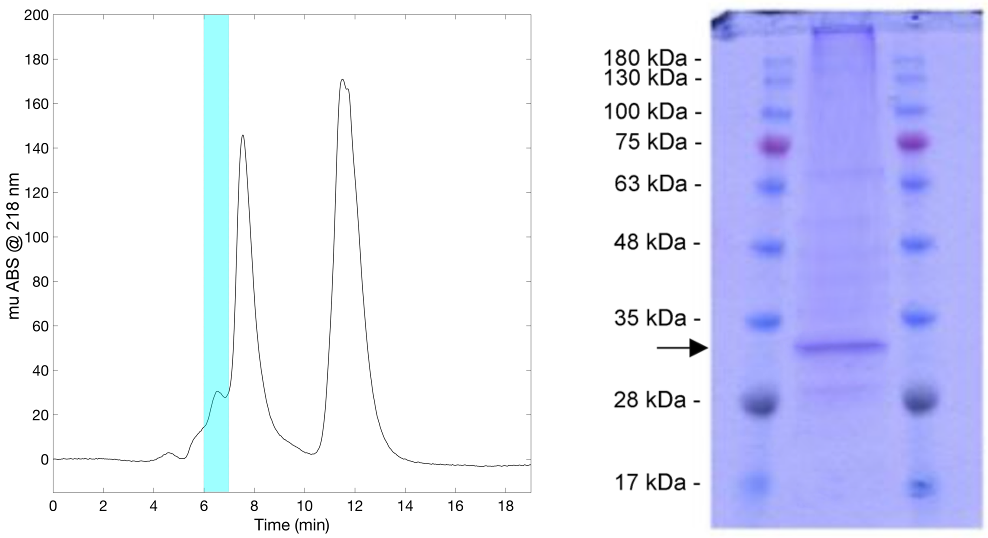

Figure S2. Purification of FNO. Size-exclusion chromatography profile of proteins obtained after ammonium sulfate precipitation (the peak corresponding to FNO is highlighted in light blue – Panel A). Polyacrylamide gel electrophoresis (PAGE) electrophoresis of the corresponding fraction (Panel B).

FNO isolation was assessed by the presence of a chromatographic peak corresponding to a 120 kDa protein, and the simultaneous presence of an electrophoretic band (SDS-PAGE) corresponding to a 30 kDa monomer, confirmed the presence of FNO (Figure S2).

Purification of F420

F420 was purified essentially as described by Ney *et al.*[2] with minor modifications. *Thermomicrobium roseum* cells were resuspended in 0.5 mL of DSM 592 medium, incubated for 30 min at RT, then inoculated in DSM 592 agar poured in a short slant with a long butt in a screw-capped tube. The culture was incubated at 72 °C for 5 days. 3 isolated colonies were inoculated in 10 mL of DSM 592 medium and incubated at 72 °C for a few days. This culture was used as inoculum to scale up the volume of the culture up to 1 L. Cells from the saturated culture were harvested by centrifugation (10000 × g, 20 min, 4 °C) and the supernatant was discarded. Pellets were resuspended in 1-to-3 mL 50 mM sodium phosphate buffer (pH 7.0) and lysed by boiling.

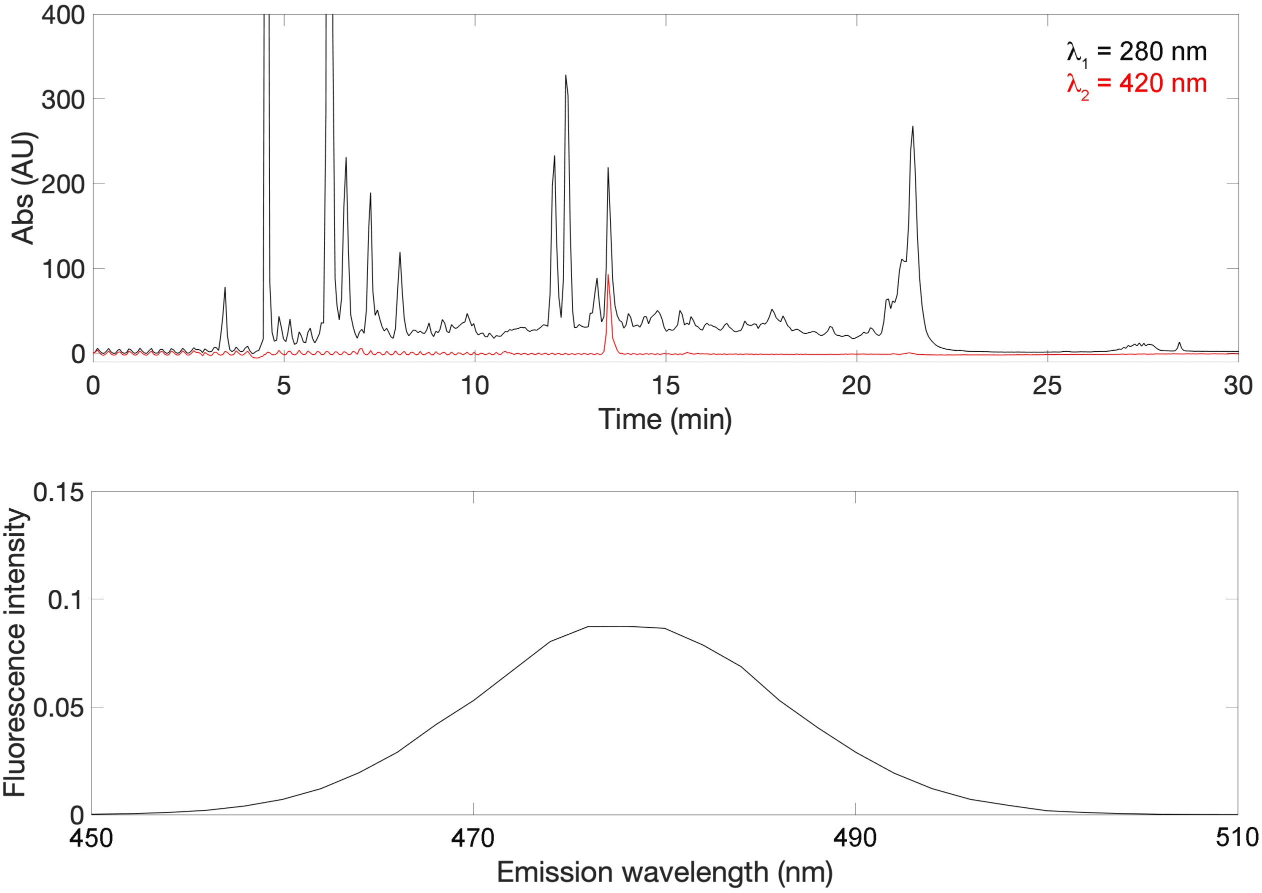

Figure S3. RP-chromatography isolation of F420 from *Thermomicrobium roseum* cell lysate (upper inset), and fluorescence emission spectrum of the peak eluted at 13.5 min upon excitation (lower inset).

Cell lysates were centrifuged at 11000 × g for 10 min, and the supernatant was collected for further purification. F420 was isolated by reversed-phase high performance liquid chromatography (HPLC) using an AKTA Basic HPLC system (Amersham Biosciences, Uppsala, Sweden) equipped with a

Luna C18 column, 5 µm particle size, 250 × 4.6 mm (Phenomenex, Bologna - Italy). Elution gradient was 0-1 min 25% B; 1-10 min from 25% to 35% B; 10-13 min 35% B; 13-16 min from 35% to 40% B; 16-19 min from 40% to 25% B. Mobile phase composition: (A) 20 mM ammonium phosphate, 10 mM tetrabutylammonium phosphate, pH 7.0, and (B) 100% acetonitrile. For fluorescence detection, isolated fractions were excited at 420 nm and emission spectra were recorded between 450 and 500 nm (Fig. S3).

Catalytic site of FNO

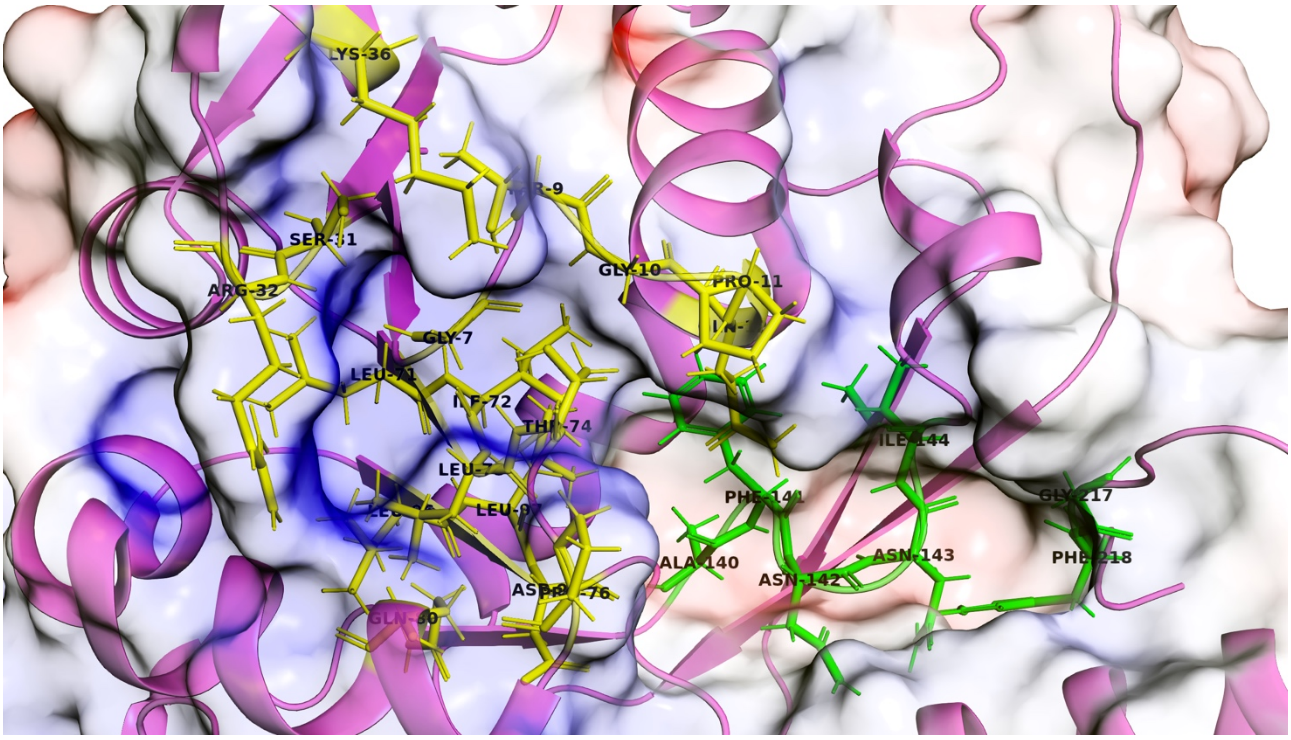

Figure S4. Close-up visualization of the catalytic site of homology modelled FNO from *M. smithii*; amino acids constituting the catalytic site are shown as solid sticks. Residues involved in the binding with the molecules screened in this study are highlighted in green.

Descriptors and affinity

H-bonding (donor and acceptor) and relative polar surface area had a minor, still significant role in establishing complex stability (Fig. S5). In particular, the higher number of H-bonds was directly associated to higher affinity of ligands for FNO.

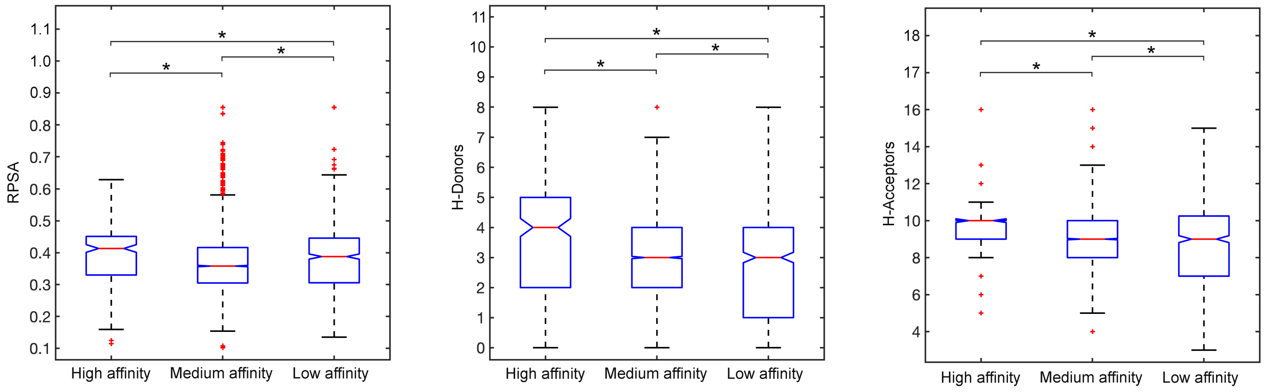

Figure S5. Boxplots summarizing the influence Relative polar surface area (RPSA), H-donors and H-acceptor descriptors on complexes affinity. Red horizontal lines are the median, the bottom and the top of the boxes are the lower and the upper quartiles, and the whiskers are the minimum and maximum values (* $P < 0.001$).

Table S1. Descriptors summary obtained from DataWarrior. (**Table S1 is provided as a separate PDF file**)

Selection of representative molecules for *in vitro* assays

10 representative ligands were selected rationally from the most populated/representative clusters constituting the ZINC Biogenic Compounds database (n_{zbc}= 8012).

First, clusters derived from SALI analysis were grouped into three major subsets constituted by:

1- clusters of structural analogs with low SALI values (ΔG_{pred}< -6.0 kcal/mol; n_1=74);

2- clusters of structural analogs with moderate SALI values (-9.5 <ΔG_{pred}< -6.0 kcal/mol; n_2= 7614), which was further divided as:

2a- clusters with moderately-low SALI values (-8.0 <ΔG_{pred}< -6.0 kcal/mol; n_{2a}= 4385)

2b- clusters with moderately-high SALI values (-9.5 <ΔG_{pred}< -8.0 kcal/mol; n_{2b}= 3229)

3- clusters of structural analogs with high SALI values (HPA compounds: -10.8 <ΔG_{pred} < -9.5 kcal/mol; n_3=324).

One compound was selected from a highly populated/representative cluster out of subset #1 (low SALI values), also based on ease-of-retrieval (Fig. S6, Panel A).

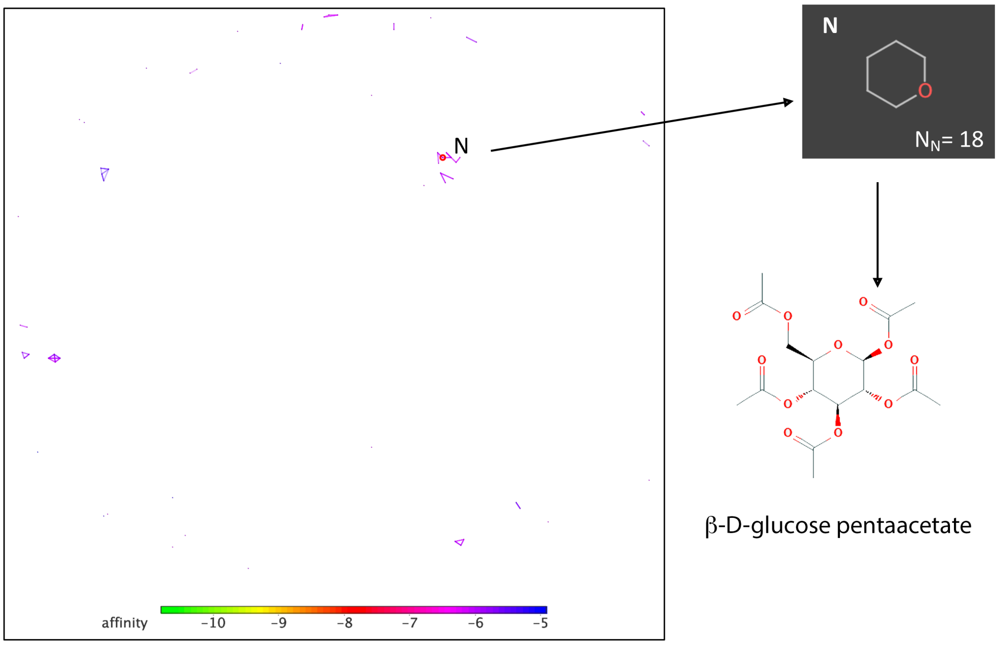

ΔG > -6 Kcal/mol N=74

Figure S6, Panel A. SALI plot clustering of the 74 LPA ligands binding to FNO and structural similarity. The compound and parent scaffold thereof selected for following experimental studies are shown.

Three compounds were selected from as many highly populated/representative clusters out of subset #2a (moderately-low SALI values), also based on ease-of-retrieval (Fig. S6, Panel B).

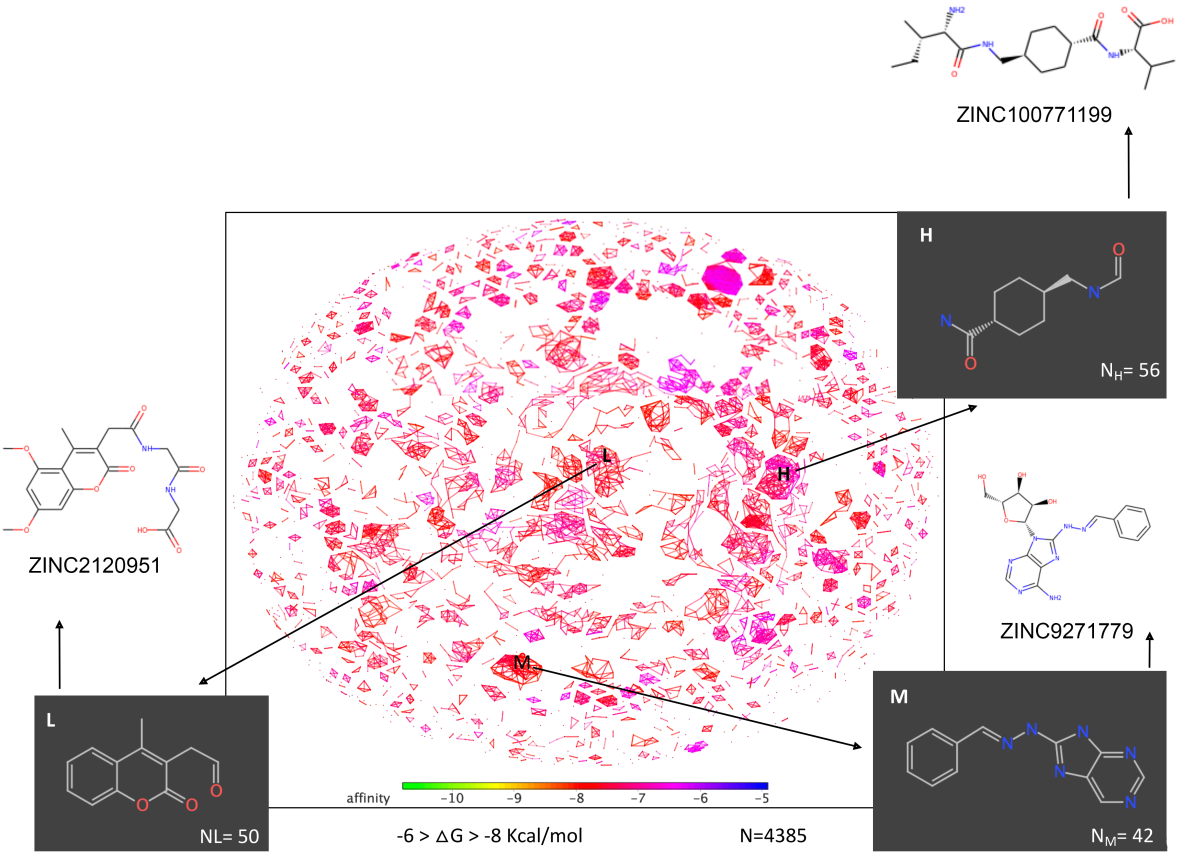

Figure S6, Panel B. SALI plot clustering of the 4385 MPA1 (moderately low scores) ligands binding to FNO and structural similarity. The compounds and parent scaffolds thereof selected for following experimental studies are shown.

Four compounds were selected the from as many highly populated/representative clusters out of subset #2b, also based on ease-of-retrieval (Fig. S6, Panel C).

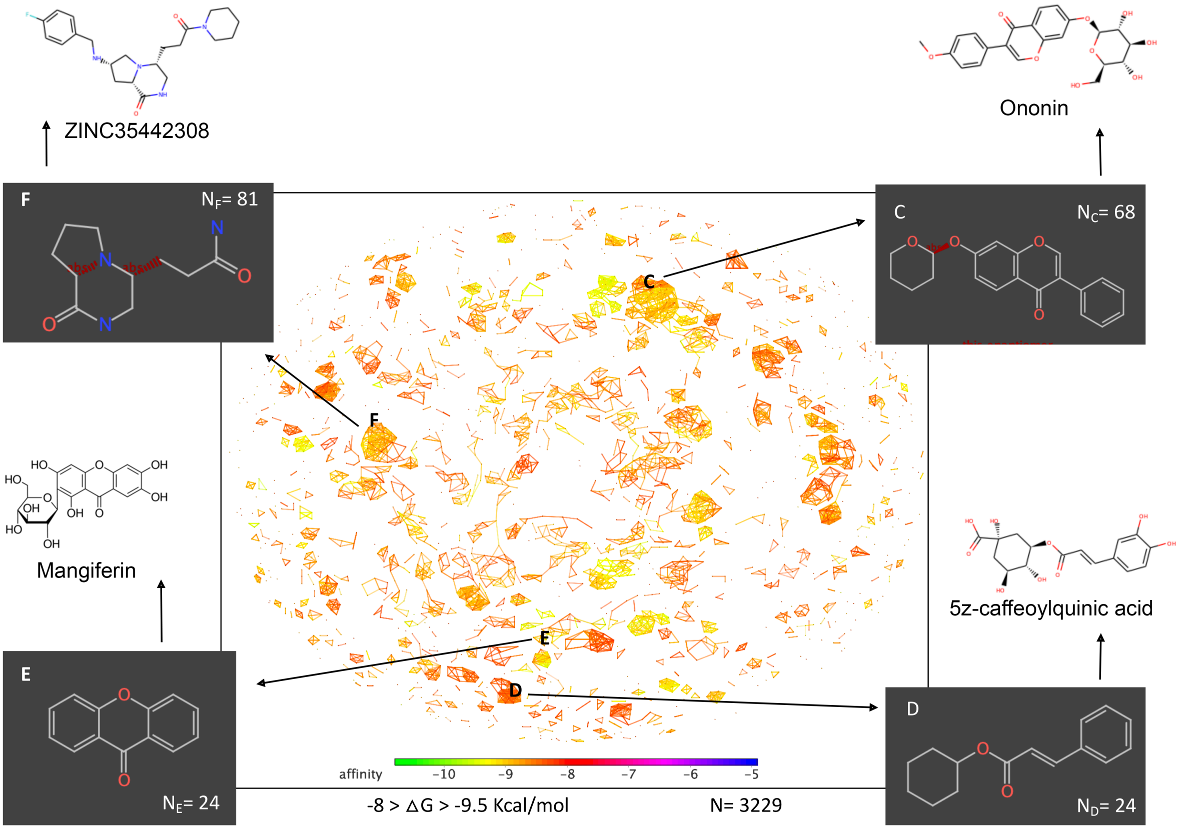

Figure S6, Panel C. SALI plot clustering of the 3229 MPA2 (moderately high scores) ligands binding to FNO and structural similarity. The compounds and parent scaffolds thereof selected for following experimental studies are shown.

Two compounds were selected the from as many highly populated/representative clusters out of subset #3 (high SALI values), also based on ease-of-retrieval (Fig. S6, Panel D).

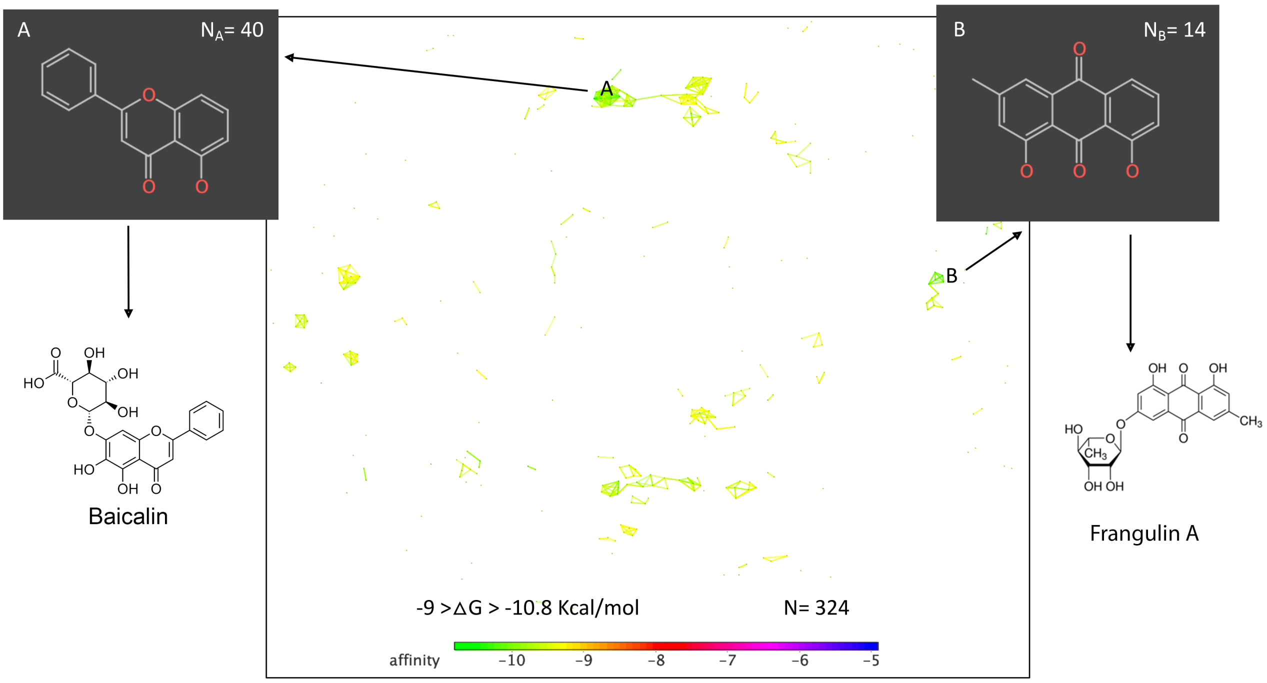

Figure S6, Panel D. SALI plot clustering of the 324 HPA ligands binding to FNO and structural similarity. The compounds and parent scaffolds thereof selected for following experimental studies are shown.

2D visualization of predictive binding mode

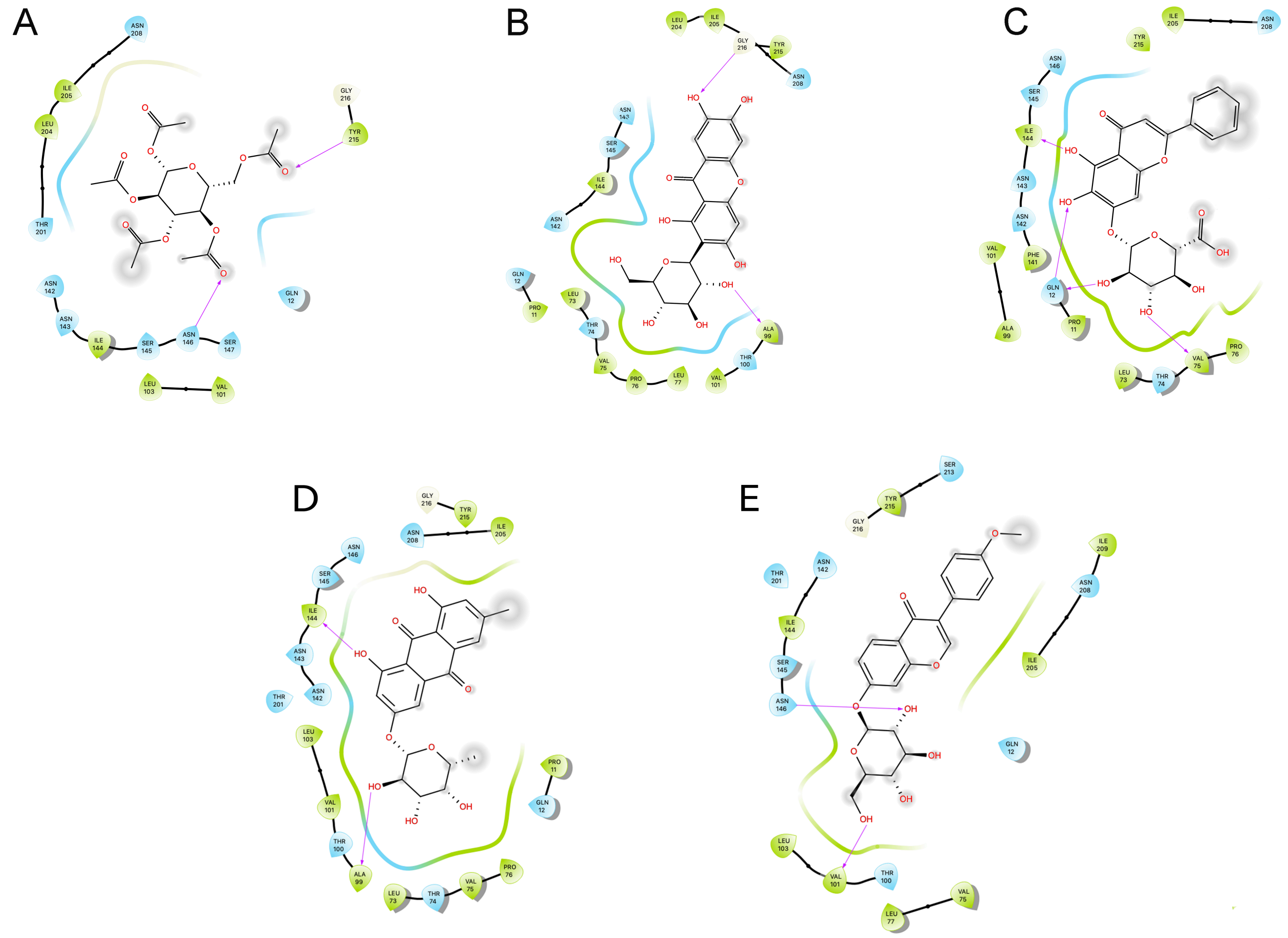

Figure S7, Panel A. 2D visualization of the binding modes of β-D-glucose pentaacetate (inset A), mangiferin (inset B), baicalin (inset C), Frangulin A (inset D) and Ononin (inset E) to FNO. Predicted H-bonds are indicated as violet arrows (donor-to-acceptor); polar and hydrophobic interactions, as well as polar and non-polar residues, are indicated in light blue and green ribbons, respectively; functional groups exposed to solvent are highlighted with grey circles.

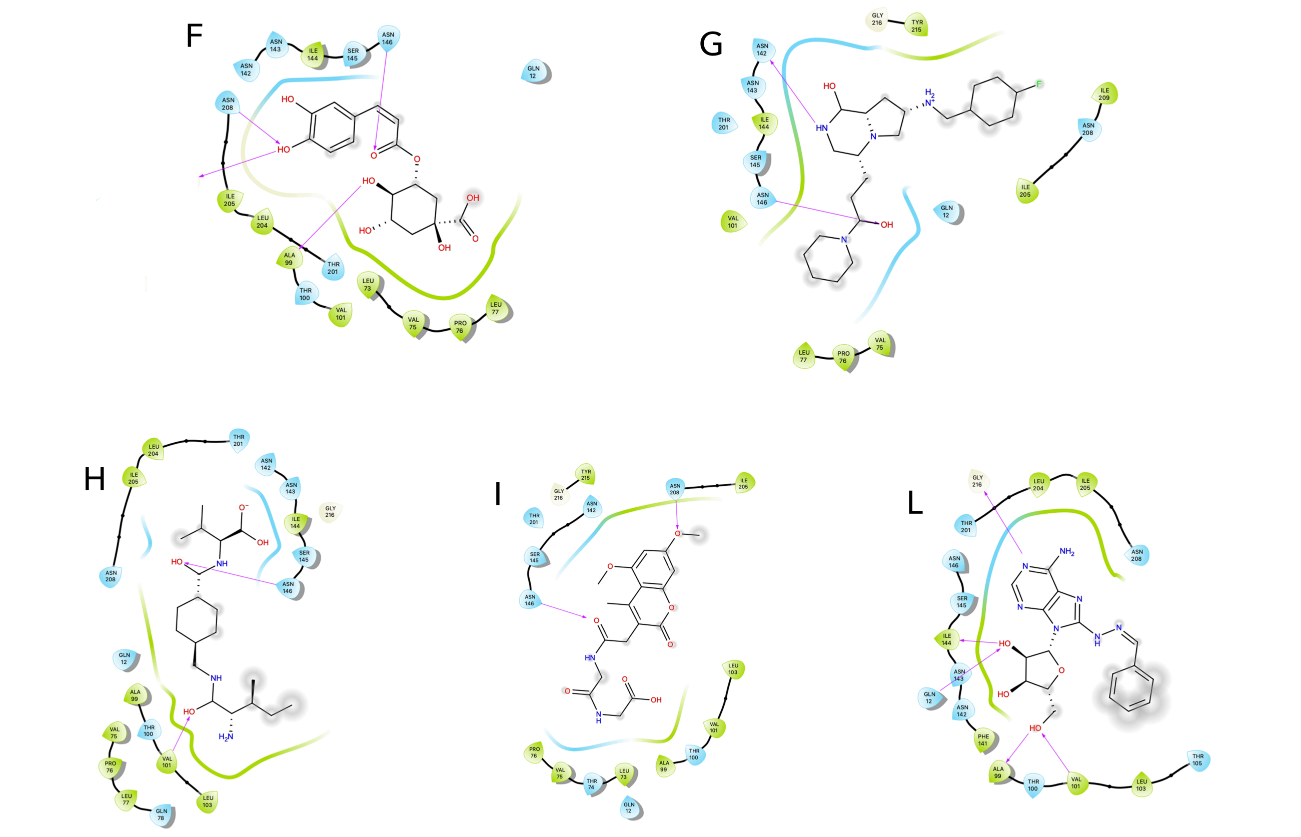

Figure S7, Panel B. 2D visualization of the binding modes of 5z-caffeolquinic acid (inset F), ZINC35442308 (inset G), ZINC248099510 (inset H), ZINC2120951 (inset I) and ZINC9271779 (inset L) to FNO. Predicted H-bonds are indicated as violet arrows (donor-to-acceptor); polar and hydrophobic interactions, as well as polar and non-polar residues, are indicated in light blue and green ribbons, respectively; functional groups exposed to solvent are highlighted with grey circles.

Binding studies

The interactions between FNO and 10 representative ligands selected rationally from the most populated/representative clusters were experimentally characterized. Kinetic and equilibrium parameters of individual complexes were measured according to the biosensor binding assay described in the manuscript. Sensorgrams are presented in the panels of Fig. S8.

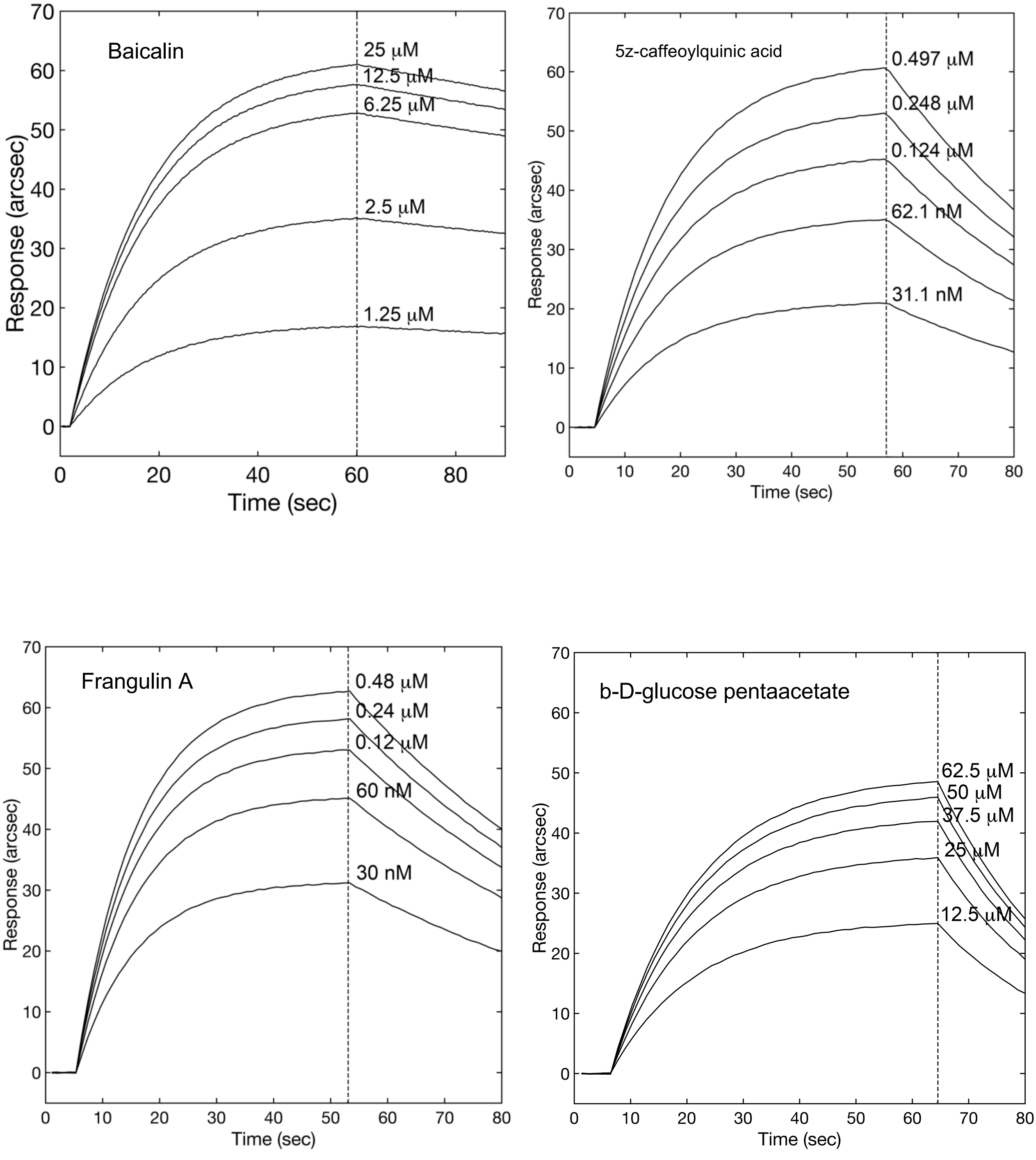

Figure S8, Panel A. Overlay of association and dissociation kinetics of binding obtained upon addition of different concentrations of the ten compounds of interest to surface-blocked FNO.

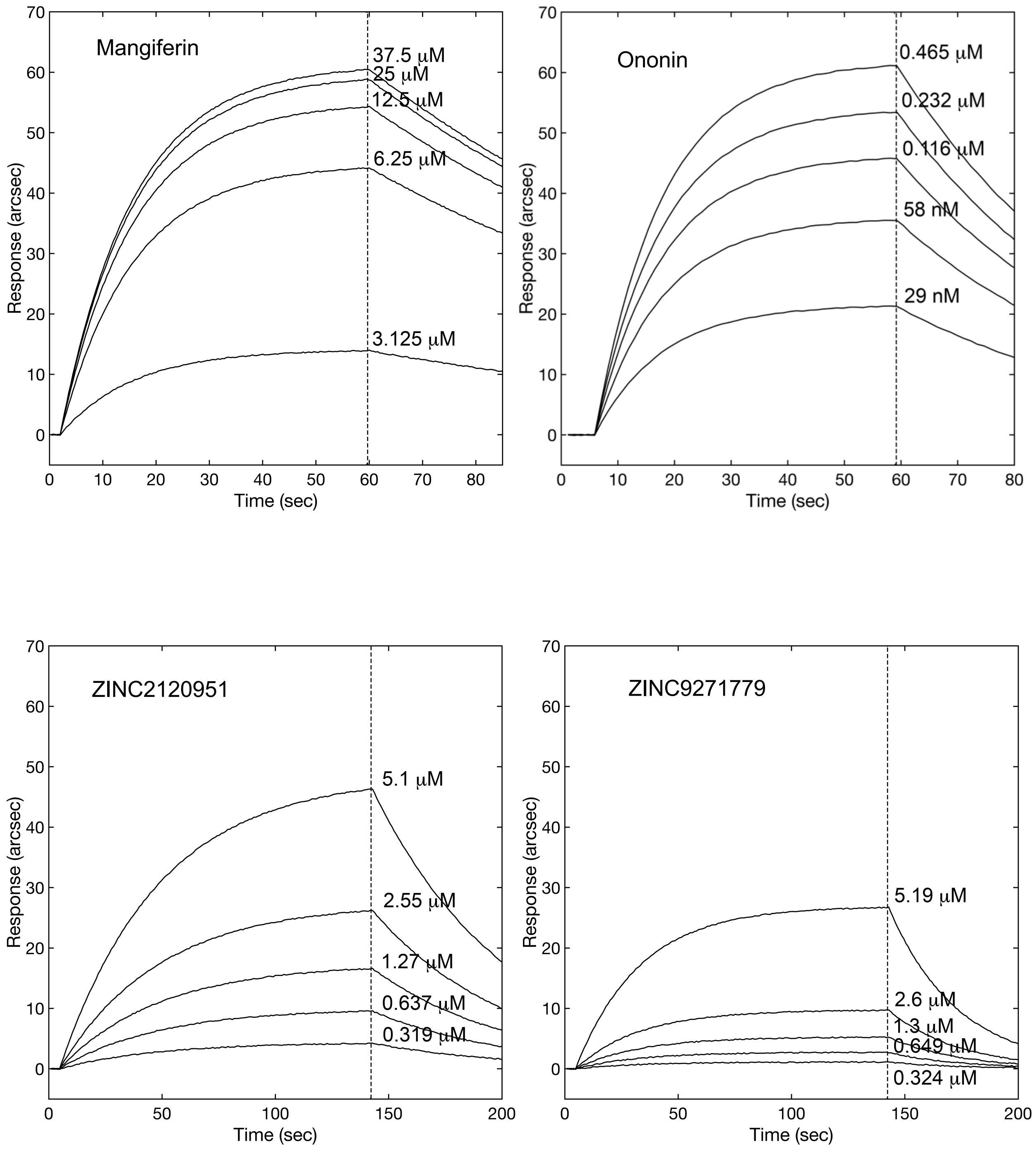

Figure S8, Panel B. Overlay of association and dissociation kinetics of binding obtained upon addition of different concentrations of the ten compounds of interest to surface-blocked FNO.

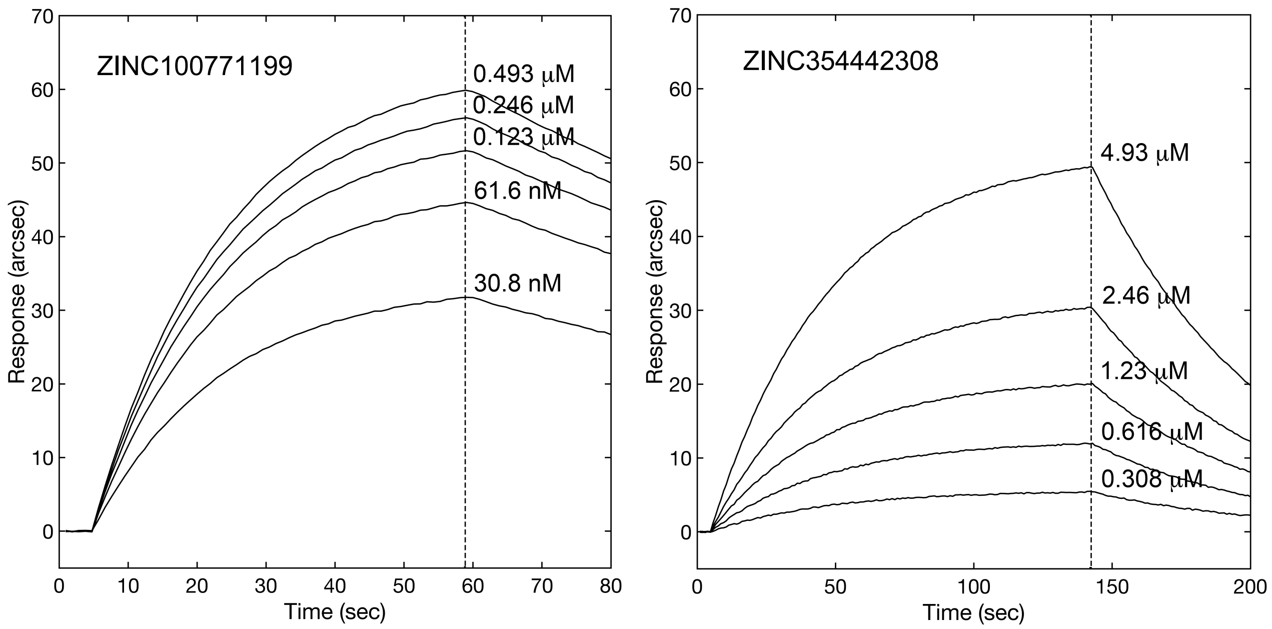

Figure S8, Panel C. Overlay of association and dissociation kinetics of binding obtained upon addition of different concentrations of the ten compounds of interest to surface-blocked FNO.

Inhibition studies

The inhibitory activity of the 10 compounds toward FNO was explored according to the fluorometric activity assay described in the Methods section of the main manuscript. Residual activity plots are shown in the panels of Fig. S6.

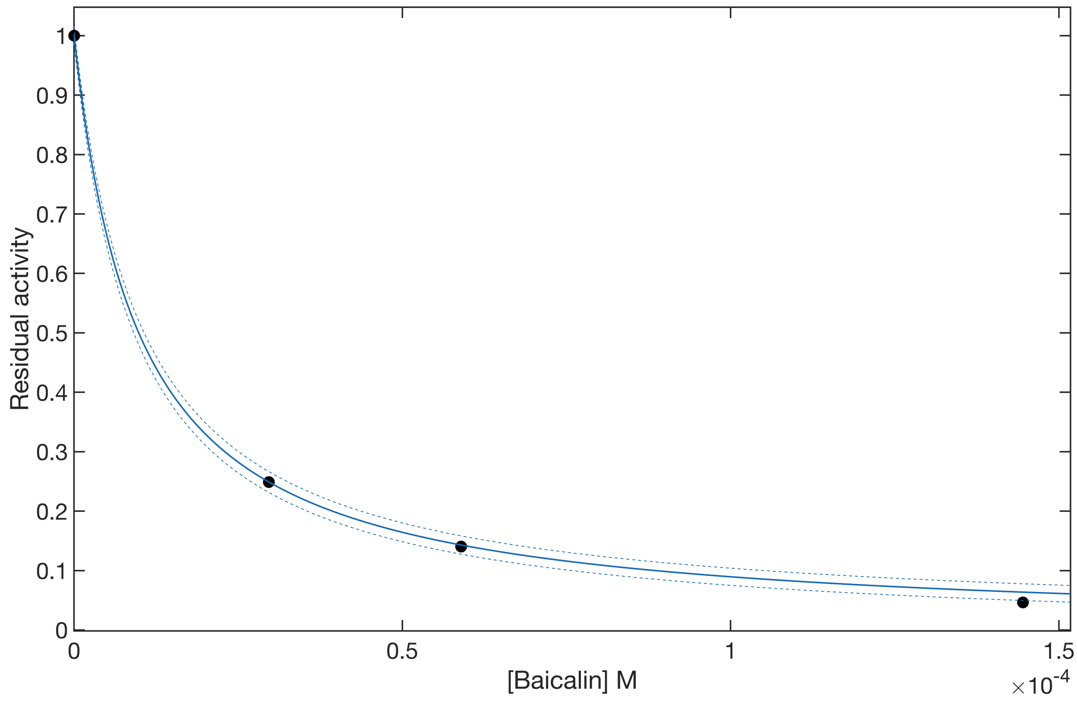

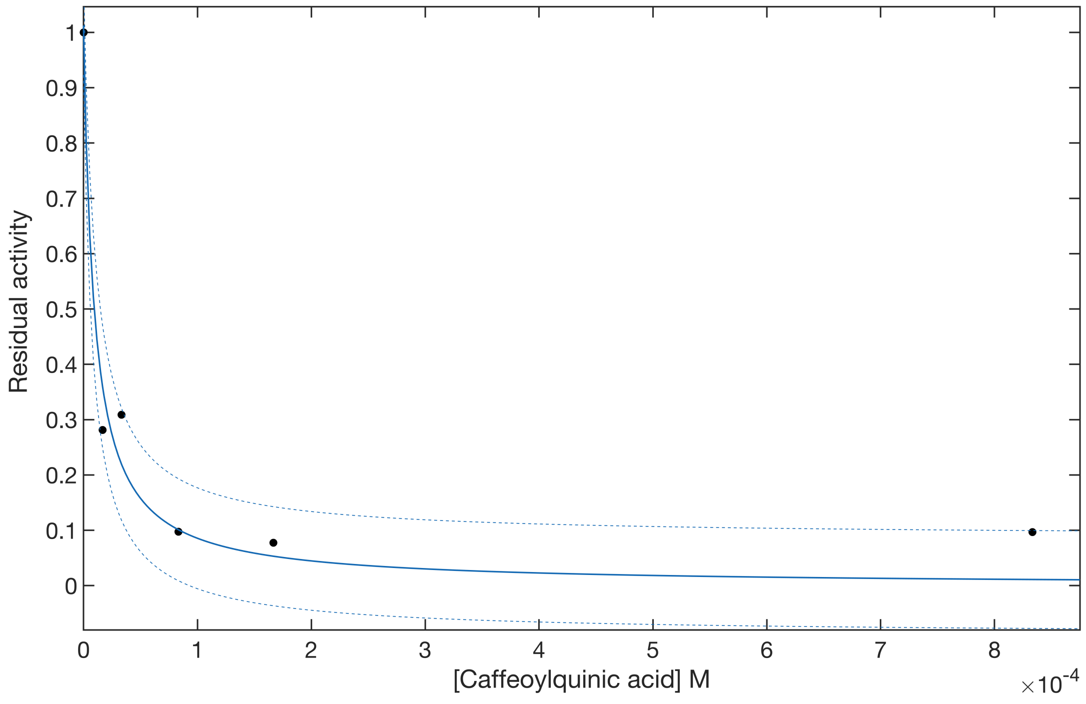

Figure S9, Panel A. Residual activity obtained by monitoring the decrease in F420 emission at 470 nm upon excitation at 420 nm in the presence and in the absence of increasing levels of the ten compounds of interest.

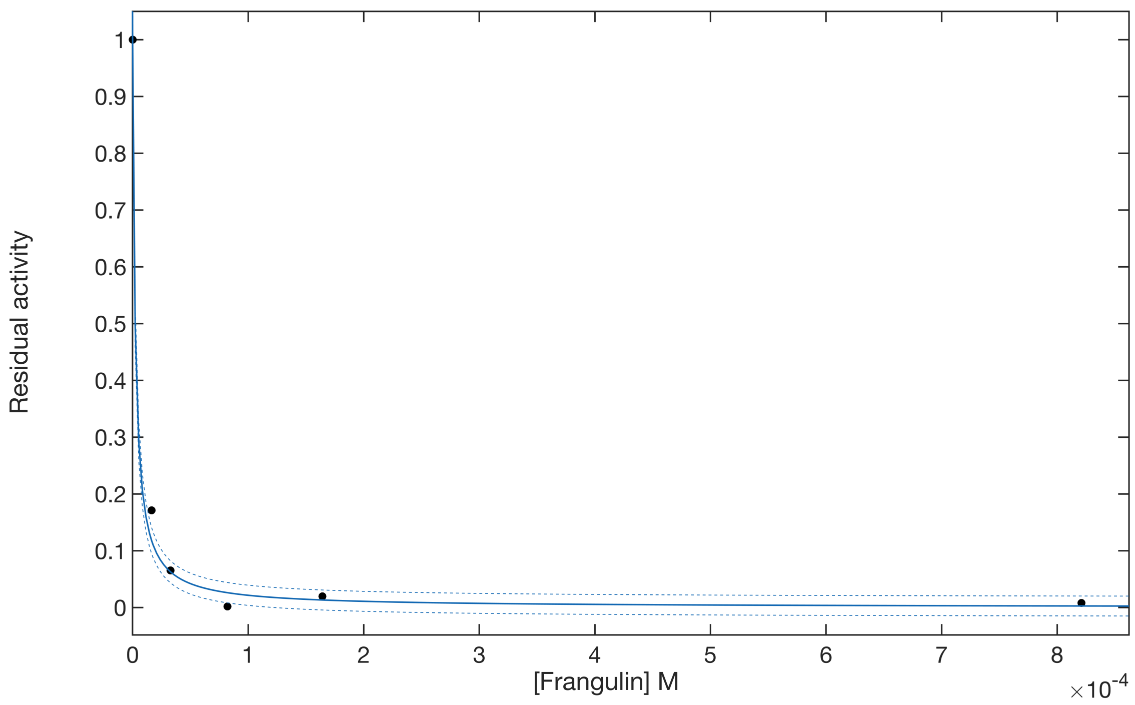

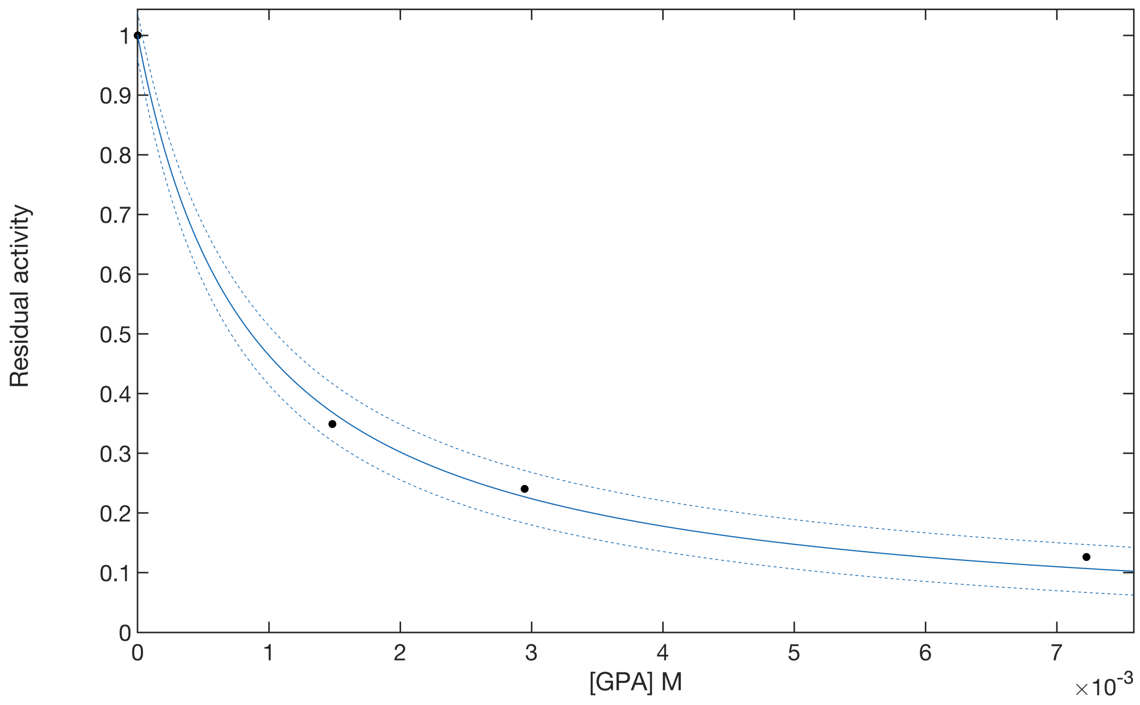

Figure S9, Panel B. Residual activity obtained by monitoring the decrease in F420 emission at 470 nm upon excitation at 420 nm in the presence and in the absence of increasing levels of the ten compounds of interest.

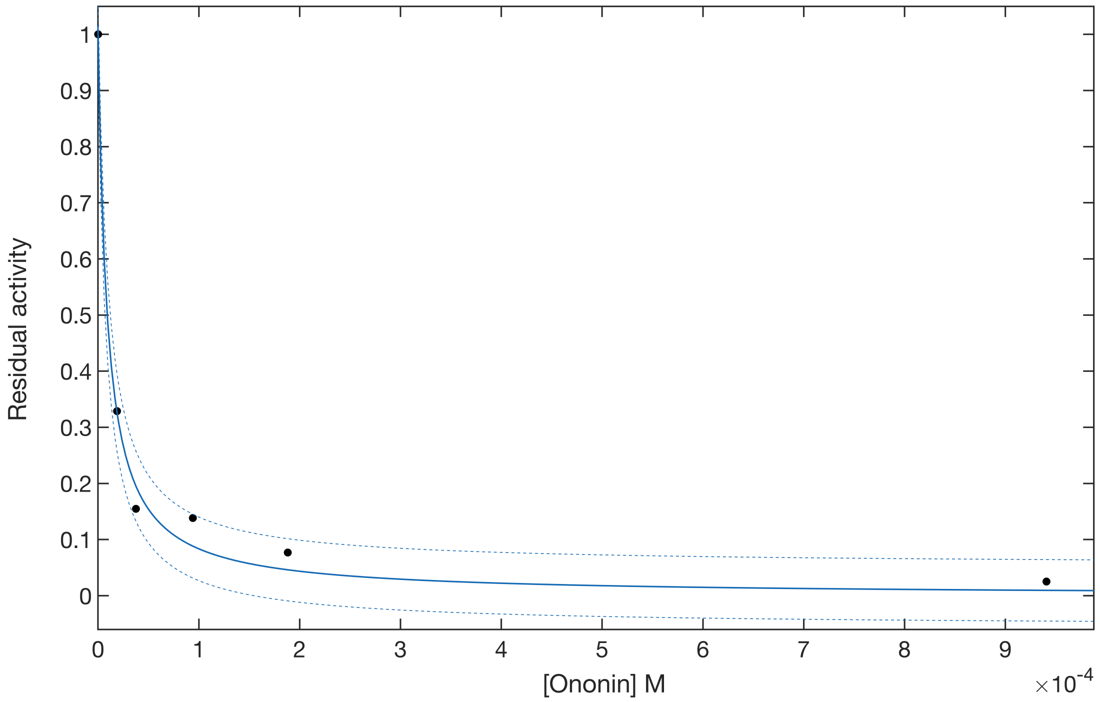

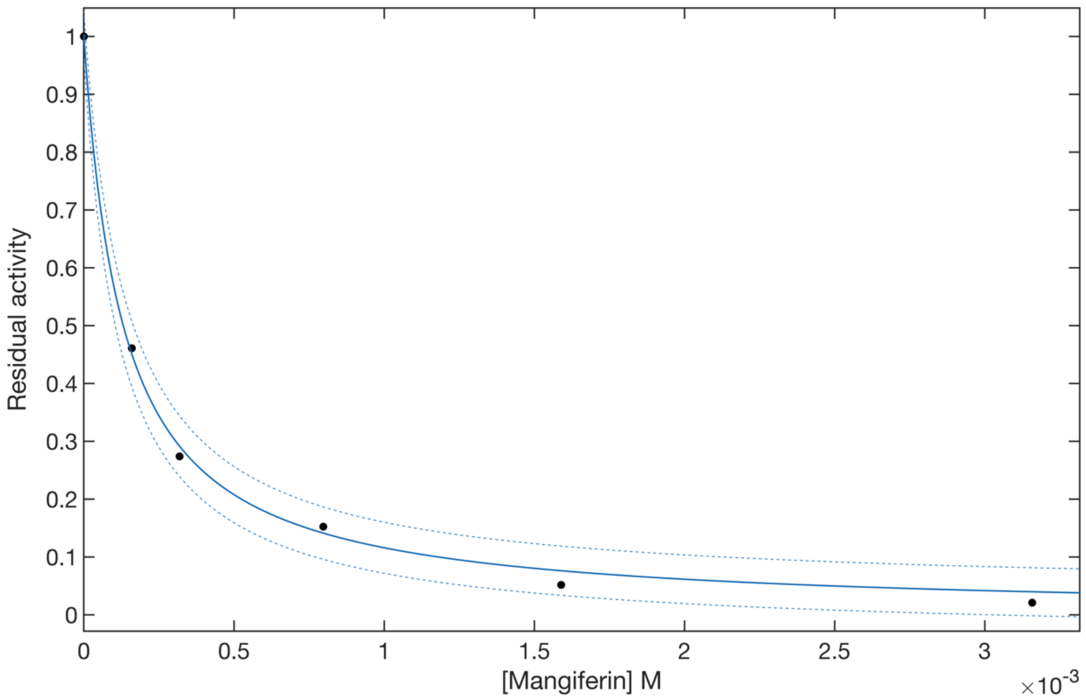

Figure S9, Panel C. Residual activity obtained by monitoring the decrease in F420 emission at 470 nm upon excitation at 420 nm in the presence and in the absence of increasing levels of the ten compounds of interest.

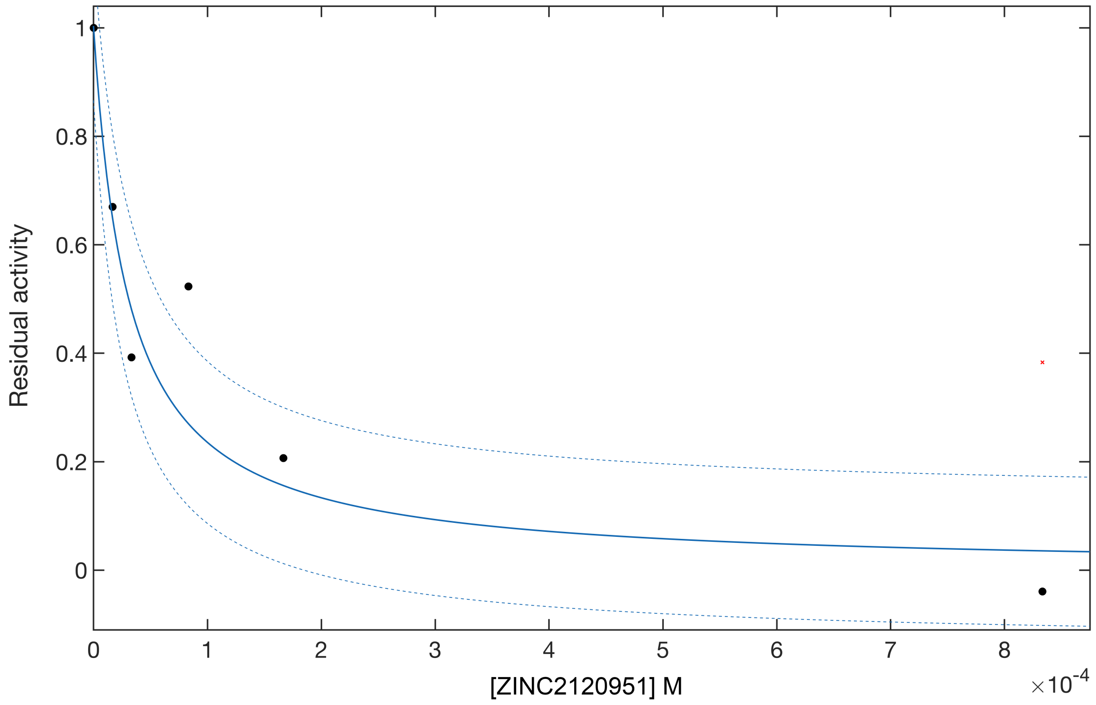

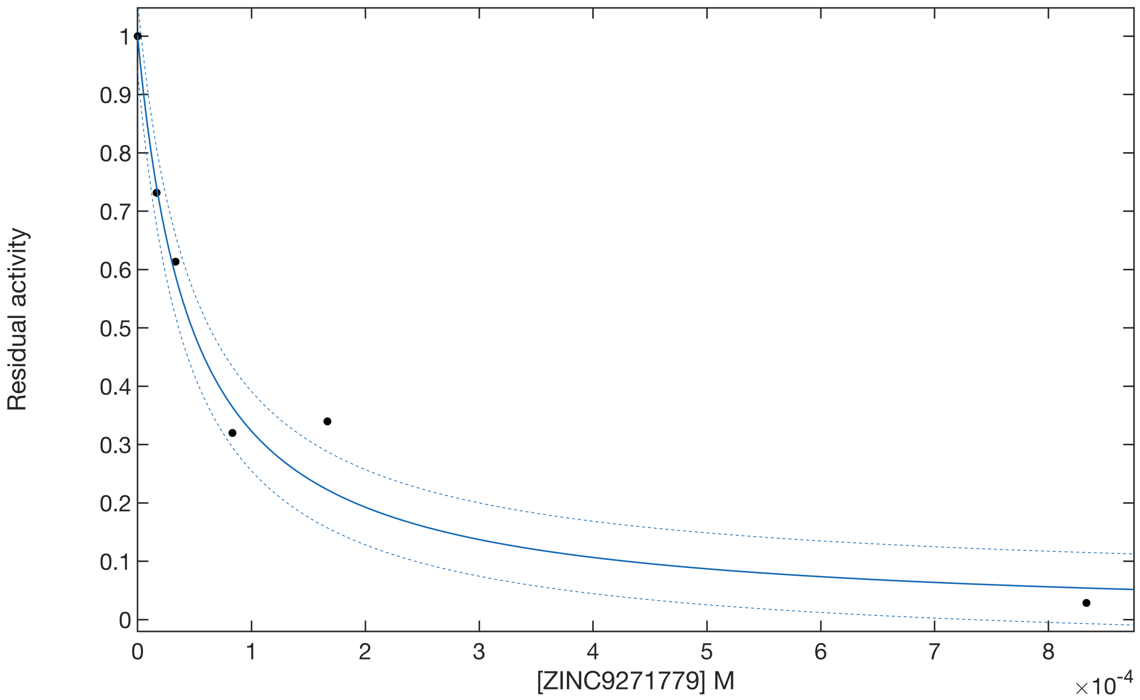

Figure S9, Panel D. Residual activity obtained by monitoring the decrease in F420 emission at 470 nm upon excitation at 420 nm in the presence and in the absence of increasing levels of the ten compounds of interest.

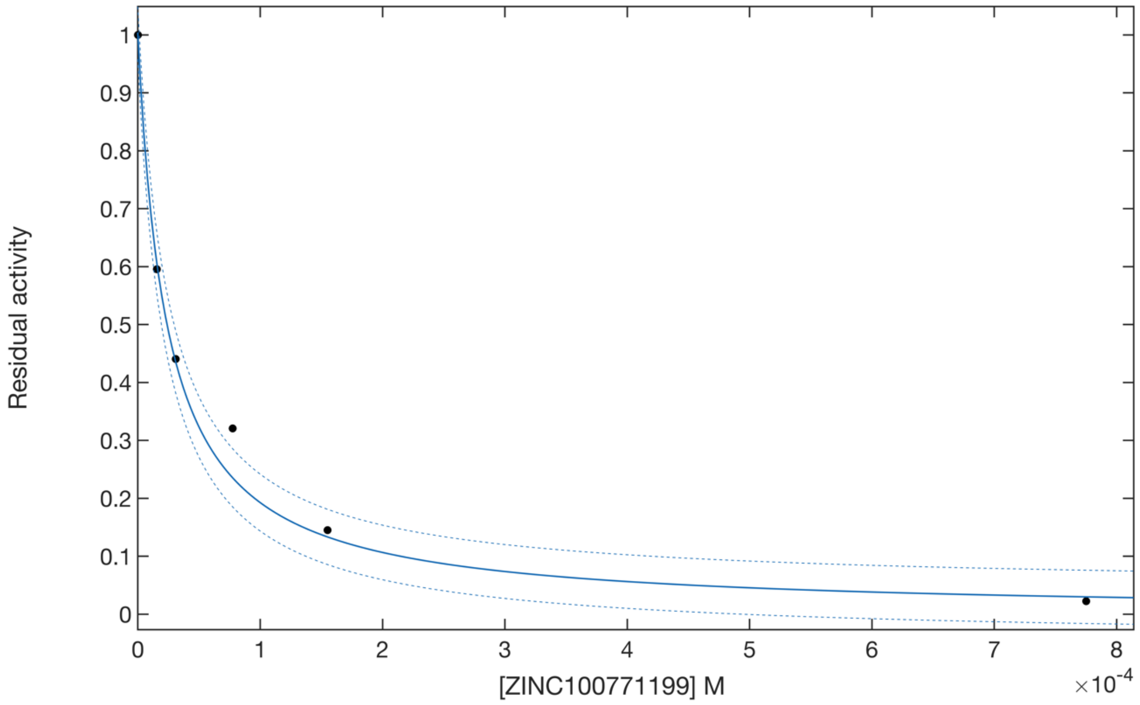

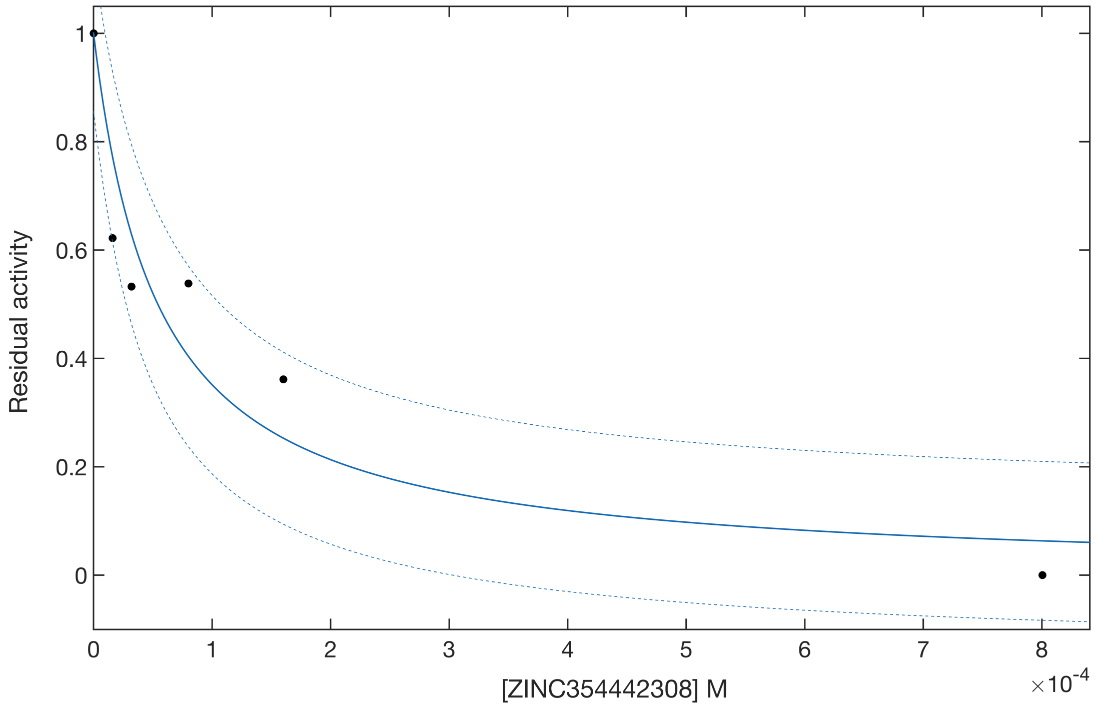

Figure S9, Panel E. Residual activity obtained by monitoring the decrease in F420 emission at 470 nm upon excitation at 420 nm in the presence and in the absence of increasing levels of the ten compounds of interest.

References

1 Berk, H. & Thauer, R. K. Function of coenzyme F420-dependent NADP reductase in methanogenic archaea containing an NADP-dependent alcohol dehydrogenase. *Arch Microbiol* **168**, 396-402, doi:10.1007/s002030050514 (1997).

2 Ney, B. *et al.* The methanogenic redox cofactor F420 is widely synthesized by aerobic soil bacteria. *ISME J* **11**, 125-137, doi:10.1038/ismej.2016.100 (2017).
