# Supplementary material for: Structure/activity virtual screening and in vitro testing of small molecule inhibitors of 8-hydroxy-5-deazaflavin:NADPH oxidoreductase from gut methanogenic bacteria
Source: Sci Rep. 2020 Aug 4;10:13150. doi: 10.1038/s41598-020-70042-w (PMC7588429; doi:10.1038/s41598-020-70042-w)
Supplement: Supplementary file 2 — Supplementary information 2 [file 41598_2020_70042_MOESM2_ESM.pdf]

Structure	Molecule Name	affinity	Total Molweight	cLogP	H-Acceptors	H-Donors	Total Surface Area	Relative PSA	Polar Surface Area	Druglikeness	Kd (nM) @ 20°C	LE from K...	LLE from Kd ...	LELP from Kd (n...	Shape Index	Molecular...	Neighbor Si...	Neig...	Neighbor	Neighbor ...	Neighbor ...	Ring Systems	frequency
	ZINC20411455	-10.8	393.398	-1.2795	8	2	265.82	0.30558	114.06	2.7555	8.870246515495	0.38091	9.3316	-3.359	0.48276	0.40085	0.92232 (max o	2	394; 45	0.77074337	0.2231622		2.0
	ZINC12153569	-10.6	445.355	-2.0792	11	5	298.01	0.45589	186.04	1.8339	12.50396780518	0.33881	9.9822	-6.1368	0.46875	0.28511	0.9642 (max of	11	5353; 272; 204; 183;	-0.1774190	0.64742494		38.0
	ZINC05742774	-10.5	416.381	1.2776	9	5	280.29	0.39809	153.75	1.4258	14.84582308777	0.35799	6.5508	3.5688	0.46667	0.32989	0.96589 (max o	5	713; 24; 20; 19; 14	0.6640411	0.09584729		3.0
	ZINC06073406	-10.4	392.374	1.0388	10	2	279.05	0.37506	124.07	5.1455	17.62628204003	0.3668	6.715	2.832	0.51724	0.32681		0		-0.2285632	-0.9484296		1.0
	ZINC59736941	-10.3	445.355	-2.0792	11	5	298.01	0.45589	186.04	1.8339	20.92745603031	0.32922	9.7585	-6.3155	0.46875	0.28742	0.95949 (max o	12	5353; 272; 204; 183;	-0.1354661	0.66775		38.0
	ZINC13424716	-10.3	445.355	-2.0792	11	5	298.01	0.45589	186.04	1.8339	20.92745603031	0.32922	9.7585	-6.3155	0.46875	0.28511	0.9642 (max of	12	5353; 272; 204; 183;	-0.1173855	0.65621376		38.0
	ZINC16248871	-10.3	444.439	-4.6138	10	6	307.33	0.45118	185.65	2.419	20.92745603031	0.32922	12.293	-14.014	0.375	0.36573	0.9497 (max of	4	1846; 1316; 400; 65	-0.7283884	-0.5468258		8.0
	ZINC16248871	-10.3	443.431	-3.2475	10	5	293.29	0.4249	184.45	4.6853	20.92745603031	0.32922	10.927	-9.8642	0.375	0.36418	0.94718 (max o	5	1850; 1849; 1318; 40	-0.1329601	0.7091215		8.0
	ZINC13432670	-10.3	384.339	1.2744	8	5	253.71	0.41236	152.36	1.6245	20.92745603031	0.37625	6.4049	3.3871	0.42857	0.25957	0.94862	1	587	-0.8374433	-0.2428699		3.0
	ZINC16248871	-10.3	442.423	-5.3235	10	4	294.47	0.42721	187.28	5.0928	20.92745603031	0.32922	13.003	-16.17	0.375	0.36418	0.94464 (max o	4	1845; 399; 66; 64	-0.4530251	-0.4863569		3.0
	ZINC16248871	-10.3	443.431	-6.6898	10	5	308.51	0.45328	188.48	2.3981	20.92745603031	0.32922	14.369	-20.32	0.375	0.36573	0.94718 (max o	3	1851; 403; 68	-0.3376061	-0.5020314		3.0
	ZINC16248871	-10.3	443.431	-6.6182	10	5	308.51	0.45328	188.48	2.5036	20.92745603031	0.32922	14.297	-20.103	0.375	0.36573	0.89541	1	1852	-0.5719966	-0.4968865		1.0
	ZINC05742777	-10.2	416.381	1.2776	9	5	280.29	0.39809	153.75	1.4258	24.84693776758	0.34776	6.3271	3.6738	0.46667	0.32989	0.96589 (max o	7	1433; 713; 370; 24; 2	0.66629326	0.0737677		3.0
	ZINC04260191	-10.2	411.461	0.2018	9	2	297.62	0.28634	97.98	6.4867	24.84693776758	0.34776	7.4029	0.58029	0.56667	0.48643	0.86238 (max o	3	3230; 217; 198	-0.2696651	0.1595179		1.0
	ZINC04349040	-10.1	445.355	-2.0792	11	5	298.01	0.45589	186.04	1.8339	29.50044691433	0.32283	9.6094	-6.4406	0.46875	0.28511	0.95709 (max o	12	5353; 272; 204; 183;	-0.1377228	0.6484102		38.0
	ZINC28630683	-10.1	443.431	-3.4078	10	5	292.46	0.42611	184.45	5.1912	29.50044691433	0.32283	10.938	-10.556	0.375	0.3646	0.9922 (max of	7	989; 389; 315; 122; 1	-0.1197029	-0.5495536		22.0
	ZINC28630683	-10.1	444.439	-4.7741	10	6	306.5	0.4524	185.65	2.9936	29.50044691433	0.32283	12.304	-14.788	0.375	0.36615	0.9922 (max of	5	1630; 314; 121; 74; 4	0.06087255	-0.5583537		22.0
	ZINC28630683	-10.1	443.431	-6.7785	10	5	307.68	0.4545	188.48	3.0758	29.50044691433	0.32283	14.309	-20.997	0.375	0.36615	0.96186 (max o	7	992; 392; 390; 316; 1	0.05708493	0.601358		5.0
	ZINC31425373	-10.1	416.381	1.2776	9	5	280.29	0.39809	153.75	1.4258	29.50044691433	0.34435	6.2526	3.7102	0.46667	0.32989	0.96589 (max o	5	713; 3; 14; 19; 20	0.64747596	0.08184440		3.0
	ZINC05742775	-10.1	416.381	1.2776	9	5	280.29	0.39809	153.75	1.4258	29.50044691433	0.34435	6.2526	3.7102	0.46667	0.32703	0.96589 (max o	5	713; 24; 3; 20; 14	0.6460809	0.10428081		3.0
	ZINC05742778	-10.1	416.381	1.2776	9	5	280.29	0.39809	153.75	1.4258	29.50044691433	0.34435	6.2526	3.7102	0.46667	0.32703	0.96589 (max o	7	713; 480; 205; 24; 3;	0.63156366	0.06770994		3.0
	ZINC03844920	-10.1	354.277	-0.845	10	4	248.02	0.50028	150.54	3.8342	29.50044691433	0.39733	8.3752	-2.1267	0.57692	0.46778	0.86285	1	54	-0.3168928	-0.9279408		2.0
	ZINC04266672	-10.1	428.534	2.1363	6	4	282.98	0.17316	86.14	-1.8087	29.50044691433	0.32283	5.3939	6.6175	0.4375	0.17217	0.92889	1	912	0.1721997	-0.843311		1.0
	ZINC04266672	-10.1	429.542	-0.108	6	4	284.58	0.15911	90.72	-2.288	29.50044691433	0.32283	7.6382	-0.33454	0.4375	0.31595	0.92348	1	913	-0.7764745	-0.5885158		1.0
	ZINC04349038	-10.0	445.355	-2.0792	11	5	298.01	0.45589	186.04	1.8339	35.02555530565	0.31963	9.5348	-6.505	0.46875	0.28511	0.95949 (max o	12	5353; 272; 204; 183;	-0.1406074	0.62872446		38.0
	ZINC13424717	-10.0	445.355	-2.0792	11	5	298.01	0.45589	186.04	1.8339	35.02555530565	0.31963	9.5348	-6.505	0.46875	0.28742	0.95949 (max o	11	5353; 272; 204; 183;	-0.1738879	0.668289		38.0
	ZINC31161007	-10.0	429.356	-1.7335	10	4	291.66	0.4209	165.81	1.8339	35.02555530565	0.32994	9.1891	-5.2539	0.48387	0.37235	0.9499 (max of	7	5353; 289; 147; 91; 4	-0.0778454	0.6447828		38.0
	ZINC34114798	-10.0	445.355	-2.0792	11	5	298.01	0.45589	186.04	1.8339	35.02555530565	0.31963	9.5348	-6.505	0.46875	0.29053	0.95465 (max o	12	5353; 272; 204; 183;	-0.1536134	0.6788694		15.0
	ZINC13424721	-10.0	448.379	-0.4237	11	8	295.54	0.4664	197.37	-1.9977	35.02555530565	0.31963	7.8793	-1.3256	0.5	0.38295	0.9642 (max of	11	3282; 3243; 2828; 28	0.7167476	0.07023124		14.0
	ZINC15957170	-10.0	432.455	-3.3242	8	2	305.72	0.30391	118.64	4.3827	35.02555530565	0.31963	10.78	-10.4	0.40625	0.29534	0.96186 (max o	9	985; 798; 636; 633; 3	0.16105498	-0.5691871		7.0
	ZINC04654944	-10.0	425.111	-5.1577	12	4	227.54	0.61352	176.51	-11.326	35.02555530565	0.42618	12.613	-12.102	0.45833	0.39802	0.95709	1	4242	-0.2796684	-0.2963932		2.0
	ZINC04235977	-10.0	409.397	0.9029	9	2	285.48	0.31799	108.41	6.406	35.02555530565	0.34094	6.5527	2.6483	0.53333	0.34637	0.89009 (max o	3	1137; 443; 261	-0.2289655	0.2844947		1.0
	ZINC12376939	-9.9	429.356	-1.7335	10	4	291.66	0.4209	165.81	1.8339	41.58545557062	0.32664	9.1146	-5.307	0.48387	0.37032	0.95771 (max o	5	289; 147; 91; 5; 31	-0.0861378	0.62563527		38.0
	ZINC13543704	-9.9	445.355	-2.0792	11	5	298.01	0.45589	186.04	1.8339	41.58545557062	0.31644	9.4603	-6.5707	0.5	0.37436	0.9499 (max of	3	270; 133; 31	-0.0146896	0.65959746		38.0
	ZINC08952594	-9.9	445.355	-2.0792	11	5	298.01	0.45589	186.04	1.8339	41.58545557062	0.31644	9.4603	-6.5707	0.46875	0.28742	0.94718 (max o	6	5353; 272; 12; 18; 31	-0.1157364	0.6759897		38.0
	ZINC65740202	-9.9	429.428	-5.118	10	6	299.76	0.46981	191.44	2.1976	41.58545557062	0.32664	12.499	-15.668	0.3871	0.36003	0.95521 (max o	4	165; 70; 69; 49	-0.0426220	-0.5306324		22.0
	ZINC65740202	-9.9	428.420	-3.7517	10	5	285.72	0.44376	190.24	4.5417	41.58545557062	0.32664	11.133	-11.486	0.3871	0.35841	0.95521 (max o	4	168; 70; 69; 48	-0.0747250	-0.5541972		22.0
	ZINC20410403	-9.9	443.431	-3.4078	10	5	292.46	0.42611	184.45	5.1912	41.58545557062	0.31644	10.789	-10.769	0.375	0.3646	0.9922 (max of	8	989; 389; 315; 168; 1	-0.1206404	-0.5900968		22.0
	ZINC20410403	-9.9	444.439	-4.7741	10	6	306.5	0.4524	185.65	2.9936	41.58545557062	0.31644	12.155	-15.087	0.375	0.36615	0.9922 (max of	6	1630; 314; 165; 121;	0.02818380	-0.5474417		22.0
	ZINC35270959	-9.9	419.416	-3.5167	9	2	290.4	0.33254	125.37	6.2316	41.58545557062	0.32664	10.898	-10.766	0.41935	0.3965	0.96018 (max o	7	508; 317; 308; 306; 2	-0.7825737	0.05213582		8.0
	ZINC85341087	-9.9	434.396	0.241	10	6	291.63	0.4171	166.14	1.8553	41.58545557062	0.32664	7.1401	0.73781	0.51613	0.37936	0.86365	1	2275	0.7268916	-0.4610636		7.0
	ZINC20410403	-9.9	444.439	-4.8782	10	5	307.48	0.45076	182.49	3.1697	41.58545557062	0.31644	12.259	-15.416	0.375	0.37716	0.9922 (max of	2	1313; 151	0.24259381	-0.2974722		6.0
	ZINC35270959	-9.9	420.424	-1.7144	9	2	286.68	0.32388	129.95	4.3355	41.58545557062	0.32664	9.0955	-5.2485	0.41935	0.3965	0.96502 (max o	5	307; 305; 237; 178; 9	-0.7655649	-0.1878583		6.0

Structure	Molecule Name	affinity	Total Molweight	cLogP	H-Acceptors	H-Donors	Total Surface Area	Relative PSA	Polar Surface Area	Druglikeness	Kd (nM) @ 20°C	LE from K...	LLE from Kd ...	LELP from Kd (n...	Shape Index	Molecular...	Neighbor Si...	Neig...	Neighbor	Neighbor ...	Neighbor ...	Ring Systems	frequency
	ZINC20410403	-9.9	443.431	-6.7785	10	5	307.68	0.4545	188.48	3.0758	41.58545557062	0.31644	14.16	-21.421	0.375	0.36615	0.99372 (max o	4	992; 392; 316; 23	0.05995281	0.58219784		5.0
	ZINC05734148	-9.9	398.366	1.3494	8	4	272.38	0.37271	141.36	1.1103	41.58545557062	0.34917	6.0317	3.8646	0.48276	0.26389		0		-0.9534667	-0.0545034		3.0
	ZINC20412362	-9.9	407.425	-1.0266	8	1	276.92	0.26477	105.27	1.5666	41.58545557062	0.33753	8.4077	-3.0415	0.46667	0.40358	0.92232 (max o	2	394; 1	0.77158713	0.24542291		2.0
	ZINC04236168	-9.9	398.369	-1.6032	8	1	279.16	0.30448	109.85	0.73627	41.58545557062	0.34917	8.9843	-4.5915	0.58621	0.40635	0.8886 (max of	4	1139; 902; 83; 55	-0.4984811	-0.5177395		2.0
	ZINC04260142	-9.9	430.478	0.6449	8	1	312.31	0.23781	90.03	6.4176	41.58545557062	0.32664	6.7362	1.9743	0.6129	0.47787	0.95243 (max o	3	2810; 198; 84	-0.2727897	0.09433079		2.0
	ZINC05892598	-9.9	412.417	-2.5003	8	5	276.11	0.3839	159.77	-1.221	41.58545557062	0.33753	9.8814	-7.4076	0.4	0.27624		0		-0.8059131	0.2162688		2.0
	ZINC04019716	-9.9	443.431	-3.1525	10	7	304.14	0.45203	182.82	-0.52664	41.58545557062	0.31644	10.534	-9.9625	0.375	0.3649	0.80895	1	156	-0.5660483	0.80441004		1.0
	ZINC29547079	-9.8	428.420	-3.7517	10	5	285.72	0.44376	190.24	4.5417	49.37387198151	0.32334	11.058	-11.603	0.3871	0.35841	0.95521 (max o	4	168; 48; 49; 69	-0.0581782	-0.5682766		22.0
	ZINC29547079	-9.8	429.428	-5.118	10	6	299.76	0.46981	191.44	2.1976	49.37387198151	0.32334	12.425	-15.828	0.3871	0.36003	0.95521 (max o	5	166; 165; 70; 48; 49	-0.0355356	-0.5635348		22.0
	ZINC30690961	-9.8	443.431	-3.4078	10	5	292.46	0.42611	184.45	5.1912	49.37387198151	0.31324	10.714	-10.879	0.375	0.3646	0.96186 (max o	7	989; 389; 315; 122; 1	-0.1400506	-0.5970838		22.0
	ZINC30690961	-9.8	444.439	-4.7741	10	6	306.5	0.4524	185.65	2.9936	49.37387198151	0.31324	12.081	-15.241	0.375	0.36615	0.95219 (max o	5	1630; 314; 121; 21; 4	0.04933669	-0.5413503		22.0
	ZINC16032786	-9.8	432.455	-3.3242	8	2	305.72	0.30391	118.64	4.3827	49.37387198151	0.31324	10.631	-10.612	0.40625	0.29534	0.96186 (max o	8	799; 636; 635; 633; 2	0.19451338	-0.5772092		7.0
	ZINC04097896	-9.8	434.396	0.1664	10	6	294.39	0.41319	166.14	-3.7146	49.37387198151	0.32334	7.1401	0.51462	0.51613	0.39353	0.96739 (max o	5	312; 220; 194; 177; 1	0.07721887	-0.3378898		7.0
	ZINC04081849	-9.8	392.403	0.4155	8	4	272.39	0.35038	125.68	-8.3754	49.37387198151	0.35799	6.891	1.1607	0.5	0.30543	0.95148 (max o	6	6710; 1120; 342; 135	0.3110069	0.4688948		7.0
	ZINC12375032	-9.8	432.380	-0.2446	10	6	289.64	0.43047	173.98	-4.2658	49.37387198151	0.32334	7.5511	-0.75647	0.48387	0.28023	0.96018 (max o	6	480; 367; 288; 205; 9	0.6396577	0.02315129		7.0
	ZINC30690961	-9.8	443.431	-6.4562	10	4	308.66	0.45286	185.32	3.1697	49.37387198151	0.31324	13.763	-20.611	0.375	0.37716		0		0.9376755	-0.3255371		6.0
	ZINC29547079	-9.8	428.420	-6.8001	10	4	301.92	0.47016	191.11	2.386	49.37387198151	0.32334	14.107	-21.031	0.3871	0.36851		0		-0.2313942	0.914636		6.0
	ZINC11866707	-9.8	430.408	0.5905	9	4	302.4	0.34868	134.91	-5.7203	49.37387198151	0.32334	6.716	1.8262	0.54839	0.40723	0.95771 (max o	2	256; 214	0.2501414	0.5262912		6.0
	ZINC12375029	-9.8	432.380	-0.2446	10	6	289.64	0.43047	173.98	-4.2658	49.37387198151	0.32334	7.5511	-0.75647	0.48387	0.28345	0.95771 (max o	4	367; 288; 62; 61	0.64493173	0.00127851		6.0
	ZINC12446748	-9.8	416.381	0.6966	9	5	281.79	0.39597	153.75	-4.2232	49.37387198151	0.33412	6.6099	2.0849	0.43333	0.26094	0.95053 (max o	4	1486; 617; 92; 60	0.6291055	-0.0139577		6.0
	ZINC16248873	-9.8	444.439	-4.6138	10	6	307.33	0.45118	185.65	2.419	49.37387198151	0.31324	11.92	-14.729	0.375	0.36573	0.9497 (max of	4	1846; 1316; 400; 8	-0.7352425	-0.5088287		6.0
	ZINC16248873	-9.8	443.431	-3.2475	10	5	293.29	0.4249	184.45	4.6853	49.37387198151	0.31324	10.554	-10.367	0.375	0.36418	0.94718 (max o	5	1850; 1849; 1318; 40	-0.1126268	0.7375078		6.0
	ZINC29547079	-9.8	428.420	-7.2981	10	4	301.92	0.47016	191.11	1.6482	49.37387198151	0.32334	14.605	-22.571	0.3871	0.36851	0.9396 (max of	2	170; 154	0.15898032	-0.2738838		6.0
	ZINC29547079	-9.8	428.420	-7.194	10	5	300.94	0.47189	194.27	2.1801	49.37387198151	0.32334	14.501	-22.249	0.3871	0.36003	0.9396	1	169	-0.3444297	0.58394456		5.0
	ZINC04236170	-9.8	413.384	-1.6093	9	2	290.62	0.33191	121.88	0.50148	49.37387198151	0.33412	8.9158	-4.8165	0.56667	0.43497	0.94529 (max o	5	1139; 902; 197; 83; 3	-0.5025042	-0.5379258		4.0
	ZINC04073649	-9.8	374.344	0.6545	8	4	255.43	0.37364	125.68	-4.3275	49.37387198151	0.37125	6.652	1.763	0.51852	0.3532	0.94905	1	436	-0.1334617	-0.7776995		3.0
	ZINC16248873	-9.8	442.423	-5.3235	10	4	294.47	0.42721	187.28	5.0928	49.37387198151	0.31324	12.63	-16.995	0.375	0.36418	0.94464 (max o	3	1845; 399; 7	-0.4569961	-0.5064770		3.0
	ZINC16248873	-9.8	443.431	-6.6898	10	5	308.51	0.45328	188.48	2.3981	49.37387198151	0.31324	13.996	-21.357	0.375	0.36573	0.94718 (max o	3	1851; 403; 10	-0.3188031	-0.5133267		3.0
	ZINC30690961	-9.8	443.431	-6.7785	10	5	307.68	0.4545	188.48	3.0017	49.37387198151	0.31324	14.085	-21.64	0.375	0.36615	0.95709 (max o	7	990; 392; 390; 316; 1	0.07869932	0.5911518		3.0
	ZINC04235292	-9.8	384.307	-2.1996	12	6	265.06	0.58334	202.58	3.8187	49.37387198151	0.35799	9.5061	-6.1444	0.53571	0.41885	0.86285	1	15	-0.2952114	-0.9325248		2.0
	ZINC08627176	-9.8	447.461	0.2801	9	3	324.88	0.29226	115.48	4.6251	49.37387198151	0.31324	7.0264	0.89421	0.625	0.52015	0.9499 (max of	5	1191; 942; 208; 108;	-0.0539802	-0.6219438		2.0
	ZINC05398559	-9.8	445.523	-1.8452	10	2	326.28	0.35984	121.05	-1.5733	49.37387198151	0.32334	9.1517	-5.7066	0.54839	0.34038	0.83999	1	910	-0.2077629	-0.0703555		1.0
	ZINC08296835	-9.8	448.454	-1.3646	9	2	334.31	0.30783	127.87	4.9086	49.37387198151	0.30375	8.6711	-4.4926	0.60606	0.36029	0.92889 (max o	4	1672; 1454; 556; 464	-0.2813885	0.42204502		1.0
	ZINC16248873	-9.8	442.423	-5.2519	10	4	294.47	0.42721	187.28	5.1694	49.37387198151	0.31324	12.558	-16.766	0.375	0.36418	0.94718 (max o	2	1847; 7	-0.4857267	-0.4561699		1.0
	ZINC12376937	-9.7	429.356	-1.7335	10	4	291.66	0.4209	165.81	1.8339	58.62105373403	0.32004	8.9654	-5.4164	0.48387	0.37032	0.95771 (max o	5	289; 147; 26; 31; 39	-0.1054031	0.62398076		38.0
	ZINC12153134	-9.7	448.379	7.0003e-4	11	7	299.19	0.45035	186.37	-3.2535	58.62105373403	0.31004	7.2312	0.0022578	0.5	0.37952	0.97319 (max o	11	434; 371; 339; 338; 3	0.04393604	0.6818591		38.0
	ZINC35328494	-9.7	448.379	0.4276	11	7	295.9	0.45536	186.37	1.8856	58.62105373403	0.31004	6.8043	1.3792	0.5	0.38067	0.90963	1	4095	-0.7565044	0.30580208		38.0
	ZINC12153137	-9.7	448.379	7.0003e-4	11	7	299.19	0.45035	186.37	-3.2535	58.62105373403	0.31004	7.2312	0.0022578	0.5	0.38151	0.95949 (max o	11	434; 371; 339; 338; 3	0.08439056	0.71504414		15.0
	ZINC35270963	-9.7	419.416	-3.5167	9	2	290.4	0.33254	125.37	6.2316	58.62105373403	0.32004	10.749	-10.988	0.41935	0.3965	0.96018 (max o	7	508; 317; 308; 306; 2	-0.7544868	0.07211473		8.0
	ZINC04073830	-9.7	378.376	0.0716	8	4	260.13	0.36689	125.68	-8.4156	58.62105373403	0.36746	7.1603	0.19485	0.51852	0.3974	0.94905 (max o	7	1120; 564; 563; 343;	0.34565547	0.45163548		7.0
	ZINC12446753	-9.7	416.381	0.6966	9	5	281.79	0.39597	153.75	-4.2232	58.62105373403	0.33071	6.5353	2.1064	0.43333	0.25743	0.95318 (max o	6	1486; 617; 480; 205;	0.6253304	0.00792112		7.0
	ZINC04236404	-9.7	406.417	-0.7253	8	1	297.91	0.28532	109.85	5.0801	58.62105373403	0.33071	7.9572	-2.1931	0.56667	0.41478	0.95549 (max o	2	353; 262	-0.1878889	0.29360345		7.0
	ZINC35270963	-9.7	420.424	-1.7144	9	2	286.68	0.32388	129.95	4.3355	58.62105373403	0.32004	8.9463	-5.3568	0.41935	0.3965	0.96262 (max o	5	307; 305; 237; 178; 4	-0.7669141	-0.1422765		6.0

Structure	Molecule Name	affinity	Total Molweight	cLogP	H-Acceptors	H-Donors	Total Surface Area	Relative PSA	Polar Surface Area	Druglikeness	Kd (nM) @ 20°C	LE from K...	LLE from Kd ...	LELP from Kd (n...	Shape Index	Molecular...	Neighbor Si...	Neig...	Neighbor	Neighbor ...	Neighbor ...	Ring Systems	frequency
	ZINC04236436	-9.7	395.394	-1.7101	9	2	284.27	0.33933	121.88	1.8415	58.62105373403	0.34212	8.942	-4.9986	0.58621	0.43036	0.94529 (max o	8	2435; 1139; 902; 265	-0.4939642	-0.4970666		4.0
	ZINC04260204	-9.7	442.514	0.4741	9	1	328.22	0.25675	99.26	7.7445	58.62105373403	0.31004	6.7578	1.5291	0.625	0.48906	0.95243 (max o	5	2810; 2063; 1421; 44(	-0.2736024	0.07130414		2.0
	ZINC04099035	-9.7	447.502	-0.3861	8	4	307.67	0.32411	139.51	0.54536	58.62105373403	0.31004	7.618	-1.2453	0.5	0.32286	0.92619 (max o	4	260; 216; 195; 160	-0.0940839	-0.5000723		2.0
	ZINC31166820	-9.7	418.397	0.9358	9	5	285.85	0.37971	145.91	-9.613	58.62105373403	0.33071	6.2961	2.8297	0.46667	0.34658	0.96092 (max o	3	180; 127; 126	-0.9138202	-0.1787301		2.0
	ZINC03839300	-9.7	445.518	-1.0479	10	2	313.43	0.31513	119.13	7.8767	58.62105373403	0.31004	8.2798	-3.3799	0.53125	0.35308	0.95267 (max o	4	2002; 1706; 898; 699	-0.5478711	0.01411426		1.0
	ZINC04221764	-9.7	441.447	-1.4323	12	3	317.34	0.4302	175.53	3.2509	58.62105373403	0.31004	8.6642	-4.6197	0.5625	0.42003	0.87593 (max o	2	899; 717	-0.7286723	0.4749624		1.0
	ZINC04265921	-9.7	442.256	-7.0274	16	2	297.94	0.62932	254.82	-1.4155	58.62105373403	0.31004	14.259	-22.666	0.46875	0.44154	0.91522 (max o	2	87; 86	0.80636626	0.24036206		1.0
	ZINC04265921	-9.7	442.256	-7.0274	16	2	297.94	0.62932	254.82	-2.0965	58.62105373403	0.31004	14.259	-22.666	0.46875	0.44154	0.91522 (max o	2	87; 85	0.80725086	0.26258832		1.0
	ZINC04265921	-9.7	443.264	-4.8578	16	3	296.65	0.62771	248.5	-1.055	58.62105373403	0.31004	12.09	-15.668	0.46875	0.44712	0.90399 (max o	2	85; 86	0.78919184	0.25388536		1.0
	ZINC05433682	-9.7	444.514	-1.9882	11	3	327.64	0.34141	141.21	4.8256	58.62105373403	0.31004	9.2201	-6.4127	0.4375	0.45868	0.92889 (max o	4	1438; 1177; 599; 143	-9.395623E	0.25973144		1.0
	ZINC20410917	-9.7	442.339	-4.5649	13	3	292.04	0.53626	190.54	4.1605	58.62105373403	0.31004	11.797	-14.723	0.375	0.33728	0.95949 (max o	5	120; 119; 118; 95; 94	-0.8710487	-0.0494453		1.0
	ZINC20410917	-9.7	442.339	-4.5649	13	3	292.04	0.53626	190.54	3.9971	58.62105373403	0.31004	11.797	-14.723	0.375	0.33728	0.95709 (max o	5	120; 119; 118; 95; 93	-0.8898294	-0.0482166		1.0
	ZINC20410917	-9.7	443.347	-2.3953	13	4	290.75	0.5342	184.22	6.7509	58.62105373403	0.31004	9.6272	-7.7257	0.375	0.34338	0.95949 (max o	5	120; 119; 118; 93; 94	-0.8791724	-0.0288556		1.0
	ZINC49601377	-9.6	446.407	0.6221	10	5	308.75	0.38394	155.14	-3.0787	69.60001670705	0.30685	6.5353	2.0274	0.46875	0.3062	0.90632	1	204	-0.1829620	0.6261582		38.0
	ZINC05742780	-9.6	432.380	0.3464	10	6	292.84	0.41538	166.14	-3.2535	69.60001670705	0.31674	6.811	1.0936	0.51613	0.37768	0.96018 (max o	7	292; 271; 270; 269; 1	0.06674	0.6521799		38.0
	ZINC03947434	-9.6	448.379	7.0003e-4	11	7	299.19	0.45035	186.37	-3.2535	69.60001670705	0.30685	7.1567	0.0022814	0.5	0.37952	0.97319 (max o	13	616; 434; 371; 339; 3	0.09402009	0.6953303		38.0
	ZINC31155604	-9.6	448.379	-0.1534	11	7	297.4	0.45306	186.37	-3.7091	69.60001670705	0.30685	7.3108	-0.49993	0.5	0.3888	0.9642 (max of	5	529; 418; 404; 244; 1	0.12347066	0.6064182		38.0
	ZINC36046318	-9.6	445.355	-2.0792	11	5	298.01	0.45589	186.04	1.8339	69.60001670705	0.30685	9.2366	-6.776	0.46875	0.28824	0.95709 (max o	11	5353; 272; 183; 147;	-0.1338686	0.687772		15.0
	ZINC18202167	-9.6	443.431	-3.4078	10	5	292.46	0.42611	184.45	5.1912	69.60001670705	0.30685	10.565	-11.106	0.375	0.3646	0.94207 (max o	6	989; 389; 122; 22; 43	-0.1185781	-0.5692053		8.0
	ZINC18202167	-9.6	444.439	-4.7741	10	6	306.5	0.4524	185.65	2.9936	69.60001670705	0.30685	11.931	-15.559	0.375	0.36615	0.93948 (max o	2	1630; 121	0.07127746	-0.5407502		8.0
	ZINC21984166	-9.6	443.431	-3.4078	10	5	292.46	0.42611	184.45	5.1912	69.60001670705	0.30685	10.565	-11.106	0.375	0.3646	0.94207 (max o	7	989; 389; 315; 22; 43	-0.1583481	-0.5648868		6.0
	ZINC21984166	-9.6	444.439	-4.7741	10	6	306.5	0.4524	185.65	2.9936	69.60001670705	0.30685	11.931	-15.559	0.375	0.36615	0.94207 (max o	6	1630; 314; 21; 41; 74	0.07969697	-0.5621186		6.0
	ZINC05409528	-9.6	422.341	-0.4299	11	8	270.83	0.50895	197.37	-3.0467	69.60001670705	0.3273	7.5873	-1.3135	0.46667	0.34508	0.95579 (max o	9	1698; 1481; 589; 494	-0.0859806	-0.7201602		5.0
	ZINC11592618	-9.6	445.447	-2.6981	10	7	305.32	0.45028	182.82	2.96	69.60001670705	0.30685	9.8555	-8.793	0.375	0.36615	0.93948 (max o	3	1788; 769; 206	0.19107048	-0.8100250		5.0
	ZINC04081667	-9.6	378.376	0.0735	8	4	258.63	0.36902	125.68	-6.0454	69.60001670705	0.36367	7.0839	0.20211	0.48148	0.25864	0.94905 (max o	5	6710; 1685; 437; 193	0.29114795	0.4749525		4.0
	ZINC15958064	-9.6	424.475	-3.1866	8	2	300	0.3097	118.64	-1.065	69.60001670705	0.31674	10.344	-10.06	0.3871	0.28661	0.96262 (max o	5	1843; 1528; 796; 382	0.37810335	-0.2105218		4.0
	ZINC15967717	-9.6	446.482	-2.8941	8	2	319.48	0.29082	118.64	4.6249	69.60001670705	0.29755	10.051	-9.7265	0.42424	0.31005	0.96343 (max o	9	1241; 985; 798; 629;	0.17480953	-0.5502310		4.0
	ZINC18202167	-9.6	443.431	-6.8501	10	5	307.68	0.4545	188.48	2.9767	69.60001670705	0.30685	14.007	-22.324	0.375	0.36615	0.9497 (max of	3	991; 391; 124	-0.3148468	0.6154		3.0
	ZINC21984166	-9.6	443.431	-6.8501	10	5	307.68	0.4545	188.48	2.9767	69.60001670705	0.30685	14.007	-22.324	0.375	0.36615	0.9497 (max of	3	991; 391; 117	-0.2991564	0.6268726		3.0
	ZINC12604168	-9.6	436.506	0.2366	8	4	301.85	0.36959	153.55	6.1013	69.60001670705	0.3273	6.9208	0.72288	0.56667	0.40929	0.97975 (max o	3	2889; 281; 144	-0.5581871	0.3238286		3.0
	ZINC08624547	-9.6	429.471	0.1793	9	3	318.53	0.29809	115.48	5.9651	69.60001670705	0.31674	6.9781	0.56607	0.6129	0.51532	0.9499 (max of	6	1829; 1764; 1540; 46(	-0.0843385	-0.5956355		2.0
	ZINC35464245	-9.6	418.397	0.9358	9	5	285.85	0.37971	145.91	-9.613	69.60001670705	0.3273	6.2216	2.8591	0.46667	0.34658	0.95837 (max o	3	180; 127; 96	-0.9122334	-0.2072558		2.0
	ZINC03839839	-9.6	361.372	0.0626	7	2	249.57	0.28361	89.95	5.803	69.60001670705	0.37766	7.0948	0.16576	0.53846	0.36752	0.94927	1	1604	-0.4744374	-0.8070205		2.0
	ZINC35464248	-9.6	418.397	0.9358	9	5	285.85	0.37971	145.91	-9.613	69.60001670705	0.3273	6.2216	2.8591	0.46667	0.34361	0.95837 (max o	3	180; 96; 126	-0.8983825	-0.192562		2.0
	ZINC02122307	-9.6	384.407	-0.2613	7	0	281.6	0.27326	95.97	1.896	69.60001670705	0.35068	7.4187	-0.74512	0.53571	0.39761	0.89582 (max o	4	4012; 1650; 552; 187	0.13688569	0.18061773		1.0
	ZINC12603820	-9.6	431.467	-2.448	10	4	306.32	0.30997	129.62	5.889	69.60001670705	0.31674	9.6054	-7.7286	0.6129	0.5015	0.91599 (max o	6	1517; 1467; 1208; 95(	-0.0879581	-0.617145		1.0
	ZINC15958948	-9.6	448.410	-3.4796	10	2	299.96	0.37642	137.1	3.3033	69.60001670705	0.29755	10.637	-11.694	0.39394	0.24517	0.92099 (max o	5	503; 502; 383; 377; 1	0.25711134	-0.5376323		1.0
	ZINC18202167	-9.6	444.439	-4.6124	10	6	306.5	0.4524	185.65	2.151	69.60001670705	0.30685	11.77	-15.032	0.375	0.34344	0.94718 (max o	2	988; 388	-0.4867346	-0.5537026		1.0
	ZINC18202167	-9.6	443.431	-6.7785	10	5	307.68	0.4545	188.48	3.0017	69.60001670705	0.30685	13.936	-22.091	0.375	0.36615	0.9497 (max of	6	992; 990; 390; 316; 1	0.03878755	0.5895205		1.0
	ZINC20410920	-9.6	442.339	-4.5649	13	3	292.04	0.53626	190.54	4.1605	69.60001670705	0.30685	11.722	-14.877	0.375	0.33728	0.95949 (max o	5	120; 93; 94; 95; 119	-0.8997492	-0.0664670		1.0
	ZINC20410920	-9.6	442.339	-4.5649	13	3	292.04	0.53626	190.54	3.9971	69.60001670705	0.30685	11.722	-14.877	0.375	0.33728	0.95709 (max o	5	120; 93; 94; 95; 118	-0.8991037	-0.0289156		1.0
	ZINC20410920	-9.6	443.347	-2.3953	13	4	290.75	0.5342	184.22	6.7509	69.60001670705	0.30685	9.5527	-7.8062	0.375	0.34338	0.95949 (max o	5	93; 94; 95; 118; 119	-0.8799866	-0.0690455		1.0
	ZINC21984166	-9.6	443.431	-6.7785	10	5	307.68	0.4545	188.48	3.0017	69.60001670705	0.30685	13.936	-22.091	0.375	0.36615	0.9497 (max of	7	990; 392; 390; 316; 2	0.06162991	0.5619474		1.0

Structure	Molecule Name	affinity	Total Molweight	cLogP	H-Acceptors	H-Donors	Total Surface Area	Relative PSA	Polar Surface Area	Druglikeness	Kd (nM) @ 20°C	LE from K...	LLE from Kd ...	LELP from Kd (n...	Shape Index	Molecular...	Neighbor Si...	Neig...	Neighbor	Neighbor ...	Neighbor ...	Ring Systems	frequency
	ZINC40309138	-9.6	434.451	0.1677	9	3	307.97	0.29016	120.86	5.4378	69.60001670705	0.30685	6.9897	0.54653	0.46875	0.48334	0.95219	1	417	0.8462113	0.34240168		1.0
	ZINC12153135	-9.5	448.379	7.0003e-4	11	7	299.19	0.45035	186.37	-3.2535	82.63533232316	0.30365	7.0821	0.0023054	0.5	0.37908	0.9642 (max of	11	434; 371; 339; 338; 3	0.06158323	0.71396756		38.0
	ZINC04096258	-9.5	448.379	7.0003e-4	11	7	299.19	0.45035	186.37	-3.2535	82.63533232316	0.30365	7.0821	0.0023054	0.5	0.37908	0.9642 (max of	14	740; 658; 434; 371; 3	0.08410534	0.6631881		38.0
	ZINC40368514	-9.5	448.379	-0.1534	11	7	297.4	0.45306	186.37	-3.7091	82.63533232316	0.30365	7.2362	-0.50519	0.5	0.38924	0.9642 (max of	6	529; 418; 404; 244; 1	0.09045097	0.6226254		38.0
	ZINC03983877	-9.5	432.380	0.3464	10	6	292.84	0.41538	166.14	-3.2535	82.63533232316	0.31345	6.7364	1.1051	0.51613	0.37723	0.96739 (max o	8	1279; 292; 271; 270;	0.04603139	0.66136926		38.0
	ZINC22066481	-9.5	414.413	-4.4419	9	6	291.63	0.43188	166.41	1.137	82.63533232316	0.32389	11.525	-13.714	0.4	0.35656	0.97975 (max o	2	69; 165	8.8230276E	-0.5737533		22.0
	ZINC22066481	-9.5	413.405	-3.0744	9	4	277.2	0.40231	164.22	4.5417	82.63533232316	0.32389	10.157	-9.492	0.4	0.36092	0.9396 (max of	5	989; 389; 43; 49; 70	-0.0972699	-0.5611279		22.0
	ZINC22066481	-9.5	414.413	-4.4407	9	5	291.24	0.43112	165.42	2.1976	82.63533232316	0.32389	11.524	-13.71	0.4	0.36259	0.97975 (max o	5	1630; 41; 48; 69; 166	-0.0066746	-0.5389408		22.0
	ZINC28367529	-9.5	434.352	0.2551	11	7	284.9	0.47294	186.37	-3.8653	82.63533232316	0.31345	6.8277	0.81386	0.41935	0.41786	0.96262 (max o	15	4095; 2776; 2566; 25	-0.701104	0.27256554		21.0
	ZINC12376936	-9.5	429.356	-1.7335	10	4	291.66	0.4209	165.81	1.8339	82.63533232316	0.31345	8.8163	-5.5305	0.48387	0.37307	0.95771 (max o	5	289; 31; 39; 91; 128	-0.0974349	0.66343385		15.0
	ZINC36046319	-9.5	445.355	-2.0792	11	5	298.01	0.45589	186.04	1.8339	82.63533232316	0.30365	9.162	-6.8474	0.46875	0.28824	0.95709 (max o	11	5353; 289; 272; 2; 5;	-0.1579011	0.6407819		15.0
	ZINC31160047	-9.5	433.443	-3.2187	9	2	300.57	0.32129	125.37	6.2494	82.63533232316	0.30365	10.302	-10.6	0.40625	0.39447	0.95709 (max o	10	1026; 508; 317; 308;	-0.7740541	0.07068585		8.0
	ZINC04348951	-9.5	434.396	0.1664	10	6	294.39	0.41319	166.14	-3.7146	82.63533232316	0.31345	6.9164	0.53087	0.51613	0.39397	0.96739 (max o	6	757; 671; 479; 220; 2	0.08984411	-0.3708397		7.0
	ZINC15259622	-9.5	432.380	-0.7865	10	7	289.19	0.43134	177.14	-2.3989	82.63533232316	0.31345	7.8693	-2.5092	0.48387	0.39235	0.95771 (max o	7	5883; 2481; 2095; 12	0.64791316	0.17021419		7.0
	ZINC13551320	-9.5	444.439	-4.8782	10	5	307.48	0.45076	182.49	3.1697	82.63533232316	0.30365	11.961	-16.065	0.375	0.37716	0.9922 (max of	2	1313; 42	0.25366792	-0.2792876		6.0
	ZINC31160047	-9.5	434.451	-1.4164	9	2	296.85	0.31278	129.95	4.3792	82.63533232316	0.30365	8.4992	-4.6646	0.40625	0.39447	0.9665 (max of	7	1025; 507; 307; 305;	-0.7837335	-0.1532732		6.0
	ZINC13551320	-9.5	443.431	-6.9542	10	4	308.66	0.45286	185.32	2.9967	82.63533232316	0.30365	14.037	-22.902	0.375	0.37716	0.92074 (max o	5	1315; 1049; 170; 71;	0.18713336	-0.2821730		6.0
	ZINC22066481	-9.5	413.405	-6.6208	9	3	293.4	0.43177	165.09	1.6482	82.63533232316	0.32389	13.704	-20.441	0.4	0.37103	0.9396 (max of	4	1315; 1049; 71; 154	0.15802434	-0.3140945		6.0
	ZINC22066481	-9.5	413.405	-6.1228	9	3	293.4	0.43177	165.09	2.3072	82.63533232316	0.32389	13.206	-18.904	0.4	0.37103		0		-0.1866458	0.9647034		6.0
	ZINC13424732	-9.5	448.379	6.9979e-4	11	7	299.19	0.45035	186.37	-3.2535	82.63533232316	0.30365	7.0821	0.0023046	0.5	0.33722	0.95949 (max o	5	1158; 740; 739; 616;	0.10531137	0.6567408		6.0
	ZINC13551320	-9.5	445.447	-2.6405	10	6	306.3	0.44865	179.66	2.134	82.63533232316	0.30365	9.7233	-8.6959	0.375	0.35489	0.8838	1	150	0.19810188	-0.2613079		6.0
	ZINC13551320	-9.5	444.439	-4.7165	10	5	307.48	0.45076	182.49	2.0633	82.63533232316	0.30365	11.799	-15.533	0.375	0.35489	0.91522 (max o	5	1311; 1045; 154; 153	0.17759788	-0.2659756		6.0
	ZINC13551320	-9.5	443.431	-3.3502	10	4	293.44	0.42448	181.29	4.8292	82.63533232316	0.30365	10.433	-11.033	0.375	0.35331	0.92889 (max o	4	1314; 1048; 1047; 15	0.13852048	-0.2825792		6.0
	ZINC22066481	-9.5	413.405	-6.5167	9	4	292.42	0.43342	168.25	2.1801	82.63533232316	0.32389	13.6	-20.12	0.4	0.36259	0.9396 (max of	3	991; 391; 73	-0.3352335	0.6192231		5.0
	ZINC15958194	-9.5	418.428	-3.2471	8	2	291.96	0.31823	118.64	3.3221	82.63533232316	0.31345	10.33	-10.359	0.3871	0.25417	0.96018 (max o	6	629; 503; 502; 383; 3	0.24327826	-0.5221402		5.0
	ZINC15967720	-9.5	446.482	-2.8941	8	2	319.48	0.29082	118.64	4.6249	82.63533232316	0.29445	9.9769	-9.8289	0.42424	0.30771	0.9615 (max of	7	799; 798; 633; 384; 2	0.14162727	-0.5701855		4.0
	ZINC28381332	-9.5	367.460	-0.4817	6	4	276.3	0.28853	121.05	-4.8501	82.63533232316	0.37372	7.5645	-1.2889	0.46154	0.56771	0.96438 (max o	3	6671; 6670; 6042	0.51067805	0.81241256		4.0
	ZINC28537532	-9.5	434.396	0.1664	10	6	294.39	0.41319	166.14	-3.7146	82.63533232316	0.31345	6.9164	0.53087	0.51613	0.31919	0.96502 (max o	5	671; 409; 299; 234; 5	0.11460955	-0.3494271		4.0
	ZINC08636507	-9.5	432.543	0.4797	8	4	307.76	0.36249	153.55	7.4151	82.63533232316	0.32389	6.6031	1.481	0.56667	0.40929	0.97975 (max o	2	281; 109	-0.5769537	0.31490278		3.0
	ZINC14584317	-9.5	446.407	1.3578	10	4	293.2	0.38336	136.3	-4.5704	82.63533232316	0.30365	5.725	4.4716	0.53125	0.28539	0.9642 (max of	3	572; 492; 311	0.3275841	0.8578964		3.0
	ZINC22066481	-9.5	414.413	-4.279	9	5	291.24	0.43112	165.42	1.2872	82.63533232316	0.32389	11.362	-13.211	0.4	0.3372	0.90289 (max o	3	2182; 988; 388	-0.4507714	-0.5645438		3.0
	ZINC13736613	-9.5	425.416	-5.1512	9	4	303.6	0.41746	168.25	2.2711	82.63533232316	0.31345	12.234	-16.434	0.3871	0.31152		0		-0.4964316	-0.8587156		2.0
	ZINC15204505	-9.5	447.502	-0.3861	8	4	307.67	0.32411	139.51	0.54536	82.63533232316	0.30365	7.4689	-1.2715	0.5	0.32083	0.92619 (max o	3	372; 80; 149	-0.1156006	-0.5083467		2.0
	ZINC31166816	-9.5	418.397	0.9358	9	5	285.85	0.37971	145.91	-9.613	82.63533232316	0.32389	6.147	2.8892	0.46667	0.34361	0.96092 (max o	3	96; 126; 127	-0.927617	-0.1929833		2.0
	ZINC04044135	-9.5	419.492	-1.2533	7	3	284.29	0.31538	127.12	0.12303	82.63533232316	0.32389	8.3361	-3.8695	0.46667	0.36524	0.95318 (max o	4	2031; 1395; 891; 562	0.28993356	0.8699797		2.0
	ZINC04082083	-9.5	406.429	0.7575	8	4	286.15	0.33353	125.68	-8.3754	82.63533232316	0.33506	6.3253	2.2608	0.48276	0.31716	0.94842 (max o	6	2407; 1122; 437; 343	0.3452494	0.50724804		2.0
	ZINC08879908	-9.5	362.364	-1.3496	7	0	265.94	0.27078	93.11	4.914	82.63533232316	0.35988	8.4324	-3.7501	0.51852	0.50368	0.88492 (max o	2	470; 291	0.7401897	-0.3388402		2.0
	ZINC13538750	-9.5	445.486	-0.3946	8	3	306.57	0.32508	136.35	0.94088	82.63533232316	0.30365	7.4774	-1.2995	0.5	0.35715	0.92619 (max o	3	372; 195; 160	-0.1016571	-0.5417772		2.0
	ZINC13736613	-9.5	425.416	-5.6492	9	4	303.6	0.41746	168.25	1.5235	82.63533232316	0.31345	12.732	-18.023	0.3871	0.31152	0.88503	1	155	-0.0110563	0.9874728		2.0
	ZINC13736613	-9.5	426.424	-3.5732	9	5	302.42	0.41518	165.42	2.1905	82.63533232316	0.31345	10.656	-11.4	0.3871	0.31152	0.88503	1	157	0.01412452	0.9879391		2.0
	ZINC35270821	-9.5	360.361	-0.1543	7	4	232.58	0.41332	127.59	0.14074	82.63533232316	0.37372	7.2371	-0.41287	0.42308	0.29718	0.96721 (max o	3	661; 660; 518	-0.8347958	0.09996614		2.0
	ZINC35454973	-9.5	438.427	0.2591	10	5	296.68	0.39956	155.14	-11.09	82.63533232316	0.31345	6.8237	0.82662	0.48387	0.45655	0.96018 (max o	7	1895; 1307; 1284; 84	-0.2233550	0.8398168		2.0
	ZINC40312900	-9.5	363.376	-0.682	8	3	260.21	0.30871	115.29	3.7493	82.63533232316	0.35988	7.7648	-1.8951	0.48148	0.48337	0.93058 (max o	4	1329; 1330; 528; 243	-0.1687832	-0.4720410		2.0

Structure	Molecule Name	affinity	Total Molweight	cLogP	H-Acceptors	H-Donors	Total Surface Area	Relative PSA	Polar Surface Area	Druglikeness	Kd (nM) @ 20°C	LE from K...	LLE from Kd ...	LELP from Kd (n...	Shape Index	Molecular...	Neighbor Si...	Neig...	Neighbor	Neighbor ...	Neighbor ...	Ring Systems	frequency
	ZINC02118703	−9.5	384.407	−0.2632	7	0	283.1	0.27181	95.97	−0.41464	82.63533232316	0.34703	7.346	−0.75844	0.60714	0.49489	0.88492 (max o	3	873; 871; 567	0.1491672	0.12096002		1.0
	ZINC03839382	−9.5	440.498	−0.0222	9	2	314.86	0.29277	108.05	7.2786	82.63533232316	0.30365	7.105	−0.07311	0.5	0.37707	0.93422 (max o	4	3598; 1375; 700; 334	−0.5086793	−0.1492023		1.0
	ZINC04098667	−9.5	372.284	1.2493	9	5	228.94	0.48738	153.75	−0.41414	82.63533232316	0.35988	5.8335	3.4714	0.44444	0.12689		0		0.5611241	0.8181387		1.0
	ZINC04098675	−9.5	402.354	0.8343	9	5	268.46	0.41563	153.75	0.75167	82.63533232316	0.33506	6.2485	2.49	0.48276	0.27356		0		0.7449415	−0.4480414		1.0
	ZINC04236435	−9.5	401.442	−1.8631	9	2	292.31	0.32999	121.88	−2.5593	82.63533232316	0.33506	8.9459	−5.5605	0.58621	0.46021	0.90617 (max o	2	6140; 83	−0.4620743	−0.5276711		1.0
	ZINC04247581	−9.5	392.359	−1.4089	9	4	258.93	0.4307	150.59	−1.9479	82.63533232316	0.34703	8.4917	−4.0599	0.46429	0.40469	0.92774 (max o	7	1716; 1715; 727; 728	−0.3117989	0.65806425		1.0
	ZINC04247581	−9.5	392.359	−1.4757	9	5	258.48	0.43168	153.75	−1.9031	82.63533232316	0.34703	8.5585	−4.2524	0.46429	0.39644	0.92774 (max o	7	1716; 1715; 727; 728	−0.2706467	0.6750145		1.0
	ZINC05414097	−9.5	442.518	−0.4196	10	2	333.66	0.31205	124.6	0.97979	82.63533232316	0.30365	7.5024	−1.3819	0.4375	0.45868	0.971 (max of 5	5	3691; 1178; 1171; 75	−0.0372209	0.24563086		1.0
	ZINC13736613	−9.5	427.432	−2.2794	9	6	298.98	0.41601	162.59	2.0586	82.63533232316	0.31345	9.3622	−7.2721	0.3871	0.35333	0.80895	1	33	−0.5830389	0.79814637		1.0
	ZINC21981188	−9.5	433.499	−2.3091	8	4	314.42	0.33144	123.72	−4.3424	82.63533232316	0.31345	9.3919	−7.3668	0.45161	0.40888		0		−0.5185398	0.84621304		1.0
	ZINC22455022	−9.5	416.547	2.8303	6	2	307.85	0.11418	52.79	8.5224	82.63533232316	0.31345	4.2525	9.0296	0.51613	0.40399		0		−0.7553946	0.63637894		1.0
	ZINC22936720	−9.5	424.567	0.089	7	2	319.22	0.18561	74.19	6.9105	82.63533232316	0.31345	6.9938	0.28394	0.54839	0.39955	0.90529 (max o	2	1564; 1019	0.32210538	−0.8323855		1.0
	ZINC22936729	−9.5	439.534	−0.3998	8	3	325.2	0.24474	102.63	5.7148	82.63533232316	0.30365	7.4826	−1.3166	0.59375	0.40306	0.89473 (max o	4	3431; 2618; 1273; 31	−0.4694531	0.07305917		1.0
	ZINC40368512	−9.4	448.379	−0.1534	11	7	297.4	0.45306	186.37	−3.7091	98.11201880741	0.30045	7.1617	−0.51056	0.5	0.38924	0.95949 (max o	6	529; 418; 404; 269; 1	0.09778172	0.6007874		38.0
	ZINC05223934	−9.4	432.380	0.3464	10	6	292.84	0.41538	166.14	−3.2535	98.11201880741	0.31015	6.6619	1.1169	0.48387	0.29334	0.93952 (max o	10	5353; 272; 2; 5; 12; 1	−0.1608168	0.6229603		38.0
	ZINC02108177	−9.4	358.369	−0.5377	7	1	262.43	0.30503	106.97	4.9328	98.11201880741	0.36979	7.546	−1.4541	0.57692	0.54079	0.95818 (max o	4	875; 646; 331; 333	0.2747093	0.13948087		22.0
	ZINC03947501	−9.4	446.407	−0.0864	10	5	308.75	0.38394	155.14	−3.4398	98.11201880741	0.30045	7.0947	−0.28757	0.5625	0.40239	0.98532 (max o	12	2890; 1773; 888; 759	0.21835609	0.66728455		16.0
	ZINC12153136	−9.4	448.379	7.0003e−4	11	7	299.19	0.45035	186.37	−3.2535	98.11201880741	0.30045	7.0076	0.0023299	0.5	0.38195	0.96186 (max o	11	434; 371; 339; 338; 3	0.05951689	0.6919514		15.0
	ZINC04349525	−9.4	448.379	7.0003e−4	11	7	299.19	0.45035	186.37	−3.2535	98.11201880741	0.30045	7.0076	0.0023299	0.5	0.38151	0.9642 (max of	11	434; 371; 339; 338; 3	0.10513635	0.7110618		15.0
	ZINC08627131	−9.4	438.498	0.1422	9	4	323.91	0.28496	114.37	5.6241	98.11201880741	0.31015	6.8661	0.4585	0.64516	0.57443	0.94768 (max o	6	1463; 1191; 942; 770	−0.0478163	−0.6025803		11.0
	ZINC15675766	−9.4	435.361	−0.916	9	2	294.18	0.31936	129.29	−3.5031	98.11201880741	0.31015	7.9243	−2.9535	0.51613	0.47166	0.93111 (max o	8	1591; 1519; 982; 792	0.3235564	−0.1040456		10.0
	ZINC11592655	−9.4	445.447	−2.5378	10	7	306.15	0.44906	182.82	2.381	98.11201880741	0.30045	9.5461	−8.4466	0.375	0.36573	0.95949 (max o	3	607; 364; 286	0.9317119	0.29248065		8.0
	ZINC31160055	−9.4	433.443	−3.2187	9	2	300.57	0.32129	125.37	6.2494	98.11201880741	0.30045	10.227	−10.713	0.40625	0.39607	0.95709 (max o	11	1026; 508; 318; 317;	−0.7453559	0.0923902		8.0
	ZINC31165610	−9.4	449.442	−1.8156	10	2	305.76	0.33281	136.81	4.1377	98.11201880741	0.29135	8.8239	−6.2317	0.39394	0.38904	0.95884 (max o	9	1026; 508; 318; 317;	−0.7956681	0.11621021		8.0
	ZINC13424711	−9.4	434.396	0.1664	10	6	294.39	0.41319	166.14	−3.7146	98.11201880741	0.31015	6.8419	0.53652	0.51613	0.39226	0.96739 (max o	3	194; 200; 220	0.05956843	−0.3249621		8.0
	ZINC04097895	−9.4	434.396	0.1664	10	6	294.39	0.41319	166.14	−3.7146	98.11201880741	0.31015	6.8419	0.53652	0.51613	0.39182	0.96739 (max o	6	1037; 299; 234; 219;	0.05660924	−0.3473788		8.0
	ZINC04348946	−9.4	434.396	0.1664	10	6	294.39	0.41319	166.14	−3.7146	98.11201880741	0.31015	6.8419	0.53652	0.51613	0.39226	0.96739 (max o	5	954; 670; 219; 142; 1	0.07330228	−0.3582787		8.0
	ZINC13424713	−9.4	434.396	0.1664	10	6	294.39	0.41319	166.14	−3.7146	98.11201880741	0.31015	6.8419	0.53652	0.51613	0.39397	0.96739 (max o	4	1751; 53; 142; 219	0.09964032	−0.3308402		7.0
	ZINC05412536	−9.4	432.380	0.3509	10	6	288.14	0.43271	173.98	−4.2232	98.11201880741	0.31015	6.6574	1.1314	0.45161	0.25678	0.99629 (max o	7	1433; 712; 480; 370;	0.654876	0.03780828		7.0
	ZINC31160055	−9.4	434.451	−1.4164	9	2	296.85	0.31278	129.95	4.3792	98.11201880741	0.30045	8.4247	−4.7142	0.40625	0.39607	0.9665 (max of	7	1025; 507; 307; 305;	−0.7498969	−0.1531775		6.0
	ZINC11866709	−9.4	430.408	0.5905	9	4	302.4	0.34868	134.91	−5.7203	98.11201880741	0.31015	6.4178	1.9039	0.54839	0.40723	0.95771 (max o	3	685; 258; 59	0.25895092	0.54471207		6.0
	ZINC15449243	−9.4	422.341	−0.4299	11	8	270.83	0.50895	197.37	−3.0467	98.11201880741	0.32048	7.4382	−1.3414	0.46667	0.3445	0.95318 (max o	9	1698; 1481; 589; 494	−0.1064022	−0.7103465		5.0
	ZINC08034271	−9.4	445.447	−2.6981	10	7	305.32	0.45028	182.82	2.96	98.11201880741	0.30045	9.7064	−8.9801	0.375	0.36615	0.93948 (max o	3	1788; 769; 107	0.17229177	−0.820715		5.0
	ZINC04236437	−9.4	425.420	−1.7801	10	2	306.53	0.34731	131.11	1.8779	98.11201880741	0.31015	8.7884	−5.7396	0.54839	0.41299	0.9228 (max of	4	1139; 902; 55; 83	−0.4777062	−0.5119802		4.0
	ZINC04081716	−9.4	364.349	−0.2704	8	4	246.37	0.38738	125.68	−6.0856	98.11201880741	0.36979	7.2787	−0.73123	0.5	0.36444	0.9464 (max of	5	1685; 708; 564; 79; 1	0.31218383	0.43364787		4.0
	ZINC15958383	−9.4	424.475	−3.1866	8	2	300	0.3097	118.64	−1.065	98.11201880741	0.31015	10.195	−10.275	0.3871	0.28378	0.96018 (max o	5	1528; 796; 634; 382;	0.3613731	−0.2226583		4.0
	ZINC15968479	−9.4	446.482	−2.8941	8	2	319.48	0.29082	118.64	4.6249	98.11201880741	0.29135	9.9024	−9.9334	0.42424	0.30771	0.9615 (max of	8	1241; 799; 798; 636;	0.1643063	−0.6045471		4.0
	ZINC08877450	−9.4	445.447	−1.6423	10	2	328.88	0.36293	143.09	−4.6767	98.11201880741	0.30045	8.6506	−5.4661	0.5	0.45001	0.92876 (max o	3	1123; 764; 361	−0.0567337	0.15306474		4.0
	ZINC15249199	−9.4	413.365	−2.6382	11	3	291.2	0.44784	166.17	1.3595	98.11201880741	0.32048	9.6465	−8.2319	0.53333	0.39424	0.95053 (max o	8	1054; 789; 669; 668;	0.28792787	−0.7844505		4.0
	ZINC15957048	−9.4	424.475	−3.1847	8	2	298.5	0.31126	118.64	1.6256	98.11201880741	0.31015	10.193	−10.268	0.3871	0.21175	0.95521 (max o	5	1527; 795; 501; 500;	0.37610084	−0.5743724		4.0
	ZINC16032783	−9.4	432.455	−3.3242	8	2	305.72	0.30391	118.64	4.3827	98.11201880741	0.30045	10.332	−11.064	0.40625	0.29791	0.9642 (max of	9	1241; 985; 799; 635;	0.19742413	−0.5543616		4.0
	ZINC31165614	−9.4	449.442	−1.8156	10	2	305.76	0.33281	136.81	4.1377	98.11201880741	0.29135	8.8239	−6.2317	0.39394	0.38904	0.9565 (max of	7	1026; 508; 318; 317;	−0.7770401	0.10884685		4.0
	ZINC12605052	−9.4	372.424	0.1472	8	3	284.96	0.31436	103.53	5.0941	98.11201880741	0.35609	6.8611	0.41338	0.48148	0.50969	0.92721 (max o	2	959; 960	−0.2448808	0.21316142		3.0

Structure	Molecule Name	affinity	Total Molweight	cLogP	H-Acceptors	H-Donors	Total Surface Area	Relative PSA	Polar Surface Area	Druglikeness	Kd (nM) @ 20°C	LE from K...	LLE from Kd ...	LELP from Kd (n...	Shape Index	Molecular...	Neighbor Si...	Neig...	Neighbor	Neighbor ...	Neighbor ...	Ring Systems	frequency
	ZINC26745313	-9.4	422.385	0.1214	10	7	282.99	0.43005	169.3	-4.0605	98.11201880741	0.32048	6.8869	0.3788	0.53333	0.38308	0.96092 (max o	7	1937; 1332; 1306; 12	0.39420262	-0.8510125		3.0
	ZINC28537536	-9.4	434.396	0.1664	10	6	294.39	0.41319	166.14	-3.7146	98.11201880741	0.31015	6.8419	0.53652	0.51613	0.31724	0.96502 (max o	5	670; 299; 241; 177; 1	0.09441624	-0.3513538		3.0
	ZINC38629302	-9.4	413.365	-2.6382	11	3	291.2	0.44784	166.17	1.3595	98.11201880741	0.32048	9.6465	-8.2319	0.53333	0.40341	0.95053 (max o	8	1054; 789; 669; 668;	0.24876204	-0.8001573		3.0
	ZINC04099004	-9.4	445.486	-0.3946	8	3	306.57	0.32508	136.35	0.94088	98.11201880741	0.30045	7.4029	-1.3133	0.5	0.35906	0.92619 (max o	4	260; 216; 80; 149	-0.0836729	-0.5352533		2.0
	ZINC31156015	-9.4	438.427	0.2591	10	5	296.68	0.39956	155.14	-11.09	98.11201880741	0.31015	6.7492	0.83541	0.48387	0.45655	0.96018 (max o	7	1895; 1307; 1284; 84	-0.2032448	0.8345791		2.0
	ZINC04260141	-9.4	390.414	0.2632	8	1	279.8	0.25457	82.19	5.026	98.11201880741	0.34338	6.7451	0.76651	0.60714	0.52885	0.94558 (max o	4	2063; 735; 13; 35	-0.282228	0.12254736		2.0
	ZINC04557376	-9.4	418.397	0.5121	9	5	288.04	0.37682	145.91	-3.7146	98.11201880741	0.32048	6.4962	1.5979	0.5	0.31467	0.95837 (max o	2	352; 241	0.16169687	-0.3793567		2.0
	ZINC35456722	-9.4	434.396	0.1664	10	6	294.39	0.41319	166.14	-3.7146	98.11201880741	0.31015	6.8419	0.53652	0.51613	0.32055	0.96502 (max o	7	670; 460; 409; 368; 3	0.11843519	-0.3855730		2.0
	ZINC08254136	-9.4	441.554	-1.8355	9	2	345.64	0.25934	88.16	3.8969	98.11201880741	0.30045	8.8438	-6.1091	0.59375	0.48848	0.97533 (max o	3	2098; 1453; 358	0.82131344	-0.1882596		2.0
	ZINC31159166	-9.4	402.398	0.6231	8	5	291.78	0.33772	136.68	-3.3049	98.11201880741	0.33154	6.3852	1.8794	0.58621	0.44156	0.95372	1	1682	0.47803617	0.4817707		2.0
	ZINC40312903	-9.4	363.376	-0.682	8	3	260.21	0.30871	115.29	3.7493	98.11201880741	0.35609	7.6903	-1.9152	0.48148	0.48236	0.93058 (max o	4	2672; 1932; 1931; 18	-0.1304222	-0.4751981		2.0
	ZINC68590104	-9.4	366.328	-0.543	8	3	240.29	0.37413	120.19	4.9827	98.11201880741	0.35609	7.5513	-1.5249	0.44444	0.22205	0.93046	1	421	-0.6348801	0.45573962		2.0
	ZINC72325196	-9.4	414.460	1.168	9	0	303.56	0.25155	90.47	2.9669	98.11201880741	0.32048	5.8403	3.6445	0.5	0.50281	0.87588	1	537	0.8626309	0.303605		2.0
	ZINC02122088	-9.4	384.407	-0.2613	7	0	281.6	0.27326	95.97	1.896	98.11201880741	0.34338	7.2696	-0.76097	0.57143	0.39761	0.89582 (max o	5	1652; 872; 551; 341;	0.14614765	0.21558435		1.0
	ZINC02124244	-9.4	414.373	0.6292	11	1	276.02	0.37135	127.77	2.4319	98.11201880741	0.32048	6.3791	1.9633	0.36667	0.33381	0.95579	1	427	0.13513976	-0.843256		1.0
	ZINC03839355	-9.4	430.527	0.2564	8	2	302.3	0.3392	127.06	7.5275	98.11201880741	0.32048	6.7519	0.80004	0.53333	0.3719	0.934 (max of 7	7	4487; 3163; 2753; 16	-0.6597774	-0.0509837		1.0
	ZINC03840200	-9.4	420.464	-0.907	10	7	305.87	0.40625	160.02	2.4162	98.11201880741	0.32048	7.9153	-2.8301	0.56667	0.46473	0.91333 (max o	5	1861; 1373; 1106; 11	-0.4556212	-0.3660047		1.0
	ZINC03844921	-9.4	358.309	-0.917	10	4	251.12	0.49411	150.54	3.6929	98.11201880741	0.36979	7.9253	-2.4798	0.57692	0.58541		0		0.59382313	0.80269307		1.0
	ZINC04150053	-9.4	446.459	-2.0874	10	1	305.9	0.36136	144.53	2.6871	98.11201880741	0.31015	9.0957	-6.7304	0.58065	0.48837	0.86981 (max o	2	1410; 1139	-0.4895526	-0.4765244		1.0
	ZINC04270879	-9.4	382.394	0.7416	7	1	276.48	0.2483	82.61	5.8593	98.11201880741	0.34338	6.2667	2.1597	0.60714	0.48018	0.8715 (max of	4	448; 447; 445; 268	-0.2970787	-0.0917154		1.0
	ZINC04349530	-9.4	436.368	-0.5213	11	7	286.98	0.46951	186.37	-3.8607	98.11201880741	0.31015	7.5296	-1.6808	0.51613	0.28482	0.90345 (max o	2	1816; 1624	-0.3036329	-0.6421547		1.0
	ZINC08917758	-9.4	427.432	-1.4661	9	1	312.31	0.32484	125.07	2.2741	98.11201880741	0.31015	8.4744	-4.7271	0.58065	0.51671	0.88201 (max o	3	3583; 1084; 567	0.1568396	0.05520302		1.0
	ZINC08918577	-9.4	436.431	2.1272	12	5	310.51	0.44536	176.82	1.3234	98.11201880741	0.30045	4.8811	7.08	0.46875	0.28991	0.92074 (max o	3	1201; 285; 212	-0.1661632	-0.7313402		1.0
	ZINC08964893	-9.4	424.420	1.8506	12	6	301.32	0.46897	179.72	1.6836	98.11201880741	0.31015	5.1577	5.9669	0.48387	0.27267	0.86981 (max o	4	5040; 1201; 285; 211	-0.1270854	-0.7384575		1.0
	ZINC12496781	-9.4	447.502	-0.5383	8	4	307.67	0.32411	139.51	1.3464	98.11201880741	0.30045	7.5466	-1.7916	0.5	0.36163	0.95949 (max o	4	372; 260; 80; 195	-0.0776197	-0.5146662		1.0
	ZINC12605024	-9.4	428.491	-0.714	10	4	320.8	0.33791	132.79	7.6073	98.11201880741	0.31015	7.7223	-2.3021	0.51613	0.5389	0.88226 (max o	3	959; 960; 13	-0.2803599	0.20008206		1.0
	ZINC15707341	-9.4	411.373	-1.6529	11	2	284.98	0.39985	147.75	3.692	98.11201880741	0.32048	8.6612	-5.1575	0.5	0.4109	0.90725 (max o	9	1591; 1519; 982; 792	0.36448786	-0.0873234		1.0
	ZINC15707341	-9.4	410.365	-1.464	11	1	295.95	0.4221	146.5	4.1935	98.11201880741	0.32048	8.4723	-4.5681	0.5	0.4109	0.97533 (max o	5	1520; 793; 643; 226;	0.3728207	-0.1243404		1.0
	ZINC15707341	-9.4	410.365	-1.464	11	1	295.95	0.4221	146.5	4.1935	98.11201880741	0.32048	8.4723	-4.5681	0.5	0.4109	0.97533 (max o	5	1521; 794; 644; 224;	0.3530842	-0.1291589		1.0
	ZINC20111604	-9.4	355.456	1.8389	5	1	252.75	0.12443	44.94	1.4431	98.11201880741	0.36979	5.1694	4.9728	0.5	0.4365		0		-0.6308022	-0.7673304		1.0
	ZINC20219093	-9.4	361.309	-1.8968	9	3	238.15	0.49549	161.08	0.61354	98.11201880741	0.38458	8.9051	-4.9321	0.52	0.50415		0		0.34753722	0.88813907		1.0
	ZINC04349021	-9.3	432.380	0.3464	10	6	292.84	0.41538	166.14	-3.2535	116.4871272894	0.30685	6.5873	1.1289	0.51613	0.37768	0.96739 (max o	6	292; 40; 105; 133; 27	0.03242596	0.6306052		38.0
	ZINC04349019	-9.3	432.380	0.3464	10	6	292.84	0.41538	166.14	-3.2535	116.4871272894	0.30685	6.5873	1.1289	0.51613	0.37768	0.96018 (max o	7	339; 292; 105; 133; 2	0.07025029	0.6323726		38.0
	ZINC04349022	-9.3	432.380	0.3464	10	6	292.84	0.41538	166.14	-3.2535	116.4871272894	0.30685	6.5873	1.1289	0.51613	0.37723	0.96018 (max o	6	350; 292; 105; 133; 2	0.04930640	0.64119893		38.0
	ZINC13424715	-9.3	432.380	0.3464	10	6	292.84	0.41538	166.14	-3.2535	116.4871272894	0.30685	6.5873	1.1289	0.51613	0.37723	0.96018 (max o	6	371; 105; 133; 269; 2	0.02834083	0.6513423		38.0
	ZINC04349042	-9.3	445.355	-2.0792	11	5	298.01	0.45589	186.04	1.8339	116.4871272894	0.29726	9.0129	-6.9946	0.46875	0.28742	0.95949 (max o	12	5353; 2; 5; 12; 18; 26	-0.1560081	0.65957177		38.0
	ZINC13424724	-9.3	400.382	1.0378	8	4	280.14	0.34069	125.68	-3.2535	116.4871272894	0.32801	5.8959	3.1639	0.51724	0.37921	0.9617 (max of	6	1228; 738; 737; 489;	0.30934897	0.5696362		38.0
	ZINC08964851	-9.3	430.380	0.7192	14	5	300.82	0.52437	209.75	-3.636	116.4871272894	0.30685	6.2145	2.3438	0.48387	0.35511	0.89402 (max o	6	5040; 3314; 1201; 76	-0.145532	-0.7265049		24.0
	ZINC35051264	-9.3	443.431	-3.4078	10	5	292.46	0.42611	184.45	5.1912	116.4871272894	0.29726	10.342	-11.464	0.375	0.3646	0.9922 (max of	6	989; 389; 22; 43; 75;	-0.1592029	-0.5849492		22.0
	ZINC35051264	-9.3	444.439	-4.7741	10	6	306.5	0.4524	185.65	2.9936	116.4871272894	0.29726	11.708	-16.06	0.375	0.36615	0.9922 (max of	5	1630; 21; 41; 74; 121	0.06392185	-0.5787641		22.0
	ZINC04404499	-9.3	432.380	-0.3621	10	6	292.84	0.41538	166.14	-3.6206	116.4871272894	0.30685	7.2958	-1.1801	0.54839	0.39018	0.95983 (max o	10	973; 759; 742; 741; 6	0.19692035	0.6362935		16.0
	ZINC04404500	-9.3	432.380	-0.3621	10	6	292.84	0.41538	166.14	-3.6206	116.4871272894	0.30685	7.2958	-1.1801	0.54839	0.39018	0.96262 (max o	9	2890; 973; 888; 741;	0.18406248	0.6528731		16.0
	ZINC12376938	-9.3	429.356	-1.7335	10	4	291.66	0.4209	165.81	1.8339	116.4871272894	0.30685	8.6672	-5.6494	0.48387	0.37307	0.95771 (max o	5	31; 39; 91; 147; 183	-0.0993579	0.6433738		15.0

Structure	Molecule Name	affinity	Total Molweight	cLogP	H-Acceptors	H-Donors	Total Surface Area	Relative PSA	Polar Surface Area	Druglikeness	Kd (nM) @ 20°C	LE from K...	LLE from Kd ...	LELP from Kd (n...	Shape Index	Molecular...	Neighbor Si...	Neig...	Neighbor	Neighbor ...	Neighbor ...	Ring Systems	frequency
	ZINC13424728	-9.3	432.380	-0.078	10	7	289.19	0.43134	177.14	-1.9977	116.4871272894	0.30685	7.0117	-0.2542	0.51613	0.3816	0.97642 (max o	12	3788; 3213; 2828; 25	0.71741396	0.11108186		14.0
	ZINC20758328	-9.3	435.415	-1.8242	10	4	314.31	0.38179	154.66	6.5169	116.4871272894	0.29726	8.7579	-6.1368	0.40625	0.46605	0.9642 (max of	10	2540; 2161; 1877; 15	0.707602	-0.1599138		14.0
	ZINC12888204	-9.3	419.416	-0.8975	9	3	306.46	0.34882	134.43	4.8339	116.4871272894	0.30685	7.8312	-2.9249	0.41935	0.45789	0.96971 (max o	12	2540; 2534; 2161; 18	0.6889358	-0.1682275		14.0
	ZINC02097966	-9.3	372.396	-0.1515	7	0	279.84	0.27498	95.97	3.8967	116.4871272894	0.35231	7.0852	-0.43003	0.62963	0.54449	0.95818 (max o	5	1480; 876; 605; 424;	0.3370449	0.14666483		11.0
	ZINC31163529	-9.3	419.416	-3.5167	9	2	290.4	0.33254	125.37	6.2316	116.4871272894	0.30685	10.45	-11.461	0.41935	0.39495	0.96018 (max o	6	47; 98; 179; 238; 239;	-0.7655390	0.09080768		8.0
	ZINC31163525	-9.3	419.416	-3.5167	9	2	290.4	0.33254	125.37	6.2316	116.4871272894	0.30685	10.45	-11.461	0.41935	0.39495	0.96018 (max o	7	1026; 47; 98; 179; 23	-0.7948983	0.07154481		8.0
	ZINC35270974	-9.3	449.442	-1.8156	10	2	305.76	0.33281	136.81	4.1377	116.4871272894	0.28825	8.7493	-6.2987	0.39394	0.39061	0.95884 (max o	9	1026; 508; 47; 98; 31	-0.783717	0.08897740		8.0
	ZINC33832983	-9.3	418.397	0.5121	9	5	288.04	0.37682	145.91	-3.7146	116.4871272894	0.31707	6.4216	1.6151	0.53333	0.39754	0.9707 (max of	7	6202; 1058; 1037; 97	0.05475250	-0.3697703		7.0
	ZINC43743261	-9.3	412.821	0.6776	8	4	275.55	0.34636	125.68	-8.307	116.4871272894	0.33972	6.2561	1.9946	0.5	0.30543	0.93686 (max o	2	710; 568	0.38894048	0.4597487		7.0
	ZINC04028953	-9.3	419.429	0.082	9	4	295.25	0.36186	134.55	-7.1226	116.4871272894	0.31707	6.8517	0.25861	0.46667	0.40837	0.89657 (max o	3	1401; 563; 79	0.345466	0.41551504		7.0
	ZINC12375030	-9.3	432.380	-0.2446	10	6	289.64	0.43047	173.98	-4.2658	116.4871272894	0.30685	7.1783	-0.79714	0.48387	0.28023	0.95771 (max o	4	1486; 367; 60; 61	0.66426516	-0.0052316		7.0
	ZINC04236407	-9.3	436.443	-0.7953	9	1	320.17	0.29672	119.08	5.088	116.4871272894	0.29726	7.729	-2.6755	0.59375	0.42754	0.93139 (max o	3	901; 353; 82	-0.2040799	0.3055101		7.0
	ZINC06143120	-9.3	432.380	-0.3621	10	6	292.84	0.41538	166.14	-3.6206	116.4871272894	0.30685	7.2958	-1.1801	0.54839	0.43127	0.96018 (max o	12	3558; 2890; 2847; 21	0.18957959	0.6963052		7.0
	ZINC31163529	-9.3	420.424	-1.7144	9	2	286.68	0.32388	129.95	4.3355	116.4871272894	0.30685	8.6481	-5.5872	0.41935	0.39495	0.96502 (max o	5	46; 97; 178; 237; 305	-0.7491875	-0.175762		6.0
	ZINC31163525	-9.3	420.424	-1.7144	9	2	286.68	0.32388	129.95	4.3355	116.4871272894	0.30685	8.6481	-5.5872	0.41935	0.39495	0.96262 (max o	5	307; 46; 97; 178; 237	-0.7664454	-0.1649300		6.0
	ZINC04073618	-9.3	400.382	0.6605	8	4	280.14	0.34069	125.68	-5.9015	116.4871272894	0.32801	6.2732	2.0137	0.51724	0.39148	0.95372 (max o	3	565; 258; 59	0.2862146	0.51149625		6.0
	ZINC04081776	-9.3	400.382	0.6605	8	4	280.14	0.34069	125.68	-5.9015	116.4871272894	0.32801	6.2732	2.0137	0.51724	0.39148	0.95372 (max o	5	2034; 1128; 214; 256;	0.26383537	0.50924945		6.0
	ZINC04074124	-9.3	414.409	1.0044	8	4	292.4	0.3264	125.68	-5.8596	116.4871272894	0.31707	5.9293	3.1677	0.5	0.3091	0.95579 (max o	4	2407; 1400; 565; 258	0.30816126	0.51592875		6.0
	ZINC11592657	-9.3	445.447	-2.5378	10	7	306.15	0.44906	182.82	2.381	116.4871272894	0.29726	9.4715	-8.5374	0.375	0.36573	0.95949 (max o	3	607; 364; 213	0.9155432	0.32063475		6.0
	ZINC35051264	-9.3	444.439	-4.8794	10	6	307.87	0.45146	183.48	2.2886	116.4871272894	0.29726	11.813	-16.415	0.375	0.37166		0		-0.5313771	0.8447378		6.0
	ZINC05409527	-9.3	422.341	-0.4299	11	8	270.83	0.50895	197.37	-3.0467	116.4871272894	0.31707	7.3636	-1.3558	0.46667	0.34508	0.95837 (max o	9	1698; 1481; 589; 494;	-0.0940767	-0.6961299		6.0
	ZINC08951913	-9.3	422.341	-0.4299	11	8	270.83	0.50895	197.37	-3.0467	116.4871272894	0.31707	7.3636	-1.3558	0.46667	0.3445	0.95837 (max o	9	1698; 1481; 589; 494;	-0.1134534	-0.6884479		5.0
	ZINC05409525	-9.3	422.341	-0.4299	11	8	270.83	0.50895	197.37	-3.0467	116.4871272894	0.31707	7.3636	-1.3558	0.46667	0.34508	0.95837 (max o	9	1698; 1481; 589; 494;	-0.0751840	-0.7018102		5.0
	ZINC04259166	-9.3	439.447	-1.8026	10	2	321.79	0.33084	131.11	4.3084	116.4871272894	0.29726	8.7363	-6.0641	0.625	0.45375	0.96877 (max o	5	4973; 2435; 1411; 90	-0.5102475	-0.482411		4.0
	ZINC15958381	-9.3	424.475	-3.1866	8	2	300	0.3097	118.64	-1.065	116.4871272894	0.30685	10.12	-10.385	0.3871	0.28661	0.96262 (max o	5	1843; 1528; 634; 110	0.36519894	-0.2429580		4.0
	ZINC15968475	-9.3	446.482	-2.8941	8	2	319.48	0.29082	118.64	4.6249	116.4871272894	0.28825	9.8278	-10.04	0.42424	0.31005	0.96343 (max o	8	1241; 799; 635; 384;	0.21385895	-0.5686810		4.0
	ZINC15249194	-9.3	413.365	-2.6382	11	3	291.2	0.44784	166.17	1.3595	116.4871272894	0.31707	9.5719	-8.3204	0.53333	0.39424	0.95053 (max o	8	1054; 789; 669; 668;	0.23050421	-0.7901197		4.0
	ZINC35270978	-9.3	449.442	-1.8156	10	2	305.76	0.33281	136.81	4.1377	116.4871272894	0.28825	8.7493	-6.2987	0.39394	0.39061	0.9565 (max of	6	1026; 508; 238; 239;	-0.7685833	0.12818472		4.0
	ZINC31158951	-9.3	360.361	0.1524	7	5	235.62	0.38868	135.29	-0.37577	116.4871272894	0.36586	6.7813	0.41656	0.42308	0.30051	0.9464 (max of	3	1898; 1024; 406	0.8140854	-0.5023244		4.0
	ZINC35051264	-9.3	443.431	-6.7785	10	5	307.68	0.4545	188.48	3.0017	116.4871272894	0.29726	13.712	-22.803	0.375	0.36615	0.95219 (max o	8	990; 392; 390; 23; 44	0.05331261	0.6209972		3.0
	ZINC08636505	-9.3	448.542	0.0658	9	4	317.76	0.38255	162.78	7.4774	116.4871272894	0.30685	6.8679	0.21444	0.58065	0.42191	0.94768 (max o	3	6182; 109; 144	-0.5527908	0.30456898		3.0
	ZINC16951757	-9.3	448.423	0.4421	10	5	310.3	0.38202	155.14	-3.5326	116.4871272894	0.29726	6.4916	1.4873	0.5	0.32641	0.95983 (max o	3	177; 194; 234	0.08166336	-0.3187572		3.0
	ZINC05413790	-9.3	393.486	0.025	9	3	300.1	0.31203	110.85	1.8016	116.4871272894	0.33972	6.9087	0.07359	0.53571	0.5476	0.95123 (max o	3	2471; 1544; 481	-0.1537603	0.27321753		3.0
	ZINC12895176	-9.3	422.416	-1.4896	9	0	310.46	0.29637	111.57	4.9237	116.4871272894	0.30685	8.4233	-4.8545	0.51613	0.4765	0.90345 (max o	2	952; 145	0.7597153	-0.3234539		2.0
	ZINC01540998	-9.3	425.400	-4.0454	11	4	317.1	0.45541	192.63	4.4441	116.4871272894	0.30685	10.979	-13.184	0.6129	0.59892	0.98065	1	1640	0.87673926	0.19571729		2.0
	ZINC03839292	-9.3	426.471	-0.0203	9	2	301.1	0.30614	108.05	7.2589	116.4871272894	0.30685	6.954	-0.066157	0.54839	0.36987	0.95053 (max o	6	1376; 701; 700; 429;	-0.4617027	-0.2028216		2.0
	ZINC04083220	-9.3	376.404	-0.0617	7	3	240.04	0.35569	113.29	-3.0006	116.4871272894	0.35231	6.9954	-0.17513	0.37037	0.32456	0.97407 (max o	3	2460; 2410; 1162	0.6465411	0.6793216		2.0
	ZINC04096817	-9.3	447.502	-0.5383	8	4	307.67	0.32411	139.51	1.3464	116.4871272894	0.29726	7.472	-1.8109	0.5	0.35906	0.95949 (max o	4	372; 80; 195; 216	-0.0645461	-0.5346366		2.0
	ZINC08627144	-9.3	445.513	-1.1626	8	3	326.24	0.23005	102.74	3.3247	116.4871272894	0.29726	8.0963	-3.9111	0.65625	0.48683	0.9499 (max of	3	3290; 1193; 467	0.08667534	-0.5817267		2.0
	ZINC08964851	-9.3	430.380	-2.2887	14	5	278.39	0.48604	198.97	-5.5004	116.4871272894	0.30685	9.2224	-7.4588	0.48387	0.39046	0.89699	1	3315	-0.9593714	0.11133226		2.0
	ZINC14806751	-9.3	447.371	-2.2592	11	5	299.56	0.45353	186.04	1.3842	116.4871272894	0.29726	9.1929	-7.6001	0.5	0.42452		0		0.8162141	-0.2424245		2.0
	ZINC20464512	-9.3	440.498	-1.9881	9	2	323.6	0.26109	119.91	4.0407	116.4871272894	0.29726	8.9218	-6.6881	0.5625	0.53001	0.96224 (max o	4	5059; 4686; 498; 301	0.22439633	-0.1856248		2.0
	ZINC20464512	-9.3	439.490	-1.7992	9	1	334.57	0.28532	118.66	4.6065	116.4871272894	0.29726	8.7329	-6.0527	0.5625	0.53001	0.96224 (max o	4	5060; 4687; 499; 300	0.24129708	-0.2228819		2.0

Structure	Molecule Name	affinity	Total Molweight	cLogP	H-Acceptors	H-Donors	Total Surface Area	Relative PSA	Polar Surface Area	Druglikeness	Kd (nM) @ 20°C	LE from K...	LLE from Kd ...	LELP from Kd (n...	Shape Index	Molecular...	Neighbor Si...	Neig...	Neighbor	Neighbor ...	Neighbor ...	Ring Systems	frequency
	ZINC31170339	−9.3	443.467	−3.2526	10	5	300.11	0.40925	168.97	−2.3795	116.4871272894	0.30685	10.186	−10.6	0.48387	0.43159	0.96018 (max o	3	1034; 1033; 1032	−0.7594119	−0.4700103		2.0
	ZINC35466134	−9.3	434.396	0.1664	10	6	294.39	0.41319	166.14	−3.7146	116.4871272894	0.30685	6.7673	0.54229	0.51613	0.40113	0.96739 (max o	5	1223; 674; 524; 523;	0.09012155	−0.4100928		2.0
	ZINC67913907	−9.3	406.386	0.3752	9	6	276.64	0.39257	149.07	−1.9967	116.4871272894	0.32801	6.5585	1.1439	0.55172	0.34267	0.95099 (max o	3	855; 854; 533	−0.8292516	0.37885687		2.0
	ZINC01020739	−9.3	392.414	−0.7574	8	0	284.02	0.23365	81.24	4.2065	116.4871272894	0.32801	7.6911	−2.3091	0.51724	0.52201		0		−0.8558883	0.24306424		1.0
	ZINC03839294	−9.3	402.474	−0.0837	8	2	274.78	0.37317	127.06	8.8522	116.4871272894	0.33972	7.0174	−0.24638	0.5	0.30664	0.93091 (max o	5	4039; 1669; 1668; 87	−0.631173	−0.0598349		1.0
	ZINC03839345	−9.3	440.498	0.3217	9	2	314.86	0.29277	108.05	5.9909	116.4871272894	0.29726	6.612	1.0822	0.5625	0.36528	0.9313 (max of	4	2374; 1376; 429; 250	−0.4391058	−0.2307693		1.0
	ZINC03839348	−9.3	416.501	0.2583	8	2	288.54	0.35538	127.06	7.6017	116.4871272894	0.32801	6.6754	0.78748	0.51724	0.30716	0.934 (max of 5	5	4487; 4039; 702; 189;	−0.6422747	−0.0393651		1.0
	ZINC04042301	−9.3	406.349	−2.3166	9	1	289.83	0.37353	142.66	4.3395	116.4871272894	0.31707	9.2503	−7.3062	0.5	0.44434	0.81782	1	2780	0.6448857	0.71818346		1.0
	ZINC04236051	−9.3	409.421	−2.6657	9	2	286.86	0.32127	115.82	4.7076	116.4871272894	0.31707	9.5994	−8.4072	0.53333	0.33669	0.91333 (max o	6	901; 723; 573; 359; 3	−0.2583746	0.25295043		1.0
	ZINC04247590	−9.3	392.359	−1.4089	9	4	258.93	0.4307	150.59	−1.9479	116.4871272894	0.33972	8.3426	−4.1472	0.46429	0.40469	0.92464 (max o	7	1716; 1715; 727; 728;	−0.2872623	0.68726677		1.0
	ZINC04247590	−9.3	392.359	−1.4757	9	5	258.48	0.43168	153.75	−1.9031	116.4871272894	0.33972	8.4094	−4.3438	0.46429	0.39644	0.92464 (max o	8	1716; 1715; 727; 728;	−0.2958410	0.64645755		1.0
	ZINC04270601	−9.3	414.484	0.7054	9	1	298.58	0.29312	97.13	−4.7722	116.4871272894	0.31707	6.2283	2.2247	0.56667	0.33099	0.8973 (max of	3	1151; 734; 267	0.03487692	−0.2830178		1.0
	ZINC04270607	−9.3	398.398	−0.4382	10	1	280.23	0.36267	110.27	0.23167	116.4871272894	0.32801	7.3719	−1.3359	0.55172	0.29359	0.91248 (max o	6	1718; 1152; 1151; 73	0.02621291	−0.2641395		1.0
	ZINC04270923	−9.3	384.410	0.6518	7	1	279.18	0.23121	74.65	7.2258	116.4871272894	0.33972	6.2819	1.9186	0.57143	0.4412	0.85346 (max o	2	1421; 199	−0.2745469	−0.0363517		1.0
	ZINC05434145	−9.3	436.471	−0.3199	10	2	325.62	0.31976	124.6	5.0549	116.4871272894	0.29726	7.2536	−1.0762	0.4375	0.46026	0.99646 (max o	6	4133; 2929; 2572; 24	−0.7280833	−0.4074776		1.0
	ZINC08789747	−9.3	445.454	−0.2809	9	2	320.82	0.30855	125.64	6.7145	116.4871272894	0.28825	7.2146	−0.9745	0.39394	0.43789	0.90399 (max o	7	2534; 1877; 1654; 13	0.7297727	−0.1114755		1.0
	ZINC12296606	−9.3	423.400	−1.9745	9	2	317.96	0.34394	133.86	−4.1588	116.4871272894	0.30685	8.9082	−6.4348	0.51613	0.46197	0.90581 (max o	2	2545; 775	0.18023585	−0.1444732		1.0
	ZINC30878826	−9.3	432.539	−1.9908	8	2	331.62	0.25291	83.75	3.0876	116.4871272894	0.30685	8.9245	−6.4879	0.54839	0.52473		0		0.8307449	−0.5101468		1.0
	ZINC31164812	−9.3	427.405	−1.9897	11	7	287.72	0.46083	174.09	1.0959	116.4871272894	0.31707	8.9234	−6.2752	0.53333	0.34322	0.88128 (max o	2	1580; 825	−0.3681837	0.47587836		1.0
	ZINC33832362	−9.3	446.407	1.3578	10	4	293.2	0.38336	136.3	−4.5704	116.4871272894	0.29726	5.5759	4.5678	0.53125	0.28539	0.91522 (max o	2	572; 159	0.33766454	0.8736299		1.0
	ZINC35442259	−9.3	420.535	0.0079002	7	2	314.16	0.20165	82.15	4.8603	116.4871272894	0.30685	6.9258	0.025747	0.58065	0.39562	0.89025 (max o	4	1911; 1597; 1595; 17	−0.4523977	0.03986719		1.0
	ZINC72320088	−9.3	424.444	1.0699	9	4	304.63	0.3561	142.75	−12.085	116.4871272894	0.31707	5.8638	3.3743	0.46667	0.4082	0.92257	1	1344	0.5911855	0.5330214		1.0
	ZINC77257258	−9.3	448.507	−1.292	10	6	304.26	0.3898	158.3	−2.7899	116.4871272894	0.30685	8.2257	−4.2106	0.51613	0.47116	0.97856	1	538	−0.5953066	−0.6620864		1.0
	ZINC13424733	−9.2	448.379	7.0003e−4	11	7	299.19	0.45035	186.37	−3.2535	138.3038816707	0.29406	6.8585	0.0023805	0.5	0.37908	0.96186 (max o	13	434; 89; 90; 102; 136	0.0631706	0.6718548		38.0
	ZINC03947432	−9.2	448.379	7.0003e−4	11	7	299.19	0.45035	186.37	−3.2535	138.3038816707	0.29406	6.8585	0.0023805	0.5	0.37952	0.96186 (max o	13	739; 434; 371; 89; 90	0.09962202	0.67569464		38.0
	ZINC04349523	−9.2	448.379	7.0003e−4	11	7	299.19	0.45035	186.37	−3.2535	138.3038816707	0.29406	6.8585	0.0023805	0.5	0.37908	0.96186 (max o	13	1158; 434; 371; 89; 9	0.07936724	0.68158424		38.0
	ZINC35466146	−9.2	448.379	7.0003e−4	11	7	299.19	0.45035	186.37	−3.2535	138.3038816707	0.29406	6.8585	0.0023805	0.5	0.3059	0.95709 (max o	3	675; 414; 412	−0.7814846	−0.2616306		38.0
	ZINC08381630	−9.2	400.382	1.0378	8	4	280.14	0.34069	125.68	−3.2535	138.3038816707	0.32448	5.8214	3.1983	0.51724	0.3797	0.9617 (max of	7	1228; 738; 489; 459;	0.28874442	0.5659802		38.0
	ZINC02112482	−9.2	429.448	−1.4081	9	2	321.21	0.34046	133.86	1.6567	138.3038816707	0.30355	8.2673	−4.6388	0.51613	0.463	0.91413 (max o	6	1989; 1096; 1083; 87	0.13552177	0.01712785		24.0
	ZINC18210358	−9.2	443.431	−3.4078	10	5	292.46	0.42611	184.45	5.1912	138.3038816707	0.29406	10.267	−11.589	0.375	0.3646	0.9922 (max of	8	989; 22; 43; 75; 115;	−0.1390356	−0.5769127		22.0
	ZINC02118733	−9.2	378.787	−0.2756	7	1	265.59	0.3014	106.97	4.9921	138.3038816707	0.36192	7.1348	−0.76149	0.57692	0.54079	0.89608 (max o	6	2729; 1121; 963; 876	0.2957842	0.14067085		22.0
	ZINC05667874	−9.2	400.382	0.3293	8	4	280.14	0.34069	125.68	−3.6206	138.3038816707	0.32448	6.5299	1.0149	0.55172	0.39121	0.95372 (max o	6	1182; 1128; 610; 595	0.24688734	0.5610664		16.0
	ZINC12374720	−9.2	416.381	−0.0164	9	5	286.49	0.37886	145.91	−3.6206	138.3038816707	0.31367	6.8756	−0.052285	0.56667	0.39445	0.95837 (max o	9	1230; 1182; 977; 955	0.21207823	0.6064991		16.0
	ZINC08844371	−9.2	430.408	0.2593	9	4	302.4	0.34868	134.91	−3.4398	138.3038816707	0.30355	6.5999	0.85423	0.58065	0.40673	0.95804 (max o	12	2135; 1426; 1203; 78	0.23198785	0.61377794		16.0
	ZINC03947431	−9.2	448.379	7.0003e−4	11	7	299.19	0.45035	186.37	−3.2535	138.3038816707	0.29406	6.8585	0.0023805	0.5	0.38195	0.95949 (max o	11	434; 371; 339; 89; 90	0.11333305	0.69073015		15.0
	ZINC40368510	−9.2	448.379	−0.1534	11	7	297.4	0.45306	186.37	−3.7091	138.3038816707	0.29406	7.0126	−0.52166	0.5	0.39164	0.95949 (max o	5	529; 125; 185; 244; 4	0.11811358	0.5876417		15.0
	ZINC31155609	−9.2	448.379	−0.1534	11	7	297.4	0.45306	186.37	−3.7091	138.3038816707	0.29406	7.0126	−0.52166	0.5	0.39121	0.9642 (max of	5	529; 418; 125; 185; 2	0.0985197	0.579946		15.0
	ZINC35466149	−9.2	448.379	7.0003e−4	11	7	299.19	0.45035	186.37	−3.2535	138.3038816707	0.29406	6.8585	0.0023805	0.5	0.30867	0.95709 (max o	3	675; 412; 413	−0.7616192	−0.2674347		15.0
	ZINC35466144	−9.2	448.379	7.0003e−4	11	7	299.19	0.45035	186.37	−3.2535	138.3038816707	0.29406	6.8585	0.0023805	0.5	0.30867	0.95709 (max o	3	675; 414; 413	−0.7742532	−0.2827548		15.0
	ZINC20758355	−9.2	405.389	−1.2568	9	3	295.46	0.36181	134.43	3.1167	138.3038816707	0.31367	8.116	−4.0068	0.43333	0.463	0.95837 (max o	8	2540; 2161; 1551; 15	0.7263295	−0.1663584		14.0
	ZINC02154383	−9.2	372.396	−0.0833	7	1	276.19	0.28984	106.97	3.8468	138.3038816707	0.34852	6.9425	−0.23901	0.59259	0.53694	0.95818 (max o	5	875; 709; 424; 186; 2	0.30916536	0.1232401		11.0
	ZINC08879924	−9.2	358.369	−0.4272	7	1	263.93	0.3033	106.97	4.2846	138.3038816707	0.36192	7.2864	−1.1804	0.57692	0.54303	0.96998 (max o	7	2484; 1480; 1080; 60	0.37820876	0.15538618		11.0
	ZINC02152388	−9.2	399.354	−3.3385	9	0	291.39	0.37194	140.01	−6.1037	138.3038816707	0.32448	10.198	−10.289	0.44828	0.53664	0.90934 (max o	5	1973; 1369; 1090; 86	0.16789071	0.44557282		11.0

Structure	Molecule Name	affinity	Total Molweight	cLogP	H-Acceptors	H-Donors	Total Surface Area	Relative PSA	Polar Surface Area	Druglikeness	Kd (nM) @ 20°C	LE from K...	LLE from Kd ...	LELP from Kd (n...	Shape Index	Molecular...	Neighbor Si...	Neig...	Neighbor	Neighbor ...	Neighbor ...	Ring Systems	frequency
	ZINC15707296	-9.2	419.799	-1.0575	9	2	286.49	0.32793	129.29	2.4097	138.3038816707	0.32448	7.9167	-3.259	0.51724	0.44004	0.92486 (max o	7	1591; 1519; 982; 792	0.34922972	-0.0718361		10.0
	ZINC05413855	-9.2	422.484	-0.9214	7	0	310.32	0.29502	129.84	1.2704	138.3038816707	0.31367	7.7806	-2.9375	0.6	0.50729	0.91599 (max o	4	4121; 4120; 2057; 59	-0.4894800	-0.3578342		10.0
	ZINC35442324	-9.2	435.974	0.0127	7	3	317.43	0.20628	89.49	5.2779	138.3038816707	0.31367	6.8465	0.040489	0.6	0.47775	0.9707 (max of	8	1303; 1051; 812; 665	-0.6099888	0.09502322		9.0
	ZINC04096280	-9.2	402.398	0.8578	8	4	281.69	0.33881	125.68	-3.7146	138.3038816707	0.32448	6.0014	2.6436	0.51724	0.39311	0.94529 (max o	3	6202; 1058; 1037	0.01697796	-0.3692144		8.0
	ZINC15956860	-9.2	448.454	-3.7381	9	2	315.72	0.32595	127.87	4.4381	138.3038816707	0.28515	10.597	-13.109	0.45455	0.33468	0.93948 (max o	5	985; 635; 629; 383; 3	0.20599307	-0.5126368		7.0
	ZINC15276048	-9.2	404.401	-4.1225	8	2	279.7	0.33218	118.64	5.555	138.3038816707	0.31367	10.982	-13.143	0.43333	0.29819	0.96545 (max o	3	985; 629; 379	0.18288049	-0.5134896		7.0
	ZINC04073807	-9.2	392.403	0.4155	8	4	272.39	0.35038	125.68	-8.3754	138.3038816707	0.33607	6.4437	1.2364	0.5	0.30543	0.95148 (max o	8	2407; 1122; 710; 563	0.33940572	0.4879409		7.0
	ZINC05396449	-9.2	438.483	-0.9879	8	1	315.4	0.32495	135.15	6.587	138.3038816707	0.30355	7.8471	-3.2545	0.58065	0.46623	0.95549 (max o	3	2802; 82; 262	-0.1910661	0.3368327		7.0
	ZINC04236054	-9.2	429.883	-2.2411	8	2	304.28	0.27001	106.59	4.8217	138.3038816707	0.31367	9.1003	-7.1449	0.5	0.36801	0.90672 (max o	5	3659; 901; 723; 573;	-0.2157507	0.24567007		7.0
	ZINC12139866	-9.2	432.380	-0.3621	10	6	292.84	0.41538	166.14	-3.6206	138.3038816707	0.30355	7.2213	-1.1929	0.54839	0.43127	0.96018 (max o	9	3558; 2847; 2138; 21	0.16725947	0.7019122		7.0
	ZINC12375031	-9.2	432.380	-0.2446	10	6	289.64	0.43047	173.98	-4.2658	138.3038816707	0.30355	7.1038	-0.80581	0.48387	0.28345	0.96018 (max o	6	1433; 617; 370; 60; 6	0.67899835	0.00905696		6.0
	ZINC12446745	-9.2	432.380	0.3509	10	6	288.14	0.43271	173.98	-4.2232	138.3038816707	0.30355	6.5083	1.156	0.45161	0.26019	0.99629 (max o	7	1433; 712; 617; 480;	0.66957176	0.05181355		6.0
	ZINC11592656	-9.2	445.447	-2.5378	10	7	306.15	0.44906	182.82	2.381	138.3038816707	0.29406	9.397	-8.6302	0.375	0.36573	0.95949 (max o	3	607; 213; 286	0.9296614	0.31340617		6.0
	ZINC18210358	-9.2	443.431	-6.7785	10	5	307.68	0.4545	188.48	3.0758	138.3038816707	0.29406	13.638	-23.051	0.375	0.36615	0.99372 (max o	7	992; 23; 44; 76; 123;	0.04269526	0.56943		5.0
	ZINC18210358	-9.2	443.431	-6.8501	10	5	307.68	0.4545	188.48	2.9767	138.3038816707	0.29406	13.709	-23.295	0.375	0.36615	0.9497 (max of	4	991; 117; 124; 169	-0.3183000	0.6365829		5.0
	ZINC39205376	-9.2	422.341	-0.4299	11	8	270.83	0.50895	197.37	-3.0467	138.3038816707	0.31367	7.2891	-1.3706	0.46667	0.3445	0.95837 (max o	10	3052; 1698; 1481; 58	-0.0851540	-0.6627718		5.0
	ZINC21984014	-9.2	444.439	-4.6138	10	6	307.33	0.45118	185.65	2.419	138.3038816707	0.29406	11.473	-15.69	0.375	0.36573	0.95219 (max o	5	2184; 1846; 1316; 8;	-0.7478922	-0.5472034		5.0
	ZINC21984014	-9.2	443.431	-3.2475	10	5	293.29	0.4249	184.45	4.6853	138.3038816707	0.29406	10.107	-11.044	0.375	0.36418	0.94718 (max o	6	2186; 1850; 1849; 13	-0.1139820	0.71774185		5.0
	ZINC15956699	-9.2	446.438	-3.7198	9	2	309.71	0.34209	135.71	3.2276	138.3038816707	0.28515	10.579	-13.045	0.42424	0.2882	0.9497 (max of	5	503; 502; 383; 111; 1	0.25912812	-0.5570896		5.0
	ZINC15958355	-9.2	434.427	-3.661	9	2	301.96	0.34081	127.87	3.3883	138.3038816707	0.29406	10.52	-12.45	0.4375	0.2733	0.94476 (max o	7	629; 503; 502; 111; 1	0.22341256	-0.5272878		5.0
	ZINC16026586	-9.2	412.421	-4.7864	9	2	283.98	0.36238	127.87	3.8701	138.3038816707	0.31367	11.646	-15.26	0.4	0.27306	0.98593 (max o	7	3418; 1878; 1262; 10	0.11537168	0.8433061		5.0
	ZINC16026509	-9.2	412.445	-1.0448	9	3	284.97	0.3646	130.83	4.2089	138.3038816707	0.31367	7.904	-3.3309	0.4	0.25945	0.98593 (max o	10	4270; 3418; 1880; 18	0.13534303	0.8413712		5.0
	ZINC20758747	-9.2	412.445	-1.0448	9	3	284.97	0.3646	130.83	4.2089	138.3038816707	0.31367	7.904	-3.3309	0.4	0.25165	0.98593 (max o	10	4270; 3418; 1880; 18	0.08294354	0.86893034		5.0
	ZINC15958066	-9.2	424.475	-3.1866	8	2	300	0.3097	118.64	-1.065	138.3038816707	0.30355	10.046	-10.498	0.3871	0.28378	0.96018 (max o	5	1843; 796; 634; 110;	0.34132013	-0.2134712		4.0
	ZINC08791136	-9.2	431.420	-2.0825	10	2	318.41	0.37486	143.09	-9.0616	138.3038816707	0.30355	8.9417	-6.8606	0.51613	0.45343	0.9617 (max of	3	1123; 764; 209	-0.0337238	0.1553618		4.0
	ZINC15249191	-9.2	413.365	-2.6382	11	3	291.2	0.44784	166.17	1.3595	138.3038816707	0.31367	9.4974	-8.4109	0.53333	0.39424	0.95053 (max o	8	1054; 789; 669; 668;	0.2684601	-0.8097222		4.0
	ZINC15957046	-9.2	424.475	-3.1847	8	2	298.5	0.31126	118.64	1.6256	138.3038816707	0.30355	10.044	-10.492	0.3871	0.2152	0.95771 (max o	5	1256; 795; 500; 395;	0.37967673	-0.5946944		4.0
	ZINC20610796	-9.2	396.422	-3.8725	8	2	273.98	0.33911	118.64	1.6685	138.3038816707	0.32448	10.732	-11.934	0.37931	0.22473	0.95642 (max o	4	1527; 1256; 501; 380	0.39141414	-0.5592705		4.0
	ZINC15957167	-9.2	432.455	-3.3242	8	2	305.72	0.30391	118.64	4.3827	138.3038816707	0.29406	10.183	-11.304	0.40625	0.29791	0.9642 (max of	9	985; 798; 635; 633; 6	0.16752416	-0.5318897		4.0
	ZINC31158954	-9.2	360.361	0.1524	7	5	235.62	0.38868	135.29	-0.37577	138.3038816707	0.36192	6.7068	0.42109	0.42308	0.30051	0.9464 (max of	3	1898; 1024; 304	0.8124721	-0.4826871		4.0
	ZINC04260093	-9.2	374.439	0.6225	8	2	282.35	0.28029	90.98	8.3915	138.3038816707	0.34852	6.2367	1.7861	0.62963	0.53326	0.96106 (max o	3	4549; 4045; 730	-0.3313622	-0.2374601		4.0
	ZINC04557377	-9.2	418.397	0.5121	9	5	288.04	0.37682	145.91	-3.7146	138.3038816707	0.31367	6.3471	1.6326	0.5	0.31641	0.95837 (max o	4	460; 409; 203; 241	0.1406445	-0.3827951		4.0
	ZINC35456726	-9.2	434.396	0.1664	10	6	294.39	0.41319	166.14	-3.7146	138.3038816707	0.30355	6.6928	0.54819	0.51613	0.32249	0.96502 (max o	5	671; 460; 177; 241; 3	0.15057279	-0.3618148		4.0
	ZINC15967715	-9.2	446.482	-2.8941	8	2	319.48	0.29082	118.64	4.6249	138.3038816707	0.28515	9.7533	-10.149	0.42424	0.31005	0.96343 (max o	8	1241; 985; 798; 633;	0.18082204	-0.5669741		4.0
	ZINC35270768	-9.2	376.360	-0.671	8	6	239.88	0.43638	155.52	0.76178	138.3038816707	0.34852	7.5302	-1.9253	0.40741	0.31342	0.9578 (max of	3	1295; 1043; 1042	-0.4737824	-0.7370150		4.0
	ZINC35458753	-9.2	376.360	-0.671	8	6	239.88	0.43638	155.52	-0.097646	138.3038816707	0.34852	7.5302	-1.9253	0.40741	0.31342	0.97149 (max o	3	1918; 1056; 522	-0.9054906	-0.3515806		4.0
	ZINC18210358	-9.2	443.431	-6.7785	10	5	307.68	0.4545	188.48	3.0017	138.3038816707	0.29406	13.638	-23.051	0.375	0.36615	0.95709 (max o	7	990; 23; 392; 76; 116	0.03597302	0.6091674		3.0
	ZINC18210358	-9.2	444.439	-4.6124	10	6	306.5	0.4524	185.65	2.151	138.3038816707	0.29406	11.472	-15.685	0.375	0.34344	0.93948 (max o	3	988; 113; 167	-0.4735877	-0.5701562		3.0
	ZINC03850729	-9.2	420.413	0.3111	9	6	291	0.39409	164.75	0.71166	138.3038816707	0.31367	6.5481	0.99182	0.46667	0.39669	0.91964 (max o	2	2476; 1504	-0.9021752	-0.1423587		3.0
	ZINC15956860	-9.2	449.462	-1.9358	9	2	312	0.31792	132.45	3.5392	138.3038816707	0.28515	8.795	-6.7887	0.45455	0.34261	0.93948 (max o	2	628; 374	-0.7044144	0.41830733		3.0
	ZINC15276048	-9.2	405.409	-2.3202	8	2	275.98	0.32318	123.22	4.6181	138.3038816707	0.31367	9.1794	-7.3971	0.43333	0.3061	0.96545 (max o	2	628; 378	-0.6918538	0.43734524		3.0
	ZINC21984014	-9.2	442.423	-5.3235	10	4	294.47	0.42721	187.28	5.0928	138.3038816707	0.29406	12.183	-18.103	0.375	0.36418	0.94718 (max o	4	2183; 1845; 7; 64	-0.4322204	-0.4804909		3.0
	ZINC21984014	-9.2	443.431	-6.6898	10	5	308.51	0.45328	188.48	2.3981	138.3038816707	0.29406	13.549	-22.75	0.375	0.36573	0.9497 (max of	4	2187; 1851; 10; 68	-0.3435728	-0.5215937		3.0

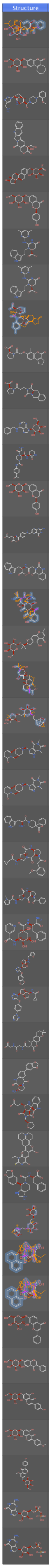

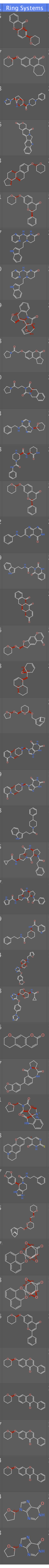

Structure	Molecule Name	affinity	Total Molweight	cLogP	H-Acceptors	H-Donors	Total Surface Area	Relative PSA	Polar Surface Area	Druglikeness	Kd (nM) @ 20°C	LE from K...	LLE from Kd ...	LELP from Kd (n...	Shape Index	Molecular...	Neighbor Si...	Neig...	Neighbor	Neighbor ...	Neighbor ...	Ring Systems	frequency
	ZINC36728545	-9.2	392.359	-1.3716	9	6	257.5	0.43355	156.91	-2.2179	138.3038816707	0.33607	8.2308	-4.0813	0.46429	0.35879	0.94573 (max o	6	3080; 2619; 1926; 81	-0.2673629	0.60222775		3.0
	ZINC04082105	-9.2	392.403	0.4136	8	4	273.89	0.34846	125.68	-8.4156	138.3038816707	0.33607	6.4456	1.2307	0.5	0.40218	0.94589 (max o	4	708; 563; 79; 135	0.3530515	0.47092727		2.0
	ZINC08254033	-9.2	441.578	-5.4041	9	2	347.02	0.26229	95.57	2.6132	138.3038816707	0.29406	12.263	-18.377	0.59375	0.48758	0.97533 (max o	3	2098; 1453; 207	0.8391953	-0.1964038		2.0
	ZINC68590099	-9.2	350.329	-0.1973	7	2	233.94	0.32829	99.96	4.9827	138.3038816707	0.36192	7.0565	-0.54515	0.46154	0.22364	0.93046	1	245	-0.6535753	0.43009716		2.0
	ZINC15204501	-9.2	447.502	-0.5383	8	4	307.67	0.32411	139.51	1.3464	138.3038816707	0.29406	7.3975	-1.8306	0.5	0.35715	0.92619 (max o	4	149; 160; 216; 260	-0.0986157	-0.5204963		2.0
	ZINC12429154	-9.2	418.397	0.5121	9	5	288.04	0.37682	145.91	-3.7146	138.3038816707	0.31367	6.3471	1.6326	0.53333	0.39876	0.9707 (max of	8	1223; 1037; 954; 945	0.07301783	-0.3798111		2.0
	ZINC01311572	-9.2	406.445	-0.5489	9	3	295.76	0.3087	128.01	3.7341	138.3038816707	0.31367	7.4081	-1.75	0.46667	0.4688	0.94785 (max o	3	1350; 1349; 326	0.00676372	-0.9848537		2.0
	ZINC01311572	-9.2	405.437	-0.5489	9	2	307.27	0.33459	126.4	4.8974	138.3038816707	0.31367	7.4081	-1.75	0.46667	0.44364	0.94515 (max o	3	1350; 1349; 325	0.01808947	-0.9909650		2.0
	ZINC04044099	-9.2	352.365	-1.6024	6	0	230.78	0.27693	78.9	1.607	138.3038816707	0.36192	8.4616	-4.4275	0.42308	0.1538	0.93046 (max o	4	1117; 894; 561; 560	-0.6786977	-0.0431639		2.0
	ZINC04064415	-9.2	370.380	-0.5479	7	0	267.84	0.2873	95.97	3.8281	138.3038816707	0.34852	7.4071	-1.5721	0.51852	0.34694	0.90377 (max o	4	1652; 1399; 1396; 18	0.16718954	0.20946938		2.0
	ZINC04090271	-9.2	370.384	-1.7893	8	0	263.51	0.29255	101.06	6.1914	138.3038816707	0.34852	8.6485	-5.134	0.51852	0.47051	0.95401 (max o	2	2419; 1695	0.21931635	-0.9134490		2.0
	ZINC04534330	-9.2	387.395	-0.5073	10	5	276.48	0.41102	145.78	-0.80219	138.3038816707	0.33607	7.3665	-1.5095	0.53571	0.39692	0.94862 (max o	6	1955; 1717; 1430; 11	-0.4091933	-0.7116793		2.0
	ZINC05433820	-9.2	445.498	-4.4461	11	4	331.3	0.36571	155.56	3.7014	138.3038816707	0.29406	11.305	-15.12	0.53125	0.54611	0.91704	1	939	-0.7831625	-0.0655168		2.0
	ZINC12429155	-9.2	418.397	0.5121	9	5	288.04	0.37682	145.91	-3.7146	138.3038816707	0.31367	6.3471	1.6326	0.53333	0.4005	0.9707 (max of	9	1223; 954; 779; 603;	0.09570514	-0.389598		2.0
	ZINC18456289	-9.2	439.387	-5.798	13	4	322.75	0.50758	214.65	3.0765	138.3038816707	0.29406	12.657	-19.717	0.625	0.60362	0.98148	1	800	-0.0209413	-0.8104092		2.0
	ZINC20611235	-9.2	405.389	-1.2568	9	3	295.46	0.36181	134.43	6.1598	138.3038816707	0.31367	8.116	-4.0068	0.53333	0.49882	0.97757 (max o	3	1089; 1008; 696	0.07859559	-0.2852699		2.0
	ZINC21984014	-9.2	444.439	-4.7179	10	5	308.31	0.44955	182.49	2.6033	138.3038816707	0.29406	11.577	-16.044	0.375	0.37412	0.93157 (max o	2	1317; 1046	0.54914695	-0.737382		2.0
	ZINC31156011	-9.2	438.427	0.2591	10	5	296.68	0.39956	155.14	-11.09	138.3038816707	0.30355	6.6001	0.85357	0.48387	0.46355	0.96018 (max o	7	1895; 1307; 1284; 84	-0.2269515	0.85775846		2.0
	ZINC35464957	-9.2	391.482	-0.7932	6	3	283.97	0.29676	113.35	-0.82856	138.3038816707	0.33607	7.6524	-2.3602	0.46429	0.37458	0.9678 (max of	4	3934; 3074; 2293; 84	-0.0713974	0.43922418		2.0
	ZINC67913874	-9.2	446.491	-1.3965	10	6	302.71	0.39179	158.3	0.12158	138.3038816707	0.30355	8.2557	-4.6006	0.51613	0.45186	0.9501	1	532	-0.4534465	0.7958111		2.0
	ZINC02091085	-9.2	384.439	-3.3444	9	2	274.27	0.27065	84.44	3.0052	138.3038816707	0.33607	10.204	-9.9515	0.5	0.47007	0.85878	1	328	0.8804167	-0.406225		1.0
	ZINC02091085	-9.2	383.431	-1.3666	9	1	285.24	0.2987	83.19	3.3835	138.3038816707	0.33607	8.2258	-4.0664	0.5	0.47007	0.85878	1	327	0.8822391	-0.3854559		1.0
	ZINC02118552	-9.2	391.470	0.207	6	2	296.46	0.24415	103.87	5.9004	138.3038816707	0.32448	6.6522	0.63794	0.55172	0.47234		0		-0.7333503	-0.6548358		1.0
	ZINC03839296	-9.2	440.498	-0.0222	9	2	314.86	0.29277	108.05	7.1983	138.3038816707	0.29406	6.8814	-0.075493	0.5	0.39302	0.95267 (max o	3	1375; 132; 250	-0.484357	-0.1741075		1.0
	ZINC03839398	-9.2	435.483	0.0847	9	2	313.54	0.28401	103.75	8.3054	138.3038816707	0.29406	6.7745	0.28804	0.5	0.3178	0.89799 (max o	5	4038; 3164; 2376; 20	-0.5655229	-0.1018627		1.0
	ZINC03840189	-9.2	442.470	-1.3943	10	6	323.71	0.39412	174.87	4.7875	138.3038816707	0.29406	8.2535	-4.7415	0.53125	0.45909	0.89631 (max o	3	1676; 704; 432	-0.4292361	-0.1739169		1.0
	ZINC04259823	-9.2	414.397	-0.3161	11	1	290.23	0.38463	119.5	0.15677	138.3038816707	0.31367	7.1753	-1.0078	0.56667	0.33629	0.90672 (max o	5	2060; 1145; 907; 715;	0.00242639	-0.2975714		1.0
	ZINC04259843	-9.2	424.420	-0.3897	12	1	301.23	0.40862	131.1	3.9965	138.3038816707	0.30355	7.2489	-1.2838	0.58065	0.35432	0.95053 (max o	5	2061; 2047; 1703; 14	-0.1384405	-0.3800018		1.0
	ZINC06623956	-9.2	415.421	-1.5242	9	2	306.62	0.35666	133.86	-1.8144	138.3038816707	0.31367	8.3834	-4.8593	0.6	0.53781	0.89699 (max o	2	4467; 764	0.01887893	0.09780696		1.0
	ZINC08296596	-9.2	352.369	-1.9352	7	2	243.79	0.30998	95.51	4.2433	138.3038816707	0.36192	8.7944	-5.347	0.5	0.26956	0.84147	1	261	-0.2655053	0.27129927		1.0
	ZINC16026586	-9.2	413.429	-2.9841	9	2	280.26	0.35392	132.45	3.0539	138.3038816707	0.31367	9.8433	-9.5136	0.4	0.28356	0.83335	1	1263	-0.7612671	-0.3799001		1.0
	ZINC20411417	-9.2	437.519	-0.2598	7	1	305.64	0.28884	120.29	3.3468	138.3038816707	0.30355	7.119	-0.85588	0.45161	0.41639	0.90825 (max o	2	1; 45	0.7536811	0.23683558		1.0
	ZINC40309141	-9.2	434.451	0.1677	9	3	307.97	0.29016	120.86	5.4378	138.3038816707	0.29406	6.6915	0.57029	0.46875	0.48228	0.95219	1	129	0.8272585	0.31409597		1.0
	ZINC67902711	-9.2	446.491	-1.2779	10	6	303.54	0.39072	158.3	-5.119	138.3038816707	0.30355	8.1371	-4.2099	0.51613	0.48603	0.87363 (max o	3	5681; 3457; 2643	-0.3931028	0.39678675		1.0
	ZINC71404542	-9.2	366.324	0.3134	7	2	233.84	0.40429	101.05	1.6898	138.3038816707	0.34852	6.5458	0.89924	0.37037	0.23527	0.91109	1	423	0.82397276	-0.2101707		1.0
	ZINC71404543	-9.2	366.324	0.3134	7	2	233.84	0.40429	101.05	1.6898	138.3038816707	0.34852	6.5458	0.89924	0.37037	0.23527	0.91109 (max o	2	1291; 422	0.8049767	-0.2022788		1.0
	ZINC04349282	-9.1	400.382	1.0378	8	4	280.14	0.34069	125.68	-3.2535	164.2064047263	0.32095	5.7468	3.2335	0.51724	0.3797	0.9617 (max of	7	738; 737; 595; 489; 2	0.28058597	0.54693896		38.0
	ZINC05667885	-9.1	400.382	0.3293	8	4	280.14	0.34069	125.68	-3.6206	164.2064047263	0.32095	6.4553	1.026	0.55172	0.38857	0.95642 (max o	7	1182; 1128; 611; 604;	0.2764243	0.5815112		30.0
	ZINC04404502	-9.1	432.380	-0.3621	10	6	292.84	0.41538	166.14	-3.6206	164.2064047263	0.30025	7.1467	-1.206	0.54839	0.3877	0.96262 (max o	10	1774; 1447; 973; 742;	0.22637221	0.6461347		30.0
	ZINC03947504	-9.1	446.407	-0.0864	10	5	308.75	0.38394	155.14	-3.4398	164.2064047263	0.29086	6.871	-0.29705	0.5625	0.40007	0.98343 (max o	12	2890; 1774; 1773; 14	0.27225778	0.66944367		30.0
	ZINC31155618	-9.1	446.407	-0.0864	10	5	308.75	0.38394	155.14	-3.4398	164.2064047263	0.29086	6.871	-0.29705	0.53125	0.38135	0.97319 (max o	6	2663; 1994; 828; 743;	0.17944252	0.63084674		30.0
	ZINC35000840	-9.1	441.185	-10.923	16	5	270.33	0.74361	273.56	-47.565	164.2064047263	0.33242	17.708	-32.861	0.5	0.50554	0.93385 (max o	9	1584; 1506; 1449; 12	-0.2996746	-0.4810414		26.0
	ZINC35000840	-9.1	440.177	-12.171	16	4	271.51	0.74472	276.39	-42.835	164.2064047263	0.33242	18.956	-36.614	0.5	0.50536	0.93385 (max o	10	1995; 1585; 1507; 14	-0.3403857	-0.4456493		26.0

Structure	Molecule Name	affinity	Total Molweight	cLogP	H-Acceptors	H-Donors	Total Surface Area	Relative PSA	Polar Surface Area	Druglikeness	Kd (nM) @ 20°C	LE from K...	LLE from Kd ...	LELP from Kd (n...	Shape Index	Molecular...	Neighbor Si...	Neig...	Neighbor	Neighbor ...	Neighbor ...	Ring Systems	frequency
	ZINC02116849	-9.1	387.343	-4.047	9	1	279	0.40208	144.89	1.9281	164.2064047263	0.33242	10.832	-12.174	0.53571	0.49073	0.97037 (max o	4	3631; 1360; 1359; 10	0.19032653	0.08305799		24.0
	ZINC08995542	-9.1	439.399	-1.5441	10	2	325.02	0.37896	157.08	5.1674	164.2064047263	0.29086	8.3287	-5.3087	0.59375	0.4474	0.97948 (max o	8	4090; 2537; 2513; 18	0.5730704	0.4376048		24.0
	ZINC03947433	-9.1	448.379	7.0003e-4	11	7	299.19	0.45035	186.37	-3.2535	164.2064047263	0.29086	6.7839	0.0024067	0.5	0.38195	0.9642 (max of	11	89; 90; 102; 136; 146	0.07518975	0.7004745		15.0
	ZINC40368513	-9.1	448.379	-0.1534	11	7	297.4	0.45306	186.37	-3.7091	164.2064047263	0.29086	6.938	-0.52739	0.5	0.39164	0.9642 (max of	5	125; 185; 244; 404; 4	0.10894717	0.61743695		15.0
	ZINC08624560	-9.1	420.508	0.0414	9	4	317.56	0.29065	114.37	6.9641	164.2064047263	0.31026	6.7432	0.13344	0.63333	0.57167	0.95579 (max o	6	1829; 1764; 1540; 77	-0.0687815	-0.6084974		11.0
	ZINC02098444	-9.1	358.369	-0.4272	7	1	263.93	0.3033	106.97	3.8468	164.2064047263	0.35799	7.2118	-1.1933	0.61538	0.54303	0.94927 (max o	5	876; 474; 249; 333; 3	0.3427957	0.10890162		11.0
	ZINC08879922	-9.1	358.369	-0.4272	7	1	263.93	0.3033	106.97	4.2846	164.2064047263	0.35799	7.2118	-1.1933	0.57692	0.54303	0.96998 (max o	7	2850; 1480; 1080; 60	0.35911074	0.14535078		11.0
	ZINC12296446	-9.1	402.422	-0.2215	8	0	302.1	0.28782	105.2	4.338	164.2064047263	0.32095	7.0061	-0.69013	0.55172	0.53177	0.97415 (max o	5	2943; 2944; 1480; 60	0.33998588	0.23709576		11.0
	ZINC02108368	-9.1	427.408	-2.4862	9	0	317.41	0.34145	140.01	-11.258	164.2064047263	0.30025	9.2708	-8.2805	0.48387	0.52689	0.91993 (max o	6	1369; 1357; 1118; 10	0.1790032	0.46719363		11.0
	ZINC15675878	-9.1	397.390	-2.4217	10	2	286.98	0.36222	138.52	3.2635	164.2064047263	0.32095	9.2063	-7.5453	0.51724	0.43483	0.95945 (max o	8	1835; 1591; 982; 792	0.31675658	-0.1257122		10.0
	ZINC35442325	-9.1	419.519	-0.4925	7	3	308.36	0.21235	89.49	3.8968	164.2064047263	0.31026	7.2771	-1.5874	0.63333	0.47775	0.9707 (max of	11	2653; 1304; 1271; 10	-0.5848415	0.1239427		9.0
	ZINC22937011	-9.1	444.598	-0.6969	8	3	336.83	0.20494	92.73	5.858	164.2064047263	0.29086	7.4815	-2.396	0.625	0.4985	0.97319 (max o	11	3438; 1278; 1277; 12	-0.5844084	0.05925994		9.0
	ZINC04235975	-9.1	365.388	0.7915	7	2	265.22	0.26687	89.95	6.5366	164.2064047263	0.34473	5.9931	2.296	0.55556	0.36327	0.92442 (max o	4	2056; 2054; 1137; 25	-0.234511	0.31989232		9.0
	ZINC35466139	-9.1	434.396	0.1664	10	6	294.39	0.41319	166.14	-3.7146	164.2064047263	0.30025	6.6182	0.55421	0.51613	0.39871	0.96739 (max o	5	954; 945; 674; 320; 5	0.08055796	-0.4317506		8.0
	ZINC12869128	-9.1	416.476	-2.1645	9	4	311.61	0.35294	153.2	3.5891	164.2064047263	0.31026	8.9491	-6.9765	0.56667	0.48229	0.9897 (max of	8	6947; 1802; 1688; 14	0.2671205	0.25495997		8.0
	ZINC04074089	-9.1	416.476	-2.1645	9	4	311.61	0.35294	153.2	3.5891	164.2064047263	0.31026	8.9491	-6.9765	0.56667	0.4843	0.9897 (max of	8	6947; 1802; 1688; 14	0.284039	0.28660098		8.0
	ZINC12429157	-9.1	418.397	0.5121	9	5	288.04	0.37682	145.91	-3.7146	164.2064047263	0.31026	6.2725	1.6506	0.53333	0.39799	0.9707 (max of	8	954; 945; 779; 674; 6	0.07395106	-0.3974589		7.0
	ZINC12446747	-9.1	432.380	0.3509	10	6	288.14	0.43271	173.98	-4.2232	164.2064047263	0.30025	6.4337	1.1687	0.45161	0.25678	0.99629 (max o	7	1433; 712; 20; 61; 92	0.6359172	0.04541109		7.0
	ZINC06041521	-9.1	432.380	-0.7865	10	7	289.19	0.43134	177.14	-2.3989	164.2064047263	0.30025	7.5711	-2.6195	0.48387	0.39235	0.96018 (max o	8	5883; 2481; 2095; 12	0.65928596	0.1879483		7.0
	ZINC02146782	-9.1	366.324	-2.5996	7	0	269.27	0.32562	115.79	1.0433	164.2064047263	0.34473	9.3842	-7.541	0.55556	0.4555	0.95201 (max o	4	1971; 1093; 543; 542	-0.3284409	-0.1245150		7.0
	ZINC15449241	-9.1	422.341	-0.4299	11	8	270.83	0.50895	197.37	-3.0467	164.2064047263	0.31026	7.2145	-1.3856	0.46667	0.3445	0.95053 (max o	10	3052; 1698; 1481; 58	-0.0988736	-0.674788		6.0
	ZINC08764449	-9.1	358.369	-0.654	7	0	266.08	0.2892	95.97	2.1725	164.2064047263	0.35799	7.4386	-1.8269	0.57692	0.51964	0.96998 (max o	3	2722; 946; 871	0.1611319	0.1541895		6.0
	ZINC15675878	-9.1	396.382	-2.2328	10	1	297.95	0.3857	137.27	3.7739	164.2064047263	0.32095	9.0174	-6.9568	0.51724	0.43483	0.97415 (max o	8	1836; 793; 653; 652;	0.33089176	-0.1691626		6.0
	ZINC15675878	-9.1	396.382	-2.2328	10	1	297.95	0.3857	137.27	3.7739	164.2064047263	0.32095	9.0174	-6.9568	0.51724	0.43483	0.97415 (max o	8	1837; 794; 653; 652;	0.31268653	-0.1661372		6.0
	ZINC15958956	-9.1	418.428	-3.2471	8	2	291.96	0.31823	118.64	3.3221	164.2064047263	0.30025	10.032	-10.815	0.41935	0.25417	0.96018 (max o	6	629; 111; 162; 377; 3	0.23577756	-0.5620624		5.0
	ZINC15957764	-9.1	404.401	-3.591	8	2	279.7	0.33218	118.64	3.3658	164.2064047263	0.31026	10.376	-11.574	0.4	0.24778	0.95031 (max o	6	629; 111; 162; 377; 3	0.2381246	-0.5427543		5.0
	ZINC13424722	-9.1	400.382	1.0378	8	4	280.14	0.34069	125.68	-3.2535	164.2064047263	0.32095	5.7468	3.2335	0.55172	0.3797	0.9617 (max of	7	1228; 737; 580; 293;	0.32235372	0.5559057		5.0
	ZINC04349271	-9.1	400.382	1.0378	8	4	280.14	0.34069	125.68	-3.2535	164.2064047263	0.32095	5.7468	3.2335	0.55172	0.3797	0.9617 (max of	7	1228; 738; 737; 580;	0.31564456	0.53593445		5.0
	ZINC04073398	-9.1	378.376	0.0735	8	4	258.63	0.36902	125.68	-6.0454	164.2064047263	0.34473	6.7111	0.21321	0.48148	0.25864	0.94905 (max o	8	2407; 2406; 1400; 11	0.32370394	0.5031068		4.0
	ZINC15957213	-9.1	396.422	-3.8725	8	2	273.98	0.33911	118.64	1.6685	164.2064047263	0.32095	10.657	-12.066	0.37931	0.22113	0.95372 (max o	5	1527; 1256; 227; 395	0.37132823	-0.5530706		4.0
	ZINC31160058	-9.1	433.443	-3.2187	9	2	300.57	0.32129	125.37	6.2494	164.2064047263	0.29086	10.003	-11.066	0.40625	0.39607	0.95949 (max o	9	1026; 47; 98; 179; 23	-0.8008318	0.09423711		4.0
	ZINC05742781	-9.1	448.423	0.4421	10	5	310.3	0.38202	155.14	-3.5326	164.2064047263	0.29086	6.3425	1.5199	0.5	0.33139	0.95983 (max o	7	1751; 757; 241; 352;	-0.12881695	-0.3655384		4.0
	ZINC05742784	-9.1	448.423	0.4421	10	5	310.3	0.38202	155.14	-3.5326	164.2064047263	0.29086	6.3425	1.5199	0.5	0.33139	0.95949 (max o	3	1751; 757; 460	0.15926753	-0.3398309		4.0
	ZINC35458758	-9.1	376.360	-0.671	8	6	239.88	0.43638	155.52	-0.097646	164.2064047263	0.34473	7.4556	-1.9465	0.40741	0.31342	0.97149 (max o	3	1918; 1056; 410	-0.8892989	-0.3599712		4.0
	ZINC03839252	-9.1	400.434	-0.3384	9	2	286.84	0.32136	108.05	5.7764	164.2064047263	0.32095	7.123	-1.0544	0.55172	0.40295	0.95053 (max o	7	2374; 1376; 1375; 87	-0.4414342	-0.2093807		4.0
	ZINC04222678	-9.1	410.405	-1.0452	9	1	301.36	0.32888	122.99	0.78313	164.2064047263	0.31026	7.8298	-3.3688	0.56667	0.4199	0.96832 (max o	6	3679; 2802; 2801; 11	-0.1700813	0.36829063		4.0
	ZINC12883352	-9.1	440.383	-0.7104	10	2	314.1	0.37555	143.45	2.4365	164.2064047263	0.29086	7.495	-2.4424	0.5625	0.3943	0.99059 (max o	7	1781; 1779; 1500; 14	0.49894306	0.38909227		4.0
	ZINC08790425	-9.1	440.383	-0.7104	10	2	314.1	0.37555	143.45	2.4365	164.2064047263	0.29086	7.495	-2.4424	0.5625	0.39633	0.99059 (max o	7	1781; 1779; 1500; 14	0.50504214	0.36899608		4.0
	ZINC08790579	-9.1	438.411	-0.5599	9	1	316.7	0.31591	125.43	6.3014	164.2064047263	0.29086	7.3445	-1.9249	0.5625	0.41531	0.9642 (max of	7	4090; 1806; 1805; 15	0.553624	0.45254397		4.0
	ZINC15957047	-9.1	424.475	-3.1847	8	2	298.5	0.31126	118.64	1.6256	164.2064047263	0.30025	9.9693	-10.607	0.3871	0.21175	0.95521 (max o	5	1527; 795; 501; 227;	0.35782292	-0.5888023		4.0
	ZINC35458038	-9.1	353.305	-0.3835	7	2	238.43	0.37579	123.96	2.5684	164.2064047263	0.35799	7.1681	-1.0713	0.46154	0.25858	0.97533 (max o	3	1916; 1609; 673	-0.8409804	-0.4993790		4.0
	ZINC36374483	-9.1	417.389	-1.405	9	5	299.31	0.37693	159.74	-3.819	164.2064047263	0.31026	8.1896	-4.5285	0.5	0.40537	0.96343 (max o	3	1324; 676; 525	0.29374847	-0.8029184		4.0
	ZINC36374479	-9.1	417.389	-1.405	9	5	299.31	0.37693	159.74	-3.819	164.2064047263	0.31026	8.1896	-4.5285	0.5	0.40537	0.95837 (max o	3	1324; 676; 526	0.30956316	-0.8169842		4.0

Structure	Molecule Name	affinity	Total Molweight	cLogP	H-Acceptors	H-Donors	Total Surface Area	Relative PSA	Polar Surface Area	Druglikeness	Kd (nM) @ 20°C	LE from K...	LLE from Kd ...	LELP from Kd (n...	Shape Index	Molecular...	Neighbor Si...	Neig...	Neighbor	Neighbor ...	Neighbor ...	Ring Systems	frequency
	ZINC49601728	-9.1	440.443	0.0769	10	6	314.91	0.38627	166.14	-6.1546	164.2064047263	0.30025	6.7077	0.25612	0.58065	0.46737	0.96262 (max o	6	3530; 3036; 1617; 13	-0.2781648	0.5512395		4.0
	ZINC04064228	-9.1	374.344	0.6545	8	4	255.43	0.37364	125.68	-4.3275	164.2064047263	0.34473	6.1301	1.8986	0.51852	0.3532	0.94905 (max o	2	597; 51	-0.1370377	-0.8165505		3.0
	ZINC14584315	-9.1	446.407	1.3578	10	4	293.2	0.38336	136.3	-4.5704	164.2064047263	0.29086	5.4268	4.6681	0.53125	0.28584	0.9642 (max of	2	572; 159	0.32106578	0.8407671		3.0
	ZINC05004354	-9.1	422.385	0.1214	10	7	282.99	0.43005	169.3	-4.0605	164.2064047263	0.31026	6.6632	0.39129	0.53333	0.38308	0.96092 (max o	7	1937; 1332; 1306; 12	0.40637198	-0.8331479		3.0
	ZINC35456519	-9.1	413.365	-2.6382	11	3	291.2	0.44784	166.17	1.3595	164.2064047263	0.31026	9.4228	-8.5033	0.53333	0.40341	0.95053 (max o	8	1054; 789; 669; 668;	0.27280462	-0.770074		3.0
	ZINC12605075	-9.1	409.509	-4.4703	10	4	309.33	0.34953	138.49	0.13155	164.2064047263	0.32095	11.255	-13.928	0.51724	0.54444	0.95123 (max o	2	2471; 277	-0.1539543	0.2524469		3.0
	ZINC35018256	-9.1	422.460	-5.2541	8	3	323.94	0.34003	107.9	3.559	164.2064047263	0.30025	12.039	-17.499	0.45161	0.3478	0.89699	1	515	0.6427722	-0.6036266		3.0
	ZINC35018256	-9.1	420.444	-5.9638	8	1	311.08	0.31275	109.53	4.9629	164.2064047263	0.30025	12.748	-19.863	0.45161	0.35774	0.88804	1	515	0.66662014	-0.5651632		3.0
	ZINC35018256	-9.1	421.452	-3.8878	8	2	309.9	0.31013	106.7	5.5508	164.2064047263	0.30025	10.672	-12.949	0.45161	0.35813	0.89699 (max o	3	516; 514; 517	0.6428724	-0.5682634		3.0
	ZINC35270820	-9.1	360.361	-0.1543	7	4	232.58	0.41332	127.59	0.14074	164.2064047263	0.35799	6.9389	-0.43102	0.42308	0.29718	0.96721 (max o	3	661; 660; 181	-0.8185056	0.08714783		2.0
	ZINC72325722	-9.1	420.489	0.1204	9	0	290.63	0.2818	106.69	9.7207	164.2064047263	0.32095	6.6642	0.37513	0.44828	0.50034	0.87588	1	246	0.8803069	0.31155133		2.0
	ZINC08627169	-9.1	427.523	-1.2634	8	3	319.89	0.23461	102.74	4.6647	164.2064047263	0.30025	8.048	-4.2079	0.64516	0.48072	0.9499 (max of	3	4172; 1193; 280	0.11299211	-0.5490436		2.0
	ZINC15676186	-9.1	424.499	-0.9868	8	2	313.6	0.23753	110.68	4.4667	164.2064047263	0.30025	7.7714	-3.2866	0.54839	0.53614	0.96224 (max o	3	4688; 499; 300	0.1915577	-0.1803173		2.0
	ZINC15676186	-9.1	423.491	-0.7979	8	1	324.57	0.2633	109.43	5.0385	164.2064047263	0.30025	7.5825	-2.6575	0.54839	0.53614	0.96224 (max o	3	4689; 301; 498	0.2059624	-0.2156585		2.0
	ZINC67913911	-9.1	406.386	0.3752	9	6	276.64	0.39257	149.07	-1.9967	164.2064047263	0.32095	6.4094	1.169	0.55172	0.34267	0.95099 (max o	3	855; 854; 322	-0.8381182	0.34606656		2.0
	ZINC35466136	-9.1	434.396	0.1664	10	6	294.39	0.41319	166.14	-3.7146	164.2064047263	0.30025	6.6182	0.55421	0.51613	0.40304	0.96739 (max o	5	779; 674; 320; 524; 3	0.11043349	-0.4055575		2.0
	ZINC67913877	-9.1	446.491	-1.3965	10	6	302.71	0.39179	158.3	0.12158	164.2064047263	0.30025	8.1811	-4.6512	0.51613	0.45186	0.9501	1	420	-0.4517069	0.81927437		2.0
	ZINC04081844	-9.1	392.403	0.4155	8	4	272.39	0.35038	125.68	-8.3754	164.2064047263	0.33242	6.3691	1.2499	0.42857	0.30543	0.95148 (max o	7	2783; 2782; 2406; 11	0.36646217	0.45470473		2.0
	ZINC04259696	-9.1	402.410	0.2116	11	1	290.07	0.37174	112.86	2.7838	164.2064047263	0.32095	6.573	0.65928	0.58621	0.39976	0.91704 (max o	3	2445; 2425; 449	-0.2223066	-0.2733241		2.0
	ZINC05433774	-9.1	433.487	-4.3873	11	4	323.55	0.36507	147.72	3.834	164.2064047263	0.30025	11.172	-14.612	0.51613	0.53479	0.9145	1	3261	0.13679698	0.33095577		2.0
	ZINC05533353	-9.1	386.355	-0.4899	8	2	250.22	0.38094	119.36	-1.0904	164.2064047263	0.33242	7.2745	-1.4738	0.35714	0.28247	0.96253 (max o	3	934; 933; 756	0.87523776	-0.1238924		2.0
	ZINC08296904	-9.1	422.416	-1.9124	9	2	307.81	0.33433	127.87	4.8089	164.2064047263	0.30025	8.697	-6.3694	0.58065	0.34915	0.93686 (max o	5	3272; 1672; 1454; 55	-0.2604457	0.45215824		2.0
	ZINC08764581	-9.1	396.378	-1.2358	9	3	291.4	0.3638	138.87	4.6514	164.2064047263	0.32095	8.0204	-3.8504	0.58621	0.49248	0.9428 (max of	3	2979; 2128; 695	0.1473914	-0.2405076		2.0
	ZINC08764756	-9.1	378.363	-1.3986	8	1	270.39	0.31436	109.85	5.0024	164.2064047263	0.33242	8.1832	-4.2074	0.5	0.50904	0.88492 (max o	2	952; 145	0.7184206	-0.3088560		2.0
	ZINC08790901	-9.1	430.504	-0.7803	8	1	299.31	0.34242	135.15	8.4506	164.2064047263	0.31026	7.5649	-2.515	0.46667	0.33829	0.93965 (max o	5	3639; 2535; 2415; 17	0.38765803	-0.6342947		2.0
	ZINC12653681	-9.1	410.402	-1.1622	10	2	261.6	0.4099	123.63	3.9896	164.2064047263	0.33242	7.9468	-3.4962	0.53571	0.41245	0.96253 (max o	2	2029; 998	-0.5674642	-0.7577615		2.0
	ZINC14855702	-9.1	434.396	0.5451	10	6	308.13	0.39477	166.14	-1.6837	164.2064047263	0.30025	6.2395	1.8155	0.6129	0.43799	0.93409 (max o	3	3036; 1897; 627	-0.2629177	0.6220953		2.0
	ZINC31160058	-9.1	434.451	-1.4164	9	2	296.85	0.31278	129.95	4.3792	164.2064047263	0.29086	8.201	-4.8696	0.40625	0.39607	0.9642 (max of	3	1025; 178; 237	-0.7832013	-0.1759026		2.0
	ZINC31166749	-9.1	434.352	0.1029	11	7	284.9	0.47294	186.37	-3.9147	164.2064047263	0.30025	6.6817	0.34272	0.48387	0.36672	0.95493 (max o	2	1566; 1030	-0.6711212	0.33810022		2.0
	ZINC31417978	-9.1	429.400	-1.5624	9	3	306.61	0.36757	153.42	1.8004	164.2064047263	0.30025	8.347	-5.2037	0.51613	0.51059	0.98065	1	827	0.9051369	0.00778875		2.0
	ZINC49600052	-9.1	416.421	-1.5798	10	6	291.9	0.4063	158.3	-5.0311	164.2064047263	0.32095	8.3644	-4.9222	0.58621	0.51096	0.96983 (max o	9	5630; 1331; 1227; 12	-0.4558876	0.68362355		2.0
	ZINC70712686	-9.1	440.400	-2.7759	11	4	252.24	0.52141	169.05	-2.076	164.2064047263	0.30025	9.5605	-9.2454	0.32258	0.091409	0.91162 (max o	3	5818; 3544; 682	-0.6729277	-0.4334681		2.0
	ZINC72320178	-9.1	390.343	-1.6259	9	5	240	0.50946	164.89	2.8143	164.2064047263	0.33242	8.4105	-4.8911	0.39286	0.31333	0.99715	1	683	-0.8550309	0.38346124		2.0
	ZINC02124246	-9.1	414.373	0.6292	11	1	276.02	0.37135	127.77	2.4319	164.2064047263	0.31026	6.1554	2.028	0.36667	0.33661	0.95579	1	188	0.09679052	-0.8451973		1.0
	ZINC03839362	-9.1	427.503	-0.357	9	2	307.82	0.28929	103.75	5.6145	164.2064047263	0.30025	7.1416	-1.189	0.48387	0.28183	0.9396 (max of	10	4488; 4037; 3818; 27	-0.5795406	-0.0306571		1.0
	ZINC03839405	-9.1	427.503	-0.357	9	2	307.82	0.28929	103.75	5.6145	164.2064047263	0.30025	7.1416	-1.189	0.48387	0.25899	0.93139 (max o	7	4038; 3164; 2376; 20	-0.5529493	-0.0630072		1.0
	ZINC03840183	-9.1	431.444	-1.2047	10	6	314.64	0.41546	175.12	5.3062	164.2064047263	0.30025	7.9893	-4.0124	0.51613	0.45091	0.89174 (max o	2	2382; 336	-0.4487049	-0.1666065		1.0
	ZINC03840197	-9.1	443.858	-0.538	9	6	303.92	0.37115	147.99	0.88632	164.2064047263	0.31026	7.3226	-1.7341	0.53333	0.442	0.90672 (max o	4	2010; 1381; 1104; 88	-0.4052041	-0.5336886		1.0
	ZINC04098544	-9.1	422.341	-0.0054996	11	7	274.48	0.49089	186.37	-4.2658	164.2064047263	0.31026	6.7901	-0.017726	0.46667	0.25093		0		0.62374264	-0.1314274		1.0
	ZINC04200781	-9.1	439.387	0.2391	13	1	305.77	0.4455	144.63	1.7605	164.2064047263	0.29086	6.5455	0.82203	0.5625	0.33087	0.90231 (max o	5	2048; 1702; 1417; 72	-0.2527403	-0.3861545		1.0
	ZINC04260029	-9.1	364.404	0.6408	7	1	270.13	0.25414	82.61	7.1993	164.2064047263	0.34473	6.1438	1.8589	0.59259	0.47471	0.86774	1	199	-0.3386544	-0.0833262		1.0
	ZINC04260207	-9.1	401.485	-0.0966	8	2	295.65	0.2551	95.56	6.9255	164.2064047263	0.32095	6.8812	-0.30098	0.62069	0.48273	0.87803 (max o	6	3662; 3491; 2063; 14	-0.2897850	0.05771625		1.0
	ZINC04260232	-9.1	409.441	1.226	8	2	299.98	0.26928	99.18	7.1552	164.2064047263	0.31026	5.5586	3.9516	0.63333	0.4975	0.8715 (max of	2	4549; 199	-0.3083687	-0.1286613		1.0

Structure	Molecule Name	affinity	Total Molweight	cLogP	H-Acceptors	H-Donors	Total Surface Area	Relative PSA	Polar Surface Area	Druglikeness	Kd (nM) @ 20°C	LE from K...	LLE from Kd ...	LELP from Kd (n...	Shape Index	Molecular...	Neighbor Si...	Neig...	Neighbor	Neighbor ...	Neighbor ...	Ring Systems	frequency
	ZINC04260257	-9.1	379.415	1.296	7	2	277.72	0.25486	89.95	7.1847	164.2064047263	0.33242	5.4886	3.8987	0.60714	0.48018	0.85377 (max o	2	578; 199	-0.2885666	-0.0539680		1.0
	ZINC04260989	-9.1	415.449	-0.2356	10	1	305.07	0.3175	99.97	-0.15894	164.2064047263	0.31026	7.0202	-0.75937	0.63333	0.43442	0.9083 (max of	5	6727; 2812; 2064; 11	-0.2283846	-0.1824138		1.0
	ZINC04270563	-9.1	430.420	0.2009	12	1	300.33	0.39234	122.09	2.8002	164.2064047263	0.30025	6.5837	0.66912	0.54839	0.35767	0.91993 (max o	4	2445; 1737; 915; 914	-0.1753038	-0.3549239		1.0
	ZINC05412574	-9.1	449.597	-0.8523	7	2	363.16	0.20415	70.22	-4.1863	164.2064047263	0.28205	7.6369	-3.0218	0.66667	0.46404	0.92348 (max o	2	4568; 1184	-0.0684234	-0.1970164		1.0
	ZINC05433674	-9.1	438.486	-0.4637	10	2	328.32	0.30464	116.64	6.4317	164.2064047263	0.29086	7.2483	-1.5942	0.40625	0.47267	0.97743 (max o	4	2082; 755; 592; 482	0.00739902	0.24144405		1.0
	ZINC06974236	-9.1	423.388	-0.6096	12	1	293.54	0.38427	136.12	2.1914	164.2064047263	0.30025	7.3942	-2.0303	0.51613	0.34582		0		0.8500735	-0.5190851		1.0
	ZINC08299537	-9.1	442.538	-0.0404	9	3	330.12	0.27239	115.43	4.8256	164.2064047263	0.29086	6.825	-0.1389	0.4375	0.49406	0.97533 (max o	3	1177; 753; 599	-0.0166856	0.3118698		1.0
	ZINC08952566	-9.1	448.521	-0.6579	8	3	340.26	0.28472	132.97	5.1302	164.2064047263	0.28205	7.4425	-2.3326	0.57576	0.42317	0.88589 (max o	6	6947; 1802; 1688; 14	0.28663415	0.24660476		1.0
	ZINC12297044	-9.1	353.309	-0.3675	8	0	252.1	0.33883	100.49	2.0507	164.2064047263	0.35799	7.1521	-1.0266	0.53846	0.37049		0		0.93231845	0.3467501		1.0
	ZINC12605293	-9.1	439.475	-1.026	11	2	327.08	0.33934	129.53	6.4317	164.2064047263	0.29086	7.8106	-3.5274	0.40625	0.47267	0.97743 (max o	5	4127; 3694; 755; 592	-0.0035943	0.22577223		1.0
	ZINC12605349	-9.1	440.502	-0.5535	10	2	331.02	0.28977	108.68	6.4317	164.2064047263	0.29086	7.3381	-1.9029	0.40625	0.44127	0.90418 (max o	4	2863; 1179; 938; 754	-0.0745782	0.2934503		1.0
	ZINC12660812	-9.1	416.356	0.5738	8	3	285.39	0.33421	122.52	-0.64903	164.2064047263	0.31026	6.2108	1.8494	0.56667	0.386		0		-0.3988380	-0.9091959		1.0
	ZINC12661173	-9.1	426.428	-0.0088001	10	0	292.43	0.31611	115.38	2.3242	164.2064047263	0.30025	6.7934	-0.02931	0.35484	0.30912	0.96262	1	693	0.7468265	0.43005648		1.0
	ZINC13831130	-9.1	445.430	-0.74	11	4	313.6	0.36623	155.12	5.2463	164.2064047263	0.29086	7.5246	-2.5441	0.5625	0.52316	0.971	1	491	0.96834356	-0.0289918		1.0
	ZINC13831130	-9.1	445.430	-0.74	11	4	313.6	0.36623	155.12	5.2463	164.2064047263	0.29086	7.5246	-2.5441	0.5625	0.52316	0.971	1	490	0.976425	-0.0100739		1.0
	ZINC31154941	-9.1	432.420	-2.0441	11	7	297.42	0.44281	178.53	0.24642	164.2064047263	0.31026	8.8287	-6.5884	0.6	0.4959	0.9167 (max of	3	5252; 1580; 825	-0.3619817	0.45650586		1.0
	ZINC32124407	-9.1	381.459	1.3274	8	3	260.76	0.25487	102.63	0.12494	164.2064047263	0.33242	5.4572	3.9932	0.53571	0.52256		0		-0.8532586	-0.1285312		1.0
	ZINC35018256	-9.1	421.452	0.2979	8	2	294.68	0.2745	102.67	6.8612	164.2064047263	0.30025	6.4867	0.99218	0.45161	0.35813	0.80997	1	515	0.62099355	-0.5707341		1.0
	ZINC38940718	-9.1	438.427	0.2338	10	5	296.68	0.39956	155.14	-11.09	164.2064047263	0.30025	6.5508	0.77869	0.48387	0.35243	0.86981	1	2263	-0.7257438	-0.6024110		1.0
	ZINC40309823	-9.1	363.376	-0.682	8	3	260.21	0.30871	115.29	3.7493	164.2064047263	0.34473	7.4666	-1.9784	0.44444	0.46124	0.98371 (max o	6	2672; 1932; 1931; 13	-0.1892434	-0.4804679		1.0
	ZINC72320204	-9.1	430.408	1.2195	9	4	313.18	0.34638	142.75	-1.6837	164.2064047263	0.30025	5.5651	4.0616	0.6129	0.42624	0.8872	1	627	-0.2365668	0.65000457		1.0
	ZINC77257255	-9.1	448.507	-1.292	10	6	304.26	0.3898	158.3	-2.7899	164.2064047263	0.30025	8.0766	-4.3031	0.51613	0.47116	0.97856	1	324	-0.5872323	-0.6908238		1.0
	ZINC02115540	-9.0	417.436	-1.4569	9	2	317.95	0.34395	133.86	2.2568	194.9604535479	0.30685	8.167	-4.748	0.53333	0.50227	0.92232 (max o	7	2725; 1989; 1983; 19	0.12212715	-2.9380416		172.0
	ZINC12405084	-9.0	426.170	-10.633	15	4	263.6	0.70467	247.54	-44.747	194.9604535479	0.34094	17.343	-31.188	0.51852	0.51581	0.93069 (max o	2	1449; 613	-0.2597092	-0.4481474		44.0
	ZINC12405084	-9.0	425.162	-11.881	15	3	264.78	0.70598	250.37	-40.017	194.9604535479	0.34094	18.591	-34.847	0.51852	0.51562	0.93069 (max o	2	1450; 612	-0.2749754	-0.4134684		44.0
	ZINC31308651	-9.0	441.185	-10.923	16	5	270.33	0.74361	273.56	-47.565	194.9604535479	0.32876	17.633	-33.226	0.5	0.51704	0.93985 (max o	10	2487; 1584; 1506; 14	-0.3140573	-0.4943844		44.0
	ZINC31308651	-9.0	440.177	-12.171	16	4	271.51	0.74472	276.39	-42.835	194.9604535479	0.32876	18.881	-37.02	0.5	0.51686	0.93985 (max o	11	4932; 2488; 1585; 15	-0.3110609	-0.4291063		44.0
	ZINC35466151	-9.0	448.379	7.0003e-4	11	7	299.19	0.45035	186.37	-3.2535	194.9604535479	0.28767	6.7094	0.0024335	0.5	0.3059	0.95709 (max o	3	412; 413; 414	-0.7933872	-0.2775598		38.0
	ZINC05667881	-9.0	400.382	0.3293	8	4	280.14	0.34069	125.68	-3.6206	194.9604535479	0.31743	6.3808	1.0374	0.55172	0.38857	0.95372 (max o	7	1182; 1128; 356; 452	0.26781145	0.563144		30.0
	ZINC04349762	-9.0	416.381	-0.0164	9	5	286.49	0.37886	145.91	-3.6206	194.9604535479	0.30685	6.7265	-0.053446	0.56667	0.39192	0.95837 (max o	9	1230; 1159; 977; 896	0.22629838	0.5599593		30.0
	ZINC04096693	-9.0	446.407	-0.0864	10	5	308.75	0.38394	155.14	-3.4398	194.9604535479	0.28767	6.7965	-0.30035	0.5625	0.39965	0.98343 (max o	12	1994; 1701; 888; 828	0.21631376	0.62768203		30.0
	ZINC04349797	-9.0	430.408	0.2593	9	4	302.4	0.34868	134.91	-3.4398	194.9604535479	0.29695	6.4508	0.87322	0.58065	0.40435	0.96018 (max o	12	2135; 1426; 1203; 78	0.28695995	0.5996279		30.0
	ZINC04097913	-9.0	432.380	-0.3621	10	6	292.84	0.41538	166.14	-3.6206	194.9604535479	0.29695	7.0722	-1.2194	0.54839	0.38725	0.96262 (max o	10	1994; 1701; 973; 828	0.1653631	0.64414996		30.0
	ZINC08844373	-9.0	430.408	0.2593	9	4	302.4	0.34868	134.91	-3.4398	194.9604535479	0.29695	6.4508	0.87322	0.58065	0.40435	0.96018 (max o	13	2135; 1774; 1426; 12	0.2817809	0.633298		30.0
	ZINC03947502	-9.0	446.407	-0.0864	10	5	308.75	0.38394	155.14	-3.4398	194.9604535479	0.28767	6.7965	-0.30035	0.5625	0.40007	0.98343 (max o	13	2890; 2847; 1774; 17	0.26747158	0.65040725		30.0
	ZINC12374721	-9.0	416.381	-0.0164	9	5	286.49	0.37886	145.91	-3.6206	194.9604535479	0.30685	6.7265	-0.053446	0.56667	0.39192	0.95837 (max o	11	7349; 1230; 1159; 97	0.22661744	0.59527177		30.0
	ZINC02140392	-9.0	401.394	-2.0808	9	2	296.45	0.3689	133.86	-1.0092	194.9604535479	0.31743	8.7909	-6.5552	0.55172	0.4288	0.96931 (max o	5	3146; 1092; 1083; 86	0.2208426	0.062944		24.0
	ZINC02119624	-9.0	416.409	-2.1384	10	3	307.97	0.40468	159.88	0.76173	194.9604535479	0.30685	8.8485	-6.969	0.6	0.49976	0.9707 (max of	4	1980; 1979; 1359; 10	0.25957823	0.10278488		24.0
	ZINC04557046	-9.0	351.286	-3.185	9	3	237.81	0.46112	145.58	-4.8874	194.9604535479	0.36822	9.8951	-8.6498	0.48	0.39472	0.94351 (max o	4	2015; 1686; 923; 747	-0.9131244	-0.3870285		24.0
	ZINC04064983	-9.0	352.338	-0.3192	8	4	243.11	0.39258	125.68	-5.4909	194.9604535479	0.36822	7.0293	-0.86688	0.52	0.40384	0.90243 (max o	3	6710; 79; 193	0.35788226	0.43316767		24.0
	ZINC20610284	-9.0	358.369	-0.5377	7	1	262.43	0.30503	106.97	4.9328	194.9604535479	0.35405	7.2478	-1.5187	0.57692	0.54079	0.94622 (max o	5	2129; 1988; 186; 331	0.27041498	0.17509304		22.0
	ZINC08879762	-9.0	358.369	-0.5377	7	1	262.43	0.30503	106.97	4.3186	194.9604535479	0.35405	7.2478	-1.5187	0.53846	0.54079	0.96998 (max o	4	2129; 1777; 1472; 64	0.26640823	0.21339959		22.0
	ZINC02118226	-9.0	388.395	-0.6759	8	0	288.34	0.30155	105.2	4.9812	194.9604535479	0.32876	7.386	-2.0559	0.57143	0.53467	0.94862 (max o	7	2943; 2944; 2729; 16	0.32061163	0.20138437		22.0

Structure	Molecule Name	affinity	Total Molweight	cLogP	H-Acceptors	H-Donors	Total Surface Area	Relative PSA	Polar Surface Area	Druglikeness	Kd (nM) @ 20°C	LE from K...	LLE from Kd ...	LELP from Kd (n...	Shape Index	Molecular...	Neighbor Si...	Neig...	Neighbor	Neighbor ...	Neighbor ...	Ring Systems	frequency
	ZINC02117365	-9.0	388.395	-0.6759	8	0	288.34	0.30155	105.2	4.9812	194.9604535479	0.32876	7.386	-2.0559	0.57143	0.53237	0.94862 (max o	7	2729; 2408; 2122; 19	0.3326923	0.18347156		22.0
	ZINC04416338	-9.0	448.379	0.5798	11	7	295.9	0.45536	186.37	1.9289	194.9604535479	0.28767	6.1303	2.0155	0.40625	0.42191	0.95949 (max o	14	4095; 2776; 2566; 25	-0.7284390	0.2879129		21.0
	ZINC12374719	-9.0	416.381	-0.0164	9	5	286.49	0.37886	145.91	-3.6206	194.9604535479	0.30685	6.7265	-0.053446	0.56667	0.39445	0.95804 (max o	11	1230; 1159; 977; 896;	0.21192536	0.5835854		16.0
	ZINC13549420	-9.0	432.380	-0.3621	10	6	292.84	0.41538	166.14	-3.6206	194.9604535479	0.29695	7.0722	-1.2194	0.54839	0.38973	0.96262 (max o	8	2663; 2120; 977; 973;	0.1611817	0.6226656		16.0
	ZINC35024718	-9.0	446.407	-0.0864	10	5	308.75	0.38394	155.14	-3.4398	194.9604535479	0.28767	6.7965	-0.30035	0.53125	0.31148	0.95465 (max o	7	7349; 1425; 1206; 11	0.15662685	0.5806144		16.0
	ZINC13431045	-9.0	401.160	-11.29	14	4	247.95	0.70688	246.95	-50.931	194.9604535479	0.36822	18	-30.662	0.56	0.6123	0.91964 (max o	11	6526; 5088; 5086; 46	-0.6074596	-0.4156233		14.0
	ZINC13431045	-9.0	400.152	-12.538	14	3	249.13	0.70826	249.78	-46.201	194.9604535479	0.36822	19.248	-34.05	0.56	0.6121	0.91964 (max o	11	6526; 5087; 5089; 46	-0.5934882	-0.4557897		14.0
	ZINC02107060	-9.0	358.369	-0.3718	7	1	263.93	0.3033	106.97	2.2299	194.9604535479	0.35405	7.0819	-1.0501	0.53846	0.54191	0.95257 (max o	4	1080; 866; 363; 474	0.36283812	0.12120847		11.0
	ZINC08879755	-9.0	372.396	-0.1515	7	0	279.84	0.27498	95.97	4.338	194.9604535479	0.34094	6.8616	-0.44436	0.59259	0.54449	0.97149 (max o	7	2850; 2484; 1480; 60	0.37607753	0.18266359		11.0
	ZINC12296445	-9.0	402.422	-0.2215	8	0	302.1	0.28782	105.2	4.338	194.9604535479	0.31743	6.9316	-0.6978	0.55172	0.53177	0.97415 (max o	5	2943; 2944; 477; 549;	0.34894636	0.22032586		11.0
	ZINC02098794	-9.0	399.378	-1.5753	9	2	296.48	0.38272	137.77	1.0496	194.9604535479	0.31743	8.2854	-4.9627	0.55172	0.51596	0.90934 (max o	2	2849; 867	0.12421252	0.22087875		11.0
	ZINC20464491	-9.0	381.391	-1.4204	9	2	276.98	0.33919	129.29	3.6716	194.9604535479	0.32876	8.1305	-4.3204	0.5	0.43733	0.95945 (max o	12	1592; 1591; 1519; 98	0.33875567	-0.1145220		10.0
	ZINC31153561	-9.0	397.390	-2.4217	10	2	286.98	0.36222	138.52	3.2635	194.9604535479	0.31743	9.1318	-7.6292	0.55172	0.43483	0.96931 (max o	11	2192; 1835; 1591; 15	0.33375832	-0.1331322		10.0
	ZINC05413562	-9.0	377.467	-0.2881	6	1	302.65	0.21256	68.85	0.48	194.9604535479	0.32876	6.9982	-0.87631	0.57143	0.42028	0.90776 (max o	5	5776; 4988; 4566; 45	-0.4740474	-0.3730559		10.0
	ZINC35442323	-9.0	401.529	-0.5933	7	3	302.01	0.21681	89.49	5.2368	194.9604535479	0.31743	7.3034	-1.8691	0.62069	0.47484	0.96931 (max o	8	3066; 1598; 1269; 83	-0.5924559	0.08152930		9.0
	ZINC22937006	-9.0	431.555	-0.6633	8	3	324.27	0.23277	98.72	5.2606	194.9604535479	0.29695	7.3734	-2.2337	0.64516	0.48679	0.97199 (max o	10	3876; 1862; 1563; 12	-0.5581566	0.09243153		9.0
	ZINC01591116	-9.0	416.381	-0.4408	9	6	282.84	0.39471	156.91	-2.3989	194.9604535479	0.30685	7.1509	-1.4365	0.5	0.39653	0.95579 (max o	7	2463; 1427; 1164; 96	0.6986481	0.14253329		9.0
	ZINC35442256	-9.0	405.948	0.5228	6	2	298.58	0.17543	69.26	6.2386	194.9604535479	0.32876	6.1873	1.5902	0.57143	0.43841	0.93686 (max o	9	1563; 1303; 1297; 10	-0.5700663	0.11104478		9.0
	ZINC35442257	-9.0	389.493	0.0176	6	2	289.51	0.18093	69.26	4.8633	194.9604535479	0.32876	6.6925	0.053534	0.60714	0.43841	0.9399 (max of	11	2653; 1563; 1304; 12	-0.5263684	0.14116785		9.0
	ZINC35454640	-9.0	445.355	-2.7877	11	5	298.01	0.45589	186.04	1.2558	194.9604535479	0.28767	9.4978	-9.6907	0.53125	0.42384	0.93952	1	1701	0.14859876	0.6544504		9.0
	ZINC36374485	-9.0	417.389	-1.405	9	5	299.31	0.37693	159.74	-3.819	194.9604535479	0.30685	8.1151	-4.5788	0.5	0.40276	0.96343 (max o	3	1324; 525; 526	0.30983457	-0.7829441		9.0
	ZINC11592654	-9.0	445.447	-2.5378	10	7	306.15	0.44906	182.82	2.381	194.9604535479	0.28767	9.2479	-8.822	0.375	0.36573	0.95949 (max o	3	213; 286; 364	0.9166612	0.29941586		8.0
	ZINC04074115	-9.0	428.531	-0.47	8	3	331.28	0.29244	132.97	5.4157	194.9604535479	0.29695	7.1801	-1.5828	0.58065	0.49018	0.94488 (max o	9	6947; 4629; 2409; 18	0.26631325	0.2987883		8.0
	ZINC04654605	-9.0	448.379	-0.4237	11	8	295.54	0.4664	197.37	-1.9977	194.9604535479	0.28767	7.1338	-1.4729	0.5	0.38342	0.95949 (max o	12	3282; 3243; 2828; 28	0.72786134	0.02931626		8.0
	ZINC15229795	-9.0	418.428	-3.6681	8	2	293.46	0.3166	118.64	4.4277	194.9604535479	0.29695	10.378	-12.353	0.41935	0.30375	0.96545 (max o	11	985; 635; 112; 162; 2	0.20529574	-0.5336672		7.0
	ZINC16032784	-9.0	432.455	-3.3242	8	2	305.72	0.30391	118.64	4.3827	194.9604535479	0.28767	10.034	-11.556	0.40625	0.29791	0.9642 (max of	9	1241; 985; 63; 230; 2	0.21848786	-0.5490235		7.0
	ZINC35466141	-9.0	434.396	0.1664	10	6	294.39	0.41319	166.14	-3.7146	194.9604535479	0.29695	6.5437	0.56037	0.51613	0.40063	0.96739 (max o	5	320; 479; 523; 524; 6	0.1022509	-0.4269872		7.0
	ZINC04064929	-9.0	378.376	0.0716	8	4	260.13	0.36689	125.68	-8.4156	194.9604535479	0.34094	6.6385	0.21001	0.51852	0.3974	0.94905 (max o	8	2034; 710; 708; 79; 2	0.32308853	0.45079362		7.0
	ZINC04236053	-9.0	383.402	-2.6763	7	2	272.95	0.26437	97.36	3.5156	194.9604535479	0.32876	9.3864	-8.1405	0.53571	0.35991	0.90883 (max o	5	1138; 901; 723; 261;	-0.2452258	0.271755		7.0
	ZINC05273634	-9.0	416.381	-0.4408	9	6	282.84	0.39471	156.91	-2.3989	194.9604535479	0.30685	7.1509	-1.4365	0.5	0.39702	0.95579 (max o	8	2463; 1427; 1164; 96	0.6810553	0.15287358		7.0
	ZINC08662730	-9.0	446.407	-0.0864	10	5	308.75	0.38394	155.14	-3.4398	194.9604535479	0.28767	6.7965	-0.30034	0.5625	0.44069	0.98532 (max o	11	2890; 1994; 1774; 17	0.23533528	0.66214734		7.0
	ZINC02096011	-9.0	366.324	-2.1249	7	0	270.53	0.3241	115.79	0.42244	194.9604535479	0.34094	8.835	-6.2325	0.62963	0.49266	0.96023 (max o	4	1093; 697; 689; 428	-0.3118747	-0.1078579		7.0
	ZINC02094767	-9.0	380.351	-2.2557	7	0	281.53	0.31144	115.79	1.0433	194.9604535479	0.32876	8.9658	-6.8612	0.53571	0.36854	0.95148 (max o	4	1971; 1093; 697; 428	-0.3324471	-0.1036074		7.0
	ZINC05438612	-9.0	429.452	-1.374	10	2	319.15	0.3739	138.64	1.3984	194.9604535479	0.29695	8.0841	-4.6271	0.58065	0.44379	0.92527 (max o	6	5425; 4576; 4125; 37	-0.6663115	-0.5991761		7.0
	ZINC12296886	-9.0	368.320	-0.9052	8	2	270.34	0.36236	124.99	0.61743	194.9604535479	0.34094	7.6153	-2.655	0.59259	0.43116	0.9617	1	1204	0.8556832	-0.1832894		7.0
	ZINC04073598	-9.0	414.409	1.0044	8	4	292.4	0.3264	125.68	-5.8596	194.9604535479	0.30685	5.7057	3.2733	0.5	0.3091	0.95579 (max o	2	256; 257	0.2723618	0.5279462		6.0
	ZINC12446752	-9.0	416.381	0.6966	9	5	281.79	0.39597	153.75	-4.2232	194.9604535479	0.30685	6.0135	2.2702	0.43333	0.26094	0.95318 (max o	6	1486; 1433; 62; 92; 3	0.6588094	0.01600526		6.0
	ZINC12428585	-9.0	448.379	6.9979e-4	11	7	299.19	0.45035	186.37	-3.2535	194.9604535479	0.28767	6.7094	0.0024326	0.5	0.33768	0.95949 (max o	5	1158; 740; 739; 102;	0.1329978	0.6876687		6.0
	ZINC05409530	-9.0	422.341	-0.4299	11	8	270.83	0.50895	197.37	-3.0467	194.9604535479	0.30685	7.14	-1.401	0.46667	0.34508	0.95053 (max o	9	1698; 1481; 104; 222;	-0.1191146	-0.669463		6.0
	ZINC02121162	-9.0	372.396	-0.2959	7	0	281.63	0.27323	95.97	2.8531	194.9604535479	0.34094	7.006	-0.86789	0.59259	0.44855	0.94622 (max o	5	1650; 1399; 872; 873;	0.11564422	0.18672588		6.0
	ZINC20464491	-9.0	380.383	-1.2315	9	1	287.95	0.36437	128.04	4.1804	194.9604535479	0.32876	7.9416	-3.7458	0.5	0.43733	0.97288 (max o	9	1520; 794; 793; 652;	0.34495342	-0.1507715		6.0
	ZINC31153561	-9.0	396.382	-2.2328	10	1	297.95	0.3857	137.27	3.7739	194.9604535479	0.31743	8.9429	-7.0341	0.55172	0.43483	0.97415 (max o	10	2193; 1836; 1520; 79	0.36168727	-0.1813127		6.0
	ZINC20464491	-9.0	380.383	-1.2315	9	1	287.95	0.36437	128.04	4.1804	194.9604535479	0.32876	7.9416	-3.7458	0.5	0.43733	0.97288 (max o	10	1593; 1521; 794; 793;	0.32419625	-0.1488066		6.0

Structure	Molecule Name	affinity	Total Molweight	cLogP	H-Acceptors	H-Donors	Total Surface Area	Relative PSA	Polar Surface Area	Druglikeness	Kd (nM) @ 20°C	LE from K...	LLE from Kd ...	LELP from Kd (n...	Shape Index	Molecular...	Neighbor Si...	Neig...	Neighbor	Neighbor ...	Neighbor ...	Ring Systems	frequency
	ZINC31153561	-9.0	396.382	-2.2328	10	1	297.95	0.3857	137.27	3.7739	194.9604535479	0.31743	8.9429	-7.0341	0.55172	0.43483	0.97415 (max o	10	2194; 1837; 1521; 79	0.34114906	-0.1918984		6.0
	ZINC20758698	-9.0	398.418	-1.4992	9	3	271.21	0.3831	130.83	4.3284	194.9604535479	0.31743	8.2093	-4.723	0.41379	0.24097	0.98525 (max o	10	4270; 3418; 1880; 18	0.10105873	0.85869277		5.0
	ZINC04349277	-9.0	400.382	1.0378	8	4	280.14	0.34069	125.68	-3.2535	194.9604535479	0.31743	5.6723	3.2694	0.55172	0.37921	0.94543 (max o	4	1228; 737; 451; 489	0.340863	0.56367016		5.0
	ZINC04260932	-9.0	372.380	0.1525	9	1	271.74	0.35457	104.57	-3.2693	194.9604535479	0.34094	6.5576	0.44729	0.62963	0.3526	0.94002 (max o	3	1735; 1724; 911	-0.2920824	-0.2406460		5.0
	ZINC06673848	-9.0	350.373	2.1326	6	2	271.88	0.25379	76.66	2.9999	194.9604535479	0.35405	4.5775	6.0234	0.61538	0.40671	0.87452	1	1754	0.24362253	-0.9496151		5.0
	ZINC08488910	-9.0	403.477	-2.4173	8	3	299.38	0.29812	126.38	5.4943	194.9604535479	0.31743	9.1274	-7.6153	0.44828	0.45871	0.9892 (max of	4	2908; 2039; 1477; 11	0.519889	-0.6949087		5.0
	ZINC35442585	-9.0	419.519	-1.3652	7	3	323.46	0.23289	77.32	4.6625	194.9604535479	0.30685	8.0753	-4.4491	0.6	0.4608	0.95031 (max o	4	1885; 1053; 838; 837	0.02826162	0.5564828		5.0
	ZINC35456738	-9.0	434.396	0.1664	10	6	294.39	0.41319	166.14	-3.7146	194.9604535479	0.29695	6.5437	0.56037	0.51613	0.3197	0.96502 (max o	5	757; 142; 177; 409; 6	0.13738084	-0.3467424		4.0
	ZINC15957169	-9.0	432.455	-3.3242	8	2	305.72	0.30391	118.64	4.3827	194.9604535479	0.28767	10.034	-11.556	0.40625	0.29534	0.96186 (max o	9	985; 799; 798; 30; 63	0.15441628	-0.5508876		4.0
	ZINC16032785	-9.0	432.455	-3.3242	8	2	305.72	0.30391	118.64	4.3827	194.9604535479	0.28767	10.034	-11.556	0.40625	0.29534	0.96186 (max o	9	1241; 799; 798; 30; 6	0.18727961	-0.5979339		4.0
	ZINC35458045	-9.0	353.305	-0.3835	7	2	238.43	0.37579	123.96	2.5684	194.9604535479	0.35405	7.0936	-1.0832	0.46154	0.26377	0.97533 (max o	3	1916; 1609; 521	-0.8530733	-0.4734453		4.0
	ZINC04259806	-9.0	411.394	0.2385	12	1	271.58	0.47802	144.3	1.974	194.9604535479	0.32876	6.4716	0.72545	0.53571	0.3459	0.89169 (max o	4	1420; 1417; 729; 577	-0.2005731	-0.4255888		4.0
	ZINC04259919	-9.0	432.368	-2.085	13	1	301.29	0.46998	158.98	0.95824	194.9604535479	0.29695	8.7951	-7.0214	0.58065	0.37405	0.95983 (max o	5	3218; 1417; 729; 441	-0.2375244	-0.4195782		4.0
	ZINC04556882	-9.0	400.307	-3.8839	12	3	262.82	0.51275	163.91	-12.61	194.9604535479	0.34094	10.594	-11.392	0.55556	0.2929	0.92739 (max o	3	2826; 1731; 746	0.06410897	-0.9753304		4.0
	ZINC13430722	-9.0	415.426	-4.3719	12	5	256.85	0.50602	182.48	-2.4143	194.9604535479	0.32876	11.082	-13.298	0.46429	0.44266	0.95983 (max o	3	2171; 1505; 780	-0.5163775	-0.6945632		4.0
	ZINC15958382	-9.0	424.475	-3.1866	8	2	300	0.3097	118.64	-1.065	194.9604535479	0.29695	9.8967	-10.731	0.3871	0.28378	0.96018 (max o	5	1528; 796; 228; 297;	0.34804437	-0.25573		4.0
	ZINC05167060	-9.0	384.339	1.2744	8	5	253.71	0.41236	152.36	1.6245	194.9604535479	0.32876	5.4357	3.8763	0.42857	0.25957	0.94862	1	6	-0.8438301	-0.2219150		3.0
	ZINC06733708	-9.0	388.371	0.9984	8	4	267.69	0.35653	125.68	-4.2822	194.9604535479	0.32876	5.7117	3.0368	0.5	0.25237	0.91482	1	436	-0.1423742	-0.8343216		3.0
	ZINC04098749	-9.0	446.407	1.3578	10	4	293.2	0.38336	136.3	-4.5704	194.9604535479	0.28767	5.3523	4.72	0.53125	0.28539	0.95465 (max o	4	2188; 159; 311; 492	0.34239092	0.84148264		3.0
	ZINC35456730	-9.0	434.396	0.1664	10	6	294.39	0.41319	166.14	-3.7146	194.9604535479	0.29695	6.5437	0.56037	0.51613	0.31775	0.96502 (max o	5	757; 200; 234; 241; 6	0.10823307	-0.3692911		3.0
	ZINC35456511	-9.0	413.365	-2.6382	11	3	291.2	0.44784	166.17	1.3595	194.9604535479	0.30685	9.3483	-8.5978	0.53333	0.40341	0.94515 (max o	8	1054; 789; 669; 221;	0.25060818	-0.7800647		3.0
	ZINC15229795	-9.0	419.436	-1.8658	8	2	289.74	0.30783	123.22	3.5	194.9604535479	0.29695	8.5759	-6.2833	0.41935	0.31294	0.96545 (max o	2	374; 378	-0.6852301	0.41779903		3.0
	ZINC03841165	-9.0	384.435	-0.1739	8	4	280.55	0.33035	114.53	5.8575	194.9604535479	0.32876	6.884	-0.52895	0.57143	0.42979	0.8973 (max of	3	4043; 2755; 750	-0.4173557	-0.3546854		3.0
	ZINC12603943	-9.0	436.507	-0.8429	10	5	331.48	0.33248	132.39	3.1857	194.9604535479	0.29695	7.553	-2.8385	0.67742	0.58406	0.95031 (max o	7	3739; 2928; 2506; 15	0.25606567	-0.3896047		3.0
	ZINC26745309	-9.0	422.385	0.1214	10	7	282.99	0.43005	169.3	-4.0605	194.9604535479	0.30685	6.5887	0.39564	0.53333	0.3809	0.96092 (max o	7	1937; 1332; 1306; 12	0.4159965	-0.8151706		3.0
	ZINC67911079	-9.0	440.400	-2.7759	11	4	252.24	0.52141	169.05	-2.076	194.9604535479	0.29695	9.486	-9.3481	0.32258	0.091409	0.91162	1	2644	-0.2498989	-0.9414429		3.0
	ZINC04044048	-9.0	368.364	-2.0163	7	0	240.78	0.30696	88.13	1.713	194.9604535479	0.34094	8.7264	-5.9139	0.44444	0.17486	0.93373 (max o	4	1394; 1393; 893; 340	-0.6619401	-0.0293978		2.0
	ZINC08295771	-9.0	387.439	0.178	9	3	292.06	0.32062	110.85	5.4831	194.9604535479	0.32876	6.5321	0.54142	0.53571	0.5217	0.89009	1	939	-0.7843982	-0.1088177		2.0
	ZINC03839539	-9.0	440.406	-1.8116	9	2	314.16	0.32757	127.87	3.4689	194.9604535479	0.28767	8.5217	-6.2975	0.5625	0.35444	0.93686 (max o	5	3272; 1672; 1454; 57	-0.2624788	0.43088344		2.0
	ZINC14855678	-9.0	418.397	0.8908	9	5	301.78	0.35967	145.91	-1.6837	194.9604535479	0.30685	5.8193	2.9031	0.63333	0.43398	0.93409 (max o	3	1513; 493; 536	-0.2414416	0.629156		2.0
	ZINC70712688	-9.0	440.400	-2.7759	11	4	252.24	0.52141	169.05	-2.076	194.9604535479	0.29695	9.486	-9.3481	0.32258	0.091409	0.91162 (max o	2	3544; 534	-0.6673106	-0.4548689		2.0
	ZINC72320177	-9.0	390.343	-1.6259	9	5	240	0.50946	164.89	2.8143	194.9604535479	0.32876	8.336	-4.9455	0.39286	0.31333	0.99715	1	535	-0.8658099	0.36295813		2.0
	ZINC02121209	-9.0	358.369	-0.7503	7	0	267.87	0.28727	95.97	-1.6318	194.9604535479	0.35405	7.4604	-2.1192	0.57692	0.43833	0.94622 (max o	4	4901; 1650; 872; 100	0.1529569	0.19512683		2.0
	ZINC04044098	-9.0	352.365	-1.6024	6	0	230.78	0.27693	78.9	1.607	194.9604535479	0.35405	8.3125	-4.5259	0.42308	0.1538	0.93046 (max o	4	1117; 894; 893; 340	-0.6996152	-0.0108032		2.0
	ZINC04044136	-9.0	419.492	-1.2533	7	3	284.29	0.31538	127.12	0.12303	194.9604535479	0.30685	7.9634	-4.0845	0.46667	0.36524	0.95318 (max o	4	2031; 1395; 891; 134	0.2689004	0.8653169		2.0
	ZINC04260255	-9.0	369.376	0.4847	8	2	267.41	0.31745	103.09	7.31	194.9604535479	0.34094	6.2254	1.4217	0.59259	0.46485	0.96297 (max o	2	3662; 448	-0.2812691	-0.0143595		2.0
	ZINC05410405	-9.0	428.486	-0.3298	8	3	327.96	0.26445	86.19	4.6695	194.9604535479	0.29695	7.0399	-1.1106	0.6129	0.44593	0.94734 (max o	2	2446; 1435	0.28482026	-0.6265814		2.0
	ZINC08299569	-9.0	394.494	-4.4109	9	3	297.87	0.3245	126.46	-0.44346	194.9604535479	0.32876	11.121	-13.417	0.5	0.50631	0.95983 (max o	5	4574; 3691; 2838; 20	-0.0842303	0.18209167		2.0
	ZINC08681833	-9.0	418.397	0.5121	9	5	288.04	0.37682	145.91	-3.7146	194.9604535479	0.30685	6.198	1.6689	0.53333	0.43031	0.97304 (max o	8	1223; 978; 954; 945;	0.05654423	-0.4320942		2.0
	ZINC13513944	-9.0	443.419	-8.3663	13	6	324.07	0.50853	212.93	4.3321	194.9604535479	0.28767	15.076	-29.083	0.625	0.56942	0.98148 (max o	5	2956; 2955; 2555; 90	0.8235151	0.2490483		2.0
	ZINC20113591	-9.0	403.414	-2.3044	10	3	302.6	0.37403	110.64	4.1076	194.9604535479	0.31743	9.0145	-7.2596	0.48276	0.41168	0.98525 (max o	3	803; 802; 639	-0.1984127	0.5844856		2.0
	ZINC20113591	-9.0	403.414	-2.3044	10	3	302.6	0.37403	110.64	4.1076	194.9604535479	0.31743	9.0145	-7.2596	0.48276	0.41168	0.98525 (max o	4	803; 801; 802; 638	-0.2051250	0.62126416		2.0
	ZINC20463655	-9.0	438.546	0.1871	7	2	322.28	0.18921	78.85	6.2154	194.9604535479	0.28767	6.523	0.6504	0.59375	0.46992	0.94488 (max o	3	6765; 5851; 1015	0.44853118	-0.1972236		2.0

Structure	Molecule Name	affinity	Total Molweight	cLogP	H-Acceptors	H-Donors	Total Surface Area	Relative PSA	Polar Surface Area	Druglikeness	Kd (nM) @ 20°C	LE from K...	LLE from Kd ...	LELP from Kd (n...	Shape Index	Molecular...	Neighbor Si...	Neig...	Neighbor	Neighbor ...	Neighbor ...	Ring Systems	frequency
	ZINC20504134	-9.0	449.594	0.4387	8	5	318.72	0.29242	133.33	6.0621	194.9604535479	0.29695	6.2714	1.4774	0.54839	0.4836	0.92566 (max o	2	3009; 1006	-0.6597361	0.6935858		2.0
	ZINC31158607	-9.0	391.482	-0.6403	6	3	280.75	0.30016	113.35	-3.3875	194.9604535479	0.32876	7.3504	-1.9476	0.42857	0.388	0.9678 (max of	4	4368; 3459; 3038; 22	-0.1476275	0.44166538		2.0
	ZINC31159157	-9.0	420.413	0.4007	9	6	299.15	0.37319	156.91	-4.8731	194.9604535479	0.30685	6.3094	1.3059	0.6	0.4825	0.92547 (max o	2	2909; 2664	0.4021184	0.4983705		2.0
	ZINC35270817	-9.0	360.361	-0.1543	7	4	232.58	0.41332	127.59	0.14074	194.9604535479	0.35405	6.8644	-0.43581	0.42308	0.29718	0.96721 (max o	3	181; 518; 660	-0.8323804	0.12203971		2.0
	ZINC35270816	-9.0	360.361	-0.1543	7	4	232.58	0.41332	127.59	0.14074	194.9604535479	0.35405	6.8644	-0.43581	0.42308	0.29718	0.96721 (max o	3	661; 181; 518	-0.8151573	0.10927693		2.0
	ZINC35442297	-9.0	405.492	-0.8044	7	2	299.51	0.20827	78.49	3.0017	194.9604535479	0.31743	7.5145	-2.5341	0.62069	0.4748	0.97415 (max o	10	2653; 1598; 1301; 13	-0.5661685	0.132791		2.0
	ZINC35456515	-9.0	413.365	-2.6382	11	3	291.2	0.44784	166.17	1.3595	194.9604535479	0.30685	9.3483	-8.5978	0.53333	0.40587	0.95053 (max o	8	1054; 789; 221; 242;	0.26787177	-0.7898662		2.0
	ZINC35457544	-9.0	370.353	-1.0161	9	5	254.3	0.42682	145.91	-4.9227	194.9604535479	0.35405	7.7262	-2.8699	0.46154	0.49901	0.95257 (max o	3	1607; 1308; 1055	-0.5926812	0.7841188		2.0
	ZINC36532582	-9.0	448.379	-0.0535	11	7	299.19	0.45035	186.37	-3.3789	194.9604535479	0.28767	6.7636	-0.18598	0.53125	0.27742	0.95709 (max o	3	2321; 1944; 1624	-0.3069516	-0.6809048		2.0
	ZINC49181878	-9.0	446.411	0.0678	11	3	310.24	0.38822	147.35	6.7757	194.9604535479	0.28767	6.6423	0.23569	0.5	0.50056	0.95465 (max o	3	4428; 3952; 849	-0.1501624	-0.9779036		2.0
	ZINC49600055	-9.0	416.421	-1.5798	10	6	291.9	0.4063	158.3	-5.0311	194.9604535479	0.31743	8.2899	-4.9769	0.58621	0.51506	0.96983 (max o	9	4409; 1583; 1331; 12	-0.4765507	0.6754162		2.0
	ZINC00892819	-9.0	371.352	0.2845	10	1	265.09	0.36033	106	4.3573	194.9604535479	0.34094	6.4256	0.83446	0.55556	0.52261	0.84855	1	540	0.69748855	-0.5395885		1.0
	ZINC00892819	-9.0	372.360	-1.6933	10	2	254.12	0.33272	107.25	3.7793	194.9604535479	0.34094	8.4034	-4.9666	0.55556	0.52261	0.84855	1	539	0.6879612	-0.5599925		1.0
	ZINC02104788	-9.0	425.392	-2.8155	9	0	308.41	0.35142	140.01	-11.241	194.9604535479	0.29695	9.5256	-9.4815	0.48387	0.50581	0.90474 (max o	4	1369; 1090; 1079; 86	0.19272465	0.5077915		1.0
	ZINC02159930	-9.0	440.411	0.5411	11	1	308.06	0.34834	125.56	5.3492	194.9604535479	0.28767	6.169	1.881	0.4375	0.38283		0		-0.6230518	-0.7769370		1.0
	ZINC03839306	-9.0	441.530	-0.015	9	2	321.58	0.27691	103.75	3.1781	194.9604535479	0.28767	6.7251	-0.052143	0.5	0.33011	0.93139 (max o	9	3818; 3709; 2749; 20	-0.6100496	-0.0243489		1.0
	ZINC04042647	-9.0	364.377	-0.3499	7	1	232.66	0.51509	155.36	3.2314	194.9604535479	0.38356	7.06	-0.91225	0.41667	0.25917	0.92619 (max o	3	5707; 4070; 3195	0.60644436	-0.4969814		1.0
	ZINC04081999	-9.0	370.380	-0.5498	7	0	269.34	0.2857	95.97	1.5147	194.9604535479	0.34094	7.2599	-1.6126	0.55556	0.45482	0.8934 (max of	3	3198; 131; 210	0.171788	0.0908006		1.0
	ZINC04082050	-9.0	412.821	0.6776	8	4	275.55	0.34636	125.68	-8.307	194.9604535479	0.32876	6.0325	2.0611	0.5	0.30943	0.92464 (max o	2	710; 321	0.38077232	0.44038796		1.0
	ZINC04097843	-9.0	408.358	-0.304	10	4	280.69	0.42591	148.05	-3.1381	194.9604535479	0.31743	7.0141	-0.9577	0.48276	0.4103	0.83981	1	5192	0.11264909	-0.6438996		1.0
	ZINC04236167	-9.0	370.340	-2.5153	9	1	262.5	0.37756	122.99	1.8802	194.9604535479	0.34094	9.2254	-7.3775	0.55556	0.38539	0.85728	1	6140	-0.4314416	-0.5578303		1.0
	ZINC04259902	-9.0	440.478	-0.9849	10	1	322.98	0.34553	131.29	-0.33312	194.9604535479	0.28767	7.695	-3.4237	0.53125	0.39738	0.92304 (max o	2	2438; 1415	-0.3000549	-0.3251705		1.0
	ZINC05433668	-9.0	439.475	-1.4106	11	2	327.08	0.33934	129.53	6.4317	194.9604535479	0.28767	8.1207	-4.9036	0.40625	0.43085	0.96186 (max o	7	2863; 2203; 2089; 11	-0.0142183	0.2731723		1.0
	ZINC05438239	-9.0	384.415	-1.4591	9	2	275.94	0.33913	107.87	-4.6497	194.9604535479	0.32876	8.1692	-4.4381	0.60714	0.34709	0.87121 (max o	2	4122; 2078	-0.1269364	-0.1926649		1.0
	ZINC08299559	-9.0	443.526	-1.0413	10	3	328.88	0.30677	128.32	4.8256	194.9604535479	0.28767	7.7514	-3.6198	0.4375	0.49406	0.97533 (max o	5	1438; 1177; 753; 88;	-0.00267647	0.2996592		1.0
	ZINC12428080	-9.0	429.391	-2.2091	10	3	289.15	0.36151	148.46	5.9839	194.9604535479	0.28767	8.9192	-7.6793	0.46875	0.24166	0.90114	1	615	0.8367378	-0.1234274		1.0
	ZINC12428080	-9.0	428.383	-3.0995	10	2	290.44	0.36434	154.78	3.9399	194.9604535479	0.28767	9.8096	-10.775	0.46875	0.24166	0.90114	1	614	0.8358137	-0.1521360		1.0
	ZINC12902346	-9.0	418.428	-0.9866	8	0	301.93	0.27162	102.34	4.5283	194.9604535479	0.29695	7.6967	-3.3225	0.51613	0.43159	0.86238 (max o	2	3348; 778	0.13318433	0.5315064		1.0
	ZINC13511674	-9.0	444.427	-9.36	13	6	313.65	0.48889	214.87	3.738	194.9604535479	0.28767	16.07	-32.537	0.625	0.57143	0.94718	1	1233	0.14158274	0.9647361		1.0
	ZINC14823616	-9.0	378.419	-0.6619	7	3	245.98	0.39694	112.05	0.16014	194.9604535479	0.34094	7.372	-1.9414	0.44444	0.303		0		0.71642405	0.21813488		1.0
	ZINC15672292	-9.0	420.444	-0.6245	8	1	299.33	0.28397	109.85	7.4098	194.9604535479	0.29695	7.3346	-2.1031	0.54839	0.43455		0		-0.9542903	0.24785979		1.0
	ZINC15674018	-9.0	359.409	-2.3241	9	2	281.81	0.37355	109.68	5.0059	194.9604535479	0.35405	9.0342	-6.5643	0.57692	0.38908		0		-0.9528492	0.07345599		1.0
	ZINC15708143	-9.0	442.562	-3.8562	9	4	329.52	0.2632	100.33	7.9995	194.9604535479	0.28767	10.566	-13.405	0.59375	0.47257	0.93157 (max o	3	6771; 3815; 1559	-0.2310940	0.13756648		1.0
	ZINC20113591	-9.0	402.406	-0.1947	10	2	288.56	0.34357	109.44	5.9634	194.9604535479	0.31743	6.9048	-0.61337	0.48276	0.41168	0.97649 (max o	3	2343; 999; 801	-0.2201605	0.63583624		1.0
	ZINC20463402	-9.0	436.554	-0.4752	7	1	330.34	0.15442	81.06	8.4675	194.9604535479	0.28767	7.1853	-1.6519	0.5625	0.54382	0.89799 (max o	10	4723; 3387; 3386; 29	-0.1601659	-0.2089985		1.0
	ZINC20759700	-9.0	412.468	-0.6242	9	4	290.09	0.29832	109.83	6.2643	194.9604535479	0.30685	7.3343	-2.0342	0.53333	0.46118	0.82392	1	3419	0.6977272	0.67597973		1.0
	ZINC33831307	-9.0	448.379	6.9991e-4	11	7	299.19	0.45035	186.37	-3.2535	194.9604535479	0.28767	6.7094	0.002433	0.5	0.3054	0.9497 (max of	3	3458; 740; 136	0.10812329	0.6374714		1.0
	ZINC36728548	-9.0	392.359	-1.3716	9	6	257.5	0.43355	156.91	-2.2179	194.9604535479	0.32876	8.0817	-4.172	0.46429	0.35879	0.9428 (max of	4	3080; 1926; 264; 415	-0.3070465	0.59685355		1.0
	ZINC72332909	-9.0	440.467	-0.4019	10	1	310.66	0.36973	132.45	-4.4543	194.9604535479	0.29695	7.112	-1.3534	0.51613	0.50333	0.92304	1	2320	0.23712002	-0.9232949		1.0
	ZINC04228246	-8.9	425.270	-8.4777	15	3	262.23	0.70705	253.29	-42.416	231.4743965740	0.33715	15.113	-25.145	0.51852	0.48501	0.98593 (max o	11	6792; 3360; 3359; 33	-0.615339	0.43582395		67.0
	ZINC13436561	-8.9	425.186	-10.071	15	4	265.24	0.70849	253.33	-44.039	231.4743965740	0.33715	16.707	-29.872	0.51852	0.52391	0.94605 (max o	10	2487; 1584; 1506; 14	-0.2739942	-0.5089092		44.0
	ZINC13436561	-8.9	424.178	-11.319	15	3	266.42	0.70978	256.16	-39.309	231.4743965740	0.33715	17.954	-33.572	0.51852	0.52373	0.94605 (max o	10	2488; 1585; 1507; 14	-0.2989741	-0.4609492		44.0
	ZINC04349286	-8.9	400.382	1.0378	8	4	280.14	0.34069	125.68	-3.2535	231.4743965740	0.3139	5.5977	3.3061	0.51724	0.37921	0.9617 (max of	6	1228; 293; 360; 451;	-0.2939905	0.5307588		38.0

Structure	Molecule Name	affinity	Total Molweight	cLogP	H-Acceptors	H-Donors	Total Surface Area	Relative PSA	Polar Surface Area	Druglikeness	Kd (nM) @ 20°C	LE from K...	LLE from Kd ...	LELP from Kd (n...	Shape Index	Molecular...	Neighbor Si...	Neig...	Neighbor	Neighbor ...	Neighbor ...	Ring Systems	frequency
	ZINC04349782	-8.9	432.380	-0.3621	10	6	292.84	0.41538	166.14	-3.6206	231.4743965740	0.29365	6.9976	-1.2331	0.54839	0.38725	0.96018 (max o	8	1159; 973; 273; 453;	0.19651735	0.6173037		30.0
	ZINC33831794	-8.9	446.407	-0.0864	10	5	308.75	0.38394	155.14	-3.4398	231.4743965740	0.28447	6.7219	-0.30372	0.5625	0.39552	0.93418 (max o	2	896; 685	0.21264134	0.54287314		30.0
	ZINC01081322	-8.9	430.408	0.2593	9	4	302.4	0.34868	134.91	-3.4398	231.4743965740	0.29365	6.3762	0.88303	0.58065	0.40392	0.96262 (max o	13	2135; 1426; 1203; 11	0.23410924	0.57786006		30.0
	ZINC04349821	-8.9	446.407	-0.0864	10	5	308.75	0.38394	155.14	-3.4398	231.4743965740	0.28447	6.7219	-0.30372	0.5625	0.39965	0.96186 (max o	11	1994; 1773; 1426; 88	0.24763726	0.64693004		30.0
	ZINC04349776	-8.9	432.380	-0.3621	10	6	292.84	0.41538	166.14	-3.6206	231.4743965740	0.29365	6.9976	-1.2331	0.54839	0.3877	0.96018 (max o	9	2847; 1773; 973; 273	0.20829628	0.64834756		30.0
	ZINC01565353	-8.9	446.407	-0.0864	10	5	308.75	0.38394	155.14	-3.4398	231.4743965740	0.28447	6.7219	-0.30372	0.5625	0.39965	0.96186 (max o	11	2890; 1994; 1774; 12	0.23859133	0.63004434		30.0
	ZINC04349794	-8.9	446.407	-0.0864	10	5	308.75	0.38394	155.14	-3.4398	231.4743965740	0.28447	6.7219	-0.30372	0.53125	0.30824	0.9497 (max of	9	7349; 1425; 1206; 11	0.15884928	0.6015594		30.0
	ZINC08918578	-8.9	431.408	1.3955	13	6	305.76	0.49195	193.39	1.4288	231.4743965740	0.29365	5.24	4.7523	0.48387	0.31466	0.89103 (max o	2	3314; 285	-0.1868374	-0.7355544		24.0
	ZINC02112653	-8.9	429.448	-1.2976	9	2	322.71	0.33888	133.86	2.5338	231.4743965740	0.29365	7.9331	-4.4189	0.58065	0.53884	0.95804 (max o	6	3145; 3143; 1974; 19	0.19099084	-0.0255793		24.0
	ZINC04557045	-8.9	351.286	-3.185	9	3	237.81	0.46112	145.58	-4.8874	231.4743965740	0.36412	9.8205	-8.747	0.48	0.39472	0.94351 (max o	4	2015; 1686; 923; 585	-0.8955499	-0.4106056		24.0
	ZINC38570094	-8.9	446.478	-1.5067	10	2	333.59	0.33847	137.1	-0.50421	231.4743965740	0.28447	8.1422	-5.2965	0.5625	0.47465	0.97319 (max o	12	4193; 3943; 3781; 26	0.51502985	0.21871695		22.0
	ZINC12881109	-8.9	446.478	-1.5067	10	2	333.59	0.33847	137.1	-0.0023535	231.4743965740	0.28447	8.1422	-5.2965	0.5625	0.47785	0.97533 (max o	12	6226; 4193; 3781; 26	0.535296	0.21159789		22.0
	ZINC13514961	-8.9	430.408	0.2593	9	4	302.4	0.34868	134.91	-3.4398	231.4743965740	0.29365	6.3762	0.88303	0.58065	0.4063	0.96262 (max o	9	2135; 1426; 1203; 97	0.2655949	0.59823006		16.0
	ZINC08844372	-8.9	430.408	0.2593	9	4	302.4	0.34868	134.91	-3.4398	231.4743965740	0.29365	6.3762	0.88303	0.58065	0.40673	0.96018 (max o	12	2890; 2135; 1426; 12	0.26056686	0.6312431		16.0
	ZINC08299538	-8.9	406.436	-4.2104	9	3	296.18	0.32636	126.46	2.2859	231.4743965740	0.3139	10.846	-13.413	0.48276	0.5205	0.96428 (max o	5	3722; 2861; 2088; 18	0.08108093	0.34503615		14.0
	ZINC03871879	-8.9	376.388	-3.4633	8	1	256.5	0.4414	154.97	10.967	231.4743965740	0.35012	10.099	-9.8918	0.5	0.35316	0.96721 (max o	5	4982; 3617; 2771; 23	0.06144226	-0.0759804		12.0
	ZINC15675802	-8.9	381.391	-1.4204	9	2	276.98	0.33919	129.29	3.6716	231.4743965740	0.32511	8.0559	-4.369	0.53571	0.43733	0.98166 (max o	10	2192; 1591; 1519; 98	0.34558293	-0.0945641		10.0
	ZINC03839675	-8.9	416.380	-1.6398	10	7	287.85	0.44492	174.01	0.57717	231.4743965740	0.3139	8.2753	-5.224	0.55172	0.47446	0.92486 (max o	3	6089; 1105; 883	-0.4042439	-0.3732563		10.0
	ZINC22936940	-8.9	428.599	0.1552	7	2	331.74	0.1686	72.5	4.8345	231.4743965740	0.29365	6.4803	0.52852	0.6129	0.49684	0.95243 (max o	10	4350; 3438; 3028; 15	-0.5653449	0.05328178		9.0
	ZINC04098745	-8.9	416.381	-0.4408	9	6	282.84	0.39471	156.91	-2.3989	231.4743965740	0.30344	7.0763	-1.4527	0.5	0.39653	0.95053 (max o	8	3464; 2463; 1427; 11	0.73390895	0.14134096		9.0
	ZINC06041522	-8.9	432.380	-0.7865	10	7	289.19	0.43134	177.14	-2.3989	231.4743965740	0.29365	7.422	-2.6784	0.48387	0.39235	0.97423 (max o	8	5883; 2481; 2095; 12	0.66076964	0.21059336		9.0
	ZINC35442255	-8.9	371.503	-0.0832	6	2	283.16	0.18498	69.26	6.2033	231.4743965740	0.33715	6.7187	-0.24677	0.59259	0.43414	0.96343 (max o	9	3066; 1599; 1598; 15	-0.5317083	0.12309326		9.0
	ZINC22936717	-8.9	414.572	-0.1868	7	2	317.98	0.17589	72.5	6.8211	231.4743965740	0.30344	6.8223	-0.61561	0.6	0.46492	0.93121 (max o	12	4350; 4346; 3438; 30	-0.5382892	0.086524		9.0
	ZINC32123758	-8.9	419.405	-1.356	9	4	302.48	0.36273	148.74	-3.5771	231.4743965740	0.30344	7.9915	-4.4688	0.6	0.47551	0.9167	1	829	0.36799908	0.587935		9.0
	ZINC32123758	-8.9	420.413	0.222	9	5	301.3	0.36024	145.91	-3.6574	231.4743965740	0.30344	6.4135	0.73162	0.6	0.47551	0.97304 (max o	9	2909; 2517; 2358; 20	0.36184856	0.5676252		9.0
	ZINC11592620	-8.9	445.447	-2.6981	10	7	305.32	0.45028	182.82	2.96	231.4743965740	0.28447	9.3336	-9.4846	0.375	0.36615	0.94464 (max o	3	1788; 107; 206	0.1722037	-0.800018		8.0
	ZINC34933704	-8.9	432.380	-0.078	10	7	289.19	0.43134	177.14	-1.9977	231.4743965740	0.29365	6.7135	-0.26562	0.51613	0.3816	0.95771 (max o	11	3788; 3213; 2453; 18	0.70013946	0.07904145		8.0
	ZINC04073376	-8.9	412.821	0.6776	8	4	275.55	0.34636	125.68	-8.307	231.4743965740	0.32511	5.9579	2.0842	0.5	0.30543	0.93686 (max o	6	1757; 321; 342; 439;	0.37443146	0.47506815		7.0
	ZINC04098672	-8.9	432.380	0.3509	10	6	288.14	0.43271	173.98	-4.2232	231.4743965740	0.29365	6.2846	1.195	0.45161	0.25616	0.93139 (max o	4	1433; 205; 370; 480	0.6504601	0.05978369		7.0
	ZINC04236052	-8.9	399.857	-2.1711	7	2	282.02	0.25587	97.36	4.874	231.4743965740	0.32511	8.8066	-6.678	0.57143	0.35669	0.9339 (max of	5	2055; 901; 261; 346;	-0.2371645	0.25002468		7.0
	ZINC15259626	-8.9	432.380	-0.7865	10	7	289.19	0.43134	177.14	-2.3989	231.4743965740	0.29365	7.422	-2.6784	0.48387	0.39186	0.95771 (max o	7	5883; 2481; 2095; 12	0.6415466	0.20322686		7.0
	ZINC06143118	-8.9	432.380	-0.3621	10	6	292.84	0.41538	166.14	-3.6206	231.4743965740	0.29365	6.9976	-1.2331	0.54839	0.43127	0.95983 (max o	12	3558; 2847; 2138; 21	0.18614624	0.67420566		7.0
	ZINC02092891	-8.9	380.351	-1.6705	7	0	284.29	0.30842	115.79	-0.64125	231.4743965740	0.32511	8.306	-5.1382	0.64286	0.49252	0.96023 (max o	2	697; 543	-0.3020777	-0.0710079		7.0
	ZINC02147918	-8.9	380.351	-1.781	7	0	282.79	0.31005	115.79	0.42244	231.4743965740	0.32511	8.4165	-5.4781	0.60714	0.41216	0.91157 (max o	4	1971; 542; 543; 689	-0.3174072	-0.0874790		7.0
	ZINC04081925	-8.9	356.357	-1.0184	8	1	249.02	0.33929	105.77	6.0912	231.4743965740	0.35012	7.6539	-2.9087	0.5	0.29006	0.90299 (max o	3	4078; 2785; 948	-0.4731301	0.3053437		7.0
	ZINC04349511	-8.9	448.379	6.9979e-4	11	7	299.19	0.45035	186.37	-3.2535	231.4743965740	0.28447	6.6348	0.00246	0.5	0.33768	0.95949 (max o	5	1158; 740; 148; 339;	0.13983425	0.66937095		6.0
	ZINC04349517	-8.9	448.379	6.9979e-4	11	7	299.19	0.45035	186.37	-3.2535	231.4743965740	0.28447	6.6348	0.00246	0.5	0.33722	0.95219 (max o	7	1279; 1158; 136; 148	0.1270015	0.65021133		6.0
	ZINC15675802	-8.9	380.383	-1.2315	9	1	287.95	0.36437	128.04	4.1804	231.4743965740	0.32511	7.867	-3.7879	0.53571	0.43733	0.97288 (max o	10	2193; 1521; 1520; 79	0.36377117	-0.1604553		6.0
	ZINC15675802	-8.9	380.383	-1.2315	9	1	287.95	0.36437	128.04	4.1804	231.4743965740	0.32511	7.867	-3.7879	0.53571	0.43733	0.97288 (max o	9	1521; 1520; 226; 497	0.34704232	-0.1714624		6.0
	ZINC04654767	-8.9	355.321	0.0168	7	3	249.54	0.37148	134.96	1.8844	231.4743965740	0.35012	6.6187	0.047984	0.5	0.45173		0		-0.9567889	0.01091020		6.0
	ZINC31165193	-8.9	446.431	-0.2867	10	5	326.96	0.38922	176.45	1.5646	231.4743965740	0.28447	6.9222	-1.0078	0.5	0.51634	0.99059 (max o	3	1029; 1028; 821	-0.9301644	-0.0394513		6.0
	ZINC31165181	-8.9	446.431	-0.2867	10	5	326.96	0.38922	176.45	1.5646	231.4743965740	0.28447	6.9222	-1.0078	0.5	0.5148	0.99059 (max o	3	1029; 1028; 822	-0.9184322	-0.0672506		6.0
	ZINC35455726	-8.9	427.405	-2.4776	11	6	298.59	0.44261	182.09	-10.383	231.4743965740	0.30344	9.1131	-8.1651	0.53333	0.48187	0.96092 (max o	9	3895; 3037; 2632; 26	-0.5069212	0.7164587		6.0

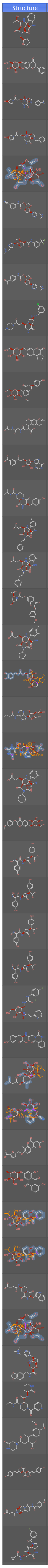

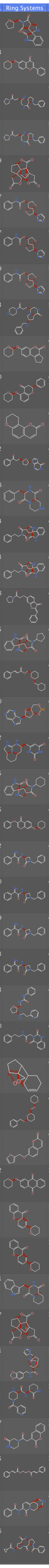

Structure	Molecule Name	affinity	Total Molweight	cLogP	H-Acceptors	H-Donors	Total Surface Area	Relative PSA	Polar Surface Area	Druglikeness	Kd (nM) @ 20°C	LE from K...	LLE from Kd ...	LELP from Kd (n...	Shape Index	Molecular...	Neighbor Si...	Neig...	Neighbor	Neighbor ...	Neighbor ...	Ring Systems	frequency
	ZINC20758733	-8.9	385.419	-0.8469	8	3	262.29	0.33814	107.97	3.0652	231.4743965740	0.32511	7.4824	-2.605	0.42857	0.22147	0.98866 (max o	8	3418; 1880; 1878; 12	0.12499587	0.8251628		5.0
	ZINC04349275	-8.9	400.382	1.0378	8	4	280.14	0.34069	125.68	-3.2535	231.4743965740	0.3139	5.5977	3.3061	0.55172	0.37921	0.9617 (max of	7	1228; 293; 451; 452;	-0.3368221	0.54244983		5.0
	ZINC35442587	-8.9	444.598	-1.5696	8	3	351.93	0.22414	80.56	6.6346	231.4743965740	0.28447	8.2051	-5.5176	0.59375	0.48437	0.92863 (max o	3	1053; 666; 838	0.03256984	0.5359051		5.0
	ZINC35442588	-8.9	401.529	-1.466	7	3	317.11	0.23755	77.32	6.0025	231.4743965740	0.3139	8.1015	-4.6703	0.58621	0.45742	0.94529 (max o	5	5625; 2988; 1053; 66	-0.0129881	0.5701149		5.0
	ZINC04245650	-8.9	408.402	-1.0717	9	2	242.06	0.4351	128.59	-1.779	231.4743965740	0.3139	7.7072	-3.4141	0.34483	0.095918	0.91333 (max o	4	5818; 2869; 1761; 72	-0.6671855	-0.3962609		5.0
	ZINC04260846	-8.9	399.430	-1.5441	10	2	313.8	0.36475	115.73	-15.549	231.4743965740	0.3139	8.1796	-4.9191	0.62069	0.35444	0.92547 (max o	6	2442; 1721; 1061; 90	-0.2547180	-0.0413077		5.0
	ZINC04260843	-8.9	442.417	-0.5314	9	2	321.55	0.31379	91.94	-18.459	231.4743965740	0.29365	7.1669	-1.8096	0.58065	0.39063	0.92547 (max o	4	2442; 1721; 733; 731	-0.2395046	-0.0247597		5.0
	ZINC04260839	-8.9	416.456	-1.5085	10	2	322.1	0.35374	109.01	-11.186	231.4743965740	0.30344	8.144	-4.9714	0.63333	0.37668	0.91934 (max o	4	2442; 1721; 733; 732	-0.2339777	-0.0458979		5.0
	ZINC22936952	-8.9	434.990	-0.1898	7	2	321.14	0.17416	72.5	9.2297	231.4743965740	0.30344	6.8253	-0.6255	0.6	0.47775	0.9707 (max of	7	1278; 1271; 1269; 10	-0.6029016	0.06629683		5.0
	ZINC04061430	-8.9	364.349	-0.2704	8	4	246.37	0.38738	125.68	-6.0856	231.4743965740	0.35012	6.9059	-0.77231	0.5	0.36444	0.9464 (max of	6	2406; 2034; 193; 343	0.3184636	0.4854254		4.0
	ZINC05742783	-8.9	448.423	0.4421	10	5	310.3	0.38202	155.14	-3.5326	231.4743965740	0.28447	6.1934	1.5541	0.5	0.32874	0.95983 (max o	6	1751; 142; 460; 461;	0.1207999	-0.3322760		4.0
	ZINC08764911	-8.9	445.447	-1.5942	10	2	328.88	0.36293	143.09	-1.6899	231.4743965740	0.28447	8.2297	-5.6041	0.5625	0.52503	0.90963 (max o	3	209; 357; 361	-0.0230727	0.135561		4.0
	ZINC15249204	-8.9	413.365	-2.6382	11	3	291.2	0.44784	166.17	1.3595	231.4743965740	0.30344	9.2737	-8.6944	0.53333	0.39424	0.94785 (max o	8	1054; 221; 242; 296;	0.2534947	-0.7612242		4.0
	ZINC04260212	-8.9	360.413	0.3468	8	3	266.44	0.30866	101.98	8.3838	231.4743965740	0.35012	6.2887	0.99052	0.61538	0.51928	0.96106 (max o	2	4550; 349	-0.3320581	-0.2574588		4.0
	ZINC15967718	-8.9	446.482	-2.8941	8	2	319.48	0.29082	118.64	4.6249	231.4743965740	0.27585	9.5296	-10.492	0.42424	0.30771	0.9615 (max of	9	30; 112; 163; 229; 38	0.1508989	-0.5889504		4.0
	ZINC15968477	-8.9	446.482	-2.8941	8	2	319.48	0.29082	118.64	4.6249	231.4743965740	0.27585	9.5296	-10.492	0.42424	0.30771	0.9615 (max of	9	1241; 63; 163; 229; 2	0.17251657	-0.5852283		4.0
	ZINC08790584	-8.9	438.411	-0.5599	9	1	316.7	0.31591	125.43	6.3014	231.4743965740	0.28447	7.1954	-1.9682	0.5625	0.41531	0.9642 (max of	7	4090; 1806; 1805; 15	0.53580815	0.46606398		4.0
	ZINC15957045	-8.9	424.475	-3.1847	8	2	298.5	0.31126	118.64	1.6256	231.4743965740	0.29365	9.8202	-10.845	0.3871	0.2152	0.95771 (max o	4	1256; 227; 380; 500	0.39647833	-0.5804105		4.0
	ZINC49601726	-8.9	440.443	0.0769	10	6	314.91	0.38627	166.14	-6.1546	231.4743965740	0.29365	6.5586	0.26188	0.58065	0.46737	0.96262 (max o	6	3089; 3036; 1617; 16	-0.2828627	0.5701478		4.0
	ZINC04556880	-8.9	400.307	-3.8839	12	3	262.82	0.51275	163.91	-12.61	231.4743965740	0.33715	10.519	-11.52	0.55556	0.2929	0.92739 (max o	3	2826; 1731; 584	0.07763652	-0.9825110		4.0
	ZINC13430721	-8.9	415.426	-4.3719	12	5	256.85	0.50602	182.48	-2.4143	231.4743965740	0.32511	11.007	-13.447	0.46429	0.44266	0.95983 (max o	3	2171; 1505; 620	-0.5373129	-0.6899892		4.0
	ZINC15958065	-8.9	424.475	-3.1866	8	2	300	0.3097	118.64	-1.065	231.4743965740	0.29365	9.8221	-10.852	0.3871	0.28378	0.96018 (max o	5	1843; 110; 228; 382;	0.3445391	-0.2352535		4.0
	ZINC08844375	-8.9	446.407	0.4497	10	4	312.4	0.36953	144.14	-3.4946	231.4743965740	0.28447	6.1858	1.5808	0.59375	0.41419	0.95465 (max o	4	1581; 1319; 1199; 58	0.31050527	0.59851235		4.0
	ZINC14614223	-8.9	398.350	-2.2584	10	4	282.84	0.42151	162.59	6.748	231.4743965740	0.3139	8.8939	-7.1946	0.58621	0.44643	0.97415 (max o	5	3085; 3084; 785; 786	-0.5028130	-0.6409922		4.0
	ZINC14614223	-8.9	398.350	-2.2584	10	4	282.84	0.42151	162.59	6.748	231.4743965740	0.3139	8.8939	-7.1946	0.58621	0.49353	0.97415 (max o	5	3085; 3084; 788; 785	-0.5058666	-0.6193360		4.0
	ZINC14614223	-8.9	397.342	-2.4419	10	3	284.02	0.42391	165.42	6.9732	231.4743965740	0.3139	9.0774	-7.7792	0.58621	0.44643	0.96931 (max o	5	3085; 3084; 788; 785	-0.4834866	-0.6091149		4.0
	ZINC14614223	-8.9	397.342	-2.4419	10	3	284.02	0.42391	165.42	6.9732	231.4743965740	0.3139	9.0774	-7.7792	0.58621	0.49353	0.96931 (max o	5	3085; 3084; 788; 786	-0.4811255	-0.6469973		4.0
	ZINC20465893	-8.9	437.465	-0.9573	8	5	335.99	0.29977	108.15	0.42057	231.4743965740	0.29365	7.5928	-3.26	0.58065	0.54509	0.93414 (max o	5	3855; 3407; 3008; 26	0.2965913	-0.2772163		4.0
	ZINC20611386	-8.9	382.351	-1.6419	9	3	277.64	0.38183	138.87	6.0279	231.4743965740	0.32511	8.2774	-5.0503	0.57143	0.54109	0.94543 (max o	4	2979; 2128; 1364; 10	0.14108321	-0.2615350		4.0
	ZINC27639108	-8.9	382.451	0.2956	7	3	268.97	0.33457	108.75	-4.0719	231.4743965740	0.33715	6.3399	0.87676	0.44444	0.35136	0.85929	1	7548	-0.1161277	0.8482937		4.0
	ZINC35455722	-8.9	427.405	-2.4776	11	6	298.59	0.44261	182.09	-10.383	231.4743965740	0.30344	9.1131	-8.1651	0.53333	0.47708	0.96092 (max o	6	3037; 1583; 1331; 12	-0.4860235	0.7258265		4.0
	ZINC36369793	-8.9	370.312	-2.5383	8	0	272.48	0.37357	128.93	1.8784	231.4743965740	0.33715	9.1738	-7.5286	0.59259	0.39652	0.96023 (max o	3	3516; 2667; 1321	-0.4263606	-0.879685		4.0
	ZINC04098673	-8.9	416.381	1.2776	9	5	280.29	0.39809	153.75	1.4258	231.4743965740	0.30344	5.3579	4.2104	0.46667	0.32703	0.96589 (max o	5	3; 14; 19; 20; 24	0.6296578	0.09006462		3.0
	ZINC28966917	-8.9	392.359	-1.3716	9	6	257.5	0.43355	156.91	-2.2179	231.4743965740	0.32511	8.0071	-4.2189	0.46429	0.35879	0.9543 (max of	5	5589; 2619; 1926; 41	-0.2467729	0.6093171		3.0
	ZINC28966920	-8.9	392.359	-1.3716	9	6	257.5	0.43355	156.91	-2.2179	231.4743965740	0.32511	8.0071	-4.2189	0.46429	0.35818	0.9543 (max of	4	5589; 2619; 415; 816	-0.2260502	0.6155841		3.0
	ZINC05396126	-8.9	411.437	-2.05	9	3	302.24	0.35369	134.43	5.4919	231.4743965740	0.30344	8.6855	-6.7559	0.6	0.45473	0.95549 (max o	2	2755; 557	-0.415761	-0.3946041		3.0
	ZINC04245649	-8.9	408.402	-1.0717	9	2	242.06	0.4351	128.59	-1.779	231.4743965740	0.3139	7.7072	-3.4141	0.34483	0.095918	0.86493	1	726	-0.6864527	-0.3832987		3.0
	ZINC08298794	-8.9	428.535	-3.2222	9	3	349.23	0.31065	87.15	0.20949	231.4743965740	0.29365	9.8577	-10.973	0.6129	0.48758	0.97423 (max o	6	3720; 3277; 2858; 21	-0.1616147	-0.0300441		3.0
	ZINC08299452	-8.9	416.480	-0.3222	10	3	315.58	0.33148	123.74	6.2676	231.4743965740	0.30344	6.9577	-1.0618	0.46667	0.53707	0.91374 (max o	2	1739; 1440	0.10025882	0.2242658		3.0
	ZINC12899074	-8.9	360.365	0.5334	8	1	266.01	0.31627	94.17	5.7863	231.4743965740	0.35012	6.1021	1.5235	0.53846	0.48097	0.90311 (max o	2	2124; 1473	0.8678924	-0.3540937		3.0
	ZINC27558828	-8.9	440.359	-3.0384	10	2	329.78	0.38486	173.32	-1.5257	231.4743965740	0.28447	9.6739	-10.681	0.625	0.43072	0.9665 (max of	2	1889; 1820	0.4962747	0.72171545		3.0
	ZINC04237055	-8.9	438.459	-0.8652	9	1	322.59	0.29449	119.08	5.1325	231.4743965740	0.28447	7.5007	-3.0414	0.625	0.445	0.9112 (max of	2	2436; 2435	-0.5157628	-0.404545		2.0
	ZINC03839295	-8.9	414.436	0.1505	8	2	285.19	0.28816	98.82	5.9522	231.4743965740	0.30344	6.485	0.49598	0.5	0.35004	0.94823 (max o	3	1377; 878; 250	-0.4530022	-0.2461256		2.0

Structure	Molecule Name	affinity	Total Molweight	cLogP	H-Acceptors	H-Donors	Total Surface Area	Relative PSA	Polar Surface Area	Druglikeness	Kd (nM) @ 20°C	LE from K...	LLE from Kd ...	LELP from Kd (n...	Shape Index	Molecular...	Neighbor Si...	Neig...	Neighbor	Neighbor ...	Neighbor ...	Ring Systems	frequency
	ZINC04534328	-8.9	387.395	-0.5073	10	5	276.48	0.41102	145.78	-0.80219	231.4743965740	0.32511	7.1428	-1.5604	0.53571	0.39692	0.94862 (max o	5	1717; 1430; 1161; 97	-0.4194408	-0.6932871		2.0
	ZINC18456291	-8.9	439.387	-5.798	13	4	322.75	0.50758	214.65	3.0765	231.4743965740	0.28447	12.433	-20.382	0.625	0.60217	0.98148	1	393	-0.0181661	-0.7907554		2.0
	ZINC35454976	-8.9	438.427	0.2591	10	5	296.68	0.39956	155.14	-11.09	231.4743965740	0.29365	6.3764	0.88235	0.48387	0.46355	0.96018 (max o	7	1895; 1307; 1284; 18	-0.1743804	0.8578342		2.0
	ZINC05515783	-8.9	386.355	-0.4899	8	2	250.22	0.38094	119.36	-1.0904	231.4743965740	0.32511	7.1254	-1.5069	0.35714	0.28247	0.96253 (max o	3	934; 933; 458	0.8644879	-0.0966344		2.0
	ZINC02127440	-8.9	380.379	-0.8901	8	2	285.05	0.32594	118.64	4.6362	231.4743965740	0.32511	7.5256	-2.7378	0.57143	0.49409	0.97346 (max o	4	2241; 1365; 1088; 46	0.18851195	-0.2256016		2.0
	ZINC31417974	-8.9	429.400	-1.5624	9	3	306.61	0.36757	153.42	1.8004	231.4743965740	0.29365	8.1979	-5.3206	0.51613	0.50116	0.98065	1	510	0.88577855	0.00565274		2.0
	ZINC31166407	-8.9	436.412	0.055	10	7	305.5	0.40831	177.14	-4.8731	231.4743965740	0.29365	6.5805	0.1873	0.58065	0.49368	0.94751	1	3179	0.7932966	-0.2391928		2.0
	ZINC49181872	-8.9	446.411	0.0678	11	3	310.24	0.38822	147.35	6.7757	231.4743965740	0.28447	6.5677	0.23834	0.5	0.50056	0.95465 (max o	3	4428; 3952; 679	-0.1637267	-0.9800638		2.0
	ZINC49600058	-8.9	416.421	-1.5798	10	6	291.9	0.4063	158.3	-5.0311	231.4743965740	0.3139	8.2153	-5.0328	0.58621	0.51506	0.96983 (max o	9	4829; 1331; 1227; 12	-0.5129723	0.67700785		2.0
	ZINC02094988	-8.9	400.434	0.0218	8	1	279.19	0.39751	140.87	10.153	231.4743965740	0.32511	6.6137	0.067054	0.5	0.36695	0.93686 (max o	5	3593; 1660; 1659; 13	0.43711573	0.65512365		2.0
	ZINC01530747	-8.9	400.434	0.0218	8	1	279.19	0.39751	140.87	10.153	231.4743965740	0.32511	6.6137	0.067054	0.5	0.36695	0.93686 (max o	5	3593; 1660; 1659; 13	0.43748987	0.6896844		2.0
	ZINC02124021	-8.9	426.408	-3.6466	11	3	312.98	0.39456	154.74	4.9479	231.4743965740	0.29365	10.282	-12.418	0.54839	0.50844	0.97856 (max o	2	2728; 2573	0.61027867	-0.5913242		2.0
	ZINC03839261	-8.9	419.480	-1.366	10	2	299.17	0.33015	119.13	6.2853	231.4743965740	0.30344	8.0015	-4.5018	0.53333	0.37915	0.95267 (max o	4	2002; 1706; 898; 78	-0.5247847	0.01581453		2.0
	ZINC03840090	-8.9	424.456	-0.6754	9	5	317.9	0.36011	154.64	4.972	231.4743965740	0.29365	7.3109	-2.3	0.54839	0.48807	0.96971 (max o	7	2758; 2428; 2006; 13	-0.4685976	-0.1600343		2.0
	ZINC03840092	-8.9	425.444	-1.6223	10	5	316.66	0.39617	167.53	4.972	231.4743965740	0.29365	8.2578	-5.5246	0.54839	0.44796	0.96971 (max o	6	3167; 2428; 2006; 13	-0.4880818	-0.1518936		2.0
	ZINC04222688	-8.9	410.405	-1.0452	9	1	301.36	0.32888	122.99	0.78313	231.4743965740	0.30344	7.6807	-3.4445	0.56667	0.4199	0.96832 (max o	6	3656; 2802; 2432; 11	-0.1882779	0.3791044		2.0
	ZINC04228265	-8.9	441.403	-6.3118	13	5	323.08	0.51025	213.26	4.3834	231.4743965740	0.28447	12.947	-22.188	0.625	0.58663	0.98148 (max o	3	922; 900; 720	0.8669605	0.2431066		2.0
	ZINC04277922	-8.9	399.450	-0.3689	9	3	300.07	0.33319	128.6	8.4978	231.4743965740	0.3139	7.0044	-1.1752	0.48276	0.51039	0.88151	1	959	-0.2516964	0.23325898		2.0
	ZINC08636403	-8.9	389.431	0.2797	8	4	274.18	0.40543	137.71	4.8689	231.4743965740	0.33715	6.3558	0.8296	0.55556	0.36319	0.88896 (max o	7	3736; 2529; 2099; 17	-0.5006938	0.32970303		2.0
	ZINC12603440	-8.9	422.484	-1.1523	11	4	315.08	0.36257	140.15	6.9641	231.4743965740	0.30344	7.7878	-3.7975	0.63333	0.55714	0.95579 (max o	3	1466; 208; 466	-0.0716248	-0.6304391		2.0
	ZINC12604035	-8.9	438.522	-0.2176	8	4	312.06	0.33554	131.72	5.078	231.4743965740	0.30344	6.8531	-0.71712	0.6	0.44025	0.97975 (max o	3	5162; 1005; 772	0.4890338	0.17249659		2.0
	ZINC12900934	-8.9	403.413	-1.9011	7	2	304.96	0.29669	125.8	2.1693	231.4743965740	0.30344	8.5366	-6.2652	0.56667	0.4717	0.95053	1	776	0.6811513	0.2639508		2.0
	ZINC12900927	-8.9	403.413	-1.9011	7	2	304.96	0.29669	125.8	2.1693	231.4743965740	0.30344	8.5366	-6.2652	0.56667	0.47166	0.95053	1	777	0.6992872	0.275613		2.0
	ZINC20113588	-8.9	403.414	-2.3044	10	3	302.6	0.37403	110.64	4.1076	231.4743965740	0.3139	8.9399	-7.3412	0.48276	0.41168	0.98525 (max o	3	638; 639; 802	-0.1916776	0.60598373		2.0
	ZINC20113588	-8.9	403.414	-2.3044	10	3	302.6	0.37403	110.64	4.1076	231.4743965740	0.3139	8.9399	-7.3412	0.48276	0.41168	0.98525 (max o	3	803; 638; 639	-0.2117559	0.60030335		2.0
	ZINC31156357	-8.9	424.401	-0.5282	10	4	300.94	0.3937	151.98	-0.28537	231.4743965740	0.30344	7.1637	-1.7407	0.6	0.42846	0.95318 (max o	3	2255; 1575; 1286	0.25144333	-0.9562282		2.0
	ZINC31157773	-8.9	430.448	-1.2551	10	6	302.9	0.39155	158.3	1.0111	231.4743965740	0.30344	7.8906	-4.1363	0.56667	0.51842	0.97122 (max o	3	2635; 2045; 1288	-0.4529441	0.36024538		2.0
	ZINC35454969	-8.9	438.427	0.2591	10	5	296.68	0.39956	155.14	-11.09	231.4743965740	0.29365	6.3764	0.88235	0.48387	0.46257	0.96018 (max o	7	1895; 1307; 1284; 84	-0.1824049	0.8395289		2.0
	ZINC35464954	-8.9	391.482	-0.7932	6	3	283.97	0.29676	113.35	-0.82856	231.4743965740	0.32511	7.4287	-2.4398	0.46429	0.37458	0.9678 (max of	4	3934; 3074; 2292; 41	-0.0715601	0.4194283		2.0
	ZINC36378600	-8.9	360.361	-0.1543	7	4	232.58	0.41332	127.59	0.18236	231.4743965740	0.35012	6.7898	-0.44071	0.42308	0.29718	0.9615 (max of	3	2668; 1925; 1612	0.05158391	0.94514954		2.0
	ZINC67903395	-8.9	443.467	-2.8577	10	5	309.29	0.3971	168.97	-2.258	231.4743965740	0.29365	9.4932	-9.7317	0.51613	0.43315	0.94751	1	2314	-0.2679041	-0.7752617		2.0
	ZINC67913908	-8.9	406.386	0.3752	9	6	276.64	0.39257	149.07	-1.9967	231.4743965740	0.3139	6.2603	1.1953	0.55172	0.34056	0.95099 (max o	3	322; 533; 854	-0.8215611	0.3605791		2.0
	ZINC67913906	-8.9	406.386	0.3752	9	6	276.64	0.39257	149.07	-1.9967	231.4743965740	0.3139	6.2603	1.1953	0.55172	0.34056	0.95099 (max o	3	855; 322; 533	-0.8461343	0.36448058		2.0
	ZINC72319973	-8.9	356.285	-2.8261	8	2	254.28	0.39657	139.18	1.0637	231.4743965740	0.35012	9.4616	-8.0718	0.57692	0.39966	0.93686 (max o	3	2324; 1625; 858	0.70342696	0.5157391		2.0
	ZINC72319976	-8.9	356.285	-2.8261	8	2	254.28	0.39657	139.18	1.0637	231.4743965740	0.35012	9.4616	-8.0718	0.57692	0.3936	0.93686 (max o	3	2324; 1625; 857	0.68455577	0.5096355		2.0
	ZINC02083422	-8.9	380.423	-0.7219	7	1	283.11	0.25418	92.78	5.2668	231.4743965740	0.32511	7.3574	-2.2205	0.53571	0.54774	0.87932 (max o	3	4943; 1972; 1655	0.16728146	0.7876274		1.0
	ZINC02111050	-8.9	386.455	-2.0293	7	1	258.82	0.42211	134.11	6.1578	231.4743965740	0.35012	8.6648	-5.796	0.46154	0.28872		0		-0.2315864	0.9545852		1.0
	ZINC02121314	-8.9	426.428	-0.0088001	10	0	292.43	0.31611	115.38	2.3242	231.4743965740	0.29365	6.6443	-0.029968	0.35484	0.31637	0.96262	1	486	0.7404345	0.40650824		1.0
	ZINC02131155	-8.9	419.416	-0.8024	9	3	309.22	0.34571	134.43	6.1452	231.4743965740	0.29365	7.4379	-2.7325	0.54839	0.50308	0.96054 (max o	2	1008; 396	0.0931068	-0.2681376		1.0
	ZINC03838439	-8.9	436.494	-4.2957	10	4	325.01	0.36863	161.71	6.1828	231.4743965740	0.28447	10.931	-15.101	0.5	0.46401		0		0.7541643	-0.6513901		1.0
	ZINC03839266	-8.9	426.471	-0.0203	9	2	301.1	0.30614	108.05	7.2589	231.4743965740	0.29365	6.6558	-0.06913	0.54839	0.33365	0.93965 (max o	5	2374; 1376; 132; 250	-0.4812981	-0.1956011		1.0
	ZINC03839352	-8.9	413.476	-0.699	9	2	294.06	0.30283	103.75	7.4055	231.4743965740	0.30344	7.3345	-2.3036	0.5	0.28889	0.9396 (max of	9	3818; 2749; 1670; 16	-0.6213925	-0.0415577		1.0
	ZINC04024908	-8.9	414.504	1.4734	8	2	264.96	0.31099	105.8	0.69584	231.4743965740	0.30344	5.1621	4.8557	0.46667	0.022941		0		0.80967116	0.30638367		1.0

Structure	Molecule Name	affinity	Total Molweight	cLogP	H-Acceptors	H-Donors	Total Surface Area	Relative PSA	Polar Surface Area	Druglikeness	Kd (nM) @ 20°C	LE from K...	LLE from Kd ...	LELP from Kd (n...	Shape Index	Molecular...	Neighbor Si...	Neig...	Neighbor	Neighbor ...	Neighbor ...	Ring Systems	frequency
	ZINC04064912	-8.9	358.369	-0.3699	7	1	262.43	0.30503	106.97	5.6302	231.4743965740	0.35012	7.0054	-1.0565	0.53846	0.49435	0.89253 (max o	2	2033; 333	0.28777382	0.12186974		1.0
	ZINC04147896	-8.9	436.451	-0.8049	11	1	317.69	0.34458	118.86	1.326	231.4743965740	0.28447	7.4404	-2.8295	0.59375	0.36841	0.9145 (max of	5	1147; 1145; 1108; 10.	-0.0356270	-0.2983836		1.0
	ZINC04217378	-8.9	432.380	-0.3621	10	6	292.84	0.41538	166.14	-3.6206	231.4743965740	0.29365	6.9976	-1.2331	0.51613	0.40085	0.91993	1	896	0.22745284	0.52532005		1.0
	ZINC04221763	-8.9	411.421	-1.3623	11	3	295.08	0.42877	166.3	3.2463	231.4743965740	0.30344	7.9978	-4.4896	0.53333	0.39436	0.88683 (max o	2	899; 81	-0.723478	0.49509013		1.0
	ZINC04221778	-8.9	425.461	-2.3788	10	5	300.22	0.43998	184.27	-10.675	231.4743965740	0.3139	9.0143	-7.5782	0.55172	0.54528		0		0.9220366	-0.3564691		1.0
	ZINC04228237	-8.9	443.419	-8.3663	13	6	324.07	0.50853	212.93	4.3321	231.4743965740	0.28447	15.002	-29.41	0.625	0.57094	0.97533 (max o	7	2956; 2955; 2555; 92.	0.8592949	0.2642678		1.0
	ZINC04247587	-8.9	392.359	-1.4089	9	4	258.93	0.4307	150.59	-1.9479	231.4743965740	0.32511	8.0444	-4.3336	0.46429	0.40469	0.92464 (max o	7	1716; 1715; 140; 141.	-0.3284029	0.67128706		1.0
	ZINC04247587	-8.9	392.359	-1.4757	9	5	258.48	0.43168	153.75	-1.9031	231.4743965740	0.32511	8.1112	-4.5391	0.46429	0.39644	0.92774 (max o	7	1716; 1715; 140; 141.	-0.2748746	0.65459937		1.0
	ZINC04259759	-8.9	401.378	0.6766	11	1	275.01	0.41555	118.85	3.3115	231.4743965740	0.3139	5.9589	2.1555	0.55172	0.31567	0.90474 (max o	5	1417; 914; 441; 575;	-0.2178562	-0.4124921		1.0
	ZINC04270599	-8.9	372.404	-0.3206	9	1	257.3	0.34015	97.13	0.76976	231.4743965740	0.33715	6.9561	-0.95091	0.55556	0.29505	0.91482 (max o	4	1151; 907; 266; 267	0.02276563	-0.3037762		1.0
	ZINC04270905	-8.9	372.424	0.1624	8	1	273.45	0.26049	82.19	6.366	231.4743965740	0.33715	6.4731	0.48168	0.59259	0.52452	0.86774 (max o	2	1153; 198	-0.292054	0.10367205		1.0
	ZINC05396121	-8.9	354.389	-1.4365	8	1	259.97	0.314	91.81	-3.1315	231.4743965740	0.35012	8.072	-4.1029	0.61538	0.32405	0.88044 (max o	3	1736; 1718; 751	-0.0431031	-0.2628653		1.0
	ZINC05409706	-8.9	383.427	-0.8747	8	1	284.97	0.28305	88.15	-3.1315	231.4743965740	0.32511	7.5102	-2.6905	0.64286	0.38281	0.89765 (max o	5	3700; 2834; 1434; 11	-0.0650199	-0.2705896		1.0
	ZINC05414102	-8.9	442.518	-0.035	10	2	333.66	0.31205	124.6	0.97979	231.4743965740	0.28447	6.6705	-0.12303	0.4375	0.4959	0.971 (max of 5	5	4574; 3691; 1171; 96	-0.0606148	0.21228573		1.0
	ZINC05415679	-8.9	442.538	-0.0404	9	3	330.12	0.27239	115.43	4.8256	231.4743965740	0.28447	6.6759	-0.14202	0.4375	0.49406	0.96877 (max o	4	1438; 1177; 465; 599	0.00257148	0.32136577		1.0
	ZINC05433770	-8.9	445.542	-1.1311	10	3	331.58	0.29191	120.36	6.2411	231.4743965740	0.28447	7.7666	-3.9761	0.40625	0.46714	0.90474 (max o	5	4598; 2863; 1179; 93	-0.0666418	0.27134714		1.0
	ZINC05433793	-8.9	437.498	0.5372	9	2	329.56	0.27021	103.75	6.4317	231.4743965740	0.28447	6.0983	1.8884	0.40625	0.47071	0.96186 (max o	4	1179; 456; 482; 592	-0.0250623	0.25972533		1.0
	ZINC12604035	-8.9	439.530	-2.3273	8	5	326.1	0.36415	132.92	1.6011	231.4743965740	0.30344	8.9628	-7.6698	0.6	0.44358	0.91871 (max o	3	5944; 5162; 771	0.48222166	0.19266617		1.0
	ZINC12899533	-8.9	423.400	-1.9745	9	2	317.96	0.34394	133.86	-2.3457	231.4743965740	0.29365	8.61	-6.724	0.58065	0.5119	0.90581 (max o	2	2947; 287	0.16474698	-0.1298265		1.0
	ZINC12901038	-8.9	392.390	-1.3869	8	1	289.07	0.31107	111.13	5.225	231.4743965740	0.3139	8.0224	-4.4183	0.55172	0.40814	0.93111 (max o	4	5494; 3348; 2546; 61	0.14860071	0.49844712		1.0
	ZINC13322014	-8.9	418.397	0.5121	9	5	288.04	0.37682	145.91	-3.7146	231.4743965740	0.30344	6.1234	1.6877	0.53333	0.43261	0.97304 (max o	8	1223; 978; 945; 368;	0.07000834	-0.4155057		1.0
	ZINC13540864	-8.9	442.360	-5.2821	15	4	299.72	0.58421	203.88	3.3719	231.4743965740	0.29365	11.918	-17.988	0.48387	0.51774		0		-0.2218722	0.9639723		1.0
	ZINC15265541	-8.9	437.432	-1.1212	12	4	291.98	0.49284	186.74	-20.852	231.4743965740	0.30344	7.7567	-3.695	0.53333	0.45215		0		0.8906278	-0.2069796		1.0
	ZINC15958253	-8.9	373.412	-2.1561	8	1	262.52	0.38458	130.44	4.2093	231.4743965740	0.35012	8.7916	-6.1582	0.53846	0.51658	0.94951	1	1636	0.5724097	0.69134015		1.0
	ZINC20113588	-8.9	402.406	-0.1947	10	2	288.56	0.34357	109.44	5.9634	231.4743965740	0.3139	6.8302	-0.62026	0.48276	0.41168	0.97649 (max o	4	2343; 999; 637; 639	-0.199122	0.64118		1.0
	ZINC20463393	-8.9	428.575	-0.9169	7	1	324.62	0.15714	81.06	6.2447	231.4743965740	0.29365	7.5524	-3.1224	0.54839	0.51877	0.9396 (max of	10	4723; 4720; 3387; 33	-0.2037853	-0.2154115		1.0
	ZINC20463985	-8.9	447.577	-0.413	6	3	352.77	0.19673	72.63	-0.27172	231.4743965740	0.27585	7.0485	-1.4972	0.60606	0.4828	0.91964 (max o	4	7849; 7847; 7629; 73	0.4184709	0.4820098		1.0
	ZINC20612431	-8.9	420.444	-0.0337	8	1	310.41	0.27383	109.85	6.2828	231.4743965740	0.29365	6.6692	-0.11476	0.45161	0.50654	0.89932	1	1255	0.761257	-0.0361118		1.0
	ZINC22936952	-8.9	437.006	-4.4092	7	4	349.22	0.24056	74.9	4.2246	231.4743965740	0.30344	11.045	-14.531	0.6	0.49816		0		0.82287186	0.561432		1.0
	ZINC31165201	-8.9	438.384	-0.3814	11	7	301.74	0.44654	186.37	-4.1057	231.4743965740	0.29365	7.0169	-1.2988	0.58065	0.41577		0		0.8226873	0.22672841		1.0
	ZINC31165470	-8.9	434.396	-0.6184	10	5	309.68	0.3926	162.98	-4.415	231.4743965740	0.29365	7.2539	-2.1059	0.6129	0.46654		0		-0.9117669	0.27727464		1.0
	ZINC31166343	-8.9	402.394	-2.1285	10	6	277.31	0.42768	158.3	0.28317	231.4743965740	0.32511	8.764	-6.547	0.57143	0.47713	0.93686 (max o	5	3528; 1580; 852; 309	-0.3839401	0.46001512		1.0
	ZINC31502299	-8.9	446.407	-0.0864	10	5	308.75	0.38394	155.14	-3.4398	231.4743965740	0.28447	6.7219	-0.30372	0.46875	0.32112	0.97319 (max o	6	2663; 1994; 506; 569	0.1451028	0.63580245		1.0
	ZINC35271099	-8.9	443.467	-3.2526	10	5	300.11	0.40925	168.97	-2.3795	231.4743965740	0.29365	9.8881	-11.077	0.48387	0.42633	0.92863	1	1588	-0.8096235	-0.5172645		1.0
	ZINC35442566	-8.9	416.544	-2.0709	8	3	329.63	0.26181	90.21	5.3245	231.4743965740	0.30344	8.7064	-6.8248	0.6	0.49319	0.9707 (max of	6	4827; 3866; 3865; 30	-0.0103557	0.5506816		1.0
	ZINC35442567	-8.9	409.549	-2.1504	8	3	321.39	0.2655	86.55	1.7389	231.4743965740	0.3139	8.7859	-6.8506	0.58621	0.50944	0.96931 (max o	7	4825; 4401; 3922; 34	-0.1586336	0.5953599		1.0
	ZINC40310000	-8.9	394.406	-1.749	8	2	305.56	0.30498	112.49	0.98724	231.4743965740	0.3139	8.3845	-5.5718	0.68966	0.54829	0.96428	1	1328	-0.1501054	-0.8600218		1.0
	ZINC49034160	-8.9	364.393	-0.3946	7	3	236.37	0.37407	121.13	0.68838	231.4743965740	0.35012	7.0301	-1.127	0.34615	0.32434	0.92032	1	1936	-0.5283789	0.7078721		1.0
	ZINC67902890	-8.9	416.421	-1.6984	10	6	291.07	0.40746	158.3	0.24642	231.4743965740	0.3139	8.3339	-5.4106	0.58621	0.48705	0.88355	1	825	-0.3894385	0.47940364		1.0
	ZINC72319896	-8.9	375.420	-2.7093	7	2	279.68	0.33642	108.17	0.94539	231.4743965740	0.33715	9.3448	-8.0358	0.44444	0.35596	0.91768 (max o	3	3113; 3112; 1949	-0.0943748	-0.7820487		1.0
	ZINC30727131	-8.9	365.424	1.5198	7	1	279.07	0.27151	93.14	7.503	231.4743965740	0.35012	5.1157	4.3408	0.42308	0.38315	0.87576 (max o	2	6286; 3858	-0.7536130	0.43830904		0.0
	ZINC02120997	-8.8	403.410	-1.8971	9	2	307.48	0.35567	133.86	-2.132	274.8265485281	0.31037	8.458	-6.1123	0.55172	0.50794	0.9561 (max of	5	2723; 1989; 1361; 32	0.11625837	-0.0229222		172.0
	ZINC02097998	-8.8	389.383	-2.1296	9	2	293.19	0.373	133.86	-0.83321	274.8265485281	0.32146	8.6905	-6.6248	0.57143	0.46856	0.9678 (max of	6	3561; 3146; 1978; 16	0.21067138	0.04455916		172.0

Structure	Molecule Name	affinity	Total Molweight	cLogP	H-Acceptors	H-Donors	Total Surface Area	Relative PSA	Polar Surface Area	Druglikeness	Kd (nM) @ 20°C	LE from K...	LLE from Kd ...	LELP from Kd (n...	Shape Index	Molecular...	Neighbor Si...	Neig...	Neighbor	Neighbor ...	Neighbor ...	Ring Systems	frequency
	ZINC02104411	-8.8	389.383	-2.0049	9	2	297.98	0.367	133.86	-2.3536	274.8265485281	0.32146	8.5658	-6.2369	0.64286	0.57145	0.9678 (max of	9	5301; 4903; 4473; 35	0.26328823	-0.0652414		172.0
	ZINC35000842	-8.8	441.185	-10.923	16	5	270.33	0.74361	273.56	-47.565	274.8265485281	0.32146	17.484	-33.981	0.5	0.51704	0.93985 (max o	10	2487; 1584; 1506; 14	-0.2621466	-0.4689442		44.0
	ZINC35000842	-8.8	440.177	-12.171	16	4	271.51	0.74472	276.39	-42.835	274.8265485281	0.32146	18.732	-37.862	0.5	0.51686	0.93985 (max o	11	4933; 2488; 1585; 15	-0.2939514	-0.4196858		44.0
	ZINC20758272	-8.8	377.376	-3.2821	10	4	278.86	0.42125	150.9	7.3778	274.8265485281	0.33336	9.843	-9.8454	0.48148	0.54969	0.95174 (max o	12	5166; 4528; 3823; 38	0.03807269	-0.6155627		42.0
	ZINC13424749	-8.8	432.380	-0.3621	10	6	292.84	0.41538	166.14	-3.6206	274.8265485281	0.29035	6.923	-1.2471	0.54839	0.38725	0.96018 (max o	9	1230; 273; 274; 453;	0.17810766	0.6100077		30.0
	ZINC04098610	-8.8	416.381	-0.0164	9	5	286.49	0.37886	145.91	-3.6206	274.8265485281	0.30003	6.5773	-0.054661	0.56667	0.39146	0.96092 (max o	11	1230; 1159; 1128; 97	0.19202515	0.5541191		30.0
	ZINC13436564	-8.8	424.178	-11.319	15	3	266.42	0.70978	256.16	-39.309	274.8265485281	0.33336	17.88	-33.954	0.51852	0.51206	0.94905 (max o	9	2488; 1585; 1507; 12	-0.2958344	-0.4417244		26.0
	ZINC13436564	-8.8	425.186	-10.071	15	4	265.24	0.70849	253.33	-44.039	274.8265485281	0.33336	16.632	-30.211	0.51852	0.51225	0.94905 (max o	10	2487; 1584; 1506; 14	-0.2666677	-0.4896269		26.0
	ZINC02102194	-8.8	401.394	-2.0808	9	2	296.45	0.3689	133.86	-1.0092	274.8265485281	0.31037	8.6417	-6.7042	0.58621	0.4288	0.97176 (max o	7	1986; 1984; 1978; 19	0.25184324	0.04784248		24.0
	ZINC02115739	-8.8	415.421	-1.752	9	2	308.95	0.35397	133.86	1.6567	274.8265485281	0.30003	8.3129	-5.8395	0.56667	0.52881	0.95804 (max o	9	3571; 3569; 1983; 19	0.1575157	-0.0146206		24.0
	ZINC12885861	-8.8	449.866	-0.7375	9	2	322.32	0.32437	136.07	5.8142	274.8265485281	0.29035	7.2984	-2.54	0.6129	0.55891	0.88025	1	331	0.32271785	0.10484746		22.0
	ZINC02154742	-8.8	372.396	-0.262	7	0	278.34	0.27646	95.97	4.9812	274.8265485281	0.33336	6.8229	-0.78593	0.59259	0.53029	0.94622 (max o	6	2033; 1988; 186; 333	0.30411762	0.16053839		22.0
	ZINC02156179	-8.8	358.369	-0.6059	7	0	266.08	0.2892	95.97	4.9812	274.8265485281	0.34619	7.1668	-1.7502	0.61538	0.54928	0.95818 (max o	10	2850; 2729; 2484; 16	0.31611335	0.14301847		22.0
	ZINC02103774	-8.8	358.369	-0.5505	7	0	266.08	0.2892	95.97	2.3158	274.8265485281	0.34619	7.1114	-1.5902	0.53846	0.54806	0.95257 (max o	5	2850; 2484; 1642; 87	0.32884145	0.1259555		22.0
	ZINC04349611	-8.8	448.379	0.5798	11	7	295.9	0.45536	186.37	1.9289	274.8265485281	0.28128	5.9811	2.0613	0.40625	0.42191	0.9665 (max of	14	4095; 2776; 2566; 25	-0.6805849	0.27578554		21.0
	ZINC20465426	-8.8	417.426	0.2903	7	3	293.05	0.23136	85.27	-1.1221	274.8265485281	0.31037	6.2706	0.93533	0.51724	0.54689	0.95318 (max o	6	4749; 4310; 3003; 25	-0.3856228	0.10778244		21.0
	ZINC08636401	-8.8	373.476	0.8561	6	4	278.44	0.3274	119.25	4.8502	274.8265485281	0.34619	5.7048	2.473	0.53846	0.39094	0.93367 (max o	7	3735; 2881; 2529; 20	-0.507505	0.35007998		17.0
	ZINC13548856	-8.8	416.381	-0.0164	9	5	286.49	0.37886	145.91	-3.6206	274.8265485281	0.30003	6.5773	-0.054661	0.56667	0.39399	0.96092 (max o	9	1230; 1159; 366; 581	0.19474697	0.59606737		16.0
	ZINC03947503	-8.8	446.407	-0.0864	10	5	308.75	0.38394	155.14	-3.4398	274.8265485281	0.28128	6.6473	-0.30717	0.5625	0.40239	0.98343 (max o	13	2890; 1774; 1773; 19	0.2525045	0.6666459		16.0
	ZINC12495612	-8.8	446.407	-0.0864	10	5	308.75	0.38394	155.14	-3.4398	274.8265485281	0.28128	6.6473	-0.30717	0.53125	0.31148	0.95709 (max o	7	7349; 1425; 1206; 11	0.17550318	0.588914		16.0
	ZINC06137871	-8.8	372.424	0.1841	8	2	280.6	0.29287	98.82	5.2839	274.8265485281	0.33336	6.3768	0.55225	0.51852	0.5184	0.9578 (max of	8	4132; 3722; 3721; 29	0.07374401	0.29955226		14.0
	ZINC02104538	-8.8	415.421	-1.7388	9	2	310.21	0.35254	133.86	-3.3392	274.8265485281	0.30003	8.2997	-5.7955	0.6	0.45238	0.97304 (max o	6	2352; 1978; 1976; 16	0.27347764	0.04776897		14.0
	ZINC02091879	-8.8	430.459	-1.613	10	4	307.54	0.35911	168.4	-0.5761	274.8265485281	0.29035	8.1739	-5.5554	0.6129	0.52678	0.9112 (max of	2	3583; 1979	0.19543621	0.06065784		14.0
	ZINC02102770	-8.8	385.327	-3.6824	9	0	279.13	0.38828	140.01	-6.1037	274.8265485281	0.32146	10.243	-11.455	0.46429	0.5437	0.96211 (max o	5	1369; 1090; 1079; 33	0.1647302	0.4833842		11.0
	ZINC02104410	-8.8	385.351	-1.9192	9	2	284.22	0.39923	137.77	1.0496	274.8265485281	0.32146	8.4801	-5.9703	0.57143	0.52209	0.96832 (max o	4	3141; 3135; 1354; 54	0.09656764	0.1994706		11.0
	ZINC15675856	-8.8	401.809	-1.1583	9	2	280.14	0.33537	129.29	3.7497	274.8265485281	0.32146	7.7192	-3.6033	0.53571	0.43733	0.97773 (max o	9	1591; 1519; 223; 224	0.33130714	-0.0851325		10.0
	ZINC03840169	-8.8	398.390	-1.7406	10	7	281.5	0.45496	174.01	0.57717	274.8265485281	0.32146	8.3015	-5.4147	0.53571	0.49726	0.9308 (max of	4	1374; 1105; 886; 703	-0.4323414	-0.3384762		10.0
	ZINC03840203	-8.8	394.426	-1.4024	10	7	290.17	0.44136	174.01	1.7681	274.8265485281	0.32146	7.9633	-4.3626	0.60714	0.52028	0.97941 (max o	6	1861; 1373; 1106; 11	-0.4218276	-0.3746951		10.0
	ZINC03840204	-8.8	398.390	-1.6455	10	7	284.26	0.45054	174.01	0.4308	274.8265485281	0.32146	8.2064	-5.1189	0.57143	0.52028	0.9308 (max of	5	6089; 1374; 1106; 88	-0.4121887	-0.3337156		10.0
	ZINC03840201	-8.8	417.820	-1.0334	9	6	288.22	0.40459	161.98	0.45749	274.8265485281	0.32146	7.5943	-3.2147	0.57143	0.49667	0.96567 (max o	5	2010; 1663; 1381; 11	-0.4210904	-0.5173999		10.0
	ZINC04963991	-8.8	448.379	-0.4237	11	8	295.54	0.4664	197.37	-1.9977	274.8265485281	0.28128	6.9846	-1.5064	0.4375	0.38342	0.95465 (max o	9	3891; 2828; 2629; 25	0.7616349	0.04718343		10.0
	ZINC20463699	-8.8	415.449	-0.3355	10	3	303.4	0.33869	127.16	1.6386	274.8265485281	0.30003	6.8964	-1.1182	0.56667	0.46966	0.93975 (max o	6	3838; 3388; 2588; 22	-0.6304931	-0.2661548		10.0
	ZINC35442308	-8.8	403.520	0.3596	6	2	303.27	0.17272	69.26	2.8741	274.8265485281	0.31037	6.2013	1.1586	0.62069	0.4748	0.97415 (max o	10	2653; 1598; 1302; 13	-0.552236	0.11934838		9.0
	ZINC04349814	-8.8	416.381	-0.4408	9	6	282.84	0.39471	156.91	-2.3989	274.8265485281	0.30003	7.0017	-1.4692	0.5	0.39653	0.95579 (max o	7	2463; 1427; 1164; 96	0.73465496	0.16277382		9.0
	ZINC12429156	-8.8	418.397	0.5121	9	5	288.04	0.37682	145.91	-3.7146	274.8265485281	0.30003	6.0488	1.7068	0.53333	0.39625	0.9707 (max of	9	1223; 1037; 200; 368	0.05382285	-0.3897863		8.0
	ZINC33832982	-8.8	418.397	0.5121	9	5	288.04	0.37682	145.91	-3.7146	274.8265485281	0.30003	6.0488	1.7068	0.53333	0.39579	0.9707 (max of	8	6202; 1058; 194; 312	0.03513922	-0.3797822		8.0
	ZINC49601392	-8.8	432.424	0.7878	9	4	303.95	0.3469	134.91	-3.5326	274.8265485281	0.29035	5.7731	2.7133	0.54839	0.40753	0.95804 (max o	4	6202; 312; 345; 1037	0.03683539	-0.3588321		8.0
	ZINC08952692	-8.8	443.478	-4.0406	10	3	333.3	0.37264	173.1	4.2721	274.8265485281	0.28128	10.602	-14.365	0.59375	0.49532	0.94476 (max o	9	6947; 4629; 3200; 18	0.30469623	0.25711724		8.0
	ZINC34933706	-8.8	432.380	-0.078	10	7	289.19	0.43134	177.14	-1.9977	274.8265485281	0.29035	6.6389	-0.26864	0.51613	0.3816	0.96018 (max o	10	3788; 3213; 1812; 14	0.6822407	0.08746667		8.0
	ZINC13136670	-8.8	432.380	-0.078	10	7	289.19	0.43134	177.14	-1.9977	274.8265485281	0.29035	6.6389	-0.26864	0.51613	0.3811	0.96018 (max o	11	3788; 3213; 2453; 21	0.6815071	0.10906887		8.0
	ZINC04654606	-8.8	448.379	-0.4237	11	8	295.54	0.4664	197.37	-1.9977	274.8265485281	0.28128	6.9846	-1.5064	0.5	0.38342	0.96186 (max o	12	3282; 3243; 2828; 28	0.6936019	0.02250579		8.0
	ZINC08551391	-8.8	448.379	-0.4237	11	8	295.54	0.4664	197.37	-1.9977	274.8265485281	0.28128	6.9846	-1.5064	0.5	0.38342	0.96186 (max o	11	3282; 3243; 2828; 28	0.6848831	0.06547989		8.0
	ZINC04557044	-8.8	351.286	-3.185	9	3	237.81	0.46112	145.58	-4.8874	274.8265485281	0.36003	9.7459	-8.8464	0.48	0.39879	0.94351 (max o	4	2015; 1686; 585; 747	-0.9045829	-0.4072053		8.0

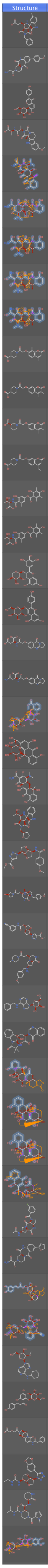

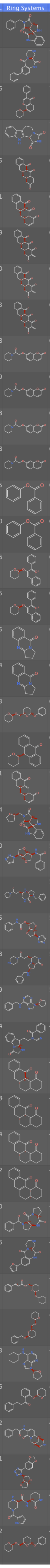

Structure	Molecule Name	affinity	Total Molweight	cLogP	H-Acceptors	H-Donors	Total Surface Area	Relative PSA	Polar Surface Area	Druglikeness	Kd (nM) @ 20°C	LE from K...	LLE from Kd ...	LELP from Kd (n...	Shape Index	Molecular...	Neighbor Si...	Neig...	Neighbor	Neighbor ...	Neighbor ...	Ring Systems	frequency
	ZINC15957168	-8.8	432.455	-3.3242	8	2	305.72	0.30391	118.64	4.3827	274.8265485281	0.28128	9.8851	-11.818	0.40625	0.29791	0.9642 (max of	10	30; 112; 230; 375; 37	0.18740346	-0.534286		7.0
	ZINC04236049	-8.8	395.438	-2.8471	8	2	288.86	0.28443	106.59	4.7938	274.8265485281	0.31037	9.408	-9.1731	0.58621	0.3741	0.9339 (max of	6	1713; 261; 262; 346;	-0.2042447	0.2625464		7.0
	ZINC12662110	-8.8	416.381	-0.4408	9	6	282.84	0.39471	156.91	-2.3989	274.8265485281	0.30003	7.0017	-1.4692	0.5	0.39653	0.93965 (max o	7	2463; 1427; 1164; 54	0.7159917	0.15326186		7.0
	ZINC08789706	-8.8	427.436	-1.5754	10	2	302.99	0.35971	134.87	5.8811	274.8265485281	0.29035	8.1363	-5.4259	0.51613	0.37777	0.9313 (max of	3	2785; 1928; 711	-0.4498782	0.339037		7.0
	ZINC35307838	-8.8	444.439	-4.7179	10	5	308.31	0.44955	182.49	2.6033	274.8265485281	0.28128	11.279	-16.773	0.375	0.37412	0.95709 (max o	2	1317; 401	0.56996286	-0.7380045		6.0
	ZINC35307838	-8.8	443.431	-6.7939	10	4	309.49	0.45165	185.32	2.064	274.8265485281	0.28128	13.355	-24.154	0.375	0.37412	0.86904 (max o	3	154; 170; 1045	0.1741439	-0.3221571		6.0
	ZINC35307838	-8.8	443.431	-3.1899	10	4	294.27	0.42328	181.29	3.986	274.8265485281	0.28128	9.7508	-11.341	0.375	0.35013	0.99867 (max o	3	1048; 153; 1045	0.12369106	-0.3139939		6.0
	ZINC35307838	-8.8	444.439	-4.5562	10	5	308.31	0.44955	182.49	1.0562	274.8265485281	0.28128	11.117	-16.198	0.375	0.35173	0.87498 (max o	4	1048; 150; 1049; 104	0.15884274	-0.2933250		6.0
	ZINC35307838	-8.8	443.431	-3.1899	10	4	294.27	0.42328	181.29	3.986	274.8265485281	0.28128	9.7508	-11.341	0.375	0.35013	0.99867 (max o	4	1314; 153; 1045; 104	0.12020329	-0.2925553		6.0
	ZINC02117968	-8.8	358.369	-0.7503	7	0	267.87	0.28727	95.97	-1.6318	274.8265485281	0.34619	7.3112	-2.1673	0.57692	0.43833	0.94622 (max o	3	187; 551; 552	0.11298299	0.20787828		6.0
	ZINC02116764	-8.8	358.369	-0.654	7	0	266.08	0.2892	95.97	2.7813	274.8265485281	0.34619	7.2149	-1.8892	0.61538	0.51964	0.94326 (max o	4	946; 131; 468; 873	0.16541207	0.13419499		6.0
	ZINC08764448	-8.8	358.369	-0.654	7	0	266.08	0.2892	95.97	2.1725	274.8265485281	0.34619	7.2149	-1.8892	0.57692	0.51964	0.96998 (max o	2	468; 871	0.14320983	0.14006518		6.0
	ZINC02119771	-8.8	358.369	-0.6398	7	0	269.37	0.28567	95.97	2.8531	274.8265485281	0.34619	7.2007	-1.8481	0.61538	0.53167	0.90719 (max o	4	1650; 131; 551; 871	0.14028987	0.16066428		6.0
	ZINC31165189	-8.8	446.431	-0.2867	10	5	326.96	0.38922	176.45	1.5646	274.8265485281	0.28128	6.8476	-1.0193	0.5	0.51411	0.99059 (max o	3	821; 822; 1028	-0.9361993	-0.0603995		6.0
	ZINC31165186	-8.8	446.431	-0.2867	10	5	326.96	0.38922	176.45	1.5646	274.8265485281	0.28128	6.8476	-1.0193	0.5	0.51261	0.99059 (max o	3	821; 822; 1029	-0.9106091	-0.0458970		6.0
	ZINC67912612	-8.8	435.360	-0.095	11	8	276.47	0.45141	180.3	-8.811	274.8265485281	0.29035	6.6559	-0.32719	0.41935	0.26184	0.85572	1	2421	-0.8280843	0.27055606		6.0
	ZINC04102353	-8.8	433.388	-0.0056002	10	7	282.62	0.39523	160.07	-8.6993	274.8265485281	0.29035	6.5665	-0.019288	0.41935	0.26223	0.93418 (max o	3	2963; 2796; 2421	-0.8425379	0.28527275		6.0
	ZINC12902007	-8.8	373.320	-5.224	10	1	268.35	0.43689	151.26	5.6478	274.8265485281	0.33336	11.785	-15.671	0.55556	0.47784	0.92418 (max o	6	5494; 3348; 2546; 21	0.17049222	0.50442505		6.0
	ZINC12899118	-8.8	373.344	-3.5458	10	2	269.34	0.43896	154.22	6.2941	274.8265485281	0.33336	10.107	-10.636	0.55556	0.46755	0.96023 (max o	6	5494; 3348; 2546; 21	0.19092824	0.53397244		6.0
	ZINC31155511	-8.8	446.403	-1.9975	12	6	304.39	0.46532	184.6	-6.8024	274.8265485281	0.29035	8.5584	-6.8796	0.51613	0.42156	0.87798 (max o	4	5613; 2328; 2273; 11	-0.3988044	0.35350603		6.0
	ZINC35271224	-8.8	434.396	-1.4258	10	8	285	0.44856	188.14	-2.6646	274.8265485281	0.29035	7.9867	-4.9106	0.35484	0.33408	0.98656 (max o	7	6314; 5513; 5512; 46	-0.5170978	0.5436708		6.0
	ZINC18152146	-8.8	443.431	-3.4078	10	5	292.46	0.42611	184.45	5.1912	274.8265485281	0.28128	9.9687	-12.116	0.375	0.3646	0.94718 (max o	8	22; 43; 75; 115; 122;	-0.1388844	-0.5573631		5.0
	ZINC20758725	-8.8	385.419	-0.8469	8	3	262.29	0.33814	107.97	3.0652	274.8265485281	0.32146	7.4078	-2.6346	0.42857	0.23091	0.98866 (max o	9	3418; 1880; 1878; 12	0.07941729	0.82913494		5.0
	ZINC04260923	-8.8	388.379	-0.2614	10	1	281.74	0.37748	113.8	-3.2548	274.8265485281	0.32146	6.8223	-0.81317	0.64286	0.3686	0.93379 (max o	5	1735; 1724; 1725; 14	-0.2716172	-0.2328510		5.0
	ZINC35442586	-8.8	431.555	-1.536	8	3	339.37	0.25144	86.55	6.0347	274.8265485281	0.29035	8.0969	-5.2902	0.6129	0.47114	0.95031 (max o	4	3026; 666; 837; 838	0.02230925	0.5774417		5.0
	ZINC04260878	-8.8	399.362	0.1123	11	1	291.19	0.41179	137.59	-17.082	274.8265485281	0.31037	6.4486	0.36182	0.62069	0.3286	0.89412 (max o	2	1062; 733	-0.2461295	-0.0041075		5.0
	ZINC22922327	-8.8	430.571	-0.8658	8	2	327.98	0.20102	81.73	9.1745	274.8265485281	0.29035	7.4267	-2.9819	0.64516	0.48679	0.97199 (max o	11	3876; 1563; 1278; 12	-0.5718946	0.07416338		5.0
	ZINC01318428	-8.8	357.369	0.0027999	9	4	257.63	0.39025	125.55	-0.31782	274.8265485281	0.34619	6.5581	0.0080878	0.57692	0.36038	0.93361 (max o	9	3229; 2807; 2417; 23	-0.4204409	-0.7281699		5.0
	ZINC31160051	-8.8	433.443	-3.2187	9	2	300.57	0.32129	125.37	6.2494	274.8265485281	0.28128	9.7796	-11.443	0.40625	0.39447	0.95949 (max o	8	179; 238; 239; 240; 3	-0.7569258	0.11067064		4.0
	ZINC31158958	-8.8	360.361	0.1524	7	5	235.62	0.38868	135.29	-0.37577	274.8265485281	0.34619	6.4085	0.44023	0.42308	0.30051	0.9464 (max of	3	1898; 304; 406	0.8301269	-0.470729		4.0
	ZINC35270769	-8.8	376.360	-0.671	8	6	239.88	0.43638	155.52	0.76178	274.8265485281	0.33336	7.2319	-2.0128	0.40741	0.31342	0.9578 (max of	3	1295; 407; 1042	-0.4629961	-0.7210898		4.0
	ZINC35270766	-8.8	376.360	-0.671	8	6	239.88	0.43638	155.52	0.76178	274.8265485281	0.33336	7.2319	-2.0128	0.40741	0.31342	0.9578 (max of	3	1295; 407; 1043	-0.4420184	-0.7252414		4.0
	ZINC35458755	-8.8	376.360	-0.671	8	6	239.88	0.43638	155.52	-0.097646	274.8265485281	0.33336	7.2319	-2.0128	0.40741	0.31342	0.97149 (max o	3	1918; 410; 522	-0.8835541	-0.3816152		4.0
	ZINC03839255	-8.8	388.398	-0.1676	8	2	270.93	0.30333	98.82	4.4287	274.8265485281	0.32146	6.7285	-0.52137	0.5	0.38093	0.94823 (max o	3	1377; 429; 701	-0.4227372	-0.2395340		4.0
	ZINC12604956	-8.8	368.348	-0.7302	9	2	261.07	0.38342	125.95	3.7847	274.8265485281	0.33336	7.2911	-2.1904	0.51852	0.33809	0.90776 (max o	4	3679; 2430; 1136; 44	-0.1514624	0.35832196		4.0
	ZINC49601732	-8.8	440.443	0.0769	10	6	314.91	0.38627	166.14	-6.1546	274.8265485281	0.29035	6.484	0.26485	0.58065	0.46737	0.96262 (max o	6	3531; 3036; 1617; 53	-0.2596256	0.566524		4.0
	ZINC34934401	-8.8	427.405	-2.4776	11	6	298.59	0.44261	182.09	-10.383	274.8265485281	0.30003	9.0385	-8.2579	0.53333	0.47708	0.95837 (max o	5	1583; 1224; 530; 841	-0.4680410	0.7163461		4.0
	ZINC00597419	-8.8	363.416	0.5683	9	3	267.82	0.36383	114.55	-3.3637	274.8265485281	0.34619	5.9926	1.6416	0.53846	0.3046	0.94008 (max o	3	4249; 2168; 1814	-0.3974566	-0.8394473		4.0
	ZINC03874670	-8.8	436.412	0.055	10	7	305.5	0.40831	177.14	-4.8731	274.8265485281	0.29035	6.5059	0.18943	0.51613	0.43522	0.95521 (max o	10	5085; 3799; 3620; 32	0.8098498	-0.3003526		4.0
	ZINC04237054	-8.8	438.459	-1.7984	9	1	324.09	0.29313	119.08	4.7225	274.8265485281	0.28128	8.3593	-6.3937	0.625	0.47435	0.97319 (max o	4	4973; 2435; 1411; 26	-0.50614	-0.4618502		4.0
	ZINC04270575	-8.8	388.383	0.3717	11	2	275.97	0.41939	121.65	1.8678	274.8265485281	0.32146	6.1892	1.1563	0.57143	0.32036	0.90883 (max o	4	1737; 1420; 450; 914	-0.1774984	-0.3956321		4.0
	ZINC08299450	-8.8	418.496	-0.412	10	3	318.28	0.31579	115.78	7.6175	274.8265485281	0.30003	6.9729	-1.3732	0.43333	0.50572	0.91248 (max o	5	5815; 2863; 1179; 48	-0.0864313	0.2724327		4.0
	ZINC13376174	-8.8	434.392	-2.3262	12	7	292.99	0.48363	187.76	-4.801	274.8265485281	0.30003	8.8871	-7.7533	0.53333	0.45643	0.96092 (max o	6	2951; 2950; 2285; 22	0.61266685	0.44084832		4.0

Structure	Molecule Name	affinity	Total Molweight	cLogP	H-Acceptors	H-Donors	Total Surface Area	Relative PSA	Polar Surface Area	Druglikeness	Kd (nM) @ 20°C	LE from K...	LLE from Kd ...	LELP from Kd (n...	Shape Index	Molecular...	Neighbor Si...	Neig...	Neighbor	Neighbor ...	Neighbor ...	Ring Systems	frequency
	ZINC13376176	-8.8	434.392	-2.3262	12	7	292.99	0.48363	187.76	-4.801	274.8265485281	0.30003	8.8871	-7.7533	0.53333	0.45643	0.96092 (max c	6	2951; 2950; 2285; 22	0.59822494	0.42691246		4.0
	ZINC15708225	-8.8	444.554	-1.8114	9	3	349.73	0.27658	95.42	4.8327	274.8265485281	0.28128	8.3723	-6.4399	0.59375	0.48712	0.92326 (max c	4	2200; 1840; 1240; 98	-0.1372725	0.1379744		4.0
	ZINC18028709	-8.8	386.267	-3.6918	10	2	252.84	0.48995	165.48	-1.8365	274.8265485281	0.32146	10.253	-11.485	0.35714	0.26587	0.93985 (max c	2	1753; 986	0.89863133	0.32657763		4.0
	ZINC18028709	-8.8	386.267	-3.1938	10	2	252.84	0.48995	165.48	-1.7419	274.8265485281	0.32146	9.7547	-9.9354	0.35714	0.27575	0.93985 (max c	2	1752; 987	0.8824943	0.35448506		4.0
	ZINC31162094	-8.8	370.312	1.0015	8	3	243.66	0.40392	130.36	0.8612	274.8265485281	0.33336	5.5594	3.0042	0.44444	0.24731	0.95493 (max c	3	4806; 2265; 1900	-0.2576971	-0.8284929		4.0
	ZINC49601716	-8.8	440.443	0.0769	10	6	314.91	0.38627	166.14	-6.4176	274.8265485281	0.29035	6.484	0.26485	0.54839	0.405	0.96502 (max c	8	3531; 3530; 3090; 30	-0.2612376	0.51217335		4.0
	ZINC67913748	-8.8	421.464	-0.9847	8	2	305.6	0.32592	133.19	-8.4075	274.8265485281	0.30003	7.5456	-3.282	0.46667	0.47687	0.96832 (max c	3	3543; 1942; 1337	-0.9194618	-0.2483282		4.0
	ZINC71404468	-8.8	434.396	0.3932	10	6	291.63	0.4171	166.14	1.8995	274.8265485281	0.29035	6.1677	1.3542	0.41935	0.35036	0.96502 (max c	4	5273; 2275; 1943; 13	0.7069482	-0.5189009		4.0
	ZINC72320457	-8.8	431.416	-0.8675	9	4	291.67	0.3866	156.58	-1.1474	274.8265485281	0.29035	7.4284	-2.9878	0.3871	0.27839	0.97199 (max c	3	3115; 2327; 2326	-0.7929103	-0.5302037		4.0
	ZINC12605018	-8.8	387.439	-1.1773	9	4	294.98	0.35545	129.55	8.1198	274.8265485281	0.32146	7.7382	-3.6624	0.53571	0.52325	0.93373 (max c	3	217; 218; 959	-0.2661835	0.21686865		3.0
	ZINC12605012	-8.8	357.413	-0.6785	8	4	271.22	0.34972	120.32	8.4009	274.8265485281	0.34619	7.2394	-1.9599	0.5	0.51093	0.93373 (max c	4	217; 218; 736; 960	-0.2731180	0.23748165		3.0
	ZINC18152146	-8.8	443.431	-6.8501	10	5	307.68	0.4545	188.48	2.9767	274.8265485281	0.28128	13.411	-24.354	0.375	0.36615	0.9497 (max of	4	117; 124; 169; 391	-0.3288978	0.5991144		3.0
	ZINC26745304	-8.8	422.385	0.1214	10	7	282.99	0.43005	169.3	-4.0605	274.8265485281	0.30003	6.4395	0.40463	0.53333	0.3809	0.96092 (max c	7	1937; 1332; 1306; 12	0.3874851	-0.8247256		3.0
	ZINC03839367	-8.8	422.843	0.4384	8	2	286.35	0.28699	98.82	5.8161	274.8265485281	0.31037	6.1225	1.4125	0.51724	0.34107	0.8973	1	1376	-0.4526113	-0.2665364		3.0
	ZINC12603833	-8.8	435.543	-1.499	9	4	321.41	0.25152	116.48	7.1204	274.8265485281	0.29035	8.0599	-5.1627	0.6129	0.54563	0.91903 (max c	4	1517; 1467; 1208; 10	-0.1228788	-0.6101289		3.0
	ZINC13412855	-8.8	449.387	-0.3513	11	8	288.97	0.43188	180.3	-8.6993	274.8265485281	0.28128	6.9122	-1.249	0.40625	0.27157	0.95709 (max c	6	3350; 3349; 2963; 27	-0.8286208	0.32826582		3.0
	ZINC13518248	-8.8	387.395	-0.5073	10	5	276.48	0.41102	145.78	-0.80219	274.8265485281	0.32146	7.0682	-1.5781	0.53571	0.40844	0.9308 (max of	6	2948; 1430; 1161; 35	-0.4403241	-0.6890567		3.0
	ZINC15268997	-8.8	434.396	-0.258	10	7	290.74	0.42904	177.14	-2.4912	274.8265485281	0.29035	6.8189	-0.88859	0.51613	0.39907	0.96018 (max c	6	3370; 1907; 1909; 19	-0.7664040	-0.2103329		3.0
	ZINC15708587	-8.8	444.554	-1.6684	9	3	349.73	0.27658	95.42	9.0329	274.8265485281	0.28128	8.2293	-5.9315	0.625	0.4813	0.93686 (max c	4	1840; 1525; 1524; 98	-0.1388423	0.11612987		3.0
	ZINC22922327	-8.8	429.563	-3.9204	8	2	347.03	0.24488	84.67	7.8679	274.8265485281	0.29035	10.481	-13.502	0.64516	0.52045	0.94501 (max c	4	1882; 1274; 1270; 12	-0.1457258	0.21148886		3.0
	ZINC31168399	-8.8	422.385	-0.7892	10	8	288.09	0.44375	188.14	-3.5267	274.8265485281	0.30003	7.3501	-2.6304	0.53333	0.49298	0.83694	1	1896	-0.6793133	0.6824259		3.0
	ZINC67910235	-8.8	389.332	-4.9553	11	4	258.42	0.51374	175.04	-4.8669	274.8265485281	0.33336	11.516	-14.865	0.44444	0.42874	0.94605 (max c	2	3101; 1064	0.60889995	0.54192406		3.0
	ZINC67910238	-8.8	389.332	-4.9553	11	4	258.42	0.51374	175.04	-4.8669	274.8265485281	0.33336	11.516	-14.865	0.44444	0.42874	0.94605 (max c	2	3957; 1063	0.5912551	0.5551965		3.0
	ZINC72326279	-8.8	396.354	-1.9403	9	1	292.45	0.37836	140.18	3.7716	274.8265485281	0.31037	8.5012	-6.2515	0.58621	0.53077	0.97878 (max c	4	3118; 2699; 1627; 10	-0.5650084	-0.3034566		3.0
	ZINC72326281	-8.8	396.354	-1.9403	9	1	292.45	0.37836	140.18	3.7716	274.8265485281	0.31037	8.5012	-6.2515	0.58621	0.53077	0.97878 (max c	4	3119; 2699; 1627; 10	-0.555119	-0.2851687		3.0
	ZINC31170345	-8.8	443.467	-3.2526	10	5	300.11	0.40925	168.97	-2.3795	274.8265485281	0.29035	9.8135	-11.202	0.48387	0.4312	0.96018 (max c	3	310; 1034; 1032	-0.7805879	-0.4978295		2.0
	ZINC08299578	-8.8	403.461	-4.3173	10	4	301.29	0.35886	138.49	3.8116	274.8265485281	0.31037	10.878	-13.91	0.51724	0.52038	0.91704 (max c	3	3697; 355; 598	-0.8192661	-0.0835067		2.0
	ZINC20758204	-8.8	405.389	-1.2568	9	3	295.46	0.36181	134.43	6.1598	274.8265485281	0.30003	7.8177	-4.1889	0.53333	0.49699	0.97757 (max c	2	396; 696	0.08670625	-0.3014911		2.0
	ZINC08879795	-8.8	438.415	-1.5386	10	1	314.91	0.33343	128.31	5.0442	274.8265485281	0.28128	8.0995	-5.4701	0.5	0.48592	0.90345 (max c	4	3787; 2164; 291; 470	0.73981506	-0.3176300		2.0
	ZINC19703028	-8.8	410.402	-1.1622	10	2	261.6	0.4099	123.63	3.9896	274.8265485281	0.32146	7.7231	-3.6154	0.53571	0.41245	0.96253 (max c	2	2029; 484	-0.5881285	-0.7536057		2.0
	ZINC31160051	-8.8	434.451	-1.4164	9	2	296.85	0.31278	129.95	4.3792	274.8265485281	0.28128	7.9773	-5.0356	0.40625	0.39447	0.9642 (max of	3	178; 237; 507	-0.7839756	-0.1308518		2.0
	ZINC31166753	-8.8	448.379	0.5308	11	6	300.81	0.43762	175.37	-3.6847	274.8265485281	0.28128	6.0301	1.8871	0.46875	0.38558	0.95493 (max c	2	1566; 509	-0.6605103	0.31836638		2.0
	ZINC04044047	-8.8	368.364	-2.0163	7	0	240.78	0.30696	88.13	1.713	274.8265485281	0.33336	8.5772	-6.0483	0.44444	0.17486	0.93058 (max c	4	1393; 1394; 560; 561	-0.6841827	0.00209087		2.0
	ZINC13859683	-8.8	418.397	0.5121	9	5	288.04	0.37682	145.91	-3.7146	274.8265485281	0.30003	6.0488	1.7068	0.53333	0.42989	0.97304 (max c	5	6202; 1037; 312; 603	0.03427707	-0.3998699		2.0
	ZINC22922137	-8.8	410.536	0.1304	6	2	304.53	0.15742	61.78	6.8344	274.8265485281	0.30003	6.4305	0.43462	0.6	0.46447	0.94488 (max c	3	2612; 641; 1014	0.4943419	-0.1700107		2.0
	ZINC20504127	-8.8	435.567	0.0948	8	5	306.46	0.30412	133.33	6.0621	274.8265485281	0.30003	6.4661	0.31597	0.6	0.48037	0.92566 (max c	2	5576; 645	-0.6435696	0.6851498		2.0
	ZINC35442296	-8.8	421.947	-0.2992	7	2	308.58	0.20215	78.49	4.3876	274.8265485281	0.31037	6.8601	-0.964	0.58621	0.4748	0.93686 (max c	7	1598; 1303; 1301; 40	-0.604995	0.11768073		2.0
	ZINC35456525	-8.8	413.365	-2.6382	11	3	291.2	0.44784	166.17	1.3595	274.8265485281	0.30003	9.1991	-8.7932	0.53333	0.40587	0.95053 (max c	8	221; 242; 296; 373; 5	0.23233789	-0.770299		2.0
	ZINC04201124	-8.8	449.506	-1.8648	11	2	322.93	0.33682	128.36	7.4561	274.8265485281	0.28128	8.4257	-6.6298	0.5625	0.39206	0.94751 (max c	6	2750; 2375; 2002; 17	-0.5538069	-0.0050345		2.0
	ZINC04517559	-8.8	441.403	-6.3118	13	5	323.08	0.51025	213.26	4.3834	274.8265485281	0.28128	12.873	-22.44	0.625	0.58514	0.98148 (max c	4	1640; 720; 722; 900	0.8568306	0.22494817		2.0
	ZINC20504127	-8.8	434.559	0.0255	8	4	317.97	0.32931	131.72	6.4008	274.8265485281	0.30003	6.5354	0.084992	0.6	0.44025	0.97975	1	771	0.5150489	0.14571314		2.0
	ZINC03977786	-8.8	434.396	-0.3162	10	7	290.74	0.42904	177.14	-4.2658	274.8265485281	0.29035	6.8771	-1.089	0.3871	0.32724	0.97642 (max c	3	2050; 2049; 1388	-0.4997979	0.6444121		2.0
	ZINC03999068	-8.8	414.453	-1.3791	10	8	289.06	0.4204	163.9	1.6633	274.8265485281	0.31037	7.94	-4.4434	0.51724	0.46749	0.95908 (max c	3	2398; 1114; 1115	0.9196807	-0.3264526		2.0

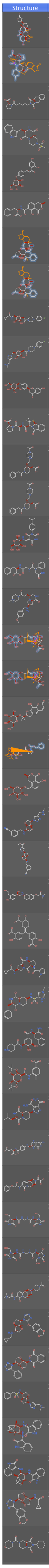

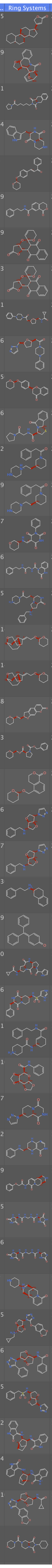

Structure	Molecule Name	affinity	Total Molweight	cLogP	H-Acceptors	H-Donors	Total Surface Area	Relative PSA	Polar Surface Area	Druglikeness	Kd (nM) @ 20°C	LE from K...	LLE from Kd ...	LELP from Kd (n...	Shape Index	Molecular...	Neighbor Si...	Neig...	Neighbor	Neighbor ...	Neighbor ...	Ring Systems	frequency
	ZINC04025854	-8.8	419.492	-1.2533	7	3	284.29	0.31538	127.12	0.12303	274.8265485281	0.30003	7.8142	-4.1773	0.46667	0.36325	0.88633 (max o	4	2031; 1395; 134; 562	0.30539206	0.85443735		2.0
	ZINC04044100	-8.8	352.365	-1.6024	6	0	230.78	0.27693	78.9	1.607	274.8265485281	0.34619	8.1633	-4.6287	0.42308	0.1538	0.93367 (max o	4	1394; 1117; 340; 561	-0.6809531	-0.0204729		2.0
	ZINC04090519	-8.8	412.464	-0.4261	8	0	304.79	0.25293	101.06	-1.2035	274.8265485281	0.30003	6.987	-1.4202	0.56667	0.47958	0.95123 (max o	3	2794; 2419; 1695	0.2207246	-0.8938381		2.0
	ZINC04270897	-8.8	376.436	-0.2008	8	2	258.87	0.33689	110.96	9.5952	274.8265485281	0.34619	6.7617	-0.58004	0.53846	0.44577	0.85775 (max o	2	3238; 1155	-0.6590583	-0.7102264		2.0
	ZINC05119394	-8.8	418.397	0.2774	9	6	298.13	0.37447	156.91	-3.3049	274.8265485281	0.30003	6.2835	0.92458	0.6	0.44836	0.95579 (max o	4	3468; 2830; 2461; 17	0.45665592	0.4518394		2.0
	ZINC05260598	-8.8	368.364	-0.2877	7	2	272.89	0.32233	115.76	2.3639	274.8265485281	0.33336	6.8486	-0.86302	0.55556	0.51163	0.96343	1	2462	0.727026	-0.4400709		2.0
	ZINC05533356	-8.8	386.355	-0.4899	8	2	250.22	0.38094	119.36	-1.0904	274.8265485281	0.32146	7.0508	-1.524	0.35714	0.28247	0.95983 (max o	3	458; 756; 933	0.8573161	-0.1175729		2.0
	ZINC05515784	-8.8	386.355	-0.4899	8	2	250.22	0.38094	119.36	-1.0904	274.8265485281	0.32146	7.0508	-1.524	0.35714	0.28247	0.95983 (max o	3	458; 934; 756	0.88205135	-0.1018103		2.0
	ZINC08627175	-8.8	421.467	-0.0343	8	3	301.95	0.26773	102.34	5.5189	274.8265485281	0.30003	6.5952	-0.11432	0.63333	0.52467	0.94529 (max o	5	1540; 1463; 1191; 58	-0.0261947	-0.5981961		2.0
	ZINC08627190	-8.8	449.505	-2.6041	10	3	326.46	0.28454	120.56	-0.021309	274.8265485281	0.28128	9.165	-9.2582	0.65625	0.53842	0.9499	1	1192	-0.8255966	-0.5031656		2.0
	ZINC08681832	-8.8	418.397	0.5121	9	5	288.04	0.37682	145.91	-3.7146	274.8265485281	0.30003	6.0488	1.7068	0.53333	0.42756	0.97304 (max o	8	6202; 1223; 954; 368	0.03625535	-0.4192045		2.0
	ZINC08790079	-8.8	430.504	-0.7803	8	1	299.31	0.34242	135.15	8.4506	274.8265485281	0.30003	7.3412	-2.6008	0.46667	0.3382	0.93965 (max o	5	6123; 4082; 2786; 25	0.3659606	-0.6316336		2.0
	ZINC20463233	-8.8	415.532	-0.7752	7	1	340.6	0.22927	85.19	4.7027	274.8265485281	0.30003	7.3361	-2.5838	0.56667	0.60939	0.95837 (max o	6	5913; 3761; 2982; 18	-0.0512248	-0.2106372		2.0
	ZINC20463233	-8.8	414.524	-0.9068	7	0	326.56	0.19614	83.99	9.4368	274.8265485281	0.30003	7.4677	-3.0224	0.56667	0.61291	0.93686 (max o	6	5914; 5912; 4209; 33	-0.1617628	-0.1762369		2.0
	ZINC20610925	-8.8	435.432	-1.4606	11	6	306.12	0.4181	168.66	4.3334	274.8265485281	0.29035	8.0215	-5.0305	0.54839	0.43428	0.93405	1	3413	0.9102671	0.19886947		2.0
	ZINC20758674	-8.8	395.394	-1.9455	9	3	296.51	0.35199	130.67	6.3396	274.8265485281	0.31037	8.5064	-6.2683	0.55172	0.56253	0.96931 (max o	3	2243; 1264; 1259	0.5940836	-0.1817916		2.0
	ZINC22922327	-8.8	431.579	-2.9755	8	3	342.02	0.23382	82.93	4.8955	274.8265485281	0.29035	9.5364	-10.248	0.64516	0.48679	0.92326 (max o	6	3876; 1563; 1277; 12	-0.5351904	0.06761825		2.0
	ZINC31170348	-8.8	443.467	-3.2526	10	5	300.11	0.40925	168.97	-2.3795	274.8265485281	0.29035	9.8135	-11.202	0.48387	0.42593	0.95771 (max o	3	310; 1032; 1033	-0.7817222	-0.4768021		2.0
	ZINC31170342	-8.8	443.467	-3.2526	10	5	300.11	0.40925	168.97	-2.3795	274.8265485281	0.29035	9.8135	-11.202	0.48387	0.42633	0.96018 (max o	3	1034; 310; 1033	-0.7642354	-0.4896641		2.0
	ZINC31502634	-8.8	424.401	-0.3217	10	5	286.21	0.41417	155.14	-7.4042	274.8265485281	0.30003	6.8826	-1.0722	0.5	0.46107	0.91333 (max o	3	1904; 1293; 1130	-0.766232	-0.3021728		2.0
	ZINC35361901	-8.8	411.405	-0.4553	10	4	291.26	0.38464	145.99	0.28886	274.8265485281	0.31037	7.0162	-1.4669	0.58621	0.50894	0.95908 (max o	3	2532; 2158; 1844	0.08351873	-0.9528063		2.0
	ZINC35457536	-8.8	370.353	-1.0161	9	5	254.3	0.42682	145.91	-4.9227	274.8265485281	0.34619	7.577	-2.9351	0.46154	0.4923	0.95257 (max o	3	1607; 1308; 672	-0.5815166	0.7717676		2.0
	ZINC67899016	-8.8	369.447	-2.0946	8	2	299.81	0.30493	89.43	-6.7896	274.8265485281	0.33336	8.6555	-6.2832	0.62963	0.45178	0.88391 (max o	3	1150; 1062; 733	-0.2126767	-0.0500946		2.0
	ZINC67899018	-8.8	369.380	-0.4382	9	1	277.2	0.34946	111.29	-8.3398	274.8265485281	0.33336	6.9991	-1.3145	0.62963	0.42729	0.88391 (max o	2	909; 1061	-0.2245103	-0.0090477		2.0
	ZINC00584203	-8.8	429.535	0.2387	7	4	330.2	0.22299	106.84	-0.36081	274.8265485281	0.29035	6.3222	0.82211	0.6129	0.46452		0		-0.3927361	-0.8999303		1.0
	ZINC03833863	-8.8	419.320	-5.6966	9	2	299.94	0.40408	177.92	1.8394	274.8265485281	0.29035	12.258	-19.62	0.35484	0.45959		0		-0.074035	-0.7743859		1.0
	ZINC03839298	-8.8	399.450	-1.041	9	2	280.3	0.3177	103.75	8.6579	274.8265485281	0.31037	7.6019	-3.354	0.48276	0.28706	0.94573 (max o	11	3818; 3164; 2749; 23	-0.5947287	-0.0712180		1.0
	ZINC03839781	-8.8	418.497	-2.1029	9	3	285.11	0.30865	123.03	8.5281	274.8265485281	0.31037	8.6638	-6.7754	0.58621	0.4477	0.88151	1	3600	0.7609314	-0.5244186		1.0
	ZINC03840093	-8.8	431.511	-1.3428	9	6	321.16	0.3464	158.36	4.8135	274.8265485281	0.29035	7.9037	-4.6248	0.54839	0.49645	0.89174 (max o	6	2760; 2759; 2007; 20	-0.5030939	-0.1674431		1.0
	ZINC04029261	-8.8	406.410	-1.225	9	1	281.87	0.36123	115.38	0.66478	274.8265485281	0.31037	7.7859	-3.9469	0.44828	0.34824		0		-0.9830631	0.06850321		1.0
	ZINC04221767	-8.8	377.404	-1.6771	11	3	272.34	0.46457	166.3	3.2718	274.8265485281	0.33336	8.238	-5.0308	0.51852	0.40216	0.88683 (max o	2	81; 717	-0.7482969	0.47650677		1.0
	ZINC04228235	-8.8	443.419	-8.3663	13	6	324.07	0.50853	212.93	4.3321	274.8265485281	0.28128	14.927	-29.744	0.625	0.58386	0.97533 (max o	7	2956; 2955; 2555; 92	0.8414538	0.25711232		1.0
	ZINC04236320	-8.8	410.405	-2.1291	9	1	296.57	0.32033	119.08	2.0442	274.8265485281	0.30003	8.69	-7.0964	0.6	0.41443	0.92527 (max o	5	1139; 34; 55; 83; 197	-0.4822975	-0.5330819		1.0
	ZINC04245710	-8.8	388.296	-4.095	16	8	261.34	0.68991	221.54	4.1581	274.8265485281	0.33336	10.656	-12.284	0.55556	0.52283	0.92094 (max o	2	3227; 905	-0.3267883	0.78155005		1.0
	ZINC04245710	-8.8	387.288	-6.2646	16	7	262.63	0.69143	227.86	4.275	274.8265485281	0.33336	12.826	-18.792	0.55556	0.52283	0.92094 (max o	3	3228; 3227; 904	-0.3442461	0.79289746		1.0
	ZINC04259185	-8.8	417.552	-3.4769	8	3	341.03	0.27229	78.6	6.1656	274.8265485281	0.30003	10.038	-11.589	0.6	0.44626	0.89979	1	4544	-0.5450855	0.8324417		1.0
	ZINC04259816	-8.8	388.403	-0.1985	10	1	267.3	0.36483	106.36	0.71105	274.8265485281	0.32146	6.7594	-0.6175	0.57143	0.34306	0.91871 (max o	5	2764; 2387; 2060; 34	0.00298650	-0.3174545		1.0
	ZINC04259901	-8.8	420.404	-1.0221	10	1	303.27	0.36799	131.29	1.9567	274.8265485281	0.29035	7.583	-3.5202	0.51613	0.34172	0.86822 (max o	3	2438; 1842; 1415	-0.2984516	-0.3040836		1.0
	ZINC04260889	-8.8	408.501	-1.9274	9	2	306.68	0.34707	108.16	-7.5269	274.8265485281	0.32146	8.4883	-5.9958	0.60714	0.41161	0.86564 (max o	3	4123; 1150; 56	-0.1703230	-0.0632695		1.0
	ZINC04266670	-8.8	428.534	2.1363	6	4	282.98	0.17316	86.14	-1.8087	274.8265485281	0.28128	4.4246	7.595	0.4375	0.17217	0.92889	1	16	0.15435758	-0.8330872		1.0
	ZINC04266670	-8.8	429.542	-0.108	6	4	284.58	0.15911	90.72	-2.288	274.8265485281	0.28128	6.6689	-0.38397	0.4375	0.31595	0.92348	1	17	-0.7869295	-0.5930624		1.0
	ZINC04270561	-8.8	385.379	0.5545	10	1	265.01	0.39349	109.62	3.3546	274.8265485281	0.32146	6.0064	1.725	0.53571	0.26478	0.90883 (max o	6	1737; 1726; 1420; 45	-0.1953366	-0.3842231		1.0
	ZINC04270855	-8.8	350.417	0.7841	8	0	254.07	0.24922	73.4	-0.44806	274.8265485281	0.36003	5.7768	2.1779	0.56	0.50944		0		-0.7269726	0.6629452		1.0

Structure	Molecule Name	affinity	Total Molweight	cLogP	H-Acceptors	H-Donors	Total Surface Area	Relative PSA	Polar Surface Area	Druglikeness	Kd (nM) @ 20°C	LE from K...	LLE from Kd ...	LELP from Kd (n...	Shape Index	Molecular...	Neighbor Si...	Neig...	Neighbor	Neighbor ...	Neighbor ...	Ring Systems	frequency
	ZINC04277716	-8.8	416.544	-0.5836	8	3	329.65	0.2631	86.19	1.6231	274.8265485281	0.30003	7.1445	-1.9452	0.6	0.46731	0.88025	1	6149	-0.8252751	-0.2869465		1.0
	ZINC04356403	-8.8	443.519	-1.0526	10	2	336.92	0.32984	131.65	-5.2455	274.8265485281	0.30003	7.6135	-3.5083	0.53333	0.47767		0		0.6572513	-0.6163323		1.0
	ZINC05396602	-8.8	428.444	-1.7031	9	1	303.85	0.37966	147.32	6.0761	274.8265485281	0.30003	8.264	-5.6765	0.56667	0.4162	0.93405 (max o	3	2802; 1454; 719	-0.2259807	0.38317537		1.0
	ZINC05399094	-8.8	424.479	-1.241	9	2	333.04	0.31531	95.85	-1.2378	274.8265485281	0.29035	7.8019	-4.2742	0.6129	0.43206	0.90776 (max o	6	4123; 3688; 3253; 20	-0.2035305	-0.0904897		1.0
	ZINC05413955	-8.8	436.471	-0.3739	10	2	325.62	0.31976	124.6	5.0549	274.8265485281	0.28128	6.9348	-1.3293	0.4375	0.49923	0.97743 (max o	6	2929; 2572; 2466; 21	-0.7048468	-0.4553266		1.0
	ZINC05414432	-8.8	436.471	-0.3199	10	2	325.62	0.31976	124.6	5.0549	274.8265485281	0.28128	6.8808	-1.1373	0.4375	0.46026	0.99646 (max o	6	4133; 2929; 2572; 24	-0.6922854	-0.4250747		1.0
	ZINC05433849	-8.8	428.444	0.4844	10	1	309.11	0.32348	104.27	3.0302	274.8265485281	0.29035	6.0765	1.6683	0.58065	0.36163	0.7988	1	2468	0.6816076	0.671361		1.0
	ZINC05807412	-8.8	413.433	0.3389	10	4	291.34	0.36624	132.44	3.7769	274.8265485281	0.30003	6.222	1.1296	0.46667	0.36185	0.9707 (max of	4	4693; 1775; 1469; 10	0.3174648	-0.4969979		1.0
	ZINC08295947	-8.8	410.454	-2.2018	11	2	288.95	0.41412	150.05	6.3333	274.8265485281	0.32146	8.7627	-6.8494	0.5	0.47013	0.96519 (max o	5	3257; 3256; 2867; 14	0.06445923	0.3775744		1.0
	ZINC08624306	-8.8	386.403	0.4347	8	3	280.71	0.3256	112.24	3.1992	274.8265485281	0.32146	6.1262	1.3523	0.57143	0.44507	0.89424 (max o	4	4606; 4601; 2877; 21	0.5313707	-0.3587701		1.0
	ZINC08764905	-8.8	432.544	-1.6844	7	0	312.38	0.35066	141.38	0.15957	274.8265485281	0.31037	8.2453	-5.427	0.58621	0.49209		0		0.43035153	0.8639084		1.0
	ZINC08790611	-8.8	445.447	-1.5922	10	1	331.31	0.33639	134.3	5.8844	274.8265485281	0.28128	8.1531	-5.6606	0.59375	0.58897	0.93965 (max o	2	2122; 548	0.3405285	0.20369503		1.0
	ZINC08879591	-8.8	372.400	-0.9674	8	2	278.5	0.33361	118.64	-0.010586	274.8265485281	0.33336	7.5283	-2.9019	0.51852	0.59934	0.95461	1	2165	0.7243469	-0.6292878		1.0
	ZINC12604698	-8.8	386.395	-0.0346	11	0	271.31	0.4834	148.15	0.6708	274.8265485281	0.33336	6.5955	-0.10379	0.59259	0.30382	0.86137	1	5394	-0.5160752	0.5239681		1.0
	ZINC12605290	-8.8	445.478	-1.4146	12	2	327.7	0.35908	137.07	4.265	274.8265485281	0.28128	7.9755	-5.0292	0.4375	0.53961	0.97743 (max o	6	2086; 1748; 1436; 11	-0.0579042	0.24846925		1.0
	ZINC12901556	-8.8	370.384	-1.4229	8	0	269.69	0.30409	102.34	4.703	274.8265485281	0.33336	7.9838	-4.2683	0.59259	0.47034	0.86639	1	5494	0.13059011	0.5106046		1.0
	ZINC13349047	-8.8	416.405	-1.5687	9	3	306.73	0.35217	137.38	-12.421	274.8265485281	0.30003	8.1296	-5.2285	0.53333	0.43785	0.96343 (max o	3	7005; 5870; 1503	0.85894954	0.18058953		1.0
	ZINC13409456	-8.8	361.320	-0.9017	10	1	262.2	0.43127	141.32	3.5845	274.8265485281	0.33336	7.4626	-2.7049	0.51852	0.19121	0.93058 (max o	2	3792; 2547	-0.3845575	-0.8252633		1.0
	ZINC15673166	-8.8	401.438	-0.2093	8	0	296.74	0.27128	99.21	3.3033	274.8265485281	0.31037	6.7702	-0.67435	0.58621	0.51766	0.82314	1	4261	-0.7511704	-0.3169123		1.0
	ZINC15675320	-8.8	402.445	-2.4903	8	2	302.27	0.29629	113.53	-0.85994	274.8265485281	0.31037	9.0512	-8.0236	0.62069	0.44389		0		0.7250716	0.68844694		1.0
	ZINC18152146	-8.8	444.439	-4.6124	10	6	306.5	0.4524	185.65	2.151	274.8265485281	0.28128	11.173	-16.398	0.375	0.34344	0.94718 (max o	3	113; 167; 388	-0.466768	-0.5494638		1.0
	ZINC18152146	-8.8	443.431	-6.7785	10	5	307.68	0.4545	188.48	3.0017	274.8265485281	0.28128	13.339	-24.099	0.375	0.36615	0.9497 (max of	6	992; 76; 116; 123; 31	0.07480918	0.61187917		1.0
	ZINC18152146	-8.8	443.431	-6.7785	10	5	307.68	0.4545	188.48	3.0758	274.8265485281	0.28128	13.339	-24.099	0.375	0.36615	0.94718 (max o	5	23; 44; 116; 392; 990	0.07973838	0.5707067		1.0
	ZINC18710083	-8.8	394.453	-1.7705	7	5	285.25	0.25371	88.8	2.0049	274.8265485281	0.31037	8.3314	-5.7044	0.55172	0.30388	0.89093	1	994	-0.8176748	0.39840865		1.0
	ZINC18710083	-8.8	393.445	0.3392	7	4	271.21	0.21507	87.6	6.315	274.8265485281	0.31037	6.2217	1.0929	0.55172	0.30388	0.89093	1	993	-0.8259104	0.41716328		1.0
	ZINC19701771	-8.8	425.396	-6.4619	10	0	277.81	0.39639	150.91	-0.14559	274.8265485281	0.29035	13.023	-22.256	0.35484	0.33934	0.81318	1	997	-0.0688219	-0.9527368		1.0
	ZINC19701771	-8.8	427.412	-4.1017	10	2	290.56	0.42287	145.79	-6.4405	274.8265485281	0.29035	10.663	-14.127	0.35484	0.33934	0.83865 (max o	2	1338; 997	-0.1113507	-0.9426357		1.0
	ZINC19701771	-8.8	426.404	-4.2333	10	1	276.52	0.39357	144.59	-3.8417	274.8265485281	0.29035	10.794	-14.58	0.35484	0.33934	0.89103 (max o	3	1338; 995; 996	-0.0902593	-0.9479876		1.0
	ZINC20412184	-8.8	418.473	0.1658	9	2	303.52	0.37592	124.46	6.7296	274.8265485281	0.31037	6.3951	0.5342	0.48276	0.41168	0.97649 (max o	3	2343; 637; 801	-0.2154481	0.6574015		1.0
	ZINC20504021	-8.8	434.559	0.1075	9	1	335.25	0.23729	95.73	7.3634	274.8265485281	0.29035	6.4534	0.37024	0.6129	0.60271	0.85831 (max o	2	1545; 1514	-0.0493052	0.8445756		1.0
	ZINC20758674	-8.8	396.402	-1.8021	9	4	285.33	0.3266	142.75	3.716	274.8265485281	0.31037	8.363	-5.8062	0.55172	0.47512	0.80776	1	2606	-0.3717035	-0.9052416		1.0
	ZINC22611954	-8.8	433.554	0.3067	7	3	331.89	0.17834	63.22	5.345	274.8265485281	0.28128	6.2542	1.0904	0.5	0.46563		0		0.7616746	0.52867746		1.0
	ZINC22922137	-8.8	411.544	-1.9793	6	3	318.57	0.19456	62.98	2.5103	274.8265485281	0.30003	8.5402	-6.5971	0.6	0.46447	0.92257 (max o	3	6846; 2612; 1015	0.5275593	-0.1605851		1.0
	ZINC22936910	-8.8	410.540	-0.4388	7	3	305.36	0.21994	82.98	5.6665	274.8265485281	0.30003	6.9997	-1.4625	0.56667	0.4025	0.93405 (max o	2	1564; 173	0.33237195	-0.8142059		1.0
	ZINC23508463	-8.8	445.435	-2.9012	11	3	311.69	0.54099	215.68	0.89821	274.8265485281	0.30003	9.4621	-9.6698	0.53333	0.46614		0		-0.3603659	0.8912422		1.0
	ZINC31154680	-8.8	411.445	-1.2648	11	1	293.52	0.36226	118.61	1.5169	274.8265485281	0.30003	7.8257	-4.2156	0.6	0.37862	0.96832 (max o	6	2808; 2764; 1213; 11	-0.0559359	-0.3248394		1.0
	ZINC31544938	-8.8	404.370	0.835	9	6	278.17	0.40518	152.98	-4.1025	274.8265485281	0.31037	5.7259	2.6903	0.51724	0.36953		0		0.05416710	0.8982837		1.0
	ZINC40312487	-8.8	369.380	-0.9387	9	2	274.8	0.36492	127.39	6.6472	274.8265485281	0.33336	7.4996	-2.8158	0.55556	0.47629	0.84311	1	4849	-0.1000157	-0.8226658		1.0
	ZINC67912703	-8.8	366.408	-0.5383	7	4	237.47	0.37259	124.29	0.78517	274.8265485281	0.34619	7.0992	-1.5549	0.34615	0.32626	0.92702 (max o	4	3542; 2316; 2317; 16	0.33823326	0.62061375		1.0
	ZINC68604906	-8.8	448.521	1.2173	8	4	334.75	0.17975	117.46	-3.3795	274.8265485281	0.27275	5.3436	4.463	0.51515	0.53338		0		-0.9044776	0.30678076		1.0
	ZINC71405130	-8.8	421.523	-1.459	8	2	338.12	0.26272	78.53	-2.4426	274.8265485281	0.29035	8.0199	-5.025	0.64516	0.47456	0.87695 (max o	3	3253; 2080; 929	-0.1876117	-0.0745199		1.0
	ZINC04062874	-8.7	389.359	-3.7496	9	0	290.86	0.35849	136.1	-6.9789	326.2985304218	0.31781	10.236	-11.798	0.53571	0.58906	0.96023 (max o	4	2718; 1973; 1357; 42	0.14338933	0.43653214		172.0
	ZINC02153171	-8.7	364.397	-1.2775	7	2	268.85	0.39223	141.06	1.7041	326.2985304218	0.35594	7.7639	-3.5891	0.56	0.56527	0.86285	1	3136	-0.7370389	0.40035093		172.0

Structure	Molecule Name	affinity	Total Molweight	cLogP	H-Acceptors	H-Donors	Total Surface Area	Relative PSA	Polar Surface Area	Druglikeness	Kd (nM) @ 20°C	LE from K...	LLE from Kd ...	LELP from Kd (n...	Shape Index	Molecular...	Neighbor Si...	Neig...	Neighbor	Neighbor ...	Neighbor ...	Ring Systems	frequency
	ZINC02113856	-8.7	381.747	-2.4544	9	3	269.44	0.41738	144.86	1.2625	326.2985304218	0.34225	8.9408	-7.1713	0.61538	0.58026	0.96106 (max o	4	4290; 3568; 3565; 16	0.10255667	0.05611709		172.0
	ZINC02125950	-8.7	404.398	-1.9604	10	4	302.56	0.42216	170.88	0.85156	326.2985304218	0.30685	8.4468	-6.3889	0.62069	0.56401	0.98795 (max o	7	5299; 4010; 3584; 31	0.02529041	0.20183119		172.0
	ZINC04221585	-8.7	395.432	-4.1482	11	6	272.29	0.53322	204.5	1.5562	326.2985304218	0.34225	10.635	-12.12	0.5	0.52319	0.92094	1	5770	0.79095966	0.4749143		127.0
	ZINC13436567	-8.7	425.186	-10.071	15	4	265.24	0.70849	253.33	-44.039	326.2985304218	0.32958	16.558	-30.558	0.51852	0.52391	0.94905 (max o	10	2487; 1584; 1506; 14	-0.2813305	-0.4744305		44.0
	ZINC13436567	-8.7	424.178	-11.319	15	3	266.42	0.70978	256.16	-39.309	326.2985304218	0.32958	17.805	-34.344	0.51852	0.52373	0.94905 (max o	10	2488; 1585; 1507; 14	-0.3174618	-0.4491121		44.0
	ZINC12889911	-8.7	404.402	-3.5751	11	4	298.78	0.44407	173.76	7.7467	326.2985304218	0.30685	10.061	-11.651	0.48276	0.55428	0.96385 (max o	10	3823; 3801; 3016; 29	0.01734892	-0.5859787		42.0
	ZINC20758433	-8.7	380.379	-0.9369	8	2	282.29	0.32913	118.64	4.6877	326.2985304218	0.31781	7.4233	-2.948	0.42857	0.49782	0.96519 (max o	11	3859; 3748; 3346; 30	0.6621644	-0.0825100		36.0
	ZINC04096303	-8.7	400.382	0.3293	8	4	280.14	0.34069	125.68	-3.6206	326.2985304218	0.30685	6.1571	1.0732	0.55172	0.38809	0.94529 (max o	7	1182; 258; 356; 459;	0.23634024	0.5427294		30.0
	ZINC13424748	-8.7	416.381	-0.0164	9	5	286.49	0.37886	145.91	-3.6206	326.2985304218	0.29662	6.5028	-0.055289	0.56667	0.39146	0.95837 (max o	10	366; 581; 610; 611; 8	0.19194517	0.5756884		30.0
	ZINC04349768	-8.7	416.381	-0.0164	9	5	286.49	0.37886	145.91	-3.6206	326.2985304218	0.29662	6.5028	-0.055289	0.56667	0.39146	0.95837 (max o	9	1426; 1230; 581; 610	0.20877278	0.56474125		30.0
	ZINC11666970	-8.7	430.408	0.2593	9	4	302.4	0.34868	134.91	-3.4398	326.2985304218	0.28705	6.2271	0.90333	0.58065	0.40392	0.96018 (max o	10	2135; 1426; 1230; 36	0.24540003	0.59673434		30.0
	ZINC04349791	-8.7	446.407	-0.0864	10	5	308.75	0.38394	155.14	-3.4398	326.2985304218	0.27808	6.5728	-0.3107	0.53125	0.30824	0.95949 (max o	7	7349; 1425; 1206; 65	0.15441643	0.5597654		30.0
	ZINC12495616	-8.7	446.407	-0.0864	10	5	308.75	0.38394	155.14	-3.4398	326.2985304218	0.27808	6.5728	-0.3107	0.53125	0.30824	0.95949 (max o	7	7349; 1425; 1230; 65	0.13940923	0.5927237		30.0
	ZINC04000431	-8.7	376.388	-1.9591	9	2	287.06	0.35947	121.72	4.4099	326.2985304218	0.32958	8.4455	-5.9443	0.55556	0.37381	0.93985 (max o	6	4501; 4500; 4425; 31	-0.2228128	0.55778646		28.0
	ZINC03947476	-8.7	353.302	-2.8445	9	5	246.47	0.47008	167.58	0.33063	326.2985304218	0.35594	9.3309	-7.9915	0.56	0.41449	0.97396 (max o	14	7407; 3467; 3051; 26	-0.2253354	-0.8486048		25.0
	ZINC02124599	-8.7	373.316	-4.4063	9	1	268	0.41858	144.89	0.9497	326.2985304218	0.32958	10.893	-13.37	0.55556	0.48449	0.96023 (max o	8	2401; 1981; 1979; 19	0.20862678	0.07722133		24.0
	ZINC02140391	-8.7	401.394	-2.0808	9	2	296.45	0.3689	133.86	-1.0092	326.2985304218	0.30685	8.5672	-6.7812	0.55172	0.43667	0.96931 (max o	5	3573; 1356; 1355; 55	0.22156239	0.02779958		24.0
	ZINC02114996	-8.7	387.367	-2.4401	9	2	285.45	0.38311	133.86	-2.0422	326.2985304218	0.31781	8.9265	-7.678	0.57143	0.43424	0.9218 (max of	7	4002; 1986; 1970; 16	0.17617007	0.04849253		24.0
	ZINC02158497	-8.7	429.448	-1.4081	9	2	321.21	0.34046	133.86	1.6567	326.2985304218	0.28705	7.8945	-4.9054	0.54839	0.463	0.92566 (max o	6	2727; 2723; 1986; 19	0.16580418	0.00549451		24.0
	ZINC02119623	-8.7	416.409	-2.1384	10	3	307.97	0.40468	159.88	0.76173	326.2985304218	0.29662	8.6248	-7.2093	0.6	0.50221	0.9707 (max of	6	4006; 3580; 1979; 13	0.2510452	0.08326986		24.0
	ZINC12882728	-8.7	426.400	-0.8918	9	2	316.1	0.34154	134.22	2.5282	326.2985304218	0.28705	7.3782	-3.1068	0.6129	0.43371	0.99016 (max o	14	3343; 3204; 2934; 27	0.5137143	0.41937503		24.0
	ZINC04081166	-8.7	366.365	0.0247	8	4	255.37	0.37373	125.68	-5.4909	326.2985304218	0.34225	6.4617	0.072169	0.5	0.28536	0.9464 (max of	7	6710; 2783; 2407; 14	0.3658138	0.5107844		24.0
	ZINC01532585	-8.7	376.368	-2.0663	10	5	264.4	0.44066	155.05	6.1929	326.2985304218	0.32958	8.5527	-6.2696	0.44444	0.47074	0.97288 (max o	17	5314; 5297; 4912; 49	0.4353737	0.77617604		24.0
	ZINC35644172	-8.7	444.439	-4.6138	10	6	307.33	0.45118	185.65	2.419	326.2985304218	0.27808	11.1	-16.592	0.375	0.36573	0.96186 (max o	5	2184; 1846; 8; 65; 40	-0.7604819	-0.5290958		22.0
	ZINC35644172	-8.7	443.431	-3.2475	10	5	293.29	0.4249	184.45	4.6853	326.2985304218	0.27808	9.7339	-11.678	0.375	0.36418	0.95949 (max o	6	2186; 1850; 1849; 9;	-0.0958768	0.7056801		22.0
	ZINC12881102	-8.7	446.478	-1.5067	10	2	333.59	0.33847	137.1	-0.0023535	326.2985304218	0.27808	7.9931	-5.4182	0.5625	0.47785	0.97533 (max o	12	4194; 4193; 3781; 26	0.5411212	0.1923685		22.0
	ZINC12882608	-8.7	446.478	-1.5067	10	2	333.59	0.33847	137.1	-0.24237	326.2985304218	0.27808	7.9931	-5.4182	0.53125	0.46794	0.97948 (max o	12	6226; 5250; 4194; 39	0.49926633	0.2045946		22.0
	ZINC04102435	-8.7	448.379	-0.0011999	11	7	297.4	0.45306	186.37	-3.6679	326.2985304218	0.27808	6.4876	-0.004315	0.40625	0.43252	0.95949 (max o	10	4260; 3174; 2768; 24	-0.7408263	0.36327362		21.0
	ZINC08636399	-8.7	405.474	0.0283	8	4	298.44	0.37247	137.71	4.9433	326.2985304218	0.31781	6.4581	0.089048	0.53571	0.42767	0.9398 (max of	4	3734; 2884; 763; 944	-0.5278241	0.35282373		17.0
	ZINC05667879	-8.7	400.382	0.3293	8	4	280.14	0.34069	125.68	-3.6206	326.2985304218	0.30685	6.1571	1.0732	0.55172	0.39121	0.95642 (max o	6	356; 366; 459; 595; 7	0.25542462	0.57952064		16.0
	ZINC13424729	-8.7	432.380	-0.078	10	7	289.19	0.43134	177.14	-1.9977	326.2985304218	0.28705	6.5644	-0.27173	0.51613	0.3811	0.96262 (max o	12	3788; 3213; 2550; 24	0.7167265	0.1316022		14.0
	ZINC20610898	-8.7	419.416	-0.8975	9	3	306.46	0.34882	134.43	4.8339	326.2985304218	0.28705	7.3839	-3.1266	0.41935	0.45817	0.96971 (max o	11	2534; 1877; 1654; 15	0.7434622	-0.1519465		14.0
	ZINC20610821	-8.7	405.389	-1.2568	9	3	295.46	0.36181	134.43	3.1167	326.2985304218	0.29662	7.7432	-4.2371	0.43333	0.46359	0.95837 (max o	7	2534; 1877; 1654; 13	0.7375645	-0.1307455		14.0
	ZINC20758378	-8.7	449.442	-1.4649	10	4	325.31	0.36888	154.66	4.2777	326.2985304218	0.26965	7.9513	-5.4325	0.39394	0.46394	0.99099 (max o	10	2540; 2534; 2161; 16	0.72715086	-0.1866649		14.0
	ZINC05414476	-8.7	397.434	0.0197	9	2	302.31	0.31669	122.61	1.0039	326.2985304218	0.30685	6.4667	0.064202	0.55172	0.52618	0.95345 (max o	6	3257; 2470; 2088; 17	0.09670455	0.33152202		14.0
	ZINC08299547	-8.7	413.457	-4.4756	10	3	311.54	0.35379	150.25	-0.65407	326.2985304218	0.29662	10.962	-15.089	0.53333	0.52246	0.95345 (max o	5	3722; 3257; 2470; 76	0.11830871	0.33948		14.0
	ZINC02117077	-8.7	429.448	-1.41	9	2	322.71	0.33888	133.86	-0.67327	326.2985304218	0.28705	7.8964	-4.912	0.58065	0.54526	0.9112 (max of	5	3583; 3571; 1983; 21	0.15532799	0.01997315		14.0
	ZINC05437829	-8.7	422.460	-0.7907	8	1	312.59	0.27192	109.85	2.8177	326.2985304218	0.28705	7.2771	-2.7546	0.54839	0.4959	0.92547 (max o	5	5778; 5393; 4972; 32	-0.4613678	-0.3075972		14.0
	ZINC03838458	-8.7	404.422	-1.2513	10	6	299.35	0.42763	170.85	1.7222	326.2985304218	0.30685	7.7377	-4.0779	0.58621	0.53859	0.93091 (max o	4	2745; 2005; 1705; 16	-0.1988819	-0.5422252		12.0
	ZINC08627125	-8.7	425.455	-0.8512	9	3	311.45	0.29167	111.57	4.4031	326.2985304218	0.29662	7.3376	-2.8697	0.66667	0.56345	0.94529 (max o	6	2109; 1764; 1463; 58;	-0.0419814	-0.5835551		11.0
	ZINC02107059	-8.7	358.369	-0.3718	7	1	263.93	0.3033	106.97	2.2299	326.2985304218	0.34225	6.8582	-1.0863	0.53846	0.542	0.95257 (max o	4	1642; 363; 474; 546	0.37806383	0.13374981		11.0
	ZINC02133556	-8.7	399.354	-3.228	9	0	292.89	0.37004	140.01	-7.1804	326.2985304218	0.30685	9.7144	-10.52	0.48276	0.5391	0.96211 (max o	7	2718; 1369; 332; 425;	0.19088581	0.45029578		11.0
	ZINC02105518	-8.7	413.381	-2.8301	9	0	305.15	0.35517	140.01	-11.258	326.2985304218	0.29662	9.3165	-9.5412	0.5	0.5329	0.96385 (max o	6	2718; 1369; 1090; 42	0.18609923	0.4877456		11.0

Structure	Molecule Name	affinity	Total Molweight	cLogP	H-Acceptors	H-Donors	Total Surface Area	Relative PSA	Polar Surface Area	Druglikeness	Kd (nM) @ 20°C	LE from K...	LLE from Kd ...	LELP from Kd (n...	Shape Index	Molecular...	Neighbor Si...	Neig...	Neighbor	Neighbor ...	Neighbor ...	Ring Systems	frequency
	ZINC04237068	-8.7	411.433	-1.5657	8	0	303.38	0.26534	99.21	3.5854	326.2985304218	0.29662	8.0521	-5.2785	0.63333	0.46768	0.97304 (max o	5	5392; 2436; 2057; 17	-0.5319273	-0.3206045		10.0
	ZINC03840205	-8.7	408.453	-0.948	10	7	303.93	0.42138	174.01	-0.54656	326.2985304218	0.30685	7.4344	-3.0895	0.62069	0.52173	0.97941 (max o	6	1861; 1373; 190; 885	-0.4364549	-0.3589437		10.0
	ZINC03840168	-8.7	394.426	-1.4975	10	7	287.41	0.4456	174.01	1.9172	326.2985304218	0.31781	7.9839	-4.712	0.57143	0.49726	0.9308 (max of	8	1861; 1673; 1373; 19	-0.4373461	-0.4012635		10.0
	ZINC03840166	-8.7	403.793	-1.4878	9	6	274.46	0.42487	161.98	1.5346	326.2985304218	0.32958	7.9742	-4.5143	0.55556	0.48971	0.96567 (max o	7	2373; 2010; 1664; 16	-0.3806897	-0.5074782		10.0
	ZINC04963988	-8.7	448.379	-0.4237	11	8	295.54	0.4664	197.37	-1.9977	326.2985304218	0.27808	6.9101	-1.5237	0.4375	0.38342	0.95465 (max o	8	4107; 3891; 2820; 26	0.7656633	0.02630174		10.0
	ZINC12883664	-8.7	363.413	-1.4073	7	2	249.83	0.39627	135.07	5.1687	326.2985304218	0.35594	7.8937	-3.9537	0.44	0.26718	0.98671 (max o	9	6409; 4523; 4524; 40	0.30708992	-0.6240254		10.0
	ZINC22922313	-8.7	415.556	0.1888	7	2	319.18	0.19544	78.49	4.2144	326.2985304218	0.29662	6.2976	0.63651	0.63333	0.48446	0.95031 (max o	11	3876; 1862; 1598; 15	-0.5428958	0.10484781		9.0
	ZINC04236077	-8.7	411.456	-0.2538	7	2	314.08	0.23965	74.16	4.5306	326.2985304218	0.29662	6.7402	-0.85565	0.53333	0.38107	0.93111 (max o	2	2056; 573	-0.2821876	0.28252158		9.0
	ZINC04235978	-8.7	425.440	0.6515	9	2	309.74	0.29308	108.41	6.4888	326.2985304218	0.28705	5.8349	2.2696	0.54839	0.39306	0.93686 (max o	3	4099; 25; 443	-0.2557662	0.3112919		9.0
	ZINC05273635	-8.7	416.381	-0.4408	9	6	282.84	0.39471	156.91	-2.3989	326.2985304218	0.29662	6.9272	-1.4861	0.5	0.39702	0.95579 (max o	8	2463; 1427; 541; 588	0.6975742	0.18653598		9.0
	ZINC15259619	-8.7	432.380	-0.7865	10	7	289.19	0.43134	177.14	-2.3989	326.2985304218	0.28705	7.2729	-2.7399	0.48387	0.39186	0.95771 (max o	7	5883; 2481; 2095; 16	0.67878586	0.19795789		9.0
	ZINC35442321	-8.7	433.546	-0.1565	7	2	324.27	0.19237	78.49	4.6683	326.2985304218	0.28705	6.6429	-0.5452	0.6129	0.46596	0.94768 (max o	8	2279; 1862; 1599; 51	-0.5632632	0.15543462		9.0
	ZINC35442320	-8.7	450.001	0.3487	7	2	333.34	0.18714	78.49	6.0576	326.2985304218	0.28705	6.1377	1.2148	0.58065	0.46596	0.94501 (max o	7	2279; 1862; 1599; 40	-0.5989223	0.13932836		9.0
	ZINC36374481	-8.7	417.389	-1.405	9	5	299.31	0.37693	159.74	-3.819	326.2985304218	0.29662	7.8914	-4.7367	0.5	0.40276	0.95837 (max o	3	525; 526; 676	0.3207994	-0.7983826		9.0
	ZINC04098629	-8.7	420.413	0.7702	9	6	302.64	0.35884	149.07	-4.1548	326.2985304218	0.29662	5.7162	2.5966	0.53333	0.42608	0.98188 (max o	8	3902; 3901; 2634; 20	-0.5393948	0.73949915		9.0
	ZINC04257737	-8.7	420.413	0.222	9	5	301.3	0.36024	145.91	-3.6574	326.2985304218	0.29662	6.2644	0.74844	0.6	0.47551	0.97304 (max o	7	2909; 2517; 2358; 20	0.40409815	0.5753483		9.0
	ZINC05440521	-8.7	429.427	-1.283	9	2	314.64	0.33441	125.5	-4.6031	326.2985304218	0.28705	7.7694	-4.4696	0.6129	0.39078	0.98126 (max o	8	3255; 2842; 2467; 20	-0.7079411	-0.4911905		9.0
	ZINC12657962	-8.7	376.044	-1.8403	5	1	160.53	0.35514	80.67	-2.4417	326.2985304218	0.55616	8.3267	-3.3089	0.4375	0.2085	0.86186 (max o	2	7904; 7701	-0.4410996	-0.8297102		9.0
	ZINC13136669	-8.7	432.380	-0.078	10	7	289.19	0.43134	177.14	-1.9977	326.2985304218	0.28705	6.5644	-0.27173	0.51613	0.3816	0.96018 (max o	11	3788; 3213; 2453; 18	0.6989496	0.12104613		8.0
	ZINC05397348	-8.7	372.400	-0.5303	8	1	271.82	0.31271	109.85	4.3881	326.2985304218	0.32958	7.0167	-1.609	0.51852	0.55155	0.94291 (max o	4	4553; 3664; 3231; 14	0.10028552	-0.565253		8.0
	ZINC72332945	-8.7	427.519	-2.0628	7	6	311.03	0.27856	108.83	2.5847	326.2985304218	0.28705	8.5492	-7.1862	0.41935	0.47997	0.97423 (max o	5	5902; 5119; 3981; 38	0.85634285	0.10246304		8.0
	ZINC02146791	-8.7	366.324	-2.5996	7	0	269.27	0.32562	115.79	1.0433	326.2985304218	0.32958	9.086	-7.8877	0.55556	0.45155	0.95201 (max o	4	1971; 428; 542; 543	-0.3482984	-0.1190483		7.0
	ZINC12296599	-8.7	425.372	-1.8215	10	3	313.31	0.39086	154.09	1.0187	326.2985304218	0.28705	8.3079	-6.3456	0.6129	0.49579	0.9617	1	609	0.8540486	-0.1609519		7.0
	ZINC01737596	-8.7	423.376	-3.1161	9	1	312.77	0.35867	144.89	2.6789	326.2985304218	0.28705	9.6025	-10.856	0.54839	0.49066	0.97423 (max o	3	3131; 1968; 1967	-0.0096061	-0.9358319		7.0
	ZINC31164855	-8.7	424.441	-2.0641	11	7	295.8	0.44523	178.53	0.47815	326.2985304218	0.30685	8.5505	-6.7268	0.55172	0.52826	0.8886 (max of	6	5695; 5287; 3910; 34	-0.6328764	0.49942967		7.0
	ZINC35644172	-8.7	444.439	-4.7179	10	5	308.31	0.44955	182.49	2.6033	326.2985304218	0.27808	11.204	-16.966	0.375	0.37412	0.95709 (max o	2	401; 1046	0.55389434	-0.7561056		6.0
	ZINC28577880	-8.7	432.380	0.3464	10	6	292.84	0.41538	166.14	-3.2535	326.2985304218	0.28705	6.14	1.2068	0.51613	0.40931	0.95521 (max o	2	133; 740	0.08773323	0.6440936		6.0
	ZINC04349516	-8.7	448.379	6.9979e-4	11	7	299.19	0.45035	186.37	-3.2535	326.2985304218	0.27808	6.4857	0.0025165	0.5	0.33722	0.95949 (max o	5	148; 350; 616; 739; 7	0.11994330	0.67080516		6.0
	ZINC04098528	-8.7	436.368	-0.1542	11	7	286.74	0.4699	186.37	-2.8589	326.2985304218	0.28705	6.6406	-0.53719	0.45161	0.35666	0.91413	1	1698	-0.1063376	-0.7306245		6.0
	ZINC13376359	-8.7	402.394	-2.0099	10	6	278.14	0.4264	158.3	-4.9799	326.2985304218	0.31781	8.4963	-6.3243	0.57143	0.50233	0.96983 (max o	8	1583; 1331; 1227; 53	-0.4804638	0.6543916		6.0
	ZINC13376361	-8.7	402.394	-2.0099	10	6	278.14	0.4264	158.3	-4.9799	326.2985304218	0.31781	8.4963	-6.3243	0.57143	0.50233	0.96983 (max o	9	4829; 1331; 530; 680	-0.4966714	0.6659353		6.0
	ZINC35271227	-8.7	434.396	-1.4258	10	8	285	0.44856	188.14	-2.6646	326.2985304218	0.28705	7.9122	-4.9671	0.35484	0.33408	0.98656 (max o	7	6314; 5513; 5512; 46	-0.5114535	0.58433574		6.0
	ZINC01643830	-8.7	424.376	-6.0843	13	4	310.95	0.5307	224.99	3.8284	326.2985304218	0.28705	12.571	-21.196	0.6129	0.58473	0.96694 (max o	5	2350; 2206; 1993; 16	-0.5669068	0.69693506		6.0
	ZINC04073877	-8.7	364.760	-0.5622	7	1	251.83	0.31787	106.97	5.7213	326.2985304218	0.35594	7.0486	-1.5795	0.52	0.49366	0.94975 (max o	3	2036; 1687; 331	0.2613373	0.15771988		6.0
	ZINC04259882	-8.7	434.436	-0.8278	13	1	297.8	0.46551	156.55	3.8561	326.2985304218	0.29662	7.3142	-2.7908	0.56667	0.38534	0.91333 (max o	3	2061; 1414; 348	-0.1035682	-0.4052144		6.0
	ZINC12604637	-8.7	399.434	-1.2236	11	1	290.26	0.36633	118.61	0.86806	326.2985304218	0.30685	7.71	-3.9877	0.58621	0.3849	0.92124 (max o	4	2808; 1022; 1108; 11	-0.0573780	-0.3463529		6.0
	ZINC04259885	-8.7	427.487	-0.4558	11	1	317.78	0.3346	118.61	0.71079	326.2985304218	0.28705	6.9422	-1.5879	0.6129	0.40012	0.92566 (max o	7	3661; 3608; 2808; 12	-0.0739586	-0.3129497		6.0
	ZINC20465165	-8.7	400.424	-0.4139	8	4	287.75	0.27545	97.3	6.2847	326.2985304218	0.31781	6.9003	-1.3024	0.57143	0.5281	0.92442 (max o	4	4744; 2225; 1866; 12	-0.3141369	-0.0292609		6.0
	ZINC20465165	-8.7	401.432	-2.5236	8	5	301.79	0.30916	98.5	2.2732	326.2985304218	0.31781	9.01	-7.9407	0.57143	0.5281	0.91204 (max o	5	4744; 2593; 2225; 18	-0.3066351	-0.0501752		6.0
	ZINC20758694	-8.7	398.418	-1.4992	9	3	271.21	0.3831	130.83	4.3284	326.2985304218	0.30685	7.9856	-4.8858	0.41379	0.24947	0.98525 (max o	10	4270; 3418; 1880; 18	0.0789062	0.8488205		5.0
	ZINC13424723	-8.7	400.382	1.0378	8	4	280.14	0.34069	125.68	-3.2535	326.2985304218	0.30685	5.4486	3.3821	0.55172	0.37921	0.9617 (max of	7	293; 360; 451; 489; 5	0.30151278	0.5499432		5.0
	ZINC04089633	-8.7	416.476	-3.0696	9	3	308.3	0.33883	149.24	7.8554	326.2985304218	0.29662	9.556	-10.349	0.46667	0.47164	0.96545 (max o	4	2908; 2039; 1477; 60	0.49354967	-0.6860392		5.0
	ZINC22922324	-8.7	418.535	-0.695	7	2	312.07	0.17922	72.5	7.8702	326.2985304218	0.29662	7.1814	-2.3431	0.63333	0.47775	0.9707 (max of	10	2653; 1278; 504; 519	-0.5764001	0.09254264		5.0

Structure	Molecule Name	affinity	Total Molweight	cLogP	H-Acceptors	H-Donors	Total Surface Area	Relative PSA	Polar Surface Area	Druglikeness	Kd (nM) @ 20°C	LE from K...	LLE from Kd ...	LELP from Kd (n...	Shape Index	Molecular...	Neighbor Si...	Neig...	Neighbor	Neighbor ...	Neighbor ...	Ring Systems	frequency
	ZINC22922319	-8.7	400.545	-0.7958	7	2	305.72	0.18295	72.5	9.2102	326.2985304218	0.30685	7.2822	-2.5935	0.62069	0.47484	0.96931 (max o	7	3066; 1598; 1278; 12	-0.5985265	0.04514477		5.0
	ZINC22936954	-8.7	443.614	-0.8994	8	2	340.54	0.17466	75.74	9.422	326.2985304218	0.27808	7.3858	-3.2343	0.625	0.4985	0.97319 (max o	10	3438; 3028; 504; 810	-0.5782175	0.03672078		5.0
	ZINC04236410	-8.7	372.400	-1.146	8	1	273.08	0.31126	109.85	4.8305	326.2985304218	0.32958	7.6324	-3.4772	0.51852	0.55105	0.91204 (max o	2	1186; 1141	-0.0388946	-0.7984148		5.0
	ZINC08296911	-8.7	372.400	-1.3318	8	2	272.98	0.34035	118.64	4.9596	326.2985304218	0.32958	7.8182	-4.041	0.55556	0.47201	0.85214 (max o	2	2656; 1140	-0.0535265	-0.7670141		5.0
	ZINC09007942	-8.7	439.535	-1.8763	10	4	310.25	0.35304	178.63	-0.25575	326.2985304218	0.29662	8.3627	-6.3256	0.63333	0.54495	0.85787 (max o	3	4894; 4234; 3996	0.58377445	-0.2797612		5.0
	ZINC20465773	-8.7	428.551	-0.3095	7	4	347.89	0.23525	86.47	2.7757	326.2985304218	0.28705	6.7959	-1.0782	0.58065	0.56344	0.93414 (max o	6	7070; 3406; 3405; 30	0.38942873	-0.2691590		5.0
	ZINC04085262	-8.7	374.368	-1.1662	8	1	275.44	0.34439	113.99	-9.4513	326.2985304218	0.32958	7.6526	-3.5385	0.48148	0.37924	0.9617 (max of	2	209; 361	-0.0633014	0.17536613		4.0
	ZINC04277721	-8.7	353.398	-0.5859	8	2	241.05	0.3581	115.4	9.5581	326.2985304218	0.37077	7.0723	-1.5802	0.58333	0.49182	0.83714	1	917	-0.6434551	-0.7267151		4.0
	ZINC15968473	-8.7	446.482	-2.8941	8	2	319.48	0.29082	118.64	4.6249	326.2985304218	0.26965	9.3805	-10.733	0.42424	0.31005	0.96343 (max o	8	112; 229; 230; 298; 3	0.20887545	-0.5904195		4.0
	ZINC35270764	-8.7	376.360	-0.671	8	6	239.88	0.43638	155.52	0.76178	326.2985304218	0.32958	7.1574	-2.036	0.40741	0.31342	0.9578 (max of	3	407; 1042; 1043	-0.4526012	-0.7410018		4.0
	ZINC03839331	-8.7	428.487	-0.0794	9	2	312.86	0.29464	108.05	6.7539	326.2985304218	0.28705	6.5658	-0.27661	0.51613	0.4054	0.92863 (max o	2	1377; 1103	-0.4143644	-0.2731668		4.0
	ZINC04222677	-8.7	428.444	-1.7622	9	1	305.09	0.38218	148.29	6.5871	326.2985304218	0.29662	8.2486	-5.941	0.56667	0.46687	0.96832 (max o	6	3679; 2802; 2801; 44	-0.1691195	0.38963503		4.0
	ZINC04085422	-8.7	396.330	-0.5023	9	1	284.25	0.3689	123.22	0.42763	326.2985304218	0.30685	6.9887	-1.637	0.62069	0.3749	0.92257 (max o	6	2788; 2414; 1779; 16	0.52603376	0.3627858		4.0
	ZINC20610799	-8.7	396.422	-3.8725	8	2	273.98	0.33911	118.64	1.6685	326.2985304218	0.30685	10.359	-12.62	0.37931	0.22473	0.95642 (max o	5	1527; 380; 395; 501;	0.35733435	-0.5689443		4.0
	ZINC03871267	-8.7	409.112	-4.2064	11	3	220.98	0.56806	160.83	-13.945	326.2985304218	0.38689	10.693	-10.872	0.47826	0.34473	0.92193 (max o	3	6405; 4053; 1384	-0.5485389	-0.7407888		4.0
	ZINC08844377	-8.7	446.407	0.4497	10	4	312.4	0.36953	144.14	-3.4946	326.2985304218	0.27808	6.0367	1.6172	0.59375	0.41419	0.97533 (max o	4	1581; 1319; 604; 767	0.31720313	0.6176338		4.0
	ZINC36368158	-8.7	446.407	0.4497	10	4	312.4	0.36953	144.14	-3.4946	326.2985304218	0.27808	6.0367	1.6172	0.59375	0.41419	0.95465 (max o	4	1586; 1581; 767; 119	0.3528764	0.6035789		4.0
	ZINC13376360	-8.7	402.394	-2.0099	10	6	278.14	0.4264	158.3	-4.9799	326.2985304218	0.31781	8.4963	-6.3243	0.57143	0.49829	0.96983 (max o	8	1331; 530; 680; 841;	-0.4925608	0.6860222		4.0
	ZINC13376358	-8.7	402.394	-2.0099	10	6	278.14	0.4264	158.3	-4.9799	326.2985304218	0.31781	8.4963	-6.3243	0.57143	0.49829	0.96983 (max o	8	1331; 1227; 1226; 53	-0.4895485	0.706609		4.0
	ZINC36369942	-8.7	356.285	-2.8822	8	0	260.22	0.39117	128.93	1.8784	326.2985304218	0.34225	9.3686	-8.4213	0.61538	0.4782	0.95818 (max o	3	3516; 2667; 844	-0.4050795	-0.8857483		4.0
	ZINC04236169	-8.7	424.432	-1.7759	9	1	308.83	0.30761	119.08	2.2928	326.2985304218	0.28705	8.2623	-6.1867	0.6129	0.47699	0.89699 (max o	6	34; 55; 83; 196; 197;	-0.5149083	-0.5031446		4.0
	ZINC04270597	-8.7	388.446	0.4884	9	1	281.56	0.31084	97.13	-0.013374	326.2985304218	0.31781	5.998	1.5368	0.57143	0.32249	0.91482 (max o	5	2816; 2060; 266; 267	0.01460939	-0.2821424		4.0
	ZINC05434226	-8.7	375.428	-0.9066	9	2	282.06	0.31571	103.75	6.6741	326.2985304218	0.32958	7.393	-2.7508	0.48148	0.47875	0.94883 (max o	7	2863; 1744; 483; 592	-0.0536571	0.2864542		4.0
	ZINC15708214	-8.7	419.475	-1.607	8	3	321.26	0.29005	92.18	2.8518	326.2985304218	0.29662	8.0934	-5.4177	0.56667	0.46973	0.94251 (max o	5	4221; 2200; 1840; 15	-0.1606352	0.11102532		4.0
	ZINC49601711	-8.7	440.443	0.0769	10	6	314.91	0.38627	166.14	-6.4176	326.2985304218	0.28705	6.4095	0.2679	0.54839	0.405	0.96262 (max o	8	3531; 3530; 3090; 30	-0.2825426	0.51268226		4.0
	ZINC49601713	-8.7	440.443	0.0769	10	6	314.91	0.38627	166.14	-6.4176	326.2985304218	0.28705	6.4095	0.2679	0.54839	0.405	0.96262 (max o	8	3531; 3530; 3090; 30	-0.2906563	0.5332945		4.0
	ZINC67913742	-8.7	421.464	-0.9847	8	2	305.6	0.32592	133.19	-8.4075	326.2985304218	0.29662	7.4711	-3.3198	0.46667	0.47687	0.9707 (max of	3	3543; 1942; 1067	-0.9004448	-0.2548490		4.0
	ZINC71404465	-8.7	434.396	0.3932	10	6	291.63	0.4171	166.14	1.8995	326.2985304218	0.28705	6.0932	1.3698	0.41935	0.35036	0.95771 (max o	4	5273; 2275; 1943; 10	0.7094261	-0.4877656		4.0
	ZINC04257488	-8.7	357.369	0.0027999	9	4	257.63	0.39025	125.55	-0.31782	326.2985304218	0.34225	6.4836	0.0081807	0.57692	0.3491	0.94951 (max o	8	3229; 2807; 2417; 23	-0.3881753	-0.7497920		4.0
	ZINC08740104	-8.7	402.410	-0.9124	11	6	284.97	0.45577	167.25	2.242	326.2985304218	0.30685	7.3988	-2.9735	0.48276	0.4037	0.94558 (max o	5	4202; 3780; 3111; 25	0.27672338	-0.5363676		4.0
	ZINC12603786	-8.7	393.438	-0.2762	9	4	292.5	0.33761	120.36	2.6343	326.2985304218	0.31781	6.7626	-0.86909	0.64286	0.56025	0.94291 (max o	6	3739; 2527; 2501; 21	0.26647636	-0.3691097		4.0
	ZINC35442338	-8.7	367.468	-2.522	8	3	292.17	0.31892	92.18	3.1217	326.2985304218	0.34225	9.0084	-7.3688	0.57692	0.5578	0.91731 (max o	4	4826; 3487; 3433; 34	-0.1479402	0.48609558		4.0
	ZINC67912740	-8.7	388.368	-1.827	10	4	263.15	0.45024	151.98	-4.331	326.2985304218	0.32958	8.3134	-5.5435	0.48148	0.36725	0.93058 (max o	2	4863; 3107	-0.7781706	-0.3647330		4.0
	ZINC79189487	-8.7	440.467	-2.7105	10	6	318.68	0.39579	150.35	-1.9787	326.2985304218	0.28705	9.1969	-9.4426	0.41935	0.44482	0.96018 (max o	5	4885; 2705; 2704; 16	0.7580236	-0.3464761		4.0
	ZINC17021318	-8.7	422.385	0.1214	10	7	282.99	0.43005	169.3	-4.0605	326.2985304218	0.29662	6.365	0.40928	0.53333	0.38308	0.96092 (max o	7	1937; 1332; 1306; 23	0.376754	-0.8092735		3.0
	ZINC12603948	-8.7	440.470	-1.086	10	5	325.57	0.33851	132.39	1.8539	326.2985304218	0.28705	7.5724	-3.7833	0.64516	0.58406	0.94768 (max o	6	5024; 3295; 2527; 14	0.2884075	-0.3989299		3.0
	ZINC12603945	-8.7	422.480	-1.1868	10	5	319.22	0.34525	132.39	3.1939	326.2985304218	0.29662	7.6732	-4.0011	0.66667	0.58197	0.95031 (max o	6	5066; 5016; 2501; 15	0.24779177	-0.4114798		3.0
	ZINC35454963	-8.7	422.385	0.1214	10	7	282.99	0.43005	169.3	-4.0605	326.2985304218	0.29662	6.365	0.40928	0.53333	0.3809	0.96092 (max o	7	1937; 1332; 233; 454	0.36569437	-0.8274072		3.0
	ZINC08298789	-8.7	400.481	-5.6447	9	3	308.1	0.34203	110.35	-1	326.2985304218	0.30685	12.131	-18.396	0.62069	0.46827	0.97176 (max o	5	3720; 3277; 2102; 76	-0.1981032	-0.0354523		3.0
	ZINC08299188	-8.7	414.508	-3.3471	9	3	320.37	0.30789	99.32	0.28325	326.2985304218	0.29662	9.8335	-11.284	0.63333	0.47816	0.97304 (max o	6	3720; 3277; 2860; 21	-0.1877724	0.00105935		3.0
	ZINC03839378	-8.7	428.487	-0.0794	9	2	312.86	0.29464	108.05	6.7539	326.2985304218	0.28705	6.5658	-0.27661	0.51613	0.36683	0.92863	1	1102	-0.3971227	-0.2889537		3.0
	ZINC04534331	-8.7	387.395	-0.5073	10	5	276.48	0.41102	145.78	-0.80219	326.2985304218	0.31781	6.9937	-1.5963	0.53571	0.40892	0.94862 (max o	5	2417; 1430; 351; 745	-0.4307928	-0.7090117		3.0
	ZINC15268999	-8.7	434.396	-0.258	10	7	290.74	0.42904	177.14	-2.4912	326.2985304218	0.28705	6.7444	-0.8988	0.51613	0.3986	0.96018 (max o	5	3370; 1909; 1908; 18	-0.7698227	-0.2468909		3.0

Structure	Molecule Name	affinity	Total Molweight	cLogP	H-Acceptors	H-Donors	Total Surface Area	Relative PSA	Polar Surface Area	Druglikeness	Kd (nM) @ 20°C	LE from K...	LLE from Kd ...	LELP from Kd (n...	Shape Index	Molecular...	Neighbor Si...	Neig...	Neighbor	Neighbor ...	Neighbor ...	Ring Systems	frequency
	ZINC22922319	-8.7	399.537	-3.8504	7	2	324.77	0.23087	75.44	7.9046	326.2985304218	0.30685	10.337	-12.548	0.62069	0.50757	0.96682 (max o	5	1882; 1299; 1274; 12	-0.1606717	0.1950034		3.0
	ZINC22922324	-8.7	417.527	-3.7496	7	2	331.12	0.22644	75.44	6.5646	326.2985304218	0.29662	10.236	-12.641	0.63333	0.51129	0.94529 (max o	4	1882; 1274; 1016; 12	-0.1514289	0.17550266		3.0
	ZINC02135911	-8.7	366.352	-1.5929	8	2	273.19	0.3405	122.13	6.1761	326.2985304218	0.32958	8.0793	-4.8332	0.59259	0.53675	0.93985 (max o	4	3152; 2851; 1985; 80	0.10422974	-0.2514210		3.0
	ZINC03808779	-8.7	383.468	-3.3779	8	2	260.44	0.37947	142.89	6.0586	326.2985304218	0.34225	9.8643	-9.8696	0.5	0.40569	0.8541 (max of	2	2393; 2392	0.45850202	-0.748537		3.0
	ZINC03848803	-8.7	397.749	-0.0936	8	0	268.24	0.34111	116.17	4.5556	326.2985304218	0.31781	6.58	-0.29452	0.57143	0.3433	0.97773 (max o	4	2390; 1382; 1243; 12	0.62416553	0.2615682		3.0
	ZINC03984839	-8.7	361.417	0.3681	7	3	249.05	0.2797	117.32	-7.0984	326.2985304218	0.34225	6.1183	1.0755	0.53846	0.37034	0.95257 (max o	2	4499; 1683	0.9769388	0.07220553		3.0
	ZINC04277720	-8.7	411.453	-0.1295	8	0	276.41	0.27246	95.61	6.1696	326.2985304218	0.31781	6.6159	-0.40748	0.57143	0.40618	0.88255 (max o	2	1720; 1149	-0.6568978	0.5669377		3.0
	ZINC04260221	-8.7	391.398	-0.6181	8	1	273.43	0.2665	90.39	4.6851	326.2985304218	0.31781	7.1045	-1.9449	0.60714	0.41001	0.96473 (max o	3	2381; 1720; 1154	-0.6607896	0.54515487		3.0
	ZINC04277769	-8.7	392.414	0.0963	8	0	292.49	0.25071	93.95	1.4785	326.2985304218	0.30685	6.3901	0.31384	0.62069	0.42618	0.95148 (max o	2	2450; 1497	-0.7283577	0.13364103		3.0
	ZINC08636506	-8.7	432.543	0.1804	8	4	310.31	0.36773	151.22	6.1785	326.2985304218	0.29662	6.306	0.60819	0.5	0.40373	0.97975 (max o	2	2885; 1465	0.43262616	0.09988184		3.0
	ZINC15675956	-8.7	423.472	-0.4634	9	2	313.41	0.29977	129.29	3.3939	326.2985304218	0.28705	6.9498	-1.6144	0.51613	0.54295	0.92821 (max o	6	4219; 3063; 2972; 18	0.26579294	-0.1496731		3.0
	ZINC19143072	-8.7	397.749	-0.0936	8	0	268.24	0.34111	116.17	4.5556	326.2985304218	0.31781	6.58	-0.29452	0.57143	0.3433	0.97773 (max o	4	2390; 1529; 1109; 12	0.62538916	0.23984976		3.0
	ZINC19143049	-8.7	377.331	-0.3557	8	0	265.08	0.34518	116.17	4.466	326.2985304218	0.31781	6.8421	-1.1192	0.57143	0.3433	0.97773 (max o	4	2390; 1529; 1109; 12	0.64341295	0.24998517		3.0
	ZINC20464836	-8.7	404.384	0.1295	7	3	280.49	0.26472	91.26	-4.2113	326.2985304218	0.31781	6.3569	0.40748	0.53571	0.56089	0.9279 (max of	5	5137; 4734; 4300; 25	-0.3715291	0.02388899		3.0
	ZINC20464836	-8.7	405.392	-1.9802	7	4	294.53	0.29977	92.46	-6.9594	326.2985304218	0.31781	8.4666	-6.2309	0.53571	0.56285	0.89081 (max o	2	5138; 4735	0.6954086	0.3590622		3.0
	ZINC22923213	-8.7	388.514	-4.1296	8	4	297.51	0.24769	83.26	8.0801	326.2985304218	0.31781	10.616	-12.994	0.57143	0.50203	0.92486 (max o	2	2654; 1601	-0.1823365	0.5081866		3.0
	ZINC36370063	-8.7	403.410	-1.5388	9	1	296.54	0.34211	125.07	5.979	326.2985304218	0.30685	8.0252	-5.0149	0.58621	0.4715	0.94515 (max o	2	1320; 1322	-0.7882992	0.28520626		3.0
	ZINC36369951	-8.7	431.463	-0.8661	9	1	321.3	0.31575	125.07	7.3832	326.2985304218	0.28705	7.3525	-3.0172	0.54839	0.49179	0.94515 (max o	2	1323; 1320	-0.7844979	0.32449093		3.0
	ZINC36369830	-8.7	389.383	-1.8981	9	1	285.54	0.35529	125.07	4.1252	326.2985304218	0.31781	8.3845	-5.9725	0.60714	0.47167	0.9279 (max of	2	1323; 1322	-0.777168	0.30500317		3.0
	ZINC40309475	-8.7	363.376	-0.682	8	3	260.21	0.30871	115.29	3.7493	326.2985304218	0.32958	7.1684	-2.0693	0.44444	0.46124	0.98371 (max o	6	2672; 1932; 1931; 18	-0.1511536	-0.4840757		2.0
	ZINC13322012	-8.7	418.397	0.5121	9	5	288.04	0.37682	145.91	-3.7146	326.2985304218	0.29662	5.9743	1.7265	0.53333	0.42987	0.97304 (max o	8	6202; 320; 368; 369;	0.05336876	-0.4098268		2.0
	ZINC35565112	-8.7	444.439	-4.8782	10	5	307.48	0.45076	182.49	3.1697	326.2985304218	0.27808	11.365	-17.542	0.375	0.37716	0.91243 (max o	3	42; 151; 1312	0.23114416	-0.2800662		2.0
	ZINC04073128	-8.7	392.403	0.4155	8	4	272.39	0.35038	125.68	-8.3754	326.2985304218	0.31781	6.0709	1.3074	0.42857	0.30543	0.95148 (max o	4	1685; 52; 79; 439	0.33489025	0.43325654		2.0
	ZINC49600056	-8.7	416.421	-1.5798	10	6	291.9	0.4063	158.3	-5.0311	326.2985304218	0.30685	8.0662	-5.1485	0.58621	0.51096	0.96983 (max o	9	6325; 530; 680; 841;	-0.5097041	0.6968831		2.0
	ZINC05415650	-8.7	449.526	-1.9047	10	2	342.94	0.3193	138.95	-1.584	326.2985304218	0.27808	8.3911	-6.8495	0.46875	0.54183	0.88944 (max o	3	4574; 2930; 600	-0.1082186	0.18481725		2.0
	ZINC31156020	-8.7	438.427	0.2591	10	5	296.68	0.39956	155.14	-11.09	326.2985304218	0.28705	6.2273	0.90263	0.48387	0.46257	0.96018 (max o	7	1895; 1307; 182; 235	-0.1879823	0.87258524		2.0
	ZINC03999066	-8.7	414.453	-1.3791	10	8	289.06	0.4204	163.9	1.6633	326.2985304218	0.30685	7.8655	-4.4944	0.51724	0.46749	0.95908 (max o	3	2398; 890; 1115	0.91514474	-0.348044		2.0
	ZINC08627141	-8.7	431.515	-2.7049	10	3	320.11	0.29018	120.56	1.3187	326.2985304218	0.28705	9.1913	-9.4231	0.64516	0.53435	0.9499	1	943	-0.8236968	-0.5226458		2.0
	ZINC20758750	-8.7	395.394	-1.9455	9	3	296.51	0.35199	130.67	6.3396	326.2985304218	0.30685	8.4319	-6.3403	0.55172	0.56375	0.96931 (max o	3	2243; 1011; 1259	0.57455707	-0.1732957		2.0
	ZINC22936954	-8.7	444.622	-3.0091	8	3	354.58	0.20734	76.94	5.5429	326.2985304218	0.27808	9.4955	-10.821	0.625	0.4985	0.92326 (max o	6	3438; 504; 810; 811;	-0.558156	0.03248760		2.0
	ZINC04097985	-8.7	408.402	0.024	9	4	279.86	0.37676	134.91	-7.4042	326.2985304218	0.30685	6.4624	0.078215	0.51724	0.46165	0.91903 (max o	7	3906; 1904; 1902; 19	-0.7359786	-0.3326702		2.0
	ZINC35457540	-8.7	370.353	-1.0161	9	5	254.3	0.42682	145.91	-4.9227	326.2985304218	0.34225	7.5025	-2.9689	0.46154	0.4923	0.95257 (max o	3	1607; 672; 1055	-0.5746546	0.78892684		2.0
	ZINC02126962	-8.7	394.406	-0.3874	8	2	298.81	0.31093	118.64	5.0485	326.2985304218	0.30685	6.8738	-1.2625	0.58621	0.53471	0.9771 (max of	4	2979; 1653; 1364; 69	0.18382859	-0.2452114		2.0
	ZINC02130102	-8.7	389.390	-1.2078	8	2	291.01	0.3227	117.69	6.3043	326.2985304218	0.30685	7.6942	-3.9362	0.55172	0.49633	0.89488 (max o	4	3421; 3152; 1265; 39	0.1017611	-0.2869114		2.0
	ZINC20763278	-8.7	449.442	-1.3478	10	2	335.53	0.33949	136.15	6.3319	326.2985304218	0.26965	7.8342	-4.9983	0.54545	0.49494	0.90055 (max o	3	6078; 3019; 1089	0.06553035	-0.3043401		2.0
	ZINC03197505	-8.7	387.319	-0.8425	9	2	262.94	0.40526	134.58	0.9819	326.2985304218	0.31781	7.3289	-2.651	0.46429	0.2766	0.9543 (max of	3	6084; 5313; 1657	-0.1908738	0.75686246		2.0
	ZINC03999067	-8.7	414.453	-1.3791	10	8	289.06	0.4204	163.9	1.6633	326.2985304218	0.30685	7.8655	-4.4944	0.51724	0.46956	0.95908 (max o	3	2398; 890; 1114	0.90449035	-0.3311994		2.0
	ZINC04044097	-8.7	352.365	-1.6024	6	0	230.78	0.27693	78.9	1.607	326.2985304218	0.34225	8.0888	-4.6819	0.42308	0.1538	0.93046 (max o	4	1393; 340; 561; 894	-0.696844	-0.0334054		2.0
	ZINC04063308	-8.7	358.369	-0.5967	7	0	264.58	0.29084	95.97	3.6644	326.2985304218	0.34225	7.0831	-1.7435	0.5	0.38226	0.89253 (max o	2	2405; 1396	0.12208930	0.14782275		2.0
	ZINC04097530	-8.7	427.405	-2.4776	11	6	298.59	0.44261	182.09	-10.383	326.2985304218	0.29662	8.964	-8.3528	0.53333	0.48862	0.85608	1	1023	-0.3949517	0.31096077		2.0
	ZINC31161541	-8.7	416.421	-1.6852	10	6	289.14	0.41018	158.3	1.0636	326.2985304218	0.30685	8.1716	-5.492	0.55172	0.50657	0.97122 (max o	3	2635; 2045; 820	-0.4432226	0.39397168		2.0
	ZINC04270660	-8.7	384.415	-0.6196	9	1	282.23	0.32466	101.04	0.3396	326.2985304218	0.31781	7.106	-1.9496	0.57143	0.32163	0.92094 (max o	4	2816; 1718; 267; 114	0.00465752	-0.2618452		2.0
	ZINC04963689	-8.7	376.404	-0.0617	7	3	240.04	0.35569	113.29	-2.0138	326.2985304218	0.32958	6.5481	-0.18721	0.37037	0.32554	0.96343 (max o	3	2460; 2410; 259	0.64610076	0.6586648		2.0

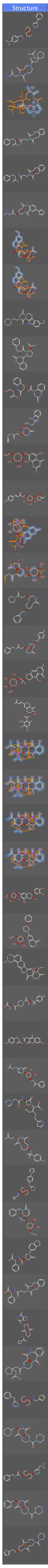

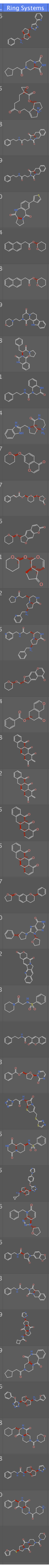

Structure	Molecule Name	affinity	Total Molweight	cLogP	H-Acceptors	H-Donors	Total Surface Area	Relative PSA	Polar Surface Area	Druglikeness	Kd (nM) @ 20°C	LE from K...	LLE from Kd ...	LELP from Kd (n...	Shape Index	Molecular...	Neighbor Si...	Neig...	Neighbor	Neighbor ...	Neighbor ...	Ring Systems	frequency
	ZINC05396445	-8.7	407.496	-0.7288	8	1	305.1	0.23294	81.91	-2.144	326.2985304218	0.29662	7.2152	-2.457	0.63333	0.38628	0.90934 (max o	2	4122; 1736	-0.1183469	-0.2498836		2.0
	ZINC05415726	-8.7	379.392	-3.1138	10	1	269.21	0.39152	144.15	4.0962	326.2985304218	0.32958	9.6002	-9.4479	0.51852	0.58287	0.93367 (max o	2	1747; 1174	0.49957976	0.5109712		2.0
	ZINC06038041	-8.7	381.423	-5.252	8	1	263.69	0.31662	108.42	5.2676	326.2985304218	0.32958	11.738	-15.936	0.37037	0.18956	0.96885 (max o	3	2480; 1442; 1443	0.66459805	0.64524025		2.0
	ZINC12888385	-8.7	420.444	-0.964	8	1	310.41	0.27383	109.85	6.487	326.2985304218	0.28705	7.4504	-3.3583	0.54839	0.49782	0.96971 (max o	4	3642; 3207; 1127; 11	0.08917298	-0.9169038		2.0
	ZINC08791828	-8.7	420.444	-0.964	8	1	310.41	0.27383	109.85	6.487	326.2985304218	0.28705	7.4504	-3.3583	0.54839	0.49905	0.96971 (max o	4	4091; 3210; 1220; 11	0.10667389	-0.9289089		2.0
	ZINC12604305	-8.7	385.467	-0.8809	7	3	274.71	0.34105	123.36	6.5869	326.2985304218	0.32958	7.3673	-2.6728	0.55556	0.27959		0		0.9652429	-0.1594770		2.0
	ZINC14612370	-8.7	405.378	-1.568	9	3	285.96	0.38348	145.58	1.7924	326.2985304218	0.30685	8.0544	-5.1101	0.55172	0.43797	0.97415 (max o	3	1823; 1511; 1234	-0.7441095	-0.6031064		2.0
	ZINC14612369	-8.7	405.378	-1.568	9	3	285.96	0.38348	145.58	1.7924	326.2985304218	0.30685	8.0544	-5.1101	0.55172	0.42628	0.97415 (max o	3	1823; 1511; 1235	-0.7291940	-0.6205548		2.0
	ZINC20412734	-8.7	412.488	0.6574	8	1	302.1	0.23409	78.11	5.9497	326.2985304218	0.29662	5.829	2.2163	0.53333	0.43269	0.92507 (max o	2	4516; 2216	-0.3375256	-0.6539889		2.0
	ZINC20610738	-8.7	406.417	-0.3203	8	1	296.65	0.28653	109.85	6.5925	326.2985304218	0.29662	6.8067	-1.0798	0.4	0.47325	0.95837 (max o	4	2126; 1684; 282; 808	0.74826604	-0.0530698		2.0
	ZINC20757684	-8.7	425.420	-2.5129	10	4	315.36	0.37249	150.9	7.7064	326.2985304218	0.28705	8.9993	-8.7542	0.41935	0.51234	0.91964 (max o	3	2243; 1264; 1011	0.576041	-0.1947681		2.0
	ZINC22920835	-8.7	424.547	-4.0001	8	4	333.06	0.23122	84.95	5.7903	326.2985304218	0.28705	10.486	-13.935	0.58065	0.42837	0.92547	1	3864	-0.3246805	-0.8550674		2.0
	ZINC31156069	-8.7	354.310	-0.6025	9	5	238.99	0.45416	145.91	-9.613	326.2985304218	0.35594	7.0889	-1.6927	0.48	0.40384		0		-0.7907611	0.61003137		2.0
	ZINC31156368	-8.7	424.401	-0.5282	10	4	300.94	0.3937	151.98	-0.28537	326.2985304218	0.29662	7.0146	-1.7807	0.6	0.42846	0.95053 (max o	3	2255; 1575; 819	0.27612817	-0.9511017		2.0
	ZINC31163748	-8.7	424.401	-0.702	10	5	284.95	0.416	155.14	-6.6063	326.2985304218	0.29662	7.1884	-2.3667	0.46667	0.36804	0.95053 (max o	4	3906; 1902; 1901; 11	-0.7737389	-0.3436066		2.0
	ZINC33830308	-8.7	388.368	-1.827	10	4	263.15	0.45024	151.98	-6.054	326.2985304218	0.32958	8.3134	-5.5435	0.48148	0.37688	0.93996 (max o	2	2332; 1905	0.8032411	0.03565881		2.0
	ZINC35442254	-8.7	372.491	-1.0301	7	2	281.92	0.22471	82.15	6.2033	326.2985304218	0.32958	7.5165	-3.1255	0.59259	0.42096	0.96343 (max o	7	2279; 1563; 1302; 13	-0.5408317	0.15878282		2.0
	ZINC35442284	-8.7	426.514	-0.6227	7	3	321.41	0.22171	90.94	2.8575	326.2985304218	0.28705	7.1091	-2.1693	0.64516	0.46666	0.94768 (max o	4	1911; 1595; 1596; 15	-0.5044681	0.01780975		2.0
	ZINC35454967	-8.7	438.427	0.2591	10	5	296.68	0.39956	155.14	-11.09	326.2985304218	0.28705	6.2273	0.90263	0.48387	0.45556	0.96018 (max o	7	1895; 182; 235; 405;	-0.1969870	0.8533657		2.0
	ZINC35466102	-8.7	445.399	-1.014	10	3	317.43	0.38654	162.65	3.082	326.2985304218	0.27808	7.5004	-3.6464	0.5	0.39902	0.99646 (max o	3	2666; 2665; 1922	-0.0433219	-0.9135404		2.0
	ZINC35565112	-8.7	443.431	-3.3502	10	4	293.44	0.42448	181.29	4.8292	326.2985304218	0.27808	9.8366	-12.048	0.375	0.35331	0.92889 (max o	3	153; 1048; 1311	0.14068048	-0.3038968		2.0
	ZINC35565112	-8.7	444.439	-4.7165	10	5	307.48	0.45076	182.49	2.0633	326.2985304218	0.27808	11.203	-16.961	0.375	0.35489	0.91522 (max o	3	1315; 150; 1314	0.1771312	-0.3008392		2.0
	ZINC35565112	-8.7	443.431	-6.9542	10	4	308.66	0.45286	185.32	2.9967	326.2985304218	0.27808	13.441	-25.008	0.375	0.37716	0.92074 (max o	4	154; 170; 1311; 1312	0.19940777	-0.3014185		2.0
	ZINC35565112	-8.7	444.439	-4.8782	10	5	307.48	0.45076	182.49	3.0955	326.2985304218	0.27808	11.365	-17.542	0.375	0.37716	0.90114 (max o	2	1315; 1313	0.20925342	-0.2812315		2.0
	ZINC49601416	-8.7	422.385	0.0295	10	7	282.99	0.43005	169.3	-1.9967	326.2985304218	0.29662	6.4569	0.099455	0.53333	0.34139	0.89867 (max o	7	1937; 233; 454; 650;	0.3986212	-0.8072007		2.0
	ZINC01507679	-8.7	413.433	0.3389	10	4	291.34	0.36624	132.44	3.7769	326.2985304218	0.29662	6.1475	1.1425	0.46667	0.36185	0.9707 (max of	4	4693; 1775; 1469; 93	0.32637253	-0.4788420		1.0
	ZINC01611274	-8.7	422.460	-0.9053	8	3	308.82	0.30565	104.4	5.1921	326.2985304218	0.28705	7.3917	-3.1538	0.45161	0.28009		0		0.8912994	0.40906012		1.0
	ZINC02110020	-8.7	424.496	-1.6686	9	2	311.66	0.34798	144.09	-3.3174	326.2985304218	0.30685	8.155	-5.4379	0.62069	0.55545		0		-0.5380086	-0.7257823		1.0
	ZINC02152659	-8.7	396.374	-0.6886	8	3	289.66	0.34889	135.99	3.9006	326.2985304218	0.30685	7.175	-2.2441	0.58621	0.52225	0.84495	1	3136	-0.757523	0.4004624		1.0
	ZINC03066102	-8.7	405.366	-2.2367	12	4	287.74	0.48175	176.14	2.1547	326.2985304218	0.30685	8.7231	-7.2893	0.62069	0.50114		0		0.49527293	-0.7545993		1.0
	ZINC03830473	-8.7	439.480	-3.4744	12	1	291.68	0.63587	237.76	10.174	326.2985304218	0.31781	9.9608	-10.932	0.57143	0.44518		0		0.737977	0.674317		1.0
	ZINC03841213	-8.7	428.872	-1.7949	9	2	290.56	0.37324	144.09	7.3951	326.2985304218	0.31781	8.2813	-5.6478	0.60714	0.46773		0		-0.8658026	-0.3898345		1.0
	ZINC03841440	-8.7	410.457	-0.6873	10	1	294.76	0.32352	105.72	1.9634	326.2985304218	0.29662	7.1737	-2.3171	0.6	0.37958	0.96832 (max o	5	2764; 1213; 1147; 71	-0.0364664	-0.3175365		1.0
	ZINC03852688	-8.7	360.369	-1.3452	9	3	252.2	0.36661	118.19	-4.0555	326.2985304218	0.34225	7.8316	-3.9304	0.53846	0.44079	0.92368 (max o	3	4495; 2765; 2014	0.8116132	0.46773946		1.0
	ZINC04085310	-8.7	381.363	-1.005	8	1	274.84	0.32168	108	6.2661	326.2985304218	0.31781	7.4914	-3.1623	0.53571	0.34114	0.96519 (max o	3	4525; 2787; 1694	-0.0644583	-0.6689396		1.0
	ZINC04090361	-8.7	406.417	-1.4667	8	1	296.65	0.28653	109.85	6.3586	326.2985304218	0.29662	7.9531	-4.9447	0.53333	0.4544	0.93409 (max o	2	1220; 1198	0.12404814	-0.9396553		1.0
	ZINC04221576	-8.7	444.451	-2.8699	13	3	302.65	0.47286	186.61	2.7466	326.2985304218	0.27808	9.3563	-10.32	0.5	0.24588		0		-0.0305279	-0.9907229		1.0
	ZINC04236411	-8.7	398.438	-1.0509	8	1	290.1	0.293	109.85	3.7408	326.2985304218	0.30685	7.5373	-3.4248	0.48276	0.50866	0.91204	1	1140	-0.0621189	-0.8268449		1.0
	ZINC04259739	-8.7	370.388	-0.4275	9	1	269.97	0.33941	101.04	0.1575	326.2985304218	0.32958	6.9139	-1.2971	0.59259	0.33665	0.89334 (max o	6	3661; 1718; 1152; 34	-0.0299023	-0.2780306		1.0
	ZINC04260112	-8.7	378.471	0.519	8	1	281.49	0.25305	82.19	1.2501	326.2985304218	0.32958	5.9674	1.5748	0.59259	0.54522	0.86499 (max o	4	4974; 3410; 3230; 19	-0.2162303	0.18923028		1.0
	ZINC04260892	-8.7	387.462	-2.1445	9	3	307.31	0.33308	94.74	-1.3699	326.2985304218	0.31781	8.6309	-6.7478	0.64286	0.45467	0.91204 (max o	4	2442; 2080; 910; 106	-0.1931080	-0.0555538		1.0
	ZINC04270908	-8.7	371.459	-0.0266	7	2	273.39	0.23929	86.33	6.9388	326.2985304218	0.32958	6.513	-0.080709	0.59259	0.4615	0.86639 (max o	2	446; 735	-0.3095267	0.08860130		1.0
	ZINC04277760	-8.7	434.515	0.0562	9	0	305.99	0.31021	111.84	6.4961	326.2985304218	0.29662	6.4302	0.18947	0.56667	0.4847	0.85025 (max o	2	3240; 1422	-0.3338634	0.7413542		1.0

Structure	Molecule Name	affinity	Total Molweight	cLogP	H-Acceptors	H-Donors	Total Surface Area	Relative PSA	Polar Surface Area	Druglikeness	Kd (nM) @ 20°C	LE from K...	LLE from Kd ...	LELP from Kd (n...	Shape Index	Molecular...	Neighbor Si...	Neig...	Neighbor	Neighbor ...	Neighbor ...	Ring Systems	frequency
	ZINC05399162	-8.7	443.526	-1.909	10	2	345.43	0.30238	95.18	-0.35126	326.2985304218	0.27808	8.3954	-6.8649	0.625	0.48817	0.90529 (max c	5	3688; 3253; 2080; 44	-0.2263384	-0.1259277		1.0
	ZINC05414707	-8.7	437.459	-0.9362	11	2	324.38	0.3548	137.49	5.0549	326.2985304218	0.27808	7.4226	-3.3667	0.4375	0.49923	0.99867 (max c	7	4133; 2929; 2572; 24	-0.6874454	-0.4471827		1.0
	ZINC05414728	-8.7	437.459	-0.9362	11	2	324.38	0.3548	137.49	5.0549	326.2985304218	0.27808	7.4226	-3.3667	0.4375	0.49923	0.99867 (max c	7	4133; 2929; 2572; 24	-0.7265275	-0.4282562		1.0
	ZINC05415634	-8.7	442.518	-0.035	10	2	333.66	0.31205	124.6	0.97979	326.2985304218	0.27808	6.5214	-0.12586	0.4375	0.4959	0.96877 (max c	6	4127; 1178; 1176; 14	-0.0265343	0.22755155		1.0
	ZINC05415798	-8.7	366.393	-2.3664	9	1	263.05	0.34286	121.29	3.2034	326.2985304218	0.34225	8.8528	-6.9142	0.53846	0.58239	0.91522 (max c	4	2840; 1747; 1175; 11	0.5185667	0.52042425		1.0
	ZINC05415816	-8.7	412.464	-0.4279	8	0	302.7	0.25467	101.06	3.0732	326.2985304218	0.29662	6.9143	-1.4426	0.53333	0.53324	0.91599 (max c	4	4130; 2840; 1441; 11	0.51808697	0.49924636		1.0
	ZINC05433673	-8.7	444.490	-0.8523	11	2	328.94	0.32438	124.18	4.265	326.2985304218	0.27808	7.3387	-3.065	0.4375	0.53961	0.97743 (max c	4	1748; 1436; 961; 117	-0.0486548	0.22855699		1.0
	ZINC05433800	-8.7	444.514	-1.9882	11	3	327.64	0.34141	141.21	4.8256	326.2985304218	0.27808	8.4746	-7.1498	0.4375	0.45673	0.96186 (max c	8	4994; 2932; 1438; 88	-0.0158251	0.29335576		1.0
	ZINC05434143	-8.7	442.518	-0.4196	10	2	333.66	0.31205	124.6	0.97979	326.2985304218	0.27808	6.906	-1.5089	0.4375	0.45673	0.9497 (max of	5	2932; 1436; 143; 117	-0.0460367	0.26473537		1.0
	ZINC06137825	-8.7	421.543	-1.7368	7	2	335.64	0.22089	70.22	-3.0795	326.2985304218	0.28705	8.2232	-6.0505	0.64516	0.45801	0.92348 (max c	5	4568; 1736; 1434; 14	-0.0889342	-0.1998909		1.0
	ZINC06624194	-8.7	406.421	-0.2521	9	2	303.62	0.32182	119.39	4.9594	326.2985304218	0.29662	6.7385	-0.84991	0.46667	0.45555	0.88683 (max c	4	5001; 4241; 3711; 22	0.57949656	-0.2385521		1.0
	ZINC08382359	-8.7	446.407	0.6221	10	5	308.75	0.38394	155.14	-3.0787	326.2985304218	0.27808	5.8643	2.2371	0.46875	0.32892		0		0.93572223	-0.2543638		1.0
	ZINC08627143	-8.7	444.525	-0.2157	7	3	327.48	0.19568	89.85	3.3247	326.2985304218	0.27808	6.7021	-0.77568	0.65625	0.4893	0.92619 (max c	4	5025; 2926; 280; 467	0.09139089	-0.5449790		1.0
	ZINC08740134	-8.7	432.480	-2.9758	10	3	296.21	0.42338	159.21	13.08	326.2985304218	0.29662	9.4622	-10.032	0.43333	0.4237	0.9707	1	2507	0.14756708	-0.1133997		1.0
	ZINC08876451	-8.7	437.427	-1.2592	9	3	326.28	0.34467	144.86	4.7386	326.2985304218	0.27808	7.7456	-4.5282	0.59375	0.56266		0		-0.7350701	0.6761937		1.0
	ZINC08964926	-8.7	436.431	2.1812	12	5	310.51	0.44536	176.82	1.3234	326.2985304218	0.27808	4.3052	7.8438	0.5	0.2553	0.92074 (max c	3	211; 212; 285	-0.1432520	-0.7079081		1.0
	ZINC12481005	-8.7	429.559	-0.4332	7	1	354.36	0.22037	85.19	4.7027	326.2985304218	0.28705	6.9196	-1.5091	0.54839	0.60929	0.94501 (max c	4	5909; 4206; 3760; 10	-0.0626541	-0.2505731		1.0
	ZINC12603433	-8.7	408.406	-0.5026	10	3	289.04	0.37119	126.79	2.3827	326.2985304218	0.30685	6.989	-1.638	0.58621	0.51186	0.90299 (max c	3	3335; 2153; 2118	0.29865792	-0.3139813		1.0
	ZINC12603668	-8.7	405.473	-2.7624	9	4	283.39	0.28526	116.48	6.7655	326.2985304218	0.30685	9.2488	-9.0026	0.62069	0.50485	0.95148 (max c	5	1540; 1517; 1467; 10	-0.1036517	-0.6033296		1.0
	ZINC12657830	-8.7	434.507	-0.7045	8	3	308.85	0.32757	139.48	4.4409	326.2985304218	0.28705	7.1909	-2.4543	0.54839	0.34943	0.94751 (max c	3	3299; 3060; 3059	-0.8320067	-0.3240468		1.0
	ZINC13511671	-8.7	444.427	-9.36	13	6	313.65	0.48889	214.87	3.738	326.2985304218	0.27808	15.846	-33.659	0.625	0.57143	0.94718	1	623	0.12243052	0.95892984		1.0
	ZINC15657778	-8.7	362.420	0.7896	6	3	220.75	0.3277	96.22	-4.6899	326.2985304218	0.34225	5.6968	2.3071	0.34615	0.35749		0		-0.9064867	-0.0966839		1.0
	ZINC20410973	-8.7	378.511	0.5118	6	1	280.31	0.21722	65.54	3.3512	326.2985304218	0.32958	5.9746	1.5529	0.55556	0.47441	0.93686 (max c	3	4701; 2978; 1246	-0.8695086	-0.1404582		1.0
	ZINC20410978	-8.7	378.511	0.5118	6	1	280.31	0.21722	65.54	3.3512	326.2985304218	0.32958	5.9746	1.5529	0.55556	0.47441	0.93686 (max c	3	4701; 2978; 1245	-0.8524734	-0.1515818		1.0
	ZINC20504121	-8.7	430.571	-0.39	7	6	315.82	0.3079	125.3	4.3656	326.2985304218	0.29662	6.8764	-1.3148	0.53333	0.41259	0.88589 (max c	3	6272; 5574; 3009	-0.6690218	0.71782875		1.0
	ZINC20504121	-8.7	429.563	-0.4593	7	5	327.33	0.33223	123.69	4.6034	326.2985304218	0.29662	6.9457	-1.5485	0.53333	0.36034	0.88589 (max c	2	6273; 5823	0.4232588	0.2146547		1.0
	ZINC20758694	-8.7	399.426	-1.7603	9	3	267.49	0.37452	135.41	3.3751	326.2985304218	0.30685	8.2467	-5.7367	0.41379	0.25889	0.83335	1	386	-0.7448634	-0.4162617		1.0
	ZINC22936792	-8.7	427.523	-1.0772	8	4	326.1	0.26832	111.42	5.0948	326.2985304218	0.28705	7.5636	-3.7527	0.64516	0.46172	0.90045 (max c	4	4349; 4345; 4343; 17	-0.4526124	0.10609121		1.0
	ZINC22936949	-8.7	400.525	-4.7973	8	2	323.53	0.26566	88.33	7.9046	326.2985304218	0.30685	11.284	-15.634	0.62069	0.50695	0.96682 (max c	6	1882; 1299; 1276; 10	-0.1395579	0.19365072		1.0
	ZINC22936949	-8.7	402.541	-4.7374	8	4	336.28	0.29351	83.21	4.1783	326.2985304218	0.30685	11.224	-15.439	0.62069	0.51524	0.90299 (max c	3	1883; 1300; 1276	-0.1689337	0.1637555		1.0
	ZINC22936949	-8.7	401.533	-2.6277	8	3	322.24	0.26272	82.01	8.6613	326.2985304218	0.30685	9.1141	-8.5636	0.62069	0.51524	0.9279 (max of	4	1524; 1300; 1274; 12	-0.1351845	0.1595996		1.0
	ZINC30879596	-8.7	435.567	-4.7908	9	4	358.42	0.29862	94.9	6.0599	326.2985304218	0.28705	11.277	-16.69	0.6129	0.50512	0.89995 (max c	5	5585; 4779; 4404; 16	-0.0207155	0.5174274		1.0
	ZINC30879596	-8.7	435.567	-4.7908	9	4	358.42	0.29862	94.9	6.1059	326.2985304218	0.28705	11.277	-16.69	0.6129	0.47861	0.90171 (max c	3	5545; 2614; 1283	-0.4621229	-0.6859146		1.0
	ZINC30879596	-8.7	434.559	-2.6811	9	3	344.38	0.27002	93.7	9.9984	326.2985304218	0.28705	9.1675	-9.3402	0.6129	0.50512	0.9167 (max of	4	5546; 5182; 1600; 12	-0.0330677	0.4817875		1.0
	ZINC30879596	-8.7	434.559	-2.6811	9	3	344.38	0.27002	93.7	9.5375	326.2985304218	0.28705	9.1675	-9.3402	0.6129	0.47861	0.90171 (max c	3	5547; 2989; 1281	-0.4717124	-0.6659024		1.0
	ZINC31156634	-8.7	428.432	-1.2487	10	6	304.64	0.38931	158.3	-4.6719	326.2985304218	0.29662	7.7351	-4.2098	0.6	0.49355	0.89867 (max c	4	5681; 2643; 1589; 13	-0.4091338	0.40610775		1.0
	ZINC31165687	-8.7	368.340	0.5888	7	2	234.86	0.40254	101.05	0.46739	326.2985304218	0.32958	5.8976	1.7865	0.37037	0.24291	0.85214	1	423	0.7896238	-0.2177736		1.0
	ZINC31165874	-8.7	432.424	0.5139	9	1	303.09	0.37253	128.73	-6.4147	326.2985304218	0.28705	5.9725	1.7903	0.41935	0.33056	0.93414	1	3044	0.17002967	0.823236		1.0
	ZINC31168002	-8.7	408.402	0.024	9	4	279.86	0.37676	134.91	-7.4042	326.2985304218	0.30685	6.4624	0.078216	0.48276	0.46854	0.9617 (max of	3	1904; 1035; 1130	-0.7480304	-0.2952421		1.0
	ZINC35442295	-8.7	387.482	-4.9067	8	2	310.97	0.29713	94.32	2.8212	326.2985304218	0.31781	11.393	-15.439	0.60714	0.50382	0.9279 (max of	3	1268; 1274; 1300	-0.1274372	0.17818607		1.0
	ZINC35442295	-8.7	388.490	-2.7371	8	3	309.68	0.29421	88	3.6938	326.2985304218	0.31781	9.2235	-8.6125	0.60714	0.51227	0.9279 (max of	4	2200; 1275; 1276; 12	-0.1535111	0.15223897		1.0
	ZINC35442295	-8.7	388.490	-1.8521	8	2	291.92	0.25127	91.38	4.3417	326.2985304218	0.31781	8.3385	-5.8278	0.60714	0.46015	0.97288 (max c	6	2279; 1598; 664; 130	-0.5900246	0.1592895		1.0
	ZINC35442305	-8.7	386.518	-0.6881	7	2	295.68	0.21425	82.15	4.2141	326.2985304218	0.31781	7.1745	-2.1652	0.60714	0.46015	0.97288 (max c	6	2279; 1598; 1052; 12	-0.5791842	0.1447363		1.0

Structure	Molecule Name	affinity	Total Molweight	cLogP	H-Acceptors	H-Donors	Total Surface Area	Relative PSA	Polar Surface Area	Druglikeness	Kd (nM) @ 20°C	LE from K...	LLE from Kd ...	LELP from Kd (n...	Shape Index	Molecular...	Neighbor Si...	Neig...	Neighbor	Neighbor ...	Neighbor ...	Ring Systems	frequency
	ZINC35466044	-8.7	428.432	-1.3406	10	6	304.64	0.38931	158.3	-2.3607	326.2985304218	0.29662	7.827	-4.5196	0.6	0.46624	0.89867	1	1287	-0.4198076	0.44680554		1.0
	ZINC36454776	-8.7	446.491	-1.406	10	5	310.41	0.39168	162.98	-11.536	326.2985304218	0.28705	7.8924	-4.8981	0.45161	0.53911		0		0.69019294	-0.7230646		1.0
	ZINC38143641	-8.7	426.460	-0.8918	9	5	288.04	0.37682	145.91	-4.9225	326.2985304218	0.29662	7.3782	-3.0066	0.43333	0.33307	0.82862	1	1927	-0.8939613	-0.2162215		1.0
	ZINC39559781	-8.7	389.423	-0.5636	7	1	285.89	0.30256	112.96	-3.4687	326.2985304218	0.31781	7.05	-1.7734	0.5	0.33872	0.95148	1	2304	-0.6271373	-0.7767023		1.0
	ZINC40309284	-8.7	394.406	-1.749	8	2	305.56	0.30498	112.49	0.98724	326.2985304218	0.30685	8.2354	-5.6999	0.65517	0.54829	0.96428	1	847	-0.1110159	-0.8608762		1.0
	ZINC40309533	-8.7	363.376	-0.628	8	3	260.21	0.30871	115.29	3.7493	326.2985304218	0.32958	7.1144	-1.9055	0.44444	0.44125	0.97149 (max o	6	2672; 1932; 1931; 18	-0.1857649	-0.4585974		1.0
	ZINC67902438	-8.7	445.542	-0.1853	9	3	323.81	0.3432	136.15	5.5539	326.2985304218	0.28705	6.6717	-0.64553	0.45161	0.48107	0.91162 (max o	2	2932; 936	0.03860673	0.2760534		1.0
	ZINC68590284	-8.7	426.404	-4.2333	10	1	276.52	0.39357	144.59	-3.8417	326.2985304218	0.28705	10.72	-14.748	0.35484	0.33934	0.89103 (max o	2	996; 997	-0.1060493	-0.9592544		1.0
	ZINC68601972	-8.7	394.418	0.7135	8	6	270	0.3877	155.52	-6.4037	326.2985304218	0.31781	5.7729	2.2451	0.42857	0.39438	0.9428	1	4439	-0.8124623	-0.2710943		1.0
	ZINC72320089	-8.7	424.444	1.0699	9	4	304.63	0.3561	142.75	-12.085	326.2985304218	0.29662	5.4165	3.607	0.46667	0.40858	0.92257	1	323	0.57308286	0.5463041		1.0
	ZINC68604488	-8.7	429.544	-6.2479	12	8	351.6	0.39852	169.1	-0.76073	326.2985304218	0.29662	12.734	-21.064	0.53333	0.45638	0.90776 (max o	2	1340; 1341	0.05644591	-0.7970531		0.0
	ZINC68604488	-8.7	430.552	-7.4726	12	8	347.88	0.39209	173.68	-0.76073	326.2985304218	0.29662	13.959	-25.193	0.53333	0.4581	1 (max of 2)	2	1342; 1340	0.03738380	-0.8055677		0.0
	ZINC68604488	-8.7	430.552	-7.4726	12	8	347.88	0.39209	173.68	-0.76073	326.2985304218	0.29662	13.959	-25.193	0.53333	0.4581	1 (max of 2)	2	1342; 1341	0.03627251	-0.8256647		0.0
	ZINC02102910	-8.6	375.332	-4.0253	9	1	274.95	0.39051	147.1	-7.0397	387.4100126532	0.32579	10.437	-12.356	0.51852	0.59942	0.94883 (max o	4	2718; 1973; 425; 111	0.14382383	0.47775915		172.0
	ZINC02097997	-8.6	389.383	-2.1296	9	2	293.19	0.373	133.86	-0.83321	387.4100126532	0.31415	8.5414	-6.7789	0.57143	0.47708	0.9678 (max of	6	3573; 2353; 2351; 16	0.198031	-0.0061108		172.0
	ZINC02118003	-8.6	417.436	-1.4427	9	2	321.24	0.34043	133.86	-1.2647	387.4100126532	0.29321	7.8545	-4.9204	0.56667	0.52248	0.9561 (max of	4	3145; 2359; 1989; 87	0.12402514	-0.0614534		172.0
	ZINC02122226	-8.6	403.386	-3.1882	9	1	307.81	0.36445	144.89	-3.6318	387.4100126532	0.30332	9.6	-10.511	0.58621	0.46411	0.96385 (max o	8	3575; 3567; 3148; 29	0.28006053	0.10305352		172.0
	ZINC35131379	-8.6	392.458	-5.1858	10	6	293.82	0.39878	187.99	0.75411	387.4100126532	0.31415	11.598	-16.507	0.64286	0.62282	0.93686	1	6666	-0.0661743	-0.4076489		134.0
	ZINC35457653	-8.6	367.416	-0.5417	7	3	280.83	0.31927	127.12	-7.3952	387.4100126532	0.33832	6.9535	-1.6012	0.46154	0.62833	0.97269 (max o	3	7437; 7436; 7089	0.07772431	0.7962013		127.0
	ZINC03869423	-8.6	425.270	-8.4777	15	3	262.23	0.70705	253.29	-42.416	387.4100126532	0.32579	14.89	-26.022	0.51852	0.48752	0.93996 (max o	6	3173; 3172; 1678; 14	-0.6349376	0.43463054		67.0
	ZINC08586020	-8.6	424.178	-11.354	15	3	264.95	0.71006	260.71	-42.337	387.4100126532	0.32579	17.766	-34.852	0.51852	0.48777	0.93996 (max o	6	3360; 3284; 2873; 24	-0.6143614	0.45661113		67.0
	ZINC12494744	-8.6	425.270	-8.4777	15	3	262.23	0.70705	253.29	-42.416	387.4100126532	0.32579	14.89	-26.022	0.51852	0.49344	0.98593 (max o	11	6792; 3360; 3359; 33	-0.5980758	0.42828855		67.0
	ZINC04217451	-8.6	382.400	-3.9574	12	5	268.08	0.55032	214.72	-1.7419	387.4100126532	0.32579	10.369	-12.147	0.55556	0.4812	0.90719 (max o	3	6015; 5841; 1712	0.60680175	0.13972317		67.0
	ZINC08215481	-8.6	441.185	-10.923	16	5	270.33	0.74361	273.56	-47.565	387.4100126532	0.31415	17.335	-34.771	0.5	0.51473	0.9308 (max of	11	2487; 1584; 1506; 51	-0.2467444	-0.4834643		44.0
	ZINC08215481	-8.6	440.177	-12.171	16	4	271.51	0.74472	276.39	-42.835	387.4100126532	0.31415	18.583	-38.742	0.5	0.51454	0.9308 (max of	10	2739; 2488; 1585; 51	-0.2770491	-0.4342043		44.0
	ZINC20233092	-8.6	362.190	-9.0309	13	4	228.04	0.69606	211.43	-31.527	387.4100126532	0.36651	15.443	-24.64	0.5	0.43249	0.92619 (max o	5	3475; 3473; 2645; 25	-0.3541098	-0.3266429		44.0
	ZINC20233092	-8.6	361.182	-10.279	13	3	229.22	0.69763	214.26	-27.565	387.4100126532	0.36651	16.69	-28.044	0.5	0.45267	0.92619 (max o	6	4932; 3476; 3474; 26	-0.3222409	-0.3532072		44.0
	ZINC25076522	-8.6	405.430	-2.4684	10	4	303.62	0.3869	150.9	5.5613	387.4100126532	0.30332	8.8802	-8.138	0.44828	0.54949	0.9892 (max of	11	4528; 3822; 3786; 37	-0.0326056	-0.6554413		42.0
	ZINC12889375	-8.6	437.431	-1.7022	10	3	323.82	0.37434	161.73	5.0293	387.4100126532	0.27488	8.114	-6.1924	0.4375	0.51049	0.9642 (max of	7	3859; 3748; 1548; 15	0.67519945	-0.1248439		36.0
	ZINC20757784	-8.6	380.379	-0.9369	8	2	282.29	0.32913	118.64	4.6877	387.4100126532	0.31415	7.3487	-2.9823	0.42857	0.49791	0.96519 (max o	11	3859; 3748; 3346; 27	0.64961743	-0.0989294		36.0
	ZINC03985275	-8.6	410.405	-1.5043	9	3	301.14	0.35203	138.87	4.1266	387.4100126532	0.29321	7.9161	-5.1305	0.4	0.50497	0.9897 (max of	11	3859; 3748; 3346; 30	0.65697634	-0.0615747		36.0
	ZINC03985237	-8.6	396.378	-1.8636	9	3	290.14	0.36538	138.87	6.3696	387.4100126532	0.30332	8.2754	-6.144	0.41379	0.50667	0.9617 (max of	11	3859; 3748; 3346; 31	0.63612336	-0.1152505		36.0
	ZINC20756237	-8.6	423.404	-2.1566	10	3	310.06	0.39096	161.73	6.3427	387.4100126532	0.28375	8.5684	-7.6003	0.41935	0.50948	0.97856 (max o	14	4471; 4328; 3412; 31	0.71411705	-0.0755036		36.0
	ZINC20610700	-8.6	408.433	-0.2642	8	2	307.05	0.30259	118.64	5.5436	387.4100126532	0.29321	6.676	-0.90106	0.4	0.50593	0.97975 (max o	13	4471; 4328; 3414; 33	0.67286795	-0.0461397		36.0
	ZINC20755847	-8.6	437.431	-1.7022	10	3	323.82	0.37434	161.73	5.0293	387.4100126532	0.27488	8.114	-6.1924	0.4375	0.50945	0.96694 (max o	9	4471; 4328; 3412; 31	0.7212956	-0.0922064		36.0
	ZINC04349806	-8.6	430.408	0.2593	9	4	302.4	0.34868	134.91	-3.4398	387.4100126532	0.28375	6.1525	0.91383	0.58065	0.40392	0.96018 (max o	10	2135; 362; 582; 604;	0.25277936	0.6144127		30.0
	ZINC04349785	-8.6	446.407	-0.0864	10	5	308.75	0.38394	155.14	-3.4398	387.4100126532	0.27488	6.4982	-0.31432	0.53125	0.30874	0.95949 (max o	6	7349; 659; 743; 955;	0.13621037	0.5729215		30.0
	ZINC04090299	-8.6	387.347	-5.3038	10	1	275.93	0.40706	149.98	6.541	387.4100126532	0.31415	11.716	-16.883	0.53571	0.52118	0.96473 (max o	11	5464; 4091; 3642; 32	0.148537	-0.8919908		29.0
	ZINC38570140	-8.6	404.441	-1.2864	9	2	311.82	0.33093	121.72	4.9671	387.4100126532	0.30332	7.6982	-4.2411	0.51724	0.40532	0.93985 (max o	6	4425; 3942; 3184; 23	-0.2292924	0.5362856		28.0
	ZINC35000839	-8.6	441.185	-10.923	16	5	270.33	0.74361	273.56	-47.565	387.4100126532	0.31415	17.335	-34.771	0.5	0.50554	0.93686 (max o	9	1585; 512; 656; 781;	-0.2786322	-0.4545604		26.0
	ZINC13436558	-8.6	424.178	-11.319	15	3	266.42	0.70978	256.16	-39.309	387.4100126532	0.32579	17.731	-34.743	0.51852	0.51206	0.94302 (max o	9	2488; 1585; 513; 657	-0.3316424	-0.4626982		26.0
	ZINC13436558	-8.6	425.186	-10.071	15	4	265.24	0.70849	253.33	-44.039	387.4100126532	0.32579	16.483	-30.914	0.51852	0.51225	0.94302 (max o	10	2487; 1584; 512; 656	-0.2873600	-0.4944654		26.0
	ZINC35000839	-8.6	440.177	-12.171	16	4	271.51	0.74472	276.39	-42.835	387.4100126532	0.31415	18.583	-38.742	0.5	0.50536	0.93686 (max o	10	3175; 513; 657; 782;	-0.3292349	-0.4293631		26.0

Structure	Molecule Name	affinity	Total Molweight	cLogP	H-Acceptors	H-Donors	Total Surface Area	Relative PSA	Polar Surface Area	Druglikeness	Kd (nM) @ 20°C	LE from K...	LLE from Kd ...	LELP from Kd (n...	Shape Index	Molecular...	Neighbor Si...	Neig...	Neighbor	Neighbor ...	Neighbor ...	Ring Systems	frequency
	ZINC13538253	-8.6	439.169	-14.205	16	3	272.69	0.74583	279.22	-23.349	387.4100126532	0.31415	20.617	-45.216	0.42857	0.44654	0.9308 (max of	5	4453; 2700; 2177; 19	-0.3842553	-0.3699440		26.0
	ZINC13538253	-8.6	440.177	-12.957	16	4	271.51	0.74472	276.39	-33.279	387.4100126532	0.31415	19.369	-41.245	0.42857	0.42993	0.9308 (max of	4	4454; 2701; 2178; 15	-0.3923379	-0.3301348		26.0
	ZINC01599733	-8.6	353.302	-2.8445	9	5	246.47	0.47008	167.58	0.33063	387.4100126532	0.35185	9.2563	-8.0844	0.56	0.41449	0.97396 (max o	13	3467; 3051; 2623; 24	-0.1597752	-0.8447932		25.0
	ZINC01532763	-8.6	353.302	-2.8445	9	5	246.47	0.47008	167.58	0.33063	387.4100126532	0.35185	9.2563	-8.0844	0.56	0.41449	0.98188 (max o	13	3467; 3051; 2623; 24	-0.1741144	-0.8330877		25.0
	ZINC02138728	-8.6	353.302	-2.8445	9	5	246.47	0.47008	167.58	0.33063	387.4100126532	0.35185	9.2563	-8.0844	0.56	0.41449	0.98188 (max o	17	5090; 3467; 3158; 30	-0.2038762	-0.8171951		25.0
	ZINC12153723	-8.6	367.329	-2.5688	9	4	262.38	0.42976	156.58	0.46026	387.4100126532	0.33832	8.9806	-7.5929	0.53846	0.41317	0.97791 (max o	10	4797; 3158; 3032; 30	-0.1799515	-0.7856243		25.0
	ZINC08740520	-8.6	431.408	1.3955	13	6	305.76	0.49195	193.39	1.4288	387.4100126532	0.28375	5.0163	4.9181	0.48387	0.33853	0.95739 (max o	8	4625; 4623; 4624; 41	-0.1540950	-0.6734992		24.0
	ZINC02116851	-8.6	387.343	-4.047	9	1	279	0.40208	144.89	1.9281	387.4100126532	0.31415	10.459	-12.882	0.53571	0.4829	0.97037 (max o	4	2402; 426; 1086; 135	0.20888232	0.09734483		24.0
	ZINC02115518	-8.6	387.343	-3.9519	9	1	281.76	0.39814	144.89	0.92074	387.4100126532	0.31415	10.364	-12.58	0.57143	0.48963	0.96023 (max o	10	3142; 3134; 1979; 19	0.22736093	0.08459452		24.0
	ZINC02102192	-8.6	401.394	-2.0808	9	2	296.45	0.3689	133.86	-1.0092	387.4100126532	0.30332	8.4926	-6.8601	0.58621	0.43667	0.97176 (max o	7	3578; 3562; 2353; 19	0.2631177	0.02917953		24.0
	ZINC12885695	-8.6	426.400	-0.8918	9	2	316.1	0.34154	134.22	2.5282	387.4100126532	0.28375	7.3036	-3.1429	0.58065	0.43371	0.99016 (max o	16	4090; 2934; 2537; 25	0.5319163	0.42477348		24.0
	ZINC08790324	-8.6	426.400	-0.8918	9	2	316.1	0.34154	134.22	2.5282	387.4100126532	0.28375	7.3036	-3.1429	0.6129	0.43588	0.99016 (max o	14	3343; 3204; 2934; 27	0.49522832	0.41011128		24.0
	ZINC04087767	-8.6	412.373	-0.7537	9	1	308.51	0.33989	123.22	0.53295	387.4100126532	0.29321	7.1655	-2.5705	0.63333	0.39496	0.96343 (max o	7	5076; 2937; 2788; 24	0.546792	0.35908094		24.0
	ZINC04081872	-8.6	379.364	-0.749	9	4	267.76	0.39901	134.55	-8.6166	387.4100126532	0.32579	7.1608	-2.299	0.44444	0.40514	0.95201 (max o	4	1822; 1728; 1635; 25	0.33415174	0.37851384		24.0
	ZINC04073248	-8.6	366.365	0.0389	8	4	258.66	0.36898	125.68	-5.4845	387.4100126532	0.33832	6.3729	0.11498	0.5	0.31084	0.96389 (max o	6	2782; 2407; 1757; 25	0.35132167	0.527052		24.0
	ZINC31163589	-8.6	352.294	-0.7593	9	3	242.07	0.43533	131.75	-3.3333	387.4100126532	0.35185	7.1711	-2.158	0.52	0.39219		0		-0.4150495	-0.8928701		24.0
	ZINC84441937	-8.6	444.439	-4.7741	10	6	306.5	0.4524	185.65	2.9936	387.4100126532	0.27488	11.186	-17.368	0.375	0.36615	0.9922 (max of	7	21; 41; 74; 114; 121;	0.04219149	-0.5614106		22.0
	ZINC08764929	-8.6	372.396	-0.262	7	0	278.34	0.27646	95.97	4.3706	387.4100126532	0.32579	6.6738	-0.80421	0.51852	0.53029	0.97149 (max o	7	2129; 2122; 2036; 19	0.27857432	0.19277371		22.0
	ZINC25763680	-8.6	434.352	0.2551	11	7	284.9	0.47294	186.37	-3.8653	387.4100126532	0.28375	6.1567	0.89903	0.41935	0.41786	0.95771 (max o	18	4095; 2776; 2566; 25	-0.6815734	0.3184669		21.0
	ZINC04416342	-8.6	448.379	0.5798	11	7	295.9	0.45536	186.37	1.9289	387.4100126532	0.27488	5.832	2.1093	0.40625	0.42191	0.95949 (max o	14	4095; 2776; 2566; 25	-0.7369413	0.30663395		21.0
	ZINC04416340	-8.6	448.379	0.5798	11	7	295.9	0.45536	186.37	1.9289	387.4100126532	0.27488	5.832	2.1093	0.40625	0.42191	0.95949 (max o	14	4095; 2776; 2566; 25	-0.7203091	0.26963857		21.0
	ZINC38396388	-8.6	418.353	0.6008	10	6	278.55	0.43669	166.14	-3.8653	387.4100126532	0.29321	5.811	2.0491	0.43333	0.4152	0.95579 (max o	7	4260; 3042; 2457; 20	-0.7122618	0.3443207		21.0
	ZINC31155769	-8.6	418.353	0.6008	10	6	278.55	0.43669	166.14	-3.8653	387.4100126532	0.29321	5.811	2.0491	0.43333	0.4152	0.95579 (max o	7	4260; 3042; 2483; 24	-0.6921823	0.3415807		21.0
	ZINC04349442	-8.6	448.379	-0.0011999	11	7	297.4	0.45306	186.37	-3.6679	387.4100126532	0.27488	6.413	-0.0043652	0.40625	0.43252	0.96186 (max o	8	4260; 3174; 2768; 24	-0.7282011	0.3814733		21.0
	ZINC14682250	-8.6	431.440	-0.1614	9	4	308.3	0.35641	142.39	0.6376	387.4100126532	0.28375	6.5732	-0.56881	0.51613	0.4269	0.97199 (max o	11	3881; 3441; 3362; 29	-0.3236089	-0.7207832		19.0
	ZINC08643826	-8.6	426.924	0.2371	8	5	300.76	0.38456	148.35	6.559	387.4100126532	0.31415	6.1747	0.75473	0.60714	0.45098	0.9398 (max of	5	5483; 4229; 3716; 25	-0.5093754	0.40784252		17.0
	ZINC08585026	-8.6	402.144	-10.74	14	4	245.67	0.70416	237.67	-50.91	387.4100126532	0.35185	17.152	-30.524	0.56	0.61742	0.92662 (max o	5	3728; 3726; 2870; 62	-0.5716853	-0.3948282		17.0
	ZINC08585026	-8.6	401.136	-11.987	14	3	246.85	0.70557	240.5	-46.18	387.4100126532	0.35185	18.399	-34.07	0.56	0.61722	0.92662 (max o	6	3729; 3727; 2916; 28	-0.5587107	-0.4347735		17.0
	ZINC02114009	-8.6	397.362	-1.5152	9	2	294.51	0.37133	133.86	-0.27777	387.4100126532	0.30332	7.927	-4.9954	0.62069	0.4219	0.9218	1	1651	0.5988104	-0.6106484		16.0
	ZINC04349421	-8.6	432.380	-0.078	10	7	289.19	0.43134	177.14	-1.9977	387.4100126532	0.28375	6.4898	-0.27489	0.51613	0.3816	0.96018 (max o	12	4107; 3788; 3213; 28	0.73476803	0.0805227		14.0
	ZINC02129034	-8.6	435.415	-1.8242	10	4	314.31	0.38179	154.66	6.5169	387.4100126532	0.27488	8.236	-6.6363	0.40625	0.46618	0.9642 (max of	8	2534; 1877; 1654; 28	0.7165302	-0.1273872		14.0
	ZINC20758452	-8.6	419.416	-0.8975	9	3	306.46	0.34882	134.43	4.8339	387.4100126532	0.28375	7.3093	-3.163	0.41935	0.45781	0.96971 (max o	9	2540; 2161; 1877; 29	0.7100708	-0.1976035		14.0
	ZINC12889236	-8.6	449.442	-1.4649	10	4	325.31	0.36888	154.66	4.2777	387.4100126532	0.26655	7.8767	-5.4957	0.39394	0.46211	0.99099 (max o	10	2540; 2534; 2161; 16	0.7448386	-0.1739980		14.0
	ZINC06137872	-8.6	408.478	-0.254	9	2	291.43	0.33531	124.27	6.3995	387.4100126532	0.31415	6.6658	-0.80853	0.5	0.52101	0.97037 (max o	5	3257; 3256; 2867; 93	0.08455329	0.36650515		14.0
	ZINC20467425	-8.6	353.421	-0.7651	9	3	269.32	0.34769	110.85	6.3927	387.4100126532	0.35185	7.1769	-2.1745	0.52	0.56215	0.93348 (max o	4	5815; 5011; 2202; 27	-0.1332737	0.28531012		13.0
	ZINC12866906	-8.6	448.494	-1.0566	10	3	342.61	0.33861	148.1	-45.666	387.4100126532	0.27488	7.4684	-3.8438	0.625	0.56425	0.9499	1	1783	0.37975886	-0.0487423		12.0
	ZINC08627177	-8.6	431.483	-0.4724	9	3	298.52	0.32286	127.79	5.4812	387.4100126532	0.30332	6.8842	-1.5574	0.62069	0.56162	0.90418 (max o	5	3290; 2112; 208; 942	-0.0197971	-0.5792596		11.0
	ZINC08879751	-8.6	372.396	-0.1515	7	0	279.84	0.27498	95.97	4.338	387.4100126532	0.32579	6.5633	-0.46503	0.59259	0.54449	0.97149 (max o	7	2850; 2484; 249; 363	0.35346314	0.18510133		11.0
	ZINC02156415	-8.6	399.354	-3.2845	9	0	291.39	0.37194	140.01	-10.183	387.4100126532	0.30332	9.6963	-10.829	0.48276	0.53664	0.96385 (max o	6	332; 425; 545; 865; 1	0.19978972	0.47011188		11.0
	ZINC02092925	-8.6	399.378	-1.5213	9	2	296.48	0.38272	137.77	-3.0203	387.4100126532	0.30332	7.9331	-5.0155	0.58621	0.51596	0.92486 (max o	2	3141; 867	0.07978804	0.18975724		11.0
	ZINC35442236	-8.6	395.418	-0.966	9	2	290.74	0.32314	129.29	-0.68108	387.4100126532	0.30332	7.3778	-3.1848	0.55172	0.44214	0.98166 (max o	10	2192; 223; 224; 376;	0.35923794	-0.1091977		10.0
	ZINC15675979	-8.6	397.390	-1.8343	10	2	286.98	0.36222	138.52	3.7664	387.4100126532	0.30332	8.2461	-6.0474	0.55172	0.44894	0.95872 (max o	11	2192; 1591; 223; 224	0.378585	-0.1037052		10.0
	ZINC04237123	-8.6	415.852	-0.8897	7	0	296.54	0.23774	89.98	3.5828	387.4100126532	0.30332	7.3015	-2.9332	0.62069	0.45728	0.93686 (max o	6	4575; 2805; 2436; 20	-0.5273489	-0.3435062		10.0

Structure	Molecule Name	affinity	Total Molweight	cLogP	H-Acceptors	H-Donors	Total Surface Area	Relative PSA	Polar Surface Area	Druglikeness	Kd (nM) @ 20°C	LE from K...	LLE from Kd ...	LELP from Kd (n...	Shape Index	Molecular...	Neighbor Si...	Neig...	Neighbor	Neighbor ...	Neighbor ...	Ring Systems	frequency
	ZINC03838465	-8.6	437.491	8.9991e-4	10	6	325.91	0.37679	157.22	2.0255	387.4100126532	0.28375	6.4109	0.0031715	0.54839	0.50918	0.93956 (max o	6	1861; 190; 1374; 885	-0.4518464	-0.3441126		10.0
	ZINC03838466	-8.6	441.454	-0.2422	10	6	320	0.38375	157.22	0.68546	387.4100126532	0.28375	6.654	-0.85357	0.51613	0.50918	0.93956 (max o	4	4914; 883; 886; 1373	-0.4407818	-0.3011998		10.0
	ZINC03840167	-8.6	417.820	-1.1285	9	6	285.46	0.4085	161.98	0.36843	387.4100126532	0.31415	7.5403	-3.5922	0.53571	0.47056	0.9426 (max of	5	2010; 1673; 433; 884	-0.4164678	-0.4958458		10.0
	ZINC22922192	-8.6	387.439	-0.3922	9	3	285.65	0.31409	110.09	2.3418	387.4100126532	0.31415	6.804	-1.2484	0.57143	0.45873	0.93975 (max o	8	5180; 4732; 3872; 38	-0.6658902	-0.2557137		10.0
	ZINC04084331	-8.6	404.466	-1.3969	8	2	284.95	0.38744	143.94	6.4109	387.4100126532	0.31415	7.8087	-4.4466	0.5	0.33153	0.96253 (max o	10	5356; 4524; 4245; 40	0.38462457	-0.6699673		10.0
	ZINC04259906	-8.6	414.441	-1.08	10	1	305.96	0.36475	131.29	0.93679	387.4100126532	0.29321	7.4918	-3.6834	0.56667	0.42407	0.92304 (max o	4	2438; 576; 908; 1416	-0.2831974	-0.3384812		10.0
	ZINC08792101	-8.6	415.429	-1.8817	11	3	305.89	0.41198	162.48	8.2206	387.4100126532	0.29321	8.2935	-6.4176	0.5	0.49178	0.96545 (max o	4	3945; 3082; 2900; 28	-0.0983586	-0.8759125		10.0
	ZINC35442306	-8.6	385.530	0.2588	6	2	296.92	0.17641	69.26	4.2141	387.4100126532	0.31415	6.153	0.82381	0.60714	0.47159	0.96519 (max o	11	3066; 664; 665; 811;	-0.5913918	0.10381422		9.0
	ZINC04349808	-8.6	416.381	-0.4408	9	6	282.84	0.39471	156.91	-2.3989	387.4100126532	0.29321	6.8526	-1.5034	0.5	0.39702	0.95579 (max o	8	2481; 2463; 541; 588	0.7162538	0.17493585		9.0
	ZINC35442319	-8.6	415.556	-0.2573	7	2	317.92	0.19621	78.49	6.0083	387.4100126532	0.29321	6.6691	-0.87753	0.6	0.46363	0.96832 (max o	5	2279; 1862; 834; 130	-0.5552488	0.17393497		9.0
	ZINC22936711	-8.6	401.529	-0.1532	7	2	305.42	0.20424	78.49	6.2064	387.4100126532	0.30332	6.565	-0.50508	0.62069	0.45028	0.9339 (max of	14	3876; 3875; 3437; 34	-0.51019	0.12033901		9.0
	ZINC06143121	-8.6	432.380	-0.3621	10	6	292.84	0.41538	166.14	-3.6206	387.4100126532	0.28375	6.7739	-1.2761	0.54839	0.42904	0.97199 (max o	12	3558; 2847; 2138; 21	0.21855983	0.696595		9.0
	ZINC12139865	-8.6	432.380	-0.3621	10	6	292.84	0.41538	166.14	-3.6206	387.4100126532	0.28375	6.7739	-1.2761	0.54839	0.42864	0.96262 (max o	9	3558; 2847; 2138; 21	0.17261256	0.68503195		9.0
	ZINC04098633	-8.6	390.387	0.8402	8	6	280.38	0.35167	139.84	-4.3466	387.4100126532	0.31415	5.5716	2.6745	0.57143	0.42303	0.9678 (max of	11	3902; 3899; 3375; 20	-0.5272103	0.7787235		9.0
	ZINC03978768	-8.6	404.414	1.1159	8	5	296.29	0.32232	128.84	-4.1548	387.4100126532	0.30332	5.2959	3.679	0.58621	0.43517	0.981 (max of 8	8	3902; 3899; 3375; 30	-0.541642	0.7895008		9.0
	ZINC03881960	-8.6	390.387	0.8402	8	6	280.38	0.35167	139.84	-4.3466	387.4100126532	0.31415	5.5716	2.6745	0.57143	0.42351	0.9678 (max of	8	4139; 4140; 3706; 28	-0.4882617	0.7965417		9.0
	ZINC35107805	-8.6	420.413	0.222	9	5	301.3	0.36024	145.91	-3.6574	387.4100126532	0.29321	6.1898	0.75714	0.6	0.47551	0.95053 (max o	8	2909; 2517; 2358; 20	0.38317093	0.571976		9.0
	ZINC04259914	-8.6	430.440	-0.9579	11	1	315.96	0.38486	140.52	0.87133	387.4100126532	0.28375	7.3697	-3.3759	0.58065	0.44439	0.90231 (max o	3	2809; 1719; 1415	-0.2724185	-0.3927568		9.0
	ZINC12867439	-8.6	428.531	-0.47	8	3	331.28	0.29244	132.97	5.9299	387.4100126532	0.28375	6.8818	-1.6564	0.58065	0.4936	0.97423 (max o	8	6947; 1802; 1688; 43	0.26648557	0.2764991		8.0
	ZINC35365984	-8.6	427.519	-2.0628	7	6	311.03	0.27856	108.83	2.5847	387.4100126532	0.28375	8.4746	-7.2698	0.41935	0.48554	0.91736 (max o	7	5902; 5119; 3981; 38	0.8822508	0.12991588		8.0
	ZINC20411558	-8.6	379.519	-1.4507	6	3	294.33	0.23912	74.44	2.1596	387.4100126532	0.32579	7.8625	-4.4529	0.55556	0.46431	0.95123 (max o	6	5176; 4771; 4704; 47	-0.0840351	0.07733717		8.0
	ZINC20411558	-8.6	379.519	-1.4507	6	3	294.33	0.23912	74.44	2.0856	387.4100126532	0.32579	7.8625	-4.4529	0.55556	0.49449	0.94605 (max o	7	5177; 4772; 4705; 47	-0.0807751	0.11835148		8.0
	ZINC35465901	-8.6	364.346	-3.2093	10	7	234.55	0.51887	169.3	-2.3427	387.4100126532	0.35185	9.6211	-9.1212	0.44	0.36432	0.98434 (max o	4	6338; 3197; 3078; 19	0.82313144	0.03706458		8.0
	ZINC12446750	-8.6	416.381	0.6966	9	5	281.79	0.39597	153.75	-4.2232	387.4100126532	0.29321	5.7152	2.3758	0.43333	0.25743	0.95053 (max o	4	62; 92; 288; 617	0.6496358	-0.0200540		7.0
	ZINC01592488	-8.6	384.339	-2.3176	8	0	288.6	0.349	132.86	0.2574	387.4100126532	0.31415	8.7294	-7.3773	0.57143	0.39524	0.95148 (max o	3	2714; 2346; 1964	-0.8318137	-0.2648215		7.0
	ZINC38890035	-8.6	443.471	-3.1577	11	2	313.34	0.40314	144.48	1.7982	387.4100126532	0.28375	9.5695	-11.128	0.45161	0.45953	0.96971 (max o	5	3083; 2778; 2030; 19	-0.2801574	-0.8600709		7.0
	ZINC05412535	-8.6	432.380	0.3509	10	6	288.14	0.43271	173.98	-4.2232	387.4100126532	0.28375	6.0609	1.2367	0.45161	0.26019	0.99629 (max o	7	14; 205; 367; 370; 48	0.67395747	0.03018557		6.0
	ZINC08951914	-8.6	422.341	-0.4299	11	8	270.83	0.50895	197.37	-3.0467	387.4100126532	0.29321	6.8417	-1.4662	0.46667	0.3445	0.95837 (max o	10	3052; 1698; 104; 222	-0.0785596	-0.6823183		6.0
	ZINC15675979	-8.6	396.382	-1.6454	10	1	297.95	0.3857	137.27	4.2687	387.4100126532	0.30332	8.0572	-5.4246	0.55172	0.44894	0.97415 (max o	9	2194; 2193; 225; 643	0.38462564	-0.1436062		6.0
	ZINC15675979	-8.6	396.382	-1.6454	10	1	297.95	0.3857	137.27	4.2687	387.4100126532	0.30332	8.0572	-5.4246	0.55172	0.44894	0.97415 (max o	9	2194; 2193; 226; 644	0.36538172	-0.1430131		6.0
	ZINC34934406	-8.6	427.405	-2.4776	11	6	298.59	0.44261	182.09	-10.383	387.4100126532	0.29321	8.8894	-8.45	0.53333	0.48187	0.95837 (max o	5	680; 841; 842; 1039;	-0.4725081	0.6961406		6.0
	ZINC08577218	-8.6	439.387	-5.2983	13	4	322.54	0.5049	219.2	2.7383	387.4100126532	0.27488	11.71	-19.275	0.625	0.58116	0.97743 (max o	5	2350; 2206; 1993; 16	-0.5869193	0.7030861		6.0
	ZINC04259845	-8.6	398.382	-0.7078	12	1	286.97	0.42893	131.1	0.94667	387.4100126532	0.30332	7.1196	-2.3335	0.58621	0.38099	0.96682 (max o	6	2808; 2061; 2047; 17	-0.1006136	-0.3843210		6.0
	ZINC02134977	-8.6	394.406	-0.7669	8	2	293.29	0.31679	118.64	5.2365	387.4100126532	0.30332	7.1787	-2.5284	0.48276	0.46742	0.93686	1	3151	0.17493285	-0.1658277		6.0
	ZINC26026012	-8.6	357.450	-2.7913	7	2	272.19	0.40821	162.3	-1.413	387.4100126532	0.36651	9.2031	-7.6159	0.66667	0.65098	0.96029 (max o	5	7916; 7716; 7589; 72	-0.1658509	0.45244628		6.0
	ZINC04260896	-8.6	374.396	-0.3438	9	1	277.75	0.33595	96.73	-1.3064	387.4100126532	0.32579	6.7556	-1.0553	0.66667	0.38335	0.92124 (max o	3	2812; 2064; 911	-0.2884891	-0.1985007		5.0
	ZINC08791366	-8.6	430.479	-4.2934	9	2	321.07	0.32227	146.28	7.3144	387.4100126532	0.28375	10.705	-15.131	0.48387	0.48295	0.93956 (max o	4	2908; 2039; 601; 112	0.47940692	-0.7103730		5.0
	ZINC20465770	-8.6	415.508	-0.2759	7	4	335.33	0.26329	92.46	2.0671	387.4100126532	0.29321	6.6877	-0.94097	0.6	0.55312	0.92507 (max o	5	5184; 3405; 3005; 12	0.42833784	-0.2649692		5.0
	ZINC20465764	-8.6	437.917	0.5009	6	4	334.84	0.23381	83.23	0.68289	387.4100126532	0.29321	5.9109	1.7083	0.56667	0.54733	0.94251 (max o	6	4787; 4320; 3405; 30	0.40759784	-0.2592325		5.0
	ZINC04963990	-8.6	448.379	-0.4237	11	8	295.54	0.4664	197.37	-1.9977	387.4100126532	0.27488	6.8355	-1.5414	0.4375	0.38342	0.95949 (max o	7	3891; 2629; 2422; 20	0.74721277	0.02543776		5.0
	ZINC12489994	-8.6	424.479	-0.9356	9	4	333.89	0.33298	120.94	-3.7059	387.4100126532	0.28375	7.3474	-3.2973	0.58065	0.48938	0.93686 (max o	5	7910; 3763; 3326; 25	0.32213765	0.02753262		5.0
	ZINC22922192	-8.6	388.446	-2.5019	9	4	299.69	0.34622	111.29	-1.8296	387.4100126532	0.31415	8.9137	-7.964	0.57143	0.45873	0.92774 (max o	7	5181; 4732; 3873; 38	-0.7016629	-0.2452610		5.0
	ZINC26977303	-8.6	407.394	-1.2596	9	4	288.08	0.38087	148.74	-3.9908	387.4100126532	0.30332	7.6714	-4.1527	0.41379	0.28911	0.82039	1	7072	-0.8471865	-0.397365		5.0

Structure	Molecule Name	affinity	Total Molweight	cLogP	H-Acceptors	H-Donors	Total Surface Area	Relative PSA	Polar Surface Area	Druglikeness	Kd (nM) @ 20°C	LE from K...	LLE from Kd ...	LELP from Kd (n...	Shape Index	Molecular...	Neighbor Si...	Neig...	Neighbor	Neighbor ...	Neighbor ...	Ring Systems	frequency
	ZINC03839329	-8.6	418.424	-0.6664	9	2	294.69	0.3128	108.05	5.4543	387.4100126532	0.29321	7.0782	-2.2728	0.53333	0.39135	0.9426 (max of	3	701; 878; 1102	-0.4326435	-0.2581208		4.0
	ZINC08790504	-8.6	438.411	-0.5599	9	1	316.7	0.31591	125.43	6.3014	387.4100126532	0.27488	6.9717	-2.0369	0.59375	0.41531	0.9642 (max of	8	3204; 2934; 1804; 47	0.49588287	0.45082033		4.0
	ZINC08790502	-8.6	438.411	-0.5599	9	1	316.7	0.31591	125.43	6.3014	387.4100126532	0.27488	6.9717	-2.0369	0.59375	0.41531	0.9642 (max of	8	3204; 2934; 1804; 47	0.51588374	0.45876095		4.0
	ZINC15957214	-8.6	396.422	-3.8725	8	2	273.98	0.33911	118.64	1.6685	387.4100126532	0.30332	10.284	-12.767	0.37931	0.22113	0.95372 (max o	5	227; 395; 500; 501; 1	0.35014808	-0.5494970		4.0
	ZINC35458035	-8.6	353.305	-0.3835	7	2	238.43	0.37579	123.96	2.5684	387.4100126532	0.33832	6.7953	-1.1335	0.46154	0.25858	0.97533 (max o	3	1916; 521; 673	-0.8548217	-0.4878670		4.0
	ZINC49601730	-8.6	440.443	0.0769	10	6	314.91	0.38627	166.14	-6.1546	387.4100126532	0.28375	6.3349	0.27101	0.58065	0.46737	0.96018 (max o	6	3090; 3036; 531; 851	-0.3026747	0.57581747		4.0
	ZINC04259929	-8.6	448.367	-3.0863	14	1	311.29	0.48701	168.21	0.45414	387.4100126532	0.27488	9.4981	-11.228	0.59375	0.37145	0.95983 (max o	7	3649; 1737; 1719; 44	-0.2155911	-0.3921512		4.0
	ZINC03871265	-8.6	409.112	-4.2064	11	3	220.98	0.56806	160.83	-13.945	387.4100126532	0.38245	10.618	-10.999	0.47826	0.34473	0.92572 (max o	3	6405; 4053; 1111	-0.5807963	-0.7176069		4.0
	ZINC13430717	-8.6	415.426	-4.3719	12	5	256.85	0.50602	182.48	-2.4143	387.4100126532	0.31415	10.784	-13.917	0.46429	0.44266	0.95983 (max o	3	2171; 620; 780	-0.5270688	-0.7099916		4.0
	ZINC15958380	-8.6	424.475	-3.1866	8	2	300	0.3097	118.64	-1.065	387.4100126532	0.28375	9.5984	-11.23	0.3871	0.28661	0.96262 (max o	5	1843; 110; 228; 297;	0.38235214	-0.2309113		4.0
	ZINC32110859	-8.6	446.407	0.4497	10	4	312.4	0.36953	144.14	-3.4946	387.4100126532	0.27488	5.9621	1.636	0.59375	0.41419	0.97533 (max o	4	767; 829; 1199; 1319	0.34671733	0.58419377		4.0
	ZINC02130507	-8.6	366.352	-1.2962	8	2	271.29	0.34247	118.64	6.0145	387.4100126532	0.32579	7.708	-3.9787	0.55556	0.54033	0.96885 (max o	5	2241; 2027; 1653; 80	0.16724333	-0.2312358		4.0
	ZINC04237053	-8.6	436.486	-0.3427	8	1	327.85	0.25926	109.85	0.84345	387.4100126532	0.27488	6.7545	-1.2467	0.625	0.48026	0.98207 (max o	3	2435; 265; 903	-0.5260497	-0.4671367		4.0
	ZINC04270592	-8.6	395.395	0.1164	11	1	261.58	0.45806	135.07	2.7399	387.4100126532	0.32579	6.2954	0.35729	0.51852	0.29224	0.89169 (max o	3	575; 914; 915	-0.180277	-0.4166875		4.0
	ZINC35454760	-8.6	448.419	-2.0505	12	6	308.9	0.44869	176.76	-4.801	387.4100126532	0.28375	8.4623	-7.2264	0.54839	0.45309	0.96262 (max o	10	4359; 2951; 2624; 22	0.6063018	0.46053353		4.0
	ZINC49601709	-8.6	440.443	0.0769	10	6	314.91	0.38627	166.14	-6.4176	387.4100126532	0.28375	6.3349	0.27101	0.54839	0.405	0.96502 (max o	8	3531; 3530; 3090; 30	-0.2603610	0.54689974		4.0
	ZINC79190948	-8.6	426.440	-2.9798	10	6	307.68	0.40994	150.35	-4.6392	387.4100126532	0.29321	9.3916	-10.163	0.43333	0.43854	0.97533 (max o	6	4885; 2705; 2704; 23	0.74176645	-0.3610044		4.0
	ZINC79189483	-8.6	440.467	-2.7105	10	6	318.68	0.39579	150.35	-1.9787	387.4100126532	0.28375	9.1223	-9.5524	0.41935	0.44482	0.96018 (max o	5	4885; 2705; 2704; 13	0.76007336	-0.3688229		4.0
	ZINC05433675	-8.6	416.480	-0.3222	10	3	315.58	0.33148	123.74	6.2676	387.4100126532	0.29321	6.734	-1.0989	0.43333	0.53924	0.97533 (max o	4	4570; 4129; 2082; 76	0.08066065	0.21296377		4.0
	ZINC14855620	-8.6	392.359	0.5616	9	5	275.28	0.39429	145.91	0.057033	387.4100126532	0.31415	5.8502	1.7877	0.60714	0.42353	0.92805	1	627	-0.2536161	0.66265464		4.0
	ZINC31159282	-8.6	406.386	0.31	9	5	289.04	0.37552	145.91	0.063551	387.4100126532	0.30332	6.1018	1.022	0.58621	0.37685	0.93101 (max o	2	2308; 2046	-0.7660115	-0.0759942		4.0
	ZINC15676216	-8.6	390.442	-2.3919	7	3	288.97	0.24002	102.4	5.5735	387.4100126532	0.30332	8.8037	-7.8857	0.55172	0.48524	0.93405 (max o	5	6538; 5893; 5185; 42	-0.2724380	0.4919255		4.0
	ZINC20411558	-8.6	378.511	0.659	6	2	280.29	0.20101	73.24	6.0263	387.4100126532	0.32579	5.7528	2.0228	0.55556	0.49191	0.94605 (max o	4	5175; 4770; 4285; 15	-0.0966834	0.14930622		4.0
	ZINC35464832	-8.6	398.494	0.9599	7	4	288.68	0.29597	116.45	-3.8942	387.4100126532	0.31415	5.4519	3.0555	0.46429	0.4288	0.96519 (max o	4	5208; 3933; 1920; 19	-0.6891451	0.04528838		4.0
	ZINC13409670	-8.6	420.413	0.3111	9	6	291	0.39409	164.75	0.71166	387.4100126532	0.29321	6.1007	1.061	0.46667	0.40373	0.94785 (max o	2	2476; 337	-0.8851324	-0.1525384		3.0
	ZINC05413797	-8.6	409.466	-1.2549	10	2	290.19	0.37455	137.16	7.3056	387.4100126532	0.31415	7.6667	-3.9946	0.5	0.52101	0.97288 (max o	6	3257; 3256; 2867; 28	0.04476611	0.36174467		3.0
	ZINC08765321	-8.6	360.365	0.5334	8	1	266.01	0.31627	94.17	5.7863	387.4100126532	0.33832	5.8784	1.5766	0.53846	0.49818	0.90311 (max o	2	2124; 774	0.88285977	-0.3638384		3.0
	ZINC03839263	-8.6	400.434	-0.3384	9	2	286.84	0.32136	108.05	7.1175	387.4100126532	0.30332	6.7502	-1.1157	0.55172	0.35324	0.93965 (max o	6	2374; 250; 252; 429;	-0.4739122	-0.2370096		3.0
	ZINC15675286	-8.6	379.435	-3.0805	9	4	269.13	0.30038	116.48	6.307	387.4100126532	0.32579	9.4923	-9.4556	0.62963	0.53013	0.95148 (max o	4	108; 956; 1208; 1467	-0.1072480	-0.6255646		3.0
	ZINC08643581	-8.6	415.489	-3.2005	10	4	279.96	0.34426	141.93	6.5823	387.4100126532	0.31415	9.6123	-10.188	0.60714	0.54285	0.91157 (max o	4	1517; 108; 956; 1208	-0.0918024	-0.6389521		3.0
	ZINC04534329	-8.6	387.395	-0.5073	10	5	276.48	0.41102	145.78	-0.80219	387.4100126532	0.31415	6.9191	-1.6148	0.53571	0.40892	0.94862 (max o	5	2417; 351; 745; 976;	-0.4518464	-0.7051384		3.0
	ZINC15708582	-8.6	414.528	-1.5984	8	3	327.47	0.26485	86.19	9.0646	387.4100126532	0.29321	8.0102	-5.4514	0.6	0.47426	0.94501 (max o	4	2200; 984; 1525; 127	-0.1565207	0.13086759		3.0
	ZINC15708585	-8.6	432.518	-1.4976	8	3	333.82	0.25981	86.19	7.7246	387.4100126532	0.28375	7.9094	-5.2779	0.58065	0.47466	0.94501 (max o	4	3816; 984; 1240; 152	-0.1639967	0.0900835		3.0
	ZINC72325800	-8.6	396.354	-1.9403	9	1	292.45	0.37836	140.18	3.7716	387.4100126532	0.30332	8.3521	-6.3969	0.62069	0.53077	0.97878 (max o	4	3119; 2699; 1072; 10	-0.5356068	-0.2759408		3.0
	ZINC03848823	-8.6	393.330	-0.7696	9	0	275.08	0.36898	125.4	4.5517	387.4100126532	0.30332	7.1814	-2.5373	0.58621	0.35677	0.9398 (max of	3	2390; 1529; 1109	0.6629269	0.2533966		3.0
	ZINC12605263	-8.6	369.420	0.9087	7	0	271.51	0.22014	70.16	-0.76387	387.4100126532	0.32579	5.5031	2.7892	0.59259	0.4257	0.85534 (max o	4	4975; 4105; 2385; 11	-0.7002485	0.15753359		3.0
	ZINC08636508	-8.6	436.506	-0.0627	8	4	304.4	0.37487	151.22	4.8594	387.4100126532	0.29321	6.4745	-0.21384	0.5	0.40373	0.97975 (max o	3	5825; 2885; 1195	0.43482047	0.07794829		3.0
	ZINC35442240	-8.6	381.391	-1.7019	9	2	278.48	0.33737	129.29	3.6869	387.4100126532	0.31415	8.1137	-5.4174	0.53571	0.49307	0.95945 (max o	3	1835; 642; 1239	0.2962346	-0.1280676		3.0
	ZINC18180158	-8.6	393.330	-0.7696	9	0	275.08	0.36898	125.4	4.5517	387.4100126532	0.30332	7.1814	-2.5373	0.58621	0.35677	0.9398 (max of	3	1243; 1244; 1382	0.643169	0.22627163		3.0
	ZINC35442496	-8.6	402.541	-3.8767	8	3	308.61	0.21315	74.47	9.9414	387.4100126532	0.30332	10.289	-12.781	0.58621	0.49995	0.94241 (max o	3	2654; 1602; 1272	-0.2035139	0.50418365		3.0
	ZINC03839256	-8.6	414.460	-0.3403	9	2	300.6	0.30665	108.05	5.7311	387.4100126532	0.29321	6.7521	-1.1606	0.5	0.42712	0.95267 (max o	3	132; 334; 429	-0.4648665	-0.1815573		3.0
	ZINC03840045	-8.6	410.445	-3.6701	10	5	302.87	0.43197	181.88	-0.76388	387.4100126532	0.30332	10.082	-12.1	0.51724	0.57679	0.93091 (max o	3	3601; 2008; 2009	0.5736153	0.7315099		3.0
	ZINC05415755	-8.6	400.453	-0.2363	8	0	296.68	0.25984	101.06	4.764	387.4100126532	0.30332	6.6481	-0.77905	0.55172	0.57169	0.91804	1	3260	0.2478877	0.95297414		3.0

Structure	Molecule Name	affinity	Total Molweight	cLogP	H-Acceptors	H-Donors	Total Surface Area	Relative PSA	Polar Surface Area	Druglikeness	Kd (nM) @ 20°C	LE from K...	LLE from Kd ...	LELP from Kd (n...	Shape Index	Molecular...	Neighbor Si...	Neig...	Neighbor	Neighbor ...	Neighbor ...	Ring Systems	frequency
	ZINC12603929	−8.6	425.455	−1.0799	9	4	314.11	0.31438	120.36	1.4302	387.4100126532	0.29321	7.4917	−3.683	0.63333	0.57166	0.94251 (max o	5	5821; 5066; 2928; 21	0.2889402	−0.3670813		3.0
	ZINC15676216	−8.6	391.450	−2.2603	7	4	303.01	0.27524	103.6	3.3254	387.4100126532	0.30332	8.6721	−7.4519	0.55172	0.43964	0.93405 (max o	4	6539; 5894; 5186; 42	0.14159536	−0.7544894		3.0
	ZINC20111270	−8.6	372.327	−2.8943	7	2	263.75	0.32409	126.33	−1.2255	387.4100126532	0.32579	9.3061	−8.884	0.55556	0.42401	0.92094 (max o	2	1530; 1531	−0.6941462	−0.6393454		3.0
	ZINC20111270	−8.6	372.327	−2.8943	7	2	263.75	0.32409	126.33	−1.2255	387.4100126532	0.32579	9.3061	−8.884	0.55556	0.42401	0.92094 (max o	2	1532; 1530	−0.6909265	−0.6199923		3.0
	ZINC20111270	−8.6	373.335	−1.3163	7	3	262.57	0.32106	123.5	−1.2255	387.4100126532	0.32579	7.7281	−4.0404	0.55556	0.42401	0.90441 (max o	2	1532; 1531	−0.7105981	−0.6223138		3.0
	ZINC35442271	−8.6	408.524	−0.6695	7	3	315.06	0.22618	90.94	4.1975	387.4100126532	0.29321	7.0813	−2.2834	0.63333	0.455	0.97304 (max o	7	5996; 1911; 319; 159	−0.4509573	0.01641770		3.0
	ZINC35442263	−8.6	426.514	−0.5687	7	3	321.41	0.22171	90.94	2.8575	387.4100126532	0.28375	6.9805	−2.0042	0.64516	0.46181	0.94768 (max o	6	5996; 1911; 1597; 15	−0.4694170	0.02963613		3.0
	ZINC35446975	−8.6	375.399	0.4905	7	1	265.48	0.25493	78.95	5.3426	387.4100126532	0.32579	5.9213	1.5056	0.55556	0.36635	0.94927 (max o	2	2380; 101	−0.4622092	−0.792567		2.0
	ZINC04044137	−8.6	419.492	−1.2533	7	3	284.29	0.31538	127.12	0.12303	387.4100126532	0.29321	7.6651	−4.2744	0.46667	0.36524	0.95579 (max o	4	2031; 134; 562; 891	0.28414705	0.8507298		2.0
	ZINC01311570	−8.6	406.445	−0.5489	9	3	295.76	0.3087	128.01	3.7341	387.4100126532	0.29321	6.9607	−1.872	0.46667	0.46887	0.94785 (max o	3	325; 326; 1350	−0.0160771	−0.9860123		2.0
	ZINC01311570	−8.6	405.437	−0.5489	9	2	307.27	0.33459	126.4	4.8974	387.4100126532	0.29321	6.9607	−1.872	0.46667	0.44371	0.94515 (max o	3	325; 326; 1349	−0.0035163	−0.9910609		2.0
	ZINC05413340	−8.6	440.522	−0.5006	9	3	343.87	0.2813	95.42	6.0627	387.4100126532	0.27488	6.9124	−1.8211	0.625	0.46123	0.94734 (max o	3	4324; 3670; 590	0.26116133	−0.6297104		2.0
	ZINC35457546	−8.6	370.353	−1.0161	9	5	254.3	0.42682	145.91	−4.9227	387.4100126532	0.33832	7.4279	−3.0034	0.46154	0.49901	0.95257 (max o	3	672; 1055; 1308	−0.5993952	0.76786363		2.0
	ZINC03840128	−8.6	440.455	−1.0211	10	6	324.25	0.39346	174.87	4.972	387.4100126532	0.27488	7.4329	−3.7147	0.5625	0.49465	0.971 (max of 4	4	3603; 2428; 704; 705	−0.4784492	−0.1215867		2.0
	ZINC08643547	−8.6	443.523	−0.8635	11	3	314.45	0.37628	153.57	7.1559	387.4100126532	0.29321	7.2753	−2.945	0.6	0.56187	0.88322	1	770	−0.0536762	−0.651759		2.0
	ZINC35330162	−8.6	446.447	−1.6498	11	6	314.16	0.40935	167.53	−4.8485	387.4100126532	0.28375	8.0616	−5.8143	0.6129	0.53116	0.8703 (max of	2	5681; 1287	−0.3988796	0.37716776		2.0
	ZINC72319974	−8.6	356.285	−2.8261	8	2	254.28	0.39657	139.18	1.0637	387.4100126532	0.33832	9.2379	−8.3534	0.57692	0.39662	0.93686 (max o	3	2324; 857; 858	0.68632305	0.5310542		2.0
	ZINC03977787	−8.6	434.396	−0.3162	10	7	290.74	0.42904	177.14	−4.2658	387.4100126532	0.28375	6.728	−1.1144	0.3871	0.32724	0.97642 (max o	3	2050; 2049; 889	−0.5324545	0.66714823		2.0
	ZINC04044050	−8.6	368.364	−2.0163	7	0	240.78	0.30696	88.13	1.713	387.4100126532	0.32579	8.4281	−6.189	0.44444	0.17486	0.93367 (max o	4	560; 893; 894; 1393	−0.6465756	−0.0158113		2.0
	ZINC20464469	−8.6	403.477	−0.1351	8	3	295.6	0.27348	102.34	6.8589	387.4100126532	0.30332	6.5469	−0.4454	0.62069	0.52072	0.94529 (max o	5	1764; 106; 466; 942;	−0.0635565	−0.5877914		2.0
	ZINC04044046	−8.6	368.364	−2.0163	7	0	240.78	0.30696	88.13	1.713	387.4100126532	0.32579	8.4281	−6.189	0.44444	0.17486	0.93373 (max o	4	560; 893; 1117; 1394	−0.66547	−0.0069158		2.0
	ZINC06031456	−8.6	381.423	−5.252	8	1	263.69	0.31662	108.42	5.2676	387.4100126532	0.32579	11.664	−16.121	0.37037	0.19001	0.96885 (max o	3	2480; 1183; 1443	0.6458221	0.61848754		2.0
	ZINC31156375	−8.6	424.401	−0.5282	10	4	300.94	0.3937	151.98	−0.28537	387.4100126532	0.29321	6.94	−1.8014	0.6	0.42846	0.95053 (max o	3	2255; 819; 1286	0.26503834	−0.9465689		2.0
	ZINC35442282	−8.6	408.524	−0.7235	7	3	315.06	0.22618	90.94	4.1975	387.4100126532	0.29321	7.1353	−2.4675	0.63333	0.46478	0.96832 (max o	6	5996; 1911; 1597; 12	−0.4860676	0.02111027		2.0
	ZINC00671043	−8.6	356.341	−0.449	10	2	257.34	0.40347	130.63	4.6343	387.4100126532	0.33832	6.8608	−1.3272	0.57692	0.53963	0.91351 (max o	2	1958; 1348	0.591288	0.67895144		2.0
	ZINC00671043	−8.6	357.349	−2.4268	10	3	246.37	0.37691	131.88	4.0499	387.4100126532	0.33832	8.8386	−7.1731	0.57692	0.53963	0.91351 (max o	2	1959; 1347	0.5721548	0.67159045		2.0
	ZINC03794622	−8.6	360.384	−1.7227	6	2	242.22	0.21414	86.84	2.1757	387.4100126532	0.33832	8.1345	−5.092	0.5	0.34931	0.9615	1	1631	0.2083069	−0.8416757		2.0
	ZINC03875439	−8.6	400.434	0.0218	8	1	279.19	0.39751	140.87	10.153	387.4100126532	0.31415	6.39	0.069393	0.5	0.36695	0.93686 (max o	5	3593; 1659; 1660; 68	0.41599056	0.68923306		2.0
	ZINC04040864	−8.6	356.353	−2.0541	7	0	238.96	0.33922	99.88	2.5289	387.4100126532	0.33832	8.4659	−6.0715	0.38462	0.26356	0.95257 (max o	3	4511; 4067; 3193	−0.4151393	0.8166016		2.0
	ZINC04061978	−8.6	358.369	−0.5967	7	0	264.58	0.29084	95.97	3.6644	387.4100126532	0.33832	7.0085	−1.7637	0.53846	0.38226	0.90377 (max o	3	1399; 341; 1119	0.15660262	0.1737988		2.0
	ZINC04062933	−8.6	358.369	−0.5825	7	0	267.87	0.28727	95.97	3.723	387.4100126532	0.33832	6.9943	−1.7218	0.53846	0.40009	0.89253 (max o	4	2405; 341; 551; 1396	0.13254322	0.2007063		2.0
	ZINC04221723	−8.6	447.518	−0.3911	11	3	333.24	0.42933	180.55	4.2772	387.4100126532	0.28375	6.8029	−1.3783	0.45161	0.47202	0.92584	1	5388	0.7212289	0.44797885		2.0
	ZINC04236520	−8.6	446.459	−2.4055	10	1	305.9	0.36136	144.53	3.2521	387.4100126532	0.28375	8.8173	−8.4775	0.58065	0.45165	0.85434	1	196	−0.4821998	−0.4356459		2.0
	ZINC04260752	−8.6	386.427	−0.6074	8	1	285.58	0.29764	109.85	4.3881	387.4100126532	0.31415	7.0192	−1.9335	0.53571	0.57902	0.90776 (max o	5	4553; 4103; 3664; 32	0.13386486	−0.5506006		2.0
	ZINC04277758	−8.6	352.390	−1.2458	9	0	247.55	0.29618	82.63	5.4483	387.4100126532	0.35185	7.6576	−3.5407	0.56	0.47972	0.85984 (max o	2	3240; 1156	−0.3375114	0.72159314		2.0
	ZINC05433718	−8.6	386.427	−0.523	8	0	285.68	0.26985	101.06	4.1889	387.4100126532	0.31415	6.9348	−1.6648	0.57143	0.58147	0.91599 (max o	3	4130; 3695; 1175	0.53661335	0.48677123		2.0
	ZINC06066740	−8.6	381.423	−5.252	8	1	263.69	0.31662	108.42	5.2676	387.4100126532	0.32579	11.664	−16.121	0.37037	0.18732	0.96885 (max o	3	2480; 1183; 1442	0.6459657	0.638497		2.0
	ZINC06137784	−8.6	364.401	−0.2911	9	1	267.52	0.36016	104.57	−7.1707	387.4100126532	0.33832	6.7029	−0.86043	0.61538	0.35129	0.87816	1	1723	−0.1444815	0.09691334		2.0
	ZINC08253590	−8.6	417.488	−0.5997	10	1	305.62	0.29798	100.37	1.1216	387.4100126532	0.29321	7.0115	−2.0453	0.56667	0.43395	0.92486 (max o	2	2387; 2386	−0.0229735	−0.390583		2.0
	ZINC08254132	−8.6	441.554	−1.8355	9	2	345.64	0.25934	88.16	3.8969	387.4100126532	0.27488	8.2473	−6.6774	0.59375	0.48848	0.97533 (max o	3	2098; 207; 358	0.83723783	−0.1742794		2.0
	ZINC08299575	−8.6	423.536	−0.9896	10	4	318.84	0.31797	127.46	6.0897	387.4100126532	0.29321	7.4014	−3.3751	0.43333	0.53255	0.89582 (max o	2	3697; 2323	−0.8359796	−0.1400814		2.0
	ZINC08624428	−8.6	367.376	−0.2251	8	5	262.73	0.36791	119.92	0.85393	387.4100126532	0.33832	6.6369	−0.66535	0.61538	0.54277	0.92032 (max o	6	5024; 4657; 3741; 32	0.29853937	−0.4488884		2.0
	ZINC08879178	−8.6	372.376	0.1094	8	2	256.08	0.32876	97.33	1.9472	387.4100126532	0.32579	6.3024	0.3358	0.55556	0.31805	0.92739 (max o	3	5035; 4198; 3308	−0.7990019	−0.4852551		2.0

Structure	Molecule Name	affinity	Total Molweight	cLogP	H-Acceptors	H-Donors	Total Surface Area	Relative PSA	Polar Surface Area	Druglikeness	Kd (nM) @ 20°C	LE from K...	LLE from Kd ...	LELP from Kd (n...	Shape Index	Molecular...	Neighbor Si...	Neig...	Neighbor	Neighbor ...	Neighbor ...	Ring Systems	frequency
	ZINC09033736	-8.6	388.447	0.1728	8	1	272.44	0.33721	118.82	5.1568	387.4100126532	0.32579	6.239	0.53041	0.51852	0.52159	0.84311	1	1636	0.5322334	0.6997362		2.0
	ZINC12296952	-8.6	364.357	-0.6284	10	1	247.45	0.36153	110.7	3.8382	387.4100126532	0.33832	7.0402	-1.8574	0.46154	0.40306	0.95559 (max o	3	3319; 2518; 2143	-0.0804445	-0.9166221		2.0
	ZINC12603621	-8.6	406.477	0.7493	8	3	305.8	0.28545	108.33	3.0663	387.4100126532	0.30332	5.6625	2.4703	0.65517	0.55185	0.93091 (max o	2	3335; 1799	0.34481394	-0.313393		2.0
	ZINC13523034	-8.6	428.420	-2.0209	10	2	317.79	0.36486	144.94	0.6894	387.4100126532	0.28375	8.4327	-7.1221	0.58065	0.55743		0		0.5332254	-0.7657852		2.0
	ZINC14612368	-8.6	405.378	-1.568	9	3	285.96	0.38348	145.58	1.7924	387.4100126532	0.30332	7.9798	-5.1695	0.55172	0.44443	0.97415 (max o	3	1823; 1234; 1235	-0.7468194	-0.6371834		2.0
	ZINC15672829	-8.6	378.427	-2.0753	9	1	284.93	0.32752	123.39	8.4	387.4100126532	0.32579	8.4871	-6.3701	0.59259	0.60895	0.96064 (max o	3	5111; 2189; 1004	-0.0721789	0.84522086		2.0
	ZINC15675171	-8.6	389.407	-0.7006	9	3	291.47	0.35122	125.13	4.8791	387.4100126532	0.31415	7.1124	-2.2301	0.60714	0.5597	0.97288 (max o	4	5454; 4654; 2920; 21	0.492718	-0.3767815		2.0
	ZINC15675177	-8.6	415.445	-0.4483	9	4	312.94	0.33927	130.01	0.98145	387.4100126532	0.29321	6.8601	-1.5289	0.66667	0.53452	0.94785 (max o	4	6752; 4652; 3289; 21	0.41612136	-0.4082227		2.0
	ZINC15675325	-8.6	417.484	-3.0591	9	5	303.24	0.34079	149.57	6.4151	387.4100126532	0.29321	9.4709	-10.433	0.5	0.49161	0.94488	1	1830	0.86846566	0.3911127		2.0
	ZINC15709514	-8.6	379.387	1.0416	8	0	277.84	0.25112	79.39	0.40258	387.4100126532	0.32579	5.3702	3.1972	0.51852	0.58948	0.85067 (max o	2	6437; 2441	-0.0736973	0.3370129		2.0
	ZINC20504099	-8.6	388.463	-2.1299	10	5	296.48	0.37173	132.39	4.324	387.4100126532	0.32579	8.5417	-6.5377	0.66667	0.62885	0.8934 (max of	3	5065; 618; 1210	0.22446032	-0.3761877		2.0
	ZINC22922292	-8.6	374.487	-5.7755	8	3	292.49	0.29977	97.62	5.9199	387.4100126532	0.32579	12.187	-17.728	0.59259	0.51656	0.96064 (max o	3	2249; 1559; 1556	-0.2228964	0.20973593		2.0
	ZINC22922292	-8.6	373.479	-2.6047	8	3	293.28	0.30166	96	7.0454	387.4100126532	0.32579	9.0165	-7.9951	0.59259	0.51656	0.91768 (max o	2	2249; 1554	-0.2303011	0.229924		2.0
	ZINC22922292	-8.6	375.495	-7.7533	8	4	281.52	0.27249	98.87	5.9199	387.4100126532	0.32579	14.165	-23.799	0.59259	0.49993	0.86993 (max o	3	2249; 632; 1554	-0.2321795	0.17436647		2.0
	ZINC22922455	-8.6	412.488	-3.8468	8	3	334.49	0.29137	106.01	4.9363	387.4100126532	0.29321	10.259	-13.12	0.63333	0.50706	0.92206 (max o	4	5950; 4339; 2280; 19	0.03581705	0.492989		2.0
	ZINC22923695	-8.6	406.529	-5.0521	9	5	308.23	0.28978	107.25	5.3719	387.4100126532	0.30332	11.464	-16.656	0.58621	0.48462	0.96931 (max o	9	6848; 5955; 5548; 55	-0.1172736	0.54299766		2.0
	ZINC22923695	-8.6	405.521	-3.0743	9	4	319.2	0.31419	106	5.522	387.4100126532	0.30332	9.4861	-10.136	0.58621	0.49809	0.96931 (max o	10	6849; 5956; 5546; 51	-0.0773833	0.51166165		2.0
	ZINC31157183	-8.6	352.382	0.2866	7	2	246.67	0.34589	110.13	-8.7646	387.4100126532	0.35185	6.1252	0.81455	0.44	0.39461	0.97118 (max o	3	6856; 4363; 4364	-0.4786646	0.5429381		2.0
	ZINC33830310	-8.6	388.368	-1.827	10	4	263.15	0.45024	151.98	-6.054	387.4100126532	0.32579	8.2388	-5.608	0.48148	0.37688	0.93686 (max o	3	5288; 4529; 3107	-0.7773347	-0.4132501		2.0
	ZINC35442240	-8.6	380.383	-1.513	9	1	289.45	0.36248	128.04	4.2036	387.4100126532	0.31415	7.9248	-4.8161	0.53571	0.49307	0.95945 (max o	3	1837; 1836; 644	0.292493	-0.1661043		2.0
	ZINC35442604	-8.6	434.555	-0.129	8	1	344.68	0.24661	109.85	5.015	387.4100126532	0.28375	6.5408	-0.45462	0.6129	0.65185	0.94785	1	3440	0.9464561	0.18238665		2.0
	ZINC35455084	-8.6	408.402	-0.1825	9	3	294.59	0.35772	131.75	-0.23288	387.4100126532	0.30332	6.5943	-0.60167	0.55172	0.42312	0.93975 (max o	3	2288; 1914; 1915	0.590497	-0.5555536		2.0
	ZINC36378602	-8.6	360.361	-0.1543	7	4	232.58	0.41332	127.59	0.18236	387.4100126532	0.33832	6.5661	-0.45608	0.42308	0.29718	0.9615 (max of	3	2668; 1925; 845	0.03618423	0.9325753		2.0
	ZINC67903438	-8.6	360.358	-2.0162	9	4	247.63	0.4258	134.91	-2.1618	387.4100126532	0.35185	8.428	-5.7303	0.48	0.50401	0.95318 (max o	3	2686; 2685; 2315	0.60874414	0.62448156		2.0
	ZINC67912723	-8.6	416.465	-1.8823	9	7	262.4	0.43727	167.91	-0.95798	387.4100126532	0.30332	8.2941	-6.2057	0.34483	0.41166	0.94823 (max o	3	3105; 3106; 1941	0.8702943	-0.1465674		2.0
	ZINC67913503	-8.6	364.261	-5.2283	10	3	255.83	0.49635	176.48	-0.72708	387.4100126532	0.33832	11.64	-15.454	0.53846	0.40838	0.93361 (max o	3	2688; 1623; 1621	-0.3739866	-0.8443443		2.0
	ZINC67913498	-8.6	364.261	-5.2283	10	3	255.83	0.49635	176.48	-0.72708	387.4100126532	0.33832	11.64	-15.454	0.53846	0.41098	0.93361 (max o	3	2688; 1623; 1622	-0.3406851	-0.8663551		2.0
	ZINC67913506	-8.6	364.261	-5.2283	10	3	255.83	0.49635	176.48	-0.72708	387.4100126532	0.33832	11.64	-15.454	0.53846	0.40838	0.93361 (max o	3	2688; 1621; 1622	-0.3519755	-0.848328		2.0
	ZINC71404536	-8.6	448.379	-0.0535	11	7	299.19	0.45035	186.37	-3.3789	387.4100126532	0.27488	6.4653	-0.19463	0.53125	0.27454	0.95709 (max o	4	2321; 1944; 202; 677	-0.3155671	-0.657975		2.0
	ZINC72320040	-8.6	441.408	-2.5571	11	4	318.14	0.4173	175.04	-2.4143	387.4100126532	0.28375	8.9689	-9.0118	0.54839	0.51495	0.85745	1	2697	-0.7812425	0.47889253		2.0
	ZINC02131462	-8.6	366.352	-1.3445	8	2	271.29	0.34247	118.64	5.5873	387.4100126532	0.32579	7.7563	-4.1269	0.55556	0.49814	0.97346 (max o	3	2241; 1653; 695	0.16984421	-0.2094191		1.0
	ZINC02146126	-8.6	437.427	-1.3909	9	2	331.19	0.3302	133.86	0.94059	387.4100126532	0.27488	7.8027	-5.06	0.65625	0.52294	0.88201	1	2733	0.1953496	-0.1582915		1.0
	ZINC02479145	-8.6	350.329	0.4208	7	1	243.99	0.29657	94.99	1.1127	387.4100126532	0.33832	5.991	1.2438	0.5	0.46383		0		-0.6953988	0.70667076		1.0
	ZINC03813328	-8.6	394.402	-0.2403	9	2	285.65	0.29522	102.42	2.3229	387.4100126532	0.31415	6.6521	-0.76492	0.64286	0.54461		0		0.94355994	0.24307021		1.0
	ZINC03840112	-8.6	444.510	-2.9173	10	7	328.31	0.38147	174.15	5.2604	387.4100126532	0.27488	9.3291	-10.613	0.53125	0.48483	0.89631 (max o	2	5386; 2006	-0.5205133	-0.1339592		1.0
	ZINC03932652	-8.6	391.430	1.4006	11	5	285.19	0.44644	163.93	-3.8297	387.4100126532	0.31415	5.0112	4.4583	0.57143	0.35828		0		-0.8952506	-0.2815453		1.0
	ZINC04062210	-8.6	374.412	0.3524	7	3	264.03	0.33379	122.41	1.9657	387.4100126532	0.32579	6.0594	1.0817	0.44444	0.28507	0.94302 (max o	3	2781; 2032; 1398	-0.8937048	0.19131489		1.0
	ZINC04062211	-8.6	374.412	0.3524	7	3	264.03	0.33379	122.41	1.9657	387.4100126532	0.32579	6.0594	1.0817	0.44444	0.28507	0.94905 (max o	3	2781; 2032; 1397	-0.8897129	0.16619131		1.0
	ZINC04098003	-8.6	424.401	-0.4739	10	5	286.21	0.41417	155.14	-6.2493	387.4100126532	0.29321	6.8857	-1.6163	0.4	0.39527		0		-0.4068462	0.8561197		1.0
	ZINC04222668	-8.6	374.455	0.3431	6	0	291.45	0.21928	78.9	3.58	387.4100126532	0.32579	6.0687	1.0531	0.55556	0.4406	0.89979	1	2201	0.44263253	-0.7878223		1.0
	ZINC04259456	-8.6	403.394	0.7133	11	1	288.27	0.39643	118.85	-14.152	387.4100126532	0.30332	5.6985	2.3516	0.58621	0.31752		0		0.7219354	0.69155455		1.0
	ZINC04270964	-8.6	396.446	0.481	8	1	295.09	0.25263	83.88	8.5596	387.4100126532	0.30332	5.9308	1.5858	0.58621	0.45596	0.89979 (max o	5	3662; 3491; 84; 268;	-0.2709904	0.02271078		1.0
	ZINC05412480	-8.6	427.591	-1.4045	7	2	343.68	0.21572	70.22	-7.8978	387.4100126532	0.28375	7.8163	-4.9498	0.64516	0.4712	0.8872 (max of	3	4568; 1434; 1184	-0.1107549	-0.2063699		1.0

Structure	Molecule Name	affinity	Total Molweight	cLogP	H-Acceptors	H-Donors	Total Surface Area	Relative PSA	Polar Surface Area	Druglikeness	Kd (nM) @ 20°C	LE from K...	LLE from Kd ...	LELP from Kd (n...	Shape Index	Molecular...	Neighbor Si...	Neig...	Neighbor	Neighbor ...	Neighbor ...	Ring Systems	frequency
	ZINC05412549	-8.6	411.504	-2.5481	8	2	325.33	0.27126	83.36	-3.1108	387.4100126532	0.29321	8.9599	-8.6904	0.63333	0.45022	0.8872 (max of	4	4568; 751; 1184; 143.	-0.0987894	-0.2412492		1.0
	ZINC05413794	-8.6	445.478	-1.7992	12	2	327.7	0.35908	137.07	4.265	387.4100126532	0.27488	8.211	-6.5453	0.4375	0.50309	0.96186 (max o	5	3699; 1748; 961; 117(	-0.0783598	0.25311446		1.0
	ZINC05413851	-8.6	443.526	-1.0413	10	3	328.88	0.30677	128.32	4.8256	387.4100126532	0.27488	7.4531	-3.7882	0.4375	0.4959	0.93157 (max o	5	1939; 88; 599; 753; 1	0.02124620	0.28589827		1.0
	ZINC06137875	-8.6	408.497	-0.4144	9	2	312.4	0.29507	108.05	0.92361	387.4100126532	0.30332	6.8262	-1.3662	0.48276	0.51681	0.9426 (max of	3	4574; 3819; 2471	-0.1276070	0.2212038		1.0
	ZINC06624453	-8.6	366.352	-1.6576	8	2	272.55	0.34089	118.64	1.4218	387.4100126532	0.32579	8.0694	-5.088	0.51852	0.54557		0		0.05554693	-0.9850847		1.0
	ZINC08253947	-8.6	383.474	-1.9865	8	1	301.73	0.27826	77.58	0.34247	387.4100126532	0.31415	8.3983	-6.3234	0.60714	0.47903	0.97037	1	2854	0.2970263	-0.8941511		1.0
	ZINC08296733	-8.6	428.444	-1.8889	9	2	303.75	0.40583	156.11	6.3201	387.4100126532	0.29321	8.3007	-6.4422	0.56667	0.34742	0.93405 (max o	6	3272; 1672; 57; 464; !	-0.2441525	0.41770312		1.0
	ZINC08299103	-8.6	424.591	-2.2761	7	2	357.09	0.22566	59.9	1.1942	387.4100126532	0.28375	8.6879	-8.0215	0.6129	0.5437	0.97423 (max o	6	4152; 3278; 3274; 32	0.44416064	-0.0678675		1.0
	ZINC08299255	-8.6	440.590	-3.0981	8	2	367.09	0.24675	69.13	0.32375	387.4100126532	0.27488	9.5099	-11.271	0.625	0.56297	0.97533 (max o	5	4152; 3278; 3274; 21(	0.42880794	-0.0860193		1.0
	ZINC08636488	-8.6	419.501	1.652	8	3	290.75	0.35512	128.92	4.1777	387.4100126532	0.30332	4.7598	5.4464	0.51724	0.38337	0.97415 (max o	3	3736; 1768; 763	-0.5163019	0.31416076		1.0
	ZINC08739977	-8.6	413.433	0.3389	10	4	291.34	0.36624	132.44	3.7769	387.4100126532	0.29321	6.0729	1.1558	0.46667	0.36277	0.9707 (max of	4	2891; 1775; 935; 107	0.3043078	-0.4808570		1.0
	ZINC08764885	-8.6	425.444	0.6655	10	3	308.43	0.34423	123.84	4.1796	387.4100126532	0.28375	5.7463	2.3454	0.54839	0.49774		0		0.9249647	-0.2731437		1.0
	ZINC12483435	-8.6	429.535	-0.1176	7	3	318.03	0.22514	91.91	5.1931	387.4100126532	0.28375	6.5294	-0.41445	0.6129	0.46427	0.89631 (max o	5	5049; 4727; 4731; 29(	0.4023276	0.01807493		1.0
	ZINC12483444	-8.6	410.536	0.2901	6	2	306.99	0.18033	71.43	4.2542	387.4100126532	0.29321	6.1217	0.9894	0.6	0.45763	0.97533 (max o	4	6216; 5049; 2986; 14(	0.42660752	-0.0130486		1.0
	ZINC12530057	-8.6	388.379	-1.978	10	2	298.12	0.42084	131.05	2.3705	387.4100126532	0.31415	8.3898	-6.2963	0.60714	0.40671	0.89839 (max o	3	3327; 2918; 2149	0.64956725	-0.3255119		1.0
	ZINC12530331	-8.6	435.479	-1.9155	8	1	321.66	0.22005	97.97	1.353	387.4100126532	0.27488	8.3273	-6.9684	0.5625	0.48683	0.87803 (max o	3	6638; 6540; 4265	-0.2246787	0.4257163		1.0
	ZINC12530331	-8.6	436.486	-1.7839	8	2	335.7	0.25267	99.17	0.4902	387.4100126532	0.27488	8.1957	-6.4897	0.5625	0.47433	0.87803	1	6542	0.10524972	-0.9644677		1.0
	ZINC12603640	-8.6	385.459	-0.66	9	3	285.62	0.31804	111.57	2.5827	387.4100126532	0.32579	7.0718	-2.0259	0.62963	0.5979	0.92394 (max o	4	4603; 3775; 3742; 29.	0.1898743	-0.4565014		1.0
	ZINC13349051	-8.6	416.405	-1.5687	9	3	306.73	0.35217	137.38	-12.421	387.4100126532	0.29321	7.9805	-5.3501	0.53333	0.43395	0.96092 (max o	3	7005; 5870; 968	0.8774992	0.17351481		1.0
	ZINC20112085	-8.6	379.519	-0.8686	6	1	281.4	0.22026	67.91	-1.6832	387.4100126532	0.32579	7.2804	-2.6662	0.55556	0.47182	0.94302 (max o	4	4088; 3831; 3832; 18!	0.09683349	0.5580811		1.0
	ZINC20463190	-8.6	427.543	-1.2613	7	1	330.55	0.23624	85.19	4.6615	387.4100126532	0.28375	7.6731	-4.4451	0.48387	0.53044	0.92584 (max o	2	5849; 2519	0.27763972	0.83251035		1.0
	ZINC20464776	-8.6	399.513	-1.3682	7	3	302.14	0.20524	85.67	3.9028	387.4100126532	0.30332	7.78	-4.5108	0.58621	0.43413	0.96682 (max o	2	4299; 1863	-0.4155721	0.23483089		1.0
	ZINC20504022	-8.6	436.575	-0.6253	9	2	343.92	0.24546	96.68	5.4007	387.4100126532	0.28375	7.0371	-2.2037	0.64516	0.61325	0.86621 (max o	2	5160; 1004	-0.0159730	0.8625926		1.0
	ZINC22922292	-8.6	374.487	-5.6436	8	3	267.48	0.2343	97.67	6.6191	387.4100126532	0.32579	12.055	-17.323	0.59259	0.4771	0.86993 (max o	2	2250; 1557	-0.6258706	-0.7409873		1.0
	ZINC22922292	-8.6	373.479	-3.6658	8	2	278.45	0.26446	96.42	6.6191	387.4100126532	0.32579	10.078	-11.252	0.59259	0.49432	0.96064 (max o	2	2250; 1555	-0.6067523	-0.7573813		1.0
	ZINC22922292	-8.6	373.479	-6.7521	8	3	283.6	0.27782	103.57	6.0413	387.4100126532	0.32579	13.164	-20.725	0.59259	0.49145		0		0.8397437	-0.2685184		1.0
	ZINC22936927	-8.6	408.524	-0.9413	7	3	316.41	0.21226	82.98	5.03	387.4100126532	0.29321	7.3531	-3.2103	0.6	0.42043	0.93405 (max o	2	173; 1019	0.34401166	-0.8307411		1.0
	ZINC28540146	-8.6	434.352	0.1632	11	7	284.9	0.47294	186.37	-1.6541	387.4100126532	0.28375	6.2486	0.57515	0.41935	0.38107	0.90289 (max o	8	2776; 2566; 2565; 24(	-0.6710919	0.29856133		1.0
	ZINC31155317	-8.6	378.332	-0.7725	9	5	250.38	0.4335	145.91	-4.1057	387.4100126532	0.32579	7.1843	-2.3712	0.44444	0.20674	0.81947	1	7072	-0.8797635	-0.4028637		1.0
	ZINC31155354	-8.6	448.419	-2.0505	12	6	308.9	0.44869	176.76	-4.801	387.4100126532	0.28375	8.4623	-7.2264	0.54839	0.45753	0.96018 (max o	5	4359; 2624; 2286; 18!	0.55463	0.4735771		1.0
	ZINC31157245	-8.6	422.428	0.7646	9	6	303.66	0.35764	149.07	-5.5121	387.4100126532	0.29321	5.6472	2.6077	0.53333	0.52875	0.90776	1	1132	-0.5234803	0.7273763		1.0
	ZINC31167772	-8.6	388.368	-2.0291	10	6	263.55	0.45001	158.3	0.28317	387.4100126532	0.32579	8.4409	-6.2283	0.55556	0.43398	0.93686 (max o	3	309; 505; 825	-0.3742422	0.4952799		1.0
	ZINC35271095	-8.6	443.467	-3.2526	10	5	300.11	0.40925	168.97	-2.3795	387.4100126532	0.28375	9.6644	-11.463	0.48387	0.42633	0.92863	1	833	-0.8068687	-0.5374622		1.0
	ZINC35442289	-8.6	394.497	-0.3789	7	3	301.3	0.23651	90.94	4.1975	387.4100126532	0.30332	6.7907	-1.2492	0.62069	0.42524	0.94251 (max o	6	3064; 1911; 319; 159	-0.4704220	0.05054507		1.0
	ZINC35442336	-8.6	405.517	-1.9685	8	3	321.82	0.27792	90.46	7.0535	387.4100126532	0.30332	8.3803	-6.4899	0.58621	0.46467	0.90776 (max o	5	4404; 4402; 3023; 12(	4.5413146E	0.4879346		1.0
	ZINC35442496	-8.6	401.533	-1.8989	8	2	319.58	0.24016	73.22	9.9414	387.4100126532	0.30332	8.3107	-6.2604	0.58621	0.5149	0.87803	1	1601	-0.2269307	0.5011098		1.0
	ZINC67912706	-8.6	366.408	-0.5383	7	4	237.47	0.37259	124.29	0.78517	387.4100126532	0.33832	6.9501	-1.5911	0.34615	0.32626	0.92702 (max o	3	3542; 2316; 1066	0.34378165	0.63998824		1.0
	ZINC26021381	-8.6	427.568	-0.6576	10	6	341.77	0.34257	170.38	-4.543	387.4100126532	0.29321	7.0694	-2.2428	0.53333	0.64816	0.9707 (max of	4	7686; 7472; 7149; 15(	-0.3598564	0.64236104		0.0
	ZINC26021367	-8.6	427.568	-0.6576	10	6	341.77	0.34257	170.38	-4.543	387.4100126532	0.29321	7.0694	-2.2428	0.53333	0.64685	0.9707 (max of	5	7686; 7472; 7149; 69(	-0.3674850	0.6738943		0.0
	ZINC02092071	-8.5	375.356	-2.4889	9	2	282.19	0.38754	133.86	-1.7607	459.9676358652	0.322	8.8262	-7.7295	0.59259	0.47442	0.91804 (max o	6	4002; 3133; 547; 863	0.1607119	0.03631067		172.0
	ZINC02112807	-8.5	417.436	-1.3464	9	2	319.45	0.34234	133.86	3.1293	459.9676358652	0.2898	7.6837	-4.646	0.6	0.57784	0.9561 (max of	6	3155; 3145; 3143; 21	0.17828828	-0.0668685		172.0
	ZINC02109729	-8.5	395.774	-2	9	3	283.2	0.3971	144.86	0.19343	459.9676358652	0.322	8.3373	-6.2112	0.62963	0.57302	0.96106 (max o	6	6390; 3565; 3135; 27	0.11353323	0.11405638		172.0
	ZINC02106053	-8.5	361.305	-4.4409	9	1	268.03	0.41854	144.89	-3.5627	459.9676358652	0.33438	10.778	-13.281	0.57692	0.53227	0.97269 (max o	8	5294; 4009; 4006; 39	0.23428673	0.13941212		172.0

Structure	Molecule Name	affinity	Total Molweight	cLogP	H-Acceptors	H-Donors	Total Surface Area	Relative PSA	Polar Surface Area	Druglikeness	Kd (nM) @ 20°C	LE from K...	LLE from Kd ...	LELP from Kd (n...	Shape Index	Molecular...	Neighbor Si...	Neig...	Neighbor	Neighbor ...	Neighbor ...	Ring Systems	frequency
	ZINC02112763	-8.5	432.408	-3.1784	11	4	323.01	0.42401	173.96	0.1125	459.9676358652	0.28045	9.5157	-11.333	0.64516	0.60885	0.96682 (max o	4	7457; 5708; 2738; 19	0.13988362	0.06091106		172.0
	ZINC03869425	-8.5	425.270	-8.4777	15	3	262.23	0.70705	253.29	-42.416	459.9676358652	0.322	14.815	-26.328	0.51852	0.48752	0.93996 (max o	7	3284; 3283; 3173; 31	-0.6168473	0.39291805		67.0
	ZINC12360703	-8.5	424.178	-11.354	15	3	264.95	0.71006	260.71	-42.337	459.9676358652	0.322	17.692	-35.262	0.51852	0.48526	0.97407 (max o	12	6792; 3360; 3359; 33	-0.5954253	0.44827223		67.0
	ZINC12360703	-8.5	425.186	-10.107	15	4	263.77	0.70876	257.88	-47.067	459.9676358652	0.322	16.444	-31.387	0.51852	0.48546	0.97407 (max o	14	6792; 3360; 3359; 33	-0.5674476	0.4161705		67.0
	ZINC04228232	-8.5	384.416	-3.7275	11	4	265.34	0.56735	212.38	-0.44329	459.9676358652	0.33438	10.065	-11.147	0.57692	0.48113	0.93686 (max o	7	5728; 5096; 3674; 36	0.59731096	0.19285004		67.0
	ZINC04887467	-8.5	351.318	-0.8696	11	2	249.96	0.50088	147.13	0.815	459.9676358652	0.34776	7.2069	-2.5006	0.48	0.37337	0.93686 (max o	3	3247; 2458; 1966	0.11343057	-0.6655916		44.0
	ZINC12889496	-8.5	418.429	-3.1207	11	4	312.54	0.42452	173.76	8.6998	459.9676358652	0.2898	9.458	-10.768	0.46667	0.56024	0.97757 (max o	12	5077; 3785; 3347; 29	0.03450342	-0.5973567		42.0
	ZINC12889917	-8.5	418.405	-4.7989	11	3	311.55	0.42269	170.8	8.0771	459.9676358652	0.2898	11.136	-16.559	0.46667	0.56864	0.97757 (max o	15	4330; 3785; 3750; 33	0.07138522	-0.6337244		42.0
	ZINC12889905	-8.5	418.405	-4.7989	11	3	311.55	0.42269	170.8	5.9994	459.9676358652	0.2898	11.136	-16.559	0.5	0.55962	0.97533 (max o	8	4330; 3823; 3750; 29	0.09132691	-0.6382385		42.0
	ZINC13533762	-8.5	449.117	-12.741	15	2	276.79	0.67954	256.38	-43.635	459.9676358652	0.322	19.078	-39.568	0.59259	0.68331		0		-0.9624553	-0.1531099		41.0
	ZINC08791253	-8.5	422.460	0.0941	8	1	318.15	0.26717	109.85	4.4481	459.9676358652	0.28045	6.2432	0.33553	0.3871	0.50513	0.96018 (max o	11	5034; 4962; 4522; 40	0.6846361	-0.0115146		36.0
	ZINC12889392	-8.5	374.372	-2.9733	9	2	268	0.36604	130.08	4.9085	459.9676358652	0.322	9.3106	-9.2339	0.51852	0.49614	0.98806 (max o	15	3642; 3345; 3210; 32	0.13372707	-0.8632988		29.0
	ZINC04090580	-8.5	374.372	-2.9733	9	2	268	0.36604	130.08	4.9085	459.9676358652	0.322	9.3106	-9.2339	0.51852	0.50041	0.98806 (max o	13	4091; 3345; 3210; 32	0.15086176	-0.8731411		29.0
	ZINC01532555	-8.5	361.206	-8.4657	13	4	230.21	0.69893	217.22	-28.905	459.9676358652	0.36225	14.803	-23.37	0.5	0.43529	0.93686 (max o	5	4933; 4932; 3175; 29	-0.3477539	-0.3778017		26.0
	ZINC03947475	-8.5	353.302	-2.8445	9	5	246.47	0.47008	167.58	0.33063	459.9676358652	0.34776	9.1818	-8.1795	0.56	0.41449	0.97396 (max o	13	3467; 3051; 2623; 24	-0.1592737	-0.8207593		25.0
	ZINC01532490	-8.5	353.302	-2.8445	9	5	246.47	0.47008	167.58	0.33063	459.9676358652	0.34776	9.1818	-8.1795	0.56	0.41449	0.98188 (max o	13	3467; 3051; 2623; 24	-0.193965	-0.8340205		25.0
	ZINC05567588	-8.5	353.302	-2.8445	9	5	246.47	0.47008	167.58	0.33063	459.9676358652	0.34776	9.1818	-8.1795	0.56	0.41449	0.98188 (max o	18	5090; 3467; 3158; 30	-0.1926338	-0.8010534		25.0
	ZINC28538287	-8.5	375.349	-4.4459	10	5	248.69	0.49387	168.97	-2.076	459.9676358652	0.33438	10.783	-13.296	0.46154	0.37855	0.91007 (max o	3	3216; 1700; 1890	-0.6180765	0.05330667		25.0
	ZINC28538287	-8.5	376.357	-2.3699	10	6	247.51	0.49145	166.14	-2.2374	459.9676358652	0.33438	8.7072	-7.0874	0.46154	0.37696	0.94927 (max o	3	3708; 2626; 1891	-0.615058	0.03274329		25.0
	ZINC04099189	-8.5	359.350	-3.6225	9	4	244.43	0.44888	148.74	-3.4303	459.9676358652	0.34776	9.9598	-10.417	0.48	0.38183	0.95 (max of 11	11	6291; 5674; 3707; 34	-0.6397565	0.07842827		25.0
	ZINC08964847	-8.5	445.435	1.6712	13	5	321.67	0.45798	182.39	1.4288	459.9676358652	0.27169	4.6661	6.1512	0.5	0.3389	0.95739 (max o	7	4624; 3757; 3756; 37	-0.1673560	-0.6422761		24.0
	ZINC02123222	-8.5	390.435	-1.0153	7	1	284.73	0.35946	130.06	0.69757	459.9676358652	0.322	7.3526	-3.1531	0.55556	0.38488	0.89081 (max o	4	4023; 4012; 187; 341	0.17310002	0.18940729		24.0
	ZINC08791135	-8.5	426.400	-0.8918	9	2	316.1	0.34154	134.22	2.5282	459.9676358652	0.28045	7.2291	-3.1799	0.58065	0.43588	0.99016 (max o	14	4090; 2934; 2537; 25	0.5325849	0.40186822		24.0
	ZINC12883147	-8.5	426.400	-0.8918	9	2	316.1	0.34154	134.22	2.5282	459.9676358652	0.28045	7.2291	-3.1799	0.58065	0.43741	0.99016 (max o	14	4090; 2934; 2537; 25	0.5337428	0.4452039		24.0
	ZINC12883139	-8.5	426.400	-0.8918	9	2	316.1	0.34154	134.22	2.5282	459.9676358652	0.28045	7.2291	-3.1799	0.58065	0.4352	0.99016 (max o	14	4090; 2934; 2537; 25	0.55191314	0.43311793		24.0
	ZINC04084677	-8.5	382.347	-0.6837	8	1	286.25	0.33139	113.99	0.53295	459.9676358652	0.3105	7.021	-2.2019	0.64286	0.41692	0.9543 (max of	13	4090; 2788; 2537; 25	0.5527226	0.395591		24.0
	ZINC12881038	-8.5	426.400	-0.8918	9	2	316.1	0.34154	134.22	2.5282	459.9676358652	0.28045	7.2291	-3.1799	0.6129	0.43741	0.99016 (max o	12	3343; 3204; 2934; 27	0.5146641	0.43917167		24.0
	ZINC09059034	-8.5	442.399	-1.3211	10	2	327.36	0.36034	143.45	4.7361	459.9676358652	0.27169	7.6584	-4.8626	0.59375	0.41307	0.9665 (max of	6	4090; 3307; 3204; 29	0.58001214	0.375077		24.0
	ZINC04073886	-8.5	351.286	-3.185	9	3	237.81	0.46112	145.58	-4.8874	459.9676358652	0.34776	9.5223	-9.1586	0.48	0.39772	0.89805 (max o	4	2015; 585; 747; 923	-0.9076066	-0.3810960		24.0
	ZINC04282223	-8.5	379.364	-0.749	9	4	267.76	0.39901	134.55	-8.6166	459.9676358652	0.322	7.0863	-2.3261	0.44444	0.40514	0.95201 (max o	3	1822; 1401; 1635	0.3449071	0.3413186		24.0
	ZINC06623670	-8.5	386.783	0.301	8	4	261.82	0.36453	125.68	-5.3756	459.9676358652	0.33438	6.0363	0.90016	0.5	0.31084	0.89253 (max o	3	2782; 710; 1400	0.38682917	0.5149548		24.0
	ZINC13507421	-8.5	406.394	-2.6337	11	6	283.25	0.45758	175.28	6.4914	459.9676358652	0.29979	8.971	-8.7851	0.44828	0.51372	0.96428 (max o	15	4912; 4908; 4896; 41	0.48119694	0.7423531		24.0
	ZINC03831422	-8.5	376.368	-2.0663	10	5	264.4	0.44066	155.05	6.1929	459.9676358652	0.322	8.4036	-6.4171	0.44444	0.48374	0.97288 (max o	20	5875; 5314; 5297; 49	0.44708785	0.80933154		24.0
	ZINC16052277	-8.5	444.439	-4.6138	10	6	307.33	0.45118	185.65	2.419	459.9676358652	0.27169	10.951	-16.982	0.375	0.36573	0.96186 (max o	5	2184; 8; 65; 400; 131	-0.7218909	-0.5278365		22.0
	ZINC16052277	-8.5	443.431	-3.2487	10	6	293.68	0.42567	185.44	3.8301	459.9676358652	0.27169	9.586	-11.958	0.375	0.35855	0.98148 (max o	6	2186; 9; 67; 402; 131	-0.1151285	0.6974478		22.0
	ZINC16052277	-8.5	443.431	-3.2475	10	5	293.29	0.4249	184.45	4.6853	459.9676358652	0.27169	9.5848	-11.953	0.375	0.36418	0.98148 (max o	6	2186; 9; 67; 402; 131	-0.0942853	0.726545		22.0
	ZINC02103773	-8.5	358.369	-0.5505	7	0	266.08	0.2892	95.97	2.3158	459.9676358652	0.33438	6.8878	-1.6463	0.53846	0.54816	0.95257 (max o	5	2850; 2484; 866; 876	0.34677172	0.12983146		22.0
	ZINC08764928	-8.5	372.396	-0.262	7	0	278.34	0.27646	95.97	4.3706	459.9676358652	0.322	6.5993	-0.81367	0.51852	0.53029	0.97149 (max o	7	2129; 2122; 2036; 19	0.2858017	0.22705732		22.0
	ZINC08764400	-8.5	388.395	-0.6759	8	0	288.34	0.30155	105.2	4.3706	459.9676358652	0.3105	7.0132	-2.1768	0.53571	0.53237	0.97288 (max o	6	2484; 2408; 2122; 54	0.31283987	0.18061832		22.0
	ZINC08789949	-8.5	432.451	-1.866	10	2	322.59	0.35001	137.1	-0.15231	459.9676358652	0.28045	8.2033	-6.6536	0.58065	0.48429	0.9702 (max of	7	4087; 3342; 3202; 26	0.52680266	0.2518036		22.0
	ZINC08995728	-8.5	446.478	-1.5067	10	2	333.59	0.33847	137.1	-0.24237	459.9676358652	0.27169	7.844	-5.5457	0.53125	0.46794	0.97948 (max o	11	6226; 5250; 4194; 41	0.51047426	0.16597085		22.0
	ZINC04085001	-8.5	418.425	-2.3204	10	2	308.83	0.36561	137.1	-2.5595	459.9676358652	0.2898	8.6577	-8.0069	0.56667	0.46386	0.9702 (max of	8	4521; 4195; 3342; 32	0.5077141	0.26253346		22.0
	ZINC04416344	-8.5	448.379	0.5798	11	7	295.9	0.45536	186.37	1.9289	459.9676358652	0.27169	5.7575	2.1341	0.40625	0.42191	0.95949 (max o	14	4095; 2776; 2566; 25	-0.7393936	0.26866558		21.0

Structure	Molecule Name	affinity	Total Molweight	cLogP	H-Acceptors	H-Donors	Total Surface Area	Relative PSA	Polar Surface Area	Druglikeness	Kd (nM) @ 20°C	LE from K...	LLE from Kd ...	LELP from Kd (n...	Shape Index	Molecular...	Neighbor Si...	Neig...	Neighbor	Neighbor ...	Neighbor ...	Ring Systems	frequency
	ZINC20465486	-8.5	418.478	-0.2255	6	3	296.34	0.17223	72.78	-3.242	459.9676358652	0.29979	6.5628	-0.75219	0.51724	0.51236	0.93069 (max o	5	6258; 5565; 4310; 22	-0.4353565	0.11714224		21.0
	ZINC17130824	-8.5	431.440	-0.1614	9	4	308.3	0.35641	142.39	0.6376	459.9676358652	0.28045	6.4987	-0.5755	0.51613	0.44611	0.97199 (max o	8	2967; 2564; 2563; 22	-0.3224459	-0.7541267		19.0
	ZINC17130822	-8.5	431.440	-0.1614	9	4	308.3	0.35641	142.39	0.6376	459.9676358652	0.28045	6.4987	-0.5755	0.51613	0.44854	0.97199 (max o	8	2967; 2564; 2563; 24	-0.2897754	-0.773753		19.0
	ZINC31161557	-8.5	431.440	-0.1614	9	4	308.3	0.35641	142.39	0.6376	459.9676358652	0.28045	6.4987	-0.5755	0.51613	0.46025	0.97199 (max o	8	4577; 2967; 2564; 25	-0.3112175	-0.7717140		19.0
	ZINC02122913	-8.5	383.335	-1.8591	9	2	282.25	0.38746	133.86	-0.2732	459.9676358652	0.3105	8.1964	-5.9875	0.64286	0.49632	0.9218	1	1358	0.6209919	-0.6081105		16.0
	ZINC13136667	-8.5	432.380	-0.078	10	7	289.19	0.43134	177.14	-1.9977	459.9676358652	0.28045	6.4153	-0.27812	0.51613	0.3816	0.97642 (max o	12	3788; 3213; 2549; 24	0.7173941	0.09082319		14.0
	ZINC20610524	-8.5	419.416	-0.8975	9	3	306.46	0.34882	134.43	4.8339	459.9676358652	0.28045	7.2348	-3.2002	0.41935	0.45825	0.96971 (max o	11	2540; 2534; 2161; 28	0.6831784	-0.1456804		14.0
	ZINC02131876	-8.5	449.442	-1.4649	10	4	325.31	0.36888	154.66	4.2777	459.9676358652	0.26345	7.8022	-5.5604	0.39394	0.46516	0.99099 (max o	11	2540; 2534; 2161; 18	0.7240219	-0.1463260		14.0
	ZINC05434131	-8.5	402.449	0.1141	9	2	302.86	0.30437	108.05	5.2959	459.9676358652	0.29979	6.2232	0.3806	0.55172	0.53702	0.94241 (max o	6	5426; 2469; 2088; 93	0.08955022	0.28880116		14.0
	ZINC02104537	-8.5	415.421	-1.7388	9	2	310.21	0.35254	133.86	-3.3392	459.9676358652	0.2898	8.0761	-6	0.6	0.45961	0.97304 (max o	6	4004; 3563; 3562; 23	0.2917341	0.00871470		14.0
	ZINC02104030	-8.5	401.394	-2.0827	9	2	297.95	0.36704	133.86	-3.3392	459.9676358652	0.29979	8.42	-6.9471	0.62069	0.52544	0.96931 (max o	4	5301; 3563; 3561; 86	0.27224034	0.00929476		14.0
	ZINC03871878	-8.5	376.388	-3.4633	8	1	256.5	0.4414	154.97	10.967	459.9676358652	0.33438	9.8006	-10.357	0.5	0.35316	0.91351 (max o	3	3178; 2391; 706	0.1020983	-0.0660776		12.0
	ZINC04151681	-8.5	447.423	-4.1492	12	6	326.13	0.47628	210.98	5.4075	459.9676358652	0.27169	10.486	-15.272	0.5625	0.55655	0.94751 (max o	6	2745; 2429; 2053; 17	-0.1774081	-0.5731917		12.0
	ZINC03838459	-8.5	390.395	-1.6106	10	6	288.35	0.44394	170.85	5.0781	459.9676358652	0.3105	7.9479	-5.1871	0.60714	0.55336	0.9673 (max of	5	3602; 2757; 2745; 17	-0.1978047	-0.5619416		12.0
	ZINC04221821	-8.5	423.376	-3.9196	11	6	302.47	0.47043	193.91	4.074	459.9676358652	0.2898	10.257	-13.525	0.53333	0.54139	0.93686 (max o	8	3607; 3606; 2757; 24	-0.2186119	-0.5547414		12.0
	ZINC08918470	-8.5	432.495	-0.7109	9	2	336.26	0.30604	127.87	-45.685	459.9676358652	0.28045	7.0482	-2.5348	0.6129	0.56322	0.9499 (max of	2	3310; 1498	0.40375906	-0.0172083		12.0
	ZINC08624545	-8.5	407.465	-0.952	9	3	305.1	0.29774	111.57	5.7431	459.9676358652	0.29979	7.2893	-3.1755	0.65517	0.56001	0.94529 (max o	6	2109; 1829; 106; 466	-0.0799182	-0.5744962		11.0
	ZINC04237201	-8.5	427.500	-1.0185	7	0	310.87	0.28304	115.28	3.6816	459.9676358652	0.2898	7.3558	-3.5145	0.63333	0.47419	0.97304 (max o	5	4972; 2436; 2057; 11	-0.517103	-0.3067974		10.0
	ZINC20464478	-8.5	423.464	-0.1223	10	6	317.67	0.38656	157.22	-8.5831	459.9676358652	0.2898	6.4596	-0.42202	0.63333	0.53042	0.94804 (max o	5	190; 885; 1105; 1106	-0.457304	-0.3881312		10.0
	ZINC03838732	-8.5	369.348	-2.0938	9	6	259.04	0.45016	161.98	1.4781	459.9676358652	0.33438	8.4311	-6.2617	0.53846	0.48649	0.92368 (max o	4	2373; 884; 1104; 166	-0.3959645	-0.4907170		10.0
	ZINC03838734	-8.5	365.385	-1.8507	9	6	264.95	0.44012	161.98	2.8025	459.9676358652	0.33438	8.188	-5.5347	0.57692	0.48649	0.93046 (max o	4	2373; 1673; 1104; 16	-0.4114369	-0.4745423		10.0
	ZINC03839674	-8.5	379.411	-1.4914	9	6	275.95	0.42258	161.98	1.6466	459.9676358652	0.322	7.8287	-4.6317	0.55556	0.4664	0.91731 (max o	4	4098; 1105; 1381; 16	-0.4247117	-0.4383192		10.0
	ZINC04349749	-8.5	432.380	-0.078	10	7	289.19	0.43134	177.14	-1.9977	459.9676358652	0.28045	6.4153	-0.27812	0.45161	0.3811	0.95771 (max o	7	4107; 3226; 2806; 25	0.77104354	0.11354821		10.0
	ZINC04084683	-8.5	376.412	-2.0596	8	2	258.75	0.44139	157.93	8.1327	459.9676358652	0.33438	8.3969	-6.1594	0.46154	0.3001	0.92315 (max o	8	6409; 4523; 4084; 40	0.310515	-0.6597124		10.0
	ZINC05397431	-8.5	402.386	-2.3885	11	1	293.29	0.41461	140.52	1.1114	459.9676358652	0.29979	8.7258	-7.9672	0.58621	0.36752	0.9561 (max of	6	3252; 2815; 2445; 24	-0.2262160	-0.3168776		10.0
	ZINC08635776	-8.5	404.489	-2.3167	9	1	315.73	0.31378	98.83	-0.67211	459.9676358652	0.29979	8.654	-7.7277	0.62069	0.51813	0.97176	1	4177	0.7556345	0.30263042		10.0
	ZINC35442252	-8.5	377.482	-0.2743	6	3	286.15	0.21069	78.05	3.9566	459.9676358652	0.322	6.6116	-0.85187	0.62963	0.46071	0.93686 (max o	6	3436; 3065; 3064; 30	-0.5035919	0.05313187		10.0
	ZINC04235945	-8.5	409.465	-0.5495	8	2	301.13	0.25046	95.56	5.938	459.9676358652	0.2898	6.8868	-1.8961	0.6	0.37958	0.93686 (max o	5	4099; 3659; 2054; 20	-0.2232109	0.26539406		9.0
	ZINC20464542	-8.5	445.582	-0.3273	8	2	340.18	0.21277	87.72	6.0083	459.9676358652	0.27169	6.6646	-1.2047	0.625	0.47445	0.94215 (max o	7	2279; 649; 1267; 130	-0.5469109	0.13954593		9.0
	ZINC08662731	-8.5	446.407	-0.0864	10	5	308.75	0.38394	155.14	-3.4398	459.9676358652	0.27169	6.4237	-0.31801	0.5625	0.43858	0.98343 (max o	11	2890; 2847; 1994; 19	0.24026819	0.7000978		9.0
	ZINC08662733	-8.5	446.407	-0.0864	10	5	308.75	0.38394	155.14	-3.4398	459.9676358652	0.27169	6.4237	-0.31801	0.5625	0.43858	0.98343 (max o	12	3558; 2890; 1994; 43	0.25539216	0.6850442		9.0
	ZINC04136964	-8.5	432.380	-0.3621	10	6	292.84	0.41538	166.14	-3.6206	459.9676358652	0.28045	6.6994	-1.2911	0.54839	0.42864	0.96262 (max o	13	3558; 2847; 2138; 21	0.16597375	0.66553324		9.0
	ZINC04098622	-8.5	406.386	0.4945	9	7	286.73	0.38957	160.07	-4.3466	459.9676358652	0.29979	5.8428	1.6495	0.55172	0.42583	0.9561 (max of	9	3902; 3899; 3901; 33	-0.5681904	0.76198226		9.0
	ZINC11851962	-8.5	420.413	0.222	9	5	301.3	0.36024	145.91	-3.6574	459.9676358652	0.2898	6.1153	0.76605	0.6	0.47511	0.95837 (max o	7	2909; 2517; 2358; 20	0.3568036	0.5478253		9.0
	ZINC31166887	-8.5	449.431	-1.8552	10	5	321.33	0.38222	168.97	-3.6061	459.9676358652	0.27169	8.1925	-6.8285	0.5625	0.48203	0.971	1	3911	0.23139487	0.9618606		9.0
	ZINC04259912	-8.5	404.358	-3.1977	12	1	291.03	0.45219	149.75	0.47667	459.9676358652	0.29979	9.535	-10.666	0.62069	0.37888	0.96682 (max o	4	3649; 2809; 1416; 14	-0.2350953	-0.3988685		9.0
	ZINC12605286	-8.5	390.414	0.2849	8	2	286.95	0.28639	98.82	3.9439	459.9676358652	0.3105	6.0524	0.91755	0.46429	0.52435	0.95983 (max o	6	2931; 2861; 2835; 20	0.05710733	0.30907616		9.0
	ZINC05414576	-8.5	426.468	-0.1532	9	2	297.78	0.32816	124.27	2.5213	459.9676358652	0.29979	6.4905	-0.51102	0.44828	0.52664	0.91292 (max o	3	3256; 2861; 1801	0.03977491	0.34121612		9.0
	ZINC08297692	-8.5	370.499	-3.4585	7	3	300.11	0.26204	80.86	0.35716	459.9676358652	0.322	9.7958	-10.741	0.66667	0.50523	0.96885 (max o	10	5009; 4588; 4153; 41	-0.13086	0.03018457		9.0
	ZINC72319879	-8.5	427.424	-2.4904	10	4	312.16	0.39326	165.81	1.5644	459.9676358652	0.2898	8.8277	-8.5935	0.6	0.60309	0.99855 (max o	9	6878; 6641; 6324; 52	-0.1913742	0.6797539		9.0
	ZINC11592619	-8.5	445.447	-2.6981	10	7	305.32	0.45028	182.82	2.96	459.9676358652	0.27169	9.0354	-9.9309	0.375	0.36615	0.94464 (max o	3	107; 206; 769	0.15367767	-0.8125084		8.0
	ZINC04081695	-8.5	414.504	-0.9244	8	3	317.52	0.30511	132.97	5.0589	459.9676358652	0.2898	7.2617	-3.1898	0.56667	0.4873	0.95804 (max o	10	6947; 3633; 2409; 18	0.2856243	0.26693073		8.0
	ZINC12871079	-8.5	446.570	-0.9935	8	3	337.77	0.3386	158.27	5.1985	459.9676358652	0.28045	7.3308	-3.5425	0.6129	0.49485	0.94476 (max o	10	6947; 6423; 4629; 43	0.30342874	0.2974436		8.0

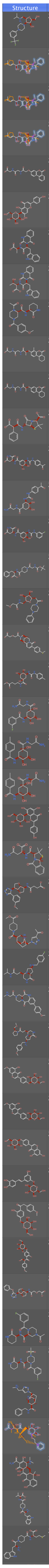

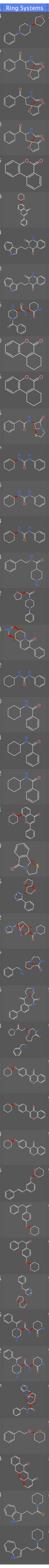

Structure	Molecule Name	affinity	Total Molweight	cLogP	H-Acceptors	H-Donors	Total Surface Area	Relative PSA	Polar Surface Area	Druglikeness	Kd (nM) @ 20°C	LE from K...	LLE from Kd ...	LELP from Kd (n...	Shape Index	Molecular...	Neighbor Si...	Neig...	Neighbor	Neighbor ...	Neighbor ...	Ring Systems	frequency
	ZINC14488852	-8.5	379.364	-0.749	9	4	267.76	0.39901	134.55	-8.6166	459.9676358652	0.322	7.0863	-2.3261	0.44444	0.40898	0.95493 (max o	3	1401; 1635; 1728	0.34669483	0.36054114		8.0
	ZINC01320008	-8.5	379.364	-0.749	9	4	267.76	0.39901	134.55	-8.6166	459.9676358652	0.322	7.0863	-2.3261	0.44444	0.40898	0.95493 (max o	3	1822; 1728; 1401	0.3274851	0.35719568		8.0
	ZINC35465905	-8.5	364.346	-3.2093	10	7	234.55	0.51887	169.3	-2.3427	459.9676358652	0.34776	9.5466	-9.2285	0.44	0.36432	0.98188 (max o	4	6338; 3197; 3078; 16	0.84689814	4.7052884E		8.0
	ZINC05438552	-8.5	401.398	-2.1581	10	2	297.98	0.40046	138.64	-3.0037	459.9676358652	0.29979	8.4954	-7.1986	0.62069	0.38681	0.97941 (max o	8	5425; 5424; 4995; 45	-0.6808841	-0.5817056		7.0
	ZINC00941350	-8.5	396.350	-1.9406	8	0	292.06	0.33445	125.02	1.3459	459.9676358652	0.29979	8.2779	-6.4731	0.44828	0.35115	0.98525 (max o	3	3126; 2711; 1960	0.17323466	0.8484118		7.0
	ZINC38569836	-8.5	425.464	-0.8327	9	2	305.49	0.32404	125.64	6.0867	459.9676358652	0.28045	7.17	-2.9691	0.45161	0.39754	0.9313 (max of	4	4078; 2784; 2511; 94	-0.455534	0.3195103		7.0
	ZINC38890031	-8.5	443.471	-3.1577	11	2	313.34	0.40314	144.48	1.7982	459.9676358652	0.28045	9.495	-11.259	0.45161	0.45576	0.96971 (max o	5	3083; 2778; 2030; 16	-0.2474840	-0.8807999		7.0
	ZINC38890024	-8.5	443.471	-3.1577	11	2	313.34	0.40314	144.48	1.7982	459.9676358652	0.28045	9.495	-11.259	0.45161	0.45585	0.96971 (max o	5	3083; 2778; 2030; 19	-0.2362874	-0.8645957		7.0
	ZINC04098535	-8.5	422.341	-0.4299	11	8	270.83	0.50895	197.37	-3.0467	459.9676358652	0.2898	6.7672	-1.4834	0.46667	0.3445	0.95318 (max o	11	3052; 104; 222; 275;	-0.1259497	-0.7006703		6.0
	ZINC12900988	-8.5	373.344	-3.6017	10	2	273.54	0.41829	140.23	2.0762	459.9676358652	0.322	9.939	-11.185	0.62963	0.50471	0.87121 (max o	3	2167; 964; 966	0.20628159	0.52193195		6.0
	ZINC02944402	-8.5	423.388	-4.9632	12	3	316.19	0.47361	198.97	2.7179	459.9676358652	0.28045	11.3	-17.697	0.64516	0.57625	0.98065 (max o	5	2350; 2206; 1993; 10	-0.5842871	0.6835262		6.0
	ZINC04081846	-8.5	374.368	-0.9625	8	0	274.58	0.31667	105.2	5.7109	459.9676358652	0.322	7.2998	-2.9891	0.51852	0.48783	0.92064 (max o	6	2943; 2944; 2408; 20	0.28645036	0.21022442		6.0
	ZINC04073934	-8.5	378.787	-0.2865	7	0	267.74	0.28741	95.97	5.7694	459.9676358652	0.33438	6.6238	-0.8568	0.53846	0.48199	0.94975 (max o	6	4011; 2408; 2036; 20	0.24826573	0.1779373		6.0
	ZINC20465171	-8.5	398.889	-0.0094996	8	4	290.47	0.27287	97.3	7.6748	459.9676358652	0.322	6.3468	-0.029502	0.55556	0.54531	0.9399 (max of	4	2995; 2592; 1868; 12	-0.3557109	-0.0310801		6.0
	ZINC20465174	-8.5	394.470	-0.6855	9	4	297.31	0.30023	106.53	7.6843	459.9676358652	0.3105	7.0228	-2.2077	0.57143	0.55683	0.9399 (max of	5	4307; 2594; 2592; 18	-0.3747265	-0.0378118		6.0
	ZINC20465171	-8.5	399.897	-2.1192	8	5	304.51	0.30639	98.5	3.6925	459.9676358652	0.322	8.4565	-6.5814	0.55556	0.54531	0.9399 (max of	5	4301; 2995; 2593; 12	-0.3246573	-0.0474194		6.0
	ZINC20465174	-8.5	395.478	-2.7952	9	5	311.35	0.33178	107.73	3.7274	459.9676358652	0.3105	9.1325	-9.0023	0.57143	0.55683	0.9399 (max of	7	5933; 4308; 4302; 25	-0.3449998	-0.0104352		6.0
	ZINC08643362	-8.5	391.379	-0.2511	10	3	288.43	0.41532	144.01	0.34279	459.9676358652	0.3105	6.5884	-0.8087	0.64286	0.45987	0.95099 (max o	3	3331; 2113; 1771	-0.2576289	-0.5680943		6.0
	ZINC08643368	-8.5	427.433	-0.3711	11	3	299.26	0.45222	169.46	-3.1358	459.9676358652	0.29979	6.7084	-1.2379	0.62069	0.47565	0.91561 (max o	4	5857; 2921; 2113; 17	-0.2817139	-0.6023176		6.0
	ZINC12603473	-8.5	447.365	0.4361	10	3	311.64	0.37463	136.17	-5.9918	459.9676358652	0.28045	5.9012	1.555	0.64516	0.51869	0.90045 (max o	3	4226; 3331; 2113	-0.2906858	-0.5841025		6.0
	ZINC16052277	-8.5	443.431	-6.6182	10	5	308.51	0.45328	188.48	2.5036	459.9676358652	0.27169	12.955	-24.36	0.375	0.36573	0.89541 (max o	2	11; 1847	-0.5452946	-0.4722930		5.0
	ZINC16052277	-8.5	442.423	-5.2519	10	4	294.47	0.42721	187.28	5.1694	459.9676358652	0.27169	11.589	-19.331	0.375	0.36418	0.94718 (max o	2	1852; 66	-0.5219562	-0.4458613		5.0
	ZINC16052277	-8.5	442.423	-5.3235	10	4	294.47	0.42721	187.28	5.0928	459.9676358652	0.27169	11.661	-19.594	0.375	0.36418	0.94718 (max o	4	2183; 7; 64; 399	-0.4369423	-0.5017269		5.0
	ZINC16052277	-8.5	443.431	-6.6898	10	5	308.51	0.45328	188.48	2.3981	459.9676358652	0.27169	13.027	-24.623	0.375	0.36573	0.9497 (max of	4	2187; 10; 68; 403	-0.3590607	-0.5042946		5.0
	ZINC20758721	-8.5	385.419	-0.8469	8	3	262.29	0.33814	107.97	3.0652	459.9676358652	0.3105	7.1842	-2.7275	0.42857	0.22685	0.98866 (max o	9	3418; 1880; 385; 387	0.10372756	0.8256902		5.0
	ZINC20758729	-8.5	385.419	-0.8469	8	3	262.29	0.33814	107.97	3.0652	459.9676358652	0.3105	7.1842	-2.7275	0.42857	0.21732	0.98866 (max o	8	3418; 385; 398; 647;	0.09551050	0.8411244		5.0
	ZINC05396073	-8.5	401.422	-0.295	10	1	294.3	0.33945	107.81	-2.4752	459.9676358652	0.29979	6.6323	-0.98401	0.62069	0.38753	0.92464 (max o	3	579; 911; 1724	-0.2947414	-0.2616428		5.0
	ZINC04260924	-8.5	358.353	-0.1914	9	1	259.48	0.37132	104.57	-3.2548	459.9676358652	0.33438	6.5287	-0.5724	0.61538	0.3475	0.94002 (max o	4	1735; 579; 911; 1725	-0.3101962	-0.2280208		5.0
	ZINC05959287	-8.5	350.373	2.1326	6	2	271.88	0.25379	76.66	2.9999	459.9676358652	0.33438	4.2047	6.3777	0.69231	0.40671	0.87452	1	596	0.23058857	-0.9630915		5.0
	ZINC08552017	-8.5	424.401	-1.9238	10	3	247.15	0.47914	148.82	-1.779	459.9676358652	0.2898	8.2611	-6.6384	0.33333	0.093597	0.91374 (max o	4	5818; 3544; 2869; 72	-0.6465316	-0.4048906		5.0
	ZINC04260834	-8.5	404.445	-1.4497	10	2	314.35	0.35279	101.17	-11.157	459.9676358652	0.29979	7.787	-4.8357	0.65517	0.36702	0.91934 (max o	4	2442; 731; 732; 733	-0.2606665	-0.0196961		5.0
	ZINC13516295	-8.5	357.369	0.0027999	9	4	257.63	0.39025	125.55	-0.31782	459.9676358652	0.33438	6.3345	0.0083732	0.57692	0.36038	0.93361 (max o	8	3229; 2807; 2417; 23	-0.3988419	-0.7665622		5.0
	ZINC43465475	-8.5	432.380	-0.078	10	7	289.19	0.43134	177.14	-1.9977	459.9676358652	0.28045	6.4153	-0.27812	0.45161	0.3816	0.93686 (max o	6	4107; 3226; 2806; 25	0.7860902	0.07542708		5.0
	ZINC08298074	-8.5	364.468	-2.4969	8	2	284.49	0.30261	84.92	-0.57226	459.9676358652	0.33438	8.8342	-7.4672	0.57692	0.53817	0.96721 (max o	6	6751; 5446; 5439; 41	0.22908163	0.2322165		5.0
	ZINC08636501	-8.5	405.517	1.0644	7	3	291.25	0.32017	119.69	5.446	459.9676358652	0.3105	5.2729	3.428	0.5	0.40938	0.89839 (max o	7	3735; 3296; 2889; 28	-0.5519892	0.360165		5.0
	ZINC05742785	-8.5	448.423	0.4421	10	5	310.3	0.38202	155.14	-3.5326	459.9676358652	0.27169	5.8952	1.6272	0.5	0.32874	0.95949 (max o	4	220; 460; 461; 757	0.14170852	-0.3260239		4.0
	ZINC31158962	-8.5	360.361	0.1524	7	5	235.62	0.38868	135.29	-0.37577	459.9676358652	0.33438	6.1849	0.45576	0.42308	0.30051	0.9464 (max of	3	304; 406; 1024	0.83209217	-0.490826		4.0
	ZINC35458761	-8.5	376.360	-0.671	8	6	239.88	0.43638	155.52	-0.097646	459.9676358652	0.322	7.0083	-2.0839	0.40741	0.31342	0.97149 (max o	3	410; 522; 1056	-0.8989587	-0.3726797		4.0
	ZINC08790788	-8.5	426.356	-1.0697	10	2	303.1	0.38918	143.45	4.6454	459.9676358652	0.28045	7.407	-3.8142	0.58065	0.39269	0.93148 (max o	7	4090; 3204; 2935; 17	0.5393301	0.37968144		4.0
	ZINC35458042	-8.5	353.305	-0.3835	7	2	238.43	0.37579	123.96	2.5684	459.9676358652	0.33438	6.7208	-1.1469	0.46154	0.26377	0.97533 (max o	3	521; 673; 1609	-0.8368295	-0.4837380		4.0
	ZINC15958063	-8.5	424.475	-3.1866	8	2	300	0.3097	118.64	-1.065	459.9676358652	0.28045	9.5239	-11.362	0.3871	0.28661	0.96262 (max o	5	110; 297; 382; 796; 1	0.35937047	-0.2016448		4.0
	ZINC13308150	-8.5	363.416	0.5683	9	3	267.82	0.36383	114.55	-3.3637	459.9676358652	0.33438	5.769	1.6995	0.53846	0.3046	0.94008 (max o	3	4249; 2168; 860	-0.4275701	-0.8497875		4.0
	ZINC05397423	-8.5	432.368	-3.2084	13	1	301.29	0.46998	158.98	0.50605	459.9676358652	0.28045	9.5457	-11.44	0.58065	0.33405	0.91993 (max o	5	3252; 450; 914; 915;	-0.1939882	-0.3640752		4.0

Structure	Molecule Name	affinity	Total Molweight	cLogP	H-Acceptors	H-Donors	Total Surface Area	Relative PSA	Polar Surface Area	Druglikeness	Kd (nM) @ 20°C	LE from K...	LLE from Kd ...	LELP from Kd (n...	Shape Index	Molecular...	Neighbor Si...	Neig...	Neighbor	Neighbor ...	Neighbor ...	Ring Systems	frequency
	ZINC05433672	-8.5	411.481	-1.3447	10	2	292.89	0.3571	129.2	7.6657	459.9676358652	0.3105	7.682	-4.3308	0.46429	0.48348	0.90171 (max o	3	3374; 2863; 1179	-0.0741149	0.31569248		4.0
	ZINC15708222	-8.5	431.511	-1.7778	9	3	337.17	0.30602	101.41	4.243	459.9676358652	0.28045	8.1151	-6.3391	0.6129	0.47695	0.93686 (max o	4	2200; 983; 984; 1240	-0.1745487	0.14497586		4.0
	ZINC05765083	-8.5	386.267	-3.1938	10	2	252.84	0.48995	165.48	-1.7419	459.9676358652	0.3105	9.5311	-10.286	0.35714	0.27575	0.93985 (max o	2	986; 1753	0.8806033	0.3330102		4.0
	ZINC05765083	-8.5	386.267	-3.6918	10	2	252.84	0.48995	165.48	-1.8365	459.9676358652	0.3105	10.029	-11.89	0.35714	0.26587	0.93985 (max o	2	987; 1752	0.9004945	0.3480647		4.0
	ZINC31162090	-8.5	370.312	1.0015	8	3	243.66	0.40392	130.36	0.8612	459.9676358652	0.322	5.3358	3.1103	0.44444	0.24731	0.95493 (max o	3	4806; 2265; 1027	-0.2897282	-0.8084020		4.0
	ZINC67913743	-8.5	421.464	-0.9847	8	2	305.6	0.32592	133.19	-8.4075	459.9676358652	0.2898	7.322	-3.3979	0.46667	0.47687	0.9707 (max of	3	3543; 1067; 1337	-0.9074787	-0.2331374		4.0
	ZINC71404467	-8.5	434.396	0.3932	10	6	291.63	0.4171	166.14	1.8995	459.9676358652	0.28045	5.9441	1.402	0.41935	0.35036	0.95771 (max o	4	5273; 2275; 1069; 13	0.69488704	-0.5034531		4.0
	ZINC04257486	-8.5	357.369	0.0027999	9	4	257.63	0.39025	125.55	-0.31782	459.9676358652	0.33438	6.3345	0.0083732	0.57692	0.3491	0.92702 (max o	10	3229; 2807; 2417; 23	-0.3879499	-0.7148881		4.0
	ZINC08643356	-8.5	415.392	-0.4185	9	4	292.94	0.3371	120.36	1.3008	459.9676358652	0.29979	6.7558	-1.396	0.62069	0.54445	0.92486 (max o	2	2527; 1209	0.29987207	-0.3484927		4.0
	ZINC35464839	-8.5	398.494	0.9599	7	4	288.68	0.29597	116.45	-3.8942	459.9676358652	0.3105	5.3774	3.0915	0.46429	0.4288	0.96519 (max o	4	6619; 3933; 1610; 19	-0.7035771	0.05739580		4.0
	ZINC35464837	-8.5	398.494	0.9599	7	4	288.68	0.29597	116.45	-3.8942	459.9676358652	0.3105	5.3774	3.0915	0.46429	0.4288	0.96519 (max o	6	6619; 5208; 3933; 22	-0.7077064	0.03479908		4.0
	ZINC02147370	-8.5	352.369	-0.4593	7	1	256.85	0.28016	92.78	3.893	459.9676358652	0.33438	6.7966	-1.3736	0.53846	0.50892	0.94951 (max o	5	5677; 3147; 1972; 68	0.21101144	0.78018063		4.0
	ZINC02115241	-8.5	352.369	-0.4593	7	1	256.85	0.28016	92.78	3.893	459.9676358652	0.33438	6.7966	-1.3736	0.57692	0.50892	0.94951 (max o	3	5677; 1972; 1655	0.1976772	0.7657826		4.0
	ZINC04556881	-8.5	400.307	-3.8839	12	3	262.82	0.51275	163.91	-12.61	459.9676358652	0.322	10.221	-12.062	0.55556	0.31092	0.92418 (max o	3	2826; 584; 746	0.08345306	-0.9680815		4.0
	ZINC19800888	-8.5	372.336	-0.7308	10	4	253.61	0.44292	145.11	1.0072	459.9676358652	0.322	7.0681	-2.2696	0.48148	0.3049	0.94302 (max o	4	2210; 2208; 1733; 18	0.6340247	-0.5135189		4.0
	ZINC05037435	-8.5	372.336	-0.7308	10	4	253.61	0.44292	145.11	1.0072	459.9676358652	0.322	7.0681	-2.2696	0.48148	0.3049	0.94905 (max o	3	2210; 2208; 1855	0.62317216	-0.5338155		4.0
	ZINC05119408	-8.5	418.397	0.2774	9	6	298.13	0.37447	156.91	-3.3049	459.9676358652	0.2898	6.0599	0.95722	0.6	0.44618	0.95837 (max o	4	3468; 2830; 2461; 92	0.44003564	0.4408505		4.0
	ZINC71405299	-8.5	373.455	-0.2291	8	3	286.57	0.28126	93.78	5.7796	459.9676358652	0.322	6.5664	-0.71149	0.59259	0.56131	0.90377 (max o	2	3682; 2322	0.15292856	0.25183356		4.0
	ZINC05433811	-8.5	418.472	-4.3812	10	3	312.09	0.34176	135.69	3.6249	459.9676358652	0.2898	10.718	-15.118	0.53333	0.53511	0.96343 (max o	6	5806; 5426; 4160; 36	0.11246051	0.26013392		4.0
	ZINC15219306	-8.5	446.363	-3.8892	11	4	322.16	0.43481	196.71	0.29536	459.9676358652	0.27169	10.226	-14.315	0.53125	0.49497	0.98343 (max o	3	2641; 1894; 1824	0.07534385	-0.8468628		4.0
	ZINC15219274	-8.5	446.363	-3.8892	11	4	322.16	0.43481	196.71	0.29536	459.9676358652	0.27169	10.226	-14.315	0.5625	0.49497	0.98343 (max o	3	2641; 1894; 1825	0.05616471	-0.8553834		4.0
	ZINC31155506	-8.5	432.336	-4.1649	11	5	306.25	0.46753	207.71	0.17356	459.9676358652	0.28045	10.502	-14.851	0.54839	0.50171	0.95983 (max o	5	6068; 6067; 2641; 18	0.05540358	-0.8744806		4.0
	ZINC34614538	-8.5	386.439	-0.5583	8	5	279.37	0.35272	136.68	-5.8875	459.9676358652	0.322	6.8956	-1.7339	0.51852	0.43737	0.99202 (max o	7	6606; 5963; 5188; 51	0.20675945	0.86760867		4.0
	ZINC35458393	-8.5	432.511	-1.9041	7	1	310.36	0.30229	134.63	0.74206	459.9676358652	0.28045	8.2414	-6.7894	0.3871	0.46879	0.94488 (max o	4	6645; 5639; 5236; 52	-0.4762985	0.44124237		4.0
	ZINC16052277	-8.5	445.447	-2.3761	10	7	306.15	0.44906	182.82	1.3997	459.9676358652	0.27169	8.7134	-8.7457	0.375	0.34301		0		0.4474279	-0.8886146		3.0
	ZINC05414495	-8.5	403.438	-1.3156	10	2	303.12	0.34029	120.94	4.9815	459.9676358652	0.29979	7.6529	-4.3884	0.48276	0.52544	0.97415 (max o	7	4570; 4132; 3263; 24	0.09276485	0.2673239		3.0
	ZINC27558826	-8.5	440.359	-3.0384	10	2	329.78	0.38486	173.32	-1.5257	459.9676358652	0.27169	9.3757	-11.183	0.625	0.40652	0.9665 (max of	2	814; 1820	0.5167134	0.7226762		3.0
	ZINC13556379	-8.5	440.359	-3.0384	10	2	329.78	0.38486	173.32	-1.5257	459.9676358652	0.27169	9.3757	-11.183	0.625	0.40616	0.9665 (max of	2	1889; 814	0.5108669	0.703611		3.0
	ZINC13412861	-8.5	449.387	-0.3513	11	8	288.97	0.43188	180.3	-8.6993	459.9676358652	0.27169	6.6886	-1.293	0.40625	0.27157	0.95709 (max o	6	3350; 3349; 2963; 27	-0.837791	0.3075694		3.0
	ZINC35271791	-8.5	434.396	-0.258	10	7	290.74	0.42904	177.14	-2.4912	459.9676358652	0.28045	6.5953	-0.91995	0.51613	0.39907	0.96502 (max o	6	3370; 1909; 979; 123	-0.748033	-0.2523871		3.0
	ZINC31156807	-8.5	436.412	-1.0455	10	8	300.59	0.4253	188.14	-1.8772	459.9676358652	0.28045	7.3828	-3.7279	0.51613	0.49349	0.96739 (max o	2	3451; 1031	-0.6835297	0.66256344		3.0
	ZINC03984838	-8.5	361.417	0.3681	7	3	249.05	0.2797	117.32	-7.0984	459.9676358652	0.33438	5.9692	1.1008	0.53846	0.36493	0.93686 (max o	3	4499; 3181; 1113	0.9715783	0.05175022		3.0
	ZINC04260227	-8.5	405.425	-0.1902	8	0	289.34	0.24113	79.39	4.4625	459.9676358652	0.29979	6.5275	-0.63444	0.62069	0.41764	0.96473 (max o	3	2381; 1149; 1154	-0.6762123	0.55735767		3.0
	ZINC15675954	-8.5	397.390	-2.7032	10	2	288.48	0.36034	138.52	3.2805	459.9676358652	0.29979	9.0405	-9.0169	0.55172	0.48719	0.95945 (max o	7	5059; 495; 651; 1837	0.3046866	-0.1459704		3.0
	ZINC05415736	-8.5	374.372	-2.4761	9	0	271.75	0.32048	110.29	4.4944	459.9676358652	0.322	8.8134	-7.6898	0.59259	0.49654	0.87932 (max o	2	4591; 3260	0.20791669	0.962745		3.0
	ZINC03839287	-8.5	429.519	0.3338	9	2	314.73	0.28294	103.75	5.14	459.9676358652	0.28045	6.0035	1.1902	0.48387	0.36981	0.92863 (max o	8	3818; 2749; 2001; 16	-0.5669249	-0.0466182		3.0
	ZINC08627180	-8.5	403.409	-0.3315	8	4	297.03	0.32377	120.78	-0.4643	459.9676358652	0.29979	6.6688	-1.1058	0.62069	0.52693	0.9398 (max of	4	5821; 3334; 3289; 21	0.38198382	-0.3874040		3.0
	ZINC12603429	-8.5	404.418	-0.3127	9	4	303.87	0.35973	130.26	1.5298	459.9676358652	0.29979	6.65	-1.0431	0.65517	0.53215	0.94543 (max o	2	5480; 2568	-0.3684929	-0.1933667		3.0
	ZINC13284239	-8.5	365.364	-1.3885	7	2	257.23	0.3152	113.79	5.8403	459.9676358652	0.322	7.7258	-4.3121	0.44444	0.34578	0.96343	1	2395	0.7046773	-0.3668048		3.0
	ZINC15268992	-8.5	434.396	-0.258	10	7	290.74	0.42904	177.14	-2.4912	459.9676358652	0.28045	6.5953	-0.91995	0.51613	0.39907	0.96018 (max o	5	3370; 1909; 1908; 97	-0.7796685	-0.2268051		3.0
	ZINC35271797	-8.5	434.396	-0.258	10	7	290.74	0.42904	177.14	-2.4912	459.9676358652	0.28045	6.5953	-0.91995	0.51613	0.39907	0.96502 (max o	6	3370; 3058; 979; 123	-0.737564	-0.2348681		3.0
	ZINC19800888	-8.5	371.328	-2.9004	10	3	254.9	0.44574	151.43	-0.58508	459.9676358652	0.322	9.2377	-9.0075	0.48148	0.2975	0.94302 (max o	3	2211; 2209; 1855	0.64875907	-0.4913559		3.0
	ZINC20465791	-8.5	435.563	-0.1754	7	4	336.16	0.27912	108.68	-3.8672	459.9676358652	0.2898	6.5127	-0.60525	0.56667	0.55001	0.97975 (max o	6	7398; 4758; 4358; 43	0.49405023	-0.3546788		3.0

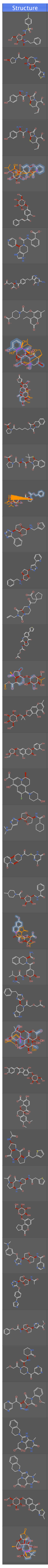

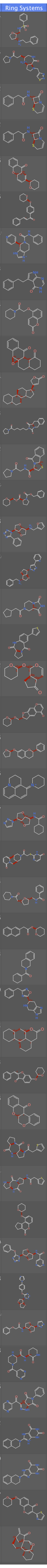

Structure	Molecule Name	affinity	Total Molweight	cLogP	H-Acceptors	H-Donors	Total Surface Area	Relative PSA	Polar Surface Area	Druglikeness	Kd (nM) @ 20°C	LE from K...	LLE from Kd ...	LELP from Kd (n...	Shape Index	Molecular...	Neighbor Si...	Neig...	Neighbor	Neighbor ...	Neighbor ...	Ring Systems	frequency
	ZINC20465785	-8.5	439.526	-0.4185	7	4	330.25	0.28412	108.68	0.074511	459.9676358652	0.2898	6.7558	-1.4441	0.56667	0.55001	0.97975 (max o	6	7398; 4758; 4358; 43	0.4745985	-0.3612964		3.0
	ZINC22923791	-8.5	409.533	-2.4812	9	3	310.57	0.37876	131.34	1.9905	459.9676358652	0.3105	8.8185	-7.991	0.57143	0.46046	0.94291 (max o	11	7405; 7085; 5998; 56	-0.0261849	0.53753364		3.0
	ZINC36378487	-8.5	388.423	-3.7931	8	3	274.1	0.41689	161.09	6.7688	459.9676358652	0.322	10.13	-11.78	0.59259	0.44815	0.93996 (max o	6	4421; 3519; 3383; 22	-0.4914070	-0.1081752		3.0
	ZINC36378487	-8.5	389.431	-1.5936	8	3	273.31	0.41521	162.71	7.0007	459.9676358652	0.322	7.9309	-4.9491	0.59259	0.43147	0.93996 (max o	4	4420; 3518; 2367; 19	-0.5178337	-0.0766462		3.0
	ZINC36728546	-8.5	392.359	-1.3716	9	6	257.5	0.43355	156.91	-2.2179	459.9676358652	0.3105	7.7089	-4.4174	0.46429	0.35879	0.94862 (max o	6	5589; 3080; 2619; 41	-0.2691645	0.58298326		3.0
	ZINC03978972	-8.5	402.398	0.6231	8	5	291.78	0.33772	136.68	-3.3049	459.9676358652	0.29979	5.7142	2.0784	0.58621	0.44156	0.95372 (max o	3	3468; 3469; 236	0.47615713	0.46171096		2.0
	ZINC40309478	-8.5	363.376	-0.682	8	3	260.21	0.30871	115.29	3.7493	459.9676358652	0.322	7.0193	-2.118	0.44444	0.46019	0.98371 (max o	6	2672; 243; 528; 1932	-0.1484521	-0.4637281		2.0
	ZINC01851132	-8.5	425.400	-4.0454	11	4	317.1	0.45541	192.63	4.4441	459.9676358652	0.28045	10.383	-14.425	0.6129	0.59736	0.98065 (max o	2	248; 922	0.8584556	0.20297593		2.0
	ZINC02122314	-8.5	372.396	-0.2959	7	0	281.63	0.27323	95.97	2.8531	459.9676358652	0.322	6.6332	-0.91895	0.55556	0.44855	0.94622 (max o	5	2405; 100; 551; 552;	0.11898285	0.16723454		2.0
	ZINC36378599	-8.5	360.361	-0.1543	7	4	232.58	0.41332	127.59	0.18236	459.9676358652	0.33438	6.4916	-0.46145	0.42308	0.29718	0.9615 (max of	3	2668; 845; 1612	0.02991724	0.9502955		2.0
	ZINC04082048	-8.5	420.500	0.1079	7	4	283.64	0.31194	124.29	-3.8679	459.9676358652	0.2898	6.2294	0.37233	0.46667	0.35381		0		0.8619804	-0.4612414		2.0
	ZINC04090564	-8.5	412.464	-0.4261	8	0	304.79	0.25293	101.06	-1.2035	459.9676358652	0.2898	6.7634	-1.4703	0.56667	0.47964	0.95401 (max o	3	3643; 344; 895	0.19875668	-0.9227523		2.0
	ZINC08790900	-8.5	430.504	-0.7803	8	1	299.31	0.34242	135.15	8.4506	459.9676358652	0.2898	7.1176	-2.6926	0.46667	0.33829	0.93965 (max o	5	4082; 3203; 2786; 47	0.36364803	-0.6085382		2.0
	ZINC16043224	-8.5	411.405	-0.4553	10	4	291.26	0.38464	145.99	0.28886	459.9676358652	0.29979	6.7926	-1.5187	0.58621	0.50894	0.95908 (max o	3	2532; 2158; 1050	0.06361658	-0.9612483		2.0
	ZINC04259601	-8.5	354.389	-0.5496	8	1	259.97	0.314	91.81	0.23114	459.9676358652	0.33438	6.8869	-1.6436	0.57692	0.30009	0.92094 (max o	4	267; 749; 1145; 1152	-0.0064285	-0.2773188		2.0
	ZINC05396119	-8.5	364.428	-0.6252	7	1	270.28	0.24982	78.67	-3.0801	459.9676358652	0.322	6.9625	-1.9416	0.62963	0.34837	0.90934 (max o	3	749; 1165; 1184	-0.0787410	-0.2364195		2.0
	ZINC05434222	-8.5	362.405	-1.1708	8	0	267.48	0.28821	101.06	0.47371	459.9676358652	0.33438	7.5081	-3.5014	0.53846	0.58207	0.93367 (max o	2	1173; 1174	0.499751	0.532458		2.0
	ZINC03839540	-8.5	428.444	-2.128	9	2	303.75	0.40583	156.11	5.2305	459.9676358652	0.2898	8.4653	-7.343	0.56667	0.34781	0.93405 (max o	5	3272; 57; 464; 556; 1	-0.2429005	0.438816		2.0
	ZINC33830309	-8.5	388.368	-1.827	10	4	263.15	0.45024	151.98	-6.054	459.9676358652	0.322	8.1643	-5.6739	0.48148	0.37688	0.93996	1	1294	0.80011266	0.05845392		2.0
	ZINC31156025	-8.5	438.427	0.2591	10	5	296.68	0.39956	155.14	-11.09	459.9676358652	0.28045	6.0782	0.92387	0.48387	0.45556	0.96018 (max o	7	182; 235; 405; 839; 8	-0.2093606	0.8684949		2.0
	ZINC49601418	-8.5	422.385	0.0028001	10	7	282.16	0.43132	169.3	1.0702	459.9676358652	0.2898	6.3345	0.0096622	0.53333	0.3425	0.89867 (max o	7	233; 454; 650; 1021;	0.37485012	-0.8434436		2.0
	ZINC00603195	-8.5	360.384	-1.7227	6	2	242.22	0.21414	86.84	2.1757	459.9676358652	0.33438	8.06	-5.1519	0.5	0.34931	0.9615	1	1371	0.18996842	-0.8306686		2.0
	ZINC04260886	-8.5	364.468	-1.9475	8	2	290.13	0.31331	82.71	-5.4013	459.9676358652	0.33438	8.2848	-5.8241	0.61538	0.3816	0.89688 (max o	2	3233; 1444	-0.1689786	0.06862077		2.0
	ZINC01323579	-8.5	373.412	-2.1561	8	1	262.52	0.38458	130.44	4.2093	459.9676358652	0.33438	8.4934	-6.448	0.53846	0.51658	0.94951 (max o	2	797; 1482	0.5527999	0.701892		2.0
	ZINC12603616	-8.5	376.451	0.8193	7	3	283.54	0.27259	99.1	2.9883	459.9676358652	0.322	5.518	2.5444	0.62963	0.53908	0.92152	1	1494	0.35739145	-0.2975054		2.0
	ZINC14612367	-8.5	405.378	-1.568	9	3	285.96	0.38348	145.58	1.7924	459.9676358652	0.29979	7.9053	-5.2303	0.55172	0.43286	0.97415 (max o	3	1234; 1235; 1511	-0.7614431	-0.6203386		2.0
	ZINC15675327	-8.5	445.538	-2.3864	9	5	328	0.31506	149.57	6.3942	459.9676358652	0.27169	8.7237	-8.7836	0.46875	0.49178	0.94488	1	1518	0.88336915	0.37545243		2.0
	ZINC15675954	-8.5	396.382	-2.5143	10	1	299.45	0.38377	137.27	3.7989	459.9676358652	0.29979	8.8516	-8.3868	0.55172	0.48719	0.97415 (max o	7	5060; 4687; 497; 653	0.30177975	-0.1828380		2.0
	ZINC67912726	-8.5	416.465	-1.8823	9	7	262.4	0.43727	167.91	-0.95798	459.9676358652	0.29979	8.2196	-6.2787	0.34483	0.41166	0.94823 (max o	3	3106; 3105; 1620	0.89104366	-0.1620135		2.0
	ZINC71404537	-8.5	448.379	-0.0535	11	7	299.19	0.45035	186.37	-3.3789	459.9676358652	0.27169	6.3908	-0.19692	0.53125	0.27454	0.95709 (max o	4	2321; 677; 1624; 181	-0.2852702	-0.6849143		2.0
	ZINC03197511	-8.5	387.319	-0.8425	9	2	262.94	0.40526	134.58	0.9819	459.9676358652	0.3105	7.1798	-2.7134	0.46429	0.2766	0.9543 (max of	3	6084; 5313; 1098	-0.1928723	0.77598566		2.0
	ZINC03839260	-8.5	390.463	-0.4037	8	2	274.28	0.37385	127.06	7.3399	459.9676358652	0.322	6.741	-1.2537	0.51852	0.41177	0.94573 (max o	5	3163; 2753; 1671; 16	-0.6719878	-0.070251		2.0
	ZINC03839424	-8.5	390.463	-0.4037	8	2	274.28	0.37385	127.06	8.7513	459.9676358652	0.322	6.741	-1.2537	0.51852	0.39212	0.93686 (max o	6	4921; 4036; 3163; 27	-0.6387497	-0.1033601		2.0
	ZINC04073402	-8.5	378.376	0.0735	8	4	258.63	0.36902	125.68	-6.0454	459.9676358652	0.322	6.2638	0.22826	0.44444	0.25864	0.94905 (max o	4	2406; 103; 193; 1120	0.30076334	0.4526708		2.0
	ZINC04147147	-8.5	438.447	0.0241	12	1	320.33	0.3739	129.41	2.5567	459.9676358652	0.27169	6.3132	0.088706	0.5625	0.37227	0.91704 (max o	8	4531; 3649; 3218; 27	-0.2661059	-0.3513669		2.0
	ZINC04147835	-8.5	397.394	-0.1303	11	1	288.21	0.38902	118.21	1.3702	459.9676358652	0.29979	6.4676	-0.43463	0.58621	0.38208	0.96682 (max o	4	2061; 2047; 348; 141	-0.1409072	-0.4011688		2.0
	ZINC04260931	-8.5	388.379	-0.6165	10	1	283.24	0.37548	113.8	-3.0685	459.9676358652	0.3105	6.9538	-1.9855	0.64286	0.36733	0.92124 (max o	4	2812; 2064; 911; 172	-0.2902436	-0.2202204		2.0
	ZINC05414503	-8.5	404.426	-1.8779	11	2	301.88	0.37803	133.83	4.9815	459.9676358652	0.29979	8.2152	-6.264	0.48276	0.52544	0.97415 (max o	3	5805; 4132; 1739	0.07633427	0.25471458		2.0
	ZINC06928299	-8.5	351.321	-1.0984	9	1	248.78	0.39223	113.85	5.0717	459.9676358652	0.33438	7.4357	-3.2848	0.5	0.2284		0		0.8704335	0.48365346		2.0
	ZINC12443285	-8.5	355.353	-2.3035	9	2	243.15	0.3242	111.85	-6.1976	459.9676358652	0.33438	8.6408	-6.8888	0.46154	0.37914	0.90377	1	2136	-0.7412409	-0.4613632		2.0
	ZINC12443285	-8.5	354.345	-2.1146	9	1	254.12	0.35338	110.6	-5.4718	459.9676358652	0.33438	8.4519	-6.3239	0.46154	0.37914	0.91059 (max o	2	4272; 2137	-0.3067761	-0.9182429		2.0
	ZINC12872105	-8.5	421.425	-0.4427	9	4	287.1	0.44155	166.81	-1.195	459.9676358652	0.29979	6.78	-1.4767	0.55172	0.38679	0.94543	1	3298	0.30126068	-0.8376997		2.0
	ZINC13815706	-8.5	364.436	0.5857	6	4	231.86	0.32537	107.22	-5.1582	459.9676358652	0.33438	5.7516	1.7516	0.34615	0.35342	0.93361 (max o	2	4869; 2531	0.8450259	0.3211588		2.0

Structure	Molecule Name	affinity	Total Molweight	cLogP	H-Acceptors	H-Donors	Total Surface Area	Relative PSA	Polar Surface Area	Druglikeness	Kd (nM) @ 20°C	LE from K...	LLE from Kd ...	LELP from Kd (n...	Shape Index	Molecular...	Neighbor Si...	Neig...	Neighbor	Neighbor ...	Neighbor ...	Ring Systems	frequency
	ZINC15707867	-8.5	444.554	-2.0458	9	3	351.23	0.2754	95.42	7.5627	459.9676358652	0.27169	8.3831	-7.53	0.625	0.51813	0.89541 (max o	2	3815; 3329	-0.2741117	0.17962235		2.0
	ZINC20411127	-8.5	381.388	-3.2599	10	1	250.79	0.47482	155.46	8.0587	459.9676358652	0.33438	9.5972	-9.749	0.5	0.27631	0.91351	1	3382	-0.7438581	0.19066495		2.0
	ZINC20465351	-8.5	419.451	0.1753	8	3	307.13	0.26669	98.41	4.5627	459.9676358652	0.2898	6.162	0.6049	0.56667	0.48096	0.91204 (max o	6	7729; 5556; 4749; 47	-0.3805247	0.14794713		2.0
	ZINC20465660	-8.5	434.966	-2.4426	7	4	306.59	0.16648	86.92	3.9702	459.9676358652	0.2898	8.7799	-8.4286	0.56667	0.43024	0.93686 (max o	4	6263; 2597; 2589; 18	-0.4325446	0.2673103		2.0
	ZINC20465660	-8.5	433.958	-0.4648	7	3	317.56	0.19527	85.67	3.9702	459.9676358652	0.2898	6.8021	-1.6039	0.56667	0.44706	0.96589 (max o	5	6263; 3394; 2598; 25	-0.4332410	0.2888909		2.0
	ZINC20465958	-8.5	446.522	-0.7821	9	5	355.29	0.3089	113.72	1.912	459.9676358652	0.27169	7.1194	-2.8787	0.53125	0.57219	0.88804	1	4323	0.22912072	-0.8110184		2.0
	ZINC22923297	-8.5	417.528	-1.7816	8	3	328.37	0.25986	86.55	4.6648	459.9676358652	0.2898	8.1189	-6.1477	0.6	0.49273	0.9396 (max of	5	3027; 3026; 2655; 19	0.05347097	0.52562785		2.0
	ZINC35442512	-8.5	444.530	-3.6385	9	2	347.3	0.28664	106.45	4.7539	459.9676358652	0.27169	9.9758	-13.392	0.59375	0.50878	0.93139 (max o	5	4339; 2655; 2280; 15	0.03538049	0.5153014		2.0
	ZINC22926903	-8.5	360.544	-2.2315	5	3	318.81	0.19833	41.22	5.0787	459.9676358652	0.33438	8.5688	-6.6735	0.65385	0.62476	0.90502 (max o	2	6270; 5587	-0.8039792	-0.2331926		2.0
	ZINC31163744	-8.5	424.401	-0.702	10	5	284.95	0.416	155.14	-6.6063	459.9676358652	0.2898	7.0393	-2.4224	0.46667	0.35991	0.94785 (max o	4	3906; 1130; 1289; 19	-0.7553552	-0.3376006		2.0
	ZINC31163741	-8.5	424.401	-0.702	10	5	284.95	0.416	155.14	-6.6063	459.9676358652	0.2898	7.0393	-2.4224	0.46667	0.35747	0.94785 (max o	4	3906; 1130; 1289; 19	-0.7582277	-0.3587795		2.0
	ZINC35455078	-8.5	408.402	-0.1825	9	3	294.59	0.35772	131.75	-0.23288	459.9676358652	0.29979	6.5198	-0.60875	0.55172	0.42312	0.93975 (max o	4	2628; 2288; 1606; 19	0.58131385	-0.5373050		2.0
	ZINC35455075	-8.5	408.402	-0.1825	9	3	294.59	0.35772	131.75	-0.23288	459.9676358652	0.29979	6.5198	-0.60875	0.55172	0.42312	0.93975 (max o	4	2628; 2288; 1606; 19	0.5601946	-0.537671		2.0
	ZINC35466100	-8.5	445.399	-1.014	10	3	317.43	0.38654	162.65	3.082	459.9676358652	0.27169	7.3513	-3.7322	0.5	0.40053	0.99646 (max o	3	2666; 2665; 1310	-0.0430436	-0.8949193		2.0
	ZINC49601568	-8.5	366.365	0.7334	8	4	256.94	0.37145	125.68	-17.662	459.9676358652	0.33438	5.6039	2.1933	0.53846	0.37974	0.97269 (max o	3	3088; 2676; 2675	-0.1179113	-0.8802563		2.0
	ZINC00607798	-8.5	357.384	-2.0668	6	1	256.85	0.25326	68.12	2.6458	459.9676358652	0.33438	8.4041	-6.1809	0.5	0.32651	0.82988	1	2707	0.15032063	-0.9806832		1.0
	ZINC00607798	-8.5	358.392	-3.1461	6	2	247.24	0.22796	71.05	1.2978	459.9676358652	0.33438	9.4834	-9.4086	0.5	0.25252	0.8412	1	2708	0.7557635	-0.5962332		1.0
	ZINC01557161	-8.5	390.442	-0.462	7	4	296.1	0.31841	128.45	2.9212	459.9676358652	0.29979	6.7993	-1.5411	0.55172	0.43898	0.88151 (max o	2	6693; 3999	0.2735763	0.7418091		1.0
	ZINC02130930	-8.5	380.379	-0.8418	8	2	285.05	0.32594	118.64	5.9996	459.9676358652	0.3105	7.1791	-2.7111	0.57143	0.54234	0.9771 (max of	5	2979; 2027; 1088; 13	0.15151033	-0.2176617		1.0
	ZINC03202703	-8.5	415.493	-5.0288	7	1	288.49	0.3779	146.96	11.056	459.9676358652	0.3105	11.366	-16.196	0.57143	0.43538		0		-0.9335082	0.25144157		1.0
	ZINC03831242	-8.5	400.434	0.0218	8	1	279.19	0.39751	140.87	10.153	459.9676358652	0.3105	6.3155	0.070209	0.5	0.36695	0.9428 (max of	5	3593; 686; 690; 1385	0.42901272	0.6728153		1.0
	ZINC03831243	-8.5	400.434	0.0218	8	1	279.19	0.39751	140.87	10.153	459.9676358652	0.3105	6.3155	0.070209	0.5	0.36695	0.9428 (max of	5	3593; 686; 690; 1385	0.41052166	0.66959625		1.0
	ZINC03838981	-8.5	384.458	-2.0213	8	4	298.3	0.32575	98.74	6.443	459.9676358652	0.3105	8.3586	-6.5098	0.57143	0.38674		0		-0.0734335	-0.9857507		1.0
	ZINC03839299	-8.5	416.501	-0.0856	8	2	288.54	0.35538	127.06	8.7787	459.9676358652	0.29979	6.4229	-0.28553	0.51724	0.37661	0.94573 (max o	8	3163; 2753; 1671; 16	-0.6346848	-0.0821483		1.0
	ZINC03839337	-8.5	402.474	-0.0837	8	2	274.78	0.37317	127.06	8.8522	459.9676358652	0.3105	6.421	-0.26957	0.5	0.27946	0.93058 (max o	6	4036; 3598; 3164; 25	-0.6003043	-0.0909357		1.0
	ZINC03839344	-8.5	441.511	-0.0487	9	2	309.48	0.35353	131.99	9.4759	459.9676358652	0.28045	6.386	-0.17365	0.48387	0.29521	0.91599 (max o	9	4036; 3818; 2749; 43	-0.6082166	-0.0565716		1.0
	ZINC03839808	-8.5	409.445	-0.2199	9	2	304.81	0.31997	113.52	6.4463	459.9676358652	0.2898	6.5572	-0.7588	0.63333	0.40089	0.88025 (max o	4	4045; 3599; 2754; 22	-0.3346546	-0.2784502		1.0
	ZINC03840148	-8.5	406.413	-0.4312	9	6	294.15	0.3779	139.79	1.2704	459.9676358652	0.29979	6.7685	-1.4383	0.55172	0.48587	0.8991 (max of	4	3606; 2757; 2005; 17	-0.2659641	-0.5465998		1.0
	ZINC03840174	-8.5	408.453	-1.7091	10	6	300.97	0.4239	174.87	4.9782	459.9676358652	0.29979	8.0464	-5.7009	0.51724	0.48296	0.89631 (max o	4	5334; 4042; 2382; 33	-0.4454227	-0.1885739		1.0
	ZINC03861779	-8.5	385.209	1.2442	6	4	234.65	0.30854	99.38	-6.4693	459.9676358652	0.378	5.0931	3.2915	0.56522	0.36162	0.93686 (max o	7	4508; 4066; 3779; 35	0.2580504	0.91528195		1.0
	ZINC03977910	-8.5	394.437	0.167	6	4	262.92	0.29849	115.06	2.4849	459.9676358652	0.3105	6.1703	0.53784	0.46429	0.31582	0.93385 (max o	4	3621; 2833; 2774; 20	0.51241326	0.6294894		1.0
	ZINC03985227	-8.5	406.417	-0.3203	8	1	296.65	0.28653	109.85	6.5925	459.9676358652	0.2898	6.6576	-1.1052	0.4	0.47397	0.95837	1	1255	0.7734427	-0.0519380		1.0
	ZINC04085311	-8.5	381.363	-1.005	8	1	274.84	0.32168	108	6.2661	459.9676358652	0.3105	7.3423	-3.2367	0.53571	0.34114	0.96519 (max o	3	4525; 2787; 1124	-0.0443174	-0.6710403		1.0
	ZINC04098354	-8.5	374.341	-2.4822	10	5	250.94	0.47238	155.14	-5.3489	459.9676358652	0.33438	8.8195	-7.4232	0.46154	0.42276	0.94951	1	5265	-0.0598095	0.8286736		1.0
	ZINC04151636	-8.5	446.423	-2.7779	14	4	323.04	0.52297	222.53	0.71546	459.9676358652	0.27169	9.1152	-10.225	0.40625	0.44707		0		0.00752881	0.9521139		1.0
	ZINC04201138	-8.5	449.506	-1.8648	11	2	322.93	0.33682	128.36	7.4561	459.9676358652	0.27169	8.2021	-6.8638	0.5625	0.3741	0.93422 (max o	5	2096; 2002; 78; 699;	-0.5706268	0.01445068		1.0
	ZINC04217500	-8.5	405.414	-2.7115	12	4	293.94	0.46081	170.59	8.2014	459.9676358652	0.29979	9.0488	-9.0446	0.44828	0.5137	0.91292 (max o	3	5375; 1708; 1709	-0.0349910	0.7509929		1.0
	ZINC04217500	-8.5	406.422	-4.6893	12	5	282.97	0.43991	171.84	7.6003	459.9676358652	0.29979	11.027	-15.642	0.44828	0.50817	0.91903 (max o	3	5379; 1707; 1709	-0.0337658	0.77186596		1.0
	ZINC04217500	-8.5	406.422	-4.6893	12	5	282.97	0.43991	171.84	7.7426	459.9676358652	0.29979	11.027	-15.642	0.44828	0.5137	0.91903 (max o	3	5380; 1707; 1708	-0.0169059	0.73762125		1.0
	ZINC04222230	-8.5	383.427	-1.0976	8	3	282.22	0.30983	108.26	6.0959	459.9676358652	0.3105	7.4349	-3.535	0.57143	0.33171	0.88178 (max o	2	5390; 4230	0.7292411	-0.5549867		1.0
	ZINC04247588	-8.5	392.359	-1.4089	9	4	258.93	0.4307	150.59	-1.9479	459.9676358652	0.3105	7.7462	-4.5375	0.46429	0.40469	0.92774 (max o	7	140; 141; 263; 264; 7	-0.2912656	0.6668452		1.0
	ZINC04247588	-8.5	392.359	-1.4757	9	5	258.48	0.43168	153.75	-1.9031	459.9676358652	0.3105	7.813	-4.7527	0.46429	0.39644	0.92464 (max o	7	140; 141; 263; 264; 7	-0.3078754	0.67911065		1.0
	ZINC04260848	-8.5	380.467	-1.5327	9	2	300.13	0.33619	91.94	-20.548	459.9676358652	0.322	7.87	-4.7599	0.62963	0.35887	0.91109 (max o	2	3233; 2442	-0.2069467	0.00679913		1.0

Structure	Molecule Name	affinity	Total Molweight	cLogP	H-Acceptors	H-Donors	Total Surface Area	Relative PSA	Polar Surface Area	Druglikeness	Kd (nM) @ 20°C	LE from K...	LLE from Kd ...	LELP from Kd (n...	Shape Index	Molecular...	Neighbor Si...	Neig...	Neighbor	Neighbor ...	Neighbor ...	Ring Systems	frequency
	ZINC04270619	-8.5	371.396	0.3731	9	1	267.01	0.3531	100.39	3.4295	459.9676358652	0.322	5.9642	1.1587	0.55556	0.29208	0.91768 (max o	5	3252; 2815; 2445; 17	-0.1941707	-0.3444208		1.0
	ZINC04277766	-8.5	373.432	0.1273	7	0	266.72	0.30043	98.4	7.5322	459.9676358652	0.33438	6.21	0.3807	0.57692	0.37599	0.89608	1	4975	-0.6625966	0.20108195		1.0
	ZINC05414660	-8.5	442.499	-0.4533	10	2	321.56	0.38711	152.84	6.634	459.9676358652	0.28045	6.7906	-1.6163	0.45161	0.44813	0.96971 (max o	3	4569; 3694; 2089	-0.0173969	0.20932908		1.0
	ZINC05434236	-8.5	443.502	0.1486	10	2	330.18	0.28993	111.29	4.265	459.9676358652	0.27169	6.1887	0.54695	0.4375	0.53788	0.96186 (max o	4	3699; 961; 1176; 143	-0.0913083	0.23532893		1.0
	ZINC06018412	-8.5	353.370	-1.2185	8	4	243.05	0.37342	109.64	-1.1456	459.9676358652	0.34776	7.5558	-3.5039	0.52	0.29163	0.84105	1	1756	-0.4737947	-0.8388616		1.0
	ZINC06018412	-8.5	354.378	-1.4796	8	4	239.33	0.36368	114.22	-4.0231	459.9676358652	0.34776	7.8169	-4.2547	0.52	0.28446	0.84105	1	1755	-0.4631257	-0.8250662		1.0
	ZINC08624460	-8.5	430.527	-1.9445	9	3	340.17	0.29159	105.15	-1.2651	459.9676358652	0.28045	8.2818	-6.9335	0.6129	0.55591	0.92602 (max o	5	5450; 4605; 3738; 25	0.24221107	-0.7098784		1.0
	ZINC08624478	-8.5	446.526	-2.7665	10	3	350.17	0.31182	114.38	-1.9602	459.9676358652	0.27169	9.1038	-10.183	0.625	0.57823	0.92602 (max o	5	5450; 4605; 3738; 25	0.24298307	-0.6905381		1.0
	ZINC08636503	-8.5	421.473	0.488	9	3	286.99	0.39461	138.15	5.4526	459.9676358652	0.29979	5.8493	1.6278	0.51724	0.38337	0.97415 (max o	8	3736; 3296; 2889; 28	-0.5369679	0.31959698		1.0
	ZINC08643471	-8.5	366.412	0.008	8	3	273.23	0.33452	112.24	3.3888	459.9676358652	0.33438	6.3293	0.023925	0.61538	0.52784	0.91999 (max o	3	4183; 3732; 2925	0.23171735	-0.4506765		1.0
	ZINC08739976	-8.5	413.433	0.3389	10	4	291.34	0.36624	132.44	3.7769	459.9676358652	0.2898	5.9984	1.1694	0.46667	0.36277	0.9707 (max of	4	2891; 935; 1074; 146	0.29535297	-0.4988236		1.0
	ZINC09109508	-8.5	431.472	0.2984	10	5	305.24	0.44526	180.52	2.0441	459.9676358652	0.2898	6.0389	1.0297	0.5	0.41559	0.92821 (max o	4	4202; 3780; 2533; 21	0.32871205	-0.5499047		1.0
	ZINC12296598	-8.5	381.391	-3.2423	9	1	268.94	0.28006	104.23	-6.7626	459.9676358652	0.3105	9.5796	-10.442	0.53571	0.40845	0.9218 (max of	2	2140; 1791	0.01606807	0.88395804		1.0
	ZINC12296598	-8.5	380.383	-1.2645	9	0	279.91	0.30828	102.98	-6.0125	459.9676358652	0.3105	7.6018	-4.0725	0.53571	0.40845	0.9218 (max of	3	3079; 2141; 1790	-0.0134089	0.9027928		1.0
	ZINC12481440	-8.5	430.547	-1.07	8	2	352.06	0.25436	97.22	5.0291	459.9676358652	0.28045	7.4073	-3.8153	0.58065	0.61535	0.95267 (max o	6	6499; 4644; 3761; 29	-0.0441801	-0.2442196		1.0
	ZINC12603934	-8.5	443.519	-1.4941	10	4	318.59	0.35874	145.81	-3.3285	459.9676358652	0.2898	7.8314	-5.1556	0.63333	0.57355	0.91522 (max o	5	6750; 5477; 5066; 46	0.21402162	-0.3584630		1.0
	ZINC13424736	-8.5	436.368	-0.5213	11	7	286.98	0.46951	186.37	-3.8607	459.9676358652	0.28045	6.8586	-1.8588	0.51613	0.28238	0.90055 (max o	2	1944; 202	-0.2828134	-0.64852		1.0
	ZINC15673216	-8.5	389.454	0.5495	9	1	291.68	0.29591	98.99	5.7948	459.9676358652	0.3105	5.7878	1.7697	0.57143	0.49769	0.87576 (max o	4	5518; 4262; 3372; 33	-0.7321377	-0.3115883		1.0
	ZINC15675209	-8.5	417.911	0.5449	6	3	298.73	0.20262	86.61	1.6475	459.9676358652	0.29979	5.7924	1.8176	0.58621	0.41601	0.89093 (max o	2	6756; 2154	0.1705731	-0.4563292		1.0
	ZINC15675227	-8.5	448.518	-0.4887	10	3	330.92	0.28524	114.81	5.1476	459.9676358652	0.27169	6.826	-1.7988	0.625	0.56695	0.89402 (max o	3	106; 466; 1764	-0.1005005	-0.5813261		1.0
	ZINC15675712	-8.5	367.408	-2.0433	8	2	270.23	0.29941	112.22	4.2409	459.9676358652	0.322	8.3806	-6.3457	0.51852	0.4485	0.88025	1	2571	0.71452236	-0.5728326		1.0
	ZINC15675902	-8.5	410.433	-1.5267	10	2	298.41	0.3516	142.18	3.3939	459.9676358652	0.2898	7.864	-5.2681	0.5	0.50144	0.90825 (max o	4	3063; 1834; 1239; 18	0.2695932	-0.1018940		1.0
	ZINC15675902	-8.5	409.425	-1.3378	10	1	309.38	0.37459	140.93	3.8739	459.9676358652	0.2898	7.6751	-4.6163	0.5	0.50144	0.97533 (max o	2	1834; 1832	0.26882216	-0.0847229		1.0
	ZINC15675902	-8.5	409.425	-1.3378	10	1	309.38	0.37459	140.93	3.8739	459.9676358652	0.2898	7.6751	-4.6163	0.5	0.50144	0.97533 (max o	2	1832; 1833	0.2842229	-0.0691122		1.0
	ZINC15675954	-8.5	396.382	-2.5143	10	1	299.45	0.38377	137.27	3.7989	459.9676358652	0.29979	8.8516	-8.3868	0.55172	0.48719	0.97415 (max o	5	496; 652; 1837; 1593	0.32056287	-0.1870035		1.0
	ZINC15707918	-8.5	390.462	-3.4687	9	4	313.07	0.35794	113.86	2.8443	459.9676358652	0.3105	9.806	-11.171	0.64286	0.50945	0.86695	1	3329	-0.2867448	0.22055292		1.0
	ZINC15708611	-8.5	449.598	-2.3097	9	4	357.34	0.35613	126.38	8.4441	459.9676358652	0.28045	8.647	-8.2357	0.6129	0.55728	0.89025 (max o	2	3816; 1884	-0.1936474	0.13776237		1.0
	ZINC15709201	-8.5	382.391	-0.9147	7	1	281.79	0.29036	96.92	1.9279	459.9676358652	0.3105	7.252	-2.9459	0.57143	0.40811	0.83335	1	908	-0.2964656	-0.2824784		1.0
	ZINC20112089	-8.5	379.519	-0.8686	6	1	281.4	0.22026	67.91	-1.6832	459.9676358652	0.322	7.2059	-2.6975	0.55556	0.47182	0.94302 (max o	3	3832; 3831; 1533	0.13484825	0.55273676		1.0
	ZINC20463230	-8.5	431.531	-1.1984	8	1	350.6	0.25125	94.42	4.5473	459.9676358652	0.28045	7.5357	-4.2731	0.58065	0.57285	0.95267 (max o	5	5183; 3761; 2982; 10	-0.0736208	-0.2163657		1.0
	ZINC20463230	-8.5	430.523	-1.33	8	0	336.56	0.22002	93.22	9.1612	459.9676358652	0.28045	7.6673	-4.7424	0.58065	0.5763	0.93686 (max o	5	5914; 4643; 3385; 29	-0.1478910	-0.1945537		1.0
	ZINC20464831	-8.5	398.525	-0.4213	6	3	303.38	0.16824	72.78	3.9028	459.9676358652	0.29979	6.7586	-1.4053	0.58621	0.44573	0.96682 (max o	2	3394; 1541	-0.4365068	0.22536036		1.0
	ZINC20464861	-8.5	414.504	-0.2049	8	3	312.55	0.23891	90.2	7.5713	459.9676358652	0.2898	6.5422	-0.70704	0.53333	0.45762	0.92835 (max o	2	6569; 3391	-0.3792942	0.17193952		1.0
	ZINC20464861	-8.5	415.512	-2.3146	8	4	326.59	0.27162	91.4	5.7493	459.9676358652	0.2898	8.6519	-7.9869	0.53333	0.46161	0.93405	1	6570	-0.2786020	-0.56544		1.0
	ZINC20758721	-8.5	386.427	-1.108	8	3	258.57	0.32862	112.55	2.0711	459.9676358652	0.3105	7.4453	-3.5684	0.42857	0.22578	0.98451	1	1881	0.8612862	0.41246894		1.0
	ZINC20758729	-8.5	386.427	-1.108	8	3	258.57	0.32862	112.55	2.0711	459.9676358652	0.3105	7.4453	-3.5684	0.42857	0.21622	0.98451	1	1879	0.8608294	0.4340404		1.0
	ZINC22922315	-8.5	419.524	-5.205	9	3	334.57	0.30544	108.81	8.4696	459.9676358652	0.2898	11.542	-17.961	0.63333	0.51972	0.9036 (max of	9	4347; 4344; 3430; 26	-0.1757130	0.18053314		1.0
	ZINC22922315	-8.5	421.540	-5.1451	9	5	347.32	0.33093	103.69	5.0996	459.9676358652	0.2898	11.482	-17.754	0.63333	0.52791	0.8956 (max of	3	4348; 1884; 1275	-0.1912013	0.161742		1.0
	ZINC22922315	-8.5	420.532	-3.0354	9	4	333.28	0.30275	102.49	9.1926	459.9676358652	0.2898	9.3727	-10.474	0.63333	0.52791	0.89867 (max o	4	4348; 1841; 1882; 18	-0.2107697	0.16927488		1.0
	ZINC22923664	-8.5	423.600	-4.1507	8	4	347.99	0.26702	81.76	5.3245	459.9676358652	0.2898	10.488	-14.323	0.6	0.50894	0.9707 (max of	7	3874; 3488; 3021; 26	-0.1839821	0.5681576		1.0
	ZINC31155157	-8.5	396.391	0.3232	9	2	274.39	0.38383	128.59	3.7448	459.9676358652	0.3105	6.0141	1.0409	0.39286	0.31345	0.88391 (max o	2	7361; 6363	0.80603254	0.19581127		1.0
	ZINC31155346	-8.5	448.419	-2.0505	12	6	308.9	0.44869	176.76	-4.801	459.9676358652	0.28045	8.3878	-7.3114	0.54839	0.45977	0.96262 (max o	5	4359; 2624; 2286; 15	0.5731437	0.48215902		1.0
	ZINC31157223	-8.5	448.423	0.2935	10	6	321.89	0.37789	166.14	-5.8199	459.9676358652	0.27169	6.0438	1.0803	0.5625	0.4201	0.88885	1	493	-0.2789289	0.63367355		1.0

Structure	Molecule Name	affinity	Total Molweight	cLogP	H-Acceptors	H-Donors	Total Surface Area	Relative PSA	Polar Surface Area	Druglikeness	Kd (nM) @ 20°C	LE from K...	LLE from Kd ...	LELP from Kd (n...	Shape Index	Molecular...	Neighbor Si...	Neig...	Neighbor	Neighbor ...	Neighbor ...	Ring Systems	frequency
	ZINC31168006	-8.5	408.402	0.024	9	4	279.86	0.37676	134.91	-7.4042	459.9676358652	0.29979	6.3133	0.080056	0.48276	0.46165	0.9617 (max of	3	1035; 1130; 1293	-0.7718563	-0.3221040		1.0
	ZINC35271788	-8.5	434.396	-0.258	10	7	290.74	0.42904	177.14	-2.4912	459.9676358652	0.28045	6.5953	-0.91995	0.51613	0.39724	0.96502 (max o	4	3460; 3058; 979; 190	-0.7302031	-0.2133023		1.0
	ZINC35442281	-8.5	409.512	-1.6704	8	3	313.82	0.26203	103.83	4.1975	459.9676358652	0.2898	8.0077	-5.764	0.63333	0.455	0.97304 (max o	7	5996; 319; 1298; 159	-0.4708148	0.00795383		1.0
	ZINC35442594	-8.5	393.550	-1.3284	7	3	311.39	0.24192	77.32	1.6475	459.9676358652	0.3105	7.6657	-4.2783	0.57143	0.47227	0.90502 (max o	2	4401; 3490	-0.1050275	0.6055701		1.0
	ZINC38143867	-8.5	424.444	-0.918	9	5	286.03	0.37947	145.91	-11.031	459.9676358652	0.2898	7.2553	-3.1677	0.43333	0.33307	0.85143 (max o	2	3100; 1326	-0.8885255	-0.2386214		1.0
	ZINC40309825	-8.5	363.376	-0.682	8	3	260.21	0.30871	115.29	3.7493	459.9676358652	0.322	7.0193	-2.118	0.44444	0.46019	0.98371 (max o	6	2672; 243; 528; 1329;	-0.1717153	-0.4924868		1.0
	ZINC49543640	-8.5	378.343	-2.9005	9	1	275.59	0.38263	131.7	4.9434	459.9676358652	0.3105	9.2378	-9.3414	0.46429	0.36005	0.95709 (max o	2	2310; 1935	0.5988661	0.31322792		1.0
	ZINC49543640	-8.5	379.351	-1.343	9	2	271.51	0.37339	123.16	4.8497	459.9676358652	0.3105	7.6803	-4.3253	0.46429	0.36005	0.94573 (max o	2	2311; 1934	0.60179454	0.29237708		1.0
	ZINC49600290	-8.5	364.393	-0.3946	7	3	236.37	0.37407	121.13	0.68838	459.9676358652	0.33438	6.7319	-1.1801	0.34615	0.32434	0.92032	1	848	-0.5296569	0.68758357		1.0
	ZINC67902434	-8.5	446.530	-1.1862	10	3	322.57	0.37852	149.04	5.5539	459.9676358652	0.28045	7.5235	-4.2296	0.41935	0.48307	0.90983 (max o	4	4570; 2932; 2082; 14	0.04085689	0.25528017		1.0
	ZINC67912308	-8.5	426.460	-0.9089	9	4	289.11	0.37522	142.75	-1.4771	459.9676358652	0.2898	7.2462	-3.1363	0.36667	0.33229	0.93686	1	3958	0.8749851	-0.3095100		1.0
	ZINC71405293	-8.5	380.511	0.1851	7	2	275.12	0.31488	107.05	2.5564	459.9676358652	0.33438	6.1522	0.55356	0.57692	0.46975	0.86499	1	1148	-0.2074419	0.22641535		1.0
	ZINC71405295	-8.5	383.472	-1.6291	9	3	276.31	0.40219	136.15	5.7961	459.9676358652	0.33438	7.9664	-4.8719	0.5	0.49172	0.89334	1	2932	-0.0337839	0.28115982		1.0
	ZINC72319895	-8.5	375.420	-2.7093	7	2	279.68	0.33642	108.17	0.94539	459.9676358652	0.322	9.0466	-8.414	0.44444	0.35596	0.92418 (max o	3	3113; 3112; 856	-0.1123215	-0.7699274		1.0
	ZINC02098866	-8.4	375.332	-4.204	9	0	277.1	0.37629	136.1	-5.9002	546.1145017766	0.31821	10.467	-13.211	0.51852	0.59901	0.96023 (max o	3	332; 1118; 1357	0.14129983	0.4552538		172.0
	ZINC02159694	-8.4	417.436	-1.4427	9	2	321.24	0.34043	133.86	2.3899	546.1145017766	0.28639	7.7054	-5.0375	0.53333	0.51227	0.95345 (max o	7	4002; 3569; 2727; 32	0.13582891	-0.0182983		172.0
	ZINC02102328	-8.4	389.383	-2.1296	9	2	293.19	0.373	133.86	-0.83321	546.1145017766	0.30685	8.3923	-6.9403	0.60714	0.46856	0.97037 (max o	7	3561; 3133; 2353; 19	0.23179702	0.04608533		172.0
	ZINC02122679	-8.4	403.410	-1.8008	9	2	305.69	0.35775	133.86	2.2568	546.1145017766	0.29627	8.0635	-6.0783	0.58621	0.56834	0.9561 (max of	7	4022; 3569; 547; 870	0.15058933	-0.0349928		172.0
	ZINC02122547	-8.4	417.436	-1.4569	9	2	317.95	0.34395	133.86	2.2568	546.1145017766	0.28639	7.7196	-5.0871	0.56667	0.50227	0.92527 (max o	7	3133; 2727; 2726; 27	0.14372362	1.8981245E		172.0
	ZINC02119475	-8.4	375.332	-3.9865	9	1	281.79	0.3981	144.89	-0.099667	546.1145017766	0.31821	10.249	-12.528	0.59259	0.54441	0.96023 (max o	5	3142; 3134; 2737; 10	0.24022272	0.1000199		172.0
	ZINC02137169	-8.4	389.383	-2.1154	9	2	296.48	0.36886	133.86	-0.45242	546.1145017766	0.30685	8.3781	-6.894	0.60714	0.48164	0.97037 (max o	9	4004; 3578; 3562; 31	0.2526994	0.01046680		172.0
	ZINC12899982	-8.4	375.356	-2.4593	9	2	284.22	0.38477	133.86	-2.9576	546.1145017766	0.31821	8.722	-7.7285	0.62963	0.5469	0.96389 (max o	3	2946; 2545; 2544	0.184354	-0.1224851		172.0
	ZINC12296512	-8.4	375.356	-2.3784	9	2	283.69	0.38549	133.86	0.74536	546.1145017766	0.31821	8.6411	-7.4743	0.66667	0.57732	0.96389 (max o	3	3579; 2946; 1648	0.21562946	-0.0749217		172.0
	ZINC02100485	-8.4	361.329	-2.7165	9	3	266.28	0.42234	144.86	1.1825	546.1145017766	0.33045	8.9792	-8.2206	0.61538	0.58026	0.96998 (max o	5	4014; 3154; 2738; 27	0.1200065	0.02762592		172.0
	ZINC02100601	-8.4	403.410	-1.661	9	2	310.24	0.3525	133.86	-2.3536	546.1145017766	0.29627	7.9237	-5.6065	0.62069	0.48459	0.98037 (max o	11	5301; 4007; 4004; 35	0.28263816	0.02841019		172.0
	ZINC02100859	-8.4	390.371	-2.4148	10	4	288.8	0.44228	170.88	2.7316	546.1145017766	0.30685	8.6775	-7.8697	0.60714	0.57007	0.98795 (max o	8	5299; 4010; 3584; 35	0.04361187	0.19099197		172.0
	ZINC02105748	-8.4	390.371	-2.6274	10	3	294.24	0.42357	159.88	-3.7367	546.1145017766	0.30685	8.8901	-8.5626	0.60714	0.53957	0.9678 (max of	5	5294; 4006; 3580; 27	0.25426003	0.13798118		172.0
	ZINC02530808	-8.4	390.442	-4.2196	10	6	281.71	0.39793	175.78	-3.5713	546.1145017766	0.30685	10.482	-13.752	0.53571	0.55489		0		-0.9438355	0.26395312		172.0
	ZINC13527614	-8.4	422.206	-10.528	14	3	268.71	0.66291	251.48	-31.197	546.1145017766	0.31821	16.791	-33.086	0.51852	0.52117	0.94905 (max o	3	3360; 1793; 2174	-0.5659094	0.4735422		67.0
	ZINC13434875	-8.4	409.187	-9.2546	14	3	258.68	0.67207	237.65	-43.413	546.1145017766	0.33045	15.517	-28.006	0.53846	0.49591	0.94326 (max o	8	3283; 2872; 2553; 25	-0.5645284	0.37772748		67.0
	ZINC13527614	-8.4	423.214	-9.2808	14	4	267.53	0.66142	248.65	-32.721	546.1145017766	0.31821	15.544	-29.166	0.51852	0.52117	0.94905 (max o	4	6792; 3358; 1792; 21	-0.552053	0.4563775		67.0
	ZINC05606971	-8.4	364.313	-2.0709	12	4	226.18	0.64152	197.08	-6.7093	546.1145017766	0.35799	8.3336	-5.7848	0.5	0.38642	0.92619 (max o	3	5429; 4136; 3703	-0.7614031	0.58774143		67.0
	ZINC01708083	-8.4	351.318	-0.8696	11	2	249.96	0.50088	147.13	0.815	546.1145017766	0.34367	7.1323	-2.5304	0.48	0.37151	0.93686 (max o	4	3247; 3234; 2458; 17	0.11303105	-0.6874289		44.0
	ZINC13538250	-8.4	440.177	-12.957	16	4	271.51	0.74472	276.39	-33.279	546.1145017766	0.30685	19.22	-42.227	0.42857	0.44429	0.93985 (max o	4	4454; 2701; 1510; 21	-0.3719376	-0.3006357		44.0
	ZINC13538250	-8.4	439.169	-14.205	16	3	272.69	0.74583	279.22	-23.349	546.1145017766	0.30685	20.467	-46.293	0.42857	0.46052	0.93686 (max o	5	4933; 4453; 2700; 21	-0.3599915	-0.3451022		44.0
	ZINC12501413	-8.4	361.206	-8.4657	13	4	230.21	0.69893	217.22	-28.905	546.1145017766	0.35799	14.728	-23.648	0.5	0.45058	0.92619 (max o	6	6997; 4933; 4932; 29	-0.3058626	-0.3669716		44.0
	ZINC38571100	-8.4	433.483	-1.9254	10	4	325.62	0.36076	150.9	3.4686	546.1145017766	0.27715	8.1881	-6.9471	0.41935	0.55687	0.99016	1	2302	0.28485882	-0.7517138		42.0
	ZINC38571099	-8.4	433.483	-1.9254	10	4	325.62	0.36076	150.9	3.4686	546.1145017766	0.27715	8.1881	-6.9471	0.41935	0.55483	0.99016	1	2303	0.27423492	-0.7356515		42.0
	ZINC20758579	-8.4	404.378	-5.2533	11	3	297.79	0.44222	170.8	7.0806	546.1145017766	0.29627	11.516	-17.732	0.48276	0.56305	0.96385 (max o	10	3823; 3801; 2903; 26	0.05375614	-0.5964560		42.0
	ZINC25076468	-8.4	391.403	-2.9228	10	4	289.86	0.40526	150.9	4.722	546.1145017766	0.30685	9.1855	-9.5253	0.46429	0.54719	0.98866 (max o	11	3823; 3822; 3801; 37	-0.0339068	-0.6180317		42.0
	ZINC12889504	-8.4	418.429	-3.1207	11	4	312.54	0.42452	173.76	8.6998	546.1145017766	0.28639	9.3834	-10.897	0.46667	0.56024	0.97757 (max o	12	5166; 5165; 5077; 37	0.05194341	-0.6277095		42.0
	ZINC20758825	-8.4	418.405	-4.7989	11	3	311.55	0.42269	170.8	8.0771	546.1145017766	0.28639	11.062	-16.757	0.46667	0.56864	0.97757 (max o	15	5166; 5165; 5077; 43	0.05649314	-0.6653586		42.0
	ZINC20758382	-8.4	408.433	-0.2642	8	2	307.05	0.30259	118.64	5.5436	546.1145017766	0.28639	6.5269	-0.92252	0.4	0.50667	0.97975 (max o	11	3859; 3748; 3346; 30	0.64300084	-0.0773518		36.0

Structure	Molecule Name	affinity	Total Molweight	cLogP	H-Acceptors	H-Donors	Total Surface Area	Relative PSA	Polar Surface Area	Druglikeness	Kd (nM) @ 20°C	LE from K...	LLE from Kd ...	LELP from Kd (n...	Shape Index	Molecular...	Neighbor Si...	Neig...	Neighbor	Neighbor ...	Neighbor ...	Ring Systems	frequency
	ZINC12884241	-8.4	437.431	-1.7983	10	2	321.16	0.35281	152.94	5.2242	546.1145017766	0.26849	8.061	-6.6978	0.40625	0.50724	0.97948 (max o	12	5034; 4962; 4522; 40	0.7253849	-0.0242709		36.0
	ZINC20757748	-8.4	403.414	-2.168	10	3	302.52	0.38811	147.74	6.1083	546.1145017766	0.29627	8.4307	-7.3178	0.51724	0.57404	0.96428 (max o	8	5163; 4761; 4678; 46	0.3444393	0.7101863		36.0
	ZINC04090204	-8.4	372.400	-1.7332	8	1	273.91	0.31032	109.85	6.3578	546.1145017766	0.31821	7.9959	-5.4467	0.51852	0.50239	0.97658 (max o	12	4091; 3209; 3210; 32	0.09389477	-0.8803099		29.0
	ZINC12889352	-8.4	374.372	-2.9733	9	2	268	0.36604	130.08	4.9085	546.1145017766	0.31821	9.236	-9.3438	0.51852	0.49786	0.98806 (max o	13	4091; 3209; 3210; 32	0.11423353	-0.8724831		29.0
	ZINC04090332	-8.4	386.427	-1.2788	8	1	287.67	0.29548	109.85	7.2507	546.1145017766	0.30685	7.5415	-4.1676	0.53571	0.51907	0.97037 (max o	13	4091; 3642; 3209; 32	0.11596783	-0.853742		29.0
	ZINC08791779	-8.4	443.478	-1.808	10	2	331.96	0.34134	152.94	-1.3332	546.1145017766	0.26849	8.0707	-6.7339	0.59375	0.51319	0.971 (max of 8	8	5464; 4632; 3749; 36	0.18222271	-0.9122413		29.0
	ZINC04000355	-8.4	362.361	-2.3184	9	2	276.06	0.3738	121.72	2.5814	546.1145017766	0.33045	8.5811	-7.0159	0.57692	0.37329	0.93686 (max o	5	4425; 3185; 3184; 11	-0.2166432	0.5201221		28.0
	ZINC38570352	-8.4	404.441	-1.2864	9	2	311.82	0.33093	121.72	4.9671	546.1145017766	0.29627	7.5491	-4.3421	0.48276	0.3914	0.93985 (max o	5	5742; 4501; 3942; 31	-0.2025518	0.5616943		28.0
	ZINC03073318	-8.4	361.206	-8.4657	13	4	230.21	0.69893	217.22	-28.905	546.1145017766	0.35799	14.728	-23.648	0.5	0.43529	0.93686 (max o	10	5735; 4933; 4932; 34	-0.351314	-0.3955643		26.0
	ZINC12153524	-8.4	353.302	-2.8445	9	5	246.47	0.47008	167.58	0.33063	546.1145017766	0.34367	9.1072	-8.2769	0.56	0.41449	0.97396 (max o	13	3467; 3051; 2623; 24	-0.1808293	-0.8509424		25.0
	ZINC04096248	-8.4	353.302	-2.8445	9	5	246.47	0.47008	167.58	0.33063	546.1145017766	0.34367	9.1072	-8.2769	0.56	0.41449	0.98188 (max o	17	5090; 3467; 3051; 30	-0.1710780	-0.8027510		25.0
	ZINC17263588	-8.4	353.302	-2.8445	9	5	246.47	0.47008	167.58	0.33063	546.1145017766	0.34367	9.1072	-8.2769	0.56	0.41449	0.96243 (max o	14	7407; 3467; 3051; 26	-0.2033340	-0.8501891		25.0
	ZINC04095885	-8.4	367.329	-2.5688	9	4	262.38	0.42976	156.58	0.39255	546.1145017766	0.33045	8.8315	-7.7736	0.57692	0.41317	0.97791 (max o	8	4797; 3158; 3032; 30	-0.2136342	-0.7616491		25.0
	ZINC31155189	-8.4	367.329	-2.5688	9	4	262.38	0.42976	156.58	0.39255	546.1145017766	0.33045	8.8315	-7.7736	0.61538	0.41317	0.94338 (max o	6	3443; 3158; 3051; 30	-0.2458107	-0.7771055		25.0
	ZINC31157010	-8.4	403.359	-4.4709	11	4	272.18	0.48777	175.04	-5.4979	546.1145017766	0.30685	10.734	-14.57	0.46429	0.4044	0.92124	1	3456	0.6900227	-0.6203947		25.0
	ZINC03978795	-8.4	375.349	-4.4746	10	5	249.52	0.49223	168.97	-1.6392	546.1145017766	0.33045	10.737	-13.541	0.46154	0.37855	0.94622 (max o	6	5674; 3707; 3445; 32	-0.6569213	0.06882904		25.0
	ZINC02105287	-8.4	416.433	-1.955	10	4	293.78	0.37593	168.4	1.7539	546.1145017766	0.28639	8.2177	-6.8264	0.6	0.50852	0.93686 (max o	5	550; 862; 1085; 1086	0.2405003	0.06617233		24.0
	ZINC02099358	-8.4	358.369	-0.8357	7	1	265.98	0.31905	104.76	0.79874	546.1145017766	0.33045	7.0984	-2.529	0.53846	0.4627	0.9543 (max of	4	692; 870; 1086; 1359	0.1921493	0.03979180		24.0
	ZINC02093563	-8.4	373.340	-2.784	9	2	273.19	0.40031	133.86	-2.0413	546.1145017766	0.31821	9.0467	-8.7489	0.62963	0.51071	0.91482 (max o	4	1986; 692; 870; 1083	0.17719668	0.02481564		24.0
	ZINC02146715	-8.4	387.367	-2.4401	9	2	285.45	0.38311	133.86	-2.0422	546.1145017766	0.30685	8.7028	-7.9522	0.60714	0.43424	0.9218 (max of	8	3581; 3153; 3133; 86	0.21249135	0.00959479		24.0
	ZINC04073459	-8.4	366.365	0.1494	8	4	260.16	0.36685	125.68	-7.351	546.1145017766	0.33045	6.1133	0.45211	0.53846	0.43062	0.91059 (max o	4	2407; 258; 563; 708	0.3002568	0.4951317		24.0
	ZINC03831424	-8.4	376.368	-2.0663	10	5	264.4	0.44066	155.05	6.1929	546.1145017766	0.31821	8.329	-6.4935	0.44444	0.47924	0.96885 (max o	18	5314; 5297; 4912; 49	0.42287612	0.7953905		24.0
	ZINC08879760	-8.4	358.369	-0.5377	7	1	262.43	0.30503	106.97	4.3186	546.1145017766	0.33045	6.8004	-1.6272	0.53846	0.54079	0.96998 (max o	4	606; 646; 1472; 1777	0.24486399	0.20267603		22.0
	ZINC02158921	-8.4	372.396	-0.262	7	0	278.34	0.27646	95.97	4.9812	546.1145017766	0.31821	6.5247	-0.82335	0.55556	0.53029	0.94622 (max o	7	2729; 2036; 548; 646	0.29210746	0.17730084		22.0
	ZINC08764399	-8.4	388.395	-0.6759	8	0	288.34	0.30155	105.2	4.3706	546.1145017766	0.30685	6.9386	-2.2027	0.53571	0.53237	0.97288 (max o	7	2850; 2408; 548; 950	0.30624616	0.21636747		22.0
	ZINC08790454	-8.4	432.451	-1.866	10	2	322.59	0.35001	137.1	-2.4605	546.1145017766	0.27715	8.1287	-6.7328	0.54839	0.46813	0.96971 (max o	11	4195; 3943; 3202; 24	0.5072653	0.24087627		22.0
	ZINC38570246	-8.4	446.478	-1.5067	10	2	333.59	0.33847	137.1	-1.1357	546.1145017766	0.26849	7.7694	-5.6117	0.53125	0.46457	0.971 (max of 1	12	6226; 5250; 4194; 39	0.50456536	0.1855204		22.0
	ZINC38570096	-8.4	446.478	-1.5067	10	2	333.59	0.33847	137.1	-0.50421	546.1145017766	0.26849	7.7694	-5.6117	0.5625	0.47465	0.97319 (max o	12	6226; 5250; 4195; 41	0.5474202	0.17219071		22.0
	ZINC13424731	-8.4	448.379	-0.0011999	11	7	297.4	0.45306	186.37	-3.6679	546.1145017766	0.26849	6.2639	-0.0044691	0.40625	0.43252	0.96186 (max o	7	3174; 2768; 2457; 11	-0.6957974	0.39917198		21.0
	ZINC04349434	-8.4	448.379	-0.0011999	11	7	297.4	0.45306	186.37	-3.6679	546.1145017766	0.26849	6.2639	-0.0044691	0.40625	0.43293	0.96186 (max o	10	4260; 3174; 2768; 24	-0.6869975	0.3805217		21.0
	ZINC13424730	-8.4	448.379	-0.0011999	11	7	297.4	0.45306	186.37	-3.6679	546.1145017766	0.26849	6.2639	-0.0044691	0.40625	0.43293	0.96186 (max o	8	4260; 3174; 2768; 24	-0.7198549	0.36283132		21.0
	ZINC20465489	-8.4	404.451	-0.8662	6	3	300.44	0.20267	60.61	-3.3273	546.1145017766	0.30685	7.1289	-2.8229	0.5	0.52217	0.94605 (max o	4	5566; 4310; 1003; 18	-0.4184454	0.12866002		21.0
	ZINC31161561	-8.4	431.440	-0.1614	9	4	308.3	0.35641	142.39	0.6376	546.1145017766	0.27715	6.4241	-0.58235	0.51613	0.44546	0.97199 (max o	9	4134; 3362; 2967; 25	-0.2795668	-0.7568378		19.0
	ZINC05464462	-8.4	431.440	-0.1614	9	4	308.3	0.35641	142.39	0.6376	546.1145017766	0.27715	6.4241	-0.58235	0.51613	0.44611	0.97199 (max o	8	4577; 4392; 4134; 34	-0.3665540	-0.7516824		19.0
	ZINC08254712	-8.4	359.449	0.5122	6	4	266.18	0.34247	119.25	4.8502	546.1145017766	0.34367	5.7505	1.4904	0.6	0.38345	0.97396 (max o	7	5483; 4229; 3716; 28	-0.4682301	0.34397197		17.0
	ZINC11666971	-8.4	430.408	0.2593	9	4	302.4	0.34868	134.91	-3.4398	546.1145017766	0.27715	6.0034	0.93559	0.58065	0.4063	0.95771 (max o	8	362; 582; 604; 685; 7	0.27425393	0.6154602		16.0
	ZINC36369723	-8.4	447.439	-0.5203	10	5	320.61	0.3591	156.99	5.6773	546.1145017766	0.26849	6.783	-1.9379	0.59375	0.56733	0.971 (max of 3	3	5243; 3515; 3514	0.6322887	-0.6798753		16.0
	ZINC12888810	-8.4	449.442	-1.4649	10	4	325.31	0.36888	154.66	4.2777	546.1145017766	0.26035	7.7276	-5.6266	0.39394	0.46523	0.99099 (max o	10	2540; 2534; 290; 302	0.6897221	-0.1912522		14.0
	ZINC05434132	-8.4	390.414	0.2849	8	2	286.95	0.28639	98.82	3.9439	546.1145017766	0.30685	5.9778	0.92848	0.5	0.52435	0.95983 (max o	9	4992; 3256; 2861; 28	0.05553927	0.3262493		14.0
	ZINC15709542	-8.4	383.447	-1.2639	10	3	293.08	0.35362	120.08	6.2697	546.1145017766	0.31821	7.5266	-3.9719	0.48148	0.57201	0.96064 (max o	5	7328; 5422; 5011; 24	-0.0960123	0.2880206		13.0
	ZINC03874671	-8.4	436.412	0.055	10	7	305.5	0.40831	177.14	-4.8731	546.1145017766	0.27715	6.2077	0.19845	0.51613	0.43296	0.97642 (max o	12	5085; 4111; 3799; 36	0.7873511	-0.2765342		13.0
	ZINC04252704	-8.4	436.412	0.055	10	7	305.5	0.40831	177.14	-4.8731	546.1145017766	0.27715	6.2077	0.19845	0.51613	0.43255	0.95771 (max o	11	6144; 5085; 3799; 36	0.7789898	-0.314331		13.0
	ZINC03840049	-8.4	380.375	-1.0217	9	6	275.69	0.41703	153.78	0.46576	546.1145017766	0.31821	7.2844	-3.2108	0.55556	0.51741	0.92394 (max o	5	3606; 2757; 1101; 16	-0.2479629	-0.5349582		12.0

Structure	Molecule Name	affinity	Total Molweight	cLogP	H-Acceptors	H-Donors	Total Surface Area	Relative PSA	Polar Surface Area	Druglikeness	Kd (nM) @ 20°C	LE from K...	LLE from Kd ...	LELP from Kd (n...	Shape Index	Molecular...	Neighbor Si...	Neig...	Neighbor	Neighbor ...	Neighbor ...	Ring Systems	frequency
	ZINC04221820	-8.4	419.413	-3.6765	11	6	308.38	0.46141	193.91	5.4078	546.1145017766	0.28639	9.9392	-12.837	0.56667	0.54139	0.94768 (max o	4	3602; 2429; 1705; 17	-0.1784612	-0.5531341		12.0
	ZINC38570553	-8.4	404.441	-2.0099	9	2	308.07	0.33405	127.87	5.8064	546.1145017766	0.29627	8.2726	-6.7841	0.58621	0.47459	0.96682 (max o	7	6658; 6425; 5752; 45	0.5089624	0.28396353		12.0
	ZINC08627150	-8.4	437.491	-1.022	10	3	327.36	0.30804	120.8	5.7431	546.1145017766	0.27715	7.2847	-3.6875	0.6129	0.56209	0.95318 (max o	5	4171; 2880; 2112; 11	-0.0512304	-0.5497551		11.0
	ZINC08636299	-8.4	443.519	-0.6432	10	3	314.43	0.33833	137.02	7.8927	546.1145017766	0.28639	6.9059	-2.2459	0.56667	0.56024	0.91934 (max o	5	4171; 2987; 2880; 14	-0.0207934	-0.5241349		11.0
	ZINC31158780	-8.4	432.424	-0.1548	9	6	295.15	0.37825	156.91	-2.8255	546.1145017766	0.27715	6.4175	-0.55854	0.35484	0.3838	0.88414 (max o	2	2049; 2050	-0.5162803	0.655818		11.0
	ZINC04214775	-8.4	418.397	-1.026	9	7	280.74	0.40871	167.91	-3.0467	546.1145017766	0.28639	7.2887	-3.5825	0.36667	0.39345	0.98188 (max o	10	3470; 3364; 2823; 28	-0.5201821	0.63538307		11.0
	ZINC04214775	-8.4	418.397	-1.026	9	7	280.74	0.40871	167.91	-3.0467	546.1145017766	0.28639	7.2887	-3.5825	0.36667	0.39345	0.98188 (max o	10	3470; 3364; 2823; 28	-0.5371333	0.6472936		11.0
	ZINC15675983	-8.4	429.456	-2.0969	10	2	304.47	0.39886	163.82	5.3596	546.1145017766	0.28639	8.3596	-7.3218	0.56667	0.49383	0.95872 (max o	6	651; 792; 2193; 2194	0.39127034	-0.1213190		10.0
	ZINC04237069	-8.4	427.500	-0.827	7	0	310.87	0.28304	115.28	5.5852	546.1145017766	0.28639	7.0897	-2.8877	0.63333	0.51803	0.96832 (max o	7	4971; 4120; 2436; 35	-0.5061417	-0.3452463		10.0
	ZINC03840170	-8.4	445.874	-0.4558	9	6	310.22	0.37589	161.98	3.3756	546.1145017766	0.28639	6.7185	-1.5915	0.5	0.48508	0.9426 (max of	4	433; 884; 1104; 1381	-0.3862132	-0.5270307		10.0
	ZINC20463691	-8.4	420.464	-0.2411	10	3	303.95	0.32637	112.6	5.9813	546.1145017766	0.28639	6.5038	-0.84186	0.6	0.47978	0.96092 (max o	6	3838; 3762; 3428; 33	-0.6341045	-0.2865705		10.0
	ZINC35442250	-8.4	359.492	-0.3751	6	3	279.8	0.21548	78.05	5.2966	546.1145017766	0.33045	6.6378	-1.1351	0.61538	0.45498	0.9615 (max of	6	3485; 3436; 3064; 30	-0.5125782	0.03435352		10.0
	ZINC04236076	-8.4	393.466	-0.3546	7	2	307.73	0.2446	74.16	5.8706	546.1145017766	0.29627	6.6173	-1.1969	0.55172	0.37439	0.9427 (max of	4	443; 1138; 2054; 205	-0.2773037	0.30358127		9.0
	ZINC04235944	-8.4	379.439	-0.4795	7	2	278.87	0.23459	86.33	5.9722	546.1145017766	0.30685	6.7422	-1.5627	0.57143	0.3588	0.9427 (max of	6	4099; 3659; 443; 205	-0.2419276	0.29843277		9.0
	ZINC04235949	-8.4	413.884	0.1265	7	2	294.29	0.2223	86.33	5.9991	546.1145017766	0.29627	6.1362	0.42698	0.58621	0.36369	0.9427 (max of	6	4099; 3659; 2056; 72	-0.2603399	0.29014245		9.0
	ZINC03055309	-8.4	446.407	-0.0864	10	5	308.75	0.38394	155.14	-3.4398	546.1145017766	0.26849	6.3491	-0.3218	0.5625	0.4382	0.98343 (max o	11	2890; 506; 569; 571;	0.19994672	0.6638808		9.0
	ZINC03983954	-8.4	420.413	0.7702	9	6	302.64	0.35884	149.07	-4.1548	546.1145017766	0.28639	5.4925	2.6893	0.56667	0.42608	0.98188 (max o	7	3902; 3901; 2634; 11	-0.5477263	0.7712595		9.0
	ZINC03881962	-8.4	390.387	0.8402	8	6	280.38	0.35167	139.84	-4.3466	546.1145017766	0.30685	5.4225	2.7382	0.57143	0.42351	0.9678 (max of	7	4139; 4140; 2846; 13	-0.5120007	0.76722986		9.0
	ZINC04257736	-8.4	420.413	0.222	9	5	301.3	0.36024	145.91	-3.6574	546.1145017766	0.28639	6.0407	0.77517	0.6	0.47551	0.95053 (max o	7	2909; 2517; 2358; 82	0.37181643	0.5313278		9.0
	ZINC05399487	-8.4	440.478	-1.9418	10	2	329.35	0.33026	128.74	1.9427	546.1145017766	0.26849	8.2045	-7.2323	0.625	0.41534	0.92326 (max o	6	2842; 2467; 2090; 20	-0.7007296	-0.4737797		9.0
	ZINC05440592	-8.4	447.466	-2	9	2	318.37	0.38543	150.8	1.1542	546.1145017766	0.27715	8.2627	-7.2163	0.6129	0.43032	0.96971 (max o	4	3255; 1181; 2081; 20	-0.6815769	-0.5260973		9.0
	ZINC05440487	-8.4	415.400	-1.7374	9	2	300.88	0.34971	125.5	-0.25493	546.1145017766	0.28639	8.0001	-6.0666	0.6	0.38719	0.98126 (max o	8	3255; 2842; 2467; 11	-0.6949696	-0.5087651		9.0
	ZINC05413922	-8.4	397.410	-1.8382	9	2	294.53	0.35725	125.5	1.0851	546.1145017766	0.29627	8.1009	-6.2046	0.62069	0.38236	0.95345 (max o	8	3255; 2842; 2467; 20	-0.6745456	-0.5073038		9.0
	ZINC05433657	-8.4	441.463	-1.4538	10	2	330.55	0.34857	134.73	-3.155	546.1145017766	0.26849	7.7165	-5.4147	0.65625	0.40255	0.98207 (max o	7	3255; 2842; 2467; 20	-0.6878179	-0.4897954		9.0
	ZINC04215221	-8.4	389.407	-1.3514	8	1	275.5	0.39543	141.14	10.412	546.1145017766	0.31821	7.6141	-4.2469	0.59259	0.47207	0.92757 (max o	2	2368; 2180	0.06912392	-0.9239841		9.0
	ZINC20444132	-8.4	388.423	-3.7931	8	3	274.1	0.41689	161.09	6.7688	546.1145017766	0.31821	10.056	-11.92	0.59259	0.41647	0.96617 (max o	5	4280; 3519; 3383; 25	-0.4970626	-0.0732293		9.0
	ZINC08551392	-8.4	448.379	-0.4237	11	8	295.54	0.4664	197.37	-1.9977	546.1145017766	0.26849	6.6864	-1.5781	0.5	0.38295	0.96186 (max o	11	3282; 3243; 2828; 28	0.6887053	0.04368195		8.0
	ZINC03864013	-8.4	351.286	-3.185	9	3	237.81	0.46112	145.58	-4.8874	546.1145017766	0.34367	9.4477	-9.2677	0.48	0.39879	0.94351 (max o	4	585; 747; 923; 1686	-0.8949809	-0.3938748		8.0
	ZINC02561003	-8.4	388.470	-3.0112	9	6	287.35	0.34474	158.71	2.6853	546.1145017766	0.30685	9.2739	-9.8134	0.53571	0.52194	0.90104	1	3156	0.63893723	0.29656774		8.0
	ZINC02560933	-8.4	372.471	-2.5819	8	6	276.09	0.32258	149.48	3.0318	546.1145017766	0.31821	8.8446	-8.1138	0.55556	0.52081	0.93046 (max o	2	7153; 3156	0.65937716	0.29368815		8.0
	ZINC40866224	-8.4	424.444	0.4226	9	5	308.56	0.35176	145.91	-7.5878	546.1145017766	0.28639	5.8401	1.4756	0.53333	0.40293	0.95837 (max o	4	3900; 3215; 3090; 23	-0.3384010	0.56415695		8.0
	ZINC40866222	-8.4	424.444	0.4226	9	5	308.56	0.35176	145.91	-7.5878	546.1145017766	0.28639	5.8401	1.4756	0.53333	0.40293	0.96092 (max o	4	3900; 3530; 3215; 23	-0.3319574	0.543377		8.0
	ZINC06041519	-8.4	432.380	-0.7865	10	7	289.19	0.43134	177.14	-2.3989	546.1145017766	0.27715	7.0492	-2.8378	0.48387	0.39235	0.96018 (max o	8	5883; 2481; 2463; 16	0.6630191	0.16169277		7.0
	ZINC08647924	-8.4	432.380	-0.3621	10	6	292.84	0.41538	166.14	-3.6206	546.1145017766	0.27715	6.6248	-1.3065	0.54839	0.43087	0.96262 (max o	10	3558; 2847; 2138; 27	0.15362632	0.68399507		7.0
	ZINC12139867	-8.4	432.380	-0.3621	10	6	292.84	0.41538	166.14	-3.6206	546.1145017766	0.27715	6.6248	-1.3065	0.54839	0.43087	0.95771 (max o	9	3558; 2847; 279; 365	0.18144935	0.7162888		7.0
	ZINC02094769	-8.4	380.351	-2.2557	7	0	281.53	0.31144	115.79	1.0433	546.1145017766	0.30685	8.5184	-7.3512	0.53571	0.36411	0.95148 (max o	4	428; 542; 697; 1093	-0.3539106	-0.0980178		7.0
	ZINC00941349	-8.4	396.350	-1.9406	8	0	292.06	0.33445	125.02	1.3459	546.1145017766	0.29627	8.2033	-6.5502	0.44828	0.35389	0.96428 (max o	3	3126; 2711; 1634	0.14529863	0.85794234		7.0
	ZINC01737607	-8.4	437.403	-2.6617	9	1	326.53	0.34355	144.89	1.6055	546.1145017766	0.26849	8.9244	-9.9136	0.5625	0.49407	0.97533 (max o	3	3131; 1078; 1967	-0.0111719	-0.9529953		7.0
	ZINC01737600	-8.4	423.376	-3.1161	9	1	312.77	0.35867	144.89	2.6789	546.1145017766	0.27715	9.3788	-11.243	0.54839	0.48399	0.97423 (max o	3	3131; 1078; 1968	0.00852775	-0.9457922		7.0
	ZINC02158774	-8.4	439.399	-1.9231	10	2	329.22	0.36255	143.09	-1.6692	546.1145017766	0.26849	8.1858	-7.1626	0.65625	0.51506	0.93139	1	2733	0.20011188	-0.1366735		7.0
	ZINC01592490	-8.4	384.339	-2.3176	8	0	288.6	0.349	132.86	0.2574	546.1145017766	0.30685	8.5803	-7.553	0.57143	0.3945	0.95148 (max o	3	3128; 2346; 1352	-0.8511208	-0.2580588		7.0
	ZINC04042176	-8.4	372.392	-2.6007	9	1	259.37	0.39257	115.38	2.2103	546.1145017766	0.33045	8.8634	-7.8702	0.42308	0.40455	0.94951 (max o	5	3083; 2778; 1615; 19	-0.2687812	-0.8446246		7.0
	ZINC15676178	-8.4	381.391	-2.8438	9	3	282.43	0.33977	143.26	0.33227	546.1145017766	0.30685	9.1065	-9.2678	0.57143	0.52949	0.91768 (max o	3	4216; 2199; 2198	0.2300824	-0.2440918		7.0

Structure	Molecule Name	affinity	Total Molweight	cLogP	H-Acceptors	H-Donors	Total Surface Area	Relative PSA	Polar Surface Area	Druglikeness	Kd (nM) @ 20°C	LE from K...	LLE from Kd ...	LELP from Kd (n...	Shape Index	Molecular...	Neighbor Si...	Neig...	Neighbor	Neighbor ...	Neighbor ...	Ring Systems	frequency
	ZINC15676178	-8.4	380.383	-2.6549	9	2	293.4	0.36445	142.01	0.91024	546.1145017766	0.30685	8.9176	-8.6522	0.57143	0.52949	0.97288 (max o	5	4218; 4217; 2199; 21	0.24201994	-0.2615786		7.0
	ZINC15675983	-8.4	428.448	-1.908	10	1	315.44	0.41976	162.57	5.8535	546.1145017766	0.28639	8.1707	-6.6622	0.56667	0.49383	0.97533 (max o	6	652; 793; 1520; 1521	0.4057798	-0.1369493		6.0
	ZINC15675983	-8.4	428.448	-1.908	10	1	315.44	0.41976	162.57	5.8535	546.1145017766	0.28639	8.1707	-6.6622	0.56667	0.49383	0.97533 (max o	5	653; 1520; 1521; 219	0.3807822	-0.1663833		6.0
	ZINC12901696	-8.4	387.371	-3.0914	10	2	283.1	0.41763	154.22	6.0613	546.1145017766	0.30685	9.3541	-10.075	0.57143	0.47406	0.96023 (max o	6	5494; 3348; 2546; 96	0.1724912	0.5461842		6.0
	ZINC31415451	-8.4	430.404	-2.0393	11	6	294.39	0.44716	175.37	-4.6332	546.1145017766	0.28639	8.302	-7.1207	0.56667	0.47685	0.92547 (max o	2	2328; 1023	-0.3953302	0.3329802		6.0
	ZINC04098736	-8.4	442.459	-0.5574	10	6	313.68	0.37809	158.3	-1.6253	546.1145017766	0.27715	6.8201	-2.0112	0.58065	0.49334	0.95031 (max o	4	2635; 2328; 820; 128	-0.4334343	0.35263842		6.0
	ZINC72320591	-8.4	428.432	-0.8821	10	6	302.68	0.39183	158.3	-7.6666	546.1145017766	0.28639	7.1448	-3.0801	0.6	0.49072	0.92547 (max o	3	1023; 2045; 2273	-0.4157394	0.34247342		6.0
	ZINC04523265	-8.4	418.397	-1.026	9	7	280.74	0.40871	167.91	-3.0467	546.1145017766	0.28639	7.2887	-3.5825	0.36667	0.39399	0.98188 (max o	7	3470; 3364; 2823; 28	-0.5069067	0.60440034		6.0
	ZINC04523265	-8.4	418.397	-1.026	9	7	280.74	0.40871	167.91	-3.0467	546.1145017766	0.28639	7.2887	-3.5825	0.36667	0.39399	0.98188 (max o	7	3470; 3364; 2823; 28	-0.5033265	0.6240079		6.0
	ZINC18847072	-8.4	438.403	-5.6299	13	4	324.71	0.50821	224.99	2.7179	546.1145017766	0.26849	11.893	-20.969	0.625	0.57966	0.98148 (max o	5	2350; 1077; 1458; 16	-0.5490877	0.7114097		6.0
	ZINC02944400	-8.4	423.388	-4.9632	12	3	316.19	0.47361	198.97	2.7179	546.1145017766	0.27715	11.226	-17.908	0.64516	0.57782	0.98065 (max o	5	2350; 2206; 1077; 14	-0.5688754	0.71576136		6.0
	ZINC04063342	-8.4	358.369	-0.5486	7	0	264.58	0.29084	95.97	5.7109	546.1145017766	0.33045	6.8113	-1.6602	0.53846	0.48199	0.92064 (max o	5	2036; 709; 875; 1687	0.2829077	0.15809236		6.0
	ZINC04081348	-8.4	358.369	-0.5486	7	0	264.58	0.29084	95.97	5.7109	546.1145017766	0.33045	6.8113	-1.6602	0.5	0.48199	0.93686 (max o	7	2408; 1121; 1472; 16	0.26058006	0.1933329		6.0
	ZINC04259840	-8.4	428.408	-1.2066	13	1	310.73	0.42831	140.33	2.6108	546.1145017766	0.27715	7.4693	-4.3536	0.6129	0.38594	0.96971 (max o	5	348; 1146; 1414; 170	-0.1209057	-0.3926717		6.0
	ZINC08879560	-8.4	368.324	-2.1446	9	3	263.88	0.40174	138.87	5.5161	546.1145017766	0.31821	8.4073	-6.7396	0.55556	0.488	0.9428 (max of	3	2851; 469; 807	0.11824289	-0.2703248		6.0
	ZINC20610544	-8.4	352.325	-1.7989	8	2	257.53	0.36077	118.64	5.5071	546.1145017766	0.33045	8.0616	-5.4438	0.53846	0.49329	0.95228 (max o	5	3151; 695; 1364; 136	0.18870057	-0.2023974		6.0
	ZINC12892134	-8.4	426.404	-1.4845	10	2	315.81	0.35753	137.1	5.1043	546.1145017766	0.27715	7.7472	-5.3563	0.51613	0.48706	0.98065 (max o	2	3787; 952	0.72227895	-0.3309891		6.0
	ZINC08643365	-8.4	421.405	-0.7499	11	3	312.19	0.41574	153.24	-0.28343	546.1145017766	0.28639	7.0126	-2.6185	0.66667	0.48252	0.95099 (max o	5	4226; 3331; 1770; 17	-0.2695526	-0.5846214		6.0
	ZINC12603970	-8.4	406.477	0.3778	8	3	305.8	0.28545	108.33	3.06	546.1145017766	0.29627	5.8849	1.2752	0.62069	0.53026	0.94558 (max o	4	2505; 2498; 2499; 21	0.26676786	-0.3339137		6.0
	ZINC08643392	-8.4	366.388	0.3088	7	3	265.13	0.29152	99.1	1.7543	546.1145017766	0.33045	5.9539	0.93448	0.57692	0.50913	0.92368 (max o	7	4167; 3334; 2505; 24	0.31116492	-0.3655724		6.0
	ZINC08636297	-8.4	391.466	0.1044	8	3	293.6	0.27534	102.34	3.7591	546.1145017766	0.30685	6.1583	0.34024	0.60714	0.53612	0.92094 (max o	7	6476; 5018; 3730; 32	0.35598505	-0.3329501		6.0
	ZINC12529733	-8.4	358.397	-1.7455	8	2	290.12	0.3635	112.59	2.3416	546.1145017766	0.33045	8.0082	-5.2822	0.65385	0.4334	0.97533 (max o	7	4650; 3328; 3327; 29	0.6399326	-0.2879846		6.0
	ZINC12529739	-8.4	390.463	-2.0081	8	2	307.61	0.39969	137.89	3.8612	546.1145017766	0.31821	8.2708	-6.3106	0.66667	0.4867	0.94622 (max o	6	4650; 3773; 3328; 33	0.6206488	-0.2977361		6.0
	ZINC04089470	-8.4	403.477	-2.4173	8	3	299.38	0.29812	126.38	5.4943	546.1145017766	0.29627	8.68	-8.1592	0.44828	0.4612	0.9892 (max of	4	2908; 601; 1126; 147	0.49418995	-0.7243517		5.0
	ZINC35442390	-8.4	428.531	-2.8165	8	2	337.3	0.26549	97.22	6.3596	546.1145017766	0.27715	9.0792	-10.162	0.58065	0.47528	0.92602 (max o	3	4339; 1560; 1912	0.05395699	0.50276		5.0
	ZINC04963987	-8.4	448.379	-0.4237	11	8	295.54	0.4664	197.37	-1.9977	546.1145017766	0.26849	6.6864	-1.5781	0.4375	0.38342	0.95949 (max o	7	3891; 2629; 2422; 58	0.75600463	0.00609068		5.0
	ZINC12489958	-8.4	429.495	-0.8412	9	4	334.44	0.32179	106.38	0.70978	546.1145017766	0.27715	7.1039	-3.0352	0.6129	0.49802	0.94501 (max o	4	3763; 3326; 2524; 14	0.29350182	0.04781776		5.0
	ZINC00967787	-8.4	360.408	-2.1145	6	2	242.11	0.1921	83.22	1.1974	546.1145017766	0.33045	8.3772	-6.3989	0.46154	0.3395	0.95857	1	2706	0.92800653	-0.1946606		5.0
	ZINC03840094	-8.4	405.473	-2.9567	9	6	304.14	0.36579	158.36	5.2604	546.1145017766	0.29627	9.2194	-9.9799	0.55172	0.51964	0.94241 (max o	8	5386; 4485; 2760; 70	-0.5006518	-0.1880325		5.0
	ZINC03840095	-8.4	433.527	-2.284	9	6	328.9	0.33825	158.36	4.9712	546.1145017766	0.27715	8.5467	-8.241	0.51613	0.51829	0.94241 (max o	4	5721; 2760; 882; 200	-0.5329062	-0.1909653		5.0
	ZINC12603502	-8.4	393.438	0.1319	9	4	292.5	0.33761	120.36	3.4058	546.1145017766	0.30685	6.1308	0.42986	0.60714	0.54369	0.9399 (max of	2	2875; 2116	0.26200706	-0.4288484		5.0
	ZINC08643412	-8.4	381.403	0.3027	8	4	276.59	0.32087	111.13	1.9915	546.1145017766	0.31821	5.96	0.95126	0.59259	0.53255	0.9399 (max of	8	5024; 3295; 2875; 25	0.3109359	-0.4004632		5.0
	ZINC08624396	-8.4	425.455	0.1286	9	4	311.35	0.31717	120.36	1.9895	546.1145017766	0.28639	6.1341	0.44904	0.6	0.54821	0.94529 (max o	5	5024; 2527; 2499; 21	0.27734476	-0.3852653		5.0
	ZINC08624394	-8.4	407.465	0.0278	9	4	305	0.32377	120.36	3.3295	546.1145017766	0.29627	6.2349	0.093835	0.62069	0.54456	0.94529 (max o	4	5449; 2875; 2498; 21	0.23518407	-0.3931426		5.0
	ZINC35454808	-8.4	434.352	0.2551	11	7	284.9	0.47294	186.37	-3.8653	546.1145017766	0.27715	6.0076	0.92044	0.41935	0.41305	0.96262 (max o	15	4095; 2776; 2566; 25	-0.6979945	0.3080065		5.0
	ZINC14684626	-8.4	434.352	0.2551	11	7	284.9	0.47294	186.37	-3.8653	546.1145017766	0.27715	6.0076	0.92044	0.41935	0.41305	0.96262 (max o	15	4095; 2776; 2566; 25	-0.6924053	0.25533396		5.0
	ZINC20464791	-8.4	387.382	-0.5233	8	4	275.19	0.31146	103.29	1.666	546.1145017766	0.31821	6.786	-1.6445	0.55556	0.5302	0.92442 (max o	4	4744; 2995; 1250; 12	-0.3216936	-0.0673695		5.0
	ZINC20465411	-8.4	434.505	-0.2197	6	3	316.08	0.16148	72.78	2.5628	546.1145017766	0.27715	6.4824	-0.79271	0.6129	0.45207	0.94768 (max o	6	6576; 3848; 2598; 25	-0.4905752	0.28945944		5.0
	ZINC20464905	-8.4	416.515	-0.3205	6	3	309.73	0.16479	72.78	2.5628	546.1145017766	0.28639	6.5832	-1.1191	0.6	0.44758	0.96589 (max o	9	6576; 4299; 3848; 33	-0.4687334	0.26370934		5.0
	ZINC62227225	-8.4	393.371	-3.2184	10	5	279.75	0.44722	164.17	4.1929	546.1145017766	0.30685	9.4811	-10.489	0.53571	0.44009	0.95148 (max o	4	3534; 3096; 3097; 26	0.52873695	-0.4213835		5.0
	ZINC03881959	-8.4	390.387	0.8402	8	6	280.38	0.35167	139.84	-4.3466	546.1145017766	0.30685	5.4225	2.7382	0.57143	0.42615	0.9678 (max of	7	4139; 4140; 3706; 20	-0.5077665	0.7876664		4.0
	ZINC03881961	-8.4	390.387	0.8402	8	6	280.38	0.35167	139.84	-4.3466	546.1145017766	0.30685	5.4225	2.7382	0.57143	0.42615	0.9678 (max of	8	4139; 4140; 3706; 28	-0.4729043	0.7859942		4.0
	ZINC04259814	-8.4	404.445	0.6105	10	1	291.56	0.33448	106.36	-0.088003	546.1145017766	0.29627	5.6522	2.0607	0.58621	0.3672	0.91871 (max o	5	3661; 3608; 347; 907	-0.0168991	-0.2924435		4.0

Structure	Molecule Name	affinity	Total Molweight	cLogP	H-Acceptors	H-Donors	Total Surface Area	Relative PSA	Polar Surface Area	Druglikeness	Kd (nM) @ 20°C	LE from K...	LLE from Kd ...	LELP from Kd (n...	Shape Index	Molecular...	Neighbor Si...	Neig...	Neighbor	Neighbor ...	Neighbor ...	Ring Systems	frequency
	ZINC13430719	-8.4	415.426	-4.3719	12	5	256.85	0.50602	182.48	-2.4143	546.1145017766	0.30685	10.635	-14.248	0.46429	0.44266	0.95983 (max o	3	620; 780; 1505	-0.5261128	-0.6743851		4.0
	ZINC03985238	-8.4	366.352	-1.2962	8	2	271.29	0.34247	118.64	6.0145	546.1145017766	0.31821	7.5589	-4.0734	0.55556	0.53877	0.96885 (max o	3	2241; 1364; 1653	0.17144178	-0.1869457		4.0
	ZINC13308148	-8.4	363.416	0.5683	9	3	267.82	0.36383	114.55	-3.3637	546.1145017766	0.33045	5.6944	1.7198	0.53846	0.3046	0.93361 (max o	3	4249; 860; 1814	-0.3844567	-0.8597529		4.0
	ZINC35454752	-8.4	448.419	-2.0505	12	6	308.9	0.44869	176.76	-4.801	546.1145017766	0.27715	8.3132	-7.3985	0.54839	0.45309	0.96262 (max o	6	2950; 969; 970; 1605;	0.6332835	0.4351768		4.0
	ZINC15708211	-8.4	401.485	-1.7078	8	3	314.91	0.29589	92.18	4.1918	546.1145017766	0.29627	7.9705	-5.7644	0.58621	0.46908	0.94251 (max o	6	5521; 983; 1240; 130	-0.1773196	0.12419717		4.0
	ZINC31162098	-8.4	370.312	1.0015	8	3	243.66	0.40392	130.36	0.8612	546.1145017766	0.31821	5.2612	3.1473	0.44444	0.24731	0.95493 (max o	3	4806; 1027; 1900	-0.267741	-0.8094207		4.0
	ZINC72320459	-8.4	431.416	-0.8675	9	4	291.67	0.3866	156.58	-1.1474	546.1145017766	0.27715	7.1302	-3.1301	0.3871	0.27235	0.97199 (max o	3	3115; 1071; 2326	-0.7746030	-0.5418369		4.0
	ZINC72320458	-8.4	431.416	-0.8675	9	4	291.67	0.3866	156.58	-1.1474	546.1145017766	0.27715	7.1302	-3.1301	0.3871	0.27955	0.97199 (max o	3	3115; 2327; 1071	-0.7970856	-0.5065856		4.0
	ZINC00580569	-8.4	357.369	0.0027999	9	4	257.63	0.39025	125.55	-0.31782	546.1145017766	0.33045	6.2599	0.0084729	0.57692	0.3491	0.94951 (max o	9	3229; 2807; 2417; 23	-0.4094248	-0.7468071		4.0
	ZINC08740161	-8.4	388.383	-1.2563	11	6	272.71	0.47626	167.25	2.2354	546.1145017766	0.30685	7.519	-4.0942	0.46429	0.39778	0.94558 (max o	7	4202; 3780; 3111; 28	0.31932703	-0.5328123		4.0
	ZINC05413953	-8.4	373.412	-0.8168	9	2	279.36	0.33344	111.71	5.2839	546.1145017766	0.31821	7.0795	-2.5669	0.48148	0.52098	0.97149 (max o	6	5426; 4570; 4127; 45	0.06109134	0.22332697		4.0
	ZINC40879757	-8.4	406.386	0.31	9	5	289.04	0.37552	145.91	0.063551	546.1145017766	0.29627	5.9527	1.0464	0.55172	0.37376	0.93686 (max o	3	7420; 1578; 2046	-0.7482734	-0.0883057		4.0
	ZINC04098879	-8.4	390.387	0.6557	8	4	282.69	0.33761	125.68	0.063551	546.1145017766	0.30685	5.607	2.1369	0.53571	0.38007	0.93686 (max o	5	7193; 6781; 5207; 23	-0.7667355	-0.0982745		4.0
	ZINC02095849	-8.4	352.369	-0.4593	7	1	256.85	0.28016	92.78	3.893	546.1145017766	0.33045	6.722	-1.3899	0.5	0.48536	0.9464 (max of	5	5677; 3147; 688; 164	0.18896483	0.7842217		4.0
	ZINC19800890	-8.4	372.336	-0.7308	10	4	253.61	0.44292	145.11	1.0072	546.1145017766	0.31821	6.9935	-2.2966	0.48148	0.3049	0.94302 (max o	4	2210; 1733; 1855; 22	0.6440414	-0.5314859		4.0
	ZINC19800892	-8.4	372.336	-0.7308	10	4	253.61	0.44292	145.11	1.0072	546.1145017766	0.31821	6.9935	-2.2966	0.48148	0.3049	0.94905 (max o	4	2211; 1733; 1855; 22	0.6138567	-0.5155372		4.0
	ZINC71405297	-8.4	360.413	-0.1955	8	3	274.01	0.31769	99.77	5.1688	546.1145017766	0.33045	6.4582	-0.59162	0.61538	0.54464	0.90377 (max o	2	1745; 1947	0.13867544	0.2355198		4.0
	ZINC03984166	-8.4	392.383	-0.816	9	4	264.77	0.3528	124.89	1.2217	546.1145017766	0.30685	7.0787	-2.6593	0.46429	0.22878	0.92774 (max o	5	3369; 2970; 2695; 26	-0.6051063	-0.6987504		4.0
	ZINC03984126	-8.4	392.383	-0.816	9	4	264.77	0.3528	124.89	1.2217	546.1145017766	0.30685	7.0787	-2.6593	0.46429	0.22878	0.92774 (max o	5	3369; 2970; 2695; 26	-0.6048174	-0.6779715		4.0
	ZINC04098340	-8.4	356.326	-1.7633	9	4	245.13	0.43014	134.91	-5.757	546.1145017766	0.34367	8.026	-5.1308	0.48	0.41551	0.9594 (max of	5	3676; 3677; 3556; 32	0.47512823	0.31476724		4.0
	ZINC04293609	-8.4	447.399	-5.6024	12	2	330.6	0.43336	185.07	3.3292	546.1145017766	0.26849	11.865	-20.866	0.59375	0.57647	0.97948 (max o	3	4976; 3671; 3242	-0.8564948	-0.0371054		4.0
	ZINC05399644	-8.4	401.489	-1.7382	9	3	321.07	0.31881	94.74	-1.3705	546.1145017766	0.29627	8.0009	-5.867	0.65517	0.49018	0.96682 (max o	7	4123; 3688; 3253; 92	-0.2227317	-0.0879888		4.0
	ZINC12603794	-8.4	378.423	-0.2701	8	3	281.04	0.3106	108.33	2.376	546.1145017766	0.31821	6.5328	-0.84881	0.62963	0.54126	0.93379 (max o	8	3335; 2928; 2570; 25	0.2883177	-0.3318817		4.0
	ZINC08643464	-8.4	382.387	-0.5132	8	3	275.13	0.31727	108.33	1.0465	546.1145017766	0.31821	6.7759	-1.6128	0.59259	0.54126	0.92739 (max o	8	5821; 3335; 2527; 24	0.32205698	-0.3478573		4.0
	ZINC10385338	-8.4	395.458	-0.1465	7	3	307.72	0.27151	110.86	2.2346	546.1145017766	0.29627	6.4092	-0.49449	0.62069	0.51994	0.96428 (max o	3	2910; 2897; 2123	-0.7000671	0.525779		4.0
	ZINC08765290	-8.4	381.431	-0.4904	7	3	295.46	0.28278	110.86	2.2489	546.1145017766	0.30685	6.7531	-1.5982	0.64286	0.5206	0.96253 (max o	3	2910; 2897; 2133	-0.6651019	0.5240481		4.0
	ZINC12481319	-8.4	373.495	-0.7786	6	1	309.09	0.21046	68.12	4.1263	546.1145017766	0.31821	7.0413	-2.4468	0.55556	0.58858	0.96064 (max o	3	4724; 4717; 2581	0.61926186	0.4199553		4.0
	ZINC12484144	-8.4	374.396	-0.5016	9	3	273.09	0.35215	116.08	-2.2838	546.1145017766	0.31821	6.7643	-1.5763	0.55556	0.45886	0.92442 (max o	8	5051; 4732; 4645; 42	-0.6704491	-0.2763993		4.0
	ZINC20464791	-8.4	388.390	-2.633	8	5	289.23	0.34488	104.49	-1.2784	546.1145017766	0.31821	8.8957	-8.2744	0.55556	0.5323	0.8934 (max of	2	5550; 2996	0.6076708	0.34847906		4.0
	ZINC31156385	-8.4	424.401	-0.4974	10	5	308.78	0.39374	162.98	-10.495	546.1145017766	0.28639	6.7601	-1.7368	0.6	0.48219	0.95837	1	2628	0.6020032	-0.5354259		4.0
	ZINC35455223	-8.4	418.397	0.8232	9	6	298.13	0.37447	156.91	-4.0044	546.1145017766	0.28639	5.4395	2.8744	0.5	0.43103	0.97533 (max o	7	3894; 3450; 3070; 26	-0.8237421	0.16941248		4.0
	ZINC31156734	-8.4	418.397	0.8232	9	6	298.13	0.37447	156.91	-4.0044	546.1145017766	0.28639	5.4395	2.8744	0.5	0.43061	0.96092 (max o	7	3894; 3450; 3070; 26	-0.7791946	0.14569041		4.0
	ZINC35455218	-8.4	418.397	0.8232	9	6	298.13	0.37447	156.91	-4.0044	546.1145017766	0.28639	5.4395	2.8744	0.5	0.42543	0.95579 (max o	7	3894; 3450; 3070; 26	-0.8289382	0.14658087		4.0
	ZINC31156742	-8.4	418.397	0.8232	9	6	298.13	0.37447	156.91	-4.0044	546.1145017766	0.28639	5.4395	2.8744	0.5	0.425	0.95837 (max o	7	3894; 3450; 3070; 26	-0.7916119	0.16142805		4.0
	ZINC31157217	-8.4	424.401	-1.2512	10	5	297.96	0.40804	162.98	-10.12	546.1145017766	0.28639	7.5139	-4.3689	0.5	0.4557	0.96589 (max o	6	6615; 5191; 4375; 43	-0.7945844	-0.0010717		4.0
	ZINC35454756	-8.4	448.419	-2.0505	12	6	308.9	0.44869	176.76	-4.801	546.1145017766	0.27715	8.3132	-7.3985	0.54839	0.45535	0.96262 (max o	9	2950; 2951; 2624; 97	0.5911853	0.4476189		4.0
	ZINC35454747	-8.4	448.419	-2.0505	12	6	308.9	0.44869	176.76	-4.801	546.1145017766	0.27715	8.3132	-7.3985	0.54839	0.45535	0.96262 (max o	6	2950; 2951; 969; 228	0.61586684	0.4772159		4.0
	ZINC67913248	-8.4	448.507	0.2945	10	6	331.27	0.35802	158.3	-0.81143	546.1145017766	0.27715	5.9682	1.0626	0.6129	0.55512	0.90581 (max o	2	3916; 2643	-0.3355864	0.3867182		4.0
	ZINC72324500	-8.4	358.325	-1.2306	8	1	253.15	0.38673	121.83	0.11443	546.1145017766	0.33045	7.4933	-3.724	0.57692	0.39474	0.93033 (max o	3	4451; 3979; 3117	-0.8912871	0.22029604		4.0
	ZINC08765441	-8.4	360.365	0.5334	8	1	266.01	0.31627	94.17	5.7863	546.1145017766	0.33045	5.7293	1.6142	0.53846	0.4954	0.90311 (max o	2	774; 1473	0.88762945	-0.3423359		3.0
	ZINC03840155	-8.4	410.445	-3.575	10	5	305.63	0.42807	181.88	1.7422	546.1145017766	0.29627	9.8377	-12.067	0.55172	0.59342	0.9673 (max of	3	3601; 1378; 2008	0.59147096	0.698815		3.0
	ZINC08627179	-8.4	385.419	-0.4323	8	4	290.68	0.33084	120.78	0.8757	546.1145017766	0.30685	6.695	-1.4088	0.64286	0.52434	0.9398 (max of	3	3289; 1516; 1765	0.4160529	-0.3850071		3.0
	ZINC08643448	-8.4	388.419	0.0238	8	3	292.71	0.31225	112.24	4.0875	546.1145017766	0.30685	6.2389	0.077563	0.60714	0.5597	0.97288 (max o	6	5454; 4654; 4606; 29	0.5119985	-0.3678639		3.0

Structure	Molecule Name	affinity	Total Molweight	cLogP	H-Acceptors	H-Donors	Total Surface Area	Relative PSA	Polar Surface Area	Druglikeness	Kd (nM) @ 20°C	LE from K...	LLE from Kd ...	LELP from Kd (n...	Shape Index	Molecular...	Neighbor Si...	Neig...	Neighbor	Neighbor ...	Neighbor ...	Ring Systems	frequency
	ZINC00967786	-8.4	409.417	-0.9747	8	3	289.6	0.32521	134.02	5.8403	546.1145017766	0.28639	7.2374	-3.4034	0.43333	0.34225	0.83638	1	2395	0.66907316	-0.3977998		3.0
	ZINC19800890	-8.4	371.328	-2.9004	10	3	254.9	0.44574	151.43	-0.58508	546.1145017766	0.31821	9.1631	-9.1147	0.48148	0.2975	0.94302 (max o	3	2211; 1856; 2208	0.6549332	-0.5114579		3.0
	ZINC19800892	-8.4	371.328	-2.9004	10	3	254.9	0.44574	151.43	-0.58508	546.1145017766	0.31821	9.1631	-9.1147	0.48148	0.2975	0.93058 (max o	3	1856; 2209; 2210	0.62767214	-0.4946031		3.0
	ZINC33943584	-8.4	362.385	-4.4497	8	3	247.6	0.46151	161.09	7.0306	546.1145017766	0.34367	10.712	-12.948	0.56	0.43729	0.93033 (max o	5	5218; 4817; 4280; 25	-0.4608542	-0.0857103		3.0
	ZINC00644891	-8.4	355.353	0.9373	9	3	256.53	0.39169	122.39	1.8667	546.1145017766	0.33045	5.3254	2.8364	0.57692	0.28996	0.93686 (max o	4	2824; 2709; 2338; 19	0.6977138	0.20817722		3.0
	ZINC03811114	-8.4	386.426	-1.3765	7	2	260.43	0.29647	117.34	1.7149	546.1145017766	0.30685	7.6392	-4.486	0.42857	0.30712	0.83447	1	1996	0.69935095	0.25276724		3.0
	ZINC03795983	-8.4	409.819	-0.5881	6	1	260.16	0.23808	91.32	1.9465	546.1145017766	0.30685	6.8508	-1.9166	0.42857	0.31257	0.96519 (max o	3	4944; 2423; 1998	0.7174462	0.24051939		3.0
	ZINC03839400	-8.4	429.519	0.3338	9	2	314.73	0.28294	103.75	5.0513	546.1145017766	0.27715	5.9289	1.2044	0.48387	0.34725	0.92863 (max o	6	4038; 3164; 2376; 33	-0.5379273	-0.0798628		3.0
	ZINC03839645	-8.4	359.405	-4.0188	8	2	253.1	0.32461	106.59	5.15	546.1145017766	0.33045	10.282	-12.162	0.57692	0.34104	0.90033 (max o	2	5328; 2003	-0.6720768	0.632434		3.0
	ZINC03839643	-8.4	397.430	0.0049001	8	1	292.94	0.26517	88.18	6.14	546.1145017766	0.29627	6.2578	0.01654	0.62069	0.33634	0.87582 (max o	2	5328; 2004	-0.6500405	0.64623874		3.0
	ZINC04073531	-8.4	424.516	-0.5042	9	1	325.12	0.2922	119.08	-60.151	546.1145017766	0.28639	6.7669	-1.7605	0.53333	0.56418	0.94543 (max o	3	6018; 5364; 4077	0.5234716	-0.0622345		3.0
	ZINC04270709	-8.4	361.420	-0.2491	8	1	270.19	0.27732	96.42	2.6677	546.1145017766	0.33045	6.5118	-0.75382	0.53846	0.52017	0.91007 (max o	3	6436; 5419; 5401	0.10267143	0.4180229		3.0
	ZINC04277709	-8.4	395.481	-0.4245	7	2	310.15	0.24269	74.16	5.7617	546.1145017766	0.29627	6.6872	-1.4328	0.58621	0.42755	0.97176 (max o	6	3857; 3670; 2449; 24	0.25121477	-0.5760739		3.0
	ZINC15709545	-8.4	374.400	-1.7637	10	2	278.12	0.37437	124.6	5.2839	546.1145017766	0.31821	8.0264	-5.5425	0.48148	0.46539	0.95201 (max o	6	4994; 2931; 2932; 59	0.01999824	0.2649183		3.0
	ZINC05433669	-8.4	417.468	-1.2691	11	3	314.34	0.36769	136.63	6.2676	546.1145017766	0.28639	7.5318	-4.4314	0.43333	0.49282	0.91248 (max o	2	4129; 2203	0.04112986	0.23245911		3.0
	ZINC08299190	-8.4	414.508	-3.3471	9	3	320.37	0.30789	99.32	0.28325	546.1145017766	0.28639	9.6098	-11.687	0.63333	0.47816	0.97304 (max o	6	3720; 3277; 2492; 76	-0.1821372	-0.0215190		3.0
	ZINC08624442	-8.4	394.425	-3.0261	9	4	289.54	0.33588	126.11	-3.4929	546.1145017766	0.30685	9.2888	-9.8619	0.67857	0.54473	0.9398 (max of	2	4604; 3774	0.7977974	-0.4516039		3.0
	ZINC15676176	-8.4	353.381	-0.5398	8	2	276.62	0.32395	111.11	2.0073	546.1145017766	0.33045	6.8025	-1.6335	0.57692	0.50875	0.93058 (max o	3	2199; 2198; 2196	0.21921751	-0.2611643		3.0
	ZINC15676178	-8.4	380.383	-2.6549	9	2	293.4	0.36445	142.01	0.91024	546.1145017766	0.30685	8.9176	-8.6522	0.57143	0.52949	0.97288 (max o	5	4218; 4217; 2195; 21	0.2640636	-0.2602915		3.0
	ZINC20463256	-8.4	444.574	-0.8769	8	1	361.66	0.22574	88.43	5.2662	546.1145017766	0.26849	7.1396	-3.266	0.5625	0.6113	0.92326 (max o	7	6800; 6496; 4211; 33	-0.0417533	-0.1710924		3.0
	ZINC20463256	-8.4	443.566	-1.0085	8	0	347.62	0.19447	87.23	9.8511	546.1145017766	0.26849	7.2712	-3.7562	0.5625	0.61463	0.92326 (max o	3	3385; 2983; 2219	-0.0983320	-0.1668443		3.0
	ZINC20465802	-8.4	436.477	-0.0104	7	5	337.23	0.26614	95.26	0.42057	546.1145017766	0.27715	6.2731	-0.037524	0.58065	0.54587	0.94768 (max o	4	3854; 3407; 806; 223	0.33291236	-0.2701040		3.0
	ZINC20465811	-8.4	430.523	-0.282	8	5	346.79	0.28764	104.49	1.9025	546.1145017766	0.27715	6.5447	-1.0175	0.58065	0.56867	0.93414 (max o	3	3854; 2600; 2237	0.31754452	-0.2814389		3.0
	ZINC31167883	-8.4	363.428	-0.939	6	3	246.64	0.32298	117.89	-4.3143	546.1145017766	0.33045	7.2017	-2.8416	0.38462	0.41417	0.96998 (max o	3	3807; 2268; 2269	0.9300098	0.25445995		3.0
	ZINC31167883	-8.4	363.428	-0.939	6	3	246.64	0.32298	117.89	-4.3143	546.1145017766	0.33045	7.2017	-2.8416	0.38462	0.41417	0.96998 (max o	3	3807; 2268; 2270	0.90626687	0.2839717		3.0
	ZINC31167883	-8.4	362.420	-3.015	6	2	247.82	0.3262	120.72	-1.8945	546.1145017766	0.33045	9.2777	-9.1239	0.38462	0.41153	0.92368 (max o	4	3806; 3807; 2270; 22	0.9238626	0.277749		3.0
	ZINC34911990	-8.4	352.341	-1.1086	6	0	277.52	0.26895	98.72	0.78259	546.1145017766	0.33045	7.3713	-3.3548	0.53846	0.49811	0.87452	1	6017	0.8290692	0.47206086		3.0
	ZINC35455431	-8.4	394.418	-0.2395	8	3	277.8	0.35428	130.36	-10.347	546.1145017766	0.30685	6.5022	-0.78052	0.46429	0.41687	0.95148 (max o	4	4365; 3494; 3454; 34	-0.4728845	0.4992328		3.0
	ZINC40312750	-8.4	404.377	-2.9582	8	1	296.77	0.32352	123.52	3.7628	546.1145017766	0.28639	9.2209	-10.329	0.5	0.41388	0.95579 (max o	5	6036; 4852; 3525; 30	0.5447254	-0.2981331		3.0
	ZINC04260203	-8.4	402.449	0.0924	9	1	295.71	0.27469	91.42	6.3988	546.1145017766	0.29627	6.1703	0.31188	0.62069	0.53811	0.94558 (max o	4	3662; 84; 198; 446	-0.2906346	0.08113219		2.0
	ZINC04044138	-8.4	419.492	-1.2533	7	3	284.29	0.31538	127.12	0.12303	546.1145017766	0.28639	7.516	-4.3762	0.46667	0.36524	0.95579 (max o	4	134; 562; 891; 1395	0.26314366	0.8469876		2.0
	ZINC71404535	-8.4	448.379	-0.0535	11	7	299.19	0.45035	186.37	-3.3789	546.1145017766	0.26849	6.3162	-0.19926	0.53125	0.27742	0.95709 (max o	3	677; 1624; 1944	-0.2950150	-0.6642561		2.0
	ZINC31156362	-8.4	424.401	-0.5282	10	4	300.94	0.3937	151.98	-0.28537	546.1145017766	0.28639	6.7909	-1.8443	0.6	0.42846	0.95318 (max o	3	819; 1286; 1575	0.2622248	-0.9584855		2.0
	ZINC67903394	-8.4	443.467	-2.8577	10	5	309.29	0.3971	168.97	-2.258	546.1145017766	0.27715	9.1204	-10.311	0.51613	0.43315	0.94751	1	853	-0.2786647	-0.7918973		2.0
	ZINC72319975	-8.4	356.285	-2.8261	8	2	254.28	0.39657	139.18	1.0637	546.1145017766	0.33045	9.0888	-8.5523	0.57692	0.39666	0.93686 (max o	3	857; 858; 1625	0.7018984	0.49552378		2.0
	ZINC20113144	-8.4	382.462	0.7274	7	1	279.84	0.21698	68.88	5.9081	546.1145017766	0.30685	5.5353	2.3706	0.5	0.43289	0.92507 (max o	4	6115; 6114; 2397; 12	-0.3423116	-0.6862056		2.0
	ZINC08790676	-8.4	420.444	0.038	8	0	307.75	0.2505	101.06	5.2139	546.1145017766	0.27715	6.2247	0.13711	0.3871	0.47043	0.91993 (max o	10	5034; 4962; 4522; 40	0.7618107	-0.0145292		2.0
	ZINC20757757	-8.4	395.394	-1.9455	9	3	296.51	0.35199	130.67	4.4525	546.1145017766	0.29627	8.2082	-6.5667	0.44828	0.5117	0.91964 (max o	4	1011; 1185; 1259; 12	0.5785715	-0.2173580		2.0
	ZINC35442318	-8.4	416.544	-1.2042	8	2	316.68	0.23162	91.38	6.0083	546.1145017766	0.28639	7.4669	-4.2048	0.6	0.45311	0.96832 (max o	7	1297; 1301; 1302; 13	-0.5768097	0.17445149		2.0
	ZINC00898099	-8.4	356.341	-0.449	10	2	257.34	0.40347	130.63	4.6343	546.1145017766	0.33045	6.7117	-1.3588	0.5	0.51063	0.91351 (max o	2	1959; 1347	0.59074116	0.6583417		2.0
	ZINC00898099	-8.4	357.349	-2.4268	10	3	246.37	0.37691	131.88	4.0499	546.1145017766	0.33045	8.6895	-7.3439	0.5	0.51063	0.91351 (max o	2	1348; 1958	0.5724647	0.6506497		2.0
	ZINC08254030	-8.4	441.578	-5.4041	9	2	347.02	0.26229	95.57	2.6132	546.1145017766	0.26849	11.667	-20.128	0.59375	0.48758	0.97533 (max o	3	207; 358; 1453	0.8188037	-0.1664353		2.0
	ZINC12296950	-8.4	364.357	-0.6284	10	1	247.45	0.36153	110.7	3.8382	546.1145017766	0.33045	6.8911	-1.9017	0.46154	0.40306	0.95559 (max o	3	3319; 2518; 1485	-0.0746007	-0.9359087		2.0

Structure	Molecule Name	affinity	Total Molweight	cLogP	H-Acceptors	H-Donors	Total Surface Area	Relative PSA	Polar Surface Area	Druglikeness	Kd (nM) @ 20°C	LE from K...	LLE from Kd ...	LELP from Kd (n...	Shape Index	Molecular...	Neighbor Si...	Neig...	Neighbor	Neighbor ...	Neighbor ...	Ring Systems	frequency
	ZINC35455087	-8.4	408.402	-0.1825	9	3	294.59	0.35772	131.75	-0.23288	546.1145017766	0.29627	6.4452	-0.616	0.55172	0.42312	0.93975 (max o	3	1606; 1914; 1915	0.5934925	-0.5173483		2.0
	ZINC04200780	-8.4	395.378	0.1277	11	1	285.51	0.40706	126.17	1.7911	546.1145017766	0.29627	6.135	0.43103	0.58621	0.33217	0.91704 (max o	2	441; 1702	-0.2892211	-0.3798968		2.0
	ZINC04147831	-8.4	427.420	-0.6291	12	1	311.97	0.39145	127.44	3.0257	546.1145017766	0.27715	6.8918	-2.2699	0.6129	0.38691	0.96971 (max o	5	4531; 2061; 348; 141	-0.1580175	-0.3869185		2.0
	ZINC11693967	-8.4	369.380	-2.0506	9	1	254.25	0.27894	103.06	-6.6933	546.1145017766	0.31821	8.3133	-6.4442	0.44444	0.37413	0.90377	1	1794	-0.7430933	-0.4390415		2.0
	ZINC11693967	-8.4	368.372	-1.8617	9	0	265.22	0.30876	101.81	-5.9311	546.1145017766	0.31821	8.1244	-5.8505	0.44444	0.37413	0.91059	1	1795	-0.3285189	-0.9137688		2.0
	ZINC22936815	-8.4	373.499	-5.126	7	3	293.73	0.26116	84.73	5.9199	546.1145017766	0.31821	11.389	-16.109	0.59259	0.51707	0.96064 (max o	3	1554; 1556; 1559	-0.2382376	0.1931043		2.0
	ZINC00538273	-8.4	361.372	-2.287	7	1	267.61	0.28045	77.35	2.8174	546.1145017766	0.33045	8.5497	-6.9209	0.5	0.40788	0.94951 (max o	5	5315; 3122; 2335; 19	0.58785313	-0.3638670		2.0
	ZINC00538273	-8.4	360.364	-2.4186	7	0	253.57	0.2406	76.15	7.0211	546.1145017766	0.33045	8.6813	-7.3191	0.5	0.40788	0.94951 (max o	5	5316; 3123; 2336; 19	0.5848046	-0.3427854		2.0
	ZINC00538273	-8.4	362.380	-3.6473	7	2	256.21	0.25901	80.28	1.7724	546.1145017766	0.33045	9.91	-11.037	0.5	0.34931	0.95857 (max o	2	3124; 2337	-0.0797625	-0.8141386		2.0
	ZINC00643293	-8.4	355.353	0.9373	9	3	256.53	0.39169	122.39	1.8667	546.1145017766	0.33045	5.3254	2.8364	0.57692	0.27801	0.9464 (max of	4	2824; 2709; 2338; 19	0.6627747	0.2324913		2.0
	ZINC03850281	-8.4	419.536	-2.7517	6	0	319.04	0.2569	120.39	-3.811	546.1145017766	0.28639	9.0144	-9.6082	0.46667	0.5131	0.94515 (max o	3	3612; 3171; 3170	-0.4510176	-0.777052		2.0
	ZINC03873157	-8.4	352.360	-2.4768	6	2	235.7	0.19733	83.22	1.5859	546.1145017766	0.34367	8.7395	-7.207	0.48	0.35916	0.96832	1	3176	0.7004953	0.45464018		2.0
	ZINC04084055	-8.4	375.467	-1.0121	7	2	293.35	0.28256	117.18	4.9133	546.1145017766	0.31821	7.2748	-3.1806	0.59259	0.4661	0.93046 (max o	3	5366; 3150; 2037	0.30871725	0.35433018		2.0
	ZINC04217387	-8.4	409.461	-3.0564	7	1	310.67	0.25419	117.18	3.3492	546.1145017766	0.28639	9.3191	-10.672	0.53333	0.48843	0.90581	1	3588	-0.77058	-0.0296139		2.0
	ZINC05398973	-8.4	373.431	-0.614	8	1	279.29	0.28497	92.08	-5.9161	546.1145017766	0.31821	6.8767	-1.9295	0.66667	0.40992	0.91731 (max o	3	4122; 3700; 593	-0.1149693	-0.2266553		2.0
	ZINC05851742	-8.4	395.434	-2.8833	7	1	299.67	0.26352	117.18	-7.3863	546.1145017766	0.29627	9.146	-9.7322	0.48276	0.48159	0.95908	1	3590	0.5108041	-0.7888706		2.0
	ZINC06131107	-8.4	406.481	-0.8669	9	2	296.38	0.31102	108.05	1.685	546.1145017766	0.29627	7.1296	-2.9261	0.55172	0.35969	0.981 (max of 8	8	4922; 4488; 4487; 40	-0.6113172	0.01295888		2.0
	ZINC06907160	-8.4	358.373	-1.0895	8	1	260.15	0.32673	109.85	6.6511	546.1145017766	0.33045	7.3522	-3.297	0.46154	0.50882		0		-0.0247198	-0.8677246		2.0
	ZINC08643398	-8.4	384.452	-0.1054	8	3	269.61	0.34431	124.55	-3.5883	546.1145017766	0.33045	6.3681	-0.31896	0.57692	0.5115	0.90719 (max o	5	6750; 4655; 4223; 33	0.20349105	-0.3402838		2.0
	ZINC12603550	-8.4	411.428	-0.8099	9	4	300.35	0.32878	120.36	1.4185	546.1145017766	0.29627	7.0726	-2.7337	0.65517	0.58153	0.92486	1	2879	0.41250512	-0.4512110		2.0
	ZINC12603816	-8.4	391.874	0.1581	6	4	285.87	0.23941	95.4	-0.085638	546.1145017766	0.31821	6.1046	0.49684	0.62963	0.45088	0.92739 (max o	8	6756; 6748; 6223; 58	0.13932443	-0.4754293		2.0
	ZINC12605077	-8.4	370.360	-1.0908	9	1	241.4	0.38782	129.47	4.965	546.1145017766	0.34367	7.3535	-3.174	0.56	0.48318	0.92993 (max o	2	3259; 2083	-0.7467510	0.5600165		2.0
	ZINC12662593	-8.4	411.405	-0.4553	10	4	291.26	0.38464	145.99	0.28886	546.1145017766	0.29627	6.718	-1.5368	0.58621	0.5072	0.95908 (max o	3	2532; 1050; 1844	0.04644650	-0.9495654		2.0
	ZINC13521034	-8.4	406.473	0.5411	7	4	272.78	0.32436	124.29	-2.2987	546.1145017766	0.29627	5.7216	1.8264	0.37931	0.34283	0.90502	1	2797	-0.8386013	0.1846057		2.0
	ZINC13532105	-8.4	428.420	-2.0472	10	4	329.82	0.37572	156.89	-9.3549	546.1145017766	0.27715	8.3099	-7.3866	0.67742	0.60085	0.96682 (max o	6	6156; 5098; 4984; 41	-0.0141943	-0.4398244		2.0
	ZINC14879985	-8.4	442.423	-5.1384	10	6	305.32	0.45415	185.65	-1.4141	546.1145017766	0.26849	11.401	-19.138	0.375	0.34214	0.87202 (max o	2	167; 2185	-0.4255392	-0.5377252		2.0
	ZINC14879985	-8.4	441.415	-3.7721	10	5	291.28	0.42784	184.45	1.5642	546.1145017766	0.26849	10.035	-14.049	0.375	0.34052	0.87202 (max o	2	2182; 2183	-0.4416059	-0.5224650		2.0
	ZINC14879985	-8.4	441.415	-7.3761	10	5	306.5	0.45625	188.48	-0.50838	546.1145017766	0.26849	13.639	-27.472	0.375	0.3649	0.91243 (max o	3	403; 1851; 2183	-0.3651196	-0.523702		2.0
	ZINC14879985	-8.4	440.407	-6.0098	10	4	292.46	0.43014	187.28	2.305	546.1145017766	0.26849	12.273	-22.384	0.375	0.36334	0.91522 (max o	4	2187; 399; 2185; 184	-0.4014986	-0.5119583		2.0
	ZINC14879985	-8.4	442.423	-5.3001	10	6	305.32	0.45415	185.65	-0.49153	546.1145017766	0.26849	11.563	-19.74	0.375	0.3649	0.91522 (max o	3	400; 1316; 1846	-0.7550374	-0.5097657		2.0
	ZINC14879985	-8.4	441.415	-3.9338	10	5	291.28	0.42784	184.45	1.8826	546.1145017766	0.26849	10.197	-14.652	0.375	0.36334	0.91799 (max o	4	402; 1318; 1849; 185	-0.0763633	0.71437514		2.0
	ZINC19702943	-8.4	355.345	-0.881	7	2	259.87	0.31246	112.5	-0.13315	546.1145017766	0.33045	7.1437	-2.6661	0.57692	0.4385	0.87576	1	2574	-0.2364522	-0.8984615		2.0
	ZINC20464813	-8.4	435.398	0.3741	9	4	301.95	0.31697	112.52	-4.994	546.1145017766	0.28639	5.8886	1.3063	0.56667	0.59676	0.9339 (max of	2	4736; 3393	-0.3846011	-0.0792977		2.0
	ZINC20465610	-8.4	443.522	-1.9434	9	4	346.15	0.29701	108.59	3.5715	546.1145017766	0.26849	8.2061	-7.2382	0.59375	0.50343	0.93139	1	3403	-0.3279682	-0.6738189		2.0
	ZINC20465610	-8.4	443.522	-1.8115	9	4	321.14	0.24226	108.64	5.5052	546.1145017766	0.26849	8.0742	-6.747	0.59375	0.48613	0.93139 (max o	4	7052; 5932; 3404; 22	-0.4269509	0.01586087		2.0
	ZINC20504107	-8.4	434.559	-0.1455	8	4	320.52	0.33464	129.39	5.1704	546.1145017766	0.28639	6.4082	-0.50805	0.53333	0.45668	0.92566 (max o	2	6743; 5161	0.4316522	0.12221707		2.0
	ZINC22923579	-8.4	437.626	-3.8147	8	3	363.9	0.24683	70.76	6.1025	546.1145017766	0.27715	10.077	-13.764	0.58065	0.49852	0.90825 (max o	7	5223; 3922; 3874; 30	-0.1590672	0.54236144		2.0
	ZINC31160379	-8.4	422.389	-0.4109	11	3	301.01	0.39975	143.86	0.32355	546.1145017766	0.28639	6.6736	-1.4348	0.53333	0.43436	0.9561 (max of	4	5606; 5196; 3050; 30	0.41500497	-0.6841038		2.0
	ZINC31167954	-8.4	398.494	0.9599	7	4	288.68	0.29597	116.45	-3.8942	546.1145017766	0.30685	5.3028	3.1283	0.46429	0.43645	0.96519 (max o	4	6619; 5611; 5208; 19	-0.6922417	0.07590411		2.0
	ZINC35456061	-8.4	391.482	-0.6403	6	3	280.75	0.30016	113.35	-3.3875	546.1145017766	0.30685	6.903	-2.0867	0.42857	0.36958	0.9678 (max of	5	4410; 3459; 2659; 84	-0.1104018	0.4191862		2.0
	ZINC35456066	-8.4	391.482	-0.6403	6	3	280.75	0.30016	113.35	-3.3875	546.1145017766	0.30685	6.903	-2.0867	0.42857	0.36958	0.9678 (max of	5	4410; 2659; 411; 654	-0.1100254	0.4396292		2.0
	ZINC67903446	-8.4	360.358	-2.0162	9	4	247.63	0.4258	134.91	-2.1618	546.1145017766	0.34367	8.2789	-5.8667	0.48	0.50401	0.95318 (max o	3	2686; 2685; 1618	0.5908078	0.6377695		2.0
	ZINC67913887	-8.4	389.332	-4.9553	11	4	258.42	0.51374	175.04	-4.8669	546.1145017766	0.31821	11.218	-15.572	0.44444	0.41256	0.95493 (max o	3	4865; 4437; 2689	-0.3443622	0.8819057		2.0

Structure	Molecule Name	affinity	Total Molweight	cLogP	H-Acceptors	H-Donors	Total Surface Area	Relative PSA	Polar Surface Area	Druglikeness	Kd (nM) @ 20°C	LE from K...	LLE from Kd ...	LELP from Kd (n...	Shape Index	Molecular...	Neighbor Si...	Neig...	Neighbor	Neighbor ...	Neighbor ...	Ring Systems	frequency
	ZINC71405302	-8.4	426.540	-1.1345	10	4	312.53	0.39225	148.18	6.7687	546.1145017766	0.29627	7.3972	-3.8293	0.44828	0.51985	0.89174 (max o	3	3697; 2837; 1457	-0.8010659	-0.1413802		2.0
	ZINC72320119	-8.4	448.507	-1.5165	10	7	305.77	0.40795	177.14	-8.1275	546.1145017766	0.27715	7.7792	-5.4717	0.41935	0.52422	0.9884 (max of	3	6913; 3973; 3972	-0.5068478	-0.783971		2.0
	ZINC77257222	-8.4	373.445	-0.9757	7	2	271.05	0.39509	149.26	-4.2177	546.1145017766	0.34367	7.2384	-2.8391	0.52	0.49896	0.97118 (max o	3	3984; 3983; 2330	0.4909287	0.63153917		2.0
	ZINC77257217	-8.4	373.445	-0.9757	7	2	271.05	0.39509	149.26	-4.2177	546.1145017766	0.34367	7.2384	-2.8391	0.52	0.49941	0.97118 (max o	3	3984; 3983; 2331	0.4592295	0.6531382		2.0
	ZINC79189880	-8.4	395.342	-0.1783	8	0	280.74	0.33269	111.19	-1.4098	546.1145017766	0.29627	6.441	-0.60182	0.51724	0.36797	0.93975 (max o	3	4457; 2703; 2702	-0.7233794	-0.3686746		2.0
	ZINC00537947	-8.4	362.360	-3.5412	8	1	265.41	0.29615	80.59	2.9498	546.1145017766	0.33045	9.8039	-10.716	0.5	0.40788	0.9066 (max of	5	5315; 3122; 2335; 19	0.62567514	-0.3573365		1.0
	ZINC00537947	-8.4	361.352	-3.6728	8	0	251.37	0.25683	79.39	7.0242	546.1145017766	0.33045	9.9355	-11.115	0.5	0.40788	0.9066 (max of	5	5316; 3123; 2336; 19	0.60717237	-0.3715093		1.0
	ZINC01530578	-8.4	376.388	-3.4633	8	1	256.5	0.4414	154.97	10.967	546.1145017766	0.33045	9.726	-10.481	0.5	0.34338	0.93361 (max o	5	4982; 4635; 4498; 36	0.05252325	-0.0360239		1.0
	ZINC01700876	-8.4	358.301	-0.3562	8	6	221.26	0.47311	155.52	0.3325	546.1145017766	0.33045	6.6189	-1.0779	0.38462	0.28654		0		0.93708736	-0.0842730		1.0
	ZINC02030982	-8.4	390.419	-0.0625	9	4	272.01	0.44351	154.5	1.9887	546.1145017766	0.31821	6.3252	-0.19641	0.48148	0.39749	0.95493 (max o	2	4693; 3376	0.34020394	-0.4960152		1.0
	ZINC02137596	-8.4	394.406	-0.6841	8	2	300.71	0.30933	122.13	5.2	546.1145017766	0.29627	6.9468	-2.3091	0.62069	0.53365	0.93985 (max o	2	2979; 1091	0.12616332	-0.2488043		1.0
	ZINC03795983	-8.4	410.827	-1.8541	6	2	248.76	0.21406	94.25	0.53	546.1145017766	0.30685	8.1168	-6.0424	0.42857	0.25826		0		0.90552044	-0.1751976		1.0
	ZINC03839328	-8.4	392.454	-1.2089	9	2	282.62	0.32616	108.05	4.1271	546.1145017766	0.30685	7.4716	-3.9398	0.53571	0.31363	0.93069 (max o	6	4488; 4037; 2752; 27	-0.5598548	-0.0255723		1.0
	ZINC03839433	-8.4	445.518	-1.0479	10	2	313.43	0.31513	119.13	7.8767	546.1145017766	0.26849	7.3106	-3.9029	0.53125	0.3368	0.93157 (max o	4	78; 699; 898; 1706	-0.5332699	-0.0030143		1.0
	ZINC03840144	-8.4	436.483	-3.0796	10	5	321.33	0.39529	167.89	0.92706	546.1145017766	0.27715	9.3423	-11.112	0.51613	0.53717	0.90776 (max o	3	3601; 1378; 2009	0.57278764	0.7108809		1.0
	ZINC03842153	-8.4	439.471	-0.1406	10	1	334.66	0.30676	121.8	5.0128	546.1145017766	0.26849	6.4033	-0.52367	0.5	0.53433	0.89799 (max o	2	6454; 4494	0.00596372	0.40206888		1.0
	ZINC03849249	-8.4	393.376	2.05	8	3	252.85	0.37457	153.05	-8.547	546.1145017766	0.31821	4.2127	6.4423	0.51852	0.3257		0		0.8286378	0.42204833		1.0
	ZINC03852689	-8.4	360.369	-1.3452	9	3	252.2	0.36661	118.19	-4.0555	546.1145017766	0.33045	7.6079	-4.0708	0.53846	0.44079	0.93033 (max o	3	4495; 2765; 1110	0.7981681	0.45384535		1.0
	ZINC03869586	-8.4	367.333	-4.5103	10	3	250.92	0.45505	152.48	4.5322	546.1145017766	0.33045	10.773	-13.649	0.5	0.36505	0.89253	1	2075	-0.6718395	0.257958		1.0
	ZINC03977911	-8.4	394.437	0.167	6	4	262.92	0.29849	115.06	2.4849	546.1145017766	0.30685	6.0957	0.54425	0.46429	0.31582	0.93385 (max o	4	3621; 2833; 2774; 16	0.49081406	0.5961498		1.0
	ZINC04039105	-8.4	362.337	-0.1746	9	3	254.7	0.39391	125.4	0.57877	546.1145017766	0.33045	6.4373	-0.52837	0.57692	0.46019	0.88391 (max o	2	484; 998	-0.5898779	-0.7340989		1.0
	ZINC04062214	-8.4	374.412	0.3524	7	3	264.03	0.33379	122.41	1.9657	546.1145017766	0.31821	5.9103	1.1074	0.44444	0.28507	0.94905 (max o	3	2781; 1397; 1398	-0.8764178	0.18235226		1.0
	ZINC04083331	-8.4	409.484	-0.6973	7	2	316.09	0.26224	117.18	5.0172	546.1145017766	0.28639	6.96	-2.4348	0.6	0.44464	0.93686 (max o	2	4081; 2038	0.30316812	0.31601256		1.0
	ZINC04260173	-8.4	359.405	0.697	7	1	257	0.35856	111.1	8.5988	546.1145017766	0.34367	5.5657	2.0281	0.56	0.4651	0.88432 (max o	2	3230; 2066	-0.2851329	0.1435578		1.0
	ZINC04260987	-8.4	374.396	-0.6989	9	1	279.25	0.33415	96.73	-1.1151	546.1145017766	0.31821	6.9616	-2.1963	0.66667	0.40375	0.98141 (max o	4	2812; 449; 1419; 172	-0.2539095	-0.2044701		1.0
	ZINC04270944	-8.4	372.448	0.4176	7	1	268.77	0.31592	102.89	9.7728	546.1145017766	0.33045	5.8451	1.2637	0.53846	0.43857	0.88432 (max o	2	2810; 2062	-0.3003080	0.12636593		1.0
	ZINC04277773	-8.4	413.472	0.8879	8	0	295.75	0.23591	79.39	6.2336	546.1145017766	0.28639	5.3748	3.1003	0.6	0.41973	0.85972 (max o	2	3168; 2817	-0.2326796	0.67085123		1.0
	ZINC04896202	-8.4	378.463	0.5647	6	4	259.68	0.30222	115.06	3.3157	546.1145017766	0.31821	5.698	1.7746	0.48148	0.32369		0		-0.7256204	-0.6879592		1.0
	ZINC05140888	-8.4	367.333	-4.5103	10	3	250.92	0.45505	152.48	4.5322	546.1145017766	0.33045	10.773	-13.649	0.5	0.37718	0.89253 (max o	3	6628; 6629; 2016	-0.6605814	0.27892372		1.0
	ZINC05396067	-8.4	422.448	-0.098	11	1	310.33	0.35372	120.18	2.6001	546.1145017766	0.27715	6.3607	-0.3536	0.58065	0.33573	0.89657 (max o	3	4493; 2426; 1702	-0.2698202	-0.3732510		1.0
	ZINC05398954	-8.4	351.425	-1.1662	8	1	259.81	0.30634	92.08	-9.6511	546.1145017766	0.34367	7.4289	-3.3934	0.64	0.39341		0		0.7558399	0.583225		1.0
	ZINC05415788	-8.4	357.361	-0.3434	8	1	235.24	0.33332	106.61	4.1073	546.1145017766	0.35799	6.6061	-0.95925	0.58333	0.47695	0.84296	1	2156	-0.7502556	0.5383076		1.0
	ZINC05433814	-8.4	397.454	-5.7905	11	3	291.91	0.37755	138.93	2.8648	546.1145017766	0.30685	12.053	-18.871	0.5	0.56539	0.93985 (max o	5	3699; 3692; 2838; 60	-0.0832720	0.21661597		1.0
	ZINC05434086	-8.4	351.365	0.178	8	0	255.23	0.28096	86.71	5.5971	546.1145017766	0.33045	6.0847	0.53866	0.57692	0.40617	0.85045 (max o	2	5678; 3270	0.5738962	0.5246891		1.0
	ZINC05434144	-8.4	442.499	-0.4533	10	2	321.56	0.38711	152.84	6.634	546.1145017766	0.27715	6.716	-1.6356	0.41935	0.44601	0.92863 (max o	4	3694; 2203; 592; 174	-0.0143458	0.24293591		1.0
	ZINC08295000	-8.4	394.470	-0.685	9	6	295.84	0.37574	139.79	-0.99133	546.1145017766	0.30685	6.9477	-2.2324	0.57143	0.50807	0.89424 (max o	4	4097; 3596; 2748; 27	-0.2303409	-0.4964185		1.0
	ZINC08299107	-8.4	424.591	-2.2761	7	2	357.09	0.22566	59.9	1.1942	546.1145017766	0.27715	8.5388	-8.2125	0.6129	0.5437	0.97423 (max o	6	5005; 3274; 3278; 32	0.41207573	-0.0558722		1.0
	ZINC08299856	-8.4	390.506	-1.9904	8	2	301.51	0.28553	84.92	-1.9808	546.1145017766	0.30685	8.2531	-6.4866	0.57143	0.50823	0.97037 (max o	4	5439; 4154; 2104; 17	0.22544391	0.2123527		1.0
	ZINC08299858	-8.4	390.506	-1.9904	8	2	301.51	0.28553	84.92	-1.9808	546.1145017766	0.30685	8.2531	-6.4866	0.57143	0.50823	0.97037 (max o	4	5440; 3717; 3275; 21	0.24781233	0.24309625		1.0
	ZINC08643702	-8.4	388.446	-2.4495	9	3	282.29	0.31648	117.32	-1.5575	546.1145017766	0.30685	8.7122	-7.9828	0.60714	0.46783	0.88589	1	2887	0.8491065	-0.3892582		1.0
	ZINC08958839	-8.4	434.359	-1.9186	10	4	314.46	0.4129	178.92	0.88599	546.1145017766	0.26849	8.1813	-7.1459	0.5625	0.48526		0		0.12476205	-0.9564133		1.0
	ZINC08964694	-8.4	414.421	1.7601	11	1	300.02	0.33058	112.73	5.5982	546.1145017766	0.28639	4.5026	6.1458	0.46667	0.36424	0.8319	1	2132	0.90171176	0.04749074		1.0
	ZINC08964694	-8.4	415.429	-0.2177	11	2	289.05	0.30517	113.98	4.9468	546.1145017766	0.28639	6.4804	-0.76015	0.46667	0.36424	0.8319	1	2131	0.893331	0.02644124		1.0

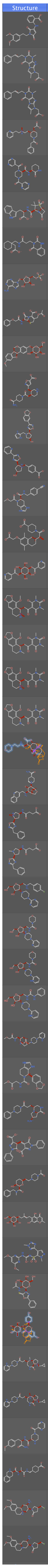

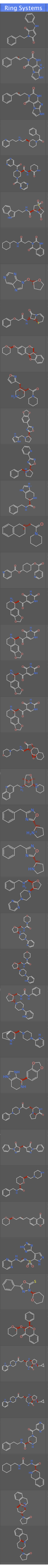

Structure	Molecule Name	affinity	Total Molweight	cLogP	H-Acceptors	H-Donors	Total Surface Area	Relative PSA	Polar Surface Area	Druglikeness	Kd (nM) @ 20°C	LE from K...	LLE from Kd ...	LELP from Kd (n...	Shape Index	Molecular...	Neighbor Si...	Neig...	Neighbor	Neighbor ...	Neighbor ...	Ring Systems	frequency
	ZINC11613110	-8.4	395.390	-0.4725	8	1	298.81	0.29587	108	3.0967	546.1145017766	0.29627	6.7352	-1.5949	0.55172	0.45436		0		0.8426045	0.43515068		1.0
	ZINC12296449	-8.4	393.402	-2.9112	9	1	281.68	0.2674	104.23	-6.8832	546.1145017766	0.29627	9.1739	-9.8263	0.55172	0.41468	0.9218 (max of	2	1790; 2141	-0.0054932	0.884581		1.0
	ZINC12296449	-8.4	392.394	-0.9334	9	0	292.65	0.29486	102.98	-6.1318	546.1145017766	0.29627	7.1961	-3.1506	0.55172	0.41468	0.9218 (max of	3	3079; 1791; 2140	0.00931419	0.90180695		1.0
	ZINC12480735	-8.4	423.555	0.0788	6	1	357.05	0.20297	77.77	4.3618	546.1145017766	0.27715	6.1839	0.28432	0.58065	0.61092	0.96018 (max o	4	6212; 5843; 5047; 50	-0.1794800	-0.1230989		1.0
	ZINC12605296	-8.4	438.447	-1.8831	12	2	323.14	0.39011	150.38	5.0549	546.1145017766	0.26849	8.1458	-7.0137	0.4375	0.46221	0.97743 (max o	8	4133; 2929; 2572; 24	-0.7103172	-0.4144242		1.0
	ZINC12884078	-8.4	418.449	-1.3073	9	2	281.5	0.39425	147.85	7.7574	546.1145017766	0.29627	7.57	-4.4126	0.44828	0.3838	0.83367	1	7685	-0.2033888	0.93605244		1.0
	ZINC12893673	-8.4	386.427	-0.513	8	2	292.26	0.3179	118.64	0.17766	546.1145017766	0.30685	6.7757	-1.6718	0.53571	0.59644	0.95461	1	951	0.7425712	-0.6297647		1.0
	ZINC13543718	-8.4	361.206	-7.7128	13	3	233.65	0.67122	210.77	-29.025	546.1145017766	0.35799	13.976	-21.545	0.54167	0.49645		0		0.40568814	0.81676626		1.0
	ZINC13544614	-8.4	405.406	-1.7746	9	1	285.5	0.4166	150.37	10.41	546.1145017766	0.30685	8.0373	-5.7834	0.60714	0.42127	0.93985 (max o	4	6697; 4258; 2368; 20	0.0713711	-0.9049765		1.0
	ZINC15657754	-8.4	432.424	1.1764	9	4	295.2	0.34688	127.07	-4.4264	546.1145017766	0.27715	5.0863	4.2446	0.54839	0.32135	0.91487	1	572	0.37736621	0.8248019		1.0
	ZINC15672793	-8.4	378.427	-2.4822	9	2	283.39	0.37066	146.17	7.0281	546.1145017766	0.31821	8.7449	-7.8005	0.59259	0.59915	0.96064 (max o	2	5111; 1514	-0.0942356	0.84615004		1.0
	ZINC15675012	-8.4	356.361	-1.26	9	3	247.04	0.35091	110.78	5.0517	546.1145017766	0.33045	7.5227	-3.813	0.53846	0.35374		0		0.38885602	-0.9115264		1.0
	ZINC15675034	-8.4	366.376	-1.739	8	2	287.5	0.36532	116.61	2.0705	546.1145017766	0.31821	8.0017	-5.4649	0.59259	0.3982	0.97149 (max o	3	2971; 2622; 2567	0.47845587	-0.6316254		1.0
	ZINC15676176	-8.4	354.389	-2.5176	8	3	265.65	0.29603	112.36	1.4229	546.1145017766	0.33045	8.7803	-7.6187	0.57692	0.50875	0.87452	1	2195	0.205719	-0.239984		1.0
	ZINC15709150	-8.4	415.484	-1.5759	7	0	319.23	0.25446	109.8	4.6925	546.1145017766	0.28639	7.8386	-5.5026	0.56667	0.47795	0.89979	1	1409	0.42853925	-0.7741074		1.0
	ZINC18169450	-8.4	390.347	-1.2056	10	4	272.94	0.4412	159.3	-2.1654	546.1145017766	0.30685	7.4683	-3.929	0.57143	0.33903		0		-0.8952606	-0.3383001		1.0
	ZINC20112959	-8.4	417.417	-2.9582	10	1	307.98	0.35096	112.88	2.6735	546.1145017766	0.28639	9.2209	-10.329	0.43333	0.452	0.91374 (max o	4	6547; 2214; 2215; 22	-0.3595285	0.5109709		1.0
	ZINC20112959	-8.4	416.409	-0.8485	10	0	293.94	0.31996	111.68	5.0321	546.1145017766	0.28639	7.1112	-2.9627	0.43333	0.4636	0.96343 (max o	3	2214; 2215; 2212	-0.3381498	0.50575393		1.0
	ZINC20112961	-8.4	417.417	-2.9582	10	1	307.98	0.35096	112.88	2.6735	546.1145017766	0.28639	9.2209	-10.329	0.43333	0.45338	0.91374 (max o	4	6548; 2212; 2213; 22	-0.3313576	0.48579413		1.0
	ZINC20112961	-8.4	416.409	-0.8485	10	0	293.94	0.31996	111.68	5.0321	546.1145017766	0.28639	7.1112	-2.9627	0.43333	0.45932	0.96343 (max o	3	2212; 2213; 2214	-0.3530418	0.49104512		1.0
	ZINC20114010	-8.4	379.519	-0.8859	6	1	278.64	0.22244	67.91	-0.50806	546.1145017766	0.31821	7.1486	-2.784	0.55556	0.47317	0.93373 (max o	2	4507; 3380	-0.6093899	0.53030884		1.0
	ZINC20463891	-8.4	413.496	-5.7487	8	3	309.56	0.31945	118.55	2.0725	546.1145017766	0.28639	12.011	-20.073	0.56667	0.43737	0.88255 (max o	4	7218; 7216; 5581; 33	0.61530995	-0.0297815		1.0
	ZINC20464247	-8.4	358.397	-2.027	8	2	291.62	0.36163	112.59	2.3499	546.1145017766	0.33045	8.2897	-6.1341	0.65385	0.49827	0.87452 (max o	4	3327; 2918; 2149; 22	0.6295564	-0.3355336		1.0
	ZINC20464247	-8.4	357.389	-2.1586	8	1	277.58	0.32935	111.39	5.4155	546.1145017766	0.33045	8.4213	-6.5323	0.65385	0.49678	0.83744	1	2223	0.64612174	-0.3484334		1.0
	ZINC20465093	-8.4	418.491	-1.5142	8	3	307.25	0.23753	98.56	2.5628	546.1145017766	0.28639	7.7769	-5.2872	0.6	0.43064	0.96589 (max o	7	6576; 3848; 3845; 25	-0.5067161	0.27350298		1.0
	ZINC20465598	-8.4	422.504	-3.3527	10	4	325.97	0.32629	111.83	3.3544	546.1145017766	0.28639	9.6154	-11.707	0.56667	0.5465	0.96589 (max o	5	7053; 5939; 5142; 33	-0.4005400	-0.6077298		1.0
	ZINC20465598	-8.4	422.504	-3.2208	10	4	300.96	0.2703	111.88	5.3067	546.1145017766	0.28639	9.4835	-11.246	0.56667	0.52857	0.89505 (max o	7	7052; 5940; 5930; 51	-0.4003431	0.07358184		1.0
	ZINC20465639	-8.4	428.511	-1.7476	9	5	310.34	0.2554	111.44	5.4344	546.1145017766	0.27715	8.0103	-6.3056	0.58065	0.49096	0.91704 (max o	6	7052; 6267; 5940; 34	-0.4076438	0.01625859		1.0
	ZINC20503886	-8.4	389.542	-3.5613	7	3	305.82	0.19355	63.39	6.6787	546.1145017766	0.30685	9.824	-11.606	0.67857	0.56261	0.87244	1	3537	-0.0544908	0.7425542		1.0
	ZINC20760997	-8.4	359.361	-0.7481	9	3	250.44	0.33972	107.03	0.41682	546.1145017766	0.33045	7.0108	-2.2639	0.5	0.46467	0.94008	1	3862	-0.3045178	-0.9391455		1.0
	ZINC22936815	-8.4	372.491	-3.0163	7	2	279.69	0.22407	83.53	6.6191	546.1145017766	0.31821	9.279	-9.4789	0.59259	0.49485	0.96064 (max o	2	1555; 1557	-0.6071738	-0.7392764		1.0
	ZINC24717028	-8.4	416.348	-3.1485	10	0	295.83	0.37467	145.6	4.7927	546.1145017766	0.27715	9.4112	-11.36	0.48387	0.33329	0.94223	1	2388	-0.5923731	-0.7887155		1.0
	ZINC30878591	-8.4	403.521	0.9303	7	1	314.87	0.19001	73.32	10.099	546.1145017766	0.29627	5.3324	3.1401	0.62069	0.59979	0.84147 (max o	2	7409; 2620	-0.3653217	0.36342704		1.0
	ZINC31161031	-8.4	438.427	0.6455	10	6	309.83	0.3926	166.14	-8.84	546.1145017766	0.27715	5.6172	2.3291	0.54839	0.50096	0.86981	1	527	-0.7059874	-0.602683		1.0
	ZINC31318699	-8.4	423.393	-2.3893	15	1	303.32	0.54527	195.63	-0.15837	546.1145017766	0.29627	8.652	-8.0647	0.51724	0.52646		0		-0.8821804	-0.266545		1.0
	ZINC32594259	-8.4	381.408	-0.7094	9	4	269.02	0.47156	155.94	-1.459	546.1145017766	0.33045	6.9721	-2.1468	0.46154	0.46213	0.84672	1	2945	0.02174350	0.91859853		1.0
	ZINC33830716	-8.4	434.396	0.3932	10	6	291.63	0.4171	166.14	1.8995	546.1145017766	0.27715	5.8695	1.4187	0.41935	0.34847	0.87288 (max o	5	5273; 50; 1069; 1343	0.72829324	-0.4819309		1.0
	ZINC35442491	-8.4	442.606	-3.1523	8	3	336.63	0.19541	74.47	8.4971	546.1145017766	0.26849	9.415	-11.741	0.59375	0.47727	0.85165 (max o	3	6320; 3486; 2282	0.75292355	0.17109756		1.0
	ZINC35442491	-8.4	443.614	-5.262	8	4	350.67	0.22762	75.67	7.6426	546.1145017766	0.26849	11.525	-19.598	0.59375	0.49349	0.85165 (max o	2	6319; 2281	0.7345417	0.1841215		1.0
	ZINC35447008	-8.4	412.449	-1.2919	10	3	314.33	0.35886	139.54	8.3428	546.1145017766	0.28639	7.5546	-4.511	0.6	0.50985	0.83844	1	1674	-0.3162886	-0.2908986		1.0
	ZINC35485054	-8.4	386.470	-3.8255	7	3	300	0.2926	110.36	4.1054	546.1145017766	0.30685	10.088	-12.467	0.64286	0.54948		0		-0.2565399	-0.7928324		1.0
	ZINC38358483	-8.4	379.474	-4.2742	6	2	290.07	0.2367	70.85	1.02	546.1145017766	0.31821	10.537	-13.432	0.55556	0.33703	0.81211	1	2297	0.18254337	0.97058094		1.0
	ZINC38358483	-8.4	378.466	-2.1645	6	1	276.03	0.19788	69.65	1.7187	546.1145017766	0.31821	8.4272	-6.8021	0.55556	0.33703	0.81211	1	2296	0.16478121	0.9688065		1.0

Structure	Molecule Name	affinity	Total Molweight	cLogP	H-Acceptors	H-Donors	Total Surface Area	Relative PSA	Polar Surface Area	Druglikeness	Kd (nM) @ 20°C	LE from K...	LLE from Kd ...	LELP from Kd (n...	Shape Index	Molecular...	Neighbor Si...	Neig...	Neighbor	Neighbor ...	Neighbor ...	Ring Systems	frequency
	ZINC39559782	-8.4	389.423	-0.5636	7	1	285.89	0.30256	112.96	-3.4687	546.1145017766	0.30685	6.8263	-1.8367	0.5	0.33872	0.95148	1	1327	-0.6126209	-0.7880441		1.0
	ZINC49180971	-8.4	430.404	-2.179	11	6	295.89	0.4449	175.37	0.02889	546.1145017766	0.28639	8.4417	-7.6085	0.56667	0.45153	0.81754	1	2961	0.8934084	-0.0745203		1.0
	ZINC49543643	-8.4	378.343	-2.9005	9	1	275.59	0.38263	131.7	4.9434	546.1145017766	0.30685	9.1632	-9.4526	0.46429	0.36416	0.95709 (max o	2	1934; 2311	0.61881214	0.30503288		1.0
	ZINC49543643	-8.4	379.351	-1.343	9	2	271.51	0.37339	123.16	4.8497	546.1145017766	0.30685	7.6057	-4.3768	0.46429	0.36416	0.94573 (max o	2	1935; 2310	0.62129533	0.28302902		1.0
	ZINC67902321	-8.4	388.447	0.2609	8	1	269.67	0.34027	115.33	6.0463	546.1145017766	0.31821	6.0018	0.8199	0.55556	0.41556		0		0.5548653	0.7646719		1.0
	ZINC67912704	-8.4	366.408	-0.5383	7	4	237.47	0.37259	124.29	0.78517	546.1145017766	0.33045	6.801	-1.629	0.34615	0.32626	0.92702 (max o	3	3542; 1066; 1619	0.3591862	0.6238153		1.0
	ZINC67912709	-8.4	368.424	-0.6037	7	5	238.11	0.37184	127.45	-0.51541	546.1145017766	0.33045	6.8664	-1.8269	0.34615	0.34106	0.92368 (max o	4	5672; 3104; 3103; 10	0.33173075	0.6005715		1.0
	ZINC70698880	-8.4	440.467	-0.4019	10	1	310.66	0.36973	132.45	-4.4543	546.1145017766	0.27715	6.6646	-1.4501	0.51613	0.50333	0.92304	1	684	0.21621053	-0.9312998		1.0
	ZINC77257455	-8.4	388.368	-1.827	10	4	263.15	0.45024	151.98	-6.054	546.1145017766	0.31821	8.0897	-5.7415	0.48148	0.37688	0.92739	1	1294	0.79495835	0.01247101		1.0
	ZINC79189446	-8.4	427.405	-2.0341	11	5	289.88	0.42117	158.38	0.38798	546.1145017766	0.28639	8.2968	-7.1026	0.43333	0.43859	0.85465 (max o	2	2704; 1629	0.725162	-0.3962993		1.0
	ZINC31167057	-8.4	367.373	-0.8653	8	2	290.28	0.34312	133.19	-7.5278	546.1145017766	0.33045	7.128	-2.6186	0.57692	0.3882	0.96998 (max o	5	6009; 5239; 4378; 43	-0.0378635	0.82748085		0.0
	ZINC31167050	-8.4	367.373	-0.8653	8	2	290.28	0.34312	133.19	-7.5278	546.1145017766	0.33045	7.128	-2.6186	0.57692	0.3849	0.97269 (max o	5	6009; 5239; 4378; 43	-0.0252451	0.8117486		0.0
	ZINC02102327	-8.3	389.383	-2.1296	9	2	293.19	0.373	133.86	-0.83321	648.3946641435	0.30319	8.3178	-7.0239	0.60714	0.47708	0.97037 (max o	7	3578; 3133; 1355; 13	0.23261772	0.01096124		172.0
	ZINC12901873	-8.3	361.329	-2.9137	9	2	270.46	0.40435	133.86	-5.3598	648.3946641435	0.32652	9.1019	-8.9236	0.61538	0.52545	0.96389 (max o	5	3132; 2947; 287; 216	0.18928026	-0.1020593		172.0
	ZINC02102576	-8.3	404.398	-2.0709	10	4	301.06	0.42427	170.88	2.7316	648.3946641435	0.29274	8.2591	-7.0742	0.58621	0.56893	0.91248 (max o	5	3574; 3139; 2601; 10	0.06185207	0.20259446		172.0
	ZINC02115854	-8.3	417.436	-1.4427	9	2	321.24	0.34043	133.86	-1.2647	648.3946641435	0.28298	7.6309	-5.0982	0.56667	0.52248	0.9561 (max of	4	3145; 2727; 2723; 13	0.14437683	-0.0564073		172.0
	ZINC02091935	-8.3	375.356	-2.4735	9	2	280.93	0.38928	133.86	-0.83321	648.3946641435	0.31442	8.6617	-7.8668	0.62963	0.55841	0.96617 (max o	7	3579; 3563; 3561; 31	0.22050938	-0.0369205		172.0
	ZINC12901539	-8.3	403.410	-1.8817	9	2	306.22	0.35713	133.86	-2.8639	648.3946641435	0.29274	8.0699	-6.4279	0.55172	0.5329	0.93091 (max o	3	4022; 2166; 2545	0.1680956	-0.1078032		172.0
	ZINC02099408	-8.3	417.436	-1.2066	9	2	324	0.33753	133.86	-5.8841	648.3946641435	0.28298	7.3948	-4.2639	0.63333	0.48743	0.98037 (max o	8	4007; 4004; 3578; 35	0.30250302	0.02812432		172.0
	ZINC02134571	-8.3	375.356	-2.4593	9	2	284.22	0.38477	133.86	-0.45242	648.3946641435	0.31442	8.6475	-7.8216	0.62963	0.56164	0.96617 (max o	8	5301; 3577; 3561; 31	0.23765537	-0.0446621		172.0
	ZINC20610344	-8.3	404.398	-2.0709	10	4	301.06	0.42427	170.88	2.7316	648.3946641435	0.29274	8.2591	-7.0742	0.58621	0.56893	0.9036 (max of	6	6835; 4010; 3586; 10	0.06250401	0.18055743		172.0
	ZINC02111943	-8.3	417.436	-1.2301	9	3	315.8	0.35611	144.86	3.7186	648.3946641435	0.28298	7.4183	-4.3469	0.56667	0.58531	0.96635 (max o	6	6073; 4008; 2738; 27	0.08424415	0.02652774		172.0
	ZINC02154371	-8.3	361.329	-2.606	9	3	267.78	0.41997	144.86	0.1125	648.3946641435	0.32652	8.7942	-7.9812	0.65385	0.5785	0.96519 (max o	4	7457; 6390; 2738; 16	0.1335481	0.10634232		172.0
	ZINC08918469	-8.3	422.440	-3.59	12	6	314.77	0.43333	200.3	-9.9373	648.3946641435	0.28298	9.7782	-12.686	0.7	0.64573	0.96519 (max o	4	6156; 5357; 4950; 41	-0.0520125	-0.4603371		134.0
	ZINC35458114	-8.3	401.457	-1.5171	7	2	324.65	0.28412	129.59	5.3725	648.3946641435	0.29274	7.7053	-5.1824	0.55172	0.54578	0.97878 (max o	3	6883; 4413; 4414	0.8239563	0.18190803		134.0
	ZINC13434878	-8.3	409.187	-9.2546	14	3	258.68	0.67207	237.65	-43.413	648.3946641435	0.32652	15.443	-28.343	0.53846	0.49591	0.9464 (max of	8	3283; 2872; 1792; 21	-0.5562931	0.39955825		67.0
	ZINC08215728	-8.3	409.187	-9.2546	14	3	258.68	0.67207	237.65	-43.413	648.3946641435	0.32652	15.443	-28.343	0.53846	0.49337	0.92702 (max o	7	3283; 2872; 2553; 25	-0.5854097	0.37517703		67.0
	ZINC04521935	-8.3	351.362	-0.6727	10	5	254.95	0.44573	145.78	-0.9947	648.3946641435	0.33958	6.8609	-1.981	0.6	0.37808	0.94678 (max o	7	4942; 4941; 4768; 43	-0.4673198	-0.5984854		67.0
	ZINC08215878	-8.3	425.186	-10.071	15	4	265.24	0.70849	253.33	-44.039	648.3946641435	0.31442	16.259	-32.031	0.51852	0.52156	0.92739 (max o	8	656; 781; 974; 1040;	-0.2964090	-0.5104474		44.0
	ZINC08215878	-8.3	424.178	-11.319	15	3	266.42	0.70978	256.16	-39.309	648.3946641435	0.31442	17.507	-35.999	0.51852	0.52138	0.92739 (max o	8	657; 782; 975; 1041;	-0.3156133	-0.4715324		44.0
	ZINC04887469	-8.3	351.318	-0.8696	11	2	249.96	0.50088	147.13	0.815	648.3946641435	0.33958	7.0578	-2.5608	0.48	0.37337	0.93686 (max o	3	3247; 1732; 1966	0.13296142	-0.6535224		44.0
	ZINC20233084	-8.3	362.190	-9.0309	13	4	228.04	0.69606	211.43	-31.527	648.3946641435	0.35373	15.219	-25.531	0.5	0.43033	0.9115 (max of	5	3475; 3473; 2645; 15	-0.3170896	-0.3116010		44.0
	ZINC33979260	-8.3	362.190	-9.0309	13	4	228.04	0.69606	211.43	-31.527	648.3946641435	0.35373	15.219	-25.531	0.5	0.43249	0.92619 (max o	5	3475; 3473; 2646; 15	-0.3357858	-0.2989685		44.0
	ZINC20233084	-8.3	361.182	-10.279	13	3	229.22	0.69763	214.26	-27.565	648.3946641435	0.35373	16.467	-29.058	0.5	0.45058	0.9115 (max of	6	3476; 3474; 2739; 26	-0.3030731	-0.3469738		44.0
	ZINC33979260	-8.3	361.182	-10.279	13	3	229.22	0.69763	214.26	-27.565	648.3946641435	0.35373	16.467	-29.058	0.5	0.45267	0.92619 (max o	6	4933; 3476; 3474; 15	-0.3191145	-0.3330304		44.0
	ZINC12889620	-8.3	418.429	-3.1207	11	4	312.54	0.42452	173.76	6.4964	648.3946641435	0.28298	9.3089	-11.028	0.5	0.55331	0.96385 (max o	10	5077; 4764; 3801; 29	0.04064595	-0.5798952		42.0
	ZINC20758560	-8.3	418.405	-4.7989	11	3	311.55	0.42269	170.8	7.0522	648.3946641435	0.28298	10.987	-16.958	0.46667	0.56452	0.94241 (max o	9	4764; 4528; 3822; 34	0.01882761	-0.6447549		42.0
	ZINC12889572	-8.3	391.403	-2.8277	10	4	292.62	0.40144	150.9	8.3596	648.3946641435	0.30319	9.0159	-9.3264	0.46429	0.55823	0.97533 (max o	13	5166; 4668; 4528; 37	0.05512574	-0.6462527		42.0
	ZINC12889511	-8.3	375.404	-1.901	9	3	284.77	0.36651	130.67	6.6384	648.3946641435	0.31442	8.0892	-6.046	0.48148	0.54845	0.97407 (max o	13	5165; 4668; 3801; 37	0.03588028	-0.6369181		42.0
	ZINC20757854	-8.3	408.433	-0.2642	8	2	307.05	0.30259	118.64	5.5436	648.3946641435	0.28298	6.4524	-0.93363	0.4	0.5082	0.96092 (max o	8	3859; 3414; 3346; 12	0.6302715	-0.0933348		36.0
	ZINC20757803	-8.3	366.352	-1.2962	8	2	271.29	0.34247	118.64	2.9625	648.3946641435	0.31442	7.4844	-4.1225	0.44444	0.50457	0.95201 (max o	5	4326; 3859; 1261; 13	0.68742627	-0.1068020		36.0
	ZINC12883529	-8.3	410.405	-1.5043	9	3	301.14	0.35203	138.87	4.1266	648.3946641435	0.28298	7.6925	-5.3159	0.4	0.50203	0.9897 (max of	12	4328; 3784; 3414; 33	0.66920966	-0.0259458		36.0
	ZINC08790231	-8.3	422.460	0.0941	8	1	318.15	0.26717	109.85	4.4481	648.3946641435	0.27385	6.0941	0.34362	0.3871	0.50363	0.98065 (max o	12	5034; 4962; 4522; 40	0.7137528	0.01516456		36.0

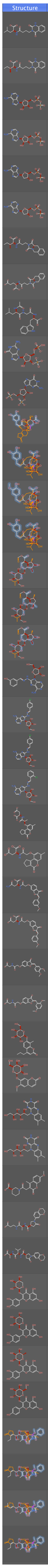

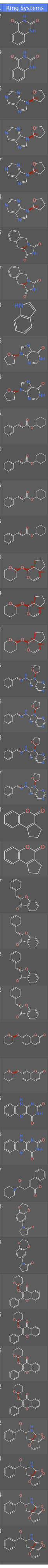

Structure	Molecule Name	affinity	Total Molweight	cLogP	H-Acceptors	H-Donors	Total Surface Area	Relative PSA	Polar Surface Area	Druglikeness	Kd (nM) @ 20°C	LE from K...	LLE from Kd ...	LELP from Kd (n...	Shape Index	Molecular...	Neighbor Si...	Neig...	Neighbor	Neighbor ...	Neighbor ...	Ring Systems	frequency
	ZINC20411992	-8.3	360.389	-0.7022	8	2	276.07	0.33655	118.64	5.1881	648.3946641435	0.32652	6.8904	-2.1506	0.57692	0.57045	0.93686 (max o	3	5123; 4019; 2485	0.6455911	0.12698522		36.0
	ZINC06908002	-8.3	360.389	-0.7022	8	2	276.07	0.33655	118.64	5.5993	648.3946641435	0.32652	6.8904	-2.1506	0.57692	0.57511	0.96721 (max o	3	5123; 4019; 2578	0.6808149	0.13070069		36.0
	ZINC08586019	-8.3	424.178	-11.354	15	3	264.95	0.71006	260.71	-42.337	648.3946641435	0.31442	17.542	-36.112	0.51852	0.47645	0.93373 (max o	6	3284; 3172; 2873; 14	-0.6071572	0.41078156		33.0
	ZINC08586019	-8.3	425.186	-10.107	15	4	263.77	0.70876	257.88	-47.067	648.3946641435	0.31442	16.295	-32.144	0.51852	0.47665	0.93373 (max o	9	3283; 3172; 2872; 25	-0.6061957	0.3739363		33.0
	ZINC13434876	-8.3	409.187	-9.2546	14	3	258.68	0.67207	237.65	-43.413	648.3946641435	0.32652	15.443	-28.343	0.53846	0.48445	0.9464 (max of	7	3283; 2872; 2553; 21	-0.595497	0.35528097		33.0
	ZINC13434873	-8.3	409.187	-9.2546	14	3	258.68	0.67207	237.65	-43.413	648.3946641435	0.32652	15.443	-28.343	0.53846	0.48445	0.94008 (max o	7	3283; 2872; 2553; 25	-0.5750062	0.35810894		33.0
	ZINC12888645	-8.3	386.427	-1.2788	8	1	287.67	0.29548	109.85	7.2507	648.3946641435	0.30319	7.467	-4.2178	0.53571	0.51907	0.97037 (max o	13	4091; 3642; 3209; 32	0.09626225	-0.8628675		29.0
	ZINC04090302	-8.3	401.374	-4.8494	10	1	289.69	0.38772	149.98	5.4336	648.3946641435	0.29274	11.038	-16.566	0.55172	0.52588	0.96473 (max o	7	5464; 4091; 3642; 32	0.14568636	-0.9105287		29.0
	ZINC05643783	-8.3	415.468	-1.797	9	4	325.9	0.35229	143.22	1.202	648.3946641435	0.28298	7.9852	-6.3503	0.53333	0.58692	0.97533 (max o	3	5431; 5430; 3266	-0.5859888	0.56153053		28.0
	ZINC72424489	-8.3	440.177	-12.957	16	4	271.51	0.74472	276.39	-33.279	648.3946641435	0.30319	19.145	-42.735	0.42857	0.42993	0.93385 (max o	4	4454; 1510; 2178; 27	-0.3888180	-0.3092536		26.0
	ZINC72424489	-8.3	439.169	-14.205	16	3	272.69	0.74583	279.22	-23.349	648.3946641435	0.30319	20.393	-46.85	0.42857	0.44654	0.9308 (max of	5	4453; 3175; 2701; 15	-0.3787398	-0.3476308		26.0
	ZINC31154929	-8.3	353.302	-2.8445	9	5	246.47	0.47008	167.58	0.27088	648.3946641435	0.33958	9.0327	-8.3766	0.6	0.41449	0.94658 (max o	15	3467; 3051; 3032; 11	-0.2142335	-0.7993055		25.0
	ZINC04102407	-8.3	353.302	-2.8445	9	5	246.47	0.47008	167.58	0.33063	648.3946641435	0.33958	9.0327	-8.3766	0.56	0.41449	0.98188 (max o	17	5090; 3467; 3158; 30	-0.1823629	-0.8174680		25.0
	ZINC03804531	-8.3	367.329	-2.5688	9	4	262.38	0.42976	156.58	0.46026	648.3946641435	0.32652	8.757	-7.8673	0.53846	0.41317	0.97791 (max o	11	5090; 4797; 3158; 30	-0.1937612	-0.7703555		25.0
	ZINC06482465	-8.3	368.337	-0.3406	9	5	261.2	0.42718	153.75	-2.4507	648.3946641435	0.32652	6.5288	-1.0431	0.57692	0.41967		0		-0.8394037	0.50844795		25.0
	ZINC68590035	-8.3	390.383	-1.9707	10	5	264.25	0.44859	155.14	-3.9213	648.3946641435	0.31442	8.1589	-6.2677	0.48148	0.37688	0.94605 (max o	2	3668; 2396	-0.5571456	-0.5145759		25.0
	ZINC31156303	-8.3	390.383	-1.942	10	5	263.42	0.45	155.14	-4.365	648.3946641435	0.31442	8.1302	-6.1764	0.48148	0.37688	0.98371 (max o	5	5966; 3919; 3708; 26	-0.6318317	0.01941044		25.0
	ZINC03978792	-8.3	390.383	-1.9707	10	5	264.25	0.44859	155.14	-3.9213	648.3946641435	0.31442	8.1589	-6.2677	0.48148	0.37688	0.95201 (max o	7	5872; 5385; 4965; 32	-0.5759852	-0.5272701		25.0
	ZINC08964833	-8.3	401.382	1.4655	12	6	283.5	0.49531	184.16	1.3234	648.3946641435	0.29274	4.7227	5.0062	0.48276	0.33593	0.95642 (max o	13	4627; 4618; 4190; 37	-0.1891463	-0.6815685		24.0
	ZINC08740499	-8.3	419.828	2.4172	11	5	292.57	0.43518	163.93	1.3996	648.3946641435	0.29274	3.771	8.2572	0.51724	0.33593	0.97878 (max o	13	4621; 4620; 4188; 37	-0.2631275	-0.6883036		24.0
	ZINC08740501	-8.3	419.828	2.4172	11	5	292.57	0.43518	163.93	1.3996	648.3946641435	0.29274	3.771	8.2572	0.51724	0.33593	0.97878 (max o	13	4627; 4626; 4621; 46	-0.2066208	-0.7146958		24.0
	ZINC08740534	-8.3	419.828	2.4172	11	5	292.57	0.43518	163.93	1.3996	648.3946641435	0.29274	3.771	8.2572	0.48276	0.31554	0.97649 (max o	11	5835; 5459; 4627; 46	-0.2409264	-0.6922507		24.0
	ZINC04037745	-8.3	401.370	-3.7031	9	1	291.26	0.38515	144.89	1.9281	648.3946641435	0.29274	9.8913	-12.65	0.48276	0.39501	0.97176 (max o	3	3631; 1360; 2401	0.1877024	0.10433663		24.0
	ZINC04037603	-8.3	387.343	-4.0624	9	1	280.26	0.40027	144.89	0.94554	648.3946641435	0.30319	10.251	-13.399	0.5	0.39986	0.9218 (max of	4	4012; 3631; 2402; 10	0.18506202	0.1264484		24.0
	ZINC08791207	-8.3	439.399	-1.5441	10	2	325.02	0.37896	157.08	5.1674	648.3946641435	0.26529	7.7323	-5.8203	0.59375	0.44897	0.97948 (max o	8	4090; 2537; 476; 150	0.58544904	0.41145337		24.0
	ZINC12886330	-8.3	439.375	-3.2223	10	1	324.03	0.37706	154.12	4.3928	648.3946641435	0.26529	9.4105	-12.146	0.59375	0.45726	0.96186 (max o	9	4090; 3343; 476; 150	0.572137	0.3955958		24.0
	ZINC04085444	-8.3	412.373	-0.7537	9	1	308.51	0.33989	123.22	0.53295	648.3946641435	0.28298	6.9419	-2.6634	0.63333	0.40106	0.92835 (max o	9	5076; 4197; 2937; 29	0.5321885	0.34203452		24.0
	ZINC04086245	-8.3	412.373	-0.7537	9	1	308.51	0.33989	123.22	0.53295	648.3946641435	0.28298	6.9419	-2.6634	0.6	0.39496	0.96343 (max o	7	5076; 3307; 2937; 27	0.5668087	0.35810772		24.0
	ZINC04065013	-8.3	380.392	0.4933	8	4	272.42	0.35034	125.68	-7.351	648.3946641435	0.31442	5.6949	1.5689	0.51852	0.32549	0.96389 (max o	7	135; 257; 342; 437; 1	0.33018288	0.5222252		24.0
	ZINC05733292	-8.3	354.310	-0.9076	9	4	244.43	0.43137	134.91	-5.8493	648.3946641435	0.33958	7.0958	-2.6727	0.48	0.2849	0.93686 (max o	5	4579; 4106; 3704; 34	-0.4211691	0.5235712		24.0
	ZINC13507423	-8.3	406.394	-2.6337	11	6	283.25	0.45758	175.28	6.4914	648.3946641435	0.29274	8.8219	-8.9968	0.44828	0.48681	0.96428 (max o	15	4912; 4908; 4896; 41	0.4614662	0.74159765		24.0
	ZINC04353343	-8.3	406.394	-2.6337	11	6	283.25	0.45758	175.28	6.4914	648.3946641435	0.29274	8.8219	-8.9968	0.44828	0.51267	0.97288 (max o	19	5875; 5314; 5297; 49	0.44209725	0.79262346		24.0
	ZINC06624273	-8.3	358.369	-0.6059	7	0	266.08	0.2892	95.97	4.3706	648.3946641435	0.32652	6.7941	-1.8557	0.57692	0.54928	0.96998 (max o	8	2850; 363; 605; 866;	0.36319882	0.16668707		22.0
	ZINC04085405	-8.3	404.398	-2.5387	10	2	297.83	0.37911	137.1	-0.72047	648.3946641435	0.29274	8.7269	-8.6722	0.62069	0.48116	0.96931 (max o	9	5369; 4087; 3342; 77	0.5460645	0.24257073		22.0
	ZINC38570073	-8.3	432.451	-1.9611	10	2	319.83	0.35303	137.1	-1.2377	648.3946641435	0.27385	8.1493	-7.1612	0.54839	0.46031	0.96971 (max o	13	6226; 5250; 4194; 39	0.49585086	0.22416568		22.0
	ZINC06569127	-8.3	434.352	0.2551	11	7	284.9	0.47294	186.37	-3.8653	648.3946641435	0.27385	5.9331	0.93153	0.41935	0.41786	0.96262 (max o	16	4095; 2776; 2566; 25	-0.7179273	0.3073729		21.0
	ZINC14684624	-8.3	434.352	0.2551	11	7	284.9	0.47294	186.37	-3.8653	648.3946641435	0.27385	5.9331	0.93153	0.41935	0.41786	0.96262 (max o	15	4095; 2776; 2566; 17	-0.7090248	0.28912255		21.0
	ZINC04349617	-8.3	448.379	0.5798	11	7	295.9	0.45536	186.37	1.9289	648.3946641435	0.26529	5.6084	2.1855	0.40625	0.42191	0.96877 (max o	14	4095; 2776; 2566; 25	-0.7479547	0.28729466		21.0
	ZINC04654800	-8.3	448.379	-0.0011999	11	7	297.4	0.45306	186.37	-3.6679	648.3946641435	0.26529	6.1894	-0.0045229	0.40625	0.43252	0.95949 (max o	10	4260; 3174; 2768; 11	-0.699146	0.36170152		21.0
	ZINC14682247	-8.3	431.440	-0.1614	9	4	308.3	0.35641	142.39	0.6376	648.3946641435	0.27385	6.3496	-0.58937	0.51613	0.42756	0.97199 (max o	9	3362; 2967; 2651; 15	-0.3121555	-0.7372482		19.0
	ZINC14682245	-8.3	431.440	-0.1614	9	4	308.3	0.35641	142.39	0.6376	648.3946641435	0.27385	6.3496	-0.58937	0.51613	0.42806	0.97199 (max o	9	3481; 3362; 2967; 15	-0.3008616	-0.7551844		19.0
	ZINC05464465	-8.3	431.440	-0.1614	9	4	308.3	0.35641	142.39	0.6376	648.3946641435	0.27385	6.3496	-0.58937	0.51613	0.44854	0.97199 (max o	8	4577; 4392; 4134; 34	-0.3331014	-0.7703404		19.0
	ZINC13660219	-8.3	431.440	-0.1614	9	4	308.3	0.35641	142.39	0.6376	648.3946641435	0.27385	6.3496	-0.58937	0.51613	0.4269	0.97199 (max o	11	4577; 4392; 4134; 38	-0.3449589	-0.7196253		19.0

Structure	Molecule Name	affinity	Total Molweight	cLogP	H-Acceptors	H-Donors	Total Surface Area	Relative PSA	Polar Surface Area	Druglikeness	Kd (nM) @ 20°C	LE from K...	LLE from Kd ...	LELP from Kd (n...	Shape Index	Molecular...	Neighbor Si...	Neig...	Neighbor	Neighbor ...	Neighbor ...	Ring Systems	frequency
	ZINC35270644	-8.3	431.440	-0.1614	9	4	308.3	0.35641	142.39	0.6376	648.3946641435	0.27385	6.3496	-0.58937	0.51613	0.42756	0.97199 (max o	9	4577; 4392; 4134; 34	-0.3664984	-0.7176471		19.0
	ZINC08636373	-8.3	422.504	-0.4389	9	5	307.6	0.40852	157.58	6.5739	648.3946641435	0.29274	6.6271	-1.4993	0.62069	0.46314	0.9398	1	1468	-0.4963854	0.446509		17.0
	ZINC12604067	-8.3	379.867	0.7743	6	4	269.34	0.33846	119.25	4.9292	648.3946641435	0.33958	5.4139	2.2802	0.6	0.38345	0.97396 (max o	9	5483; 4229; 3716; 33	-0.4732491	0.36374906		17.0
	ZINC03871880	-8.3	376.388	-3.4633	8	1	256.5	0.4414	154.97	10.967	648.3946641435	0.32652	9.6515	-10.607	0.5	0.35316	0.96721 (max o	4	4939; 4498; 3617; 16	0.09477357	-0.0862023		17.0
	ZINC04038341	-8.3	425.996	-1.1969	7	5	318.59	0.34176	128.76	0.91283	648.3946641435	0.31442	7.3851	-3.8067	0.51852	0.47313	0.99202 (max o	10	7679; 7464; 7460; 72	0.741394	-0.2587905		15.0
	ZINC04349428	-8.3	432.380	-0.078	10	7	289.19	0.43134	177.14	-1.9977	648.3946641435	0.27385	6.2662	-0.28483	0.51613	0.3811	0.96018 (max o	11	3788; 3213; 294; 832	0.73574775	0.10017851		14.0
	ZINC08551389	-8.3	448.379	-0.4237	11	8	295.54	0.4664	197.37	-1.9977	648.3946641435	0.26529	6.6119	-1.5971	0.5	0.38342	0.97743 (max o	12	3282; 3243; 2828; 28	0.70811737	0.03540132		14.0
	ZINC04349261	-8.3	448.379	-0.4237	11	8	295.54	0.4664	197.37	-1.9977	648.3946641435	0.26529	6.6119	-1.5971	0.5	0.38295	0.96186 (max o	11	3282; 3243; 2828; 28	0.7327891	0.06141255		14.0
	ZINC12888804	-8.3	449.442	-1.4649	10	4	325.31	0.36888	154.66	4.2777	648.3946641435	0.25725	7.6531	-5.6943	0.39394	0.46337	0.99099 (max o	10	290; 302; 397; 1260;	0.70741576	-0.1784014		14.0
	ZINC12878646	-8.3	449.442	-1.4649	10	4	325.31	0.36888	154.66	4.2777	648.3946641435	0.25725	7.6531	-5.6943	0.39394	0.4633	0.99099 (max o	11	2540; 282; 290; 1257	0.7029894	-0.1426414		14.0
	ZINC05434138	-8.3	427.460	-0.4791	10	2	326.07	0.32429	131.84	0.70151	648.3946641435	0.27385	6.6673	-1.7495	0.51613	0.53191	0.94241 (max o	6	4181; 4132; 1168; 11	0.12149084	0.29626623		14.0
	ZINC05434136	-8.3	432.475	-0.3847	10	2	326.62	0.31284	117.28	4.9815	648.3946641435	0.27385	6.5729	-1.4048	0.51613	0.54173	0.94241 (max o	6	5806; 4181; 4132; 24	0.12708144	0.2761778		14.0
	ZINC05397262	-8.3	390.418	-0.6588	7	0	292.83	0.25291	104.54	-2.2557	648.3946641435	0.29274	6.847	-2.2505	0.51724	0.46821	0.9561 (max of	6	5778; 5393; 4990; 45	-0.4870426	-0.3355254		14.0
	ZINC08643600	-8.3	361.397	-0.4845	8	5	279.69	0.3456	119.92	1.4809	648.3946641435	0.32652	6.6727	-1.4838	0.69231	0.56953	0.93367 (max o	4	3739; 3295; 2927; 61	0.28516835	-0.4334025		14.0
	ZINC12483096	-8.3	446.444	-0.3731	8	2	301.98	0.23505	88.08	-1.7846	648.3946641435	0.27385	6.5613	-1.3624	0.58065	0.53336	0.94241 (max o	6	6503; 6500; 5851; 55	0.41879272	-0.2254680		14.0
	ZINC13521023	-8.3	436.412	0.055	10	7	305.5	0.40831	177.14	-4.8731	648.3946641435	0.27385	6.1332	0.20084	0.51613	0.43255	0.96018 (max o	10	5085; 3799; 3620; 32	0.82511353	-0.3079556		13.0
	ZINC01703076	-8.3	376.388	-3.4633	8	1	256.5	0.4414	154.97	10.967	648.3946641435	0.32652	9.6515	-10.607	0.5	0.34338	0.96721 (max o	5	4982; 4635; 2771; 70	0.03955465	-0.0740766		12.0
	ZINC01562698	-8.3	365.409	-1.8794	8	3	245.3	0.46262	162.71	9.2567	648.3946641435	0.33958	8.0676	-5.5345	0.56	0.38598	0.91611 (max o	3	4354; 4025; 2779	0.11011011	-0.1781781		12.0
	ZINC04221827	-8.3	433.440	-3.2221	11	6	322.14	0.4417	193.91	4.3314	648.3946641435	0.27385	9.4103	-11.766	0.58065	0.54229	0.94768 (max o	5	4097; 3602; 1705; 17	-0.1775094	-0.5332252		12.0
	ZINC72320617	-8.3	350.322	-0.2569	8	3	236.42	0.41629	130.36	-2.1313	648.3946641435	0.33958	6.4451	-0.75653	0.48	0.40379	0.97118 (max o	9	6058; 6057; 5279; 48	0.7494135	-0.1121636		12.0
	ZINC31169933	-8.3	448.507	0.4131	10	6	332.1	0.35712	158.3	-6.0909	648.3946641435	0.27385	5.7751	1.5085	0.6129	0.57895	0.90289 (max o	6	5681; 3916; 3457; 41	-0.3758503	0.38404542		11.0
	ZINC67912189	-8.3	438.468	-1.8092	11	6	308.91	0.42614	175.37	0.52337	648.3946641435	0.28298	7.9974	-6.3934	0.53333	0.56079	0.96589	1	4433	0.9426598	0.20599376		11.0
	ZINC04097528	-8.3	409.386	-3.6382	12	7	280.11	0.51858	202.32	-13.537	648.3946641435	0.30319	9.8264	-12	0.5	0.55798		0		-0.9158668	0.14391391		11.0
	ZINC04237066	-8.3	395.434	-0.5644	7	0	293.38	0.2403	89.98	4.1501	648.3946641435	0.29274	6.7526	-1.928	0.62069	0.47064	0.9561 (max of	7	5778; 3681; 724; 114	-0.5289990	-0.3660284		10.0
	ZINC03838744	-8.3	381.384	-2.2646	10	6	274.95	0.46048	171.21	2.8906	648.3946641435	0.31442	8.4528	-7.2024	0.59259	0.5001	0.93046 (max o	3	1104; 1663; 1664	-0.3756486	-0.4876036		10.0
	ZINC31156576	-8.3	448.379	-0.4237	11	8	295.54	0.4664	197.37	-1.9977	648.3946641435	0.26529	6.6119	-1.5971	0.4375	0.38295	0.96186 (max o	6	3891; 925; 1163; 143	0.77562696	0.00792654		10.0
	ZINC13424746	-8.3	432.380	-0.078	10	7	289.19	0.43134	177.14	-1.9977	648.3946641435	0.27385	6.2662	-0.28483	0.45161	0.3816	0.95771 (max o	9	4107; 3226; 2806; 29	0.75265706	0.08563921		10.0
	ZINC04098560	-8.3	448.379	-0.4237	11	8	295.54	0.4664	197.37	-1.9977	648.3946641435	0.26529	6.6119	-1.5971	0.4375	0.38295	0.96186 (max o	8	3891; 3243; 2806; 26	0.7807021	0.05266207		10.0
	ZINC13424747	-8.3	432.380	-0.078	10	7	289.19	0.43134	177.14	-1.9977	648.3946641435	0.27385	6.2662	-0.28483	0.45161	0.3811	0.95771 (max o	7	4107; 3226; 2806; 12	0.75352734	0.10672508		10.0
	ZINC20463706	-8.3	420.464	-0.2411	10	3	303.95	0.32637	112.6	5.9813	648.3946641435	0.28298	6.4293	-0.852	0.56667	0.47978	0.96092 (max o	6	4214; 3872; 3838; 33	-0.6227014	-0.2250506		10.0
	ZINC04084685	-8.3	376.412	-2.0596	8	2	258.75	0.44139	157.93	8.1327	648.3946641435	0.32652	8.2478	-6.3078	0.46154	0.3001	0.92315 (max o	8	5356; 4524; 4245; 40	0.33799434	-0.6922298		10.0
	ZINC04259900	-8.3	372.360	-2.3185	10	1	271.03	0.41176	131.29	1.0253	648.3946641435	0.31442	8.5067	-7.3738	0.59259	0.34856	0.92464 (max o	5	576; 908; 1415; 1738	-0.2628598	-0.3101922		10.0
	ZINC04147899	-8.3	415.429	-2.4221	11	1	305.85	0.37649	134.53	1.7728	648.3946641435	0.28298	8.6103	-8.5592	0.6	0.38544	0.91522 (max o	6	5002; 4493; 3218; 24	-0.2465252	-0.3433581		10.0
	ZINC35442315	-8.3	391.509	0.0392	6	2	300.01	0.17459	69.26	4.7596	648.3946641435	0.30319	6.149	0.12929	0.60714	0.50236	0.9399 (max of	10	3876; 3438; 3065; 30	-0.5241248	0.10453347		10.0
	ZINC08299700	-8.3	391.494	-3.1808	9	3	305.85	0.34134	118.78	0.79107	648.3946641435	0.30319	9.369	-10.491	0.64286	0.5263	0.98451 (max o	11	4599; 4178; 4164; 41	0.19900285	0.29814866		10.0
	ZINC31169357	-8.3	413.397	-2.9205	10	4	298.4	0.41139	165.81	1.5959	648.3946641435	0.29274	9.1087	-9.9765	0.58621	0.59812	0.99848 (max o	7	6878; 6641; 6324; 43	-0.1698706	0.7088966		10.0
	ZINC06041520	-8.3	432.380	-0.7865	10	7	289.19	0.43134	177.14	-2.3989	648.3946641435	0.27385	6.9747	-2.872	0.48387	0.39235	0.95267 (max o	8	5883; 161; 462; 758;	0.67841864	0.17592283		9.0
	ZINC05433656	-8.3	427.436	-1.9082	10	2	316.79	0.36371	134.73	1.1989	648.3946641435	0.27385	8.0964	-6.968	0.64516	0.39998	0.98207 (max o	7	3255; 2842; 1181; 20	-0.6691619	-0.4890086		9.0
	ZINC08299325	-8.3	388.489	-3.3577	7	3	306.46	0.25661	80.86	-0.98284	648.3946641435	0.30319	9.5459	-11.074	0.67857	0.50714	0.97037 (max o	8	3714; 2860; 2857; 28	-0.1727511	0.01321037		9.0
	ZINC08298678	-8.3	402.516	-3.2328	7	3	335.32	0.2639	68.69	-1.069	648.3946641435	0.29274	9.421	-11.043	0.65517	0.51789	0.97176 (max o	8	3713; 3277; 2857; 28	-0.1678933	-0.0090517		9.0
	ZINC08298261	-8.3	414.552	-3.4036	8	3	351.23	0.28041	77.92	0.29222	648.3946641435	0.28298	9.5918	-12.028	0.6	0.51401	0.97304 (max o	8	4157; 4155; 3713; 33	-0.1543507	0.02401589		9.0
	ZINC19795953	-8.3	362.385	-4.4497	8	3	247.6	0.46151	161.09	7.0306	648.3946641435	0.33958	10.638	-13.104	0.56	0.44329	0.9654 (max of	7	4817; 4280; 3383; 26	-0.4899887	-0.0534704		9.0
	ZINC03830391	-8.3	363.393	-2.2502	8	3	246.81	0.45979	162.71	7.158	648.3946641435	0.33958	8.4384	-6.6265	0.56	0.41871	0.88969 (max o	5	4280; 3589; 3518; 25	-0.5253886	-0.0401596		9.0

Structure	Molecule Name	affinity	Total Molweight	cLogP	H-Acceptors	H-Donors	Total Surface Area	Relative PSA	Polar Surface Area	Druglikeness	Kd (nM) @ 20°C	LE from K...	LLE from Kd ...	LELP from Kd (n...	Shape Index	Molecular...	Neighbor Si...	Neig...	Neighbor	Neighbor ...	Neighbor ...	Ring Systems	frequency
	ZINC35024381	-8.3	366.804	-4.1249	7	2	244.41	0.41394	140.86	7.1659	648.3946641435	0.35373	10.313	-11.661	0.54167	0.43407	0.94718 (max o	5	4390; 3920; 3589; 34	-0.5196036	-0.0204675		9.0
	ZINC05733290	-8.3	354.310	-0.9076	9	4	244.43	0.43137	134.91	-5.8493	648.3946641435	0.33958	7.0958	-2.6727	0.48	0.28946	0.93686 (max o	4	4579; 3704; 3448; 24	-0.4427068	0.5515414		8.0
	ZINC12655829	-8.3	410.453	-3.8871	12	6	300.93	0.46376	162.9	2.9357	648.3946641435	0.29274	10.075	-13.278	0.44828	0.43201	0.86904 (max o	3	5832; 5485; 4001	-0.5044373	-0.5779038		8.0
	ZINC31156311	-8.3	390.383	-1.942	10	5	263.42	0.45	155.14	-4.365	648.3946641435	0.31442	8.1302	-6.1764	0.48148	0.37688	0.98371 (max o	4	6290; 3919; 3708; 26	-0.6512803	0.02755117		8.0
	ZINC05273633	-8.3	416.381	-0.4408	9	6	282.84	0.39471	156.91	-2.3989	648.3946641435	0.28298	6.629	-1.5577	0.5	0.39702	0.95579 (max o	8	541; 588; 714; 920; 9	0.6975119	0.16475466		7.0
	ZINC08789898	-8.3	425.464	-0.7376	9	2	308.25	0.32114	125.64	4.7791	648.3946641435	0.27385	6.9258	-2.6934	0.45161	0.40779	0.93686 (max o	4	4078; 2784; 2785; 19	-0.4135992	0.32048792		7.0
	ZINC31164867	-8.3	440.484	-1.7456	11	7	311.11	0.42332	178.53	-1.2172	648.3946641435	0.28298	7.9338	-6.1686	0.53333	0.56681	0.98786 (max o	6	5695; 5287; 3910; 34	-0.6365046	0.47720551		7.0
	ZINC31164863	-8.3	440.484	-1.7456	11	7	311.11	0.42332	178.53	0.71929	648.3946641435	0.28298	7.9338	-6.1686	0.53333	0.53671	0.9897 (max of	6	5695; 5287; 3910; 34	-0.6572914	0.4700277		7.0
	ZINC01632794	-8.3	384.339	-2.3176	8	0	288.6	0.349	132.86	0.2574	648.3946641435	0.30319	8.5058	-7.644	0.57143	0.42546	0.95148 (max o	3	3991; 1352; 1964	-0.8639347	-0.2735370		7.0
	ZINC09033636	-8.3	401.414	-2.5193	11	3	282.77	0.43675	133.45	2.3161	648.3946641435	0.30319	8.7075	-8.3092	0.53571	0.49242		0		0.1775572	-0.9330368		7.0
	ZINC03839678	-8.3	409.437	-1.9072	10	6	299.71	0.42244	171.21	3.9307	648.3946641435	0.29274	8.0954	-6.515	0.55172	0.51891		0		0.7298543	0.56145203		7.0
	ZINC31157947	-8.3	427.405	-2.4776	11	6	298.59	0.44261	182.09	-10.383	648.3946641435	0.28298	8.6658	-8.7554	0.53333	0.48187	0.96589 (max o	4	3895; 3037; 842; 263	-0.4815700	0.74513227		6.0
	ZINC04097731	-8.3	449.387	-0.3513	11	8	288.97	0.43188	180.3	-8.6993	648.3946641435	0.26529	6.5395	-1.3242	0.40625	0.26804	0.95949 (max o	8	3350; 3349; 2963; 27	-0.8085992	0.2810792		6.0
	ZINC12901943	-8.3	358.373	-1.6534	8	1	266.33	0.33763	111.13	5.2828	648.3946641435	0.32652	7.8416	-5.0638	0.53846	0.45544	0.89688 (max o	5	5494; 778; 964; 966;	0.17259045	0.52470696		6.0
	ZINC31159557	-8.3	428.432	-0.8829	10	6	299.92	0.39544	158.3	-1.6264	648.3946641435	0.28298	7.0711	-3.12	0.56667	0.47557	0.95031 (max o	3	820; 1288; 2045	-0.4365094	0.37453413		6.0
	ZINC02036915	-8.3	438.403	-5.6299	13	4	324.71	0.50821	224.99	2.7179	648.3946641435	0.26529	11.818	-21.221	0.625	0.58116	0.98148 (max o	5	1077; 1458; 1656; 19	-0.5501106	0.6871344		6.0
	ZINC04082114	-8.3	374.368	-0.9625	8	0	274.58	0.31667	105.2	5.7109	648.3946641435	0.31442	7.1507	-3.0612	0.51852	0.48513	0.93686 (max o	6	548; 1687; 1689; 177	0.30070692	0.19607277		6.0
	ZINC20465161	-8.3	364.444	-0.6155	8	4	275.05	0.28817	97.3	7.6247	648.3946641435	0.32652	6.8037	-1.8851	0.57692	0.5395	0.93367 (max o	4	2594; 1866; 1868; 22	-0.3782782	-0.0018538		6.0
	ZINC20465168	-8.3	448.440	0.4835	9	4	314.51	0.28381	106.53	-0.37533	648.3946641435	0.27385	5.7047	1.7656	0.58065	0.5959	0.93409 (max o	4	3393; 1868; 2592; 25	-0.3991089	-0.0059662		6.0
	ZINC20465168	-8.3	449.448	-1.6262	9	5	328.55	0.31441	107.73	-4.3868	648.3946641435	0.27385	7.8144	-5.9382	0.58065	0.5959	0.90825 (max o	4	3393; 1869; 2593; 25	-0.3663546	-0.0164339		6.0
	ZINC20465161	-8.3	365.452	-2.7252	8	5	289.09	0.32274	98.5	3.6132	648.3946641435	0.32652	8.9134	-8.3463	0.57692	0.5395	0.93367 (max o	6	6819; 5929; 1251; 18	-0.3237396	-0.0098889		6.0
	ZINC08624381	-8.3	410.440	0.1347	8	3	299.89	0.29107	108.33	1.7254	648.3946641435	0.29274	6.0535	0.46014	0.58621	0.53026	0.9398 (max of	5	2107; 2114; 2118; 21	0.27806452	-0.350987		6.0
	ZINC08624378	-8.3	392.450	0.0339	8	3	293.54	0.29737	108.33	3.0654	648.3946641435	0.30319	6.1543	0.11181	0.60714	0.52726	0.94558 (max o	4	3287; 2106; 2155; 24	0.23487525	-0.3558418		6.0
	ZINC08643416	-8.3	362.424	0.5519	7	3	271.04	0.28516	99.1	3.0773	648.3946641435	0.32652	5.6363	1.6903	0.61538	0.50913	0.92368 (max o	5	4168; 2111; 2114; 21	0.3112171	-0.3301856		6.0
	ZINC20465128	-8.3	418.434	-1.8708	7	4	307.09	0.2665	86.47	-3.6156	648.3946641435	0.29274	8.059	-6.3907	0.55172	0.55806	0.91599 (max o	8	6819; 5935; 5933; 55	-0.3574396	0.04159518		6.0
	ZINC04089257	-8.3	357.369	0.0027999	9	4	257.63	0.39025	125.55	-0.31782	648.3946641435	0.32652	6.1854	0.008575	0.57692	0.36096	0.94951 (max o	10	3229; 2807; 861; 114	-0.4310695	-0.7441486		5.0
	ZINC04236422	-8.3	378.791	-1.3241	8	1	267.33	0.31796	109.85	0.80084	648.3946641435	0.32652	7.5123	-4.0552	0.57692	0.45984	0.93686 (max o	3	3656; 719; 2370	-0.1896263	0.35792217		5.0
	ZINC03838543	-8.3	379.799	0.7254	8	1	268.3	0.30086	96.02	-2.6746	648.3946641435	0.32652	5.4628	2.2216	0.57692	0.45717	0.91007	1	2432	-0.2117673	0.32613686		5.0
	ZINC35442828	-8.3	372.356	-2.8873	9	2	273.05	0.37689	127.87	6.7754	648.3946641435	0.31442	9.0755	-9.1829	0.59259	0.35124	0.81211	1	1186	-0.0885352	-0.7412914		5.0
	ZINC12489988	-8.3	417.459	-0.6704	8	4	318.53	0.30647	97.15	-0.76593	648.3946641435	0.28298	6.8586	-2.3691	0.6	0.49109	0.94529 (max o	6	6996; 6218; 3763; 33	0.32527924	0.06775188		5.0
	ZINC03607120	-8.3	376.407	-2.6266	7	2	252.11	0.22415	92.45	1.2327	648.3946641435	0.31442	8.8148	-8.3537	0.44444	0.36084	0.96885 (max o	4	4423; 3998; 3246; 23	-0.1326032	-0.9378875		5.0
	ZINC15657718	-8.3	434.352	0.2551	11	7	284.9	0.47294	186.37	-3.8653	648.3946641435	0.27385	5.9331	0.93153	0.41935	0.41305	0.96262 (max o	15	4095; 2776; 175; 583	-0.6892733	0.2921885		5.0
	ZINC20465577	-8.3	446.541	-0.3905	7	3	331.99	0.18386	82.01	2.6493	648.3946641435	0.26529	6.5787	-1.472	0.5625	0.45453	0.92602 (max o	5	4312; 2598; 2228; 22	-0.4870158	0.26816863		5.0
	ZINC20464908	-8.3	432.970	0.1847	6	3	318.8	0.1601	72.78	3.9702	648.3946641435	0.28298	6.0035	0.6527	0.56667	0.44758	0.96589 (max o	6	6263; 4299; 3394; 18	-0.4347019	0.24565777		5.0
	ZINC62227230	-8.3	393.371	-3.2184	10	5	279.75	0.44722	164.17	4.1929	648.3946641435	0.30319	9.4066	-10.615	0.53571	0.44009	0.95148 (max o	4	3534; 3097; 2312; 26	0.54493177	-0.4308572		5.0
	ZINC62227206	-8.3	352.318	-3.2288	9	5	244.63	0.46482	155.3	-0.044643	648.3946641435	0.33958	9.417	-9.5083	0.52	0.40478	0.94351 (max o	4	3956; 3955; 3096; 26	0.5695023	-0.4006275		5.0
	ZINC01646901	-8.3	419.417	-1.766	12	3	298.49	0.38655	161.33	-1.1402	648.3946641435	0.28298	7.9542	-6.2407	0.56667	0.53365	0.96092 (max o	3	3557; 3130; 2715	0.55873376	-0.6719253		5.0
	ZINC02033818	-8.3	351.405	-0.5014	6	3	271.93	0.29526	112.66	2.9212	648.3946641435	0.32652	6.6896	-1.5356	0.57692	0.46855	0.96438 (max o	5	6693; 3999; 3804; 32	0.27802542	0.7791744		5.0
	ZINC04260488	-8.3	420.416	-1.7279	9	0	302.86	0.28756	110.29	1.2835	648.3946641435	0.28298	7.9161	-6.1061	0.5	0.50651	0.9707 (max of	4	6142; 4551; 3663; 24	-0.3366268	0.8318125		5.0
	ZINC04260487	-8.3	418.444	-0.2722	8	0	306.62	0.25142	101.06	-2.6436	648.3946641435	0.28298	6.4604	-0.9619	0.5	0.51407	0.98037 (max o	4	6142; 4551; 3663; 24	-0.3529675	0.8425258		5.0
	ZINC35270644	-8.3	430.432	-2.331	9	3	309.59	0.35909	148.71	-0.33782	648.3946641435	0.27385	8.5192	-8.5119	0.51613	0.42756	0.96502 (max o	4	4393; 3482; 3363; 25	-0.3436738	-0.7867465		5.0
	ZINC13660219	-8.3	430.432	-2.331	9	3	309.59	0.35909	148.71	-0.33782	648.3946641435	0.27385	8.5192	-8.5119	0.51613	0.4269	0.96502 (max o	4	4393; 3482; 3363; 26	-0.3331399	-0.8040951		5.0
	ZINC20463366	-8.3	374.483	-1.919	7	1	282.84	0.18035	81.06	8.2861	648.3946641435	0.31442	8.1072	-6.1032	0.55556	0.5766	0.93985 (max o	9	4723; 3387; 3386; 29	-0.1823443	-0.2122982		5.0

Structure	Molecule Name	affinity	Total Molweight	cLogP	H-Acceptors	H-Donors	Total Surface Area	Relative PSA	Polar Surface Area	Druglikeness	Kd (nM) @ 20°C	LE from K...	LLE from Kd ...	LELP from Kd (n...	Shape Index	Molecular...	Neighbor Si...	Neig...	Neighbor	Neighbor ...	Neighbor ...	Ring Systems	frequency
	ZINC20463373	−8.3	430.591	−0.2261	7	1	331.53	0.15386	81.06	5.1382	648.3946641435	0.27385	6.4143	−0.82563	0.54839	0.59489	0.94501 (max o	10	5912; 4723; 3387; 33	−0.1722120	−0.1937903		5.0
	ZINC20465128	−8.3	417.426	0.2389	7	3	293.05	0.23136	85.27	0.44743	648.3946641435	0.29274	5.9493	0.81609	0.55172	0.55806	0.9279 (max of	8	5934; 5932; 5561; 47	−0.3807263	0.0662263		5.0
	ZINC20465903	−8.3	431.511	−1.2289	9	5	345.55	0.32042	117.38	1.9025	648.3946641435	0.27385	7.4171	−4.4875	0.58065	0.56792	0.94768 (max o	4	3855; 3008; 806; 223	0.27563444	−0.2765582		4.0
	ZINC36369868	−8.3	370.312	−2.4278	8	0	273.98	0.37152	128.93	0.81006	648.3946641435	0.31442	8.616	−7.7215	0.62963	0.47945	0.95818 (max o	3	3516; 844; 1321	−0.4181491	−0.868516		4.0
	ZINC04237052	−8.3	422.460	−0.7971	8	1	314.09	0.27062	109.85	5.1827	648.3946641435	0.27385	6.9853	−2.9107	0.6129	0.47719	0.98207 (max o	5	83; 265; 724; 903; 14	−0.5025474	−0.4411211		4.0
	ZINC01081235	−8.3	357.369	0.0027999	9	4	257.63	0.39025	125.55	−0.31782	648.3946641435	0.32652	6.1854	0.008575	0.57692	0.34851	0.92702 (max o	8	3229; 2807; 2417; 86	−0.4198652	−0.7628161		4.0
	ZINC12603790	−8.3	397.402	−0.5193	9	4	286.59	0.34457	120.36	1.3008	648.3946641435	0.30319	6.7075	−1.7128	0.60714	0.56025	0.9399 (max of	8	5024; 1209; 1211; 17	0.30034417	−0.3834058		4.0
	ZINC08624407	−8.3	379.411	−0.6201	9	4	280.24	0.35238	120.36	2.6408	648.3946641435	0.31442	6.8083	−1.9722	0.62963	0.55611	0.94291 (max o	6	5016; 3288; 2875; 25	0.2986254	−0.4153226		4.0
	ZINC04270015	−8.3	405.331	−6.081	12	6	260.53	0.57176	206.27	−2.1079	648.3946641435	0.30319	12.269	−20.057	0.42857	0.38316	0.93985 (max o	3	5396; 3236; 2443	−0.1318969	−0.8674019		4.0
	ZINC04270013	−8.3	405.331	−6.081	12	6	260.53	0.57176	206.27	−2.1079	648.3946641435	0.30319	12.269	−20.057	0.42857	0.38316	0.93985 (max o	3	5396; 3236; 2444	−0.1027345	−0.8433862		4.0
	ZINC79191471	−8.3	440.467	−2.7105	10	6	318.68	0.39579	150.35	−0.96405	648.3946641435	0.27385	8.8987	−9.8977	0.45161	0.44482	0.94232 (max o	5	4885; 1346; 1628; 16	0.7227425	−0.3528250		4.0
	ZINC05119400	−8.3	418.397	0.2774	9	6	298.13	0.37447	156.91	−3.3049	648.3946641435	0.28298	5.9108	0.98028	0.6	0.44618	0.95579 (max o	4	3468; 2830; 926; 173	0.44014543	0.4186657		4.0
	ZINC31166504	−8.3	430.364	−3.5435	10	3	315.81	0.40208	176.48	0.29536	648.3946641435	0.27385	9.7317	−12.939	0.54839	0.4884	0.93418 (max o	3	1824; 1825; 1894	0.05535607	−0.8358801		4.0
	ZINC71404396	−8.3	408.382	−1.7427	10	5	272.62	0.39069	145.12	−4.341	648.3946641435	0.29274	7.9309	−5.9531	0.44828	0.24826	0.94823 (max o	5	3369; 2970; 2025; 20	−0.6232277	−0.7046788		4.0
	ZINC15675304	−8.3	407.465	−0.7176	9	3	303.6	0.29921	111.57	3.0574	648.3946641435	0.29274	6.9058	−2.4513	0.62069	0.56376	0.92464 (max o	6	6476; 3730; 3335; 32	0.33357644	−0.3306559		4.0
	ZINC31156380	−8.3	424.401	−0.4974	10	5	308.78	0.39374	162.98	−10.495	648.3946641435	0.28298	6.6856	−1.7577	0.6	0.48449	0.95837 (max o	3	1914; 1915; 2256	0.57346714	−0.5188216		4.0
	ZINC35455213	−8.3	418.397	0.8232	9	6	298.13	0.37447	156.91	−4.0044	648.3946641435	0.28298	5.365	2.909	0.5	0.43103	0.95318 (max o	7	3894; 3450; 3070; 22	−0.79695	0.13857047		4.0
	ZINC02112110	−8.3	371.368	−1.1309	8	3	286.85	0.34659	127.79	−9.3549	648.3946641435	0.31442	7.3191	−3.5967	0.66667	0.56137	0.96617 (max o	3	4231; 2363; 2176	−0.0294003	−0.4088961		4.0
	ZINC02516112	−8.3	371.368	−1.1309	8	3	286.85	0.34659	127.79	−9.3549	648.3946641435	0.31442	7.3191	−3.5967	0.66667	0.56296	0.96682 (max o	5	5098; 4984; 4112; 21	0.016065	−0.4119156		4.0
	ZINC04023819	−8.3	386.534	0.4278	5	3	304.67	0.15945	71.91	−1.7559	648.3946641435	0.30319	5.7604	1.411	0.67857	0.45323	0.99414 (max o	3	3623; 3624; 3157	0.7622776	−0.4807827		4.0
	ZINC04044720	−8.3	368.364	−0.7314	7	0	252.03	0.29326	88.13	−7.9854	648.3946641435	0.31442	6.9196	−2.3262	0.44444	0.39185	0.93058 (max o	3	4073; 4072; 4071	−0.2490558	−0.9136129		4.0
	ZINC20462840	−8.3	419.471	−2.7576	7	1	330.66	0.26861	105.01	3.56	648.3946641435	0.28298	8.9458	−9.7448	0.56667	0.59941	0.94529 (max o	5	4711; 4712; 4293; 42	0.03996399	−0.2086209		4.0
	ZINC20760360	−8.3	400.434	−1.0932	9	3	282.97	0.36718	130.83	4.7611	648.3946641435	0.29274	7.2814	−3.7344	0.41379	0.26844	0.95372 (max o	3	4335; 4333; 3860	−0.7875164	0.51676834		4.0
	ZINC31158565	−8.3	420.413	0.7702	9	6	302.64	0.35884	149.07	−4.1548	648.3946641435	0.28298	5.418	2.7217	0.53333	0.34454	0.95872 (max o	5	3899; 3901; 1132; 16	−0.5781745	0.7424274		4.0
	ZINC35457926	−8.3	400.810	1.2997	8	5	282.47	0.35961	144.52	0.48888	648.3946641435	0.31442	4.8885	4.1336	0.40741	0.33851	0.97149 (max o	3	3931; 3503; 3071	−0.8553499	0.40508705		4.0
	ZINC38571024	−8.3	387.439	−0.3965	9	3	280.91	0.30878	120.64	5.956	648.3946641435	0.30319	6.5847	−1.3077	0.5	0.4755	0.96253 (max o	3	3944; 3522; 3081	−0.7024525	0.19576876		4.0
	ZINC67902760	−8.3	404.367	−2.6504	11	5	267.41	0.49205	172.21	−5.6455	648.3946641435	0.30319	8.8386	−8.7416	0.46429	0.36295	0.94573	1	3098	−0.8191859	−0.1292877		4.0
	ZINC67902760	−8.3	403.359	−4.7264	11	4	268.59	0.49428	175.04	−5.9792	648.3946641435	0.30319	10.915	−15.589	0.46429	0.36295	0.94573	1	3099	−0.1772192	0.9736205		4.0
	ZINC67903432	−8.3	430.536	0.8705	8	4	305.55	0.32744	121.14	−5.1488	648.3946641435	0.28298	5.3177	3.0762	0.43333	0.49097	0.96343 (max o	3	5670; 5263; 3538	0.5901766	0.61697704		4.0
	ZINC05434140	−8.3	423.512	−0.4738	10	3	323.86	0.32001	120.08	1.5359	648.3946641435	0.28298	6.662	−1.6743	0.5	0.55396	0.93975 (max o	6	3819; 3693; 277; 481	−0.1184248	0.25625867		3.0
	ZINC05664819	−8.3	420.413	0.3111	9	6	291	0.39409	164.75	0.71166	648.3946641435	0.28298	5.8771	1.0994	0.46667	0.40373	0.94785 (max o	2	337; 1504	−0.9000572	−0.1653128		3.0
	ZINC33359859	−8.3	440.400	−2.7759	11	4	252.24	0.52141	169.05	−2.076	648.3946641435	0.27385	8.9641	−10.136	0.32258	0.091409	0.91162	1	681	−0.2371856	−0.9309517		3.0
	ZINC72325799	−8.3	396.354	−1.9403	9	1	292.45	0.37836	140.18	3.7716	648.3946641435	0.29274	8.1285	−6.6281	0.62069	0.53077	0.97878 (max o	4	3118; 1072; 1073; 16	−0.5495934	−0.3121064		3.0
	ZINC03922770	−8.3	419.502	−4.2998	10	3	258.73	0.48734	191.12	5.2533	648.3946641435	0.31442	10.488	−13.675	0.51852	0.50106	0.85929 (max o	2	1099; 2392	0.4662747	−0.7288878		3.0
	ZINC03922770	−8.3	420.510	−3.955	10	4	270.19	0.50909	203.15	6.0752	648.3946641435	0.31442	10.143	−12.579	0.51852	0.47565	0.85929 (max o	2	1099; 2393	0.48025876	−0.7419517		3.0
	ZINC03849229	−8.3	377.331	−0.3557	8	0	265.08	0.34518	116.17	4.466	648.3946641435	0.30319	6.5439	−1.1732	0.57143	0.3433	0.97773 (max o	4	1109; 1243; 1244; 13	0.64151764	0.27324584		3.0
	ZINC04277737	−8.3	368.392	0.0691	8	0	267.18	0.27446	93.95	1.8264	648.3946641435	0.31442	6.1191	0.21977	0.59259	0.43482	0.95148	1	1157	−0.7486814	0.13104239		3.0
	ZINC35442493	−8.3	430.595	−3.1111	8	3	333.37	0.19732	74.47	8.8043	648.3946641435	0.27385	9.2993	−11.361	0.58065	0.50743	0.94241 (max o	3	5584; 1272; 1601	−0.1733941	0.52729005		3.0
	ZINC03839420	−8.3	446.526	−1.1912	9	3	314.68	0.35836	144.35	7.0911	648.3946641435	0.27385	7.3794	−4.3498	0.54839	0.40992	0.97856	1	2751	−0.2252294	0.93144065		3.0
	ZINC15675167	−8.3	404.418	−0.3127	9	4	303.87	0.35973	130.26	1.5298	648.3946641435	0.29274	6.5009	−1.0682	0.65517	0.53215	0.94543 (max o	3	6180; 4613; 1797	−0.3846811	−0.2059667		3.0
	ZINC03954245	−8.3	365.364	−1.3885	7	2	257.23	0.3152	113.79	5.8403	648.3946641435	0.31442	7.5767	−4.416	0.44444	0.34578	0.96343 (max o	2	1813; 1961	0.6865193	−0.3807287		3.0
	ZINC28966907	−8.3	392.359	−1.3716	9	6	257.5	0.43355	156.91	−2.2179	648.3946641435	0.30319	7.5598	−4.5238	0.46429	0.35879	0.9543 (max of	5	5589; 415; 816; 817;	−0.2315971	0.5947178		3.0
	ZINC00644889	−8.3	355.353	0.9373	9	3	256.53	0.39169	122.39	1.8667	648.3946641435	0.32652	5.2509	2.8706	0.57692	0.2893	0.92032 (max o	4	2824; 2709; 1956; 19	0.69873065	0.23027398		3.0

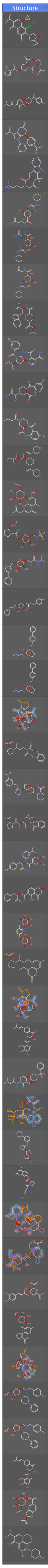

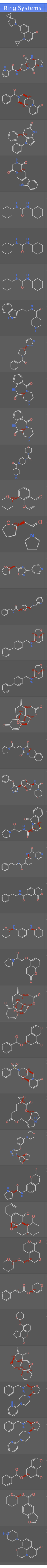

Structure	Molecule Name	affinity	Total Molweight	cLogP	H-Acceptors	H-Donors	Total Surface Area	Relative PSA	Polar Surface Area	Druglikeness	Kd (nM) @ 20°C	LE from K...	LLE from Kd ...	LELP from Kd (n...	Shape Index	Molecular...	Neighbor Si...	Neig...	Neighbor	Neighbor ...	Neighbor ...	Ring Systems	frequency
	ZINC04099036	-8.3	409.819	-0.5881	6	1	260.16	0.23808	91.32	1.9465	648.3946641435	0.30319	6.7763	-1.9397	0.42857	0.31257	0.96519	1	1996	0.735372	0.22781895		3.0
	ZINC03839397	-8.3	373.412	-1.3591	9	2	266.04	0.33472	103.75	8.5337	648.3946641435	0.31442	7.5473	-4.3225	0.48148	0.25988	0.93686 (max o	8	4038; 4036; 3164; 27	-0.586034	-0.1053320		3.0
	ZINC04277711	-8.3	413.471	-0.3237	7	2	316.5	0.23782	74.16	4.4217	648.3946641435	0.28298	6.5119	-1.1439	0.6	0.43664	0.94529 (max o	6	3670; 2449; 590; 244	0.26535386	-0.5910965		3.0
	ZINC40313256	-8.3	374.351	-2.8882	7	1	274.51	0.31332	114.29	3.7179	648.3946641435	0.30319	9.0764	-9.5259	0.46429	0.40006	0.95148 (max o	4	4852; 3525; 3086; 23	0.55452275	-0.2537403		3.0
	ZINC02114809	-8.3	369.487	0.1712	6	2	291.62	0.22207	60.85	6.0854	648.3946641435	0.31442	6.017	0.54449	0.51852	0.38854	0.88639 (max o	3	6115; 3138; 3137	-0.4041456	-0.6424482		3.0
	ZINC03840202	-8.3	386.447	-1.8993	10	7	285.95	0.44788	174.01	-2.8418	648.3946641435	0.31442	8.0875	-6.0406	0.59259	0.54526	0.96389 (max o	2	2371; 2372	0.2835469	0.9313254		3.0
	ZINC03838738	-8.3	386.447	-1.9944	10	7	283.19	0.45224	174.01	-2.7287	648.3946641435	0.31442	8.1826	-6.3431	0.55556	0.52262	0.91731 (max o	2	2383; 2371	0.3036031	0.93223965		3.0
	ZINC03838737	-8.3	372.420	-2.3537	10	7	272.19	0.47052	174.01	-1.8238	648.3946641435	0.32652	8.5419	-7.2085	0.57692	0.54096	0.96389 (max o	2	2383; 2372	0.2857597	0.9423769		3.0
	ZINC04082731	-8.3	414.504	-0.9244	8	3	317.52	0.30511	132.97	5.3113	648.3946641435	0.28298	7.1126	-3.2667	0.56667	0.47404	0.91704 (max o	4	4629; 4015; 566; 168	0.24921076	0.2885108		3.0
	ZINC04221562	-8.3	404.357	-0.8251	11	2	285.71	0.43772	163.5	-2.2589	648.3946641435	0.29274	7.0133	-2.8186	0.44828	0.41476	0.93395 (max o	2	6725; 4536	-0.7085183	-0.5461125		3.0
	ZINC04235872	-8.3	381.185	-0.8899	8	2	230.25	0.37346	112.81	2.0838	648.3946641435	0.3691	7.0781	-2.411	0.52174	0.34767	0.89454 (max o	3	2801; 958; 2431	-0.1701157	0.32622096		3.0
	ZINC04235895	-8.3	382.213	0.2854	7	1	240	0.282	78.95	4.3354	648.3946641435	0.3691	5.9028	0.77322	0.56522	0.38909	0.90468 (max o	2	2801; 2430	-0.1884804	0.3163609		3.0
	ZINC05398621	-8.3	440.519	-1.2845	11	2	327.48	0.3115	125.1	0.80954	648.3946641435	0.27385	7.4727	-4.6905	0.45161	0.57174	0.91561 (max o	5	7697; 6660; 5802; 57	-0.1513524	0.293881		3.0
	ZINC05890342	-8.3	394.375	-0.4815	9	5	272.76	0.40908	153.75	-2.8773	648.3946641435	0.30319	6.6697	-1.5881	0.42857	0.44532	0.95148 (max o	6	5809; 5760; 5496; 54	-0.7940947	0.43717095		3.0
	ZINC08624383	-8.3	366.433	-1.5299	9	3	256.87	0.40032	133.78	4.0938	648.3946641435	0.35373	7.7181	-4.3251	0.58333	0.57671	0.87932	1	3287	0.17127758	-0.3596713		3.0
	ZINC08643372	-8.3	377.400	-0.6704	10	4	283.4	0.42054	142.63	3.2023	648.3946641435	0.31442	6.8586	-2.1322	0.62963	0.47882	0.90719 (max o	2	5857; 4226	-0.2735828	-0.6195981		3.0
	ZINC15675217	-8.3	390.434	-1.5846	7	4	289.32	0.27447	108.29	-1.0286	648.3946641435	0.30319	7.7728	-5.2264	0.67857	0.47863	0.9398 (max of	5	5017; 4173; 4169; 33	0.16091989	-0.5137923		3.0
	ZINC22922206	-8.3	413.560	-1.6145	6	3	329.07	0.18835	62.98	3.4155	648.3946641435	0.28298	7.8027	-5.7053	0.6	0.51797	0.92257 (max o	3	7520; 1014; 1015	0.5221889	-0.1937547		3.0
	ZINC22922206	-8.3	413.560	-5.0568	6	3	344.29	0.22423	67.01	-0.85607	648.3946641435	0.28298	11.245	-17.87	0.6	0.50999	0.87809 (max o	3	7519; 7517; 7220	0.6160267	0.0524428		3.0
	ZINC04083216	-8.3	376.404	-0.0617	7	3	240.04	0.35569	113.29	-3.0006	648.3946641435	0.31442	6.2499	-0.19623	0.37037	0.32456	0.97407 (max o	3	2460; 259; 1162	0.62785757	0.65221006		2.0
	ZINC04090582	-8.3	370.384	-1.7893	8	0	263.51	0.29255	101.06	6.1914	648.3946641435	0.31442	7.9775	-5.6907	0.51852	0.47042	0.95123 (max o	2	344; 895	0.23796715	-0.9020829		2.0
	ZINC04147660	-8.3	445.478	0.108	12	1	324.89	0.34282	116.1	3.4345	648.3946641435	0.26529	6.0802	0.4071	0.5625	0.43129	0.91704 (max o	6	4531; 3649; 3218; 27	-0.2449227	-0.3231929		2.0
	ZINC12881624	-8.3	430.504	-0.7803	8	1	299.31	0.34242	135.15	8.4506	648.3946641435	0.28298	6.9685	-2.7574	0.46667	0.3382	0.93965 (max o	5	3639; 3201; 473; 949	0.39548463	-0.6510829		2.0
	ZINC04221812	-8.3	441.443	-1.968	11	6	323.01	0.42893	187.76	4.972	648.3946641435	0.26529	8.1562	-7.4182	0.5625	0.45689	0.971 (max of 4	4	4540; 704; 705; 1380	-0.458006	-0.1228035		2.0
	ZINC05260601	-8.3	368.364	-0.2877	7	2	272.89	0.32233	115.76	2.3639	648.3946641435	0.31442	6.4759	-0.91501	0.55556	0.50985	0.96343	1	927	0.70907325	-0.4318492		2.0
	ZINC03999065	-8.3	414.453	-1.3791	10	8	289.06	0.4204	163.9	1.6633	648.3946641435	0.29274	7.5673	-4.711	0.51724	0.46956	0.95908 (max o	3	890; 1114; 1115	0.9003793	-0.3535196		2.0
	ZINC04063453	-8.3	358.369	-0.5825	7	0	267.87	0.28727	95.97	3.723	648.3946641435	0.32652	6.7707	-1.784	0.5	0.40009	0.89253 (max o	3	1119; 1399; 1650	0.09881354	0.17744438		2.0
	ZINC04963686	-8.3	376.404	-0.0617	7	3	240.04	0.35569	113.29	-2.0138	648.3946641435	0.31442	6.2499	-0.19623	0.37037	0.32554	0.96343 (max o	3	259; 1162; 2410	0.6282508	0.67265725		2.0
	ZINC35466098	-8.3	445.399	-1.014	10	3	317.43	0.38654	162.65	3.082	648.3946641435	0.26529	7.2022	-3.8222	0.5	0.39902	0.99646 (max o	3	2666; 1310; 1922	-0.0436615	-0.8762144		2.0
	ZINC04236529	-8.3	431.535	-1.056	8	2	320.98	0.28291	99.61	-0.53963	648.3946641435	0.28298	7.2442	-3.7317	0.56667	0.41711	0.88492	1	3225	0.07505587	-0.8269039		2.0
	ZINC06031602	-8.3	381.423	-5.252	8	1	263.69	0.31662	108.42	5.2676	648.3946641435	0.31442	11.44	-16.704	0.37037	0.18778	0.96885 (max o	3	1183; 1442; 1443	0.66401	0.6245253		2.0
	ZINC03842034	-8.3	375.408	-3.6629	10	1	268.59	0.36416	111.4	-0.021499	648.3946641435	0.31442	9.8511	-11.65	0.55556	0.44335	0.92486 (max o	2	1451; 2387	-0.0028278	-0.3805748		2.0
	ZINC04899897	-8.3	402.493	-5.9292	8	3	305.02	0.30519	130.99	-3.0855	648.3946641435	0.29274	12.117	-20.254	0.58621	0.48942	0.88922 (max o	4	5410; 4906; 4557; 28	-0.6543013	-0.6350828		2.0
	ZINC36378601	-8.3	360.361	-0.1543	7	4	232.58	0.41332	127.59	0.18236	648.3946641435	0.32652	6.3425	-0.47257	0.42308	0.29718	0.9615 (max of	3	845; 1612; 1925	0.0470456	0.96213686		2.0
	ZINC67903441	-8.3	360.358	-2.0162	9	4	247.63	0.4258	134.91	-2.1618	648.3946641435	0.33958	8.2044	-5.9374	0.48	0.50401	0.95318 (max o	3	1618; 2315; 2686	0.6092587	0.64522094		2.0
	ZINC67913502	-8.3	364.261	-5.2283	10	3	255.83	0.49635	176.48	-0.72708	648.3946641435	0.32652	11.416	-16.012	0.53846	0.41098	0.93361 (max o	3	1621; 1622; 1623	-0.3628955	-0.8636712		2.0
	ZINC04064305	-8.3	378.376	0.0735	8	4	258.63	0.36902	125.68	-6.0454	648.3946641435	0.31442	6.1147	0.23376	0.44444	0.25864	0.94905 (max o	6	2783; 2782; 437; 439	0.35963464	0.49058172		2.0
	ZINC12661785	-8.3	364.436	0.5857	6	4	231.86	0.32537	107.22	-5.1582	648.3946641435	0.32652	5.6025	1.7938	0.34615	0.35342	0.93361 (max o	3	4869; 3622; 1821	0.84376955	0.2998545		2.0
	ZINC20465657	-8.3	418.511	-2.9478	7	4	297.52	0.17155	86.92	2.5628	648.3946641435	0.28298	9.136	-10.417	0.6	0.43024	0.93686 (max o	3	6576; 2598; 1872	-0.4534406	0.29855812		2.0
	ZINC20465657	-8.3	417.503	-0.97	7	3	308.49	0.20101	85.67	2.5628	648.3946641435	0.28298	7.1582	-3.4278	0.6	0.44706	0.96589 (max o	9	6576; 3848; 3394; 18	-0.4711118	0.28466934		2.0
	ZINC35466104	-8.3	445.399	-1.014	10	3	317.43	0.38654	162.65	3.082	648.3946641435	0.26529	7.2022	-3.8222	0.5	0.40053	0.99646 (max o	3	1310; 1922; 2665	-0.0626711	-0.9049312		2.0
	ZINC49601569	-8.3	366.365	0.7334	8	4	256.94	0.37145	125.68	-17.662	648.3946641435	0.32652	5.4548	2.2461	0.53846	0.38283	0.97269 (max o	3	3088; 2676; 1938	-0.1365445	-0.8885133		2.0
	ZINC00537891	-8.3	361.372	-2.287	7	1	267.61	0.28045	77.35	2.8174	648.3946641435	0.32652	8.4752	-7.0043	0.5	0.40788	0.94951 (max o	5	5315; 3122; 1950; 19	0.6092922	-0.3939047		2.0

Structure	Molecule Name	affinity	Total Molweight	cLogP	H-Acceptors	H-Donors	Total Surface Area	Relative PSA	Polar Surface Area	Druglikeness	Kd (nM) @ 20°C	LE from K...	LLE from Kd ...	LELP from Kd (n...	Shape Index	Molecular...	Neighbor Si...	Neig...	Neighbor	Neighbor ...	Neighbor ...	Ring Systems	frequency
	ZINC00537891	-8.3	360.364	-2.4186	7	0	253.57	0.2406	76.15	7.0211	648.3946641435	0.32652	8.6068	-7.4073	0.5	0.40788	0.94951 (max o	5	5316; 3123; 1951; 19	0.5695155	-0.3780062		2.0
	ZINC00537891	-8.3	362.380	-3.6473	7	2	256.21	0.25901	80.28	1.7724	648.3946641435	0.32652	9.8355	-11.17	0.5	0.34931	0.95857 (max o	2	3124; 1954	-0.0823835	-0.8346229		2.0
	ZINC12662595	-8.3	411.405	-0.4553	10	4	291.26	0.38464	145.99	0.28886	648.3946641435	0.29274	6.6435	-1.5553	0.58621	0.5072	0.95908 (max o	3	1050; 1844; 2158	0.06642091	-0.9429000		2.0
	ZINC19702869	-8.3	373.335	-0.7802	7	2	266.22	0.30501	112.5	-1.4732	648.3946641435	0.31442	6.9684	-2.4814	0.59259	0.44071	0.87576	1	2207	-0.2271414	-0.9167912		2.0
	ZINC35456072	-8.3	391.482	-0.6403	6	3	280.75	0.30016	113.35	-3.3875	648.3946641435	0.30319	6.8285	-2.1119	0.42857	0.36958	0.9678 (max of	5	4410; 4368; 3934; 22	-0.1300729	0.4100195		2.0
	ZINC67903444	-8.3	360.358	-2.0162	9	4	247.63	0.4258	134.91	-2.1618	648.3946641435	0.33958	8.2044	-5.9374	0.48	0.50401	0.95318 (max o	3	1618; 2315; 2685	0.62763083	0.63202214		2.0
	ZINC67913884	-8.3	389.332	-4.9553	11	4	258.42	0.51374	175.04	-4.8669	648.3946641435	0.31442	11.143	-15.76	0.44444	0.41256	0.94302 (max o	3	4865; 4437; 2319	-0.3276589	0.87115026		2.0
	ZINC79189886	-8.3	395.342	-0.1783	8	0	280.74	0.33269	111.19	-1.4098	648.3946641435	0.29274	6.3665	-0.60908	0.51724	0.37549	0.9426 (max of	3	4457; 2703; 2334	-0.7260175	-0.3885152		2.0
	ZINC11851965	-8.3	420.413	0.222	9	5	301.3	0.36024	145.91	-3.6574	648.3946641435	0.28298	5.9662	0.78451	0.6	0.47734	0.95579 (max o	7	2909; 829; 1144; 158	0.39872313	0.5557703		2.0
	ZINC02115109	-8.3	420.413	0.222	9	5	301.3	0.36024	145.91	-3.6574	648.3946641435	0.28298	5.9662	0.78451	0.6	0.47774	0.95837 (max o	7	2909; 2517; 829; 114	0.37782672	0.55205363		2.0
	ZINC03839323	-8.3	406.481	-0.8669	9	2	296.38	0.31102	108.05	1.685	648.3946641435	0.29274	7.0551	-2.9613	0.55172	0.37004	0.97649 (max o	6	4922; 3709; 2750; 89	-0.5733709	-0.0080638		2.0
	ZINC03839847	-8.3	360.413	-0.701	8	1	260.59	0.28608	83.88	8.1593	648.3946641435	0.32652	6.8892	-2.1469	0.53846	0.29513	0.84147 (max o	2	4926; 1604	-0.4394962	-0.7953251		2.0
	ZINC04085476	-8.3	359.425	-0.2233	6	0	245.34	0.31789	106.05	8.0296	648.3946641435	0.33958	6.4115	-0.65758	0.44	0.24828	0.92124 (max o	3	3203; 3201; 473	0.42426226	-0.6274579		2.0
	ZINC04259620	-8.3	429.499	-2.0696	10	2	333.89	0.33505	104.83	0.90274	648.3946641435	0.27385	8.2578	-7.5574	0.58065	0.37205	0.87477 (max o	2	2974; 2815	-0.1750101	-0.3749029		2.0
	ZINC08295675	-8.3	374.372	-1.3	9	0	273.01	0.319	110.29	0.10042	648.3946641435	0.31442	7.4882	-4.1346	0.59259	0.48882	0.96343 (max o	3	6906; 5004; 4151	0.53700054	0.55050504		2.0
	ZINC12296953	-8.3	364.357	-0.6284	10	1	247.45	0.36153	110.7	3.8382	648.3946641435	0.32652	6.8166	-1.9246	0.46154	0.38146	0.95559 (max o	3	3319; 1485; 2143	-0.0598739	-0.9234814		2.0
	ZINC12483106	-8.3	413.496	-1.1561	8	2	295.88	0.24317	91.74	6.2154	648.3946641435	0.28298	7.3443	-4.0854	0.56667	0.44411	0.93975 (max o	2	3426; 2915	0.5362756	-0.7055443		2.0
	ZINC12494765	-8.3	352.342	-7.7802	9	3	240.72	0.39868	134.24	2.8108	648.3946641435	0.33958	13.968	-22.911	0.48	0.44503	0.93348 (max o	4	4647; 4052; 4051; 36	-0.7548520	-0.0055758		2.0
	ZINC12603872	-8.3	369.411	-1.8203	7	3	267.42	0.26374	95.84	-0.06504	648.3946641435	0.32652	8.0085	-5.5749	0.65385	0.48902	0.92368 (max o	5	6755; 4228; 4222; 41	0.06614678	-0.5050227		2.0
	ZINC12862693	-8.3	439.879	0.1186	10	5	294.16	0.46203	180.52	1.9978	648.3946641435	0.29274	6.0696	0.40514	0.48276	0.40059	0.97878 (max o	5	4202; 3780; 1196; 17	0.30692694	-0.5517114		2.0
	ZINC13513946	-8.3	443.419	-8.3663	13	6	324.07	0.50853	212.93	4.3321	648.3946641435	0.26529	14.554	-31.536	0.625	0.58236	0.98148 (max o	5	2956; 2955; 624; 720	0.8251164	0.27085814		2.0
	ZINC13660199	-8.3	388.455	-0.0883	8	5	281.14	0.3505	136.68	-3.7924	648.3946641435	0.31442	6.2765	-0.28083	0.51852	0.4588	0.97407 (max o	6	5103; 4679; 3790; 33	-0.3601005	0.20188971		2.0
	ZINC20463366	-8.3	374.483	-1.5985	7	1	307.85	0.24694	81.01	4.1263	648.3946641435	0.31442	7.7867	-5.0839	0.55556	0.58806	0.96064 (max o	3	4724; 2584; 2145	0.60630226	0.40563145		2.0
	ZINC20463670	-8.3	401.485	-1.4327	8	3	286.69	0.2615	94.64	6.502	648.3946641435	0.29274	7.6209	-4.8941	0.55172	0.42735	0.93686	1	3869	-0.1022252	-0.9725241		2.0
	ZINC20465833	-8.3	406.952	-0.907	5	4	320.78	0.19181	70.74	-0.9372	648.3946641435	0.30319	7.0952	-2.9915	0.57143	0.5012	0.9308 (max of	3	7412; 4317; 3006	-0.4163034	-0.6578145		2.0
	ZINC20760360	-8.3	401.442	-1.3543	9	3	279.25	0.35875	135.41	3.8291	648.3946641435	0.29274	7.5425	-4.6263	0.41379	0.28829	0.93101 (max o	3	4334; 4336; 3861	-0.686555	0.59116274		2.0
	ZINC22927066	-8.3	400.565	-0.6812	6	3	322.16	0.20459	66.38	0.70801	648.3946641435	0.29274	6.8694	-2.327	0.44828	0.37208	0.9617	1	3435	0.91914797	0.23875034		2.0
	ZINC22936700	-8.3	390.482	-4.4924	8	3	312.01	0.31614	105.57	5.5413	648.3946641435	0.30319	10.681	-14.817	0.60714	0.48378	0.90725 (max o	4	4347; 4344; 3430; 18	-0.1815384	0.20123643		2.0
	ZINC22936700	-8.3	391.490	-1.4378	8	3	292.96	0.27168	102.63	6.9614	648.3946641435	0.30319	7.626	-4.7422	0.60714	0.43029	0.90725 (max o	5	4349; 4345; 4343; 34	-0.4523785	0.0835086		2.0
	ZINC31155429	-8.3	370.440	0.6306	7	4	275.33	0.31032	116.45	-4.553	648.3946641435	0.32652	5.5576	1.9313	0.53846	0.47666	0.97269 (max o	5	5189; 3885; 3884; 38	0.25005275	-0.5025569		2.0
	ZINC31157778	-8.3	396.478	0.2159	7	4	273.46	0.31244	116.45	-7.2753	648.3946641435	0.30319	5.9723	0.71209	0.39286	0.29698	0.96519 (max o	3	6328; 3926; 3455	0.7061768	0.3167858		2.0
	ZINC31157933	-8.3	427.405	-2.4776	11	6	298.59	0.44261	182.09	-10.383	648.3946641435	0.28298	8.6658	-8.7554	0.53333	0.48614	0.96343 (max o	4	3895; 3037; 842; 263	-0.4969158	0.7561641		2.0
	ZINC31162312	-8.3	391.351	-1.8148	9	5	272.81	0.41355	159.74	-4.1818	648.3946641435	0.30319	8.003	-5.9856	0.46429	0.38246	0.8991	1	2636	-0.0188218	0.96820796		2.0
	ZINC31162312	-8.3	392.359	-0.2368	9	6	271.63	0.411	156.91	-4.2658	648.3946641435	0.30319	6.425	-0.78102	0.46429	0.38246	0.8991	1	2637	-0.0148052	0.95261836		2.0
	ZINC31163364	-8.3	445.483	-2.0251	10	4	327.72	0.37459	165.81	-2.0398	648.3946641435	0.27385	8.2133	-7.3949	0.51613	0.62779	0.96739 (max o	5	6007; 4810; 3504; 30	0.19243445	0.34575513		2.0
	ZINC34660468	-8.3	383.404	-1.3187	11	6	275.35	0.46029	166.01	-5.8806	648.3946641435	0.31442	7.5069	-4.194	0.55556	0.43941	0.97903 (max o	2	2671; 2648	0.36327213	0.9044589		2.0
	ZINC34660466	-8.3	383.404	-1.3187	11	6	275.35	0.46029	166.01	-5.8806	648.3946641435	0.31442	7.5069	-4.194	0.55556	0.43941	0.97903 (max o	2	2671; 2649	0.37036943	0.8890291		2.0
	ZINC35465282	-8.3	431.369	-4.62	12	6	288.83	0.50521	198.43	0.77107	648.3946641435	0.28298	10.808	-16.326	0.56667	0.44536	0.95837 (max o	4	4814; 3507; 3076; 30	0.30226567	-0.6793332		2.0
	ZINC49181193	-8.3	430.492	-0.5521	9	6	296.67	0.37631	156.91	-2.4223	648.3946641435	0.28298	6.7403	-1.951	0.43333	0.40838	0.96092 (max o	3	5660; 5659; 3087	0.65454084	-0.548789		2.0
	ZINC49601571	-8.3	366.365	0.7334	8	4	256.94	0.37145	125.68	-17.662	648.3946641435	0.32652	5.4548	2.2461	0.53846	0.38794	0.97269 (max o	3	3088; 1938; 2675	-0.1233548	-0.9040035		2.0
	ZINC49604876	-8.3	353.413	0.5349	7	2	251.11	0.30198	96.3	6.0667	648.3946641435	0.33958	5.6533	1.5752	0.36	0.22028	0.99111 (max o	3	6537; 6361; 3092	-0.5356406	0.5138824		2.0
	ZINC71404395	-8.3	407.374	0.2351	10	4	283.59	0.41426	143.87	-4.1473	648.3946641435	0.29274	5.9531	0.80311	0.44828	0.25198	0.94823 (max o	3	3368; 2969; 2694	0.86285585	0.04240133		2.0
	ZINC71404395	-8.3	408.382	-1.7427	10	5	272.62	0.39069	145.12	-4.341	648.3946641435	0.29274	7.9309	-5.9531	0.44828	0.25198	0.94823 (max o	5	3369; 2970; 2695; 20	-0.6423349	-0.690839		2.0

Structure	Molecule Name	affinity	Total Molweight	cLogP	H-Acceptors	H-Donors	Total Surface Area	Relative PSA	Polar Surface Area	Druglikeness	Kd (nM) @ 20°C	LE from K...	LLE from Kd ...	LELP from Kd (n...	Shape Index	Molecular...	Neighbor Si...	Neig...	Neighbor	Neighbor ...	Neighbor ...	Ring Systems	frequency
	ZINC71404396	-8.3	407.374	0.2351	10	4	283.59	0.41426	143.87	-4.1473	648.3946641435	0.29274	5.9531	0.80311	0.44828	0.24826	0.94823 (max o	3	3368; 2969; 2692	0.85426146	0.02131854		2.0
	ZINC79189889	-8.3	395.342	-0.1783	8	0	280.74	0.33269	111.19	-1.4098	648.3946641435	0.29274	6.3665	-0.60908	0.51724	0.36797	0.93975 (max o	3	4457; 2334; 2702	-0.7426029	-0.3739203		2.0
	ZINC00753726	-8.3	357.436	-0.4935	8	2	267.23	0.2264	76.89	1.2135	648.3946641435	0.32652	6.6817	-1.5114	0.5	0.4759		0		0.7777097	0.6207816		1.0
	ZINC01090002	-8.3	386.371	-0.5214	12	0	270.7	0.37695	116.88	4.3686	648.3946641435	0.30319	6.7096	-1.7197	0.42857	0.53461	0.85534	1	2342	-0.0588859	-0.9805725		1.0
	ZINC01090002	-8.3	387.379	-2.4992	12	1	259.73	0.35063	118.13	4.121	648.3946641435	0.30319	8.6874	-8.2429	0.42857	0.53461	0.85534	1	2341	-0.0522759	-0.9890567		1.0
	ZINC01323478	-8.3	418.473	0.1658	9	2	303.52	0.37592	124.46	6.7296	648.3946641435	0.29274	6.0224	0.56638	0.48276	0.41168	0.97649 (max o	3	637; 801; 999	-0.1945140	0.66078746		1.0
	ZINC01610008	-8.3	364.380	0.0312	7	1	260.4	0.2573	91.37	2.4976	648.3946641435	0.31442	6.157	0.099229	0.55556	0.53096	0.90236	1	3523	-0.6461428	0.7476445		1.0
	ZINC02136900	-8.3	350.353	-1.2955	7	1	266.84	0.29951	101.9	5.755	648.3946641435	0.32652	7.4837	-3.9676	0.57692	0.49427	0.86721	1	3152	0.04776782	-0.2651355		1.0
	ZINC03826253	-8.3	402.445	-2.3085	7	2	266.37	0.21215	92.45	0.5591	648.3946641435	0.29274	8.4967	-7.8859	0.44828	0.31216	0.87695 (max o	4	4423; 3998; 3246; 23	-0.1540937	-0.9367314		1.0
	ZINC03830511	-8.3	422.461	-2.1376	9	1	291.75	0.47095	179.33	10.36	648.3946641435	0.30319	8.3258	-7.0503	0.60714	0.49807	0.9428 (max of	4	6697; 4258; 2051; 21	0.05126884	-0.9124452		1.0
	ZINC03838472	-8.3	440.451	0.2375	9	3	321.67	0.32468	129.31	2.9736	648.3946641435	0.26529	5.9507	0.89523	0.46875	0.46033		0		0.53293246	-0.5752157		1.0
	ZINC03839269	-8.3	440.498	0.3217	9	2	314.86	0.29277	108.05	5.9909	648.3946641435	0.26529	5.8665	1.2126	0.5625	0.33412	0.92889 (max o	4	252; 429; 700; 1376	-0.4778248	-0.2166979		1.0
	ZINC03839662	-8.3	386.450	-0.3186	8	1	274.85	0.27124	83.88	8.4344	648.3946641435	0.30319	6.5068	-1.0508	0.53571	0.24302	0.87218 (max o	3	6702; 6092; 4923	-0.3248727	0.6907577		1.0
	ZINC03839850	-8.3	446.454	-1.9787	9	1	319.78	0.29708	119.08	3.9874	648.3946641435	0.26529	8.1669	-7.4585	0.625	0.42519	0.8417 (max of	3	5654; 1149; 1720	-0.681021	0.5360934		1.0
	ZINC03840171	-8.3	397.426	-1.5195	10	6	291.9	0.44782	175.12	5.5928	648.3946641435	0.30319	7.7077	-5.0117	0.5	0.46985	0.90299 (max o	4	5334; 4042; 432; 167	-0.4249874	-0.1958819		1.0
	ZINC03841211	-8.3	350.394	-2.6416	8	2	258.59	0.35929	118.64	1.3971	648.3946641435	0.33958	8.8298	-7.7791	0.6	0.45648		0		0.6564371	0.74005735		1.0
	ZINC03841221	-8.3	413.472	-0.0070999	8	2	304.02	0.26571	99.18	0.22719	648.3946641435	0.28298	6.1953	-0.02509	0.6	0.40208	0.83981	1	1497	-0.7202971	0.15466243		1.0
	ZINC03842036	-8.3	429.499	-0.6409	10	1	308.88	0.29484	100.37	1.7	648.3946641435	0.27385	6.8291	-2.3403	0.58065	0.42317	0.9228 (max of	4	2764; 907; 1451; 238	-0.0190426	-0.3491296		1.0
	ZINC03845636	-8.3	416.348	-3.1485	10	0	295.83	0.37467	145.6	4.7927	648.3946641435	0.27385	9.3367	-11.497	0.48387	0.33329	0.94223	1	2251	-0.5778228	-0.788432		1.0
	ZINC03849143	-8.3	369.332	-0.833	8	0	248.76	0.44967	144.41	6.4135	648.3946641435	0.32652	7.0212	-2.5512	0.53846	0.30518	0.9066	1	4274	-0.8427363	0.5307965		1.0
	ZINC03952881	-8.3	355.305	-2.1276	9	2	269.68	0.4274	154.31	-2.9415	648.3946641435	0.32652	8.3158	-6.5161	0.65385	0.54377		0		0.8733794	-0.3321690		1.0
	ZINC03984507	-8.3	364.428	-1.2815	7	2	254.75	0.21213	75.48	6.167	648.3946641435	0.31442	7.4697	-4.0757	0.48148	0.39481	0.88831 (max o	3	6115; 6114; 2216	-0.3554476	-0.7019017		1.0
	ZINC04025519	-8.3	350.346	0.1017	7	3	223.98	0.45928	138.59	0.27969	648.3946641435	0.35373	6.0865	0.28751	0.5	0.33674		0		0.8470228	-0.4766528		1.0
	ZINC04083912	-8.3	373.451	-1.6551	7	2	284.12	0.26802	106.15	5.4222	648.3946641435	0.31442	7.8433	-5.2639	0.59259	0.44943	0.90502 (max o	4	4663; 4515; 3638; 36	0.29212853	0.36897317		1.0
	ZINC04236532	-8.3	437.564	-1.0669	8	2	316.92	0.35078	127.85	6.803	648.3946641435	0.29274	7.2551	-3.6445	0.55172	0.42636	0.89081	1	3225	0.13380857	-0.8034535		1.0
	ZINC04260763	-8.3	376.412	0.9347	9	1	282.95	0.28708	91.42	1.9834	648.3946641435	0.31442	5.2535	2.9727	0.55556	0.58411	0.84495	1	1526	-0.0931875	0.30675626		1.0
	ZINC04260850	-8.3	388.446	-1.6703	9	2	305.85	0.3299	91.94	-14.091	648.3946641435	0.30319	7.8585	-5.509	0.64286	0.37783	0.91204 (max o	6	731; 732; 733; 1150;	-0.2184797	-0.0296844		1.0
	ZINC04270622	-8.3	416.437	0.0195	11	1	302.33	0.35666	112.86	2.8711	648.3946641435	0.28298	6.1687	0.06891	0.56667	0.38804	0.91993 (max o	6	3252; 2815; 444; 450	-0.2074589	-0.3079228		1.0
	ZINC04277712	-8.3	396.469	-1.3714	8	2	308.91	0.27918	87.05	5.7617	648.3946641435	0.29274	7.5596	-4.6847	0.58621	0.37159	0.97415 (max o	6	3857; 3670; 2449; 20	0.25801829	-0.6108024		1.0
	ZINC04277713	-8.3	397.458	-2.3723	9	2	307.67	0.31596	99.94	5.7617	648.3946641435	0.29274	8.5605	-8.1038	0.58621	0.37159	0.97176 (max o	6	3857; 3670; 2449; 20	0.22947969	-0.5825063		1.0
	ZINC04277714	-8.3	396.469	-1.4254	8	2	308.91	0.27918	87.05	5.7617	648.3946641435	0.29274	7.6136	-4.8692	0.58621	0.42755	0.97415 (max o	6	3857; 3670; 2067; 24	0.23944594	-0.6174102		1.0
	ZINC04277742	-8.3	377.463	-0.7596	8	1	273.53	0.24681	86.77	-0.98797	648.3946641435	0.31442	6.9478	-2.4159	0.59259	0.40988	0.90441 (max o	2	4975; 4105	-0.6697169	0.18081735		1.0
	ZINC05414704	-8.3	437.459	-1.3208	11	2	324.38	0.3548	137.49	5.0549	648.3946641435	0.26529	7.509	-4.9786	0.4375	0.46221	0.97743 (max o	10	4133; 2929; 2572; 24	-0.7078230	-0.4360474		1.0
	ZINC05433803	-8.3	397.454	0.1597	8	2	280.8	0.28896	91.34	0.70444	648.3946641435	0.29274	6.0285	0.54554	0.58621	0.33604	0.7988	1	932	0.6998664	0.65745074		1.0
	ZINC05434229	-8.3	438.447	-2.2677	12	2	323.14	0.39011	150.38	5.0549	648.3946641435	0.26529	8.4559	-8.5479	0.4375	0.42081	0.971 (max of 6	6	4133; 2572; 278; 931	-0.7069737	-0.3847452		1.0
	ZINC05496652	-8.3	429.471	0.5811	9	4	292.22	0.33013	126.33	6.1715	648.3946641435	0.27385	5.6071	2.1219	0.41935	0.31023		0		-0.9520952	-0.1398682		1.0
	ZINC08462770	-8.3	354.345	-0.3346	9	0	252.96	0.32373	101.81	-6.0036	648.3946641435	0.32652	6.5228	-1.0248	0.5	0.38475		0		-0.7746396	0.6300796		1.0
	ZINC08627187	-8.3	377.439	-0.874	8	4	284.96	0.33749	120.78	-0.70945	648.3946641435	0.31442	7.0622	-2.7797	0.62963	0.50179	0.92739 (max o	3	6998; 5020; 2879	0.41046235	-0.4715632		1.0
	ZINC08740133	-8.3	432.480	-2.9758	10	3	296.21	0.42338	159.21	13.08	648.3946641435	0.28298	9.164	-10.516	0.43333	0.41799	0.9707 (max of	2	4939; 1197	0.13159633	-0.0972355		1.0
	ZINC12480829	-8.3	431.531	-2.0782	8	1	340.05	0.25905	94.42	3.6925	648.3946641435	0.27385	8.2664	-7.5888	0.51613	0.5676	0.92584 (max o	2	5849; 1539	0.29911327	0.836606		1.0
	ZINC12481047	-8.3	424.540	-1.9106	9	1	342.68	0.26742	97.66	3.6352	648.3946641435	0.28298	8.0988	-6.7517	0.53333	0.63181	0.89412 (max o	2	4641; 4296	-0.1377644	-0.2551124		1.0
	ZINC12489917	-8.3	414.480	-0.5945	8	2	299.23	0.25185	89.89	4.3452	648.3946641435	0.28298	6.7827	-2.1009	0.56667	0.46622	0.9103 (max of	7	7850; 7848; 7630; 73	0.6628794	0.5638195		1.0
	ZINC12603458	-8.3	416.500	-2.4723	9	4	326.31	0.32822	113.94	-1.572	648.3946641435	0.28298	8.6605	-8.7366	0.63333	0.56614	0.91076 (max o	5	5450; 4605; 3738; 17	0.22345279	-0.6982658		1.0

Structure	Molecule Name	affinity	Total Molweight	cLogP	H-Acceptors	H-Donors	Total Surface Area	Relative PSA	Polar Surface Area	Druglikeness	Kd (nM) @ 20°C	LE from K...	LLE from Kd ...	LELP from Kd (n...	Shape Index	Molecular...	Neighbor Si...	Neig...	Neighbor	Neighbor ...	Neighbor ...	Ring Systems	frequency
	ZINC12888593	-8.3	429.452	-1.5514	10	2	316.88	0.34556	138.95	7.6805	648.3946641435	0.27385	7.7396	-5.6651	0.48387	0.54384	0.8956 (max of	6	4761; 4678; 4665; 46	0.3299797	0.7255845		1.0
	ZINC13409452	-8.3	366.360	-1.3073	9	1	258.38	0.46497	153.2	3.7915	648.3946641435	0.32652	7.4955	-4.0038	0.5	0.157	0.85984 (max o	2	971; 2548	-0.4068814	-0.8200627		1.0
	ZINC13409452	-8.3	367.368	-0.4169	9	2	257.09	0.46229	146.88	6.6044	648.3946641435	0.32652	6.6051	-1.2768	0.5	0.157	0.85984	1	2547	-0.3954099	-0.8037474		1.0
	ZINC13548131	-8.3	377.205	-8.9994	14	5	231.42	0.73939	231.74	-25.168	648.3946641435	0.33958	15.188	-26.502	0.48	0.50338	0.84884	1	5876	-0.2812121	-0.3173524		1.0
	ZINC13863965	-8.3	355.305	-2.6325	9	2	270.44	0.42579	150.82	5.0531	648.3946641435	0.32652	8.8207	-8.0624	0.65385	0.49932		0		-0.920599	0.37610045		1.0
	ZINC13885108	-8.3	350.398	-3.6074	8	1	232.65	0.40877	127.67	5.2739	648.3946641435	0.35373	9.7956	-10.198	0.5	0.33754	0.93334	1	4026	0.6884138	0.7082096		1.0
	ZINC15675046	-8.3	366.376	-1.83	8	2	287.5	0.36532	116.61	2.0705	648.3946641435	0.31442	8.0182	-5.8202	0.59259	0.43598	0.98371 (max o	3	2971; 2622; 2191	0.44939947	-0.6589941		1.0
	ZINC15675630	-8.3	402.413	-2.4777	9	2	291.73	0.31495	125.11	4.2628	648.3946641435	0.28298	8.6659	-8.7557	0.53333	0.41114	0.88025	1	1831	0.7096128	-0.5538923		1.0
	ZINC15709548	-8.3	437.459	-1.3208	11	2	324.38	0.3548	137.49	5.0549	648.3946641435	0.26529	7.509	-4.9786	0.4375	0.46026	0.97743 (max o	9	4133; 278; 930; 931;	-0.6786481	-0.4120724		1.0
	ZINC19535444	-8.3	369.356	-2.7303	9	2	270.01	0.36662	125.64	3.3257	648.3946641435	0.31442	8.9185	-8.6835	0.51852	0.42944	0.93395 (max o	2	2728; 694	0.5998484	-0.5735515		1.0
	ZINC20463387	-8.3	400.521	-1.6009	7	1	297.1	0.17169	81.06	9.2968	648.3946641435	0.29274	7.7891	-5.4687	0.55172	0.54002	0.93385 (max o	10	5916; 4723; 3387; 33	-0.1966414	-0.2494859		1.0
	ZINC20463387	-8.3	400.521	-1.2804	7	1	322.11	0.23601	81.01	5.3001	648.3946641435	0.29274	7.4686	-4.3739	0.55172	0.55024	0.93385 (max o	2	4724; 2581	0.6273365	0.3994456		1.0
	ZINC20463423	-8.3	442.582	-0.6086	7	1	326.28	0.21874	109.3	9.8467	648.3946641435	0.27385	6.7968	-2.2224	0.54839	0.53697	0.91292 (max o	10	5530; 4723; 3387; 33	-0.1746239	-0.2458304		1.0
	ZINC20463670	-8.3	400.477	-0.208	8	3	290.41	0.27096	90.06	7.1009	648.3946641435	0.29274	6.3962	-0.71053	0.55172	0.45149		0		-0.3223610	0.890744		1.0
	ZINC20611984	-8.3	378.511	0.4945	6	1	277.55	0.21938	65.54	4.5685	648.3946641435	0.31442	5.6937	1.5727	0.55556	0.47599	0.89424	1	3020	-0.5615622	0.6176893		1.0
	ZINC20758848	-8.3	438.482	-0.8184	9	4	321.09	0.29023	142.75	3.4063	648.3946641435	0.26529	7.0066	-3.0849	0.46875	0.46074	0.80776	1	1010	-0.3586867	-0.9177146		1.0
	ZINC20760451	-8.3	385.466	0.1656	8	3	292.24	0.2576	91.91	9.5077	648.3946641435	0.30319	6.0226	0.54619	0.64286	0.57927		0		0.74701244	-0.6621293		1.0
	ZINC20760761	-8.3	423.559	-1.5397	7	4	370.43	0.25832	79.44	3.3891	648.3946641435	0.27385	7.7279	-5.6224	0.67742	0.61367		0		0.39843282	0.91245645		1.0
	ZINC22923472	-8.3	422.615	-4.3532	8	3	351.7	0.23705	64.77	9.4625	648.3946641435	0.28298	10.541	-15.383	0.6	0.50894	0.9707 (max of	7	5584; 3874; 3025; 30	-0.1694361	0.55686235		1.0
	ZINC22923472	-8.3	422.615	-4.3532	8	3	351.7	0.23705	64.77	7.8637	648.3946641435	0.28298	10.541	-15.383	0.6	0.49703	0.84335 (max o	2	5545; 1281	-0.4286502	-0.6735098		1.0
	ZINC22923472	-8.3	423.623	-6.4629	8	4	365.74	0.26634	65.97	5.2194	648.3946641435	0.28298	12.651	-22.839	0.6	0.50894	0.90725 (max o	8	6284; 5583; 3874; 30	-0.1433563	0.5844598		1.0
	ZINC30878790	-8.3	369.460	-0.5188	8	1	280.63	0.24883	82.55	7.9918	648.3946641435	0.32652	6.707	-1.5889	0.61538	0.58013	0.89162 (max o	4	7732; 6287; 2621; 22	-0.3343699	0.34046686		1.0
	ZINC30878790	-8.3	370.468	-2.6285	8	2	294.67	0.28462	83.75	3.6866	648.3946641435	0.32652	8.8167	-8.0501	0.61538	0.57563	0.89524 (max o	3	7733; 6288; 2620	-0.3188736	0.35414183		1.0
	ZINC31153514	-8.3	367.364	-2.7399	9	2	286.26	0.40523	129.5	2.0705	648.3946641435	0.31442	8.9281	-8.7141	0.59259	0.3982	0.96885 (max o	3	2971; 2191; 2567	0.45784518	-0.6390526		1.0
	ZINC31155343	-8.3	448.419	-2.0505	12	6	308.9	0.44869	176.76	-4.801	648.3946641435	0.27385	8.2387	-7.4876	0.54839	0.45752	0.96262 (max o	5	4359; 1573; 1605; 18	0.5934489	0.4900178		1.0
	ZINC31157261	-8.3	380.392	0.1928	8	4	270.7	0.35257	125.68	-6.4044	648.3946641435	0.31442	5.9954	0.61319	0.55556	0.50204		0		-0.7602956	0.6482789		1.0
	ZINC34554784	-8.3	381.380	-1.4059	9	4	270.71	0.39467	134.55	-8.4237	648.3946641435	0.31442	7.5941	-4.4714	0.44444	0.40473	0.93996 (max o	3	4816; 3479; 3478	0.6659384	-0.3776036		1.0
	ZINC35442498	-8.3	402.517	-2.3828	8	2	320.78	0.25937	79.21	4.6521	648.3946641435	0.29274	8.571	-8.1397	0.58621	0.48912	0.93686 (max o	6	5950; 3866; 3865; 83	-0.0049806	0.5304391		1.0
	ZINC35442829	-8.3	361.440	-0.0116	7	1	253.91	0.26655	78.95	6.0196	648.3946641435	0.32652	6.1998	-0.035526	0.57692	0.25749	0.91351	1	4923	-0.3039463	0.69923687		1.0
	ZINC35465657	-8.3	446.407	-0.0864	10	5	308.75	0.38394	155.14	-3.4398	648.3946641435	0.26529	6.2746	-0.32568	0.46875	0.3238	0.95709 (max o	3	506; 625; 828	0.14242543	0.6146587		1.0
	ZINC35465985	-8.3	420.413	0.4007	9	6	299.15	0.37319	156.91	-4.8731	648.3946641435	0.28298	5.7875	1.416	0.6	0.50177	0.91076 (max o	2	3468; 655	0.4386847	0.48590165		1.0
	ZINC40165547	-8.3	377.356	-1.3186	11	5	266.17	0.47996	158.92	-0.38159	648.3946641435	0.31442	7.5068	-4.1937	0.51852	0.38631	0.88737 (max o	2	2648; 2649	0.38474408	0.8993604		1.0
	ZINC40309537	-8.3	363.376	-0.628	8	3	260.21	0.30871	115.29	3.7493	648.3946641435	0.31442	6.8162	-1.9973	0.44444	0.44125	0.97149 (max o	6	243; 528; 1329; 1330	-0.1657780	-0.4506764		1.0
	ZINC49604876	-8.3	354.421	-1.5748	7	3	265.15	0.33894	97.5	3.7131	648.3946641435	0.33958	7.763	-4.6375	0.36	0.22028	0.88322	1	3805	-0.2652747	0.9534661		1.0
	ZINC56874303	-8.3	406.382	-2.2357	11	5	274.25	0.4687	164.37	-3.886	648.3946641435	0.30319	8.4239	-7.3738	0.46429	0.42827	0.92152	1	4884	0.7517443	0.14937168		1.0
	ZINC70686861	-8.3	358.305	-0.8493	9	0	242.15	0.38117	110.29	-16.951	648.3946641435	0.32652	7.0375	-2.6011	0.46154	0.39216	0.93361	1	3965	-0.3862593	0.8648096		1.0
	ZINC71405136	-8.3	406.481	0.0855	7	3	287.09	0.3293	115.84	5.65	648.3946641435	0.30319	6.1027	0.282	0.60714	0.42715	0.88492	1	4044	0.74086744	0.4511644		1.0
	ZINC72320465	-8.3	445.396	-2.9563	12	4	313.9	0.45479	184.27	-0.61557	648.3946641435	0.27385	9.1445	-10.795	0.48387	0.51896	0.85745	1	1626	-0.7821330	0.45686397		1.0
	ZINC79189965	-8.3	412.413	-3.3218	10	6	293.92	0.42913	150.35	-2.4648	648.3946641435	0.29274	9.51	-11.347	0.41379	0.38287	0.93111 (max o	6	4885; 2705; 1346; 16	0.72311836	-0.3742230		1.0
	ZINC02104681	-8.2	375.332	-4.0935	9	0	278.6	0.37426	136.1	-6.9789	769.8318345753	0.31063	10.207	-13.178	0.55556	0.60638	0.91157 (max o	4	1079; 1090; 1118; 13	0.15929712	0.46362856		172.0
	ZINC11866812	-8.2	445.466	-1.8392	9	1	345.8	0.32441	144.89	-10.973	769.8318345753	0.2621	7.9528	-7.0172	0.625	0.46554	0.89329	1	1362	0.3157754	0.08624188		172.0
	ZINC02115888	-8.2	403.410	-1.8971	9	2	307.48	0.35567	133.86	-2.132	769.8318345753	0.28921	8.0107	-6.5596	0.55172	0.50794	0.9561 (max of	6	4022; 874; 1096; 198	0.13018608	-0.0398281		172.0
	ZINC08952410	-8.2	446.502	-0.7369	10	4	333.59	0.33107	168.4	-2.8293	769.8318345753	0.2621	6.8505	-2.8115	0.625	0.48986	0.90581 (max o	2	3148; 1362	0.26193738	0.06589293		172.0

Structure	Molecule Name	affinity	Total Molweight	cLogP	H-Acceptors	H-Donors	Total Surface Area	Relative PSA	Polar Surface Area	Druglikeness	Kd (nM) @ 20°C	LE from K...	LLE from Kd ...	LELP from Kd (n...	Shape Index	Molecular...	Neighbor Si...	Neig...	Neighbor	Neighbor ...	Neighbor ...	Ring Systems	frequency
	ZINC02123151	-8.2	417.436	-1.4427	9	2	321.24	0.34043	133.86	2.3899	769.8318345753	0.27957	7.5563	-5.1604	0.56667	0.51227	0.95345 (max o	7	3569; 3153; 1096; 19	0.17018801	-0.0304745		172.0
	ZINC12900450	-8.2	361.329	-2.9137	9	2	270.46	0.40435	133.86	-3.6715	769.8318345753	0.32258	9.0273	-9.0324	0.61538	0.55654	0.93686 (max o	5	4022; 3145; 775; 254	0.17405453	-0.0878095		172.0
	ZINC12899689	-8.2	361.329	-2.8186	9	2	273.22	0.40026	133.86	-3.653	769.8318345753	0.32258	8.9322	-8.7376	0.65385	0.57988	0.91731 (max o	4	3577; 2142; 2166; 29	0.21068999	-0.0953519		172.0
	ZINC02157050	-8.2	375.356	-2.2621	9	3	280.04	0.40159	144.86	0.1125	769.8318345753	0.31063	8.3757	-7.2822	0.62963	0.57302	0.96682 (max o	7	5708; 4014; 1646; 16	0.12110356	0.06886447		172.0
	ZINC02112530	-8.2	364.397	-1.408	7	1	269.21	0.38019	130.06	1.3654	769.8318345753	0.33549	7.5216	-4.1969	0.6	0.51898	0.89723 (max o	3	4017; 4009; 468	0.19711323	0.16081604		172.0
	ZINC02126056	-8.2	417.413	-2.8289	9	1	318.81	0.35187	144.89	-2.6493	769.8318345753	0.27957	8.9425	-10.119	0.56667	0.45866	0.97533 (max o	5	3575; 3567; 3148; 13	0.2934762	0.08540541		172.0
	ZINC02117005	-8.2	431.440	-2.3745	9	1	332.57	0.33731	144.89	-2.6702	769.8318345753	0.27055	8.4881	-8.7765	0.58065	0.47045	0.97642 (max o	5	3575; 3567; 3148; 27	0.28284553	0.06659013		172.0
	ZINC02152399	-8.2	361.305	-4.4409	9	1	268.03	0.41854	144.89	1.9718	769.8318345753	0.32258	10.555	-13.767	0.57692	0.54177	0.93686 (max o	4	4017; 3134; 1981; 27	0.21971008	0.12871566		172.0
	ZINC02134570	-8.2	375.356	-2.4593	9	2	284.22	0.38477	133.86	-0.45242	769.8318345753	0.31063	8.5729	-7.917	0.62963	0.56912	0.96617 (max o	8	5301; 3577; 3578; 31	0.2552963	-0.0490910		172.0
	ZINC02106054	-8.2	361.305	-4.4409	9	1	268.03	0.41854	144.89	-3.5627	769.8318345753	0.32258	10.555	-13.767	0.57692	0.52989	0.97269 (max o	8	5294; 4009; 3992; 31	0.20297809	0.14060917		172.0
	ZINC01891591	-8.2	361.329	-2.9137	9	2	270.46	0.40435	133.86	-5.1919	769.8318345753	0.32258	9.0273	-9.0324	0.61538	0.55286	0.96438 (max o	5	5301; 3561; 3132; 27	0.21903059	-0.0560038		172.0
	ZINC02117115	-8.2	417.436	-1.4088	9	2	317.95	0.34395	133.86	4.8467	769.8318345753	0.27957	7.5224	-5.0391	0.53333	0.59229	0.95345 (max o	7	4759; 4466; 4465; 40	0.06749552	-1.4722468		172.0
	ZINC02118941	-8.2	417.436	-1.4088	9	2	317.95	0.34395	133.86	4.8467	769.8318345753	0.27957	7.5224	-5.0391	0.56667	0.59229	0.95345 (max o	10	6385; 5304; 4899; 44	0.10243393	-0.0073464		172.0
	ZINC02108895	-8.2	403.410	-1.6845	9	3	302.04	0.37233	144.86	4.7799	769.8318345753	0.28921	7.7981	-5.8245	0.55172	0.59732	0.96635 (max o	9	6385; 5304; 4899; 40	0.08049776	-0.0140568		172.0
	ZINC04556770	-8.2	376.455	-6.5458	8	4	290.66	0.34749	139.78	-6.9405	769.8318345753	0.31063	12.659	-21.072	0.62963	0.51596	0.96064 (max o	4	5410; 4906; 4557; 24	-0.6741919	-0.6391269		172.0
	ZINC02160568	-8.2	447.462	-1.3683	10	2	341.71	0.3493	143.09	5.6987	769.8318345753	0.2621	7.4819	-5.2206	0.5625	0.60402	0.95983 (max o	7	6076; 5698; 5699; 49	0.00581195	0.03906892		172.0
	ZINC02130933	-8.2	417.436	-1.2983	9	2	319.45	0.34234	133.86	3.7862	769.8318345753	0.27957	7.4119	-4.6439	0.6	0.59058	0.96635 (max o	5	6390; 6073; 5698; 48	0.06904108	0.06048298		172.0
	ZINC02111570	-8.2	417.436	-1.1196	9	3	317.3	0.35443	144.86	3.8899	769.8318345753	0.27957	7.2332	-4.0047	0.6	0.5919	0.93686 (max o	2	6073; 4464	0.00712739	0.01616267		172.0
	ZINC08488911	-8.2	428.504	-2.01	10	4	332.27	0.37295	156.89	-57.266	769.8318345753	0.27957	8.1236	-7.1896	0.6	0.62293	0.9707 (max of	2	5031; 4083	0.5424132	-0.1989967		127.0
	ZINC13522362	-8.2	399.451	-3.9384	11	4	266.86	0.49858	187.08	-0.78796	769.8318345753	0.31063	10.052	-12.679	0.55556	0.53374	0.98371 (max o	4	4638; 4637; 3224; 29	0.5863307	0.23911795		67.0
	ZINC13522357	-8.2	399.451	-3.9384	11	4	266.86	0.49858	187.08	-0.78796	769.8318345753	0.31063	10.052	-12.679	0.55556	0.53374	0.98371 (max o	5	5096; 4638; 4637; 32	0.60656637	0.23050629		67.0
	ZINC13513635	-8.2	421.358	-4.449	13	3	279.97	0.54999	208.65	-19.779	769.8318345753	0.29954	10.563	-14.853	0.5	0.47588		0		-0.8839758	0.3525489		49.0
	ZINC08918337	-8.2	441.503	-2.3405	11	3	338.53	0.38762	170.96	-59.386	769.8318345753	0.27055	8.4541	-8.6508	0.54839	0.60576	0.94785 (max o	5	6020; 6018; 5866; 54	0.50940394	-0.1002039		47.0
	ZINC70665340	-8.2	396.446	0.0039	11	6	294.01	0.44175	167.25	-12.241	769.8318345753	0.29954	6.1097	0.01302	0.53571	0.42173	0.87582 (max o	2	1196; 2121	0.28608838	-0.5167858		44.0
	ZINC02159505	-8.2	361.206	-8.4657	13	4	230.21	0.69893	217.22	-28.905	769.8318345753	0.34946	14.579	-24.225	0.5	0.45058	0.92619 (max o	8	5876; 5796; 4933; 49	-0.2938902	-0.4010239		44.0
	ZINC08876761	-8.2	418.405	-4.7989	11	3	311.55	0.42269	170.8	5.9994	769.8318345753	0.27957	10.913	-17.165	0.5	0.56151	0.97533 (max o	11	4330; 3750; 2941; 29	0.08267524	-0.6170664		42.0
	ZINC12889936	-8.2	432.432	-4.3445	11	3	325.31	0.40481	170.8	6.9913	769.8318345753	0.27055	10.458	-16.058	0.48387	0.56679	0.97856 (max o	11	4330; 3750; 1808; 18	0.10294285	-0.6210843		42.0
	ZINC12889834	-8.2	432.432	-4.3445	11	3	325.31	0.40481	170.8	6.9913	769.8318345753	0.27055	10.458	-16.058	0.48387	0.56497	0.97856 (max o	9	4330; 3750; 2941; 18	0.09440201	-0.6586817		42.0
	ZINC12889643	-8.2	432.455	-2.6663	11	4	326.3	0.40662	173.76	7.4436	769.8318345753	0.27055	8.7799	-9.855	0.48387	0.55892	0.97856 (max o	9	5077; 4764; 1808; 18	0.07486424	-0.5980493		42.0
	ZINC20757808	-8.2	391.403	-2.9228	10	4	289.86	0.40526	150.9	4.722	769.8318345753	0.29954	9.0364	-9.7576	0.46429	0.5495	0.98866 (max o	11	3823; 3822; 3801; 37	-0.0149411	-0.6160517		42.0
	ZINC12889585	-8.2	375.404	-1.901	9	3	284.77	0.36651	130.67	6.6384	769.8318345753	0.31063	8.0146	-6.1197	0.48148	0.54836	0.97407 (max o	12	5165; 4668; 3823; 37	0.03722550	-0.6564323		42.0
	ZINC08585028	-8.2	401.136	-11.987	14	3	246.85	0.70557	240.5	-46.18	769.8318345753	0.33549	18.101	-35.732	0.56	0.61685	0.93348 (max o	6	3795; 3729; 3727; 29	-0.5388204	-0.4299968		41.0
	ZINC12493522	-8.2	401.136	-11.987	14	3	246.85	0.70557	240.5	-46.18	769.8318345753	0.33549	18.101	-35.732	0.56	0.6143	0.91611 (max o	5	3767; 3729; 3727; 14	-0.5416153	-0.4509382		41.0
	ZINC08585028	-8.2	402.144	-10.74	14	4	245.67	0.70416	237.67	-50.91	769.8318345753	0.33549	16.854	-32.013	0.56	0.61705	0.93348 (max o	5	3794; 3728; 3726; 14	-0.5349535	-0.4084515		41.0
	ZINC08215882	-8.2	400.172	-9.4476	13	3	251.05	0.63689	217.44	-40.879	769.8318345753	0.33549	15.561	-28.161	0.56	0.62019	0.91964 (max o	5	4387; 3471; 3055; 30	-0.7139567	0.07619062		41.0
	ZINC33979246	-8.2	400.172	-9.4476	13	3	251.05	0.63689	217.44	-40.879	769.8318345753	0.33549	15.561	-28.161	0.56	0.62269	0.94351 (max o	5	4387; 3471; 3056; 28	-0.7047327	0.09633522		41.0
	ZINC08215882	-8.2	399.164	-10.695	13	2	252.23	0.63858	220.27	-36.149	769.8318345753	0.33549	16.809	-31.88	0.56	0.61999	0.91964 (max o	5	4388; 3472; 3056; 30	-0.6854268	0.0995394		41.0
	ZINC33979246	-8.2	399.164	-10.695	13	2	252.23	0.63858	220.27	-36.149	769.8318345753	0.33549	16.809	-31.88	0.56	0.6225	0.94351 (max o	5	4388; 3472; 2853; 30	-0.6779058	0.12163336		41.0
	ZINC20612182	-8.2	410.405	-1.5043	9	3	301.14	0.35203	138.87	4.1266	769.8318345753	0.27957	7.6179	-5.3807	0.4	0.50423	0.9897 (max of	13	4471; 4328; 3414; 33	0.67656416	-0.0661835		36.0
	ZINC02136214	-8.2	380.379	-0.9369	8	2	282.29	0.32913	118.64	4.6877	769.8318345753	0.29954	7.0505	-3.1278	0.42857	0.49759	0.96519 (max o	15	4471; 4328; 4326; 36	0.6922613	-0.0511277		36.0
	ZINC20612196	-8.2	423.404	-2.1566	10	3	310.06	0.39096	161.73	6.3427	769.8318345753	0.27055	8.2702	-7.9711	0.41935	0.5079	0.97856 (max o	9	4471; 4328; 3414; 31	0.7120206	-0.0565592		36.0
	ZINC12883516	-8.2	438.482	-0.4255	9	3	332.8	0.32269	146.28	1.3218	769.8318345753	0.2621	6.5391	-1.6234	0.46875	0.50505	0.95243 (max o	7	4471; 4328; 3412; 31	0.7081994	-0.1089539		36.0
	ZINC20611675	-8.2	380.379	-0.9369	8	2	282.29	0.32913	118.64	4.6877	769.8318345753	0.29954	7.0505	-3.1278	0.42857	0.4975	0.96519 (max o	13	4471; 4328; 4326; 40	0.6890038	-0.0315861		36.0

Structure	Molecule Name	affinity	Total Molweight	cLogP	H-Acceptors	H-Donors	Total Surface Area	Relative PSA	Polar Surface Area	Druglikeness	Kd (nM) @ 20°C	LE from K...	LLE from Kd ...	LELP from Kd (n...	Shape Index	Molecular...	Neighbor Si...	Neig...	Neighbor	Neighbor ...	Neighbor ...	Ring Systems	frequency
	ZINC08791255	-8.2	437.431	-1.7983	10	2	321.16	0.35281	152.94	5.2242	769.8318345753	0.2621	7.9119	-6.8612	0.40625	0.5057	0.97948 (max o	11	5034; 4962; 4522; 40	0.74162686	-0.0100944		36.0
	ZINC02133503	-8.2	376.348	-3.3681	11	4	276.78	0.47937	173.76	4.4122	769.8318345753	0.31063	9.4817	-10.843	0.59259	0.5895	0.90952	1	6274	0.6293562	0.16113709		36.0
	ZINC08586021	-8.2	424.178	-11.354	15	3	264.95	0.71006	260.71	-42.337	769.8318345753	0.31063	17.468	-36.552	0.51852	0.47645	0.93373 (max o	6	3284; 3173; 1461; 17	-0.6254371	0.41508523		33.0
	ZINC08586021	-8.2	425.186	-10.107	15	4	263.77	0.70876	257.88	-47.067	769.8318345753	0.31063	16.22	-32.536	0.51852	0.47665	0.93373 (max o	10	3283; 3173; 2873; 17	-0.5962897	0.3928882		33.0
	ZINC03869383	-8.2	384.416	-3.7275	11	4	265.34	0.56735	212.38	-0.44329	769.8318345753	0.32258	9.8411	-11.555	0.57692	0.47164	0.96775 (max o	8	5728; 5405; 5095; 45	0.606119	0.21021399		33.0
	ZINC04090376	-8.2	372.400	-1.7332	8	1	273.91	0.31032	109.85	6.3578	769.8318345753	0.31063	7.8468	-5.5795	0.51852	0.50417	0.97658 (max o	15	3642; 3345; 3209; 32	0.1528414	-0.8535655		29.0
	ZINC04090413	-8.2	386.427	-1.2788	8	1	287.67	0.29548	109.85	6.5707	769.8318345753	0.29954	7.3924	-4.2692	0.53571	0.5148	0.96519 (max o	15	4091; 3642; 3209; 32	0.09136786	-0.8985354		29.0
	ZINC04090872	-8.2	390.439	-2.2567	8	1	280.4	0.36551	135.15	6.4005	769.8318345753	0.31063	8.3703	-7.2648	0.55556	0.50983	0.96567 (max o	17	4091; 3749; 3642; 32	0.12872589	-0.9006251		29.0
	ZINC08876246	-8.2	430.440	-3.0359	11	3	315.9	0.39497	164.97	4.6258	769.8318345753	0.27055	9.1495	-11.221	0.58065	0.5413	0.92527 (max o	5	4091; 3210; 2127; 27	0.1431927	-0.9297075		29.0
	ZINC04090438	-8.2	386.427	-1.0427	8	1	290.43	0.29267	109.85	-2.8056	769.8318345753	0.29954	7.1563	-3.481	0.60714	0.48736	0.94543 (max o	8	5464; 4632; 3749; 36	0.20495729	-0.8830755		29.0
	ZINC12501415	-8.2	361.206	-8.4657	13	4	230.21	0.69893	217.22	-28.905	769.8318345753	0.34946	14.579	-24.225	0.5	0.43314	0.91522 (max o	4	1638; 1995; 2148; 27	-0.3103716	-0.3882478		26.0
	ZINC33830037	-8.2	353.302	-2.8445	9	5	246.47	0.47008	167.58	0.27088	769.8318345753	0.33549	8.9581	-8.4788	0.6	0.41449	0.94338 (max o	14	3467; 1112; 1351; 13	-0.2251803	-0.8147297		25.0
	ZINC31155181	-8.2	367.329	-2.5688	9	4	262.38	0.42976	156.58	0.46026	769.8318345753	0.32258	8.6824	-7.9632	0.57692	0.41317	0.97791 (max o	9	3443; 3158; 1484; 17	-0.2034125	-0.7849837		25.0
	ZINC31155197	-8.2	367.329	-2.5688	9	4	262.38	0.42976	156.58	0.39255	769.8318345753	0.32258	8.6824	-7.9632	0.61538	0.41317	0.97791 (max o	8	3443; 3158; 1484; 20	-0.2357958	-0.7611736		25.0
	ZINC85341114	-8.2	389.376	-4.0467	10	4	265.43	0.45104	157.97	-2.1256	769.8318345753	0.31063	10.16	-13.027	0.44444	0.37887	0.94622 (max o	4	5674; 3707; 1700; 20	-0.6530972	0.09013641		25.0
	ZINC28538323	-8.2	390.383	-1.9707	10	5	264.25	0.44859	155.14	-3.9213	769.8318345753	0.31063	8.0843	-6.3441	0.48148	0.37688	0.93373 (max o	3	5960; 2396; 2814	-0.5509856	-0.4939949		25.0
	ZINC08740505	-8.2	401.382	1.4655	12	6	283.5	0.49531	184.16	1.3234	769.8318345753	0.28921	4.6481	5.0672	0.51724	0.33593	0.97878 (max o	15	5458; 4622; 4624; 41	-0.2269781	-0.6463084		24.0
	ZINC08740488	-8.2	415.409	1.7412	12	5	299.41	0.45864	173.16	1.4288	769.8318345753	0.27957	4.3724	6.2281	0.53333	0.34885	0.9561 (max of	13	4624; 4619; 4190; 41	-0.2169316	-0.6628714		24.0
	ZINC08964843	-8.2	401.382	1.4655	12	6	283.5	0.49531	184.16	1.3234	769.8318345753	0.28921	4.6481	5.0672	0.48276	0.31555	0.97649 (max o	12	6205; 4201; 4188; 37	-0.2464358	-0.6412869		24.0
	ZINC08740543	-8.2	385.383	1.8112	11	5	277.15	0.45939	163.93	1.3234	769.8318345753	0.29954	4.3024	6.0466	0.5	0.33348	0.97288 (max o	11	5460; 4627; 4626; 46	-0.229649	-0.7101853		24.0
	ZINC12881033	-8.2	426.400	-0.8918	9	2	316.1	0.34154	134.22	2.5282	769.8318345753	0.27055	7.0054	-3.2962	0.6129	0.4352	0.99016 (max o	12	3343; 3204; 1218; 14	0.4956087	0.43088582		24.0
	ZINC04085645	-8.2	382.347	-0.6837	8	1	286.25	0.33139	113.99	0.53295	769.8318345753	0.29954	6.7973	-2.2825	0.67857	0.41692	0.9543 (max of	11	3343; 3204; 2934; 11	0.52021545	0.38299793		24.0
	ZINC12882659	-8.2	442.399	-1.3211	10	2	327.36	0.36034	143.45	4.7361	769.8318345753	0.2621	7.4347	-5.0405	0.59375	0.41833	0.93422 (max o	6	4090; 3307; 3204; 17	0.5599193	0.3769873		24.0
	ZINC12884109	-8.2	442.399	-0.8237	10	1	330.77	0.34725	132.45	0.53295	769.8318345753	0.2621	6.9373	-3.1427	0.59375	0.378	0.92889 (max o	5	5076; 4197; 1403; 24	0.57399684	0.33973926		24.0
	ZINC13515701	-8.2	367.285	-3.3332	10	3	249.6	0.47941	154.81	-0.84954	769.8318345753	0.32258	9.4468	-10.333	0.46154	0.27744	0.89885	1	3448	-0.4436718	0.5723252		24.0
	ZINC31167087	-8.2	370.309	-1.2533	10	5	250.78	0.47269	155.14	-5.8493	769.8318345753	0.32258	7.3669	-3.8852	0.46154	0.41647	0.9615 (max of	7	6630; 4952; 3893; 35	-0.4013702	0.51992047		24.0
	ZINC12419746	-8.2	370.309	-1.2533	10	5	250.78	0.47269	155.14	-5.8493	769.8318345753	0.32258	7.3669	-3.8852	0.46154	0.4171	0.9615 (max of	8	5471; 5408; 4983; 45	-0.4365918	0.5085776		24.0
	ZINC11565587	-8.2	376.368	-2.0663	10	5	264.4	0.44066	155.05	6.1929	769.8318345753	0.31063	8.1799	-6.6519	0.44444	0.48479	0.96064 (max o	15	4912; 4908; 4896; 41	0.4204976	0.74096596		24.0
	ZINC04353345	-8.2	406.394	-2.6337	11	6	283.25	0.45758	175.28	6.4914	769.8318345753	0.28921	8.7473	-9.1065	0.44828	0.509	0.96428 (max o	15	4912; 4908; 4896; 41	0.4413624	0.74154884		24.0
	ZINC35271344	-8.2	388.455	-0.2632	8	5	281.14	0.3505	136.68	-5.3631	769.8318345753	0.31063	6.3768	-0.8473	0.51852	0.43395	0.96064 (max o	3	6315; 4821; 4396	-0.0423150	0.592657		23.0
	ZINC02125701	-8.2	388.395	-0.6759	8	0	288.34	0.30155	105.2	4.9812	769.8318345753	0.29954	6.7895	-2.2565	0.57143	0.52047	0.91204 (max o	5	331; 548; 549; 876; 1	0.32434994	0.16366617		22.0
	ZINC12896086	-8.2	388.395	-0.6759	8	0	288.34	0.30155	105.2	4.3706	769.8318345753	0.29954	6.7895	-2.2565	0.53571	0.53467	0.97288 (max o	5	477; 549; 608; 1689;	0.30612686	0.23558444		22.0
	ZINC12896081	-8.2	388.395	-0.6759	8	0	288.34	0.30155	105.2	4.3706	769.8318345753	0.29954	6.7895	-2.2565	0.53571	0.53467	0.97288 (max o	5	477; 549; 608; 1689;	0.3262051	0.22286457		22.0
	ZINC06624272	-8.2	358.369	-0.6059	7	0	266.08	0.2892	95.97	4.3706	769.8318345753	0.32258	6.7195	-1.8783	0.57692	0.54928	0.96998 (max o	8	474; 605; 866; 876; 1	0.34464228	0.16527264		22.0
	ZINC03977894	-8.2	448.379	0.5798	11	7	295.9	0.45536	186.37	1.9289	769.8318345753	0.2621	5.5338	2.2122	0.40625	0.42191	0.9665 (max of	15	4095; 175; 583; 919;	-0.7045602	0.32539046		21.0
	ZINC20465429	-8.2	447.452	-0.2085	8	3	316.81	0.24557	94.5	-1.5807	769.8318345753	0.27055	6.3221	-0.77064	0.54839	0.55983	0.95318 (max o	4	7729; 6814; 4310; 10	-0.4004227	0.13992602		21.0
	ZINC14682249	-8.2	431.440	-0.1614	9	4	308.3	0.35641	142.39	0.6376	769.8318345753	0.27055	6.275	-0.59656	0.51613	0.44016	0.97199 (max o	10	4392; 3881; 3362; 15	-0.2915267	-0.7384133		19.0
	ZINC08636396	-8.2	388.491	0.0647	7	4	288.74	0.32801	122.49	5.5865	769.8318345753	0.31063	6.0489	0.20828	0.59259	0.42961	0.93686 (max o	7	5824; 4610; 3292; 94	-0.4787691	0.3259056		17.0
	ZINC33979245	-8.2	400.172	-9.4476	13	3	251.05	0.63689	217.44	-40.879	769.8318345753	0.33549	15.561	-28.161	0.56	0.6231	0.94351 (max o	5	4387; 3471; 3055; 28	-0.7251517	0.0944213		17.0
	ZINC33979245	-8.2	399.164	-10.695	13	2	252.23	0.63858	220.27	-36.149	769.8318345753	0.33549	16.809	-31.88	0.56	0.62291	0.94351 (max o	5	4388; 3472; 3056; 28	-0.697505	0.11799825		17.0
	ZINC04042226	-8.2	365.409	-1.8794	8	3	245.3	0.46262	162.71	9.2567	769.8318345753	0.33549	7.993	-5.602	0.56	0.38598	0.91611 (max o	4	4700; 4480; 4275; 23	0.13333912	-0.1798138		17.0
	ZINC03875033	-8.2	412.442	-3.6694	9	1	267.33	0.48165	180.42	5.6501	769.8318345753	0.31063	9.783	-11.813	0.48148	0.39445	0.94302 (max o	3	6130; 4939; 4498	0.11001721	-0.0454143		17.0
	ZINC04654607	-8.2	448.379	-0.4237	11	8	295.54	0.4664	197.37	-1.9977	769.8318345753	0.2621	6.5373	-1.6166	0.5	0.38342	0.97743 (max o	12	3282; 3243; 29; 294;	0.72141784	0.04831803		14.0

Structure	Molecule Name	affinity	Total Molweight	cLogP	H-Acceptors	H-Donors	Total Surface Area	Relative PSA	Polar Surface Area	Druglikeness	Kd (nM) @ 20°C	LE from K...	LLE from Kd ...	LELP from Kd (n...	Shape Index	Molecular...	Neighbor Si...	Neig...	Neighbor	Neighbor ...	Neighbor ...	Ring Systems	frequency
	ZINC04349248	-8.2	448.379	-0.4237	11	8	295.54	0.4664	197.37	-1.9977	769.8318345753	0.2621	6.5373	-1.6166	0.5	0.38342	0.96186 (max o	12	3282; 3243; 2828; 29	0.7421354	0.04436731		14.0
	ZINC12603861	-8.2	395.478	-2.1782	9	6	318.5	0.35871	124.36	1.2785	769.8318345753	0.29954	8.2918	-7.2718	0.67857	0.60067	0.94291 (max o	5	4657; 4656; 3776; 37	0.32215497	-0.4418203		14.0
	ZINC03874669	-8.2	436.412	0.055	10	7	305.5	0.40831	177.14	-4.8731	769.8318345753	0.27055	6.0586	0.20329	0.51613	0.43296	0.95771 (max o	11	5085; 4101; 3799; 36	0.7739946	-0.2944759		13.0
	ZINC13521018	-8.2	436.412	0.055	10	7	305.5	0.40831	177.14	-4.8731	769.8318345753	0.27055	6.0586	0.20329	0.51613	0.43296	0.97642 (max o	12	5085; 4111; 3799; 36	0.79226357	-0.2973747		13.0
	ZINC03874215	-8.2	360.413	-1.5337	7	1	254.02	0.38347	127.2	11.036	769.8318345753	0.33549	7.6473	-4.5716	0.52	0.39204	0.90896 (max o	5	5346; 3178; 706; 234	0.07387315	-0.0912938		12.0
	ZINC03874215	-8.2	361.421	-1.5337	7	2	242.51	0.35421	128.81	10.469	769.8318345753	0.33549	7.6473	-4.5716	0.52	0.39112	0.90896 (max o	4	5345; 4025; 3177; 27	0.09090219	-0.1079093		12.0
	ZINC03838461	-8.2	404.422	-1.1562	10	6	302.11	0.42372	170.85	4.0393	769.8318345753	0.28921	7.2698	-3.9978	0.62069	0.55713	0.9673 (max of	5	3606; 3602; 1101; 16	-0.2146939	-0.5751336		12.0
	ZINC03840037	-8.2	366.348	-1.381	9	6	264.69	0.43436	153.78	3.7628	769.8318345753	0.32258	7.4946	-4.2811	0.57692	0.53373	0.96389 (max o	8	3606; 3602; 3595; 31	-0.2348432	-0.5715884		12.0
	ZINC08627149	-8.2	407.465	-0.5232	9	3	303.6	0.29921	111.57	6.4495	769.8318345753	0.28921	6.6368	-1.8091	0.58621	0.54989	0.95318 (max o	4	4171; 2987; 2109; 21	-0.0283983	-0.5439999		11.0
	ZINC20464465	-8.2	408.517	-1.039	8	3	306.89	0.24139	99.08	4.737	769.8318345753	0.28921	7.1526	-3.5925	0.58621	0.52025	0.93069 (max o	3	4171; 2112; 2880	0.00731309	-0.5532177		11.0
	ZINC04523267	-8.2	418.397	-1.026	9	7	280.74	0.40871	167.91	-3.0467	769.8318345753	0.27957	7.1396	-3.6699	0.36667	0.39399	0.98188 (max o	7	3470; 3364; 2049; 20	-0.5411422	0.6267568		11.0
	ZINC04523267	-8.2	418.397	-1.026	9	7	280.74	0.40871	167.91	-3.0467	769.8318345753	0.27957	7.1396	-3.6699	0.36667	0.39399	0.98188 (max o	9	6314; 4394; 3470; 33	-0.5278231	0.5950976		11.0
	ZINC04237224	-8.2	439.510	-2.5799	8	1	343.74	0.25627	94.42	3.2697	769.8318345753	0.2621	8.6935	-9.8433	0.5625	0.49211	0.95983 (max o	3	4543; 4542; 1412	-0.546948	-0.3532343		10.0
	ZINC04245684	-8.2	432.380	-0.078	10	7	289.19	0.43134	177.14	-1.9977	769.8318345753	0.27055	6.1916	-0.2883	0.45161	0.3811	0.95267 (max o	9	4107; 3464; 3226; 32	0.770102	0.09139605		10.0
	ZINC04084330	-8.2	404.466	-1.3969	8	2	284.95	0.38744	143.94	6.4109	769.8318345753	0.29954	7.5105	-4.6635	0.5	0.33153	0.96253 (max o	10	6409; 4523; 4084; 40	0.3490431	-0.6211641		10.0
	ZINC04270656	-8.2	374.463	0.307	8	1	283.56	0.27338	87.9	0.095764	769.8318345753	0.31063	5.8066	0.9883	0.59259	0.35642	0.9083 (max of	3	3661; 1151; 1152	-0.0190662	-0.2609526		10.0
	ZINC04270618	-8.2	375.384	-0.4438	10	1	276.51	0.37713	109.62	1.954	769.8318345753	0.31063	6.5574	-1.4287	0.59259	0.33349	0.93686 (max o	5	3252; 1726; 1738; 24	-0.1804996	-0.3333794		10.0
	ZINC38571036	-8.2	402.430	-1.2294	10	3	296.97	0.37314	139.62	5.2213	769.8318345753	0.28921	7.343	-4.2509	0.48276	0.48106	0.9892 (max of	4	3945; 1478; 2899; 29	-0.0794382	-0.876906		10.0
	ZINC08792085	-8.2	429.456	-1.4273	11	3	319.65	0.39424	162.48	6.9674	769.8318345753	0.27055	7.5409	-5.2755	0.51613	0.49282	0.96545 (max o	4	3945; 3082; 1478; 28	-0.0631601	-0.8663524		10.0
	ZINC08792079	-8.2	429.432	-3.1055	11	2	318.66	0.39236	159.52	6.4929	769.8318345753	0.27055	9.2191	-11.478	0.51613	0.50076	0.93956 (max o	4	3945; 3082; 2900; 14	-0.0856982	-0.8572368		10.0
	ZINC35442313	-8.2	373.519	-0.0616	6	2	293.66	0.17837	69.26	6.0996	769.8318345753	0.31063	6.1752	-0.1983	0.59259	0.49928	0.9399 (max of	8	3876; 3438; 665; 834	-0.5537523	0.07126215		10.0
	ZINC22936695	-8.2	402.561	-0.4787	7	3	314.62	0.20291	81.29	5.951	769.8318345753	0.28921	6.5923	-1.6552	0.62069	0.49201	0.93975 (max o	9	4350; 4346; 3438; 34	-0.5371018	0.03201443		10.0
	ZINC08299707	-8.2	405.545	-4.757	9	3	334.6	0.32543	102.99	2.4402	769.8318345753	0.28921	10.871	-16.448	0.62069	0.53024	0.97176 (max o	9	4483; 4163; 4162; 37	0.1794195	0.33171192		10.0
	ZINC08299703	-8.2	391.470	-3.5442	9	2	304.86	0.3392	115.82	0.0027639	769.8318345753	0.29954	9.6578	-11.832	0.64286	0.54334	0.98451 (max o	7	5014; 5012; 4164; 37	0.20105675	0.25807583		10.0
	ZINC20465429	-8.2	448.460	-2.3182	8	4	330.85	0.27759	95.7	-3.5563	769.8318345753	0.27055	8.4318	-8.5684	0.54839	0.56465	0.8973 (max of	3	7730; 6815; 5938	-0.3643066	-0.5597313		10.0
	ZINC35454665	-8.2	390.471	-0.232	8	6	282.24	0.34935	139.84	-2.1613	769.8318345753	0.31063	6.3456	-0.74686	0.51852	0.45759	0.96343 (max o	8	5628; 5497; 5276; 52	-0.3080126	0.2254931		10.0
	ZINC06143119	-8.2	432.380	-0.3621	10	6	292.84	0.41538	166.14	-3.6206	769.8318345753	0.27055	6.4757	-1.3384	0.54839	0.42904	0.95983 (max o	12	3558; 279; 365; 558;	0.20573078	0.68317187		9.0
	ZINC09033213	-8.2	420.413	0.222	9	5	301.3	0.36024	145.91	-3.6574	769.8318345753	0.27957	5.8916	0.79407	0.6	0.47511	0.93686 (max o	8	655; 829; 1144; 1586	0.39273256	0.5355251		9.0
	ZINC05439001	-8.2	427.436	-1.9082	10	2	316.79	0.36371	134.73	1.1989	769.8318345753	0.27055	8.0218	-7.053	0.58065	0.38041	0.92584 (max o	6	1181; 2079; 2081; 20	-0.6811039	-0.4694501		9.0
	ZINC04259917	-8.2	420.425	-2.459	11	1	298.52	0.46593	165.82	2.3525	769.8318345753	0.28921	8.5726	-8.5024	0.62069	0.4314	0.96682 (max o	2	1416; 1719	-0.2571848	-0.4266687		9.0
	ZINC03842023	-8.2	374.400	0.1435	10	1	275.31	0.35534	103.63	0.99667	769.8318345753	0.31063	5.9701	0.46196	0.55556	0.39447	0.9428 (max of	6	4531; 4493; 3649; 32	-0.2273520	-0.3365280		9.0
	ZINC08299441	-8.2	406.436	-4.2104	9	3	296.18	0.32636	126.46	2.2859	769.8318345753	0.28921	10.324	-14.558	0.44828	0.5205	0.96428 (max o	7	4994; 3721; 762; 174	0.03792486	0.31952137		9.0
	ZINC12605346	-8.2	372.424	0.1841	8	2	280.6	0.29287	98.82	5.2839	769.8318345753	0.31063	5.9295	0.59266	0.48148	0.5184	0.9578 (max of	7	4991; 3722; 3721; 32	0.05786480	0.26547772		9.0
	ZINC05414531	-8.2	420.440	-0.2139	9	2	310.71	0.29668	108.05	3.6415	769.8318345753	0.27957	6.3275	-0.7651	0.43333	0.53053	0.94529 (max o	5	5799; 3263; 2861; 18	0.07482819	0.32391736		9.0
	ZINC08298670	-8.2	374.462	-5.6553	7	3	294.19	0.29022	91.89	-2.2555	769.8318345753	0.31063	11.769	-18.206	0.66667	0.49579	0.96885 (max o	8	4147; 2919; 2860; 28	-0.1382429	0.01313273		9.0
	ZINC08299020	-8.2	400.525	-3.5285	8	3	322.37	0.27496	90.09	0.36709	769.8318345753	0.28921	9.6421	-12.2	0.62069	0.50363	0.97176 (max o	8	4157; 4155; 3714; 33	-0.1585314	0.05374255		9.0
	ZINC03838676	-8.2	384.526	-3.3336	7	3	328.97	0.26899	68.69	0.27097	769.8318345753	0.29954	9.4472	-11.129	0.64286	0.51667	0.97037 (max o	8	4484; 4149; 3718; 37	-0.1294732	-0.0048980		9.0
	ZINC08298158	-8.2	356.472	-5.7561	7	3	287.84	0.29662	91.89	-0.91546	769.8318345753	0.32258	11.87	-17.844	0.65385	0.49307	0.96721 (max o	9	4153; 4148; 4147; 37	-0.1103732	0.03139241		9.0
	ZINC12602706	-8.2	386.498	-5.8261	8	3	310.1	0.30758	101.12	-0.915	769.8318345753	0.29954	11.94	-19.45	0.60714	0.49273	0.97037 (max o	8	4157; 4155; 4147; 33	-0.1370636	0.06117412		9.0
	ZINC67913349	-8.2	385.344	-3.1037	10	4	275.73	0.44522	165.81	-7.8503	769.8318345753	0.31063	9.2173	-9.9915	0.62963	0.56974		0		0.12320925	0.9232852		9.0
	ZINC08298673	-8.2	374.462	-5.6553	7	3	294.19	0.29022	91.89	-2.2555	769.8318345753	0.31063	11.769	-18.206	0.66667	0.49579	0.96885 (max o	8	4148; 3330; 3276; 28	-0.1458224	0.04199736		9.0
	ZINC08298675	-8.2	402.516	-3.2328	7	3	335.32	0.2639	68.69	-1.069	769.8318345753	0.28921	9.3464	-11.178	0.65517	0.51789	0.97176 (max o	8	4484; 4155; 4149; 76	-0.1541823	0.00215406		9.0
	ZINC08299324	-8.2	388.489	-3.3577	7	3	306.46	0.25661	80.86	-0.98284	769.8318345753	0.29954	9.4713	-11.21	0.67857	0.50714	0.97037 (max o	8	4588; 4157; 4153; 11	-0.1691573	0.03396524		9.0

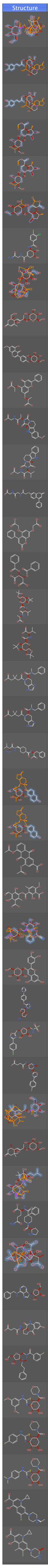

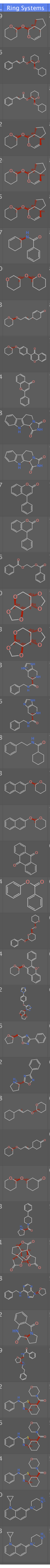

Structure	Molecule Name	affinity	Total Molweight	cLogP	H-Acceptors	H-Donors	Total Surface Area	Relative PSA	Polar Surface Area	Druglikeness	Kd (nM) @ 20°C	LE from K...	LLE from Kd ...	LELP from Kd (n...	Shape Index	Molecular...	Neighbor Si...	Neig...	Neighbor	Neighbor ...	Neighbor ...	Ring Systems	frequency
	ZINC35465903	-8.2	364.346	-3.2093	10	7	234.55	0.51887	169.3	-2.3427	769.8318345753	0.33549	9.3229	-9.5661	0.44	0.36432	0.98434 (max o	4	6338; 3197; 1611; 19	0.8345165	0.01869389		8.0
	ZINC49601722	-8.2	440.443	0.0769	10	6	314.91	0.38627	166.14	-7.5878	769.8318345753	0.27055	6.0367	0.28423	0.51613	0.405	0.96262 (max o	9	3531; 3530; 1059; 13	-0.3113570	0.53832066		8.0
	ZINC49601718	-8.2	440.443	0.0769	10	6	314.91	0.38627	166.14	-7.5878	769.8318345753	0.27055	6.0367	0.28423	0.51613	0.405	0.96262 (max o	9	3531; 3530; 3215; 30	-0.2975380	0.5537967		8.0
	ZINC04268365	-8.2	390.383	-1.9707	10	5	264.25	0.44859	155.14	-3.9213	769.8318345753	0.31063	8.0843	-6.3441	0.48148	0.37688	0.94605 (max o	3	3235; 3030; 2813	-0.5567636	-0.5360092		8.0
	ZINC04268363	-8.2	390.383	-1.9707	10	5	264.25	0.44859	155.14	-3.9213	769.8318345753	0.31063	8.0843	-6.3441	0.48148	0.37688	0.94605 (max o	3	3668; 3235; 2814	-0.5225244	-0.5445652		8.0
	ZINC35455104	-8.2	359.350	-3.6225	9	4	244.43	0.44888	148.74	-3.4303	769.8318345753	0.33549	9.7361	-10.798	0.48	0.38183	0.94678 (max o	6	6291; 5967; 3493; 34	-0.6373076	0.12852035		8.0
	ZINC03840032	-8.2	385.778	-0.7689	8	5	268.65	0.3853	141.75	3.8073	769.8318345753	0.32258	6.8825	-2.3836	0.57692	0.50888	0.91652 (max o	3	3607; 3595; 3165	-0.2133125	-0.5087767		8.0
	ZINC04556958	-8.2	356.323	-4.03	11	7	235.73	0.55869	178.53	-8.3431	769.8318345753	0.34946	10.144	-11.532	0.5	0.45757	0.94718 (max o	8	7275; 6732; 6731; 64	0.5759432	-0.4802430		8.0
	ZINC31168190	-8.2	402.438	-1.1195	9	6	287.44	0.38839	156.91	-9.0196	769.8318345753	0.29954	7.2331	-3.7374	0.5	0.46405	0.97773 (max o	6	5641; 5210; 5209; 47	0.66255	-0.1419553		8.0
	ZINC08662732	-8.2	446.407	-0.0864	10	5	308.75	0.38394	155.14	-3.4398	769.8318345753	0.2621	6.2	-0.32965	0.5625	0.44069	0.98343 (max o	12	192; 274; 279; 435; 5	0.23368634	0.6816702		7.0
	ZINC00941352	-8.2	396.350	-1.9406	8	0	292.06	0.33445	125.02	1.3459	769.8318345753	0.28921	8.0542	-6.71	0.44828	0.3484	0.96428 (max o	3	3126; 1634; 1960	0.14712383	0.82416064		7.0
	ZINC04084030	-8.2	413.409	-1.9347	10	2	291.99	0.37327	134.87	4.4784	769.8318345753	0.27957	8.0483	-6.9202	0.53333	0.37381	0.9313 (max of	4	711; 948; 2511; 2784	-0.434558	0.30991048		7.0
	ZINC04082529	-8.2	411.437	-1.192	9	2	294.49	0.33614	125.64	4.4741	769.8318345753	0.27957	7.3056	-4.2637	0.46667	0.39493	0.93686 (max o	4	4078; 1928; 2511; 27	-0.4139516	0.2996912		7.0
	ZINC02132878	-8.2	409.373	-1.8531	9	2	306.96	0.35627	133.86	-1.7804	769.8318345753	0.27957	7.9667	-6.6284	0.63333	0.50992	0.93139 (max o	3	4473; 1368; 1987	0.20550483	-0.1156367		7.0
	ZINC00942393	-8.2	368.296	-3.0365	8	0	270.06	0.3617	125.02	-2.3215	769.8318345753	0.31063	9.1501	-9.7752	0.48148	0.37917	0.89424	1	3196	-0.0688908	0.9288516		7.0
	ZINC01632637	-8.2	356.285	-3.0054	8	0	264.08	0.3814	132.86	0.2672	769.8318345753	0.32258	9.119	-9.3167	0.53846	0.39318	0.9464 (max of	3	3991; 3128; 1352	-0.8380218	-0.3022276		7.0
	ZINC38890028	-8.2	443.471	-3.1577	11	2	313.34	0.40314	144.48	1.7982	769.8318345753	0.27055	9.2713	-11.671	0.45161	0.45961	0.96971 (max o	5	1615; 1929; 1930; 20	-0.2583934	-0.8632510		7.0
	ZINC04042177	-8.2	372.392	-2.6007	9	1	259.37	0.39257	115.38	2.2103	769.8318345753	0.32258	8.7143	-8.0621	0.42308	0.40726	0.94951 (max o	5	3083; 1615; 1929; 19	-0.2698612	-0.8786893		7.0
	ZINC15676116	-8.2	412.488	-1.1744	8	2	306.75	0.24284	110.68	5.2695	769.8318345753	0.27957	7.288	-4.2007	0.5	0.6176	0.92527 (max o	5	4688; 4686; 4219; 12	0.23102225	-0.1656653		7.0
	ZINC15676116	-8.2	411.480	-0.9855	8	1	317.72	0.26898	109.43	5.7981	769.8318345753	0.27957	7.0991	-3.525	0.5	0.6176	0.92527 (max o	4	4687; 4689; 4220; 29	0.22720239	-0.2053676		7.0
	ZINC04089455	-8.2	403.521	-0.6129	7	3	320.77	0.28307	125.97	2.4898	769.8318345753	0.28921	6.7265	-2.1192	0.62069	0.53963	0.92232 (max o	4	6475; 6427; 4960; 32	0.55382353	-0.0403668		7.0
	ZINC12957072	-8.2	385.209	1.2442	6	4	234.65	0.30854	99.38	-6.4693	769.8318345753	0.36466	4.8694	3.412	0.56522	0.36597	0.96125 (max o	7	4508; 4066; 3779; 35	0.23605701	0.91248673		7.0
	ZINC04028707	-8.2	385.209	1.2442	6	4	234.65	0.30854	99.38	-6.4693	769.8318345753	0.36466	4.8694	3.412	0.56522	0.36676	0.93686 (max o	7	4508; 4066; 3779; 35	0.23188464	0.8952704		7.0
	ZINC03812842	-8.2	367.288	0.4159	8	0	260.32	0.38215	126.87	-0.88349	769.8318345753	0.31063	5.6977	1.3389	0.44444	0.20601		0		-0.7807361	0.6232108		6.0
	ZINC08740080	-8.2	418.309	-2.5777	11	3	296.7	0.47192	193.55	-3.1876	769.8318345753	0.27957	8.6913	-9.2202	0.43333	0.51307		0		-0.8714588	-0.4834844		6.0
	ZINC31157942	-8.2	427.405	-2.4776	11	6	298.59	0.44261	182.09	-10.383	769.8318345753	0.27957	8.5912	-8.8621	0.53333	0.48187	0.96343 (max o	5	3895; 841; 842; 2632	-0.5025285	0.7363494		6.0
	ZINC04097706	-8.2	449.387	-0.3513	11	8	288.97	0.43188	180.3	-8.6993	769.8318345753	0.2621	6.4649	-1.3403	0.40625	0.26804	0.95949 (max o	7	3350; 3349; 2963; 89	-0.817904	0.31176314		6.0
	ZINC04259828	-8.2	357.353	-4.2868	11	1	253.23	0.44651	129.64	-0.28992	769.8318345753	0.32258	10.4	-13.289	0.57692	0.39204	0.92124 (max o	4	1022; 1147; 1213; 14	-0.0771712	-0.3553642		6.0
	ZINC12603466	-8.2	387.387	-0.1833	9	3	265.6	0.42278	143.16	1.8095	769.8318345753	0.32258	6.2969	-0.56823	0.61538	0.45797	0.91999 (max o	3	5857; 3331; 1771	-0.3132730	-0.6214466		6.0
	ZINC12529971	-8.2	362.360	-1.9886	8	2	284.21	0.37106	112.59	1.0099	769.8318345753	0.32258	8.1022	-6.1646	0.65385	0.4334	0.97533 (max o	7	4650; 3328; 3327; 14	0.65685195	-0.3028172		6.0
	ZINC13660035	-8.2	390.471	-0.232	8	6	282.24	0.34935	139.84	-2.1613	769.8318345753	0.31063	6.3456	-0.74686	0.51852	0.46026	0.97407 (max o	5	5509; 5508; 5103; 46	-0.3972748	0.22427218		6.0
	ZINC13660208	-8.2	388.455	-0.0883	8	5	281.14	0.3505	136.68	-3.7924	769.8318345753	0.31063	6.2019	-0.28426	0.51852	0.46226	0.97407 (max o	6	5509; 5508; 5103; 37	-0.3791832	0.2133041		6.0
	ZINC35464799	-8.2	434.524	0.0313	9	6	308.42	0.36197	156.91	-5.985	769.8318345753	0.27957	6.0823	0.11196	0.46667	0.49711	0.97304 (max o	7	6618; 6336; 4813; 39	-0.1563094	0.8071499		6.0
	ZINC08968624	-8.2	430.503	-2.6152	9	3	322.06	0.32435	149.24	7.5737	769.8318345753	0.27055	8.7288	-9.6661	0.48387	0.47416	0.96545 (max o	4	601; 1126; 1477; 203	0.51606125	-0.7165533		5.0
	ZINC08552018	-8.2	424.401	-1.9238	10	3	247.15	0.47914	148.82	-1.779	769.8318345753	0.27957	8.0374	-6.8813	0.33333	0.093597	0.91333 (max o	3	5818; 726; 1761	-0.6502715	-0.3866211		5.0
	ZINC04257489	-8.2	357.369	0.0027999	9	4	257.63	0.39025	125.55	-0.31782	769.8318345753	0.32258	6.1108	0.0086796	0.57692	0.36096	0.94951 (max o	8	3229; 861; 1143; 171	-0.3988989	-0.7307718		5.0
	ZINC08299605	-8.2	409.215	-1.8451	8	2	256.68	0.36197	118.64	3.5337	769.8318345753	0.33549	7.9587	-5.4998	0.56	0.36385	0.93686 (max o	2	3272; 3271	-0.2898959	0.47794262		5.0
	ZINC20465761	-8.2	403.472	-0.1051	6	4	319.42	0.2451	83.23	0.59056	769.8318345753	0.28921	6.2187	-0.3634	0.55172	0.54575	0.9398 (max of	5	5962; 3405; 1252; 15	0.38599375	-0.2505069		5.0
	ZINC08636498	-8.2	437.515	0.2366	9	3	311.25	0.36386	138.15	5.5275	769.8318345753	0.27957	5.877	0.8463	0.5	0.4355	0.90725 (max o	5	3734; 3296; 1194; 17	-0.5641667	0.34150562		5.0
	ZINC08643911	-8.2	395.454	0.4774	7	3	273.08	0.34148	119.69	4.1235	769.8318345753	0.31063	5.6362	1.5369	0.55556	0.40472	0.97658 (max o	6	5483; 3296; 109; 176	-0.5431735	0.34008676		5.0
	ZINC08636496	-8.2	420.532	0.273	8	3	301.55	0.32101	122.93	6.1014	769.8318345753	0.28921	5.8406	0.94395	0.55172	0.43759	0.91838 (max o	8	5824; 4610; 3296; 32	-0.5218587	0.33376694		5.0
	ZINC00597112	-8.2	360.408	-2.754	6	2	263.26	0.21408	71.05	0.95726	769.8318345753	0.32258	8.8676	-8.5374	0.53846	0.35483	0.8412	1	1633	0.74008757	-0.6128400		5.0
	ZINC00587541	-8.2	360.408	-2.1145	6	2	242.11	0.1921	83.22	1.1974	769.8318345753	0.32258	8.2281	-6.5549	0.46154	0.3395	0.95857	1	1962	0.9451078	-0.1885883		5.0

Structure	Molecule Name	affinity	Total Molweight	cLogP	H-Acceptors	H-Donors	Total Surface Area	Relative PSA	Polar Surface Area	Druglikeness	Kd (nM) @ 20°C	LE from K...	LLE from Kd ...	LELP from Kd (n...	Shape Index	Molecular...	Neighbor Si...	Neig...	Neighbor	Neighbor ...	Neighbor ...	Ring Systems	frequency
	ZINC03840097	-8.2	447.554	-1.8296	9	6	342.66	0.32467	158.36	4.9028	769.8318345753	0.2621	7.9432	-6.9806	0.53125	0.52628	0.94215 (max o	4	6090; 882; 2006; 200	-0.515411	-0.1998889		5.0
	ZINC08624290	-8.2	363.412	0.2019	8	4	270.24	0.32841	111.13	3.3315	769.8318345753	0.32258	5.9117	0.62589	0.61538	0.5272	0.93686 (max o	4	2106; 2116; 2151; 25	0.2665767	-0.4045334		5.0
	ZINC03869764	-8.2	448.379	-0.0011999	11	7	297.4	0.45306	186.37	-3.6679	769.8318345753	0.2621	6.1148	-0.0045781	0.40625	0.42752	0.95465 (max o	8	4260; 3174; 1133; 14	-0.707826	0.38088807		5.0
	ZINC20464750	-8.2	385.847	-0.1189	8	4	277.91	0.30841	103.29	3.078	769.8318345753	0.32258	6.2325	-0.36859	0.53846	0.54803	0.92064 (max o	5	5549; 5139; 1866; 18	-0.3608843	-0.0758026		5.0
	ZINC62227202	-8.2	352.318	-3.2288	9	5	244.63	0.46482	155.3	-0.044643	769.8318345753	0.33549	9.3424	-9.6243	0.52	0.40478	0.94351 (max o	4	3956; 3955; 2312; 26	0.56628627	-0.4232406		5.0
	ZINC01646904	-8.2	419.417	-1.766	12	3	298.49	0.38655	161.33	-1.1402	769.8318345753	0.27957	7.8796	-6.3168	0.56667	0.53607	0.96092 (max o	3	3557; 3130; 2347	0.5392594	-0.6837392		5.0
	ZINC05273905	-8.2	351.405	-0.5014	6	3	271.93	0.29526	112.66	2.9212	769.8318345753	0.32258	6.615	-1.5543	0.57692	0.46679	0.96438 (max o	5	6693; 3999; 3804; 32	0.29536211	0.74270815		5.0
	ZINC20463369	-8.2	416.564	-0.6805	7	1	317.77	0.16052	81.06	4.1882	769.8318345753	0.27957	6.7941	-2.4341	0.53333	0.58926	0.94501 (max o	10	5526; 4723; 3387; 33	-0.1690728	-0.2277196		5.0
	ZINC03871970	-8.2	382.400	-2.5815	10	2	258.21	0.59645	203.58	12.827	769.8318345753	0.33549	8.6951	-7.6948	0.52	0.34527	0.97118 (max o	4	5322; 4968; 4187; 36	-0.3758815	0.3000893		5.0
	ZINC08643668	-8.2	419.471	-0.7077	7	4	312.82	0.25075	104.63	-0.90707	769.8318345753	0.27957	6.8213	-2.5314	0.7	0.50241	0.94251 (max o	6	6755; 5017; 4169; 33	0.16053744	-0.4758276		5.0
	ZINC20504122	-8.2	407.557	-0.9659	7	5	317.25	0.34279	123.69	4.5934	769.8318345753	0.29954	7.0795	-3.2246	0.53571	0.41762	0.89505 (max o	4	6273; 5944; 5575; 51	0.48166716	0.23506035		5.0
	ZINC20465899	-8.2	435.930	-0.5529	8	5	338.71	0.29736	108.15	1.8554	769.8318345753	0.27957	6.6665	-1.9777	0.56667	0.56059	0.94768 (max o	3	3855; 806; 2600	0.2874963	-0.2562925		4.0
	ZINC08299554	-8.2	391.450	-5.4019	10	3	291.29	0.35542	131.39	5.024	769.8318345753	0.29954	11.516	-18.034	0.46429	0.47694	0.94883 (max o	6	483; 592; 754; 938; 1	-0.0557757	0.30709192		4.0
	ZINC72320460	-8.2	431.416	-0.8675	9	4	291.67	0.3866	156.58	-1.1474	769.8318345753	0.27055	6.9811	-3.2064	0.3871	0.27352	0.97199 (max o	3	1071; 2326; 2327	-0.7793987	-0.5192874		4.0
	ZINC08740068	-8.2	373.368	-0.8314	10	5	265.98	0.4309	141.23	2.3159	769.8318345753	0.31063	6.945	-2.6765	0.48148	0.40116	0.93069 (max o	5	4693; 3376; 1469; 17	0.30843177	-0.516009		4.0
	ZINC67912737	-8.2	388.368	-1.827	10	4	263.15	0.45024	151.98	-4.331	769.8318345753	0.31063	7.9406	-5.8815	0.48148	0.36725	0.93058 (max o	3	4436; 1336; 1582	-0.7790284	-0.3867074		4.0
	ZINC05414710	-8.2	409.466	-1.2549	10	2	290.19	0.37455	137.16	7.3056	769.8318345753	0.29954	7.3685	-4.1894	0.46429	0.5235	0.97288 (max o	2	4128; 1437	0.02116330	0.33045444		4.0
	ZINC04556883	-8.2	400.307	-3.8839	12	3	262.82	0.51275	163.91	-12.61	769.8318345753	0.31063	9.9975	-12.503	0.55556	0.31092	0.92418 (max o	3	584; 746; 1731	0.09746296	-0.9769174		4.0
	ZINC05037522	-8.2	356.326	-1.7633	9	4	245.13	0.43014	134.91	-5.757	769.8318345753	0.33549	7.8769	-5.256	0.48	0.41551	0.94678 (max o	5	3676; 3677; 3556; 32	0.4562906	0.30583292		4.0
	ZINC10385344	-8.2	395.458	-0.1465	7	3	307.72	0.27151	110.86	2.2346	769.8318345753	0.28921	6.2601	-0.50655	0.62069	0.52259	0.96428 (max o	3	2123; 2133; 2897	-0.6845743	0.51412165		4.0
	ZINC08765291	-8.2	381.431	-0.4904	7	3	295.46	0.28278	110.86	2.2489	769.8318345753	0.29954	6.604	-1.6372	0.64286	0.52337	0.96253 (max o	3	2910; 2123; 2133	-0.6959661	0.5476242		4.0
	ZINC20464750	-8.2	386.855	-2.2286	8	5	291.95	0.34167	104.49	0.15743	769.8318345753	0.32258	8.3422	-6.9086	0.53846	0.55021	0.90033 (max o	3	5550; 5140; 2226	0.6142254	0.38487214		4.0
	ZINC13507070	-8.2	356.326	-0.7789	9	5	254.28	0.42685	145.91	-5.8493	769.8318345753	0.33549	6.8925	-2.3217	0.56	0.40945	0.8996	1	3645	-0.8548273	-0.0827728		4.0
	ZINC35455209	-8.2	418.397	0.8232	9	6	298.13	0.37447	156.91	-4.0044	769.8318345753	0.27957	5.2904	2.9445	0.5	0.42543	0.95053 (max o	7	3894; 3450; 2257; 22	-0.8146781	0.1316833		4.0
	ZINC13376175	-8.2	434.392	-2.3262	12	7	292.99	0.48363	187.76	-4.801	769.8318345753	0.27957	8.4398	-8.3206	0.53333	0.45857	0.96092 (max o	6	969; 970; 1605; 2284;	0.5901225	0.4700682		4.0
	ZINC13376173	-8.2	434.392	-2.3262	12	7	292.99	0.48363	187.76	-4.801	769.8318345753	0.27957	8.4398	-8.3206	0.53333	0.45857	0.96092 (max o	6	969; 970; 2284; 2285;	0.6260266	0.45593703		4.0
	ZINC72324503	-8.2	358.325	-1.2306	8	1	253.15	0.38673	121.83	0.11443	769.8318345753	0.32258	7.3442	-3.8148	0.57692	0.39474	0.94326 (max o	3	4451; 3979; 2329	-0.8737473	0.23270516		4.0
	ZINC20462838	-8.2	444.550	-2.962	8	1	359.13	0.2572	108.25	5.613	769.8318345753	0.2621	9.0756	-11.301	0.5625	0.61634	0.92863 (max o	6	6496; 4711; 4712; 42	6.335091E-	-0.1750461		4.0
	ZINC35457933	-8.2	400.810	1.2997	8	5	282.47	0.35961	144.52	0.48888	769.8318345753	0.31063	4.8139	4.184	0.40741	0.33554	0.97149 (max o	3	3931; 3503; 2660	-0.8435557	0.42457443		4.0
	ZINC38571036	-8.2	403.438	-1.4183	10	4	286	0.34909	140.87	4.5684	769.8318345753	0.28921	7.5319	-4.904	0.48276	0.48106	0.9892 (max of	3	3944; 3522; 2670	-0.7115108	0.17538331		4.0
	ZINC67902761	-8.2	404.367	-2.6504	11	5	267.41	0.49205	172.21	-5.6455	769.8318345753	0.29954	8.764	-8.8482	0.46429	0.36295	0.94573	1	2682	-0.8363086	-0.1170715		4.0
	ZINC67902761	-8.2	403.359	-4.7264	11	4	268.59	0.49428	175.04	-5.9792	769.8318345753	0.29954	10.84	-15.779	0.46429	0.36295	0.94573	1	2683	-0.1887051	0.9757542		4.0
	ZINC02038927	-8.2	423.452	-5.2627	13	5	297.18	0.46214	219.64	1.2977	769.8318345753	0.27957	11.376	-18.824	0.6	0.61616	0.97533 (max o	3	4931; 4050; 3129	0.57732075	0.7918004		4.0
	ZINC03838789	-8.2	368.432	-1.1804	9	6	280.14	0.4104	153.78	-0.27861	769.8318345753	0.32258	7.294	-3.6592	0.61538	0.5642	0.96196 (max o	4	4097; 3596; 2100; 27	-0.2059033	-0.4666648		4.0
	ZINC03838769	-8.2	368.432	-1.2755	9	6	277.38	0.41449	153.78	-2.5535	769.8318345753	0.32258	7.3891	-3.954	0.57692	0.54234	0.91999 (max o	4	4097; 3596; 2100; 27	-0.2097030	-0.4882761		4.0
	ZINC03839797	-8.2	418.425	-3.1607	10	2	310.33	0.36384	137.1	4.8824	769.8318345753	0.27957	9.2743	-11.306	0.66667	0.50331	0.96832 (max o	3	4045; 3599; 1674	-0.3545616	-0.2868562		4.0
	ZINC04237153	-8.2	387.435	-1.6291	7	0	277.06	0.32794	118.22	5.0114	769.8318345753	0.31063	7.7427	-5.2444	0.59259	0.44836	0.96343 (max o	2	4100; 3660	-0.8679106	-0.4762599		4.0
	ZINC35363827	-8.2	388.368	-1.827	10	4	263.15	0.45024	151.98	-6.054	769.8318345753	0.31063	7.9406	-5.8815	0.48148	0.35896	0.94605 (max o	3	4398; 3636; 2933	-0.7587694	0.26783442		4.0
	ZINC12871773	-8.2	388.368	-1.827	10	4	263.15	0.45024	151.98	-6.054	769.8318345753	0.31063	7.9406	-5.8815	0.48148	0.35896	0.94605 (max o	3	4398; 3636; 3061	-0.7680117	0.28650922		4.0
	ZINC20504122	-8.2	408.565	-0.8966	7	6	305.74	0.31805	125.3	4.3523	769.8318345753	0.29954	7.0102	-2.9933	0.53571	0.48126	0.88737 (max o	4	6272; 5574; 645; 125	-0.6741833	0.70035475		4.0
	ZINC20760298	-8.2	401.461	-0.0253	8	3	287.81	0.30815	107.97	4.3334	769.8318345753	0.28921	6.1389	-0.08748	0.48276	0.28336	0.95642 (max o	3	6841; 4332; 3420	0.8275118	0.40111497		4.0
	ZINC31165580	-8.2	426.416	-1.3949	10	6	299.06	0.40674	166.14	-8.9951	769.8318345753	0.27957	7.5085	-4.9894	0.5	0.455	0.96092 (max o	5	6615; 5191; 4375; 43	-0.8093534	-0.0166298		4.0
	ZINC35442302	-8.2	375.466	-0.5102	6	3	275.65	0.21872	78.05	3.5603	769.8318345753	0.31063	6.6238	-1.6424	0.62963	0.44269	0.93686 (max o	5	4346; 3485; 3437; 19	-0.4856190	0.08585932		4.0

Structure	Molecule Name	affinity	Total Molweight	cLogP	H-Acceptors	H-Donors	Total Surface Area	Relative PSA	Polar Surface Area	Druglikeness	Kd (nM) @ 20°C	LE from K...	LLE from Kd ...	LELP from Kd (n...	Shape Index	Molecular...	Neighbor Si...	Neig...	Neighbor	Neighbor ...	Neighbor ...	Ring Systems	frequency
	ZINC35465285	-8.2	431.369	-4.62	12	6	288.83	0.50521	198.43	0.77107	769.8318345753	0.27957	10.734	-16.525	0.56667	0.44315	0.95837 (max o	6	4814; 4385; 4384; 35	0.28952906	-0.6629106		4.0
	ZINC62227222	-8.2	393.371	-3.2184	10	5	279.75	0.44722	164.17	4.1929	769.8318345753	0.29954	9.332	-10.744	0.53571	0.44317	0.95148 (max o	4	3955; 3534; 2312; 26	0.54911554	-0.4105843		4.0
	ZINC72320493	-8.2	368.337	-0.2243	9	4	265.68	0.40831	142.75	-1.8996	769.8318345753	0.32258	6.3379	-0.69533	0.57692	0.39767	0.94326 (max o	3	4448; 4449; 3975	-0.4472416	0.83863425		4.0
	ZINC03839811	-8.2	443.503	-2.3126	9	3	319.73	0.38905	159.73	6.962	769.8318345753	0.27055	8.4262	-8.5477	0.6129	0.495	0.95549 (max o	3	4925; 557; 750	-0.4394712	-0.3810118		3.0
	ZINC13551787	-8.2	449.387	-0.3513	11	8	288.97	0.43188	180.3	-8.6993	769.8318345753	0.2621	6.4649	-1.3403	0.40625	0.27103	0.95709 (max o	7	3349; 3350; 897; 972	-0.8001578	0.30263382		3.0
	ZINC72326191	-8.2	411.389	0.5636	9	1	307.18	0.34628	126.35	1.417	769.8318345753	0.27957	5.55	2.0159	0.56667	0.50679	0.97975 (max o	3	1072; 3119; 2699	-0.5475066	-0.3334404		3.0
	ZINC72326192	-8.2	411.389	0.5636	9	1	307.18	0.34628	126.35	1.417	769.8318345753	0.27957	5.55	2.0159	0.56667	0.50679	0.97975 (max o	3	1073; 1627; 3118	-0.5359357	-0.2971063		3.0
	ZINC20763233	-8.2	410.405	-1.3872	9	1	311.36	0.32091	120.36	6.1904	769.8318345753	0.27957	7.5008	-4.9619	0.56667	0.52705	0.90055 (max o	3	3466; 3152; 1265	0.05741255	-0.2837526		3.0
	ZINC08636504	-8.2	448.542	-0.2335	9	4	320.31	0.38747	160.45	6.2543	769.8318345753	0.27055	6.3471	-0.86305	0.51613	0.41693	0.94232 (max o	3	6988; 1195; 1465	0.41483957	0.11155196		3.0
	ZINC03839332	-8.2	430.459	-1.8392	10	3	307.19	0.34272	128.28	6.9465	769.8318345753	0.27055	7.9528	-6.7979	0.54839	0.39435	0.97856	1	2377	-0.2471562	0.925257		3.0
	ZINC12603952	-8.2	421.492	-0.8368	9	4	320.02	0.30857	120.36	2.7579	769.8318345753	0.27957	6.9504	-2.9932	0.66667	0.57166	0.94804 (max o	4	5066; 618; 1496; 215	0.25597084	-0.3528546		3.0
	ZINC03839258	-8.2	373.412	-1.3591	9	2	266.04	0.33472	103.75	7.1954	769.8318345753	0.31063	7.4727	-4.3752	0.48148	0.30736	0.94573 (max o	9	3818; 3164; 430; 555	-0.5729247	-0.0666765		3.0
	ZINC00644887	-8.2	355.353	0.9373	9	3	256.53	0.39169	122.39	1.8667	769.8318345753	0.32258	5.1763	2.9056	0.57692	0.28996	0.9464 (max of	4	2824; 1956; 1957; 23	0.6800198	0.21993038		3.0
	ZINC08643706	-8.2	390.462	-2.8604	9	3	294.29	0.30358	117.32	-0.66558	769.8318345753	0.29954	8.974	-9.5493	0.64286	0.58438	0.88589 (max o	2	4604; 2119	0.8317824	-0.4030125		3.0
	ZINC20463250	-8.2	401.505	-0.7733	7	1	326.84	0.23892	85.19	4.534	769.8318345753	0.28921	6.8869	-2.6738	0.55172	0.5916	0.94251 (max o	9	6763; 4211; 3761; 37	-0.0277333	-0.2061031		3.0
	ZINC20463250	-8.2	400.497	-0.9049	7	0	312.8	0.20476	83.99	9.3194	769.8318345753	0.28921	7.0185	-3.1289	0.55172	0.59549	0.934 (max of 6	6	6764; 3385; 640; 100	-0.1410976	-0.1755786		3.0
	ZINC04222680	-8.2	423.242	-1.2049	8	1	270.54	0.31419	109.85	-1.0242	769.8318345753	0.32258	7.3185	-3.7352	0.57692	0.45984	0.92368 (max o	5	3656; 442; 1136; 243	-0.1709288	0.3471257		3.0
	ZINC02575132	-8.2	367.404	-0.8471	7	4	278.28	0.3356	132.89	2.9327	769.8318345753	0.31063	6.9607	-2.727	0.59259	0.47862	0.93686 (max o	2	6693; 4024	0.26636738	0.79415584		3.0
	ZINC05415594	-8.2	442.515	-3.4712	11	3	327.35	0.42294	176.28	6.6908	769.8318345753	0.27957	9.5848	-12.416	0.46667	0.63482	0.88255 (max o	2	3697; 2323	-0.8024888	-0.1192134		3.0
	ZINC05415683	-8.2	394.494	-4.4109	9	3	297.87	0.3245	126.46	-0.44346	769.8318345753	0.29954	10.525	-14.726	0.46429	0.50631	0.95983 (max o	4	4574; 3691; 600; 208	-0.0732879	0.19842947		3.0
	ZINC05923208	-8.2	390.387	0.8402	8	6	280.38	0.35167	139.84	-4.3466	769.8318345753	0.29954	5.2734	2.805	0.57143	0.46085	0.9678 (max of	7	4139; 4140; 3706; 33	-0.4922731	0.7765865		3.0
	ZINC08624414	-8.2	352.361	-0.219	7	4	251.27	0.33908	107.89	0.34673	769.8318345753	0.33549	6.3326	-0.65279	0.6	0.51779	0.91611 (max o	7	5821; 4184; 4170; 41	0.3398163	-0.4211865		3.0
	ZINC12603519	-8.2	366.388	-0.4563	7	4	265.03	0.32147	107.89	1	769.8318345753	0.32258	6.5699	-1.4145	0.65385	0.52693	0.93367 (max o	3	5519; 5451; 4613	-0.3926226	-0.2672949		3.0
	ZINC20464581	-8.2	392.502	-5.3941	9	5	294.47	0.30332	107.25	6.0521	769.8318345753	0.29954	11.508	-18.008	0.57143	0.44491	0.94291 (max o	10	6848; 5955; 5625; 55	-0.0849329	0.529073		3.0
	ZINC22922653	-8.2	376.503	-4.542	8	4	289.38	0.26339	87.02	6.3292	769.8318345753	0.31063	10.656	-14.622	0.55556	0.43768	0.94291 (max o	10	6848; 5953; 5955; 55	-0.0962362	0.51126814		3.0
	ZINC20464581	-8.2	391.494	-3.4163	9	4	305.44	0.32835	106	6.2088	769.8318345753	0.29954	9.5299	-11.405	0.57143	0.46046	0.94291 (max o	9	6849; 5956; 5625; 55	-0.0693130	0.4956133		3.0
	ZINC22922653	-8.2	375.495	-2.5642	8	3	300.35	0.29029	85.77	6.465	769.8318345753	0.31063	8.6778	-8.2547	0.55556	0.45432	0.94291 (max o	10	6849; 5956; 5954; 55	-0.0517632	0.46949273		3.0
	ZINC40313259	-8.2	374.351	-2.8882	7	1	274.51	0.31332	114.29	3.7179	769.8318345753	0.29954	9.0018	-9.6421	0.46429	0.40224	0.95148 (max o	4	6036; 3525; 2305; 26	0.56665546	-0.2690239		3.0
	ZINC59487917	-8.2	440.927	-3.5869	8	3	312.08	0.35395	147.1	7.5816	769.8318345753	0.28921	9.7005	-12.402	0.44828	0.49833	0.95642 (max o	5	7448; 6904; 6667; 63	0.58753985	0.35482776		3.0
	ZINC35464951	-8.2	391.482	-0.7932	6	3	283.97	0.29676	113.35	-0.82856	769.8318345753	0.29954	6.9068	-2.6481	0.46429	0.37458	0.9678 (max of	4	4410; 3934; 411; 843	-0.0910073	0.42981467		2.0
	ZINC13513942	-8.2	443.419	-8.3663	13	6	324.07	0.50853	212.93	4.3321	769.8318345753	0.2621	14.48	-31.921	0.625	0.57094	0.98148 (max o	5	624; 720; 900; 2956;	0.8429839	0.27862743		2.0
	ZINC31158618	-8.2	391.482	-0.6403	6	3	280.75	0.30016	113.35	-3.3875	769.8318345753	0.29954	6.7539	-2.1376	0.42857	0.388	0.9678 (max of	4	4410; 4368; 3459; 65	-0.1666973	0.43122372		2.0
	ZINC02124019	-8.2	426.408	-3.6466	11	3	312.98	0.39456	154.74	4.9479	769.8318345753	0.27055	9.7602	-13.478	0.54839	0.51019	0.97856 (max o	2	694; 2573	0.5880057	-0.5933086		2.0
	ZINC04222687	-8.2	428.444	-1.7622	9	1	305.09	0.38218	148.29	6.5871	769.8318345753	0.27957	7.8758	-6.3032	0.56667	0.46687	0.96832 (max o	5	353; 442; 719; 928; 1	-0.2079014	0.37022835		2.0
	ZINC05119406	-8.2	418.397	0.2774	9	6	298.13	0.37447	156.91	-3.3049	769.8318345753	0.27957	5.8362	0.99224	0.6	0.44836	0.95837 (max o	4	3468; 926; 1734; 246	0.4580897	0.42982486		2.0
	ZINC20411997	-8.2	410.405	-0.7331	9	3	305.16	0.34739	138.87	5.0647	769.8318345753	0.27957	6.8467	-2.6222	0.6	0.53764	0.94543 (max o	5	469; 807; 1088; 1653	0.16279669	-0.2530788		2.0
	ZINC31167042	-8.2	410.374	-1.0226	10	5	284.26	0.42771	162.98	-4.2008	769.8318345753	0.28921	7.1362	-3.5358	0.44828	0.45734		0		0.89801544	0.4325973		2.0
	ZINC12603431	-8.2	389.407	-0.7006	9	3	291.47	0.35122	125.13	4.8791	769.8318345753	0.29954	6.8142	-2.3389	0.60714	0.5597	0.9428 (max of	2	1515; 2117	0.5311174	-0.3794242		2.0
	ZINC31156476	-8.2	356.326	-0.6267	9	5	254.28	0.42685	145.91	-4.3551	769.8318345753	0.33549	6.7403	-1.868	0.6	0.4809	0.90236 (max o	5	493; 531; 851; 1060;	-0.2876176	0.59187335		2.0
	ZINC03839430	-8.2	436.556	-0.2545	8	2	305.53	0.39286	152.36	8.5528	769.8318345753	0.28921	6.3681	-0.87998	0.55172	0.42541	0.93686 (max o	6	4039; 3163; 189; 166	-0.6744931	-0.0931462		2.0
	ZINC04260902	-8.2	374.396	-0.6989	9	1	279.25	0.33415	96.73	-1.1151	769.8318345753	0.31063	6.8125	-2.2499	0.66667	0.381	0.98141 (max o	4	449; 1419; 1725; 206	-0.2678904	-0.1897644		2.0
	ZINC08300704	-8.2	410.454	-1.8172	11	2	288.95	0.41412	150.05	6.3333	769.8318345753	0.29954	7.9308	-6.0666	0.5	0.52101	0.97288 (max o	6	4128; 3257; 3256; 93	0.05992735	0.34284037		2.0
	ZINC04529117	-8.2	355.353	0.9373	9	3	256.53	0.39169	122.39	1.8667	769.8318345753	0.32258	5.1763	2.9056	0.57692	0.27801	0.93686 (max o	4	1956; 1957; 2338; 27	0.68170595	0.24223827		2.0

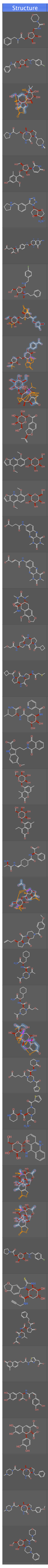

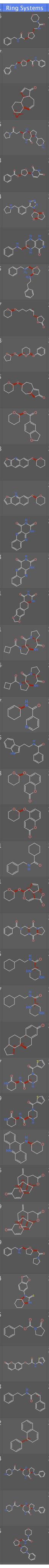

Structure	Molecule Name	affinity	Total Molweight	cLogP	H-Acceptors	H-Donors	Total Surface Area	Relative PSA	Polar Surface Area	Druglikeness	Kd (nM) @ 20°C	LE from K...	LLE from Kd ...	LELP from Kd (n...	Shape Index	Molecular...	Neighbor Si...	Neig...	Neighbor	Neighbor ...	Neighbor ...	Ring Systems	frequency
	ZINC12603603	-8.2	407.465	-0.6442	9	3	305.1	0.29774	111.57	4.1892	769.83183457753	0.28921	6.7578	-2.2274	0.62069	0.61528	0.90561 (max o	2	5454; 3288	0.43406895	-0.3987486		2.0
	ZINC12603729	-8.2	375.419	-0.3471	6	4	276.8	0.24725	95.4	-1.5145	769.83183457753	0.31063	6.4607	-1.1174	0.66667	0.45088	0.92739 (max o	7	6755; 5025; 5017; 46	0.09897264	-0.5130617		2.0
	ZINC04097928	-8.2	364.436	0.0565	6	5	239.12	0.32845	118.22	-2.8615	769.83183457753	0.32258	6.0571	0.17515	0.34615	0.30202	0.90502 (max o	2	4985; 2173	-0.8612713	0.1955229		2.0
	ZINC22922391	-8.2	393.574	-3.6406	7	3	329.14	0.24251	61.53	6.2872	769.83183457753	0.29954	9.7542	-12.154	0.57143	0.46552	0.91561 (max o	7	6284; 4401; 3874; 18	-0.1787752	0.58930725		2.0
	ZINC31160371	-8.2	408.362	-0.6866	11	4	285.1	0.43294	154.86	0.32355	769.83183457753	0.28921	6.8002	-2.374	0.51724	0.42334	0.9561 (max of	4	5606; 5196; 3050; 22	0.41564074	-0.6467563		2.0
	ZINC15709224	-8.2	358.444	-5.1498	8	2	276.39	0.33232	94.14	-2.0251	769.83183457753	0.32258	11.263	-15.964	0.53846	0.37432	0.84324	1	2437	-0.1604704	-0.4083784		2.0
	ZINC13513943	-8.2	443.419	-8.3663	13	6	324.07	0.50853	212.93	4.3321	769.83183457753	0.2621	14.48	-31.921	0.625	0.58386	0.98148 (max o	5	624; 720; 900; 2555;	0.84010565	0.2361518		2.0
	ZINC20465827	-8.2	390.497	-1.4122	5	4	311.71	0.1974	70.74	-2.4063	769.83183457753	0.29954	7.5258	-4.7146	0.60714	0.5012	0.9308 (max of	4	7056; 4317; 3395; 25	-0.3951414	-0.6621245		2.0
	ZINC31163360	-8.2	445.483	-2.0251	10	4	327.72	0.37459	165.81	-2.0398	769.83183457753	0.27055	8.1387	-7.485	0.51613	0.62779	0.96739 (max o	5	4836; 4810; 3072; 30	0.18111554	0.36109537		2.0
	ZINC35465289	-8.2	431.369	-4.62	12	6	288.83	0.50521	198.43	0.77107	769.83183457753	0.27957	10.734	-16.525	0.56667	0.44536	0.95837 (max o	6	4814; 4385; 4384; 35	0.26172134	-0.6839298		2.0
	ZINC49181196	-8.2	430.492	-0.5521	9	6	296.67	0.37631	156.91	-2.4223	769.83183457753	0.27957	6.6657	-1.9748	0.43333	0.40838	0.96092 (max o	3	5660; 5659; 2674	0.653447	-0.5839885		2.0
	ZINC49601572	-8.2	366.365	0.7334	8	4	256.94	0.37145	125.68	-17.662	769.83183457753	0.32258	5.3802	2.2735	0.53846	0.39099	0.97269 (max o	3	1938; 2675; 2676	-0.1043663	-0.89482		2.0
	ZINC15222553	-8.2	407.374	0.2351	10	4	283.59	0.41426	143.87	-4.1473	769.83183457753	0.28921	5.8785	0.8129	0.44828	0.25198	0.95099 (max o	3	3368; 2692; 2694	0.84990644	0.06027118		2.0
	ZINC15222553	-8.2	408.382	-1.7427	10	5	272.62	0.39069	145.12	-4.341	769.83183457753	0.28921	7.8563	-6.0257	0.44828	0.25198	0.95099 (max o	5	3369; 2025; 2026; 26	-0.6416955	-0.671283		2.0
	ZINC01228678	-8.2	356.317	-1.7736	10	1	254.51	0.42018	135.63	0.51167	769.83183457753	0.32258	7.8872	-5.4981	0.57692	0.40643	0.91693	1	2710	0.7270719	0.6828701		2.0
	ZINC00723627	-8.2	354.301	-2.0258	10	1	253.49	0.42187	135.63	0.365	769.83183457753	0.32258	8.1394	-6.28	0.57692	0.3349	0.91693	1	2713	0.7249596	0.68438303		2.0
	ZINC02126220	-8.2	375.380	0.5316	9	1	261.3	0.33555	97.41	3.1899	769.83183457753	0.31063	5.582	1.7113	0.37037	0.33473	0.96617 (max o	3	4018; 3576; 3149	0.9486822	0.00945711		2.0
	ZINC03839326	-8.2	378.427	-1.5509	9	2	268.86	0.34286	108.05	5.9081	769.83183457753	0.31063	7.6645	-4.9927	0.55556	0.31944	0.97407 (max o	5	3598; 898; 2752; 200	-0.5467191	-0.0428321		2.0
	ZINC03839416	-8.2	394.494	-0.9029	8	2	276.35	0.36067	124.12	6.0714	769.83183457753	0.31063	7.0165	-2.9066	0.55556	0.34286	0.97407 (max o	5	4922; 4921; 4487; 20	-0.5996505	-0.0379506		2.0
	ZINC03840061	-8.2	390.438	-0.9902	9	5	295.16	0.38786	154.64	5.1601	769.83183457753	0.29954	7.1038	-3.3057	0.53571	0.51199	0.96519 (max o	4	3603; 3167; 2761; 70	-0.4732466	-0.1391407		2.0
	ZINC04042470	-8.2	380.355	-2.2118	8	2	286.74	0.35966	134.38	2.8782	769.83183457753	0.29954	8.3254	-7.384	0.5	0.52643	0.93379 (max o	2	3127; 255	0.62690914	0.73162836		2.0
	ZINC04074052	-8.2	366.365	0.0247	8	4	255.37	0.37373	125.68	-5.4909	769.83183457753	0.32258	6.0889	0.07657	0.46154	0.28536	0.89688 (max o	4	439; 1122; 2406; 278	0.3957994	0.47914088		2.0
	ZINC04073614	-8.2	366.365	0.0389	8	4	258.66	0.36898	125.68	-5.4845	769.83183457753	0.32258	6.0747	0.12059	0.46154	0.31084	0.89253 (max o	5	439; 2783; 1400; 175	0.3808063	0.4948124		2.0
	ZINC04090024	-8.2	361.440	-1.7883	7	3	282.25	0.3217	125.97	2.5895	769.83183457753	0.32258	7.9019	-5.5437	0.61538	0.4718	0.90502 (max o	2	5759; 4960	0.5325421	-0.0422291		2.0
	ZINC04098332	-8.2	414.362	-2.5155	11	4	272.1	0.47218	161.21	-2.4751	769.83183457753	0.28921	8.6291	-8.6978	0.44828	0.35871	0.95908 (max o	3	5600; 3646; 3237	0.89691025	-0.0125541		2.0
	ZINC04277756	-8.2	399.446	0.8387	8	0	293.77	0.2375	79.39	-0.5335	769.83183457753	0.28921	5.2749	2.9	0.62069	0.45386	0.85972 (max o	2	5402; 2068	-0.2468384	0.7037748		2.0
	ZINC04277849	-8.2	366.460	-0.0517	8	2	283.94	0.30285	112.81	3.2612	769.83183457753	0.32258	6.1653	-0.16027	0.5	0.57506	0.93373 (max o	2	3241; 2819	-0.3120308	0.16958785		2.0
	ZINC04277856	-8.2	396.486	-0.5505	9	2	307.7	0.31196	122.04	3.235	769.83183457753	0.29954	6.6641	-1.8378	0.5	0.58787	0.93373 (max o	3	6834; 3241; 2818	-0.3048790	0.14811797		2.0
	ZINC05275966	-8.2	394.437	0.167	6	4	262.92	0.29849	115.06	2.4849	769.83183457753	0.29954	5.9466	0.55752	0.46429	0.31582	0.9543 (max of	4	3621; 1681; 2022; 27	0.5230243	0.6100529		2.0
	ZINC12605331	-8.2	449.507	-1.9384	10	2	330.84	0.39252	167.19	2.2337	769.83183457753	0.27055	8.052	-7.1646	0.45161	0.53503	0.88451 (max o	3	5428; 1172; 2841	-0.1141262	0.16233797		2.0
	ZINC05433776	-8.2	394.475	-4.4446	9	3	285.77	0.40949	154.7	5.2323	769.83183457753	0.31063	10.558	-14.308	0.48148	0.49931	0.90045 (max o	3	5428; 3696; 2930	-0.1162585	0.14228779		2.0
	ZINC05685482	-8.2	357.385	-1.3033	7	6	239.87	0.30792	116.32	-0.30516	769.83183457753	0.32258	7.4169	-4.0402	0.46154	0.46139	0.95257 (max o	3	4997; 4137; 3267	-0.2255207	0.7319992		2.0
	ZINC05707339	-8.2	408.402	-1.7372	9	5	249.39	0.44741	153.75	-6.2295	769.83183457753	0.28921	7.8508	-6.0067	0.34483	0.33202	0.97415	1	2844	0.02877129	-0.9382366		2.0
	ZINC05707337	-8.2	408.402	-1.7372	9	5	249.39	0.44741	153.75	-6.2295	769.83183457753	0.28921	7.8508	-6.0067	0.34483	0.33202	0.97415	1	2845	0.03101915	-0.9202329		2.0
	ZINC08624294	-8.2	353.412	-0.9983	6	3	257.42	0.23514	86.61	0.63602	769.83183457753	0.33549	7.1119	-2.9757	0.64	0.44889	0.91964 (max o	3	6755; 3731; 2528	0.09511892	-0.4798759		2.0
	ZINC08636402	-8.2	389.431	-0.0196	8	4	276.73	0.41091	135.38	4.0035	769.83183457753	0.31063	6.1332	-0.063097	0.48148	0.35033	0.88538 (max o	5	7165; 6183; 6171; 58	0.48970056	0.07254834		2.0
	ZINC12296162	-8.2	432.451	-1.6066	10	2	330.08	0.35128	144.94	-2.4205	769.83183457753	0.27055	7.7202	-5.9382	0.54839	0.54037	0.94488	1	4203	0.7391162	0.64590985		2.0
	ZINC12893322	-8.2	373.364	-0.6155	8	1	270.95	0.42882	145.89	-3.1608	769.83183457753	0.32258	6.7291	-1.908	0.61538	0.46468	0.90599	1	3306	-0.1187907	0.8828284		2.0
	ZINC13403577	-8.2	358.325	-1.1	8	5	265.67	0.39229	150.15	2.235	769.83183457753	0.32258	7.2136	-3.41	0.61538	0.52785	0.9615 (max of	6	6068; 6067; 5083; 50	-0.0036567	-0.8785023		2.0
	ZINC14726937	-8.2	356.285	-3.7815	8	4	245.19	0.43656	161.18	1.0482	769.83183457753	0.32258	9.8951	-11.723	0.42308	0.38525	0.95257 (max o	3	5516; 5515; 5108	-0.8662272	0.31553602		2.0
	ZINC20464572	-8.2	414.572	-1.3265	7	2	334.58	0.1966	60.33	9.4625	769.83183457753	0.27957	7.4401	-4.7448	0.6	0.49382	0.96832 (max o	5	3866; 3026; 3027; 83	0.01125281	0.54324764		2.0
	ZINC22923479	-8.2	444.598	-1.3965	8	2	356.84	0.21236	69.56	9.4319	769.83183457753	0.2621	7.5101	-5.3282	0.625	0.50495	0.93686 (max o	5	3866; 1053; 1885; 29	0.04992461	0.5473727		2.0
	ZINC20464796	-8.2	420.508	-0.5935	9	3	313.17	0.25976	97.74	5.3822	769.83183457753	0.27957	6.7071	-2.1229	0.56667	0.54355	0.95579 (max o	4	5930; 5141; 3397; 22	-0.3833822	0.08717035		2.0

Structure	Molecule Name	affinity	Total Molweight	cLogP	H-Acceptors	H-Donors	Total Surface Area	Relative PSA	Polar Surface Area	Druglikeness	Kd (nM) @ 20°C	LE from K...	LLE from Kd ...	LELP from Kd (n...	Shape Index	Molecular...	Neighbor Si...	Neig...	Neighbor	Neighbor ...	Neighbor ...	Ring Systems	frequency
	ZINC20464796	-8.2	421.516	-2.7032	9	4	327.21	0.29153	98.94	3.3544	769.8318345753	0.27957	8.8168	-9.6691	0.56667	0.54695	0.96589 (max o	3	5142; 3398; 2232	-0.4093594	-0.5896731		2.0
	ZINC31156132	-8.2	415.370	-2.8558	11	3	295.72	0.44874	171.88	2.9919	769.8318345753	0.28921	8.9694	-9.8744	0.55172	0.5642	0.99848	1	3890	-0.1989810	0.96934265		2.0
	ZINC31156426	-8.2	388.368	-1.9969	10	5	262.24	0.45203	155.14	-7.5663	769.8318345753	0.31063	8.1105	-6.4285	0.48148	0.37688	0.94605 (max o	3	6326; 5596; 4361	-0.5985296	0.23942931		2.0
	ZINC31156455	-8.2	359.350	-3.6225	9	4	244.43	0.44888	148.74	-3.4303	769.8318345753	0.33549	9.7361	-10.798	0.48	0.37141	0.95 (max of 6)	6	6291; 5967; 3493; 34	-0.6447596	0.10877117		2.0
	ZINC31163367	-8.2	445.483	-2.0251	10	4	327.72	0.37459	165.81	-2.0398	769.8318345753	0.27055	8.1387	-7.485	0.51613	0.62878	0.96739 (max o	5	4836; 4810; 3072; 26	0.17413682	0.3804291		2.0
	ZINC31170029	-8.2	422.389	-0.4109	11	3	301.01	0.39975	143.86	-1.2018	769.8318345753	0.27957	6.5245	-1.4698	0.5	0.43436	0.9561 (max of	4	5606; 5196; 2262; 30	0.42752388	-0.6620832		2.0
	ZINC35271794	-8.2	434.396	-0.258	10	7	290.74	0.42904	177.14	-2.4912	769.8318345753	0.27055	6.3716	-0.9536	0.51613	0.39724	0.96502 (max o	3	3460; 1907; 1909	-0.7584707	-0.2320365		2.0
	ZINC35455101	-8.2	388.368	-1.9969	10	5	262.24	0.45203	155.14	-7.5663	769.8318345753	0.31063	8.1105	-6.4285	0.48148	0.37688	0.94302 (max o	4	6326; 5229; 5190; 43	-0.5613432	0.24952987		2.0
	ZINC35458300	-8.2	445.483	-2.0251	10	4	327.72	0.37459	165.81	-2.0398	769.8318345753	0.27055	8.1387	-7.485	0.51613	0.62375	0.96739 (max o	5	6007; 4836; 3504; 30	0.14319424	0.3506675		2.0
	ZINC38896528	-8.2	384.367	-5.1862	9	4	274.54	0.40023	156.02	4.6443	769.8318345753	0.29954	11.3	-17.314	0.60714	0.49963	0.90236 (max o	5	785; 786; 787; 788; 3	-0.4881184	-0.6277773		2.0
	ZINC38896528	-8.2	385.375	-2.9867	9	4	273.75	0.3985	157.64	4.9357	769.8318345753	0.29954	9.1003	-9.9709	0.60714	0.48654	0.90236 (max o	5	785; 786; 787; 788; 3	-0.4685245	-0.631067		2.0
	ZINC61989702	-8.2	366.369	-1.6201	9	6	258.12	0.44437	158.26	-14.14	769.8318345753	0.32258	7.7337	-5.0223	0.57692	0.53263	0.9464	1	3533	0.06812321	0.8136625		2.0
	ZINC67910229	-8.2	389.332	-4.9553	11	4	258.42	0.51374	175.04	-4.8669	769.8318345753	0.31063	11.069	-15.952	0.44444	0.42077	0.94605 (max o	2	3957; 1063	0.6266583	0.5499492		2.0
	ZINC67912135	-8.2	446.447	-2.1818	11	7	310.75	0.42381	178.53	-5.8124	769.8318345753	0.27055	8.2954	-8.0642	0.48387	0.47499	0.91964 (max o	3	4382; 3903; 3528	-0.4140195	0.42631626		2.0
	ZINC67912719	-8.2	416.465	-1.8823	9	7	262.4	0.43727	167.91	-0.95798	769.8318345753	0.28921	7.9959	-6.5084	0.34483	0.41166	0.94823 (max o	3	1620; 1941; 3105	0.8719357	-0.1691366		2.0
	ZINC67912718	-8.2	416.465	-1.8823	9	7	262.4	0.43727	167.91	-0.95798	769.8318345753	0.28921	7.9959	-6.5084	0.34483	0.41166	0.94823 (max o	3	1620; 1941; 3106	0.8891561	-0.1400842		2.0
	ZINC72320348	-8.2	361.413	-0.5052	6	1	266.74	0.2868	103.73	-2.1259	769.8318345753	0.32258	6.6188	-1.5661	0.46154	0.51711	0.95257 (max o	3	6061; 5282; 5283	0.912747	0.15385126		2.0
	ZINC00597112	-8.2	358.392	-1.8063	6	0	258.83	0.19708	66.92	6.2532	769.8318345753	0.32258	7.9199	-5.5995	0.53846	0.42068	0.82988	1	1632	0.13163468	-0.9784033		1.0
	ZINC02665387	-8.2	353.390	-1.2508	7	0	242.2	0.31924	86.28	-4.0652	769.8318345753	0.33549	7.3644	-3.7283	0.48	0.31411		0		0.17243095	-0.7799826		1.0
	ZINC03649853	-8.2	358.325	-1.1	8	5	265.67	0.39229	150.15	2.235	769.8318345753	0.32258	7.2136	-3.41	0.61538	0.45626	0.9615 (max of	6	6068; 6067; 5083; 50	0.03511441	-0.8447675		1.0
	ZINC03840063	-8.2	397.494	-1.6576	9	6	298.42	0.3728	158.36	5.0326	769.8318345753	0.29954	7.7712	-5.5338	0.53571	0.51839	0.89174 (max o	4	6699; 6400; 4040; 88	-0.5200378	-0.1765523		1.0
	ZINC03840142	-8.2	374.396	-1.5268	9	5	275.1	0.40229	140.65	2.3537	769.8318345753	0.31063	7.6404	-4.9151	0.55556	0.45902	0.88391 (max o	3	4482; 3603; 2758	-0.4679415	-0.1042756		1.0
	ZINC03840198	-8.2	415.509	-3.3999	10	7	305.67	0.39435	164.6	2.1117	769.8318345753	0.28921	9.5135	-11.756	0.51724	0.47546	0.86695	1	4927	-0.5729964	-0.2704356		1.0
	ZINC03842026	-8.2	387.462	-0.4135	9	1	281.86	0.28763	91.14	1.5655	769.8318345753	0.29954	6.5271	-1.3804	0.57143	0.39252	0.91871 (max o	5	3608; 907; 1022; 110	-0.0166670	-0.3100646		1.0
	ZINC03852692	-8.2	360.369	-1.3452	9	3	252.2	0.36661	118.19	-4.0555	769.8318345753	0.32258	7.4588	-4.1701	0.53846	0.44079	0.93033 (max o	3	4495; 1110; 2014	0.8127968	0.4374881		1.0
	ZINC03869572	-8.2	352.342	-7.7802	9	3	240.72	0.39868	134.24	2.8108	769.8318345753	0.33549	13.894	-23.191	0.48	0.44672	0.93007 (max o	2	4052; 4051	-0.772061	0.03299425		1.0
	ZINC03882038	-8.2	394.437	0.167	6	4	262.92	0.29849	115.06	2.4849	769.8318345753	0.29954	5.9466	0.55752	0.46429	0.31582	0.93385 (max o	4	3621; 2833; 1681; 20	0.48017853	0.6150835		1.0
	ZINC03916663	-8.2	398.271	-7.7326	13	3	256.92	0.58742	204.25	-26.394	769.8318345753	0.31063	13.846	-24.893	0.48148	0.38806		0		0.16411738	-0.973225		1.0
	ZINC04062213	-8.2	374.412	0.3524	7	3	264.03	0.33379	122.41	1.9657	769.8318345753	0.31063	5.7612	1.1345	0.44444	0.28507	0.94302 (max o	3	1397; 1398; 2032	-0.9072254	0.17385262		1.0
	ZINC04085269	-8.2	395.390	-0.5023	8	1	288.6	0.30634	108	6.3948	769.8318345753	0.28921	6.6159	-1.7368	0.55172	0.40531	0.96682 (max o	5	5367; 4959; 4525; 11	-0.0575190	-0.6895005		1.0
	ZINC04090164	-8.2	392.390	-1.01	8	1	281.39	0.30207	109.85	5.2066	769.8318345753	0.28921	7.1236	-3.4923	0.55172	0.44263		0		0.2808373	-0.8628920		1.0
	ZINC04213546	-8.2	388.296	-4.095	16	8	261.34	0.68991	221.54	4.1581	769.8318345753	0.31063	10.209	-13.183	0.55556	0.52283	0.92094 (max o	3	3228; 3227; 2800	-0.3405753	0.81234664		1.0
	ZINC04213546	-8.2	387.288	-6.2646	16	7	262.63	0.69143	227.86	4.275	769.8318345753	0.31063	12.378	-20.167	0.55556	0.52283	0.93058 (max o	3	3228; 3227; 2799	-0.3234319	0.8015352		1.0
	ZINC04222740	-8.2	350.326	-0.8983	9	2	243.28	0.4226	118.34	2.4531	769.8318345753	0.33549	7.0119	-2.6776	0.6	0.35366	0.82459	1	5391	-0.8715365	-0.3681290		1.0
	ZINC04260180	-8.2	418.516	0.4107	8	1	301.9	0.31345	118.27	9.2193	769.8318345753	0.28921	5.7029	1.4201	0.58621	0.47856	0.86774 (max o	4	3230; 35; 84; 2066	-0.2670273	0.11513927		1.0
	ZINC04260729	-8.2	358.397	0.5621	8	1	259.69	0.27429	82.19	5.3563	769.8318345753	0.32258	5.5515	1.7425	0.53846	0.51454	0.83367	1	4103	0.08112759	-0.5230302		1.0
	ZINC05157983	-8.2	358.342	-1.6588	9	4	246.68	0.42744	134.91	-5.8033	769.8318345753	0.33549	7.7724	-4.9445	0.48	0.4306		0		0.77270126	-0.5821008		1.0
	ZINC05413177	-8.2	357.433	-0.7571	7	1	262.04	0.25397	75.01	-2.3462	769.8318345753	0.32258	6.8707	-2.347	0.65385	0.39651	0.89765 (max o	3	4568; 3700; 751	-0.0824820	-0.2562236		1.0
	ZINC05415715	-8.2	360.325	-0.4487	10	1	244.48	0.40118	122.48	6.4828	769.8318345753	0.32258	6.5623	-1.391	0.53846	0.47299	0.84869	1	4989	-0.2063058	-0.7436276		1.0
	ZINC05415827	-8.2	432.519	-0.1312	8	0	312.4	0.31194	129.3	4.9827	769.8318345753	0.27957	6.2448	-0.46929	0.53333	0.53841	0.91599 (max o	3	3695; 1174; 1175	0.5372788	0.5081326		1.0
	ZINC06623689	-8.2	362.381	0.0489	8	4	262.16	0.3615	121.14	-3.0133	769.8318345753	0.32258	6.0647	0.15159	0.46154	0.3623		0		-0.9071303	0.2067696		1.0
	ZINC06624054	-8.2	399.378	-1.5753	9	2	296.48	0.38272	137.77	1.0496	769.8318345753	0.28921	7.6889	-5.4469	0.55172	0.51596	0.89093	1	544	0.16024774	0.23025012		1.0
	ZINC06624610	-8.2	352.325	-2.0956	8	2	259.43	0.35856	122.13	5.6906	769.8318345753	0.32258	8.2092	-6.4963	0.57692	0.4807	0.91059 (max o	2	1091; 2128	0.0822975	-0.2496217		1.0

Structure	Molecule Name	affinity	Total Molweight	cLogP	H-Acceptors	H-Donors	Total Surface Area	Relative PSA	Polar Surface Area	Druglikeness	Kd (nM) @ 20°C	LE from K...	LLE from Kd ...	LELP from Kd (n...	Shape Index	Molecular...	Neighbor Si...	Neig...	Neighbor	Neighbor ...	Neighbor ...	Ring Systems	frequency
	ZINC08253945	-8.2	383.474	-1.9865	8	1	301.73	0.27826	77.58	0.34247	769.8318345753	0.29954	8.1001	-6.6318	0.60714	0.47903	0.97037	1	1452	0.27554253	-0.9014881		1.0
	ZINC08299485	-8.2	448.566	-0.1738	9	3	326.06	0.33822	143.67	6.4546	769.8318345753	0.27055	6.2874	-0.64239	0.45161	0.48347	0.96502 (max o	2	5428; 3696	-0.1059674	0.08834511		1.0
	ZINC08623885	-8.2	369.400	-0.8742	8	1	288.03	0.31465	109.17	3.8096	769.8318345753	0.31063	6.9878	-2.8142	0.62963	0.35791		0		-0.9483422	-0.1622371		1.0
	ZINC08624300	-8.2	360.409	0.1203	7	3	257.78	0.29983	99.1	4.0957	769.8318345753	0.32258	5.9933	0.37293	0.57692	0.44151	0.91351 (max o	4	4654; 4601; 3285; 94	0.5304978	-0.4001216		1.0
	ZINC08627140	-8.2	394.426	-1.9116	10	4	292.76	0.37478	133.25	2.7585	769.8318345753	0.29954	8.0252	-6.3818	0.64286	0.57886	0.92486 (max o	4	5020; 3288; 2152; 25(	0.392471	-0.4631158		1.0
	ZINC08643715	-8.2	384.455	-3.6616	10	3	284.81	0.34879	126.55	-1.2742	769.8318345753	0.31063	9.7752	-11.787	0.62963	0.54456	0.87816 (max o	2	6186; 6185	-0.1181297	0.93496025		1.0
	ZINC08762256	-8.2	417.377	-1.264	12	5	291.94	0.4715	167.53	0.46018	769.8318345753	0.27957	7.3776	-4.5212	0.56667	0.38729		0		0.78028315	-0.4875458		1.0
	ZINC08830711	-8.2	354.269	-2.2098	8	0	265.58	0.37925	132.86	-6.5679	769.8318345753	0.32258	8.3234	-6.8504	0.53846	0.32744		0		0.4332219	0.72392243		1.0
	ZINC08964843	-8.2	402.390	0.849	12	7	271.99	0.47395	185.77	-0.63587	769.8318345753	0.28921	5.2646	2.9356	0.48276	0.32071	0.93101	1	6206	0.04754047	0.9163321		1.0
	ZINC12483987	-8.2	372.443	-1.3222	7	2	265.57	0.24611	80.66	1.9896	769.8318345753	0.31063	7.4358	-4.2564	0.55556	0.43536	0.92442 (max o	5	5582; 5535; 3426; 34	0.55267465	-0.7158989		1.0
	ZINC12603613	-8.2	385.459	-0.7731	9	4	288.28	0.34255	120.36	-1.748	769.8318345753	0.31063	6.8867	-2.4888	0.62963	0.57803	0.90719 (max o	3	6749; 4602; 3293	0.2037015	-0.4168277		1.0
	ZINC12603643	-8.2	368.472	0.3776	7	3	277.82	0.2782	99.1	1.1875	769.8318345753	0.32258	5.736	1.1706	0.61538	0.51311	0.87218 (max o	3	3740; 1495; 1772	0.2109647	-0.454483		1.0
	ZINC12605315	-8.2	436.471	0.0647	10	2	325.62	0.31976	124.6	5.0549	769.8318345753	0.2621	6.0489	0.24685	0.4375	0.49923	0.97533 (max o	7	278; 930; 931; 1169;	-0.7241	-0.4494743		1.0
	ZINC12605351	-8.2	447.518	-2.1331	11	3	321.33	0.41412	161.93	5.5539	769.8318345753	0.27055	8.2467	-7.8842	0.41935	0.44039	0.91162 (max o	7	4994; 1177; 1178; 13	0.00401898	0.28027394		1.0
	ZINC12898957	-8.2	420.489	0.0752	9	4	307.62	0.39142	149.95	-2.8041	769.8318345753	0.28921	6.0384	0.26002	0.41379	0.50503	0.84672	1	2274	0.01393199	0.93557745		1.0
	ZINC12956064	-8.2	381.388	-1.1828	11	6	273.8	0.46289	166.01	-1.4319	769.8318345753	0.31063	7.2964	-3.8077	0.55556	0.43523	0.86774 (max o	4	4768; 4337; 976; 245	-0.4470422	-0.6350677		1.0
	ZINC13537964	-8.2	446.403	-2.6818	12	7	300.15	0.48223	195.6	-2.1336	769.8318345753	0.27055	8.7954	-9.9123	0.51613	0.46503	0.89979 (max o	2	7787; 2309	0.91361946	-0.0696610		1.0
	ZINC13543937	-8.2	436.467	-1.1291	9	4	330.12	0.30386	137.49	-9.2761	769.8318345753	0.2621	7.2427	-4.3079	0.53125	0.49748	0.90055 (max o	2	7189; 6736	-0.0491038	-0.4175956		1.0
	ZINC13818943	-8.2	390.359	-2.4947	13	5	270.36	0.54257	186.46	3.6172	769.8318345753	0.29954	8.6083	-8.3284	0.53571	0.31883		0		-0.8738078	0.38165247		1.0
	ZINC15675048	-8.2	366.376	-1.83	8	2	287.5	0.36532	116.61	2.0705	769.8318345753	0.31063	7.9436	-5.8912	0.59259	0.43598	0.98371 (max o	3	2191; 2567; 2622	0.47226444	-0.6526334		1.0
	ZINC16040550	-8.2	373.364	-0.2528	9	1	265.54	0.36017	106.53	3.1691	769.8318345753	0.31063	6.3664	-0.81382	0.55556	0.34281	0.98141	1	4271	0.6478035	-0.6916041		1.0
	ZINC19703003	-8.2	416.520	0.4033	8	1	320.2	0.22245	82.19	2.8153	769.8318345753	0.27957	5.7103	1.4426	0.63333	0.56995		0		0.69444025	0.6933583		1.0
	ZINC20112134	-8.2	384.351	0.9984	11	4	274.09	0.44376	161.45	4.255	769.8318345753	0.29954	5.1152	3.3331	0.57143	0.39867		0		-0.1045339	-0.9817957		1.0
	ZINC20410981	-8.2	378.511	0.5118	6	1	280.31	0.21722	65.54	3.3512	769.8318345753	0.31063	5.6018	1.6476	0.55556	0.47441	0.93686 (max o	3	4701; 1245; 1246	-0.8509419	-0.1741513		1.0
	ZINC20462874	-8.2	437.535	-3.4899	8	1	335.14	0.31139	130.46	-3.0372	769.8318345753	0.27957	9.6035	-12.483	0.53333	0.58587	0.86162	1	7038	0.8628418	-0.0576177		1.0
	ZINC20463723	-8.2	372.396	0.2294	7	2	260.39	0.27205	82.99	-0.35062	769.8318345753	0.31063	5.8842	0.73849	0.55556	0.38955	0.87218	1	7791	0.18417068	0.89611006		1.0
	ZINC20463769	-8.2	410.536	0.2361	6	2	306.99	0.18033	71.43	4.2542	769.8318345753	0.27957	5.8775	0.84451	0.6	0.46758	0.97533 (max o	4	6557; 5049; 1487; 14	0.41538838	0.00248582		1.0
	ZINC20464572	-8.2	414.572	-1.3265	7	2	334.58	0.1966	60.33	9.032	769.8318345753	0.27957	7.4401	-4.7448	0.6	0.4805	0.86945 (max o	2	5547; 1283	-0.4942134	-0.6618475		1.0
	ZINC20464578	-8.2	396.530	-4.6021	8	4	321.67	0.30892	87.75	1.7742	769.8318345753	0.29954	10.716	-15.364	0.57143	0.47451	0.82039	1	2991	-0.3977386	-0.6959007		1.0
	ZINC20464578	-8.2	395.522	-2.4924	8	3	307.63	0.27738	86.55	2.6915	769.8318345753	0.29954	8.606	-8.3207	0.57143	0.45924	0.85189 (max o	2	2994; 2990	-0.4075873	-0.6775855		1.0
	ZINC20464581	-8.2	391.494	-3.4163	9	4	305.44	0.32835	106	6.4808	769.8318345753	0.29954	9.5299	-11.405	0.57143	0.43419	0.89979 (max o	3	5547; 5545; 2991	-0.4379909	-0.6544434		1.0
	ZINC20465076	-8.2	434.519	-0.5296	10	4	316.01	0.38467	151.32	6.3456	769.8318345753	0.27957	6.6432	-1.8943	0.56667	0.48187	0.89839 (max o	3	6260; 5556; 5141	-0.4168812	0.15075731		1.0
	ZINC20465076	-8.2	435.527	-2.6393	10	5	330.05	0.41085	152.52	4.457	769.8318345753	0.27957	8.7529	-9.4405	0.56667	0.48561	0.87908	1	5557	-0.4182278	-0.572293		1.0
	ZINC20465210	-8.2	391.466	-0.6532	8	3	289.11	0.2691	94.5	3.9545	769.8318345753	0.29954	6.7668	-2.1807	0.53571	0.51811	0.87344 (max o	3	7635; 6577; 4309	-0.6451087	0.5330073		1.0
	ZINC20465210	-8.2	392.474	-2.7629	8	4	303.15	0.30295	95.7	3.3361	769.8318345753	0.29954	8.8765	-9.2238	0.53571	0.52146	0.89424 (max o	4	7636; 6579; 5590; 51	0.31118065	-0.0870201		1.0
	ZINC20465827	-8.2	389.489	-0.0459	5	3	297.67	0.15954	69.54	2.4256	769.8318345753	0.29954	6.1595	-0.15324	0.60714	0.49567	0.96519 (max o	3	7057; 4318; 3396	-0.5344553	0.22342029		1.0
	ZINC20504724	-8.2	423.492	-0.0395	10	0	309.32	0.37156	122.2	2.4986	769.8318345753	0.28921	6.1531	-0.13658	0.62069	0.3804	0.86774 (max o	3	4589; 4546; 4102	-0.5265665	0.4224708		1.0
	ZINC20759465	-8.2	362.381	0.2807	8	3	262.91	0.31372	110.54	8.5686	769.8318345753	0.32258	5.8329	0.87017	0.61538	0.54434		0		-0.6544583	0.7475067		1.0
	ZINC22457672	-8.2	378.511	0.4945	6	1	277.55	0.21938	65.54	4.5685	769.8318345753	0.31063	5.6191	1.5919	0.55556	0.47599	0.89424	1	2602	-0.5650673	0.5964468		1.0
	ZINC22923476	-8.2	409.573	-4.4626	8	3	339.14	0.26485	70.76	1.587	769.8318345753	0.28921	10.576	-15.43	0.58621	0.50944	0.90502 (max o	7	5583; 4825; 4401; 38	-0.1260043	0.59582216		1.0
	ZINC22923476	-8.2	408.565	-2.3529	8	2	325.1	0.2331	69.56	5.7812	769.8318345753	0.28921	8.4665	-8.1356	0.58621	0.50944	0.96931 (max o	7	5584; 4825; 4401; 39	-0.1476623	0.562695		1.0
	ZINC22923691	-8.2	431.555	-1.4697	8	4	337.22	0.26223	97.55	5.3245	769.8318345753	0.27055	7.5833	-5.4322	0.6129	0.49645	0.9396 (max of	4	835; 1885; 2988; 302	0.01534716	0.52265364		1.0
	ZINC25668354	-8.2	387.263	-5.0032	13	4	260.17	0.60337	208.34	-1.4839	769.8318345753	0.29954	11.117	-16.703	0.42857	0.48553		0		-0.7618358	0.6440504		1.0

Structure	Molecule Name	affinity	Total Molweight	cLogP	H-Acceptors	H-Donors	Total Surface Area	Relative PSA	Polar Surface Area	Druglikeness	Kd (nM) @ 20°C	LE from K...	LLE from Kd ...	LELP from Kd (n...	Shape Index	Molecular...	Neighbor Si...	Neig...	Neighbor	Neighbor ...	Neighbor ...	Ring Systems	frequency
	ZINC31164633	-8.2	418.353	0.5089	10	6	278.55	0.43669	166.14	-1.6541	769.8318345753	0.27957	5.6047	1.8203	0.43333	0.37304	0.88944 (max o	2	1574; 1613	-0.679613	0.35871696		1.0
	ZINC31165870	-8.2	432.424	0.5139	9	1	303.09	0.37253	128.73	-6.4147	769.8318345753	0.27055	5.5997	1.8994	0.41935	0.33056	0.93414	1	1292	0.16501251	0.8668422		1.0
	ZINC31168284	-8.2	448.423	-0.4938	10	4	322.1	0.37727	159.82	-3.8997	769.8318345753	0.2621	6.6074	-1.884	0.53125	0.48799		0		0.9622386	-0.0045168		1.0
	ZINC31169224	-8.2	442.415	-0.5911	11	4	311.39	0.40284	153.37	-3.0962	769.8318345753	0.27055	6.7047	-2.1848	0.41935	0.4905	0.90474	1	5981	0.78567636	-0.5467183		1.0
	ZINC33832006	-8.2	422.341	-0.4299	11	8	270.83	0.50895	197.37	-3.0467	769.8318345753	0.27957	6.5435	-1.5377	0.43333	0.3445	0.94515 (max o	4	416; 494; 1481; 1698	-0.1040422	-0.6545638		1.0
	ZINC35361161	-8.2	434.507	-0.7045	8	3	308.85	0.32757	139.48	4.4409	769.8318345753	0.27055	6.8181	-2.6039	0.54839	0.34943	0.94488 (max o	3	3299; 3060; 1214	-0.8118588	-0.3305983		1.0
	ZINC35361163	-8.2	434.507	-0.7045	8	3	308.85	0.32757	139.48	4.4409	769.8318345753	0.27055	6.8181	-2.6039	0.54839	0.34943	0.94751 (max o	3	3299; 1214; 3059	-0.8257397	-0.3453473		1.0
	ZINC35424635	-8.2	383.454	-3.4776	9	3	271.32	0.24746	98.7	3.295	769.8318345753	0.29954	9.5912	-11.61	0.57143	0.41201		0		-0.6315076	-0.6137771		1.0
	ZINC35442242	-8.2	375.428	-1.0923	9	2	276.91	0.33928	129.29	3.483	769.8318345753	0.31063	7.2059	-3.5163	0.48148	0.56166	0.88025 (max o	2	1239; 1832	0.2530444	-0.1365906		1.0
	ZINC35442249	-8.2	360.480	-1.322	7	3	278.56	0.25582	90.94	5.2966	769.8318345753	0.32258	7.4356	-4.0982	0.61538	0.43956	0.9615 (max of	4	3436; 1597; 1910; 22	-0.4897493	0.03994911		1.0
	ZINC35465073	-8.2	404.414	1.1159	8	5	296.29	0.32232	128.84	-4.1548	769.8318345753	0.28921	4.9977	3.8584	0.58621	0.4838	0.97176 (max o	4	3902; 3899; 3375; 13	-0.5325739	0.7594484		1.0
	ZINC36369809	-8.2	396.382	-1.6749	10	0	288.41	0.33386	112.21	-6.1498	769.8318345753	0.28921	7.7885	-5.7913	0.55172	0.43049	0.86023 (max o	2	1791; 2141	-5.186942E	0.9191273		1.0
	ZINC36728547	-8.2	392.359	-1.3716	9	6	257.5	0.43355	156.91	-2.2179	769.8318345753	0.29954	7.4852	-4.579	0.46429	0.35879	0.9428 (max of	3	415; 678; 1926	-0.2864130	0.6127551		1.0
	ZINC52969269	-8.2	425.464	-4.5729	9	4	334.49	0.34707	153.45	-0.44472	769.8318345753	0.27055	10.687	-16.902	0.54839	0.59521		0		-0.7403304	0.6454853		1.0
	ZINC59101983	-8.2	353.413	0.5349	7	2	251.11	0.30198	96.3	6.0667	769.8318345753	0.33549	5.5787	1.5944	0.36	0.22509	0.93348 (max o	3	6537; 6361; 2677	-0.5543567	0.5403492		1.0
	ZINC62001289	-8.2	382.799	0.257	8	4	265.32	0.35719	121.14	-2.9168	769.8318345753	0.32258	5.8566	0.7967	0.42308	0.25698		0		0.8102346	0.41618848		1.0
	ZINC67902774	-8.2	426.460	-0.8631	9	5	287.21	0.37791	145.91	-10.34	769.8318345753	0.27957	6.9767	-3.0872	0.43333	0.33791	0.85143	1	1927	-0.8708005	-0.2510717		1.0
	ZINC67912711	-8.2	368.424	-0.6037	7	5	238.11	0.37184	127.45	-0.51541	769.8318345753	0.32258	6.7173	-1.8715	0.34615	0.34106	0.92368 (max o	4	5672; 3542; 3104; 23	0.2964675	0.616314		1.0
	ZINC67912716	-8.2	368.424	-0.6037	7	5	238.11	0.37184	127.45	-0.51541	769.8318345753	0.32258	6.7173	-1.8715	0.34615	0.34106	0.92368 (max o	3	5672; 2317; 3103	0.32542235	0.5808381		1.0
	ZINC67913817	-8.2	381.467	-0.7857	7	3	289.97	0.29072	123.5	-3.2548	769.8318345753	0.31063	6.8993	-2.5293	0.44444	0.45552	0.94302	1	6047	-0.4854070	-0.5870027		1.0
	ZINC70664673	-8.2	437.531	-0.5347	8	3	282.84	0.30367	108.69	3.9677	769.8318345753	0.27055	6.6483	-1.9763	0.32258	0.32115		0		-0.4366697	0.8933917		1.0
	ZINC72319897	-8.2	375.420	-2.7093	7	2	279.68	0.33642	108.17	0.94539	769.8318345753	0.31063	8.8229	-8.7218	0.44444	0.35596	0.92739 (max o	3	856; 3113; 1949	-0.0915174	-0.7614491		1.0
	ZINC72319898	-8.2	375.420	-2.7093	7	2	279.68	0.33642	108.17	0.94539	769.8318345753	0.31063	8.8229	-8.7218	0.44444	0.35596	0.92739 (max o	3	856; 1949; 3112	-0.1091945	-0.7504341		1.0
	ZINC77257607	-8.2	356.326	-0.9889	9	6	238.39	0.46831	156.91	-4.7468	769.8318345753	0.33549	7.1025	-2.9477	0.44	0.39992		0		-0.9174292	-0.1560034		1.0
	ZINC20610353	-8.2	441.475	-0.0128	10	2	323.63	0.3458	139.67	4.6336	769.8318345753	0.27055	6.1264	-0.04731	0.3871	0.42831	0.93139	1	6836	-0.2397108	0.94104284		0.0
	ZINC01891589	-8.1	361.329	-2.9137	9	2	270.46	0.40435	133.86	-5.1919	914.0113514704	0.31865	8.9527	-9.1439	0.61538	0.56074	0.96438 (max o	6	868; 2351; 2360; 254	0.23135643	-0.0870061		172.0
	ZINC02120846	-8.1	389.359	-3.5321	9	1	295.55	0.37956	144.89	-1.174	914.0113514704	0.29589	9.5711	-11.937	0.60714	0.54585	0.96567 (max o	6	3148; 1359; 1645; 19	0.22367546	0.10869233		172.0
	ZINC02093056	-8.1	375.356	-2.4889	9	2	282.19	0.38754	133.86	-1.7607	914.0113514704	0.30685	8.5279	-8.1112	0.62963	0.47442	0.91804 (max o	7	3579; 3153; 1641; 19	0.2001158	0.02408785		172.0
	ZINC02123347	-8.1	403.410	-1.661	9	2	310.24	0.3525	133.86	-2.3536	914.0113514704	0.28568	7.7	-5.8141	0.55172	0.48459	0.96931 (max o	5	5301; 3573; 553; 863	0.24318649	0.02885945		172.0
	ZINC02122464	-8.1	403.410	-1.7866	9	2	308.98	0.35394	133.86	-1.2647	914.0113514704	0.28568	7.8256	-6.2538	0.58621	0.57879	0.95401 (max o	9	4022; 3569; 3155; 69	0.1586246	-0.0723197		172.0
	ZINC02121040	-8.1	417.436	-1.3322	9	2	322.74	0.33885	133.86	3.1624	914.0113514704	0.27616	7.3712	-4.824	0.6	0.58574	0.9561 (max of	6	3571; 3569; 3155; 31	0.197494	-0.0611923		172.0
	ZINC02096142	-8.1	375.332	-3.9865	9	1	281.79	0.3981	144.89	0.89819	914.0113514704	0.30685	10.026	-12.992	0.59259	0.5422	0.96567 (max o	8	4017; 3580; 3142; 13	0.2034038	0.11771385		172.0
	ZINC02159848	-8.1	389.383	-1.8648	9	2	298.51	0.36635	133.86	2.8383	914.0113514704	0.29589	7.9038	-6.3024	0.60714	0.5798	0.95174 (max o	4	1648; 3143; 3144; 31	0.19473585	-0.0818444		172.0
	ZINC02126011	-8.1	417.413	-2.7338	9	1	321.57	0.34885	144.89	-3.6553	914.0113514704	0.27616	8.7728	-9.8993	0.6	0.46718	0.96385 (max o	7	3575; 3567; 1362; 27	0.27182	0.08473357		172.0
	ZINC02146650	-8.1	375.356	-2.4747	9	2	285.48	0.38307	133.86	-1.4895	914.0113514704	0.30685	8.5137	-8.0649	0.62963	0.48776	0.96567 (max o	10	4007; 4003; 4002; 35	0.21103509	-0.0214288		172.0
	ZINC02107277	-8.1	404.398	-2.1391	10	3	304.71	0.40901	159.88	2.792	914.0113514704	0.28568	8.1781	-7.4876	0.62069	0.57531	0.95401 (max o	8	6690; 4010; 3586; 35	0.02570526	0.17847401		172.0
	ZINC02121165	-8.1	375.356	-2.3192	9	2	284.75	0.38406	133.86	1.9524	914.0113514704	0.30685	8.3582	-7.5582	0.59259	0.57169	0.95174 (max o	3	4022; 3569; 3155	0.15324976	-0.0930258		172.0
	ZINC02109807	-8.1	394.423	-2.6399	8	2	286.05	0.38693	152.04	1.8042	914.0113514704	0.30685	8.6789	-8.6033	0.59259	0.55345	0.99202 (max o	4	4076; 3634; 3584; 35	-0.0428148	0.17228499		172.0
	ZINC20610007	-8.1	403.410	-1.6845	9	3	302.04	0.37233	144.86	4.7799	914.0113514704	0.28568	7.7235	-5.8964	0.55172	0.59732	0.9561 (max of	6	5304; 4759; 4290; 35	0.07366206	-0.0335644		172.0
	ZINC02156076	-8.1	375.356	-2.4408	9	2	282.19	0.38754	133.86	1.2511	914.0113514704	0.30685	8.4798	-7.9545	0.62963	0.57002	0.96297 (max o	7	5708; 5300; 4014; 40	0.14027886	0.03843965		172.0
	ZINC08964539	-8.1	432.475	-1.1759	10	4	318.57	0.34667	168.4	0.56747	914.0113514704	0.26725	7.2149	-4.3999	0.6129	0.55441	0.97423	1	4235	0.12312489	0.7923467		172.0
	ZINC12296969	-8.1	362.357	-1.1247	8	1	273.98	0.34623	113.99	1.7607	914.0113514704	0.31865	7.1637	-3.5296	0.57692	0.50172	0.8996 (max of	4	6925; 5306; 4469; 35	0.10273041	0.13403799		172.0
	ZINC04090516	-8.1	369.504	-0.9277	7	3	298.03	0.30467	125.97	2.9235	914.0113514704	0.31865	6.9667	-2.9114	0.61538	0.59818	0.96721 (max o	11	7501; 7313; 6954; 61	0.51599604	0.01268682		127.0

Structure	Molecule Name	affinity	Total Molweight	cLogP	H-Acceptors	H-Donors	Total Surface Area	Relative PSA	Polar Surface Area	Druglikeness	Kd (nM) @ 20°C	LE from K...	LLE from Kd ...	LELP from Kd (n...	Shape Index	Molecular...	Neighbor Si...	Neig...	Neighbor	Neighbor ...	Neighbor ...	Ring Systems	frequency
	ZINC08586022	-8.1	424.178	-11.354	15	3	264.95	0.71006	260.71	-42.337	914.0113514704	0.30685	17.393	-37.003	0.51852	0.48777	0.93996 (max c	6	1461; 1678; 1793; 24	-0.5868654	0.41216505		67.0
	ZINC08586022	-8.1	425.186	-10.107	15	4	263.77	0.70876	257.88	-47.067	914.0113514704	0.30685	16.146	-32.937	0.51852	0.48797	0.92418 (max c	10	3284; 1678; 1792; 21	-0.575889	0.39504153		67.0
	ZINC13539741	-8.1	440.245	-8.694	14	3	250.67	0.69354	237.65	-38.717	914.0113514704	0.30685	14.733	-28.333	0.51852	0.48526	0.97407 (max c	9	6792; 721; 1461; 149	-0.5728373	0.45221227		67.0
	ZINC13539741	-8.1	441.253	-8.694	14	3	273.28	0.7189	276.45	-38.679	914.0113514704	0.30685	14.733	-28.333	0.51852	0.48526	0.98593 (max c	8	6792; 3360; 721; 149	-0.585964	0.46676204		67.0
	ZINC13539741	-8.1	442.261	-7.0794	14	5	277.55	0.72322	270.07	-34.477	914.0113514704	0.30685	13.118	-23.071	0.51852	0.48526	0.90104 (max c	8	6792; 4257; 721; 336	-0.5797035	0.43326735		67.0
	ZINC04228231	-8.1	399.451	-3.9384	11	4	266.86	0.49858	187.08	-0.78796	914.0113514704	0.30685	9.9774	-12.835	0.55556	0.5327	0.94905 (max c	3	4636; 2959; 2960	0.60542893	0.25074726		67.0
	ZINC13507279	-8.1	355.318	-3.0071	9	4	255.46	0.4295	148.74	-3.2919	914.0113514704	0.33139	9.0461	-9.0741	0.56	0.34593	0.98188 (max c	3	6607; 6295; 5694	0.19360764	-0.6512573		49.0
	ZINC04887468	-8.1	351.318	-0.8696	11	2	249.96	0.50088	147.13	0.815	914.0113514704	0.33139	6.9086	-2.6241	0.48	0.37151	0.93686 (max c	4	1732; 1966; 2458; 32	0.13194771	-0.6751425		44.0
	ZINC04261902	-8.1	409.354	-1.2371	13	2	288.71	0.51346	173.43	1.289	914.0113514704	0.28568	7.2761	-4.3303	0.41379	0.35259	0.9339 (max of	4	4634; 4617; 3247; 19	0.09447206	-0.6794715		44.0
	ZINC12889881	-8.1	405.430	-2.4684	10	4	303.62	0.3869	150.9	5.7254	914.0113514704	0.28568	8.5074	-8.6403	0.44828	0.55607	0.9892 (max of	12	3822; 3785; 3786; 36	-0.0012403	-0.6349851		42.0
	ZINC20758716	-8.1	375.404	-1.901	9	3	284.77	0.36651	130.67	6.1083	914.0113514704	0.30685	7.94	-6.1953	0.48148	0.54346	0.96064 (max c	8	4528; 3823; 3822; 15	-0.0215185	-0.6745674		42.0
	ZINC20758816	-8.1	375.404	-1.901	9	3	284.77	0.36651	130.67	6.1083	914.0113514704	0.30685	7.94	-6.1953	0.48148	0.54355	0.96064 (max c	9	5165; 4764; 4528; 38	-0.0214617	-0.6359988		42.0
	ZINC20758596	-8.1	375.404	-1.901	9	3	284.77	0.36651	130.67	6.6384	914.0113514704	0.30685	7.94	-6.1953	0.48148	0.54836	0.97407 (max c	12	5166; 5167; 5165; 43	0.01755116	-0.6848725		42.0
	ZINC33979244	-8.1	400.172	-9.4476	13	3	251.05	0.63689	217.44	-40.879	914.0113514704	0.33139	15.487	-28.509	0.56	0.62269	0.94351 (max c	5	4387; 3472; 2852; 30	-0.7171155	0.11518039		41.0
	ZINC33979244	-8.1	399.164	-10.695	13	2	252.23	0.63858	220.27	-36.149	914.0113514704	0.33139	16.734	-32.273	0.56	0.6225	0.94351 (max c	5	4388; 2853; 3054; 30	-0.6888339	0.139479		41.0
	ZINC12889360	-8.1	410.405	-1.5043	9	3	301.14	0.35203	138.87	4.1266	914.0113514704	0.27616	7.5433	-5.4472	0.4	0.50278	0.9897 (max of	11	3859; 3748; 1261; 13	0.63770044	-0.0562216		36.0
	ZINC20756552	-8.1	408.433	-0.2642	8	2	307.05	0.30259	118.64	5.5436	914.0113514704	0.27616	6.3032	-0.95669	0.4	0.50746	0.96092 (max c	10	4328; 1547; 1782; 25	0.65354216	-0.0409506		36.0
	ZINC03985178	-8.1	396.378	-1.8636	9	3	290.14	0.36538	138.87	6.3696	914.0113514704	0.28568	7.9026	-6.5233	0.41379	0.50613	0.9617 (max of	16	4522; 4471; 4328; 43	0.6812463	-0.0866523		36.0
	ZINC12885825	-8.1	424.432	-1.146	9	2	312.24	0.31418	130.08	3.0071	914.0113514704	0.26725	7.185	-4.2881	0.3871	0.50198	0.99016 (max c	12	5034; 4962; 4522; 40	0.70480835	-0.0176646		36.0
	ZINC03869422	-8.1	425.270	-8.4777	15	3	262.23	0.70705	253.29	-42.416	914.0113514704	0.30685	14.517	-27.628	0.51852	0.4762	0.93373 (max c	7	3173; 721; 1383; 149	-0.6361247	0.39457873		33.0
	ZINC03869424	-8.1	425.270	-8.4777	15	3	262.23	0.70705	253.29	-42.416	914.0113514704	0.30685	14.517	-27.628	0.51852	0.4762	0.93373 (max c	7	721; 1383; 1490; 167	-0.6449199	0.4130811		33.0
	ZINC11616626	-8.1	363.197	-7.1787	12	3	230	0.63839	201.54	-29.747	914.0113514704	0.3452	13.218	-20.796	0.5	0.38815	0.91522 (max c	2	7786; 6107	-0.3502251	-0.9095219		33.0
	ZINC04090497	-8.1	386.427	-1.2788	8	1	287.67	0.29548	109.85	6.5707	914.0113514704	0.29589	7.3178	-4.3219	0.53571	0.51303	0.96519 (max c	16	4091; 3642; 3210; 32	0.11152722	-0.8909488		29.0
	ZINC04090597	-8.1	360.345	-3.3326	9	2	257	0.38171	130.08	7.1531	914.0113514704	0.31865	9.3716	-10.459	0.53846	0.49416	0.94883 (max c	18	4091; 3643; 3642; 11	0.10901218	-0.9099692		29.0
	ZINC04090592	-8.1	374.372	-2.9733	9	2	268	0.36604	130.08	4.9085	914.0113514704	0.30685	9.0123	-9.6899	0.51852	0.49865	0.98806 (max c	15	3642; 3345; 1404; 16	0.17017101	-0.8631733		29.0
	ZINC12888345	-8.1	416.452	-1.6101	9	2	309.28	0.31719	130.08	-3.3694	914.0113514704	0.27616	7.6491	-5.8303	0.56667	0.50256	0.9897 (max of	11	5464; 4632; 3749; 36	0.16834843	-0.883393		29.0
	ZINC04090201	-8.1	416.452	-1.6101	9	2	309.28	0.31719	130.08	-3.3694	914.0113514704	0.27616	7.6491	-5.8303	0.56667	0.50433	0.9897 (max of	11	5464; 4632; 3749; 36	0.18786706	-0.873182		29.0
	ZINC04000270	-8.1	390.415	-1.6457	9	2	300.82	0.34303	121.72	4.0203	914.0113514704	0.29589	7.6847	-5.5619	0.53571	0.3907	0.93686 (max c	6	5742; 4949; 4425; 11	-0.2389869	0.5159581		28.0
	ZINC04000429	-8.1	362.361	-2.3184	9	2	276.06	0.3738	121.72	2.5814	914.0113514704	0.31865	8.3574	-7.2757	0.53846	0.35473	0.93686 (max c	4	5742; 4501; 2028; 23	-0.2084650	0.5413871		28.0
	ZINC05643758	-8.1	415.468	-1.797	9	4	325.9	0.35229	143.22	1.202	914.0113514704	0.27616	7.836	-6.5071	0.53333	0.58657	0.97533 (max c	3	5431; 5430; 2475	-0.569993	0.5511148		28.0
	ZINC33979259	-8.1	361.182	-10.279	13	3	229.22	0.69763	214.26	-27.565	914.0113514704	0.3452	16.318	-29.775	0.5	0.43529	0.91891 (max c	6	1535; 1995; 2577; 26	-0.3383744	-0.3398092		26.0
	ZINC33979258	-8.1	362.190	-9.0309	13	4	228.04	0.69606	211.43	-31.527	914.0113514704	0.3452	15.07	-26.161	0.5	0.41453	0.91891 (max c	5	3475; 1534; 3474; 25	-0.3351879	-0.3192023		26.0
	ZINC33979259	-8.1	362.190	-9.0309	13	4	228.04	0.69606	211.43	-31.527	914.0113514704	0.3452	15.07	-26.161	0.5	0.41453	0.91891 (max c	5	1534; 2576; 2645; 34	-0.3541397	-0.3079240		26.0
	ZINC33979258	-8.1	361.182	-10.279	13	3	229.22	0.69763	214.26	-27.565	914.0113514704	0.3452	16.318	-29.775	0.5	0.43529	0.91891 (max c	6	1535; 3476; 2577; 26	-0.3439930	-0.359319		26.0
	ZINC03869963	-8.1	361.206	-8.4657	13	4	230.21	0.69893	217.22	-28.905	914.0113514704	0.3452	14.505	-24.524	0.5	0.43529	0.91891 (max c	7	6406; 4932; 3474; 15	-0.3711980	-0.3882225		26.0
	ZINC33830038	-8.1	353.302	-2.8445	9	5	246.47	0.47008	167.58	0.27088	914.0113514704	0.33139	8.8835	-8.5834	0.6	0.41449	0.94338 (max c	14	1112; 1351; 1353; 13	-0.2149788	-0.8327586		25.0
	ZINC03804533	-8.1	367.329	-2.5688	9	4	262.38	0.42976	156.58	0.46026	914.0113514704	0.31865	8.6078	-8.0616	0.57692	0.41317	0.97791 (max c	11	5090; 3443; 1367; 14	-0.2239134	-0.7794637		25.0
	ZINC31155186	-8.1	367.329	-2.5688	9	4	262.38	0.42976	156.58	0.39255	914.0113514704	0.31865	8.6078	-8.0616	0.61538	0.41317	0.94338 (max c	5	3467; 2254; 3031; 30	-0.2463486	-0.8120466		25.0
	ZINC31155033	-8.1	353.302	-2.8445	9	5	246.47	0.47008	167.58	-0.49467	914.0113514704	0.33139	8.8835	-8.5834	0.56	0.49406	0.94658	1	4797	-0.1717984	-0.7694092		25.0
	ZINC31158055	-8.1	390.383	-2.0453	10	5	267.01	0.44395	155.14	-9.3795	914.0113514704	0.30685	8.0843	-6.6656	0.48148	0.394	0.92124	1	2259	0.6703694	-0.6298992		25.0
	ZINC04102143	-8.1	359.350	-3.6225	9	4	244.43	0.44888	148.74	-3.4303	914.0113514704	0.33139	9.6615	-10.931	0.48	0.38183	0.95 (max of 1	10	5967; 5674; 3707; 34	-0.6198885	0.0756027		25.0
	ZINC04104827	-8.1	390.383	-1.9707	10	5	264.25	0.44859	155.14	-3.9213	914.0113514704	0.30685	8.0097	-6.4224	0.48148	0.37688	0.95201 (max c	3	5385; 4965; 2396	-0.5965217	-0.560664		25.0
	ZINC08964853	-8.1	429.436	2.1475	12	5	313.17	0.43848	173.16	0.0079414	914.0113514704	0.26725	3.8915	8.0354	0.54839	0.35709	0.91736 (max c	3	5040; 285; 768	-0.1629825	-0.7127918		24.0

Structure	Molecule Name	affinity	Total Molweight	cLogP	H-Acceptors	H-Donors	Total Surface Area	Relative PSA	Polar Surface Area	Druglikeness	Kd (nM) @ 20°C	LE from K...	LLE from Kd ...	LELP from Kd (n...	Shape Index	Molecular...	Neighbor Si...	Neig...	Neighbor	Neighbor ...	Neighbor ...	Ring Systems	frequency
	ZINC08964831	-8.1	401.382	1.4655	12	6	283.5	0.49531	184.16	1.3234	914.0113514704	0.28568	4.5735	5.1298	0.48276	0.33593	0.95642 (max o	13	4624; 4188; 3753; 37	-0.2337275	-0.6257114		24.0
	ZINC08740528	-8.1	441.447	2.4164	12	5	325.45	0.42194	173.16	-4.1017	914.0113514704	0.2589	3.6226	9.3333	0.5625	0.37372	0.95267 (max o	7	5029; 4626; 3304; 25	-0.2184793	-0.6959032		24.0
	ZINC08740507	-8.1	401.382	1.4655	12	6	283.5	0.49531	184.16	1.3234	914.0113514704	0.28568	4.5735	5.1298	0.51724	0.33593	0.97878 (max o	15	5458; 4627; 4626; 46.	-0.1973578	-0.6987107		24.0
	ZINC08740541	-8.1	385.383	1.8112	11	5	277.15	0.45939	163.93	1.3234	914.0113514704	0.29589	4.2278	6.1212	0.5	0.33348	0.97288 (max o	12	6194; 5460; 4188; 37	-0.2513324	-0.6738832		24.0
	ZINC08740532	-8.1	419.828	2.4172	11	5	292.57	0.43518	163.93	1.3996	914.0113514704	0.28568	3.6218	8.4611	0.48276	0.31554	0.97649 (max o	11	5835; 5459; 4188; 37	-0.2736190	-0.6689817		24.0
	ZINC12880996	-8.1	439.375	-3.2223	10	1	324.03	0.37706	154.12	4.3928	914.0113514704	0.2589	9.2613	-12.446	0.625	0.45726	0.96186 (max o	7	1218; 1474; 1804; 25	0.5136304	0.4010727		24.0
	ZINC04085608	-8.1	412.373	-1.2511	9	2	305.1	0.35385	134.22	4.7361	914.0113514704	0.27616	7.2901	-4.5303	0.63333	0.43325	0.95837 (max o	13	4090; 3343; 3307; 12	0.5482279	0.4138851		24.0
	ZINC08790685	-8.1	442.399	-1.3211	10	2	327.36	0.36034	143.45	4.7361	914.0113514704	0.2589	7.3601	-5.1027	0.5625	0.41307	0.9665 (max of	5	4090; 1786; 2416; 29	0.59320825	0.39087644		24.0
	ZINC67912320	-8.1	386.308	-1.599	11	6	257.13	0.51196	175.37	-5.8493	914.0113514704	0.30685	7.638	-5.2111	0.44444	0.43134	0.94589 (max o	4	6035; 4952; 3893; 30	-0.3665775	0.53179663		24.0
	ZINC13507418	-8.1	406.394	-2.6337	11	6	283.25	0.45758	175.28	6.4914	914.0113514704	0.28568	8.6727	-9.2189	0.44828	0.49834	0.97288 (max o	15	4912; 4908; 4896; 41	0.41609755	0.7768593		24.0
	ZINC12880930	-8.1	432.451	-1.866	10	2	322.59	0.35001	137.1	-0.15231	914.0113514704	0.26725	7.905	-6.9821	0.58065	0.48429	0.9702 (max of	7	5250; 4195; 4087; 16	0.5664735	0.24793491		22.0
	ZINC04085003	-8.1	418.425	-2.3204	10	2	308.83	0.36561	137.1	-2.5595	914.0113514704	0.27616	8.3594	-8.4023	0.56667	0.46386	0.9702 (max of	8	5753; 5250; 4195; 40	0.5468424	0.2611992		22.0
	ZINC12603765	-8.1	350.345	-0.7215	7	4	262.32	0.32479	107.89	-0.39614	914.0113514704	0.33139	6.7605	-2.1772	0.64	0.54507	0.9302 (max of	8	5821; 4652; 4184; 41	0.35310015	-0.4035177		22.0
	ZINC08964832	-8.1	401.382	1.4655	12	6	283.5	0.49531	184.16	1.3234	914.0113514704	0.28568	4.5735	5.1298	0.48276	0.33438	0.95642 (max o	13	6192; 5460; 5459; 50	-0.1443540	-0.6403733		20.0
	ZINC08740529	-8.1	441.447	2.4164	12	5	325.45	0.42194	173.16	-4.1017	914.0113514704	0.2589	3.6226	9.3333	0.5625	0.37253	0.95267 (max o	7	5460; 5029; 4626; 46.	-0.1554717	-0.6934576		20.0
	ZINC13435044	-8.1	385.161	-10.438	13	3	242.86	0.66775	226.72	-47.338	914.0113514704	0.3452	16.477	-30.238	0.58333	0.61972	0.94034 (max o	10	5088; 5086; 5056; 46	-0.6008818	-0.4370931		20.0
	ZINC13435044	-8.1	384.153	-11.686	13	2	244.04	0.66936	229.55	-42.608	914.0113514704	0.3452	17.725	-33.852	0.58333	0.61952	0.94034 (max o	10	5087; 5089; 5057; 46	-0.5850810	-0.4778981		20.0
	ZINC13660217	-8.1	431.440	-0.1614	9	4	308.3	0.35641	142.39	0.6376	914.0113514704	0.26725	6.2004	-0.60392	0.51613	0.4269	0.95771 (max o	8	3881; 3441; 1512; 22	-0.3031536	-0.7193721		19.0
	ZINC35270647	-8.1	431.440	-0.1614	9	4	308.3	0.35641	142.39	0.6376	914.0113514704	0.26725	6.2004	-0.60392	0.51613	0.42806	0.97199 (max o	8	4577; 4392; 4134; 20	-0.3445370	-0.7531287		19.0
	ZINC31154974	-8.1	417.413	-0.5893	9	5	292.39	0.3864	153.39	0.85416	914.0113514704	0.27616	6.6283	-2.1339	0.53333	0.41819	0.95318 (max o	7	4392; 4134; 3881; 15	-0.3341256	-0.7034825		19.0
	ZINC71404534	-8.1	409.386	-3.6382	12	7	280.11	0.51858	202.32	-14.737	914.0113514704	0.29589	9.6772	-12.296	0.42857	0.47924	0.95148 (max o	4	7275; 6054; 5412; 39	0.58552825	-0.4989553		19.0
	ZINC03830215	-8.1	365.409	-1.8794	8	3	245.3	0.46262	162.71	9.2567	914.0113514704	0.33139	7.9184	-5.6712	0.56	0.38362	0.90896 (max o	4	5120; 4480; 3830; 35	0.12231728	-0.1384730		17.0
	ZINC04025280	-8.1	370.312	-2.3239	8	0	267.35	0.36536	125.02	-0.16309	914.0113514704	0.30685	8.3629	-7.5735	0.44444	0.31043	0.96064 (max o	3	3626; 3186; 3187	-0.1516125	0.7609521		16.0
	ZINC04025279	-8.1	370.312	-2.3239	8	0	267.35	0.36536	125.02	-0.16309	914.0113514704	0.30685	8.3629	-7.5735	0.44444	0.31384	0.98371 (max o	3	3626; 3186; 3188	-0.1510635	0.741036		16.0
	ZINC04025276	-8.1	370.312	-2.3239	8	0	267.35	0.36536	125.02	-0.16309	914.0113514704	0.30685	8.3629	-7.5735	0.44444	0.31722	0.96064 (max o	3	3626; 3187; 3188	-0.1319622	0.7512661		16.0
	ZINC36369724	-8.1	447.439	-0.5203	10	5	320.61	0.3591	156.99	5.6773	914.0113514704	0.2589	6.5593	-2.0096	0.59375	0.57382	0.971 (max of 3	3	5243; 2295; 3514	0.60034674	-0.6755894		16.0
	ZINC36369722	-8.1	447.439	-0.5203	10	5	320.61	0.3591	156.99	5.6773	914.0113514704	0.2589	6.5593	-2.0096	0.59375	0.57597	0.971 (max of 3	3	5243; 2295; 3515	0.6121021	-0.6905550		16.0
	ZINC32124078	-8.1	376.388	-1.702	9	1	288.62	0.3462	120.36	6.2973	914.0113514704	0.30685	7.741	-5.5468	0.55556	0.54824	0.93686 (max o	4	5173; 3863; 3019; 34	0.04001107	-0.3178956		15.0
	ZINC20762004	-8.1	362.361	-2.1564	9	1	274.86	0.36353	120.36	5.6851	914.0113514704	0.31865	8.1954	-6.7673	0.53846	0.53507	0.94927 (max o	4	6276; 5173; 3863; 34	0.02056642	-0.3261201		15.0
	ZINC04095704	-8.1	432.380	-0.078	10	7	289.19	0.43134	177.14	-1.9977	914.0113514704	0.26725	6.117	-0.29186	0.51613	0.3811	0.95771 (max o	13	3788; 3464; 3243; 29	0.7356686	0.12050173		14.0
	ZINC04349262	-8.1	448.379	-0.4237	11	8	295.54	0.4664	197.37	-1.9977	914.0113514704	0.2589	6.4627	-1.6365	0.5	0.38295	0.95949 (max o	12	3282; 29; 586; 924; 9	0.750353	0.06466895		14.0
	ZINC08551390	-8.1	448.379	-0.4237	11	8	295.54	0.4664	197.37	-1.9977	914.0113514704	0.2589	6.4627	-1.6365	0.5	0.38295	0.9642 (max of	11	3788; 29; 586; 924; 9	0.7031752	0.05688256		14.0
	ZINC13435042	-8.1	385.161	-10.438	13	3	242.86	0.66775	226.72	-47.338	914.0113514704	0.3452	16.477	-30.238	0.58333	0.6201	0.94034 (max o	9	5088; 5086; 5056; 46	-0.6207155	-0.4300226		14.0
	ZINC13435042	-8.1	384.153	-11.686	13	2	244.04	0.66936	229.55	-42.608	914.0113514704	0.3452	17.725	-33.852	0.58333	0.6199	0.94034 (max o	9	5087; 5089; 5057; 46	-0.6053902	-0.4712296		14.0
	ZINC05414612	-8.1	433.488	-0.4184	10	2	313.14	0.35537	148.06	-5.4179	914.0113514704	0.27616	6.4574	-1.5151	0.53333	0.5283	0.92507 (max o	7	937; 1168; 1189; 143	0.10131792	0.35280317		14.0
	ZINC05414608	-8.1	426.468	-0.1532	9	2	297.78	0.32816	124.27	2.5213	914.0113514704	0.28568	6.1922	-0.53626	0.48276	0.52664	0.9398 (max of	8	6974; 3257; 937; 143	0.06548648	0.35961592		14.0
	ZINC04027034	-8.1	374.344	-2.9068	8	0	272.05	0.35905	125.02	-4.0072	914.0113514704	0.30685	8.9458	-9.4731	0.44444	0.36794	0.96064 (max o	3	3628; 3627; 3190	-0.0957900	0.94955015		14.0
	ZINC04027031	-8.1	374.344	-2.9068	8	0	272.05	0.35905	125.02	-4.0072	914.0113514704	0.30685	8.9458	-9.4731	0.44444	0.37407	0.96064 (max o	3	3628; 3627; 3191	-0.1008413	0.96455055		14.0
	ZINC05437786	-8.1	409.508	-0.42	7	2	322.41	0.23346	74.16	3.4555	914.0113514704	0.27616	6.459	-1.5208	0.53333	0.46656	0.92206 (max o	4	6742; 5777; 4566; 11	-0.4470108	-0.3227427		14.0
	ZINC08643506	-8.1	365.360	-0.7276	8	5	273.78	0.35306	119.92	0.14186	914.0113514704	0.31865	6.7666	-2.2834	0.65385	0.56953	0.93367 (max o	7	5024; 4657; 3334; 12	0.32064316	-0.4197701		14.0
	ZINC04083870	-8.1	404.441	-4.08	9	2	309.13	0.35652	157.31	4.1482	914.0113514704	0.28568	10.119	-14.281	0.62069	0.52822	0.96211 (max o	8	6423; 4584; 4519; 44	0.341785	0.2573343		14.0
	ZINC08917865	-8.1	432.495	-0.7109	9	2	336.26	0.30604	127.87	-51.553	914.0113514704	0.26725	6.7499	-2.66	0.51613	0.57114	0.92876 (max o	5	6422; 5465; 4518; 45	0.4204936	0.02462728		14.0
	ZINC04083870	-8.1	403.434	-6.2795	9	2	309.92	0.35816	155.69	1.8712	914.0113514704	0.28568	12.319	-21.981	0.62069	0.57085	0.93686 (max o	3	6424; 4518; 3200	0.36244342	0.20233342		14.0

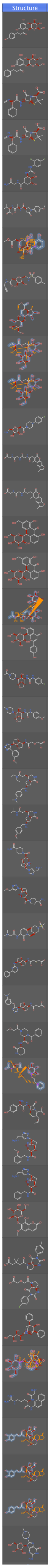

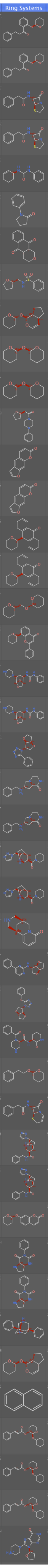

Structure	Molecule Name	affinity	Total Molweight	cLogP	H-Acceptors	H-Donors	Total Surface Area	Relative PSA	Polar Surface Area	Druglikeness	Kd (nM) @ 20°C	LE from K...	LLE from Kd ...	LELP from Kd (n...	Shape Index	Molecular...	Neighbor Si...	Neig...	Neighbor	Neighbor ...	Neighbor ...	Ring Systems	frequency
	ZINC03875408	-8.1	436.412	0.055	10	7	305.5	0.40831	177.14	-4.8731	914.0113514704	0.26725	5.984	0.2058	0.51613	0.43255	0.95267 (max o	11	5085; 3799; 3620; 32	0.82073665	-0.2633237		13.0
	ZINC14652612	-8.1	420.413	0.4007	9	6	299.15	0.37319	156.91	-4.8731	914.0113514704	0.27616	5.6383	1.451	0.5	0.42134	0.97533 (max o	7	6144; 4673; 4111; 41	0.78066397	-0.2566385		13.0
	ZINC03874214	-8.1	360.413	-1.5337	7	1	254.02	0.38347	127.2	11.036	914.0113514704	0.33139	7.5727	-4.628	0.52	0.39204	0.90896 (max o	5	4939; 3878; 1679; 27	0.11215089	-0.1028345		12.0
	ZINC03874214	-8.1	361.421	-1.5337	7	2	242.51	0.35421	128.81	10.469	914.0113514704	0.33139	7.5727	-4.628	0.52	0.39112	0.90896 (max o	4	4938; 3587; 3178; 27	0.07044989	-0.1121294		12.0
	ZINC03838759	-8.1	384.338	-1.2802	9	6	271.04	0.42418	153.78	3.7628	914.0113514704	0.30685	7.3192	-4.1721	0.59259	0.50823	0.91731 (max o	2	3602; 2757	-0.2482416	-0.5877525		12.0
	ZINC38570560	-8.1	404.441	-2.0099	9	2	308.07	0.33405	127.87	6.2137	914.0113514704	0.28568	8.0489	-7.0354	0.58621	0.48282	0.96931 (max o	7	6425; 6022; 5756; 57	0.5278199	0.31209722		12.0
	ZINC72320619	-8.1	350.322	-0.2569	8	3	236.42	0.41629	130.36	-2.1313	914.0113514704	0.33139	6.2959	-0.77521	0.48	0.40379	0.97668 (max o	10	6679; 6058; 6057; 52	0.7770392	-0.0907171		12.0
	ZINC12603685	-8.1	382.436	-0.7918	8	4	279.06	0.361	133.34	-3.8656	914.0113514704	0.31865	6.8308	-2.4849	0.65385	0.55406	0.93686 (max o	4	5482; 5064; 4655; 46	0.27656513	-0.3145411		12.0
	ZINC36377991	-8.1	390.383	-1.9707	10	5	264.25	0.44859	155.14	-3.9213	914.0113514704	0.30685	8.0097	-6.4224	0.48148	0.36873	0.95201 (max o	4	5248; 4616; 4615; 41	-0.2028350	0.45337665		12.0
	ZINC77257635	-8.1	424.397	-2.7103	12	7	290.45	0.49833	195.6	-5.5159	914.0113514704	0.28568	8.7493	-9.487	0.51724	0.49386	0.96428 (max o	3	5289; 4456; 3552	0.37702176	-0.773906		12.0
	ZINC77257632	-8.1	424.397	-2.7103	12	7	290.45	0.49833	195.6	-5.5159	914.0113514704	0.28568	8.7493	-9.487	0.51724	0.49386	0.96428 (max o	3	5289; 4456; 3553	0.38809678	-0.7904304		12.0
	ZINC08627200	-8.1	354.400	-3.9314	7	3	253.95	0.27887	100.88	2.1047	914.0113514704	0.33139	9.9704	-11.863	0.64	0.51689	0.88432 (max o	3	4171; 280; 1463	0.02221253	-0.5678553		11.0
	ZINC02112867	-8.1	442.403	-2.8355	11	3	327.19	0.42168	166.87	1.0496	914.0113514704	0.2589	8.8745	-10.952	0.59375	0.57774	0.96832 (max o	3	867; 1354; 3135	0.08091372	0.1668499		11.0
	ZINC02100435	-8.1	399.378	-1.4648	9	2	297.98	0.3808	137.77	-0.017291	914.0113514704	0.28568	7.5038	-5.1273	0.58621	0.52006	0.96473 (max o	3	3141; 867; 1646	0.10035124	0.15472916		11.0
	ZINC33913484	-8.1	418.397	-1.026	9	7	280.74	0.40871	167.91	-3.0467	914.0113514704	0.27616	7.065	-3.7152	0.36667	0.39399	0.97304 (max o	7	2049; 2050; 2071; 20	-0.5246326	0.61600816		11.0
	ZINC14417215	-8.1	418.397	-1.026	9	7	280.74	0.40871	167.91	-3.0467	914.0113514704	0.27616	7.065	-3.7152	0.36667	0.39399	0.97304 (max o	8	5512; 3470; 2049; 20	-0.5447064	0.60589737		11.0
	ZINC31158225	-8.1	448.507	0.1589	10	6	328.58	0.36095	158.3	-11.43	914.0113514704	0.26725	5.8801	0.59457	0.54839	0.60189	0.90289 (max o	2	419; 2643	-0.3927127	0.42008114		11.0
	ZINC31170020	-8.1	416.381	0.2677	9	6	282.84	0.39471	156.91	-1.9977	914.0113514704	0.27616	5.7713	0.96936	0.46667	0.38541	0.9228 (max of	3	714; 2806; 3213	0.75393516	0.12803125		10.0
	ZINC20463695	-8.1	408.429	-0.0703	9	3	288.04	0.30968	103.37	4.5786	914.0113514704	0.28568	6.1093	-0.24608	0.58621	0.46898	0.9427 (max of	6	4645; 3870; 3838; 10	-0.6103058	-0.2606314		10.0
	ZINC22922175	-8.1	392.454	-0.2978	9	3	286.2	0.30105	95.53	6.6631	914.0113514704	0.29589	6.3368	-1.0065	0.60714	0.46989	0.95709 (max o	7	5180; 3872; 3870; 37	-0.6461410	-0.2506866		10.0
	ZINC05397465	-8.1	418.385	-3.3898	12	1	303.29	0.43391	149.75	0.59099	914.0113514704	0.27616	9.4288	-12.275	0.6	0.36575	0.9561 (max of	5	1726; 1737; 1738; 24	-0.2064480	-0.3283915		10.0
	ZINC22936978	-8.1	416.588	-0.1652	7	2	328.48	0.17027	72.5	6.7267	914.0113514704	0.27616	6.2042	-0.5982	0.6	0.52639	0.93686 (max o	11	4350; 4346; 3876; 50	-0.5465283	0.05042312		10.0
	ZINC22936690	-8.1	389.518	-0.4451	7	3	302.06	0.2327	87.28	5.3227	914.0113514704	0.29589	6.4841	-1.5043	0.64286	0.47483	0.93686 (max o	8	3876; 3875; 1563; 19	-0.5015600	0.07657617		10.0
	ZINC08299705	-8.1	377.491	-7.1795	9	3	293.47	0.36045	126.19	1.2496	914.0113514704	0.30685	13.219	-23.398	0.62963	0.50503	0.96885 (max o	8	4163; 4162; 3733; 37	0.15692894	0.33107316		10.0
	ZINC08299767	-8.1	391.494	-3.183	9	2	308	0.3289	107.78	2.3227	914.0113514704	0.29589	9.222	-10.757	0.64286	0.5187	0.98451 (max o	11	5440; 4599; 4178; 41	0.1973309	0.2787437		10.0
	ZINC04236530	-8.1	369.465	-2.3064	8	2	273.48	0.33205	99.61	7.2135	914.0113514704	0.33139	8.3454	-6.9597	0.56	0.40692	0.89081 (max o	2	2433; 2434	0.0953312	-0.8101551		10.0
	ZINC05413930	-8.1	429.476	-2.1008	9	2	312.02	0.39328	150.8	2.4942	914.0113514704	0.27616	8.1398	-7.6071	0.63333	0.42782	0.95345 (max o	7	6459; 1181; 2081; 20	-0.6610302	-0.5233644		9.0
	ZINC04147905	-8.1	431.428	-2.3	12	1	315.85	0.39623	143.76	1.7161	914.0113514704	0.26725	8.339	-8.6061	0.58065	0.4222	0.95983 (max o	7	4531; 3649; 577; 170	-0.2497219	-0.3649085		9.0
	ZINC05434234	-8.1	402.449	-0.3147	9	2	304.36	0.30286	108.05	4.9815	914.0113514704	0.28568	6.3537	-1.1016	0.44828	0.52544	0.9617 (max of	6	5799; 4132; 3721; 17	0.07405764	0.2762793		9.0
	ZINC72319878	-8.1	427.424	-2.4904	10	4	312.16	0.39326	165.81	1.5644	914.0113514704	0.27616	8.5294	-9.0179	0.6	0.60309	0.99855 (max o	9	6878; 6641; 6324; 52	-0.1894319	0.6988502		9.0
	ZINC20444127	-8.1	388.423	-3.7931	8	3	274.1	0.41689	161.09	6.7688	914.0113514704	0.30685	9.8321	-12.362	0.59259	0.44815	0.96617 (max o	6	4421; 4280; 3519; 19	-0.4826129	-0.0889436		9.0
	ZINC12602709	-8.1	386.498	-5.8261	8	3	310.1	0.30758	101.12	-0.915	914.0113514704	0.29589	11.865	-19.69	0.60714	0.49273	0.97037 (max o	8	4157; 4155; 4148; 24	-0.1287591	0.08260439		9.0
	ZINC08298162	-8.1	356.472	-5.7561	7	3	287.84	0.29662	91.89	-0.91546	914.0113514704	0.31865	11.795	-18.064	0.65385	0.49307	0.96721 (max o	9	4588; 4484; 4153; 41	-0.0989456	0.06801195		9.0
	ZINC12419744	-8.1	370.309	-1.2533	10	5	250.78	0.47269	155.14	-5.8493	914.0113514704	0.31865	7.2923	-3.9332	0.46154	0.4206	0.9615 (max of	7	6211; 5408; 4983; 37	-0.4521746	0.4935115		8.0
	ZINC04260683	-8.1	386.427	-0.1864	8	1	284.08	0.29921	109.85	4.3881	914.0113514704	0.29589	6.2254	-0.62997	0.53571	0.55046	0.97773 (max o	6	6434; 4553; 3665; 36	0.13046241	-0.5893814		8.0
	ZINC04260806	-8.1	364.308	-2.6693	9	1	253.24	0.37514	119.08	2.9762	914.0113514704	0.31865	8.7083	-8.377	0.57692	0.46358	0.97533 (max o	4	6434; 4552; 4553; 36	0.12296753	-0.6248665		8.0
	ZINC35365982	-8.1	427.519	-2.0628	7	6	311.03	0.27856	108.83	2.5847	914.0113514704	0.26725	8.1018	-7.7185	0.41935	0.4885	0.94751 (max o	3	3828; 3827; 1590	0.8451458	0.12225886		8.0
	ZINC04082157	-8.1	364.346	-3.2093	10	7	234.55	0.51887	169.3	-2.3427	914.0113514704	0.33139	9.2483	-9.6842	0.44	0.36432	0.9402 (max of	5	6338; 5038; 1611; 19	0.8142048	0.01622300		8.0
	ZINC02561004	-8.1	358.444	-2.9412	8	6	265.09	0.33596	149.48	2.6721	914.0113514704	0.31865	8.9802	-9.2302	0.57692	0.52979	0.93046 (max o	3	7153; 1991; 1992	0.66162	0.27350068		8.0
	ZINC49601720	-8.1	440.443	0.0769	10	6	314.91	0.38627	166.14	-7.5878	914.0113514704	0.26725	5.9621	0.28774	0.51613	0.405	0.96262 (max o	9	531; 1059; 1333; 133	-0.3039254	0.5179535		8.0
	ZINC49601724	-8.1	440.443	0.0769	10	6	314.91	0.38627	166.14	-7.5878	914.0113514704	0.26725	5.9621	0.28774	0.51613	0.405	0.96262 (max o	9	3900; 1059; 1060; 13	-0.2712471	0.52993476		8.0
	ZINC04098733	-8.1	424.444	0.4226	9	5	308.56	0.35176	145.91	-7.5878	914.0113514704	0.27616	5.6164	1.5303	0.53333	0.40293	0.96092 (max o	4	3900; 2306; 2307; 30	-0.3227112	0.5797711		8.0
	ZINC05224212	-8.1	382.400	-4.6527	12	6	276.17	0.50534	162.9	2.8692	914.0113514704	0.30685	10.692	-15.163	0.44444	0.411	0.93996 (max o	3	6158; 5795; 4987	-0.6586883	-0.4749504		8.0

Structure	Molecule Name	affinity	Total Molweight	cLogP	H-Acceptors	H-Donors	Total Surface Area	Relative PSA	Polar Surface Area	Druglikeness	Kd (nM) @ 20°C	LE from K...	LLE from Kd ...	LELP from Kd (n...	Shape Index	Molecular...	Neighbor Si...	Neig...	Neighbor	Neighbor ...	Neighbor ...	Ring Systems	frequency
	ZINC04268368	-8.1	390.383	-1.9707	10	5	264.25	0.44859	155.14	-3.9213	914.0113514704	0.30685	8.0097	-6.4224	0.48148	0.37688	0.95201 (max o	4	3668; 2396; 2813; 28	-0.5388652	-0.5295805		8.0
	ZINC35455106	-8.1	359.350	-3.6225	9	4	244.43	0.44888	148.74	-3.4303	914.0113514704	0.33139	9.6615	-10.931	0.48	0.38183	0.94678 (max o	6	6291; 5967; 1700; 30	-0.6233468	0.11302331		8.0
	ZINC03839695	-8.1	412.239	-0.7505	8	5	265.51	0.38985	141.75	3.3008	914.0113514704	0.33139	6.7895	-2.2647	0.56	0.51273	0.89805 (max o	2	3595; 2756	-0.2141809	-0.528277		8.0
	ZINC03838796	-8.1	361.397	-0.6774	8	5	272.9	0.3793	141.75	3.987	914.0113514704	0.31865	6.7164	-2.1259	0.61538	0.51632	0.93046 (max o	2	4033; 4032	-0.2347220	-0.5185143		8.0
	ZINC20463887	-8.1	447.452	-2.8897	8	4	314.48	0.30762	112.06	-5.6592	914.0113514704	0.26725	8.9287	-10.813	0.58065	0.52723	0.93686 (max o	6	7043; 5542; 5540; 42	0.5744609	-0.1002350		8.0
	ZINC20463887	-8.1	446.444	-4.9657	8	3	315.66	0.31021	114.89	-5.9275	914.0113514704	0.26725	11.005	-18.581	0.58065	0.52723	0.89995 (max o	5	7218; 7216; 5951; 22	0.60331744	-0.0662795		8.0
	ZINC35454853	-8.1	422.469	-0.5759	10	6	308.96	0.38387	158.3	-18.912	914.0113514704	0.28568	6.6149	-2.0159	0.58621	0.53336	0.97415 (max o	5	5630; 5226; 4828; 44	-0.4095693	0.72009444		8.0
	ZINC00941351	-8.1	396.350	-1.9406	8	0	292.06	0.33445	125.02	1.3459	914.0113514704	0.28568	7.9796	-6.7928	0.44828	0.35115	0.98525 (max o	3	1634; 1960; 2711	0.15633318	0.83988255		7.0
	ZINC04073414	-8.1	368.296	-2.6592	8	0	270.06	0.3617	125.02	0.55543	914.0113514704	0.30685	8.6982	-8.6662	0.48148	0.3625	0.89424	1	2712	-0.0406836	0.9090738		7.0
	ZINC01737611	-8.1	437.403	-2.6617	9	1	326.53	0.34355	144.89	1.6055	914.0113514704	0.2589	8.7007	-10.281	0.5625	0.48737	0.97533 (max o	3	1078; 1967; 1968	-0.0301890	-0.9436969		7.0
	ZINC31164871	-8.1	440.484	-1.7456	11	7	311.11	0.42332	178.53	-1.2172	914.0113514704	0.27616	7.7846	-6.3209	0.53333	0.56681	0.98786 (max o	6	5695; 5287; 3910; 12	-0.6686311	0.5026881		7.0
	ZINC01545269	-8.1	356.285	-3.0054	8	0	264.08	0.3814	132.86	0.2672	914.0113514704	0.31865	9.0444	-9.4317	0.53846	0.39224	0.9464 (max of	3	3991; 1964; 2714	-0.8444007	-0.2801566		7.0
	ZINC04089741	-8.1	417.548	-0.1585	7	3	334.53	0.27143	125.97	3.606	914.0113514704	0.27616	6.1975	-0.57394	0.63333	0.55127	0.95053 (max o	5	6991; 6475; 6427; 49	0.564228	-0.0578937		7.0
	ZINC04521285	-8.1	385.209	1.2442	6	4	234.65	0.30854	99.38	-6.4693	914.0113514704	0.36021	4.7948	3.4541	0.56522	0.36597	0.93686 (max o	7	4508; 4066; 3779; 35	0.24183048	0.9283453		7.0
	ZINC04090921	-8.1	401.442	-1.2844	9	2	299.13	0.32247	121.88	7.465	914.0113514704	0.28568	7.3234	-4.4959	0.44828	0.51817	0.93975 (max o	3	4666; 4527; 3211	-0.7343681	0.07381645		7.0
	ZINC04090620	-8.1	401.442	-1.2844	9	2	299.13	0.32247	121.88	8.3132	914.0113514704	0.28568	7.3234	-4.4959	0.44828	0.52214	0.97649 (max o	4	4763; 4666; 4527; 32	-0.7423887	0.05329625		7.0
	ZINC31157286	-8.1	394.418	-0.2395	8	3	277.8	0.35428	130.36	-10.347	914.0113514704	0.29589	6.2785	-0.80943	0.46429	0.41687	0.95983 (max o	5	4796; 4365; 3494; 22	-0.4957383	0.5281042		7.0
	ZINC35455434	-8.1	394.418	-0.2395	8	3	277.8	0.35428	130.36	-10.347	914.0113514704	0.29589	6.2785	-0.80943	0.46429	0.41687	0.95148 (max o	4	4796; 4365; 2291; 34	-0.5047501	0.48828793		7.0
	ZINC31157197	-8.1	381.400	-2.088	8	2	266.7	0.37345	133.19	-8.3912	914.0113514704	0.30685	8.127	-6.8047	0.44444	0.4277	0.97407 (max o	4	6856; 5598; 4364; 43	-0.4800871	0.56414163		7.0
	ZINC14828630	-8.1	360.317	1.4386	8	6	246.9	0.42398	155.52	-5.8339	914.0113514704	0.31865	4.6004	4.5147	0.5	0.35002	0.98284	1	6168	-0.6661252	0.60146195		6.0
	ZINC13412864	-8.1	449.387	-0.3513	11	8	288.97	0.43188	180.3	-8.6993	914.0113514704	0.2589	6.3903	-1.3569	0.40625	0.26858	0.95709 (max o	6	972; 1815; 2421; 279	-0.8032909	0.3246694		6.0
	ZINC13412858	-8.1	449.387	-0.3513	11	8	288.97	0.43188	180.3	-8.6993	914.0113514704	0.2589	6.3903	-1.3569	0.40625	0.26858	0.95709 (max o	6	972; 1815; 2421; 279	-0.8239662	0.2921826		6.0
	ZINC02133130	-8.1	366.352	-1.4396	8	2	268.53	0.34599	118.64	5.543	914.0113514704	0.30685	7.4786	-4.6916	0.51852	0.46338	0.93686 (max o	2	1366; 2241	0.1531446	-0.1946592		6.0
	ZINC12603462	-8.1	381.359	-0.5621	9	3	278.53	0.38326	126.94	0.66821	914.0113514704	0.30685	6.6011	-1.8319	0.66667	0.46259	0.92394 (max o	5	4226; 1770; 1798; 21	-0.3016451	-0.6041636		6.0
	ZINC12529916	-8.1	374.396	-2.1594	9	2	300.12	0.38471	121.82	2.4482	914.0113514704	0.30685	8.1984	-7.0374	0.66667	0.44764	0.95174 (max o	7	4650; 3328; 1491; 21	0.619029	-0.3193943		6.0
	ZINC12529969	-8.1	378.359	-2.9899	9	2	294.21	0.39244	121.82	0.49056	914.0113514704	0.30685	9.0289	-9.744	0.66667	0.42731	0.94927 (max o	5	4650; 2149; 2150; 29	0.62281924	-0.2758399		6.0
	ZINC35456939	-8.1	406.470	-1.1587	9	7	290.09	0.38505	160.07	-3.1565	914.0113514704	0.29589	7.1977	-3.916	0.5	0.47465	0.96519 (max o	3	4833; 4411; 3929	0.89285046	0.20765378		6.0
	ZINC13660204	-8.1	388.455	-0.0883	8	5	281.14	0.3505	136.68	-3.7924	914.0113514704	0.30685	6.1273	-0.28777	0.51852	0.45195	0.97407 (max o	8	5509; 5508; 5497; 51	-0.3569406	0.18039109		6.0
	ZINC35464796	-8.1	434.524	0.0313	9	6	308.42	0.36197	156.91	-5.985	914.0113514704	0.27616	6.0077	0.11334	0.46667	0.49711	0.97304 (max o	7	6618; 6336; 4813; 39	-0.1397123	0.7938472		6.0
	ZINC35464793	-8.1	434.524	0.0313	9	6	308.42	0.36197	156.91	-5.985	914.0113514704	0.27616	6.0077	0.11334	0.46667	0.49711	0.97304 (max o	7	6618; 6336; 4813; 39	-0.1747232	0.7899699		6.0
	ZINC05433908	-8.1	419.500	-0.714	9	2	316.89	0.33204	125.5	-1.8158	914.0113514704	0.27616	6.753	-2.5854	0.56667	0.49031	0.9218 (max of	5	6976; 6459; 5424; 41	-0.6413432	-0.577818		6.0
	ZINC20758822	-8.1	412.421	-4.7864	9	2	283.98	0.36238	127.87	3.8701	914.0113514704	0.27616	10.825	-17.332	0.4	0.26549	0.98593 (max o	9	385; 387; 398; 647; 8	0.12279792	0.8618129		5.0
	ZINC70712689	-8.1	440.400	-2.7759	11	4	252.24	0.52141	169.05	-2.076	914.0113514704	0.26725	8.8149	-10.387	0.32258	0.091409	0.90873 (max o	3	534; 682; 1761	-0.6535728	-0.4399987		5.0
	ZINC04257487	-8.1	357.369	0.0027999	9	4	257.63	0.39025	125.55	-0.31782	914.0113514704	0.31865	6.0362	0.0087867	0.57692	0.36096	0.93361 (max o	8	861; 1143; 1717; 181	-0.3778148	-0.7340273		5.0
	ZINC04222705	-8.1	396.264	-1.0542	7	2	248.74	0.26301	86.33	4.4085	914.0113514704	0.3452	7.0932	-3.0539	0.54167	0.36644	0.85658	1	3656	-0.2293390	0.33998358		5.0
	ZINC08296787	-8.1	380.763	-2.9656	9	2	263.47	0.39059	127.87	4.8517	914.0113514704	0.31865	9.0046	-9.3068	0.57692	0.36529	0.94658 (max o	6	464; 556; 1454; 1672	-0.2807558	0.44336843		5.0
	ZINC08296785	-8.1	364.764	-1.9643	8	2	253.47	0.36655	118.64	5.367	914.0113514704	0.33139	8.0033	-5.9274	0.56	0.36385	0.94658 (max o	2	3272; 2864	-0.2997547	0.45717752		5.0
	ZINC20465757	-8.1	385.482	-0.2059	6	4	313.07	0.25007	83.23	1.9306	914.0113514704	0.29589	6.2449	-0.69587	0.57143	0.54401	0.9398 (max of	6	7411; 7055; 1252; 15	0.4319786	-0.2836043		5.0
	ZINC04245683	-8.1	432.380	-0.078	10	7	289.19	0.43134	177.14	-1.9977	914.0113514704	0.26725	6.117	-0.29186	0.45161	0.3811	0.93686 (max o	6	4107; 1729; 1933; 25	0.78747535	0.09874356		5.0
	ZINC12490007	-8.1	429.495	-0.8412	9	4	334.44	0.32179	106.38	0.70978	914.0113514704	0.26725	6.8802	-3.1476	0.58065	0.49802	0.93686 (max o	5	7910; 3763; 1489; 21	0.3332649	0.04692674		5.0
	ZINC22922175	-8.1	393.462	-2.4075	9	4	300.24	0.33373	96.73	2.5535	914.0113514704	0.29589	8.4465	-8.1365	0.60714	0.46989	0.95709 (max o	7	5919; 5181; 3873; 38	-0.6857096	-0.2606389		5.0
	ZINC08643907	-8.1	411.909	0.9826	7	3	282.15	0.3305	119.69	5.5162	914.0113514704	0.30685	5.0564	3.2023	0.55556	0.40472	0.97658 (max o	6	4229; 1767; 1768; 28	-0.533112	0.36959153		5.0
	ZINC03869766	-8.1	448.379	-0.0011999	11	7	297.4	0.45306	186.37	-3.6679	914.0113514704	0.2589	6.0402	-0.0046346	0.40625	0.42752	0.95709 (max o	7	1133; 1424; 2070; 21	-0.7165646	0.39995316		5.0

Structure	Molecule Name	affinity	Total Molweight	cLogP	H-Acceptors	H-Donors	Total Surface Area	Relative PSA	Polar Surface Area	Druglikeness	Kd (nM) @ 20°C	LE from K...	LLE from Kd ...	LELP from Kd (n...	Shape Index	Molecular...	Neighbor Si...	Neig...	Neighbor	Neighbor ...	Neighbor ...	Ring Systems	frequency
	ZINC20464815	-8.1	435.398	0.3741	9	4	301.95	0.31697	112.52	-4.994	914.0113514704	0.27616	5.6649	1.3546	0.56667	0.59676	0.93409 (max c	4	5549; 2227; 2594; 25	-0.3945526	-0.0436389		5.0
	ZINC20464833	-8.1	398.525	-0.4213	6	3	303.38	0.16824	72.78	3.9028	914.0113514704	0.28568	6.4603	-1.4747	0.58621	0.44573	0.96682 (max c	8	4299; 3848; 3845; 18	-0.4512860	0.25617278		5.0
	ZINC01646903	-8.1	419.417	-1.766	12	3	298.49	0.38655	161.33	-1.1402	914.0113514704	0.27616	7.805	-6.3948	0.56667	0.53461	0.96092 (max c	3	3557; 2347; 2715	0.55282164	-0.6522876		5.0
	ZINC05273906	-8.1	351.405	-0.5014	6	3	271.93	0.29526	112.66	2.9212	914.0113514704	0.31865	6.5404	-1.5735	0.57692	0.46605	0.96438 (max c	6	6693; 4024; 3999; 38	0.25714839	0.77504003		5.0
	ZINC35270647	-8.1	430.432	-2.331	9	3	309.59	0.35909	148.71	-0.33782	914.0113514704	0.26725	8.37	-8.7221	0.51613	0.42806	0.96502 (max c	3	4393; 2560; 2652	-0.3003563	-0.7900329		5.0
	ZINC13660217	-8.1	430.432	-2.331	9	3	309.59	0.35909	148.71	-0.33782	914.0113514704	0.26725	8.37	-8.7221	0.51613	0.4269	0.93414 (max c	3	4393; 2560; 2652	-0.3219114	-0.7880187		5.0
	ZINC20463436	-8.1	417.552	-1.4244	8	2	319.06	0.19579	93.09	9.377	914.0113514704	0.27616	7.4634	-5.1578	0.56667	0.58799	0.91333 (max c	10	5918; 4723; 640; 804	-0.1480915	-0.2228206		5.0
	ZINC12603718	-8.1	407.436	-0.5369	6	4	296.91	0.23051	95.4	-1.0286	914.0113514704	0.28568	6.5759	-1.8793	0.68966	0.48988	0.94251 (max c	5	6755; 5017; 2569; 28	0.11909792	-0.5126769		5.0
	ZINC08624372	-8.1	435.926	-0.2025	7	4	321.89	0.24369	104.63	0.51837	914.0113514704	0.27616	6.2415	-0.73327	0.66667	0.50241	0.93686 (max c	5	7015; 6223; 4174; 41	0.14957277	-0.4565843		5.0
	ZINC04147952	-8.1	412.466	-1.0216	10	1	299.23	0.35835	134.36	6.8682	914.0113514704	0.29589	7.0606	-3.4527	0.46429	0.55784	0.93975 (max c	4	6438; 6131; 4094; 32	-0.0323041	0.41982496		5.0
	ZINC04147955	-8.1	449.506	-0.5877	11	2	348.38	0.32479	130.17	6.0552	914.0113514704	0.2589	6.6267	-2.27	0.46875	0.58077	0.94488 (max c	5	7328; 5339; 4930; 40	-0.0536795	0.37026483		5.0
	ZINC04147960	-8.1	448.520	-1.4597	11	1	310.06	0.39596	159.81	5.6517	914.0113514704	0.28568	7.4987	-5.1095	0.44828	0.56321	0.89009 (max c	4	7598; 6131; 4094; 32	-0.0321250	0.44072393		5.0
	ZINC08254273	-8.1	357.436	-3.1884	8	3	285.22	0.33297	97.37	0.415	914.0113514704	0.31865	9.2274	-10.006	0.65385	0.49437	0.96721 (max c	6	4147; 3715; 3714; 37	-0.0683026	-0.0039027		5.0
	ZINC08254146	-8.1	371.463	-2.7583	8	3	298.98	0.31765	97.37	-1.3433	914.0113514704	0.30685	8.7973	-8.9892	0.66667	0.51343	0.96885 (max c	6	4147; 3715; 3714; 37	-0.0878453	-0.0144503		5.0
	ZINC08298077	-8.1	364.468	-2.4969	8	2	284.49	0.30261	84.92	-0.57226	914.0113514704	0.31865	8.5359	-7.8359	0.57692	0.53817	0.96721 (max c	6	5452; 5440; 4596; 37	0.24925615	0.26549003		5.0
	ZINC08299819	-8.1	358.444	-5.8112	8	4	280.43	0.35118	112.12	-1.022	914.0113514704	0.31865	11.85	-18.237	0.61538	0.46214	0.96721 (max c	5	5442; 5441; 4597; 41	-0.1488375	-0.0724140		5.0
	ZINC36378488	-8.1	389.431	-1.5936	8	3	273.31	0.41521	162.71	7.0007	914.0113514704	0.30685	7.6326	-5.1935	0.59259	0.43147	0.92094 (max c	4	4420; 1923; 2367; 35	-0.5322485	-0.0599475		5.0
	ZINC36378488	-8.1	388.423	-3.7931	8	3	274.1	0.41689	161.09	6.7688	914.0113514704	0.30685	9.8321	-12.362	0.59259	0.44815	0.93996 (max c	7	5218; 4817; 4421; 19	-0.5117675	-0.0570850		5.0
	ZINC35024379	-8.1	366.804	-4.1249	7	2	244.41	0.41394	140.86	7.1659	914.0113514704	0.3452	10.164	-11.949	0.54167	0.42833	0.94718 (max c	4	4817; 4390; 3920; 26	-0.4982431	-0.0178455		5.0
	ZINC20611589	-8.1	440.499	-0.3333	8	2	327.3	0.33731	143.94	5.5165	914.0113514704	0.26725	6.3723	-1.2471	0.48387	0.5229	0.96971 (max c	8	4471; 4328; 3859; 37	0.69503087	-0.1254844		4.0
	ZINC36369977	-8.1	384.339	-2.0839	8	0	286.24	0.35561	128.93	0.81006	914.0113514704	0.29589	8.1229	-7.0429	0.60714	0.40345	0.96023 (max c	3	844; 1321; 2667	-0.3960034	-0.8733495		4.0
	ZINC08681548	-8.1	436.412	0.055	10	7	305.5	0.40831	177.14	-4.8731	914.0113514704	0.26725	5.984	0.2058	0.51613	0.43522	0.95771 (max c	11	5085; 3799; 3620; 33	0.8058675	-0.2793723		4.0
	ZINC67913746	-8.1	421.464	-0.9847	8	2	305.6	0.32592	133.19	-8.4075	914.0113514704	0.27616	7.0237	-3.5657	0.46667	0.47687	0.96832 (max c	3	1067; 1337; 1942	-0.9121029	-0.2697662		4.0
	ZINC15952926	-8.1	373.368	-0.8314	10	5	265.98	0.4309	141.23	2.3159	914.0113514704	0.30685	6.8704	-2.7095	0.48148	0.40168	0.92739 (max c	3	4693; 1969; 2891	0.34174868	-0.5314334		4.0
	ZINC35442549	-8.1	381.495	-2.2691	8	2	303.27	0.28117	83.39	5.0787	914.0113514704	0.30685	8.3081	-7.3949	0.59259	0.55576	0.93686 (max c	4	4826; 1305; 3433; 34	-0.1611133	0.5068833		4.0
	ZINC35442578	-8.1	367.512	-1.7765	7	3	294.37	0.2559	77.32	6.1707	914.0113514704	0.31865	7.8155	-5.5751	0.57692	0.52864	0.96438 (max c	7	5997; 5584; 4826; 48	-0.1279523	0.55807614		4.0
	ZINC02125220	-8.1	412.421	-0.5993	9	1	301.37	0.30514	111.24	3.9444	914.0113514704	0.27616	6.6383	-2.1701	0.5	0.48148	0.86564 (max c	2	1655; 1972	0.23518036	0.77452886		4.0
	ZINC33832004	-8.1	418.397	0.2774	9	6	298.13	0.37447	156.91	-3.3049	914.0113514704	0.27616	5.7616	1.0045	0.6	0.44579	0.93121 (max c	7	926; 1682; 1734; 246	0.45640123	0.47261286		4.0
	ZINC33832004	-8.1	417.389	-1.3006	9	5	299.31	0.37693	159.74	-3.2279	914.0113514704	0.27616	7.3396	-4.7096	0.6	0.44579	0.92547 (max c	2	1682; 3468	0.47637412	0.44130224		4.0
	ZINC35465756	-8.1	386.439	-0.5583	8	5	279.37	0.35272	136.68	-5.8875	914.0113514704	0.30685	6.5973	-1.8195	0.51852	0.43737	0.99202 (max c	7	6606; 5963; 5188; 51	0.18640496	0.861409		4.0
	ZINC35465754	-8.1	386.439	-0.5583	8	5	279.37	0.35272	136.68	-5.8875	914.0113514704	0.30685	6.5973	-1.8195	0.51852	0.43737	0.99202 (max c	7	6606; 5963; 5188; 51	0.20128162	0.8481794		4.0
	ZINC35465752	-8.1	386.439	-0.5583	8	5	279.37	0.35272	136.68	-5.8875	914.0113514704	0.30685	6.5973	-1.8195	0.51852	0.43737	0.99202 (max c	7	6606; 5963; 5188; 51	0.22504897	0.87859637		4.0
	ZINC15222551	-8.1	408.382	-1.7427	10	5	272.62	0.39069	145.12	-4.341	914.0113514704	0.28568	7.7817	-6.1001	0.44828	0.24826	0.95099 (max c	5	2025; 2026; 2693; 26	-0.6223832	-0.665362		4.0
	ZINC04293612	-8.1	447.399	-5.6024	12	2	330.6	0.43336	185.07	3.3292	914.0113514704	0.2589	11.641	-21.639	0.59375	0.5769	0.97948 (max c	3	4976; 3671; 2069	-0.8387375	-0.0476776		4.0
	ZINC05399129	-8.1	402.473	-2.3723	9	2	319.61	0.3157	91.94	-1.3024	914.0113514704	0.28568	8.4113	-8.3039	0.65517	0.47654	0.96682 (max c	6	4123; 3688; 929; 107	-0.1914956	-0.1081404		4.0
	ZINC12603774	-8.1	394.422	-0.684	9	3	291.04	0.33428	117.56	2.4652	914.0113514704	0.29589	6.723	-2.3117	0.64286	0.55105	0.93379 (max c	5	1207; 1494; 2118; 21	0.32301837	-0.3119201		4.0
	ZINC12481465	-8.1	433.547	-1.3474	8	1	355.11	0.2395	86.58	4.1982	914.0113514704	0.26725	7.3864	-5.0417	0.54839	0.59428	0.91964 (max c	3	5848; 4724; 4717	0.65347105	0.42894822		4.0
	ZINC31156729	-8.1	418.397	0.8232	9	6	298.13	0.37447	156.91	-4.0044	914.0113514704	0.27616	5.2158	2.9809	0.5	0.43103	0.97533 (max c	7	3894; 2257; 2258; 22	-0.8100925	0.15424803		4.0
	ZINC35457135	-8.1	440.400	-1.5969	11	6	304.31	0.44258	183.21	-10.12	914.0113514704	0.26725	7.6359	-5.9752	0.48387	0.5205	0.95521 (max c	4	4834; 2260; 3496; 34	-0.7918748	0.03247398		4.0
	ZINC35457125	-8.1	440.400	-1.5969	11	6	304.31	0.44258	183.21	-10.12	914.0113514704	0.26725	7.6359	-5.9752	0.48387	0.5205	0.95267 (max c	3	4834; 3496; 3498	-0.8097625	0.02475479		4.0
	ZINC03470954	-8.1	386.534	0.4278	5	3	304.67	0.15945	71.91	-1.7559	914.0113514704	0.29589	5.6112	1.4458	0.67857	0.45547	0.99414 (max c	3	3623; 3624; 2399	0.76234686	-0.4560220		4.0
	ZINC35457936	-8.1	400.810	1.2997	8	5	282.47	0.35961	144.52	0.48888	914.0113514704	0.30685	4.7393	4.2357	0.40741	0.33485	0.97149 (max c	3	3931; 2660; 3071	-0.8591376	0.43163458		4.0
	ZINC38571055	-8.1	387.439	-0.3965	9	3	280.91	0.30878	120.64	5.956	914.0113514704	0.29589	6.4355	-1.34	0.5	0.47559	0.96253 (max c	3	3944; 2670; 3081	-0.722721	0.19306083		4.0

Structure	Molecule Name	affinity	Total Molweight	cLogP	H-Acceptors	H-Donors	Total Surface Area	Relative PSA	Polar Surface Area	Druglikeness	Kd (nM) @ 20°C	LE from K...	LLE from Kd ...	LELP from Kd (n...	Shape Index	Molecular...	Neighbor Si...	Neig...	Neighbor	Neighbor ...	Neighbor ...	Ring Systems	frequency
	ZINC67903434	-8.1	430.536	0.8705	8	4	305.55	0.32744	121.14	-5.1488	914.0113514704	0.27616	5.1685	3.1521	0.43333	0.49097	0.96343 (max o	3	5670; 5263; 2684	0.627183	0.6115986		4.0
	ZINC01612188	-8.1	423.452	-5.2627	13	5	297.18	0.46214	219.64	1.2977	914.0113514704	0.27616	11.302	-19.057	0.6	0.6156	0.97533 (max o	3	4931; 4050; 2717	0.58801377	0.775005		4.0
	ZINC20760288	-8.1	401.461	-0.0253	8	3	287.81	0.30815	107.97	4.3334	914.0113514704	0.28568	6.0643	-0.08856	0.48276	0.28196	0.95908 (max o	3	6841; 4332; 3018	0.8449051	0.40682158		4.0
	ZINC35442300	-8.1	357.476	-0.611	6	3	269.3	0.22388	78.05	4.9003	914.0113514704	0.31865	6.65	-1.9175	0.61538	0.43754	0.93686 (max o	4	4346; 2278; 3065; 34	-0.4865803	0.06277785		4.0
	ZINC22936902	-8.1	387.502	-0.681	7	3	291.56	0.24108	87.28	4.9212	914.0113514704	0.29589	6.72	-2.3015	0.64286	0.45595	0.93069 (max o	7	4346; 3876; 3875; 34	-0.4878768	0.10742132		4.0
	ZINC35465293	-8.1	431.369	-4.62	12	6	288.83	0.50521	198.43	0.77107	914.0113514704	0.27616	10.659	-16.729	0.56667	0.44315	0.9707 (max of	7	4814; 4385; 4384; 26	0.2815173	-0.6826365		4.0
	ZINC62227227	-8.1	393.371	-3.2184	10	5	279.75	0.44722	164.17	4.1929	914.0113514704	0.29589	9.2574	-10.877	0.53571	0.44317	0.95148 (max o	4	3956; 2312; 2681; 30	0.5506725	-0.3898902		4.0
	ZINC03843350	-8.1	368.381	-3.3848	8	0	280.16	0.35951	132.86	-11.135	914.0113514704	0.31865	9.4238	-10.622	0.53846	0.62243	0.96438 (max o	3	6661; 4046; 3611	-0.7774711	-0.4352129		4.0
	ZINC03977882	-8.1	414.414	-5.2749	11	2	288.77	0.52201	208.91	5.0035	914.0113514704	0.29589	11.314	-17.827	0.60714	0.50083	0.94573 (max o	3	5467; 4945; 4054	0.47985154	0.50170416		4.0
	ZINC04268377	-8.1	405.331	-6.081	12	6	260.53	0.57176	206.27	-2.1079	914.0113514704	0.29589	12.12	-20.552	0.42857	0.38316	0.93985 (max o	3	5396; 2443; 2444	-0.1202994	-0.8295550		4.0
	ZINC08297558	-8.1	398.553	-3.0671	7	2	340.07	0.23695	59.9	1.0543	914.0113514704	0.28568	9.1061	-10.736	0.62069	0.58809	0.95099 (max o	6	4152; 1455; 1456; 21	0.40670213	-0.0792045		4.0
	ZINC08297553	-8.1	398.553	-3.0671	7	2	340.07	0.23695	59.9	1.0543	914.0113514704	0.28568	9.1061	-10.736	0.62069	0.58809	0.95099 (max o	5	5005; 1455; 3274; 21	0.41809723	-0.0334413		4.0
	ZINC31155664	-8.1	372.456	0.8828	7	4	276.35	0.30917	116.45	-5.701	914.0113514704	0.31865	5.1562	2.7705	0.53846	0.5126	0.97533 (max o	7	5280; 5281; 4794; 38	0.28125224	-0.4826919		4.0
	ZINC31156088	-8.1	387.403	-2.5561	9	4	285.25	0.3953	156.58	-5.0739	914.0113514704	0.30685	8.5951	-8.3302	0.59259	0.62974	0.94905 (max o	7	5965; 5629; 5228; 52	-0.9189373	0.03996939		4.0
	ZINC31161216	-8.1	386.439	-0.2961	8	5	283.18	0.34798	136.68	-5.5643	914.0113514704	0.30685	6.3351	-0.96498	0.51852	0.48761	0.97149 (max o	3	5189; 3904; 3884	0.20248172	-0.4749093		4.0
	ZINC49181271	-8.1	374.472	0.91	7	5	276.92	0.30875	119.61	-4.4688	914.0113514704	0.31865	5.129	2.8558	0.53846	0.55128	0.97791 (max o	8	6620; 6299; 5977; 52	0.75796336	0.34606743		4.0
	ZINC49601634	-8.1	361.365	-3.7858	9	5	255.01	0.44241	159.74	-3.3297	914.0113514704	0.33139	9.8248	-11.424	0.48	0.58091	0.97396 (max o	3	6900; 6665; 4430	0.80404484	-0.0659400		4.0
	ZINC31156803	-8.1	436.412	-1.0455	10	8	300.59	0.4253	188.14	-1.8772	914.0113514704	0.26725	7.0845	-3.912	0.51613	0.47504	0.96739	1	1896	-0.6979360	0.67066556		3.0
	ZINC02137593	-8.1	350.353	-1.2472	7	1	266.84	0.29951	101.9	6.1706	914.0113514704	0.31865	7.2862	-3.914	0.57692	0.53585	0.93686 (max o	4	1089; 1091; 2361; 30	0.07005694	-0.2658310		3.0
	ZINC05433706	-8.1	358.373	-1.4748	8	0	261.75	0.29452	101.06	5.0086	914.0113514704	0.31865	7.5138	-4.6283	0.57692	0.50414	0.91804 (max o	2	1439; 1743	0.22673641	0.95424587		3.0
	ZINC08627181	-8.1	428.487	-0.5359	9	4	325.5	0.30636	124.02	1.6807	914.0113514704	0.26725	6.5749	-2.0052	0.64516	0.546	0.91333 (max o	8	6476; 5021; 5018; 37	0.39742807	-0.3742211		3.0
	ZINC15268994	-8.1	434.396	-0.258	10	7	290.74	0.42904	177.14	-2.4912	914.0113514704	0.26725	6.297	-0.96538	0.51613	0.3986	0.96018 (max o	6	3460; 979; 1237; 182	-0.7480092	-0.2162411		3.0
	ZINC08298797	-8.1	428.535	-3.2222	9	3	349.23	0.31065	87.15	0.20949	914.0113514704	0.26725	9.2612	-12.057	0.6129	0.48758	0.97423 (max o	6	3720; 760; 1187; 118	-0.1762091	-0.0428779		3.0
	ZINC20463253	-8.1	431.531	-0.8433	8	1	349.1	0.25233	94.42	4.5806	914.0113514704	0.26725	6.8823	-3.1554	0.58065	0.60096	0.94734 (max o	5	5913; 4211; 4206; 22	-0.0469653	-0.1915031		3.0
	ZINC20463253	-8.1	430.523	-0.9749	8	0	335.06	0.22101	93.22	9.257	914.0113514704	0.26725	7.0139	-3.6478	0.58065	0.60448	0.93952 (max o	6	5914; 5846; 1001; 18	-0.1333796	-0.1555935		3.0
	ZINC31157271	-8.1	394.418	-0.2395	8	3	277.8	0.35428	130.36	-10.347	914.0113514704	0.29589	6.2785	-0.80943	0.46429	0.41333	0.95983 (max o	4	4796; 4365; 3454; 22	-0.4867386	0.48251265		3.0
	ZINC02104955	-8.1	355.460	-0.2214	6	2	281.45	0.23009	60.85	6.3081	914.0113514704	0.31865	6.2604	-0.69481	0.53846	0.3882	0.94008 (max o	2	2357; 3137	-0.4255081	-0.6384530		3.0
	ZINC02104954	-8.1	355.460	-0.2214	6	2	281.45	0.23009	60.85	6.3081	914.0113514704	0.31865	6.2604	-0.69481	0.53846	0.3882	0.94008 (max o	2	3138; 2357	-0.4133739	-0.6223721		3.0
	ZINC12483741	-8.1	385.482	-0.307	6	3	291.37	0.21382	76.56	1.4201	914.0113514704	0.29589	6.346	-1.0376	0.64286	0.48945		0		-0.9553049	-0.1175388		3.0
	ZINC15919781	-8.1	390.387	0.8402	8	6	280.38	0.35167	139.84	-4.3466	914.0113514704	0.29589	5.1988	2.8396	0.57143	0.46039	0.9678 (max of	9	4139; 4140; 3899; 37	-0.5222959	0.79853225		3.0
	ZINC08636292	-8.1	377.439	-0.4234	8	4	279.74	0.31726	111.13	2.4501	914.0113514704	0.30685	6.4624	-1.3798	0.62963	0.54717	0.92418 (max o	9	6476; 5021; 5018; 41	0.35400134	-0.3673963		3.0
	ZINC40312753	-8.1	404.377	-2.9582	8	1	296.77	0.32352	123.52	3.7628	914.0113514704	0.27616	8.9972	-10.712	0.5	0.41582	0.95579 (max o	5	6036; 4852; 2305; 26	0.5282197	-0.2843969		3.0
	ZINC04042640	-8.1	364.377	-0.3499	7	1	232.66	0.51509	155.36	3.2314	914.0113514704	0.3452	6.3889	-1.0136	0.41667	0.25917	0.92619 (max o	4	5707; 4070; 4069; 55	0.60778576	-0.4587093		3.0
	ZINC04041895	-8.1	350.351	-0.6256	7	2	216.75	0.5672	166.36	3.1728	914.0113514704	0.36021	6.6646	-1.7368	0.3913	0.26346	0.93319 (max o	3	4956; 4069; 4068	0.6293863	-0.4191689		3.0
	ZINC04098344	-8.1	389.332	-5.648	11	6	255.52	0.53193	189.2	-2.7509	914.0113514704	0.30685	11.687	-18.407	0.44444	0.39572		0		0.8238023	0.5635095		3.0
	ZINC08643467	-8.1	434.896	-0.3214	9	3	295.03	0.34854	133.78	3.8475	914.0113514704	0.29589	6.3604	-1.0862	0.60714	0.54755	0.97773 (max o	2	3337; 3336	0.2216007	-0.3002029		3.0
	ZINC12603780	-8.1	400.451	-0.9274	9	3	279.61	0.36776	133.78	-4.2693	914.0113514704	0.30685	6.9664	-3.0224	0.59259	0.54358	0.94291 (max o	6	6750; 1800; 2115; 33	0.18162836	-0.3407344		3.0
	ZINC12603783	-8.1	414.477	-0.5835	9	3	291.87	0.35231	133.78	-3.2922	914.0113514704	0.29589	6.6225	-1.972	0.60714	0.54755	0.97773 (max o	3	4607; 3294; 3336	0.19374405	-0.3218137		3.0
	ZINC08879478	-8.1	445.538	-0.244	9	3	356.88	0.28912	101.41	6.7127	914.0113514704	0.2589	6.283	-0.94244	0.5	0.50653	0.97948	1	4669	-0.0565493	-0.7869599		3.0
	ZINC15673214	-8.1	379.435	-2.2	9	1	284.01	0.31178	113.09	4.9293	914.0113514704	0.30685	8.239	-7.1697	0.59259	0.57207	0.92702 (max o	4	5518; 4261; 1827; 33	-0.7125649	-0.3068564		3.0
	ZINC15673197	-8.1	351.449	0.3788	8	2	268.69	0.27053	84.99	6.4747	914.0113514704	0.33139	5.6602	1.1431	0.6	0.56904	0.95631 (max o	4	5885; 5518; 1827; 33	-0.6974091	-0.3231435		3.0
	ZINC20465409	-8.1	410.508	-1.3469	7	3	300.25	0.2033	82.01	-0.79551	914.0113514704	0.28568	7.3859	-4.7146	0.58621	0.45254	0.9427 (max of	6	5565; 5156; 5143; 47	-0.4345415	0.13936931		3.0
	ZINC20465866	-8.1	429.539	-1.2564	8	4	346.65	0.26773	99.36	2.7757	914.0113514704	0.26725	7.2954	-4.7012	0.58065	0.56266	0.93414 (max o	4	7070; 7055; 4320; 12	0.3721292	-0.2826301		3.0

Structure	Molecule Name	affinity	Total Molweight	cLogP	H-Acceptors	H-Donors	Total Surface Area	Relative PSA	Polar Surface Area	Druglikeness	Kd (nM) @ 20°C	LE from K...	LLE from Kd ...	LELP from Kd (n...	Shape Index	Molecular...	Neighbor Si...	Neig...	Neighbor	Neighbor ...	Neighbor ...	Ring Systems	frequency
	ZINC35442592	-8.1	353.485	-2.1185	7	3	280.61	0.26845	77.32	6.8696	914.0113514704	0.33139	8.1575	-6.3927	0.56	0.48479	0.90033 (max c	6	5997; 5625; 5583; 55	-0.0900640	0.576468		3.0
	ZINC08964853	-8.1	429.436	-0.8604	12	5	290.74	0.39516	162.38	-1.9228	914.0113514704	0.26725	6.8994	-3.2194	0.54839	0.39202	0.89699	1	284	-0.9641326	0.08932742		2.0
	ZINC04082209	-8.1	356.353	-0.8918	7	0	255.58	0.30108	95.97	3.8447	914.0113514704	0.31865	6.9308	-2.7987	0.53846	0.43331	0.8934	1	567	0.15181388	0.09793410		2.0
	ZINC04270894	-8.1	370.408	-0.2615	8	2	271.8	0.30048	94.74	7.1969	914.0113514704	0.30685	6.3005	-0.85222	0.59259	0.45518	0.85775	1	917	-0.6606831	-0.7249946		2.0
	ZINC20611638	-8.1	411.341	-2.5468	11	6	275.45	0.46466	168.66	2.9699	914.0113514704	0.28568	8.5858	-8.9147	0.55172	0.40375	0.93405	1	1007	0.8936013	0.18483824		2.0
	ZINC04277759	-8.1	378.427	-0.5706	9	0	273.59	0.26799	82.63	-1.065	914.0113514704	0.30685	6.6096	-1.8596	0.59259	0.4847	0.85984 (max c	3	5402; 1156; 1422	-0.3166423	0.7307351		2.0
	ZINC05433685	-8.1	416.521	-1.9223	10	3	293.45	0.35938	140.88	6.1444	914.0113514704	0.29589	7.9613	-6.4967	0.46429	0.51268	0.88538 (max c	2	5900; 5274	-0.1307184	0.3890341		2.0
	ZINC22923547	-8.1	448.566	-4.7791	10	4	341.89	0.29033	113.32	3.1762	914.0113514704	0.2589	10.818	-18.459	0.59375	0.51433	0.93686 (max c	9	6848; 5955; 5544; 48.	-0.1402253	0.50607383		2.0
	ZINC22923547	-8.1	447.558	-2.8013	10	3	352.86	0.31239	112.07	3.3459	914.0113514704	0.2589	8.8403	-10.82	0.59375	0.52517	0.93686 (max c	9	6849; 5956; 5546; 48.	-0.1287026	0.47171444		2.0
	ZINC30880881	-8.1	406.545	-1.1122	7	1	326.93	0.22011	92.78	6.0926	914.0113514704	0.28568	7.1512	-3.8931	0.62069	0.64338	0.94785	1	1603	0.946599	0.15970422		2.0
	ZINC03839334	-8.1	420.489	-0.9025	9	2	298.04	0.3776	136.29	8.3954	914.0113514704	0.28568	6.9415	-3.1591	0.55172	0.4192	0.93985 (max c	5	189; 1666; 1668; 167	-0.6549495	-0.0877415		2.0
	ZINC12653996	-8.1	409.357	-1.1314	11	1	288.79	0.41767	140.15	2.6217	914.0113514704	0.27616	7.1704	-4.0969	0.5	0.36586		0		-0.3059367	0.7906561		2.0
	ZINC08681851	-8.1	421.425	-0.4427	9	4	287.1	0.44155	166.81	-1.195	914.0113514704	0.28568	6.4817	-1.5496	0.55172	0.38679	0.94543	1	1803	0.30806124	-0.8555235		2.0
	ZINC20411124	-8.1	381.388	-3.2599	10	1	250.79	0.47482	155.46	8.0587	914.0113514704	0.31865	9.2989	-10.23	0.5	0.27631	0.91351	1	1858	-0.7552391	0.20828031		2.0
	ZINC20465505	-8.1	431.487	0.0045	9	3	323.04	0.28452	107.64	5.9953	914.0113514704	0.26725	6.0345	0.016838	0.51613	0.4825	0.91599 (max c	5	6825; 5937; 5556; 43	-0.3702876	0.12488194		2.0
	ZINC03873156	-8.1	352.360	-2.4768	6	2	235.7	0.19733	83.22	1.5859	914.0113514704	0.33139	8.5158	-7.4739	0.48	0.35916	0.96832	1	2017	0.7080134	0.43344063		2.0
	ZINC02132196	-8.1	361.440	-1.4665	7	2	279.59	0.29647	117.18	4.9047	914.0113514704	0.31865	7.5055	-4.6023	0.57692	0.4449	0.93046 (max c	2	3637; 2038	0.27483523	0.38153824		2.0
	ZINC08624385	-8.1	428.504	-0.2795	9	3	304.37	0.33785	133.78	-3.5737	914.0113514704	0.28568	6.3185	-0.97835	0.58621	0.53235	0.89839 (max c	5	6750; 3336; 2115; 24	0.1931738	-0.3600291		2.0
	ZINC05433692	-8.1	352.370	-1.1916	9	1	235.05	0.3983	129.47	0.1124	914.0113514704	0.3452	7.2306	-3.4519	0.54167	0.4759	0.92993	1	2156	-0.763311	0.5673949		2.0
	ZINC20465604	-8.1	413.496	-1.8734	8	4	323.89	0.28655	99.36	3.4624	914.0113514704	0.27616	7.9124	-6.7837	0.56667	0.49108	0.93139 (max c	2	7053; 2234	-0.3249987	-0.6376089		2.0
	ZINC20465604	-8.1	413.496	-1.7415	8	4	298.88	0.22685	99.41	5.4263	914.0113514704	0.27616	7.7805	-6.3061	0.56667	0.47151	0.93139 (max c	4	7052; 6267; 2235; 22	-0.4152348	0.03717261		2.0
	ZINC04084593	-8.1	359.425	-0.2233	6	0	245.34	0.31789	106.05	8.0296	914.0113514704	0.33139	6.2623	-0.67382	0.44	0.2481	0.92124 (max c	3	6123; 2415; 2535	0.40443403	-0.6219285		2.0
	ZINC12296951	-8.1	364.357	-0.6284	10	1	247.45	0.36153	110.7	3.8382	914.0113514704	0.31865	6.6674	-1.9721	0.46154	0.38146	0.95559 (max c	3	1485; 2143; 2518	-0.0956710	-0.9305090		2.0
	ZINC22920754	-8.1	385.486	-1.2128	7	2	278.13	0.21181	74.67	6.8344	914.0113514704	0.29589	7.2518	-4.0989	0.57143	0.43456	0.93975 (max c	4	5582; 2522; 2915; 34	0.5563842	-0.6947559		2.0
	ZINC22927068	-8.1	400.565	-0.6812	6	3	322.16	0.20459	66.38	0.70801	914.0113514704	0.28568	6.7202	-2.3844	0.44828	0.37208	0.9617	1	2616	0.9013309	0.24719404		2.0
	ZINC22922353	-8.1	434.535	-4.6665	9	3	346.77	0.31329	114.8	5.5231	914.0113514704	0.26725	10.706	-17.461	0.6129	0.50852	0.90725 (max c	4	4347; 4344; 1882; 26	-0.2015822	0.2053725		2.0
	ZINC22922353	-8.1	435.543	-1.6119	9	3	327.72	0.27337	111.86	6.9235	914.0113514704	0.26725	7.6509	-6.0314	0.6129	0.46064	0.90725 (max c	3	4343; 174; 2618	-0.4355867	0.0943595		2.0
	ZINC31157783	-8.1	396.478	0.2159	7	4	273.46	0.31244	116.45	-7.2753	914.0113514704	0.29589	5.8231	0.72967	0.39286	0.29698	0.93385 (max c	3	6328; 3926; 2631	0.7379542	0.29353884		2.0
	ZINC15222551	-8.1	407.374	0.2351	10	4	283.59	0.41426	143.87	-4.1473	914.0113514704	0.28568	5.8039	0.82294	0.44828	0.24826	0.95099 (max c	3	2692; 2694; 2969	0.84303224	0.03925308		2.0
	ZINC01293552	-8.1	364.356	-1.7979	7	2	276.74	0.33653	125.15	2.7818	914.0113514704	0.30685	7.8369	-5.8593	0.48148	0.52269	0.93379	1	2780	0.609836	0.7258199		2.0
	ZINC03842128	-8.1	386.450	0.1299	8	0	280.29	0.22591	73.4	6.7673	914.0113514704	0.29589	5.9091	0.43902	0.57143	0.47297	0.83822	1	2068	-0.2578503	0.64234495		2.0
	ZINC08765455	-8.1	359.337	-0.6937	8	1	260.48	0.44606	145.89	0.95666	914.0113514704	0.33139	6.7327	-2.0933	0.64	0.46623	0.90599	1	2942	-0.1176408	0.9020017		2.0
	ZINC20465348	-8.1	438.498	-0.4927	9	3	319.52	0.2546	97.74	4.0422	914.0113514704	0.26725	6.5317	-1.8436	0.58065	0.54535	0.94768 (max c	8	7729; 5930; 5141; 47	-0.3675496	0.10368712		2.0
	ZINC20465348	-8.1	439.506	-2.6024	9	4	333.56	0.28598	98.94	2.0144	914.0113514704	0.26725	8.6414	-9.7376	0.58065	0.54889	0.94768 (max c	5	7730; 5142; 4750; 22	-0.3664665	-0.5949012		2.0
	ZINC31156450	-8.1	359.350	-3.6225	9	4	244.43	0.44888	148.74	-3.4303	914.0113514704	0.33139	9.6615	-10.931	0.48	0.37141	0.95 (max of 6)	6	6291; 5967; 1700; 30	-0.6731365	0.08297487		2.0
	ZINC31159286	-8.1	434.396	-0.258	10	7	290.74	0.42904	177.14	-2.4912	914.0113514704	0.26725	6.297	-0.96538	0.51613	0.39676	0.94223 (max c	3	1907; 3058; 3370	-0.7465880	-0.1965951		2.0
	ZINC35458303	-8.1	445.483	-2.0251	10	4	327.72	0.37459	165.81	-2.0398	914.0113514704	0.26725	8.0641	-7.5775	0.51613	0.62375	0.96739 (max c	5	6007; 4836; 4810; 26	0.15927373	0.3653165		2.0
	ZINC61989700	-8.1	366.369	-1.6201	9	6	258.12	0.44437	158.26	-14.14	914.0113514704	0.31865	7.6591	-5.0843	0.57692	0.53263	0.9464	1	3094	0.05935664	0.8394058		2.0
	ZINC00803232	-8.1	358.460	-0.4344	6	2	269.54	0.20145	71.31	5.3478	914.0113514704	0.31865	6.4734	-1.3633	0.5	0.39959	0.98517 (max c	3	4887; 4886; 3555	0.1163845	-0.8351351		2.0
	ZINC02126221	-8.1	375.380	0.5316	9	1	261.3	0.33555	97.41	3.1899	914.0113514704	0.30685	5.5074	1.7325	0.37037	0.33473	0.96617 (max c	3	4018; 3576; 2731	0.94937885	0.03184070		2.0
	ZINC03840062	-8.1	391.427	-1.9371	10	5	293.92	0.42682	167.53	5.1601	914.0113514704	0.29589	7.9761	-6.5467	0.53571	0.45645	0.96519 (max c	4	5716; 4540; 705; 275	-0.4534831	-0.1447435		2.0
	ZINC03850286	-8.1	419.536	-2.7517	6	0	319.04	0.2569	120.39	-3.811	914.0113514704	0.27616	8.7907	-9.9641	0.46667	0.50937	0.94515 (max c	3	3612; 2013; 3170	-0.4415992	-0.7601108		2.0
	ZINC03850285	-8.1	419.536	-2.7517	6	0	319.04	0.2569	120.39	-3.811	914.0113514704	0.27616	8.7907	-9.9641	0.46667	0.5131	0.94515 (max c	3	3612; 3171; 2013	-0.4729824	-0.7731383		2.0

Structure	Molecule Name	affinity	Total Molweight	cLogP	H-Acceptors	H-Donors	Total Surface Area	Relative PSA	Polar Surface Area	Druglikeness	Kd (nM) @ 20°C	LE from K...	LLE from Kd ...	LELP from Kd (n...	Shape Index	Molecular...	Neighbor Si...	Neig...	Neighbor	Neighbor ...	Neighbor ...	Ring Systems	frequency
	ZINC04026906	-8.1	421.448	-0.09	7	3	320.03	0.26341	123.5	0.7285	914.0113514704	0.26725	6.129	-0.33676	0.58065	0.4978	0.95267	1	4506	0.68291384	-0.6434248		2.0
	ZINC04037323	-8.1	393.438	0.1128	6	2	291.12	0.24457	103.27	-4.785	914.0113514704	0.28568	5.9262	0.39484	0.51724	0.48469	0.94823 (max o	7	6379; 6074; 6070; 57	-0.0435062	0.6880819		2.0
	ZINC04040867	-8.1	356.353	-2.0541	7	0	238.96	0.33922	99.88	2.5289	914.0113514704	0.31865	8.0931	-6.4463	0.38462	0.27186	0.95257 (max o	3	4511; 4067; 1392	-0.4311911	0.8274695		2.0
	ZINC04085474	-8.1	359.425	-0.2233	6	0	245.34	0.31789	106.05	8.0296	914.0113514704	0.33139	6.2623	-0.67382	0.44	0.24828	0.92124 (max o	3	6123; 1780; 2415	0.40020654	-0.601344		2.0
	ZINC04654976	-8.1	389.426	-1.3596	7	1	277.27	0.23515	89.52	2.3577	914.0113514704	0.29589	7.3986	-4.595	0.46429	0.41125	0.96253 (max o	2	4027; 3997	0.8506338	-0.0390131		2.0
	ZINC04654976	-8.1	390.434	-2.4408	7	2	265.87	0.21255	92.45	0.32387	914.0113514704	0.29589	8.4798	-8.2491	0.46429	0.36237	0.95983 (max o	4	4423; 3998; 2364; 23	-0.1269292	-0.9541681		2.0
	ZINC05015109	-8.1	388.368	-1.827	10	4	263.15	0.45024	151.98	-6.054	914.0113514704	0.30685	7.866	-5.9541	0.48148	0.36873	0.93686 (max o	3	4115; 3675; 3248	-0.2067637	0.39165294		2.0
	ZINC05015108	-8.1	388.368	-1.827	10	4	263.15	0.45024	151.98	-6.054	914.0113514704	0.30685	7.866	-5.9541	0.48148	0.36873	0.93686 (max o	2	4115; 3249	-0.2252331	0.4042108		2.0
	ZINC05601204	-8.1	356.326	-1.7633	9	4	245.13	0.43014	134.91	-5.757	914.0113514704	0.33139	7.8023	-5.3209	0.48	0.4074	0.95631 (max o	7	4135; 3988; 3702; 36	0.4909781	0.29771373		2.0
	ZINC05685484	-8.1	357.385	-1.3033	7	6	239.87	0.30792	116.32	-0.30516	914.0113514704	0.31865	7.3423	-4.0901	0.46154	0.46139	0.94326 (max o	3	4997; 4137; 2843	-0.2078349	0.7235448		2.0
	ZINC08624322	-8.1	370.425	-0.3178	8	3	254.35	0.36497	124.55	4.6514	914.0113514704	0.33139	6.3568	-0.95898	0.56	0.48274	0.87324 (max o	3	5062; 4601; 2877	0.5108585	-0.4101126		2.0
	ZINC12604183	-8.1	365.428	0.7716	6	4	249.22	0.36578	119.25	3.9887	914.0113514704	0.33139	5.2674	2.3283	0.56	0.36252	0.91007 (max o	3	5483; 2529; 3292	-0.4588188	0.3798231		2.0
	ZINC08636525	-8.1	390.506	0.5672	7	4	277.69	0.34106	122.49	6.0268	914.0113514704	0.30685	5.4718	1.8485	0.55556	0.40645	0.92418 (max o	5	5824; 4610; 3338; 28	-0.4872076	0.34627342		2.0
	ZINC08643408	-8.1	369.460	0.0489	8	4	278.28	0.31892	111.13	-0.022475	914.0113514704	0.31865	5.9901	0.15346	0.61538	0.55037	0.90719 (max o	6	6749; 5829; 4602; 42	0.17360555	-0.3976697		2.0
	ZINC08879176	-8.1	372.376	0.1094	8	2	256.08	0.32876	97.33	1.9472	914.0113514704	0.30685	5.9296	0.35653	0.55556	0.31805	0.91768 (max o	3	5035; 4198; 1479	-0.8167496	-0.4676044		2.0
	ZINC11726787	-8.1	421.472	0.635	10	4	308.22	0.31646	142.79	0.93809	914.0113514704	0.27616	5.404	2.2994	0.7	0.58107	0.94515	1	3317	0.65492195	-0.6710595		2.0
	ZINC11726787	-8.1	420.464	0.4515	10	3	309.4	0.31907	145.62	0.97374	914.0113514704	0.27616	5.5875	1.6349	0.7	0.58107	0.94515	1	3318	0.64138955	-0.6575836		2.0
	ZINC13424931	-8.1	370.309	-1.2533	10	5	250.78	0.47269	155.14	-5.8493	914.0113514704	0.31865	7.2923	-3.9332	0.42308	0.41997	0.94326 (max o	7	6430; 5408; 4983; 49	-0.425656	0.4674718		2.0
	ZINC15218654	-8.1	366.369	-3.9926	9	6	254.04	0.43871	162.68	-2.6869	914.0113514704	0.31865	10.032	-12.53	0.53846	0.5131	0.94951	1	3808	0.22052743	0.9274653		2.0
	ZINC15709555	-8.1	414.506	-0.3874	9	2	287.37	0.4109	152.51	7.8648	914.0113514704	0.30685	6.4264	-1.2625	0.48148	0.50342	0.90171 (max o	4	6460; 5423; 4573; 17	-0.0732073	0.35794964		2.0
	ZINC20410984	-8.1	411.561	-0.9837	7	2	302.27	0.22308	84.84	0.89296	914.0113514704	0.28568	7.0227	-3.4433	0.51724	0.49255	0.95908 (max o	3	4284; 4283; 3833	-0.2471735	0.8624155		2.0
	ZINC20465250	-8.1	387.473	-0.3802	5	3	285.67	0.16624	69.54	0.80407	914.0113514704	0.29589	6.4192	-1.2849	0.57143	0.40645	0.9308 (max of	4	7057; 6822; 4748; 30	-0.5502005	0.20963518		2.0
	ZINC20465948	-8.1	422.450	-0.4405	7	5	323.47	0.27746	95.26	0.43196	914.0113514704	0.27616	6.4795	-1.5951	0.56667	0.53852	0.94768 (max o	3	5159; 806; 2237	0.3352293	-0.2928508		2.0
	ZINC21983589	-8.1	404.347	-0.3308	7	1	273.68	0.26641	104.21	1.4728	914.0113514704	0.28568	6.3698	-1.1579	0.44828	0.32937	0.9617 (max of	2	3769; 3423	-0.7312428	-0.6384027		2.0
	ZINC21983587	-8.1	404.347	-0.3308	7	1	273.68	0.26641	104.21	1.4728	914.0113514704	0.28568	6.3698	-1.1579	0.44828	0.32937	0.9617 (max of	2	3769; 3424	-0.7611301	-0.6041125		2.0
	ZINC31158623	-8.1	391.482	-0.6403	6	3	280.75	0.30016	113.35	-3.3875	914.0113514704	0.29589	6.6793	-2.164	0.42857	0.388	0.9678 (max of	4	4368; 654; 2292; 303	-0.1484507	0.42053723		2.0
	ZINC31169453	-8.1	431.369	-4.62	12	6	288.83	0.50521	198.43	0.77107	914.0113514704	0.27616	10.659	-16.729	0.56667	0.44793	0.95318 (max o	4	4814; 4385; 4384; 35	0.26830825	-0.6646822		2.0
	ZINC35361273	-8.1	427.448	0.0981	10	5	311.83	0.38463	154.78	-52.971	914.0113514704	0.27616	5.9409	0.35523	0.46667	0.56473	0.96092 (max o	3	4823; 4659; 4232	0.00333878	0.9678049		2.0
	ZINC35457120	-8.1	440.400	-1.5969	11	6	304.31	0.44258	183.21	-10.12	914.0113514704	0.26725	7.6359	-5.9752	0.48387	0.5224	0.95267 (max o	3	4834; 3497; 3498	-0.7812326	0.01404448		2.0
	ZINC35457494	-8.1	438.384	-0.3814	11	7	301.74	0.44654	186.37	-4.1057	914.0113514704	0.26725	6.4204	-1.4271	0.48387	0.40596	0.95771 (max o	3	3930; 3501; 3499	0.67461526	-0.5075801		2.0
	ZINC35457485	-8.1	438.384	-0.3814	11	7	301.74	0.44654	186.37	-4.1057	914.0113514704	0.26725	6.4204	-1.4271	0.48387	0.40596	0.95521 (max o	3	3930; 3501; 3500	0.66549784	-0.5287640		2.0
	ZINC35457498	-8.1	438.384	-0.3814	11	7	301.74	0.44654	186.37	-4.1057	914.0113514704	0.26725	6.4204	-1.4271	0.48387	0.40368	0.95771 (max o	3	3930; 3499; 3500	0.67625064	-0.5447398		2.0
	ZINC35457690	-8.1	371.317	-3.7779	10	4	260.71	0.47087	165.81	1.2603	914.0113514704	0.31865	9.8169	-11.856	0.57692	0.54724	0.98284	1	4412	0.29289755	0.81862056		2.0
	ZINC35614469	-8.1	433.504	-3.1318	8	4	317.17	0.36268	161.4	-7.2057	914.0113514704	0.27616	9.1708	-11.34	0.43333	0.57251	0.95837 (max o	5	7096; 6676; 6359; 63	-0.6667288	-0.1759174		2.0
	ZINC35614469	-8.1	434.511	-4.3565	8	4	313.45	0.35511	165.98	2.3821	914.0113514704	0.27616	10.396	-15.775	0.43333	0.59693	0.95579 (max o	3	7094; 7095; 6344	-0.5150329	-0.8284029		2.0
	ZINC38549932	-8.1	374.499	0.8471	5	3	312.02	0.20444	63.36	-6.941	914.0113514704	0.30685	5.1919	2.7607	0.51852	0.47399	0.97407 (max o	3	7563; 7445; 5648	0.00257232	-0.7815942		2.0
	ZINC40165236	-8.1	381.400	-1.2602	8	3	279.88	0.35608	136.35	1.6811	914.0113514704	0.30685	7.2992	-4.1069	0.55556	0.50979	0.96885	1	4426	-0.6592831	-0.7481922		2.0
	ZINC49181913	-8.1	416.421	-1.6984	10	6	291.07	0.40746	158.3	-1.0377	914.0113514704	0.28568	7.7374	-5.945	0.51724	0.43931	0.94529 (max o	4	5252; 3903; 825; 310	-0.3985575	0.4416543		2.0
	ZINC72325680	-8.1	359.401	-1.7532	6	0	264.73	0.35168	127.68	-2.7108	914.0113514704	0.33139	7.7922	-5.2904	0.44	0.49515	0.97118 (max o	2	5692; 3550	-0.7210518	-0.5838284		2.0
	ZINC72325680	-8.1	360.409	-3.731	6	1	253.76	0.32365	128.93	-2.9727	914.0113514704	0.33139	9.77	-11.258	0.44	0.49515	0.97118 (max o	2	5693; 3549	-0.7004914	-0.5829909		2.0
	ZINC77257587	-8.1	369.352	-7.6274	8	2	282.96	0.33574	133.47	2.0393	914.0113514704	0.30685	13.666	-24.857	0.62963	0.47541	0.96617	1	3986	-0.0311437	0.7920071		2.0
	ZINC00538328	-8.1	363.412	-2.99	6	1	264.1	0.31253	93.42	2.1271	914.0113514704	0.33139	9.029	-9.0225	0.52	0.41008	0.88064 (max o	5	5315; 1950; 1952; 23	0.58944976	-0.3848611		1.0
	ZINC00538328	-8.1	362.404	-3.1216	6	0	250.06	0.27393	92.22	6.6561	914.0113514704	0.33139	9.1606	-9.4196	0.52	0.41008	0.88064 (max o	5	5316; 1951; 1953; 23	0.5683594	-0.3561675		1.0

Structure	Molecule Name	affinity	Total Molweight	cLogP	H-Acceptors	H-Donors	Total Surface Area	Relative PSA	Polar Surface Area	Druglikeness	Kd (nM) @ 20°C	LE from K...	LLE from Kd ...	LELP from Kd (n...	Shape Index	Molecular...	Neighbor Si...	Neig...	Neighbor	Neighbor ...	Neighbor ...	Ring Systems	frequency
	ZINC00538328	-8.1	364.420	-3.4357	6	2	252.7	0.29224	96.35	2.7382	914.0113514704	0.33139	9.4747	-10.367	0.52	0.34778	0.87324 (max o	2	1954; 2337	-0.0594806	-0.8070268		1.0
	ZINC02101677	-8.1	396.374	-0.7849	8	3	291.45	0.34675	135.99	1.6968	914.0113514704	0.28568	6.8239	-2.7474	0.55172	0.52802	0.86285 (max o	2	1094; 1095	-0.7493568	0.38189092		1.0
	ZINC03809801	-8.1	407.534	-0.0612	6	1	294.71	0.36819	153.64	0.050831	914.0113514704	0.30685	6.1002	-0.19945	0.51852	0.42745	0.81711	1	4028	-0.8605029	-0.2089763		1.0
	ZINC03839401	-8.1	399.450	-1.041	9	2	280.3	0.3177	103.75	8.6579	914.0113514704	0.28568	7.08	-3.6439	0.48276	0.25421	0.93385 (max o	8	4038; 335; 431; 879;	-0.5795880	-0.0858791		1.0
	ZINC03840053	-8.1	379.411	-0.8006	9	5	286.09	0.41113	154.89	5.2903	914.0113514704	0.30685	6.8396	-2.6091	0.51852	0.50169	0.87816 (max o	3	6699; 6400; 3605	-0.5486495	-0.2199932		1.0
	ZINC03984841	-8.1	361.417	0.3681	7	3	249.05	0.2797	117.32	-7.0984	914.0113514704	0.31865	5.6709	1.1552	0.53846	0.36672	0.84869 (max o	2	4499; 1683	0.9562482	0.05149019		1.0
	ZINC03995410	-8.1	358.357	0.1603	10	5	252.39	0.45885	151.57	-0.71634	914.0113514704	0.31865	5.8787	0.50306	0.53846	0.34843		0		0.2272973	-0.943523		1.0
	ZINC04221575	-8.1	435.440	-2.0235	13	2	313.95	0.45119	183.81	0.22172	914.0113514704	0.26725	8.0625	-7.5715	0.48387	0.41134	0.89334 (max o	2	6726; 4535	-0.7214461	-0.4712633		1.0
	ZINC04245708	-8.1	388.296	-4.095	16	8	261.34	0.68991	221.54	4.1581	914.0113514704	0.30685	10.134	-13.345	0.55556	0.52283	0.92094 (max o	5	904; 905; 2799; 2800;	-0.3647893	0.78381765		1.0
	ZINC04245708	-8.1	387.288	-6.2646	16	7	262.63	0.69143	227.86	4.275	914.0113514704	0.30685	12.304	-20.416	0.55556	0.52283	0.93058 (max o	4	905; 2799; 2800; 322	-0.3476342	0.7728265		1.0
	ZINC04260179	-8.1	378.452	0.029	8	1	269.39	0.33999	110.43	7.9263	914.0113514704	0.31865	6.01	0.091009	0.57692	0.53507	0.86774 (max o	4	13; 1148; 2062; 2810	-0.2489259	0.151973		1.0
	ZINC04260894	-8.1	379.483	-2.0069	9	3	301.59	0.3394	94.74	-6.0278	914.0113514704	0.30685	8.0459	-6.5404	0.62963	0.43663	0.91109 (max o	2	1722; 1723	-0.1913792	0.02511196		1.0
	ZINC04270034	-8.1	412.390	-1.5491	10	4	275.86	0.42949	151.98	-2.117	914.0113514704	0.28568	7.5881	-5.4224	0.44828	0.35871	0.95908 (max o	3	5600; 3646; 2798	0.8780181	-0.0154910		1.0
	ZINC04277747	-8.1	390.482	0.7496	8	1	291.01	0.24477	82.19	-4.7082	914.0113514704	0.29589	5.2894	2.5334	0.60714	0.46711	0.89839	1	4105	-0.6683356	0.1425289		1.0
	ZINC04277864	-8.1	437.539	-0.0872	10	2	333.52	0.29845	125.28	2.4844	914.0113514704	0.26725	6.1262	-0.32628	0.48387	0.59201	0.8886 (max of	3	3410; 2818; 2819	-0.2951205	0.18414634		1.0
	ZINC05412980	-8.1	378.408	-0.3267	9	1	269.18	0.43358	132.81	-1.1866	914.0113514704	0.31865	6.3657	-1.0253	0.61538	0.40971	0.85614	1	3666	0.9191473	-0.0905601		1.0
	ZINC05433790	-8.1	433.487	-4.3873	11	4	323.55	0.36507	147.72	3.834	914.0113514704	0.26725	10.426	-16.416	0.48387	0.53681	0.9145 (max of	2	4160; 457	0.14075522	0.29277495		1.0
	ZINC08295572	-8.1	367.364	0.1851	9	0	263.73	0.30982	95.94	5.5386	914.0113514704	0.30685	5.8539	0.60323	0.55556	0.37562	0.85045 (max o	2	6906; 2087	0.55577284	0.5380812		1.0
	ZINC08299258	-8.1	440.590	-3.0981	8	2	367.09	0.24675	69.13	0.32375	914.0113514704	0.2589	9.1371	-11.966	0.625	0.56297	0.97533 (max o	6	5005; 1455; 1456; 21	0.4316802	-0.0493106		1.0
	ZINC08624539	-8.1	379.411	-0.6201	9	4	280.24	0.35238	120.36	2.7585	914.0113514704	0.30685	6.6591	-2.0209	0.62963	0.55815	0.93996 (max o	3	2501; 2879; 2923	0.37938315	-0.4283862		1.0
	ZINC08740215	-8.1	434.507	-0.7045	8	3	308.85	0.32757	139.48	4.4409	914.0113514704	0.26725	6.7435	-2.6361	0.54839	0.34943	0.94488 (max o	3	1214; 3059; 3060	-0.8063860	-0.3526925		1.0
	ZINC08765390	-8.1	373.364	-0.4029	8	2	265.51	0.44929	156.89	4.0021	914.0113514704	0.31865	6.4419	-1.2644	0.61538	0.49005		0		-0.8747045	-0.2235663		1.0
	ZINC12480761	-8.1	415.576	-0.019	6	1	351.33	0.20627	77.77	-0.23447	914.0113514704	0.27616	6.058	-0.068799	0.56667	0.5916	0.9112 (max of	3	7032; 5843; 3835	-0.2789069	-0.1540977		1.0
	ZINC12483169	-8.1	396.486	-0.3241	9	3	289.73	0.30787	103.37	1.6237	914.0113514704	0.29589	6.3631	-1.0953	0.57143	0.49191	0.88639 (max o	2	7385; 5055	-0.566783	-0.3264243		1.0
	ZINC12530393	-8.1	401.485	-1.7904	8	3	313.41	0.29731	92.18	4.6816	914.0113514704	0.28568	7.8294	-6.2671	0.58621	0.4421	0.87488 (max o	2	1838; 1839	-0.2595948	0.19593202		1.0
	ZINC12653494	-8.1	371.345	-1.1939	11	7	265.46	0.5009	173.1	0.41134	914.0113514704	0.31865	7.2329	-3.7468	0.57692	0.41677	0.83573	1	5484	0.30008492	-0.8749161		1.0
	ZINC12657432	-8.1	444.412	-0.8216	11	6	291.05	0.52283	208.51	-1.0932	914.0113514704	0.27616	6.8606	-2.9751	0.46667	0.41648		0		0.8515229	-0.3439927		1.0
	ZINC12902253	-8.1	406.417	-0.8842	8	1	302.83	0.29693	111.13	5.6236	914.0113514704	0.27616	6.9232	-3.2017	0.56667	0.45639	0.93111 (max o	6	5494; 619; 778; 964;	0.15331092	0.53836626		1.0
	ZINC15671846	-8.1	392.430	-1.3753	9	3	300.93	0.34217	131.03	5.269	914.0113514704	0.29589	7.4143	-4.648	0.60714	0.55393		0		0.41177478	0.7234222		1.0
	ZINC19368515	-8.1	369.400	0.1602	7	2	249.37	0.33986	140.43	4.6233	914.0113514704	0.31865	5.8788	0.50275	0.61538	0.52919		0		-0.5876777	0.6634336		1.0
	ZINC19999157	-8.1	375.424	-2.1927	8	3	271.19	0.27814	108.06	6.4631	914.0113514704	0.30685	8.2317	-7.1459	0.55556	0.34686	0.93686 (max o	4	6799; 6523; 5468; 33	-0.0244855	-0.8488999		1.0
	ZINC19999160	-8.1	375.424	-2.1927	8	3	271.19	0.27814	108.06	6.4631	914.0113514704	0.30685	8.2317	-7.1459	0.55556	0.36351	0.93686 (max o	4	6799; 6523; 5468; 33	-0.0437976	-0.837311		1.0
	ZINC20114008	-8.1	379.519	-0.8859	6	1	278.64	0.22244	67.91	-0.50806	914.0113514704	0.30685	6.9249	-2.8871	0.55556	0.47317	0.93373 (max o	2	7182; 2217	-0.6288349	0.5205809		1.0
	ZINC20463390	-8.1	414.548	-1.2589	7	1	310.86	0.16409	81.06	8.0526	914.0113514704	0.27616	7.2979	-4.5586	0.56667	0.53174	0.9396 (max of	10	5133; 4723; 640; 804;	-0.1897766	-0.2305722		1.0
	ZINC20464734	-8.1	385.483	-0.678	8	3	274.42	0.3037	110.72	7.8715	914.0113514704	0.31865	6.717	-2.1277	0.53846	0.5241	0.91007 (max o	2	5552; 1864	-0.3455579	0.15909153		1.0
	ZINC20464734	-8.1	386.491	-2.7877	8	4	288.46	0.33759	111.92	5.189	914.0113514704	0.31865	8.8267	-8.7485	0.53846	0.52943	0.92702 (max o	2	6265; 5553	-0.3244195	-0.5307862		1.0
	ZINC20465250	-8.1	388.481	-2.4899	5	4	299.71	0.2053	70.74	-2.4642	914.0113514704	0.29589	8.5289	-8.415	0.57143	0.41042	0.91157 (max o	3	7056; 4747; 3006	-0.3599796	-0.6499225		1.0
	ZINC20465505	-8.1	432.495	-2.1052	9	4	337.08	0.31432	108.84	4.1337	914.0113514704	0.26725	8.1442	-7.8772	0.51613	0.48683	0.91599 (max o	5	7050; 6826; 5938; 55	-0.3875422	-0.5549201		1.0
	ZINC20465580	-8.1	420.572	-0.3537	7	3	319.92	0.1908	82.01	-1.1061	914.0113514704	0.27616	6.3927	-1.2808	0.53333	0.45668	0.97533 (max o	3	6585; 4754; 4314	-0.4527524	0.19355628		1.0
	ZINC20503577	-8.1	389.542	-3.5336	7	3	323.28	0.24691	61.53	5.9169	914.0113514704	0.29589	9.5726	-11.942	0.60714	0.42189		0		0.67345035	0.7242651		1.0
	ZINC20504730	-8.1	373.412	-0.2935	9	1	270.97	0.31897	94.76	1.956	914.0113514704	0.30685	6.3325	-0.9565	0.59259	0.42523		0		-0.2665348	-0.9585988		1.0
	ZINC20504965	-8.1	395.502	-0.3968	9	3	304.67	0.30988	117	2.3687	914.0113514704	0.29589	6.4358	-1.341	0.53571	0.60048	0.8886 (max of	3	6834; 1148; 3241	-0.2526336	0.17420532		1.0
	ZINC20759426	-8.1	358.421	-1.5469	8	4	259.52	0.31077	104.51	6.421	914.0113514704	0.31865	7.5859	-4.8546	0.53846	0.46013	0.82392	1	648	0.68005043	0.6906047		1.0

Structure	Molecule Name	affinity	Total Molweight	cLogP	H-Acceptors	H-Donors	Total Surface Area	Relative PSA	Polar Surface Area	Druglikeness	Kd (nM) @ 20°C	LE from K...	LLE from Kd ...	LELP from Kd (n...	Shape Index	Molecular...	Neighbor Si...	Neig...	Neighbor	Neighbor ...	Neighbor ...	Ring Systems	frequency
	ZINC20761909	-8.1	376.391	-1.1985	7	2	280.01	0.29428	105.33	6.1868	914.0113514704	0.29589	7.2375	-4.0505	0.53571	0.47877	0.87363	1	1089	0.10375178	-0.3094564		1.0
	ZINC22920754	-8.1	386.494	-3.3225	7	3	292.17	0.24968	75.87	2.5103	914.0113514704	0.29589	9.3615	-11.229	0.57143	0.43456	0.91522 (max o	3	5582; 3426; 2915	0.5776594	-0.6944588		1.0
	ZINC22921063	-8.1	413.524	-5.1823	9	5	321.28	0.296	108.7	4.8596	914.0113514704	0.27616	11.221	-18.765	0.6	0.47506	0.89582 (max o	2	5953; 3432	-0.1288172	0.45140126		1.0
	ZINC22922583	-8.1	402.497	-4.9927	9	5	312.21	0.31466	108.95	4.9067	914.0113514704	0.28568	11.032	-17.476	0.58621	0.46796	0.9083 (max of	2	5953; 3427	-0.1098297	0.4596628		1.0
	ZINC30879291	-8.1	389.518	-0.8856	7	2	288.17	0.16237	70.39	8.7762	914.0113514704	0.29589	6.9246	-2.993	0.60714	0.50778	0.89169	1	4784	-0.3220090	0.93228936		1.0
	ZINC31155922	-8.1	375.349	-4.4746	10	5	249.52	0.49223	168.97	-1.6392	914.0113514704	0.31865	10.514	-14.042	0.46154	0.36926	0.94326	1	2023	-0.6363802	0.05995725		1.0
	ZINC31156007	-8.1	424.401	-0.7273	10	5	284.95	0.416	155.14	-7.7578	914.0113514704	0.27616	6.7663	-2.6336	0.43333	0.35484		0		0.8260835	-0.1030000		1.0
	ZINC31156113	-8.1	354.310	-0.9076	9	4	244.43	0.43137	134.91	-5.8493	914.0113514704	0.33139	6.9466	-2.7387	0.52	0.28407	0.89885 (max o	7	4952; 4579; 4391; 24	-0.4188847	0.5633461		1.0
	ZINC31158310	-8.1	448.379	-0.3766	11	7	299.19	0.45035	186.37	-5.9015	914.0113514704	0.2589	6.4156	-1.4546	0.4375	0.32223	0.91243	1	658	0.12661187	0.628125		1.0
	ZINC32124053	-8.1	361.397	0.6417	8	1	264.92	0.28175	80.34	6.0985	914.0113514704	0.31865	5.3973	2.0138	0.46154	0.42332		0		0.7925489	-0.5620389		1.0
	ZINC34054657	-8.1	352.425	0.9315	6	3	253.92	0.29687	104.06	-1.3444	914.0113514704	0.33139	5.1075	2.8109	0.44	0.46196	0.93686	1	4815	0.3182047	-0.728944		1.0
	ZINC34554785	-8.1	381.380	-1.4059	9	4	270.71	0.39467	134.55	-8.4237	914.0113514704	0.30685	7.4449	-4.5818	0.44444	0.40473	0.93996 (max o	3	4816; 3479; 2647	0.64632696	-0.3706296		1.0
	ZINC34554787	-8.1	381.380	-1.4059	9	4	270.71	0.39467	134.55	-8.4237	914.0113514704	0.30685	7.4449	-4.5818	0.44444	0.40473	0.93996 (max o	3	4816; 2647; 3478	0.62863594	-0.3986517		1.0
	ZINC35442545	-8.1	421.560	-1.5447	8	2	331.29	0.25739	83.39	3.7513	914.0113514704	0.27616	7.5837	-5.5935	0.6	0.52196	0.89253 (max o	2	6320; 2281	0.76992655	0.15723957		1.0
	ZINC35442584	-8.1	423.560	-2.1392	9	3	324.33	0.36269	131.34	1.2975	914.0113514704	0.28568	8.1782	-7.488	0.58621	0.49809	0.96931 (max o	9	7085; 5998; 5586; 55	-0.0483194	0.5404037		1.0
	ZINC35454535	-8.1	361.420	-4.0081	8	2	266.36	0.30845	106.59	5.9766	914.0113514704	0.31865	10.047	-12.578	0.61538	0.49758	0.87695 (max o	3	3662; 446; 1421	-0.2869932	0.0345986		1.0
	ZINC35614469	-8.1	434.511	-1.1599	8	4	316.97	0.35899	155.41	-7.2548	914.0113514704	0.27616	7.1989	-4.2001	0.43333	0.57119	0.96589 (max o	5	7096; 7093; 6676; 63	-0.6603278	-0.1317180		1.0
	ZINC38896804	-8.1	392.434	0.94	7	1	287.92	0.2327	91.37	2.7041	914.0113514704	0.28568	5.099	3.2903	0.58621	0.49609	0.90236	1	2345	-0.6620301	0.7326773		1.0
	ZINC49181368	-8.1	420.366	-3.3769	12	6	279.72	0.50636	184.6	-0.17686	914.0113514704	0.28568	9.4159	-11.82	0.51724	0.49453		0		0.11049465	-0.9824140		1.0
	ZINC59410029	-8.1	438.482	0.4636	9	0	314.85	0.25558	94.38	3.4006	914.0113514704	0.2589	5.5754	1.7906	0.40625	0.49873		0		-0.6021379	0.57278395		1.0
	ZINC67870854	-8.1	388.514	-0.018	8	1	301.51	0.22251	73.83	7.1747	914.0113514704	0.29589	6.057	-0.060834	0.67857	0.54544	0.83798 (max o	2	3537; 3536	-0.1110503	0.7564578		1.0
	ZINC67870854	-8.1	389.522	-2.1277	8	2	315.55	0.25711	75.03	4.1904	914.0113514704	0.29589	8.1667	-7.1909	0.67857	0.51954	0.83798	1	3535	-0.1323683	0.73081374		1.0
	ZINC67870854	-8.1	389.522	-2.1277	8	2	315.55	0.25711	75.03	6.6787	914.0113514704	0.29589	8.1667	-7.1909	0.67857	0.56298	0.87244 (max o	2	2239; 3535	-0.0728876	0.75523925		1.0
	ZINC67912688	-8.1	396.434	-1.0197	8	5	255.49	0.39759	144.52	-2.8974	914.0113514704	0.29589	7.0587	-3.4462	0.35714	0.39239	0.93985 (max o	3	4434; 3954; 3541	0.2612309	-0.8537419		1.0
	ZINC67912692	-8.1	396.434	-1.0197	8	5	255.49	0.39759	144.52	-2.8974	914.0113514704	0.29589	7.0587	-3.4462	0.35714	0.39239	0.93985 (max o	3	4434; 3954; 3540	0.24605033	-0.8403105		1.0
	ZINC67912700	-8.1	366.408	-0.5383	7	4	237.47	0.37259	124.29	0.78517	914.0113514704	0.31865	6.5773	-1.6893	0.34615	0.32626	0.92702 (max o	4	1066; 1619; 2316; 31	0.32289767	0.6370122		1.0
	ZINC72320164	-8.1	434.524	-0.8391	9	7	299.02	0.37355	160.07	-4.7016	914.0113514704	0.27616	6.8781	-3.0384	0.36667	0.44569	0.96092 (max o	3	5682; 5195; 4803	-0.5056388	-0.7137012		1.0
	ZINC02123345	-8.0	403.410	-1.661	9	2	310.24	0.3525	133.86	-2.3536	1085.195598354	0.28216	7.6255	-5.8868	0.55172	0.49252	0.96931 (max o	5	868; 1092; 1355; 314	0.23201919	-0.0228706		172.0
	ZINC02091937	-8.0	375.356	-2.4735	9	2	280.93	0.38928	133.86	-0.83321	1085.195598354	0.30306	8.438	-8.1618	0.62963	0.55071	0.96617 (max o	7	3579; 863; 1643; 197	0.22209525	-0.0060955		172.0
	ZINC02137167	-8.0	389.383	-2.1154	9	2	296.48	0.36886	133.86	-0.45242	1085.195598354	0.29223	8.0799	-7.2387	0.60714	0.48989	0.97037 (max o	9	4004; 1356; 1976; 19	0.26217985	-0.0097961		172.0
	ZINC02100599	-8.0	403.410	-1.661	9	2	310.24	0.3525	133.86	-2.3536	1085.195598354	0.28216	7.6255	-5.8868	0.62069	0.49252	0.98037 (max o	11	4007; 4004; 3581; 35	0.28859434	-0.0299921		172.0
	ZINC02117006	-8.0	431.440	-2.3745	9	1	332.57	0.33731	144.89	-2.6702	1085.195598354	0.26395	8.339	-8.9959	0.58065	0.46206	0.97642 (max o	6	3575; 1362; 2352; 27	0.30436784	0.06728277		172.0
	ZINC02126055	-8.0	417.413	-2.8289	9	1	318.81	0.35187	144.89	-2.6493	1085.195598354	0.27275	8.7934	-10.372	0.56667	0.46707	0.97533 (max o	5	1362; 2724; 2730; 31	0.30146536	0.10382025		172.0
	ZINC02120334	-8.0	403.410	-1.7866	9	2	308.98	0.35394	133.86	2.3899	1085.195598354	0.28216	7.7511	-6.3319	0.58621	0.57702	0.96881 (max o	9	4022; 3571; 870; 198	0.16324446	-0.0511844		172.0
	ZINC02137456	-8.0	361.329	-2.8328	9	2	269.93	0.40514	133.86	-1.7607	1085.195598354	0.31471	8.7973	-9.0012	0.65385	0.55616	0.96389 (max o	4	2142; 2351; 3133; 35	0.20196679	-0.0413299		172.0
	ZINC02146401	-8.0	404.398	-2.173	10	3	308	0.40464	159.88	0.70764	1085.195598354	0.28216	8.1375	-7.7014	0.62069	0.54997	0.95123 (max o	5	4017; 4006; 1085; 19	0.24280702	0.11988439		172.0
	ZINC02121826	-8.0	417.436	-1.3322	9	2	322.74	0.33885	133.86	0.49078	1085.195598354	0.27275	7.2967	-4.8843	0.6	0.5782	0.96881 (max o	6	4473; 4022; 870; 108	0.1826966	-0.0461648		172.0
	ZINC02155714	-8.0	404.398	-1.9604	10	4	302.56	0.42216	170.88	2.7113	1085.195598354	0.28216	7.9249	-6.9479	0.62069	0.56051	0.96211 (max o	7	4076; 3634; 1087; 19	-0.0094033	0.18985489		172.0
	ZINC02146550	-8.0	389.383	-2.0203	9	2	299.24	0.36546	133.86	-3.3869	1085.195598354	0.29223	7.9848	-6.9133	0.64286	0.49055	0.97941 (max o	7	4473; 4007; 4003; 19	0.24351563	-0.0069727		172.0
	ZINC02126625	-8.0	361.329	-2.8186	9	2	273.22	0.40026	133.86	-1.4895	1085.195598354	0.31471	8.7831	-8.9561	0.65385	0.56722	0.96389 (max o	6	4903; 4473; 2360; 27	0.24988921	-0.0791505		172.0
	ZINC04073354	-8.0	394.423	-2.6399	8	2	286.05	0.38693	152.04	1.8042	1085.195598354	0.30306	8.6044	-8.7109	0.59259	0.55345	0.99202 (max o	4	4076; 3140; 3564; 35	-0.0390913	0.20997842		172.0
	ZINC02109808	-8.0	394.423	-2.6399	8	2	286.05	0.38693	152.04	1.8042	1085.195598354	0.30306	8.6044	-8.7109	0.59259	0.55122	0.99202 (max o	4	4076; 3634; 3584; 31	-0.0305662	0.19091132		172.0
	ZINC02118064	-8.0	423.828	-1.4224	9	3	305.2	0.36848	144.86	4.8568	1085.195598354	0.28216	7.3869	-5.0412	0.55172	0.59732	0.9339 (max of	6	5304; 4899; 4465; 10	0.08588315	0.00710252		172.0

Structure	Molecule Name	affinity	Total Molweight	cLogP	H-Acceptors	H-Donors	Total Surface Area	Relative PSA	Polar Surface Area	Druglikeness	Kd (nM) @ 20°C	LE from K...	LLE from Kd ...	LELP from Kd (n...	Shape Index	Molecular...	Neighbor Si...	Neig...	Neighbor	Neighbor ...	Neighbor ...	Ring Systems	frequency
	ZINC02113067	-8.0	395.774	-2.1787	9	2	285.35	0.38325	133.86	1.3311	1085.195598354	0.30306	8.1432	-7.189	0.62963	0.57002	0.95228 (max o	6	5702; 5300; 4467; 44	0.10288583	0.09719545		172.0
	ZINC02123398	-8.0	434.424	-2.2091	11	3	326.97	0.41175	169.11	2.792	1085.195598354	0.26395	8.1736	-8.3693	0.58065	0.56795	0.92304 (max o	8	6925; 5306; 4010; 35	0.06300127	0.15827133		172.0
	ZINC02120951	-8.0	391.355	-2.8547	10	2	292.19	0.4085	143.09	1.2511	1085.195598354	0.29223	8.8192	-9.7685	0.60714	0.57178	0.9399 (max of	5	5700; 4467; 4466; 40	0.03577275	0.06344174		172.0
	ZINC02160326	-8.0	434.424	-2.2091	11	3	326.97	0.41175	169.11	2.792	1085.195598354	0.26395	8.1736	-8.3693	0.58065	0.56613	0.92304 (max o	7	6924; 5749; 5701; 40	0.02419586	0.15988383		172.0
	ZINC02122925	-8.0	362.357	-1.1247	8	1	273.98	0.34623	113.99	4.8454	1085.195598354	0.31471	7.0892	-3.5737	0.53846	0.51328	0.96438 (max o	7	6925; 5307; 5306; 44	0.08200541	0.14550179		172.0
	ZINC02114384	-8.0	391.355	-2.8547	10	2	292.19	0.4085	143.09	1.2511	1085.195598354	0.29223	8.8192	-9.7685	0.60714	0.574	0.92442 (max o	7	6385; 6076; 5307; 50	0.08698355	0.06966156		172.0
	ZINC02159644	-8.0	376.384	-0.9064	8	1	284.98	0.33287	113.99	3.9973	1085.195598354	0.30306	6.8709	-2.9908	0.51852	0.51073	0.95372 (max o	7	7120; 5709; 5699; 49	0.05470223	0.09773101		172.0
	ZINC03869382	-8.0	384.416	-3.7275	11	4	265.34	0.56735	212.38	-0.44329	1085.195598354	0.31471	9.692	-11.844	0.57692	0.48318	0.96775 (max o	8	6015; 5788; 5728; 45	0.56989706	0.1647028		67.0
	ZINC04514038	-8.0	370.389	-4.1819	11	4	251.58	0.59838	212.38	-0.65956	1085.195598354	0.3273	10.146	-12.777	0.56	0.46938	0.96775 (max o	7	6015; 5788; 5728; 54	0.609599	0.15766051		67.0
	ZINC04228234	-8.0	423.170	-13.388	15	2	266.13	0.71134	263.54	-22.848	1085.195598354	0.30306	19.352	-44.176	0.44444	0.42067	0.92739 (max o	9	6403; 6098; 6094; 57	0.637713	0.46991313		67.0
	ZINC04228234	-8.0	424.178	-12.14	15	3	264.95	0.71006	260.71	-32.8	1085.195598354	0.30306	18.105	-40.06	0.44444	0.40268	0.92739 (max o	9	6404; 6099; 6095; 57	0.6098171	0.5207888		67.0
	ZINC31413936	-8.0	380.768	-1.513	12	4	237.34	0.61136	197.08	-1.9876	1085.195598354	0.34094	7.4775	-4.4377	0.5	0.38826	0.86507	1	7786	-0.3516691	-0.8812156		67.0
	ZINC12494556	-8.0	381.300	-7.0703	13	4	260.93	0.59472	205.81	1.1475	1085.195598354	0.30306	13.035	-23.33	0.51852	0.38004		0		0.14605959	-0.9701985		67.0
	ZINC31167905	-8.0	444.431	-0.9145	11	5	319.48	0.41186	172.21	-7.1849	1085.195598354	0.26395	6.879	-3.4646	0.6129	0.60414	1 (max of 3)	3	6298; 5610; 4380	0.2959103	-0.733101		49.0
	ZINC77257566	-8.0	446.447	-1.1579	11	6	319.08	0.41256	175.37	-5.239	1085.195598354	0.26395	7.1224	-4.3868	0.54839	0.4628	0.99343 (max o	5	6682; 6376; 6065; 60	0.07279783	0.5087895		49.0
	ZINC04073587	-8.0	384.451	-1.4801	9	2	299.76	0.34331	127.87	-45.76	1085.195598354	0.30306	7.4446	-4.8839	0.59259	0.62053	0.91482 (max o	3	6019; 5748; 5074	0.5187938	-0.1188089		47.0
	ZINC17028944	-8.0	361.377	-2.3554	9	3	271.01	0.38511	130.67	5.6323	1085.195598354	0.31471	8.3199	-7.4843	0.5	0.53855	0.9615 (max of	10	1009; 1221; 1809; 22	0.01912620	-0.6249882		42.0
	ZINC13548227	-8.0	361.377	-2.3554	9	3	271.01	0.38511	130.67	5.6323	1085.195598354	0.31471	8.3199	-7.4843	0.5	0.53865	0.9615 (max of	10	5165; 3823; 1009; 12	-0.0037923	-0.5952030		42.0
	ZINC12889868	-8.0	405.430	-2.4684	10	4	303.62	0.3869	150.9	5.7254	1085.195598354	0.28216	8.4329	-8.7483	0.44828	0.55387	0.9892 (max of	12	3822; 1565; 1808; 18	0.00217122	-0.6157224		42.0
	ZINC17028736	-8.0	405.430	-2.4684	10	4	303.62	0.3869	150.9	5.5613	1085.195598354	0.28216	8.4329	-8.7483	0.44828	0.55169	0.9892 (max of	11	4528; 1565; 2252; 26	-0.0417607	-0.6372455		42.0
	ZINC12889875	-8.0	405.430	-2.4684	10	4	303.62	0.3869	150.9	5.7254	1085.195598354	0.28216	8.4329	-8.7483	0.44828	0.55387	0.9892 (max of	12	5166; 5165; 3822; 15	-0.0138633	-0.6545818		42.0
	ZINC04090870	-8.0	405.430	-2.4684	10	4	303.62	0.3869	150.9	5.7254	1085.195598354	0.28216	8.4329	-8.7483	0.44828	0.55607	0.9892 (max of	12	5166; 5165; 3822; 37	-7.6670985	-0.6721304		42.0
	ZINC08876713	-8.0	432.432	-4.3445	11	3	325.31	0.40481	170.8	6.9913	1085.195598354	0.26395	10.309	-16.459	0.48387	0.56679	0.97856 (max o	11	5166; 5165; 5077; 43	0.07486307	-0.6534020		42.0
	ZINC08585032	-8.0	401.136	-11.987	14	3	246.85	0.70557	240.5	-46.18	1085.195598354	0.3273	17.952	-36.625	0.56	0.61685	0.93348 (max o	6	5089; 1460; 2871; 29	-0.5189636	-0.4250755		41.0
	ZINC08585032	-8.0	402.144	-10.74	14	4	245.67	0.70416	237.67	-50.91	1085.195598354	0.3273	16.704	-32.813	0.56	0.61705	0.93348 (max o	5	5088; 1459; 3729; 28	-0.5520924	-0.3929918		41.0
	ZINC36367779	-8.0	438.169	0.0808	9	1	249.79	0.38901	111.24	3.9348	1085.195598354	0.35576	5.8837	0.22712	0.52174	0.48074	0.92193 (max o	3	7759; 6544; 6083	0.6912983	0.55101895		41.0
	ZINC20758493	-8.0	426.472	-0.7877	8	2	313.54	0.35211	143.94	5.5822	1085.195598354	0.27275	6.7522	-2.888	0.43333	0.51304	0.9702 (max of	13	4471; 4328; 1261; 13	0.6686625	-0.1037601		36.0
	ZINC08876275	-8.0	440.499	-0.3333	8	2	327.3	0.33731	143.94	5.5165	1085.195598354	0.26395	6.2978	-1.2627	0.45161	0.51625	0.9702 (max of	11	4471; 4328; 3859; 12	0.65573627	-0.1205431		36.0
	ZINC12885820	-8.0	424.432	-1.146	9	2	312.24	0.31418	130.08	3.0071	1085.195598354	0.26395	7.1105	-4.3417	0.3871	0.49984	0.99016 (max o	12	5034; 4962; 4522; 40	0.6988922	0.00213165		36.0
	ZINC04085486	-8.0	394.406	-0.5786	8	1	293.39	0.28972	109.85	3.573	1085.195598354	0.28216	6.5431	-2.0506	0.41379	0.49528	0.96682 (max o	12	5034; 4962; 4522; 40	0.7203391	-0.0045469		36.0
	ZINC05606962	-8.0	364.313	-2.0709	12	4	226.18	0.64152	197.08	-6.7093	1085.195598354	0.34094	8.0354	-6.0741	0.5	0.36859	0.91891 (max o	3	5429; 4136; 2093	-0.7524994	0.60788846		33.0
	ZINC03869381	-8.0	384.416	-3.7275	11	4	265.34	0.56735	212.38	-0.44329	1085.195598354	0.31471	9.692	-11.844	0.57692	0.47164	0.96775 (max o	7	5728; 5405; 4556; 39	0.5768951	0.20296194		33.0
	ZINC04492892	-8.0	351.362	-0.6727	10	5	254.95	0.44573	145.78	-0.9947	1085.195598354	0.3273	6.6372	-2.0553	0.6	0.36445	0.92315 (max o	6	4941; 4942; 4768; 43	-0.4352396	-0.6194910		33.0
	ZINC37253577	-8.0	382.400	-3.9574	12	5	268.08	0.55032	214.72	-1.7419	1085.195598354	0.30306	9.9219	-13.058	0.55556	0.47161	0.93373 (max o	5	6015; 5841; 4556; 36	0.55246365	0.15240979		33.0
	ZINC37253583	-8.0	382.400	-3.9574	12	5	268.08	0.55032	214.72	-1.7419	1085.195598354	0.30306	9.9219	-13.058	0.55556	0.47161	0.93373 (max o	5	6015; 5841; 5405; 27	0.5949765	0.17136349		33.0
	ZINC04090229	-8.0	404.466	-1.8023	8	1	294.16	0.34842	135.15	6.3348	1085.195598354	0.29223	7.7668	-6.1673	0.57143	0.51837	0.96519 (max o	14	4091; 3749; 1220; 14	0.13143429	-0.8823088		29.0
	ZINC04090368	-8.0	402.426	-1.9694	9	2	298.28	0.32889	130.08	-1.1293	1085.195598354	0.28216	7.9339	-6.9798	0.58621	0.50153	0.95345 (max o	10	5464; 4632; 4091; 37	0.16282354	-0.9217438		29.0
	ZINC08876682	-8.0	432.519	-0.8935	8	1	321.68	0.31861	135.15	-1.8804	1085.195598354	0.27275	6.858	-3.2759	0.6	0.51226	0.95345 (max o	10	5464; 4632; 4091; 21	0.16565822	-0.9025557		29.0
	ZINC38570179	-8.0	374.416	-1.2164	8	2	289.56	0.32183	112.49	4.9671	1085.195598354	0.30306	7.1809	-4.0138	0.55556	0.41951	0.89253 (max o	3	4949; 1614; 2300	-0.1869872	0.54541236		28.0
	ZINC06031801	-8.0	375.349	-4.4746	10	5	249.52	0.49223	168.97	-1.6392	1085.195598354	0.31471	10.439	-14.218	0.46154	0.37855	0.92702 (max o	4	1700; 2023; 3121; 32	-0.654812	0.04885725		25.0
	ZINC06032117	-8.0	390.383	-1.942	10	5	263.42	0.45	155.14	-4.365	1085.195598354	0.30306	7.9065	-6.408	0.48148	0.37688	0.98371 (max o	4	3919; 1890; 2626; 26	-0.6349213	0.03975406		25.0
	ZINC08964849	-8.0	445.435	1.6712	13	5	321.67	0.45798	182.39	1.4288	1085.195598354	0.25571	4.2933	6.5356	0.5	0.3389	0.95739 (max o	7	4190; 1470; 1784; 25	-0.1357746	-0.6831179		24.0
	ZINC08964845	-8.0	401.382	1.4655	12	6	283.5	0.49531	184.16	1.3234	1085.195598354	0.28216	4.499	5.1939	0.48276	0.31555	0.97649 (max o	13	6205; 4627; 4618; 42	-0.2083859	-0.6801681		24.0

Structure	Molecule Name	affinity	Total Molweight	cLogP	H-Acceptors	H-Donors	Total Surface Area	Relative PSA	Polar Surface Area	Druglikeness	Kd (nM) @ 20°C	LE from K...	LLE from Kd ...	LELP from Kd (n...	Shape Index	Molecular...	Neighbor Si...	Neig...	Neighbor	Neighbor ...	Neighbor ...	Ring Systems	frequency
	ZINC04037732	-8.0	401.370	-3.7031	9	1	291.26	0.38515	144.89	1.9281	1085.195598354	0.28216	9.6676	-13.124	0.48276	0.40382	0.97176 (max o	3	426; 2401; 2402	0.16865698	0.11260833		24.0
	ZINC13507420	-8.0	406.394	-2.6337	11	6	283.25	0.45758	175.28	6.4914	1085.195598354	0.28216	8.5982	-9.3341	0.44828	0.48702	0.97037 (max o	15	4912; 4908; 4896; 41	0.44875094	0.75937015		24.0
	ZINC04353342	-8.0	406.394	-2.6337	11	6	283.25	0.45758	175.28	6.4914	1085.195598354	0.28216	8.5982	-9.3341	0.44828	0.48571	0.97288 (max o	21	6010; 5875; 5314; 52	0.4755962	0.776532		24.0
	ZINC35653108	-8.0	405.410	-2.5138	11	5	286.96	0.41839	158.29	6.8607	1085.195598354	0.28216	8.4783	-8.9092	0.41379	0.49847	0.97176 (max o	5	6010; 4842; 4417; 16	0.45089695	0.8266193		24.0
	ZINC72320113	-8.0	372.456	0.5602	7	4	276.88	0.30858	116.45	-6.4652	1085.195598354	0.31471	5.4043	1.78	0.53846	0.44971	0.96998 (max o	3	4879; 4878; 4424	-0.0541702	0.64469004		23.0
	ZINC72319913	-8.0	406.470	-1.0841	9	7	287.33	0.38875	160.07	-4.0127	1085.195598354	0.29223	7.0486	-3.7097	0.46429	0.44224	0.95148 (max o	7	6916; 6678; 6680; 63	0.07070169	-0.7365409		23.0
	ZINC72319912	-8.0	406.470	-1.0841	9	7	287.33	0.38875	160.07	-4.0127	1085.195598354	0.29223	7.0486	-3.7097	0.46429	0.44224	0.94862 (max o	7	6916; 6678; 6680; 63	0.06517714	-0.757593		23.0
	ZINC19327778	-8.0	357.469	-2.4049	7	5	264.45	0.3274	105.67	3.1491	1085.195598354	0.3273	8.3694	-7.3476	0.44	0.61182	0.97118 (max o	4	7372; 7201; 6798; 65	-0.2578236	0.7373427		23.0
	ZINC12881094	-8.0	446.478	-1.5067	10	2	333.59	0.33847	137.1	-0.0023535	1085.195598354	0.25571	7.4712	-5.8923	0.5625	0.47785	0.97533 (max o	11	6226; 5250; 4194; 41	0.55688447	0.20655024		22.0
	ZINC38570249	-8.0	446.478	-1.5067	10	2	333.59	0.33847	137.1	-1.1357	1085.195598354	0.25571	7.4712	-5.8923	0.53125	0.46457	0.971 (max of 1	13	6226; 5250; 4194; 41	0.5202414	0.19861084		22.0
	ZINC08624261	-8.0	379.455	-0.1875	8	4	290.24	0.30578	111.13	2.9004	1085.195598354	0.30306	6.152	-0.61869	0.62963	0.57264	0.93373 (max o	10	6476; 5021; 5018; 46	0.36669326	-0.3511934		22.0
	ZINC12603870	-8.0	380.463	-2.1721	8	5	307.04	0.33478	112.33	0.71839	1085.195598354	0.30306	8.1366	-7.1673	0.66667	0.58386	0.94002 (max o	7	5455; 5021; 4614; 42	0.35988945	-0.4205146		22.0
	ZINC08964848	-8.0	445.435	1.6712	13	5	321.67	0.45798	182.39	1.4288	1085.195598354	0.25571	4.2933	6.5356	0.5	0.3376	0.95739 (max o	7	4625; 4622; 4189; 17	-0.1228718	-0.6478332		20.0
	ZINC08964846	-8.0	445.435	1.6712	13	5	321.67	0.45798	182.39	1.4288	1085.195598354	0.25571	4.2933	6.5356	0.5	0.3376	0.95739 (max o	7	5458; 4623; 4619; 17	-0.1422884	-0.6181327		20.0
	ZINC08964830	-8.0	401.382	1.4655	12	6	283.5	0.49531	184.16	1.3234	1085.195598354	0.28216	4.499	5.1939	0.48276	0.33438	0.95642 (max o	13	6759; 6484; 6205; 61	-0.1793061	-0.5934731		20.0
	ZINC13431047	-8.0	401.160	-11.29	14	4	247.95	0.70688	246.95	-50.931	1085.195598354	0.3273	17.255	-34.495	0.56	0.61192	0.92662 (max o	10	5088; 5086; 4674; 62	-0.572847	-0.4171696		20.0
	ZINC13431047	-8.0	400.152	-12.538	14	3	249.13	0.70826	249.78	-46.201	1085.195598354	0.3273	18.502	-38.307	0.56	0.61172	0.92662 (max o	10	5087; 5089; 4676; 62	-0.5768407	-0.4612472		20.0
	ZINC12495268	-8.0	400.152	-12.538	14	3	249.13	0.70826	249.78	-46.201	1085.195598354	0.3273	18.502	-38.307	0.56	0.60915	0.91611 (max o	8	5087; 5089; 5057; 37	-0.5646178	-0.4777015		20.0
	ZINC13435048	-8.0	385.161	-10.438	13	3	242.86	0.66775	226.72	-47.338	1085.195598354	0.34094	16.403	-30.616	0.58333	0.61972	0.94378 (max o	8	5088; 5056; 4675; 62	-0.6137973	-0.4509105		20.0
	ZINC13435048	-8.0	384.153	-11.686	13	2	244.04	0.66936	229.55	-42.608	1085.195598354	0.34094	17.65	-34.275	0.58333	0.61952	0.94378 (max o	8	5089; 5057; 4676; 62	-0.5976188	-0.4916969		20.0
	ZINC12495268	-8.0	401.160	-11.29	14	4	247.95	0.70688	246.95	-50.931	1085.195598354	0.3273	17.255	-34.495	0.56	0.60935	0.91611 (max o	7	5088; 5086; 5056; 37	-0.589573	-0.4231444		20.0
	ZINC31154969	-8.0	417.413	-0.5893	9	5	292.39	0.3864	153.39	0.85416	1085.195598354	0.27275	6.5538	-2.1606	0.53333	0.41819	0.95318 (max o	5	1512; 2559; 2967; 33	-0.3146863	-0.6998405		19.0
	ZINC48056993	-8.0	382.273	-8.8812	13	5	246.07	0.65079	218.33	-2.9931	1085.195598354	0.31471	14.846	-28.22	0.46154	0.43576	0.94951 (max o	3	6663; 6033; 3950	0.20078598	0.9206875		19.0
	ZINC48056990	-8.0	382.273	-8.8812	13	5	246.07	0.65079	218.33	-2.9931	1085.195598354	0.31471	14.846	-28.22	0.46154	0.43405	0.94951 (max o	3	6663; 6033; 3951	0.16227987	0.9233279		19.0
	ZINC71404533	-8.0	409.386	-3.6382	12	7	280.11	0.51858	202.32	-14.737	1085.195598354	0.29223	9.6027	-12.45	0.42857	0.47846	0.95148 (max o	4	7275; 6054; 5411; 35	0.54379857	-0.4996524		19.0
	ZINC08636315	-8.0	425.504	-0.0914	9	4	310.65	0.39002	146.94	3.6911	1085.195598354	0.28216	6.0559	-0.32393	0.55172	0.45482	0.9398 (max of	3	1194; 3735; 2884	-0.5444032	0.38343522		17.0
	ZINC08636318	-8.0	393.506	0.7364	7	4	290.65	0.34805	128.48	3.6811	1085.195598354	0.30306	5.2281	2.4299	0.55556	0.4258	0.93367 (max o	8	7334; 5483; 4610; 42	-0.5219480	0.3887447		17.0
	ZINC08254657	-8.0	379.479	0.3925	7	4	278.39	0.36338	128.48	3.6811	1085.195598354	0.31471	5.572	1.2472	0.61538	0.42094	0.97533 (max o	9	7334; 5483; 4610; 46	-0.480438	0.38460857		17.0
	ZINC08585030	-8.0	401.136	-11.987	14	3	246.85	0.70557	240.5	-46.18	1085.195598354	0.3273	17.952	-36.625	0.56	0.61722	0.92662 (max o	6	5087; 3729; 1460; 28	-0.5543483	-0.4135733		17.0
	ZINC08585030	-8.0	402.144	-10.74	14	4	245.67	0.70416	237.67	-50.91	1085.195598354	0.3273	16.704	-32.813	0.56	0.61742	0.92662 (max o	5	5086; 1459; 3728; 28	-0.5698703	-0.3724784		17.0
	ZINC03871881	-8.0	376.388	-3.4633	8	1	256.5	0.4414	154.97	10.967	1085.195598354	0.31471	9.4278	-11.005	0.5	0.35316	0.96721 (max o	5	5346; 4635; 706; 196	0.06596152	-0.0531583		17.0
	ZINC19850111	-8.0	364.401	-4.0789	8	3	246.09	0.46434	161.09	9.1306	1085.195598354	0.3273	10.043	-12.462	0.56	0.40396	0.9654 (max of	4	5120; 4700; 3878; 31	0.14324014	-0.1350430		17.0
	ZINC04025278	-8.0	370.312	-2.3239	8	0	267.35	0.36536	125.02	-0.16309	1085.195598354	0.30306	8.2884	-7.6682	0.44444	0.31384	0.98371 (max o	3	3186; 3187; 3188	-0.1707076	0.7497729		16.0
	ZINC72399611	-8.0	444.011	-2.8381	8	6	314.2	0.33256	123.69	0.91283	1085.195598354	0.29223	8.8026	-9.7117	0.5	0.49672	0.92757 (max o	4	7679; 7460; 6930; 68	0.68036056	-0.2788120		15.0
	ZINC20763623	-8.0	376.388	-1.702	9	1	288.62	0.3462	120.36	6.6121	1085.195598354	0.30306	7.6665	-5.6161	0.55556	0.5537	0.96617 (max o	4	7063; 5173; 3422; 34	0.03882332	-0.3369091		15.0
	ZINC13136668	-8.0	432.380	-0.078	10	7	289.19	0.43134	177.14	-1.9977	1085.195598354	0.26395	6.0425	-0.29551	0.51613	0.3811	0.96262 (max o	11	294; 832; 967; 1038;	0.6994872	0.10015571		14.0
	ZINC08299533	-8.0	388.446	-4.3112	9	3	289.83	0.33351	126.46	3.6259	1085.195598354	0.29223	10.276	-14.753	0.5	0.51462	0.96253 (max o	6	4132; 762; 936; 1189;	0.09041751	0.31221578		14.0
	ZINC02150586	-8.0	415.421	-1.7234	9	2	308.95	0.35397	133.86	-1.7907	1085.195598354	0.27275	7.6879	-6.3186	0.6	0.50548	0.8872 (max of	3	210; 862; 1084	0.17643446	0.06895331		14.0
	ZINC02104029	-8.0	401.394	-2.0827	9	2	297.95	0.36704	133.86	-3.3392	1085.195598354	0.28216	8.0472	-7.3813	0.62069	0.53202	0.96931 (max o	4	868; 1643; 1644; 235	0.26886246	-0.0302600		14.0
	ZINC04027033	-8.0	374.344	-2.9068	8	0	272.05	0.35905	125.02	-4.0072	1085.195598354	0.30306	8.8713	-9.5916	0.44444	0.37101	0.98371 (max o	3	3190; 3191; 3627	-0.0796807	0.9616739		14.0
	ZINC04027032	-8.0	374.344	-2.9068	8	0	272.05	0.35905	125.02	-4.0072	1085.195598354	0.30306	8.8713	-9.5916	0.44444	0.37101	0.98371 (max o	3	3628; 3190; 3191	-0.1165576	0.9525468		14.0
	ZINC08643376	-8.0	381.427	-0.6042	9	5	291.9	0.3654	129.15	0.36871	1085.195598354	0.30306	6.5687	-1.9937	0.7037	0.58854	0.94002 (max o	7	7332; 5024; 5016; 61	0.27865115	-0.4171792		14.0
	ZINC20463863	-8.0	418.434	-0.4298	7	2	284.23	0.20385	71.01	-1.1656	1085.195598354	0.28216	6.3943	-1.5233	0.58621	0.52884	0.94241 (max o	7	7065; 6847; 6503; 59	0.43472123	-0.2109022		14.0

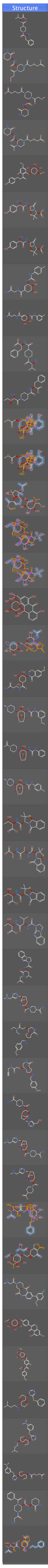

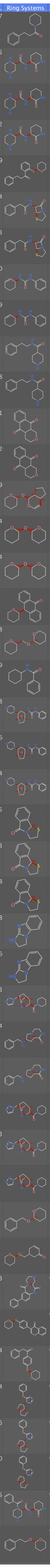

Structure	Molecule Name	affinity	Total Molweight	cLogP	H-Acceptors	H-Donors	Total Surface Area	Relative PSA	Polar Surface Area	Druglikeness	Kd (nM) @ 20°C	LE from K...	LLE from Kd ...	LELP from Kd (n...	Shape Index	Molecular...	Neighbor Si...	Neig...	Neighbor	Neighbor ...	Neighbor ...	Ring Systems	frequency
	ZINC04064795	-8.0	375.467	-0.9638	7	2	293.35	0.28256	117.18	4.9182	1085.195598354	0.30306	6.9283	-3.1802	0.59259	0.52005	0.95174 (max o	7	6423; 4584; 4519; 44	0.32288295	0.24659987		14.0
	ZINC05413938	-8.0	429.472	-4.4646	11	2	314.89	0.43504	173.48	5.4283	1085.195598354	0.28216	10.429	-15.823	0.51724	0.62313	0.9892 (max of	3	7179; 3777; 3689	0.03352739	0.47167575		13.0
	ZINC12605312	-8.0	411.433	-3.7476	11	2	311.16	0.38405	148.18	0.59247	1085.195598354	0.28216	9.7121	-13.282	0.51724	0.58034	0.96682 (max o	2	7179; 3690	0.05256503	0.4807012		13.0
	ZINC05413935	-8.0	399.447	-3.9658	10	2	291.13	0.4362	164.25	5.6951	1085.195598354	0.30306	9.9303	-13.086	0.55556	0.62033	0.93686 (max o	3	7179; 6741; 3690	-0.0130729	0.45313722		13.0
	ZINC31164435	-8.0	449.431	-1.2473	10	5	322.59	0.38073	168.97	-4.6088	1085.195598354	0.25571	7.2118	-4.8779	0.5	0.43647		0		0.3873433	-0.8697229		13.0
	ZINC03604145	-8.0	365.409	-1.8794	8	3	245.3	0.46262	162.71	9.2567	1085.195598354	0.3273	7.8439	-5.7421	0.56	0.38362	0.90896 (max o	4	4025; 3878; 3160; 31	0.08760818	-0.1302894		12.0
	ZINC28630997	-8.0	364.401	-4.0789	8	3	246.09	0.46434	161.09	9.1306	1085.195598354	0.3273	10.043	-12.462	0.56	0.40396	0.90896 (max o	4	4354; 3178; 3587; 38	0.10698324	-0.1222803		12.0
	ZINC03840036	-8.0	362.385	-1.1379	9	6	270.6	0.42487	153.78	5.0915	1085.195598354	0.31471	7.1024	-3.6157	0.61538	0.53373	0.93686 (max o	6	1662; 2053; 2429; 27	-0.1948680	-0.5810770		12.0
	ZINC03840158	-8.0	380.375	-0.9266	9	6	278.45	0.41289	153.78	2.6926	1085.195598354	0.30306	6.8911	-3.0575	0.59259	0.53962	0.96389 (max o	6	4033; 1675; 1710; 20	-0.2433236	-0.5541269		12.0
	ZINC04082740	-8.0	375.467	-0.9638	7	2	293.35	0.28256	117.18	5.1731	1085.195598354	0.30306	6.9283	-3.1802	0.59259	0.50902	0.95174 (max o	7	6121; 5490; 4663; 45	0.24971579	0.3525614		12.0
	ZINC04083408	-8.0	389.494	-0.5094	7	2	307.11	0.2699	117.18	6.1636	1085.195598354	0.29223	6.4739	-1.7431	0.60714	0.51837	0.97288 (max o	8	6121; 5490; 5448; 53	0.27083543	0.36059758		12.0
	ZINC72320618	-8.0	350.322	-0.2569	8	3	236.42	0.41629	130.36	-2.1313	1085.195598354	0.3273	6.2214	-0.7849	0.48	0.40379	0.97118 (max o	9	6679; 5279; 5277; 48	0.766784	-0.0722151		12.0
	ZINC72320620	-8.0	350.322	-0.2569	8	3	236.42	0.41629	130.36	-2.1313	1085.195598354	0.3273	6.2214	-0.7849	0.48	0.40379	0.97668 (max o	10	6679; 6058; 5279; 52	0.748871	-0.0743652		12.0
	ZINC35456531	-8.0	448.419	-2.3095	12	5	302.17	0.46854	181.44	-5.7219	1085.195598354	0.26395	8.274	-8.7496	0.41935	0.38595	0.95521 (max o	3	6329; 5233; 5232	-0.6233454	-0.7239709		12.0
	ZINC38664847	-8.0	421.267	-8.9493	14	8	261.12	0.6744	248.7	-32.77	1085.195598354	0.30306	14.914	-29.53	0.48148	0.5106	0.95201 (max o	4	6027; 5652; 5650; 39	-0.04839180	-0.4533584		12.0
	ZINC38664847	-8.0	420.259	-10.197	14	7	262.3	0.67587	251.53	-27.673	1085.195598354	0.30306	16.161	-33.647	0.48148	0.52676	0.95201 (max o	5	7943; 6026; 5651; 56	-0.03915479	-0.4728284		12.0
	ZINC14442632	-8.0	434.396	-1.4258	10	8	285	0.44856	188.14	-2.6646	1085.195598354	0.26395	7.3903	-5.4017	0.35484	0.35869	0.96018 (max o	7	6314; 5513; 5512; 46	-0.5357596	0.5534069		11.0
	ZINC13548018	-8.0	367.350	-3.5208	11	7	247.09	0.53867	178.17	-0.5245	1085.195598354	0.3273	9.4853	-10.757	0.56	0.5381		0		-0.4392972	-0.8833858		11.0
	ZINC04221892	-8.0	441.503	-1.333	10	6	318.46	0.45249	196.51	2.0283	1085.195598354	0.27275	7.2975	-4.8872	0.56667	0.51902	0.92257	1	4098	-0.4214050	-0.4163279		10.0
	ZINC22922202	-8.0	392.454	-0.2978	9	3	286.2	0.30105	95.53	6.6631	1085.195598354	0.29223	6.2623	-1.019	0.57143	0.46989	0.95709 (max o	7	5180; 4214; 1552; 38	-0.6572341	-0.2139258		10.0
	ZINC20463693	-8.0	390.438	-0.1711	9	3	281.69	0.31666	103.37	5.9186	1085.195598354	0.29223	6.1356	-0.58549	0.57143	0.46511	0.9427 (max of	6	7385; 5180; 1002; 22	-0.594349	-0.2753056		10.0
	ZINC22922187	-8.0	380.418	-0.127	8	3	270.29	0.28177	86.3	5.2818	1085.195598354	0.30306	6.0915	-0.41906	0.59259	0.45723	0.93686 (max o	7	5180; 4645; 3872; 15	-0.6417537	-0.2297124		10.0
	ZINC12883732	-8.0	363.413	-1.4073	7	2	249.83	0.39627	135.07	5.1687	1085.195598354	0.3273	7.3718	-4.2997	0.44	0.26373	0.98671 (max o	9	6409; 4523; 4245; 40	0.34052706	-0.6388995		10.0
	ZINC04084293	-8.0	404.466	-1.3969	8	2	284.95	0.38744	143.94	6.4109	1085.195598354	0.29223	7.3614	-4.7801	0.5	0.33141	0.96253 (max o	9	4524; 4245; 4085; 40	0.3745708	-0.6507983		10.0
	ZINC12883673	-8.0	363.413	-1.4073	7	2	249.83	0.39627	135.07	5.1687	1085.195598354	0.3273	7.3718	-4.2997	0.44	0.26718	0.98671 (max o	9	5356; 4524; 4523; 42	0.3539332	-0.6522788		10.0
	ZINC38571037	-8.0	402.430	-1.2294	10	3	296.97	0.37314	139.62	5.2213	1085.195598354	0.28216	7.1939	-4.3571	0.48276	0.4833	0.9892 (max of	4	1478; 2899; 2900; 30	-0.0644388	-0.8472468		10.0
	ZINC06624240	-8.0	372.404	-0.5669	9	2	280.88	0.34787	119.39	5.069	1085.195598354	0.30306	6.5314	-1.8706	0.51852	0.47809	0.93373 (max o	4	6469; 5001; 4241; 11	0.58401424	-0.2586076		10.0
	ZINC08299706	-8.0	405.545	-4.757	9	3	334.6	0.32543	102.99	2.4402	1085.195598354	0.28216	10.721	-16.859	0.62069	0.53024	0.97176 (max o	9	5719; 4599; 4178; 41	0.1704387	0.31396133		10.0
	ZINC22936972	-8.0	403.545	-0.1316	7	2	315.92	0.19746	78.49	6.1236	1085.195598354	0.28216	6.0961	-0.4664	0.62069	0.51274	0.93405 (max o	11	649; 1017; 1018; 126	-0.5197542	0.08515854		10.0
	ZINC22936919	-8.0	385.486	-1.1835	7	3	302.61	0.23228	87.28	4.2568	1085.195598354	0.29223	7.148	-4.0498	0.67857	0.47841	0.93686 (max o	5	4350; 3876; 1563; 34	-0.5042948	0.0986315		10.0
	ZINC08635779	-8.0	377.467	-3.6109	9	3	292.09	0.35742	118.78	2.5568	1085.195598354	0.30306	9.5754	-11.915	0.62963	0.50634	0.96885 (max o	8	4599; 4178; 4161; 24	0.19337721	0.3187229		10.0
	ZINC08299761	-8.0	391.518	-4.8819	9	3	305.74	0.32394	115.16	2.5202	1085.195598354	0.29223	10.846	-16.705	0.64286	0.51805	0.97037 (max o	10	4596; 4163; 4162; 37	0.20932713	0.33355963		10.0
	ZINC08299763	-8.0	391.494	-4.1803	9	2	304.75	0.32174	112.2	1.7218	1085.195598354	0.29223	10.145	-14.305	0.64286	0.53531	0.97037 (max o	5	5013; 4596; 4164; 28	0.21566072	0.2882855		10.0
	ZINC35454688	-8.0	413.397	-2.9205	10	4	298.4	0.41139	165.81	1.5959	1085.195598354	0.28216	8.885	-10.351	0.58621	0.60027	0.99848 (max o	7	6878; 6641; 6324; 43	-0.1713361	0.6888944		10.0
	ZINC35456128	-8.0	386.439	-0.2961	8	5	283.18	0.34798	136.68	-4.6035	1085.195598354	0.30306	6.2606	-0.97704	0.48148	0.53809	0.96617 (max o	5	5970; 5969; 5603; 52	-0.0850747	0.9150453		10.0
	ZINC04235952	-8.0	443.910	0.0565	8	2	316.55	0.23826	95.56	5.9729	1085.195598354	0.26395	5.908	0.21405	0.51613	0.37368	0.92326 (max o	5	4099; 346; 1713; 205	-0.2088983	0.28458223		9.0
	ZINC01806960	-8.0	432.380	-0.3621	10	6	292.84	0.41538	166.14	-3.6206	1085.195598354	0.26395	6.3266	-1.3718	0.54839	0.42904	0.97199 (max o	10	279; 365; 759; 1447;	0.2030205	0.7117047		9.0
	ZINC31166891	-8.0	449.431	-1.8552	10	5	321.33	0.38222	168.97	-3.6061	1085.195598354	0.25571	7.8197	-7.2552	0.5625	0.47999	0.971	1	1903	0.25611058	0.95805514		9.0
	ZINC04259731	-8.0	360.436	0.4991	8	1	271.3	0.28574	87.9	-0.083368	1085.195598354	0.31471	5.4654	1.5859	0.61538	0.37241	0.90502 (max o	5	1145; 1147; 2060; 28	-0.0503114	-0.2824584		9.0
	ZINC03842025	-8.0	403.505	0.3955	9	1	306.12	0.26483	91.14	0.79232	1085.195598354	0.28216	5.569	1.4017	0.58621	0.41854	0.91871 (max o	4	3661; 1147; 2060; 27	-0.0543988	-0.3040726		9.0
	ZINC04147909	-8.0	447.427	-3.3013	13	1	325.85	0.41476	152.99	1.2263	1085.195598354	0.25571	9.2658	-12.911	0.59375	0.41748	0.95983 (max o	7	4531; 1417; 1702; 17	-0.2309377	-0.3578870		9.0
	ZINC08299439	-8.0	388.446	-4.3112	9	3	289.83	0.33351	126.46	3.6259	1085.195598354	0.29223	10.276	-14.753	0.46429	0.51462	0.96253 (max o	6	4994; 936; 3722; 286	0.05669672	0.28782615		9.0
	ZINC72320406	-8.0	427.424	-2.4904	10	4	312.16	0.39326	165.81	1.3048	1085.195598354	0.27275	8.4549	-9.1306	0.56667	0.56302	0.99855 (max o	3	5284; 1948; 3546	-0.2121494	0.6790956		9.0

Structure	Molecule Name	affinity	Total Molweight	cLogP	H-Acceptors	H-Donors	Total Surface Area	Relative PSA	Polar Surface Area	Druglikeness	Kd (nM) @ 20°C	LE from K...	LLE from Kd ...	LELP from Kd (n...	Shape Index	Molecular...	Neighbor Si...	Neig...	Neighbor	Neighbor ...	Neighbor ...	Ring Systems	frequency
	ZINC31169349	-8.0	359.306	-4.0291	10	6	245.66	0.51258	179.97	-2.9939	1085.195598354	0.3273	9.9936	-12.31	0.52	0.50378	0.96243	1	5215	0.78553283	-0.5863526		9.0
	ZINC03812869	-8.0	367.812	-1.9254	7	2	243.62	0.41204	142.48	7.3145	1085.195598354	0.34094	7.8899	-5.6473	0.54167	0.41073	0.88969 (max o	3	3920; 2367; 2650	-0.5397754	-0.0227405		9.0
	ZINC35024383	-8.0	366.804	-4.1249	7	2	244.41	0.41394	140.86	7.1659	1085.195598354	0.34094	10.089	-12.099	0.54167	0.42833	0.94718 (max o	5	4390; 4280; 2650; 34	-0.5046439	-0.0372553		9.0
	ZINC05733294	-8.0	354.310	-0.9076	9	4	244.43	0.43137	134.91	-5.8493	1085.195598354	0.3273	6.8721	-2.773	0.48	0.28946	0.9402 (max of	6	6211; 4983; 4579; 24	-0.4594032	0.5360863		8.0
	ZINC04260662	-8.0	402.426	-0.6003	9	1	294.08	0.32304	119.08	4.4651	1085.195598354	0.28216	6.5648	-2.1275	0.51724	0.55706	0.92152 (max o	4	4553; 1166; 1418; 32	0.1214168	-0.5691550		8.0
	ZINC04260716	-8.0	372.400	-0.5818	8	1	273.91	0.31032	109.85	4.5839	1085.195598354	0.30306	6.5463	-1.9198	0.55556	0.49575	0.87932 (max o	4	6434; 4552; 3231; 32	0.14090319	-0.6095053		8.0
	ZINC19331503	-8.0	427.519	-2.0628	7	6	311.03	0.27856	108.83	2.5847	1085.195598354	0.26395	8.0273	-7.815	0.41935	0.48602	0.94751 (max o	3	1590; 3484; 3827	0.85726374	0.08092681		8.0
	ZINC72332946	-8.0	427.519	-2.0628	7	6	311.03	0.27856	108.83	2.5847	1085.195598354	0.26395	8.0273	-7.815	0.41935	0.48919	0.97423 (max o	5	5902; 5119; 1345; 15	0.8696747	0.11488778		8.0
	ZINC19331495	-8.0	427.519	-2.0628	7	6	311.03	0.27856	108.83	2.5847	1085.195598354	0.26395	8.0273	-7.815	0.41935	0.47959	0.90581 (max o	7	5902; 5119; 3981; 13	0.8744632	0.09201557		8.0
	ZINC04102138	-8.0	422.381	-3.6175	12	7	272.77	0.53063	195.6	-3.0562	1085.195598354	0.28216	9.582	-12.821	0.44828	0.36066		0		-0.9518896	0.13585615		8.0
	ZINC31159830	-8.0	424.444	0.4226	9	5	308.56	0.35176	145.91	-7.5878	1085.195598354	0.27275	5.5419	1.5494	0.53333	0.40293	0.96092 (max o	4	2306; 2307; 3215; 35	-0.3174540	0.5590532		8.0
	ZINC04268364	-8.0	390.383	-1.9707	10	5	264.25	0.44859	155.14	-3.9213	1085.195598354	0.30306	7.9352	-6.5027	0.48148	0.37688	0.95201 (max o	3	2690; 2813; 3235	-0.5191574	-0.5236625		8.0
	ZINC34166788	-8.0	390.383	-1.942	10	5	263.42	0.45	155.14	-4.365	1085.195598354	0.30306	7.9065	-6.408	0.48148	0.37688	0.98371 (max o	3	2626; 2627; 3708	-0.6289618	-0.0010447		8.0
	ZINC03840164	-8.0	442.806	-3.3075	10	5	306.43	0.42695	181.88	3.8704	1085.195598354	0.27275	9.272	-12.126	0.53333	0.52252	0.88922 (max o	2	1710; 2756	-0.1947712	-0.5214517		8.0
	ZINC03838750	-8.0	351.333	-1.3749	8	5	253.23	0.40876	141.75	3.7508	1085.195598354	0.3273	7.3394	-4.2007	0.56	0.50727	0.96196 (max o	4	4033; 2756; 2757; 31	-0.2289719	-0.5389758		8.0
	ZINC03838766	-8.0	377.396	-1.1864	9	5	280.14	0.40519	150.98	1.6237	1085.195598354	0.30306	7.1509	-3.9148	0.59259	0.50223	0.91109	1	4032	-0.2808088	-0.5287214		8.0
	ZINC04270612	-8.0	362.361	-2.5268	9	2	276.57	0.37209	127.87	-8.9358	1085.195598354	0.31471	8.4913	-8.0289	0.65385	0.58485	0.91731 (max o	3	5414; 4560; 4104	0.03742613	-0.4394542		8.0
	ZINC13424699	-8.0	370.309	-1.2533	10	5	250.78	0.47269	155.14	-5.8493	1085.195598354	0.31471	7.2178	-3.9823	0.42308	0.41647	0.94951 (max o	7	6430; 5408; 4983; 49	-0.3956389	0.49967286		8.0
	ZINC31156650	-8.0	384.336	-0.9776	10	4	266.69	0.43286	144.14	-5.8493	1085.195598354	0.30306	6.9421	-3.2258	0.40741	0.42813	0.98371 (max o	6	6630; 6035; 5471; 49	-0.3867892	0.53588843		8.0
	ZINC31155162	-8.0	376.357	-2.3979	10	7	261.49	0.46541	169.3	-7.5627	1085.195598354	0.31471	8.3624	-7.6193	0.53846	0.57293	0.9615 (max of	5	6301; 5982; 4793; 47	0.14649934	0.7039708		8.0
	ZINC31158144	-8.0	352.425	1.0294	6	4	262.95	0.2869	107.22	4.8399	1085.195598354	0.3273	4.9351	3.1451	0.56	0.44603	0.97118 (max o	6	5999; 5230; 4830; 47	-0.7047359	-0.1890401		8.0
	ZINC31158154	-8.0	352.425	1.0294	6	4	262.95	0.2869	107.22	4.8399	1085.195598354	0.3273	4.9351	3.1451	0.56	0.45151	0.97118 (max o	5	6880; 5999; 4799; 47	-0.6909583	-0.2039866		8.0
	ZINC05438529	-8.0	387.371	-2.6125	10	2	284.22	0.41985	138.64	1.3694	1085.195598354	0.29223	8.577	-8.9397	0.60714	0.38218	0.97941 (max o	8	5425; 4995; 4576; 41	-0.6611818	-0.5799705		7.0
	ZINC31164859	-8.0	440.484	-1.7456	11	7	311.11	0.42332	178.53	0.71929	1085.195598354	0.27275	7.7101	-6.3999	0.53333	0.53967	0.9897 (max of	6	5695; 5287; 1290; 26	-0.6493019	0.5121706		7.0
	ZINC12660035	-8.0	385.209	1.2442	6	4	234.65	0.30854	99.38	-6.4693	1085.195598354	0.35576	4.7203	3.4973	0.56522	0.36597	0.96125 (max o	7	4508; 4066; 1677; 27	0.278897	0.91721064		7.0
	ZINC02149682	-8.0	385.209	1.2442	6	4	234.65	0.30854	99.38	-6.4693	1085.195598354	0.35576	4.7203	3.4973	0.56522	0.36676	0.93686 (max o	7	4508; 4066; 3779; 16	0.27458474	0.9006031		7.0
	ZINC04024381	-8.0	428.544	-2.1249	8	6	324.6	0.32696	131.89	-4.0306	1085.195598354	0.27275	8.0894	-7.7906	0.53333	0.54938	0.94515	1	4397	0.8069335	-0.1051309		7.0
	ZINC00601247	-8.0	399.426	1.616	9	3	292.15	0.30207	136.55	2.7817	1085.195598354	0.28216	4.3485	5.7273	0.68966	0.5691		0		0.7655753	-0.6416465		6.0
	ZINC12892141	-8.0	426.404	-1.4845	10	2	315.81	0.35753	137.1	5.1043	1085.195598354	0.26395	7.449	-5.6241	0.51613	0.48498	0.98065 (max o	2	952; 2164	0.7363002	-0.2972436		6.0
	ZINC35456935	-8.0	406.470	-1.1587	9	7	290.09	0.38505	160.07	-3.1565	1085.195598354	0.29223	7.1232	-3.965	0.5	0.47465	0.96519 (max o	3	4833; 4411; 3495	0.8751395	0.21817018		6.0
	ZINC13333979	-8.0	388.455	-0.0883	8	5	281.14	0.3505	136.68	-3.7924	1085.195598354	0.30306	6.0528	-0.29136	0.51852	0.45195	0.96617 (max o	9	5497; 5225; 5103; 46	-0.3400595	0.1944655		6.0
	ZINC20463861	-8.0	442.534	-0.4493	8	3	320.88	0.22139	91.24	4.6027	1085.195598354	0.25571	6.4138	-1.7571	0.59375	0.45634	0.93414 (max o	4	6559; 5853; 4646; 42	0.39732143	-0.1003556		6.0
	ZINC20463863	-8.0	419.442	-2.5395	7	3	298.27	0.24132	72.21	-5.4897	1085.195598354	0.28216	8.504	-9.0003	0.58621	0.52884	0.93069 (max o	7	7064; 6846; 6503; 62	0.4721514	-0.2224341		6.0
	ZINC31167838	-8.0	434.524	0.0313	9	6	308.42	0.36197	156.91	-5.985	1085.195598354	0.27275	5.9332	0.11476	0.46667	0.49711	0.97304 (max o	7	6618; 6336; 4813; 30	-0.1689586	0.82481945		6.0
	ZINC31167830	-8.0	434.524	0.0313	9	6	308.42	0.36197	156.91	-5.985	1085.195598354	0.27275	5.9332	0.11476	0.46667	0.49711	0.97304 (max o	7	6618; 6336; 4813; 39	-0.1339216	0.8117802		6.0
	ZINC04222690	-8.0	423.242	-1.2049	8	1	270.54	0.31419	109.85	-1.0242	1085.195598354	0.31471	7.1694	-3.8286	0.57692	0.45984	0.93686 (max o	4	719; 2432; 2801; 322	-0.2097372	0.34867525		5.0
	ZINC31156581	-8.0	448.379	-0.4237	11	8	295.54	0.4664	197.37	-1.9977	1085.195598354	0.25571	6.3882	-1.657	0.4375	0.38295	0.94464 (max o	6	925; 1163; 1431; 207	0.78426045	0.03080616		5.0
	ZINC12489963	-8.0	399.469	-0.7712	8	4	312.18	0.3127	97.15	0.57407	1085.195598354	0.28216	6.7357	-2.7332	0.58621	0.49019	0.94529 (max o	5	6996; 1489; 2147; 25	0.31342292	0.04798716		5.0
	ZINC22922202	-8.0	393.462	-2.4075	9	4	300.24	0.33373	96.73	2.5535	1085.195598354	0.29223	8.372	-8.2382	0.57143	0.46989	0.95709 (max o	7	5543; 5181; 4214; 15	-0.6964051	-0.2245091		5.0
	ZINC22922187	-8.0	381.426	-2.2367	8	4	284.33	0.31724	87.5	1.1104	1085.195598354	0.30306	8.2012	-7.3804	0.59259	0.45723	0.93686 (max o	7	6565; 5181; 4645; 38	-0.6818539	-0.240268		5.0
	ZINC77257606	-8.0	381.312	-2.7633	10	6	254	0.49575	179.97	-4.1365	1085.195598354	0.30306	8.7278	-9.1181	0.40741	0.40591	0.85775 (max o	2	7072; 4366	-0.8486970	-0.4387841		5.0
	ZINC01646902	-8.0	419.417	-1.766	12	3	298.49	0.38655	161.33	-1.1402	1085.195598354	0.27275	7.7305	-6.4747	0.56667	0.53658	0.96092 (max o	3	2347; 2715; 3130	0.5331026	-0.6644739		5.0
	ZINC13209484	-8.0	358.421	-0.9827	8	2	266.97	0.26917	95.28	4.0255	1085.195598354	0.31471	6.9472	-3.1225	0.57692	0.52483		0		-0.5693615	-0.7756457		5.0

Structure	Molecule Name	affinity	Total Molweight	cLogP	H-Acceptors	H-Donors	Total Surface Area	Relative PSA	Polar Surface Area	Druglikeness	Kd (nM) @ 20°C	LE from K...	LLE from Kd ...	LELP from Kd (n...	Shape Index	Molecular...	Neighbor Si...	Neig...	Neighbor	Neighbor ...	Neighbor ...	Ring Systems	frequency
	ZINC04260486	-8.0	404.417	-0.7266	8	0	292.86	0.26323	101.06	1.6982	1085.195598354	0.28216	6.6911	-2.5752	0.48276	0.51295	0.98037 (max c	4	6142; 4551; 2439; 24	-0.3613039	0.80373013		5.0
	ZINC03842133	-8.0	419.480	-0.0889	10	2	324.62	0.31776	120.94	6.2103	1085.195598354	0.27275	6.0534	-0.32594	0.5	0.57749	0.94488 (max c	2	4930; 3220	-0.0343779	0.36063665		5.0
	ZINC08254179	-8.0	385.514	-4.3345	8	3	327.73	0.30348	81.58	0.27097	1085.195598354	0.29223	10.299	-14.832	0.64286	0.51667	0.97037 (max c	10	4484; 4149; 4147; 37	-0.123616	-0.0225366		5.0
	ZINC08254215	-8.0	371.487	-4.4594	8	3	298.87	0.29983	93.75	0.35716	1085.195598354	0.30306	10.424	-14.715	0.66667	0.50523	0.96885 (max c	12	5009; 4588; 4153; 41	-0.1189663	0.01174988		5.0
	ZINC08298038	-8.0	350.441	-2.94	8	2	277.08	0.3107	84.92	-1.3803	1085.195598354	0.3273	8.9045	-8.9825	0.64	0.53456	0.98188 (max c	8	5440; 5439; 4596; 41	0.23290277	0.27725825		5.0
	ZINC08298307	-8.0	370.499	-3.043	7	3	315.21	0.28073	68.69	0.15824	1085.195598354	0.30306	9.0075	-10.041	0.62963	0.49308	0.96885 (max c	5	5442; 5009; 5006; 27	-0.141419	-0.0374974		5.0
	ZINC03861793	-8.0	408.631	0.6952	7	5	239.64	0.3605	115.17	-5.48	1085.195598354	0.35576	5.2693	1.9541	0.47826	0.23842	0.91037 (max c	4	6081; 5790; 5407; 52	-0.4831529	0.6323968		5.0
	ZINC05398634	-8.0	449.501	0.2956	9	2	331.75	0.26665	112.63	0.10405	1085.195598354	0.25571	5.6689	1.156	0.4375	0.53129	0.90109 (max c	2	7156; 4992	0.00501716	0.3629252		5.0
	ZINC08254276	-8.0	357.436	-3.1884	8	3	285.22	0.33297	97.37	0.415	1085.195598354	0.31471	9.1529	-10.131	0.65385	0.49437	0.96721 (max c	6	4588; 4149; 4148; 32	-0.0678625	0.01802176		5.0
	ZINC08254147	-8.0	371.463	-2.7583	8	3	298.98	0.31765	97.37	-1.3433	1085.195598354	0.30306	8.7228	-9.1016	0.66667	0.51343	0.96885 (max c	6	4588; 4149; 4148; 37	-0.0684897	0.04015214		5.0
	ZINC20464839	-8.0	390.431	-0.9314	8	3	268.21	0.33478	116.71	0.94286	1085.195598354	0.31471	6.8959	-2.9595	0.53846	0.53208	0.97533 (max c	6	5551; 4740; 4739; 43	-0.4084001	-0.1043087		5.0
	ZINC20465149	-8.0	419.928	-0.3168	8	3	289.84	0.28754	110.72	8.604	1085.195598354	0.30306	6.2813	-1.0453	0.55556	0.52967	0.97658 (max c	5	5563; 5144; 4740; 43	-0.4414015	-0.0809327		5.0
	ZINC20465149	-8.0	420.936	-2.4265	8	4	303.88	0.32046	111.92	4.8242	1085.195598354	0.30306	8.391	-8.0067	0.55556	0.52967	0.97658 (max c	6	6259; 5564; 5145; 47	-0.4188237	-0.1368684		5.0
	ZINC05396198	-8.0	369.400	-1.2538	8	2	268.56	0.29613	99.47	5.8914	1085.195598354	0.30306	7.2183	-4.1372	0.55556	0.34866	0.90776 (max c	3	442; 958; 1136	-0.1506400	0.3793023		4.0
	ZINC20465890	-8.0	401.485	-1.1589	8	5	323.29	0.31155	108.15	1.7606	1085.195598354	0.28216	7.1234	-4.1073	0.58621	0.55807	0.94251 (max c	6	6831; 5159; 806; 260	0.3106566	-0.2620728		4.0
	ZINC13521027	-8.0	436.412	0.055	10	7	305.5	0.40831	177.14	-4.8731	1085.195598354	0.26395	5.9095	0.20837	0.51613	0.43481	0.95521 (max c	10	5085; 887; 2018; 205	0.7982878	-0.3190853		4.0
	ZINC03874668	-8.0	436.412	0.055	10	7	305.5	0.40831	177.14	-4.8731	1085.195598354	0.26395	5.9095	0.20837	0.51613	0.43522	0.95267 (max c	10	5085; 3799; 887; 201	0.82477766	-0.2848896		4.0
	ZINC35464842	-8.0	398.494	0.9599	7	4	288.68	0.29597	116.45	-3.8942	1085.195598354	0.29223	5.0046	3.2847	0.46429	0.4288	0.96519 (max c	5	6619; 5208; 1610; 19	-0.7028912	0.01183650		4.0
	ZINC05397696	-8.0	431.515	-4.4148	10	3	324.65	0.30867	129.7	4.2382	1085.195598354	0.26395	10.379	-16.726	0.51613	0.55037	0.91993 (max c	2	1745; 1947	0.14615378	0.27236605		4.0
	ZINC05037523	-8.0	356.326	-1.7633	9	4	245.13	0.43014	134.91	-5.757	1085.195598354	0.3273	7.7278	-5.3874	0.48	0.41551	0.94678 (max c	5	2044; 2829; 3265; 35	0.45746928	0.32777172		4.0
	ZINC05037524	-8.0	356.326	-1.7633	9	4	245.13	0.43014	134.91	-5.757	1085.195598354	0.3273	7.7278	-5.3874	0.48	0.41551	0.94678 (max c	5	3702; 2044; 2829; 32	0.4725119	0.29147503		4.0
	ZINC04293610	-8.0	447.399	-5.6024	12	2	330.6	0.43336	185.07	3.3292	1085.195598354	0.25571	11.567	-21.91	0.59375	0.57826	0.97948 (max c	3	4976; 2069; 3242	-0.8367879	-0.0709976		4.0
	ZINC05412402	-8.0	429.475	-3.6578	10	2	336.39	0.35144	122.84	-2.163	1085.195598354	0.26395	9.6223	-13.858	0.64516	0.48951	0.93405 (max c	5	4123; 929; 1167; 208	-0.2141646	-0.1077488		4.0
	ZINC12484097	-8.0	379.411	-0.4072	9	3	273.64	0.33844	101.52	2.0768	1085.195598354	0.30306	6.3717	-1.3436	0.59259	0.47039	0.95493 (max c	7	6215; 4645; 4214; 21	-0.653779	-0.2915337		4.0
	ZINC04095571	-8.0	386.352	-0.8489	10	5	276.54	0.42865	155.14	-5.8493	1085.195598354	0.30306	6.8134	-2.8011	0.51852	0.4143	0.91007 (max c	2	4677; 2953	-0.8540739	-0.1055193		4.0
	ZINC31156738	-8.0	418.397	0.8232	9	6	298.13	0.37447	156.91	-4.0044	1085.195598354	0.27275	5.1413	3.0181	0.5	0.42543	0.95837 (max c	7	2257; 2258; 2289; 22	-0.8041452	0.17683628		4.0
	ZINC31169930	-8.0	448.507	0.3212	10	6	332.1	0.35712	158.3	-3.599	1085.195598354	0.26395	5.6433	1.2169	0.6129	0.55437	0.90581 (max c	2	2318; 2643	-0.3558572	0.39686513		4.0
	ZINC72324501	-8.0	358.325	-1.2306	8	1	253.15	0.38673	121.83	0.11443	1085.195598354	0.31471	7.1951	-3.9102	0.57692	0.39474	0.93033 (max c	3	4451; 2329; 3117	-0.8605634	0.2177983		4.0
	ZINC04023825	-8.0	386.534	0.4278	5	3	304.67	0.15945	71.91	-1.7559	1085.195598354	0.29223	5.5367	1.4639	0.67857	0.45323	0.99414 (max c	3	2399; 3157; 3623	0.74587667	-0.4697764		4.0
	ZINC04023822	-8.0	386.534	0.4278	5	3	304.67	0.15945	71.91	-1.7559	1085.195598354	0.29223	5.5367	1.4639	0.67857	0.45547	0.99414 (max c	3	2399; 3157; 3624	0.7788096	-0.4654656		4.0
	ZINC31160316	-8.0	404.414	1.1159	8	5	296.29	0.32232	128.84	-4.1548	1085.195598354	0.28216	4.8486	3.9549	0.58621	0.44204	0.981 (max of 7	7	1132; 1389; 1406; 16	-0.5529673	0.75264955		4.0
	ZINC31160312	-8.0	406.386	0.4945	9	7	286.73	0.38957	160.07	-4.3466	1085.195598354	0.28216	5.47	1.7526	0.55172	0.34968	0.95872 (max c	5	1132; 1699; 2024; 26	-0.5608237	0.73425734		4.0
	ZINC31159448	-8.0	390.387	0.8402	8	6	280.38	0.35167	139.84	-4.3466	1085.195598354	0.29223	5.1243	2.8751	0.57143	0.43069	0.95401 (max c	8	3902; 1389; 1406; 16	-0.5617704	0.7808416		4.0
	ZINC35457930	-8.0	400.810	1.2997	8	5	282.47	0.35961	144.52	0.48888	1085.195598354	0.30306	4.6648	4.2886	0.40741	0.33782	0.97149 (max c	3	2660; 3071; 3503	-0.8710270	0.41228792		4.0
	ZINC38571037	-8.0	403.438	-1.4183	10	4	286	0.34909	140.87	4.5684	1085.195598354	0.28216	7.3828	-5.0266	0.48276	0.4833	0.9892 (max of	3	2670; 3081; 3522	-0.7325905	0.17291237		4.0
	ZINC03838758	-8.0	354.405	-1.6348	9	6	266.38	0.4316	153.78	0.73096	1085.195598354	0.3273	7.5993	-4.9948	0.6	0.55844	0.96196 (max c	4	4097; 2100; 2747; 27	-0.2261355	-0.4748792		4.0
	ZINC03839798	-8.0	434.492	-2.422	9	2	317.82	0.37883	153.17	6.8539	1085.195598354	0.27275	8.3865	-8.8799	0.66667	0.55146	0.96832 (max c	3	4045; 1674; 2754	-0.3529567	-0.267049		4.0
	ZINC04237154	-8.0	403.502	-0.8904	6	0	284.55	0.34563	134.29	6.7445	1085.195598354	0.30306	6.8549	-2.9381	0.59259	0.50845	0.96343 (max c	3	6143; 4100; 2804	-0.8585346	-0.4959900		4.0
	ZINC04082212	-8.0	388.368	-1.827	10	4	263.15	0.45024	151.98	-6.054	1085.195598354	0.30306	7.7915	-6.0285	0.48148	0.35896	0.94302 (max c	3	4398; 2933; 3061	-0.7694101	0.24792226		4.0
	ZINC20760352	-8.0	400.434	-1.0932	9	3	282.97	0.36718	130.83	4.7611	1085.195598354	0.28216	7.0577	-3.8744	0.41379	0.26844	0.95372 (max c	3	4335; 4333; 2607	-0.8064567	0.5131467		4.0
	ZINC62227199	-8.0	352.318	-3.2288	9	5	244.63	0.46482	155.3	-0.044643	1085.195598354	0.3273	9.1933	-9.8649	0.52	0.40876	0.94351 (max c	4	3956; 2680; 3096; 30	0.5872506	-0.4241604		4.0
	ZINC62227204	-8.0	352.318	-3.2288	9	5	244.63	0.46482	155.3	-0.044643	1085.195598354	0.3273	9.1933	-9.8649	0.52	0.40876	0.94351 (max c	4	2680; 3096; 3534; 39	0.5897308	-0.4045218		4.0
	ZINC72320494	-8.0	368.337	-0.2243	9	4	265.68	0.40831	142.75	-1.8996	1085.195598354	0.31471	6.1888	-0.71271	0.57692	0.39767	0.94008 (max c	3	4448; 4449; 3116	-0.4269066	0.8468849		4.0

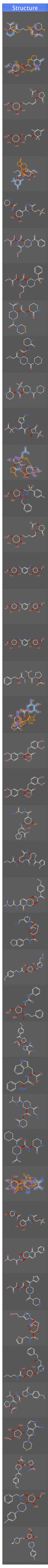

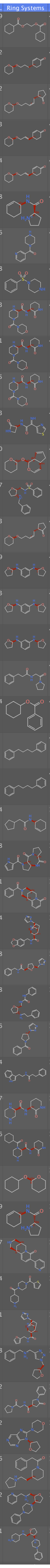

Structure	Molecule Name	affinity	Total Molweight	cLogP	H-Acceptors	H-Donors	Total Surface Area	Relative PSA	Polar Surface Area	Druglikeness	Kd (nM) @ 20°C	LE from K...	LLE from Kd ...	LELP from Kd (n...	Shape Index	Molecular...	Neighbor Si...	Neig...	Neighbor	Neighbor ...	Neighbor ...	Ring Systems	frequency
	ZINC03843346	-8.0	368.381	-3.3848	8	0	280.16	0.35951	132.86	-11.135	1085.195598354	0.31471	9.3493	-10.755	0.53846	0.62243	0.96438 (max o	3	6661; 4046; 3169	-0.7602342	-0.4264289		4.0
	ZINC31155668	-8.0	372.456	0.8828	7	4	276.35	0.30917	116.45	-5.701	1085.195598354	0.31471	5.0817	2.8051	0.53846	0.51537	0.97533 (max o	7	5280; 5281; 4794; 26	0.2729053	-0.5005972		4.0
	ZINC31156084	-8.0	387.403	-2.5561	9	4	285.25	0.3953	156.58	-5.0739	1085.195598354	0.30306	8.5206	-8.4344	0.59259	0.63006	0.94905 (max o	7	5965; 5629; 5228; 52	-0.884333	0.06586412		4.0
	ZINC31155443	-8.0	370.440	0.6306	7	4	275.33	0.31032	116.45	-4.553	1085.195598354	0.31471	5.3339	2.0037	0.53846	0.47687	0.97269 (max o	7	5189; 4794; 3904; 26	0.22359867	-0.4713363		4.0
	ZINC31161212	-8.0	386.439	-0.2961	8	5	283.18	0.34798	136.68	-5.5643	1085.195598354	0.30306	6.2606	-0.97704	0.51852	0.47785	0.97149 (max o	3	5189; 3461; 3884	0.21468301	-0.4917234		4.0
	ZINC04200822	-8.0	433.504	-3.9404	11	6	302.24	0.46265	200.88	1.4613	1085.195598354	0.28216	9.9049	-13.965	0.51724	0.47959	0.91292 (max o	12	6431; 6133; 5764; 49	-0.5849754	-0.1349098		4.0
	ZINC05397699	-8.0	392.411	-1.0804	10	1	298.4	0.34078	118.14	1.3805	1085.195598354	0.29223	7.0449	-3.697	0.5	0.57348	0.93373 (max o	6	6973; 6454; 6452; 61	0.04507013	0.40470046		4.0
	ZINC05397706	-8.0	427.476	-0.711	10	1	310.47	0.34226	130.7	-4.9187	1085.195598354	0.28216	6.6755	-2.5199	0.48276	0.56032	0.93686 (max o	3	6163; 5800; 5418	0.1229104	0.42650908		4.0
	ZINC05437522	-8.0	381.431	-1.2952	10	2	282.68	0.33865	111.29	4.5368	1085.195598354	0.30306	7.2597	-4.2738	0.51852	0.5673	0.93686 (max o	6	5010; 3819; 3820; 14	-0.1123668	0.23734523		4.0
	ZINC15709537	-8.0	411.457	-1.794	11	2	306.44	0.34503	120.52	4.3528	1085.195598354	0.28216	7.7585	-6.3581	0.48276	0.57215	0.9426 (max of	4	5010; 1446; 2471; 36	-0.1340052	0.24262081		4.0
	ZINC15709558	-8.0	417.485	-1.7333	11	2	293.51	0.3791	136.74	5.5536	1085.195598354	0.29223	7.6978	-5.9312	0.5	0.56955	0.88831	1	3699	-0.1502322	0.23049775		4.0
	ZINC12503091	-8.0	433.421	-5.9852	13	2	286.79	0.62548	243.86	5.3236	1085.195598354	0.29223	11.95	-20.481	0.46429	0.44003	0.97533 (max o	3	6248; 5320; 3821	-0.5386910	-0.6573933		4.0
	ZINC13374266	-8.0	388.368	-2.1091	10	5	265.99	0.44566	155.14	-3.1573	1085.195598354	0.30306	8.0736	-6.9594	0.48148	0.39433	0.94302 (max o	3	6787; 5238; 4377	-0.9120134	0.10382800		4.0
	ZINC30879717	-8.0	421.536	-0.6055	7	4	322.4	0.29104	108.68	-4.0514	1085.195598354	0.28216	6.57	-2.146	0.55172	0.54225	0.97878 (max o	7	5592; 4788; 4758; 43	0.48218367	-0.3190767		4.0
	ZINC31156079	-8.0	387.403	-2.5561	9	4	285.25	0.3953	156.58	-5.0739	1085.195598354	0.30306	8.5206	-8.4344	0.59259	0.63406	0.95201 (max o	7	5965; 5629; 5228; 52	-0.8996458	0.07890803		4.0
	ZINC36367438	-8.0	372.373	-2.5562	10	8	254.8	0.47692	163.9	-1.9752	1085.195598354	0.31471	8.5207	-8.1223	0.57692	0.44167	0.9464 (max of	3	4269; 3921; 3937	-0.8506974	-0.3172379		4.0
	ZINC36367437	-8.0	372.373	-2.5562	10	8	254.8	0.47692	163.9	-1.9752	1085.195598354	0.31471	8.5207	-8.1223	0.57692	0.44167	0.9464 (max of	3	4269; 3921; 3938	-0.8757673	-0.2884333		4.0
	ZINC35361010	-8.0	372.373	-2.5562	10	8	254.8	0.47692	163.9	-1.9752	1085.195598354	0.31471	8.5207	-8.1223	0.57692	0.44167	0.94326 (max o	3	4269; 3937; 3938	-0.8703669	-0.310432		4.0
	ZINC35464606	-8.0	375.443	-1.0588	7	1	289.31	0.2544	100.98	7.6353	1085.195598354	0.30306	7.0233	-3.4937	0.51852	0.64008	0.96617 (max o	3	6885; 5640; 5240	-0.1656869	0.8766649		4.0
	ZINC35465643	-8.0	358.298	-1.0155	10	6	241.05	0.51724	173.98	-0.59921	1085.195598354	0.3273	6.98	-3.1026	0.52	0.41264	0.98188 (max o	3	5643; 4839; 4838	-0.0522238	0.78511554		4.0
	ZINC72320083	-8.0	391.395	-0.7635	8	4	292.41	0.34103	139.51	-1.3035	1085.195598354	0.29223	6.728	-2.6126	0.5	0.56831	0.98006 (max o	3	5278; 4444; 3969	0.70077384	0.5966221		4.0
	ZINC72320081	-8.0	391.395	-0.7635	8	4	292.41	0.34103	139.51	-1.3035	1085.195598354	0.29223	6.728	-2.6126	0.5	0.56831	0.98006 (max o	3	5278; 4444; 3970	0.69875467	0.5755		4.0
	ZINC03840035	-8.0	396.418	-4.0294	10	5	291.87	0.44825	181.88	2.7551	1085.195598354	0.29223	9.9939	-13.788	0.53571	0.59191	0.9673 (max of	3	1378; 2008; 2009	0.5915707	0.71863204		3.0
	ZINC15708819	-8.0	403.438	-1.8579	10	2	289.8	0.34179	112.98	8.2472	1085.195598354	0.28216	7.8224	-6.5846	0.51724	0.33009	0.93985 (max o	8	4038; 430; 555; 702;	-0.5863892	-0.0518688		3.0
	ZINC04277707	-8.0	425.507	-0.4945	8	2	332.41	0.25652	83.39	5.7953	1085.195598354	0.26395	6.459	-1.8734	0.6129	0.45333	0.9396 (max of	7	4324; 1435; 2067; 24	0.2773611	-0.6086561		3.0
	ZINC08298786	-8.0	400.481	-5.6447	9	3	308.1	0.34203	110.35	-1	1085.195598354	0.28216	11.609	-20.005	0.62069	0.46827	0.97176 (max o	5	760; 1187; 1188; 210	-0.2032045	-0.0144284		3.0
	ZINC12603451	-8.0	406.461	-3.1969	10	4	305.45	0.35112	135.34	-2.0559	1085.195598354	0.28216	9.1614	-11.33	0.68966	0.55477	0.9398 (max of	2	4604; 2108	0.79706067	-0.4294208		3.0
	ZINC20465799	-8.0	400.497	-0.212	7	5	324.53	0.27655	95.26	1.7606	1085.195598354	0.28216	6.1765	-0.75135	0.58621	0.55891	0.9427 (max of	5	6268; 5159; 3855; 22	0.32723004	-0.2487058		3.0
	ZINC04221571	-8.0	416.393	-0.9959	12	2	301.62	0.44778	172.73	-0.94321	1085.195598354	0.27275	6.9604	-3.6513	0.5	0.43448	0.9103 (max of	3	6726; 4536; 4535	-0.7153124	-0.5096406		3.0
	ZINC14651456	-8.0	351.405	-0.5014	6	3	271.93	0.29526	112.66	2.9212	1085.195598354	0.31471	6.4659	-1.5932	0.57692	0.47478	0.93033 (max o	5	6693; 3999; 2349; 28	0.2725978	0.76093554		3.0
	ZINC05433818	-8.0	369.444	-5.2604	10	4	278.55	0.38815	138.49	4.8665	1085.195598354	0.31471	11.225	-16.715	0.5	0.56144	0.89582 (max o	4	939; 1457; 2323; 283	-0.8197925	-0.1065487		3.0
	ZINC05414438	-8.0	378.471	0.0844	8	2	288.64	0.28471	98.82	1.2177	1085.195598354	0.30306	5.8801	0.2785	0.48148	0.50808	0.94589 (max o	7	6455; 4574; 143; 600	-0.0701979	0.23247856		3.0
	ZINC04216682	-8.0	380.372	-6.2025	12	8	237.3	0.5799	225	-2.0341	1085.195598354	0.31471	12.167	-19.708	0.53846	0.49507	0.86721 (max o	2	6016; 5647	0.6085463	0.7820716		3.0
	ZINC04221598	-8.0	449.503	-4.7925	12	7	307.33	0.49761	221.11	2.5113	1085.195598354	0.27275	10.757	-17.571	0.53333	0.47455	0.91292 (max o	10	6431; 6133; 5764; 49	-0.6278770	-0.1641679		3.0
	ZINC05397187	-8.0	366.396	-1.4953	7	0	269.88	0.26482	93.64	4.1077	1085.195598354	0.30306	7.4598	-4.934	0.59259	0.45753	0.94883 (max o	3	4543; 4120; 2436	-0.5322066	-0.3876767		3.0
	ZINC05398609	-8.0	437.539	0.0614	9	2	321.34	0.33864	140.87	3.1465	1085.195598354	0.27275	5.9031	0.22511	0.46667	0.51339	0.91157 (max o	3	7697; 6660; 5784	-0.1699738	0.30473554		3.0
	ZINC08298553	-8.0	360.436	-2.5687	8	3	289.91	0.33842	97.62	-1.4007	1085.195598354	0.31471	8.5332	-8.162	0.65385	0.51399	0.96721 (max o	5	6473; 5443; 4592; 45	0.0733856	-0.1919991		3.0
	ZINC08643351	-8.0	434.515	-3.0945	10	4	349.57	0.33498	123.17	-3.2218	1085.195598354	0.26395	9.059	-11.724	0.67742	0.60366	0.92602 (max o	5	5450; 4605; 1762; 17	0.2653594	-0.7033308		3.0
	ZINC08790218	-8.0	430.479	-1.3933	9	1	322.43	0.29464	119.08	7.6495	1085.195598354	0.26395	7.3578	-5.2786	0.54839	0.45492	0.96971 (max o	7	5492; 5032; 4664; 46	0.4948127	0.31832162		3.0
	ZINC08918576	-8.0	352.350	-1.7011	11	4	243.01	0.48517	152.01	0.0425	1085.195598354	0.3273	7.6656	-5.1973	0.44	0.37618	0.91999 (max o	6	6990; 6989; 6758; 64	-0.8513220	0.11382742		3.0
	ZINC20463001	-8.0	431.531	-1.5387	8	2	347.56	0.28717	117.2	4.7116	1085.195598354	0.26395	7.5032	-5.8294	0.54839	0.58833	0.93405 (max o	4	6992; 5913; 5524; 42	0.01110943	-0.1910736		3.0
	ZINC20465403	-8.0	417.503	-1.2674	7	3	308.49	0.20101	85.67	2.5628	1085.195598354	0.27275	7.2319	-4.6467	0.6	0.43945	0.94529 (max o	8	4312; 4299; 2228; 22	-0.4797643	0.24637842		3.0
	ZINC20465406	-8.0	424.559	-4.2322	7	5	344.61	0.25069	73.84	1.5772	1085.195598354	0.27275	10.197	-15.517	0.6	0.50777	0.94529 (max o	6	7730; 5558; 5154; 47	-0.3001161	-0.5657981		3.0

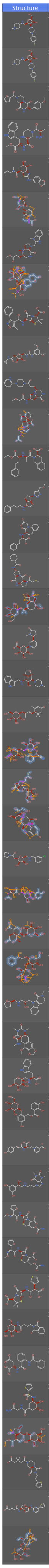

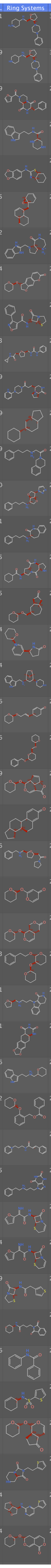

Structure	Molecule Name	affinity	Total Molweight	cLogP	H-Acceptors	H-Donors	Total Surface Area	Relative PSA	Polar Surface Area	Druglikeness	Kd (nM) @ 20°C	LE from K...	LLE from Kd ...	LELP from Kd (n...	Shape Index	Molecular...	Neighbor Si...	Neig...	Neighbor	Neighbor ...	Neighbor ...	Ring Systems	frequency
	ZINC20465406	-8.0	423.551	-2.1225	7	4	330.57	0.21886	72.64	3.5378	1085.195598354	0.27275	8.087	-7.7818	0.6	0.5037	0.94529 (max o	4	5560; 5152; 4743; 43	0.63976765	-0.4365891		3.0
	ZINC20465406	-8.0	423.551	-1.2375	7	3	312.81	0.17451	76.02	6.0901	1085.195598354	0.27275	7.202	-4.5371	0.6	0.4486	0.94529 (max o	5	5565; 5559; 4742; 43	-0.4709234	0.11753809		3.0
	ZINC04260198	-8.0	383.403	0.7604	8	1	283.32	0.28868	92.09	7.3488	1085.195598354	0.29223	5.2041	2.602	0.60714	0.47843	0.96297 (max o	5	446; 578; 1421; 2063;	-0.2719559	0.04730909		2.0
	ZINC35447025	-8.0	402.473	-3.9888	9	4	305.19	0.34097	131.17	3.9859	1085.195598354	0.28216	9.9533	-14.137	0.51724	0.50246		0		-0.7466615	0.6272023		2.0
	ZINC08636387	-8.0	405.474	1.1942	8	3	286.01	0.361	128.92	4.0942	1085.195598354	0.29223	4.7703	4.0864	0.53571	0.41124	0.90299 (max o	6	6182; 5824; 4150; 76	-0.5110107	0.2939545		2.0
	ZINC35464960	-8.0	391.482	-0.7932	6	3	283.97	0.29676	113.35	-0.82856	1085.195598354	0.29223	6.7577	-2.7143	0.46429	0.37458	0.9678 (max of	4	411; 843; 2659; 3074	-0.0912282	0.40908605		2.0
	ZINC22920848	-8.0	401.509	-3.5531	8	3	306.29	0.24666	82.15	-2.2529	1085.195598354	0.28216	9.5176	-12.593	0.55172	0.42105	0.92547	1	1266	-0.3294314	-0.8841392		2.0
	ZINC31163752	-8.0	424.401	-0.702	10	5	284.95	0.416	155.14	-6.6063	1085.195598354	0.27275	6.6665	-2.5738	0.46667	0.37044	0.95053 (max o	4	1130; 1289; 1901; 19	-0.7389093	-0.3529444		2.0
	ZINC03831241	-8.0	400.434	0.0218	8	1	279.19	0.39751	140.87	10.153	1085.195598354	0.29223	5.9427	0.074597	0.5	0.36695	0.93686 (max o	5	686; 690; 1385; 1659;	0.4497354	0.6720823		2.0
	ZINC08643523	-8.0	409.437	-0.4659	10	5	300.9	0.3877	138.38	2.2926	1085.195598354	0.28216	6.4304	-1.6512	0.58621	0.56557	0.86078	1	1462	0.28930703	-0.4648003		2.0
	ZINC15708139	-8.0	446.550	-4.6731	10	4	339.02	0.28532	109.56	7.5627	1085.195598354	0.25571	10.638	-18.275	0.625	0.50484	0.93157 (max o	2	632; 1838	-0.2666043	0.13820049		2.0
	ZINC03850283	-8.0	419.536	-2.7517	6	0	319.04	0.2569	120.39	-3.811	1085.195598354	0.27275	8.7162	-10.089	0.46667	0.50937	0.94515 (max o	3	2013; 3170; 3171	-0.4630728	-0.7565189		2.0
	ZINC03801163	-8.0	438.522	-0.1462	7	1	339.16	0.22022	103.35	-0.32321	1085.195598354	0.25571	6.1107	-0.57175	0.5	0.47407	0.90581 (max o	2	6695; 2052	-0.7748328	-0.0067879		2.0
	ZINC05438181	-8.0	359.448	-0.6964	7	1	275.3	0.24174	75.01	-4.2088	1085.195598354	0.31471	6.6609	-2.2128	0.69231	0.42486	0.91731 (max o	3	751; 2078; 2834	-0.102141	-0.2622230		2.0
	ZINC03814184	-8.0	395.434	-2.8833	7	1	299.67	0.26352	117.18	-7.3863	1085.195598354	0.28216	8.8478	-10.219	0.48276	0.47963	0.95908	1	2094	0.5250729	-0.7998361		2.0
	ZINC06131108	-8.0	422.548	-0.2189	8	2	303.87	0.328	124.12	1.8442	1085.195598354	0.28216	6.1834	-0.77581	0.55172	0.37791	0.981 (max of 7	7	4922; 4488; 4487; 40	-0.5939791	0.02441635		2.0
	ZINC72320120	-8.0	448.507	-1.5165	10	7	305.77	0.40795	177.14	-8.1275	1085.195598354	0.26395	7.481	-5.7453	0.41935	0.52422	0.9884 (max of	3	6913; 3973; 2325	-0.4847396	-0.7875165		2.0
	ZINC03869573	-8.0	352.342	-7.7802	9	3	240.72	0.39868	134.24	2.8108	1085.195598354	0.3273	13.745	-23.771	0.48	0.44503	0.93348 (max o	4	4647; 4052; 4051; 25	-0.7189643	-0.0015674		2.0
	ZINC22922151	-8.0	373.475	-1.4894	7	3	268.94	0.23027	77.57	7.0876	1085.195598354	0.30306	7.4539	-4.9146	0.55556	0.4145	0.93686 (max o	3	6561; 2587; 3867	-0.0806090	-0.9768564		2.0
	ZINC31155433	-8.0	370.440	0.6306	7	4	275.33	0.31032	116.45	-4.553	1085.195598354	0.31471	5.3339	2.0037	0.53846	0.48626	0.97269 (max o	5	5189; 3885; 3884; 26	0.24534844	-0.4683186		2.0
	ZINC31157937	-8.0	427.405	-2.4776	11	6	298.59	0.44261	182.09	-10.383	1085.195598354	0.27275	8.4421	-9.0837	0.53333	0.48614	0.96589 (max o	4	842; 2632; 2633; 303	-0.5177826	0.74731106		2.0
	ZINC04098349	-8.0	446.428	-1.9048	11	4	289.59	0.50406	186.51	-2.8226	1085.195598354	0.27275	7.8693	-6.9836	0.46667	0.39985	0.95076 (max o	3	5600; 2798; 3237	0.8897887	-0.0339009		2.0
	ZINC03882036	-8.0	394.437	0.167	6	4	262.92	0.29849	115.06	2.4849	1085.195598354	0.29223	5.7975	0.57146	0.46429	0.31582	0.9543 (max of	4	1681; 2022; 2774; 28	0.5126703	0.5922932		2.0
	ZINC08624292	-8.0	369.867	-0.4931	6	3	266.49	0.22714	86.61	2.0382	1085.195598354	0.3273	6.4576	-1.5066	0.6	0.44889	0.91964 (max o	6	7015; 6748; 6223; 58	0.09273521	-0.4459836		2.0
	ZINC31156127	-8.0	415.370	-2.8558	11	3	295.72	0.44874	171.88	2.9919	1085.195598354	0.28216	8.8203	-10.121	0.55172	0.5642	0.99848	1	3033	-0.2086146	0.9602573		2.0
	ZINC67910232	-8.0	389.332	-4.9553	11	4	258.42	0.51374	175.04	-4.8669	1085.195598354	0.30306	10.92	-16.351	0.44444	0.42077	0.94605 (max o	2	1064; 3101	0.6089843	0.5632881		2.0
	ZINC31160820	-8.0	416.421	-1.5798	10	6	291.9	0.4063	158.3	-6.1605	1085.195598354	0.28216	7.5443	-5.599	0.51724	0.46992	0.96983 (max o	4	7421; 4382; 3102; 35	-0.4346298	0.43037573		2.0
	ZINC00803231	-8.0	358.460	-0.4344	6	2	269.54	0.20145	71.31	5.3478	1085.195598354	0.31471	6.3989	-1.3803	0.5	0.38664	0.98284 (max o	3	4887; 4886; 3125	0.13528042	-0.8232361		2.0
	ZINC02126223	-8.0	375.380	0.5316	9	1	261.3	0.33555	97.41	3.1899	1085.195598354	0.30306	5.4329	1.7541	0.37037	0.33473	0.96617 (max o	3	4018; 2731; 3149	0.92946327	0.01070011		2.0
	ZINC15218659	-8.0	366.369	-3.9926	9	6	254.04	0.43871	162.68	-2.6869	1085.195598354	0.31471	9.9571	-12.686	0.53846	0.5131	0.94951	1	3367	0.20126182	0.93784386		2.0
	ZINC35457489	-8.0	438.384	-0.3814	11	7	301.74	0.44654	186.37	-4.1057	1085.195598354	0.26395	6.3459	-1.4449	0.48387	0.40368	0.95521 (max o	3	3499; 3500; 3501	0.685969	-0.5239432		2.0
	ZINC77257588	-8.0	369.352	-7.6274	8	2	282.96	0.33574	133.47	2.0393	1085.195598354	0.30306	13.592	-25.168	0.62963	0.47052	0.96617	1	3551	-0.0528918	0.76347		2.0
	ZINC02019839	-8.0	390.419	-1.579	10	4	282.43	0.3472	135.74	3.7713	1085.195598354	0.29223	7.5435	-5.4032	0.57143	0.51188	0.9678 (max of	2	4890; 3559	-0.1709473	-0.9017765		2.0
	ZINC03830484	-8.0	423.381	-2.8488	12	2	292.92	0.54564	201.89	13.42	1085.195598354	0.28216	8.8133	-10.096	0.51724	0.38037	0.981 (max of 5	5	6395; 4496; 4030; 40	-0.2993987	0.8291605		2.0
	ZINC03871977	-8.0	423.381	-2.8488	12	2	292.92	0.54564	201.89	13.42	1085.195598354	0.28216	8.8133	-10.096	0.51724	0.41769	0.981 (max of 6	6	6395; 5878; 4496; 40	-0.2797726	0.83777654		2.0
	ZINC03831539	-8.0	382.416	-3.6789	8	1	252.44	0.52916	183.21	11.228	1085.195598354	0.3273	9.6434	-11.24	0.48	0.33609	0.90896 (max o	4	6978; 6491; 5323; 44	-0.4065148	-0.4327579		2.0
	ZINC04027984	-8.0	408.406	-0.851	10	4	290.12	0.39098	145.63	-0.16973	1085.195598354	0.28216	6.8155	-3.016	0.55172	0.49608	0.95675 (max o	2	4065; 4064	-0.0580190	0.9588728		2.0
	ZINC04040672	-8.0	354.317	-3.2387	8	2	268.26	0.38634	138.46	3.4589	1085.195598354	0.31471	9.2032	-10.291	0.53846	0.53917	0.96721	1	4510	0.7825123	-0.1509446		2.0
	ZINC04221776	-8.0	375.425	-1.8	9	5	253.65	0.54962	195.44	7.2775	1085.195598354	0.34094	7.7645	-5.2795	0.54167	0.53033	0.91567	1	4096	-0.8451521	0.4884682		2.0
	ZINC05015106	-8.0	388.368	-1.827	10	4	263.15	0.45024	151.98	-6.054	1085.195598354	0.30306	7.7915	-6.0285	0.48148	0.36873	0.93686 (max o	2	4115; 3249	-0.1872178	0.4007412		2.0
	ZINC05415823	-8.0	406.482	-0.2263	8	0	295.38	0.32991	129.3	6.2086	1085.195598354	0.29223	6.1908	-0.77438	0.57143	0.58667	0.91599 (max o	3	6975; 1441; 2840	0.51720935	0.4788651		2.0
	ZINC05433873	-8.0	417.465	-1.3603	9	2	304.23	0.41278	153.74	-1.2664	1085.195598354	0.28216	7.3248	-4.8211	0.62069	0.35002	0.96682 (max o	6	5424; 4995; 4576; 41	-0.668596	-0.5431884		2.0
	ZINC05601207	-8.0	356.326	-1.7633	9	4	245.13	0.43014	134.91	-5.757	1085.195598354	0.3273	7.7278	-5.3874	0.48	0.39769	0.95631 (max o	5	4135; 3988; 3265; 35	0.48869735	0.25452304		2.0

Structure	Molecule Name	affinity	Total Molweight	cLogP	H-Acceptors	H-Donors	Total Surface Area	Relative PSA	Polar Surface Area	Druglikeness	Kd (nM) @ 20°C	LE from K...	LLE from Kd ...	LELP from Kd (n...	Shape Index	Molecular...	Neighbor Si...	Neig...	Neighbor	Neighbor ...	Neighbor ...	Ring Systems	frequency
	ZINC05784598	-8.0	359.309	-0.6258	8	4	264.21	0.38893	147.35	-1.3192	1085.195598354	0.31471	6.5903	-1.9885	0.61538	0.44901	0.9615 (max of	6	6068; 6067; 5083; 50	0.01652533	-0.8705041		2.0
	ZINC05923201	-8.0	390.387	0.8402	8	6	280.38	0.35167	139.84	-4.3466	1085.195598354	0.29223	5.1243	2.8751	0.57143	0.46334	0.9678 (max of	7	4139; 4140; 1386; 20	-0.4839111	0.81619054		2.0
	ZINC08643349	-8.0	390.438	-0.563	9	3	296.45	0.33567	118.73	-1.106	1085.195598354	0.29223	6.5275	-1.9265	0.64286	0.53651	0.90983 (max o	2	5831; 4605	0.25434676	-0.7420897		2.0
	ZINC08643484	-8.0	357.449	-0.1299	8	4	272.26	0.32598	111.13	4.4055	1085.195598354	0.3273	6.0944	-0.39688	0.64	0.60167	0.86951 (max o	2	5449; 2925	0.21809264	-0.4340439		2.0
	ZINC08740537	-8.0	386.371	0.8103	12	5	275.91	0.50121	176.82	1.3234	1085.195598354	0.29223	5.1542	2.7728	0.5	0.33348	0.98451 (max o	12	6484; 5030; 4627; 46	-0.2672015	-0.6370439		2.0
	ZINC08880016	-8.0	371.392	-0.7527	8	3	263.53	0.33655	107.97	4.2775	1085.195598354	0.30306	6.7172	-2.4837	0.59259	0.37988	0.92739	1	4199	0.88246423	0.04498057		2.0
	ZINC12296697	-8.0	358.464	-0.871	7	4	297.83	0.28248	81.67	5.0813	1085.195598354	0.31471	6.8355	-2.7676	0.65385	0.5922	0.96389	1	5044	-0.8965319	-0.4271284		2.0
	ZINC12481053	-8.0	415.532	-0.4313	7	1	340.6	0.22927	85.19	4.534	1085.195598354	0.27275	6.3958	-1.5813	0.53333	0.59278	0.94251 (max o	3	4206; 1205; 2982	-0.0254157	-0.2425434		2.0
	ZINC12481447	-8.0	416.520	-0.7794	8	2	338.3	0.26471	97.22	4.7001	1085.195598354	0.27275	6.7439	-2.8575	0.56667	0.58931	0.95837 (max o	6	6499; 4644; 1000; 17	-0.0583621	-0.2295587		2.0
	ZINC12603509	-8.0	357.405	-1.2713	9	3	260.86	0.34823	111.57	2.6575	1085.195598354	0.3273	7.2358	-3.8842	0.6	0.57091	0.90243 (max o	4	4603; 1495; 3293; 37	0.19611667	-0.4370429		2.0
	ZINC12654793	-8.0	413.421	-0.3992	10	5	301.66	0.3976	154.78	-17.14	1085.195598354	0.28216	6.3637	-1.4148	0.58621	0.54808	0.95908 (max o	3	6224; 5222; 4822	-0.7269369	0.57017106		2.0
	ZINC15060918	-8.0	363.428	-0.939	6	3	246.64	0.32298	117.89	-4.3143	1085.195598354	0.31471	6.9035	-2.9837	0.38462	0.41417	0.92368 (max o	4	2268; 2269; 2270; 38	0.9130967	0.2623698		2.0
	ZINC15060918	-8.0	362.420	-3.015	6	2	247.82	0.3262	120.72	-1.8945	1085.195598354	0.31471	8.9795	-9.5801	0.38462	0.41153	0.92368 (max o	2	2268; 3807	0.9383309	0.2688746		2.0
	ZINC15675202	-8.0	428.935	-1.8818	8	4	315.47	0.2599	107.87	2.3469	1085.195598354	0.28216	7.8463	-6.6693	0.65517	0.53465	0.93395 (max o	4	7015; 6223; 4228; 42	0.08254923	-0.4645311		2.0
	ZINC20410987	-8.0	411.561	-0.9837	7	2	302.27	0.22308	84.84	0.89296	1085.195598354	0.28216	6.9482	-3.4864	0.51724	0.49255	0.95908 (max o	3	4284; 4283; 3381	-0.2630818	0.8899066		2.0
	ZINC20463861	-8.0	443.542	-2.559	8	4	334.92	0.25403	92.44	2.2729	1085.195598354	0.25571	8.5235	-10.008	0.59375	0.45972	0.93148 (max o	3	7212; 6558; 6556	-0.5595953	-0.6030342		2.0
	ZINC20760352	-8.0	401.442	-1.3543	9	3	279.25	0.35875	135.41	3.8291	1085.195598354	0.28216	7.3188	-4.7998	0.41379	0.28829	0.93101 (max o	3	4336; 4334; 2608	-0.6988174	0.6228181		2.0
	ZINC31155673	-8.0	372.456	0.8828	7	4	276.35	0.30917	116.45	-5.701	1085.195598354	0.31471	5.0817	2.8051	0.53846	0.50545	0.97533 (max o	6	5280; 5189; 4794; 34	0.26744348	-0.4671476		2.0
	ZINC31155896	-8.0	358.342	-0.7325	9	5	248.05	0.42532	138.07	-4.5704	1085.195598354	0.3273	6.697	-2.238	0.52	0.47272	0.98671 (max o	3	6301; 5982; 5595	0.13291326	0.7751411		2.0
	ZINC31156646	-8.0	403.359	-3.9731	11	4	283.89	0.46765	175.04	-6.0644	1085.195598354	0.29223	9.9376	-13.596	0.46429	0.47814	0.91561	1	4372	0.8741218	0.02401280		2.0
	ZINC31157977	-8.0	364.393	1.4512	7	2	267.37	0.31911	110.13	-16.562	1085.195598354	0.31471	4.5133	4.6112	0.42308	0.31382	0.97791 (max o	3	6611; 6292; 5601	-0.7642775	0.18778531		2.0
	ZINC31163784	-8.0	365.400	-1.6171	7	2	267.98	0.35156	119.42	-3.3681	1085.195598354	0.31471	7.5816	-5.1383	0.53846	0.59699	0.95857	1	3907	0.5350267	-0.6223331		2.0
	ZINC31163780	-8.0	365.400	-1.6171	7	2	267.98	0.35156	119.42	-3.3681	1085.195598354	0.31471	7.5816	-5.1383	0.53846	0.59391	0.95857	1	3908	0.5530074	-0.6308246		2.0
	ZINC35442551	-8.0	423.576	-1.8144	8	2	337.3	0.24379	75.55	2.6381	1085.195598354	0.27275	7.7789	-6.6522	0.56667	0.49963	0.90825 (max o	6	5223; 4825; 4401; 83	-0.1258527	0.5755024		2.0
	ZINC35455680	-8.0	396.478	0.2159	7	4	273.46	0.31244	116.45	-7.2753	1085.195598354	0.29223	5.7486	0.73879	0.39286	0.29698	0.93385 (max o	3	6328; 2631; 3455	0.73894125	0.31592658		2.0
	ZINC40309436	-8.0	362.385	-1.6987	8	2	273.46	0.43023	148.69	1.8006	1085.195598354	0.3273	7.6632	-5.19	0.64	0.54609	0.91007	1	4847	-0.1938254	-0.9642578		2.0
	ZINC49181875	-8.0	446.411	0.0678	11	3	310.24	0.38822	147.35	6.7757	1085.195598354	0.25571	5.8967	0.26515	0.5	0.50056	0.95465 (max o	3	4428; 679; 849	-0.1836779	-0.9751144		2.0
	ZINC72320122	-8.0	448.507	-1.5165	10	7	305.77	0.40795	177.14	-8.1275	1085.195598354	0.26395	7.481	-5.7453	0.41935	0.52422	0.98656 (max o	3	6913; 2325; 3972	-0.5175413	-0.7983455		2.0
	ZINC72332861	-8.0	394.530	-1.6827	6	4	272.72	0.28194	83.59	-0.33179	1085.195598354	0.29223	7.6472	-5.758	0.35714	0.32121	0.79631	1	7451	0.53869057	0.73764724		2.0
	ZINC77257228	-8.0	373.445	-0.9757	7	2	271.05	0.39509	149.26	-4.2177	1085.195598354	0.3273	6.9402	-2.981	0.52	0.49896	0.97118 (max o	3	2330; 2331; 3983	0.49172702	0.6683818		2.0
	ZINC77257225	-8.0	373.445	-0.9757	7	2	271.05	0.39509	149.26	-4.2177	1085.195598354	0.3273	6.9402	-2.981	0.52	0.49941	0.97118 (max o	3	3984; 2330; 2331	0.47024482	0.6351146		2.0
	ZINC01609239	-8.0	356.326	-1.7633	9	4	245.13	0.43014	134.91	-5.757	1085.195598354	0.3273	7.7278	-5.3874	0.48	0.40593	0.9594 (max of	4	3702; 3676; 2044; 28	0.453628	0.28390938		1.0
	ZINC02019839	-8.0	391.427	-3.5568	10	5	271.46	0.32082	136.99	3.3663	1085.195598354	0.29223	9.5213	-12.171	0.57143	0.51188	0.85878	1	3560	-0.1584822	-0.8859914		1.0
	ZINC03830508	-8.0	395.435	-1.4848	8	1	271.44	0.47635	169.38	11.089	1085.195598354	0.31471	7.4493	-4.7179	0.57692	0.4758	0.94008 (max o	4	7459; 5467; 4911; 49	0.40727174	0.5189311		1.0
	ZINC03839322	-8.0	364.401	-1.8929	9	2	255.1	0.36135	108.05	7.1765	1085.195598354	0.31471	7.8574	-6.0147	0.53846	0.31336	0.89688 (max o	3	132; 1669; 2750	-0.5452277	-0.0997667		1.0
	ZINC03839831	-8.0	384.479	-2.4177	9	3	262.37	0.3354	123.03	7.8647	1085.195598354	0.31471	8.3822	-7.6822	0.57692	0.47229	0.88151 (max o	2	4490; 881	0.7588876	-0.5031486		1.0
	ZINC03840075	-8.0	430.484	-0.8088	9	5	313.84	0.42965	182.88	6.4223	1085.195598354	0.27275	6.7733	-2.9653	0.53333	0.49225	0.96832 (max o	6	6189; 4540; 1380; 36	-0.5104204	-0.1140096		1.0
	ZINC03840080	-8.0	423.512	-1.7628	9	6	303.34	0.43387	186.6	5.228	1085.195598354	0.28216	7.7273	-6.2476	0.51724	0.46567	0.90299 (max o	7	6189; 5332; 4540; 45	-0.5406334	-0.1371545		1.0
	ZINC03840137	-8.0	363.369	-1.3372	9	5	266.03	0.42781	140.9	2.4113	1085.195598354	0.31471	7.3017	-4.2489	0.53846	0.44448	0.87218	1	3166	-0.5139175	-0.2221621		1.0
	ZINC03842164	-8.0	444.511	0.7269	9	1	331.84	0.33766	137.15	6.7146	1085.195598354	0.26395	5.2376	2.7539	0.45161	0.52511	0.91292 (max o	5	6972; 5339; 4966; 49	-0.0134359	0.41170675		1.0
	ZINC03871967	-8.0	408.414	-4.8643	10	2	276.73	0.57753	222.12	8.4031	1085.195598354	0.30306	10.829	-16.051	0.48148	0.45978	0.94302 (max o	4	5322; 5292; 4092; 27	-0.3558362	0.33028698		1.0
	ZINC03982482	-8.0	364.436	0.5857	6	4	231.86	0.32537	107.22	-5.1582	1085.195598354	0.31471	5.3788	1.8611	0.34615	0.35342	0.88178 (max o	2	4869; 2531	0.86301035	0.32576716		1.0
	ZINC04027359	-8.0	360.321	0.9481	9	2	255.86	0.38001	114.4	3.2797	1085.195598354	0.31471	5.0164	3.0126	0.5	0.39618	0.92702	1	5706	-0.5454914	0.47693187		1.0

Structure	Molecule Name	affinity	Total Molweight	cLogP	H-Acceptors	H-Donors	Total Surface Area	Relative PSA	Polar Surface Area	Druglikeness	Kd (nM) @ 20°C	LE from K...	LLE from Kd ...	LELP from Kd (n...	Shape Index	Molecular...	Neighbor Si...	Neig...	Neighbor	Neighbor ...	Neighbor ...	Ring Systems	frequency
	ZINC04085635	-8.0	354.465	-0.9097	6	1	262.82	0.26037	73.9	-11.215	1085.195598354	0.3273	6.8742	-2.7794	0.52	0.43218	0.98434 (max o	3	6951; 6715; 4088	0.09465051	0.536571		1.0
	ZINC04099102	-8.0	447.555	-2.4274	7	1	325.6	0.35878	170.72	-4.7035	1085.195598354	0.27275	8.3919	-8.8997	0.5	0.50237		0		-0.5676769	0.8121469		1.0
	ZINC04260949	-8.0	385.376	-4.3413	11	1	270.01	0.5228	170	-2.5761	1085.195598354	0.31471	10.306	-13.794	0.65385	0.4758	0.85614	1	3254	0.9321039	-0.1068852		1.0
	ZINC04268250	-8.0	406.473	1.148	7	3	269.11	0.31727	113.29	-5.0575	1085.195598354	0.28216	4.8165	4.0686	0.31034	0.35279		0		-0.9347383	0.13869907		1.0
	ZINC05370519	-8.0	382.379	0.303	10	2	276.96	0.36525	131.9	1.93	1085.195598354	0.29223	5.6615	1.0368	0.5	0.35147		0		-0.0769179	-0.7941607		1.0
	ZINC05397153	-8.0	363.397	-1.3482	9	1	254.23	0.41907	142.21	6.9508	1085.195598354	0.3273	7.3127	-4.1191	0.6	0.56525	0.83096	1	5421	0.20870759	-0.8637529		1.0
	ZINC05398388	-8.0	352.453	0.3101	7	1	265.71	0.24071	83.53	0.040415	1085.195598354	0.3273	5.6544	0.94744	0.52	0.46426	0.85658 (max o	2	6729; 5397	-0.1920898	0.24473211		1.0
	ZINC05414444	-8.0	449.550	0.0489	10	2	338.22	0.28304	111.29	0.20341	1085.195598354	0.25571	5.9156	0.19124	0.4375	0.53649	0.8872 (max of	4	4974; 4574; 2086; 36	-0.119719	0.20161198		1.0
	ZINC05414751	-8.0	423.512	-0.4738	10	3	323.86	0.32001	120.08	1.5359	1085.195598354	0.27275	6.4383	-1.7371	0.46667	0.55601	0.9167 (max of	2	4574; 2471	-0.1054130	0.2179167		1.0
	ZINC05415606	-8.0	442.499	-0.0687	10	2	321.56	0.38711	152.84	6.634	1085.195598354	0.26395	6.0332	-0.26027	0.41935	0.48833	0.96502 (max o	5	4569; 4127; 482; 174	0.02041803	0.22348392		1.0
	ZINC05433809	-8.0	448.566	-0.1738	9	3	326.06	0.33822	143.67	6.4546	1085.195598354	0.26395	6.1383	-0.65845	0.41935	0.48347	0.96502 (max o	2	2841; 2862	-0.1009756	0.10820614		1.0
	ZINC06623755	-8.0	423.464	-0.8299	10	4	308.69	0.33671	131.8	1.681	1085.195598354	0.27275	6.7944	-3.0427	0.53333	0.49779	0.86078	1	5794	0.42006356	0.56146824		1.0
	ZINC08624376	-8.0	382.411	-0.7774	9	3	283.23	0.35801	121.47	3.5023	1085.195598354	0.30306	6.7419	-2.5652	0.59259	0.51687	0.88538 (max o	2	5820; 1772	0.23974863	-0.4308056		1.0
	ZINC08643591	-8.0	373.404	-2.0933	10	3	270.86	0.3723	120.8	2.6846	1085.195598354	0.31471	8.0578	-6.6514	0.61538	0.59783	0.92394 (max o	4	5479; 4603; 3775; 14	0.16139105	-0.4366810		1.0
	ZINC08740325	-8.0	366.401	-3.7014	11	5	269.61	0.46545	147.22	0.60016	1085.195598354	0.31471	9.6659	-11.761	0.46154	0.37603	0.88432 (max o	2	6410; 3752	-0.8654126	0.12932472		1.0
	ZINC08740333	-8.0	445.463	-2.1077	11	8	309.21	0.43065	181.33	-6.437	1085.195598354	0.26395	8.0722	-7.9851	0.51613	0.44038	0.95267 (max o	3	6370; 5457; 5026	-0.1761044	-0.933379		1.0
	ZINC08765461	-8.0	442.443	-1.3835	9	1	325.29	0.31301	115.38	0.94	1085.195598354	0.25571	7.348	-5.4105	0.46875	0.38684	0.9665 (max of	3	5868; 5493; 4628	-0.9480569	-0.2220998		1.0
	ZINC09033373	-8.0	357.318	-3.5513	11	8	247.65	0.549	180.61	-1.1977	1085.195598354	0.3273	9.5158	-10.85	0.56	0.52326		0		-0.6877139	0.6417047		1.0
	ZINC12489979	-8.0	405.517	-0.9242	8	4	320.22	0.30485	97.15	-3.2988	1085.195598354	0.28216	6.8887	-3.2755	0.58621	0.51385	0.89424	1	7910	0.33980417	-0.0131213		1.0
	ZINC12503149	-8.0	416.358	-0.4274	7	1	271.42	0.26862	104.21	1.0835	1085.195598354	0.27275	6.3919	-1.567	0.46667	0.31752	0.91333 (max o	2	3423; 3424	-0.7463739	-0.6210469		1.0
	ZINC12529669	-8.0	405.449	-0.5429	9	3	284.96	0.33836	115.51	6.3962	1085.195598354	0.28216	6.5074	-1.9241	0.55172	0.46811	0.90299 (max o	5	7217; 7042; 6220; 55	-0.7698893	0.3817398		1.0
	ZINC12529669	-8.0	407.465	-3.8773	9	4	295.28	0.36149	121.29	2.3474	1085.195598354	0.28216	9.8418	-13.742	0.55172	0.44088	0.90617 (max o	6	7043; 6221; 5542; 55	0.5843607	-0.1180126		1.0
	ZINC12529669	-8.0	406.457	-2.6526	9	4	299	0.36943	116.71	3.44	1085.195598354	0.28216	8.6171	-9.4011	0.55172	0.47107	0.87919 (max o	2	7173; 6222	0.5602726	-0.2320678		1.0
	ZINC12529784	-8.0	377.424	-3.2619	9	2	294.11	0.45534	150.78	3.873	1085.195598354	0.31471	9.2264	-10.365	0.65385	0.44591	0.90719	1	2150	0.60158044	-0.3302756		1.0
	ZINC12662939	-8.0	419.461	-0.0327	10	5	292.5	0.46465	180.52	1.8241	1085.195598354	0.28216	5.9972	-0.11589	0.48276	0.44088	0.93686 (max o	5	4202; 1196; 1787; 21	0.29749385	-0.5345545		1.0
	ZINC13409379	-8.0	360.332	-2.5234	9	1	263.94	0.38482	128.96	3.4345	1085.195598354	0.30306	8.4879	-8.3265	0.51852	0.19183	0.93058	1	971	-0.3738342	-0.8098562		1.0
	ZINC14455258	-8.0	399.402	-0.9163	9	2	290.54	0.37819	149.31	-1.7264	1085.195598354	0.28216	6.8808	-3.2475	0.48276	0.40672		0		-0.8824213	-0.4209647		1.0
	ZINC14770076	-8.0	354.421	-1.5748	7	3	265.15	0.33894	97.5	3.7131	1085.195598354	0.3273	7.5393	-4.8114	0.36	0.22509	0.88322	1	2678	-0.2866163	0.9476646		1.0
	ZINC15672728	-8.0	358.397	-2.0597	8	2	276.52	0.34576	124.76	4.0129	1085.195598354	0.31471	8.0242	-6.5447	0.61538	0.58047	0.81601	1	5111	-0.1252676	0.8304742		1.0
	ZINC15676144	-8.0	390.482	-1.3033	8	3	289.93	0.28421	119.47	-0.37117	1085.195598354	0.29223	7.2678	-4.4598	0.5	0.57474	0.88589 (max o	3	5887; 3812; 3813	0.8137313	-0.3724279		1.0
	ZINC15676144	-8.0	389.474	-1.1144	8	2	300.9	0.3103	118.22	0.15266	1085.195598354	0.29223	7.0789	-3.8134	0.5	0.57474	0.97288 (max o	3	5888; 3811; 3813	0.831781	-0.3813749		1.0
	ZINC15676144	-8.0	389.474	-1.1144	8	2	300.9	0.3103	118.22	0.15266	1085.195598354	0.29223	7.0789	-3.8134	0.5	0.57474	0.97288 (max o	2	3811; 3812	0.8141226	-0.3945430		1.0
	ZINC15676274	-8.0	382.462	-2.2543	7	3	283.25	0.24487	102.4	0.88417	1085.195598354	0.29223	8.2188	-7.714	0.53571	0.47487	0.87809 (max o	3	5893; 4399; 1522	-0.2956609	0.4972626		1.0
	ZINC15708580	-8.0	420.556	-1.7318	8	3	323.41	0.33113	114.43	10.438	1085.195598354	0.28216	7.6963	-6.1377	0.58621	0.46426	0.9279 (max of	5	5899; 4267; 4221; 15	-0.1811358	0.10398020		1.0
	ZINC15708783	-8.0	365.392	-0.4919	8	2	268.79	0.32564	104.29	6.6737	1085.195598354	0.30306	6.4564	-1.6231	0.59259	0.33003	0.84311	1	4486	0.3700488	0.66295683		1.0
	ZINC16958002	-8.0	433.421	-5.9852	13	2	286.79	0.62548	243.86	5.3236	1085.195598354	0.29223	11.95	-20.481	0.46429	0.44003	0.95148 (max o	2	6248; 3768	-0.522238	-0.6295446		1.0
	ZINC19323563	-8.0	361.464	-0.6851	7	3	267.89	0.3105	82.95	5.1418	1085.195598354	0.31471	6.6496	-2.1769	0.38462	0.50767	0.88896	1	3825	-0.8676849	-0.4621802		1.0
	ZINC19323563	-8.0	361.464	-0.6851	7	3	267.89	0.3105	82.95	5.1418	1085.195598354	0.31471	6.6496	-2.1769	0.38462	0.50767	0.88896	1	3824	-0.8799459	-0.4510168		1.0
	ZINC19795964	-8.0	388.467	-2.3099	7	1	262.95	0.34018	118.08	9.6591	1085.195598354	0.30306	8.2744	-7.622	0.48148	0.38375	0.93373 (max o	2	6305; 6285	0.4780679	0.84267765		1.0
	ZINC20112087	-8.0	379.519	-0.8686	6	1	281.4	0.22026	67.91	-1.6832	1085.195598354	0.30306	6.8331	-2.8661	0.55556	0.47182	0.94302 (max o	4	6715; 1533; 1857; 38	0.11495357	0.54532766		1.0
	ZINC20112091	-8.0	379.519	-0.8686	6	1	281.4	0.22026	67.91	-1.6832	1085.195598354	0.30306	6.8331	-2.8661	0.55556	0.47182	0.94302 (max o	3	1533; 1857; 3831	0.11648767	0.56623036		1.0
	ZINC20462832	-8.0	410.489	-4.3377	9	1	326.39	0.31364	117.48	3.9461	1085.195598354	0.28216	10.302	-15.373	0.55172	0.63792	0.9426 (max of	3	5125; 4714; 4710	-0.2771916	-0.2745432		1.0
	ZINC20462983	-8.0	430.591	-0.0784	7	2	362.79	0.23135	89.8	0.37029	1085.195598354	0.26395	6.0429	-0.29702	0.58065	0.61438	0.91635 (max o	2	5131; 3322	-0.3061370	-0.1854735		1.0

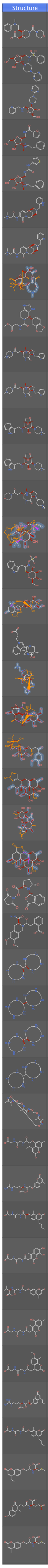

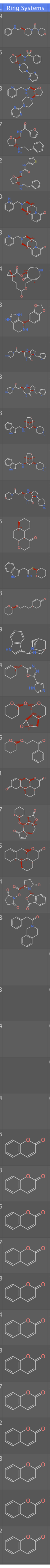

Structure	Molecule Name	affinity	Total Molweight	cLogP	H-Acceptors	H-Donors	Total Surface Area	Relative PSA	Polar Surface Area	Druglikeness	Kd (nM) @ 20°C	LE from K...	LLE from Kd ...	LELP from Kd (n...	Shape Index	Molecular...	Neighbor Si...	Neig...	Neighbor	Neighbor ...	Neighbor ...	Ring Systems	frequency
	ZINC20463486	-8.0	388.486	0.0988	7	0	305.05	0.2	76.15	5.4031	1085.195598354	0.29223	5.8657	0.33809	0.53571	0.62612		0		-0.7242123	-0.6862659		1.0
	ZINC20465070	-8.0	449.530	-0.6213	10	3	319.44	0.32958	136.5	-0.28776	1085.195598354	0.26395	6.5858	-2.3538	0.54839	0.47792	0.90299 (max o	2	6267; 5563	-0.4082690	-0.0274355		1.0
	ZINC20465084	-8.0	401.489	-2.5619	9	3	299.66	0.28015	111.45	3.9028	1085.195598354	0.28216	8.5264	-9.0797	0.58621	0.41591	0.95372 (max o	5	4312; 4299; 3848; 22	-0.5009927	0.25243163		1.0
	ZINC20465752	-8.0	375.443	-1.0172	7	4	302.76	0.30519	96.37	2.6141	1085.195598354	0.30306	6.9817	-3.3565	0.55556	0.5359	0.96343 (max o	3	5591; 4319; 4316	0.21028909	-0.1716987		1.0
	ZINC20465797	-8.0	406.525	-0.4276	7	5	320.47	0.34359	123.5	3.0597	1085.195598354	0.29223	6.3921	-1.4632	0.57143	0.55347	0.9399 (max of	3	6605; 6268; 4322	0.32485196	-0.2284122		1.0
	ZINC20503569	-8.0	383.494	-3.4316	7	3	293.95	0.2117	71.23	5.7617	1085.195598354	0.29223	9.3961	-11.743	0.60714	0.39094	0.88589	1	3857	0.2373766	-0.6371000		1.0
	ZINC20503569	-8.0	382.486	-1.4538	7	2	304.92	0.24006	69.98	5.7617	1085.195598354	0.29223	7.4183	-4.9748	0.60714	0.40674	0.91248 (max o	6	4324; 2067; 2447; 24	0.22405218	-0.6041443		1.0
	ZINC20610432	-8.0	381.423	0.089	8	1	281.42	0.33196	105.67	8.9299	1085.195598354	0.30306	5.8755	0.29367	0.40741	0.31692	0.9144 (max of	2	6286; 818	-0.7410471	0.45664564		1.0
	ZINC20760993	-8.0	359.361	-0.7481	9	3	250.44	0.33972	107.03	0.41682	1085.195598354	0.31471	6.7126	-2.3771	0.5	0.46647	0.94008	1	2247	-0.2832043	-0.9443383		1.0
	ZINC22921177	-8.0	416.568	-4.3831	8	3	347.38	0.26136	74.42	5.2194	1085.195598354	0.27275	10.348	-16.07	0.6	0.49319	0.90725 (max o	5	5585; 4779; 835; 386	-0.0320599	0.5744894		1.0
	ZINC22921177	-8.0	415.560	-2.2734	8	2	333.34	0.23025	73.22	9.4625	1085.195598354	0.27275	8.2379	-8.335	0.6	0.49319	0.9707 (max of	5	835; 2655; 2988; 302	0.00717358	0.56350213		1.0
	ZINC22922151	-8.0	374.483	-3.5991	7	4	282.98	0.26846	78.77	2.8907	1085.195598354	0.30306	9.5636	-11.876	0.55556	0.4145	0.91109 (max o	2	6561; 3869	-0.0638629	-0.9682183		1.0
	ZINC22922151	-8.0	373.475	-2.3744	7	4	286.7	0.27796	74.19	3.42	1085.195598354	0.30306	8.3389	-7.8348	0.55556	0.44136	0.90776	1	6563	0.42528966	0.6391847		1.0
	ZINC22923279	-8.0	409.573	-4.4626	8	3	339.14	0.26485	70.76	4.6521	1085.195598354	0.28216	10.427	-15.816	0.58621	0.50432	0.93686 (max o	9	6284; 5997; 4825; 18	-0.1631612	0.5752431		1.0
	ZINC25725270	-8.0	368.468	0.836	6	4	255.32	0.29547	107.22	-5.1021	1085.195598354	0.31471	5.1285	2.6564	0.38462	0.43107	0.93686	1	6601	0.10563409	0.96875155		1.0
	ZINC30160081	-8.0	447.464	-1.9575	11	5	295.92	0.53477	218.41	-3.1062	1085.195598354	0.28216	7.922	-6.9376	0.44828	0.50276	0.87809	1	5374	-0.3743791	0.5527063		1.0
	ZINC31161972	-8.0	388.455	0.0594	8	5	280.61	0.35116	136.68	-4.727	1085.195598354	0.30306	5.9051	0.196	0.51852	0.54983		0		0.9155357	0.34139383		1.0
	ZINC32124326	-8.0	368.455	-2.6711	6	1	261.69	0.20784	71.77	4.6135	1085.195598354	0.30306	8.6356	-8.8138	0.48148	0.45962		0		-0.0910837	-0.9881999		1.0
	ZINC35442744	-8.0	416.612	-0.5014	7	2	372.7	0.23899	65.62	1.6991	1085.195598354	0.27275	6.4659	-1.8383	0.56667	0.55507		0		-0.9151243	0.37965444		1.0
	ZINC44418123	-8.0	364.346	-3.3428	10	6	235.7	0.53564	161.6	-4.3182	1085.195598354	0.3273	9.3073	-10.213	0.44	0.37198	0.90171	1	5436	0.50186574	0.6122729		1.0
	ZINC49600040	-8.0	358.342	-1.1943	9	5	255.3	0.42515	145.91	-3.6721	1085.195598354	0.3273	7.1588	-3.6489	0.56	0.50799	0.88322 (max o	4	6301; 5982; 4793; 47	0.13834357	0.72326356		1.0
	ZINC49785214	-8.0	396.434	-1.0197	8	5	255.49	0.39759	144.52	-2.8974	1085.195598354	0.29223	6.9842	-3.4893	0.35714	0.39239	0.93985 (max o	3	4434; 3540; 3541	0.2410792	-0.8633441		1.0
	ZINC67912310	-8.0	426.460	-0.9089	9	4	289.11	0.37522	142.75	-1.4771	1085.195598354	0.27275	6.8734	-3.3323	0.36667	0.33069	0.93686	1	1940	0.8583744	-0.3220947		1.0
	ZINC67912695	-8.0	398.450	-0.9925	8	6	256.06	0.39694	147.68	-1.5022	1085.195598354	0.29223	6.957	-3.3962	0.35714	0.39548	0.92774	1	4435	0.8148123	0.51671755		1.0
	ZINC70686864	-8.0	358.305	-0.8493	9	0	242.15	0.38117	110.29	-16.951	1085.195598354	0.31471	6.8138	-2.6986	0.46154	0.39216	0.93361	1	2691	-0.3903319	0.84507614		1.0
	ZINC72324475	-8.0	397.406	-1.2034	8	1	297.42	0.31007	121.99	5.2937	1085.195598354	0.28216	7.1679	-4.265	0.48276	0.51467		0		0.12936278	-0.9846818		1.0
	ZINC68605103	-8.0	373.611	-7.8395	8	8	312.18	0.25793	109.98	1.345	1085.195598354	0.31471	13.804	-24.91	0.53846	0.49088	0.88896 (max o	3	3961; 3962; 3963	0.62836707	0.692842		0.0
	ZINC68605103	-8.0	374.619	-9.0642	8	8	308.46	0.24898	114.56	1.345	1085.195598354	0.31471	15.029	-28.801	0.53846	0.48684	0.89253 (max o	3	3961; 3962; 3964	0.627832	0.71241623		0.0
	ZINC68605103	-8.0	374.619	-9.0642	8	8	308.46	0.24898	114.56	0.65056	1085.195598354	0.31471	15.029	-28.801	0.53846	0.49217	0.89253 (max o	2	3963; 3964	0.6100141	0.68606204		0.0
	ZINC68605103	-8.0	374.619	-9.0642	8	8	308.46	0.24898	114.56	0.74063	1085.195598354	0.31471	15.029	-28.801	0.53846	0.48146	0.89253 (max o	2	3963; 3964	0.609752	0.7056631		0.0
	ZINC68576608	-8.0	384.392	3.8526	12	0	312.66	0.41278	129.54	0.235	1085.195598354	0.30306	2.1119	12.712	0.44444	0.24331		0		0.8651274	0.34812024		0.0
	ZINC02093285	-7.9	375.356	-2.4747	9	2	285.48	0.38307	133.86	-1.4895	1288.439712237	0.29927	8.3646	-8.2691	0.59259	0.48776	0.90299 (max o	4	1083; 1641; 1989; 31	0.17794432	-0.0100336		172.0
	ZINC02105747	-7.9	390.371	-2.6274	10	3	294.24	0.42357	159.88	-3.7367	1288.439712237	0.28858	8.5173	-9.1045	0.60714	0.5423	0.9678 (max of	5	1085; 1645; 1980; 35	0.26596397	0.12071743		172.0
	ZINC02099405	-7.9	417.436	-1.2066	9	2	324	0.33753	133.86	-5.8841	1288.439712237	0.26934	7.0965	-4.4798	0.63333	0.495	0.98037 (max o	7	4007; 1644; 1976; 19	0.30092552	-0.0109686		172.0
	ZINC02123788	-7.9	363.301	-3.4061	10	4	260.37	0.48224	165.09	1.1825	1288.439712237	0.31078	9.296	-10.96	0.61538	0.58026	0.96998 (max o	4	1975; 2738; 3154; 35	0.12192671	0.04791277		172.0
	ZINC02159276	-7.9	389.383	-2.241	9	2	295.22	0.37044	133.86	-2.132	1288.439712237	0.28858	8.1309	-7.7656	0.57143	0.57089	0.95401 (max o	8	1983; 2544; 2723; 29	0.13841115	-0.0774088		172.0
	ZINC20412612	-7.9	361.329	-2.7165	9	3	266.28	0.42234	144.86	1.1825	1288.439712237	0.31078	8.6064	-8.7409	0.61538	0.58026	0.94622 (max o	3	4466; 1082; 3411	0.10294608	0.01408584		172.0
	ZINC02099065	-7.9	361.329	-2.881	9	2	271.72	0.40247	133.86	-1.2823	1288.439712237	0.31078	8.7709	-9.2702	0.61538	0.5828	0.90719 (max o	3	3153; 3154; 3581	0.18790916	0.00863548		172.0
	ZINC12660764	-7.9	432.475	-1.1759	10	4	318.57	0.34667	168.4	0.56747	1288.439712237	0.26065	7.0658	-4.5113	0.6129	0.56211	0.97423	1	3311	0.09032860	0.81180507		172.0
	ZINC02107535	-7.9	403.410	-1.5659	9	2	313	0.34939	133.86	-6.9366	1288.439712237	0.27863	7.4558	-5.62	0.65517	0.4932	0.97941 (max o	7	4903; 1976; 2352; 31	0.28098857	-0.0106922		172.0
	ZINC02125338	-7.9	364.397	-1.3938	7	1	272.5	0.3756	130.06	1.4363	1288.439712237	0.32321	7.2837	-4.3123	0.6	0.53316	0.94027 (max o	6	2722; 2737; 3134; 35	0.21837386	0.15335758		172.0
	ZINC04073355	-7.9	394.423	-2.6399	8	2	286.05	0.38693	152.04	1.8042	1288.439712237	0.29927	8.5298	-8.8211	0.59259	0.55122	0.99202 (max o	4	3140; 3564; 3584; 36	-0.0513620	0.1920031		172.0
	ZINC02112484	-7.9	350.370	-1.8482	7	1	258.74	0.39557	130.06	-3.0392	1288.439712237	0.33668	7.7381	-5.4895	0.58333	0.52485	0.95983 (max o	7	5294; 4023; 4017; 16	0.21137981	0.17647192		172.0

Structure	Molecule Name	affinity	Total Molweight	cLogP	H-Acceptors	H-Donors	Total Surface Area	Relative PSA	Polar Surface Area	Druglikeness	Kd (nM) @ 20°C	LE from K...	LLE from Kd ...	LELP from Kd (n...	Shape Index	Molecular...	Neighbor Si...	Neig...	Neighbor	Neighbor ...	Neighbor ...	Ring Systems	frequency
	ZINC02159392	-7.9	364.397	-1.5043	7	1	271	0.37768	130.06	-3.0392	1288.439712237	0.32321	7.3942	-4.6542	0.56	0.42964	0.89723 (max o	3	4901; 1652; 4009	0.19308148	0.1838489		172.0
	ZINC02118087	-7.9	352.749	-0.9031	7	1	253.38	0.33491	104.76	2.9068	1288.439712237	0.33668	6.793	-2.6824	0.58333	0.48895	0.89637 (max o	2	5702; 1687	0.18125738	0.14705965		172.0
	ZINC02117467	-7.9	434.424	-2.2091	11	3	326.97	0.41175	169.11	2.792	1288.439712237	0.26065	8.099	-8.4752	0.58065	0.5567	0.92304 (max o	9	7120; 5709; 4474; 10	0.04450972	0.17002955		172.0
	ZINC01750410	-7.9	364.397	-1.3938	7	1	272.5	0.3756	130.06	-2.5187	1288.439712237	0.32321	7.2837	-4.3123	0.6	0.52942	0.96243 (max o	7	5308; 5294; 4017; 40	0.24091935	0.15619445		172.0
	ZINC02120117	-7.9	433.436	-1.8227	10	2	327.95	0.36396	143.09	4.8467	1288.439712237	0.26065	7.7126	-6.9928	0.54839	0.59308	0.95983 (max o	6	4899; 4900; 4465; 27	0.02444171	0.02670462		172.0
	ZINC02111341	-7.9	417.436	-1.2301	9	3	315.8	0.35611	144.86	5.6379	1288.439712237	0.26934	7.12	-4.567	0.56667	0.60548	0.9561 (max of	7	6076; 5698; 4759; 44	0.04535548	0.00151355		172.0
	ZINC02125144	-7.9	384.815	-1.0296	7	2	268.72	0.39242	141.06	3.9881	1288.439712237	0.32321	6.9195	-3.1855	0.56	0.54145	0.91928 (max o	4	7120; 6835; 6690; 53	-0.0121534	0.15290357		172.0
	ZINC13151994	-7.9	379.352	-3.5312	12	4	291.12	0.49801	194.56	1.2927	1288.439712237	0.29927	9.4211	-11.799	0.66667	0.62765	0.87344	1	4247	0.8482498	0.36367175		134.0
	ZINC13151994	-7.9	378.344	-4.7559	12	3	279.66	0.47744	182.53	0.8563	1288.439712237	0.29927	10.646	-15.892	0.66667	0.63425	0.87344	1	4248	0.8664695	0.36957687		134.0
	ZINC35458111	-7.9	401.457	-1.5171	7	2	324.65	0.28412	129.59	5.3725	1288.439712237	0.27863	7.407	-5.4448	0.55172	0.53691	0.97878 (max o	3	6883; 2661; 4413	0.8235123	0.20447496		134.0
	ZINC35458107	-7.9	401.457	-1.5171	7	2	324.65	0.28412	129.59	5.3725	1288.439712237	0.27863	7.407	-5.4448	0.55172	0.54578	0.97878 (max o	3	6883; 2661; 4414	0.84119475	0.19193047		134.0
	ZINC01875567	-7.9	399.471	-2.4614	10	5	282.13	0.41626	187.42	-4.3873	1288.439712237	0.29927	8.3513	-8.2247	0.62963	0.59946	0.96343 (max o	4	6515; 6195; 4894; 12	0.6013075	-0.3093232		134.0
	ZINC04899619	-7.9	379.415	-2.3144	10	5	282.8	0.39569	171.2	-10.386	1288.439712237	0.29927	8.2043	-7.7335	0.66667	0.57648	0.9339 (max of	10	7323; 6080; 5797; 57	-0.0705415	-0.4496117		134.0
	ZINC03874914	-7.9	441.440	-6.0726	12	3	324.93	0.51097	237.22	-4.4889	1288.439712237	0.26934	11.963	-22.546	0.56667	0.68882	0.97304 (max o	3	6104; 5737; 5347	-0.6371415	0.63119465		134.0
	ZINC04084854	-7.9	428.504	-2.01	10	4	332.27	0.37295	156.89	-57.266	1288.439712237	0.26934	7.8999	-7.4626	0.6	0.62477	0.9707 (max of	2	5031; 2868	0.55983025	-0.2085480		127.0
	ZINC21985931	-7.9	351.362	-0.6727	10	5	254.95	0.44573	145.78	-0.9947	1288.439712237	0.32321	6.5626	-2.0813	0.6	0.37808	0.94678 (max o	7	4941; 4942; 4768; 24	-0.4566377	-0.5828569		67.0
	ZINC04492894	-7.9	351.362	-0.6727	10	5	254.95	0.44573	145.78	-0.9947	1288.439712237	0.32321	6.5626	-2.0813	0.6	0.37878	0.93007 (max o	6	4941; 4942; 4768; 43	-0.4446783	-0.5999892		67.0
	ZINC13543489	-7.9	363.290	-3.2037	11	5	236.25	0.67484	210.9	-10.687	1288.439712237	0.35132	9.0936	-9.1191	0.52174	0.39545	0.89805 (max o	2	6927; 3359	-0.5472299	0.41998595		67.0
	ZINC31158983	-7.9	358.385	0.0798	8	6	260.16	0.379	139.84	-11.814	1288.439712237	0.32321	5.8101	0.2469	0.56	0.51537	0.98897	1	4801	-0.8107504	-0.4063712		49.0
	ZINC31167898	-7.9	429.396	-3.4184	11	5	304.75	0.44581	186.04	-2.5195	1288.439712237	0.26934	9.3083	-12.692	0.6	0.60577	0.99855 (max o	3	6298; 5610; 3914	0.29248258	-0.6988601		49.0
	ZINC72320193	-7.9	416.421	-1.0879	10	6	296.82	0.40981	166.14	-5.3918	1288.439712237	0.27863	6.9778	-3.9045	0.58621	0.54079	0.99279 (max o	7	6376; 6065; 6063; 60	0.08745318	0.47322518		49.0
	ZINC05152238	-7.9	354.358	-0.9613	9	5	258.01	0.41975	137.35	-3.0133	1288.439712237	0.32321	6.8512	-2.9742	0.44	0.33001	0.90535 (max o	2	5794; 5404	0.4105243	0.5939279		49.0
	ZINC77257436	-7.9	402.394	-1.5423	10	6	283.06	0.42973	166.14	-10.796	1288.439712237	0.28858	7.4322	-5.3444	0.57143	0.5244	0.94573 (max o	5	6376; 6063; 6059; 60	0.10440756	0.4595687		49.0
	ZINC37538901	-7.9	413.397	-1.9035	10	4	297.49	0.41265	165.81	-7.7249	1288.439712237	0.27863	7.7934	-6.8316	0.51724	0.41638		0		-0.869538	-0.4839738		49.0
	ZINC72424490	-7.9	440.177	-12.957	16	4	271.51	0.74472	276.39	-33.279	1288.439712237	0.28858	18.847	-44.899	0.42857	0.44429	0.93985 (max o	4	1510; 2178; 2701; 44	-0.372995	-0.3247235		44.0
	ZINC72424490	-7.9	439.169	-14.205	16	3	272.69	0.74583	279.22	-23.349	1288.439712237	0.28858	20.095	-49.222	0.42857	0.46052	0.93686 (max o	5	4932; 1509; 2177; 27	-0.3652584	-0.3663976		44.0
	ZINC03872749	-7.9	361.206	-8.4657	13	4	230.21	0.69893	217.22	-20.626	1288.439712237	0.33668	14.356	-25.145	0.41667	0.39574	0.92257 (max o	6	6997; 6406; 6101; 57	-0.3608177	-0.4100920		44.0
	ZINC03881603	-7.9	362.139	-1.7236	10	5	206.36	0.57385	155.22	-0.33304	1288.439712237	0.38478	7.6135	-4.4795	0.47619	0.39655	0.91628 (max o	3	7466; 5876; 5710	-0.254746	-0.4058295		44.0
	ZINC20758793	-7.9	432.432	-4.3445	11	3	325.31	0.40481	170.8	6.9913	1288.439712237	0.26065	10.234	-16.668	0.48387	0.56497	0.97856 (max o	9	5166; 1809; 1810; 22	0.07577725	-0.6732435		42.0
	ZINC20758833	-7.9	361.377	-2.2603	9	3	273.77	0.38123	130.67	6.4851	1288.439712237	0.31078	8.1502	-7.273	0.53846	0.59602		0		-0.8518739	0.514551		42.0
	ZINC20756353	-7.9	426.472	-0.7877	8	2	313.54	0.35211	143.94	5.5822	1288.439712237	0.26934	6.6776	-2.9245	0.43333	0.51231	0.9702 (max of	17	5034; 4471; 1547; 15	0.6950403	-0.0707061		36.0
	ZINC04087900	-7.9	394.406	-0.5786	8	1	293.39	0.28972	109.85	3.573	1288.439712237	0.27863	6.4685	-2.0766	0.41379	0.49519	0.96682 (max o	12	5034; 4962; 4522; 17	0.73495704	0.00950176		36.0
	ZINC20611262	-7.9	366.352	-1.2962	8	2	271.29	0.34247	118.64	2.9625	1288.439712237	0.29927	7.1861	-4.3312	0.44444	0.5045	0.95201 (max o	6	4962; 4328; 2603; 27	0.7307694	-0.0669386		36.0
	ZINC12878410	-7.9	374.416	-0.579	8	2	284.31	0.32679	118.64	5.8124	1288.439712237	0.29927	6.4689	-1.9347	0.48148	0.55949	0.96885 (max o	4	4472; 4244; 4020; 40	0.30790228	0.6547697		36.0
	ZINC02131608	-7.9	374.416	-0.579	8	2	284.31	0.32679	118.64	5.8124	1288.439712237	0.29927	6.4689	-1.9347	0.48148	0.55949	0.96885 (max o	4	4760; 4472; 4243; 40	0.28735036	0.65190566		36.0
	ZINC20758075	-7.9	445.494	-1.0409	10	3	341.04	0.34427	147.74	7.897	1288.439712237	0.25251	6.9308	-4.1222	0.5	0.58512	0.97533 (max o	10	5947; 5163; 4760; 47	0.3233457	0.7085902		36.0
	ZINC02126235	-7.9	378.428	-1.2257	8	2	282.56	0.39071	143.94	4.9437	1288.439712237	0.31078	7.1156	-3.9439	0.61538	0.57507	0.97331 (max o	5	6274; 5905; 5123; 24	0.6628499	0.11784594		36.0
	ZINC02132171	-7.9	374.416	-0.579	8	2	284.31	0.32679	118.64	6.082	1288.439712237	0.29927	6.4689	-1.9347	0.48148	0.55401	0.93373 (max o	5	6591; 5578; 4472; 42	0.32809073	0.65621		36.0
	ZINC12878528	-7.9	388.442	-0.1246	8	2	298.07	0.31171	118.64	6.5136	1288.439712237	0.28858	6.0145	-0.43177	0.46429	0.5616	0.97037 (max o	12	7062; 6691; 6408; 60	0.2821102	0.7062109		36.0
	ZINC05606969	-7.9	364.313	-2.0709	12	4	226.18	0.64152	197.08	-6.7093	1288.439712237	0.33668	7.9608	-6.151	0.5	0.36859	0.91891 (max o	3	5429; 2093; 3703	-0.7454103	0.5806993		33.0
	ZINC03869249	-7.9	423.170	-13.388	15	2	266.13	0.71134	263.54	-22.848	1288.439712237	0.29927	19.278	-44.736	0.44444	0.4085	0.94605 (max o	9	6403; 6098; 6094; 57	0.658641	0.46331075		33.0
	ZINC03869249	-7.9	424.178	-12.14	15	3	264.95	0.71006	260.71	-32.8	1288.439712237	0.29927	18.03	-40.567	0.44444	0.39014	0.94605 (max o	9	6404; 6099; 6095; 57	0.6288274	0.5104211		33.0
	ZINC04090579	-7.9	404.466	-1.8023	8	1	294.16	0.34842	135.15	6.3348	1288.439712237	0.28858	7.6922	-6.2454	0.57143	0.5201	0.96567 (max o	16	1198; 1404; 1696; 20	0.1260946	-0.9199154		29.0

Structure	Molecule Name	affinity	Total Molweight	cLogP	H-Acceptors	H-Donors	Total Surface Area	Relative PSA	Polar Surface Area	Druglikeness	Kd (nM) @ 20°C	LE from K...	LLE from Kd ...	LELP from Kd (n...	Shape Index	Molecular...	Neighbor Si...	Neig...	Neighbor	Neighbor ...	Neighbor ...	Ring Systems	frequency
	ZINC12902571	-7.9	377.396	-0.4796	9	2	277.5	0.32714	108.41	5.767	1288.439712237	0.29927	6.3695	-1.6026	0.59259	0.42171	0.97346 (max o	3	5869; 5078; 4670	0.8560023	-0.2310700		29.0
	ZINC39024486	-7.9	404.441	-1.2864	9	2	311.82	0.33093	121.72	5.1314	1288.439712237	0.27863	7.1763	-4.6169	0.51724	0.391	0.93985 (max o	4	1116; 1614; 2028; 31	-0.2497292	0.53108966		28.0
	ZINC04000254	-7.9	350.781	-1.2131	7	2	257.96	0.32249	103.26	4.4132	1288.439712237	0.33668	7.103	-3.6031	0.58333	0.37912		0		0.97017324	0.12995854		28.0
	ZINC08764305	-7.9	377.376	-2.1649	10	4	287.95	0.43612	158.6	3.2239	1288.439712237	0.29927	8.0548	-7.2339	0.55556	0.48125		0		0.09651655	0.9874945		28.0
	ZINC67912143	-7.9	422.381	-3.7495	12	7	277.19	0.52217	195.6	-5.5578	1288.439712237	0.27863	9.6394	-13.457	0.44828	0.38765	0.94842 (max o	2	5671; 4862	0.776815	-0.3349217		25.0
	ZINC31156724	-7.9	390.383	-1.9013	10	5	263.95	0.4491	155.14	-4.419	1288.439712237	0.29927	7.7912	-6.3531	0.44444	0.38684		0		-0.8666723	-0.4304497		25.0
	ZINC08740491	-7.9	415.409	1.7412	12	5	299.41	0.45864	173.16	1.4288	1288.439712237	0.26934	4.1487	6.4646	0.53333	0.34885	0.9561 (max of	13	4627; 4626; 4619; 46	-0.1767225	-0.6986970		24.0
	ZINC02119613	-7.9	390.435	-1.0153	7	1	284.73	0.35946	130.06	0.69757	1288.439712237	0.29927	6.9052	-3.3926	0.51852	0.38488	0.92124 (max o	4	4901; 100; 1652; 240	0.1780662	0.16886266		24.0
	ZINC04088879	-7.9	412.373	-1.2511	9	2	305.1	0.35385	134.22	4.7361	1288.439712237	0.26934	7.141	-4.645	0.6	0.43325	0.95837 (max o	15	472; 476; 765; 1500;	0.56724596	0.41734347		24.0
	ZINC35051316	-7.9	408.402	0.2582	9	5	293.01	0.37043	145.91	-6.7495	1288.439712237	0.27863	5.6317	0.92668	0.44828	0.37687	0.88391 (max o	2	3448; 4370	-0.4249439	0.58444583		24.0
	ZINC31160232	-7.9	408.402	0.2582	9	5	293.01	0.37043	145.91	-6.7495	1288.439712237	0.27863	5.6317	0.92668	0.44828	0.36623	0.87477	1	4391	-0.4101337	0.6206027		24.0
	ZINC04353344	-7.9	406.394	-2.6337	11	6	283.25	0.45758	175.28	6.4914	1288.439712237	0.27863	8.5236	-9.4523	0.44828	0.49787	0.96428 (max o	18	5875; 5314; 5297; 49	0.46864274	0.75943524		24.0
	ZINC01769096	-7.9	376.368	-2.0663	10	5	264.4	0.44066	155.05	6.1929	1288.439712237	0.29927	7.9562	-6.9045	0.44444	0.45936	0.96885 (max o	19	5875; 5314; 5297; 49	0.48915592	0.7596476		24.0
	ZINC35653112	-7.9	405.410	-2.5138	11	5	286.96	0.41839	158.29	6.8607	1288.439712237	0.27863	8.4037	-9.022	0.41379	0.49442	0.97176 (max o	4	6010; 4842; 1999; 39	0.4298641	0.83281857		24.0
	ZINC38544245	-7.9	372.456	0.5602	7	4	276.88	0.30858	116.45	-6.4652	1288.439712237	0.31078	5.3297	1.8026	0.53846	0.44971	0.96998 (max o	4	4879; 4878; 3971; 43	-0.0425790	0.62692285		23.0
	ZINC35271337	-7.9	388.455	-0.2632	8	5	281.14	0.3505	136.68	-5.3631	1288.439712237	0.29927	6.1531	-0.87947	0.51852	0.43395	0.9578 (max of	4	6315; 4821; 4424; 30	-0.0529317	0.60895425		23.0
	ZINC72320402	-7.9	390.471	-0.2607	8	6	283.07	0.34832	139.84	-5.146	1288.439712237	0.29927	6.1506	-0.87112	0.48148	0.45157	0.95493 (max o	3	6917; 6062; 4446	-0.3989649	0.80517286		23.0
	ZINC72320401	-7.9	390.471	-0.2607	8	6	283.07	0.34832	139.84	-5.146	1288.439712237	0.29927	6.1506	-0.87112	0.48148	0.45157	0.95493 (max o	3	6917; 6062; 4447	-0.3781151	0.8142527		23.0
	ZINC08790455	-7.9	432.451	-1.866	10	2	322.59	0.35001	137.1	-2.4605	1288.439712237	0.26065	7.7559	-7.1589	0.54839	0.46813	0.96971 (max o	7	1693; 2125; 2298; 32	0.54941726	0.22483227		22.0
	ZINC08789976	-7.9	446.478	-1.5067	10	2	333.59	0.33847	137.1	-0.0023535	1288.439712237	0.25251	7.3966	-5.9669	0.5625	0.47785	0.97533 (max o	11	6226; 5250; 4194; 77	0.5612367	0.18621595		22.0
	ZINC08790292	-7.9	446.478	-1.5067	10	2	333.59	0.33847	137.1	-0.24237	1288.439712237	0.25251	7.3966	-5.9669	0.53125	0.46794	0.97948 (max o	11	6226; 5250; 1216; 12	0.5314524	0.15940222		22.0
	ZINC04085407	-7.9	404.398	-2.5387	10	2	297.83	0.37911	137.1	-0.72047	1288.439712237	0.27863	8.4286	-9.1113	0.62069	0.48116	0.96931 (max o	9	5370; 4193; 4195; 17	0.56959325	0.2254177		22.0
	ZINC08624284	-7.9	350.413	0.26	7	4	267.68	0.31829	107.89	2.1592	1288.439712237	0.32321	5.6299	0.80443	0.64	0.5419	0.92683 (max o	5	4658; 2505; 3730; 37	0.33282605	-0.3659539		22.0
	ZINC08624256	-7.9	354.377	0.0169	7	4	261.77	0.32548	107.89	0.81916	1288.439712237	0.32321	5.873	0.052288	0.6	0.5419	0.92662 (max o	8	5821; 4658; 4184; 21	0.343181	-0.3852143		22.0
	ZINC08643744	-7.9	384.426	-2.4152	8	5	301.13	0.34135	112.33	-0.62565	1288.439712237	0.29927	8.3051	-8.0703	0.62963	0.58386	0.93996 (max o	11	5821; 5455; 5021; 46	0.3427561	-0.4401578		22.0
	ZINC20464940	-7.9	420.450	-1.506	7	4	317.59	0.25769	86.47	-2.7901	1288.439712237	0.27863	7.3959	-5.405	0.55172	0.62669	0.91599 (max o	8	7521; 7397; 7222; 70	-0.3415334	0.05798651		22.0
	ZINC12603662	-7.9	384.426	-2.4152	8	5	301.13	0.34135	112.33	-0.62565	1288.439712237	0.29927	8.3051	-8.0703	0.62963	0.58386	0.93996 (max o	3	7599; 5063; 4184	0.35595474	-0.4576892		22.0
	ZINC04175638	-7.9	448.379	0.5798	11	7	295.9	0.45536	186.37	1.9289	1288.439712237	0.25251	5.3101	2.2962	0.40625	0.42191	0.96877 (max o	16	4260; 99; 175; 583; 9	-0.7248223	0.325765		21.0
	ZINC15657732	-7.9	432.380	0.9255	10	6	289.55	0.4201	166.14	1.9289	1288.439712237	0.26065	4.9644	3.5507	0.41935	0.41946	0.93418 (max o	9	1133; 1424; 1574; 16	-0.7328273	0.34466353		21.0
	ZINC20465423	-7.9	376.398	-3.2887	6	3	259.31	0.22282	83.81	-4.5103	1288.439712237	0.31078	9.1786	-10.582	0.53846	0.5162	0.94605 (max o	4	1003; 1871; 2231; 30	-0.4191222	0.10544591		21.0
	ZINC20465551	-7.9	422.524	-0.1334	9	4	322.07	0.27714	106.53	6.8014	1288.439712237	0.26934	6.0233	-0.49528	0.53333	0.54391	0.91871 (max o	5	6825; 5937; 5554; 47	-0.3586534	0.14127034		21.0
	ZINC08740490	-7.9	415.409	1.7412	12	5	299.41	0.45864	173.16	1.4288	1288.439712237	0.26934	4.1487	6.4646	0.53333	0.34743	0.9561 (max of	13	6192; 5460; 5030; 46	-0.1375395	-0.6614082		20.0
	ZINC08964844	-7.9	401.382	1.4655	12	6	283.5	0.49531	184.16	1.3234	1288.439712237	0.27863	4.4244	5.2597	0.48276	0.31394	0.97649 (max o	12	6205; 6192; 5460; 54	-0.1598347	-0.6255119		20.0
	ZINC05464459	-7.9	431.440	-0.1614	9	4	308.3	0.35641	142.39	0.6376	1288.439712237	0.26065	6.0513	-0.61921	0.51613	0.44546	0.97199 (max o	9	4577; 4392; 2092; 22	-0.3339846	-0.7372477		19.0
	ZINC35270642	-7.9	431.440	-0.1614	9	4	308.3	0.35641	142.39	0.6376	1288.439712237	0.26065	6.0513	-0.61921	0.51613	0.44016	0.97199 (max o	9	4577; 2092; 2473; 25	-0.3558452	-0.7357375		19.0
	ZINC08636371	-7.9	426.924	-0.0622	8	5	303.31	0.38973	146.02	5.7631	1288.439712237	0.28858	5.9521	-0.21554	0.53571	0.44729	0.9339 (max of	4	7164; 6743; 6477; 50	0.46779987	0.17881428		18.0
	ZINC12604003	-7.9	399.898	0.6546	7	4	281.55	0.3593	128.48	3.756	1288.439712237	0.31078	5.2353	2.1063	0.61538	0.42094	0.97533 (max o	10	7334; 5483; 4610; 46	-0.5010699	0.38812217		17.0
	ZINC08254704	-7.9	386.475	0.9184	7	3	287.46	0.33678	134.25	-0.17099	1288.439712237	0.29927	4.9715	3.0688	0.55556	0.44205	0.90441 (max o	3	6182; 5824; 3736	-0.5317415	0.2989653		17.0
	ZINC33979243	-7.9	400.172	-9.4476	13	3	251.05	0.63689	217.44	-40.879	1288.439712237	0.32321	15.338	-29.23	0.56	0.6231	0.94351 (max o	5	4388; 2852; 3053; 30	-0.7369594	0.11289138		17.0
	ZINC33979243	-7.9	399.164	-10.695	13	2	252.23	0.63858	220.27	-36.149	1288.439712237	0.32321	16.585	-33.09	0.56	0.62291	0.94351 (max o	5	2853; 3054; 3056; 34	-0.7089116	0.13638212		17.0
	ZINC19203864	-7.9	364.401	-4.0789	8	3	246.09	0.46434	161.09	9.1306	1288.439712237	0.32321	9.9688	-12.62	0.56	0.41051	0.9654 (max of	4	5120; 4700; 4480; 27	0.1582145	-0.1498647		17.0
	ZINC02100722	-7.9	355.389	0.3809	6	2	263.68	0.27002	103.27	0.93543	1288.439712237	0.31078	5.509	1.2256	0.5	0.44815	0.96438 (max o	5	6072; 5298; 4897; 44	0.6827874	0.6314202		16.0
	ZINC05434135	-7.9	402.449	-0.3147	9	2	304.36	0.30286	108.05	4.9815	1288.439712237	0.27863	6.2046	-1.1295	0.48276	0.52544	0.97176 (max o	8	4181; 936; 1739; 174	0.10753931	0.27926415		14.0

Structure	Molecule Name	affinity	Total Molweight	cLogP	H-Acceptors	H-Donors	Total Surface Area	Relative PSA	Polar Surface Area	Druglikeness	Kd (nM) @ 20°C	LE from K...	LLE from Kd ...	LELP from Kd (n...	Shape Index	Molecular...	Neighbor Si...	Neig...	Neighbor	Neighbor ...	Neighbor ...	Ring Systems	frequency
	ZINC08636552	-7.9	445.518	-0.4183	10	2	339.18	0.28224	111.29	5.6002	1288.439712237	0.25251	6.3082	-1.6566	0.5	0.55591	0.9665 (max of	5	5799; 4991; 2469; 24	0.13065608	0.31216136		14.0
	ZINC05397285	-7.9	422.484	-0.9214	7	0	310.32	0.29502	129.84	-0.80046	1288.439712237	0.26934	6.8113	-3.4209	0.53333	0.51617	0.96832 (max o	5	4990; 4971; 4566; 35	-0.5136246	-0.3279789		14.0
	ZINC20464974	-7.9	401.913	-1.7544	8	5	315.01	0.29618	98.5	4.5081	1288.439712237	0.29927	7.6443	-5.8623	0.55556	0.61699	0.9399 (max of	5	6571; 5929; 4744; 18	-0.3017491	-0.0110992		14.0
	ZINC20464977	-7.9	397.494	-2.4304	9	5	321.85	0.32096	107.73	4.5399	1288.439712237	0.28858	8.3203	-8.4219	0.57143	0.62486	0.9399 (max of	6	7521; 6571; 5929; 47	-0.3128404	0.00681659		14.0
	ZINC20463680	-7.9	408.473	-1.8878	9	3	291.13	0.28881	108.31	6.279	1288.439712237	0.27863	7.7777	-6.7753	0.58621	0.48338	0.92464 (max o	5	6500; 6253; 5850; 38	0.42043936	-0.1491466		14.0
	ZINC04085344	-7.9	389.494	-0.5094	7	2	307.11	0.2699	117.18	5.8028	1288.439712237	0.28858	6.3993	-1.7652	0.60714	0.52733	0.95174 (max o	7	6947; 6423; 4584; 45	0.32243297	0.268022		14.0
	ZINC08299028	-7.9	365.456	-3.6855	9	4	295.69	0.36694	113.58	0.28989	1288.439712237	0.31078	9.5754	-11.859	0.69231	0.5778	0.96721 (max o	6	6175; 6174; 5813; 50	0.10122416	-0.2126804		14.0
	ZINC05414511	-7.9	376.433	-2.1966	10	2	267.69	0.40241	133.5	6.3132	1288.439712237	0.32321	8.0865	-6.7962	0.48	0.54855	0.89438 (max o	2	7157; 6460	-0.0919298	0.3893858		13.0
	ZINC04557300	-7.9	420.413	0.4007	9	6	299.15	0.37319	156.91	-4.8731	1288.439712237	0.26934	5.4892	1.4877	0.5	0.42134	0.97533 (max o	6	6144; 4673; 2018; 29	0.7684391	-0.2748904		13.0
	ZINC04252531	-7.9	420.413	0.4007	9	6	299.15	0.37319	156.91	-4.8731	1288.439712237	0.26934	5.4892	1.4877	0.5	0.42134	0.95579 (max o	5	6144; 4673; 4111; 27	0.7501654	-0.2790222		13.0
	ZINC03604143	-7.9	365.409	-1.8794	8	3	245.3	0.46262	162.71	9.2567	1288.439712237	0.32321	7.7693	-5.8148	0.56	0.38362	0.91611 (max o	5	4480; 4354; 2344; 27	0.10285380	-0.1433941		12.0
	ZINC25073484	-7.9	364.401	-4.0789	8	3	246.09	0.46434	161.09	9.1306	1288.439712237	0.32321	9.9688	-12.62	0.56	0.40396	0.90896 (max o	4	4700; 2344; 3878; 40	0.11944028	-0.1596461		12.0
	ZINC06469312	-7.9	375.349	-4.4746	10	5	249.52	0.49223	168.97	-1.6392	1288.439712237	0.31078	10.365	-14.398	0.46154	0.36996	0.94326 (max o	4	4586; 3517; 4143; 41	-0.2138811	0.48658872		12.0
	ZINC06468452	-7.9	375.349	-4.4746	10	5	249.52	0.49223	168.97	-1.6392	1288.439712237	0.31078	10.365	-14.398	0.46154	0.36996	0.93686 (max o	4	4615; 4586; 4145; 41	-0.2214113	0.46678597		12.0
	ZINC06468485	-7.9	375.349	-4.4746	10	5	249.52	0.49223	168.97	-1.6392	1288.439712237	0.31078	10.365	-14.398	0.46154	0.36996	0.93686 (max o	4	5248; 4586; 4145; 41	-0.2011522	0.4713876		12.0
	ZINC77257636	-7.9	424.397	-2.7103	12	7	290.45	0.49833	195.6	-5.5159	1288.439712237	0.27863	8.6002	-9.7272	0.51724	0.49589	0.95908 (max o	3	5289; 3552; 3553	0.41051874	-0.7881797		12.0
	ZINC08643337	-7.9	412.413	-0.714	9	4	306.04	0.35368	134.19	-9.0846	1288.439712237	0.27863	6.6039	-2.5625	0.68966	0.58705	0.94823 (max o	3	6511; 5519; 5480	-0.3513728	-0.2468765		12.0
	ZINC35271307	-7.9	412.433	0.2102	9	4	295.62	0.37724	150.59	-8.6504	1288.439712237	0.27863	5.6797	0.7544	0.41379	0.52952	0.96682 (max o	8	7900; 7250; 7106; 70	0.43616635	0.29732716		12.0
	ZINC08627148	-7.9	366.436	-4.1022	8	3	269.86	0.29949	110.11	3.4961	1288.439712237	0.31078	9.9921	-13.2	0.57692	0.51382	0.93069 (max o	5	2109; 2112; 2880; 29	-0.0137892	-0.5591539		11.0
	ZINC35271235	-7.9	434.396	-1.4258	10	8	285	0.44856	188.14	-2.6646	1288.439712237	0.26065	7.3157	-5.4701	0.35484	0.35923	0.98656 (max o	8	6314; 5513; 5512; 46	-0.5485586	0.585176		11.0
	ZINC67912192	-7.9	438.468	-1.8092	11	6	308.91	0.42614	175.37	0.52337	1288.439712237	0.26934	7.6991	-6.7171	0.53333	0.5609	0.96589	1	2687	0.92885303	0.19479074		11.0
	ZINC04221832	-7.9	441.503	-1.4281	10	6	315.7	0.45645	196.51	1.9235	1288.439712237	0.26934	7.318	-5.3022	0.53333	0.49732	0.92257 (max o	2	1673; 3655	-0.4016207	-0.4108140		10.0
	ZINC04349745	-7.9	432.380	-0.078	10	7	289.19	0.43134	177.14	-1.9977	1288.439712237	0.26065	5.9679	-0.29925	0.45161	0.3816	0.95771 (max o	8	1163; 1423; 1729; 19	0.76842606	0.06939665		10.0
	ZINC04084291	-7.9	404.466	-1.3969	8	2	284.95	0.38744	143.94	6.4109	1288.439712237	0.28858	7.2868	-4.8406	0.5	0.33141	0.96253 (max o	9	4523; 4084; 949; 121	0.328717	-0.6219047		10.0
	ZINC04085299	-7.9	363.413	-1.4073	7	2	249.83	0.39627	135.07	5.1687	1288.439712237	0.32321	7.2972	-4.3541	0.44	0.26876	0.98671 (max o	9	6409; 4523; 4245; 12	0.31895992	-0.6404562		10.0
	ZINC12883744	-7.9	363.413	-1.4073	7	2	249.83	0.39627	135.07	5.1687	1288.439712237	0.32321	7.2972	-4.3541	0.44	0.26373	0.98671 (max o	9	5356; 4524; 1402; 24	0.34508413	-0.6727717		10.0
	ZINC04085300	-7.9	363.413	-1.4073	7	2	249.83	0.39627	135.07	5.1687	1288.439712237	0.32321	7.2972	-4.3541	0.44	0.26876	0.98671 (max o	9	5356; 4524; 4245; 14	0.36444488	-0.6686722		10.0
	ZINC08253593	-7.9	359.452	-0.4944	8	1	268.6	0.26459	81.91	0.99105	1288.439712237	0.31078	6.3843	-1.5908	0.57692	0.34691	0.8412 (max of	2	5002; 4493	-0.1977209	-0.4044007		10.0
	ZINC12664457	-7.9	372.404	-0.5669	9	2	280.88	0.34787	119.39	5.608	1288.439712237	0.29927	6.4568	-1.8943	0.51852	0.48258	0.97407 (max o	4	6469; 5001; 1185; 37	0.6188967	-0.2554054		10.0
	ZINC08635778	-7.9	377.467	-3.6109	9	3	292.09	0.35742	118.78	2.5568	1288.439712237	0.29927	9.5008	-12.066	0.62963	0.50634	0.96885 (max o	8	4599; 2493; 3280; 37	0.16092195	0.29176205		10.0
	ZINC08299704	-7.9	377.491	-7.1795	9	3	293.47	0.36045	126.19	1.2496	1288.439712237	0.29927	13.069	-23.99	0.62963	0.50503	0.96885 (max o	8	4599; 4178; 2866; 32	0.15057568	0.31138402		10.0
	ZINC08299698	-7.9	391.494	-3.1808	9	3	305.85	0.34134	118.78	0.79107	1288.439712237	0.28858	9.0707	-11.022	0.64286	0.5263	0.98451 (max o	11	5014; 5012; 4599; 41	0.16458283	0.2680049		10.0
	ZINC08299759	-7.9	391.518	-4.8819	9	3	305.74	0.32394	115.16	2.5202	1288.439712237	0.28858	10.772	-16.917	0.64286	0.51805	0.97037 (max o	10	5446; 5013; 4599; 41	0.18058018	0.297945		10.0
	ZINC22936924	-7.9	398.529	-1.2171	7	3	315.17	0.20256	81.29	4.9277	1288.439712237	0.27863	7.107	-4.3681	0.65517	0.49515	0.93975 (max o	6	810; 811; 3028; 3438	-0.5258509	0.04873390		10.0
	ZINC08635777	-7.9	404.489	-2.3167	9	1	315.73	0.31378	98.83	-0.67211	1288.439712237	0.27863	8.2066	-8.3146	0.62069	0.51813	0.97176	1	1766	0.7219156	0.32875752		10.0
	ZINC08299770	-7.9	391.470	-3.5464	9	1	307.01	0.32673	104.82	1.5368	1288.439712237	0.28858	9.4363	-12.289	0.64286	0.53594	0.98451 (max o	7	5440; 5014; 5012; 24	0.21376401	0.24307464		10.0
	ZINC31169353	-7.9	413.397	-2.9205	10	4	298.4	0.41139	165.81	1.5959	1288.439712237	0.27863	8.8104	-10.482	0.58621	0.59812	0.99848 (max o	7	6878; 6641; 6324; 19	-0.1511259	0.7198347		10.0
	ZINC04235948	-7.9	439.490	-0.6195	9	2	323.39	0.26414	104.79	5.938	1288.439712237	0.25251	6.5094	-2.4534	0.5625	0.38797	0.93686 (max o	5	1137; 1713; 2054; 20	-0.2218754	0.304542		9.0
	ZINC19594535	-7.9	362.385	-4.4497	8	3	247.6	0.46151	161.09	7.0306	1288.439712237	0.32321	10.34	-13.767	0.56	0.43729	0.9654 (max of	8	5218; 4817; 2218; 22	-0.4844601	-0.0336463		9.0
	ZINC08298255	-7.9	414.552	-3.4036	8	3	351.23	0.28041	77.92	0.29222	1288.439712237	0.26934	9.2935	-12.637	0.6	0.51401	0.97304 (max o	8	4484; 4157; 2490; 28	-0.1238379	0.04822011		9.0
	ZINC08299018	-7.9	400.525	-3.5285	8	3	322.37	0.27496	90.09	0.36709	1288.439712237	0.27863	9.4184	-12.664	0.62069	0.50363	0.97176 (max o	8	4588; 2490; 2859; 28	-0.1487433	0.07514150		9.0
	ZINC08297688	-7.9	370.499	-3.4585	7	3	300.11	0.26204	80.86	0.35716	1288.439712237	0.29927	9.3484	-11.556	0.66667	0.50523	0.96885 (max o	10	5447; 4588; 4484; 41	-0.1172894	0.06729227		9.0
	ZINC20411560	-7.9	379.519	-1.4507	6	3	294.33	0.23912	74.44	2.1596	1288.439712237	0.29927	7.3406	-4.8475	0.55556	0.46431	0.95123 (max o	7	5176; 4771; 4704; 47	-0.0652797	0.08461043		8.0

Structure	Molecule Name	affinity	Total Molweight	cLogP	H-Acceptors	H-Donors	Total Surface Area	Relative PSA	Polar Surface Area	Druglikeness	Kd (nM) @ 20°C	LE from K...	LLE from Kd ...	LELP from Kd (n...	Shape Index	Molecular...	Neighbor Si...	Neig...	Neighbor	Neighbor ...	Neighbor ...	Ring Systems	frequency
	ZINC20411560	-7.9	379.519	-1.4507	6	3	294.33	0.23912	74.44	2.0856	1288.439712237	0.29927	7.3406	-4.8475	0.55556	0.49449	0.94605 (max o	8	5177; 4772; 4705; 47	-0.0607978	0.12878546		8.0
	ZINC31614071	-7.9	352.301	-3.13	8	2	269.84	0.38408	138.46	-0.13705	1288.439712237	0.31078	9.0199	-10.071	0.61538	0.4224		0		0.79887503	-0.1350982		8.0
	ZINC08762259	-7.9	367.385	-4.2278	11	5	269.44	0.46129	136.88	2.9437	1288.439712237	0.31078	10.118	-13.604	0.46154	0.41265	0.94951 (max o	6	6492; 5864; 5487; 49	-0.5973930	-0.5245104		8.0
	ZINC12661595	-7.9	367.385	-4.2278	11	5	269.44	0.46129	136.88	2.9437	1288.439712237	0.31078	10.118	-13.604	0.46154	0.41325	0.92368 (max o	6	6492; 6158; 5864; 54	-0.641062	-0.5200527		8.0
	ZINC03838797	-7.9	377.396	-1.0913	9	5	282.9	0.40124	150.98	4.0925	1288.439712237	0.29927	6.9812	-3.6465	0.62963	0.52639	0.93046 (max o	3	4033; 3162; 3597	-0.2512152	-0.5040859		8.0
	ZINC03838798	-7.9	365.360	-0.9205	8	5	266.99	0.38769	141.75	2.6546	1288.439712237	0.31078	6.8104	-2.9619	0.57692	0.51632	0.96196 (max o	4	3162; 3595; 3606; 40	-0.2606546	-0.5234346		8.0
	ZINC35464916	-7.9	402.438	-1.1195	9	6	287.44	0.38839	156.91	-9.0196	1288.439712237	0.28858	7.0094	-3.8793	0.5	0.4598	0.97773 (max o	7	5641; 5594; 5210; 52	0.62793994	-0.1533043		8.0
	ZINC31168186	-7.9	402.438	-1.1195	9	6	287.44	0.38839	156.91	-9.0196	1288.439712237	0.28858	7.0094	-3.8793	0.5	0.47017	0.97533 (max o	7	5641; 5594; 5210; 52	0.6682606	-0.1617288		8.0
	ZINC35455015	-7.9	396.431	-1.2041	10	6	285.68	0.41515	158.3	-17.323	1288.439712237	0.29927	7.094	-4.0235	0.62963	0.54055	0.95493 (max o	8	6325; 5630; 5226; 48.	-0.4348297	0.6915451		8.0
	ZINC35454840	-7.9	422.469	-0.5759	10	6	308.96	0.38387	158.3	-18.912	1288.439712237	0.27863	6.4658	-2.0669	0.58621	0.53097	0.97415 (max o	5	5630; 5226; 4828; 44	-0.4269724	0.73266864		8.0
	ZINC04270581	-7.9	418.468	-1.0404	9	2	326.09	0.31559	127.87	-5.8355	1288.439712237	0.26934	6.9303	-3.8627	0.6	0.58461	0.9279 (max of	3	6156; 4560; 3669	0.01975800	-0.4344691		8.0
	ZINC04348410	-7.9	370.309	-1.2533	10	5	250.78	0.47269	155.14	-5.8493	1288.439712237	0.31078	7.1432	-4.0328	0.42308	0.4171	0.9464 (max of	8	6430; 5408; 4983; 49	-0.4105155	0.48328194		8.0
	ZINC03838508	-7.9	362.381	-0.754	8	4	268.68	0.37982	138.95	2.7114	1288.439712237	0.31078	6.6439	-2.4262	0.53846	0.4976	0.9066	1	4915	0.2394064	-0.8823227		8.0
	ZINC03839699	-7.9	410.223	-0.4237	8	4	268.01	0.38271	145.67	-0.13769	1288.439712237	0.32321	6.3136	-1.3109	0.6	0.41073		0		0.4818458	-0.8701454		8.0
	ZINC70666303	-7.9	359.357	-0.8644	8	2	260.62	0.37929	120.55	0.018054	1288.439712237	0.31078	6.7543	-2.7814	0.5	0.32755	0.93361 (max o	4	7109; 6908; 6053; 52	0.4844635	0.37688524		8.0
	ZINC05413712	-7.9	403.370	-3.6138	11	2	294.22	0.43957	147.87	0.72265	1288.439712237	0.27863	9.5037	-12.97	0.62069	0.37944	0.96931 (max o	7	5425; 4995; 4576; 59	-0.6949451	-0.5641691		7.0
	ZINC04081792	-7.9	354.385	-0.2757	7	1	251.52	0.29616	96.54	5.9975	1288.439712237	0.31078	6.1656	-0.88712	0.42308	0.32515	0.89334 (max o	4	711; 1928; 2511; 278	-0.4328052	0.32946676		7.0
	ZINC01632636	-7.9	356.285	-3.0054	8	0	264.08	0.3814	132.86	0.2672	1288.439712237	0.31078	8.8953	-9.6705	0.53846	0.42431	0.9464 (max of	3	2346; 2714; 3128	-0.8573238	-0.2955077		7.0
	ZINC12530151	-7.9	398.461	-1.4409	8	3	295.65	0.27871	119.47	4.2819	1288.439712237	0.27863	7.3308	-5.1714	0.51724	0.57898	0.92527 (max o	5	5059; 1239; 4220; 29	0.2608635	-0.1836710		7.0
	ZINC12530150	-7.9	356.381	-2.6794	8	3	260.72	0.31605	119.47	4.6123	1288.439712237	0.31078	8.5693	-8.6215	0.53846	0.52289	0.95228 (max o	5	5059; 4688; 4218; 21	0.24924171	-0.2047193		7.0
	ZINC12530150	-7.9	355.373	-2.4905	8	2	271.69	0.34366	118.22	5.1902	1288.439712237	0.31078	8.3804	-8.0137	0.53846	0.52289	0.96998 (max o	6	5060; 4689; 4220; 21	0.25022513	-0.2411301		7.0
	ZINC12530151	-7.9	397.454	-1.252	8	2	306.62	0.30451	118.22	4.8308	1288.439712237	0.27863	7.1419	-4.4934	0.51724	0.57898	0.92527 (max o	5	5060; 2973; 4217; 42	0.26347682	-0.2224053		7.0
	ZINC04028710	-7.9	385.209	1.2442	6	4	234.65	0.30854	99.38	-6.4693	1288.439712237	0.35132	4.6457	3.5415	0.56522	0.36597	0.93686 (max o	7	4508; 1677; 2777; 29	0.2627709	0.9308521		7.0
	ZINC31157201	-7.9	381.400	-2.088	8	2	266.7	0.37345	133.19	-8.3912	1288.439712237	0.29927	7.9779	-6.977	0.44444	0.4277	0.97407 (max o	4	5598; 1576; 3452; 43	-0.4615340	0.55813265		7.0
	ZINC31157192	-7.9	381.400	-2.088	8	2	266.7	0.37345	133.19	-8.3912	1288.439712237	0.29927	7.9779	-6.977	0.44444	0.4277	0.97407 (max o	4	5598; 1576; 3452; 43	-0.4644400	0.57978886		7.0
	ZINC35360970	-7.9	428.544	-2.1249	8	6	324.6	0.32696	131.89	-4.0306	1288.439712237	0.26934	8.0148	-7.8892	0.53333	0.54938	0.94515	1	3625	0.8169	-0.1233411		7.0
	ZINC31155350	-7.9	448.419	-2.0505	12	6	308.9	0.44869	176.76	-4.801	1288.439712237	0.26065	7.9404	-7.8667	0.54839	0.45528	0.96018 (max o	4	1573; 1605; 1893; 26	0.5727534	0.45997995		6.0
	ZINC20465177	-7.9	424.496	-0.7555	10	4	319.57	0.3106	115.76	7.6843	1288.439712237	0.26934	6.6454	-2.805	0.53333	0.57	0.93111 (max o	4	6571; 5139; 1868; 43	-0.3608506	-0.0538482		6.0
	ZINC20465177	-7.9	425.504	-2.8652	10	5	333.61	0.33962	116.96	3.7274	1288.439712237	0.26934	8.7551	-10.638	0.53333	0.57	0.92507 (max o	4	6571; 5139; 1869; 43	-0.3432521	-0.0453945		6.0
	ZINC35456943	-7.9	406.470	-1.1587	9	7	290.09	0.38505	160.07	-3.1565	1288.439712237	0.28858	7.0486	-4.0152	0.5	0.47465	0.97288 (max o	3	4833; 3495; 3929	0.88596535	0.23609728		6.0
	ZINC05433904	-7.9	377.420	-1.9525	9	2	281.96	0.37317	125.5	-1.8948	1288.439712237	0.29927	7.8424	-6.5242	0.59259	0.42405	0.97837 (max o	5	6976; 6459; 5424; 32	-0.61678	-0.5578821		6.0
	ZINC49180888	-7.9	418.481	-0.2872	9	6	293.11	0.38088	156.91	-5.2779	1288.439712237	0.27863	6.1771	-1.0308	0.37931	0.4602	0.95372 (max o	3	7747; 7565; 6034	-0.5560826	-0.6671418		6.0
	ZINC03984280	-7.9	370.388	-1.3826	9	2	258.51	0.27504	93.56	3.1164	1288.439712237	0.29927	7.2725	-4.6199	0.51852	0.36244	0.9543 (max of	4	5355; 5073; 4662; 42	0.55407023	0.6622502		6.0
	ZINC12427922	-7.9	368.416	-0.4303	8	1	265.92	0.21811	73.33	2.8517	1288.439712237	0.29927	6.3202	-1.4378	0.44444	0.36948	0.92152 (max o	3	5842; 5355; 4062	0.5456628	0.643523		6.0
	ZINC14616881	-7.9	422.469	-0.5759	10	6	308.96	0.38387	158.3	-18.912	1288.439712237	0.27863	6.4658	-2.0669	0.58621	0.54046	0.97415 (max o	5	5881; 5514; 5511; 46	-0.3944352	0.6463346		6.0
	ZINC34235760	-7.9	388.324	-6.5696	11	4	269.23	0.50901	188.87	-5.5881	1288.439712237	0.29927	12.46	-21.952	0.44444	0.48967	0.96343 (max o	3	6632; 5621; 5219	-0.7996924	0.55046874		6.0
	ZINC06007196	-7.9	410.425	1.9926	8	2	316.4	0.28129	95.12	2.9999	1288.439712237	0.26934	3.8973	7.398	0.6	0.38012	0.8319 (max of	2	7326; 4844	0.2137461	-0.759895		5.0
	ZINC12660351	-7.9	439.535	-1.8209	10	4	310.25	0.35304	178.63	-2.4047	1288.439712237	0.26934	7.7108	-6.7605	0.56667	0.59183	0.85972 (max o	3	6515; 6195; 1202	0.56212085	-0.3117897		5.0
	ZINC31157466	-7.9	365.313	-2.4176	9	5	247.65	0.45556	159.74	-4.1365	1288.439712237	0.31078	8.3075	-7.7791	0.42308	0.27854	0.85775	1	3987	-0.8431819	-0.4625680		5.0
	ZINC08298035	-7.9	350.441	-2.94	8	2	277.08	0.3107	84.92	-1.3803	1288.439712237	0.32321	8.8299	-9.0962	0.64	0.53456	0.98188 (max o	8	5446; 5440; 5439; 50	0.19296286	0.23939721		5.0
	ZINC38197764	-7.9	376.407	-2.6266	7	2	252.11	0.22415	92.45	1.2327	1288.439712237	0.29927	8.5165	-8.7767	0.44444	0.36084	0.96885 (max o	4	2364; 2366; 3246; 39	-0.1671662	-0.9495255		5.0
	ZINC13523811	-7.9	352.342	-3.1539	9	5	246.01	0.46783	162.71	0.41536	1288.439712237	0.32321	9.0438	-9.758	0.52	0.39912		0		-0.3350408	-0.8376093		5.0
	ZINC02384987	-7.9	367.404	-0.8471	7	4	278.28	0.3356	132.89	2.9327	1288.439712237	0.29927	6.737	-2.8306	0.59259	0.4716	0.93686 (max o	3	2741; 3251; 3999	0.24555382	0.7914296		5.0

Structure	Molecule Name	affinity	Total Molweight	cLogP	H-Acceptors	H-Donors	Total Surface Area	Relative PSA	Polar Surface Area	Druglikeness	Kd (nM) @ 20°C	LE from K...	LLE from Kd ...	LELP from Kd (n...	Shape Index	Molecular...	Neighbor Si...	Neig...	Neighbor	Neighbor ...	Neighbor ...	Ring Systems	frequency
	ZINC02033819	-7.9	351.405	-0.5014	6	3	271.93	0.29526	112.66	2.9212	1288.439712237	0.31078	6.3913	-1.6134	0.57692	0.46782	0.96438 (max c	7	6693; 4024; 1639; 23	0.2548288	0.75592345		5.0
	ZINC35270642	-7.9	430.432	-2.331	9	3	309.59	0.35909	148.71	-0.33782	1288.439712237	0.26065	8.2209	-8.9429	0.51613	0.44016	0.96502 (max c	4	2560; 2652; 3363; 34	-0.3116421	-0.806734		5.0
	ZINC03927198	-7.9	394.411	-2.0757	10	3	265.05	0.59276	214.58	11.303	1288.439712237	0.31078	7.9656	-6.679	0.53846	0.37147	0.87816 (max c	2	5321; 4968	-0.3368906	0.2991867		5.0
	ZINC04147954	-7.9	442.491	-1.5204	11	1	322.99	0.36295	143.59	6.5685	1288.439712237	0.26934	7.4103	-5.6449	0.46667	0.56879	0.93975 (max c	4	7157; 3219; 3220; 32	-0.0521915	0.41028276		5.0
	ZINC08254172	-7.9	357.460	-6.757	8	3	286.6	0.33618	104.78	-0.91546	1288.439712237	0.31078	12.647	-21.742	0.65385	0.49307	0.96721 (max c	11	4588; 1759; 2855; 28	-0.0872612	0.03058023		5.0
	ZINC08299297	-7.9	372.471	-3.5136	8	4	292.7	0.31343	101.09	0.24876	1288.439712237	0.29927	9.4035	-11.741	0.62963	0.47727	0.96885 (max c	5	5442; 5009; 4597; 41	-0.1545652	-0.0513060		5.0
	ZINC35024376	-7.9	366.804	-4.1249	7	2	244.41	0.41394	140.86	7.1659	1288.439712237	0.33668	10.015	-12.252	0.54167	0.43407	0.94718 (max c	3	2650; 3480; 3920	-0.5114132	-0.0012995		5.0
	ZINC08254174	-7.9	357.460	-6.757	8	3	286.6	0.33618	104.78	-0.91546	1288.439712237	0.31078	12.647	-21.742	0.65385	0.49307	0.96721 (max c	11	4588; 4153; 2855; 28	-0.0836811	0.05287029		5.0
	ZINC08254176	-7.9	385.514	-4.3345	8	3	327.73	0.30348	81.58	0.27097	1288.439712237	0.28858	10.224	-15.02	0.64286	0.51667	0.97037 (max c	10	5006; 4588; 4484; 41	-0.0852441	0.00591420		5.0
	ZINC20464756	-7.9	386.467	-0.6883	8	3	274.12	0.32756	116.71	-3.0257	1288.439712237	0.31078	6.5782	-2.2147	0.53846	0.53208	0.97533 (max c	6	5551; 4740; 4739; 43	-0.4258879	-0.1151736		5.0
	ZINC20465146	-7.9	403.473	-0.822	8	3	280.77	0.29683	110.72	5.4639	1288.439712237	0.29927	6.7119	-2.7467	0.55556	0.52967	0.97658 (max c	5	5563; 5144; 3843; 38	-0.3994719	-0.0651073		5.0
	ZINC20465152	-7.9	399.510	-0.5789	8	3	286.68	0.29071	110.72	1.4637	1288.439712237	0.29927	6.4688	-1.9344	0.55556	0.52967	0.97658 (max c	5	5563; 5144; 3846; 42	-0.4342785	-0.0595943		5.0
	ZINC20465146	-7.9	404.481	-2.9317	8	4	294.81	0.33031	111.92	1.5867	1288.439712237	0.29927	8.8216	-9.7962	0.55556	0.52967	0.97658 (max c	6	5928; 5564; 5145; 43	-0.3842448	-0.1286355		5.0
	ZINC20465152	-7.9	400.518	-2.6886	8	4	300.72	0.32382	111.92	-2.3742	1288.439712237	0.29927	8.5785	-8.9839	0.55556	0.52967	0.97658 (max c	6	7045; 5564; 5145; 38	-0.4037305	-0.1231743		5.0
	ZINC08299821	-7.9	358.444	-5.8112	8	4	280.43	0.35118	112.12	-1.022	1288.439712237	0.31078	11.701	-18.699	0.61538	0.46214	0.96721 (max c	5	5442; 5441; 4597; 32	-0.1111957	-0.0674361		5.0
	ZINC36369858	-7.9	379.213	-1.0197	7	3	234.08	0.29631	102.4	0.1157	1288.439712237	0.35132	6.9096	-2.9025	0.52174	0.50802	0.92193 (max c	2	5244; 4419	-0.9625594	-0.0734428		5.0
	ZINC13308152	-7.9	363.416	0.5683	9	3	267.82	0.36383	114.55	-3.3637	1288.439712237	0.31078	5.3216	1.8286	0.53846	0.3046	0.93686 (max c	3	860; 1814; 2168	-0.4063286	-0.8562197		4.0
	ZINC15676244	-7.9	420.468	-2.4619	8	3	311.23	0.25499	111.63	5.6012	1288.439712237	0.26065	8.3518	-9.4451	0.58065	0.49953	0.93686 (max c	4	6538; 5893; 1492; 15	-0.2414150	0.46029994		4.0
	ZINC20411560	-7.9	378.511	0.659	6	2	280.29	0.20101	73.24	6.0263	1288.439712237	0.29927	5.2309	2.202	0.55556	0.49191	0.94605 (max c	5	5175; 4770; 1536; 42	-0.0769756	0.16113478		4.0
	ZINC03871266	-7.9	409.112	-4.2064	11	3	220.98	0.56806	160.83	-13.945	1288.439712237	0.35132	10.096	-11.973	0.47826	0.3478	0.92572 (max c	3	6405; 1111; 1384	-0.5700251	-0.7377025		4.0
	ZINC05399531	-7.9	408.501	-1.9935	9	2	306.68	0.34707	108.16	-0.76942	1288.439712237	0.28858	7.8834	-6.9079	0.60714	0.47716	0.88683 (max c	5	910; 929; 2080; 3253	-0.1822897	-0.0923056		4.0
	ZINC12484160	-7.9	379.411	-0.4072	9	3	273.64	0.33844	101.52	2.0768	1288.439712237	0.29927	6.2971	-1.3606	0.55556	0.47039	0.95493 (max c	7	5052; 4645; 2146; 25	-0.6619865	-0.2352572		4.0
	ZINC72324497	-7.9	358.325	-1.2306	8	1	253.15	0.38673	121.83	0.11443	1288.439712237	0.31078	7.1205	-3.9597	0.57692	0.39474	0.94326 (max c	3	2329; 3117; 3979	-0.8779965	0.20515908		4.0
	ZINC04044726	-7.9	368.364	-0.7314	7	0	252.03	0.29326	88.13	-7.9854	1288.439712237	0.29927	6.6213	-2.4439	0.44444	0.39185	0.93058 (max c	3	2404; 4071; 4072	-0.2591605	-0.8960152		4.0
	ZINC04044724	-7.9	368.364	-0.7314	7	0	252.03	0.29326	88.13	-7.9854	1288.439712237	0.29927	6.6213	-2.4439	0.44444	0.39185	0.93058 (max c	3	4073; 2404; 4071	-0.2713345	-0.9107472		4.0
	ZINC04044721	-7.9	368.364	-0.7314	7	0	252.03	0.29326	88.13	-7.9854	1288.439712237	0.29927	6.6213	-2.4439	0.44444	0.39185	0.93058 (max c	3	4073; 2404; 4072	-0.2605996	-0.9272318		4.0
	ZINC20462834	-7.9	401.481	-2.8584	7	1	324.31	0.27387	105.01	4.9	1288.439712237	0.27863	8.7483	-10.259	0.55172	0.59748	0.94529 (max c	7	6763; 4711; 4707; 47	0.01886606	-0.2094998		4.0
	ZINC20760365	-7.9	400.434	-1.0932	9	3	282.97	0.36718	130.83	4.7611	1288.439712237	0.27863	6.9831	-3.9235	0.41379	0.27221	0.95642 (max c	3	2607; 3860; 4333	-0.8107958	0.49226314		4.0
	ZINC20760293	-7.9	401.461	-0.0253	8	3	287.81	0.30815	107.97	4.3334	1288.439712237	0.27863	5.9152	-0.090802	0.48276	0.28336	0.95642 (max c	3	6841; 3018; 3420	0.8513179	0.38537064		4.0
	ZINC03869447	-7.9	423.452	-5.2627	13	5	297.18	0.46214	219.64	1.2977	1288.439712237	0.26934	11.153	-19.539	0.6	0.6156	0.97533 (max c	3	4931; 2717; 3129	0.57204455	0.77059776		4.0
	ZINC04221826	-7.9	425.460	-3.719	11	6	317.92	0.44757	193.91	0.73303	1288.439712237	0.26934	9.6089	-13.808	0.56667	0.5673	0.89582 (max c	5	2100; 2429; 2747; 27	-0.1923636	-0.5007515		4.0
	ZINC03841147	-7.9	375.424	-0.2833	8	3	278.64	0.3183	107.97	5.7602	1288.439712237	0.29927	6.1732	-0.94664	0.62963	0.49056	0.88683 (max c	5	349; 1674; 2754; 359	-0.3756453	-0.2799532		4.0
	ZINC03841141	-7.9	377.438	-1.3835	7	3	276.38	0.26532	95.12	5.1745	1288.439712237	0.29927	7.2734	-4.6229	0.62963	0.45225	0.88492	1	2696	0.7268342	0.4680049		4.0
	ZINC04237152	-7.9	371.436	-0.6278	6	0	267.06	0.30278	108.99	5.5858	1288.439712237	0.31078	6.5177	-2.0201	0.57692	0.44972	0.94927 (max c	3	6143; 2804; 3660	-0.8671933	-0.4862412		4.0
	ZINC35363826	-7.9	388.368	-1.827	10	4	263.15	0.45024	151.98	-6.054	1288.439712237	0.29927	7.7169	-6.1049	0.48148	0.35896	0.94605 (max c	3	2933; 3061; 3636	-0.7781349	0.266793		4.0
	ZINC20760356	-7.9	400.434	-1.0932	9	3	282.97	0.36718	130.83	4.7611	1288.439712237	0.27863	6.9831	-3.9235	0.41379	0.27221	0.95642 (max c	3	4335; 2607; 3860	-0.7912692	0.49575478		4.0
	ZINC31165584	-7.9	426.416	-1.3949	10	6	299.06	0.40674	166.14	-8.9951	1288.439712237	0.26934	7.2848	-5.1789	0.5	0.455	0.96092 (max c	5	6615; 5191; 2260; 30	-0.8035122	-0.0365403		4.0
	ZINC31165576	-7.9	426.416	-1.3949	10	6	299.06	0.40674	166.14	-8.9951	1288.439712237	0.26934	7.2848	-5.1789	0.5	0.455	0.96092 (max c	5	6615; 5191; 2260; 30	-0.7893135	-0.0220567		4.0
	ZINC22936907	-7.9	400.545	-0.7146	7	3	304.12	0.20992	81.29	5.5682	1288.439712237	0.27863	6.6045	-2.5647	0.62069	0.4721	0.93101 (max c	7	4350; 810; 3028; 306	-0.5167466	0.06616910		4.0
	ZINC31169461	-7.9	431.369	-4.62	12	6	288.83	0.50521	198.43	0.77107	1288.439712237	0.26934	10.51	-17.153	0.56667	0.44315	0.95318 (max c	6	4814; 3076; 3077; 34	0.25357705	-0.6498318		4.0
	ZINC72320496	-7.9	368.337	-0.2243	9	4	265.68	0.40831	142.75	-1.8996	1288.439712237	0.31078	6.1142	-0.72173	0.57692	0.39767	0.94326 (max c	3	3116; 3975; 4448	-0.4488231	0.8559172		4.0
	ZINC72320495	-7.9	368.337	-0.2243	9	4	265.68	0.40831	142.75	-1.8996	1288.439712237	0.31078	6.1142	-0.72173	0.57692	0.39767	0.94326 (max c	3	3116; 3975; 4449	-0.4279334	0.8646758		4.0
	ZINC03843348	-7.9	368.381	-3.3848	8	0	280.16	0.35951	132.86	-11.135	1288.439712237	0.31078	9.2747	-10.891	0.53846	0.62243	0.96438 (max c	3	6661; 3169; 3611	-0.7943863	-0.4210704		4.0

Structure	Molecule Name	affinity	Total Molweight	cLogP	H-Acceptors	H-Donors	Total Surface Area	Relative PSA	Polar Surface Area	Druglikeness	Kd (nM) @ 20°C	LE from K...	LLE from Kd ...	LELP from Kd (n...	Shape Index	Molecular...	Neighbor Si...	Neig...	Neighbor	Neighbor ...	Neighbor ...	Ring Systems	frequency
	ZINC03871995	-7.9	414.414	-5.2749	11	2	288.77	0.52201	208.91	5.0035	1288.439712237	0.28858	11.165	-18.279	0.60714	0.49964	0.94573 (max o	3	5467; 4497; 3180	0.45933783	0.49258584		4.0
	ZINC67912734	-7.9	388.368	-1.827	10	4	263.15	0.45024	151.98	-4.331	1288.439712237	0.29927	7.7169	-6.1049	0.48148	0.36725	0.93058 (max o	2	4863; 3107	-0.7958918	-0.3724385		4.0
	ZINC08297642	-7.9	428.579	-3.4261	8	3	366.49	0.26874	77.92	-0.88368	1288.439712237	0.26065	9.316	-13.144	0.67742	0.58847	0.95521 (max o	4	5005; 1455; 1456; 32	0.41654924	-0.0982317		4.0
	ZINC49601640	-7.9	361.365	-3.7858	9	5	255.01	0.44241	159.74	-3.3297	1288.439712237	0.32321	9.6757	-11.713	0.48	0.58091	0.97396 (max o	3	6900; 6665; 3529	0.81160414	-0.0446673		4.0
	ZINC03839692	-7.9	383.467	-1.9442	9	6	284.66	0.39082	158.36	3.9826	1288.439712237	0.29927	7.8341	-6.4965	0.51852	0.47599	0.92418 (max o	9	6699; 6400; 6029; 57	-0.5410153	-0.1732220		4.0
	ZINC31166055	-7.9	388.368	-2.1091	10	5	265.99	0.44566	155.14	-3.1573	1288.439712237	0.29927	7.999	-7.0475	0.48148	0.39433	0.94302 (max o	3	6787; 5238; 3791	-0.8848396	0.12766166		4.0
	ZINC30879705	-7.9	425.499	-0.8486	7	4	316.49	0.29647	108.68	-0.11036	1288.439712237	0.27863	6.7385	-3.0456	0.55172	0.54225	0.97878 (max o	7	5592; 4788; 4758; 18	0.47806188	-0.3405914		4.0
	ZINC35454995	-7.9	387.403	-2.5561	9	4	285.25	0.3953	156.58	-5.0739	1288.439712237	0.29927	8.446	-8.5411	0.59259	0.63438	0.97149 (max o	7	5965; 5629; 5228; 52	-0.885432	0.04351782		4.0
	ZINC15885487	-7.9	372.373	-2.5562	10	8	254.8	0.47692	163.9	-1.9752	1288.439712237	0.31078	8.4461	-8.2251	0.57692	0.44167	0.94008 (max o	3	3921; 3937; 3938	-0.8829939	-0.3247023		4.0
	ZINC72320080	-7.9	391.395	-0.7635	8	4	292.41	0.34103	139.51	-1.3035	1288.439712237	0.28858	6.6534	-2.6457	0.5	0.56809	0.98006 (max o	3	5278; 3969; 3970	0.71678627	0.5803052		4.0
	ZINC01081597	-7.9	365.389	-0.7942	10	4	257.6	0.41421	132.44	4.4154	1288.439712237	0.31078	6.6841	-2.5555	0.46154	0.41325	0.93361 (max o	4	6201; 5739; 5740; 53	-0.5413248	-0.5950288		4.0
	ZINC04060955	-7.9	396.462	-1.1769	9	1	300.36	0.31629	119.08	-31.455	1288.439712237	0.28858	7.0668	-4.0782	0.57143	0.55816	0.9426 (max of	4	5837; 5697; 5361; 40	0.51150507	-0.0436431		4.0
	ZINC04064113	-7.9	424.516	-0.5042	9	1	325.12	0.2922	119.08	-30.388	1288.439712237	0.26934	6.3941	-1.872	0.53333	0.55832	0.9426 (max of	4	6019; 5837; 5361; 40	0.50402075	-0.0633523		4.0
	ZINC05121256	-7.9	364.436	0.0565	6	5	239.12	0.32845	118.22	-2.8615	1288.439712237	0.31078	5.8334	0.1818	0.34615	0.30202	0.94008 (max o	3	4985; 4565; 4116	-0.8251221	0.21130033		4.0
	ZINC05121255	-7.9	364.436	0.0565	6	5	239.12	0.32845	118.22	-2.8615	1288.439712237	0.31078	5.8334	0.1818	0.34615	0.30202	0.94008 (max o	3	4985; 4565; 4117	-0.823439	0.2332638		4.0
	ZINC05414719	-7.9	374.400	-1.3791	10	2	278.12	0.37437	124.6	5.2839	1288.439712237	0.29927	7.269	-4.6082	0.48148	0.52098	0.97149 (max o	7	5805; 4129; 482; 117	0.02517050	0.24538271		4.0
	ZINC05414732	-7.9	410.454	-1.8172	11	2	288.95	0.41412	150.05	6.3333	1288.439712237	0.28858	7.7071	-6.297	0.46429	0.5235	0.97288 (max o	3	2836; 2867; 4127	0.038825	0.2981937		4.0
	ZINC05414737	-7.9	417.468	-0.8845	11	3	314.34	0.36769	136.63	6.2676	1288.439712237	0.26934	6.7744	-3.2839	0.43333	0.53924	0.97533 (max o	4	5805; 1440; 2085; 41	0.04201323	0.21295941		4.0
	ZINC12659939	-7.9	446.503	-2.5422	8	2	329.5	0.4207	189.06	2.8383	1288.439712237	0.26934	8.4321	-9.4385	0.6	0.66601	1 (max of 3)	3	5793; 4563; 4562	0.16077504	-0.7231982		4.0
	ZINC13348409	-7.9	383.349	-4.4475	12	8	254.94	0.57347	198.4	-3.1137	1288.439712237	0.31078	10.337	-14.311	0.46154	0.52717	0.94326 (max o	3	7185; 5082; 4250	-0.7405027	0.152023		4.0
	ZINC13348401	-7.9	383.349	-4.4475	12	8	254.94	0.57347	198.4	-3.1137	1288.439712237	0.31078	10.337	-14.311	0.46154	0.52717	0.94326 (max o	3	7185; 5082; 4251	-0.7723787	0.16675441		4.0
	ZINC31158104	-7.9	388.324	-6.5696	11	4	269.23	0.50901	188.87	-5.5881	1288.439712237	0.29927	12.46	-21.952	0.44444	0.4979	0.95201 (max o	3	6612; 5968; 5602	0.7521789	-0.2379198		4.0
	ZINC31163585	-7.9	432.511	-1.9041	7	1	310.36	0.30229	134.63	0.74206	1288.439712237	0.26065	7.794	-7.3051	0.3871	0.46682	0.93956 (max o	4	6645; 5202; 5200; 52	-0.4827686	0.46254882		4.0
	ZINC49601555	-7.9	445.396	-4.3457	12	6	310.43	0.47985	206.27	-2.4636	1288.439712237	0.26065	10.236	-16.672	0.3871	0.49582	0.98065 (max o	3	7566; 5261; 4854	-0.4953801	-0.7335603		4.0
	ZINC15676244	-7.9	421.476	-2.3303	8	4	325.27	0.28715	112.83	3.3876	1288.439712237	0.26065	8.2202	-8.9402	0.58065	0.45115	0.93686 (max o	3	6539; 5894; 1523	0.16093169	-0.7640347		3.0
	ZINC35442392	-7.9	393.534	-1.6291	8	2	305.48	0.34218	111.11	2.2405	1288.439712237	0.29927	7.519	-5.4436	0.55556	0.45432	0.94291 (max o	12	7405; 7085; 7068; 59	-0.0360537	0.5028731		3.0
	ZINC03839407	-7.9	419.505	-1.2099	9	2	297.29	0.35837	129.05	8.3881	1288.439712237	0.27863	7.0998	-4.3423	0.51724	0.31726	0.93686 (max o	6	335; 431; 2001; 2376	-0.5588028	-0.0826033		3.0
	ZINC04073628	-7.9	396.462	-1.1769	9	1	300.36	0.31629	119.08	-53.141	1288.439712237	0.28858	7.0668	-4.0782	0.57143	0.54843	0.94543	1	2035	0.53179395	-0.1021901		3.0
	ZINC12530150	-7.9	355.373	-2.4905	8	2	271.69	0.34366	118.22	5.1902	1288.439712237	0.31078	8.3804	-8.0137	0.53846	0.52289	0.96998 (max o	7	5060; 4689; 4220; 21	0.27173677	-0.2421732		3.0
	ZINC31157276	-7.9	394.418	-0.2395	8	3	277.8	0.35428	130.36	-10.347	1288.439712237	0.28858	6.1294	-0.82992	0.46429	0.41687	0.95983 (max o	5	4796; 2291; 3453; 34	-0.4762188	0.5208465		3.0
	ZINC02125089	-7.9	372.424	-1.9564	8	3	281.76	0.34384	132.97	5.3856	1288.439712237	0.29927	7.8463	-6.5372	0.59259	0.47368	0.90617 (max o	2	4629; 2409	0.24823934	0.31117046		3.0
	ZINC12603677	-7.9	364.357	-1.6638	10	3	270.94	0.43449	139.83	2.0082	1288.439712237	0.31078	7.5537	-5.3536	0.65385	0.45895	0.92394 (max o	5	5857; 1798; 2113; 25	-0.2605641	-0.6037550		3.0
	ZINC08624369	-7.9	402.469	-1.7554	8	4	305.23	0.29293	117.52	0.43293	1288.439712237	0.27863	7.6453	-6.3001	0.68966	0.49258	0.9398 (max of	4	4173; 2569; 2886; 32	0.18093614	-0.4754592		3.0
	ZINC08627168	-7.9	386.470	-1.4189	7	3	294.07	0.24314	99.5	2.5454	1288.439712237	0.28858	7.3088	-4.9168	0.64286	0.51829	0.91871 (max o	5	6756; 5025; 4609; 46	0.14693256	-0.5321101		3.0
	ZINC05923204	-7.9	390.387	0.8402	8	6	280.38	0.35167	139.84	-4.3466	1288.439712237	0.28858	5.0497	2.9115	0.57143	0.46085	0.9678 (max of	8	1386; 2019; 2020; 20	-0.5029036	0.8069507		3.0
	ZINC08624416	-7.9	364.397	-0.3898	8	4	267.18	0.35631	117.12	1.7841	1288.439712237	0.31078	6.2797	-1.2543	0.65385	0.53096	0.92032 (max o	5	5455; 4658; 4652; 28	0.37622026	-0.4067374		3.0
	ZINC04041889	-7.9	350.351	-0.6256	7	2	216.75	0.5672	166.36	3.1728	1288.439712237	0.35132	6.5155	-1.7807	0.3913	0.26346	0.93319 (max o	4	4956; 3194; 3195; 40	0.5984218	-0.4407383		3.0
	ZINC20465863	-7.9	438.905	-0.446	7	4	333.6	0.26757	96.12	0.68289	1288.439712237	0.26934	6.3359	-1.6559	0.56667	0.54648	0.93121 (max o	4	7055; 4787; 1542; 34	0.36852545	-0.2630475		3.0
	ZINC05397198	-7.9	398.462	-1.7579	7	0	287.37	0.30957	118.94	5.5504	1288.439712237	0.28858	7.6478	-6.0915	0.60714	0.51089	0.94883 (max o	3	354; 2057; 3681	-0.5123915	-0.3839303		3.0
	ZINC08791211	-7.9	444.506	-0.9389	9	1	336.19	0.28258	119.08	8.6017	1288.439712237	0.25251	6.8288	-3.7183	0.53125	0.46174	0.97743 (max o	5	5492; 5032; 4664; 46	0.48970753	0.35594842		3.0
	ZINC20464779	-7.9	399.513	-1.3682	7	3	302.14	0.20524	85.67	3.9028	1288.439712237	0.27863	7.2581	-4.9104	0.58621	0.43413	0.96682 (max o	8	4318; 4312; 1541; 22	-0.4567759	0.23668814		3.0
	ZINC20465569	-7.9	429.539	-1.4382	8	3	324.4	0.22198	94.9	3.9893	1288.439712237	0.26065	7.3281	-5.5177	0.54839	0.44265	0.92602 (max o	5	6585; 2596; 3845; 38	-0.4748902	0.2250056		3.0
	ZINC20465572	-7.9	436.595	-4.403	8	5	360.52	0.26736	83.07	3.0316	1288.439712237	0.26065	10.293	-16.892	0.54839	0.50928	0.91964 (max o	7	7050; 6826; 5938; 55	-0.3318446	-0.5471083		3.0

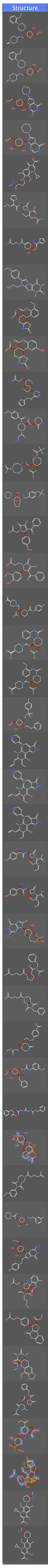

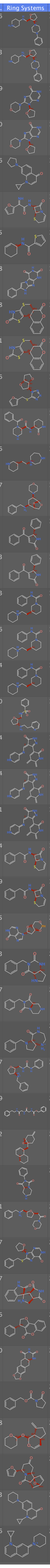

Structure	Molecule Name	affinity	Total Molweight	cLogP	H-Acceptors	H-Donors	Total Surface Area	Relative PSA	Polar Surface Area	Druglikeness	Kd (nM) @ 20°C	LE from K...	LLE from Kd ...	LELP from Kd (n...	Shape Index	Molecular...	Neighbor Si...	Neig...	Neighbor	Neighbor ...	Neighbor ...	Ring Systems	frequency
	ZINC20465572	-7.9	435.587	-2.2933	8	4	346.48	0.23768	81.87	4.9624	1288.439712237	0.26065	8.1832	-8.7982	0.54839	0.5051	0.91964 (max o	4	5560; 5152; 4743; 38	0.64989316	-0.4544316		3.0
	ZINC20465572	-7.9	435.587	-1.4083	8	3	328.72	0.19649	85.25	7.4824	1288.439712237	0.26065	7.2982	-5.4029	0.54839	0.45276	0.93409 (max o	6	6585; 5559; 4754; 47	-0.4869080	0.17090523		3.0
	ZINC02090819	-7.9	368.349	-2.6524	12	5	249.57	0.52879	167.69	2.18	1288.439712237	0.31078	8.5423	-8.5346	0.42308	0.41055	0.96998 (max o	5	5832; 5485; 4468; 42	-0.540798	-0.5748179		3.0
	ZINC12661024	-7.9	353.334	-2.2275	11	4	242.84	0.48056	141.67	2.2599	1288.439712237	0.32321	8.1174	-6.8918	0.44	0.41303	0.92342 (max o	4	6201; 5485; 4001; 41	-0.5574454	-0.5629170		3.0
	ZINC03795958	-7.9	403.452	-1.1476	7	1	288.27	0.22618	89.52	3.4046	1288.439712237	0.27863	7.0375	-4.1187	0.48276	0.38429	0.88683 (max o	2	3245; 3997	0.8309867	-0.0416566		3.0
	ZINC03830485	-7.9	423.381	-2.8488	12	2	292.92	0.54564	201.89	13.42	1288.439712237	0.27863	8.7387	-10.224	0.51724	0.38037	0.981 (max of 7	7	6395; 5878; 4875; 44	-0.2809702	0.8186846		3.0
	ZINC03838799	-7.9	353.398	-1.1547	8	5	256.58	0.48277	169.99	5.4708	1288.439712237	0.33668	7.0446	-3.4297	0.58333	0.4973	0.91928 (max o	3	6093; 5333; 5325	-0.5703403	-0.2493855		3.0
	ZINC03984280	-7.9	369.380	0.5952	9	1	269.48	0.30455	92.31	3.8826	1288.439712237	0.29927	5.2947	1.9888	0.51852	0.36244	0.93385 (max o	2	5072; 4661	-0.6856531	-0.6004758		3.0
	ZINC04042644	-7.9	364.377	-0.3499	7	1	232.66	0.51509	155.36	3.2314	1288.439712237	0.33668	6.2398	-1.0393	0.41667	0.25917	0.92619 (max o	4	5707; 559; 3195; 406	0.61780965	-0.4767968		3.0
	ZINC04041885	-7.9	350.351	-0.6256	7	2	216.75	0.5672	166.36	3.1728	1288.439712237	0.35132	6.5155	-1.7807	0.3913	0.26346	0.93319 (max o	4	4956; 4070; 4069; 31	0.6186142	-0.4384821		3.0
	ZINC04259336	-7.9	367.385	-0.9761	10	1	259.99	0.49883	152.82	1.5965	1288.439712237	0.32321	6.866	-3.02	0.6	0.31085	0.93686 (max o	4	4589; 4547; 4546; 30	-0.5176552	0.44834936		3.0
	ZINC08299494	-7.9	418.472	-4.3812	10	3	312.09	0.34176	135.69	3.6249	1288.439712237	0.26934	10.271	-16.266	0.5	0.53511	0.96343 (max o	4	5806; 5426; 1745; 32	0.13153829	0.2542386		3.0
	ZINC12480996	-7.9	395.542	-0.6366	7	1	328.03	0.23806	85.19	0.67097	1288.439712237	0.28858	6.5265	-2.206	0.5	0.65142	0.9398 (max of 8	8	5909; 5525; 4642; 46	-0.1511340	-0.3048316		3.0
	ZINC12483978	-7.9	405.392	-0.5392	7	2	271.67	0.23702	77	-6.0104	1288.439712237	0.28858	6.4291	-1.8684	0.57143	0.53114	0.93686 (max o	7	7174; 6995; 6503; 59	0.4401473	-0.2322957		3.0
	ZINC20611663	-7.9	365.340	-3.268	7	0	269.57	0.30133	109.8	-6.2595	1288.439712237	0.29927	9.1579	-10.92	0.48148	0.4761	0.91768 (max o	3	5946; 5904; 4282	0.03753489	0.8852125		3.0
	ZINC19794584	-7.9	395.366	-3.1593	8	1	289.68	0.32564	130.03	4.1467	1288.439712237	0.27863	9.0492	-11.339	0.41379	0.48481	0.91903 (max o	3	6247; 5904; 4327	0.02768200	0.86770713		3.0
	ZINC20462884	-7.9	434.486	-2.7637	8	2	342.12	0.29311	117.04	3.6935	1288.439712237	0.26065	8.6536	-10.603	0.58065	0.59634	0.94768 (max o	5	4707; 4708; 4294; 25	0.01704486	-0.2445543		3.0
	ZINC20462879	-7.9	416.496	-2.8645	8	2	335.77	0.29866	117.04	5.0335	1288.439712237	0.26934	8.7544	-10.635	0.56667	0.59479	0.95837 (max o	4	4707; 4294; 4291; 42	0.038507	-0.2467006		3.0
	ZINC20462887	-7.9	446.522	-2.9345	9	2	358.03	0.30802	126.27	5.0862	1288.439712237	0.25251	8.8244	-11.621	0.53125	0.57716	0.92584 (max o	5	4707; 4708; 4644; 42	0.00674313	-0.2262524		3.0
	ZINC20465881	-7.9	436.551	-1.1223	8	4	334.92	0.31291	121.57	-3.9113	1288.439712237	0.26934	7.0122	-4.1668	0.56667	0.54916	0.97975 (max o	7	7398; 5941; 4758; 43	0.4983268	-0.3334570		3.0
	ZINC24737498	-7.9	376.395	-2.1023	8	2	294.3	0.3053	109.22	1.1019	1288.439712237	0.28858	7.9922	-7.2849	0.53571	0.21924	0.9678 (max of 2	2	4351; 4352	0.7433357	-0.4041234		3.0
	ZINC24737498	-7.9	376.395	-2.1023	8	2	294.3	0.3053	109.22	1.1019	1288.439712237	0.28858	7.9922	-7.2849	0.53571	0.21924	0.9678 (max of 2	2	4351; 4353	0.7258096	-0.4176854		3.0
	ZINC24737498	-7.9	376.395	-2.1023	8	2	294.3	0.3053	109.22	1.1019	1288.439712237	0.28858	7.9922	-7.2849	0.53571	0.21924	0.9678 (max of 2	2	4352; 4353	0.744107	-0.4260451		3.0
	ZINC36378490	-7.9	389.431	-1.5936	8	3	273.31	0.41521	162.71	7.0007	1288.439712237	0.29927	7.4835	-5.325	0.59259	0.43147	0.93996 (max o	3	4421; 1923; 3518	-0.5251822	-0.0971764		3.0
	ZINC36378490	-7.9	388.423	-3.7931	8	3	274.1	0.41689	161.09	6.7688	1288.439712237	0.29927	9.683	-12.675	0.59259	0.44815	0.93996 (max o	5	5218; 1924; 3383; 35	-0.5036857	-0.0927079		3.0
	ZINC12726075	-7.9	425.111	-5.1577	12	4	227.54	0.61352	176.51	-11.326	1288.439712237	0.33668	11.048	-15.319	0.45833	0.39802	0.95709 (max o	2	5876; 27	-0.2637094	-0.3301545		2.0
	ZINC04260751	-7.9	358.373	-1.3915	8	1	264.41	0.32147	109.85	0.39731	1288.439712237	0.31078	7.2814	-4.4774	0.57692	0.51286	0.90776 (max o	2	1418; 2811	0.10452922	-0.5305163		2.0
	ZINC05433717	-7.9	360.345	-2.7628	9	0	260.75	0.334	110.29	3.8989	1288.439712237	0.31078	8.6527	-8.8899	0.61538	0.50694	0.88391 (max o	3	5678; 1175; 1441	0.55514824	0.49501827		2.0
	ZINC22922532	-7.9	389.450	-3.3998	8	2	307.72	0.31197	103.21	-3.07	1288.439712237	0.28858	9.2897	-11.781	0.60714	0.50832	0.92206 (max o	3	1560; 1912; 2280	0.01745940	0.5026214		2.0
	ZINC20465955	-7.9	416.496	-0.7121	8	5	333.03	0.29952	104.49	1.912	1288.439712237	0.26934	6.602	-2.6438	0.56667	0.56325	0.88804	1	1876	0.21063717	-0.8003387		2.0
	ZINC04762991	-7.9	412.421	-1.7015	9	3	323.47	0.3426	136.66	-9.3872	1288.439712237	0.26934	7.5914	-6.3172	0.66667	0.59916	0.94768 (max o	4	6736; 6156; 2176; 23	-0.0323912	-0.4300979		2.0
	ZINC35456056	-7.9	391.482	-0.6403	6	3	280.75	0.30016	113.35	-3.3875	1288.439712237	0.28858	6.5302	-2.2188	0.42857	0.36958	0.9678 (max of 5	5	2292; 2293; 2659; 30	-0.1294026	0.43071342		2.0
	ZINC08295676	-7.9	392.411	-2.017	9	0	276.74	0.3779	135.59	5.9268	1288.439712237	0.29927	7.9069	-6.7397	0.59259	0.54292	0.96343 (max o	2	6906; 2489	0.5188641	0.5411787		2.0
	ZINC12603437	-7.9	385.866	-1.3151	7	3	276.49	0.25509	95.84	1.3417	1288.439712237	0.31078	7.205	-4.2316	0.61538	0.48902	0.92368 (max o	5	7015; 2528; 3731; 38	0.07347879	-0.4843071		2.0
	ZINC09034011	-7.9	423.424	-0.3866	10	5	285.09	0.47673	180.52	0.57753	1288.439712237	0.27863	6.2765	-1.3875	0.48276	0.40059	0.97878 (max o	5	1196; 1787; 2121; 25	0.28433123	-0.5535227		2.0
	ZINC20760365	-7.9	401.442	-1.3543	9	3	279.25	0.35875	135.41	3.8291	1288.439712237	0.27863	7.2442	-4.8606	0.41379	0.28532	0.93101 (max o	3	2608; 3861; 4334	-0.6822499	0.61285067		2.0
	ZINC79189899	-7.9	395.342	-0.1783	8	0	280.74	0.33269	111.19	-1.4098	1288.439712237	0.27863	6.0682	-0.63991	0.51724	0.37549	0.9426 (max of 3	3	2334; 2702; 2703	-0.7450301	-0.3945586		2.0
	ZINC02126218	-7.9	375.380	0.5316	9	1	261.3	0.33555	97.41	3.1899	1288.439712237	0.29927	5.3583	1.7763	0.37037	0.33473	0.96617 (max o	3	2731; 3149; 3576	0.93074596	0.03314180		2.0
	ZINC12296161	-7.9	432.451	-1.6066	10	2	330.08	0.35128	144.94	-2.4205	1288.439712237	0.26065	7.4965	-6.1637	0.54839	0.53739	0.94488	1	2913	0.7448613	0.652513		2.0
	ZINC31156431	-7.9	388.368	-1.9969	10	5	262.24	0.45203	155.14	-7.5663	1288.439712237	0.29927	7.8868	-6.6726	0.48148	0.37688	0.93996 (max o	3	5190; 3034; 3068	-0.5775156	0.23389268		2.0
	ZINC04040870	-7.9	356.353	-2.0541	7	0	238.96	0.33922	99.88	2.5289	1288.439712237	0.31078	7.944	-6.6095	0.38462	0.27186	0.95257 (max o	3	4511; 1392; 3193	-0.4107837	0.8361473		2.0
	ZINC02015981	-7.9	389.426	-1.3596	7	1	277.27	0.23515	89.52	2.3577	1288.439712237	0.28858	7.2495	-4.7113	0.46429	0.41125	0.96253 (max o	2	4027; 3245	0.84284425	-0.0600423		2.0
	ZINC02015981	-7.9	390.434	-2.4408	7	2	265.87	0.21255	92.45	0.32387	1288.439712237	0.28858	8.3307	-8.4579	0.46429	0.36237	0.95983 (max o	4	4423; 2364; 2366; 32	-0.1473730	-0.9569156		2.0

Structure	Molecule Name	affinity	Total Molweight	cLogP	H-Acceptors	H-Donors	Total Surface Area	Relative PSA	Polar Surface Area	Druglikeness	Kd (nM) @ 20°C	LE from K...	LLE from Kd ...	LELP from Kd (n...	Shape Index	Molecular...	Neighbor Si...	Neig...	Neighbor	Neighbor ...	Neighbor ...	Ring Systems	frequency
	ZINC05601178	-7.9	356.326	-1.7633	9	4	245.13	0.43014	134.91	-5.757	1288.439712237	0.32321	7.6532	-5.4556	0.48	0.4074	0.95631 (max o	3	3265; 3702; 3988	0.489357	0.27589646		2.0
	ZINC05685483	-7.9	357.385	-1.3033	7	6	239.87	0.30792	116.32	-0.30516	1288.439712237	0.31078	7.1932	-4.1936	0.46154	0.46139	0.94326 (max o	3	4997; 2843; 3267	-0.2093747	0.7449507		2.0
	ZINC08879174	-7.9	372.376	0.1094	8	2	256.08	0.32876	97.33	1.9472	1288.439712237	0.29927	5.7805	0.36556	0.55556	0.31805	0.91768 (max o	3	5035; 1479; 3308	-0.816442	-0.4888779		2.0
	ZINC20410989	-7.9	411.561	-0.9837	7	2	302.27	0.22308	84.84	0.89296	1288.439712237	0.27863	6.8736	-3.5305	0.51724	0.49255	0.95908 (max o	3	4284; 3381; 3833	-0.2463227	0.879353		2.0
	ZINC31158613	-7.9	391.482	-0.6403	6	3	280.75	0.30016	113.35	-3.3875	1288.439712237	0.28858	6.5302	-2.2188	0.42857	0.388	0.9678 (max of	4	654; 2659; 3038; 345	-0.1678735	0.41059205		2.0
	ZINC31169458	-7.9	431.369	-4.62	12	6	288.83	0.50521	198.43	0.77107	1288.439712237	0.26934	10.51	-17.153	0.56667	0.44793	0.95318 (max o	6	4814; 4385; 3076; 30	0.24705566	-0.6695238		2.0
	ZINC35457686	-7.9	371.317	-3.7779	10	4	260.71	0.47087	165.81	1.2603	1288.439712237	0.31078	9.6678	-12.156	0.57692	0.54682	0.98284	1	3502	0.28793755	0.7978566		2.0
	ZINC40165237	-7.9	381.400	-1.2602	8	3	279.88	0.35608	136.35	1.6811	1288.439712237	0.29927	7.1501	-4.2109	0.55556	0.5206	0.96885	1	3524	-0.6734725	-0.7355307		2.0
	ZINC04027688	-7.9	394.379	-1.2789	10	5	274.21	0.42497	156.63	1.978	1288.439712237	0.28858	7.1688	-4.4317	0.57143	0.50116	0.97595 (max o	2	4065; 3630	-0.0403652	0.97031873		2.0
	ZINC04221777	-7.9	389.452	-1.4407	9	5	264.65	0.52677	195.44	4.1283	1288.439712237	0.32321	7.3306	-4.4575	0.52	0.51078	0.91567	1	3654	-0.8520193	0.46752936		2.0
	ZINC05015104	-7.9	388.368	-1.827	10	4	263.15	0.45024	151.98	-6.054	1288.439712237	0.29927	7.7169	-6.1049	0.48148	0.36873	0.93686 (max o	5	5248; 4615; 3248; 32	-0.2058807	0.4128938		2.0
	ZINC00689809	-7.9	356.326	-1.7633	9	4	245.13	0.43014	134.91	-5.757	1288.439712237	0.32321	7.6532	-5.4556	0.48	0.39769	0.95631 (max o	3	4135; 3265; 3702	0.47134486	0.2696372		2.0
	ZINC05923206	-7.9	390.387	0.8402	8	6	280.38	0.35167	139.84	-4.3466	1288.439712237	0.28858	5.0497	2.9115	0.57143	0.46334	0.9678 (max of	8	1386; 2019; 2020; 20	-0.4688974	0.80592716		2.0
	ZINC12603623	-7.9	350.434	-0.7445	8	3	246.87	0.37603	124.55	4.0257	1288.439712237	0.35132	6.6344	-2.1192	0.6087	0.58768		0		0.6026458	0.7772095		2.0
	ZINC08880018	-7.9	371.392	-0.7527	8	3	263.53	0.33655	107.97	4.2775	1288.439712237	0.29927	6.6426	-2.5151	0.59259	0.37988	0.92739	1	3751	0.8697041	0.06353432		2.0
	ZINC12481058	-7.9	445.558	-0.5013	8	1	362.86	0.24277	94.42	4.5806	1288.439712237	0.25251	6.3912	-1.9853	0.5625	0.60142	0.94734 (max o	3	1205; 3384; 3760	-0.0367841	-0.2250143		2.0
	ZINC12603955	-7.9	412.480	-2.387	8	4	306.4	0.26759	107.87	0.94373	1288.439712237	0.27863	8.2769	-8.5669	0.68966	0.53465	0.93395 (max o	3	6755; 2528; 3810	0.08604991	-0.5002713		2.0
	ZINC20410992	-7.9	411.561	-0.9837	7	2	302.27	0.22308	84.84	0.89296	1288.439712237	0.27863	6.8736	-3.5305	0.51724	0.49255	0.95908 (max o	3	3381; 3833; 4283	-0.2669812	0.8702245		2.0
	ZINC20760356	-7.9	401.442	-1.3543	9	3	279.25	0.35875	135.41	3.8291	1288.439712237	0.27863	7.2442	-4.8606	0.41379	0.28532	0.93101 (max o	3	2608; 4336; 3861	-0.7022435	0.6013604		2.0
	ZINC31161343	-7.9	418.393	-1.4692	11	4	298.62	0.43025	161.21	-9.4231	1288.439712237	0.27863	7.3591	-5.2729	0.44828	0.48001	0.91561	1	3892	0.8667765	0.00299028		2.0
	ZINC35442334	-7.9	379.523	-1.6403	7	2	302.54	0.23875	66.32	2.6507	1288.439712237	0.29927	7.5302	-5.481	0.55556	0.46565	0.91157 (max o	8	6640; 4825; 836; 191	-0.1446795	0.6081071		2.0
	ZINC49181881	-7.9	446.411	0.0678	11	3	310.24	0.38822	147.35	6.7757	1288.439712237	0.25251	5.8221	0.26851	0.5	0.50056	0.95465 (max o	3	679; 849; 3952	-0.1701848	-0.9720539		2.0
	ZINC02034076	-7.9	398.391	-0.9735	10	3	267.32	0.43345	150.85	-15.529	1288.439712237	0.29927	6.8634	-3.2529	0.51852	0.48357	0.94605 (max o	3	6465; 6466; 3994	-0.7541485	-0.5658966		2.0
	ZINC01783006	-7.9	398.391	-0.9735	10	3	267.32	0.43345	150.85	-15.529	1288.439712237	0.29927	6.8634	-3.2529	0.51852	0.48357	0.94605 (max o	3	6465; 6466; 4000	-0.7599392	-0.5850642		2.0
	ZINC03839040	-7.9	431.491	0.1636	10	2	329.1	0.30097	112.98	3.6439	1288.439712237	0.26065	5.7263	0.62765	0.48387	0.48243	0.93414 (max o	2	6934; 6091	0.01235807	-0.0407070		2.0
	ZINC03839357	-7.9	392.454	-1.2089	9	2	282.62	0.32616	108.05	4.1271	1288.439712237	0.28858	7.0988	-4.1891	0.53571	0.30628	0.98006 (max o	6	4488; 4487; 430; 200	-0.5901411	0.00484806		2.0
	ZINC03840172	-7.9	427.520	-0.8435	9	6	311.91	0.43913	190.22	6.8545	1288.439712237	0.27863	6.7334	-3.0273	0.51724	0.53504	0.90299 (max o	4	5334; 4914; 1676; 23	-0.4578367	-0.2237375		2.0
	ZINC03841128	-7.9	379.843	0.3927	7	3	271.8	0.28951	98.74	5.7654	1288.439712237	0.31078	5.4972	1.2636	0.61538	0.47026	0.88538 (max o	4	4548; 4491; 557; 404	-0.4069363	-0.3121630		2.0
	ZINC03869571	-7.9	352.342	-7.7802	9	3	240.72	0.39868	134.24	2.8108	1288.439712237	0.32321	13.67	-24.072	0.48	0.44503	0.93007 (max o	4	4647; 2525; 2767; 36	-0.7519501	0.03215420		2.0
	ZINC03875872	-7.9	363.413	-0.6024	7	1	253.48	0.37833	124.07	10.887	1288.439712237	0.32321	6.4923	-1.8638	0.56	0.35723	0.9654	1	4057	0.752953	-0.5769744		2.0
	ZINC03875871	-7.9	363.413	-0.6024	7	1	253.48	0.37833	124.07	10.887	1288.439712237	0.32321	6.4923	-1.8638	0.56	0.36713	0.9654	1	4058	0.7376801	-0.5943857		2.0
	ZINC04081845	-7.9	351.398	0.8397	7	2	255.91	0.29632	96.3	4.9272	1288.439712237	0.32321	5.0502	2.598	0.4	0.29216	0.88798 (max o	2	4530; 4080	-0.0228227	0.9208116		2.0
	ZINC06032132	-7.9	362.377	-0.1664	7	1	251.77	0.37526	97.89	-7.4006	1288.439712237	0.31078	6.0563	-0.53543	0.42308	0.22201	0.98517 (max o	4	6634; 6633; 5761; 56	0.53852755	-0.2419251		2.0
	ZINC08298876	-7.9	376.547	-3.196	7	3	323.25	0.27375	68.69	-4.2377	1288.439712237	0.29927	9.0859	-10.679	0.62963	0.50925	0.96885 (max o	4	5445; 5444; 4916; 45	0.00330393	-0.0584930		2.0
	ZINC08627171	-7.9	406.889	-1.1334	7	4	298.39	0.26613	108.29	0.39664	1288.439712237	0.28858	7.0233	-3.9275	0.64286	0.48291	0.9308 (max of	7	7015; 6756; 6223; 50	0.11785433	-0.4772947		2.0
	ZINC08636279	-7.9	437.491	-0.5232	10	4	333.38	0.35758	147.08	-5.7659	1288.439712237	0.26065	6.4131	-2.0073	0.64516	0.62597	0.95267	1	4264	-0.8183658	0.19145572		2.0
	ZINC15675181	-7.9	437.491	-0.5232	10	4	333.38	0.35758	147.08	-5.7659	1288.439712237	0.26065	6.4131	-2.0073	0.64516	0.62597	0.95267 (max o	2	5856; 4179	-0.8422949	0.16201295		2.0
	ZINC08740484	-7.9	386.371	0.8103	12	5	275.91	0.50121	176.82	1.3234	1288.439712237	0.28858	5.0796	2.8079	0.5	0.33348	0.98451 (max o	12	6759; 6192; 4627; 46	-0.2602884	-0.6555084		2.0
	ZINC12481147	-7.9	373.471	-1.0729	6	0	296.25	0.19396	72.91	4.2075	1288.439712237	0.29927	6.9628	-3.5851	0.55556	0.60773	0.95201 (max o	7	6764; 6213; 6214; 47	-0.1764921	-0.1595403		2.0
	ZINC12481156	-7.9	403.497	-1.1429	7	0	318.51	0.2118	82.14	4.2489	1288.439712237	0.27863	7.0328	-4.1018	0.58621	0.62221	0.95642 (max o	5	6214; 5914; 5846; 47	-0.1267090	-0.1358512		2.0
	ZINC12483138	-7.9	406.457	-1.3607	9	2	282.78	0.28637	97.31	6.2018	1288.439712237	0.27863	7.2506	-4.8835	0.55172	0.44808	0.93686 (max o	6	7172; 6500; 5851; 51	0.44179156	-0.1455443		2.0
	ZINC12604815	-7.9	351.813	-1.3765	7	3	248.19	0.29546	95.12	6.0874	1288.439712237	0.33668	7.2664	-4.0885	0.58333	0.33952	0.92978 (max o	2	5390; 1711	0.7209273	-0.5330296		2.0
	ZINC12658531	-7.9	427.448	0.0981	10	5	311.83	0.38463	154.78	-52.971	1288.439712237	0.26934	5.7918	0.36422	0.46667	0.56298	0.96092 (max o	3	4823; 4659; 3483	0.02556280	0.9666341		2.0

Structure	Molecule Name	affinity	Total Molweight	cLogP	H-Acceptors	H-Donors	Total Surface Area	Relative PSA	Polar Surface Area	Druglikeness	Kd (nM) @ 20°C	LE from K...	LLE from Kd ...	LELP from Kd (n...	Shape Index	Molecular...	Neighbor Si...	Neig...	Neighbor	Neighbor ...	Neighbor ...	Ring Systems	frequency
	ZINC13523529	-7.9	362.389	-2.3544	10	4	267.39	0.44452	150.28	9.5534	1288.439712237	0.31078	8.2443	-7.5758	0.46154	0.49374	0.96998 (max o	8	6527; 6231; 6230; 61	0.00583677	0.78476405		2.0
	ZINC13523529	-7.9	362.389	-2.3544	10	4	267.39	0.44452	150.28	9.5534	1288.439712237	0.31078	8.2443	-7.5758	0.46154	0.49374	0.96998 (max o	9	6528; 6231; 6230; 61	0.02731688	0.7915178		2.0
	ZINC13523529	-7.9	363.397	-4.3322	10	5	256.42	0.42076	151.53	8.9317	1288.439712237	0.31078	10.222	-13.94	0.46154	0.48699	0.9615 (max of	8	6529; 6233; 6232; 61	-0.0025308	0.81535816		2.0
	ZINC13523529	-7.9	363.397	-4.3322	10	5	256.42	0.42076	151.53	9.0745	1288.439712237	0.31078	10.222	-13.94	0.46154	0.49374	0.96998 (max o	8	6530; 6233; 6232; 61	-0.0100798	0.7975003		2.0
	ZINC15675162	-7.9	410.445	-2.4046	10	5	315.04	0.38976	130.79	0.72584	1288.439712237	0.27863	8.2945	-8.6301	0.62069	0.54674	0.94543 (max o	5	5455; 5063; 4614; 37	0.3772319	-0.4509602		2.0
	ZINC17918626	-7.9	410.401	-0.733	8	1	303.24	0.30698	124.04	-0.21215	1288.439712237	0.26934	6.6229	-2.7214	0.43333	0.44006		0		-0.7576924	0.6501688		2.0
	ZINC19370362	-7.9	379.519	-1.4507	6	3	294.33	0.23912	74.44	2.1596	1288.439712237	0.29927	7.3406	-4.8475	0.55556	0.46431	0.95123 (max o	4	5176; 4694; 4286; 42	-0.0653783	0.06144996		2.0
	ZINC19370362	-7.9	379.519	-1.4507	6	3	294.33	0.23912	74.44	2.0856	1288.439712237	0.29927	7.3406	-4.8475	0.55556	0.49449	0.94558 (max o	5	5177; 4695; 4287; 42	-0.0832397	0.096743		2.0
	ZINC22059926	-7.9	389.386	-1.5082	9	1	272.97	0.3459	125.8	3.0344	1288.439712237	0.28858	7.3981	-5.2262	0.5	0.34135	0.9428	1	4769	-0.6365694	-0.7560599		2.0
	ZINC22936916	-7.9	375.447	-2.4681	8	4	290.15	0.30157	111.42	5.1445	1288.439712237	0.29927	8.358	-8.2471	0.66667	0.45562	0.93373 (max o	4	1273; 2618; 4343; 43	-0.4516836	0.061096		2.0
	ZINC22936678	-7.9	379.479	-1.7297	8	4	289.6	0.30214	111.42	6.1644	1288.439712237	0.29927	7.6196	-5.7797	0.62963	0.45131	0.93373 (max o	5	4349; 1273; 2618; 34	-0.4688672	0.09556454		2.0
	ZINC30879097	-7.9	449.614	-0.7858	8	2	369.74	0.20117	69.92	8.885	1288.439712237	0.25251	6.6757	-3.112	0.59375	0.60104	0.91522 (max o	2	7409; 4355	-0.3541473	0.41691524		2.0
	ZINC30879097	-7.9	449.614	-0.7858	8	2	369.74	0.20117	69.92	7.9602	1288.439712237	0.25251	6.6757	-3.112	0.59375	0.59919	0.91522 (max o	2	7410; 4356	-0.3183791	0.44370583		2.0
	ZINC31162117	-7.9	381.423	-0.1984	8	6	269.11	0.35476	135.54	-0.95599	1288.439712237	0.29927	6.0883	-0.66295	0.44444	0.48415	0.94905	1	4807	-0.8337281	-0.5269021		2.0
	ZINC67913882	-7.9	389.332	-4.9553	11	4	258.42	0.51374	175.04	-4.8669	1288.439712237	0.29927	10.845	-16.558	0.44444	0.41256	0.95493 (max o	3	4865; 2319; 2689	-0.3321427	0.851244		2.0
	ZINC71404493	-7.9	389.332	-5.648	11	6	255.52	0.53193	189.2	-2.7509	1288.439712237	0.29927	11.538	-18.873	0.44444	0.3782	0.94302 (max o	3	6747; 6372; 6371	0.163254	0.90471977		2.0
	ZINC01532072	-7.9	410.410	-3.5417	12	2	285.96	0.43489	168.66	3.1017	1288.439712237	0.28858	9.4316	-12.273	0.5	0.58797		0		0.06326730	0.92911726		1.0
	ZINC01853205	-7.9	430.543	0.1157	7	1	332.18	0.22485	103.35	-5.3693	1288.439712237	0.26065	5.7742	0.44388	0.48387	0.48423	0.9112 (max of	3	7578; 6695; 5317	-0.8318857	0.01074229		1.0
	ZINC03784073	-7.9	350.398	-3.6074	8	1	232.65	0.40877	127.67	5.2739	1288.439712237	0.33668	9.4973	-10.715	0.5	0.33754	0.93334	1	2562	0.68682384	0.71770835		1.0
	ZINC03820336	-7.9	377.483	-0.6107	6	1	286.89	0.31726	128.34	-10.012	1288.439712237	0.31078	6.5006	-1.9651	0.53846	0.45551	0.81711	1	3159	-0.8572478	-0.2361202		1.0
	ZINC03830487	-7.9	423.381	-2.8488	12	2	292.92	0.54564	201.89	13.42	1288.439712237	0.27863	8.7387	-10.224	0.51724	0.38037	0.94823 (max o	5	6395; 4496; 3591; 36	-0.3198640	0.8207987		1.0
	ZINC03839336	-7.9	376.436	-0.4018	8	2	260.52	0.3936	127.06	8.7421	1288.439712237	0.31078	6.2917	-1.2929	0.5	0.28989	0.93058 (max o	4	1669; 1670; 1671; 23	-0.6191205	-0.0976629		1.0
	ZINC03839414	-7.9	422.529	-0.2526	8	2	291.77	0.41139	152.36	8.5452	1288.439712237	0.28858	6.1425	-0.87532	0.53571	0.37145	0.91838 (max o	4	4921; 251; 253; 2753	-0.6499857	-0.0675681		1.0
	ZINC03843490	-7.9	351.330	-1.8439	8	0	256.52	0.38781	126.87	-1.0935	1288.439712237	0.32321	7.7338	-5.7049	0.6	0.622		0		0.87578	-0.4460919		1.0
	ZINC03869574	-7.9	352.342	-7.7802	9	3	240.72	0.39868	134.24	2.8108	1288.439712237	0.32321	13.67	-24.072	0.48	0.44672	0.93007 (max o	3	2525; 2767; 3616	-0.7619133	0.01339635		1.0
	ZINC04027801	-7.9	380.352	-1.7333	10	5	260.45	0.44742	156.63	2.863	1288.439712237	0.29927	7.6232	-5.7918	0.55556	0.5022	0.97595 (max o	2	3630; 4064	-0.0364153	0.9553091		1.0
	ZINC04081845	-7.9	352.405	-1.27	7	3	269.95	0.33291	97.5	2.5603	1288.439712237	0.32321	7.1599	-3.9293	0.4	0.29216	0.82533 (max o	2	4530; 4079	-0.0083897	0.93624365		1.0
	ZINC04083366	-7.9	423.511	-0.1946	7	2	329.85	0.2513	117.18	5.2718	1288.439712237	0.26065	6.0845	-0.74658	0.6129	0.48805	0.93686 (max o	5	6423; 4584; 4515; 44	0.3426884	0.32100847		1.0
	ZINC04085633	-7.9	354.465	-0.9097	6	1	262.82	0.26037	73.9	-11.215	1288.439712237	0.32321	6.7996	-2.8146	0.52	0.43218	0.98434 (max o	4	6951; 6715; 1533; 36	0.07404707	0.5297644		1.0
	ZINC04097462	-7.9	408.414	-4.8643	10	2	276.73	0.57753	222.12	8.4031	1288.439712237	0.29927	10.754	-16.254	0.48148	0.45978	0.91109 (max o	2	5292; 3618	-0.3531999	0.34955958		1.0
	ZINC04117655	-7.9	369.396	-0.951	7	3	287.85	0.31065	118.56	0.14307	1288.439712237	0.29927	6.8409	-3.1777	0.55556	0.55761		0		0.30146334	0.9193901		1.0
	ZINC04277728	-7.9	375.467	0.809	7	0	279.55	0.21381	70.16	-5.8622	1288.439712237	0.29927	5.0809	2.7032	0.59259	0.40988	0.90441 (max o	4	4975; 1497; 2451; 32	-0.6794051	0.16043766		1.0
	ZINC04521518	-7.9	398.394	-2.3611	9	3	315.9	0.36382	140.57	1.7225	1288.439712237	0.27863	8.251	-8.4739	0.55172	0.50947		0		0.6721726	0.7354471		1.0
	ZINC04878448	-7.9	375.380	-1.1409	9	5	266.14	0.39453	142.69	6.1386	1288.439712237	0.29927	7.0308	-3.8123	0.44444	0.45827	0.94905 (max o	8	5875; 5314; 5297; 49	0.49547213	0.7772037		1.0
	ZINC05376846	-7.9	383.471	-3.5436	10	5	275.42	0.36936	169.79	5.7345	1288.439712237	0.29927	9.4335	-11.841	0.62963	0.50156	0.88896	1	6165	-0.1119804	0.77409965		1.0
	ZINC05399386	-7.9	388.446	-1.6021	9	1	286.59	0.2901	95.32	-3.9628	1288.439712237	0.28858	7.492	-5.5516	0.64286	0.42524	0.86493 (max o	3	593; 1165; 2078	-0.1329267	-0.2125411		1.0
	ZINC05413326	-7.9	415.536	-0.5598	7	2	319.85	0.29898	102.4	7.2677	1288.439712237	0.27863	6.4497	-2.0091	0.58621	0.46168	0.90561 (max o	2	6149; 4555	-0.7976929	-0.3166522		1.0
	ZINC05434219	-7.9	438.447	-1.8831	12	2	323.14	0.39011	150.38	5.0549	1288.439712237	0.25251	7.773	-7.4576	0.4375	0.46026	0.97743 (max o	8	278; 931; 1169; 1170	-0.6957793	-0.4023178		1.0
	ZINC05916569	-7.9	354.313	-2.018	7	0	272.35	0.32194	115.79	0.39665	1288.439712237	0.31078	7.9079	-6.4933	0.5	0.43416		0		-0.5570370	0.8233559		1.0
	ZINC08623781	-7.9	430.615	-1.8778	7	4	379.22	0.24392	80.62	3.5483	1288.439712237	0.26065	7.7677	-7.2042	0.64516	0.62819	0.80261	1	7510	0.03305825	0.8351926		1.0
	ZINC08627170	-7.9	373.431	-2.6863	8	4	281.73	0.3208	121.18	0.31135	1288.439712237	0.29927	8.5762	-8.9762	0.66667	0.47055	0.92124 (max o	7	7491; 5017; 4609; 41	0.17157988	-0.4944309		1.0
	ZINC08627193	-7.9	352.409	-2.868	8	3	259.83	0.31367	108.73	1.275	1288.439712237	0.32321	8.7579	-8.8734	0.64	0.47295	0.91999 (max o	4	5822; 4222; 2528; 41	0.05847298	-0.5235476		1.0
	ZINC08627194	-7.9	364.464	-1.4347	7	3	274.59	0.26039	99.5	1.8784	1288.439712237	0.31078	7.3246	-4.6164	0.65385	0.47799	0.91999 (max o	2	5822; 4175	0.03565686	-0.5276093		1.0

Structure	Molecule Name	affinity	Total Molweight	cLogP	H-Acceptors	H-Donors	Total Surface Area	Relative PSA	Polar Surface Area	Druglikeness	Kd (nM) @ 20°C	LE from K...	LLE from Kd ...	LELP from Kd (n...	Shape Index	Molecular...	Neighbor Si...	Neig...	Neighbor	Neighbor ...	Neighbor ...	Ring Systems	frequency
	ZINC08643593	-7.9	354.358	-1.4253	9	3	258.47	0.39231	121.47	2.7907	1288.439712237	0.32321	7.3152	-4.4098	0.6	0.52509	0.91999	1	1772	0.25385988	-0.4489917		1.0
	ZINC08740037	-7.9	425.457	-0.1172	9	5	299.76	0.43325	177.81	-2.04	1288.439712237	0.27863	6.0071	-0.42063	0.51724	0.37768		0		-0.8756672	0.29430315		1.0
	ZINC08740123	-7.9	417.409	-2.7955	11	8	284.69	0.46774	181.33	-6.4753	1288.439712237	0.27863	8.6854	-10.033	0.51724	0.42581	0.95372 (max o	3	6370; 5457; 5026	-0.2064197	-0.9527082		1.0
	ZINC08740416	-7.9	382.400	-2.3829	10	2	258.21	0.59645	203.58	8.6611	1288.439712237	0.32321	8.2728	-7.3726	0.52	0.34527	0.91255 (max o	2	5322; 2769	-0.3750333	0.34300154		1.0
	ZINC08876779	-7.9	442.399	-0.8237	10	1	330.77	0.34725	132.45	0.53295	1288.439712237	0.25251	6.7136	-3.2621	0.59375	0.41223	0.90399 (max o	3	5076; 2414; 2937	0.5789197	0.32114178		1.0
	ZINC08918280	-7.9	437.404	-1.3926	12	4	312.58	0.42197	159.38	-0.45211	1288.439712237	0.26065	7.2825	-5.3427	0.54839	0.45869		0		0.09070928	-0.9872234		1.0
	ZINC12481086	-7.9	405.493	-1.2426	8	1	330.29	0.27915	98.33	4.9851	1288.439712237	0.27863	7.1325	-4.4597	0.51724	0.58737	0.96682 (max o	7	5844; 5527; 4716; 46	-0.1663431	-0.3204636		1.0
	ZINC12481093	-7.9	421.560	-0.5647	7	1	336.54	0.29254	113.43	6.4595	1288.439712237	0.27863	6.4546	-2.0267	0.51724	0.58502	0.96682 (max o	6	5844; 5529; 4640; 46	-0.1468867	-0.3258376		1.0
	ZINC12481423	-7.9	436.938	-1.0708	8	1	341.02	0.26116	98.08	4.5875	1288.439712237	0.26934	6.9607	-3.9756	0.53333	0.59377	0.92821 (max o	5	6250; 4712; 2219; 29	-0.0112471	-0.1904112		1.0
	ZINC12529639	-7.9	390.400	-1.8269	6	3	275.71	0.2482	68.97	-9.2111	1288.439712237	0.29927	7.7168	-6.1045	0.55556	0.50603	0.87803 (max o	2	5058; 3389	0.60407746	-0.1248284		1.0
	ZINC12530523	-7.9	407.513	-1.8412	8	3	310.85	0.36526	120.42	5.9062	1288.439712237	0.28858	7.7311	-6.3802	0.57143	0.45774	0.96519 (max o	5	7363; 5521; 4267; 12	-0.1844531	0.08353463		1.0
	ZINC12603443	-7.9	370.425	-0.6332	8	4	255.75	0.3939	133.34	-4.4124	1288.439712237	0.32321	6.5231	-1.9591	0.6	0.52042	0.91611 (max o	4	6750; 6187; 4655; 21	0.21300833	-0.3189346		1.0
	ZINC12603891	-7.9	355.433	-0.4789	8	5	264.42	0.36555	119.92	-2.3848	1288.439712237	0.32321	6.3688	-1.4817	0.64	0.56009	0.91611 (max o	4	6749; 6188; 5829; 32	0.13187356	-0.4025266		1.0
	ZINC12657787	-7.9	425.416	-0.7212	9	2	316.2	0.33065	136.07	-0.66506	1288.439712237	0.26065	6.6111	-2.7669	0.58065	0.50159	0.86493	1	2356	-0.0456908	-0.3981005		1.0
	ZINC12663599	-7.9	354.369	-0.9563	9	3	274.12	0.37232	119.55	4.8367	1288.439712237	0.31078	6.8462	-3.0771	0.65385	0.58087	0.8996	1	4239	0.5531243	-0.6096921		1.0
	ZINC12663599	-7.9	355.377	-2.9341	9	4	263.15	0.34615	120.8	4.6119	1288.439712237	0.31078	8.824	-9.4411	0.65385	0.58087	0.8996	1	4238	0.5379655	-0.5959772		1.0
	ZINC12663605	-7.9	354.389	-1.6548	8	3	256.17	0.29898	100.93	3.6896	1288.439712237	0.31078	7.5447	-5.3247	0.61538	0.43621		0		-0.7213747	0.69141763		1.0
	ZINC13544613	-7.9	405.406	-1.7746	9	1	285.5	0.4166	150.37	10.41	1288.439712237	0.28858	7.6645	-6.1494	0.60714	0.42127	0.93985 (max o	3	6697; 2180; 2368	0.04895901	-0.9313891		1.0
	ZINC15672879	-7.9	352.409	-2.2488	8	1	271.95	0.31256	109.85	5.4496	1288.439712237	0.32321	8.1387	-6.9577	0.64	0.61686	0.88896 (max o	2	980; 3373	-0.7285448	-0.2910955		1.0
	ZINC15672903	-7.9	372.447	-0.9047	8	2	275.27	0.26247	96.58	4.3902	1288.439712237	0.29927	6.7946	-3.023	0.59259	0.46412	0.86427	1	1827	-0.7436716	-0.2746767		1.0
	ZINC15708207	-7.9	391.446	-2.5191	9	3	304.6	0.35223	105.32	4.5679	1288.439712237	0.28858	8.409	-8.7292	0.57143	0.46045	0.96519 (max o	5	7363; 5898; 5521; 38	-0.1982504	0.1164258		1.0
	ZINC15708794	-7.9	432.479	-0.6031	11	2	323.88	0.35235	133.83	5.6182	1288.439712237	0.26065	6.493	-2.3138	0.45161	0.45168	0.8886 (max of	2	6700; 5765	-0.0875321	-0.0581726		1.0
	ZINC16026456	-7.9	368.392	-0.6694	8	3	259.23	0.36223	121.6	2.1174	1288.439712237	0.29927	6.5593	-2.2368	0.40741	0.25171	0.90934 (max o	4	385; 398; 647; 1262	0.10452559	0.8769077		1.0
	ZINC16040552	-7.9	373.364	-0.2528	9	1	265.54	0.36017	106.53	3.1691	1288.439712237	0.29927	6.1427	-0.84472	0.55556	0.34426	0.98141	1	2975	0.62759846	-0.7020346		1.0
	ZINC17144401	-7.9	354.345	-1.1362	9	1	254.12	0.35338	110.6	-6.63	1288.439712237	0.31078	7.0261	-3.656	0.42308	0.37914	0.8996	1	1795	-0.2936139	-0.9073252		1.0
	ZINC18203137	-7.9	369.332	-0.833	8	0	248.76	0.44967	144.41	6.4135	1288.439712237	0.31078	6.7229	-2.6803	0.53846	0.30518	0.9066	1	2389	-0.8535551	0.5129753		1.0
	ZINC19370362	-7.9	378.511	0.659	6	2	280.29	0.20101	73.24	6.0263	1288.439712237	0.29927	5.2309	2.202	0.55556	0.49191	0.92094 (max o	4	5175; 4770; 4285; 42	-0.0773238	0.14024994		1.0
	ZINC19632901	-7.9	354.386	-1.7864	8	2	248.77	0.48583	161.44	3.4583	1288.439712237	0.35132	7.6763	-5.0849	0.65217	0.54144		0		-0.1297432	0.96335965		1.0
	ZINC20411620	-7.9	371.459	-1.7331	7	2	294.21	0.2541	70.08	2.4628	1288.439712237	0.29927	7.623	-5.7911	0.51852	0.46314	0.88991	1	4289	0.89388907	-0.2737952		1.0
	ZINC20412427	-7.9	357.409	0.2672	7	1	267.61	0.251	74.87	2.0584	1288.439712237	0.31078	5.6227	0.85977	0.5	0.45408	0.88991	1	4288	0.88817596	-0.2523433		1.0
	ZINC20462994	-7.9	417.504	-1.8919	8	2	335.3	0.29767	117.2	4.5113	1288.439712237	0.26934	7.7818	-7.0241	0.53333	0.53305	0.934 (max of 2	2	5524; 3836	0.06044684	-0.2087769		1.0
	ZINC20463201	-7.9	407.553	-0.873	7	1	334.88	0.23319	85.19	2.5416	1288.439712237	0.27863	6.7629	-3.1332	0.51724	0.56343	0.9398 (max of	6	5909; 4719; 4640; 46	-0.1353628	-0.2899755		1.0
	ZINC20465212	-7.9	372.420	0.0148	7	3	276.72	0.28317	95.17	4.4379	1288.439712237	0.29927	5.8751	0.049454	0.51852	0.4378	0.88896 (max o	3	7635; 6577; 3001	-0.6254722	0.5420512		1.0
	ZINC20465754	-7.9	391.510	-0.3393	6	4	309.01	0.31925	111.47	4.2321	1288.439712237	0.29927	6.2292	-1.1338	0.55556	0.5332	0.96343 (max o	4	7399; 5569; 4319; 38	0.2164361	-0.1512063		1.0
	ZINC20465818	-7.9	373.495	-2.4599	6	4	304.12	0.23839	83.63	-0.87513	1288.439712237	0.29927	8.3498	-8.2197	0.59259	0.48937	0.92094 (max o	4	7224; 4747; 2599; 30	-0.3916920	-0.6275499		1.0
	ZINC20465818	-7.9	372.487	-1.0936	6	3	290.08	0.20153	82.43	3.7656	1288.439712237	0.29927	6.9835	-3.6542	0.59259	0.48344	0.96343 (max o	5	7225; 7057; 4748; 30	-0.4926787	0.21189326		1.0
	ZINC20465857	-7.9	376.431	-1.9641	8	4	301.52	0.34283	109.26	2.6141	1288.439712237	0.29927	7.854	-6.563	0.55556	0.53487	0.96343 (max o	4	6830; 5591; 3852; 43	0.2504479	-0.1630318		1.0
	ZINC20465887	-7.9	407.513	-1.3745	8	5	319.23	0.37929	136.39	3.0597	1288.439712237	0.28858	7.2644	-4.7629	0.57143	0.55255	0.92464 (max o	3	6831; 6605; 3853	0.30591622	-0.2213907		1.0
	ZINC20503571	-7.9	411.524	-0.5769	7	2	328.42	0.21993	66.32	5.7953	1288.439712237	0.26934	6.4668	-2.1419	0.63333	0.43275	0.9228 (max of	3	1435; 3670; 3857	0.24438018	-0.5967345		1.0
	ZINC20610297	-7.9	377.464	0.7218	7	0	267.23	0.27774	87.62	10.123	1288.439712237	0.31078	5.1681	2.3225	0.53846	0.35816	0.92368	1	6837	-0.7701448	0.60104734		1.0
	ZINC22923301	-7.9	392.502	-5.364	9	4	299.38	0.288	96.25	4.7032	1288.439712237	0.28858	11.254	-18.587	0.57143	0.47937	0.9339 (max of	10	6848; 6321; 5955; 55	-0.1074274	0.5268664		1.0
	ZINC22923301	-7.9	391.494	-3.3862	9	3	310.35	0.31316	95	4.8638	1288.439712237	0.28858	9.2761	-11.734	0.57143	0.49369	0.9339 (max of	10	6849; 6321; 5956; 55	-0.0884853	0.49039307		1.0
	ZINC22923338	-7.9	409.533	-2.4511	9	2	315.48	0.36303	120.34	0.64183	1288.439712237	0.28858	8.341	-8.4936	0.57143	0.49369	0.9339 (max of	10	7405; 7085; 6321; 59	-0.0410661	0.52198166		1.0

Structure	Molecule Name	affinity	Total Molweight	cLogP	H-Acceptors	H-Donors	Total Surface Area	Relative PSA	Polar Surface Area	Druglikeness	Kd (nM) @ 20°C	LE from K...	LLE from Kd ...	LELP from Kd (n...	Shape Index	Molecular...	Neighbor Si...	Neig...	Neighbor	Neighbor ...	Neighbor ...	Ring Systems	frequency
	ZINC22936894	-7.9	376.455	-5.0202	8	4	298.15	0.35737	114.36	3.4896	1288.439712237	0.29927	10.91	-16.775	0.62963	0.48977	0.92418 (max o	4	4347; 1882; 2617; 34	-0.1871267	0.22286557		1.0
	ZINC22936894	-7.9	377.463	-1.9656	8	4	279.1	0.31351	111.42	5.7689	1288.439712237	0.29927	7.8555	-6.568	0.62963	0.43357	0.92418 (max o	4	4349; 1273; 2618; 43	-0.4344268	0.07190265		1.0
	ZINC22936916	-7.9	374.439	-5.5227	8	4	309.2	0.3446	114.36	2.8445	1288.439712237	0.29927	11.413	-18.454	0.66667	0.51449	0.92418 (max o	5	1882; 2617; 3430; 43	-0.1956660	0.18383195		1.0
	ZINC22936916	-7.9	375.447	-3.3531	8	5	307.91	0.34185	108.04	3.3638	1288.439712237	0.29927	9.243	-11.204	0.66667	0.52453	0.88044 (max o	4	5898; 1883; 1884; 43	-0.2101709	0.14846338		1.0
	ZINC30879281	-7.9	362.492	-0.2969	6	2	301.12	0.22665	67.1	5.1168	1288.439712237	0.31078	6.1868	-0.95534	0.61538	0.56532	0.94326	1	4785	0.6421557	0.73528504		1.0
	ZINC31155453	-7.9	394.418	-0.2801	8	1	268.29	0.34373	108.36	-0.891	1288.439712237	0.28858	6.17	-0.97061	0.35714	0.29282	0.88255	1	4406	-0.5274456	-0.8115315		1.0
	ZINC31160999	-7.9	378.376	0.3865	8	2	267.27	0.37389	114.82	-7.4618	1288.439712237	0.29927	5.5034	1.2915	0.40741	0.29859		0		0.60943764	-0.7158543		1.0
	ZINC31169037	-7.9	402.394	-2.0099	10	6	278.14	0.4264	158.3	-6.1289	1288.439712237	0.28858	7.8998	-6.9648	0.5	0.45079	0.96983 (max o	3	7421; 3102; 3903	-0.4498214	0.41492337		1.0
	ZINC35442243	-7.9	370.408	-3.5745	8	2	265.83	0.26878	102.84	4.8439	1288.439712237	0.29927	9.4644	-11.944	0.51852	0.49923	0.88044 (max o	3	5893; 4400; 3814	-0.3101647	0.48061895		1.0
	ZINC35442243	-7.9	369.400	-3.3856	8	1	276.8	0.29776	101.59	5.4097	1288.439712237	0.29927	9.2755	-11.313	0.51852	0.49923	0.88044	1	4399	-0.3457988	0.47087544		1.0
	ZINC35442533	-7.9	390.482	-5.1085	8	5	333.05	0.33445	100.45	3.4799	1288.439712237	0.28858	10.998	-17.702	0.64286	0.47686		0		0.24145176	-0.9642517		1.0
	ZINC35442533	-7.9	389.474	-2.9988	8	4	319.01	0.30516	99.25	5.7212	1288.439712237	0.28858	8.8887	-10.392	0.64286	0.49467	0.89765 (max o	4	7068; 5954; 1280; 16	-0.0141683	0.47357687		1.0
	ZINC35454629	-7.9	437.463	-0.941	9	3	323.23	0.34867	153.42	-0.45976	1288.439712237	0.26065	6.8309	-3.6101	0.48387	0.48961	0.95521 (max o	3	6877; 6323; 5627	-0.8865478	-0.1299659		1.0
	ZINC35454797	-7.9	394.418	-0.2801	8	1	268.29	0.34373	108.36	-0.891	1288.439712237	0.28858	6.17	-0.97061	0.35714	0.29414	0.88255	1	4360	-0.5385239	-0.7935206		1.0
	ZINC35459007	-7.9	388.368	-1.5759	10	4	277.62	0.42677	151.98	-7.1253	1288.439712237	0.29927	7.4658	-5.2658	0.44444	0.44832	0.96617 (max o	3	7439; 6335; 4837	0.83272105	-0.3382697		1.0
	ZINC36369858	-7.9	378.205	-0.8308	7	2	245.05	0.32781	101.15	0.90232	1288.439712237	0.35132	6.7207	-2.3648	0.52174	0.50802	0.94762 (max o	2	5245; 4418	-0.944883	-0.0800614		1.0
	ZINC62001440	-7.9	352.342	-0.4508	9	4	243.35	0.41253	128.56	-6.9771	1288.439712237	0.32321	6.3407	-1.3948	0.4	0.34778		0		-0.9173549	0.24782656		1.0
	ZINC67912690	-7.9	396.434	-1.0197	8	5	255.49	0.39759	144.52	-2.8974	1288.439712237	0.28858	6.9096	-3.5335	0.35714	0.39239	0.93985 (max o	3	3540; 3541; 3954	0.2262857	-0.8507026		1.0
	ZINC67912696	-7.9	398.450	-0.9925	8	6	256.06	0.39694	147.68	-1.5022	1288.439712237	0.28858	6.8824	-3.4392	0.35714	0.39548	0.92774	1	3959	0.8136811	0.49598226		1.0
	ZINC68590696	-7.9	362.330	-3.6103	10	6	234.68	0.53797	161.6	-4.3947	1288.439712237	0.32321	9.5002	-11.17	0.44	0.33846	0.94351 (max o	4	6907; 6677; 6050; 54	0.53524595	0.5714278		1.0
	ZINC68601975	-7.9	394.418	0.7135	8	6	270	0.3877	155.52	-6.4037	1288.439712237	0.28858	5.1764	2.4724	0.42857	0.39438	0.9428	1	1339	-0.8237257	-0.2270865		1.0
	ZINC70666463	-7.9	388.419	0.1486	8	1	293.71	0.25971	88.54	4.9592	1288.439712237	0.28858	5.7413	0.51493	0.53571	0.53411		0		0.9504162	-0.0673413		1.0
	ZINC71404563	-7.9	367.361	-1.0424	11	4	260.5	0.47854	151.33	-41.178	1288.439712237	0.31078	6.9323	-3.3541	0.53846	0.30417		0		-0.5934838	0.75230455		1.0
	ZINC72324436	-7.9	414.421	1.1726	11	1	314.11	0.34074	118.36	6.6661	1288.439712237	0.26934	4.7173	4.3536	0.56667	0.47345		0		0.34516543	0.9163706		1.0
	ZINC72325802	-7.9	372.404	-1.9298	9	3	275.17	0.31468	110.16	4.3198	1288.439712237	0.29927	7.8197	-6.4484	0.59259	0.49119	0.8934 (max of	3	7113; 5691; 5690	-0.1406398	-0.3461553		1.0
	ZINC31167054	-7.9	367.373	-0.8653	8	2	290.28	0.34312	133.19	-7.5278	1288.439712237	0.31078	6.7552	-2.7843	0.57692	0.38944	0.96998 (max o	5	6009; 5239; 2266; 22	-0.0270201	0.8452128		0.0
	ZINC31167046	-7.9	367.373	-0.8653	8	2	290.28	0.34312	133.19	-7.5278	1288.439712237	0.31078	6.7552	-2.7843	0.57692	0.38615	0.96998 (max o	5	6009; 5239; 2266; 22	0.00720680	0.83267367		0.0
	ZINC19419005	-7.9	377.325	-14.226	12	1	306.86	0.49019	186.66	-19.4	1288.439712237	0.31078	20.116	-45.774	0.61538	0.79069		0		0.7796321	-0.6251906		0.0
	ZINC20609966	-7.8	417.436	-1.2301	9	3	315.8	0.35611	144.86	5.6379	1529.749009844	0.26593	7.0455	-4.6256	0.56667	0.60548	0.9561 (max of	5	2725; 2740; 3411; 40	0.04049935	-0.0171580		172.0
	ZINC02140328	-7.8	375.356	-2.3642	9	2	286.98	0.38107	133.86	-3.3869	1529.749009844	0.29548	8.1796	-8.0012	0.66667	0.56695	0.97837 (max o	7	5301; 4903; 868; 273	0.23719223	-0.0648971		172.0
	ZINC02117283	-7.8	375.356	-2.4408	9	2	282.19	0.38754	133.86	1.2511	1529.749009844	0.29548	8.2562	-8.2604	0.59259	0.57002	0.94622 (max o	4	2725; 3565; 3570; 42	0.08460783	0.04759075		172.0
	ZINC04556772	-7.8	376.455	-6.5458	8	4	290.66	0.34749	139.78	-6.9405	1529.749009844	0.29548	12.361	-22.153	0.62963	0.51416	0.96064 (max o	4	5410; 4906; 2459; 28	-0.6340509	-0.6328216		172.0
	ZINC02118008	-7.8	391.355	-2.8547	10	2	292.19	0.4085	143.09	1.2511	1529.749009844	0.28493	8.6701	-10.019	0.60714	0.56029	0.92442 (max o	8	6387; 5699; 5300; 35	0.05184555	0.07407614		172.0
	ZINC02115904	-7.8	433.436	-1.8227	10	2	327.95	0.36396	143.09	4.8467	1529.749009844	0.25735	7.6381	-7.0824	0.54839	0.58427	0.95983 (max o	10	6385; 5699; 5304; 48	0.06354817	0.03729592		172.0
	ZINC02124925	-7.8	376.384	-0.9064	8	1	284.98	0.33287	113.99	3.9973	1529.749009844	0.29548	6.7218	-3.0675	0.51852	0.52821	0.95372 (max o	7	6925; 6385; 6076; 53	0.08619349	0.09074516		172.0
	ZINC02108406	-7.8	403.410	-1.574	9	3	303.54	0.37049	144.86	5.6379	1529.749009844	0.2751	7.3894	-5.7215	0.58621	0.60537	0.9561 (max of	9	6076; 6073; 5699; 56	0.02586763	0.00416365		172.0
	ZINC02159256	-7.8	362.357	-1.1247	8	1	273.98	0.34623	113.99	4.8454	1529.749009844	0.30685	6.9401	-3.6654	0.53846	0.49394	0.96438 (max o	7	7120; 6387; 5709; 57	0.06097258	0.1380604		172.0
	ZINC04899835	-7.8	392.478	0.4164	9	5	301.29	0.32401	148.14	-3.8733	1529.749009844	0.28493	5.399	1.4614	0.53571	0.60123	0.89424	1	4578	-0.0831136	-0.3950129		134.0
	ZINC05707434	-7.8	363.416	-2.4938	9	5	272.8	0.37353	161.97	-0.15312	1529.749009844	0.30685	8.3092	-8.1272	0.57692	0.6185	0.90236 (max o	5	6113; 6041; 5358; 41	-0.1058039	-0.4259742		134.0
	ZINC04025664	-7.8	378.404	-1.9604	9	3	296.53	0.38657	150.65	-9.7358	1529.749009844	0.29548	7.7758	-6.6346	0.51852	0.55566	0.96617 (max o	4	6415; 6416; 6080; 45	-0.0525758	-0.4986693		134.0
	ZINC04025662	-7.8	378.404	-1.9604	9	3	296.53	0.38657	150.65	-9.7358	1529.749009844	0.29548	7.7758	-6.6346	0.51852	0.55598	0.96617 (max o	4	6415; 6416; 6080; 45	-0.0322011	-0.4904469		134.0
	ZINC39205604	-7.8	357.287	-7.4085	12	8	236.1	0.6443	220.43	-5.5171	1529.749009844	0.33242	13.224	-22.287	0.54167	0.62032	0.94034 (max o	3	6895; 6350; 6349	0.4871831	-0.7763408		120.0
	ZINC12371980	-7.8	399.451	-3.9384	11	4	266.86	0.49858	187.08	-0.78796	1529.749009844	0.29548	9.7538	-13.329	0.55556	0.53374	0.98371 (max o	4	2959; 2960; 4636; 46	0.5846877	0.25949758		67.0

Structure	Molecule Name	affinity	Total Molweight	cLogP	H-Acceptors	H-Donors	Total Surface Area	Relative PSA	Polar Surface Area	Druglikeness	Kd (nM) @ 20°C	LE from K...	LLE from Kd ...	LELP from Kd (n...	Shape Index	Molecular...	Neighbor Si...	Neig...	Neighbor	Neighbor ...	Neighbor ...	Ring Systems	frequency
	ZINC12371979	-7.8	399.451	-3.9384	11	4	266.86	0.49858	187.08	-0.78796	1529.749009844	0.29548	9.7538	-13.329	0.55556	0.53374	0.98371 (max o	4	4638; 2959; 2960; 46	0.60345393	0.27110767		67.0
	ZINC21985929	-7.8	351.362	-0.6727	10	5	254.95	0.44573	145.78	-0.9947	1529.749009844	0.31912	6.4881	-2.108	0.6	0.37808	0.92315 (max o	7	4941; 4942; 2456; 29	-0.4234814	-0.6026294		67.0
	ZINC12371978	-7.8	399.451	-3.9384	11	4	266.86	0.49858	187.08	-0.78796	1529.749009844	0.29548	9.7538	-13.329	0.55556	0.5327	0.94905 (max o	3	4638; 3224; 4637	0.58380413	0.28059033		67.0
	ZINC31158986	-7.8	358.385	0.0798	8	6	260.16	0.379	139.84	-11.814	1529.749009844	0.31912	5.7356	0.25006	0.56	0.51455	0.98897	1	4369	-0.8292358	-0.4312714		49.0
	ZINC14486265	-7.8	386.356	-4.8719	11	6	266.96	0.53383	210.24	-4.0803	1529.749009844	0.29548	10.687	-16.488	0.51852	0.42376	0.94905	1	4683	-0.3695981	0.8537631		49.0
	ZINC62001292	-7.8	367.397	0.3556	8	4	277.56	0.33794	117.48	-5.0536	1529.749009844	0.30685	5.4598	1.1589	0.5	0.31985	0.94658 (max o	3	6117; 5000; 4999	-0.8307769	-0.3848569		49.0
	ZINC04043063	-7.8	353.413	0.9764	7	4	267.32	0.31348	108.25	-2.6319	1529.749009844	0.31912	4.839	3.0597	0.52	0.46473	0.96832 (max o	3	6487; 6419; 5745	0.48096707	0.54515177		49.0
	ZINC38557563	-7.8	428.504	-1.6882	10	3	329.61	0.35196	148.1	-62.155	1529.749009844	0.26593	7.5036	-6.3482	0.53333	0.60334	0.9897 (max of	13	6021; 6020; 6019; 60	0.47747895	-0.0812371		47.0
	ZINC04061677	-7.8	398.478	-1.0257	9	2	313.52	0.32824	127.87	-56.322	1529.749009844	0.28493	6.8411	-3.5998	0.57143	0.62001	0.93069 (max o	7	6709; 6422; 6384; 60	0.4389371	-0.0292651		47.0
	ZINC09013130	-7.8	394.339	-0.8122	12	1	281.98	0.47156	147.41	4.1075	1529.749009844	0.28493	6.6276	-2.8505	0.42857	0.34713	0.9339 (max of	3	6245; 5039; 3234	0.05559560	-0.7054930		44.0
	ZINC04090838	-7.8	391.403	-2.8277	10	4	292.62	0.40144	150.9	7.8461	1529.749009844	0.28493	8.6431	-9.9242	0.46429	0.55363	0.94589 (max o	9	5166; 4764; 1009; 15	0.00365629	-0.653287		42.0
	ZINC12889925	-7.8	361.377	-2.2603	9	3	273.77	0.38123	130.67	5.1228	1529.749009844	0.30685	8.0757	-7.3662	0.5	0.55339	0.97269 (max o	4	5167; 2541; 2542; 29	0.05655755	-0.6849490		42.0
	ZINC20758844	-7.8	432.455	-2.6663	11	4	326.3	0.40662	173.76	6.4454	1529.749009844	0.25735	8.4817	-10.36	0.48387	0.55508	0.94488 (max o	6	5077; 2543; 2605; 29	0.01711128	-0.6052642		42.0
	ZINC02131759	-7.8	440.499	-0.3333	8	2	327.3	0.33731	143.94	5.5165	1529.749009844	0.25735	6.1487	-1.2951	0.45161	0.51536	0.9702 (max of	13	1547; 1548; 1549; 27	0.7004979	-0.0907840		36.0
	ZINC04084948	-7.8	410.405	-1.5053	9	2	301.24	0.32565	130.08	5.2502	1529.749009844	0.26593	7.3207	-5.6604	0.4	0.50371	0.95549 (max o	12	5034; 4962; 1782; 21	0.7092566	-0.0372274		36.0
	ZINC02136482	-7.8	360.389	-1.0334	8	2	270.55	0.34341	118.64	5.2365	1529.749009844	0.30685	6.8488	-3.3678	0.46154	0.54838	0.94927 (max o	3	4020; 4021; 4243	0.30251402	0.6352599		36.0
	ZINC13548218	-7.8	403.414	-2.168	10	3	302.52	0.38811	147.74	6.1083	1529.749009844	0.2751	7.9834	-7.8807	0.51724	0.57411	0.96428 (max o	8	5163; 4761; 2242; 25	0.38179934	0.70130974		36.0
	ZINC08876185	-7.8	445.494	-1.0409	10	3	341.04	0.34427	147.74	7.0161	1529.749009844	0.24931	6.8563	-4.1751	0.5	0.58155	0.97319 (max o	8	5163; 4761; 4678; 46	0.3598462	0.6945541		36.0
	ZINC20758333	-7.8	431.467	-1.4953	10	3	327.28	0.35874	147.74	7.217	1529.749009844	0.25735	7.3107	-5.8103	0.48387	0.57859	0.96262 (max o	9	5947; 5163; 2242; 25	0.364855	0.71167684		36.0
	ZINC12888837	-7.8	445.494	-1.0409	10	3	341.04	0.34427	147.74	7.897	1529.749009844	0.24931	6.8563	-4.1751	0.5	0.58512	0.97533 (max o	10	5947; 5163; 4761; 46	0.33913013	0.69347626		36.0
	ZINC08876415	-7.8	431.467	-1.4953	10	3	327.28	0.35874	147.74	7.217	1529.749009844	0.25735	7.3107	-5.8103	0.48387	0.57987	0.96262 (max o	7	5163; 4761; 4678; 46	0.35422242	0.677099		36.0
	ZINC20611253	-7.8	388.442	-0.1246	8	2	298.07	0.31171	118.64	6.5136	1529.749009844	0.28493	5.94	-0.4373	0.46429	0.5616	0.97037 (max o	12	7062; 6691; 6408; 60	0.29716146	0.6898632		36.0
	ZINC04514036	-7.8	370.389	-4.1819	11	4	251.58	0.59838	212.38	-0.65956	1529.749009844	0.31912	9.9973	-13.104	0.56	0.45626	0.96775 (max o	7	5788; 5405; 2766; 36	0.57958126	0.1826038		33.0
	ZINC08876252	-7.8	443.478	-1.808	10	2	331.96	0.34134	152.94	-1.3332	1529.749009844	0.24931	7.6234	-7.2519	0.59375	0.51171	0.971 (max of 7	7	5464; 2127; 2794; 32	0.201897	-0.9036808		29.0
	ZINC12901336	-7.8	391.423	-0.0252	9	2	291.26	0.31168	108.41	3.7647	1529.749009844	0.28493	5.8406	-0.088444	0.60714	0.44427	0.97346 (max o	3	5869; 5078; 4246	0.8713826	-0.1963619		29.0
	ZINC04200817	-7.8	377.417	-3.4293	10	5	266.48	0.49568	184.27	1.8266	1529.749009844	0.31912	9.2447	-10.746	0.52	0.56262	0.93033 (max o	9	6431; 6133; 5764; 49	-0.6258765	-0.1395501		29.0
	ZINC04221597	-7.8	449.547	-4.595	11	6	323.74	0.43192	200.88	2.7641	1529.749009844	0.26593	10.41	-17.279	0.5	0.54837	0.93975 (max o	11	6659; 6431; 6133; 57	-0.5914143	-0.1520109		29.0
	ZINC04221782	-7.8	416.473	-4.1392	10	5	306.93	0.48324	207.18	5.7729	1529.749009844	0.28493	9.9546	-14.527	0.57143	0.63854	0.88286	1	6699	-0.4975334	-0.2102626		29.0
	ZINC04000369	-7.8	406.414	-2.0291	10	2	309.32	0.36593	130.95	4.4099	1529.749009844	0.2751	7.8445	-7.3758	0.51724	0.38438	0.87244 (max o	2	1116; 4501	-0.2380358	0.57255983		28.0
	ZINC04000436	-7.8	376.388	-1.9591	9	2	287.06	0.35947	121.72	4.4099	1529.749009844	0.29548	7.7745	-6.6302	0.51852	0.35668	0.93985 (max o	5	5742; 1116; 2300; 31	-0.2176610	0.5781338		28.0
	ZINC40309281	-7.8	367.178	-2.3719	7	2	240.67	0.34566	103.26	-0.60035	1529.749009844	0.36264	8.1873	-6.5407	0.63636	0.6	0.93686 (max o	2	6896; 5579	-0.2693759	0.9199841		28.0
	ZINC40312880	-7.8	361.377	-2.5215	9	3	279.08	0.38863	138.51	3.8076	1529.749009844	0.30685	8.3369	-8.2175	0.61538	0.56046	0.91693	1	7254	-0.3881803	0.7536084		28.0
	ZINC62236103	-7.8	435.480	-5.1116	10	4	325.43	0.4597	207.19	0.57289	1529.749009844	0.26593	10.927	-19.221	0.53333	0.61775		0		0.6829565	0.652051		28.0
	ZINC08740064	-7.8	409.354	-1.2371	13	2	288.71	0.51346	173.43	1.289	1529.749009844	0.2751	7.0525	-4.4969	0.41379	0.3364	0.9339 (max of	2	5039; 3234	0.09432141	-0.7008322		26.0
	ZINC31157706	-7.8	367.329	-2.5688	9	4	262.38	0.42976	156.58	-0.35579	1529.749009844	0.30685	8.3842	-8.3716	0.53846	0.48773	0.94658 (max o	4	1484; 2042; 2365; 34	-0.1885513	-0.7542243		25.0
	ZINC67912150	-7.8	406.382	-2.8228	11	6	269.34	0.48875	175.37	-4.5827	1529.749009844	0.28493	8.6382	-9.907	0.46429	0.37398	0.94842 (max o	2	4432; 4858	0.7960724	-0.3414392		25.0
	ZINC31159570	-7.8	448.419	-2.3095	12	5	302.17	0.46854	181.44	-2.9612	1529.749009844	0.25735	8.1249	-8.974	0.41935	0.3669	0.93111	1	4795	-0.6887396	0.49269614		25.0
	ZINC31156436	-7.8	406.382	-2.7941	11	6	268.51	0.49026	175.37	-3.5666	1529.749009844	0.28493	8.6095	-9.8063	0.46429	0.37398	0.93111	1	4804	-0.6880724	0.47111642		25.0
	ZINC49878546	-7.8	406.382	-2.8228	11	6	269.34	0.48875	175.37	-4.5827	1529.749009844	0.28493	8.6382	-9.907	0.46429	0.37398	0.94558 (max o	2	5671; 4862	0.7961719	-0.3635227		25.0
	ZINC49774719	-7.8	422.381	-3.6462	12	7	273.6	0.52902	195.6	-3.701	1529.749009844	0.2751	9.4616	-13.254	0.44828	0.37123		0		0.98662496	-0.0131647		25.0
	ZINC08740518	-7.8	431.408	1.3955	13	6	305.76	0.49195	193.39	1.4288	1529.749009844	0.25735	4.4199	5.4225	0.48387	0.33853	0.95739 (max o	7	4625; 1470; 1784; 28	-0.1810948	-0.6279939		24.0
	ZINC08740530	-7.8	441.447	2.4164	12	5	325.45	0.42194	173.16	-4.1017	1529.749009844	0.24931	3.399	9.6922	0.5625	0.37372	0.95267 (max o	7	5029; 2509; 2895; 33	-0.1847327	-0.7168433		24.0
	ZINC05733295	-7.8	354.310	-0.9076	9	4	244.43	0.43137	134.91	-5.8493	1529.749009844	0.31912	6.723	-2.8441	0.48	0.2849	0.9402 (max of	7	5471; 5408; 2477; 24	-0.4251641	0.5440201		24.0

Structure	Molecule Name	affinity	Total Molweight	cLogP	H-Acceptors	H-Donors	Total Surface Area	Relative PSA	Polar Surface Area	Druglikeness	Kd (nM) @ 20°C	LE from K...	LLE from Kd ...	LELP from Kd (n...	Shape Index	Molecular...	Neighbor Si...	Neig...	Neighbor	Neighbor ...	Neighbor ...	Ring Systems	frequency
	ZINC35653110	-7.8	405.410	-2.5138	11	5	286.96	0.41839	158.29	6.8607	1529.749009844	0.2751	8.3292	-9.1376	0.41379	0.48455	0.95908 (max o	4	6010; 4912; 3936; 44	0.42701703	0.81406474		24.0
	ZINC72320114	-7.8	372.456	0.5602	7	4	276.88	0.30858	116.45	-6.4652	1529.749009844	0.30685	5.2552	1.8257	0.53846	0.44971	0.96998 (max o	3	3971; 4424; 4878	-0.0320296	0.64665127		23.0
	ZINC72320112	-7.8	372.456	0.5602	7	4	276.88	0.30858	116.45	-6.4652	1529.749009844	0.30685	5.2552	1.8257	0.53846	0.44971	0.96998 (max o	3	4879; 3971; 4424	-0.0644530	0.6269469		23.0
	ZINC35271348	-7.8	388.455	-0.2632	8	5	281.14	0.3505	136.68	-5.3631	1529.749009844	0.29548	6.0786	-0.89075	0.51852	0.43395	0.96064 (max o	3	6315; 3057; 4396	-0.0746127	0.6095529		23.0
	ZINC72320398	-7.8	406.470	-1.0841	9	7	287.33	0.38875	160.07	-4.0503	1529.749009844	0.28493	6.8995	-3.8048	0.5	0.44224	0.95148 (max o	7	6916; 6678; 6680; 63	0.02755101	-0.7544524		23.0
	ZINC31155045	-7.8	406.470	-1.0554	9	7	286.5	0.38988	160.07	-3.6839	1529.749009844	0.28493	6.8708	-3.7041	0.5	0.50094		0		-0.5690635	-0.7025866		23.0
	ZINC12603445	-7.8	362.381	-0.8923	8	4	278.23	0.34216	117.12	1.0505	1529.749009844	0.30685	6.7077	-2.908	0.69231	0.55834	0.93033 (max o	6	5455; 5018; 4658; 15	0.398222	-0.3973437		22.0
	ZINC12603900	-7.8	366.412	-0.1539	8	4	277.68	0.34284	117.12	2.2539	1529.749009844	0.30685	5.9693	-0.50155	0.65385	0.55562	0.93033 (max o	6	5455; 3730; 4167; 41	0.37539127	-0.3692600		22.0
	ZINC08643739	-7.8	366.436	-2.516	8	5	294.78	0.3487	112.33	0.71435	1529.749009844	0.30685	8.3314	-8.1995	0.65385	0.58116	0.94002 (max o	6	5455; 5021; 4656; 37	0.3622676	-0.4392759		22.0
	ZINC20465339	-7.8	367.419	-0.4572	7	3	269.94	0.25117	85.27	4.7279	1529.749009844	0.30685	6.2726	-1.49	0.57692	0.51876	0.94605 (max o	7	7729; 6825; 4751; 10	-0.3487239	0.09940757		21.0
	ZINC20465385	-7.8	410.488	0.0374	8	4	306.16	0.25888	97.3	5.3844	1529.749009844	0.2751	5.778	0.13595	0.58621	0.54349	0.9427 (max of	6	7729; 5554; 1870; 33	-0.3876941	0.12847394		21.0
	ZINC20465370	-7.8	403.473	-0.5772	8	3	280.77	0.29683	110.72	5.048	1529.749009844	0.29548	6.3926	-1.9534	0.55556	0.529	0.93686 (max o	4	7729; 7049; 5552; 47	-0.3131707	0.10986990		21.0
	ZINC08740519	-7.8	431.408	1.3955	13	6	305.76	0.49195	193.39	1.4288	1529.749009844	0.25735	4.4199	5.4225	0.48387	0.33716	0.95739 (max o	8	1470; 3313; 3756; 41	-0.1262067	-0.6294967		20.0
	ZINC08740500	-7.8	419.828	2.4172	11	5	292.57	0.43518	163.93	1.3996	1529.749009844	0.2751	3.3982	8.7865	0.51724	0.33438	0.97878 (max o	12	6192; 5460; 5459; 50	-0.1710708	-0.6799337		20.0
	ZINC08740517	-7.8	431.408	1.3955	13	6	305.76	0.49195	193.39	1.4288	1529.749009844	0.25735	4.4199	5.4225	0.48387	0.33716	0.95739 (max o	8	6205; 5458; 4625; 14	-0.1587067	-0.6041848		20.0
	ZINC08740506	-7.8	401.382	1.4655	12	6	283.5	0.49531	184.16	1.3234	1529.749009844	0.2751	4.3499	5.3271	0.51724	0.33438	0.97878 (max o	14	6192; 5460; 5459; 54	-0.1560237	-0.6552965		20.0
	ZINC08740487	-7.8	415.409	1.7412	12	5	299.41	0.45864	173.16	1.4288	1529.749009844	0.26593	4.0742	6.5475	0.53333	0.34743	0.9561 (max of	13	6759; 6484; 6205; 61	-0.1757138	-0.6123344		20.0
	ZINC08740498	-7.8	419.828	2.4172	11	5	292.57	0.43518	163.93	1.3996	1529.749009844	0.2751	3.3982	8.7865	0.51724	0.33438	0.97878 (max o	12	6759; 6484; 6205; 61	-0.2163796	-0.630734		20.0
	ZINC13431052	-7.8	445.237	-10.296	15	4	286.85	0.65069	263.59	-43.957	1529.749009844	0.28493	16.111	-36.136	0.60714	0.65107	0.93686 (max o	4	6526; 6228; 5873; 37	-0.5895536	-0.3801326		20.0
	ZINC03806413	-7.8	359.353	0.4743	9	3	260.05	0.37993	120.69	-21.328	1529.749009844	0.31912	5.3411	1.4863	0.64	0.44605		0		0.4945395	0.82815915		20.0
	ZINC05464460	-7.8	431.440	-0.1614	9	4	308.3	0.35641	142.39	0.6376	1529.749009844	0.25735	5.9768	-0.62715	0.51613	0.46025	0.97199 (max o	8	1899; 2092; 2473; 25	-0.3552221	-0.7692871		19.0
	ZINC08636313	-7.8	408.521	-0.055	8	4	300.95	0.34793	131.72	4.4037	1529.749009844	0.28493	5.8704	-0.19303	0.60714	0.45778	0.93686 (max o	10	7334; 5824; 5483; 28	-0.4938727	0.3675374		17.0
	ZINC03830216	-7.8	365.409	-1.8794	8	3	245.3	0.46262	162.71	9.2567	1529.749009844	0.31912	7.6948	-5.8893	0.56	0.38362	0.91611 (max o	5	4700; 2779; 3160; 40	0.13983954	-0.1579261		17.0
	ZINC11616718	-7.8	376.388	-3.4633	8	1	256.5	0.4414	154.97	10.967	1529.749009844	0.30685	9.2787	-11.287	0.5	0.34338	0.96721 (max o	6	5346; 4982; 1963; 23	0.04514516	-0.0549256		17.0
	ZINC03978033	-7.8	376.388	-3.4633	8	1	256.5	0.4414	154.97	10.967	1529.749009844	0.30685	9.2787	-11.287	0.5	0.34338	0.96721 (max o	6	6130; 4939; 4635; 19	0.08872176	-0.0505006		17.0
	ZINC20410372	-7.8	364.401	-4.0789	8	3	246.09	0.46434	161.09	9.1306	1529.749009844	0.31912	9.8943	-12.782	0.56	0.40396	0.9654 (max of	5	2779; 3830; 4275; 43	0.1547896	-0.1732677		17.0
	ZINC02090528	-7.8	355.389	0.3809	6	2	263.68	0.27002	103.27	0.15053	1529.749009844	0.30685	5.4345	1.2413	0.5	0.4614	0.94951 (max o	5	6072; 5298; 4897; 40	0.7011629	0.63822776		16.0
	ZINC02090526	-7.8	355.389	0.3809	6	2	263.68	0.27002	103.27	0.15053	1529.749009844	0.30685	5.4345	1.2413	0.5	0.46057	0.94951 (max o	5	6072; 5298; 4897; 40	0.6832339	0.61154944		16.0
	ZINC35271195	-7.8	426.460	0.0489	9	4	309.91	0.35985	150.59	-12.343	1529.749009844	0.26593	5.7665	0.18388	0.43333	0.53443	0.96589 (max o	5	7106; 7083; 6874; 52	0.39670864	0.28298157		16.0
	ZINC13435046	-7.8	385.161	-10.438	13	3	242.86	0.66775	226.72	-47.338	1529.749009844	0.33242	16.254	-31.401	0.58333	0.6201	0.94378 (max o	7	5086; 5056; 621; 335	-0.6399638	-0.4249982		14.0
	ZINC13435046	-7.8	384.153	-11.686	13	2	244.04	0.66936	229.55	-42.608	1529.749009844	0.33242	17.501	-35.154	0.58333	0.6199	0.94378 (max o	8	5087; 5057; 622; 335	-0.626228	-0.4667558		14.0
	ZINC06262879	-7.8	359.421	-0.202	6	2	268.38	0.2653	103.27	-4.56	1529.749009844	0.30685	6.0174	-0.65831	0.5	0.50195	0.92702 (max o	3	6070; 4955; 4895	-0.0564761	0.72272724		14.0
	ZINC05397027	-7.8	377.467	-0.2881	6	1	302.65	0.21256	68.85	-1.6254	1529.749009844	0.28493	6.1035	-1.0111	0.5	0.43149	0.90236 (max o	7	6742; 5777; 4990; 59	-0.4671149	-0.3292055		14.0
	ZINC05437546	-7.8	415.852	-0.8897	7	0	296.54	0.23774	89.98	1.5028	1529.749009844	0.2751	6.7051	-3.2341	0.55172	0.46678	0.9427 (max of	8	5778; 5393; 5392; 51	-0.515961	-0.2858319		14.0
	ZINC20464970	-7.8	403.448	-2.1588	8	5	312.29	0.29876	98.5	3.0936	1529.749009844	0.28493	7.9742	-7.5766	0.57143	0.59688	0.91204 (max o	6	5929; 1250; 1251; 22	-0.2943564	-0.0321756		14.0
	ZINC12603865	-7.8	399.441	-2.4213	9	6	312.59	0.36549	124.36	-0.0675	1529.749009844	0.28493	8.2367	-8.4979	0.64286	0.60067	0.9399 (max of	6	5024; 1462; 2927; 32	0.31303793	-0.4619012		14.0
	ZINC12603864	-7.8	381.451	-2.5221	9	6	306.24	0.37307	124.36	1.2725	1529.749009844	0.29548	8.3375	-8.5356	0.66667	0.5975	0.94302 (max o	5	5828; 5016; 4657; 29	0.3345633	-0.4596412		14.0
	ZINC04074027	-7.8	389.430	-5.1116	9	3	301.35	0.35898	144.66	-0.10036	1529.749009844	0.28493	10.927	-17.94	0.67857	0.59494	0.90236 (max o	3	3199; 3310; 4517	0.37934715	0.11090058		14.0
	ZINC02129888	-7.8	390.415	-4.5344	9	2	295.37	0.37313	157.31	5.2615	1529.749009844	0.28493	10.35	-15.914	0.60714	0.53124	0.96211 (max o	7	6423; 4584; 4519; 32	0.35839087	0.28579417		14.0
	ZINC06262872	-7.8	389.494	-0.5094	7	2	307.11	0.2699	117.18	5.2815	1529.749009844	0.28493	6.3248	-1.7878	0.60714	0.52286	0.97037 (max o	8	6423; 5366; 3200; 36	0.34066966	0.27810356		14.0
	ZINC04074027	-7.8	390.438	-2.9121	9	3	300.56	0.3573	146.28	2.1725	1529.749009844	0.28493	8.7275	-10.22	0.67857	0.55348	0.90236	1	4518	0.39504525	0.14443463		14.0
	ZINC04074066	-7.8	389.494	-0.5094	7	2	307.11	0.2699	117.18	5.2815	1529.749009844	0.28493	6.3248	-1.7878	0.60714	0.52104	0.97037 (max o	9	6423; 6121; 5490; 53	0.32225937	0.29035178		14.0
	ZINC08299774	-7.8	363.440	-3.6471	9	3	291.83	0.37159	110.42	2.3539	1529.749009844	0.30685	9.4625	-11.886	0.65385	0.5479	0.96721 (max o	5	6175; 6174; 5816; 58	0.13351631	-0.2275005		14.0

Structure	Molecule Name	affinity	Total Molweight	cLogP	H-Acceptors	H-Donors	Total Surface Area	Relative PSA	Polar Surface Area	Druglikeness	Kd (nM) @ 20°C	LE from K...	LLE from Kd ...	LELP from Kd (n...	Shape Index	Molecular...	Neighbor Si...	Neig...	Neighbor	Neighbor ...	Neighbor ...	Ring Systems	frequency
	ZINC20463972	-7.8	390.462	-2.1371	9	4	310.19	0.3345	111.29	-0.99464	1529.749009844	0.28493	7.9525	-7.5005	0.57143	0.52524	0.92774 (max o	8	6809; 6810; 6565; 59	-0.6897120	-0.2811187		14.0
	ZINC05433802	-7.8	362.430	-6.1931	10	3	253.16	0.4432	151.91	4.9264	1529.749009844	0.33242	12.008	-18.631	0.5	0.53055	0.90837 (max o	4	6741; 6460; 5900; 33	-0.0529361	0.39006773		13.0
	ZINC13424694	-7.8	420.413	0.4007	9	6	299.15	0.37319	156.91	-4.8731	1529.749009844	0.26593	5.4147	1.5068	0.5	0.4209	0.95579 (max o	5	6144; 5085; 3365; 41	0.76114666	-0.2564188		13.0
	ZINC04064073	-7.8	407.533	-1.0329	7	2	313.6	0.32009	142.48	5.0647	1529.749009844	0.28493	6.8483	-3.6251	0.64286	0.5279	0.96519 (max o	8	6946; 6423; 4663; 46	0.30539128	0.33465025		12.0
	ZINC12877838	-7.8	389.494	-0.5094	7	2	307.11	0.2699	117.18	6.1636	1529.749009844	0.28493	6.3248	-1.7878	0.60714	0.51837	0.97288 (max o	8	6121; 5490; 5448; 53	0.26755223	0.34050992		12.0
	ZINC04084422	-7.8	390.415	-2.3692	9	2	297.07	0.34642	127.87	4.485	1529.749009844	0.28493	8.1846	-8.3151	0.60714	0.47922	0.96519 (max o	6	5753; 5369; 1693; 23	0.51032954	0.30511624		12.0
	ZINC72320134	-7.8	350.322	-0.2569	8	3	236.42	0.41629	130.36	-2.1313	1529.749009844	0.31912	6.0723	-0.80503	0.48	0.40379	0.97118 (max o	10	6058; 6057; 5279; 52	0.76868576	-0.1113765		12.0
	ZINC72320135	-7.8	350.322	-0.2569	8	3	236.42	0.41629	130.36	-2.1313	1529.749009844	0.31912	6.0723	-0.80503	0.48	0.40379	0.97118 (max o	10	6679; 6057; 5279; 52	0.758741	-0.1312826		12.0
	ZINC72320137	-7.8	350.322	-0.2569	8	3	236.42	0.41629	130.36	-2.1313	1529.749009844	0.31912	6.0723	-0.80503	0.48	0.40379	0.97668 (max o	10	6679; 6058; 5279; 52	0.78817964	-0.1095923		12.0
	ZINC12603771	-7.8	368.409	-1.1357	8	4	266.8	0.37759	133.34	-4.9319	1529.749009844	0.31912	6.9511	-3.5588	0.64	0.54807	0.93686 (max o	6	6750; 6187; 1800; 21	0.24514726	-0.3357173		12.0
	ZINC08624591	-7.8	402.466	-0.9115	9	4	291.27	0.3802	142.57	-4.9768	1529.749009844	0.29548	6.7269	-3.0848	0.66667	0.57581	0.94002 (max o	4	6750; 5064; 3332; 33	0.23394786	-0.3175650		12.0
	ZINC06469287	-7.8	375.349	-4.4746	10	5	249.52	0.49223	168.97	-1.6392	1529.749009844	0.30685	10.29	-14.583	0.46154	0.36996	0.94326 (max o	4	4616; 4143; 4144; 41	-0.2357574	0.4806776		12.0
	ZINC08681472	-7.8	390.383	-1.9707	10	5	264.25	0.44859	155.14	-3.9213	1529.749009844	0.29548	7.7861	-6.6695	0.48148	0.36873	0.94605 (max o	5	5248; 4616; 3517; 41	-0.2044766	0.43290642		12.0
	ZINC08681473	-7.8	390.383	-1.9707	10	5	264.25	0.44859	155.14	-3.9213	1529.749009844	0.29548	7.7861	-6.6695	0.48148	0.36873	0.95201 (max o	4	5248; 3517; 4586; 46	-0.2232143	0.44608256		12.0
	ZINC12603532	-7.8	376.404	-0.8944	8	4	261.6	0.38509	133.34	2.6637	1529.749009844	0.31912	6.7098	-2.8027	0.64	0.57539	0.9302 (max of	3	6481; 4608; 4612	-0.3892431	-0.1868092		12.0
	ZINC08643330	-7.8	376.404	-0.8944	8	4	261.6	0.38509	133.34	2.6637	1529.749009844	0.31912	6.7098	-2.8027	0.64	0.57539	0.93007 (max o	2	6511; 4653	-0.3644449	-0.2135409		12.0
	ZINC04228284	-7.8	383.349	-4.4475	12	8	254.94	0.57347	198.4	-4.7796	1529.749009844	0.30685	10.263	-14.494	0.46154	0.46983	0.95559 (max o	7	7010; 6958; 6720; 65	0.21226753	0.72879404		12.0
	ZINC12504462	-7.8	356.323	-4.03	11	7	235.73	0.55869	178.53	-13.883	1529.749009844	0.33242	9.8454	-12.123	0.5	0.46793	0.86078 (max o	2	7777; 6370	-0.1751008	-0.9614284		12.0
	ZINC31162155	-7.8	394.415	-1.3673	10	6	278.15	0.42639	158.3	-17.104	1529.749009844	0.29548	7.1827	-4.6274	0.55556	0.49914		0		-0.8290825	0.4918492		12.0
	ZINC04237223	-7.8	423.511	-1.5786	7	1	333.74	0.23398	85.19	3.8448	1529.749009844	0.25735	7.394	-6.1339	0.54839	0.49638	0.95983 (max o	3	2805; 3681; 4542	-0.5491922	-0.3728798		10.0
	ZINC04237219	-7.8	410.560	-1.8671	6	2	343.56	0.19898	49.5	4.76	1529.749009844	0.26593	7.6825	-7.0209	0.53333	0.46735	0.8872 (max of	5	5776; 4988; 591; 454	-0.5091188	-0.364037		10.0
	ZINC04085256	-7.8	363.413	-1.4073	7	2	249.83	0.39627	135.07	5.1687	1529.749009844	0.31912	7.2227	-4.4099	0.44	0.27227	0.98671 (max o	9	6409; 1219; 1692; 45	0.29780355	-0.6435225		10.0
	ZINC04085258	-7.8	363.413	-1.4073	7	2	249.83	0.39627	135.07	5.1687	1529.749009844	0.31912	7.2227	-4.4099	0.44	0.27227	0.98671 (max o	9	5356; 1219; 1402; 24	0.32370576	-0.6763385		10.0
	ZINC03842002	-7.8	358.401	0.0214	9	1	265.31	0.33105	94.4	1.0467	1529.749009844	0.30685	5.794	0.069742	0.57692	0.34101	0.91522 (max o	5	5002; 2076; 2426; 27	-0.2329007	-0.3785148		10.0
	ZINC08299765	-7.8	391.494	-3.183	9	2	308	0.3289	107.78	2.3227	1529.749009844	0.28493	8.9984	-11.171	0.64286	0.5187	0.98451 (max o	11	5439; 5014; 5012; 24	0.17775784	0.28004396		10.0
	ZINC20465339	-7.8	368.427	-2.5669	7	4	283.98	0.28819	86.47	2.5468	1529.749009844	0.30685	8.3823	-8.3654	0.57692	0.52464	0.94605 (max o	6	7730; 6826; 5927; 47	-0.3333419	-0.5823617		10.0
	ZINC20465370	-7.8	404.481	-2.6869	8	4	294.81	0.33031	111.92	2.8796	1529.749009844	0.29548	8.5023	-9.0933	0.55556	0.53447	0.93686 (max o	5	7730; 7050; 6265; 55	-0.3515812	-0.5415973		10.0
	ZINC72319869	-7.8	406.470	-1.0554	9	7	286.5	0.38988	160.07	-1.8323	1529.749009844	0.28493	6.8708	-3.7041	0.5	0.56656	0.97288 (max o	7	5680; 5628; 5276; 52	-0.3376841	0.2549771		10.0
	ZINC72319866	-7.8	406.470	-1.0554	9	7	286.5	0.38988	160.07	-1.8323	1529.749009844	0.28493	6.8708	-3.7041	0.5	0.57544	0.97288 (max o	3	5680; 5276; 4877	-0.3576981	0.2672835		10.0
	ZINC04277708	-7.8	389.518	-0.1754	7	2	311.34	0.24176	74.16	2.7635	1529.749009844	0.28493	5.9908	-0.61559	0.57143	0.47577	0.91157 (max o	2	6149; 4124	-0.8182065	-0.3089551		10.0
	ZINC31158776	-7.8	386.439	-0.2961	8	5	283.18	0.34798	136.68	-4.6035	1529.749009844	0.29548	6.1115	-1.0021	0.48148	0.53586	0.99202 (max o	5	5969; 5970; 5603; 52	-0.0628268	0.91137993		10.0
	ZINC35465980	-7.8	441.451	-2.6414	10	5	318.58	0.39507	176.81	-2.3234	1529.749009844	0.25735	8.4568	-10.264	0.48387	0.44136	0.97642 (max o	3	6339; 5242; 4840	0.20008804	-0.7265847		10.0
	ZINC35465978	-7.8	441.451	-2.6414	10	5	318.58	0.39507	176.81	-2.3234	1529.749009844	0.25735	8.4568	-10.264	0.48387	0.44136	0.97642 (max o	3	6339; 5242; 4841	0.18207581	-0.7142975		10.0
	ZINC04147658	-7.8	404.426	-0.3553	11	1	299.07	0.36055	112.86	2.6608	1529.749009844	0.2751	6.1707	-1.2915	0.58621	0.40159	0.9428 (max of	6	1702; 2047; 2425; 27	-0.2120426	-0.3501415		9.0
	ZINC03838675	-7.8	384.526	-3.3336	7	3	328.97	0.26899	68.69	0.27097	1529.749009844	0.28493	9.149	-11.7	0.64286	0.51667	0.97037 (max o	8	5006; 2746; 2858; 32	-0.0998365	0.01431477		9.0
	ZINC04260809	-7.8	390.390	-0.4295	8	1	278.17	0.30557	109.85	3.0481	1529.749009844	0.28493	6.2449	-1.5074	0.53571	0.55046	0.97773 (max o	6	1166; 1418; 3231; 32	0.10868153	-0.5855287		8.0
	ZINC04260807	-7.8	380.375	-1.9306	8	1	260.73	0.39309	135.15	4.9715	1529.749009844	0.30685	7.746	-6.2917	0.57692	0.53162	0.9615 (max of	4	6434; 3232; 3665; 45	0.09611837	-0.600848		8.0
	ZINC20412620	-7.8	423.572	-1.5432	7	3	331.85	0.24222	83.67	-1.1127	1529.749009844	0.26593	7.3586	-5.803	0.6	0.51463	0.95318 (max o	6	4775; 4773; 4704; 15	-0.0304183	0.09872293		8.0
	ZINC20412623	-7.8	423.572	-1.5432	7	3	331.85	0.24222	83.67	-1.1127	1529.749009844	0.26593	7.3586	-5.803	0.6	0.51463	0.95318 (max o	7	4775; 4773; 1537; 47	-0.0304815	0.05811124		8.0
	ZINC22457071	-7.8	379.519	-1.4507	6	3	294.33	0.23912	74.44	2.1596	1529.749009844	0.29548	7.2661	-4.9096	0.55556	0.46431	0.95123 (max o	6	5176; 4775; 4773; 15	-0.0494269	0.07233921		8.0
	ZINC22457322	-7.8	423.572	-1.5432	7	3	331.85	0.24222	83.67	-1.1127	1529.749009844	0.26593	7.3586	-5.803	0.6	0.51463	0.95318 (max o	7	5176; 4776; 4694; 47	-0.0127761	0.04894314		8.0
	ZINC22457317	-7.8	423.572	-1.5432	7	3	331.85	0.24222	83.67	-1.1127	1529.749009844	0.26593	7.3586	-5.803	0.6	0.51463	0.95318 (max o	7	5176; 4775; 4694; 47	-0.0132992	0.07073091		8.0
	ZINC20412620	-7.8	423.572	-1.5432	7	3	331.85	0.24222	83.67	-1.2522	1529.749009844	0.26593	7.3586	-5.803	0.6	0.54484	0.95318 (max o	6	4776; 4774; 4705; 15	-0.0425807	0.13635898		8.0

Structure	Molecule Name	affinity	Total Molweight	cLogP	H-Acceptors	H-Donors	Total Surface Area	Relative PSA	Polar Surface Area	Druglikeness	Kd (nM) @ 20°C	LE from K...	LLE from Kd ...	LELP from Kd (n...	Shape Index	Molecular...	Neighbor Si...	Neig...	Neighbor	Neighbor ...	Neighbor ...	Ring Systems	frequency
	ZINC20412623	-7.8	423.572	-1.5432	7	3	331.85	0.24222	83.67	-1.2522	1529.749009844	0.26593	7.3586	-5.803	0.6	0.54484	0.95318 (max o	7	4776; 4774; 1538; 42	-0.0420823	0.1162697		8.0
	ZINC22457071	-7.8	379.519	-1.4507	6	3	294.33	0.23912	74.44	2.0856	1529.749009844	0.29548	7.2661	-4.9096	0.55556	0.49449	0.94605 (max o	6	5177; 1538; 4287; 47	-0.0631947	0.10745051		8.0
	ZINC22457317	-7.8	423.572	-1.5432	7	3	331.85	0.24222	83.67	-1.2522	1529.749009844	0.26593	7.3586	-5.803	0.6	0.54484	0.95318 (max o	7	5177; 4776; 4695; 47	-0.0185306	0.11650425		8.0
	ZINC22457322	-7.8	423.572	-1.5432	7	3	331.85	0.24222	83.67	-1.2522	1529.749009844	0.26593	7.3586	-5.803	0.6	0.54484	0.95318 (max o	6	5177; 4695; 4703; 47	-0.0140035	0.09540678		8.0
	ZINC31154891	-7.8	386.439	-0.1928	8	5	279.59	0.35244	136.68	-3.8197	1529.749009844	0.29548	6.0082	-0.65249	0.51852	0.45355	0.97407 (max o	6	5641; 5594; 5209; 30	0.64318043	-0.1377767		8.0
	ZINC35455022	-7.8	396.431	-1.2041	10	6	285.68	0.41515	158.3	-17.323	1529.749009844	0.29548	7.0195	-4.075	0.62963	0.54055	0.9578 (max of	6	6325; 5630; 5511; 85	-0.4401952	0.6713412		8.0
	ZINC35454849	-7.8	422.469	-0.5759	10	6	308.96	0.38387	158.3	-18.912	1529.749009844	0.2751	6.3913	-2.0934	0.58621	0.53573	0.97415 (max o	5	5630; 5226; 3492; 44	-0.4302665	0.7117904		8.0
	ZINC04899824	-7.8	376.388	-2.1675	9	2	287.57	0.35786	127.87	-6.9985	1529.749009844	0.29548	7.9829	-7.3355	0.62963	0.57977	0.9279 (max of	3	6393; 3669; 4104	0.00429447	-0.4682042		8.0
	ZINC31155176	-7.8	376.357	-2.3979	10	7	261.49	0.46541	169.3	-7.5627	1529.749009844	0.30685	8.2133	-7.8147	0.53846	0.55924	0.9615 (max of	6	6301; 5982; 3882; 39	0.15952669	0.72070897		8.0
	ZINC31155167	-7.8	376.357	-2.3979	10	7	261.49	0.46541	169.3	-7.5627	1529.749009844	0.30685	8.2133	-7.8147	0.53846	0.55678	0.9615 (max of	6	6301; 5982; 4793; 47	0.11707599	0.72649395		8.0
	ZINC31155173	-7.8	376.357	-2.3979	10	7	261.49	0.46541	169.3	-7.5627	1529.749009844	0.30685	8.2133	-7.8147	0.53846	0.54963	0.9615 (max of	5	6301; 5982; 4793; 38	0.12571694	0.70753664		8.0
	ZINC31158149	-7.8	352.425	1.0294	6	4	262.95	0.2869	107.22	4.8399	1529.749009844	0.31912	4.786	3.2257	0.56	0.44603	0.97118 (max o	6	6880; 5230; 4830; 47	-0.718401	-0.1746928		8.0
	ZINC31158159	-7.8	352.425	1.0294	6	4	262.95	0.2869	107.22	4.8399	1529.749009844	0.31912	4.786	3.2257	0.56	0.45151	0.97118 (max o	5	6880; 5999; 3897; 38	-0.6855417	-0.1825926		8.0
	ZINC35455849	-7.8	352.425	1.0294	6	4	262.95	0.2869	107.22	4.8399	1529.749009844	0.31912	4.786	3.2257	0.56	0.44603	0.97118 (max o	5	6880; 5999; 5230; 38	-0.7113497	-0.2087932		8.0
	ZINC70666365	-7.8	359.357	-0.8644	8	2	260.62	0.37929	120.55	0.018054	1529.749009844	0.30685	6.6798	-2.817	0.42308	0.30714	0.93033 (max o	4	7274; 7110; 6053; 52	0.46956438	0.36268958		8.0
	ZINC12660219	-7.8	436.507	-1.659	9	2	329.82	0.36505	153.17	2.1105	1529.749009844	0.26593	7.4744	-6.2384	0.6	0.54661	0.9707 (max of	3	5488; 5068; 5069	-0.7142778	0.6325577		8.0
	ZINC14486269	-7.8	386.356	-4.8719	11	6	266.96	0.53383	210.24	-4.0803	1529.749009844	0.29548	10.687	-16.488	0.51852	0.42717	0.94905	1	4682	-0.3654140	0.87353605		8.0
	ZINC05438633	-7.8	419.437	-2.8751	10	2	301.71	0.45348	163.94	2.7251	1529.749009844	0.2751	8.6905	-10.451	0.62069	0.43096	0.96682 (max o	8	6459; 5425; 4995; 59	-0.6751971	-0.5625946		7.0
	ZINC05433647	-7.8	361.353	-0.1936	9	1	265.98	0.37804	104.94	-1.3902	1529.749009844	0.30685	6.009	-0.63093	0.61538	0.34481	0.87324 (max o	2	6458; 5425	-0.6466404	-0.5969773		7.0
	ZINC15676114	-7.8	370.408	-2.4129	8	2	271.82	0.27404	110.68	5.6094	1529.749009844	0.29548	8.2283	-8.166	0.51852	0.57421	0.95461 (max o	5	498; 2972; 4216; 468	0.20783356	-0.1949254		7.0
	ZINC15676112	-7.8	386.407	-3.4142	9	2	281.82	0.2998	119.91	5.1841	1529.749009844	0.28493	9.2296	-11.983	0.53571	0.56455	0.95461 (max o	5	5059; 4688; 300; 468	0.24260667	-0.1845673		7.0
	ZINC15676112	-7.8	385.399	-3.2253	9	1	292.79	0.32604	118.66	5.733	1529.749009844	0.28493	9.0407	-11.32	0.53571	0.56455	0.95461 (max o	6	5060; 301; 1837; 297	0.2687543	-0.2031953		7.0
	ZINC15676114	-7.8	369.400	-2.224	8	1	282.79	0.3022	109.43	6.1638	1529.749009844	0.29548	8.0394	-7.5267	0.51852	0.57421	0.95461 (max o	6	499; 2973; 4217; 421	0.2221366	-0.2259805		7.0
	ZINC04028709	-7.8	385.209	1.2442	6	4	234.65	0.30854	99.38	-6.4693	1529.749009844	0.34687	4.5712	3.5869	0.56522	0.36597	0.93686 (max o	7	1677; 2777; 2949; 32	0.25339317	0.8984277		7.0
	ZINC12889439	-7.8	401.442	-1.2844	9	2	299.13	0.32247	121.88	8.3132	1529.749009844	0.2751	7.0998	-4.6688	0.44828	0.52214	0.97649 (max o	4	3211; 3212; 4667; 45	-0.7230093	0.05505799		7.0
	ZINC04090650	-7.8	387.415	-1.7388	9	2	285.37	0.33802	121.88	7.361	1529.749009844	0.28493	7.5542	-6.1026	0.46429	0.51653	0.95401 (max o	4	5169; 4666; 3211; 32	-0.7621515	0.05183808		7.0
	ZINC20758642	-7.8	401.442	-1.2844	9	2	299.13	0.32247	121.88	8.3132	1529.749009844	0.2751	7.0998	-4.6688	0.44828	0.52206	0.97649 (max o	3	5169; 3211; 4667	-0.7410142	0.01168758		7.0
	ZINC12889600	-7.8	401.442	-1.2844	9	2	299.13	0.32247	121.88	8.3132	1529.749009844	0.2751	7.0998	-4.6688	0.44828	0.52206	0.97649 (max o	3	5169; 4763; 4666	-0.7219249	0.01883308		7.0
	ZINC31157281	-7.8	394.418	-0.2395	8	3	277.8	0.35428	130.36	-10.347	1529.749009844	0.28493	6.0549	-0.84056	0.46429	0.41333	0.95983 (max o	4	3453; 3454; 3494; 43	-0.4938661	0.5067373		7.0
	ZINC30879339	-7.8	361.460	-0.8804	6	3	308.05	0.24408	72.23	2.87	1529.749009844	0.30685	6.6958	-2.8692	0.69231	0.59213	0.94326 (max o	2	7641; 6289	-0.3359341	0.42984223		7.0
	ZINC14442634	-7.8	434.396	-1.4258	10	8	285	0.44856	188.14	-2.6646	1529.749009844	0.25735	7.2412	-5.5402	0.35484	0.33408	0.96018 (max o	7	6314; 5513; 5512; 10	-0.4983496	0.57126886		6.0
	ZINC12529915	-7.8	390.395	-3.1607	10	2	310.12	0.40455	131.05	1.9251	1529.749009844	0.28493	8.9761	-11.093	0.67857	0.44085	0.95174 (max o	5	2149; 2150; 2918; 33	0.63726074	-0.3101737		6.0
	ZINC20465125	-7.8	402.872	-2.0123	7	4	299.4	0.27335	86.47	2.3125	1529.749009844	0.29548	7.8277	-6.8102	0.55556	0.52637	0.92064 (max o	8	7044; 6819; 5935; 59	-0.3496544	0.02225244		6.0
	ZINC35456947	-7.8	406.470	-1.1587	9	7	290.09	0.38505	160.07	-3.1565	1529.749009844	0.28493	6.9741	-4.0666	0.5	0.47465	0.97288 (max o	3	3495; 3929; 4411	0.9029926	0.22470646		6.0
	ZINC13660037	-7.8	390.471	-0.232	8	6	282.24	0.34935	139.84	-2.1613	1529.749009844	0.29548	6.0474	-0.78516	0.51852	0.47044	0.97407 (max o	5	5509; 5508; 5103; 25	-0.3792583	0.23542881		6.0
	ZINC13333978	-7.8	388.455	-0.0883	8	5	281.14	0.3505	136.68	-3.7924	1529.749009844	0.29548	5.9037	-0.29883	0.51852	0.45195	0.96617 (max o	6	5628; 5497; 5224; 33	-0.3338616	0.17503068		6.0
	ZINC13333975	-7.8	388.455	-0.0883	8	5	281.14	0.3505	136.68	-3.7924	1529.749009844	0.29548	5.9037	-0.29883	0.51852	0.45612	0.96617 (max o	7	5628; 5497; 5225; 52	-0.3017887	0.20472316		6.0
	ZINC20463811	-7.8	426.535	-0.1036	7	2	314.53	0.18421	71.01	4.6027	1529.749009844	0.25735	5.919	-0.40256	0.54839	0.45421	0.97642 (max o	7	6557; 6559; 6255; 58	0.3590703	-0.0518033		6.0
	ZINC12484182	-7.8	412.508	-0.3793	7	3	298.62	0.20441	82.01	4.5421	1529.749009844	0.26593	6.1947	-1.4263	0.56667	0.44581	0.95549 (max o	7	7213; 6557; 6559; 62	0.393133	-0.0634119		6.0
	ZINC31167827	-7.8	434.524	0.0313	9	6	308.42	0.36197	156.91	-5.985	1529.749009844	0.26593	5.7841	0.1177	0.46667	0.49711	0.97304 (max o	7	6618; 6336; 3073; 35	-0.1787189	0.8079109		6.0
	ZINC12663497	-7.8	398.398	-1.3043	10	1	278.41	0.29108	99.63	-0.78034	1529.749009844	0.2751	7.1197	-4.7411	0.48276	0.36192	0.96385 (max o	3	5355; 5073; 4062	0.5237843	0.68244225		6.0
	ZINC14616883	-7.8	422.469	-0.5759	10	6	308.96	0.38387	158.3	-18.912	1529.749009844	0.2751	6.3913	-2.0934	0.58621	0.53573	0.97415 (max o	5	5881; 5514; 5511; 42	-0.3890755	0.6687935		6.0
	ZINC14438554	-7.8	396.431	-1.2041	10	6	285.68	0.41515	158.3	-17.323	1529.749009844	0.29548	7.0195	-4.075	0.62963	0.54169	0.9578 (max of	8	6325; 6238; 5880; 58	-0.4188686	0.6780735		6.0

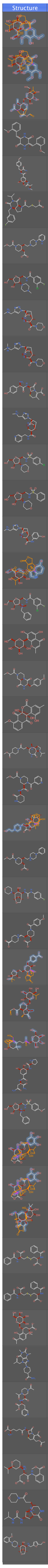

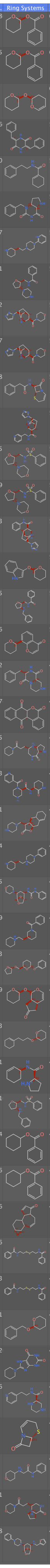

Structure	Molecule Name	affinity	Total Molweight	cLogP	H-Acceptors	H-Donors	Total Surface Area	Relative PSA	Polar Surface Area	Druglikeness	Kd (nM) @ 20°C	LE from K...	LLE from Kd ...	LELP from Kd (n...	Shape Index	Molecular...	Neighbor Si...	Neig...	Neighbor	Neighbor ...	Neighbor ...	Ring Systems	frequency
	ZINC13540671	-7.8	360.314	-1.1781	10	5	250.04	0.47408	155.14	-4.135	1529.749009844	0.31912	6.9935	-3.6917	0.48	0.39614	0.91007	1	3645	-0.8371887	-0.0941428		6.0
	ZINC31159265	-7.8	360.314	-1.1781	10	5	250.04	0.47408	155.14	-4.135	1529.749009844	0.31912	6.9935	-3.6917	0.52	0.41908	0.95318 (max c	6	6788; 6001; 5634; 56	-0.6932061	-0.1014475		6.0
	ZINC31160597	-7.8	403.359	-4.0657	11	4	283.96	0.46753	175.04	-7.5769	1529.749009844	0.28493	9.8811	-14.269	0.46429	0.50044	0.9678 (max of	3	7237; 5973; 5972	-0.4915708	0.8638945		6.0
	ZINC05023646	-7.8	350.373	2.1326	6	2	271.88	0.25379	76.66	2.9999	1529.749009844	0.30685	3.6828	6.9501	0.53846	0.33757	0.83798	1	7326	0.19299358	-0.7695756		5.0
	ZINC03838819	-7.8	435.499	-3.0267	10	6	326.4	0.37148	167.59	5.3567	1529.749009844	0.25735	8.8421	-11.761	0.58065	0.53802	0.92326 (max c	2	4920; 2006	-0.4944785	-0.2326060		5.0
	ZINC04260489	-7.8	436.483	-0.9892	8	0	310.35	0.30475	126.36	3.2827	1529.749009844	0.26593	6.8046	-3.7197	0.5	0.55948	0.96832 (max c	4	6142; 2439; 2440; 36	-0.3736709	0.8336999		5.0
	ZINC20463375	-7.8	404.509	-2.4178	8	1	306.6	0.19899	90.29	8.255	1529.749009844	0.2751	8.2332	-8.7887	0.58621	0.58026	0.93985 (max c	10	640; 804; 2580; 2582	-0.1531393	-0.2421007		5.0
	ZINC20465125	-7.8	401.865	0.0974	7	3	285.36	0.23759	85.27	6.3452	1529.749009844	0.29548	5.718	0.32963	0.55556	0.52637	0.92064 (max c	7	7044; 5934; 5932; 55	-0.3770360	0.04544231		5.0
	ZINC08299247	-7.8	350.465	-4.6411	8	2	276.97	0.29148	81.3	0.32375	1529.749009844	0.31912	10.456	-14.543	0.64	0.52506	0.9654 (max of	7	5446; 5440; 3275; 37	0.23133035	0.30013892		5.0
	ZINC03838655	-7.8	364.492	-4.5162	8	2	305.83	0.29618	69.13	0.25249	1529.749009844	0.30685	10.332	-14.718	0.61538	0.53856	0.96721 (max c	3	5719; 4596; 2866	0.22953433	0.32309577		5.0
	ZINC35024386	-7.8	362.385	-4.4497	8	3	247.6	0.46151	161.09	7.0306	1529.749009844	0.31912	10.265	-13.944	0.56	0.43729	0.93361 (max c	6	5218; 2276; 2575; 34	-0.4696315	-0.0492368		5.0
	ZINC08254213	-7.8	371.487	-4.4594	8	3	298.87	0.29983	93.75	0.35716	1529.749009844	0.29548	10.275	-15.092	0.66667	0.50523	0.96885 (max c	12	5447; 1759; 2860; 32	-0.1028972	0.048273		5.0
	ZINC20464855	-7.8	406.886	-0.4262	8	3	277.28	0.32382	116.71	4.1533	1529.749009844	0.30685	6.2416	-1.389	0.53846	0.53208	0.97533 (max c	6	5551; 3843; 3846; 38	-0.4454112	-0.1035985		5.0
	ZINC20464810	-7.8	372.441	-1.0322	8	3	261.86	0.34289	116.71	-4.0749	1529.749009844	0.31912	6.8476	-3.2345	0.52	0.52628	0.93686 (max c	6	5563; 5564; 5551; 47	-0.4025209	-0.0868049		5.0
	ZINC08299295	-7.8	372.471	-3.5136	8	4	292.7	0.31343	101.09	0.24876	1529.749009844	0.29548	9.329	-11.891	0.62963	0.47727	0.96885 (max c	5	5447; 5441; 3281; 41	-0.1210717	-0.0433513		5.0
	ZINC14860993	-7.8	374.186	0.0892	7	5	224.22	0.38529	115.17	-5.5938	1529.749009844	0.36264	5.7262	0.24598	0.5	0.24725	0.90912 (max c	4	6154; 6155; 5406; 49	-0.4281289	0.605758		5.0
	ZINC30879657	-7.8	423.891	0.0708	6	4	321.08	0.24383	83.23	0.69731	1529.749009844	0.2751	5.7446	0.25736	0.55172	0.53938	0.94251 (max c	3	5962; 1542; 4320	0.40282238	-0.2389405		5.0
	ZINC31165116	-7.8	354.310	-1.0269	9	6	235.34	0.47438	156.91	-8.4272	1529.749009844	0.31912	6.8423	-3.2179	0.4	0.41325	0.87932	1	5760	-0.7457625	0.41943726		5.0
	ZINC04260084	-7.8	361.397	-0.3709	8	1	269.89	0.28782	88.18	7.0744	1529.749009844	0.30685	6.1863	-1.2087	0.65385	0.5174	0.90377 (max c	2	349; 447	-0.3243955	-0.1965052		4.0
	ZINC31162102	-7.8	370.312	1.0015	8	3	243.66	0.40392	130.36	0.8612	1529.749009844	0.29548	4.8139	3.3894	0.44444	0.24731	0.95493 (max c	3	1027; 1900; 2265	-0.2789801	-0.8255867		4.0
	ZINC35442547	-7.8	409.549	-1.5035	8	2	328.03	0.25995	83.39	3.9962	1529.749009844	0.2751	7.3189	-5.4652	0.58621	0.5614	0.93686 (max c	7	5997; 5584; 1305; 34	-0.1512477	0.52391845		4.0
	ZINC05414683	-7.8	403.438	-1.3156	10	2	303.12	0.34029	120.94	4.9815	1529.749009844	0.2751	7.131	-4.7822	0.44828	0.52776	0.97415 (max c	6	5805; 5806; 1440; 17	0.07961941	0.23426783		4.0
	ZINC22457071	-7.8	378.511	0.659	6	2	280.29	0.20101	73.24	6.0263	1529.749009844	0.29548	5.1564	2.2303	0.55556	0.49191	0.94605 (max c	5	5175; 4772; 1536; 42	-0.0980529	0.12906861		4.0
	ZINC20463221	-7.8	403.521	-1.2774	7	1	332.85	0.22548	77.35	4.1982	1529.749009844	0.2751	7.0928	-4.6433	0.58621	0.59108	0.96428 (max c	4	5848; 4724; 2145; 33	0.6614097	0.40843394		4.0
	ZINC12484133	-7.8	367.376	-0.2364	8	3	257.73	0.32053	92.29	0.65625	1529.749009844	0.30685	6.0518	-0.77042	0.57692	0.45731	0.93367 (max c	7	7498; 2146; 3388; 37	-0.6506047	-0.2713955		4.0
	ZINC20462836	-7.8	431.507	-2.9284	8	1	346.57	0.28514	114.24	4.9255	1529.749009844	0.25735	8.7438	-11.379	0.58065	0.60629	0.9396 (max of	5	2579; 2980; 4291; 47	0.03133422	-0.1898029		4.0
	ZINC31169464	-7.8	431.369	-4.62	12	6	288.83	0.50521	198.43	0.77107	1529.749009844	0.26593	10.435	-17.373	0.56667	0.44315	0.9707 (max of	7	2662; 3076; 3077; 34	0.27575472	-0.6454903		4.0
	ZINC67912736	-7.8	388.368	-1.827	10	4	263.15	0.45024	151.98	-4.331	1529.749009844	0.29548	7.6424	-6.1831	0.48148	0.36725	0.93058 (max c	2	1336; 4436	-0.7928473	-0.3386969		4.0
	ZINC38289022	-7.8	374.472	0.91	7	5	276.92	0.30875	119.61	-4.4688	1529.749009844	0.30685	4.9054	2.9657	0.53846	0.55128	0.97791 (max c	8	6620; 6299; 5977; 52	0.7569936	0.32500803		4.0
	ZINC04221602	-7.8	384.411	-4.291	11	7	273.91	0.50951	201.45	4.1291	1529.749009844	0.29548	10.106	-14.522	0.51852	0.47437	0.92418 (max c	7	5771; 5387; 5386; 50	-0.5502360	-0.1551211		4.0
	ZINC30879700	-7.8	437.535	-1.0194	8	4	332.4	0.31236	117.91	2.6435	1529.749009844	0.26593	6.8348	-3.8333	0.56667	0.5508	0.9397 (max of	4	5592; 5184; 3880; 43	0.46641326	-0.3052541		4.0
	ZINC35465645	-7.8	358.298	-1.0155	10	6	241.05	0.51724	173.98	-0.59921	1529.749009844	0.31912	6.8309	-3.1822	0.52	0.41264	0.98188 (max c	3	5643; 3935; 4838	-0.0681936	0.81073684		4.0
	ZINC35465639	-7.8	358.298	-1.0155	10	6	241.05	0.51724	173.98	-0.59921	1529.749009844	0.31912	6.8309	-3.1822	0.52	0.41264	0.97118 (max c	3	5643; 4839; 3935	-0.0471136	0.80552226		4.0
	ZINC05121258	-7.8	364.436	0.0565	6	5	239.12	0.32845	118.22	-2.8615	1529.749009844	0.30685	5.7589	0.18413	0.34615	0.30202	0.94008 (max c	3	4985; 4116; 4117	-0.8425228	0.22792546		4.0
	ZINC04995786	-7.8	446.503	-2.5422	8	2	329.5	0.4207	189.06	2.8383	1529.749009844	0.26593	8.3576	-9.5595	0.6	0.66601	1 (max of 3)	3	5793; 4233; 4562	0.11729821	-0.7283456		4.0
	ZINC04995783	-7.8	446.503	-2.5422	8	2	329.5	0.4207	189.06	2.8383	1529.749009844	0.26593	8.3576	-9.5595	0.6	0.65926	0.97533 (max c	3	5793; 4563; 4233	0.13147701	-0.6960773		4.0
	ZINC49601557	-7.8	445.396	-4.3457	12	6	310.43	0.47985	206.27	-2.4636	1529.749009844	0.25735	10.161	-16.886	0.3871	0.49433	0.98268 (max c	3	7566; 5261; 4429	-0.4952687	-0.7682891		4.0
	ZINC00631635	-7.8	360.417	0.7506	9	1	267.72	0.30958	104.77	-1.5116	1529.749009844	0.30685	5.0648	2.4462	0.46154	0.45034	0.92032	1	6493	0.05584149	-0.8162812		4.0
	ZINC03842152	-7.8	376.412	-0.5835	9	1	288.4	0.31793	108.91	5.0128	1529.749009844	0.29548	6.3989	-1.9747	0.48148	0.5551	0.93686 (max c	7	6131; 5403; 5339; 49	-0.0328908	0.40045416		4.0
	ZINC03871994	-7.8	414.414	-5.2749	11	2	288.77	0.52201	208.91	5.0035	1529.749009844	0.28493	11.09	-18.513	0.60714	0.49964	0.94573 (max c	3	6203; 4945; 4054	0.44667783	0.5279196		4.0
	ZINC04043929	-7.8	392.450	-1.0059	8	2	301.56	0.30707	121.37	-1.6404	1529.749009844	0.28493	6.8213	-3.5304	0.5	0.46542	0.96519 (max c	3	5747; 5360; 4957	0.06963438	0.9100626		4.0
	ZINC15708241	-7.8	382.483	-2.657	9	4	303.63	0.34463	104.21	5.2912	1529.749009844	0.29548	8.4724	-8.9921	0.59259	0.52617	0.92442 (max c	5	7363; 7020; 6243; 58	-0.1793006	0.04596071		4.0
	ZINC20463814	-7.8	446.522	-1.4582	9	3	324.33	0.26254	104.38	5.4919	1529.749009844	0.24931	7.2736	-5.8489	0.5625	0.45148	0.93686 (max c	5	6255; 5852; 5135; 14	0.36172253	0.02285288		4.0

Structure	Molecule Name	affinity	Total Molweight	cLogP	H-Acceptors	H-Donors	Total Surface Area	Relative PSA	Polar Surface Area	Druglikeness	Kd (nM) @ 20°C	LE from K...	LLE from Kd ...	LELP from Kd (n...	Shape Index	Molecular...	Neighbor Si...	Neig...	Neighbor	Neighbor ...	Neighbor ...	Ring Systems	frequency
	ZINC20463848	-7.8	416.496	-1.3882	8	3	302.07	0.24878	95.15	5.4154	1529.749009844	0.26593	7.2036	-5.2201	0.6	0.4439	0.93101 (max o	7	7402; 7213; 6768; 65	0.3857515	0.04776678		4.0
	ZINC35271220	-7.8	377.411	-0.7314	7	3	282.84	0.317	127.12	-10.534	1529.749009844	0.29548	6.5468	-2.4753	0.48148	0.52687	0.96617 (max o	3	6313; 5623; 4819	0.02224184	-0.9725088		4.0
	ZINC35271214	-7.8	377.411	-0.7314	7	3	282.84	0.317	127.12	-10.534	1529.749009844	0.29548	6.5468	-2.4753	0.48148	0.52687	0.96617 (max o	3	6313; 5623; 4820	0.04231087	-0.9657228		4.0
	ZINC70673665	-7.8	435.299	0.8783	7	3	287.01	0.29748	113.29	-5.5588	1529.749009844	0.28493	4.9371	3.0825	0.42857	0.35175	0.9984 (max of	5	6162; 5417; 5272; 52	0.5018695	-0.3113138		4.0
	ZINC70673661	-7.8	435.299	0.8783	7	3	287.01	0.29748	113.29	-5.5588	1529.749009844	0.28493	4.9371	3.0825	0.42857	0.35175	0.9984 (max of	5	6162; 5417; 5272; 52	0.5070397	-0.2910507		4.0
	ZINC72320407	-7.8	404.367	-2.4514	11	4	273.15	0.47036	161.21	-7.1638	1529.749009844	0.28493	8.2668	-8.6036	0.46429	0.42659	0.92152	1	2679	0.7720444	0.13541898		3.0
	ZINC03984843	-7.8	361.417	0.3681	7	3	249.05	0.2797	117.32	-7.0984	1529.749009844	0.30685	5.4473	1.1996	0.53846	0.3694	0.95257 (max o	3	1113; 1683; 3181	0.96359444	0.07174802		3.0
	ZINC20464722	-7.8	366.412	-0.7888	8	3	273.29	0.30828	100.49	3.0761	1529.749009844	0.30685	6.6042	-2.5707	0.57692	0.53657	0.92064 (max o	6	5932; 5933; 5549; 55	-0.3875691	-0.0223963		3.0
	ZINC20464722	-7.8	367.420	-2.8985	8	4	287.33	0.34208	101.69	0.36912	1529.749009844	0.30685	8.7139	-9.4461	0.57692	0.53887	0.96998 (max o	6	6264; 5567; 5555; 55	0.67562044	0.36584023		3.0
	ZINC05398713	-7.8	432.499	-0.8061	10	2	324.16	0.30673	125.52	1.444	1529.749009844	0.25735	6.6215	-3.1323	0.45161	0.52854	0.9279 (max of	5	7158; 6973; 6454; 54	0.02579112	0.37341285		3.0
	ZINC38314992	-7.8	412.393	-1.2486	8	0	322.04	0.29388	117.18	0.78259	1529.749009844	0.26593	7.064	-4.6952	0.53333	0.46489	0.8319	1	4141	0.17973007	-0.7346948		3.0
	ZINC49543415	-7.8	434.403	-3.0282	9	1	319.03	0.33229	132.75	3.7628	1529.749009844	0.24931	8.8436	-12.146	0.46875	0.40384	0.95219 (max o	4	6036; 2305; 2673; 35	0.5333732	-0.2630020		3.0
	ZINC08789885	-7.8	446.570	-0.9935	8	3	337.77	0.3386	158.27	5.1985	1529.749009844	0.25735	6.8089	-3.8604	0.6129	0.4906	0.94223 (max o	7	6947; 566; 953; 1802	0.28396976	0.30766985		3.0
	ZINC04221570	-7.8	404.357	-0.8251	11	2	285.71	0.43772	163.5	-2.2832	1529.749009844	0.2751	6.6405	-2.9992	0.44828	0.41476	0.93395 (max o	4	6726; 2427; 3652; 45	-0.7412593	-0.5280669		3.0
	ZINC05434240	-7.8	408.497	-0.4144	9	2	312.4	0.29507	108.05	0.92361	1529.749009844	0.2751	6.2298	-1.5063	0.44828	0.51916	0.93686 (max o	8	600; 752; 1172; 1446	-0.0959454	0.20008275		3.0
	ZINC12603572	-7.8	362.424	-0.2906	7	3	269.78	0.28649	99.1	4.933	1529.749009844	0.30685	6.106	-0.94705	0.61538	0.57194	0.91351 (max o	5	5454; 5062; 1515; 21	0.49231938	-0.3980677		3.0
	ZINC08643426	-7.8	378.423	-0.6271	8	4	280.94	0.33886	117.12	2.4285	1529.749009844	0.29548	6.4425	-2.1223	0.66667	0.54033	0.9464 (max of	5	6481; 6241; 6180; 25	-0.4081991	-0.2524191		3.0
	ZINC12891473	-7.8	445.538	-0.244	9	3	356.88	0.28912	101.41	6.7127	1529.749009844	0.24931	6.0594	-0.97869	0.5	0.50653	0.97948	1	3309	-0.0358342	-0.7784552		3.0
	ZINC06645561	-7.8	391.423	-1.1307	8	1	283.06	0.37413	133.3	5.9517	1529.749009844	0.29548	6.9461	-3.8266	0.51852	0.44686		0		0.84650046	-0.4570135		3.0
	ZINC20465575	-7.8	422.544	-1.5177	8	3	316.16	0.2247	91.24	0.64821	1529.749009844	0.26593	7.3331	-5.7071	0.53333	0.45668	0.97533 (max o	7	6585; 5156; 5143; 33	-0.4344473	0.16123232		3.0
	ZINC20464804	-7.8	392.518	-1.4477	7	3	293.9	0.20769	82.01	0.54449	1529.749009844	0.28493	7.2631	-5.0809	0.57143	0.44591	0.96253 (max o	6	6258; 5156; 5143; 47	-0.4529746	0.12813698		3.0
	ZINC08297631	-7.8	360.460	-4.2698	8	3	289.8	0.32005	94	0.26445	1529.749009844	0.30685	10.085	-13.915	0.65385	0.50507	0.96721 (max o	4	6472; 5438; 3719; 45	0.06589807	-0.1707913		3.0
	ZINC08298574	-7.8	374.487	-4.1449	8	3	318.66	0.32197	81.83	0.21435	1529.749009844	0.29548	9.9603	-14.028	0.62963	0.51785	0.96885 (max o	5	7293; 6173; 3719; 45	0.09897508	-0.1611297		3.0
	ZINC08624449	-7.8	418.516	-2.2364	9	4	336.81	0.31798	113.94	-1.1347	1529.749009844	0.26593	8.0518	-8.4096	0.63333	0.58897	0.92566 (max o	6	5450; 1762; 1763; 25	0.25906417	-0.7219354		3.0
	ZINC12886078	-7.8	444.506	-0.9389	9	1	336.19	0.28258	119.08	8.6017	1529.749009844	0.24931	6.7543	-3.766	0.53125	0.46174	0.97743 (max o	5	5492; 5032; 3747; 41	0.47462422	0.339718		3.0
	ZINC20462796	-7.8	401.481	-3.1469	7	1	324.31	0.27387	105.01	3.7967	1529.749009844	0.2751	8.9623	-11.439	0.51724	0.58238	0.91903	1	5524	0.05977569	-0.2467643		3.0
	ZINC20464902	-7.8	406.569	-4.333	7	5	338.26	0.2554	73.84	2.9172	1529.749009844	0.2751	10.148	-15.75	0.58621	0.5038	0.95372 (max o	4	5558; 5154; 3849; 43	-0.2890312	-0.5476752		3.0
	ZINC20464902	-7.8	405.561	-2.2233	7	4	324.22	0.22315	72.64	4.8778	1529.749009844	0.2751	8.0387	-8.0817	0.58621	0.49987	0.96428 (max o	5	5560; 5152; 5151; 38	0.63818145	-0.4739084		3.0
	ZINC20464902	-7.8	405.561	-1.3383	7	3	306.46	0.17813	76.02	7.4301	1529.749009844	0.2751	7.1537	-4.8647	0.58621	0.44207	0.95372 (max o	6	6258; 5559; 5150; 38	-0.4870973	0.1484409		3.0
	ZINC03871978	-7.8	423.381	-2.8488	12	2	292.92	0.54564	201.89	13.42	1529.749009844	0.2751	8.6642	-10.355	0.51724	0.41769	0.981 (max of 7	7	6395; 5878; 4875; 35	-0.3024	0.81008965		3.0
	ZINC04200815	-7.8	445.516	-2.1189	10	5	309.92	0.4919	212.51	3.9215	1529.749009844	0.2751	7.9343	-7.7022	0.51724	0.49864	0.90776 (max o	9	6431; 6133; 5764; 53	-0.6014873	-0.1697485		3.0
	ZINC12663497	-7.8	397.390	0.6735	10	0	289.38	0.31796	98.38	-0.017525	1529.749009844	0.2751	5.1419	2.4482	0.48276	0.36192	0.96385 (max o	2	5072; 4061	-0.6511113	-0.6161664		3.0
	ZINC08295028	-7.8	367.361	-2.6543	10	0	259	0.49691	149.86	1.3132	1529.749009844	0.31912	8.4697	-8.3176	0.6	0.31262	0.91964 (max o	3	3011; 4102; 4546	-0.5092274	0.42963243		3.0
	ZINC04259334	-7.8	382.396	-0.1504	10	0	273.73	0.45454	136.03	-1.0849	1529.749009844	0.30685	5.9658	-0.49015	0.61538	0.30704	0.93686 (max o	5	5780; 5781; 4589; 30	-0.5376428	0.4402028		3.0
	ZINC12481127	-7.8	396.530	-1.3805	8	2	329.32	0.27192	97.22	5.7828	1529.749009844	0.28493	7.1959	-4.8451	0.53571	0.64867	0.93686 (max o	5	5917; 5909; 4205; 46	-0.1218652	-0.2717137		3.0
	ZINC20465875	-7.8	440.514	-1.3654	8	4	329.01	0.31853	121.57	0.03248	1529.749009844	0.26593	7.1808	-5.1344	0.56667	0.54916	0.97975 (max o	7	7398; 5941; 1874; 18	0.47237185	-0.3816074		3.0
	ZINC04073816	-7.8	385.486	-0.2235	7	2	302.88	0.24683	70.08	5.8068	1529.749009844	0.28493	6.0389	-0.78441	0.53571	0.39962	0.91768 (max o	3	6115; 6114; 1247	-0.350798	-0.6685979		3.0
	ZINC08295578	-7.8	440.606	0.3898	9	2	369.37	0.24046	86.63	5.4998	1529.749009844	0.25735	5.4256	1.5146	0.6129	0.72298	0.86222 (max o	2	6740; 4993	0.7880785	0.23153347		3.0
	ZINC08298571	-7.8	374.487	-4.1449	8	3	318.66	0.32197	81.83	0.21435	1529.749009844	0.29548	9.9603	-14.028	0.62963	0.51785	0.96885 (max o	5	7292; 6984; 5443; 54	0.05827828	-0.1532996		3.0
	ZINC08627139	-7.8	359.402	-1.9961	9	4	254.01	0.43979	146.23	3.3261	1529.749009844	0.33242	7.8115	-6.0048	0.625	0.56927	0.90837 (max o	2	6481; 4653	-0.4102465	-0.1822307		3.0
	ZINC08791212	-7.8	444.506	-0.9389	9	1	336.19	0.28258	119.08	8.6017	1529.749009844	0.24931	6.7543	-3.766	0.53125	0.46174	0.97743 (max o	5	5492; 5032; 4664; 37	0.4953518	0.33693582		3.0
	ZINC15708590	-7.8	388.511	-3.1622	9	3	290.8	0.35168	111.64	10.075	1529.749009844	0.30685	8.9776	-10.305	0.57692	0.52408	0.91652 (max o	3	5897; 5522; 5061	-0.2292424	0.15698606		3.0
	ZINC15708590	-7.8	387.503	-3.507	9	2	279.34	0.32509	99.61	9.3465	1529.749009844	0.30685	9.3224	-11.429	0.57692	0.56098		0		0.9260513	-0.2196765		3.0

Structure	Molecule Name	affinity	Total Molweight	cLogP	H-Acceptors	H-Donors	Total Surface Area	Relative PSA	Polar Surface Area	Druglikeness	Kd (nM) @ 20°C	LE from K...	LLE from Kd ...	LELP from Kd (n...	Shape Index	Molecular...	Neighbor Si...	Neig...	Neighbor	Neighbor ...	Neighbor ...	Ring Systems	frequency
	ZINC20463814	-7.8	447.530	-3.5679	9	4	338.37	0.29314	105.58	3.3893	1529.749009844	0.24931	9.3833	-14.311	0.5625	0.45556	0.93686 (max o	5	6254; 5537; 5134; 47	-0.6124211	-0.6090024		3.0
	ZINC20463848	-7.8	417.504	-3.4979	8	4	316.11	0.28215	96.35	3.2832	1529.749009844	0.26593	9.3133	-13.153	0.6	0.44775	0.92304 (max o	3	7212; 6554; 4726	-0.5989370	-0.5801241		3.0
	ZINC04040862	-7.8	356.353	-2.0541	7	0	238.96	0.33922	99.88	2.5289	1529.749009844	0.30685	7.8695	-6.6942	0.38462	0.26356	0.95257 (max o	3	1392; 3193; 4067	-0.4353257	0.8078773		2.0
	ZINC04270784	-7.8	391.423	0.8708	9	0	293.75	0.27156	88.62	1.7426	1529.749009844	0.28493	4.9446	3.0562	0.57143	0.6028	0.90104	1	6147	-0.3004984	0.93718207		2.0
	ZINC04098356	-7.8	388.368	-1.827	10	4	263.15	0.45024	151.98	-6.054	1529.749009844	0.29548	7.6424	-6.1831	0.48148	0.37688	0.93686	1	1582	-0.7944473	-0.4424936		2.0
	ZINC20464725	-7.8	381.427	-0.7949	9	4	284.75	0.33612	112.52	3.1071	1529.749009844	0.29548	6.6103	-2.6902	0.59259	0.55967	0.9339 (max of	2	5549; 2227	-0.3729300	-0.0942801		2.0
	ZINC20463375	-7.8	404.509	-2.0973	8	1	331.61	0.2594	90.24	4.1982	1529.749009844	0.2751	7.9127	-7.6237	0.58621	0.59061	0.96428 (max o	6	5848; 2145; 2581; 25	0.64034754	0.4141499		2.0
	ZINC03838471	-7.8	377.396	-0.7421	9	4	286.9	0.38066	137.85	-6.3969	1529.749009844	0.29548	6.5575	-2.5115	0.62963	0.53344	0.88391	1	2761	-0.4387441	-0.1294304		2.0
	ZINC31163371	-7.8	445.483	-2.0251	10	4	327.72	0.37459	165.81	-2.0398	1529.749009844	0.25735	7.8405	-7.8689	0.51613	0.62878	0.96739 (max o	5	6007; 2638; 3040; 30	0.21004578	0.35663188		2.0
	ZINC04026907	-7.8	421.448	-0.09	7	3	320.03	0.26341	123.5	0.7285	1529.749009844	0.25735	5.9054	-0.34971	0.58065	0.49759	0.95267	1	3189	0.66201824	-0.6500985		2.0
	ZINC08624303	-7.8	364.397	-0.6966	8	3	267.28	0.32659	108.33	2.8851	1529.749009844	0.30685	6.512	-2.2702	0.61538	0.48584	0.91351 (max o	4	5454; 941; 2877; 328	0.51135087	-0.3887016		2.0
	ZINC08624391	-7.8	413.513	-0.1252	9	4	313.04	0.31545	120.36	-0.0072673	1529.749009844	0.2751	5.9406	-0.4551	0.62069	0.56755	0.9083 (max of	5	6749; 5449; 2924; 32	0.19316979	-0.3986897		2.0
	ZINC12658529	-7.8	427.448	0.0981	10	5	311.83	0.38463	154.78	-52.971	1529.749009844	0.26593	5.7173	0.36889	0.46667	0.56473	0.96092 (max o	3	4823; 3483; 4232	0.02456718	0.9794866		2.0
	ZINC35457130	-7.8	440.400	-1.5969	11	6	304.31	0.44258	183.21	-10.12	1529.749009844	0.25735	7.4123	-6.205	0.48387	0.5224	0.95521 (max o	3	3496; 3497; 3498	-0.8018688	0.05099490		2.0
	ZINC03831538	-7.8	382.416	-3.6789	8	1	252.44	0.52916	183.21	11.228	1529.749009844	0.31912	9.4943	-11.528	0.48	0.33609	0.90896 (max o	3	6961; 4913; 3594	-0.3706886	-0.4290049		2.0
	ZINC04040674	-7.8	354.317	-3.2387	8	2	268.26	0.38634	138.46	3.4589	1529.749009844	0.30685	9.0541	-10.555	0.53846	0.53717	0.96721	1	3632	0.7630331	-0.1518554		2.0
	ZINC08740539	-7.8	386.371	0.8103	12	5	275.91	0.50121	176.82	1.3234	1529.749009844	0.28493	5.0051	2.8439	0.5	0.33348	0.98451 (max o	12	6484; 5030; 2509; 25	-0.2385471	-0.6600522		2.0
	ZINC12481463	-7.8	446.546	-0.8494	9	2	360.56	0.2761	106.45	4.7697	1529.749009844	0.24931	6.6648	-3.407	0.53125	0.57201	0.92584 (max o	4	5913; 1796; 3761; 42	-0.0154010	-0.2244424		2.0
	ZINC08624399	-7.8	401.458	-1.4454	10	3	295.62	0.34111	120.8	2.9559	1529.749009844	0.28493	7.2608	-5.0728	0.60714	0.58592	0.8934 (max of	4	1495; 3742; 3775; 46	0.18224326	-0.4242994		2.0
	ZINC35360985	-7.8	413.421	-0.3992	10	5	301.66	0.3976	154.78	-17.14	1529.749009844	0.2751	6.2146	-1.4511	0.58621	0.54808	0.9617 (max of	3	6224; 5222; 3778	-0.7113473	0.5590793		2.0
	ZINC31155678	-7.8	372.456	0.8828	7	4	276.35	0.30917	116.45	-5.701	1529.749009844	0.30685	4.9326	2.877	0.53846	0.50825	0.97533 (max o	6	5281; 5189; 3444; 38	0.2589758	-0.4850675		2.0
	ZINC40309274	-7.8	392.411	-1.7687	9	2	295.72	0.43166	157.92	1.8006	1529.749009844	0.29548	7.5841	-5.9858	0.62963	0.52417	0.91007	1	3948	-0.2156423	-0.961109		2.0
	ZINC03839363	-7.8	408.521	-0.5609	8	2	290.11	0.34356	124.12	4.2875	1529.749009844	0.28493	6.3763	-1.9686	0.53571	0.32805	0.98006 (max o	6	430; 2000; 2096; 370	-0.5922634	-0.0161228		2.0
	ZINC03841155	-7.8	377.464	0.1576	7	3	275.13	0.34958	124.04	6.5345	1529.749009844	0.30685	5.6578	0.51361	0.65385	0.492	0.88538	1	4043	-0.4361297	-0.2803126		2.0
	ZINC12494774	-7.8	352.342	-7.7802	9	3	240.72	0.39868	134.24	2.8108	1529.749009844	0.31912	13.596	-24.38	0.48	0.44503	0.93007 (max o	3	2525; 3616; 4051	-0.73509	-0.0120168		2.0
	ZINC04098666	-7.8	367.397	-0.1337	8	2	257.78	0.30142	110.13	0.26035	1529.749009844	0.30685	5.9491	-0.43572	0.38462	0.29035	0.88798 (max o	2	4079; 4080	-0.0303479	0.938453		2.0
	ZINC08298899	-7.8	362.520	-3.3209	7	3	294.39	0.26713	80.86	-4.1638	1529.749009844	0.30685	9.1363	-10.823	0.65385	0.49452	0.96721 (max o	3	5445; 5444; 4156	0.01040692	-0.0794956		2.0
	ZINC08740486	-7.8	386.371	0.8103	12	5	275.91	0.50121	176.82	1.3234	1529.749009844	0.28493	5.0051	2.8439	0.5	0.33348	0.98451 (max o	12	6759; 6192; 4627; 25	-0.2292292	-0.6775868		2.0
	ZINC35361270	-7.8	427.448	0.0981	10	5	311.83	0.38463	154.78	-52.971	1529.749009844	0.26593	5.7173	0.36889	0.46667	0.56298	0.96092 (max o	3	3483; 4232; 4659	0.00211254	0.9804927		2.0
	ZINC19369923	-7.8	423.572	-1.5432	7	3	331.85	0.24222	83.67	-1.1127	1529.749009844	0.26593	7.3586	-5.803	0.6	0.51463	0.95123 (max o	5	4775; 4773; 4704; 46	-0.0293380	0.03637999		2.0
	ZINC19369923	-7.8	423.572	-1.5432	7	3	331.85	0.24222	83.67	-1.2522	1529.749009844	0.26593	7.3586	-5.803	0.6	0.54484	0.94558 (max o	5	4776; 4774; 4705; 42	-0.0314116	0.08102149		2.0
	ZINC22059930	-7.8	389.386	-1.5082	9	1	272.97	0.3459	125.8	3.0344	1529.749009844	0.28493	7.3236	-5.2933	0.5	0.34135	0.9428	1	4338	-0.6192968	-0.7701098		2.0
	ZINC31162113	-7.8	381.423	-0.1984	8	6	269.11	0.35476	135.54	-0.95599	1529.749009844	0.29548	6.0138	-0.67145	0.44444	0.48673	0.94905	1	4373	-0.8461655	-0.5088998		2.0
	ZINC67913880	-7.8	389.332	-4.9553	11	4	258.42	0.51374	175.04	-4.8669	1529.749009844	0.29548	10.771	-16.77	0.44444	0.41256	0.94302 (max o	3	2319; 2689; 4437	-0.3486758	0.8623021		2.0
	ZINC00631635	-7.8	361.425	-1.2272	9	2	256.75	0.28008	106.02	-2.1694	1529.749009844	0.30685	7.0426	-3.9994	0.46154	0.45034	0.92032	1	6494	0.55537146	0.72370696		2.0
	ZINC01532360	-7.8	370.428	-0.3474	7	4	295.68	0.27733	119.19	-2.5311	1529.749009844	0.29548	6.1628	-1.1757	0.7037	0.56793	0.9578	1	7283	0.9060111	-0.2413767		2.0
	ZINC04212489	-7.8	388.442	-5.1589	8	1	290.49	0.28407	120.42	3.8091	1529.749009844	0.28493	10.974	-18.106	0.5	0.52143	0.8973	1	4476	0.39627865	0.68626434		2.0
	ZINC03781951	-7.8	417.504	-2.2487	8	1	318.98	0.24528	106.59	-0.018701	1529.749009844	0.26593	8.0641	-8.4559	0.46667	0.50456	0.8973 (max of	2	4534; 4477	0.4023107	0.70593435		2.0
	ZINC04026725	-7.8	379.411	-0.2292	6	2	277.36	0.25671	103.27	-2.455	1529.749009844	0.28493	6.0446	-0.80441	0.5	0.46706	0.94573 (max o	6	6379; 6112; 6074; 57	-0.0462191	0.66436887		2.0
	ZINC04028636	-7.8	354.465	-0.927	6	1	260.06	0.26313	73.9	-9.96	1529.749009844	0.31912	6.7424	-2.9049	0.52	0.43249	0.92662 (max o	2	7182; 2217	-0.5892496	0.5399206		2.0
	ZINC04082896	-7.8	381.515	-0.6972	7	2	301.39	0.27503	117.18	3.6596	1529.749009844	0.29548	6.5126	-2.3595	0.66667	0.58351	0.93046	1	5309	-0.9534745	0.05080577		2.0
	ZINC04259305	-7.8	363.397	-0.3051	9	1	256.54	0.45053	139.68	1.3967	1529.749009844	0.31912	6.1205	-0.95607	0.6	0.34954	0.93686 (max o	3	5780; 5394; 4547	-0.5165586	0.5034375		2.0
	ZINC04259413	-7.8	355.418	-0.3635	9	1	250.82	0.46081	139.68	0.033184	1529.749009844	0.33242	6.1789	-1.0935	0.58333	0.29694	0.93341 (max o	3	5781; 4102; 4545	-0.5045265	0.4668316		2.0

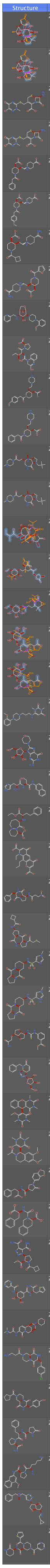

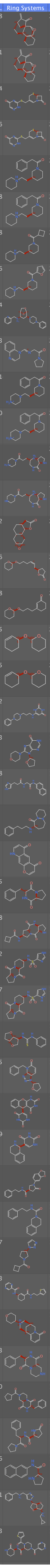

Structure	Molecule Name	affinity	Total Molweight	cLogP	H-Acceptors	H-Donors	Total Surface Area	Relative PSA	Polar Surface Area	Druglikeness	Kd (nM) @ 20°C	LE from K...	LLE from Kd ...	LELP from Kd (n...	Shape Index	Molecular...	Neighbor Si...	Neig...	Neighbor	Neighbor ...	Neighbor ...	Ring Systems	frequency
	ZINC05741430	-7.8	364.436	0.5857	6	4	231.86	0.32537	107.22	-5.1582	1529.749009844	0.30685	5.2297	1.9088	0.34615	0.35342	0.93686	1	4580	-0.0476336	-0.9576218		2.0
	ZINC05734580	-7.8	364.436	0.5857	6	4	231.86	0.32537	107.22	-5.1582	1529.749009844	0.30685	5.2297	1.9088	0.34615	0.35342	0.93686	1	4581	-0.0270037	-0.9634261		2.0
	ZINC19702837	-7.8	370.389	-6.1229	10	1	238.26	0.61806	206.77	6.8173	1529.749009844	0.33242	11.938	-18.419	0.54167	0.48299	0.88866	1	4698	0.42056996	-0.8467394		2.0
	ZINC19702837	-7.8	369.381	-8.3224	10	1	239.05	0.61933	205.15	6.197	1529.749009844	0.33242	14.138	-25.036	0.54167	0.48299	0.88866	1	4699	0.4094846	-0.8641546		2.0
	ZINC20462819	-7.8	415.508	-2.8603	7	1	338.07	0.26273	105.01	5.0634	1529.749009844	0.26593	8.6757	-10.756	0.56667	0.61471	0.95837 (max c	5	4291; 4292; 4293; 42	0.02873132	-0.227559		2.0
	ZINC20462821	-7.8	445.534	-2.9303	8	1	360.33	0.27425	114.24	5.0897	1529.749009844	0.24931	8.7457	-11.754	0.59375	0.62659	0.93952 (max c	6	6992; 5913; 4711; 42	-0.0036876	-0.2073268		2.0
	ZINC20463135	-7.8	379.499	-1.9639	7	2	305.82	0.29367	107.97	5.3457	1529.749009844	0.29548	7.7793	-6.6464	0.55556	0.56437	0.93058 (max c	5	5908; 5132; 5125; 47	-0.1999061	-0.2714398		2.0
	ZINC20463157	-7.8	391.466	-1.9915	8	2	314.99	0.32992	121.11	6.2223	1529.749009844	0.28493	7.8069	-6.9895	0.53571	0.57504	0.93385 (max c	5	5908; 5527; 5126; 42	-0.1910256	-0.2932796		2.0
	ZINC20463824	-7.8	427.523	-1.0505	8	2	313.29	0.21996	83.9	4.6027	1529.749009844	0.25735	6.8659	-4.0819	0.54839	0.44422	0.97642 (max c	6	6255; 6216; 5853; 55	0.36720452	-0.0319304		2.0
	ZINC20758642	-7.8	401.442	-1.3137	9	2	299.35	0.32297	128.86	7.7037	1529.749009844	0.2751	7.1291	-4.7753	0.44828	0.44519	0.95401	1	5168	0.4988276	-0.6063693		2.0
	ZINC22920596	-7.8	403.521	-1.5475	7	1	316.25	0.20617	89.52	4.405	1529.749009844	0.2751	7.3629	-5.6252	0.58621	0.62221	0.93091 (max c	4	7376; 5910; 4210; 47	-0.1260666	-0.1006511		2.0
	ZINC22920592	-7.8	373.495	-1.4775	6	1	293.99	0.18776	80.29	4.381	1529.749009844	0.29548	7.2929	-5.0003	0.55556	0.60773	0.92774 (max c	4	7524; 6851; 4209; 47	-0.1589527	-0.1213940		2.0
	ZINC22923491	-7.8	395.590	-3.2095	7	3	336.05	0.23752	61.53	2.2633	1529.749009844	0.28493	9.0249	-11.264	0.57143	0.53882	0.90441 (max c	8	6321; 5625; 5583; 55	-0.0515532	0.5749174		2.0
	ZINC22923491	-7.8	394.582	-1.0998	7	2	322.01	0.20428	60.33	6.4409	1529.749009844	0.28493	6.9152	-3.8599	0.57143	0.53882	0.92442 (max c	8	6321; 5625; 5586; 55	-0.0971095	0.5436449		2.0
	ZINC35458255	-7.8	380.435	0.7597	7	2	268.7	0.33469	105.59	-1.5895	1529.749009844	0.29548	5.0557	2.5711	0.44444	0.35504	0.93996	1	5235	0.06878947	-0.9894342		2.0
	ZINC35458291	-7.8	445.483	-2.0251	10	4	327.72	0.37459	165.81	-2.0398	1529.749009844	0.25735	7.8405	-7.8689	0.51613	0.62475	0.96739 (max c	5	6007; 3040; 3041; 30	0.15540467	0.38506916		2.0
	ZINC49181280	-7.8	372.456	1.0537	7	4	275.82	0.30977	116.45	-4.0371	1529.749009844	0.30685	4.7617	3.434	0.5	0.55248	0.97533 (max c	3	5256; 3526; 4843	0.77626675	0.35514688		2.0
	ZINC49601625	-7.8	388.455	0.0267	8	5	280.91	0.35079	136.68	-6.634	1529.749009844	0.29548	5.7887	0.090361	0.48148	0.49366	0.94302 (max c	3	5664; 5663; 4855	0.55201477	0.60184455		2.0
	ZINC49601623	-7.8	388.455	0.0267	8	5	280.91	0.35079	136.68	-6.634	1529.749009844	0.29548	5.7887	0.090361	0.48148	0.49366	0.94605 (max c	3	5664; 5663; 4856	0.57154226	0.60938895		2.0
	ZINC01544805	-7.8	387.482	1.2662	8	1	302.23	0.20428	68.36	6.3768	1529.749009844	0.28493	4.5492	4.4439	0.60714	0.59815		0		-0.5890518	-0.7711472		1.0
	ZINC02124467	-7.8	368.349	-2.6524	12	5	249.57	0.52879	167.69	2.18	1529.749009844	0.30685	8.4678	-8.6441	0.42308	0.40932	0.88538 (max c	2	5808; 4001	-0.5414582	-0.5519183		1.0
	ZINC02561058	-7.8	368.416	-3.4059	8	5	280.97	0.36139	134.75	4.2904	1529.749009844	0.29548	9.2213	-11.527	0.59259	0.46544	0.87363	1	5312	0.7177792	0.28541288		1.0
	ZINC03781951	-7.8	416.496	-3.4801	8	1	322.7	0.25398	102.01	0.81083	1529.749009844	0.26593	9.2955	-13.086	0.46667	0.5608	0.83518	1	4476	0.4243874	0.7069745		1.0
	ZINC03782807	-7.8	369.328	-2.7796	8	0	267.84	0.35196	126.87	1.2833	1529.749009844	0.29548	8.595	-9.407	0.40741	0.39317		0		-0.3390318	0.8991578		1.0
	ZINC03838956	-7.8	360.369	-1.0653	9	2	262.7	0.36547	124.33	1.6237	1529.749009844	0.30685	6.8807	-3.4718	0.61538	0.46625	0.84311	1	3817	0.3648702	0.64361185		1.0
	ZINC03839353	-7.8	394.494	-0.9029	8	2	276.35	0.36067	124.12	6.0714	1529.749009844	0.29548	6.7183	-3.0557	0.55556	0.33881	0.93069 (max c	7	189; 253; 2096; 4488	-0.6284852	-0.0218078		1.0
	ZINC03839756	-7.8	367.429	-1.1091	9	1	249.18	0.36544	112.99	8.0643	1529.749009844	0.31912	6.9245	-3.4755	0.52	0.39468	0.84105	1	4490	0.739482	-0.5155192		1.0
	ZINC03839756	-7.8	368.437	-3.0869	9	2	238.21	0.33622	114.24	7.2201	1529.749009844	0.31912	8.9023	-9.6732	0.52	0.39468	0.85067 (max c	2	3600; 4489	0.7433077	-0.4933177		1.0
	ZINC03841706	-7.8	395.435	-0.7061	9	3	289.45	0.45697	153.23	-1.126	1529.749009844	0.29548	6.5215	-2.3897	0.62963	0.51335		0		-0.6792207	0.7201574		1.0
	ZINC03852691	-7.8	360.369	-1.3452	9	3	252.2	0.36661	118.19	-4.0555	1529.749009844	0.30685	7.1606	-4.384	0.53846	0.44079	0.92368 (max c	3	1110; 2014; 2765	0.82636625	0.45094565		1.0
	ZINC04026322	-7.8	412.401	2.0625	10	0	284.6	0.30344	99.7	2.5635	1529.749009844	0.26593	3.7529	7.7557	0.36667	0.36995	0.95579	1	4951	0.57405823	0.5028876		1.0
	ZINC04029810	-7.8	389.407	1.247	9	1	281	0.29491	99.62	4.6354	1529.749009844	0.28493	4.5684	4.3765	0.42857	0.38093	0.93385	1	6116	0.6049677	-0.6522189		1.0
	ZINC04085266	-7.8	395.390	-0.5023	8	1	288.6	0.30634	108	6.3948	1529.749009844	0.2751	6.3177	-1.8259	0.55172	0.40531	0.96682 (max c	5	5367; 4959; 1124; 16	-0.0364959	-0.688308		1.0
	ZINC04089137	-7.8	366.372	-3.0084	7	1	275.87	0.29974	112.6	5.0728	1529.749009844	0.29548	8.8238	-10.181	0.51852	0.55699		0		-0.0729567	-0.64873		1.0
	ZINC04221567	-7.8	364.361	-1.7096	11	2	259.88	0.48122	163.5	-1.3894	1529.749009844	0.30685	7.525	-5.5715	0.46154	0.33889	0.89885 (max c	4	6726; 4536; 3222; 36	-0.7459244	-0.4883307		1.0
	ZINC04221784	-7.8	431.472	-1.7557	10	5	312.6	0.46644	195.77	6.4223	1529.749009844	0.26593	7.5711	-6.602	0.53333	0.44903	0.96832 (max c	5	6189; 2428; 3167; 36	-0.4972261	-0.1310803		1.0
	ZINC04259188	-7.8	376.499	-3.643	7	3	310.72	0.27765	67.52	1.8923	1529.749009844	0.29548	9.4584	-12.329	0.59259	0.42095	0.89979	1	906	-0.5386030	0.8347837		1.0
	ZINC04260050	-7.8	365.840	-3.237	7	2	262.28	0.27513	97.36	5.7162	1529.749009844	0.31912	9.0524	-10.144	0.64	0.50307	0.83537	1	4043	-0.4186861	-0.2945273		1.0
	ZINC04260224	-7.8	371.436	0.8543	7	2	272	0.26022	89.95	4.5013	1529.749009844	0.29548	4.9611	2.8912	0.59259	0.43183	0.86859	1	730	-0.3147721	-0.2701350		1.0
	ZINC04899474	-7.8	372.424	-0.5686	8	3	279.06	0.33649	121.6	8.9902	1529.749009844	0.29548	6.384	-1.9243	0.44444	0.48841		0		-0.7964949	0.5697323		1.0
	ZINC04899481	-7.8	400.521	-4.7208	7	3	310.66	0.25768	113.92	3.3402	1529.749009844	0.2751	10.536	-17.16	0.58621	0.45757	0.88922	1	6684	0.6393793	0.74936706		1.0
	ZINC05412454	-7.8	385.510	-2.4305	7	2	302.4	0.24517	70.22	-2.3462	1529.749009844	0.28493	8.2459	-8.5302	0.64286	0.46357	0.88128 (max c	5	455; 1184; 1432; 143	-0.0943413	-0.2209651		1.0
	ZINC05414663	-7.8	442.499	-0.0687	10	2	321.56	0.38711	152.84	6.634	1529.749009844	0.25735	5.8841	-0.26695	0.45161	0.48833	0.96971 (max c	2	1742; 3694	0.00451169	0.20821163		1.0

Structure	Molecule Name	affinity	Total Molweight	cLogP	H-Acceptors	H-Donors	Total Surface Area	Relative PSA	Polar Surface Area	Druglikeness	Kd (nM) @ 20°C	LE from K...	LLE from Kd ...	LELP from Kd (n...	Shape Index	Molecular...	Neighbor Si...	Neig...	Neighbor	Neighbor ...	Neighbor ...	Ring Systems	frequency
	ZINC05415723	-7.8	358.398	-1.2025	9	1	230.99	0.49344	157.71	7.5893	1529.749009844	0.34687	7.0179	-3.4667	0.52174	0.49338		0		-0.0376713	-0.9833949		1.0
	ZINC06062920	-7.8	413.476	-4.7447	9	6	300.57	0.33593	146.15	1.9164	1529.749009844	0.26593	10.56	-17.842	0.53333	0.40618		0		0.89055854	-0.3201555		1.0
	ZINC08295663	-7.8	362.385	-1.947	8	0	254.48	0.37166	126.36	5.9183	1529.749009844	0.31912	7.7624	-6.1012	0.6	0.53496	0.83367	1	1743	0.18779254	0.9608505		1.0
	ZINC08299530	-7.8	410.493	-1.983	10	3	306.38	0.32613	124.66	4.7911	1529.749009844	0.2751	7.7984	-7.2082	0.44828	0.51681	0.89025	1	754	-0.0988223	0.25628787		1.0
	ZINC08624445	-7.8	377.424	-4.1278	10	4	281.95	0.38383	139	-2.1529	1529.749009844	0.29548	9.9432	-13.97	0.66667	0.54023	0.92124 (max o	3	2108; 2887; 3774	0.8149894	-0.4168008		1.0
	ZINC08624513	-7.8	360.365	0.0479	8	4	267.85	0.37077	121.03	1.4119	1529.749009844	0.30685	5.7675	0.1561	0.61538	0.4844	0.90104 (max o	2	941; 2117	0.51380885	-0.3463913		1.0
	ZINC08627147	-7.8	358.417	-1.3948	7	4	269.21	0.29497	108.29	-0.17452	1529.749009844	0.30685	7.2102	-4.5456	0.65385	0.43483	0.91059 (max o	4	2154; 2926; 4172; 41	0.13968594	-0.5133233		1.0
	ZINC08636321	-7.8	431.515	-0.7826	9	4	313.85	0.37916	146.3	5.9857	1529.749009844	0.26593	6.598	-2.9428	0.6	0.42722	0.91292 (max o	5	7334; 5483; 3716; 42	-0.465336	0.3997941		1.0
	ZINC08765460	-7.8	442.443	-1.3835	9	1	325.29	0.31301	115.38	0.94	1529.749009844	0.24931	7.1989	-5.5493	0.46875	0.38684	0.9665 (max of	3	5868; 5493; 3746	-0.9358628	-0.2373288		1.0
	ZINC12480774	-7.8	399.489	-0.7306	7	1	332.98	0.26002	90.91	4.5992	1529.749009844	0.2751	6.546	-2.6557	0.55172	0.59339	0.87803 (max o	2	5961; 5131	-0.3736796	-0.1718932		1.0
	ZINC12481024	-7.8	379.499	-1.557	7	1	307.36	0.25407	85.19	5.5966	1529.749009844	0.29548	7.3724	-5.2694	0.51852	0.58903	0.93367 (max o	9	5915; 5909; 5908; 46	-0.1313420	-0.3110719		1.0
	ZINC12481033	-7.8	393.526	-1.215	7	1	321.12	0.24318	85.19	4.3084	1529.749009844	0.28493	7.0304	-4.2642	0.53571	0.57899	0.93686 (max o	10	5909; 5132; 4715; 46	-0.1574608	-0.2850167		1.0
	ZINC12481140	-7.8	389.470	-1.4961	7	0	306.25	0.22028	82.14	4.2122	1529.749009844	0.28493	7.3115	-5.2508	0.57143	0.56546	0.92757 (max o	4	6764; 6213; 1860; 42	-0.1846093	-0.1768018		1.0
	ZINC12529672	-7.8	380.438	-1.7782	7	3	267.33	0.27195	101.63	4.6783	1529.749009844	0.29548	7.5936	-6.018	0.59259	0.46187	0.96064 (max o	2	6847; 5851	0.39523482	-0.1773286		1.0
	ZINC12530603	-7.8	393.506	-2.1856	8	3	306.43	0.30408	92.18	6.2425	1529.749009844	0.28493	8.001	-7.6707	0.57143	0.46845	0.88391 (max o	3	6771; 5521; 5061	-0.2519644	0.10471274		1.0
	ZINC15952927	-7.8	373.368	-0.8314	10	5	265.98	0.4309	141.23	2.3159	1529.749009844	0.29548	6.6468	-2.8137	0.48148	0.40008	0.95493 (max o	5	935; 1074; 1969; 289	0.3305842	-0.5137769		1.0
	ZINC19570994	-7.8	395.458	-2.6455	7	1	317.93	0.2594	87	3.4563	1529.749009844	0.2751	8.4609	-9.6164	0.48276	0.50366	0.91903	1	4697	0.01076042	-0.9273115		1.0
	ZINC19570997	-7.8	395.458	-2.6455	7	1	317.93	0.2594	87	3.4563	1529.749009844	0.2751	8.4609	-9.6164	0.48276	0.50467	0.91903	1	4696	0.01297084	-0.9082437		1.0
	ZINC20410976	-7.8	378.511	0.5118	6	1	280.31	0.21722	65.54	3.3512	1529.749009844	0.29548	5.3036	1.7321	0.55556	0.47441	0.93686 (max o	3	1245; 1246; 2978	-0.8685065	-0.1642011		1.0
	ZINC20462828	-7.8	393.502	-3.3001	7	1	318.59	0.27879	105.01	2.6264	1529.749009844	0.28493	9.1155	-11.582	0.53571	0.56589	0.93069 (max o	2	5125; 4710	-0.2543484	-0.2453203		1.0
	ZINC20462830	-7.8	407.529	-2.9581	7	1	332.35	0.26725	105.01	0.1747	1529.749009844	0.2751	8.7735	-10.753	0.55172	0.59005	0.9426 (max of	4	5125; 4714; 3834; 47	-0.274827	-0.2536194		1.0
	ZINC20462870	-7.8	436.914	-3.1559	8	1	338.49	0.29481	117.9	4.9467	1529.749009844	0.26593	8.9713	-11.867	0.53333	0.59941	0.92821 (max o	6	6250; 2579; 2980; 42	0.02108469	-0.1679284		1.0
	ZINC20462872	-7.8	421.536	-2.9937	7	1	334.01	0.32688	133.25	6.873	1529.749009844	0.2751	8.8091	-10.882	0.55172	0.62255	0.93091 (max o	3	6249; 5126; 5127	-0.2575282	-0.2665251		1.0
	ZINC20462876	-7.8	422.544	-3.0175	8	2	343.81	0.29167	117.04	1.0105	1529.749009844	0.26593	8.8329	-11.347	0.56667	0.61753	0.90725 (max o	3	7375; 3834; 4710	-0.3125969	-0.2492105		1.0
	ZINC20463221	-7.8	402.513	-1.409	7	0	318.81	0.19137	76.15	8.3938	1529.749009844	0.2751	7.2244	-5.1217	0.58621	0.59575	0.88128	1	4723	-0.1331426	-0.2347366		1.0
	ZINC20463244	-7.8	393.526	-1.215	7	1	321.12	0.24318	85.19	2.5416	1529.749009844	0.28493	7.0304	-4.2642	0.53571	0.55926	0.9398 (max of	3	5132; 4296; 4721	-0.1601298	-0.2629709		1.0
	ZINC20463244	-7.8	392.518	-1.3466	7	0	307.08	0.20858	83.99	7.0783	1529.749009844	0.28493	7.162	-4.7261	0.53571	0.56364	0.93069 (max o	6	6497; 5916; 5912; 51	-0.2353612	-0.2019240		1.0
	ZINC20463246	-7.8	407.553	-0.873	7	1	334.88	0.23319	85.19	0.094341	1529.749009844	0.2751	6.6884	-3.1733	0.55172	0.58409	0.92152 (max o	2	5132; 4719	-0.1768663	-0.2794936		1.0
	ZINC20463246	-7.8	406.545	-1.0046	7	0	320.84	0.19963	83.99	4.6408	1529.749009844	0.2751	6.82	-3.6517	0.55172	0.58803	0.92152 (max o	3	6498; 5133; 4720	-0.2499324	-0.2245594		1.0
	ZINC20463824	-7.8	428.531	-3.1602	8	3	327.33	0.25341	85.1	2.2729	1529.749009844	0.25735	8.9756	-12.28	0.54839	0.44845	0.97642 (max o	6	6558; 6556; 6254; 55	-0.5768181	-0.633437		1.0
	ZINC20464680	-7.8	449.516	-0.2813	6	3	346.14	0.19455	66.6	-0.054125	1529.749009844	0.24931	6.0967	-1.1283	0.4375	0.48542	0.93948	1	6256	0.50022656	-0.7042489		1.0
	ZINC20464725	-7.8	382.435	-2.9046	9	5	298.79	0.36731	113.72	0.20551	1529.749009844	0.29548	8.72	-9.8301	0.59259	0.56165	0.91768	1	5550	0.6339561	0.31922796		1.0
	ZINC20465244	-7.8	371.479	-3.5376	6	4	292.12	0.24819	83.63	-0.93076	1529.749009844	0.29548	9.353	-11.972	0.55556	0.39177	0.91157 (max o	3	7224; 3395; 4317	-0.3817956	-0.6458137		1.0
	ZINC20465244	-7.8	370.471	-1.4279	6	3	278.08	0.21023	82.43	2.1441	1529.749009844	0.29548	7.2433	-4.8324	0.55556	0.38744	0.91482 (max o	4	7225; 6822; 3396; 43	-0.529211	0.20221724		1.0
	ZINC20465601	-7.8	403.457	-2.5528	9	4	288.57	0.28385	112.55	5.8322	1529.749009844	0.2751	8.3682	-9.2794	0.55172	0.45912	0.8991 (max of	4	7052; 5940; 5556; 22	-0.4185393	0.08285066		1.0
	ZINC20465601	-7.8	403.457	-2.6847	9	4	313.58	0.34097	112.5	4.0149	1529.749009844	0.2751	8.5001	-9.7589	0.55172	0.48066	0.96428 (max o	4	7053; 5939; 5557; 34	-0.3879381	-0.5919798		1.0
	ZINC20465854	-7.8	395.478	-2.6321	9	4	313.91	0.32751	108.59	3.3594	1529.749009844	0.28493	8.4475	-9.2377	0.57143	0.5992	0.92464 (max o	3	6589; 5590; 5157	0.2971518	-0.0500210		1.0
	ZINC20761557	-7.8	362.341	0.4563	10	3	258.65	0.41388	118.23	3.2492	1529.749009844	0.30685	5.3591	1.4871	0.46154	0.44343	0.92702	1	6842	-0.1953555	0.9513721		1.0
	ZINC20762895	-7.8	402.430	-1.4407	10	3	295.21	0.33566	127.76	-3.5341	1529.749009844	0.2751	7.2561	-5.2369	0.48276	0.54984	0.93686 (max o	5	6308; 5990; 5619; 56	-0.5983597	0.17697224		1.0
	ZINC20763400	-7.8	396.439	-1.0158	8	2	290.57	0.39732	150.29	1.7857	1529.749009844	0.29548	6.8312	-3.4378	0.55556	0.53245		0		0.6063961	0.76193756		1.0
	ZINC25759920	-7.8	429.475	-2.5377	10	5	326.59	0.35154	157.36	5.5343	1529.749009844	0.25735	8.3531	-9.8607	0.51613	0.57092	0.92304	1	4782	-0.6929841	-0.7016955		1.0
	ZINC25759920	-7.8	428.467	-2.3488	10	4	337.56	0.37262	156.11	6.0779	1529.749009844	0.25735	8.1642	-9.1267	0.51613	0.57092	0.92304	1	4781	-0.6766232	-0.7082051		1.0
	ZINC27849754	-7.8	355.845	0.5371	6	1	248.24	0.27119	89.39	8.5398	1529.749009844	0.34687	5.2783	1.5484	0.6087	0.55332		0		0.89461344	0.16237058		1.0

Structure	Molecule Name	affinity	Total Molweight	cLogP	H-Acceptors	H-Donors	Total Surface Area	Relative PSA	Polar Surface Area	Druglikeness	Kd (nM) @ 20°C	LE from K...	LLE from Kd ...	LELP from Kd (n...	Shape Index	Molecular...	Neighbor Si...	Neig...	Neighbor	Neighbor ...	Neighbor ...	Ring Systems	frequency
	ZINC30878784	-7.8	377.507	-1.1775	7	3	284.81	0.19206	79.18	8.9253	1529.749009844	0.29548	6.9929	-3.985	0.62963	0.53625	0.89169	1	3439	-0.3418004	0.92264986		1.0
	ZINC30879243	-7.8	361.504	0.65	5	2	302.36	0.18944	54.21	5.1168	1529.749009844	0.30685	5.1654	2.1183	0.61538	0.56626	0.94326	1	4357	0.62443155	0.7487303		1.0
	ZINC31159429	-7.8	432.508	-0.6954	9	6	297.92	0.37473	156.91	-3.8603	1529.749009844	0.26593	6.5108	-2.6149	0.36667	0.44165	0.95318 (max o	3	5682; 5195; 3547	-0.4734007	-0.7015197		1.0
	ZINC31163178	-7.8	420.366	-2.9159	12	6	279.72	0.50636	184.6	0.6027	1529.749009844	0.2751	8.7313	-10.599	0.44828	0.35539		0		0.98304236	0.05349192		1.0
	ZINC31166156	-7.8	424.426	-2.3426	11	5	278.1	0.56584	222.85	-4.2817	1529.749009844	0.29548	8.158	-7.9281	0.48148	0.5472	0.8541	1	5374	-0.3594984	0.5681288		1.0
	ZINC34054656	-7.8	352.425	0.9315	6	3	253.92	0.29687	104.06	-1.3444	1529.749009844	0.31912	4.8839	2.919	0.44	0.46196	0.93686	1	3477	0.34134147	-0.7262627		1.0
	ZINC34554786	-7.8	381.380	-1.4059	9	4	270.71	0.39467	134.55	-8.4237	1529.749009844	0.29548	7.2213	-4.758	0.44444	0.40473	0.93996 (max o	3	2647; 3478; 3479	0.6495431	-0.3929590		1.0
	ZINC35361433	-7.8	416.433	0.2595	10	3	313.97	0.3587	129.83	-5.3551	1529.749009844	0.26593	5.5559	0.97581	0.5	0.5261		0		0.06814182	0.95487934		1.0
	ZINC35442499	-7.8	395.522	-2.4623	8	2	312.54	0.2631	75.55	2.8266	1529.749009844	0.28493	8.2777	-8.6418	0.57143	0.50468	0.9339 (max of	8	6640; 5997; 836; 302	-0.1080529	0.5848986		1.0
	ZINC35442576	-7.8	379.523	-1.8177	7	3	297.63	0.2531	77.32	6.0627	1529.749009844	0.29548	7.6331	-6.1516	0.59259	0.49512	0.90236 (max o	5	835; 1561; 1562; 348	-0.0708944	0.56891865		1.0
	ZINC35456403	-7.8	446.403	-2.5373	12	7	299.91	0.48261	195.6	-5.4287	1529.749009844	0.25735	8.3527	-9.8591	0.48387	0.36687		0		0.45351774	-0.8859448		1.0
	ZINC35456909	-7.8	404.367	-3.0019	11	6	270.55	0.48656	175.37	-5.2776	1529.749009844	0.28493	8.8173	-10.536	0.46429	0.37407	0.96519 (max o	3	6332; 6331; 5637	-0.1806639	-0.8839977		1.0
	ZINC35459010	-7.8	388.368	-1.5759	10	4	277.62	0.42677	151.98	-7.1253	1529.749009844	0.29548	7.3913	-5.3333	0.44444	0.44832	0.96617 (max o	3	7439; 6335; 4415	0.8142112	-0.3504553		1.0
	ZINC40310039	-7.8	358.353	-0.6345	9	2	276.94	0.37163	119.39	-2.5815	1529.749009844	0.30685	6.4499	-2.0678	0.65385	0.53525	0.84311	1	1057	-0.0972019	-0.8027350		1.0
	ZINC67902341	-7.8	380.855	0.2956	7	2	256.24	0.33808	107.05	6.8307	1529.749009844	0.31912	5.5198	0.9263	0.52	0.36214		0		-0.6643117	-0.7412936		1.0
	ZINC68590555	-7.8	402.394	-1.763	10	5	276.16	0.42924	155.14	-4.6523	1529.749009844	0.28493	7.5784	-6.1875	0.46429	0.39394	0.93385 (max o	2	5267; 5266	0.71946	0.6847154		1.0
	ZINC68606315	-7.8	352.529	-6.1384	8	5	286.23	0.24617	92.44	4.9563	1529.749009844	0.31912	11.954	-19.235	0.6	0.55663	0.80946	1	4868	0.5247729	0.83439714		1.0
	ZINC68606315	-7.8	353.537	-7.5047	8	6	300.27	0.28141	93.64	4.5324	1529.749009844	0.31912	13.32	-23.517	0.6	0.56007	0.80946	1	4867	0.5018163	0.841088		1.0
	ZINC70454188	-7.8	364.436	0.5857	6	4	231.86	0.32537	107.22	-5.1582	1529.749009844	0.30685	5.2297	1.9088	0.34615	0.35342	0.8996 (max of	3	1821; 2531; 3622	0.8284166	0.33573043		1.0
	ZINC70666241	-7.8	361.464	-2.0406	7	1	285.07	0.26422	74.49	-1.3465	1529.749009844	0.30685	7.856	-6.6502	0.57692	0.47751	0.96721	1	4871	0.6969269	-0.6557300		1.0
	ZINC70666241	-7.8	361.464	-2.0406	7	1	285.07	0.26422	74.49	-1.3465	1529.749009844	0.30685	7.856	-6.6502	0.57692	0.47751	0.96721	1	4870	0.69881654	-0.6738355		1.0
	ZINC71789555	-7.8	423.381	-2.9028	12	2	292.92	0.54564	201.89	13.432	1529.749009844	0.2751	8.7182	-10.552	0.51724	0.442	0.93101 (max o	2	4029; 4496	-0.2848767	0.7995012		1.0
	ZINC79194737	-7.8	444.479	-3.7369	10	6	311.41	0.46119	175.65	-1.089	1529.749009844	0.26593	9.5523	-14.052	0.43333	0.43854	0.97533 (max o	5	1346; 1628; 1629; 27	0.7422588	-0.3823527		1.0
	ZINC05957367	-7.8	444.487	-5.6526	13	6	341.12	0.48165	213.27	1.0103	1529.749009844	0.25735	11.468	-21.964	0.6129	0.61605	0.95401	1	5789	0.9086755	-0.2846248		0.0
	ZINC49600150	-7.8	369.476	0.8703	6	4	297.04	0.26838	121.05	1.0099	1529.749009844	0.30685	4.9451	2.8363	0.57692	0.68415	0.97791 (max o	3	6356; 6357; 6039	0.32035983	-0.7638082		0.0
	ZINC67913529	-7.8	429.576	0.7542	7	2	335.06	0.31466	148.83	-1.2855	1529.749009844	0.2751	5.0612	2.7415	0.44828	0.55891	0.97176 (max o	3	7571; 6675; 5673	0.75437427	0.28042004		0.0
	ZINC02126076	-7.7	364.397	-1.5043	7	1	271	0.37768	130.06	-3.0392	1816.254130103	0.31503	7.2451	-4.7751	0.56	0.42964	0.88696 (max o	3	552; 4012; 4023	0.18987232	0.2058964		172.0
	ZINC02140440	-7.7	389.383	-1.9098	9	2	300.74	0.36364	133.86	-6.9366	1816.254130103	0.28128	7.6506	-6.7898	0.67857	0.56624	0.97837 (max o	5	5301; 868; 3577; 400	0.2766794	-0.0489142		172.0
	ZINC02522646	-7.7	376.455	-6.5458	8	4	290.66	0.34749	139.78	-6.9405	1816.254130103	0.29169	12.287	-22.441	0.62963	0.51114	0.96064 (max o	4	5410; 2459; 2825; 45	-0.6582815	-0.6554072		172.0
	ZINC02122729	-7.7	403.410	-1.7527	9	2	305.69	0.35775	133.86	4.8467	1816.254130103	0.27158	7.4935	-6.4538	0.58621	0.60298	0.96635 (max o	11	6385; 6073; 5698; 53	0.06416347	0.01771732		172.0
	ZINC02123855	-7.7	376.384	-0.9064	8	1	284.98	0.33287	113.99	3.9973	1816.254130103	0.29169	6.6472	-3.1074	0.51852	0.52539	0.95372 (max o	6	6924; 5749; 5701; 27	0.02001466	0.07518532		172.0
	ZINC08790236	-7.7	447.462	-1.3683	10	2	341.71	0.3493	143.09	4.122	1816.254130103	0.24612	7.1091	-5.5596	0.53125	0.57089	0.91243 (max o	2	6076; 3566	0.07031517	0.08351885		172.0
	ZINC01875566	-7.7	399.471	-2.4614	10	5	282.13	0.41626	187.42	-4.3873	1816.254130103	0.29169	8.2022	-8.4383	0.62963	0.59645	0.96343 (max o	4	6515; 6195; 1202; 39	0.60289943	-0.2885820		134.0
	ZINC04024245	-7.7	365.389	-2.6737	10	5	271.8	0.4117	171.2	-9.9373	1816.254130103	0.30291	8.4145	-8.8266	0.69231	0.61474	0.96519 (max o	5	5797; 5743; 5357; 25	-0.0900277	-0.4590797		134.0
	ZINC38573629	-7.7	413.900	-1.3058	8	4	313.26	0.33167	146.2	1.8294	1816.254130103	0.28128	7.0466	-4.6424	0.46429	0.57055	0.98866 (max o	4	6196; 6025; 5469; 54	0.6815944	0.3442267		134.0
	ZINC01579829	-7.7	350.326	-4.3113	9	2	270.78	0.41968	147.69	-11.164	1816.254130103	0.31503	10.052	-13.685	0.64	0.56333	0.92721 (max o	5	7323; 7189; 6779; 60	-0.0716747	-0.4704631		134.0
	ZINC31168608	-7.7	350.278	-4.1042	9	1	263.19	0.43246	153.09	-6.0092	1816.254130103	0.31503	9.845	-13.028	0.6	0.53405	0.97396 (max o	7	7897; 7798; 7644; 72	0.8381537	0.10058313		134.0
	ZINC08790050	-7.7	441.503	-2.6623	11	4	341.19	0.40778	179.75	-54.702	1816.254130103	0.25406	8.4031	-10.479	0.6129	0.62511	0.90529 (max o	3	5466; 2868; 4083	0.5375967	-0.1780342		127.0
	ZINC04089842	-7.7	355.477	-1.3821	7	3	284.27	0.31941	125.97	2.3079	1816.254130103	0.31503	7.1229	-4.3872	0.6	0.5902	0.95 (max of 6)	6	6521; 6426; 6127; 61	0.50982803	0.06794168		127.0
	ZINC13508220	-7.7	415.052	-19.03	15	4	239.36	0.85587	304.55	-31.825	1816.254130103	0.32816	24.771	-57.991	0.41667	0.55758	0.97812 (max o	24	7929; 7883; 7884; 78	0.31757092	0.7424507		127.0
	ZINC13508220	-7.7	415.052	-19.03	15	4	239.36	0.85587	304.55	-31.825	1816.254130103	0.32816	24.771	-57.991	0.41667	0.55758	0.97812 (max o	25	7929; 7885; 7883; 78	0.3080697	0.7585513		127.0
	ZINC13508220	-7.7	415.052	-19.03	15	4	239.36	0.85587	304.55	-31.825	1816.254130103	0.32816	24.771	-57.991	0.41667	0.54974	0.95709 (max o	21	7930; 7929; 7885; 78	0.35988218	0.74323577		127.0
	ZINC13508220	-7.7	414.044	-20.278	15	3	240.54	0.85657	307.38	-20.593	1816.254130103	0.32816	26.018	-61.793	0.41667	0.58345	0.95709 (max o	10	7928; 7882; 7833; 76	0.38689208	0.8391045		127.0

Structure	Molecule Name	affinity	Total Molweight	cLogP	H-Acceptors	H-Donors	Total Surface Area	Relative PSA	Polar Surface Area	Druglikeness	Kd (nM) @ 20°C	LE from K...	LLE from Kd ...	LELP from Kd (n...	Shape Index	Molecular...	Neighbor Si...	Neig...	Neighbor	Neighbor ...	Neighbor ...	Ring Systems	frequency
	ZINC04046324	-7.7	393.431	-0.2167	10	5	289.09	0.41489	154.78	-57.489	1816.254130103	0.29169	5.9575	-0.7429	0.51852	0.60755		0		-0.1261597	-0.9784229		120.0
	ZINC13509104	-7.7	384.416	-3.7275	11	4	265.34	0.56735	212.38	-0.44329	1816.254130103	0.30291	9.4683	-12.306	0.57692	0.48231	0.93686 (max c	3	1712; 2959; 5095	0.58860373	0.21827048		67.0
	ZINC03875160	-7.7	351.362	-0.6727	10	5	254.95	0.44573	145.78	-0.9947	1816.254130103	0.31503	6.4135	-2.1354	0.6	0.37878	0.93007 (max c	6	2456; 3673; 4109; 43	-0.4565417	-0.6157319		67.0
	ZINC03871404	-7.7	423.170	-13.388	15	2	266.13	0.71134	263.54	-22.95	1816.254130103	0.29169	19.129	-45.898	0.44444	0.42253	0.9578 (max of	9	6403; 6098; 6094; 57	0.66761124	0.4998144		67.0
	ZINC03871404	-7.7	424.178	-12.14	15	3	264.95	0.71006	260.71	-32.906	1816.254130103	0.29169	17.881	-41.62	0.44444	0.40459	0.9578 (max of	9	6404; 6099; 6095; 57	0.64716715	0.53879356		67.0
	ZINC08616445	-7.7	393.355	-0.2858	12	1	282.15	0.47553	157.75	1.9052	1816.254130103	0.28128	6.0266	-1.0161	0.42857	0.30182	0.94573 (max c	5	6179; 5819; 5767; 54	-0.6588815	0.35668		67.0
	ZINC14414164	-7.7	351.318	-0.7704	11	2	248.49	0.49994	151.68	1.3398	1816.254130103	0.31503	6.5112	-2.4455	0.44	0.27264	0.93348 (max c	6	7478; 6536; 5879; 49	-0.6096823	0.29775736		67.0
	ZINC14414154	-7.7	351.318	-0.7704	11	2	248.49	0.49994	151.68	1.3398	1816.254130103	0.31503	6.5112	-2.4455	0.44	0.2717	0.9402 (max of	8	7478; 6965; 6536; 58	-0.6392273	0.31809795		67.0
	ZINC06623781	-7.7	369.369	-0.8638	9	4	268.81	0.39746	134.55	-2.7155	1816.254130103	0.30291	6.6046	-2.8516	0.46154	0.32819	0.9302 (max of	3	6117; 4859; 4999	-0.8102449	-0.3854971		49.0
	ZINC06623779	-7.7	411.405	-0.4928	10	4	305.31	0.38269	143.78	-5.689	1816.254130103	0.27158	6.2336	-1.8146	0.51724	0.34432	0.93379 (max c	3	6117; 5000; 4859	-0.8388427	-0.3611152		49.0
	ZINC38557595	-7.7	442.531	-1.2338	10	3	343.37	0.33786	148.1	-55.361	1816.254130103	0.25406	6.9746	-4.8564	0.51613	0.6107	0.99016 (max c	11	6021; 6020; 6019; 58	0.44526005	-0.0914934		47.0
	ZINC12865799	-7.7	442.531	-1.2338	10	3	343.37	0.33786	148.1	-57.405	1816.254130103	0.25406	6.9746	-4.8564	0.51613	0.61534	0.99016 (max c	11	6021; 6020; 6019; 60	0.44909102	-0.1276823		47.0
	ZINC12865752	-7.7	400.450	-2.3609	10	3	304.85	0.38055	148.1	-54.914	1816.254130103	0.28128	8.1017	-8.3935	0.57143	0.58529	0.98866 (max c	16	7602; 6384; 6021; 60	0.48581576	-0.1021072		47.0
	ZINC08918145	-7.7	413.449	-3.0691	11	3	317.97	0.4007	156.97	-55.589	1816.254130103	0.27158	8.8099	-11.301	0.65517	0.61647	0.94905 (max c	2	6384; 5866	0.49147204	-0.0436184		47.0
	ZINC03869964	-7.7	361.206	-8.4657	13	4	230.21	0.69893	217.22	-28.905	1816.254130103	0.32816	14.207	-25.798	0.5	0.45267	0.92619 (max c	10	657; 1535; 1638; 493	-0.3300743	-0.3927068		44.0
	ZINC03869965	-7.7	361.206	-8.4657	13	4	230.21	0.69893	217.22	-28.905	1816.254130103	0.32816	14.207	-25.798	0.5	0.45267	0.92619 (max c	9	6101; 1041; 1638; 19	-0.3268678	-0.373297		44.0
	ZINC12889649	-7.7	432.455	-2.6663	11	4	326.3	0.40662	173.76	7.4436	1816.254130103	0.25406	8.4071	-10.495	0.48387	0.55892	0.97856 (max c	9	5166; 5165; 1808; 21	0.06163976	-0.6140576		42.0
	ZINC20758591	-7.7	391.403	-2.8277	10	4	292.62	0.40144	150.9	8.3596	1816.254130103	0.28128	8.5685	-10.053	0.46429	0.55823	0.97533 (max c	13	1009; 2163; 2246; 25	0.03772636	-0.675982		42.0
	ZINC20758572	-7.7	375.404	-1.901	9	3	284.77	0.36651	130.67	6.6384	1816.254130103	0.29169	7.6418	-6.5171	0.48148	0.54845	0.97407 (max c	13	5166; 5167; 2163; 22	0.01961704	-0.6654044		42.0
	ZINC20758829	-7.7	361.377	-2.2603	9	3	273.77	0.38123	130.67	5.1228	1816.254130103	0.30291	8.0011	-7.4619	0.5	0.55339	0.97269 (max c	4	3415; 4668; 5165; 51	0.03644934	-0.6951669		42.0
	ZINC11422512	-7.7	399.164	-11.481	13	2	252.23	0.63858	220.27	-29.713	1816.254130103	0.31503	17.222	-36.445	0.48	0.55509	0.91964 (max c	2	5041; 5042	-0.0273652	-0.9249482		41.0
	ZINC11422512	-7.7	399.164	-11.481	13	2	252.23	0.63858	220.27	-30.963	1816.254130103	0.31503	17.222	-36.445	0.48	0.55509	0.91964 (max c	2	5043; 5041	-0.0243565	-0.9055321		41.0
	ZINC11422512	-7.7	398.156	-12.729	13	1	253.41	0.64027	223.1	-19.789	1816.254130103	0.31503	18.47	-40.406	0.48	0.57299	0.90171 (max c	3	6167; 5043; 5042	-0.0071615	-0.9171964		41.0
	ZINC08790496	-7.7	440.499	-0.4294	8	1	324.64	0.3157	135.15	4.4864	1816.254130103	0.25406	6.1702	-1.6902	0.41935	0.50988	0.95549 (max c	12	1782; 2126; 2160; 25	0.7289257	-0.0453291		36.0
	ZINC04087150	-7.7	380.379	-0.9379	8	1	282.39	0.301	109.85	1.8805	1816.254130103	0.28128	6.6787	-3.3344	0.42857	0.50186	0.92486 (max c	12	5034; 1782; 2126; 21	0.7435773	-0.0308146		36.0
	ZINC20411995	-7.7	360.389	-0.7022	8	2	276.07	0.33655	118.64	5.5993	1816.254130103	0.30291	6.443	-2.3182	0.57692	0.57511	0.96721 (max c	3	2485; 2578; 4019	0.66344887	0.13994084		36.0
	ZINC20757976	-7.7	445.494	-1.0409	10	3	341.04	0.34427	147.74	7.0161	1816.254130103	0.24612	6.7817	-4.2293	0.5	0.58023	0.97319 (max c	8	5948; 2242; 4329; 46	0.3756681	0.6816541		36.0
	ZINC20758114	-7.7	376.388	-1.8191	9	3	278.4	0.38078	138.87	4.1721	1816.254130103	0.29169	7.5599	-6.2363	0.44444	0.54911	0.98806 (max c	9	6691; 6408; 6023; 59	0.27646485	0.6879717		36.0
	ZINC13509082	-7.7	384.416	-3.7275	11	4	265.34	0.56735	212.38	-0.44329	1816.254130103	0.30291	9.4683	-12.306	0.57692	0.47284	0.88538 (max c	2	5096; 2766	0.6248793	0.2180741		33.0
	ZINC03875158	-7.7	351.362	-0.6727	10	5	254.95	0.44573	145.78	-0.9947	1816.254130103	0.31503	6.4135	-2.1354	0.6	0.36445	0.92315 (max c	6	2456; 3673; 4109; 43	-0.4349402	-0.582106		33.0
	ZINC04517521	-7.7	351.318	-0.7704	11	2	248.49	0.49994	151.68	1.3398	1816.254130103	0.31503	6.5112	-2.4455	0.44	0.25022	0.9402 (max of	7	7478; 6965; 5879; 54	-0.597899	0.31743807		33.0
	ZINC04517516	-7.7	351.318	-0.7704	11	2	248.49	0.49994	151.68	1.3398	1816.254130103	0.31503	6.5112	-2.4455	0.44	0.2512	0.93348 (max c	6	7478; 6965; 5879; 51	-0.5896793	0.29696295		33.0
	ZINC12899187	-7.7	375.400	-0.6257	8	0	280.98	0.2865	99.21	-3.0843	1816.254130103	0.29169	6.3665	-2.1451	0.59259	0.4031	0.89505 (max c	3	5869; 4246; 4670	0.85438275	-0.2090026		29.0
	ZINC04200819	-7.7	435.520	-6.8926	11	6	311.47	0.47058	211.91	1.4742	1816.254130103	0.27158	12.633	-25.38	0.51724	0.54883	0.92527 (max c	11	6431; 6133; 5764; 57	-0.6050398	-0.1333737		29.0
	ZINC04221596	-7.7	421.493	-5.2677	11	6	298.98	0.46769	200.88	3.0006	1816.254130103	0.28128	11.009	-18.728	0.53571	0.54744	0.93975 (max c	10	6431; 6189; 6133; 57	-0.5735637	-0.1201955		29.0
	ZINC04000404	-7.7	374.416	-1.1213	8	2	292.32	0.31879	112.49	6.3794	1816.254130103	0.29169	6.8621	-3.8441	0.59259	0.44348	0.86639 (max c	2	3184; 3942	-0.1950861	0.52503175		28.0
	ZINC02384977	-7.7	361.425	-2.3899	9	6	263.23	0.4022	176.3	2.7776	1816.254130103	0.30291	8.1307	-7.8897	0.61538	0.54389		0		-0.9885900	-0.0126878		28.0
	ZINC09013131	-7.7	394.339	-0.8122	12	1	281.98	0.47156	147.41	4.1075	1816.254130103	0.28128	6.553	-2.8876	0.42857	0.33174	0.9339 (max of	3	6245; 4617; 4634	0.07443751	-0.7154512		26.0
	ZINC04104826	-7.7	390.383	-1.9707	10	5	264.25	0.44859	155.14	-3.9213	1816.254130103	0.29169	7.7115	-6.7561	0.48148	0.37688	0.95201 (max c	3	5385; 2396; 3217	-0.6081624	-0.5409087		25.0
	ZINC09317472	-7.7	428.452	1.7076	12	5	311.97	0.4195	167.17	2.1023	1816.254130103	0.25406	4.0332	6.7214	0.51613	0.36426	0.91736 (max c	3	212; 285; 3314	-0.1251952	-0.7198700		24.0
	ZINC12885456	-7.7	442.399	-0.8237	10	1	330.77	0.34725	132.45	0.53295	1816.254130103	0.24612	6.5645	-3.3468	0.59375	0.40554	0.92889 (max c	5	1403; 2414; 2416; 29	0.5550179	0.33876008		24.0
	ZINC03650334	-7.7	376.368	-2.0663	10	5	264.4	0.44066	155.05	6.1929	1816.254130103	0.29169	7.8071	-7.0838	0.44444	0.45962	0.97037 (max c	15	4912; 1075; 1661; 18	0.428216	0.758489		24.0
	ZINC03831423	-7.7	376.368	-2.0663	10	5	264.4	0.44066	155.05	6.1929	1816.254130103	0.29169	7.8071	-7.0838	0.44444	0.46827	0.9578 (max of	20	5875; 5314; 5297; 10	0.45598212	0.77680486		24.0

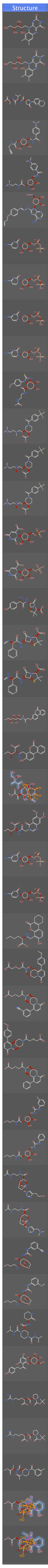

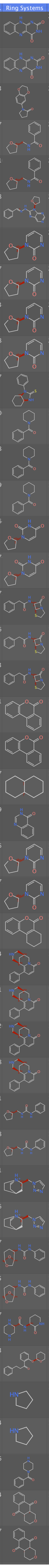

Structure	Molecule Name	affinity	Total Molweight	cLogP	H-Acceptors	H-Donors	Total Surface Area	Relative PSA	Polar Surface Area	Druglikeness	Kd (nM) @ 20°C	LE from K...	LLE from Kd ...	LELP from Kd (n...	Shape Index	Molecular...	Neighbor Si...	Neig...	Neighbor	Neighbor ...	Neighbor ...	Ring Systems	frequency
	ZINC02036848	-7.7	376.368	-2.0663	10	5	264.4	0.44066	155.05	6.1929	1816.254130103	0.29169	7.8071	-7.0838	0.44444	0.45827	0.97407 (max o	21	6010; 5875; 5314; 52	0.46233907	0.7938288		24.0
	ZINC13542423	-7.7	390.395	-1.8134	10	4	275.5	0.39419	146.26	4.701	1816.254130103	0.28128	7.5542	-6.4471	0.42857	0.45263	0.91804 (max o	2	3672; 4896	0.5022002	0.7952393		24.0
	ZINC38570076	-7.7	432.451	-1.9611	10	2	319.83	0.35303	137.1	-1.2377	1816.254130103	0.25406	7.7019	-7.7192	0.54839	0.46031	0.96971 (max o	13	6658; 6226; 1217; 17	0.52830523	0.23174743		22.0
	ZINC08627173	-7.7	375.424	-0.9259	8	4	290.79	0.3052	111.13	1.744	1816.254130103	0.29169	6.6667	-3.1742	0.66667	0.57492	0.93686 (max o	7	6476; 5021; 2111; 32	0.38913724	-0.3545687		22.0
	ZINC08627192	-7.7	409.505	-2.6196	9	5	329.6	0.32263	115.57	1.5191	1816.254130103	0.27158	8.3604	-9.6459	0.65517	0.60467	0.92464 (max o	9	6476; 5455; 3289; 32	0.36352918	-0.3874411		22.0
	ZINC08740527	-7.7	441.447	2.4164	12	5	325.45	0.42194	173.16	-4.1017	1816.254130103	0.24612	3.3244	9.8181	0.5625	0.37253	0.95267 (max o	7	6194; 5458; 3301; 33	-0.1957589	-0.6640008		20.0
	ZINC13431049	-7.7	400.152	-12.538	14	3	249.13	0.70826	249.78	-46.201	1816.254130103	0.31503	18.279	-39.799	0.56	0.61172	0.92662 (max o	9	622; 3353; 3355; 372	-0.5598461	-0.455498		20.0
	ZINC13431049	-7.7	401.160	-11.29	14	4	247.95	0.70688	246.95	-50.931	1816.254130103	0.31503	17.031	-35.839	0.56	0.61192	0.92662 (max o	10	5873; 621; 3352; 335	-0.5905251	-0.4021217		20.0
	ZINC12495027	-7.7	385.161	-10.438	13	3	242.86	0.66775	226.72	-47.338	1816.254130103	0.32816	16.179	-31.809	0.58333	0.61712	0.91522 (max o	6	3352; 3354; 5057; 37	-0.6338717	-0.4459463		20.0
	ZINC12495027	-7.7	384.153	-11.686	13	2	244.04	0.66936	229.55	-42.608	1816.254130103	0.32816	17.427	-35.611	0.58333	0.61691	0.91522 (max o	6	3353; 3355; 3767; 37	-0.6178056	-0.4867758		20.0
	ZINC08636372	-7.7	422.504	-0.7382	9	5	310.15	0.41338	155.25	5.7893	1816.254130103	0.27158	6.479	-2.7182	0.55172	0.46028	0.9339 (max of	5	7775; 7160; 7161; 69	0.43893635	0.20074347		18.0
	ZINC20462955	-7.7	443.508	-1.3863	6	1	349.05	0.18636	68.12	-0.65061	1816.254130103	0.25406	7.1271	-5.4567	0.54839	0.68462	0.92021 (max o	6	7379; 7206; 7034; 68	-0.0705130	-0.0685630		18.0
	ZINC20462955	-7.7	443.508	-1.3863	6	1	349.05	0.18636	68.12	-1.5247	1816.254130103	0.25406	7.1271	-5.4567	0.54839	0.68254	0.92021 (max o	6	7380; 7207; 7035; 68	-0.0696754	-0.0883958		18.0
	ZINC20462955	-7.7	444.516	-0.9796	6	2	363.09	0.21782	69.32	-3.0803	1816.254130103	0.25406	6.7204	-3.8558	0.54839	0.67194	0.91934 (max o	6	7381; 7208; 7036; 68	-0.0293014	-0.0862639		18.0
	ZINC03870125	-7.7	366.174	-9.7178	13	4	227.86	0.69784	218.63	-30.796	1816.254130103	0.32816	15.459	-29.613	0.5	0.57479	0.92257 (max o	5	6717; 6096; 5729; 57	-0.2403895	-0.7258776		17.0
	ZINC03870125	-7.7	365.166	-10.965	13	3	229.04	0.6994	221.46	-26.867	1816.254130103	0.32816	16.706	-33.415	0.5	0.59344	0.91891 (max o	5	6718; 6097; 5732; 57	-0.2824663	-0.7216297		17.0
	ZINC19795949	-7.7	364.401	-4.0789	8	3	246.09	0.46434	161.09	9.1306	1816.254130103	0.31503	9.8197	-12.948	0.56	0.41051	0.9654 (max of	3	3160; 3830; 4275	0.12857133	-0.1193137		17.0
	ZINC03874216	-7.7	360.413	-1.5337	7	1	254.02	0.38347	127.2	11.036	1816.254130103	0.31503	7.2745	-4.8684	0.52	0.39204	0.90896 (max o	7	6130; 5346; 2391; 25	0.11631807	-0.0814086		17.0
	ZINC03874216	-7.7	361.421	-1.5337	7	2	242.51	0.35421	128.81	10.469	1816.254130103	0.31503	7.2745	-4.8684	0.52	0.39112	0.90896 (max o	2	5345; 3177	0.03241120	-0.1083643		17.0
	ZINC36369721	-7.7	447.439	-0.5203	10	5	320.61	0.3591	156.99	5.6773	1816.254130103	0.24612	6.2611	-2.114	0.59375	0.56029	0.971 (max of 3	3	2295; 3514; 3515	0.61999995	-0.6646791		16.0
	ZINC02100721	-7.7	355.389	0.3809	6	2	263.68	0.27002	103.27	0.93543	1816.254130103	0.30291	5.3599	1.2575	0.5	0.44815	0.96438 (max o	5	6072; 5298; 4005; 44	0.7184988	0.6031185		16.0
	ZINC35271203	-7.7	426.460	0.0489	9	4	309.91	0.35985	150.59	-12.343	1816.254130103	0.26252	5.6919	0.18627	0.43333	0.53443	0.96589 (max o	5	7083; 6874; 6046; 52	0.37970236	0.31606567		16.0
	ZINC20763618	-7.7	376.388	-1.702	9	1	288.62	0.3462	120.36	6.6121	1816.254130103	0.29169	7.4428	-5.8349	0.55556	0.5537	0.96617 (max o	4	6277; 3422; 3466; 38	0.01897246	-0.3468019		15.0
	ZINC13431048	-7.7	400.152	-12.538	14	3	249.13	0.70826	249.78	-46.201	1816.254130103	0.31503	18.279	-39.799	0.56	0.6121	0.91964 (max o	9	622; 3353; 3355; 372	-0.5783214	-0.4406686		14.0
	ZINC13431048	-7.7	401.160	-11.29	14	4	247.95	0.70688	246.95	-50.931	1816.254130103	0.31503	17.031	-35.839	0.56	0.6123	0.91964 (max o	10	6228; 5088; 5087; 62	-0.6103775	-0.3957057		14.0
	ZINC01891581	-7.7	359.421	0.0341	6	2	271.14	0.26259	103.27	-9.0811	1816.254130103	0.30291	5.7067	0.11257	0.53846	0.48974	0.95257 (max o	3	6070; 4955; 4585	-0.0365495	0.7298463		14.0
	ZINC04237476	-7.7	427.500	-1.0185	7	0	310.87	0.28304	115.28	1.609	1816.254130103	0.26252	6.7593	-3.8796	0.56667	0.48353	0.97304 (max o	8	5778; 5392; 5110; 49	-0.4753594	-0.2741244		14.0
	ZINC05397271	-7.7	406.417	-1.6601	8	0	302.83	0.27758	113.77	-2.8319	1816.254130103	0.26252	7.4009	-6.3236	0.53333	0.46566	0.96832 (max o	7	5392; 5110; 2464; 41	-0.4979546	-0.2978651		14.0
	ZINC04237368	-7.7	427.500	-0.827	7	0	310.87	0.28304	115.28	3.5218	1816.254130103	0.26252	6.5678	-3.1502	0.56667	0.52676	0.96832 (max o	7	5778; 5777; 5392; 49	-0.4807535	-0.3135592		14.0
	ZINC15672337	-7.7	381.407	-1.4957	7	0	281.12	0.25078	89.98	1.4481	1816.254130103	0.28128	7.2365	-5.3176	0.53571	0.46463	0.95174 (max o	5	5393; 5392; 4575; 49	-0.4936706	-0.2554668		14.0
	ZINC08643589	-7.7	385.391	-0.8473	9	5	285.99	0.37295	129.15	-0.97514	1816.254130103	0.29169	6.5881	-2.9048	0.66667	0.58854	0.93686 (max o	10	5821; 1211; 1462; 21	0.30584672	-0.4322611		14.0
	ZINC08624491	-7.7	367.400	-0.9481	9	5	279.64	0.38142	129.15	0.36486	1816.254130103	0.30291	6.6889	-3.1299	0.69231	0.58435	0.94002 (max o	5	5024; 1210; 2501; 37	0.27471346	-0.4478348		14.0
	ZINC08299031	-7.7	365.456	-3.6855	9	4	295.69	0.36694	113.58	0.28989	1816.254130103	0.30291	9.4263	-12.167	0.69231	0.5778	0.96721 (max o	6	5816; 5814; 5812; 54	0.07094093	-0.2292781		14.0
	ZINC08299052	-7.7	379.507	-5.2617	9	4	324.44	0.34826	97.79	1.9232	1816.254130103	0.29169	11.003	-18.038	0.66667	0.58029	0.96885 (max o	7	7292; 6175; 6174; 58	0.09485174	-0.1936386		14.0
	ZINC12483833	-7.7	362.385	-0.8296	9	4	272.49	0.38196	124.87	-2.8424	1816.254130103	0.30291	6.5704	-2.7387	0.61538	0.48405	0.92032 (max o	4	7498; 6215; 2146; 50	-0.6938203	-0.3021867		14.0
	ZINC12483852	-7.7	367.400	-0.7352	9	4	273.04	0.36815	110.31	1.4376	1816.254130103	0.30291	6.476	-2.4271	0.61538	0.497	0.95257 (max o	4	7498; 6215; 4214; 50	-0.6741422	-0.2969105		14.0
	ZINC08299453	-7.7	396.490	-0.2705	10	4	305.54	0.34398	122.88	6.2676	1816.254130103	0.28128	6.0113	-0.96169	0.46429	0.58655	0.9083 (max of	6	5815; 5803; 5422; 15	-0.1329621	0.30633527		13.0
	ZINC13424697	-7.7	436.412	0.055	10	7	305.5	0.40831	177.14	-4.8731	1816.254130103	0.25406	5.6858	0.21649	0.51613	0.43255	0.95771 (max o	11	887; 2018; 2058; 255	0.8016216	-0.2588933		13.0
	ZINC04027397	-7.7	371.436	-1.9697	10	4	270.83	0.38397	162.41	-30.487	1816.254130103	0.30291	7.7105	-6.5025	0.57692	0.6005	0.95857	1	4954	-0.2028608	-0.44573		13.0
	ZINC04027631	-7.7	371.436	-1.9697	10	4	270.83	0.38397	162.41	-30.487	1816.254130103	0.30291	7.7105	-6.5025	0.57692	0.60392	0.95857 (max o	2	6945; 4953	-0.1829394	-0.4376164		13.0
	ZINC05398424	-7.7	379.387	0.5201	8	1	282.23	0.28601	96.02	0.21194	1816.254130103	0.29169	5.2207	1.783	0.51852	0.5714	0.93686 (max o	4	7156; 6974; 2088; 36	0.00372548	0.34186736		13.0
	ZINC72320136	-7.7	350.322	-0.2569	8	3	236.42	0.41629	130.36	-2.1313	1816.254130103	0.31503	5.9977	-0.81548	0.48	0.40379	0.97668 (max o	10	6679; 6058; 6057; 26	0.7396056	-0.0926694		12.0
	ZINC72319950	-7.7	364.349	0.171	8	2	252.33	0.37776	119.36	-2.1253	1816.254130103	0.30291	5.5698	0.56452	0.46154	0.40419	0.95559 (max o	8	6679; 6058; 6057; 39	0.79605	-0.0874917		12.0

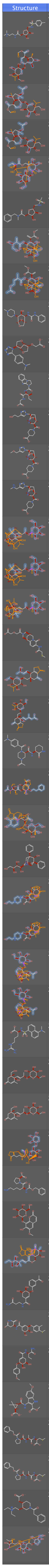

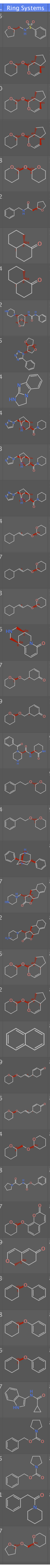

Structure	Molecule Name	affinity	Total Molweight	cLogP	H-Acceptors	H-Donors	Total Surface Area	Relative PSA	Polar Surface Area	Druglikeness	Kd (nM) @ 20°C	LE from K...	LLE from Kd ...	LELP from Kd (n...	Shape Index	Molecular...	Neighbor Si...	Neig...	Neighbor	Neighbor ...	Neighbor ...	Ring Systems	frequency
	ZINC12603856	-7.7	416.517	-2.4855	9	5	317.87	0.37226	137.78	-4.0368	1816.254130103	0.28128	8.2263	-8.8365	0.64286	0.58969	0.97773 (max o	5	6187; 5830; 5482; 33	0.26511368	-0.2965045		12.0
	ZINC36377990	-7.7	390.383	-1.9707	10	5	264.25	0.44859	155.14	-3.9213	1816.254130103	0.29169	7.7115	-6.7561	0.48148	0.36873	0.94605 (max o	5	3517; 4115; 4144; 46	-0.1849837	0.4438431		12.0
	ZINC35456539	-7.7	448.419	-2.3095	12	5	302.17	0.46854	181.44	-5.7219	1816.254130103	0.25406	8.0503	-9.0905	0.41935	0.38595	0.95521 (max o	3	6329; 3928; 5232	-0.6052628	-0.7182590		12.0
	ZINC35456535	-7.7	448.419	-2.3095	12	5	302.17	0.46854	181.44	-5.7219	1816.254130103	0.25406	8.0503	-9.0905	0.41935	0.38595	0.95521 (max o	3	6329; 3928; 5233	-0.6407733	-0.71		12.0
	ZINC77257634	-7.7	424.397	-2.7103	12	7	290.45	0.49833	195.6	-5.5159	1816.254130103	0.27158	8.4511	-9.9799	0.51724	0.49589	0.95908 (max o	3	3552; 3553; 4456	0.39959022	-0.7719338		12.0
	ZINC12603586	-7.7	372.441	-0.7287	8	3	266.35	0.34853	124.55	5.4188	1816.254130103	0.31503	6.4695	-2.3131	0.6	0.62528	0.87324 (max o	3	5454; 3285; 4654	0.49133852	-0.4191042		12.0
	ZINC35271315	-7.7	412.433	0.2102	9	4	295.62	0.37724	150.59	-8.6504	1816.254130103	0.27158	5.5306	0.774	0.41379	0.52952	0.96682 (max o	7	7105; 7084; 6875; 60	0.41825762	0.3099863		12.0
	ZINC31170278	-7.7	438.471	1.6499	9	2	324.65	0.33377	136.43	-5.8488	1816.254130103	0.25406	4.0909	6.4942	0.45161	0.53187	0.96971 (max o	8	6876; 6869; 6626; 59	0.37621257	0.25093794		12.0
	ZINC22922179	-7.7	362.428	-0.2278	8	3	263.94	0.28855	86.3	6.6218	1816.254130103	0.30291	5.9686	-0.75203	0.57692	0.45217	0.93686 (max o	7	6504; 1552; 3428; 38	-0.6262030	-0.2454662		10.0
	ZINC08253595	-7.7	374.376	-2.5071	10	0	272.88	0.36884	114.66	1.6341	1816.254130103	0.29169	8.2479	-8.595	0.59259	0.35684	0.86023 (max o	3	2426; 4146; 4493	-0.2139379	-0.3720105		10.0
	ZINC06624169	-7.7	372.404	-0.5669	9	2	280.88	0.34787	119.39	5.608	1816.254130103	0.29169	6.3077	-1.9435	0.51852	0.48258	0.97407 (max o	4	6469; 1185; 3711; 42	0.6020013	-0.2676394		10.0
	ZINC08299701	-7.7	391.470	-3.5442	9	2	304.86	0.3392	115.82	0.0027639	1816.254130103	0.28128	9.285	-12.6	0.64286	0.54334	0.98451 (max o	7	5014; 2865; 4154; 41	0.17205122	0.24459504		10.0
	ZINC08299768	-7.7	391.470	-3.5464	9	1	307.01	0.32673	104.82	1.5368	1816.254130103	0.28128	9.2872	-12.608	0.64286	0.53594	0.98451 (max o	7	5439; 2865; 4161; 41	0.18183108	0.22444333		10.0
	ZINC08299762	-7.7	391.494	-4.1803	9	2	304.75	0.32174	112.2	1.7218	1816.254130103	0.28128	9.9211	-14.862	0.64286	0.53531	0.97037 (max o	5	5446; 5014; 3725; 41	0.1831295	0.2602867		10.0
	ZINC35454673	-7.7	390.471	-0.232	8	6	282.24	0.34935	139.84	-2.1613	1816.254130103	0.29169	5.9728	-0.79536	0.51852	0.45759	0.97149 (max o	8	5628; 5497; 5276; 30	-0.3271971	0.22916184		10.0
	ZINC35454669	-7.7	390.471	-0.232	8	6	282.24	0.34935	139.84	-2.1613	1816.254130103	0.29169	5.9728	-0.79536	0.51852	0.45759	0.97149 (max o	8	5628; 5497; 5276; 52	-0.3423646	0.21413627		10.0
	ZINC72319867	-7.7	406.470	-1.0554	9	7	286.5	0.38988	160.07	-1.8323	1816.254130103	0.28128	6.7962	-3.7522	0.5	0.56656	0.97288 (max o	7	5680; 5628; 3067; 48	-0.3441284	0.23611833		10.0
	ZINC04259162	-7.7	408.454	-3.0911	9	1	294.08	0.38251	144.38	4.6786	1816.254130103	0.28128	8.8319	-10.99	0.64286	0.49754	0.96519 (max o	3	5884; 265; 903	-0.4730484	-0.4911366		10.0
	ZINC35456133	-7.7	386.439	-0.2961	8	5	283.18	0.34798	136.68	-4.6035	1816.254130103	0.29169	6.0369	-1.0151	0.48148	0.53353	0.96885 (max o	5	5969; 5970; 5603; 39	-0.0741429	0.94602567		10.0
	ZINC35465976	-7.7	441.451	-2.6414	10	5	318.58	0.39507	176.81	-2.3234	1816.254130103	0.25406	8.3822	-10.397	0.48387	0.44414	0.97642 (max o	3	6339; 4840; 4841	0.19759306	-0.7474109		10.0
	ZINC05397309	-7.7	415.492	0.0805	9	2	315.42	0.2718	102.06	5.8895	1816.254130103	0.26252	5.6603	0.30664	0.5	0.55278	0.94488 (max o	3	5799; 2931; 4181	0.10422611	0.3004957		9.0
	ZINC72320405	-7.7	427.424	-2.4904	10	4	312.16	0.39326	165.81	1.3048	1816.254130103	0.26252	8.2312	-9.4864	0.56667	0.56302	0.99855 (max o	3	1948; 3546; 3974	-0.2094223	0.7009116		9.0
	ZINC31169345	-7.7	359.306	-4.0291	10	6	245.66	0.51258	179.97	-2.9939	1816.254130103	0.31503	9.7699	-12.79	0.52	0.4848	0.96243	1	3915	0.7696237	-0.6012994		9.0
	ZINC19331507	-7.7	427.519	-2.0628	7	6	311.03	0.27856	108.83	2.5847	1816.254130103	0.25406	7.8036	-8.1195	0.41935	0.48045	0.97423 (max o	5	5902; 1345; 1590; 38	0.88751256	0.1080968		8.0
	ZINC22457076	-7.7	379.519	-1.4507	6	3	294.33	0.23912	74.44	2.1596	1816.254130103	0.29169	7.1915	-4.9734	0.55556	0.46431	0.95123 (max o	7	1537; 4277; 4286; 47	-0.0488113	0.04922137		8.0
	ZINC22457076	-7.7	379.519	-1.4507	6	3	294.33	0.23912	74.44	2.0856	1816.254130103	0.29169	7.1915	-4.9734	0.55556	0.49449	0.94605 (max o	7	1538; 4278; 4287; 47	-0.0479177	0.09287092		8.0
	ZINC08952339	-7.7	406.382	-2.7247	11	6	268.21	0.49081	175.37	-4.1099	1816.254130103	0.28128	8.4655	-9.6869	0.42857	0.38767	0.93686 (max o	2	3197; 5037	0.8073451	-0.0053525		8.0
	ZINC08952338	-7.7	406.382	-2.7247	11	6	268.21	0.49081	175.37	-4.1099	1816.254130103	0.28128	8.4655	-9.6869	0.42857	0.38767	0.93686	1	5038	0.8188449	-0.0234282		8.0
	ZINC02575474	-7.7	407.493	-1.1575	9	4	285.32	0.33818	161.56	-2.3353	1816.254130103	0.28128	6.8983	-4.1152	0.53571	0.52457		0		0.7812858	0.6226218		8.0
	ZINC31168193	-7.7	402.438	-1.1195	9	6	287.44	0.38839	156.91	-9.0196	1816.254130103	0.28128	6.8603	-3.9801	0.5	0.47435	0.97533 (max o	6	5641; 5594; 3047; 43	0.6513002	-0.1828499		8.0
	ZINC31168182	-7.7	402.438	-1.1195	9	6	287.44	0.38839	156.91	-9.0196	1816.254130103	0.28128	6.8603	-3.9801	0.5	0.4598	0.97773 (max o	7	5641; 5594; 3047; 43	0.64793205	-0.1599326		8.0
	ZINC35454845	-7.7	422.469	-0.5759	10	6	308.96	0.38387	158.3	-18.912	1816.254130103	0.27158	6.3167	-2.1206	0.58621	0.52858	0.97415 (max o	5	5630; 3492; 4407; 44	-0.4472117	0.72360474		8.0
	ZINC13544334	-7.7	377.439	-0.7398	8	2	295.11	0.31476	126.41	-10.487	1816.254130103	0.29169	6.4806	-2.5362	0.51852	0.51217	0.85572 (max o	2	6418; 5359	-0.7608795	0.36288288		8.0
	ZINC04027246	-7.7	370.309	-1.2533	10	5	250.78	0.47269	155.14	-5.8493	1816.254130103	0.30291	6.9941	-4.1375	0.42308	0.41647	0.9615 (max of	11	6630; 6430; 5408; 49	-0.3804958	0.51531047		8.0
	ZINC35455854	-7.7	352.425	1.0294	6	4	262.95	0.2869	107.22	4.8399	1816.254130103	0.31503	4.7114	3.2676	0.56	0.44603	0.97118 (max o	5	6880; 5999; 3897; 47	-0.7339433	-0.1644419		8.0
	ZINC03838511	-7.7	362.381	-0.6589	8	4	271.44	0.37596	138.95	4.8888	1816.254130103	0.30291	6.3997	-2.1752	0.57692	0.52464	0.9066	1	4031	0.26001993	-0.8724013		8.0
	ZINC15675336	-7.7	423.465	-2.702	9	4	307.46	0.43524	187.31	6.5831	1816.254130103	0.27158	8.4428	-9.9493	0.55172	0.51723	0.88922 (max o	2	6242; 5718	0.7840299	-0.1732558		8.0
	ZINC03840231	-7.7	364.397	-0.2408	8	4	274.69	0.36044	131.11	6.6315	1816.254130103	0.30291	5.9816	-0.79495	0.57692	0.49528	0.83203	1	6242	0.80795294	-0.2238196		8.0
	ZINC70666304	-7.7	359.357	-0.8644	8	2	260.62	0.37929	120.55	0.018054	1816.254130103	0.30291	6.6052	-2.8536	0.5	0.32755	0.93361 (max o	4	7109; 6908; 4440; 48	0.46019545	0.40830737		8.0
	ZINC12660220	-7.7	436.507	-1.659	9	2	329.82	0.36505	153.17	2.1105	1816.254130103	0.26252	7.3998	-6.3194	0.6	0.54677	0.9707 (max of	3	5488; 4660; 5068	-0.7021366	0.6508202		8.0
	ZINC12660217	-7.7	436.507	-1.659	9	2	329.82	0.36505	153.17	2.1105	1816.254130103	0.26252	7.3998	-6.3194	0.6	0.54868	0.9707 (max of	3	5488; 4660; 5069	-0.7158028	0.65823275		8.0
	ZINC12480640	-7.7	360.496	-0.0098995	5	1	310.79	0.19788	64.88	3.14	1816.254130103	0.30291	5.7507	-0.032681	0.53846	0.65655	0.94951 (max o	4	7719; 6992; 6853; 51	-0.0618601	-0.1762341		8.0
	ZINC77257446	-7.7	442.456	-3.1267	12	8	305.2	0.47444	198.76	-4.7867	1816.254130103	0.26252	8.8675	-11.91	0.53333	0.5664	0.98188 (max o	6	5695; 1290; 2639; 26	-0.6518737	0.49053517		7.0

Structure	Molecule Name	affinity	Total Molweight	cLogP	H-Acceptors	H-Donors	Total Surface Area	Relative PSA	Polar Surface Area	Druglikeness	Kd (nM) @ 20°C	LE from K...	LLE from Kd ...	LELP from Kd (n...	Shape Index	Molecular...	Neighbor Si...	Neig...	Neighbor	Neighbor ...	Neighbor ...	Ring Systems	frequency
	ZINC12530147	-7.7	372.380	-3.6807	9	3	270.72	0.34131	128.7	4.1953	1816.254130103	0.29169	9.4215	-12.618	0.55556	0.51601	0.95228 (max o	6	300; 1835; 4216; 421	0.27321896	-0.1684765		7.0
	ZINC12530147	-7.7	371.372	-3.4918	9	2	281.69	0.36696	127.45	4.7671	1816.254130103	0.29169	9.2326	-11.971	0.55556	0.51601	0.95228 (max o	7	301; 1837; 4217; 421	0.2852683	-0.2130009		7.0
	ZINC04084993	-7.7	375.467	-1.2856	7	3	296.01	0.30675	125.97	2.8948	1816.254130103	0.29169	7.0264	-4.4074	0.62963	0.53126	0.92464 (max o	6	6991; 6475; 6427; 27	0.543357	-0.0591825		7.0
	ZINC20758853	-7.7	387.415	-1.7388	9	2	285.37	0.33802	121.88	7.361	1816.254130103	0.28128	7.4796	-6.1818	0.46429	0.51645	0.95401 (max o	3	4527; 4667; 4763	-0.7315194	0.03552787		7.0
	ZINC12483897	-7.7	444.525	-0.0027997	7	2	320.88	0.18057	71.01	3.2627	1816.254130103	0.24612	5.7436	-0.011376	0.5625	0.45643	0.9499 (max of	5	6255; 5853; 5538; 47	0.33866048	-0.0519182		6.0
	ZINC12663491	-7.7	412.425	-0.898	10	1	292.17	0.27737	99.63	-2.1646	1816.254130103	0.26252	6.6388	-3.4206	0.46667	0.37029	0.96385 (max o	3	5355; 4062; 4662	0.5470895	0.6827206		6.0
	ZINC34235758	-7.7	388.324	-6.5696	11	4	269.23	0.50901	188.87	-5.5881	1816.254130103	0.29169	12.31	-22.522	0.44444	0.48967	0.96343 (max o	3	6632; 5621; 4389	-0.7826431	0.56215155		6.0
	ZINC31159273	-7.7	360.314	-1.1781	10	5	250.04	0.47408	155.14	-4.135	1816.254130103	0.31503	6.9189	-3.7397	0.52	0.41839	0.95318 (max o	6	6788; 6001; 5634; 56	-0.6970165	-0.1456707		6.0
	ZINC03831540	-7.7	382.416	-3.6789	8	1	252.44	0.52916	183.21	11.228	1816.254130103	0.31503	9.4197	-11.678	0.48	0.33609	0.98188 (max o	6	7143; 6978; 6961; 53	-0.3703646	-0.4670221		6.0
	ZINC20465279	-7.7	383.438	-2.0798	7	4	296.18	0.2981	92.46	-0.083214	1816.254130103	0.29169	7.8206	-7.1301	0.51852	0.49113	0.8541	1	6582	0.6872432	0.43890414		6.0
	ZINC31167407	-7.7	417.456	-1.3541	8	0	322.92	0.28252	119.03	4.2354	1816.254130103	0.26252	7.0949	-5.158	0.43333	0.51425	0.96092 (max o	5	7245; 6863; 6616; 66	-0.025668	0.8774951		6.0
	ZINC35459270	-7.7	388.368	-2.1091	10	5	265.99	0.44566	155.14	-3.1573	1816.254130103	0.29169	7.8499	-7.2305	0.48148	0.40236	0.94302 (max o	3	6787; 3791; 4377	-0.904039	0.12460632		6.0
	ZINC22922179	-7.7	363.436	-2.3375	8	4	277.98	0.32448	87.5	2.4504	1816.254130103	0.30291	8.0783	-7.7168	0.57692	0.45217	0.93686 (max o	6	6809; 1553; 3429; 38	-0.6764276	-0.2193733		5.0
	ZINC03838834	-7.7	449.526	-4.6516	10	6	338.89	0.37767	178.62	3.8088	1816.254130103	0.24612	10.392	-18.9	0.5625	0.54029	0.92326	1	4485	-0.5142243	-0.2652992		5.0
	ZINC20464879	-7.7	411.453	-0.8649	10	4	307.01	0.34432	121.75	3.1071	1816.254130103	0.27158	6.6057	-3.1847	0.51724	0.57008	0.93111 (max o	5	6571; 5549; 2995; 43	-0.3416693	-0.0643679		5.0
	ZINC04215237	-7.7	396.427	-2.1413	10	2	268.68	0.57321	203.58	14.606	1816.254130103	0.30291	7.8821	-7.069	0.5	0.37809	0.93058 (max o	4	5321; 5322; 2769; 40	-0.3560829	0.28801528		5.0
	ZINC08643636	-7.7	403.472	-0.3712	6	3	301.66	0.20066	86.61	1.2054	1816.254130103	0.27158	6.112	-1.3668	0.65517	0.5273	0.93395 (max o	5	6756; 1193; 2926; 41	0.12597625	-0.5312676		5.0
	ZINC20504120	-7.7	397.493	-1.5529	7	5	299.08	0.36362	123.69	3.2634	1816.254130103	0.29169	7.2937	-5.3237	0.59259	0.41629	0.97658 (max o	7	6273; 5944; 5575; 55	0.46060324	0.20162573		5.0
	ZINC08299280	-7.7	356.472	-3.1679	7	3	286.35	0.27463	80.86	0.25153	1816.254130103	0.30291	8.9087	-10.458	0.65385	0.47862	0.96721 (max o	5	5447; 1759; 3714; 37	-0.1461467	-0.0172085		5.0
	ZINC20465143	-7.7	415.509	-0.9928	9	3	296.68	0.31462	119.95	8.1202	1816.254130103	0.28128	6.7336	-3.5296	0.57143	0.54193	0.93379 (max o	6	5563; 5551; 3846; 43	-0.4259157	-0.0935312		5.0
	ZINC20465143	-7.7	416.517	-3.1025	9	4	310.72	0.34558	121.15	4.3076	1816.254130103	0.28128	8.8433	-11.03	0.57143	0.54193	0.93379 (max o	6	5564; 5551; 3847; 43	-0.3888119	-0.1061559		5.0
	ZINC08298304	-7.7	370.499	-3.043	7	3	315.21	0.28073	68.69	0.15824	1816.254130103	0.29169	8.7838	-10.432	0.62963	0.49308	0.96885 (max o	5	5447; 5441; 3718; 41	-0.1062780	-0.0273753		5.0
	ZINC04534181	-7.7	374.186	0.0892	7	5	224.22	0.38529	115.17	-5.5938	1816.254130103	0.35799	5.6516	0.24917	0.5	0.24832	0.92913 (max o	5	6154; 6155; 5406; 54	-0.4662638	0.6009723		5.0
	ZINC30879668	-7.7	401.481	-0.706	7	4	321.57	0.27456	92.46	2.0802	1816.254130103	0.27158	6.4468	-2.5996	0.58621	0.54578	0.92232 (max o	4	7411; 7070; 1543; 47	0.42617863	-0.3021174		5.0
	ZINC05890339	-7.7	394.375	-0.4815	9	5	272.76	0.40908	153.75	-2.8773	1816.254130103	0.28128	6.2223	-1.7118	0.42857	0.44532	0.94573 (max o	6	5809; 5760; 5496; 54	-0.7859815	0.41875514		5.0
	ZINC13533924	-7.7	397.375	-4.0196	12	7	270.85	0.52834	187.4	-6.2954	1816.254130103	0.29169	9.7604	-13.78	0.40741	0.4674	0.94622 (max o	6	7010; 6958; 6720; 53	0.24583532	0.7191218		5.0
	ZINC04097367	-7.7	383.349	-4.4475	12	8	254.94	0.57347	198.4	-4.7202	1816.254130103	0.30291	10.188	-14.682	0.42308	0.46983	0.94951 (max o	6	7010; 6958; 6720; 53	0.23623359	0.75362164		5.0
	ZINC12659247	-7.7	427.409	0.2644	12	0	314.69	0.39343	141.11	4.2655	1816.254130103	0.26252	5.4764	1.0071	0.46667	0.49641	0.95053 (max o	3	6999; 6516; 6486	0.471542	-0.4246841		5.0
	ZINC31159269	-7.7	360.314	-1.1781	10	5	250.04	0.47408	155.14	-4.135	1816.254130103	0.31503	6.9189	-3.7397	0.52	0.40974	0.95 (max of 7)	7	6788; 6001; 5882; 56	-0.7140443	-0.1324279		5.0
	ZINC36369857	-7.7	379.213	-1.0197	7	3	234.08	0.29631	102.4	0.1157	1816.254130103	0.34242	6.7605	-2.9779	0.52174	0.50623	0.92193 (max o	2	5245; 4418	-0.9564351	-0.0950460		5.0
	ZINC49600305	-7.7	363.341	-1.4804	8	3	270.63	0.35702	128.51	-0.3333	1816.254130103	0.30291	7.2212	-4.8872	0.57692	0.50734	0.9615 (max of	3	5662; 5258; 5259	0.33015698	-0.8492282		5.0
	ZINC49600303	-7.7	363.341	-1.4804	8	3	270.63	0.35702	128.51	-0.3333	1816.254130103	0.30291	7.2212	-4.8872	0.57692	0.50393	0.9615 (max of	3	5662; 5260; 5258	0.3466079	-0.8674627		5.0
	ZINC49600301	-7.7	363.341	-1.4804	8	3	270.63	0.35702	128.51	-0.3333	1816.254130103	0.30291	7.2212	-4.8872	0.57692	0.50929	0.9615 (max of	3	5662; 5260; 5259	0.3238461	-0.8689197		5.0
	ZINC71404466	-7.7	434.396	0.3932	10	6	291.63	0.4171	166.14	1.8995	1816.254130103	0.25406	5.3476	1.5477	0.41935	0.35036	0.96502 (max o	4	1069; 1343; 1943; 22	0.72385526	-0.5037901		4.0
	ZINC31153654	-7.7	404.469	-2.1254	7	2	300.07	0.20479	93.61	6.8949	1816.254130103	0.26252	7.8662	-8.096	0.53333	0.52624	0.92527	1	1522	-0.2496094	0.49565175		4.0
	ZINC22457076	-7.7	378.511	0.659	6	2	280.29	0.20101	73.24	6.0263	1816.254130103	0.29169	5.0818	2.2592	0.55556	0.49191	0.94605 (max o	4	1536; 4276; 4285; 47	-0.0961006	0.16851032		4.0
	ZINC35458401	-7.7	432.511	-1.9041	7	1	310.36	0.30229	134.63	0.74206	1816.254130103	0.25406	7.6449	-7.4948	0.3871	0.46879	0.94488 (max o	4	6645; 5639; 1917; 52	-0.4406553	0.45142934		4.0
	ZINC04293611	-7.7	447.399	-5.6024	12	2	330.6	0.43336	185.07	3.3292	1816.254130103	0.24612	11.343	-22.763	0.59375	0.5751	0.97948 (max o	3	2069; 3242; 3671	-0.8544408	-0.0603549		4.0
	ZINC20464879	-7.7	412.461	-2.9746	10	5	321.05	0.37299	122.95	0.20551	1816.254130103	0.27158	8.7154	-10.953	0.51724	0.57182	0.90236 (max o	2	5550; 2996	0.63512105	0.37863395		4.0
	ZINC31157210	-7.7	424.401	-1.2512	10	5	297.96	0.40804	162.98	-10.12	1816.254130103	0.26252	6.992	-4.766	0.5	0.4557	0.96589 (max o	5	6615; 2260; 3043; 43	-0.822905	-0.0335028		4.0
	ZINC49600285	-7.7	422.469	-0.6945	10	6	308.13	0.3849	158.3	-13.648	1816.254130103	0.27158	6.4353	-2.5573	0.58621	0.50602	0.97415	1	6040	0.8761598	0.41757455		4.0
	ZINC04915431	-7.7	442.423	-3.2151	10	3	338.39	0.37454	167.92	-10.491	1816.254130103	0.24612	8.9559	-13.063	0.5625	0.53641	0.9499 (max of	4	7189; 6736; 2176; 23	-0.0143729	-0.4187393		4.0
	ZINC67903433	-7.7	430.536	0.8705	8	4	305.55	0.32744	121.14	-5.1488	1816.254130103	0.26252	4.8703	3.3159	0.43333	0.49097	0.96589 (max o	3	5670; 2684; 3538	0.6087638	0.6043512		4.0

Structure	Molecule Name	affinity	Total Molweight	cLogP	H-Acceptors	H-Donors	Total Surface Area	Relative PSA	Polar Surface Area	Druglikeness	Kd (nM) @ 20°C	LE from K...	LLE from Kd ...	LELP from Kd (n...	Shape Index	Molecular...	Neighbor Si...	Neig...	Neighbor	Neighbor ...	Neighbor ...	Ring Systems	frequency
	ZINC03869446	-7.7	423.452	-5.2627	13	5	297.18	0.46214	219.64	1.2977	1816.254130103	0.26252	11.004	-20.047	0.6	0.61616	0.97533 (max o	3	2717; 3129; 4050	0.562662	0.7884742		4.0
	ZINC08297667	-7.7	428.579	-3.4261	8	3	366.49	0.26874	77.92	-0.88368	1816.254130103	0.25406	9.1669	-13.486	0.67742	0.58847	0.95521 (max o	4	2101; 3273; 3278; 41	0.42449307	-0.0686714		4.0
	ZINC72320326	-7.7	401.430	-2.4695	9	4	289.37	0.38967	156.58	-3.0756	1816.254130103	0.28128	8.2103	-8.7796	0.5	0.52725	0.97773 (max o	4	3444; 3885; 3886; 52	0.23683582	-0.4878833		4.0
	ZINC72320327	-7.7	401.430	-2.4695	9	4	289.37	0.38967	156.58	-3.0756	1816.254130103	0.28128	8.2103	-8.7796	0.5	0.53007	0.97773 (max o	4	3444; 3885; 4794; 52	0.26474848	-0.5187359		4.0
	ZINC35454991	-7.7	387.403	-2.5561	9	4	285.25	0.3953	156.58	-5.0739	1816.254130103	0.29169	8.2969	-8.763	0.59259	0.63006	0.95201 (max o	7	5965; 5629; 3447; 38	-0.9018523	0.05590392		4.0
	ZINC35454983	-7.7	387.403	-2.5561	9	4	285.25	0.3953	156.58	-5.0739	1816.254130103	0.29169	8.2969	-8.763	0.59259	0.63006	0.94605 (max o	7	5965; 5629; 5228; 34	-0.9197929	0.06139072		4.0
	ZINC31155438	-7.7	370.440	0.6306	7	4	275.33	0.31032	116.45	-4.553	1816.254130103	0.30291	5.1102	2.0818	0.53846	0.46712	0.97269 (max o	7	2625; 3461; 3883; 38	0.22818165	-0.5072293		4.0
	ZINC49181277	-7.7	374.472	0.91	7	5	276.92	0.30875	119.61	-4.4688	1816.254130103	0.30291	4.8308	3.0042	0.53846	0.54867	0.97791 (max o	8	6620; 6299; 5977; 52	0.75898904	0.36817893		4.0
	ZINC49181274	-7.7	374.472	0.91	7	5	276.92	0.30875	119.61	-4.4688	1816.254130103	0.30291	4.8308	3.0042	0.53846	0.54867	0.97791 (max o	8	6620; 6299; 5977; 52	0.7420023	0.359132		4.0
	ZINC08295013	-7.7	384.451	-1.5729	9	5	284.64	0.37233	141.57	0.43676	1816.254130103	0.29169	7.3137	-5.3923	0.51852	0.47529	0.8934 (max of	5	6132; 6030; 5717; 40	-0.5326319	-0.2109464		4.0
	ZINC08299449	-7.7	424.500	-0.8006	11	3	318.9	0.33612	123.32	5.4193	1816.254130103	0.26252	6.5414	-3.0496	0.46667	0.58225	0.91333 (max o	3	5011; 3699; 3819	-0.1349723	0.2645046		4.0
	ZINC35464609	-7.7	375.443	-1.0588	7	1	289.31	0.2544	100.98	7.6353	1816.254130103	0.29169	6.7996	-3.6298	0.51852	0.63645	0.96617 (max o	7	6885; 5640; 3932	-0.1805464	0.8904926		4.0
	ZINC72320082	-7.7	391.395	-0.7635	8	4	292.41	0.34103	139.51	-1.3035	1816.254130103	0.28128	6.5043	-2.7144	0.5	0.56809	0.98006 (max o	3	3969; 3970; 4444	0.6821845	0.59083843		4.0
	ZINC05121257	-7.7	364.436	0.0565	6	5	239.12	0.32845	118.22	-2.8615	1816.254130103	0.30291	5.6843	0.18652	0.34615	0.30202	0.94008 (max o	4	2797; 4116; 4117; 45	-0.8441786	0.20595464		4.0
	ZINC13348405	-7.7	383.349	-4.4475	12	8	254.94	0.57347	198.4	-3.1137	1816.254130103	0.30291	10.188	-14.682	0.46154	0.52717	0.94951 (max o	3	7185; 4250; 4251	-0.7527937	0.1702074		4.0
	ZINC31163581	-7.7	432.511	-1.9041	7	1	310.36	0.30229	134.63	0.74206	1816.254130103	0.25406	7.6449	-7.4948	0.3871	0.47217	0.94488 (max o	4	1917; 4374; 5200; 52	-0.4670104	0.47806254		4.0
	ZINC31163574	-7.7	432.511	-1.9041	7	1	310.36	0.30229	134.63	0.74206	1816.254130103	0.25406	7.6449	-7.4948	0.3871	0.47217	0.94488 (max o	4	5236; 5202; 4374; 52	-0.446737	0.47273564		4.0
	ZINC31163578	-7.7	432.511	-1.9041	7	1	310.36	0.30229	134.63	0.74206	1816.254130103	0.25406	7.6449	-7.4948	0.3871	0.46682	0.93956 (max o	4	5639; 5202; 4374; 52	-0.4617896	0.45708808		4.0
	ZINC49601558	-7.7	445.396	-4.3457	12	6	310.43	0.47985	206.27	-2.4636	1816.254130103	0.25406	10.087	-17.105	0.3871	0.49243	0.98268 (max o	3	7566; 4429; 4854	-0.4846152	-0.7528334		4.0
	ZINC03842166	-7.7	419.480	-0.0889	10	2	324.62	0.31776	120.94	6.2103	1816.254130103	0.26252	5.8297	-0.33864	0.46667	0.58067	0.91374 (max o	7	6973; 5339; 4966; 32	-0.0335887	0.38090676		4.0
	ZINC03977881	-7.7	414.414	-5.2749	11	2	288.77	0.52201	208.91	5.0035	1816.254130103	0.28128	11.016	-18.753	0.60714	0.50083	0.94573 (max o	3	6203; 3180; 4497	0.4820611	0.5226579		4.0
	ZINC04043931	-7.7	392.450	-1.0059	8	2	301.56	0.30707	121.37	-1.6404	1816.254130103	0.28128	6.7467	-3.5762	0.5	0.46306	0.96519 (max o	3	5747; 5360; 4513	0.10404795	0.9143802		4.0
	ZINC20463838	-7.7	416.496	-0.9149	8	2	304.22	0.23684	84.15	4.7082	1816.254130103	0.26252	6.6557	-3.485	0.53333	0.44315	0.93686 (max o	5	6768; 6255; 5852; 55	0.35085374	0.00542834		4.0
	ZINC01543916	-7.7	355.374	-4.8756	11	4	263.55	0.49228	168.7	-0.63512	1816.254130103	0.31503	10.616	-15.477	0.56	0.58741	0.98897 (max o	3	5296; 5295; 4891	0.789027	0.12111179		4.0
	ZINC01543916	-7.7	354.366	-1.7048	11	4	264.34	0.4938	167.08	-16.91	1816.254130103	0.31503	7.4456	-5.4116	0.56	0.60937	0.98671 (max o	3	5296; 5295; 4892	0.79014325	0.14324404		4.0
	ZINC05224220	-7.7	382.400	-4.6527	12	6	276.17	0.50534	162.9	2.8692	1816.254130103	0.29169	10.394	-15.951	0.44444	0.40925	0.93373 (max o	3	6158; 5795; 3250	-0.6463274	-0.460845		4.0
	ZINC02151124	-7.7	367.385	-4.2278	11	5	269.44	0.46129	136.88	2.9437	1816.254130103	0.30291	9.9686	-13.957	0.46154	0.41144	0.92683 (max o	7	6492; 5864; 5808; 54	-0.6085669	-0.5065997		4.0
	ZINC12483914	-7.7	406.481	-0.105	9	3	299.96	0.28724	95.53	5.035	1816.254130103	0.27158	5.8458	-0.38663	0.55172	0.48842	0.9613 (max of	2	6504; 5475	-0.6380552	-0.3071170		4.0
	ZINC12530608	-7.7	403.522	-2.6237	9	3	303	0.35881	117.63	7.0907	1816.254130103	0.29169	8.3645	-8.9947	0.55556	0.51543	0.88798 (max o	4	5897; 5522; 4651; 46	-0.2497171	0.12829886		4.0
	ZINC12663468	-7.7	365.234	-7.4348	12	3	245.3	0.60306	202.06	-30.618	1816.254130103	0.32816	13.176	-22.656	0.58333	0.48729	0.92619 (max o	3	6519; 6517; 6199	-0.4782061	-0.4146149		4.0
	ZINC12663468	-7.7	366.242	-7.6237	12	4	234.33	0.58447	203.31	-31.599	1816.254130103	0.32816	13.365	-23.232	0.58333	0.51178	0.93334 (max o	3	6520; 6518; 6200	-0.4691489	-0.4710877		4.0
	ZINC30879429	-7.7	376.495	-0.4331	6	1	320.79	0.22289	74.11	2.9872	1816.254130103	0.29169	6.1739	-1.4848	0.55556	0.60461	0.92394 (max o	5	7719; 7030; 7029; 18	-0.0830910	-0.1808976		4.0
	ZINC31155124	-7.7	386.439	-0.5583	8	5	279.37	0.35272	136.68	-6.1261	1816.254130103	0.29169	6.2991	-1.914	0.44444	0.51342	0.97407 (max o	7	6606; 5963; 1906; 35	0.18662271	0.8782228		4.0
	ZINC31155111	-7.7	386.439	-0.5583	8	5	279.37	0.35272	136.68	-6.1261	1816.254130103	0.29169	6.2991	-1.914	0.44444	0.51342	0.97407 (max o	7	6606; 5963; 5188; 19	0.22765478	0.8604055		4.0
	ZINC31159001	-7.7	397.355	-2.4322	10	4	280.77	0.44104	161.88	-4.6331	1816.254130103	0.28128	8.173	-8.647	0.42857	0.36086	0.97037 (max o	4	7547; 6855; 6293; 57	0.13945751	-0.6329368		4.0
	ZINC31164237	-7.7	365.313	-2.4855	9	4	257.49	0.44208	152.65	-3.5303	1816.254130103	0.30291	8.2263	-8.2053	0.46154	0.28526	0.91351 (max o	4	6607; 6293; 5608; 52	0.1975239	-0.6308853		4.0
	ZINC31160850	-7.7	389.419	-1.9705	9	2	290.57	0.36673	134.58	-13.144	1816.254130103	0.29169	7.7113	-6.7554	0.55556	0.51846	0.94905 (max o	7	6882; 6860; 6644; 66	-0.7293151	-0.0781340		4.0
	ZINC31168779	-7.7	412.433	0.3716	9	5	305.3	0.35552	145.91	-6.6975	1816.254130103	0.27158	5.3692	1.3683	0.62069	0.49148	0.98316 (max o	3	5980; 5612; 5213	-0.7130499	0.4372617		4.0
	ZINC31168767	-7.7	412.433	0.3716	9	5	305.3	0.35552	145.91	-6.6975	1816.254130103	0.27158	5.3692	1.3683	0.62069	0.49148	0.98316 (max o	3	5980; 5612; 5214	-0.7075658	0.4757339		4.0
	ZINC35458995	-7.7	410.414	-2.1178	11	6	284.15	0.46328	175.37	-2.7727	1816.254130103	0.28128	7.8586	-7.5293	0.5	0.43791	0.9678 (max of	3	6884; 6334; 6008	0.6802234	-0.4653919		4.0
	ZINC35465101	-7.7	373.420	-1.7731	8	4	278.5	0.35806	139.51	-9.7968	1816.254130103	0.30291	7.5139	-5.8535	0.5	0.51159	0.94951 (max o	4	7247; 7092; 6648; 56	0.4541019	0.5826966		4.0
	ZINC67913285	-7.7	410.417	0.3539	9	3	294.52	0.37845	147.43	-9.4267	1816.254130103	0.27158	5.3869	1.3031	0.41379	0.5305	0.95908 (max o	5	7900; 7105; 6875; 60	0.41913915	0.34937552		4.0
	ZINC72320477	-7.7	354.354	-0.5368	8	0	240.95	0.35725	89.52	-3.7722	1816.254130103	0.31503	6.2776	-1.704	0.48	0.30378	0.96243 (max o	3	6918; 6681; 5688	0.7605934	0.41359496		4.0

Structure	Molecule Name	affinity	Total Molweight	cLogP	H-Acceptors	H-Donors	Total Surface Area	Relative PSA	Polar Surface Area	Druglikeness	Kd (nM) @ 20°C	LE from K...	LLE from Kd ...	LELP from Kd (n...	Shape Index	Molecular...	Neighbor Si...	Neig...	Neighbor	Neighbor ...	Neighbor ...	Ring Systems	frequency
	ZINC20464808	-7.7	388.822	-0.012	7	3	272.8	0.27218	91.26	1.7092	1816.254130103	0.30291	5.7528	-0.039614	0.53846	0.52866	0.92064 (max o	4	1248; 4734; 4745; 47	-0.3878357	0.01304088		3.0
	ZINC20464808	-7.7	389.830	-2.1217	7	4	286.84	0.3078	92.46	-1.0161	1816.254130103	0.30291	7.8625	-7.0043	0.53846	0.53101	0.88178 (max o	2	1249; 4735	0.70241797	0.33839273		3.0
	ZINC12603926	-7.7	407.465	-1.1807	9	4	307.76	0.32087	120.36	2.7702	1816.254130103	0.27158	6.9215	-4.3476	0.65517	0.5702	0.94804 (max o	4	1210; 1496; 1800; 29	0.24562527	-0.3732375		3.0
	ZINC05433799	-7.7	390.423	-6.259	11	3	287.35	0.41274	152.24	3.6259	1816.254130103	0.28128	12	-22.252	0.46429	0.46383	0.9543 (max of	5	1177; 2203; 2861; 29	0.02043244	0.30802122		3.0
	ZINC59487915	-7.7	440.927	-3.5869	8	3	312.08	0.35395	147.1	7.5816	1816.254130103	0.27158	9.3277	-13.208	0.44828	0.49833	0.95642 (max o	5	7448; 6904; 6667; 63	0.6009101	0.36992922		3.0
	ZINC35442564	-7.7	353.485	-2.2061	7	2	285.52	0.25298	66.32	8.0885	1816.254130103	0.31503	7.9469	-7.0028	0.56	0.51563	0.88831 (max o	5	7085; 5956; 5955; 22	-0.1298906	0.52607083		3.0
	ZINC03838832	-7.7	357.385	-3.9234	10	7	251.23	0.49496	178.59	1.3944	1816.254130103	0.31503	9.6642	-12.454	0.56	0.43003	0.90171 (max o	4	6659; 6352; 5326; 36	-0.6333535	-0.1861374		3.0
	ZINC05396950	-7.7	353.445	-1.1246	6	1	279.7	0.22074	57.95	4.76	1816.254130103	0.30291	6.8654	-3.7126	0.57692	0.41554	0.96998 (max o	3	5776; 591; 4542	-0.4928138	-0.3791333		3.0
	ZINC03882444	-7.7	365.791	-0.7447	6	1	244.4	0.25344	91.32	2.0837	1816.254130103	0.31503	6.4855	-2.3639	0.48	0.3225	0.9594 (max of	2	1996; 4888	0.7350152	0.24982217		3.0
	ZINC01278767	-7.7	365.791	-0.7447	6	1	244.4	0.25344	91.32	2.0837	1816.254130103	0.31503	6.4855	-2.3639	0.48	0.3225	0.9594	1	4944	0.7171795	0.26285148		3.0
	ZINC12663491	-7.7	411.417	1.0798	10	0	303.14	0.30352	98.38	-1.4369	1816.254130103	0.26252	4.661	4.1131	0.46667	0.37029	0.96385 (max o	2	4061; 4661	-0.6704542	-0.6185106		3.0
	ZINC04041892	-7.7	350.351	-0.6256	7	2	216.75	0.5672	166.36	3.1728	1816.254130103	0.34242	6.3664	-1.827	0.3913	0.26346	0.93319 (max o	3	3194; 4068; 4069	0.6084939	-0.4169609		3.0
	ZINC33342870	-7.7	362.385	-4.4497	8	3	247.6	0.46151	161.09	7.0306	1816.254130103	0.31503	10.191	-14.125	0.56	0.43729	0.93033 (max o	5	2276; 3519; 4280; 44	-0.4760589	-0.0695213		3.0
	ZINC08627185	-7.7	379.455	-0.2946	8	4	295.46	0.32549	120.78	2.7954	1816.254130103	0.29169	6.0354	-1.01	0.62963	0.56649	0.92739 (max o	3	2502; 2879; 5019	0.40753743	-0.4910345		3.0
	ZINC08627183	-7.7	366.416	-1.3815	9	5	279.4	0.38522	132.81	2.7633	1816.254130103	0.30291	7.1223	-4.5607	0.65385	0.58109	0.8934	1	5020	0.4186957	-0.5079188		3.0
	ZINC08790219	-7.7	430.479	-1.3933	9	1	322.43	0.29464	119.08	7.6495	1816.254130103	0.25406	7.1341	-5.4842	0.54839	0.45492	0.96971 (max o	7	6658; 5753; 5492; 37	0.5145995	0.32740167		3.0
	ZINC20463838	-7.7	417.504	-3.0246	8	3	318.26	0.2705	85.35	2.5284	1816.254130103	0.26252	8.7654	-11.521	0.53333	0.44769	0.93686 (max o	4	6254; 5537; 4726; 47	-0.5954132	-0.6417911		3.0
	ZINC08740334	-7.7	445.463	-2.1077	11	8	309.21	0.43065	181.33	-6.437	1816.254130103	0.25406	7.8485	-8.2962	0.51613	0.43854	0.95267 (max o	4	6370; 5457; 3744; 41	-0.1982432	-0.9297347		3.0
	ZINC13335253	-7.7	410.374	-0.6661	10	5	285.96	0.41453	155.14	-5.6381	1816.254130103	0.27158	6.4069	-2.4527	0.44828	0.36343	0.95099 (max o	4	5499; 5498; 5433; 50	-0.2712033	0.7683686		3.0
	ZINC13335251	-7.7	410.374	-0.6661	10	5	285.96	0.41453	155.14	-5.6381	1816.254130103	0.27158	6.4069	-2.4527	0.44828	0.36343	0.94823 (max o	4	5499; 5498; 5433; 50	-0.2882773	0.7799337		3.0
	ZINC08879181	-7.7	372.376	0.1094	8	2	256.08	0.32876	97.33	1.9472	1816.254130103	0.29169	5.6314	0.37505	0.55556	0.31805	0.92739 (max o	3	1479; 3308; 4198	-0.7980133	-0.4632333		2.0
	ZINC15672815	-7.7	350.417	-3.0585	8	1	267.18	0.30047	106.32	9.5443	1816.254130103	0.31503	8.7993	-9.7086	0.6	0.59872	0.93686 (max o	3	1514; 2189; 3809	-0.1035833	0.82872593		2.0
	ZINC02130731	-7.7	372.400	-0.6351	8	1	273.91	0.31032	109.85	6.5995	1816.254130103	0.29169	6.3759	-2.1773	0.44444	0.51133		0		0.25788194	-0.8916373		2.0
	ZINC20504114	-7.7	448.586	0.1984	8	4	332.78	0.32232	129.39	5.1704	1816.254130103	0.25406	5.5424	0.78093	0.48387	0.45821	0.92566 (max o	3	7492; 5826; 2240	0.41674262	0.08939014		2.0
	ZINC71405300	-7.7	419.526	-2.0672	10	3	287.14	0.44114	161.6	6.8226	1816.254130103	0.29169	7.808	-7.0869	0.44444	0.49888	0.88151 (max o	3	5900; 5423; 3258	-0.1314495	0.3683464		2.0
	ZINC03839412	-7.7	422.548	-0.2189	8	2	303.87	0.328	124.12	1.8442	1816.254130103	0.27158	5.9597	-0.80603	0.55172	0.38835	0.97649 (max o	4	2096; 2375; 2752; 37	-0.6092821	-0.0055667		2.0
	ZINC03839858	-7.7	433.531	-0.9693	9	2	311.05	0.34252	129.05	7.7864	1816.254130103	0.26252	6.7101	-3.6922	0.56667	0.34729	0.90418 (max o	2	5330; 2380	-0.4287672	-0.7800849		2.0
	ZINC13660201	-7.7	388.455	-0.0883	8	5	281.14	0.3505	136.68	-3.7924	1816.254130103	0.29169	5.8291	-0.30272	0.51852	0.469	0.97407 (max o	6	2558; 2964; 2965; 33	-0.3615435	0.22405888		2.0
	ZINC05685481	-7.7	357.385	-1.3033	7	6	239.87	0.30792	116.32	-0.30516	1816.254130103	0.30291	7.0441	-4.3026	0.46154	0.46139	0.95257 (max o	3	2843; 3267; 4137	-0.1896315	0.73721313		2.0
	ZINC13403582	-7.7	358.325	-1.1	8	5	265.67	0.39229	150.15	2.235	1816.254130103	0.30291	6.8408	-3.6314	0.61538	0.52559	0.9615 (max of	6	6068; 6067; 2743; 29	0.01503733	-0.8896762		2.0
	ZINC72320349	-7.7	361.413	-0.5052	6	1	266.74	0.2868	103.73	-2.1259	1816.254130103	0.30291	6.246	-1.6678	0.46154	0.51711	0.95257 (max o	3	6061; 3114; 5283	0.91237915	0.17629966		2.0
	ZINC04037319	-7.7	393.438	0.1128	6	2	291.12	0.24457	103.27	-4.785	1816.254130103	0.27158	5.628	0.41535	0.51724	0.48493	0.94823 (max o	7	7287; 7119; 7117; 61	-0.0391287	0.70941824		2.0
	ZINC04544767	-7.7	370.309	-1.2533	10	5	250.78	0.47269	155.14	-5.8493	1816.254130103	0.30291	6.9941	-4.1375	0.42308	0.4206	0.9615 (max of	10	6430; 6211; 5408; 29	-0.4312914	0.48789257		2.0
	ZINC05434134	-7.7	408.478	-0.4481	9	2	300.3	0.37476	136.29	6.6275	1816.254130103	0.28128	6.1889	-1.5931	0.46429	0.50988	0.92152	1	5428	-0.1226408	0.10238864		2.0
	ZINC20465945	-7.7	386.470	-0.6421	7	5	310.77	0.2888	95.26	1.772	1816.254130103	0.28128	6.3829	-2.2828	0.57143	0.55194	0.9427 (max of	4	7411; 3407; 3854; 38	0.35177004	-0.2777481		2.0
	ZINC39560293	-7.7	432.420	-2.0441	11	7	297.42	0.44281	178.53	-1.0377	1816.254130103	0.26252	7.7849	-7.7863	0.53333	0.44723	0.94529 (max o	2	505; 3528	-0.3723847	0.4235741		2.0
	ZINC01542931	-7.7	390.419	-1.579	10	4	282.43	0.3472	135.74	3.7713	1816.254130103	0.28128	7.3198	-5.6137	0.57143	0.51293	0.9678	1	3560	-0.2067862	-0.9124768		2.0
	ZINC05433876	-7.7	435.504	-2.0773	9	2	307.96	0.46457	179.04	4.1281	1816.254130103	0.27158	7.8181	-7.649	0.62069	0.39689	0.96682 (max o	6	6459; 1749; 3698; 37	-0.6887586	-0.5452216		2.0
	ZINC13341234	-7.7	359.309	-0.6258	8	4	264.21	0.38893	147.35	-1.3192	1816.254130103	0.30291	6.3666	-2.0659	0.61538	0.46464	0.9615 (max of	6	6068; 6067; 5083; 27	0.01479460	-0.8516925		2.0
	ZINC12297056	-7.7	372.491	-0.4166	7	4	311.59	0.27	81.67	4.9471	1816.254130103	0.29169	6.1574	-1.4282	0.66667	0.59158	0.96389	1	3759	-0.8901392	-0.4435437		2.0
	ZINC03838462	-7.7	428.504	-0.4722	9	5	311.89	0.42226	173.43	3.7036	1816.254130103	0.27158	6.213	-1.7387	0.51724	0.53493	0.90045 (max o	3	5717; 1374; 4042	-0.4722110	-0.2557277		2.0
	ZINC08627172	-7.7	390.434	-1.6386	7	4	289.32	0.27447	108.29	-1.0286	1816.254130103	0.28128	7.3794	-5.8256	0.67857	0.48291	0.9308 (max of	8	6755; 5025; 2569; 28	0.12937446	-0.4938964		2.0
	ZINC22922147	-7.7	378.447	-1.4174	8	2	265.03	0.25635	80.24	6.8035	1816.254130103	0.29169	7.1582	-4.8592	0.55556	0.43758	0.93686 (max o	7	7172; 7065; 6847; 65	0.46382183	-0.1807901		2.0

Structure	Molecule Name	affinity	Total Molweight	cLogP	H-Acceptors	H-Donors	Total Surface Area	Relative PSA	Polar Surface Area	Druglikeness	Kd (nM) @ 20°C	LE from K...	LLE from Kd ...	LELP from Kd (n...	Shape Index	Molecular...	Neighbor Si...	Neig...	Neighbor	Neighbor ...	Neighbor ...	Ring Systems	frequency
	ZINC12603665	-7.7	410.445	-2.4046	10	5	315.04	0.38976	130.79	0.72584	1816.254130103	0.27158	8.1454	-8.8542	0.62069	0.54674	0.94543 (max o	3	7599; 4225; 4263	0.3714313	-0.4721599		2.0
	ZINC16946265	-7.7	441.502	-0.0884	8	2	347.58	0.2686	97.58	0.07	1816.254130103	0.24612	5.8292	-0.35918	0.4375	0.43839	0.92889 (max o	2	6543; 5117	-0.1490015	-0.9037989		2.0
	ZINC20462826	-7.7	379.475	-3.6421	7	1	304.83	0.29138	105.01	4.3973	1816.254130103	0.29169	9.3829	-12.486	0.55556	0.58245	0.93069 (max o	6	5906; 3834; 4709; 47	-0.2378728	-0.2591686		2.0
	ZINC20462866	-7.7	391.442	-3.6697	8	1	314	0.3278	118.15	5.2692	1816.254130103	0.28128	9.4105	-13.047	0.53571	0.59159	0.96519 (max o	3	4713; 4716; 5127	-0.2262037	-0.2954855		2.0
	ZINC20758853	-7.7	387.415	-1.7681	9	2	285.59	0.33853	128.86	6.7131	1816.254130103	0.28128	7.5089	-6.286	0.46429	0.43568	0.95401	1	4762	0.5166103	-0.5917142		2.0
	ZINC35458252	-7.7	380.435	0.7597	7	2	268.7	0.33469	105.59	-1.5895	1816.254130103	0.29169	4.9811	2.6044	0.44444	0.35385	0.93996	1	4835	0.04027754	-0.9905004		2.0
	ZINC49181283	-7.7	372.456	1.0537	7	4	275.82	0.30977	116.45	-4.0371	1816.254130103	0.30291	4.6871	3.4786	0.5	0.54987	0.97533 (max o	3	4851; 5254; 5255	0.77604455	0.37759128		2.0
	ZINC00803234	-7.7	358.460	-0.4344	6	2	269.54	0.20145	71.31	5.3478	1816.254130103	0.30291	6.1752	-1.4341	0.5	0.39959	0.98517 (max o	3	3125; 3555; 4886	0.11582641	-0.8167013		2.0
	ZINC00803233	-7.7	358.460	-0.4344	6	2	269.54	0.20145	71.31	5.3478	1816.254130103	0.30291	6.1752	-1.4341	0.5	0.38664	0.98284 (max o	3	4887; 3125; 3555	0.0965062	-0.8274708		2.0
	ZINC03838719	-7.7	367.448	-0.5655	9	3	281.64	0.34601	124.84	0.55	1816.254130103	0.30291	6.3063	-1.8669	0.5	0.59665	0.92721	1	6457	-0.0361675	0.3203101		2.0
	ZINC03839827	-7.7	370.449	-2.6668	8	2	272.82	0.40466	143.94	6.9021	1816.254130103	0.31503	8.4076	-8.4653	0.64	0.58591	0.9594 (max of	3	5329; 2755; 4924	-0.4760932	-0.3942709		2.0
	ZINC03839825	-7.7	354.382	-3.4055	9	2	265.33	0.38786	127.87	4.9263	1816.254130103	0.31503	9.1463	-10.81	0.64	0.5137	0.9594	1	4925	-0.4960476	-0.4000188		2.0
	ZINC03840143	-7.7	397.494	-2.681	9	6	299.86	0.3583	144.37	2.2969	1816.254130103	0.28128	8.4218	-9.5316	0.53571	0.49984	0.93069 (max o	7	6703; 6698; 6396; 63	-0.6063118	-0.2404376		2.0
	ZINC03840407	-7.7	419.480	-1.1458	10	4	318.17	0.37078	154.72	6.3564	1816.254130103	0.26252	6.8866	-4.3646	0.46667	0.52551	0.84809	1	5653	-0.2144669	-0.866168		2.0
	ZINC03874934	-7.7	429.511	0.1874	7	1	362.23	0.2428	89.33	-3.757	1816.254130103	0.25406	5.5534	0.73763	0.58065	0.544	0.98065	1	5713	0.8514354	-0.1387583		2.0
	ZINC03984579	-7.7	358.524	1.9934	4	2	242.34	0.10762	62.08	-2.6151	1816.254130103	0.30291	3.7474	6.5808	0.53846	0.45447	0.92394 (max o	2	5741; 4948	0.93646246	0.09298979		2.0
	ZINC04085599	-7.7	369.476	-0.9701	6	3	267.09	0.28687	110.05	-5.7297	1816.254130103	0.30291	6.7109	-3.2026	0.42308	0.46411	0.93686 (max o	3	6407; 6105; 5368	-0.8584844	-0.3539401		2.0
	ZINC04336476	-7.7	415.440	-0.3009	8	1	330.97	0.29595	98.56	-0.53287	1816.254130103	0.26252	6.0417	-1.1462	0.46667	0.48146	0.90873	1	6380	0.24804662	-0.9360176		2.0
	ZINC08636404	-7.7	411.485	-0.9622	8	4	304.19	0.36671	134.74	6.2221	1816.254130103	0.27158	6.703	-3.543	0.51724	0.39568	0.94251 (max o	4	6478; 6181; 6171; 61	0.49521992	0.1522075		2.0
	ZINC08740476	-7.7	391.411	1.6778	11	5	273.09	0.54077	192.17	3.1748	1816.254130103	0.29169	4.063	5.7519	0.48148	0.29519	0.96885 (max o	5	6483; 6482; 5834; 58	-0.2947116	0.24107073		2.0
	ZINC08740526	-7.7	375.344	0.9999	12	5	266.84	0.53002	177.07	1.5916	1816.254130103	0.29169	4.7409	3.4279	0.48148	0.31128	0.96885 (max o	5	7338; 6193; 5834; 58	-0.3016384	0.27874497		2.0
	ZINC08740538	-7.7	386.371	0.8103	12	5	275.91	0.50121	176.82	1.3234	1816.254130103	0.28128	4.9305	2.8808	0.5	0.33184	0.98451 (max o	12	6759; 6484; 6192; 54	-0.1995733	-0.6275607		2.0
	ZINC12483569	-7.7	358.460	-0.6202	6	3	283.83	0.25762	68.05	-0.030137	1816.254130103	0.30291	6.361	-2.0475	0.53846	0.50349	0.87087 (max o	2	6567; 5536	0.6232698	-0.0716369		2.0
	ZINC13508092	-7.7	353.302	-2.8445	9	5	246.47	0.47008	167.58	0.33063	1816.254130103	0.31503	8.5853	-9.0293	0.56	0.43889	0.96243 (max o	7	7407; 1367; 1750; 20	-0.2349201	-0.7957496		2.0
	ZINC13541443	-7.7	426.496	-4.7437	12	5	308.42	0.42157	203.5	5.9116	1816.254130103	0.26252	10.485	-18.07	0.46667	0.52942	0.91964	1	5100	-0.0237271	-0.8863944		2.0
	ZINC13541443	-7.7	426.496	-4.1113	12	5	303.46	0.41211	201.13	6.1865	1816.254130103	0.26252	9.8521	-15.661	0.46667	0.55478	0.91964 (max o	2	7484; 5099	-0.0620207	-0.8860910		2.0
	ZINC13660244	-7.7	404.367	-3.1441	11	6	270.25	0.4871	175.37	-5.0325	1816.254130103	0.28128	8.8849	-11.178	0.46429	0.38571	0.95148 (max o	3	6535; 6534; 6533	0.3996462	0.6319666		2.0
	ZINC14726935	-7.7	356.285	-3.7815	8	4	245.19	0.43656	161.18	1.0482	1816.254130103	0.30291	9.5223	-12.484	0.42308	0.3825	0.95257 (max o	3	5516; 5515; 2968	-0.8479588	0.32525542		2.0
	ZINC15707440	-7.7	441.619	-2.827	9	5	357.57	0.36653	131.26	4.8097	1816.254130103	0.26252	8.5678	-10.769	0.63333	0.6169	0.94515 (max o	3	7018; 5115; 5116	-0.2670732	0.00155189		2.0
	ZINC15708467	-7.7	437.587	-3.5654	9	5	358.12	0.36597	131.26	3.8048	1816.254130103	0.26252	9.3062	-13.581	0.66667	0.6181	0.94515 (max o	4	7365; 6244; 5114; 51	-0.2551154	0.03713390		2.0
	ZINC16946261	-7.7	441.502	-0.0884	8	2	347.58	0.2686	97.58	0.07	1816.254130103	0.24612	5.8292	-0.35918	0.4375	0.43688	0.92889 (max o	3	6794; 6543; 5118	-0.1391646	-0.919727		2.0
	ZINC20113041	-7.7	361.353	-3.0669	9	2	265.37	0.38799	104.6	3.003	1816.254130103	0.30291	8.8077	-10.125	0.42308	0.34623	0.90377 (max o	2	6548; 5903	-0.3170637	0.5012539		2.0
	ZINC20462815	-7.7	381.491	-3.0637	7	1	311.74	0.28492	105.01	0.9629	1816.254130103	0.29169	8.8045	-10.503	0.51852	0.65912	0.93686 (max o	3	6249; 5906; 5125	-0.2412331	-0.2804707		2.0
	ZINC20462981	-7.7	390.526	-0.8685	7	2	332.01	0.25279	89.8	5.6121	1816.254130103	0.28128	6.6093	-3.0877	0.57143	0.65073	0.91635 (max o	3	5961; 3835; 4639	-0.3466032	-0.2007810		2.0
	ZINC20762688	-7.7	374.396	-0.2697	9	2	280.55	0.34611	109.33	5.5386	1816.254130103	0.29169	6.0105	-0.9246	0.59259	0.50241	0.92739 (max o	3	6843; 6592; 6278	-0.0804517	-0.3748260		2.0
	ZINC22016783	-7.7	370.400	-1.5547	6	2	283.25	0.2854	120.72	-0.71819	1816.254130103	0.29169	7.2955	-5.3299	0.40741	0.55949		0		-0.8460178	-0.5275653		2.0
	ZINC31156421	-7.7	388.368	-1.9969	10	5	262.24	0.45203	155.14	-7.5663	1816.254130103	0.29169	7.7377	-6.8459	0.48148	0.37688	0.93996 (max o	3	5229; 3068; 4361	-0.5569445	0.22957791		2.0
	ZINC35455096	-7.7	388.368	-1.9969	10	5	262.24	0.45203	155.14	-7.5663	1816.254130103	0.29169	7.7377	-6.8459	0.48148	0.37688	0.94302 (max o	3	5631; 3068; 5190	-0.5857776	0.19552508		2.0
	ZINC31163722	-7.7	431.369	-4.1689	12	4	300.81	0.48469	192.11	0.77139	1816.254130103	0.26252	9.9097	-15.88	0.53333	0.61541	0.99855	1	5203	0.4635048	-0.6734043		2.0
	ZINC31163718	-7.7	431.369	-4.1689	12	4	300.81	0.48469	192.11	0.77139	1816.254130103	0.26252	9.9097	-15.88	0.53333	0.61541	0.99855	1	5204	0.45823348	-0.7100270		2.0
	ZINC31167962	-7.7	398.494	0.9599	7	4	288.68	0.29597	116.45	-3.8942	1816.254130103	0.28128	4.7809	3.4127	0.46429	0.43645	0.96519 (max o	6	6619; 5611; 1610; 19	-0.6707111	0.0375124		2.0
	ZINC31168496	-7.7	374.472	0.91	7	5	276.92	0.30875	119.61	-10.488	1816.254130103	0.30291	4.8308	3.0042	0.53846	0.49164	0.99159 (max o	7	6620; 6299; 5977; 52	0.739071	0.33815035		2.0
	ZINC35360988	-7.7	413.421	-0.3992	10	5	301.66	0.3976	154.78	-17.14	1816.254130103	0.27158	6.14	-1.4699	0.58621	0.54981	0.9617 (max of	3	6224; 3778; 4822	-0.7069479	0.58043706		2.0

Structure	Molecule Name	affinity	Total Molweight	cLogP	H-Acceptors	H-Donors	Total Surface Area	Relative PSA	Polar Surface Area	Druglikeness	Kd (nM) @ 20°C	LE from K...	LLE from Kd ...	LELP from Kd (n...	Shape Index	Molecular...	Neighbor Si...	Neig...	Neighbor	Neighbor ...	Neighbor ...	Ring Systems	frequency
	ZINC35456888	-7.7	376.357	-2.1895	10	4	250.11	0.47371	151.98	-5.795	1816.254130103	0.30291	7.9303	-7.2282	0.46154	0.45048	0.96721	1	5605	-0.1380481	0.8984797		2.0
	ZINC36369968	-7.7	387.454	-3.4461	7	1	311.54	0.286	98.86	3.1333	1816.254130103	0.28128	9.1869	-12.252	0.42857	0.48412	0.93385 (max c	4	6893; 6012; 5646; 52	0.31889233	-0.6047058		2.0
	ZINC36369967	-7.7	387.454	-3.4461	7	1	311.54	0.286	98.86	3.1333	1816.254130103	0.28128	9.1869	-12.252	0.42857	0.48497	0.93385 (max c	4	7269; 6891; 6655; 52	0.2866462	-0.5891753		2.0
	ZINC68590560	-7.7	402.394	-1.763	10	5	276.16	0.42924	155.14	-4.6523	1816.254130103	0.28128	7.5038	-6.2679	0.46429	0.39394	0.93385 (max c	2	6049; 4866	0.73342544	0.6713172		2.0
	ZINC70666331	-7.7	357.317	-2.5166	8	1	271.07	0.36574	127.82	1.1832	1816.254130103	0.30291	8.2574	-8.308	0.5	0.53104		0		0.24775955	-0.8201839		2.0
	ZINC70666569	-7.7	370.336	0.0403	8	1	281.29	0.34804	121.83	-1.1245	1816.254130103	0.29169	5.7005	0.13816	0.48148	0.53622		0		0.90925914	-0.0286167		2.0
	ZINC70673673	-7.7	435.299	0.8783	7	3	287.01	0.29748	113.29	-5.5588	1816.254130103	0.28128	4.8625	3.1226	0.42857	0.35175	0.9984 (max of	3	4873; 4874; 5271	0.52379876	-0.3049938		2.0
	ZINC70673669	-7.7	435.299	0.8783	7	3	287.01	0.29748	113.29	-5.5588	1816.254130103	0.28128	4.8625	3.1226	0.42857	0.35175	0.9984 (max of	3	5272; 4873; 4874	0.51840305	-0.3250141		2.0
	ZINC72320350	-7.7	361.413	-0.5052	6	1	266.74	0.2868	103.73	-2.1259	1816.254130103	0.30291	6.246	-1.6678	0.46154	0.51711	0.95257 (max c	3	6061; 3114; 5282	0.9315222	0.1503941		2.0
	ZINC01530702	-7.7	382.416	-3.6789	8	1	252.44	0.52916	183.21	11.228	1816.254130103	0.31503	9.4197	-11.678	0.48	0.32631	0.93007 (max c	4	6978; 6961; 6491; 53	-0.3844996	-0.4488302		1.0
	ZINC02115954	-7.7	421.449	-1.8453	9	4	305.03	0.38704	164.24	-4.4502	1816.254130103	0.27158	7.5861	-6.7948	0.58621	0.54604		0		-0.9673098	0.06196676		1.0
	ZINC03806644	-7.7	397.403	0.8592	9	1	262.29	0.43181	136.9	-2.482	1816.254130103	0.29169	4.8816	2.9456	0.48148	0.38535		0		0.8658906	-0.3759444		1.0
	ZINC03830507	-7.7	395.435	-1.4848	8	1	271.44	0.47635	169.38	11.089	1816.254130103	0.30291	7.2256	-4.9017	0.57692	0.4758	0.93686 (max c	4	7459; 6203; 4911; 35	0.41335523	0.54025996		1.0
	ZINC03830509	-7.7	395.435	-1.4848	8	1	271.44	0.47635	169.38	11.089	1816.254130103	0.30291	7.2256	-4.9017	0.57692	0.4758	0.93686 (max c	4	7459; 6204; 3592; 49	0.427443	0.52361214		1.0
	ZINC03838657	-7.7	362.520	-3.538	7	3	309.49	0.28592	68.69	-1.8964	1816.254130103	0.30291	9.2788	-11.68	0.61538	0.48198	0.96721 (max c	2	6398; 4156	0.01108333	-0.1008673		1.0
	ZINC03838743	-7.7	357.386	-2.328	9	6	248.63	0.5509	190.22	4.6157	1816.254130103	0.32816	8.0688	-7.0942	0.54167	0.4584		0		-0.0250773	0.98061985		1.0
	ZINC03839411	-7.7	354.430	-1.563	8	2	248.33	0.40136	124.12	7.2103	1816.254130103	0.32816	7.3038	-4.763	0.54167	0.36164	0.90952 (max c	3	1671; 2752; 4039	-0.6158098	-0.0759167		1.0
	ZINC03839615	-7.7	361.440	-0.0116	7	1	253.91	0.26655	78.95	6.363	1816.254130103	0.30291	5.7524	-0.038295	0.57692	0.25749	0.91351 (max c	4	6702; 6092; 2378; 26	-0.3420863	0.70151675		1.0
	ZINC03881755	-7.7	364.445	-1.5602	7	1	266.86	0.33519	118.08	5.1785	1816.254130103	0.31503	7.301	-4.9526	0.56	0.57693	0.90535 (max c	3	6382; 6383; 688	0.14555347	0.79066885		1.0
	ZINC03984763	-7.7	372.551	2.3527	4	2	253.34	0.10294	62.08	-2.6151	1816.254130103	0.29169	3.3881	8.0657	0.51852	0.43143	0.91768 (max c	2	5741; 4948	0.9246214	0.11050805		1.0
	ZINC03984903	-7.7	372.551	2.4478	4	2	256.1	0.10184	62.08	-2.6151	1816.254130103	0.29169	3.293	8.3917	0.55556	0.47262	0.91768 (max c	3	5741; 4946; 4947	0.935047	0.12860174		1.0
	ZINC04026323	-7.7	412.401	2.0625	10	0	284.6	0.30344	99.7	2.5635	1816.254130103	0.26252	3.6783	7.8564	0.36667	0.36355	0.95579	1	4504	0.5918151	0.51102865		1.0
	ZINC04084925	-7.7	381.407	-0.6837	7	1	290.6	0.26982	98.77	6.4717	1816.254130103	0.28128	6.4245	-2.4307	0.60714	0.4439	0.96519 (max c	3	5367; 2787; 4525	-0.0433019	-0.7068841		1.0
	ZINC04147979	-7.7	447.490	-0.619	11	1	337.98	0.31138	121.38	4.375	1816.254130103	0.24612	6.3598	-2.5151	0.46875	0.57427	0.88804 (max c	5	6456; 5339; 3610; 44	-0.0142514	0.37219122		1.0
	ZINC04221605	-7.7	448.498	-4.4605	11	6	336.99	0.41363	187.49	-4.6706	1816.254130103	0.24612	10.201	-18.124	0.46875	0.45334		0		-0.9235585	0.2167998		1.0
	ZINC04270755	-7.7	367.444	0.8411	8	0	279.53	0.2496	79.39	-2.7691	1816.254130103	0.30291	4.8997	2.7767	0.53846	0.57687	0.8996 (max of	3	5785; 1148; 3692	-0.1665631	0.21640788		1.0
	ZINC04277731	-7.7	359.381	0.0974	8	0	261.2	0.28285	83.3	-0.5617	1816.254130103	0.30291	5.6434	0.32155	0.57692	0.39962	0.89608 (max c	4	1497; 1727; 2451; 41	-0.690618	0.17810482		1.0
	ZINC04403891	-7.7	432.487	-2.2172	13	8	300.47	0.42247	212.41	5.0164	1816.254130103	0.25406	7.958	-8.7272	0.64516	0.45048		0		-0.6378444	0.76099485		1.0
	ZINC04535676	-7.7	376.388	-3.4633	8	1	256.5	0.4414	154.97	10.967	1816.254130103	0.30291	9.2041	-11.433	0.5	0.34338	0.93686 (max c	4	706; 1963; 2348; 463	0.03163811	-0.0378533		1.0
	ZINC05161616	-7.7	392.148	-0.8627	8	4	208.69	0.44746	126.65	-0.64769	1816.254130103	0.39379	6.6035	-2.1908	0.5	0.27806		0		-0.5685996	-0.8183169		1.0
	ZINC05397148	-7.7	359.385	-0.6637	9	1	259.04	0.31513	107.26	7.2183	1816.254130103	0.30291	6.4045	-2.1911	0.57692	0.54448	0.84869	1	2839	-0.2192596	-0.7278365		1.0
	ZINC05414247	-7.7	353.461	1.3789	7	0	275.54	0.20589	62.32	4.0995	1816.254130103	0.31503	4.3619	4.3771	0.6	0.62538	0.83321 (max c	2	6740; 4590	0.78786105	0.20938142		1.0
	ZINC08295570	-7.7	374.396	0.6536	9	0	268.29	0.27329	82.63	4.7682	1816.254130103	0.29169	5.0872	2.2407	0.55556	0.49233	0.86499 (max c	2	6906; 2489	0.5181788	0.5628501		1.0
	ZINC12480725	-7.7	439.554	-0.3444	7	1	367.05	0.22468	87	4.1771	1816.254130103	0.24612	6.0852	-1.3993	0.59375	0.57803	0.93952 (max c	3	7029; 6212; 2144	-0.1541613	-0.1572388		1.0
	ZINC12480790	-7.7	444.961	-0.2168	7	1	357.47	0.23342	90.66	4.24	1816.254130103	0.25406	5.9576	-0.85336	0.54839	0.59754	0.88292 (max c	2	5472; 2144	-0.1642352	-0.0831472		1.0
	ZINC12481227	-7.7	394.878	-1.3685	7	0	296.67	0.23066	85.8	4.0966	1816.254130103	0.29169	7.1093	-4.6916	0.51852	0.58923	0.91731 (max c	3	6764; 5846; 5473	-0.1481962	-0.0964545		1.0
	ZINC12483459	-7.7	375.531	0.8979	5	2	285.49	0.15549	58.54	4.1332	1816.254130103	0.29169	4.8429	3.0782	0.59259	0.49716	0.89657 (max c	3	1487; 1488; 2986	0.43453586	0.00909554		1.0
	ZINC12484127	-7.7	355.433	-0.4902	8	3	259.42	0.31844	92.29	-2.1624	1816.254130103	0.31503	6.231	-1.556	0.56	0.47796	0.88639 (max c	2	6564; 3324	-0.5787459	-0.2898055		1.0
	ZINC12529647	-7.7	350.413	-2.8145	7	3	256.51	0.30576	78.2	-1.1908	1816.254130103	0.31503	8.5553	-8.9341	0.52	0.40493	0.86809 (max c	3	6806; 3771; 4215	0.608275	-0.1465497		1.0
	ZINC12603923	-7.7	371.432	-2.3064	9	4	274.52	0.35972	120.36	3.9845	1816.254130103	0.30291	8.0472	-7.6141	0.65385	0.57408	0.8934 (max of	2	5477; 1546	0.18256381	-0.3779466		1.0
	ZINC13407156	-7.7	364.436	0.6461	6	4	232.69	0.32421	107.22	-5.3203	1816.254130103	0.30291	5.0947	2.133	0.34615	0.34783		0		0.41311494	0.88132143		1.0
	ZINC13538065	-7.7	441.463	-4.9186	10	5	340.84	0.37903	173.68	1.6657	1816.254130103	0.24612	10.659	-19.985	0.65625	0.62945	0.91204 (max c	2	2176; 2363	-0.0039342	-0.401628		1.0
	ZINC14555621	-7.7	360.405	0.6314	6	4	267.44	0.27071	99.38	-2.627	1816.254130103	0.30291	5.1094	2.0844	0.61538	0.46248		0		-0.9863726	-0.0562807		1.0

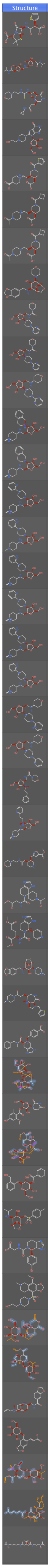

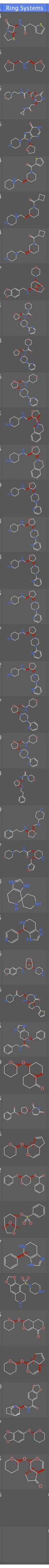

Structure	Molecule Name	affinity	Total Molweight	cLogP	H-Acceptors	H-Donors	Total Surface Area	Relative PSA	Polar Surface Area	Druglikeness	Kd (nM) @ 20°C	LE from K...	LLE from Kd ...	LELP from Kd (n...	Shape Index	Molecular...	Neighbor Si...	Neig...	Neighbor	Neighbor ...	Neighbor ...	Ring Systems	frequency
	ZINC15449281	-7.7	382.416	-3.5967	8	1	252.44	0.52916	183.21	11.519	1816.254130103	0.31503	9.3375	-11.417	0.48	0.32811	0.91611 (max o	2	6961; 4913	-0.3335305	-0.4824974		1.0
	ZINC15675160	-7.7	448.535	-3.9914	11	7	327.14	0.39475	174.19	1.1969	1816.254130103	0.25406	9.7322	-15.711	0.6129	0.42688		0		-0.5491521	-0.8327676		1.0
	ZINC15708344	-7.7	439.603	-3.0629	9	5	347.07	0.37762	131.26	4.4235	1816.254130103	0.26252	8.8037	-11.667	0.63333	0.59278	0.93405 (max o	4	7364; 6243; 5114; 51	-0.2299238	0.01086506		1.0
	ZINC20111426	-7.7	396.403	-2.6385	12	5	277.01	0.44529	155.91	6.2749	1816.254130103	0.28128	8.3793	-9.3805	0.5	0.44146	0.89688 (max o	2	5808; 5462	-0.5883125	-0.5434736		1.0
	ZINC20462868	-7.7	407.509	-2.9918	7	1	320.25	0.34092	133.25	6.7627	1816.254130103	0.28128	8.7326	-10.637	0.53571	0.58908	0.96519 (max o	2	4713; 5126	-0.2444995	-0.3031994		1.0
	ZINC20463242	-7.7	379.499	-1.557	7	1	307.36	0.25407	85.19	4.3084	1816.254130103	0.29169	7.2978	-5.3378	0.55556	0.5755	0.93686 (max o	5	5915; 4641; 4715; 47	-0.1808664	-0.2621024		1.0
	ZINC20463242	-7.7	378.491	-1.6886	7	0	293.32	0.21836	83.99	8.8854	1816.254130103	0.29169	7.4294	-5.789	0.55556	0.58009	0.93069 (max o	9	5918; 5916; 5912; 55	-0.2164353	-0.2002194		1.0
	ZINC20463842	-7.7	365.405	-1.5268	8	2	252.47	0.29465	86.23	1.98	1816.254130103	0.30291	7.2676	-5.0404	0.53846	0.43824	0.9302 (max of	7	7347; 7172; 6995; 68	0.48676026	-0.1491247		1.0
	ZINC20465065	-7.7	422.484	-1.7872	11	3	310.69	0.33245	123.52	5.3822	1816.254130103	0.26252	7.528	-6.8078	0.56667	0.52892	0.95579 (max o	6	5930; 5556; 2233; 29	-0.4023312	0.09518751		1.0
	ZINC20465065	-7.7	423.492	-3.8969	11	4	324.73	0.36132	124.72	3.3544	1816.254130103	0.26252	9.6377	-14.844	0.56667	0.5324	0.96589 (max o	3	2232; 2998; 3398	-0.3973911	-0.5725288		1.0
	ZINC20465090	-7.7	394.494	-2.6414	9	3	291.42	0.28474	107.79	0.54449	1816.254130103	0.28128	8.3822	-9.3908	0.57143	0.42647	0.96253 (max o	6	6818; 5559; 5156; 33	-0.4694425	0.15989786		1.0
	ZINC20465246	-7.7	377.527	-4.3927	6	5	314.2	0.26365	70.6	1.3058	1816.254130103	0.29169	10.134	-15.059	0.55556	0.4668	0.8934 (max of	2	6579; 6269	0.3191943	-0.0122251		1.0
	ZINC20465246	-7.7	376.519	-2.283	6	4	300.16	0.22921	69.4	3.2775	1816.254130103	0.29169	8.0238	-7.8267	0.55556	0.46281	0.86564	1	5152	0.651158	-0.4153745		1.0
	ZINC20465651	-7.7	407.557	-4.0975	8	4	319.26	0.24933	90.11	4.0701	1816.254130103	0.27158	9.8383	-15.088	0.58621	0.4415	0.90299 (max o	3	6587; 5156; 5153	-0.4903227	0.19001679		1.0
	ZINC20465651	-7.7	407.557	-3.9656	8	4	294.25	0.18552	90.16	7.4301	1816.254130103	0.27158	9.7064	-14.602	0.58621	0.42299	0.9218 (max of	5	6586; 5559; 5155; 51	-0.520436	0.16012233		1.0
	ZINC20465651	-7.7	407.557	-4.8506	8	5	312.01	0.23188	86.78	4.8778	1816.254130103	0.27158	10.591	-17.861	0.58621	0.48248	0.88128 (max o	3	5560; 5152; 4743	0.660142	-0.4713418		1.0
	ZINC20465651	-7.7	406.549	-2.8728	8	4	322.98	0.25797	85.53	4.8778	1816.254130103	0.27158	8.6136	-10.578	0.58621	0.49935	0.96428 (max o	6	5560; 3850; 4315; 47	0.6598913	-0.4352225		1.0
	ZINC20465651	-7.7	408.565	-6.0753	8	5	308.29	0.22262	91.36	4.0701	1816.254130103	0.27158	11.816	-22.37	0.58621	0.42299	0.90299 (max o	4	6586; 5155; 5149; 51	-0.5106481	0.17989267		1.0
	ZINC20465651	-7.7	408.565	-6.9603	8	6	326.05	0.26496	87.98	2.9172	1816.254130103	0.27158	12.701	-25.629	0.58621	0.48653	0.88128 (max o	4	5558; 3849; 4313; 47	-0.3024055	-0.5293212		1.0
	ZINC20465654	-7.7	394.514	-4.075	8	4	281.69	0.21669	96.15	0.54449	1816.254130103	0.28128	9.8158	-14.488	0.57143	0.426	0.9218 (max of	3	6586; 5150; 5153	-0.5310696	0.17869437		1.0
	ZINC20465654	-7.7	393.506	-2.0972	8	3	292.66	0.24605	94.9	0.54449	1816.254130103	0.28128	7.838	-7.456	0.57143	0.44532	0.96253 (max o	6	6587; 3399; 4738; 47	-0.4524232	0.17182669		1.0
	ZINC20465749	-7.7	394.490	-1.6852	8	4	315.15	0.29142	95.7	3.3594	1816.254130103	0.28128	7.426	-5.9913	0.57143	0.60006	0.92464 (max o	5	6828; 5590; 5568; 30	0.3041196	-0.0693024		1.0
	ZINC20465912	-7.7	380.531	-4.2619	7	5	324.96	0.28868	83.49	1.3058	1816.254130103	0.29169	10.003	-14.611	0.59259	0.55688	0.91768 (max o	2	6589; 6269	0.33011666	0.00685259		1.0
	ZINC20504004	-7.7	395.478	-0.3042	9	2	308.33	0.30458	110.51	3.3503	1816.254130103	0.28128	6.045	-1.0815	0.64286	0.61393	0.86621 (max o	2	7510; 1545	0.00538925	0.86683697		1.0
	ZINC20762743	-7.7	350.398	-2.0259	9	3	250.18	0.32644	108.88	2.6338	1816.254130103	0.31503	7.7667	-6.4308	0.52	0.48083		0		0.9078032	-0.2191408		1.0
	ZINC20762890	-7.7	402.430	-1.4407	10	3	295.21	0.33566	127.76	-3.5341	1816.254130103	0.27158	7.1815	-5.3049	0.48276	0.55072	0.93686 (max o	4	5989; 5987; 5985; 47	-0.6481207	0.18288435		1.0
	ZINC22922147	-7.7	379.455	-3.5271	8	3	279.07	0.29376	81.44	2.5083	1816.254130103	0.29169	9.2679	-12.092	0.55556	0.43758	0.91109 (max o	7	7172; 7064; 7066; 68	0.50761265	-0.1548206		1.0
	ZINC22922404	-7.7	420.600	-1.4599	7	2	330.52	0.26062	88.57	10.817	1816.254130103	0.27158	7.2007	-5.3756	0.58621	0.49173	0.9617 (max of	5	5586; 5546; 1282; 15	-0.0707991	0.47809476		1.0
	ZINC31153654	-7.7	405.477	-1.9938	7	3	314.11	0.24033	94.81	5.2522	1816.254130103	0.26252	7.7346	-7.5947	0.53333	0.48089	0.8433	1	1523	0.10334685	-0.7591945		1.0
	ZINC31159425	-7.7	432.508	-0.6954	9	6	297.92	0.37473	156.91	-3.8603	1816.254130103	0.26252	6.4362	-2.6489	0.36667	0.44165	0.95318 (max o	3	5682; 3547; 4803	-0.4945157	-0.6973489		1.0
	ZINC31160367	-7.7	408.362	-0.6866	11	4	285.1	0.43294	154.86	0.32355	1816.254130103	0.27158	6.4274	-2.5282	0.51724	0.4237	0.93686 (max o	4	5606; 2262; 3039; 30	0.437226	-0.6784495		1.0
	ZINC31163175	-7.7	404.367	-2.9732	11	6	269.72	0.48806	175.37	-4.4552	1816.254130103	0.28128	8.714	-10.57	0.46429	0.38026		0		0.7941345	0.43207276		1.0
	ZINC31167890	-7.7	390.387	0.6557	8	4	282.69	0.33761	125.68	-0.21208	1816.254130103	0.28128	5.0851	2.3312	0.5	0.31218	0.9543 (max of	4	7420; 7193; 6781; 20	-0.7493945	-0.1312522		1.0
	ZINC32109689	-7.7	358.434	-0.9102	7	2	244.47	0.32487	107.05	-14.377	1816.254130103	0.32816	6.651	-2.7737	0.54167	0.45793	0.95053	1	6306	-0.2891536	0.8660699		1.0
	ZINC36369857	-7.7	378.205	-0.8308	7	2	245.05	0.32781	101.15	0.90232	1816.254130103	0.34242	6.5716	-2.4262	0.52174	0.50623	0.94762 (max o	2	4419; 5244	-0.9400356	-0.1021247		1.0
	ZINC40310036	-7.7	413.493	0.0515	9	1	290.71	0.2832	100.16	7.5368	1816.254130103	0.28128	5.6893	0.18309	0.5	0.48458		0		-0.7692988	0.5276708		1.0
	ZINC68590454	-7.7	374.341	-2.4822	10	5	250.94	0.47238	155.14	-5.3489	1816.254130103	0.30291	8.223	-8.1944	0.46154	0.4136	0.94951	1	1697	-0.0821524	0.82705945		1.0
	ZINC68590543	-7.7	402.394	-1.763	10	5	276.16	0.42924	155.14	-4.6523	1816.254130103	0.28128	7.5038	-6.2679	0.46429	0.39394	0.93686 (max o	2	6049; 4866	0.71439266	0.6810795		1.0
	ZINC71405306	-7.7	372.487	0.965	6	2	300.99	0.21808	55.24	1.443	1816.254130103	0.29169	4.7758	3.3083	0.62963	0.51551		0		0.03323748	-0.9014500		1.0
	ZINC72320590	-7.7	369.301	-2.8257	10	4	253.46	0.47234	157.97	-3.4238	1816.254130103	0.30291	8.5665	-9.3284	0.46154	0.28526	0.8996 (max of	2	6607; 5205	0.18092714	-0.6177227		1.0
	ZINC77257457	-7.7	388.368	-1.827	10	4	263.15	0.45024	151.98	-6.054	1816.254130103	0.29169	7.5678	-6.2634	0.48148	0.37688	0.92094	1	1582	-0.777591	-0.4566945		1.0
	ZINC35464299	-7.7	367.373	-0.8653	8	2	290.28	0.34312	133.19	-7.5278	1816.254130103	0.30291	6.6061	-2.8566	0.57692	0.3849	0.97269 (max o	5	6009; 2266; 2267; 43	-0.0042570	0.84836006		0.0
	ZINC31163090	-7.7	447.463	-4.3629	13	5	344.88	0.43145	199.64	-0.0014118	1816.254130103	0.25406	10.104	-17.173	0.67742	0.7663	1	1	5975	0.32407418	0.9295616		0.0

Structure	Molecule Name	affinity	Total Molweight	cLogP	H-Acceptors	H-Donors	Total Surface Area	Relative PSA	Polar Surface Area	Druglikeness	Kd (nM) @ 20°C	LE from K...	LLE from Kd ...	LELP from Kd (n...	Shape Index	Molecular...	Neighbor Si...	Neig...	Neighbor	Neighbor ...	Neighbor ...	Ring Systems	frequency
	ZINC02104412	-7.6	389.383	-2.0049	9	2	297.98	0.367	133.86	-2.3536	2156.416734724	0.27762	7.6712	-7.2217	0.64286	0.56433	0.9678 (max of	9	868; 1643; 1976; 236	0.25061664	-0.0279894		172.0
	ZINC01750412	-7.6	364.397	-1.3938	7	1	272.5	0.3756	130.06	-2.5187	2156.416734724	0.31094	7.0601	-4.4826	0.6	0.52734	0.96243 (max o	6	6075; 1645; 1980; 27	0.22917612	0.1704214		172.0
	ZINC04556771	-7.6	376.455	-6.5458	8	4	290.66	0.34749	139.78	-6.9405	2156.416734724	0.28791	12.212	-22.736	0.62963	0.51295	0.96064 (max o	4	2459; 2825; 4557; 49	-0.6386795	-0.6510784		172.0
	ZINC02102367	-7.6	364.397	-1.1812	7	2	267.06	0.39486	141.06	3.9328	2156.416734724	0.31094	6.8475	-3.7988	0.6	0.55754	0.95983 (max o	4	6690; 5303; 1087; 19	0.00982806	0.18950434		172.0
	ZINC02111643	-7.6	389.383	-2.0284	9	3	289.78	0.38809	144.86	4.7799	2156.416734724	0.27762	7.6947	-7.3063	0.57143	0.59906	0.96473 (max o	8	6073; 2720; 2726; 34	0.06018696	-0.0175821		172.0
	ZINC02103439	-7.6	361.329	-2.7847	9	2	269.93	0.40514	133.86	1.2511	2156.416734724	0.29898	8.451	-9.314	0.65385	0.58784	0.96106 (max o	4	6390; 3154; 3565; 44	0.1035075	0.07712785		172.0
	ZINC02122926	-7.6	362.357	-1.1247	8	1	273.98	0.34623	113.99	4.8454	2156.416734724	0.29898	6.791	-3.7618	0.53846	0.51192	0.96438 (max o	7	6925; 5709; 3320; 35	0.07891559	0.12718578		172.0
	ZINC02124195	-7.6	350.370	-1.472	7	1	262.03	0.3906	130.06	1.467	2156.416734724	0.32389	7.1383	-4.5447	0.625	0.53126	0.96029 (max o	2	6075; 3992	0.2289593	0.19074048		172.0
	ZINC02123021	-7.6	362.357	-1.0296	8	1	276.74	0.34278	113.99	1.0104	2156.416734724	0.29898	6.6959	-3.4437	0.61538	0.54759	0.91768 (max o	5	6925; 6387; 3566; 35	0.09242555	0.11396368		172.0
	ZINC02108761	-7.6	350.370	-1.6356	7	2	253.3	0.4163	141.06	3.9109	2156.416734724	0.32389	7.3019	-5.0498	0.58333	0.54839	0.95983 (max o	3	6690; 4016; 5299	-0.0198639	0.17247525		172.0
	ZINC04024248	-7.6	365.389	-2.6737	10	5	271.8	0.4117	171.2	-9.9373	2156.416734724	0.29898	8.34	-8.9428	0.69231	0.61211	0.9615 (max of	5	7339; 5746; 2514; 41	-0.0914793	-0.4798032		134.0
	ZINC03874917	-7.6	441.440	-6.0726	12	3	324.93	0.51097	237.22	-4.4889	2156.416734724	0.25911	11.739	-23.436	0.56667	0.69865	0.97304 (max o	3	6104; 5737; 4056	-0.6075524	0.65678513		134.0
	ZINC04899823	-7.6	378.404	-2.0163	9	3	300.73	0.3685	136.66	-10.582	2156.416734724	0.28791	7.6826	-7.0033	0.62963	0.60158	0.96064 (max o	3	6737; 6156; 3669	0.00480607	-0.4491511		134.0
	ZINC61991205	-7.6	394.426	-1.7705	10	5	305.31	0.40913	159.85	-3.2206	2156.416734724	0.27762	7.4368	-6.3774	0.67857	0.62936	0.96253	1	5666	-0.3625829	-0.8290765		134.0
	ZINC61991204	-7.6	394.426	-1.7705	10	5	305.31	0.40913	159.85	-3.2206	2156.416734724	0.27762	7.4368	-6.3774	0.67857	0.62979	0.96253	1	5667	-0.3449674	-0.8196789		134.0
	ZINC09136056	-7.6	411.928	-0.0657	7	3	319.17	0.28449	125.97	3.5604	2156.416734724	0.27762	5.732	-0.23665	0.46429	0.57248	0.97773 (max o	4	6196; 6025; 5251; 54	0.68744063	0.32326034		134.0
	ZINC04027282	-7.6	421.496	-1.1444	10	5	317.73	0.35219	171.2	-51.722	2156.416734724	0.25911	6.8107	-4.4166	0.5	0.60455	0.97533 (max o	4	7467; 6113; 5743; 45	-0.1279806	-0.4550804		134.0
	ZINC13533969	-7.6	395.371	-3.9676	12	4	296.11	0.49211	200.89	-3.1588	2156.416734724	0.27762	9.6339	-14.291	0.67857	0.62774	0.8325	1	6229	-0.9363212	0.04792147		134.0
	ZINC04545850	-7.6	392.430	-4.3105	9	3	305.77	0.3863	166.1	2.1659	2156.416734724	0.27762	9.9768	-15.526	0.57143	0.59636	0.96253 (max o	3	6730; 6444; 6443	-0.0089724	0.64264095		134.0
	ZINC01532336	-7.6	422.196	-2.3837	10	2	295.14	0.4011	161.58	-6.5473	2156.416734724	0.28791	8.05	-8.2795	0.55556	0.60796		0		-0.9666319	0.12897207		134.0
	ZINC03840175	-7.6	417.525	-3.3177	10	7	311.97	0.3986	178.59	5.1105	2156.416734724	0.26805	8.984	-12.377	0.48276	0.52014	0.86023 (max o	3	1676; 2382; 4042	-0.4221437	-0.2174141		127.0
	ZINC04090033	-7.6	383.531	-0.4733	7	3	311.79	0.29122	125.97	2.6006	2156.416734724	0.28791	6.1396	-1.6439	0.62963	0.60909	0.91396 (max o	3	7312; 6125; 5373	0.5447276	0.07829431		127.0
	ZINC04090537	-7.6	384.475	-2.8201	9	4	301.04	0.39566	169.06	3.2287	2156.416734724	0.28791	8.4864	-9.7952	0.62963	0.60585	0.92094 (max o	3	7312; 6955; 5372	0.57350993	0.11941142		127.0
	ZINC03838730	-7.6	359.421	-1.5862	9	6	264.88	0.44024	161.98	-1.2862	2156.416734724	0.31094	7.2525	-5.1013	0.52	0.55729		0		-0.8880173	-0.4310175		127.0
	ZINC72320395	-7.6	441.495	-0.6212	9	4	338.12	0.33349	156.58	-7.5289	2156.416734724	0.25076	6.2875	-2.4773	0.54839	0.6916	0.97642 (max o	3	6373; 5686; 5684	0.8484627	-0.4329406		120.0
	ZINC72320396	-7.6	441.495	-0.6212	9	4	338.12	0.33349	156.58	-7.5289	2156.416734724	0.25076	6.2875	-2.4773	0.54839	0.6916	0.97642 (max o	3	6374; 5684; 5685	0.86420256	-0.4188769		120.0
	ZINC72320394	-7.6	441.495	-0.6212	9	4	338.12	0.33349	156.58	-7.5289	2156.416734724	0.25076	6.2875	-2.4773	0.54839	0.6916	0.97642 (max o	3	6373; 5686; 5685	0.8484189	-0.4109924		120.0
	ZINC05606964	-7.6	364.313	-2.0709	12	4	226.18	0.64152	197.08	-6.7093	2156.416734724	0.32389	7.7372	-6.3938	0.5	0.38642	0.92619 (max o	3	2093; 3703; 4136	-0.7373583	0.6009434		67.0
	ZINC02105819	-7.6	393.355	-0.2858	12	1	282.15	0.47553	157.75	1.9052	2156.416734724	0.27762	5.9521	-1.0295	0.42857	0.30366	0.9543 (max of	5	6179; 5819; 5767; 54	-0.6470105	0.37530077		67.0
	ZINC31167901	-7.6	429.396	-3.4184	11	5	304.75	0.44581	186.04	-2.5195	2156.416734724	0.25911	9.0847	-13.193	0.6	0.60577	0.99855 (max o	3	6298; 3914; 4380	0.28193057	-0.7170684		49.0
	ZINC04347030	-7.6	355.342	-0.752	9	4	256.55	0.41645	134.55	-4.9901	2156.416734724	0.31094	6.4183	-2.4185	0.44	0.33971	0.88044 (max o	2	6368; 4118	0.38922587	0.591767		49.0
	ZINC77257395	-7.6	355.318	-3.0071	9	4	255.46	0.4295	148.74	-3.2919	2156.416734724	0.31094	8.6734	-9.6711	0.52	0.34593	0.98188 (max o	3	6607; 6295; 3356	0.23176435	-0.6571677		49.0
	ZINC35456639	-7.6	374.384	-1.0158	9	7	261.66	0.42689	160.07	-3.6927	2156.416734724	0.29898	6.6821	-3.3976	0.46154	0.44044	0.9464 (max of	3	6330; 6002; 5635	-0.2384629	0.8968869		49.0
	ZINC04073833	-7.6	398.478	-1.2136	9	1	310.86	0.3056	119.08	-65.767	2156.416734724	0.27762	6.8799	-4.3714	0.57143	0.64515	0.95401 (max o	6	6709; 6018; 5434; 20	0.47191304	-0.0435738		47.0
	ZINC08918273	-7.6	413.449	-3.0132	11	3	313.77	0.4182	170.96	-52.386	2156.416734724	0.26805	8.6795	-11.241	0.58621	0.58921	0.94785 (max o	7	7602; 6019; 5748; 56	0.523134	-0.1390281		47.0
	ZINC12865795	-7.6	442.531	-1.2338	10	3	343.37	0.33786	148.1	-57.405	2156.416734724	0.25076	6.9001	-4.9203	0.51613	0.61534	0.99016 (max o	11	6021; 6020; 6019; 60	0.43252838	-0.1098089		47.0
	ZINC04073787	-7.6	384.451	-1.4801	9	2	299.76	0.34331	127.87	-62.775	2156.416734724	0.28791	7.1464	-5.1409	0.59259	0.61502	0.95372 (max o	8	6422; 6384; 6020; 60	0.4514621	-0.0467490		47.0
	ZINC06596392	-7.6	412.505	-0.7592	9	1	324.62	0.29265	119.08	-61.023	2156.416734724	0.26805	6.4255	-2.8323	0.55172	0.65338	0.97649 (max o	4	6709; 5465; 5362; 53	0.4667198	-0.0057727		47.0
	ZINC04073151	-7.6	412.505	-0.7592	9	1	324.62	0.29265	119.08	-61.023	2156.416734724	0.26805	6.4255	-2.8323	0.55172	0.65338	0.97649 (max o	4	6709; 5465; 5434; 53	0.48752192	-0.0053235		47.0
	ZINC13518175	-7.6	448.057	-7.4795	11	3	230.04	0.5776	181.33	-25.795	2156.416734724	0.35334	13.146	-21.168	0.54545	0.57006	0.87379	1	7116	0.542745	0.8278107		41.0
	ZINC03943903	-7.6	445.355	-2.0792	11	5	298.01	0.45589	186.04	1.8339	2156.416734724	0.24292	7.7455	-8.5592	0.46875	0.28742	0.95709 (max o	13	2; 5; 12; 18; 26; 28; 3	-0.1207883	0.63666534		38.0
	ZINC20757971	-7.6	374.416	-0.579	8	2	284.31	0.32679	118.64	5.7836	2156.416734724	0.28791	6.2453	-2.0111	0.44444	0.5533	0.97658 (max o	10	7062; 6408; 6023; 59	0.29222107	0.6712789		36.0
	ZINC13548192	-7.6	376.388	-1.8191	9	3	278.4	0.38078	138.87	4.1721	2156.416734724	0.28791	7.4854	-6.3184	0.44444	0.54436	0.98806 (max o	9	7062; 6408; 6023; 59	0.2880031	0.7247099		36.0

Structure	Molecule Name	affinity	Total Molweight	cLogP	H-Acceptors	H-Donors	Total Surface Area	Relative PSA	Polar Surface Area	Druglikeness	Kd (nM) @ 20°C	LE from K...	LLE from Kd ...	LELP from Kd (n...	Shape Index	Molecular...	Neighbor Si...	Neig...	Neighbor	Neighbor ...	Neighbor ...	Ring Systems	frequency
	ZINC04514042	-7.6	370.389	-4.1819	11	4	251.58	0.59838	212.38	-0.65956	2156.416734724	0.31094	9.8482	-13.449	0.56	0.45626	0.96775 (max o	7	5788; 5728; 2766; 36	0.62065655	0.19738725		33.0
	ZINC03871403	-7.6	423.170	-13.388	15	2	266.13	0.71134	263.54	-22.95	2156.416734724	0.28791	19.054	-46.501	0.44444	0.4085	0.94605 (max o	9	6403; 6098; 6094; 57	0.6881442	0.48162407		33.0
	ZINC03871403	-7.6	424.178	-12.14	15	3	264.95	0.71006	260.71	-32.906	2156.416734724	0.28791	17.807	-42.168	0.44444	0.39014	0.94605 (max o	9	6404; 6099; 6095; 57	0.6660218	0.5219228		33.0
	ZINC08740077	-7.6	393.355	-0.2858	12	1	282.15	0.47553	157.75	1.9052	2156.416734724	0.27762	5.9521	-1.0295	0.42857	0.28503	0.93046 (max o	8	7478; 6965; 6179; 58	-0.6279782	0.33681482		33.0
	ZINC08876308	-7.6	443.455	-3.4862	10	1	330.97	0.33937	149.98	-1.7485	2156.416734724	0.24292	9.1525	-14.351	0.59375	0.51805	0.95549 (max o	9	1404; 2127; 2418; 27	0.1852567	-0.8922565		29.0
	ZINC04221599	-7.6	372.400	-5.6183	11	7	270.65	0.51565	201.45	5.6349	2156.416734724	0.29898	11.285	-18.792	0.53846	0.54851	0.92032 (max o	7	6189; 6029; 5771; 53	-0.5286881	-0.1556303		29.0
	ZINC04221600	-7.6	386.427	-7.2432	11	7	283.14	0.51671	212.48	4.0911	2156.416734724	0.28791	12.909	-25.158	0.51852	0.55009	0.92418 (max o	5	5771; 5720; 4538; 49	-0.5623129	-0.1373465		29.0
	ZINC05643780	-7.6	415.468	-1.797	9	4	325.9	0.35229	143.22	1.202	2156.416734724	0.25911	7.4633	-6.9352	0.53333	0.5887	0.97533 (max o	3	2475; 3266; 5430	-0.5827101	0.5833025		28.0
	ZINC05643756	-7.6	415.468	-1.797	9	4	325.9	0.35229	143.22	1.202	2156.416734724	0.25911	7.4633	-6.9352	0.53333	0.58835	0.97533 (max o	3	2475; 3266; 5431	-0.5668606	0.57282835		28.0
	ZINC20760890	-7.6	367.178	-2.3719	7	2	240.67	0.34566	103.26	-0.60035	2156.416734724	0.35334	8.0382	-6.7128	0.68182	0.6	0.94057 (max o	3	6032; 5580; 4848	-0.3003606	0.8975674		28.0
	ZINC20761471	-7.6	381.205	-1.9175	7	2	254.43	0.32697	103.26	-0.15442	2156.416734724	0.33798	7.5838	-5.6735	0.65217	0.59384	0.95111 (max o	3	6896; 6032; 5579	-0.2771644	0.9032737		28.0
	ZINC04899456	-7.6	430.547	-0.2461	8	4	341.68	0.30669	141.76	3.3527	2156.416734724	0.25076	5.9124	-0.98143	0.41935	0.53731		0		-0.7760001	-0.6111855		28.0
	ZINC04104828	-7.6	390.383	-1.9707	10	5	264.25	0.44859	155.14	-3.9213	2156.416734724	0.28791	7.637	-6.845	0.48148	0.37688	0.95201 (max o	3	2396; 3217; 4965	-0.5773974	-0.5686077		25.0
	ZINC67912141	-7.6	422.381	-3.7495	12	7	277.19	0.52217	195.6	-5.5578	2156.416734724	0.26805	9.4158	-13.988	0.44828	0.38765	0.94558 (max o	2	4432; 4858	0.77787477	-0.3558451		25.0
	ZINC12419756	-7.6	384.336	-0.9776	10	4	266.69	0.43286	144.14	-5.8493	2156.416734724	0.28791	6.6439	-3.3956	0.44444	0.42873	0.94605 (max o	6	6630; 6211; 2914; 38	-0.4409006	0.5295818		24.0
	ZINC03652836	-7.6	441.237	-1.685	10	5	270.77	0.43029	155.05	4.4029	2156.416734724	0.28791	7.3513	-5.8526	0.44444	0.45827	0.97407 (max o	13	6010; 5875; 1075; 16	0.4819808	0.79456687		24.0
	ZINC02004116	-7.6	396.786	-1.8042	10	5	267.56	0.43545	155.05	6.2533	2156.416734724	0.28791	7.4705	-6.2666	0.44444	0.45827	0.97658 (max o	13	6010; 5875; 5314; 10	0.46758312	0.81162643		24.0
	ZINC37867490	-7.6	380.372	-6.2025	12	8	237.3	0.5799	225	-2.0341	2156.416734724	0.29898	11.869	-20.746	0.53846	0.50398	0.86721 (max o	2	6016; 3651	0.59041035	0.79207903		23.0
	ZINC72320400	-7.6	406.470	-1.0841	9	7	287.33	0.38875	160.07	-4.0503	2156.416734724	0.27762	6.7504	-3.9049	0.5	0.44224	0.95148 (max o	7	6916; 6678; 6680; 63	0.04388237	-0.7647756		23.0
	ZINC08643751	-7.6	396.462	-2.586	9	5	317.04	0.35576	121.56	0.83312	2156.416734724	0.27762	8.2523	-9.3148	0.67857	0.59257	0.9428 (max of	9	7599; 3776; 4170; 41	0.39645332	-0.4180482		22.0
	ZINC20465420	-7.6	354.444	-1.6137	6	3	277.33	0.21956	60.61	2.5227	2156.416734724	0.31094	7.28	-5.1898	0.56	0.48596	0.90243 (max o	3	7051; 2231; 5565	-0.4159614	0.17171144		21.0
	ZINC20464737	-7.6	385.483	-0.678	8	3	274.42	0.3037	110.72	7.8715	2156.416734724	0.29898	6.3443	-2.2677	0.53846	0.5241	0.93686 (max o	3	7049; 3391; 4751	-0.3194917	0.132035		21.0
	ZINC20465417	-7.6	368.471	-0.973	6	3	273.23	0.1868	72.78	2.608	2156.416734724	0.29898	6.6393	-3.2544	0.57692	0.47705	0.93355 (max o	8	6818; 6587; 6585; 62	-0.4516815	0.14943801		21.0
	ZINC20464894	-7.6	392.498	-0.0634	8	4	299.81	0.26437	97.3	6.7244	2156.416734724	0.27762	5.7297	-0.22837	0.57143	0.53999	0.9427 (max of	3	6814; 4311; 4753	-0.3979270	0.16105953		21.0
	ZINC08740542	-7.6	385.383	1.8112	11	5	277.15	0.45939	163.93	1.3234	2156.416734724	0.27762	3.8551	6.524	0.5	0.33184	0.97288 (max o	12	6192; 6194; 2895; 33	-0.1751514	-0.6625179		20.0
	ZINC08740533	-7.6	419.828	2.4172	11	5	292.57	0.43518	163.93	1.3996	2156.416734724	0.26805	3.2491	9.0177	0.48276	0.31394	0.97649 (max o	10	6192; 5835; 5460; 25	-0.2061168	-0.6471178		20.0
	ZINC08740504	-7.6	401.382	1.4655	12	6	283.5	0.49531	184.16	1.3234	2156.416734724	0.26805	4.2008	5.4673	0.51724	0.33438	0.97878 (max o	14	6759; 6484; 6205; 61	-0.1947767	-0.6090043		20.0
	ZINC04556961	-7.6	356.323	-4.03	11	7	235.73	0.55869	178.53	-8.3431	2156.416734724	0.32389	9.6963	-12.442	0.5	0.45941	0.94718 (max o	8	6731; 6732; 6446; 64	0.5870312	-0.4605668		19.0
	ZINC04556960	-7.6	356.323	-4.03	11	7	235.73	0.55869	178.53	-8.3431	2156.416734724	0.32389	9.6963	-12.442	0.5	0.45757	0.94718 (max o	8	6731; 6732; 6446; 64	0.54389334	-0.4606847		19.0
	ZINC12604009	-7.6	383.443	0.1494	7	4	272.48	0.37126	128.48	2.3511	2156.416734724	0.29898	5.5169	0.4997	0.61538	0.42094	0.97533 (max o	11	7334; 1468; 2099; 25	-0.5130089	0.3698437		17.0
	ZINC03874217	-7.6	360.413	-1.5337	7	1	254.02	0.38347	127.2	11.036	2156.416734724	0.31094	7.2	-4.9325	0.52	0.39204	0.90896 (max o	5	2771; 3617; 4635; 49	0.07964218	-0.0694828		17.0
	ZINC03874217	-7.6	361.421	-1.5337	7	2	242.51	0.35421	128.81	10.469	2156.416734724	0.31094	7.2	-4.9325	0.52	0.39112	0.90896 (max o	3	2770; 4938; 5346	0.05127732	-0.0944236		17.0
	ZINC02100719	-7.6	355.389	0.3809	6	2	263.68	0.27002	103.27	0.93543	2156.416734724	0.29898	5.2854	1.274	0.5	0.45418	0.96438 (max o	5	6072; 4005; 4462; 44	0.7012643	0.6177425		16.0
	ZINC72320311	-7.6	353.433	-0.2864	6	2	262.54	0.29161	106.89	-1.0973	2156.416734724	0.31094	5.9527	-0.92108	0.44	0.46819	0.93686 (max o	3	7573; 6915; 6377	0.38824293	-0.7552306		16.0
	ZINC04258780	-7.6	365.408	-0.4944	6	0	271.12	0.22315	80.75	2.0243	2156.416734724	0.28791	6.1607	-1.7172	0.51852	0.46733	0.95174 (max o	5	5778; 1180; 2464; 45	-0.4780148	-0.2929893		14.0
	ZINC04237367	-7.6	411.433	-1.5657	8	0	303.38	0.26534	99.21	1.508	2156.416734724	0.25911	7.232	-6.0425	0.56667	0.47712	0.97304 (max o	8	5778; 5777; 1142; 45	-0.4954555	-0.2769982		14.0
	ZINC20463677	-7.6	405.517	-1.5757	8	2	297.34	0.21702	82.09	6.8803	2156.416734724	0.26805	7.242	-5.8784	0.58621	0.50161	0.93686 (max o	7	7384; 7346; 6765; 65	0.3985538	-0.2188772		14.0
	ZINC08918216	-7.6	446.522	-0.4444	9	1	347.36	0.27349	119.08	-54.529	2156.416734724	0.24292	6.1107	-1.8294	0.5	0.59492	0.92876 (max o	4	6709; 3310; 5362; 54	0.45410785	0.01280598		14.0
	ZINC08743671	-7.6	423.939	0.1648	7	2	322.53	0.257	117.18	4.8616	2156.416734724	0.26805	5.5015	0.61481	0.51724	0.56899	0.86904 (max o	4	6196; 6025; 5469; 52	0.6541973	0.31392458		14.0
	ZINC08635292	-7.6	352.457	-2.8249	8	3	283.89	0.33111	93.71	-1.6975	2156.416734724	0.31094	8.4912	-9.0851	0.64	0.57252	0.9654 (max of	2	6751; 3275	0.26739436	0.23451655		14.0
	ZINC20463978	-7.6	395.478	-2.0427	9	4	310.74	0.32246	96.73	3.3803	2156.416734724	0.27762	7.709	-7.3578	0.57143	0.5364	0.95709 (max o	6	6809; 6810; 6565; 59	-0.7254427	-0.2704556		14.0
	ZINC12483712	-7.6	361.420	-1.0243	8	4	287.63	0.3484	108.05	-4.8125	2156.416734724	0.29898	6.6906	-3.426	0.53846	0.49149	0.92032 (max o	3	6994; 6993; 6767	0.8866512	0.28979632		14.0
	ZINC05414618	-7.6	383.447	-1.2639	10	3	293.08	0.35362	120.08	6.2697	2156.416734724	0.28791	6.9302	-4.39	0.48148	0.57201	0.96064 (max o	3	5815; 2202; 5011	-0.1125393	0.27457297		13.0

Structure	Molecule Name	affinity	Total Molweight	cLogP	H-Acceptors	H-Donors	Total Surface Area	Relative PSA	Polar Surface Area	Druglikeness	Kd (nM) @ 20°C	LE from K...	LLE from Kd ...	LELP from Kd (n...	Shape Index	Molecular...	Neighbor Si...	Neig...	Neighbor	Neighbor ...	Neighbor ...	Ring Systems	frequency
	ZINC04270785	-7.6	356.398	0.4682	8	0	255	0.32063	101.6	4.1094	2156.416734724	0.32389	5.1981	1.4455	0.54167	0.60089	0.88866	1	7145	0.16130206	0.4254217		13.0
	ZINC08481005	-7.6	405.493	-0.8551	8	3	313.46	0.30623	137.41	6.1749	2156.416734724	0.26805	6.5214	-3.1901	0.62069	0.52259	0.97415 (max o	4	5490; 3638; 4663; 53	0.26606038	0.32017875		12.0
	ZINC04083303	-7.6	389.494	-0.5094	7	2	307.11	0.2699	117.18	5.1784	2156.416734724	0.27762	6.1757	-1.8349	0.60714	0.52092	0.93985 (max o	6	6946; 6121; 5490; 20	0.32306105	0.31307426		12.0
	ZINC04082427	-7.6	405.493	-0.8551	8	3	313.46	0.30623	137.41	6.1749	2156.416734724	0.26805	6.5214	-3.1901	0.62069	0.52259	0.97415 (max o	4	6121; 5448; 3638; 46	0.24792247	0.33273667		12.0
	ZINC12871402	-7.6	389.494	-0.5094	7	2	307.11	0.2699	117.18	6.1636	2156.416734724	0.27762	6.1757	-1.8349	0.60714	0.51663	0.97288 (max o	8	6946; 6121; 3637; 36	0.2882041	0.34830922		12.0
	ZINC04088003	-7.6	376.388	-2.5875	9	2	286.07	0.35974	127.87	6.3279	2156.416734724	0.28791	8.2538	-8.9873	0.66667	0.49945	0.96617 (max o	6	5756; 5752; 2413; 35	0.5467421	0.2811914		12.0
	ZINC04088009	-7.6	376.388	-2.5875	9	2	286.07	0.35974	127.87	6.3279	2156.416734724	0.28791	8.2538	-8.9873	0.66667	0.49945	0.96617 (max o	6	6714; 6425; 6022; 57	0.5653911	0.26959577		12.0
	ZINC12603858	-7.6	436.935	-2.2234	9	5	321.03	0.36859	137.78	3.1396	2156.416734724	0.27762	7.8897	-8.0087	0.64286	0.58969	0.97773 (max o	4	6187; 5830; 3332; 50	0.28620303	-0.2958398		12.0
	ZINC38664852	-7.6	421.267	-8.9493	14	8	261.12	0.6744	248.7	-32.77	2156.416734724	0.28791	14.616	-31.084	0.48148	0.51117	0.95201 (max o	4	6027; 3947; 5650; 56	0.02471457	-0.4564955		12.0
	ZINC38664850	-7.6	421.267	-8.9493	14	8	261.12	0.6744	248.7	-32.77	2156.416734724	0.28791	14.616	-31.084	0.48148	0.5106	0.95201 (max o	4	6027; 5652; 3947; 56	0.06951206	-0.4490777		12.0
	ZINC38664850	-7.6	420.259	-10.197	14	7	262.3	0.67587	251.53	-27.673	2156.416734724	0.28791	15.863	-35.418	0.48148	0.52676	0.95201 (max o	5	6026; 5791; 5651; 56	0.05216133	-0.4892377		12.0
	ZINC38664852	-7.6	420.259	-10.197	14	7	262.3	0.67587	251.53	-27.673	2156.416734724	0.28791	15.863	-35.418	0.48148	0.52732	0.95201 (max o	5	7696; 6026; 3946; 56	0.01886524	-0.4774820		12.0
	ZINC08643651	-7.6	366.412	-1.1075	8	3	279.28	0.31255	108.33	3.7849	2156.416734724	0.29898	6.7738	-3.7043	0.65385	0.6191	0.91351 (max o	6	1515; 2117; 2923; 46	0.4721618	-0.4027071		12.0
	ZINC12603647	-7.6	440.447	-1.8861	11	4	331.95	0.38632	152.65	-2.823	2156.416734724	0.25076	7.5524	-7.5217	0.70968	0.58492	0.95267 (max o	3	6241; 1797; 4182	-0.3486122	-0.2244975		12.0
	ZINC15675164	-7.6	412.413	-0.714	9	4	306.04	0.35368	134.19	-9.0846	2156.416734724	0.26805	6.3803	-2.6637	0.68966	0.58705	0.94823 (max o	4	6241; 2922; 4182; 54	-0.3878550	-0.2450820		12.0
	ZINC08624492	-7.6	370.376	-1.2732	8	4	274.53	0.34677	117.12	-0.30707	2156.416734724	0.29898	6.9395	-4.2585	0.69231	0.57286	0.94008 (max o	3	6511; 5519; 2922	-0.3716188	-0.2581759		12.0
	ZINC13545062	-7.6	383.349	-4.4475	12	8	254.94	0.57347	198.4	-4.7796	2156.416734724	0.29898	10.114	-14.876	0.46154	0.46983	0.94008 (max o	3	7010; 6531; 6428	0.19296663	0.73158574		12.0
	ZINC14442630	-7.6	434.396	-1.4258	10	8	285	0.44856	188.14	-2.6646	2156.416734724	0.25076	7.0921	-5.686	0.35484	0.35923	0.96018 (max o	8	6314; 1044; 1296; 33	-0.5319835	0.5745749		11.0
	ZINC72320090	-7.6	382.404	-1.8946	10	6	269.16	0.44063	158.3	-4.5161	2156.416734724	0.29898	7.5609	-6.3369	0.57692	0.55602	0.87363 (max o	5	419; 1287; 1589; 264	-0.4188330	0.38765684		11.0
	ZINC31169041	-7.6	354.350	-2.5673	10	6	244.4	0.48527	158.3	-5.0334	2156.416734724	0.32389	8.2336	-7.9264	0.58333	0.54319	0.93046 (max o	4	7421; 6043; 5681; 10	-0.4162884	0.36511162		11.0
	ZINC03985311	-7.6	365.453	-0.9071	6	1	248.34	0.43694	153.64	2.7368	2156.416734724	0.32389	6.5734	-2.8006	0.45833	0.255	0.92315 (max o	7	6409; 1402; 2412; 37	0.3605008	-0.6884372		10.0
	ZINC08792088	-7.6	420.469	-1.6578	10	3	306.22	0.41898	164.92	8.1063	2156.416734724	0.26805	7.3241	-6.1847	0.48276	0.49258		0		-0.6235523	-0.7570021		10.0
	ZINC20464737	-7.6	386.491	-2.7877	8	4	288.46	0.33759	111.92	5.189	2156.416734724	0.29898	8.454	-9.3241	0.53846	0.52943	0.95857 (max o	6	7050; 6815; 6265; 59	-0.3217117	-0.5658432		10.0
	ZINC13660042	-7.6	390.471	-0.232	8	6	282.24	0.34935	139.84	-2.1613	2156.416734724	0.28791	5.8983	-0.80582	0.51852	0.46372	0.97407 (max o	5	2964; 2965; 3361; 46	-0.3777792	0.19190112		10.0
	ZINC13660040	-7.6	390.471	-0.232	8	6	282.24	0.34935	139.84	-2.1613	2156.416734724	0.28791	5.8983	-0.80582	0.51852	0.45343	0.97407 (max o	5	5509; 2964; 2965; 33	-0.3982507	0.20349163		10.0
	ZINC35454662	-7.6	390.471	-0.232	8	6	282.24	0.34935	139.84	-2.1613	2156.416734724	0.28791	5.8983	-0.80582	0.51852	0.45759	0.96343 (max o	8	3067; 4671; 4672; 48	-0.3227128	0.20873986		10.0
	ZINC72319868	-7.6	406.470	-1.0554	9	7	286.5	0.38988	160.07	-1.8323	2156.416734724	0.27762	6.7217	-3.8016	0.5	0.57544	0.97288 (max o	3	4876; 4877; 5276	-0.3604579	0.24667068		10.0
	ZINC31158769	-7.6	386.439	-0.2961	8	5	283.18	0.34798	136.68	-4.6035	2156.416734724	0.28791	5.9624	-1.0285	0.48148	0.5404	0.99202 (max o	5	5970; 5969; 3927; 48	-0.0523939	0.94318813		10.0
	ZINC12658809	-7.6	367.385	-4.2278	11	5	269.44	0.46129	136.88	2.9437	2156.416734724	0.29898	9.8941	-14.141	0.46154	0.41265	0.94008 (max o	5	6492; 5864; 4191; 42	-0.6294038	-0.5026828		8.0
	ZINC35464913	-7.6	402.438	-1.1195	9	6	287.44	0.38839	156.91	-9.0196	2156.416734724	0.27762	6.7858	-4.0324	0.5	0.46405	0.97773 (max o	6	3047; 4381; 4416; 47	0.6710599	-0.1817033		8.0
	ZINC31154896	-7.6	386.439	-0.1928	8	5	279.59	0.35244	136.68	-3.8197	2156.416734724	0.28791	5.8591	-0.66967	0.51852	0.46384	0.97407 (max o	5	4381; 4416; 4789; 52	0.6316173	-0.1749365		8.0
	ZINC20463877	-7.6	363.456	-3.9887	7	4	275.02	0.3154	102.83	2.3408	2156.416734724	0.29898	9.655	-13.341	0.57692	0.46057	0.92394 (max o	5	7043; 6221; 3389; 37	0.57155263	-0.1308602		8.0
	ZINC20463880	-7.6	393.482	-4.0587	8	4	297.28	0.32542	112.06	2.4571	2156.416734724	0.27762	9.725	-14.619	0.60714	0.47848	0.93686 (max o	6	7176; 7043; 6221; 33	0.5584906	-0.1158135		8.0
	ZINC35455011	-7.6	396.431	-1.2041	10	6	285.68	0.41515	158.3	-17.323	2156.416734724	0.28791	6.8704	-4.1823	0.62963	0.53653	0.95493 (max o	8	6325; 530; 3492; 440	-0.4514699	0.70387393		8.0
	ZINC04042893	-7.6	395.455	-1.4997	8	2	294.7	0.36987	144.3	-7.167	2156.416734724	0.28791	7.166	-5.209	0.51852	0.52885	0.95201 (max o	4	7133; 6417; 6418; 51	-0.7725583	0.34372044		8.0
	ZINC04544768	-7.6	370.309	-1.2533	10	5	250.78	0.47269	155.14	-5.8493	2156.416734724	0.29898	6.9196	-4.1919	0.42308	0.4171	0.9615 (max of	10	6430; 5471; 2914; 33	-0.4159157	0.50330126		8.0
	ZINC49181947	-7.6	422.428	1.04	9	4	302.11	0.34901	134.91	-7.8186	2156.416734724	0.25911	4.6263	4.0137	0.46667	0.46379		0		-0.801299	0.4759774		8.0
	ZINC35456155	-7.6	355.362	-2.4289	8	4	260.48	0.38283	139.51	-4.6528	2156.416734724	0.31094	8.0952	-7.8115	0.52	0.53864	0.98434 (max o	7	7086; 6532; 6237; 62	0.66531706	-0.3373885		8.0
	ZINC35456150	-7.6	355.362	-2.4289	8	4	260.48	0.38283	139.51	-4.6528	2156.416734724	0.31094	8.0952	-7.8115	0.52	0.5396	0.98434 (max o	7	7086; 6532; 6237; 62	0.6889262	-0.3133829		8.0
	ZINC12660221	-7.6	436.507	-1.659	9	2	329.82	0.36505	153.17	2.1105	2156.416734724	0.25911	7.3253	-6.4026	0.6	0.5447	0.9707 (max of	3	4660; 5068; 5069	-0.7283017	0.6402957		8.0
	ZINC02139975	-7.6	377.415	-0.9394	8	2	287.38	0.31843	119	-1.1305	2156.416734724	0.28791	6.6057	-3.2629	0.59259	0.57355	0.95493 (max o	3	5705; 5704; 5310	-0.8185258	0.46287322		8.0
	ZINC02139972	-7.6	377.415	-0.9394	8	2	287.38	0.31843	119	-1.1305	2156.416734724	0.28791	6.6057	-3.2629	0.59259	0.57337	0.95493 (max o	3	5705; 5704; 5311	-0.8137604	0.43612245		8.0
	ZINC35456631	-7.6	374.384	-1.0158	9	7	261.66	0.42689	160.07	-3.6927	2156.416734724	0.29898	6.6821	-3.3976	0.46154	0.44399	0.9464 (max of	3	6330; 6002; 5636	-0.2023588	0.88663566		8.0

Structure	Molecule Name	affinity	Total Molweight	cLogP	H-Acceptors	H-Donors	Total Surface Area	Relative PSA	Polar Surface Area	Druglikeness	Kd (nM) @ 20°C	LE from K...	LLE from Kd ...	LELP from Kd (n...	Shape Index	Molecular...	Neighbor Si...	Neig...	Neighbor	Neighbor ...	Neighbor ...	Ring Systems	frequency
	ZINC03812863	-7.6	405.493	-3.2679	8	2	315.18	0.2965	144.82	2.4951	2156.416734724	0.26805	8.9342	-12.191	0.48276	0.53097	0.92507 (max o	3	6941; 6706; 6103	-0.8134004	-0.4475203		8.0
	ZINC15676494	-7.6	447.917	-0.2885	8	1	321.5	0.22961	105.81	0.71917	2156.416734724	0.25076	5.9548	-1.1505	0.51613	0.57624	0.95243 (max o	4	7613; 6789; 6317; 58	-0.3004880	0.3892707		8.0
	ZINC31156459	-7.6	392.487	-0.0831	8	6	286.85	0.34373	139.84	-10.396	2156.416734724	0.28791	5.7494	-0.28864	0.48148	0.56222	0.99202 (max o	7	7261; 6879; 6642; 66	-0.7034465	0.23377614		8.0
	ZINC05433921	-7.6	360.369	-0.6678	9	2	267.44	0.38143	107.74	2.1526	2156.416734724	0.29898	6.3341	-2.2336	0.61538	0.35147	0.93379 (max o	7	6976; 594; 1749; 370	-0.6555142	-0.5606893		7.0
	ZINC77257444	-7.6	442.456	-3.1267	12	8	305.2	0.47444	198.76	-4.7867	2156.416734724	0.25911	8.793	-12.067	0.53333	0.5664	0.98188 (max o	6	1290; 2639; 2640; 34	-0.6717265	0.482126		7.0
	ZINC03843351	-7.6	356.285	-2.2012	8	0	266.6	0.37779	132.86	-7.8406	2156.416734724	0.29898	7.8675	-7.3624	0.53846	0.51578		0		-0.2817212	-0.8935205		7.0
	ZINC31157187	-7.6	381.400	-2.088	8	2	266.7	0.37345	133.19	-8.3912	2156.416734724	0.28791	7.7543	-7.2524	0.44444	0.4277	0.97407 (max o	4	6856; 3452; 4363; 43	-0.4845104	0.5879553		7.0
	ZINC02115249	-7.6	367.400	-0.278	6	2	274.1	0.25976	103.27	-1.389	2156.416734724	0.28791	5.9443	-0.96559	0.51852	0.51018	0.91059 (max o	5	6379; 6074; 5703; 31	-0.0781647	0.6940613		7.0
	ZINC15708861	-7.6	405.434	-3.1741	11	3	311.31	0.40574	156.45	5.2013	2156.416734724	0.26805	8.8404	-11.841	0.48276	0.50657	0.85007 (max o	2	7317; 6962	-0.0490744	0.02782851		7.0
	ZINC12901484	-7.6	390.439	-1.7225	8	1	286.58	0.3748	136.43	5.5197	2156.416734724	0.28791	7.3888	-5.9829	0.59259	0.47527	0.93069 (max o	7	778; 964; 965; 966; 2	0.15189318	0.5182215		6.0
	ZINC14442636	-7.6	434.396	-1.4258	10	8	285	0.44856	188.14	-2.6646	2156.416734724	0.25076	7.0921	-5.686	0.35484	0.3335	0.96018 (max o	7	6314; 1044; 1296; 38	-0.551304	0.5633112		6.0
	ZINC20465122	-7.6	368.427	-2.6183	7	4	283.98	0.28819	86.47	2.2344	2156.416734724	0.29898	8.2846	-8.7575	0.53846	0.52135	0.9302 (max of	6	6819; 6816; 5935; 59	-0.322362	0.02717093		6.0
	ZINC13333976	-7.6	388.455	-0.0883	8	5	281.14	0.3505	136.68	-3.7924	2156.416734724	0.28791	5.7546	-0.3067	0.51852	0.45612	0.96617 (max o	8	5628; 3067; 3361; 37	-0.3173618	0.18911438		6.0
	ZINC05433905	-7.6	391.446	-1.4981	9	2	295.72	0.35581	125.5	-6.2582	2156.416734724	0.27762	7.1644	-5.3962	0.60714	0.42753	0.97837 (max o	6	6976; 6459; 1749; 32	-0.6357907	-0.5588004		6.0
	ZINC03984241	-7.6	398.442	-0.5003	9	1	288.18	0.23596	82.56	2.8517	2156.416734724	0.26805	6.1666	-1.8664	0.48276	0.39037	0.9543 (max of	5	7285; 4062; 4204; 46	0.533614	0.6628405		6.0
	ZINC14616882	-7.6	422.469	-0.5759	10	6	308.96	0.38387	158.3	-18.912	2156.416734724	0.26805	6.2422	-2.1485	0.58621	0.53336	0.97415 (max o	5	5881; 4259; 4680; 46	-0.4093166	0.6613358		6.0
	ZINC14438552	-7.6	396.431	-1.2041	10	6	285.68	0.41515	158.3	-17.323	2156.416734724	0.28791	6.8704	-4.1823	0.62963	0.54567	0.9578 (max of	8	6238; 5880; 5881; 55	-0.3975261	0.68654305		6.0
	ZINC34235759	-7.6	388.324	-6.5696	11	4	269.23	0.50901	188.87	-5.5881	2156.416734724	0.28791	12.236	-22.819	0.44444	0.48967	0.96343 (max o	3	6632; 4389; 5219	-0.7913505	0.53603303		6.0
	ZINC35456329	-7.6	360.314	-1.1781	10	5	250.04	0.47408	155.14	-4.135	2156.416734724	0.31094	6.8444	-3.7889	0.52	0.41908	0.94975 (max o	6	6347; 6001; 4802; 51	-0.6772321	-0.1160215		6.0
	ZINC31158094	-7.6	388.324	-6.5696	11	4	269.23	0.50901	188.87	-5.5881	2156.416734724	0.28791	12.236	-22.819	0.44444	0.50591	0.95493 (max o	3	6612; 5968; 4367	0.7734853	-0.2370154		6.0
	ZINC03831541	-7.6	382.416	-3.6789	8	1	252.44	0.52916	183.21	11.228	2156.416734724	0.31094	9.3452	-11.832	0.48	0.33609	0.98188 (max o	6	6978; 6961; 6491; 35	-0.3906413	-0.4694702		6.0
	ZINC20465014	-7.6	351.421	-2.0765	7	4	277.33	0.31836	92.46	1.2407	2156.416734724	0.31094	7.7428	-6.6782	0.56	0.50915	0.90599 (max o	4	6572; 6264; 5567; 47	0.6884767	0.38112783		6.0
	ZINC31167404	-7.6	417.456	-1.3541	8	0	322.92	0.28252	119.03	4.2354	2156.416734724	0.25911	7.0204	-5.2259	0.43333	0.51674	0.96092 (max o	5	7245; 6863; 6616; 66	-0.0330354	0.8929717		6.0
	ZINC31157455	-7.6	431.369	-5.0212	12	5	289.48	0.50387	195.27	-2.1759	2156.416734724	0.25911	10.687	-19.378	0.43333	0.40785	0.91413	1	5971	-0.7032073	0.69244206		6.0
	ZINC04102166	-7.6	373.333	-4.6537	10	5	250.73	0.48985	168.97	-2.6261	2156.416734724	0.29898	10.32	-15.565	0.46154	0.39662		0		-0.8859313	0.3118334		6.0
	ZINC39962693	-7.6	412.509	-0.7544	10	3	314.12	0.31809	124.66	2.4253	2156.416734724	0.26805	6.4207	-2.8144	0.44828	0.57408	0.94823 (max o	5	7697; 7328; 6660; 58	-0.1127380	0.33787915		6.0
	ZINC05398662	-7.6	405.494	-1.6871	10	2	288.73	0.3602	138.08	2.8203	2156.416734724	0.28791	7.3534	-5.8599	0.51852	0.5535	0.90776 (max o	2	7697; 6164	-0.1319047	0.34778744		6.0
	ZINC08298079	-7.6	350.441	-2.9422	8	1	279.23	0.29721	73.92	0.26159	2156.416734724	0.31094	8.6085	-9.4623	0.64	0.5258	0.98188 (max o	8	5446; 1760; 2103; 54	0.2057896	0.22181256		5.0
	ZINC08299245	-7.6	350.465	-4.6411	8	2	276.97	0.29148	81.3	0.32375	2156.416734724	0.31094	10.307	-14.926	0.64	0.52506	0.9654 (max of	7	5719; 1760; 4154; 41	0.21618897	0.26720333		5.0
	ZINC20464728	-7.6	381.427	-0.7949	9	4	284.75	0.33612	112.52	3.1071	2156.416734724	0.28791	6.4612	-2.761	0.59259	0.55967	0.9339 (max of	5	2995; 3393; 4734; 47	-0.3790402	-0.0595414		5.0
	ZINC20465122	-7.6	367.419	-0.5086	7	3	269.94	0.25117	85.27	6.2974	2156.416734724	0.29898	6.1749	-1.7011	0.53846	0.52135	0.92064 (max o	4	5934; 5932; 2590; 47	-0.3974843	0.05198717		5.0
	ZINC03830477	-7.6	382.400	-2.5815	10	2	258.21	0.59645	203.58	12.827	2156.416734724	0.31094	8.2478	-8.3023	0.52	0.37714	0.97118 (max o	5	2769; 3618; 4187; 49	-0.3751692	0.32104492		5.0
	ZINC03830453	-7.6	426.453	-2.6401	11	2	292.44	0.56083	212.81	16.217	2156.416734724	0.27762	8.3064	-9.5097	0.53571	0.40226	0.93058 (max o	3	4060; 4968; 5322	-0.3558657	0.3095777		5.0
	ZINC20504118	-7.6	409.529	-1.7237	8	5	314.99	0.377	132.92	4.6993	2156.416734724	0.27762	7.39	-6.2088	0.60714	0.42881	0.93069 (max o	5	6273; 5944; 3010; 51	0.47878253	0.21282147		5.0
	ZINC08298082	-7.6	350.441	-2.9422	8	1	279.23	0.29721	73.92	0.26159	2156.416734724	0.31094	8.6085	-9.4623	0.64	0.5258	0.98188 (max o	8	2104; 3275; 3280; 37	0.23139991	0.25463504		5.0
	ZINC08298374	-7.6	386.498	-3.3887	8	4	321.56	0.31593	88.92	0.15993	2156.416734724	0.27762	9.055	-12.206	0.60714	0.49117	0.97037 (max o	5	3281; 3718; 4159; 41	-0.1332544	-0.0593618		5.0
	ZINC04534182	-7.6	374.186	0.0892	7	5	224.22	0.38529	115.17	-5.5938	2156.416734724	0.35334	5.5771	0.25245	0.5	0.24235	0.92913 (max o	5	6154; 6155; 5790; 46	-0.4464238	0.59314126		5.0
	ZINC05398396	-7.6	360.432	0.7518	7	1	271.43	0.23564	83.53	2.6677	2156.416734724	0.29898	4.9145	2.5146	0.53846	0.52017	0.91007 (max o	2	2065; 5401	0.06435052	0.397245		5.0
	ZINC04270797	-7.6	390.458	0.6818	8	1	293.69	0.25183	92.76	2.6677	2156.416734724	0.27762	4.9845	2.4558	0.57143	0.54491	0.91838 (max o	3	6436; 5401; 5338	0.12090759	0.38366798		5.0
	ZINC03842121	-7.6	403.501	0.6482	8	1	306.25	0.22044	86.77	3.3828	2156.416734724	0.26805	5.0181	2.4182	0.55172	0.56148	0.91248 (max o	4	7582; 6936; 5401; 54	0.1217807	0.36109436		5.0
	ZINC20464731	-7.6	402.466	-1.1022	9	3	284.12	0.35122	125.94	3.6498	2156.416734724	0.28791	6.7685	-3.8283	0.55556	0.54453	0.93046 (max o	7	3843; 4298; 4734; 47	-0.4192939	-0.0732009		5.0
	ZINC20465140	-7.6	385.483	-0.9228	8	3	274.42	0.3037	110.72	0.54912	2156.416734724	0.29898	6.5891	-3.0865	0.53846	0.5239	0.94002 (max o	7	6267; 3844; 3846; 43	-0.4145617	-0.0506403		5.0
	ZINC20465140	-7.6	386.491	-3.0325	8	4	288.46	0.33759	111.92	-3.4712	2156.416734724	0.29898	8.6988	-10.143	0.53846	0.5239	0.94002 (max o	6	6817; 3847; 4304; 43	-0.369241	-0.1145617		5.0

Structure	Molecule Name	affinity	Total Molweight	cLogP	H-Acceptors	H-Donors	Total Surface Area	Relative PSA	Polar Surface Area	Druglikeness	Kd (nM) @ 20°C	LE from K...	LLE from Kd ...	LELP from Kd (n...	Shape Index	Molecular...	Neighbor Si...	Neig...	Neighbor	Neighbor ...	Neighbor ...	Ring Systems	frequency
	ZINC08298371	-7.6	386.498	-3.3887	8	4	321.56	0.31593	88.92	0.15993	2156.416734724	0.27762	9.055	-12.206	0.60714	0.49117	0.97037 (max o	5	5442; 3281; 4165; 45	-0.1055454	-0.0490136		5.0
	ZINC08299279	-7.6	356.472	-3.1679	7	3	286.35	0.27463	80.86	0.25153	2156.416734724	0.29898	8.8342	-10.596	0.65385	0.47862	0.96721 (max o	5	4153; 4588; 4597; 50	-0.1061026	-0.0061471		5.0
	ZINC04534215	-7.6	408.631	0.6952	7	5	239.64	0.3605	115.17	-5.48	2156.416734724	0.33798	4.9711	2.057	0.47826	0.24519	0.92947 (max o	5	6081; 5790; 3613; 49	-0.4462219	0.6363312		5.0
	ZINC01045361	-7.6	408.631	0.6952	7	5	239.64	0.3605	115.17	-5.48	2156.416734724	0.33798	4.9711	2.057	0.47826	0.24519	0.92947 (max o	5	6154; 6081; 5790; 54	-0.4851699	0.6106433		5.0
	ZINC13139345	-7.6	394.375	-0.4815	9	5	272.76	0.40908	153.75	-2.8773	2156.416734724	0.27762	6.1478	-1.7344	0.42857	0.44471	0.95148 (max o	6	5809; 5760; 2479; 54	-0.8058652	0.41777128		5.0
	ZINC05890346	-7.6	394.375	-0.4815	9	5	272.76	0.40908	153.75	-2.8773	2156.416734724	0.27762	6.1478	-1.7344	0.42857	0.44532	0.95148 (max o	6	5809; 5760; 5496; 54	-0.7982999	0.39963642		5.0
	ZINC04097368	-7.6	383.349	-4.4475	12	8	254.94	0.57347	198.4	-4.7202	2156.416734724	0.29898	10.114	-14.876	0.42308	0.46983	0.94951 (max o	7	7831; 7010; 6958; 67	0.22555421	0.71482164		5.0
	ZINC31159278	-7.6	360.314	-1.1781	10	5	250.04	0.47408	155.14	-4.135	2156.416734724	0.31094	6.8444	-3.7889	0.52	0.40903	0.95 (max of 6)	6	6001; 5882; 5634; 48	-0.7310988	-0.1208652		5.0
	ZINC32123826	-7.6	417.441	-0.9642	10	3	304.95	0.32176	124.1	-0.11909	2156.416734724	0.25911	6.6305	-3.7211	0.5	0.55244	0.95837 (max o	7	7424; 6308; 5990; 59	-0.6286391	0.14916158		5.0
	ZINC49600306	-7.6	363.341	-1.4804	8	3	270.63	0.35702	128.51	-0.3333	2156.416734724	0.29898	7.1467	-4.9515	0.57692	0.51407	0.9615 (max of	3	5258; 5259; 5260	0.35290167	-0.8480167		5.0
	ZINC03873057	-7.6	375.421	-4.4053	9	4	267.35	0.49766	181.16	-2.7923	2156.416734724	0.31094	10.072	-14.168	0.44	0.65461	0.91611 (max o	4	7678; 7188; 6102; 57	-0.8513942	0.01877900		5.0
	ZINC61997000	-7.6	395.562	-0.3464	6	3	305.79	0.19795	86.61	-4.4229	2156.416734724	0.27762	6.0127	-1.2477	0.57143	0.55438	0.94573 (max o	3	7272; 7102; 6901	-0.6830851	0.23753454		5.0
	ZINC61997000	-7.6	395.562	-0.488	6	4	323.55	0.24197	83.23	-3.5843	2156.416734724	0.27762	6.1543	-1.7578	0.57143	0.58716	0.94573 (max o	3	7273; 7103; 6902	-0.3814021	0.88251543		5.0
	ZINC35458405	-7.6	432.511	-1.9041	7	1	310.36	0.30229	134.63	0.74206	2156.416734724	0.25076	7.5704	-7.5934	0.3871	0.4634	0.93956 (max o	4	6645; 1917; 5201; 52	-0.4705260	0.42005473		4.0
	ZINC20464728	-7.6	382.435	-2.9046	9	5	298.79	0.36731	113.72	0.20551	2156.416734724	0.28791	8.5709	-10.089	0.59259	0.56165	0.91768 (max o	5	2226; 2996; 4735; 47	0.641332	0.3568232		4.0
	ZINC67903437	-7.6	430.536	0.8705	8	4	305.55	0.32744	121.14	-5.1488	2156.416734724	0.25911	4.7958	3.3595	0.43333	0.49097	0.96589 (max o	3	2684; 3538; 5263	0.60853034	0.5837908		4.0
	ZINC20504118	-7.6	409.529	-0.2881	8	5	289.44	0.322	133.33	6.8098	2156.416734724	0.27762	5.9544	-1.0377	0.60714	0.48852	0.90236 (max o	2	1006; 5574	-0.6397339	0.70591545		4.0
	ZINC20504118	-7.6	410.537	-1.6544	8	6	303.48	0.35337	134.53	4.4561	2156.416734724	0.27762	7.3207	-5.9592	0.60714	0.49066	0.92418 (max o	4	6272; 1253; 5576; 30	-0.6560345	0.71275777		4.0
	ZINC08927234	-7.6	384.364	-4.7824	10	1	268.54	0.50741	181.27	4.9262	2156.416734724	0.29898	10.449	-15.996	0.61538	0.49233	0.93686 (max o	6	6204; 6203; 5839; 31	0.44243237	0.5067172		4.0
	ZINC04270012	-7.6	405.331	-6.081	12	6	260.53	0.57176	206.27	-2.1079	2156.416734724	0.27762	11.747	-21.904	0.42857	0.38316	0.93985 (max o	3	2443; 2444; 3236	-0.1278507	-0.8487412		4.0
	ZINC05397712	-7.6	397.451	-0.2122	9	1	286.71	0.33574	121.47	-4.7636	2156.416734724	0.28791	5.8785	-0.73705	0.48148	0.55356	0.90441 (max o	3	7692; 5800; 3684	0.16077062	0.40318444		4.0
	ZINC03830264	-7.6	434.429	-4.7376	13	3	285.61	0.62393	241.03	9.3538	2156.416734724	0.27762	10.404	-17.065	0.46429	0.48641	0.8991 (max of	2	6248; 3768	-0.5580936	-0.6457564		4.0
	ZINC30879694	-7.6	407.509	-0.9494	7	4	310.14	0.30254	108.68	-5.2701	2156.416734724	0.27762	6.6157	-3.4197	0.53571	0.53948	0.94558 (max o	6	7411; 7398; 5941; 38	0.44259143	-0.3359125		4.0
	ZINC35454987	-7.6	387.403	-2.5561	9	4	285.25	0.3953	156.58	-5.0739	2156.416734724	0.28791	8.2224	-8.8783	0.59259	0.63438	0.94605 (max o	7	5965; 3447; 3888; 38	-0.9174313	0.08282257		4.0
	ZINC35464603	-7.6	375.443	-1.0588	7	1	289.31	0.2544	100.98	7.6353	2156.416734724	0.28791	6.7251	-3.6776	0.51852	0.63637	0.96617 (max o	3	6885; 3932; 5240	-0.1729070	0.90926105		4.0
	ZINC35465641	-7.6	358.298	-1.0155	10	6	241.05	0.51724	173.98	-0.59921	2156.416734724	0.31094	6.6818	-3.2659	0.52	0.41264	0.97118 (max o	3	3935; 4838; 4839	-0.0736156	0.79290104		4.0
	ZINC12653223	-7.6	366.377	-1.4884	11	5	253.33	0.48147	158.46	1.1585	2156.416734724	0.29898	7.1547	-4.9783	0.42308	0.41055	0.96998 (max o	4	5832; 2530; 4001; 42	-0.5240886	-0.5661279		4.0
	ZINC03984113	-7.6	365.389	-0.7942	10	4	257.6	0.41421	132.44	4.4154	2156.416734724	0.29898	6.4605	-2.6564	0.46154	0.41325	0.93361 (max o	3	5739; 5740; 3989	-0.522933	-0.6072604		4.0
	ZINC04064751	-7.6	382.435	-1.5362	9	1	289.36	0.32831	119.08	-33.243	2156.416734724	0.28791	7.2025	-5.3358	0.59259	0.56569	0.92124 (max o	5	6422; 6384; 5837; 40	0.4791864	-0.0244453		4.0
	ZINC03842163	-7.6	406.437	-1.0823	10	1	312.16	0.32576	118.14	5.0031	2156.416734724	0.26805	6.7486	-4.0377	0.48276	0.56719	0.93686 (max o	8	6439; 6131; 3220; 36	-0.0134895	0.39198187		4.0
	ZINC04043933	-7.6	392.450	-1.0059	8	2	301.56	0.30707	121.37	-1.6404	2156.416734724	0.27762	6.6722	-3.6233	0.5	0.46306	0.96519 (max o	3	5747; 4513; 4957	0.08319245	0.89557415		4.0
	ZINC15708229	-7.6	375.468	-3.2716	9	3	278.24	0.39074	117.63	5.4177	2156.416734724	0.31094	8.9379	-10.522	0.56	0.51765	0.91652 (max o	3	5897; 4692; 5061	-0.2338303	0.11633378		4.0
	ZINC35271210	-7.6	377.411	-0.7314	7	3	282.84	0.317	127.12	-10.534	2156.416734724	0.28791	6.3977	-2.5404	0.48148	0.52687	0.96617 (max o	3	6313; 4819; 4820	0.02587551	-0.9552607		4.0
	ZINC05395464	-7.6	435.299	0.8783	7	3	287.01	0.29748	113.29	-5.5588	2156.416734724	0.27762	4.788	3.1636	0.42857	0.35175	0.9984 (max of	4	6162; 5761; 4873; 48	0.48609212	-0.2973464		4.0
	ZINC01995484	-7.6	355.374	-4.8756	11	4	263.55	0.49228	168.7	-0.63512	2156.416734724	0.31094	10.542	-15.68	0.56	0.58587	0.98897 (max o	3	4891; 4892; 5295	0.8075012	0.15121798		4.0
	ZINC01995484	-7.6	354.366	-1.7048	11	4	264.34	0.4938	167.08	-16.91	2156.416734724	0.31094	7.3711	-5.4828	0.56	0.60788	0.98671 (max o	3	5296; 4891; 4892	0.80715823	0.12915419		4.0
	ZINC08762235	-7.6	353.358	-2.6321	11	5	240.58	0.47568	149.05	2.9222	2156.416734724	0.31094	8.2984	-8.465	0.44	0.41176	0.92683 (max o	3	5808; 4904; 5121	-0.5862618	-0.5089993		4.0
	ZINC12483964	-7.6	418.492	0.1639	9	3	312.24	0.27594	95.53	2.2582	2156.416734724	0.25911	5.5024	0.63254	0.56667	0.50053	0.9613 (max of	2	6505; 5054	-0.6182622	-0.3022481		4.0
	ZINC31168775	-7.6	412.433	0.3716	9	5	305.3	0.35552	145.91	-6.6975	2156.416734724	0.26805	5.2947	1.3863	0.62069	0.48911	0.98316 (max o	3	5980; 5213; 5214	-0.7205018	0.4563153		4.0
	ZINC35465104	-7.6	373.420	-1.7731	8	4	278.5	0.35806	139.51	-9.7968	2156.416734724	0.29898	7.4394	-5.9305	0.5	0.51159	0.95857 (max o	5	7092; 6865; 6648; 63	0.4309528	0.58346206		4.0
	ZINC72320479	-7.6	354.354	-0.5368	8	0	240.95	0.35725	89.52	-3.7722	2156.416734724	0.31094	6.2031	-1.7264	0.48	0.30378	0.96243 (max o	3	6918; 6681; 5285	0.7754323	0.42643		4.0
	ZINC03840150	-7.6	367.425	-1.1566	8	5	270.34	0.4582	169.99	5.7725	2156.416734724	0.31094	6.8229	-3.7197	0.6	0.57249	0.95983 (max o	3	6093; 4034; 5331	-0.5527723	-0.2632830		4.0
	ZINC03840039	-7.6	367.425	-1.2517	8	5	267.58	0.46293	169.99	3.2312	2156.416734724	0.31094	6.918	-4.0256	0.56	0.54897	0.91928 (max o	4	6093; 5717; 5333; 53	-0.5512081	-0.2419989		4.0

Structure	Molecule Name	affinity	Total Molweight	cLogP	H-Acceptors	H-Donors	Total Surface Area	Relative PSA	Polar Surface Area	Druglikeness	Kd (nM) @ 20°C	LE from K...	LLE from Kd ...	LELP from Kd (n...	Shape Index	Molecular...	Neighbor Si...	Neig...	Neighbor	Neighbor ...	Neighbor ...	Ring Systems	frequency
	ZINC13407160	-7.6	419.448	-1.2708	8	2	284.75	0.36046	141.03	-0.21816	2156.416734724	0.25911	6.9371	-4.9044	0.4	0.42818	0.95579 (max o	3	6435; 5871; 5395	0.8123206	0.37215617		4.0
	ZINC04268253	-7.6	419.448	-1.2708	8	2	284.75	0.36046	141.03	-0.21816	2156.416734724	0.25911	6.9371	-4.9044	0.4	0.42818	0.95579 (max o	3	6435; 5871; 5501	0.79447025	0.36381197		4.0
	ZINC04995219	-7.6	358.394	-4.462	9	2	245.75	0.51963	182.61	4.8789	2156.416734724	0.32389	10.128	-13.776	0.58333	0.44402	0.90399 (max o	3	7324; 6719; 6381	-0.2906737	-0.8421731		4.0
	ZINC05273539	-7.6	353.354	-4.454	10	3	258.34	0.46923	161.73	9.1102	2156.416734724	0.31094	10.12	-14.324	0.48	0.53522	0.97118 (max o	8	7152; 6449; 6233; 62	0.04879992	0.82138026		4.0
	ZINC20463224	-7.6	381.515	-0.9786	7	1	314.27	0.24848	85.19	0.67097	2156.416734724	0.28791	6.6449	-3.399	0.51852	0.65204	0.93686 (max o	3	5915; 5911; 4205	-0.1158489	-0.2938823		4.0
	ZINC20463789	-7.6	360.432	-1.6502	7	3	264.97	0.27652	89.45	1.4201	2156.416734724	0.29898	7.3165	-5.5195	0.61538	0.45738	0.89688	1	2915	0.5327216	-0.7270156		4.0
	ZINC22920774	-7.6	388.510	-2.9577	7	3	302.67	0.24102	75.87	3.4155	2156.416734724	0.27762	8.624	-10.654	0.57143	0.49539	0.91522 (max o	3	2915; 3425; 3426	0.5743682	-0.7158214		4.0
	ZINC22920774	-7.6	388.510	-6.4	7	3	317.89	0.27736	79.9	-0.66115	2156.416734724	0.27762	12.066	-23.053	0.57143	0.48578	0.85214 (max o	3	7519; 7517; 2222	0.59205765	-0.0021068		4.0
	ZINC20463789	-7.6	361.440	-2.8749	7	3	261.25	0.26622	94.03	-0.21329	2156.416734724	0.29898	8.5412	-9.6157	0.61538	0.44832		0		0.73812675	0.60424864		4.0
	ZINC20463880	-7.6	391.466	-0.7243	8	3	286.96	0.30116	106.28	6.4734	2156.416734724	0.27762	6.3906	-2.6089	0.60714	0.50519	0.92418 (max o	5	7217; 7042; 6220; 37	-0.8096114	0.37998945		4.0
	ZINC20463877	-7.6	361.440	-0.6543	7	3	264.7	0.2887	97.05	6.4079	2156.416734724	0.29898	6.3206	-2.1885	0.57692	0.48409	0.96998 (max o	5	7217; 7042; 6220; 55	-0.7926439	0.34374174		4.0
	ZINC35271772	-7.6	438.471	1.6499	9	2	324.65	0.33377	136.43	-5.8488	2156.416734724	0.25076	4.0164	6.5797	0.45161	0.53187	0.96971 (max o	7	6876; 6869; 5995; 59	0.40097326	0.2100015		4.0
	ZINC35493984	-7.6	406.382	-2.7247	11	6	268.21	0.49081	175.37	-4.1099	2156.416734724	0.27762	8.391	-9.8144	0.42857	0.37997	0.95148 (max o	3	6346; 6342; 6011	0.3649358	-0.7226867		4.0
	ZINC36369864	-7.6	388.399	-5.6761	9	2	300.79	0.39034	141.95	3.1709	2156.416734724	0.27762	11.342	-20.445	0.42857	0.43636	0.95174 (max o	5	6893; 6891; 6662; 56	0.2976656	-0.6072657		4.0
	ZINC40313294	-7.6	374.372	-6.1305	9	2	287.03	0.40905	141.95	-7.2883	2156.416734724	0.28791	11.797	-21.293	0.40741	0.40422	0.95174 (max o	3	6891; 6662; 5646	0.30787575	-0.5875712		4.0
	ZINC28966911	-7.6	392.359	-1.3716	9	6	257.5	0.43355	156.91	-2.2179	2156.416734724	0.27762	7.0379	-4.9405	0.46429	0.35818	0.9543 (max of	4	816; 817; 1926; 2619	-0.2508086	0.5878306		3.0
	ZINC03839646	-7.6	373.408	-0.0223	8	1	267.63	0.29025	88.18	6.8804	2156.416734724	0.28791	5.6886	-0.077456	0.59259	0.33904	0.90033 (max o	2	2003; 2004	-0.6675077	0.65378624		3.0
	ZINC04270798	-7.6	361.420	-0.2491	8	1	270.19	0.27732	96.42	2.6677	2156.416734724	0.29898	5.9154	-0.83317	0.53846	0.52541	0.9279 (max of	5	5419; 2065; 4567; 53	0.08403654	0.386995		3.0
	ZINC04270691	-7.6	369.440	-0.7275	9	1	273.51	0.28339	96	2.1516	2156.416734724	0.29898	6.3938	-2.4333	0.53846	0.57204	0.91693 (max o	3	5785; 2465; 3685	-0.1701696	0.28251836		3.0
	ZINC13139347	-7.6	394.375	-0.4815	9	5	272.76	0.40908	153.75	-2.8773	2156.416734724	0.27762	6.1478	-1.7344	0.42857	0.44471	0.95148 (max o	6	5809; 5760; 2479; 49	-0.7778322	0.40017712		3.0
	ZINC08624389	-7.6	373.448	-0.9153	9	4	282.26	0.34985	120.36	4.4876	2156.416734724	0.29898	6.5816	-3.0614	0.61538	0.58957	0.9083 (max of	4	5820; 2106; 3740; 46	0.21424305	-0.3965258		3.0
	ZINC15673208	-7.6	352.433	-0.2553	8	1	267.23	0.26655	82.19	5.9822	2156.416734724	0.31094	5.9216	-0.82106	0.6	0.55167	0.95631 (max o	4	7194; 1827; 3372; 33	-0.7165452	-0.3277868		3.0
	ZINC35442591	-7.6	381.539	-1.2393	7	3	304.54	0.24736	77.32	3.0783	2156.416734724	0.28791	6.9056	-4.3045	0.55556	0.49859	0.9083 (max of	8	6321; 838; 1887; 299	-0.0542888	0.5570192		3.0
	ZINC03838843	-7.6	357.385	-3.8283	10	7	253.99	0.48959	178.59	3.8547	2156.416734724	0.31094	9.4946	-12.312	0.6	0.46397	0.90171 (max o	3	6703; 6399; 4919	-0.6709587	-0.1978456		3.0
	ZINC08624452	-7.6	448.566	-4.6685	10	5	376.17	0.33147	118.38	-2.1887	2156.416734724	0.24292	10.335	-19.218	0.65625	0.61222	0.92602 (max o	7	7336; 5831; 1762; 17	0.22101207	-0.7188482		3.0
	ZINC20462998	-7.6	401.505	-1.4687	7	2	325.3	0.27608	107.97	4.6832	2156.416734724	0.26805	7.135	-5.4792	0.51724	0.57243	0.93405 (max o	3	3836; 4295; 4706	0.04956249	-0.2272661		3.0
	ZINC03838763	-7.6	353.398	-1.2498	8	5	253.82	0.48802	169.99	2.9567	2156.416734724	0.32389	6.9161	-3.8587	0.54167	0.46452	0.91928 (max o	3	5331; 4034; 4532	-0.5843985	-0.2111555		3.0
	ZINC05437506	-7.6	402.449	0.1141	9	2	302.86	0.30437	108.05	5.2959	2156.416734724	0.26805	5.5522	0.42567	0.51724	0.5393	0.95076 (max o	5	5806; 1745; 1746; 20	0.0964996	0.2460093		3.0
	ZINC08298557	-7.6	360.436	-2.5687	8	3	289.91	0.33842	97.62	-1.4007	2156.416734724	0.29898	8.235	-8.5916	0.65385	0.51399	0.96721 (max o	5	6474; 3719; 4593; 50	0.05250892	-0.1894000		3.0
	ZINC08297629	-7.6	360.460	-4.2698	8	3	289.8	0.32005	94	0.26445	2156.416734724	0.29898	9.9361	-14.281	0.65385	0.50507	0.96721 (max o	4	7162; 5443; 4592; 45	0.03598798	-0.1536392		3.0
	ZINC12886072	-7.6	444.506	-0.9389	9	1	336.19	0.28258	119.08	8.6017	2156.416734724	0.24292	6.6052	-3.8651	0.53125	0.46174	0.97743 (max o	5	3747; 4196; 4630; 46	0.5116599	0.34902185		3.0
	ZINC08740345	-7.6	417.409	-2.7955	11	8	284.69	0.46774	181.33	-6.4753	2156.416734724	0.26805	8.4618	-10.429	0.51724	0.43239	0.95372 (max o	4	6370; 3744; 4186; 50	-0.2158065	-0.9385355		3.0
	ZINC06070013	-7.6	410.374	-0.6661	10	5	285.96	0.41453	155.14	-5.6381	2156.416734724	0.26805	6.3324	-2.485	0.44828	0.36286	0.92805 (max o	4	5499; 5498; 5079; 50	-0.2513579	0.7774993		3.0
	ZINC03872805	-7.6	422.520	-1.4272	8	8	321.68	0.29246	154.6	-7.0465	2156.416734724	0.25911	7.0935	-5.508	0.73333	0.49236	0.97533 (max o	2	6940; 6705	0.83814126	-0.2237809		3.0
	ZINC04222745	-7.6	396.395	-1.2197	10	2	289.8	0.38927	127.57	2.5547	2156.416734724	0.27762	6.886	-4.3934	0.57143	0.40081	0.94558 (max o	2	6433; 2803	-0.864145	-0.3320849		3.0
	ZINC04277814	-7.6	405.449	0.3474	9	1	311.9	0.29086	105.25	5.0128	2156.416734724	0.26805	5.3189	1.296	0.51724	0.5667	0.94241 (max o	5	7069; 6438; 6439; 61	-0.0131113	0.43241745		3.0
	ZINC06624058	-7.6	359.448	-0.0706	7	2	295.63	0.26097	67.6	3.5639	2156.416734724	0.29898	5.7369	-0.23614	0.57692	0.43245	0.90377 (max o	2	7456; 6077	0.74519163	-0.1964968		3.0
	ZINC09088460	-7.6	364.397	-1.8807	8	3	269.13	0.30424	114.05	1.6759	2156.416734724	0.29898	7.547	-6.2904	0.61538	0.4393	0.89688 (max o	4	6799; 6523; 3378; 33	-0.0231846	-0.8297539		3.0
	ZINC12481217	-7.6	377.434	-0.9702	6	0	288.84	0.19893	72.91	2.7121	2156.416734724	0.28791	6.6365	-3.3699	0.55556	0.58923	0.93686 (max o	3	6764; 5846; 5048	-0.1673139	-0.1049287		3.0
	ZINC20463224	-7.6	380.507	-1.1102	7	0	300.23	0.21334	83.99	5.1161	2156.416734724	0.28791	6.7765	-3.8561	0.51852	0.65672	0.93686 (max o	7	5918; 5916; 5912; 55	-0.2116711	-0.2342185		3.0
	ZINC03197508	-7.6	387.319	-0.8425	9	2	262.94	0.40526	134.58	0.9819	2156.416734724	0.27762	6.5088	-3.0347	0.46429	0.2766	0.9543 (max of	3	6084; 1098; 1657	-0.1726280	0.76938576		2.0
	ZINC04221681	-7.6	447.518	-0.4862	11	3	330.48	0.43292	180.55	1.7954	2156.416734724	0.25076	6.1525	-1.9389	0.41935	0.4506	0.92584 (max o	2	6136; 1408	0.7279079	0.4261988		2.0
	ZINC12603424	-7.6	381.448	-2.7445	10	4	271.09	0.42159	145.81	4.3318	2156.416734724	0.31094	8.4108	-8.8265	0.64	0.6228	0.88492 (max o	2	1800; 5065	0.20382147	-0.3789677		2.0

Structure	Molecule Name	affinity	Total Molweight	cLogP	H-Acceptors	H-Donors	Total Surface Area	Relative PSA	Polar Surface Area	Druglikeness	Kd (nM) @ 20°C	LE from K...	LLE from Kd ...	LELP from Kd (n...	Shape Index	Molecular...	Neighbor Si...	Neig...	Neighbor	Neighbor ...	Neighbor ...	Ring Systems	frequency
	ZINC31167958	-7.6	398.494	0.9599	7	4	288.68	0.29597	116.45	-3.8942	2156.416734724	0.27762	4.7064	3.4576	0.46429	0.43645	0.96519 (max c	3	6619; 2271; 5208	-0.6757219	0.06039066		2.0
	ZINC14726939	-7.6	356.285	-3.7815	8	4	245.19	0.43656	161.18	1.0482	2156.416734724	0.29898	9.4478	-12.648	0.42308	0.38525	0.95257 (max c	3	2968; 5108; 5515	-0.8765123	0.33386973		2.0
	ZINC31160363	-7.6	408.362	-0.6866	11	4	285.1	0.43294	154.86	-1.2018	2156.416734724	0.26805	6.3529	-2.5615	0.48276	0.42334	0.9561 (max of	4	2262; 3039; 3050; 51	0.4060146	-0.6674011		2.0
	ZINC72325681	-7.6	359.401	-1.7532	6	0	264.73	0.35168	127.68	-2.7108	2156.416734724	0.31094	7.4195	-5.6384	0.44	0.49726	0.97118 (max c	2	3549; 5693	-0.7346654	-0.5657204		2.0
	ZINC72325681	-7.6	360.409	-3.731	6	1	253.76	0.32365	128.93	-2.9727	2156.416734724	0.31094	9.3973	-11.999	0.44	0.49726	0.97118 (max c	2	3550; 5692	-0.7145954	-0.5649524		2.0
	ZINC31155902	-7.6	358.342	-0.7325	9	5	248.05	0.42532	138.07	-4.5704	2156.416734724	0.31094	6.3988	-2.3558	0.52	0.47032	0.98671 (max c	3	6301; 5982; 3887	0.1548651	0.77267253		2.0
	ZINC31157969	-7.6	364.393	1.4512	7	2	267.37	0.31911	110.13	-16.562	2156.416734724	0.29898	4.2151	4.8539	0.42308	0.31559	0.97791 (max c	3	6611; 6292; 3896	-0.7964951	0.1984874		2.0
	ZINC34302824	-7.6	362.377	-0.1664	7	1	251.77	0.37526	97.89	-7.4006	2156.416734724	0.29898	5.8327	-0.55656	0.42308	0.22723	0.98517 (max c	4	6634; 6633; 5761; 41	-0.5177706	-0.2507084		2.0
	ZINC04222232	-7.6	396.264	-1.2573	7	3	251.4	0.29169	95.12	4.2596	2156.416734724	0.32389	6.9236	-3.8818	0.58333	0.33952	0.92978 (max c	2	1711; 4230	0.7422871	-0.5362504		2.0
	ZINC02138340	-7.6	367.488	-1.1516	7	2	287.63	0.28818	117.18	3.6514	2156.416734724	0.29898	6.8179	-3.8518	0.65385	0.5723	0.93046	1	4520	-0.9447736	0.02739225		2.0
	ZINC22923496	-7.6	367.536	-4.0887	7	3	312.12	0.25573	61.53	6.103	2156.416734724	0.29898	9.755	-13.676	0.57692	0.52864	0.90502 (max c	7	5997; 2615; 3024; 34	-0.0875489	0.5954866		2.0
	ZINC22923496	-7.6	366.528	-1.979	7	2	298.08	0.22068	60.33	10.133	2156.416734724	0.29898	7.6453	-6.6192	0.57692	0.52864	0.96438 (max c	8	5997; 2613; 2654; 30	-0.1394885	0.54257226		2.0
	ZINC39563707	-7.6	448.542	-1.5911	10	3	345.56	0.31063	134.31	4.0575	2156.416734724	0.24292	7.2574	-6.5499	0.46875	0.51445	0.89631 (max c	2	7856; 4929	-0.2251192	-0.8824057		2.0
	ZINC20463789	-7.6	360.432	-1.6502	7	3	264.97	0.27652	89.45	1.129	2156.416734724	0.29898	7.3165	-5.5195	0.61538	0.48789	0.87087 (max c	2	7497; 5050	0.5900136	-0.0831497		2.0
	ZINC14726933	-7.6	356.285	-3.7815	8	4	245.19	0.43656	161.18	1.0482	2156.416734724	0.29898	9.4478	-12.648	0.42308	0.3825	0.95257 (max c	3	5516; 2968; 5108	-0.8578183	0.3439533		2.0
	ZINC31160213	-7.6	376.357	-2.1895	10	4	250.11	0.47371	151.98	-5.795	2156.416734724	0.29898	7.8558	-7.3233	0.46154	0.45048	0.96721	1	5234	-0.1428281	0.8798272		2.0
	ZINC03840077	-7.6	439.555	-2.4174	9	6	324.84	0.40515	186.6	6.4284	2156.416734724	0.25911	8.0837	-9.3295	0.5	0.52341	0.93975 (max c	5	6659; 6189; 5721; 36	-0.5713042	-0.1541873		2.0
	ZINC03841215	-7.6	434.492	-1.6803	9	2	316.32	0.38063	153.17	5.5382	2156.416734724	0.25911	7.3466	-6.4848	0.6	0.48912	0.92821	1	5336	-0.3837500	-0.8903726		2.0
	ZINC03841212	-7.6	420.465	-2.1347	9	2	302.56	0.39794	153.17	6.6416	2156.416734724	0.26805	7.801	-7.9638	0.58621	0.48791	0.92821	1	5337	-0.3738179	-0.8779703		2.0
	ZINC03939857	-7.6	416.405	-1.2429	9	3	306.89	0.37179	153.06	-1.0653	2156.416734724	0.25911	6.9092	-4.7967	0.5	0.30536	0.98394 (max c	3	5349; 5350; 5351	-0.3652818	-0.7855767		2.0
	ZINC03939857	-7.6	416.405	-1.2429	9	3	306.89	0.37179	153.06	-1.0653	2156.416734724	0.25911	6.9092	-4.7967	0.5	0.30536	0.98394 (max c	3	5352; 5349; 5350	-0.3551092	-0.8024524		2.0
	ZINC04085598	-7.6	369.476	-0.9701	6	3	267.09	0.28687	110.05	-5.7297	2156.416734724	0.29898	6.6364	-3.2447	0.42308	0.46411	0.93686 (max c	3	6407; 6105; 4961	-0.8526901	-0.3757386		2.0
	ZINC04096261	-7.6	362.389	-2.3544	10	4	267.39	0.44452	150.28	9.5534	2156.416734724	0.29898	8.0207	-7.8748	0.46154	0.49175	0.96998 (max c	9	7152; 6527; 6528; 62	0.00224676	0.7444636		2.0
	ZINC04096261	-7.6	362.389	-2.3544	10	4	267.39	0.44452	150.28	9.5534	2156.416734724	0.29898	8.0207	-7.8748	0.46154	0.49175	0.96998 (max c	8	7152; 6527; 6528; 62	0.02053316	0.75426245		2.0
	ZINC04096261	-7.6	363.397	-4.3322	10	5	256.42	0.42076	151.53	8.9317	2156.416734724	0.29898	9.9985	-14.49	0.46154	0.48498	0.9615 (max of	9	7152; 6530; 6529; 62	-0.0140778	0.7780302		2.0
	ZINC04096261	-7.6	363.397	-4.3322	10	5	256.42	0.42076	151.53	9.0745	2156.416734724	0.29898	9.9985	-14.49	0.46154	0.49175	0.96998 (max c	9	7152; 6530; 6529; 62	-0.0158325	0.75833625		2.0
	ZINC04221729	-7.6	414.421	-1.4704	11	3	313.27	0.41482	160.52	6.9756	2156.416734724	0.25911	7.1367	-5.6747	0.5	0.4488	0.92257	1	5772	0.75797707	0.39155644		2.0
	ZINC04270782	-7.6	362.385	-0.0601	9	0	270.25	0.29876	92.28	1.7426	2156.416734724	0.29898	5.7264	-0.20102	0.53846	0.59124	0.93686	1	6147	-0.3136738	0.90982527		2.0
	ZINC05440750	-7.6	408.478	-0.4481	9	2	300.3	0.37476	136.29	6.6275	2156.416734724	0.27762	6.1144	-1.6141	0.46429	0.51243	0.92152 (max c	4	2841; 2862; 2930; 49	-0.1193683	0.12306347		2.0
	ZINC05414665	-7.6	414.506	-0.3874	9	2	287.37	0.4109	152.51	7.8648	2156.416734724	0.28791	6.0537	-1.3456	0.44444	0.50618	0.89081 (max c	3	5900; 3374; 5274	-0.1123265	0.35862017		2.0
	ZINC05437694	-7.6	355.369	-1.3057	7	0	260.81	0.28607	93.89	2.0118	2156.416734724	0.29898	6.972	-4.3672	0.5	0.46488	0.94927	1	5807	0.07357995	-0.8860518		2.0
	ZINC08298894	-7.6	362.520	-3.3209	7	3	294.39	0.26713	80.86	-4.1638	2156.416734724	0.29898	8.9872	-11.107	0.65385	0.49452	0.96721 (max c	3	4156; 4595; 5444	0.02411480	-0.0599975		2.0
	ZINC08298874	-7.6	376.547	-3.196	7	3	323.25	0.27375	68.69	-4.2377	2156.416734724	0.28791	8.8623	-11.101	0.62963	0.50925	0.96885 (max c	4	6398; 5445; 4156; 45	0.02861927	-0.0886308		2.0
	ZINC11867897	-7.6	423.421	-1.7886	10	2	316.05	0.44601	172.3	-2.4775	2156.416734724	0.26805	7.4549	-6.6726	0.65517	0.60961	0.90725	1	6207	0.6339674	-0.6232675		2.0
	ZINC12484168	-7.6	402.557	0.2987	6	2	300.31	0.15964	61.78	-0.39326	2156.416734724	0.26805	5.3676	1.1143	0.58621	0.46061	0.91964 (max c	6	7213; 6768; 6766; 65	0.40149897	0.03697589		2.0
	ZINC12877310	-7.6	445.534	-0.8085	8	4	360.74	0.27247	101.69	1.7223	2156.416734724	0.24292	6.4748	-3.3283	0.4375	0.43181	0.85572	1	6120	-0.7290919	-0.6789687		2.0
	ZINC13335252	-7.6	410.374	-0.6661	10	5	285.96	0.41453	155.14	-5.6381	2156.416734724	0.26805	6.3324	-2.485	0.44828	0.36656	0.95099 (max c	4	5079; 5080; 5433; 54	-0.2669037	0.7890875		2.0
	ZINC13335249	-7.6	410.374	-0.6661	10	5	285.96	0.41453	155.14	-5.6381	2156.416734724	0.26805	6.3324	-2.485	0.44828	0.36656	0.94823 (max c	4	5499; 5079; 5080; 54	-0.2536434	0.757641		2.0
	ZINC13386381	-7.6	351.717	-0.1115	7	1	248.22	0.34848	112.96	-3.798	2156.416734724	0.32389	5.7778	-0.34425	0.45833	0.48588	0.97246	1	6614	-0.1343428	0.98141104		2.0
	ZINC13528740	-7.6	350.330	-2.8891	10	2	253.74	0.44656	163	0.36393	2156.416734724	0.31094	8.5554	-9.2916	0.6	0.53811	0.86499	1	7009	0.9094492	-0.3882625		2.0
	ZINC20463826	-7.6	428.531	-3.2142	8	3	327.33	0.25341	85.1	2.2729	2156.416734724	0.25076	8.8805	-12.818	0.54839	0.45838	0.97642 (max c	6	6558; 6556; 6254; 47	-0.5954404	-0.6005750		2.0
	ZINC20463826	-7.6	427.523	-1.1045	8	2	313.29	0.21996	83.9	4.6027	2156.416734724	0.25076	6.7708	-4.4047	0.54839	0.45421	0.97642 (max c	7	6557; 6255; 5853; 47	0.3483218	-0.0324169		2.0
	ZINC31156417	-7.6	388.368	-1.9969	10	5	262.24	0.45203	155.14	-7.5663	2156.416734724	0.28791	7.6632	-6.936	0.48148	0.37688	0.93996 (max c	2	5631; 3034	-0.5712678	0.21309139		2.0

Structure	Molecule Name	affinity	Total Molweight	cLogP	H-Acceptors	H-Donors	Total Surface Area	Relative PSA	Polar Surface Area	Druglikeness	Kd (nM) @ 20°C	LE from K...	LLE from Kd ...	LELP from Kd (n...	Shape Index	Molecular...	Neighbor Si...	Neig...	Neighbor	Neighbor ...	Neighbor ...	Ring Systems	frequency
	ZINC35455092	-7.6	388.368	-1.9969	10	5	262.24	0.45203	155.14	-7.5663	2156.416734724	0.28791	7.6632	-6.936	0.48148	0.37688	0.93996 (max o	3	6326; 5229; 5596	-0.5925349	0.21706563		2.0
	ZINC31160895	-7.6	371.412	-1.5001	7	2	288.87	0.28217	109.77	6.0291	2156.416734724	0.28791	7.1664	-5.2104	0.51852	0.60384	0.94905	1	5974	0.24835683	0.8620031		2.0
	ZINC31164241	-7.6	365.313	-2.4855	9	4	257.49	0.44208	152.65	-3.5303	2156.416734724	0.29898	8.1518	-8.3133	0.46154	0.41219	0.91351 (max o	2	6607; 5205	0.2028987	-0.6111649		2.0
	ZINC38549937	-7.6	374.499	0.8471	5	3	312.02	0.20444	63.36	-6.941	2156.416734724	0.28791	4.8192	2.9423	0.51852	0.47399	0.97407 (max o	3	7563; 7445; 3520	0.01996284	-0.7938627		2.0
	ZINC44021506	-7.6	385.372	-7.8363	11	4	274.38	0.43946	166.83	2.0067	2156.416734724	0.28791	13.503	-27.218	0.55556	0.57766	0.96885	1	5657	-0.5980852	0.7967075		2.0
	ZINC44021503	-7.6	385.372	-7.8363	11	4	274.38	0.43946	166.83	2.0067	2156.416734724	0.28791	13.503	-27.218	0.55556	0.56343	0.96885	1	5658	-0.6007979	0.79521334		2.0
	ZINC49181190	-7.6	430.492	-0.5521	9	6	296.67	0.37631	156.91	-2.4223	2156.416734724	0.25911	6.2184	-2.1307	0.43333	0.40996	0.95837 (max o	3	2674; 3087; 5659	0.63134545	-0.5876644		2.0
	ZINC49181187	-7.6	430.492	-0.5521	9	6	296.67	0.37631	156.91	-2.4223	2156.416734724	0.25911	6.2184	-2.1307	0.43333	0.40996	0.95837 (max o	3	2674; 3087; 5660	0.6331304	-0.5513856		2.0
	ZINC49601629	-7.6	388.455	0.0267	8	5	280.91	0.35079	136.68	-6.634	2156.416734724	0.28791	5.6396	0.092739	0.48148	0.49366	0.94302 (max o	3	4855; 4856; 5663	0.55360436	0.6226303		2.0
	ZINC49601627	-7.6	388.455	0.0267	8	5	280.91	0.35079	136.68	-6.634	2156.416734724	0.28791	5.6396	0.092739	0.48148	0.49366	0.94605 (max o	3	5664; 4855; 4856	0.57181984	0.63007826		2.0
	ZINC56874249	-7.6	350.394	-5.2192	7	2	254.67	0.42443	146.69	5.329	2156.416734724	0.32389	10.885	-16.114	0.54167	0.5751	0.93334 (max o	3	6056; 6055; 5679	0.03156278	-0.7344255		2.0
	ZINC71758441	-7.6	350.394	-5.2192	7	2	254.67	0.42443	146.69	5.329	2156.416734724	0.32389	10.885	-16.114	0.54167	0.5751	0.93334 (max o	3	6056; 6055; 5665	0.03445812	-0.7140438		2.0
	ZINC00538083	-7.6	406.461	-0.5383	10	3	327.23	0.32317	123.14	-5.8993	2156.416734724	0.26805	6.2046	-2.0082	0.62069	0.69637		0		-0.2472819	-0.8474198		1.0
	ZINC01530999	-7.6	408.414	-4.8643	10	2	276.73	0.57753	222.12	8.4031	2156.416734724	0.28791	10.531	-16.895	0.48148	0.45978	0.94302 (max o	2	3618; 4092	-0.3358564	0.3204524		1.0
	ZINC02560996	-7.6	409.445	-1.4496	9	4	313.66	0.36906	154.65	2.5737	2156.416734724	0.25911	7.1159	-5.5944	0.53333	0.47933	0.87363 (max o	2	7153; 4475	0.69614637	0.2982572		1.0
	ZINC03786299	-7.6	369.342	-2.3292	6	1	272.81	0.23844	68.12	1.7785	2156.416734724	0.29898	7.9955	-7.7905	0.5	0.44519	0.88064 (max o	5	1950; 1952; 2335; 31	0.62636954	-0.3785435		1.0
	ZINC03786299	-7.6	368.334	-2.4608	6	0	258.77	0.19712	66.92	6.55	2156.416734724	0.29898	8.1271	-8.2307	0.5	0.44519	0.88064 (max o	5	1951; 1953; 2336; 31	0.60545444	-0.3500054		1.0
	ZINC03798757	-7.6	416.516	-0.2263	7	1	318.42	0.23456	103.35	-3.0393	2156.416734724	0.25911	5.8926	-0.87336	0.46667	0.46238	0.9112 (max of	2	6695; 3995	-0.8192105	0.04268781		1.0
	ZINC03809827	-7.6	430.527	-2.3143	9	4	309.96	0.29123	157.76	1.7035	2156.416734724	0.25076	7.9806	-9.2293	0.54839	0.54423		0		-0.6410568	-0.7428254		1.0
	ZINC03839171	-7.6	416.480	-0.6882	10	4	319.46	0.33939	124.57	3.4973	2156.416734724	0.25911	6.3545	-2.656	0.46667	0.46954	0.86552 (max o	2	6935; 6934	-0.0122843	0.02802125		1.0
	ZINC03839755	-7.6	354.406	-3.336	8	1	248.66	0.41217	135.15	7.1827	2156.416734724	0.32389	9.0023	-10.3	0.58333	0.50505	0.84296	1	4925	-0.498683	-0.4201071		1.0
	ZINC03839856	-7.6	436.556	-0.012	8	2	305.53	0.39286	152.36	7.9484	2156.416734724	0.26805	5.6783	-0.044768	0.58621	0.37103	0.90418	1	4926	-0.4176649	-0.7994674		1.0
	ZINC03840356	-7.6	357.821	0.9104	8	1	246.51	0.40558	123.74	3.4389	2156.416734724	0.33798	4.7559	2.6937	0.56522	0.39472		0		-0.6575209	0.6224942		1.0
	ZINC03939857	-7.6	415.397	-4.1123	9	1	309.05	0.37282	152.73	-0.93878	2156.416734724	0.25911	9.7786	-15.871	0.5	0.42924		0		0.59008133	-0.7271870		1.0
	ZINC03939857	-7.6	415.397	-4.0082	9	2	308.07	0.3742	155.89	-0.74118	2156.416734724	0.25911	9.6745	-15.469	0.5	0.42048	0.8956 (max of	4	7191; 5352; 5350; 53	-0.385412	-0.7875544		1.0
	ZINC03939857	-7.6	416.405	-1.2429	9	3	306.89	0.37179	153.06	-0.97625	2156.416734724	0.25911	6.9092	-4.7967	0.5	0.30536	0.91964 (max o	3	5352; 5349; 5351	-0.3772640	-0.7683653		1.0
	ZINC04084926	-7.6	381.407	-0.6837	7	1	290.6	0.26982	98.77	6.4717	2156.416734724	0.27762	6.35	-2.4627	0.60714	0.4439	0.96519 (max o	3	2787; 4525; 4959	-0.0628533	-0.7154287		1.0
	ZINC04089958	-7.6	385.527	-1.5073	7	2	294.12	0.34129	142.48	5.4131	2156.416734724	0.29898	7.1736	-5.0415	0.65385	0.573	0.86427	1	7469	0.95190483	0.13249196		1.0
	ZINC04095807	-7.6	408.427	-1.9969	10	4	271.75	0.53086	202.62	-4.7406	2156.416734724	0.29898	7.6632	-6.6791	0.46154	0.54727	0.87809 (max o	4	7504; 7474; 3879; 48	-0.3814585	0.57322836		1.0
	ZINC04096261	-7.6	362.389	-2.2765	10	5	268	0.44578	158.25	6.4682	2156.416734724	0.29898	7.9428	-7.6143	0.46154	0.45177	0.9615 (max of	3	5382; 5381; 5378	0.9528344	-0.0235887		1.0
	ZINC04096261	-7.6	362.389	-2.2765	10	5	268	0.44578	158.25	6.4682	2156.416734724	0.29898	7.9428	-7.6143	0.46154	0.45541	0.9615 (max of	3	5382; 5381; 5377	0.92875314	-0.0284605		1.0
	ZINC04096261	-7.6	363.397	-4.2543	10	6	257.03	0.42213	159.5	5.9644	2156.416734724	0.29898	9.9206	-14.229	0.46154	0.45177	0.9615 (max of	3	5382; 5377; 5378	0.94332075	-0.0432538		1.0
	ZINC04096261	-7.6	363.397	-4.2543	10	6	257.03	0.42213	159.5	5.9644	2156.416734724	0.29898	9.9206	-14.229	0.46154	0.45541	0.9615 (max of	3	5377; 5378; 5381	0.9384986	-0.0093042		1.0
	ZINC04259363	-7.6	379.400	-0.1664	11	1	269.26	0.49658	151.47	-6.9983	2156.416734724	0.29898	5.8327	-0.55656	0.61538	0.30879	0.86137 (max o	3	5780; 957; 4545	-0.53719	0.49491322		1.0
	ZINC04277762	-7.6	350.417	-0.0405	8	1	260.23	0.27372	82.19	1.0258	2156.416734724	0.31094	5.7068	-0.13025	0.6	0.45165	0.85984 (max o	2	2817; 3240	-0.2789240	0.72850955		1.0
	ZINC05399131	-7.6	350.398	-0.6008	8	1	248.07	0.36816	119.35	6.0496	2156.416734724	0.32389	6.2671	-1.8549	0.625	0.56439	0.83096	1	3680	0.19025074	-0.8533584		1.0
	ZINC08234298	-7.6	362.330	-3.6103	10	6	234.68	0.53797	161.6	-4.3947	2156.416734724	0.31094	9.2766	-11.611	0.44	0.35739	0.92315 (max o	3	6050; 3949; 4438	0.5008489	0.5761629		1.0
	ZINC08295744	-7.6	365.453	0.0486	7	4	266.13	0.38011	128.48	4.911	2156.416734724	0.31094	5.6177	0.1563	0.6	0.41529	0.94678	1	7334	-0.4908809	0.4248132		1.0
	ZINC08636487	-7.6	419.501	1.3527	8	3	293.3	0.36072	126.59	2.8625	2156.416734724	0.26805	4.3136	5.0465	0.44828	0.37507	0.97415 (max o	3	6183; 5827; 2882	0.45503253	0.04815436		1.0
	ZINC12480769	-7.6	439.554	0.0107	7	1	365.55	0.22561	87	4.2217	2156.416734724	0.24292	5.6556	0.044049	0.59375	0.60261	0.89329 (max o	2	7380; 5047	-0.1082665	-0.0890862		1.0
	ZINC12603454	-7.6	382.464	-3.2603	9	4	279.13	0.42134	154.35	-0.57943	2156.416734724	0.29898	8.9266	-10.905	0.65385	0.53617	0.87695	1	7336	0.2378844	-0.7303003		1.0
	ZINC12603614	-7.6	358.433	-0.4763	8	3	273.56	0.31909	108.33	4.0113	2156.416734724	0.31094	6.1426	-1.5318	0.6	0.58322	0.88064	1	3742	0.12365322	-0.4224709		1.0
	ZINC12603674	-7.6	376.435	-2.7114	9	4	298.29	0.35905	113.94	-2.3532	2156.416734724	0.28791	8.3777	-9.4177	0.62963	0.52366		0		0.95653486	0.19538121		1.0

Structure	Molecule Name	affinity	Total Molweight	cLogP	H-Acceptors	H-Donors	Total Surface Area	Relative PSA	Polar Surface Area	Druglikeness	Kd (nM) @ 20°C	LE from K...	LLE from Kd ...	LELP from Kd (n...	Shape Index	Molecular...	Neighbor Si...	Neig...	Neighbor	Neighbor ...	Neighbor ...	Ring Systems	frequency
	ZINC12653078	-7.6	385.372	-0.9182	11	6	281.37	0.46156	162.1	0.41134	2156.416734724	0.28791	6.5845	-3.1892	0.59259	0.41538	0.83573	1	3339	0.27853328	-0.8822968		1.0
	ZINC12654467	-7.6	426.448	-1.9448	9	3	334.47	0.33133	136.66	4.4412	2156.416734724	0.25076	7.6111	-7.7557	0.54839	0.55381		0		-0.5479748	-0.7061075		1.0
	ZINC12901063	-7.6	432.404	-2.1948	10	1	314.98	0.36806	128.52	1.222	2156.416734724	0.25076	7.8611	-8.7527	0.45161	0.35526	0.96739 (max o	3	5868; 3746; 4628	-0.9245341	-0.2223576		1.0
	ZINC13704173	-7.6	440.583	3.0188	8	1	313.53	0.26699	115.42	1.3833	2156.416734724	0.25911	2.6475	11.65	0.56667	0.46623	0.96832	1	5768	0.5635348	-0.8223443		1.0
	ZINC14828379	-7.6	392.403	1.1199	8	2	289.19	0.32961	119.36	-11.485	2156.416734724	0.27762	4.5464	4.0339	0.42857	0.53096	0.92774	1	6721	0.8738322	-0.0763260		1.0
	ZINC15708204	-7.6	365.452	-2.8335	8	3	281.67	0.33081	92.18	4.5731	2156.416734724	0.29898	8.4998	-9.4773	0.57692	0.46868	0.91999 (max o	8	7363; 6771; 6243; 22	-0.2001283	0.09387673		1.0
	ZINC20463258	-7.6	391.466	-1.5846	8	1	316.53	0.29128	98.33	4.9851	2156.416734724	0.27762	7.2509	-5.7077	0.53571	0.58523	0.96519 (max o	3	4207; 4716; 5529	-0.1881893	-0.3149529		1.0
	ZINC20463258	-7.6	390.458	-1.7162	8	0	302.49	0.25839	97.13	9.2851	2156.416734724	0.27762	7.3825	-6.1818	0.53571	0.58944	0.96519 (max o	4	5916; 5133; 5526; 55	-0.2335180	-0.2395077		1.0
	ZINC20463261	-7.6	407.533	-0.9067	7	1	322.78	0.30501	113.43	6.4595	2156.416734724	0.27762	6.573	-3.2659	0.53571	0.58269	0.96519 (max o	2	4208; 5527	-0.1604738	-0.3420110		1.0
	ZINC20463261	-7.6	406.525	-1.0383	7	0	308.74	0.2734	112.23	10.681	2156.416734724	0.27762	6.7046	-3.74	0.53571	0.58692	0.96519 (max o	5	5916; 2585; 5133; 55	-0.2171788	-0.2551518		1.0
	ZINC20463651	-7.6	440.518	-0.7855	8	2	348.47	0.26811	85.54	4.5014	2156.416734724	0.24292	6.4518	-3.2336	0.46875	0.49013		0		0.06611029	0.97038543		1.0
	ZINC20463651	-7.6	440.518	-4.2278	8	2	363.69	0.29874	89.57	-0.15375	2156.416734724	0.24292	9.8941	-17.404	0.46875	0.49427		0		-0.0430383	-0.9723242		1.0
	ZINC20464575	-7.6	405.545	-5.2546	9	4	311.94	0.25572	90.26	9.4886	2156.416734724	0.26805	10.921	-19.603	0.58621	0.48462	0.96931 (max o	10	6848; 5955; 5548; 55	-0.1191690	0.5099753		1.0
	ZINC20464575	-7.6	405.545	-5.3865	9	4	336.95	0.31097	90.21	5.4689	2156.416734724	0.26805	11.053	-20.095	0.58621	0.47397	0.90171 (max o	4	1281; 2614; 2994; 55	-0.4502999	-0.6700297		1.0
	ZINC20464575	-7.6	404.537	-3.2768	9	3	322.91	0.28101	89.01	9.5775	2156.416734724	0.26805	8.9431	-12.225	0.58621	0.49809	0.96931 (max o	12	6849; 5956; 5586; 12	-0.0898477	0.46906582		1.0
	ZINC20464575	-7.6	404.537	-3.2768	9	3	322.91	0.28101	89.01	9.1362	2156.416734724	0.26805	8.9431	-12.225	0.58621	0.47397	0.90171 (max o	4	1283; 2989; 2994; 55	-0.4595515	-0.6505177		1.0
	ZINC20464575	-7.6	406.553	-7.3643	9	5	325.98	0.28778	91.46	5.2664	2156.416734724	0.26805	13.031	-27.474	0.58621	0.48462	0.90883 (max o	9	6848; 5583; 5585; 15	-0.0868628	0.55714935		1.0
	ZINC20465067	-7.6	403.438	-1.1192	10	3	298.3	0.34814	124.19	5.9027	2156.416734724	0.26805	6.7855	-4.1753	0.55172	0.45954	0.90725 (max o	5	1870; 2999; 3400; 47	-0.4029410	0.11699106		1.0
	ZINC20465067	-7.6	404.445	-3.2289	10	4	312.34	0.37744	125.39	4.0149	2156.416734724	0.26805	8.8952	-12.046	0.55172	0.46373	0.96428 (max o	3	3000; 3401; 4756	-0.4094572	-0.5536884		1.0
	ZINC20465087	-7.6	408.545	-5.5267	9	5	335.78	0.32262	99.62	2.9172	2156.416734724	0.26805	11.193	-20.618	0.58621	0.48693	0.95372 (max o	4	3849; 4313; 4741; 51	-0.3104251	-0.5475896		1.0
	ZINC20465087	-7.6	407.537	-2.532	9	3	303.98	0.25176	101.8	7.4301	2156.416734724	0.26805	8.1983	-9.446	0.58621	0.42344	0.95372 (max o	6	6818; 3851; 4314; 47	-0.5068874	0.14128804		1.0
	ZINC20465087	-7.6	407.537	-3.417	9	4	321.74	0.29306	98.42	4.8778	2156.416734724	0.26805	9.0833	-12.748	0.58621	0.48289	0.96428 (max o	5	3850; 4315; 4743; 51	0.6287513	-0.4564305		1.0
	ZINC20465695	-7.6	365.448	-1.7345	7	4	291.09	0.30331	92.46	-0.89656	2156.416734724	0.29898	7.4008	-5.8014	0.57692	0.53887	0.96998 (max o	3	6264; 4735; 5555	0.7072548	0.37452292		1.0
	ZINC20465746	-7.6	377.503	-0.6476	6	4	307.35	0.25473	83.23	0.60121	2156.416734724	0.28791	6.3139	-2.2494	0.55556	0.52234	0.96343 (max o	4	7223; 7054; 6832; 51	0.28507593	-0.1108101		1.0
	ZINC20465776	-7.6	427.564	-0.5302	7	4	319.84	0.35702	136.92	2.6255	2156.416734724	0.27762	6.1965	-1.9098	0.53571	0.56206	0.96519 (max o	3	6604; 5572; 4316	0.2266783	-0.1076336		1.0
	ZINC20465830	-7.6	364.528	-1.3754	5	4	299.64	0.20535	70.74	-5.9967	2156.416734724	0.29898	7.0417	-4.6003	0.57692	0.50304	0.9615 (max of	3	6827; 6588; 5942	0.5164055	0.7785106		1.0
	ZINC20465830	-7.6	363.520	-0.0090996	5	3	285.6	0.16628	69.54	-1.2889	2156.416734724	0.29898	5.6754	-0.030436	0.57692	0.49671	0.96998 (max o	3	6829; 6590; 5943	-0.4052316	0.76442915		1.0
	ZINC20465869	-7.6	428.552	-1.4771	8	4	318.6	0.39284	149.81	2.5823	2156.416734724	0.27762	7.1434	-5.3205	0.53571	0.56111	0.96519 (max o	2	6604; 5569	0.23910831	-0.1215803		1.0
	ZINC20503815	-7.6	446.682	-0.0528	6	3	371.97	0.23015	76.3	3.5063	2156.416734724	0.25076	5.7191	-0.21056	0.67742	0.64278	0.86162 (max o	2	7780; 6668	0.71778274	-0.5923902		1.0
	ZINC20504123	-7.6	445.566	-2.4849	9	5	340.45	0.37183	141.51	6.9412	2156.416734724	0.25076	8.1512	-9.9096	0.58065	0.41216	0.89093 (max o	4	6273; 5944; 5162; 55	0.44443378	0.219198		1.0
	ZINC22923520	-7.6	423.584	-4.4514	9	3	342.08	0.35699	115.55	1.2119	2156.416734724	0.26805	10.118	-16.607	0.58621	0.49809	0.90883 (max o	11	5998; 5586; 1280; 18	-0.0324034	0.555808		1.0
	ZINC22923520	-7.6	422.576	-2.3417	9	2	328.04	0.32947	114.35	5.305	2156.416734724	0.26805	8.008	-8.7361	0.58621	0.49809	0.96931 (max o	10	7085; 5998; 1887; 34	-0.0554845	0.50876427		1.0
	ZINC22926908	-7.6	404.553	-2.1201	7	3	339.07	0.24547	59.68	4.9976	2156.416734724	0.26805	7.7864	-7.9094	0.62069	0.5852	0.88492 (max o	2	6270; 1888	-0.8185227	-0.2489678		1.0
	ZINC22936962	-7.6	379.547	-0.195	6	2	289.6	0.25117	97.5	7.56	2156.416734724	0.29898	5.8613	-0.65222	0.57692	0.49404		0		-0.6046834	-0.773127		1.0
	ZINC30879632	-7.6	380.463	-2.1153	8	4	301.39	0.30472	95.7	3.3764	2156.416734724	0.28791	7.7816	-7.3472	0.55556	0.59508	0.91804 (max o	4	6832; 3002; 4757; 51	0.28994435	-0.0893403		1.0
	ZINC30879638	-7.6	361.417	-1.4473	7	4	289	0.31972	96.37	2.6188	2156.416734724	0.29898	7.1136	-4.8408	0.53846	0.52454	0.9615 (max of	4	7399; 6832; 3852; 43	0.23695444	-0.1455132		1.0
	ZINC30879796	-7.6	370.919	-2.0963	5	4	287.54	0.21399	70.74	-0.21108	2156.416734724	0.31094	7.7626	-6.7419	0.56	0.4612	0.85446	1	6584	0.31156364	0.8720403		1.0
	ZINC31157461	-7.6	414.362	-2.5155	11	4	272.1	0.47218	161.21	-2.4751	2156.416734724	0.26805	8.1818	-9.3845	0.44828	0.35136	0.95372 (max o	3	2798; 3237; 3646	0.8698253	-0.0364626		1.0
	ZINC32123853	-7.6	362.424	-2.8007	7	2	268.93	0.30328	86.5	3.9633	2156.416734724	0.29898	8.467	-9.3676	0.46154	0.46995	0.81466	1	5616	-0.8109669	0.5310232		1.0
	ZINC32123854	-7.6	360.409	-2.657	7	1	267.83	0.3043	83.34	3.5553	2156.416734724	0.29898	8.3233	-8.8869	0.46154	0.47347	0.81466	1	5615	-0.8239215	0.5123999		1.0
	ZINC32123854	-7.6	359.401	-2.7886	7	0	253.79	0.26581	82.14	3.8953	2156.416734724	0.29898	8.4549	-9.3271	0.46154	0.51491		0		0.32319885	0.91797835		1.0
	ZINC32123973	-7.6	387.395	0.1367	10	2	292.42	0.37638	126.51	0.40949	2156.416734724	0.27762	5.5296	0.49239	0.46429	0.53047	0.9543 (max of	4	5988; 5986; 5619; 56	-0.6326853	0.21874541		1.0
	ZINC32123973	-7.6	388.403	-1.8411	10	3	281.45	0.35207	127.76	-0.11909	2156.416734724	0.27762	7.5074	-6.6317	0.46429	0.53047	0.92774 (max o	8	6308; 5988; 5990; 59	-0.6372768	0.16632813		1.0

Structure	Molecule Name	affinity	Total Molweight	cLogP	H-Acceptors	H-Donors	Total Surface Area	Relative PSA	Polar Surface Area	Druglikeness	Kd (nM) @ 20°C	LE from K...	LLE from Kd ...	LELP from Kd (n...	Shape Index	Molecular...	Neighbor Si...	Neig...	Neighbor	Neighbor ...	Neighbor ...	Ring Systems	frequency
	ZINC32123973	-7.6	388.403	-1.8411	10	3	281.45	0.35207	127.76	-0.11909	2156.416734724	0.27762	7.5074	-6.6317	0.46429	0.53047	0.93686 (max o	5	5988; 5987; 5986; 56	-0.6067825	0.19594026		1.0
	ZINC35442733	-7.6	389.542	-1.3331	7	3	319.22	0.23059	82.66	1.1649	2156.416734724	0.27762	6.9994	-4.8018	0.57143	0.54439		0		-0.4608951	-0.8834019		1.0
	ZINC35454637	-7.6	437.463	-0.941	9	3	323.23	0.34867	153.42	-0.45976	2156.416734724	0.25076	6.6073	-3.7526	0.48387	0.48961	0.95771 (max o	3	6877; 6323; 4405	-0.8707712	-0.1174175		1.0
	ZINC35456916	-7.6	404.367	-3.0019	11	6	270.55	0.48656	175.37	-5.2776	2156.416734724	0.27762	8.6682	-10.813	0.46429	0.37407	0.96519 (max o	3	6332; 6331; 4832	-0.2032477	-0.8826224		1.0
	ZINC35485056	-7.6	402.423	-1.6076	10	6	280.65	0.50846	196.87	-11.086	2156.416734724	0.28791	7.2739	-5.5838	0.62963	0.65799		0		0.19178723	-0.7896452		1.0
	ZINC39962692	-7.6	408.429	-3.7745	10	1	290.19	0.36183	128.31	5.4137	2156.416734724	0.26805	9.4408	-14.081	0.62069	0.40181	0.82079	1	2381	-0.7045872	0.5043492		1.0
	ZINC67912714	-7.6	368.424	-0.6037	7	5	238.11	0.37184	127.45	-0.51541	2156.416734724	0.29898	6.27	-2.0192	0.34615	0.34106	0.92368 (max o	3	2317; 3103; 3104	0.29803118	0.5844607		1.0
	ZINC67913641	-7.6	373.333	-4.3309	10	4	248.42	0.49416	165.81	-2.0548	2156.416734724	0.29898	9.9972	-14.486	0.46154	0.37855	0.91652 (max o	4	1700; 2023; 3121; 32	-0.6657792	0.10485297		1.0
	ZINC68606257	-7.6	420.643	-4.8135	7	3	369.1	0.18212	70.9	5.3915	2156.416734724	0.25911	10.48	-18.577	0.56667	0.57936	0.82862	1	5676	0.77786773	0.51252574		1.0
	ZINC68606257	-7.6	421.651	-6.1798	7	4	383.14	0.21209	72.1	4.4132	2156.416734724	0.25911	11.846	-23.85	0.56667	0.58499	0.82862	1	5675	0.7760326	0.4917517		1.0
	ZINC70666217	-7.6	351.381	-0.4551	6	0	259.15	0.23346	80.75	3.4863	2156.416734724	0.29898	6.1214	-1.5222	0.5	0.54113	0.9464 (max of	3	1649; 1655; 1972	0.17649326	0.7698875		1.0
	ZINC71405304	-7.6	360.437	0.3126	7	1	253.42	0.31063	98.26	6.2433	2156.416734724	0.31094	5.3537	1.0053	0.56	0.43427	0.84304 (max o	3	6906; 2087; 4130	0.55585235	0.5163355		1.0
	ZINC72320163	-7.6	434.524	-0.8391	9	7	299.02	0.37355	160.07	-4.7016	2156.416734724	0.25911	6.5054	-3.2383	0.36667	0.44569	0.96092 (max o	3	3547; 4803; 5195	-0.4846021	-0.7177905		1.0
	ZINC72325410	-7.6	363.412	-2.758	8	2	290.23	0.3203	95.37	6.1898	2156.416734724	0.29898	8.4243	-9.2247	0.57692	0.56964		0		0.7371689	0.62409544		1.0
	ZINC72325570	-7.6	390.438	-0.3456	9	1	299.89	0.28727	92.7	4.9305	2156.416734724	0.27762	6.0119	-1.2449	0.60714	0.55513	0.86222 (max o	3	7113; 5691; 4452	-0.1554898	-0.3643830		1.0
	ZINC72325570	-7.6	391.446	-2.4553	9	2	313.93	0.31915	93.9	2.2683	2156.416734724	0.27762	8.1216	-8.844	0.60714	0.55849	0.88737 (max o	3	7113; 4452; 5690	-0.1343007	-0.3616663		1.0
	ZINC67913531	-7.6	429.576	0.7542	7	2	335.06	0.31466	148.83	-1.2855	2156.416734724	0.26805	4.9121	2.8137	0.44828	0.55891	0.97176 (max o	3	7571; 6675; 4864	0.73567855	0.272106		0.0
	ZINC35457502	-7.6	436.540	0.256	9	8	340.73	0.33692	171.07	-3.3718	2156.416734724	0.25911	5.4103	0.98798	0.66667	0.73168	0.99612 (max o	3	7263; 7088; 6006	-0.9251812	-0.2809800		0.0
	ZINC13535643	-7.6	402.335	-11.312	13	3	300.83	0.57428	246.36	1.9917	2156.416734724	0.27762	16.978	-40.746	0.53571	0.65879		0		0.6545678	-0.7500779		0.0
	ZINC02157495	-7.5	389.383	-1.9864	9	2	295.95	0.36952	133.86	0.18211	2560.287713445	0.27397	7.5781	-7.2504	0.64286	0.56407	0.96297 (max o	4	6390; 1647; 2738; 31	0.15708356	0.0764296		172.0
	ZINC02113556	-7.5	417.436	-1.2983	9	2	319.45	0.34234	133.86	5.6987	2560.287713445	0.25571	6.89	-5.0773	0.6	0.6106	0.9561 (max of	7	6076; 2732; 2740; 40	0.04068480	0.04064831		172.0
	ZINC02117617	-7.5	447.462	-1.3683	10	2	341.71	0.3493	143.09	5.6987	2560.287713445	0.23972	6.96	-5.7078	0.5625	0.596	0.95983 (max o	7	6076; 2740; 3585; 44	0.02182701	0.04956492		172.0
	ZINC02120847	-7.5	352.749	-0.808	7	1	256.14	0.3313	104.76	3.3896	2560.287713445	0.31963	6.3997	-2.5279	0.625	0.53199	0.91652 (max o	3	6387; 3565; 4011	0.12668718	0.12782519		172.0
	ZINC02159258	-7.5	362.357	-1.1247	8	1	273.98	0.34623	113.99	4.8454	2560.287713445	0.29504	6.7164	-3.812	0.53846	0.49253	0.96438 (max o	7	7120; 6387; 5749; 35	0.04068128	0.12944078		172.0
	ZINC02118762	-7.5	362.357	-1.1247	8	1	273.98	0.34623	113.99	4.8454	2560.287713445	0.29504	6.7164	-3.812	0.53846	0.51016	0.96438 (max o	7	6924; 6760; 5749; 35	0.03764958	0.10954398		172.0
	ZINC04080978	-7.5	362.357	-1.1247	8	1	273.98	0.34623	113.99	4.8454	2560.287713445	0.29504	6.7164	-3.812	0.53846	0.50879	0.96438 (max o	8	6924; 6760; 6197; 35	9.0173475E	0.10860015		172.0
	ZINC02118647	-7.5	362.357	-1.0296	8	1	276.74	0.34278	113.99	1.0104	2560.287713445	0.29504	6.6213	-3.4896	0.61538	0.54483	0.91768 (max o	7	6924; 6760; 6387; 61	0.03621132	0.08725167		172.0
	ZINC03874916	-7.5	441.440	-6.0726	12	3	324.93	0.51097	237.22	-4.4889	2560.287713445	0.25571	11.664	-23.748	0.56667	0.68953	0.97304 (max o	3	6104; 4056; 5347	-0.6149409	0.63658077		134.0
	ZINC04027453	-7.5	421.496	-1.1873	10	5	321.32	0.34825	171.2	-11.406	2560.287713445	0.25571	6.779	-4.6432	0.6	0.58554	0.96832 (max o	6	6041; 5797; 5746; 41	-0.1100914	-0.4679430		134.0
	ZINC04043726	-7.5	421.496	-1.1873	10	5	321.32	0.34825	171.2	-11.406	2560.287713445	0.25571	6.779	-4.6432	0.6	0.58305	0.96832 (max o	5	6666; 5797; 4114; 53	-0.0884414	-0.4388223		134.0
	ZINC38573628	-7.5	413.900	-1.3058	8	4	313.26	0.33167	146.2	1.8294	2560.287713445	0.27397	6.8975	-4.7662	0.46429	0.56805	0.98866 (max o	4	6196; 5251; 5461; 54	0.6753405	0.30745214		134.0
	ZINC05294153	-7.5	421.496	-1.1873	10	5	321.32	0.34825	171.2	-11.406	2560.287713445	0.25571	6.779	-4.6432	0.6	0.58417	0.96832 (max o	7	7252; 6779; 6041; 41	-0.1078177	-0.4463704		134.0
	ZINC61996990	-7.5	434.539	-4.0587	10	6	332.34	0.35256	187.99	1.0827	2560.287713445	0.24746	9.6504	-16.402	0.58065	0.62513	0.92304 (max o	5	7252; 6666; 4578; 57	-0.1256368	-0.4345640		134.0
	ZINC04029401	-7.5	364.377	-2.4707	9	3	286.97	0.38617	136.66	-9.8067	2560.287713445	0.29504	8.0624	-8.374	0.65385	0.63416	0.92721 (max o	3	6415; 6416; 6156	-0.0116800	-0.4822623		134.0
	ZINC39321750	-7.5	406.481	-2.424	9	4	319.2	0.36122	155.07	2.9612	2560.287713445	0.26452	8.0157	-9.1637	0.58621	0.57667	0.89169 (max o	2	6391; 6309	0.01198006	0.63947666		134.0
	ZINC38573615	-7.5	397.901	-0.284	7	3	308.17	0.29464	125.97	3.5239	2560.287713445	0.28412	5.8757	-0.99959	0.48148	0.5813	0.93686 (max o	3	7166; 6485; 6196	0.6674949	0.32891104		134.0
	ZINC31166571	-7.5	366.277	-4.9637	10	2	267.45	0.47456	173.32	-1.6285	2560.287713445	0.29504	10.555	-16.824	0.53846	0.5543	0.96438 (max o	3	6862; 6861; 6296	-0.6211667	0.71892995		134.0
	ZINC31168621	-7.5	350.278	-4.1042	9	1	263.19	0.43246	153.09	-6.0092	2560.287713445	0.30685	9.6959	-13.375	0.6	0.52844	0.97396 (max o	7	7897; 7798; 7644; 72	0.81649363	0.06995225		134.0
	ZINC31168616	-7.5	350.278	-4.1042	9	1	263.19	0.43246	153.09	-6.0092	2560.287713445	0.30685	9.6959	-13.375	0.6	0.52844	0.96832 (max o	7	7897; 7798; 7644; 72	0.8054068	0.10673437		134.0
	ZINC31155516	-7.5	363.385	-0.0989	7	3	289.14	0.31009	127.12	-4.6148	2560.287713445	0.29504	5.6906	-0.3352	0.61538	0.61092		0		-0.819221	-0.5416342		134.0
	ZINC04221586	-7.5	440.540	-3.6162	11	7	312.47	0.5492	231.55	3.8067	2560.287713445	0.27397	9.2079	-13.199	0.53571	0.58636	0.92094	1	1135	0.79481244	0.4962546		127.0
	ZINC04085243	-7.5	355.477	-1.3821	7	3	284.27	0.31941	125.97	2.3079	2560.287713445	0.30685	6.9738	-4.5042	0.6	0.5916	0.95 (max of 8)	8	6746; 6128; 6126; 61	0.4751668	0.01378664		127.0
	ZINC12889065	-7.5	369.504	-0.9277	7	3	298.03	0.30467	125.97	2.9235	2560.287713445	0.29504	6.5194	-3.1443	0.61538	0.59818	0.96721 (max o	10	7331; 6227; 6177; 61	0.50660443	-0.0054238		127.0

Structure	Molecule Name	affinity	Total Molweight	cLogP	H-Acceptors	H-Donors	Total Surface Area	Relative PSA	Polar Surface Area	Druglikeness	Kd (nM) @ 20°C	LE from K...	LLE from Kd ...	LELP from Kd (n...	Shape Index	Molecular...	Neighbor Si...	Neig...	Neighbor	Neighbor ...	Neighbor ...	Ring Systems	frequency
	ZINC04089155	-7.5	355.477	-1.3821	7	3	284.27	0.31941	125.97	2.3079	2560.287713445	0.30685	6.9738	-4.5042	0.6	0.5881	0.95 (max of 4)	4	7137; 4963; 5751; 57	0.495731	0.05187099		127.0
	ZINC04084110	-7.5	369.504	-0.9277	7	3	298.03	0.30467	125.97	3.0831	2560.287713445	0.29504	6.5194	-3.1443	0.61538	0.60405	0.95257 (max o	12	7331; 7310; 6746; 65	0.52523834	0.02995082		127.0
	ZINC04084712	-7.5	383.531	-0.4733	7	3	311.79	0.29122	125.97	3.7029	2560.287713445	0.28412	6.065	-1.6659	0.62963	0.60187	0.94927 (max o	9	7331; 7311; 7135; 67	0.5470768	0.02862289		127.0
	ZINC04085103	-7.5	359.489	-2.1239	7	3	279.76	0.38708	151.27	1.071	2560.287713445	0.31963	7.7156	-6.6448	0.70833	0.61376	0.95384 (max o	11	7764; 7587; 7470; 74	0.53792304	0.11883205		127.0
	ZINC67913941	-7.5	424.441	-1.0613	11	5	315.59	0.41693	172.21	-20.492	2560.287713445	0.26452	6.653	-4.0121	0.58621	0.56882	0.96682 (max o	2	7749; 7660	-0.6569463	0.3944657		120.0
	ZINC37253580	-7.5	382.400	-3.9574	12	5	268.08	0.55032	214.72	-1.7419	2560.287713445	0.28412	9.5491	-13.929	0.55556	0.48268	0.93996 (max o	6	1407; 3615; 3674; 39	0.5715747	0.14219517		67.0
	ZINC03869384	-7.5	384.416	-3.7275	11	4	265.34	0.56735	212.38	-0.44329	2560.287713445	0.29504	9.3192	-12.634	0.57692	0.48318	0.96775 (max o	8	5841; 5788; 1712; 27	0.61401063	0.17801799		67.0
	ZINC11726792	-7.5	382.400	-3.9574	12	5	268.08	0.55032	214.72	-1.7419	2560.287713445	0.28412	9.5491	-13.929	0.55556	0.48268	0.93996 (max o	6	6015; 1407; 3940; 39	0.5903006	0.13061154		67.0
	ZINC04514046	-7.5	370.389	-4.1819	11	4	251.58	0.59838	212.38	-0.65956	2560.287713445	0.30685	9.7736	-13.629	0.56	0.46938	0.96775 (max o	6	5841; 3615; 3674; 45	0.58838224	0.15228175		67.0
	ZINC03871402	-7.5	423.170	-13.388	15	2	266.13	0.71134	263.54	-22.95	2560.287713445	0.28412	18.98	-47.121	0.44444	0.42253	0.93996 (max o	9	6403; 6098; 6094; 36	0.6461204	0.44948485		67.0
	ZINC03871402	-7.5	424.178	-12.14	15	3	264.95	0.71006	260.71	-32.906	2560.287713445	0.28412	17.732	-42.73	0.44444	0.40459	0.93996 (max o	9	6404; 6099; 6095; 36	0.6115256	0.49941355		67.0
	ZINC08616446	-7.5	393.355	-0.2858	12	1	282.15	0.47553	157.75	1.9052	2560.287713445	0.27397	5.8775	-1.0432	0.42857	0.30366	0.9543 (max of	5	6179; 5015; 5302; 54	-0.6495744	0.33736017		67.0
	ZINC14414157	-7.5	351.318	-0.7704	11	2	248.49	0.49994	151.68	1.3398	2560.287713445	0.30685	6.3621	-2.5107	0.44	0.2717	0.9402 (max of	7	6965; 6536; 4979; 49	-0.5849519	0.33591107		67.0
	ZINC04204377	-7.5	393.355	-0.2858	12	1	282.15	0.47553	157.75	1.9052	2560.287713445	0.27397	5.8775	-1.0432	0.42857	0.30182	0.9428 (max of	8	6536; 6179; 5879; 58	-0.6169470	0.35539204		67.0
	ZINC12655077	-7.5	355.350	-2.2067	11	5	250.69	0.47401	157.22	0.75202	2560.287713445	0.30685	7.7984	-7.1915	0.52	0.45357		0		0.14486276	0.97531736		67.0
	ZINC05152239	-7.5	354.358	-0.9613	9	5	258.01	0.41975	137.35	-3.0133	2560.287713445	0.30685	6.553	-3.1328	0.52	0.45182	0.90535 (max o	4	6487; 6419; 3710; 41	0.44185373	0.56540287		49.0
	ZINC77257434	-7.5	402.394	-1.5423	10	6	283.06	0.42973	166.14	-10.796	2560.287713445	0.27397	7.134	-5.6295	0.57143	0.52229	0.94573 (max o	5	6376; 4445; 4455; 60	0.1239973	0.46873185		49.0
	ZINC77257568	-7.5	446.447	-1.1579	11	6	319.08	0.41256	175.37	-5.239	2560.287713445	0.24746	6.7496	-4.6792	0.54839	0.4628	0.99343 (max o	5	6682; 6376; 3985; 44	0.07089598	0.48700407		49.0
	ZINC72320192	-7.5	416.421	-1.0879	10	6	296.82	0.40981	166.14	-5.3918	2560.287713445	0.26452	6.6796	-4.1127	0.58621	0.5384	0.99279 (max o	7	6682; 6376; 6064; 60	0.12703902	0.48993808		49.0
	ZINC72320191	-7.5	416.421	-1.0879	10	6	296.82	0.40981	166.14	-5.3918	2560.287713445	0.26452	6.6796	-4.1127	0.58621	0.5384	0.99279 (max o	7	6682; 6376; 6064; 60	0.10689045	0.4809084		49.0
	ZINC77257564	-7.5	446.447	-1.1579	11	6	319.08	0.41256	175.37	-5.239	2560.287713445	0.24746	6.7496	-4.6792	0.54839	0.46022	0.99343 (max o	5	6682; 3985; 6065; 60	0.09245195	0.5160938		49.0
	ZINC04043066	-7.5	353.413	0.9764	7	4	267.32	0.31348	108.25	-2.6319	2560.287713445	0.30685	4.6153	3.182	0.52	0.46255	0.96832 (max o	3	6487; 6419; 4512	0.50104165	0.5530004		49.0
	ZINC13537304	-7.5	379.213	-7.336	12	3	247.57	0.5869	207.2	-28.381	2560.287713445	0.30685	12.928	-23.908	0.48	0.44573		0		0.6484714	-0.7604598		49.0
	ZINC12870894	-7.5	441.503	-2.3964	11	3	342.73	0.37175	156.97	-62.466	2560.287713445	0.24746	7.9881	-9.6841	0.6129	0.62954	0.95372 (max o	3	2904; 5036; 5363	0.4841559	-0.0626345		47.0
	ZINC38557523	-7.5	398.478	-1.1208	9	2	310.76	0.33116	127.87	-61.255	2560.287713445	0.27397	6.7125	-4.091	0.57143	0.60735	0.9426 (max of	13	6020; 6019; 2035; 29	0.4964023	-0.0830199		47.0
	ZINC38557565	-7.5	428.504	-1.6882	10	3	329.61	0.35196	148.1	-62.155	2560.287713445	0.25571	7.2799	-6.6021	0.53333	0.60559	0.9897 (max of	13	6021; 2904; 4845; 50	0.466068	-0.0981216		47.0
	ZINC38557597	-7.5	442.531	-1.2338	10	3	343.37	0.33786	148.1	-55.361	2560.287713445	0.24746	6.8255	-4.9859	0.51613	0.6128	0.99016 (max o	11	4514; 4845; 5074; 50	0.45890254	-0.0799716		47.0
	ZINC38557554	-7.5	398.478	-1.1208	9	2	310.76	0.33116	127.87	-53.284	2560.287713445	0.27397	6.7125	-4.091	0.57143	0.58974	0.97773 (max o	16	7602; 6021; 3635; 40	0.49703762	-0.1161907		47.0
	ZINC04073604	-7.5	400.450	-2.3609	10	3	304.85	0.38055	148.1	-54.914	2560.287713445	0.27397	7.9526	-8.6174	0.57143	0.58779	0.98866 (max o	14	7602; 6021; 6020; 60	0.4835147	-0.1300000		47.0
	ZINC12865807	-7.5	442.531	-1.2338	10	3	343.37	0.33786	148.1	-57.405	2560.287713445	0.24746	6.8255	-4.9859	0.51613	0.61744	0.99016 (max o	11	6021; 6020; 6019; 60	0.45141035	-0.1093964		47.0
	ZINC08918351	-7.5	442.531	-1.2338	10	3	343.37	0.33786	148.1	-57.405	2560.287713445	0.24746	6.8255	-4.9859	0.51613	0.61744	0.99016 (max o	11	6021; 6020; 6019; 60	0.46926716	-0.1188457		47.0
	ZINC02109058	-7.5	370.424	-1.7935	9	2	286	0.35983	127.87	-53.594	2560.287713445	0.29504	7.3852	-6.0787	0.61538	0.58769	0.93996 (max o	8	7602; 6384; 6019; 60	0.5161635	-0.0820242		47.0
	ZINC13543247	-7.5	440.102	-7.4984	13	4	248.84	0.6466	217.22	-27.898	2560.287713445	0.30685	13.09	-24.437	0.48	0.49754	0.90097 (max o	4	2557; 2739; 4059; 42	-0.2862757	-0.3599186		44.0
	ZINC05224737	-7.5	361.206	-8.4657	13	4	230.21	0.69893	217.22	-20.626	2560.287713445	0.31963	14.057	-26.486	0.41667	0.39494	0.92257 (max o	4	6997; 6101; 2739; 40	-0.3156457	-0.4097608		44.0
	ZINC03870126	-7.5	366.174	-9.7178	13	4	227.86	0.69784	218.63	-30.796	2560.287713445	0.31963	15.31	-30.403	0.5	0.57412	0.92619 (max o	5	6717; 6096; 4934; 57	-0.2704529	-0.7405979		41.0
	ZINC03870126	-7.5	365.166	-10.965	13	3	229.04	0.6994	221.46	-26.867	2560.287713445	0.31963	16.557	-34.306	0.5	0.59279	0.92619 (max o	5	6718; 6097; 5732; 49	-0.2939147	-0.7019138		41.0
	ZINC20757905	-7.5	449.507	-2.0188	10	3	333.77	0.40417	173.04	6.9	2560.287713445	0.24746	7.6105	-8.1582	0.48387	0.58224	0.93686 (max o	3	4329; 4665; 4761	0.3509566	0.7267496		36.0
	ZINC20426259	-7.5	364.401	-1.6801	8	2	268.8	0.41071	143.94	5.8769	2560.287713445	0.30685	7.2718	-5.4754	0.6	0.58457	0.97331 (max o	3	6274; 5949; 4019	0.626535	0.13747449		36.0
	ZINC38570794	-7.5	376.388	-1.8191	9	3	278.4	0.38078	138.87	4.1721	2560.287713445	0.28412	7.4108	-6.4026	0.44444	0.54628	0.98806 (max o	8	6408; 4244; 4760; 51	0.26110137	0.70350474		36.0
	ZINC13548190	-7.5	376.388	-1.8191	9	3	278.4	0.38078	138.87	4.1721	2560.287713445	0.28412	7.4108	-6.4026	0.44444	0.54716	0.98806 (max o	9	7062; 6408; 6023; 59	0.2674721	0.72293144		36.0
	ZINC20757947	-7.5	388.442	-0.1246	8	2	298.07	0.31171	118.64	5.7639	2560.287713445	0.27397	5.7163	-0.45479	0.46429	0.5544	0.93069 (max o	11	7062; 6691; 6408; 60	0.31778288	0.69134516		36.0
	ZINC20758386	-7.5	350.374	-2.1345	8	2	255.04	0.43287	143.94	5.8909	2560.287713445	0.31963	7.7262	-6.678	0.58333	0.58408	0.96029 (max o	2	6274; 5905	0.62856746	0.11392268		36.0
	ZINC03869247	-7.5	423.170	-13.388	15	2	266.13	0.71134	263.54	-22.848	2560.287713445	0.28412	18.98	-47.121	0.44444	0.4085	0.95493 (max o	9	6403; 6098; 6094; 57	0.6799052	0.460196		33.0

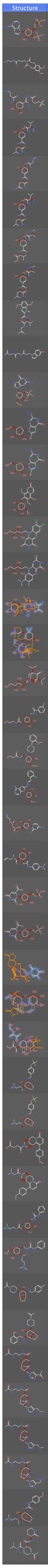

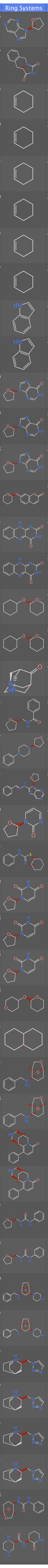

Structure	Molecule Name	affinity	Total Molweight	cLogP	H-Acceptors	H-Donors	Total Surface Area	Relative PSA	Polar Surface Area	Druglikeness	Kd (nM) @ 20°C	LE from K...	LLE from Kd ...	LELP from Kd (n...	Shape Index	Molecular...	Neighbor Si...	Neig...	Neighbor	Neighbor ...	Neighbor ...	Ring Systems	frequency
	ZINC03869247	-7.5	424.178	-12.14	15	3	264.95	0.71006	260.71	-32.8	2560.287713445	0.28412	17.732	-42.73	0.44444	0.39014	0.95493 (max o	9	6404; 6099; 6095; 57	0.64761555	0.50065106		33.0
	ZINC12902033	-7.5	391.423	-0.0252	9	2	291.26	0.31168	108.41	4.821	2560.287713445	0.27397	5.6169	-0.091981	0.60714	0.43286	0.97346 (max o	3	4246; 4670; 5078	0.8739664	-0.2188667		29.0
	ZINC04200818	-7.5	407.466	-7.4702	11	6	289.47	0.50634	211.91	1.354	2560.287713445	0.28412	13.062	-26.293	0.55556	0.57387	0.93373 (max o	9	6431; 6133; 3650; 36	-0.6118339	-0.1528896		29.0
	ZINC03838851	-7.5	399.510	-2.5988	9	6	306.16	0.36337	158.36	5.2903	2560.287713445	0.27397	8.1905	-9.4857	0.5	0.55109	0.9339 (max of	8	6659; 6400; 6090; 60	-0.5914640	-0.1881619		29.0
	ZINC04221601	-7.5	358.374	-5.9776	11	7	259.65	0.53749	201.45	5.1901	2560.287713445	0.30685	11.569	-19.481	0.56	0.55731	0.91652 (max o	5	6400; 6133; 4538; 53	-0.5526883	-0.1193464		29.0
	ZINC03838850	-7.5	385.483	-4.8964	9	6	293.89	0.40148	169.39	4.0161	2560.287713445	0.28412	10.488	-17.234	0.51852	0.55181	0.92418 (max o	8	6699; 6400; 6090; 60	-0.5811238	-0.1711930		29.0
	ZINC39560953	-7.5	372.440	-2.9002	9	5	281.38	0.37664	141.57	1.7936	2560.287713445	0.29504	8.4919	-9.8297	0.53846	0.55036	0.8934 (max of	3	6132; 5003; 6029	-0.5682565	-0.2284936		29.0
	ZINC39560950	-7.5	371.456	-3.2715	9	6	281.4	0.39534	158.36	5.4293	2560.287713445	0.29504	8.8632	-11.088	0.53846	0.55034	0.93373 (max o	9	6699; 6400; 6352; 60	-0.5727430	-0.1913267		29.0
	ZINC04000250	-7.5	390.415	-1.6457	9	2	300.82	0.34303	121.72	4.0203	2560.287713445	0.27397	7.2374	-6.0069	0.5	0.37475	0.93686 (max o	4	2300; 3184; 3185; 45	-0.2423878	0.5529592		28.0
	ZINC40313061	-7.5	381.205	-1.9175	7	2	254.43	0.32697	103.26	-0.15442	2560.287713445	0.33353	7.5092	-5.7491	0.69565	0.59384	0.95111 (max o	3	6896; 5579; 5580	-0.2847964	0.8850321		28.0
	ZINC03872750	-7.5	361.206	-8.4657	13	4	230.21	0.69893	217.22	-20.626	2560.287713445	0.31963	14.057	-26.486	0.41667	0.37572	0.9115 (max of	4	6406; 6101; 1995; 40	-0.3806801	-0.4081089		26.0
	ZINC02461679	-7.5	362.139	-1.7236	10	5	206.36	0.57385	155.22	-0.33304	2560.287713445	0.36529	7.3153	-4.7184	0.47619	0.39342	0.90776 (max o	4	7466; 7301; 5840; 40	-0.2203472	-0.4331919		26.0
	ZINC09209380	-7.5	362.139	-1.7236	10	5	206.36	0.57385	155.22	-0.33304	2560.287713445	0.36529	7.3153	-4.7184	0.47619	0.39446	0.90776 (max o	3	7466; 7301; 5710	-0.2425626	-0.4615928		26.0
	ZINC49180912	-7.5	400.335	-1.3233	11	5	273.04	0.47077	164.37	-5.8493	2560.287713445	0.27397	6.915	-4.8301	0.42857	0.44178	0.94589 (max o	3	6630; 3539; 3893	-0.3966472	0.5579252		24.0
	ZINC13543025	-7.5	417.204	-1.5421	10	5	270.72	0.43037	155.05	6.2637	2560.287713445	0.28412	7.1338	-5.4277	0.44444	0.45827	0.97658 (max o	11	6010; 1661; 2455; 36	0.4880889	0.81262463		24.0
	ZINC35653106	-7.5	405.410	-2.5138	11	5	286.96	0.41839	158.29	6.8607	2560.287713445	0.26452	8.1055	-9.5032	0.41379	0.47557	0.95908 (max o	8	3672; 3936; 4417; 48	0.47203505	0.8283308		24.0
	ZINC37874880	-7.5	380.372	-6.2025	12	8	237.3	0.5799	225	-2.0341	2560.287713445	0.29504	11.794	-21.022	0.53846	0.50398	0.85984 (max o	2	3651; 5647	0.59526646	0.7955279		23.0
	ZINC72320403	-7.5	390.471	-0.2607	8	6	283.07	0.34832	139.84	-5.146	2560.287713445	0.28412	5.8524	-0.91758	0.48148	0.45742	0.95493 (max o	3	6917; 4446; 4447	-0.3820142	0.7946455		23.0
	ZINC19323151	-7.5	357.469	-2.4049	7	5	264.45	0.3274	105.67	3.1491	2560.287713445	0.30685	7.9966	-7.8375	0.44	0.62148	0.97118 (max o	6	7368; 7367; 7200; 70	-0.2832696	0.7077775		23.0
	ZINC08624584	-7.5	370.376	-0.8412	8	4	274.53	0.34677	117.12	-1.516	2560.287713445	0.29504	6.4329	-2.8511	0.65385	0.56877	0.9302 (max of	8	1496; 1765; 2118; 28	0.3317784	-0.4018125		22.0
	ZINC20465497	-7.5	409.481	-1.1268	9	3	309.61	0.28358	103.73	5.6093	2560.287713445	0.26452	6.7185	-4.2597	0.55172	0.53019	0.94862 (max o	6	7729; 7049; 6825; 68	-0.3390735	0.13744387		21.0
	ZINC08740531	-7.5	419.828	2.4172	11	5	292.57	0.43518	163.93	1.3996	2560.287713445	0.26452	3.1745	9.138	0.48276	0.31394	0.97649 (max o	10	6759; 6484; 6205; 61	-0.2530344	-0.6219805		20.0
	ZINC13431055	-7.5	445.237	-10.296	15	4	286.85	0.65069	263.59	-43.957	2560.287713445	0.27397	15.888	-37.581	0.60714	0.65107	0.93686 (max o	4	6526; 6228; 4674; 50	-0.6301565	-0.3857090		20.0
	ZINC08636383	-7.5	404.533	0.9792	7	3	300.57	0.28878	113.7	4.3836	2560.287713445	0.27397	4.6125	3.5741	0.57143	0.47659	0.9083 (max of	7	6182; 2881; 2883; 32	-0.4945917	0.30972674		17.0
	ZINC03870127	-7.5	366.174	-9.7178	13	4	227.86	0.69784	218.63	-30.796	2560.287713445	0.31963	15.31	-30.403	0.5	0.57479	0.92257 (max o	5	6717; 6096; 5732; 49	-0.2277326	-0.7441719		17.0
	ZINC03870127	-7.5	365.166	-10.965	13	3	229.04	0.6994	221.46	-26.867	2560.287713445	0.31963	16.557	-34.306	0.5	0.59344	0.91891 (max o	5	6718; 6097; 4935; 57	-0.2517291	-0.7073538		17.0
	ZINC03586101	-7.5	354.095	-0.8884	7	3	182.47	0.42358	99.1	3.3731	2560.287713445	0.45124	6.4801	-1.9688	0.58824	0.50202	0.90064 (max o	4	7769; 7669; 7505; 73	0.43426389	0.36072746		17.0
	ZINC04556850	-7.5	356.323	-4.03	11	7	235.73	0.55869	178.53	-7.2031	2560.287713445	0.31963	9.6217	-12.608	0.45833	0.45757	0.94718 (max o	7	6731; 6732; 6446; 64	0.5771163	-0.4426688		17.0
	ZINC67913293	-7.5	444.475	-0.5622	10	6	314.74	0.39614	173.98	-11.767	2560.287713445	0.24746	6.1539	-2.2719	0.41935	0.54324	0.96739 (max o	5	7449; 7106; 6674; 52	0.38199785	0.33782405		16.0
	ZINC22922211	-7.5	421.458	-2.1747	7	3	308.77	0.23312	72.21	-4.5845	2560.287713445	0.26452	7.7664	-8.2212	0.58621	0.5935	0.93069 (max o	7	7520; 7518; 7221; 65	0.4609181	-0.2404924		16.0
	ZINC22922211	-7.5	421.458	-5.617	7	3	323.99	0.26914	76.24	-8.8561	2560.287713445	0.26452	11.209	-21.234	0.58621	0.58484	0.93069 (max o	5	7519; 7517; 7220; 65	0.6103832	-0.0078028		16.0
	ZINC04237366	-7.5	395.434	-0.5644	7	0	293.38	0.2403	89.98	2.0753	2560.287713445	0.26452	6.1561	-2.1337	0.55172	0.48052	0.9561 (max of	9	1180; 2436; 2464; 45	-0.4987782	-0.3182101		14.0
	ZINC04237361	-7.5	382.482	-0.1937	6	1	303.2	0.20043	54.29	2.6952	2560.287713445	0.27397	5.7854	-0.70701	0.53571	0.44553	0.92206 (max o	6	6742; 3264; 4566; 49	-0.4571003	-0.2871145		14.0
	ZINC08643327	-7.5	381.451	-2.5221	9	6	306.24	0.37307	124.36	1.2725	2560.287713445	0.28412	8.1138	-8.877	0.66667	0.5975	0.94302	1	4656	0.34776878	-0.4756297		14.0
	ZINC20464967	-7.5	367.468	-2.3604	8	5	299.59	0.31143	98.5	4.4336	2560.287713445	0.29504	7.9521	-8.0001	0.57692	0.61452	0.93367 (max o	5	7397; 2593; 4301; 43	-0.2878972	0.00730066		14.0
	ZINC12483145	-7.5	380.438	-1.3713	7	2	268.87	0.2268	78.85	4.8754	2560.287713445	0.28412	6.963	-4.8265	0.59259	0.47414	0.96064 (max o	12	7384; 6847; 6765; 65	0.41080388	-0.1877409		14.0
	ZINC12483133	-7.5	378.447	-1.8178	8	3	268.87	0.27552	99.08	6.2154	2560.287713445	0.28412	7.4095	-6.3981	0.55556	0.46871	0.94883 (max o	8	7384; 6765; 6553; 65	0.40016812	-0.1570057		14.0
	ZINC08299772	-7.5	363.440	-3.6471	9	3	291.83	0.37159	110.42	2.3539	2560.287713445	0.29504	9.2388	-12.361	0.65385	0.5479	0.96721 (max o	5	4600; 5007; 5008; 58	0.12012665	-0.2151534		14.0
	ZINC08299075	-7.5	365.480	-5.3866	9	4	295.58	0.34894	109.96	1.9922	2560.287713445	0.29504	10.978	-18.257	0.69231	0.5688	0.96721 (max o	6	6175; 6174; 5814; 41	0.11235556	-0.2331057		14.0
	ZINC08299078	-7.5	365.480	-5.3866	9	4	295.58	0.34894	109.96	1.9922	2560.287713445	0.29504	10.978	-18.257	0.69231	0.5688	0.96721 (max o	6	6174; 5816; 5007; 50	0.08169427	-0.2119814		14.0
	ZINC08299048	-7.5	351.453	-7.6842	9	4	283.31	0.38784	120.99	0.70912	2560.287713445	0.30685	13.276	-25.043	0.68	0.55685	0.9654 (max of	7	6175; 6174; 5816; 58	0.0924678	-0.2324721		14.0
	ZINC20463958	-7.5	395.478	-2.0427	9	4	310.74	0.32246	96.73	3.3803	2560.287713445	0.27397	7.6344	-7.4559	0.60714	0.5364	0.95709 (max o	6	6809; 6810; 6565; 34	-0.7092625	-0.2861498		14.0
	ZINC08299447	-7.5	353.421	-0.7651	9	3	269.32	0.34769	110.85	6.3927	2560.287713445	0.30685	6.3568	-2.4934	0.48	0.56215	0.93348 (max o	4	938; 1544; 5011; 542	-0.1141668	0.29601932		13.0

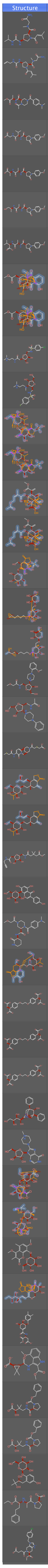

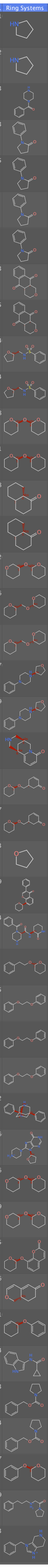

Structure	Molecule Name	affinity	Total Molweight	cLogP	H-Acceptors	H-Donors	Total Surface Area	Relative PSA	Polar Surface Area	Druglikeness	Kd (nM) @ 20°C	LE from K...	LLE from Kd ...	LELP from Kd (n...	Shape Index	Molecular...	Neighbor Si...	Neig...	Neighbor	Neighbor ...	Neighbor ...	Ring Systems	frequency
	ZINC03839046	-7.5	367.448	-0.3712	9	3	287.83	0.33857	124.84	6.1052	2560.287713445	0.29504	5.9629	-1.2581	0.5	0.61612	0.92032 (max o	4	7461; 7296; 5765; 57	-0.0701270	-0.0252261		13.0
	ZINC03839053	-7.5	365.432	-0.4616	9	3	289.11	0.33707	124.84	2.6186	2560.287713445	0.29504	6.0533	-1.5645	0.53846	0.63834	0.92032 (max o	4	7581; 7294; 5766; 57	-0.0506727	-0.0352002		13.0
	ZINC05396049	-7.5	404.465	0.3157	9	1	310.7	0.27123	99.26	2.1762	2560.287713445	0.26452	5.276	1.1935	0.55172	0.60387	0.94241 (max o	3	6453; 6454; 6452	0.06390142	0.41858113		13.0
	ZINC04084424	-7.5	390.415	-2.3692	9	2	297.07	0.34642	127.87	4.485	2560.287713445	0.27397	7.9609	-8.6477	0.60714	0.47922	0.96519 (max o	6	6658; 6022; 3202; 45	0.52816236	0.29186603		12.0
	ZINC04084383	-7.5	376.388	-2.6826	9	2	283.31	0.36324	127.87	6.0104	2560.287713445	0.28412	8.2743	-9.4419	0.62963	0.46807	0.96617 (max o	7	6658; 6425; 6022; 57	0.5420547	0.32348335		12.0
	ZINC04085151	-7.5	363.412	-0.257	8	3	278.14	0.31887	107.97	4.8383	2560.287713445	0.29504	5.8487	-0.87105	0.65385	0.46209	0.96438 (max o	4	6714; 3521; 5369; 57	0.5614751	0.3145424		12.0
	ZINC38570563	-7.5	404.441	-2.0099	9	2	308.07	0.33405	127.87	6.2137	2560.287713445	0.26452	7.6016	-7.5982	0.58621	0.48282	0.96931 (max o	7	6714; 6658; 6425; 35	0.56491095	0.2919017		12.0
	ZINC72319951	-7.5	364.349	0.171	8	2	252.33	0.37776	119.36	-2.1253	2560.287713445	0.29504	5.4207	0.57957	0.46154	0.40419	0.95857 (max o	9	6679; 2698; 3548; 39	0.7855329	-0.0694103		12.0
	ZINC72319949	-7.5	364.349	0.171	8	2	252.33	0.37776	119.36	-2.1253	2560.287713445	0.29504	5.4207	0.57957	0.46154	0.40419	0.95559 (max o	8	6679; 2698; 6058; 35	0.7795738	-0.1299625		12.0
	ZINC08643571	-7.5	436.935	-2.2234	9	5	321.03	0.36859	137.78	3.1396	2560.287713445	0.27397	7.8151	-8.1155	0.64286	0.58969	0.93985 (max o	2	5064; 5482	0.30806905	-0.2972894		12.0
	ZINC20464952	-7.5	406.497	-2.5669	8	4	305.31	0.31895	111.92	2.3771	2560.287713445	0.28412	8.1586	-9.0347	0.55556	0.60588	0.97658 (max o	4	7045; 6817; 6259; 43	-0.3953051	-0.1662694		12.0
	ZINC38664849	-7.5	421.267	-8.9493	14	8	261.12	0.6744	248.7	-32.77	2560.287713445	0.28412	14.541	-31.499	0.48148	0.51117	0.95201 (max o	4	3947; 5650; 5652; 60	0.06094633	-0.4690548		12.0
	ZINC38664849	-7.5	420.259	-10.197	14	7	262.3	0.67587	251.53	-27.673	2560.287713445	0.28412	15.789	-35.89	0.48148	0.52732	0.95201 (max o	5	7322; 6027; 3946; 56	0.03022099	-0.4932371		12.0
	ZINC31170272	-7.5	438.471	1.6499	9	2	324.65	0.33377	136.43	-5.8488	2560.287713445	0.24746	3.9418	6.6674	0.45161	0.53187	0.97199 (max o	8	7423; 6876; 6869; 59	0.40806523	0.22851078		12.0
	ZINC31170269	-7.5	438.471	1.6499	9	2	324.65	0.33377	136.43	-5.8488	2560.287713445	0.24746	3.9418	6.6674	0.45161	0.53187	0.97199 (max o	8	7422; 6876; 6869; 59	0.3933147	0.2424785		12.0
	ZINC04556806	-7.5	420.259	-10.197	14	7	262.3	0.67587	251.53	-31.289	2560.287713445	0.28412	15.789	-35.89	0.48148	0.52563	0.95201 (max o	4	7943; 7696; 7322; 56	0.00645983	-0.4952552		12.0
	ZINC14438556	-7.5	396.431	-1.2041	10	6	285.68	0.41515	158.3	-17.323	2560.287713445	0.28412	6.7958	-4.238	0.62963	0.54308	0.95493 (max o	3	6238; 4680; 5511	-0.4309767	0.65356886		11.0
	ZINC67912090	-7.5	424.485	-0.2953	10	6	313.2	0.37867	158.3	-25.333	2560.287713445	0.26452	5.887	-1.1164	0.65517	0.54153	0.93046	1	5613	-0.3834629	0.3637988		11.0
	ZINC20465497	-7.5	410.489	-3.2365	9	4	323.65	0.31466	104.93	3.6337	2560.287713445	0.26452	8.8282	-12.235	0.55172	0.53531	0.94862 (max o	8	7730; 7053; 7050; 68	-0.3762959	-0.5750047		10.0
	ZINC20464770	-7.5	350.437	-2.6677	7	4	277.63	0.29478	86.47	3.8868	2560.287713445	0.30685	8.2594	-8.6939	0.56	0.51871	0.94326 (max o	6	7053; 6826; 6815; 65	-0.3350905	-0.6183929		10.0
	ZINC15672341	-7.5	424.521	-2.3524	8	1	301.57	0.39785	160.45	6.4882	2560.287713445	0.27397	7.9441	-8.5863	0.64286	0.55701	0.96519	1	4973	-0.4484084	-0.465286		10.0
	ZINC31158772	-7.5	386.439	-0.2961	8	5	283.18	0.34798	136.68	-4.6035	2560.287713445	0.28412	5.8878	-1.0422	0.48148	0.53353	0.99202 (max o	5	3927; 4800; 5231; 56	-0.0904925	0.9330356		10.0
	ZINC31158765	-7.5	386.439	-0.2961	8	5	283.18	0.34798	136.68	-4.6035	2560.287713445	0.28412	5.8878	-1.0422	0.48148	0.53809	0.99202 (max o	5	3927; 4800; 5231; 56	-0.0470067	0.92564267		10.0
	ZINC12603449	-7.5	384.427	-0.9672	9	4	298.67	0.36241	134.19	-5.6862	2560.287713445	0.28412	6.5589	-3.4042	0.66667	0.67801	0.9036	1	4264	-0.8583457	0.17365123		10.0
	ZINC15259616	-7.5	432.380	-0.7865	10	7	289.19	0.43134	177.14	-2.3989	2560.287713445	0.24746	6.3782	-3.1783	0.48387	0.39235	0.97423 (max o	7	161; 462; 758; 790; 1	0.63451105	0.18317749		9.0
	ZINC05397322	-7.5	445.518	-0.4183	10	2	339.18	0.28224	111.29	5.6002	2560.287713445	0.23972	6.01	-1.7449	0.46875	0.55591	0.9665 (max of	4	2835; 3263; 4181; 49	0.11252805	0.31894884		9.0
	ZINC31169449	-7.5	360.358	-1.2596	9	6	256.4	0.42356	149.07	-4.4264	2560.287713445	0.30685	6.8513	-4.105	0.52	0.50865	0.98671 (max o	10	6887; 6337; 6301; 38	0.15101095	0.7393605		9.0
	ZINC12655155	-7.5	443.383	-5.0358	10	1	333	0.38468	176.15	-6.2561	2560.287713445	0.23972	10.628	-21.007	0.625	0.62914	0.97743 (max o	8	7170; 7169; 7168; 62	-0.6371214	0.57557195		9.0
	ZINC12655155	-7.5	443.383	-5.0358	10	1	333	0.38468	176.15	-7.6209	2560.287713445	0.23972	10.628	-21.007	0.625	0.62641	0.97743 (max o	8	7170; 7169; 7168; 62	-0.6002101	0.6228467		9.0
	ZINC12655155	-7.5	442.375	-7.1118	10	0	334.18	0.38686	178.98	-6.6469	2560.287713445	0.23972	12.704	-29.667	0.625	0.63082	0.97743 (max o	8	7170; 7169; 7168; 62	-0.5979509	0.5939922		9.0
	ZINC19331499	-7.5	427.519	-2.0628	7	6	311.03	0.27856	108.83	2.5847	2560.287713445	0.24746	7.6545	-8.336	0.41935	0.48029	0.97423 (max o	5	1345; 1590; 3827; 39	0.8610065	0.13542812		8.0
	ZINC12658812	-7.5	367.385	-4.2278	11	5	269.44	0.46129	136.88	2.9437	2560.287713445	0.29504	9.8195	-14.329	0.46154	0.41265	0.94951 (max o	5	6492; 4191; 4237; 49	-0.6294265	-0.5394246		8.0
	ZINC48056987	-7.5	382.273	-8.8812	13	5	246.07	0.65079	218.33	-2.9931	2560.287713445	0.29504	14.473	-30.101	0.46154	0.43576	0.94951 (max o	3	6663; 3950; 3951	0.18180032	0.91376096		8.0
	ZINC71404532	-7.5	409.386	-3.6382	12	7	280.11	0.51858	202.32	-14.737	2560.287713445	0.27397	9.2299	-13.28	0.42857	0.47924	0.94862 (max o	4	7275; 6732; 3545; 39	0.5646448	-0.4996359		8.0
	ZINC31169259	-7.5	412.390	-0.0778	10	4	287.63	0.40135	144.14	-3.1079	2560.287713445	0.26452	5.6695	-0.29411	0.41379	0.42494	0.90474	1	3049	0.7704765	-0.5633045		8.0
	ZINC35455839	-7.5	352.425	1.0294	6	4	262.95	0.2869	107.22	4.8399	2560.287713445	0.30685	4.5623	3.3548	0.56	0.45151	0.97118 (max o	6	6880; 3897; 3898; 47	-0.7268464	-0.1924416		8.0
	ZINC03838499	-7.5	406.410	-2.0096	9	3	303.71	0.36568	145.22	3.1725	2560.287713445	0.26452	7.6013	-7.5971	0.51724	0.51791	0.90934 (max o	2	6397; 5113	0.8001138	-0.1581381		8.0
	ZINC70666364	-7.5	359.357	-0.8644	8	2	260.62	0.37929	120.55	0.018054	2560.287713445	0.29504	6.4561	-2.9297	0.42308	0.30714	0.93033 (max o	4	7274; 7110; 4440; 48	0.45052406	0.37293094		8.0
	ZINC02139974	-7.5	377.415	-0.9394	8	2	287.38	0.31843	119	-1.1305	2560.287713445	0.28412	6.5311	-3.3064	0.59259	0.57627	0.95493 (max o	3	5310; 5311; 5704	-0.8308592	0.44359863		8.0
	ZINC02139970	-7.5	377.415	-0.9394	8	2	287.38	0.31843	119	-1.1305	2560.287713445	0.28412	6.5311	-3.3064	0.59259	0.57609	0.95493 (max o	3	5705; 5310; 5311	-0.8016588	0.45470494		8.0
	ZINC35456627	-7.5	374.384	-1.0158	9	7	261.66	0.42689	160.07	-3.6927	2560.287713445	0.29504	6.6075	-3.4429	0.46154	0.44101	0.9464 (max of	3	6330; 5635; 5636	-0.2244359	0.88194317		8.0
	ZINC03830748	-7.5	376.451	-0.8691	7	1	293.16	0.25478	103.35	1.9037	2560.287713445	0.28412	6.4608	-3.059	0.51852	0.50029	0.95201 (max o	2	6695; 6085	-0.8354019	0.03150849		8.0
	ZINC15676492	-7.5	405.837	-1.527	8	1	286.57	0.2576	105.81	0.9468	2560.287713445	0.27397	7.1187	-5.5736	0.53571	0.51745	0.95675 (max o	5	7016; 6789; 6769; 58	-0.3010819	0.36769333		8.0

Structure	Molecule Name	affinity	Total Molweight	cLogP	H-Acceptors	H-Donors	Total Surface Area	Relative PSA	Polar Surface Area	Druglikeness	Kd (nM) @ 20°C	LE from K...	LLE from Kd ...	LELP from Kd (n...	Shape Index	Molecular...	Neighbor Si...	Neig...	Neighbor	Neighbor ...	Neighbor ...	Ring Systems	frequency
	ZINC34361065	-7.5	356.370	0.1552	8	1	277.9	0.33185	108.36	-3.5573	2560.287713445	0.30685	5.4365	0.50579	0.52	0.56618	0.96243 (max o	6	7360; 7012; 6786; 66	-0.0289324	-0.7397826		8.0
	ZINC34361063	-7.5	356.370	0.1552	8	1	277.9	0.33185	108.36	-3.5573	2560.287713445	0.30685	5.4365	0.50579	0.52	0.57411	0.96243 (max o	7	7360; 7012; 6786; 67	-0.0255769	-0.7198126		8.0
	ZINC02139623	-7.5	367.400	-0.2638	6	2	277.39	0.25668	103.27	-1.2836	2560.287713445	0.28412	5.8555	-0.92849	0.51852	0.52187	0.96567 (max o	7	7287; 7119; 6379; 60	-0.0974860	0.68517554		7.0
	ZINC04200887	-7.5	391.430	-2.1382	11	3	295.88	0.40351	150.62	9.5005	2560.287713445	0.27397	7.7299	-7.8045	0.46429	0.51969	0.92774 (max o	5	7461; 7317; 5766; 42	-0.0883682	-0.0369430		7.0
	ZINC04200888	-7.5	389.415	-2.2286	11	3	297.16	0.40177	150.62	6.1322	2560.287713445	0.27397	7.8203	-8.1345	0.5	0.54014	0.92774 (max o	4	7318; 7294; 5723; 57	-0.0507797	-0.0151559		7.0
	ZINC20465131	-7.5	380.463	-2.7891	8	4	299.89	0.30625	95.7	3.6854	2560.287713445	0.28412	8.3808	-9.8167	0.59259	0.53407	0.91351 (max o	8	7521; 6819; 5935; 18	-0.3586007	0.00500958		6.0
	ZINC20465134	-7.5	393.506	-2.8227	8	4	312.45	0.27329	89.71	4.3417	2560.287713445	0.27397	8.4144	-10.303	0.57143	0.54969	0.91204 (max o	7	7222; 6819; 2591; 47	-0.3374171	0.03760675		6.0
	ZINC49180891	-7.5	418.481	-0.2872	9	6	293.11	0.38088	156.91	-5.2779	2560.287713445	0.26452	5.8789	-1.0857	0.37931	0.4602	0.95908 (max o	3	7747; 7565; 4427	-0.5767367	-0.6747262		6.0
	ZINC12483882	-7.5	442.534	-0.4493	8	3	320.88	0.22139	91.24	4.6027	2560.287713445	0.23972	6.041	-1.8742	0.53125	0.4526	0.93952 (max o	8	6559; 6255; 3840; 46	0.37127408	-0.0680392		6.0
	ZINC12413147	-7.5	382.443	-0.0447	8	2	276.03	0.22135	84.33	1.5892	2560.287713445	0.27397	5.6364	-0.16316	0.42857	0.35142	0.85377	1	4204	0.5333908	0.62663066		6.0
	ZINC14616880	-7.5	422.469	-0.5759	10	6	308.96	0.38387	158.3	-18.912	2560.287713445	0.26452	6.1676	-2.1771	0.58621	0.5381	0.97415 (max o	5	4259; 4680; 4684; 55	-0.4150052	0.6408656		6.0
	ZINC06379175	-7.5	388.368	-1.8524	10	4	278.22	0.42585	151.98	-10.162	2560.287713445	0.28412	7.4441	-6.5199	0.48148	0.46782	0.9578 (max of	3	7490; 6467; 6468	0.9136038	-0.1123372		6.0
	ZINC34034965	-7.5	388.368	-1.8524	10	4	278.22	0.42585	151.98	-10.162	2560.287713445	0.28412	7.4441	-6.5199	0.48148	0.48454	0.93686	1	7315	-0.4890027	0.84786606		6.0
	ZINC31159654	-7.5	446.403	-2.5173	12	5	304.21	0.4654	181.44	-4.4513	2560.287713445	0.24746	8.109	-10.173	0.41935	0.40529	0.91413	1	5599	-0.6927247	0.6887587		6.0
	ZINC03882101	-7.5	388.368	-2.1498	10	5	265.46	0.44655	155.14	-4.9044	2560.287713445	0.28412	7.7415	-7.5666	0.48148	0.39408	0.94905	1	6707	-0.1646305	0.9688965		6.0
	ZINC15676492	-7.5	404.829	-1.3381	8	0	297.54	0.28497	104.56	1.5012	2560.287713445	0.27397	6.9298	-4.8841	0.53571	0.51745	0.95675 (max o	5	7195; 7017; 6790; 67	-0.2855855	0.33665425		6.0
	ZINC08552016	-7.5	424.401	-1.9238	10	3	247.15	0.47914	148.82	-1.779	2560.287713445	0.25571	7.5155	-7.5235	0.33333	0.093597	0.91374 (max o	4	534; 726; 1761; 2869	-0.6596876	-0.4185800		5.0
	ZINC03838654	-7.5	364.492	-4.5162	8	2	305.83	0.29618	69.13	0.25249	2560.287713445	0.29504	10.108	-15.307	0.61538	0.53856	0.96721 (max o	3	3723; 4483; 5446	0.2135671	0.311829		5.0
	ZINC20465131	-7.5	379.455	-0.6794	8	3	285.85	0.27217	94.5	7.6916	2560.287713445	0.28412	6.2711	-2.3913	0.59259	0.53407	0.92064 (max o	6	2235; 2590; 5934; 47	-0.3932658	0.03234107		5.0
	ZINC20465134	-7.5	392.498	-0.713	8	3	298.41	0.2391	88.51	7.9004	2560.287713445	0.27397	6.3047	-2.6025	0.57143	0.54969	0.9083 (max of	6	7222; 2590; 5935; 47	-0.3612454	0.06215079		5.0
	ZINC20504117	-7.5	413.948	-1.0477	7	5	308.15	0.35291	123.69	4.6699	2560.287713445	0.28412	6.6394	-3.6876	0.59259	0.41629	0.97658 (max o	6	6273; 772; 3010; 516	0.46365985	0.22568378		5.0
	ZINC04534216	-7.5	408.631	0.6952	7	5	239.64	0.3605	115.17	-5.48	2560.287713445	0.33353	4.8965	2.0844	0.47826	0.23946	0.92947 (max o	5	6081; 3613; 5291; 54	-0.4477873	0.6138469		5.0
	ZINC32124085	-7.5	417.441	-0.9642	10	3	304.95	0.32176	124.1	-0.11909	2560.287713445	0.25571	6.5559	-3.7707	0.46667	0.55362	0.95837 (max o	7	7424; 6308; 5172; 56	-0.6586721	0.16324168		5.0
	ZINC32123825	-7.5	417.441	-0.9642	10	3	304.95	0.32176	124.1	-0.11909	2560.287713445	0.25571	6.5559	-3.7707	0.5	0.55362	0.95837 (max o	7	7424; 6308; 5989; 59	-0.6483910	0.14573619		5.0
	ZINC30879650	-7.5	389.445	-0.5352	6	4	305.66	0.25613	83.23	0.60578	2560.287713445	0.27397	6.1269	-1.9535	0.53571	0.53724	0.9398 (max of	3	7411; 3005; 4787	0.41135094	-0.2799224		5.0
	ZINC04097839	-7.5	394.375	-0.4815	9	5	272.76	0.40908	153.75	-2.8773	2560.287713445	0.27397	6.0732	-1.7575	0.42857	0.44471	0.9428 (max of	7	5809; 2479; 4811; 49	-0.7737848	0.43818772		5.0
	ZINC35456324	-7.5	360.314	-1.1781	10	5	250.04	0.47408	155.14	-4.135	2560.287713445	0.30685	6.7698	-3.8394	0.52	0.40974	0.94975 (max o	7	6347; 6014; 4802; 51	-0.7126503	-0.1108458		5.0
	ZINC15251357	-7.5	374.341	-0.9024	10	4	265.95	0.43407	144.14	-4.135	2560.287713445	0.29504	6.4941	-3.0585	0.5	0.40946	0.94975 (max o	5	6788; 6347; 6014; 51	-0.7153027	-0.1536282		5.0
	ZINC36374591	-7.5	374.341	-0.9024	10	4	265.95	0.43407	144.14	-4.135	2560.287713445	0.29504	6.4941	-3.0585	0.5	0.40946	0.94975 (max o	4	6788; 6347; 5882; 60	-0.7321739	-0.1423879		5.0
	ZINC32124086	-7.5	417.441	-0.9642	10	3	304.95	0.32176	124.1	-0.11909	2560.287713445	0.25571	6.5559	-3.7707	0.46667	0.55244	0.95837 (max o	7	7424; 6308; 4766; 56	-0.6173006	0.13427128		5.0
	ZINC03873054	-7.5	375.421	-4.4053	9	4	267.35	0.49766	181.16	-2.7923	2560.287713445	0.30685	9.997	-14.357	0.44	0.65461	0.92315 (max o	4	7678; 7188; 6102; 53	-0.8686953	0.00699515		5.0
	ZINC04260475	-7.5	406.482	-0.4113	8	0	298.97	0.32595	129.3	1.9423	2560.287713445	0.27397	6.003	-1.5013	0.5	0.51105	0.97837 (max o	4	7477; 6963; 6145; 57	-0.1519132	-0.8027973		5.0
	ZINC04260474	-7.5	392.455	-0.8657	8	0	285.21	0.34168	129.3	6.2987	2560.287713445	0.28412	6.4574	-3.047	0.48148	0.509	0.97837 (max o	4	7477; 6963; 6145; 57	-0.1169922	-0.8092419		5.0
	ZINC05398475	-7.5	385.463	-0.0353	10	3	300.82	0.34452	120.08	2.3603	2560.287713445	0.28412	5.627	-0.12424	0.48148	0.65081	0.90033 (max o	5	7328; 6660; 5011; 56	-0.1129787	0.31721953		5.0
	ZINC08636493	-7.5	395.454	0.1781	7	3	275.63	0.34757	117.36	2.8295	2560.287713445	0.28412	5.4136	0.62685	0.48148	0.39785	0.97658 (max o	7	7164; 6479; 6184; 61	0.45220754	0.08945451		5.0
	ZINC08636500	-7.5	405.517	0.7651	7	3	293.8	0.32607	117.36	4.1585	2560.287713445	0.27397	4.8266	2.7926	0.42857	0.40252	0.91333 (max o	6	7335; 6479; 6184; 51	0.4369194	0.05588295		5.0
	ZINC12655678	-7.5	384.336	-0.9694	10	0	269.14	0.43933	139.34	-6.1644	2560.287713445	0.28412	6.5611	-3.412	0.40741	0.58847	0.95201 (max o	2	7864; 7680	-0.303788	-0.8894943		5.0
	ZINC67903052	-7.5	367.460	-0.4817	6	4	276.3	0.28853	121.05	-4.8501	2560.287713445	0.29504	6.0734	-1.6326	0.46154	0.57414	0.96438 (max o	3	6670; 6671; 176	0.54287064	0.7927605		4.0
	ZINC12481665	-7.5	421.511	-1.1766	7	1	339.2	0.22126	77.35	2.8582	2560.287713445	0.25571	6.7683	-4.6014	0.6	0.59522	0.94529 (max o	3	3323; 4717; 4724	0.6743306	0.42339674		4.0
	ZINC49600283	-7.5	422.469	-0.6945	10	6	308.13	0.3849	158.3	-13.648	2560.287713445	0.26452	6.2862	-2.6255	0.58621	0.50855	0.97415	1	5257	0.8837278	0.39630252		4.0
	ZINC08927238	-7.5	384.364	-4.7824	10	1	268.54	0.50741	181.27	4.9262	2560.287713445	0.29504	10.374	-16.209	0.61538	0.49233	0.93686 (max o	4	7459; 6204; 6203; 54	0.46260118	0.5132947		4.0
	ZINC05398282	-7.5	397.451	-0.2122	9	1	286.71	0.33574	121.47	-4.7636	2560.287713445	0.28412	5.8039	-0.74688	0.51852	0.55356	0.93686 (max o	3	3684; 5418; 5801	0.13886036	0.3948323		4.0
	ZINC04217565	-7.5	435.393	-6.281	14	2	285.79	0.66265	253.09	6.428	2560.287713445	0.27397	11.873	-22.926	0.5	0.5101		0		0.7180121	0.6438176		4.0

Structure	Molecule Name	affinity	Total Molweight	cLogP	H-Acceptors	H-Donors	Total Surface Area	Relative PSA	Polar Surface Area	Druglikeness	Kd (nM) @ 20°C	LE from K...	LLE from Kd ...	LELP from Kd (n...	Shape Index	Molecular...	Neighbor Si...	Neig...	Neighbor	Neighbor ...	Neighbor ...	Ring Systems	frequency
	ZINC31156074	-7.5	387.403	-2.5561	9	4	285.25	0.3953	156.58	-5.0739	2560.287713445	0.28412	8.1478	-8.9966	0.59259	0.63438	0.97149 (max o	7	3447; 3888; 3889; 44	-0.9011074	0.03314193		4.0
	ZINC08740170	-7.5	380.404	-1.2191	11	5	264.33	0.46143	158.46	4.3394	2560.287713445	0.28412	6.8108	-4.2908	0.44444	0.41043	0.94314 (max o	3	2530; 4001; 5485	-0.5054096	-0.5585896		4.0
	ZINC08918277	-7.5	442.555	-1.0277	9	1	331.61	0.33922	144.38	-30.482	2560.287713445	0.25571	6.6194	-4.0191	0.56667	0.56414	0.93686 (max o	3	4074; 4075; 5361	0.49933583	-0.0245221		4.0
	ZINC05415602	-7.5	404.426	-1.8779	11	2	301.88	0.37803	133.83	4.9815	2560.287713445	0.26452	7.4696	-7.0992	0.44828	0.52776	0.97415 (max o	4	1740; 4127; 4129; 45	0.05927689	0.24408832		4.0
	ZINC04995790	-7.5	446.503	-2.5422	8	2	329.5	0.4207	189.06	2.8383	2560.287713445	0.25571	8.1339	-9.9419	0.6	0.67268	0.97533 (max o	3	4233; 4562; 4563	0.14986381	-0.7071559		4.0
	ZINC31158099	-7.5	388.324	-6.5696	11	4	269.23	0.50901	188.87	-5.5881	2560.287713445	0.28412	12.161	-23.123	0.44444	0.49851	0.95201 (max o	3	6612; 4367; 5602	0.7718746	-0.2146142		4.0
	ZINC00631634	-7.5	362.432	0.7495	9	2	272.39	0.33331	113.56	7.3025	2560.287713445	0.29504	4.8422	2.5403	0.46154	0.46776	0.92342	1	6069	-3.4720835	-0.8017781		4.0
	ZINC04043932	-7.5	392.450	-1.0059	8	2	301.56	0.30707	121.37	-1.6404	2560.287713445	0.27397	6.5976	-3.6716	0.5	0.46542	0.96519 (max o	3	4513; 4957; 5360	0.08485174	0.9237543		4.0
	ZINC05224204	-7.5	382.400	-4.6527	12	6	276.17	0.50534	162.9	2.8692	2560.287713445	0.28412	10.244	-16.376	0.44444	0.40925	0.93373 (max o	3	6158; 3250; 4987	-0.6384227	-0.4822020		4.0
	ZINC15707497	-7.5	359.469	-2.4496	8	3	268.24	0.36803	108.4	7.2377	2560.287713445	0.31963	8.0413	-7.6638	0.54167	0.47067	0.89723 (max o	4	7362; 4692; 5061; 55	-0.214793	0.1277997		4.0
	ZINC31155119	-7.5	386.439	-0.5583	8	5	279.37	0.35272	136.68	-6.1261	2560.287713445	0.28412	6.15	-1.965	0.44444	0.51342	0.97407 (max o	7	6606; 1906; 3508; 35	0.22130895	0.8437674		4.0
	ZINC31168771	-7.5	412.433	0.3716	9	5	305.3	0.35552	145.91	-6.6975	2560.287713445	0.26452	5.2201	1.4048	0.62069	0.49384	0.98316 (max o	3	5213; 5214; 5612	-0.7012018	0.4554721		4.0
	ZINC35458998	-7.5	410.414	-2.1178	11	6	284.15	0.46328	175.37	-2.7727	2560.287713445	0.27397	7.7095	-7.73	0.5	0.43791	0.9678 (max of	3	6884; 6334; 5237	0.6959812	-0.4494642		4.0
	ZINC67913283	-7.5	410.417	0.3539	9	3	294.52	0.37845	147.43	-9.4267	2560.287713445	0.26452	5.2378	1.3379	0.41379	0.5305	0.95908 (max o	5	7900; 7105; 7084; 43	0.43842667	0.31804797		4.0
	ZINC03838489	-7.5	410.489	0.2467	8	4	306.08	0.38748	153.2	3.3672	2560.287713445	0.27397	5.345	0.90046	0.53571	0.56244	0.88286 (max o	5	6132; 6093; 4914; 50	-0.5132149	-0.2443120		4.0
	ZINC13407164	-7.5	419.448	-1.2708	8	2	284.75	0.36046	141.03	-0.21816	2560.287713445	0.25571	6.8625	-4.9698	0.4	0.42818	0.95579 (max o	3	6435; 5395; 5501	0.8099759	0.39501056		4.0
	ZINC20463264	-7.5	382.503	-1.7225	8	2	315.56	0.28378	97.22	5.7828	2560.287713445	0.28412	7.3142	-6.0626	0.55556	0.64904	0.93686 (max o	3	6499; 4642; 5911	-0.0926218	-0.3001428		4.0
	ZINC20463226	-7.5	395.542	-0.5242	7	1	328.03	0.23806	85.19	1.5998	2560.287713445	0.27397	6.1159	-1.9133	0.53571	0.65786	0.9398 (max of	4	6499; 5917; 5525; 59	-0.0712370	-0.2915093		4.0
	ZINC35271770	-7.5	438.471	1.6499	9	2	324.65	0.33377	136.43	-5.8488	2560.287713445	0.24746	3.9418	6.6674	0.45161	0.53187	0.96971 (max o	7	6876; 6869; 5216; 56	0.36841172	0.22289501		4.0
	ZINC35271767	-7.5	438.471	1.6499	9	2	324.65	0.33377	136.43	-5.8488	2560.287713445	0.24746	3.9418	6.6674	0.45161	0.53187	0.96971 (max o	7	6876; 6869; 5216; 59	0.3810934	0.20414445		4.0
	ZINC35780712	-7.5	406.382	-2.7247	11	6	268.21	0.49081	175.37	-4.1099	2560.287713445	0.27397	8.3164	-9.9453	0.42857	0.37997	0.95148 (max o	3	6346; 6342; 5645	0.3515586	-0.7079243		4.0
	ZINC04293782	-7.5	410.338	-0.3298	13	2	285.6	0.51106	164.15	4.6806	2560.287713445	0.26452	5.9215	-1.2468	0.41379	0.43252	0.93975 (max o	3	7146; 6441; 6151	-0.9077542	0.01354563		4.0
	ZINC20464009	-7.5	359.425	-3.7348	7	2	287.6	0.30741	84.02	1.0333	2560.287713445	0.29504	9.3265	-12.658	0.57692	0.52107	0.89688 (max o	3	7391; 6811; 5926	0.38156068	0.06890888		4.0
	ZINC20464009	-7.5	360.432	-1.6588	7	3	286.42	0.30455	81.19	1.4597	2560.287713445	0.29504	7.2505	-5.6222	0.57692	0.52107	0.89688 (max o	4	7392; 6996; 6813; 59	0.3650534	0.05889325		4.0
	ZINC20464009	-7.5	359.425	-3.5932	7	1	269.84	0.26182	87.4	4.1937	2560.287713445	0.29504	9.1849	-12.179	0.57692	0.4825	0.92418 (max o	2	7390; 5925	0.34261423	-0.7596948		4.0
	ZINC20464009	-7.5	360.432	-1.5172	7	2	268.66	0.25858	84.57	4.5014	2560.287713445	0.29504	7.1089	-5.1423	0.57692	0.4825	0.92418 (max o	2	7393; 5923	0.35436293	-0.7761426		4.0
	ZINC20465634	-7.5	394.494	-2.6907	9	5	287.6	0.27559	111.44	6.6451	2560.287713445	0.27397	8.2824	-9.8212	0.57143	0.52027	0.9083 (max of	4	7052; 2233; 2236; 47	-0.4157006	0.06013179		4.0
	ZINC35456145	-7.5	355.362	-2.4289	8	4	260.48	0.38283	139.51	-4.6528	2560.287713445	0.30685	8.0206	-7.9157	0.52	0.5318	0.98434 (max o	7	7086; 6532; 6237; 62	0.6696221	-0.3165766		4.0
	ZINC35457085	-7.5	389.419	-1.9705	9	2	290.57	0.36673	134.58	-13.144	2560.287713445	0.28412	7.5622	-6.9355	0.55556	0.52531	0.94905 (max o	9	7435; 7262; 6882; 68	-0.7295871	-0.0566729		4.0
	ZINC36370069	-7.5	414.480	-4.6842	8	1	334.85	0.27669	102.1	-5.4264	2560.287713445	0.25571	10.276	-18.319	0.5	0.51797	0.94241 (max o	3	6893; 6655; 5247	0.33793625	-0.5677427		4.0
	ZINC35442267	-7.5	438.550	-0.7395	8	3	337.32	0.2409	100.17	4.2168	2560.287713445	0.23972	6.3312	-3.0848	0.65625	0.47366	0.94734 (max o	4	1594; 1595; 1596; 19	-0.4908659	0.00132799		3.0
	ZINC38205251	-7.5	352.341	-1.1086	6	0	277.52	0.26895	98.72	0.78259	2560.287713445	0.29504	6.7003	-3.7574	0.61538	0.49811	0.87452	1	2277	0.84379846	0.45545304		3.0
	ZINC04270795	-7.5	369.440	-0.7275	9	1	273.51	0.28339	96	2.1516	2560.287713445	0.29504	6.3192	-2.4657	0.53846	0.57687	0.91693 (max o	3	2465; 4974; 5397	-0.1736767	0.26005232		3.0
	ZINC05890334	-7.5	394.375	-0.4815	9	5	272.76	0.40908	153.75	-2.8773	2560.287713445	0.27397	6.0732	-1.7575	0.42857	0.44532	0.95148 (max o	6	2479; 4998; 5432; 54	-0.7660774	0.41942656		3.0
	ZINC12603680	-7.5	370.385	-1.285	10	3	258.01	0.47773	156.05	2.5468	2560.287713445	0.30685	6.8767	-4.1878	0.6	0.45372	0.91999 (max o	4	1771; 2504; 2921; 42	-0.2922229	-0.6250633		3.0
	ZINC15709581	-7.5	362.430	-6.1931	10	3	253.16	0.4432	151.91	4.9264	2560.287713445	0.31963	11.785	-19.376	0.45833	0.53422	0.88538 (max o	4	3258; 4573; 5274; 54	-0.0924969	0.36866668		3.0
	ZINC22923611	-7.5	420.556	-4.7161	9	4	324.14	0.266	96.25	6.4017	2560.287713445	0.25571	10.308	-18.444	0.56667	0.47464	0.90299 (max o	10	7085; 6848; 1561; 59	-0.1273596	0.4912542		3.0
	ZINC22923611	-7.5	419.548	-2.7383	9	3	335.11	0.29002	95	6.5701	2560.287713445	0.25571	8.33	-10.709	0.56667	0.48768	0.92257 (max o	10	7085; 6849; 1562; 29	-0.1084142	0.47835445		3.0
	ZINC49543417	-7.5	434.403	-3.0282	9	1	319.03	0.33229	132.75	3.7628	2560.287713445	0.23972	8.6199	-12.632	0.46875	0.40566	0.95219 (max o	4	2305; 3086; 3525; 48	0.5647307	-0.290537		3.0
	ZINC68590550	-7.5	402.394	-1.763	10	5	276.16	0.42924	155.14	-4.6523	2560.287713445	0.27397	7.3547	-6.435	0.46429	0.39394	0.98006 (max o	3	5266; 5267; 5762	0.7302952	0.66914856		3.0
	ZINC04098339	-7.5	404.367	-3.1441	11	6	270.25	0.4871	175.37	-5.0325	2560.287713445	0.27397	8.7358	-11.476	0.46429	0.3933	0.98006	1	6049	0.72487473	0.66326547		3.0
	ZINC04270687	-7.5	366.461	0.6184	7	1	267.37	0.31537	111.77	4.5709	2560.287713445	0.30685	4.9733	2.0153	0.52	0.49868	0.95318 (max o	2	6729; 3686	-0.1888378	0.27315265		3.0
	ZINC13552105	-7.5	380.356	-2.7109	10	2	263	0.51186	169.8	12.458	2560.287713445	0.29504	8.3026	-9.1881	0.5	0.40416	0.94291 (max o	3	3619; 4029; 4496	-0.3159434	0.84025794		3.0

Structure	Molecule Name	affinity	Total Molweight	cLogP	H-Acceptors	H-Donors	Total Surface Area	Relative PSA	Polar Surface Area	Druglikeness	Kd (nM) @ 20°C	LE from K...	LLE from Kd ...	LELP from Kd (n...	Shape Index	Molecular...	Neighbor Si...	Neig...	Neighbor	Neighbor ...	Neighbor ...	Ring Systems	frequency
	ZINC05434231	-7.5	432.475	-0.3847	10	2	326.62	0.31284	117.28	4.9815	2560.287713445	0.24746	5.9764	-1.5546	0.48387	0.5438	0.94241 (max o	5	1745; 2469; 4160; 45	0.11645065	0.23931256		3.0
	ZINC20463197	-7.5	409.569	-0.1822	7	1	341.79	0.22847	85.19	1.5998	2560.287713445	0.26452	5.7739	-0.68879	0.51724	0.65677	0.9398 (max of	8	5911; 1205; 4205; 42	-0.1030437	-0.2811760		3.0
	ZINC20113180	-7.5	395.366	-3.338	8	0	291.83	0.31261	119.03	-6.2595	2560.287713445	0.26452	8.9297	-12.619	0.44828	0.43972	0.91292 (max o	3	7027; 4282; 4327	0.04194312	0.85174084		3.0
	ZINC20465872	-7.5	422.524	-1.4662	8	4	322.66	0.3248	121.57	-5.0973	2560.287713445	0.26452	7.0579	-5.5428	0.55172	0.54695	0.96682 (max o	4	7398; 4321; 4758; 55	0.45780408	-0.3487207		3.0
	ZINC15708599	-7.5	395.526	-2.5476	9	4	316.19	0.31054	98.22	9.8925	2560.287713445	0.27397	8.1393	-9.2988	0.60714	0.53131	0.92442 (max o	2	3816; 4690	-0.1876336	0.0626292		3.0
	ZINC12481203	-7.5	389.470	-1.141	7	0	304.75	0.22136	82.14	4.0931	2560.287713445	0.27397	6.7327	-4.1647	0.57143	0.59789	0.93091 (max o	7	6764; 6214; 5910; 33	-0.1401105	-0.1162188		3.0
	ZINC20463226	-7.5	394.534	-0.6558	7	0	313.99	0.20399	83.99	6.0974	2560.287713445	0.27397	6.2475	-2.3937	0.53571	0.66217	0.93686 (max o	8	7345; 5918; 5916; 10	-0.2070905	-0.1794112		3.0
	ZINC20463264	-7.5	381.495	-1.8541	8	1	301.52	0.25043	96.02	10.127	2560.287713445	0.28412	7.4458	-6.5258	0.55556	0.65363	0.89169 (max o	5	3387; 5133; 5526; 59	-0.1945257	-0.1961701		3.0
	ZINC03830748	-7.5	375.443	-2.1005	7	1	296.88	0.26411	98.77	3.6019	2560.287713445	0.28412	7.6922	-7.3931	0.51852	0.61293	0.96343 (max o	2	6696; 6086	-0.5821642	0.60906273		3.0
	ZINC04222260	-7.5	381.472	0.1456	7	3	272.05	0.40195	150.82	0.34281	2560.287713445	0.29504	5.4461	0.49349	0.42308	0.3964	0.93686	1	6137	0.85885483	-0.0179980		3.0
	ZINC03842077	-7.5	411.589	0.1278	7	5	319.41	0.35969	141.15	0.43217	2560.287713445	0.27397	5.4639	0.46648	0.46429	0.48033		0		-0.2283137	0.9673177		3.0
	ZINC03984111	-7.5	365.389	-0.7942	10	4	257.6	0.41421	132.44	4.4154	2560.287713445	0.29504	6.3859	-2.6918	0.46154	0.41144	0.92702 (max o	3	3989; 5354; 5740	-0.5409446	-0.6159816		3.0
	ZINC05886092	-7.5	351.362	-1.0635	10	4	246.6	0.43268	132.44	1.2465	2560.287713445	0.30685	6.6552	-3.4659	0.44	0.41176	0.89688 (max o	5	4468; 4904; 5121; 54	-0.5705343	-0.5483965		3.0
	ZINC03984112	-7.5	365.389	-0.7942	10	4	257.6	0.41421	132.44	4.4154	2560.287713445	0.29504	6.3859	-2.6918	0.46154	0.41144	0.92702 (max o	4	5808; 3989; 5354; 57	-0.5601547	-0.5830383		3.0
	ZINC08551993	-7.5	425.996	-1.1969	7	5	318.59	0.34176	128.76	0.91283	2560.287713445	0.28412	6.7886	-4.2127	0.51852	0.46665	0.96343 (max o	10	7679; 7460; 7163; 69	0.7156598	-0.2286850		3.0
	ZINC04245609	-7.5	425.996	-1.1969	7	5	318.59	0.34176	128.76	0.91283	2560.287713445	0.28412	6.7886	-4.2127	0.51852	0.46166	0.92739 (max o	9	7679; 7464; 7298; 69	0.7278642	-0.2449825		3.0
	ZINC08643504	-7.5	353.417	-0.9814	8	5	275.47	0.35089	119.92	-2.7618	2560.287713445	0.30685	6.5731	-3.1983	0.68	0.58787	0.9302 (max of	4	6749; 6188; 3293; 42	0.15285978	-0.4002138		3.0
	ZINC13407266	-7.5	390.383	-1.9707	10	5	264.25	0.44859	155.14	-3.9213	2560.287713445	0.28412	7.5624	-6.9362	0.48148	0.37688	0.93058 (max o	2	5960; 2396	-0.5359286	-0.5091889		3.0
	ZINC28538317	-7.5	390.383	-1.9707	10	5	264.25	0.44859	155.14	-3.9213	2560.287713445	0.28412	7.5624	-6.9362	0.48148	0.37688	0.93058 (max o	2	3030; 5872	-0.5306912	-0.4882615		3.0
	ZINC31156298	-7.5	390.383	-1.942	10	5	263.42	0.45	155.14	-4.365	2560.287713445	0.28412	7.5337	-6.8352	0.48148	0.37688	0.93686 (max o	2	6290; 2626	-0.6490508	0.00620709		3.0
	ZINC15672914	-7.5	352.477	0.5088	7	1	277	0.21007	65.12	5.3754	2560.287713445	0.30685	5.0829	1.6582	0.64	0.59415	0.95631 (max o	3	7194; 6240; 3372	-0.7003370	-0.3439510		3.0
	ZINC20465634	-7.5	394.494	-2.8226	9	5	312.61	0.33355	111.39	4.651	2560.287713445	0.27397	8.4143	-10.303	0.57143	0.54386	0.9083 (max of	3	7053; 2232; 4756	-0.3788890	-0.6115384		3.0
	ZINC20611665	-7.5	365.340	-3.268	7	0	269.57	0.30133	109.8	-6.2595	2560.287713445	0.28412	8.8597	-11.502	0.48148	0.47449	0.91768 (max o	3	7027; 6247; 4327	0.06699373	0.88219905		3.0
	ZINC35442531	-7.5	395.550	-1.6075	8	2	315.98	0.33081	111.11	2.6137	2560.287713445	0.28412	7.1992	-5.6579	0.55556	0.52497	0.92774 (max o	11	7405; 7085; 7068; 68	-0.0512379	0.48920777		3.0
	ZINC20463236	-7.5	445.558	-0.8452	8	1	362.86	0.24277	94.42	4.7493	2560.287713445	0.23972	6.4369	-3.5257	0.59375	0.62183	0.93952 (max o	7	7377; 6992; 1000; 33	-0.0290295	-0.1851812		2.0
	ZINC20463236	-7.5	444.550	-0.9768	8	0	348.82	0.21229	93.22	9.3782	2560.287713445	0.23972	6.5685	-4.0747	0.59375	0.62497	0.93952 (max o	4	1001; 1860; 3385; 42	-0.1202979	-0.1729142		2.0
	ZINC04089179	-7.5	421.560	-1.1847	7	3	327.26	0.3309	151.27	2.0218	2560.287713445	0.26452	6.7764	-4.4786	0.65517	0.51536	0.90502 (max o	2	6991; 2790	0.53598404	-0.0811961		2.0
	ZINC03838477	-7.5	392.411	-1.5658	10	4	293.9	0.40891	150.74	1.5762	2560.287713445	0.27397	7.1575	-5.7152	0.53571	0.45562	0.89582	1	3167	-0.4346261	-0.1519691		2.0
	ZINC08643574	-7.5	420.488	-2.9951	10	4	335.81	0.34871	123.17	-2.2757	2560.287713445	0.25571	8.5868	-11.713	0.66667	0.56618	0.90983 (max o	2	3737; 5450	0.23431021	-0.7506555		2.0
	ZINC04259304	-7.5	378.408	0.5206	9	0	270.28	0.40813	122.89	-1.3653	2560.287713445	0.29504	5.0711	1.7645	0.61538	0.3433	0.93686 (max o	4	4545; 4546; 5394; 57	-0.5234207	0.48058388		2.0
	ZINC04259410	-7.5	370.429	0.4622	9	0	264.56	0.41696	122.89	-2.6787	2560.287713445	0.30685	5.1295	1.5063	0.6	0.29362	0.93341 (max o	3	4546; 4547; 5780	-0.5316299	0.46158814		2.0
	ZINC35458295	-7.5	445.483	-2.0251	10	4	327.72	0.37459	165.81	-2.0398	2560.287713445	0.24746	7.6168	-8.1837	0.51613	0.62475	0.96739 (max o	5	2638; 3072; 3504; 48	0.16710392	0.34719026		2.0
	ZINC05414149	-7.5	383.467	-3.1354	9	6	286.1	0.37553	144.37	2.3688	2560.287713445	0.28412	8.7271	-11.036	0.51852	0.48462	0.93069 (max o	3	6659; 6399; 4927	-0.6202962	-0.2040966		2.0
	ZINC03798720	-7.5	429.511	0.1874	7	1	362.23	0.2428	89.33	-3.757	2560.287713445	0.24746	5.4043	0.7573	0.58065	0.54194	0.98065	1	4940	0.8171493	-0.1446431		2.0
	ZINC03984761	-7.5	372.551	2.3527	4	2	253.34	0.10294	62.08	-2.6151	2560.287713445	0.28412	3.239	8.2807	0.51852	0.43143	0.92394 (max o	3	4946; 4947; 4948	0.9470401	0.11152868		2.0
	ZINC08740473	-7.5	391.411	1.6778	11	5	273.09	0.54077	192.17	3.1748	2560.287713445	0.28412	3.9139	5.9053	0.48148	0.29519	0.96885 (max o	5	6483; 6482; 5834; 50	-0.2810101	0.25884715		2.0
	ZINC08740523	-7.5	375.344	0.9999	12	5	266.84	0.53002	177.07	1.5916	2560.287713445	0.28412	4.5918	3.5193	0.48148	0.31128	0.96885 (max o	5	7338; 6193; 5027; 50	-0.3207426	0.2677215		2.0
	ZINC30879461	-7.5	403.565	0.3298	6	1	344.48	0.21038	77.77	1.1962	2560.287713445	0.26452	5.2619	1.2468	0.55172	0.65685	0.89657 (max o	2	4639; 5131	-0.3531328	-0.1781853		2.0
	ZINC31168500	-7.5	374.472	0.91	7	5	276.92	0.30875	119.61	-10.488	2560.287713445	0.29504	4.6817	3.0843	0.53846	0.49076	0.99159 (max o	7	6620; 6299; 3526; 48	0.741499	0.38105008		2.0
	ZINC72320351	-7.5	361.413	-0.5052	6	1	266.74	0.2868	103.73	-2.1259	2560.287713445	0.29504	6.0969	-1.7123	0.46154	0.51711	0.95257 (max o	3	3114; 5282; 5283	0.93182147	0.1726243		2.0
	ZINC04221685	-7.5	414.421	-1.5655	11	3	310.51	0.41851	160.52	4.6932	2560.287713445	0.25571	7.1572	-6.1223	0.46667	0.42585	0.92257 (max o	2	6136; 5389	0.72458655	0.39324585		2.0
	ZINC05437700	-7.5	371.368	-2.307	8	0	270.81	0.31243	103.12	1.4158	2560.287713445	0.28412	7.8987	-8.1199	0.51852	0.46257	0.94927	1	5427	0.0752092	-0.8663987		2.0
	ZINC12483876	-7.5	432.583	0.2287	7	2	322.57	0.17962	71.01	-0.32602	2560.287713445	0.24746	5.363	0.9242	0.54839	0.46871	0.91964 (max o	4	6255; 4727; 5135; 54	0.37342155	0.00588499		2.0

Structure	Molecule Name	affinity	Total Molweight	cLogP	H-Acceptors	H-Donors	Total Surface Area	Relative PSA	Polar Surface Area	Druglikeness	Kd (nM) @ 20°C	LE from K...	LLE from Kd ...	LELP from Kd (n...	Shape Index	Molecular...	Neighbor Si...	Neig...	Neighbor	Neighbor ...	Neighbor ...	Ring Systems	frequency
	ZINC31160891	-7.5	371.412	-1.5001	7	2	288.87	0.28217	109.77	6.0291	2560.287713445	0.28412	7.0918	-5.2799	0.51852	0.60529	0.94905	1	5607	0.26947877	0.8833399		2.0
	ZINC71758440	-7.5	350.394	-5.2192	7	2	254.67	0.42443	146.69	5.329	2560.287713445	0.31963	10.811	-16.329	0.54167	0.5751	0.93334 (max o	3	5665; 5679; 6056	0.01355459	-0.7237698		2.0
	ZINC71758442	-7.5	350.394	-5.2192	7	2	254.67	0.42443	146.69	5.329	2560.287713445	0.31963	10.811	-16.329	0.54167	0.5751	0.93334 (max o	3	5665; 5679; 6055	0.05296648	-0.7249754		2.0
	ZINC05398287	-7.5	376.412	0.4132	9	2	287.34	0.3208	108.05	1.7983	2560.287713445	0.28412	5.1785	1.4543	0.55556	0.56853	0.93686 (max o	3	6454; 6163; 5800	0.10321332	0.37441054		2.0
	ZINC05398468	-7.5	413.473	-0.5654	11	2	314.18	0.33653	120.52	1.5725	2560.287713445	0.26452	6.1571	-2.1374	0.44828	0.63368	0.87803 (max o	4	7328; 2465; 5803; 56	-0.1321477	0.3269754		2.0
	ZINC06385550	-7.5	375.373	-2.4613	10	5	269.21	0.44226	161.86	-7.4413	2560.287713445	0.29504	8.053	-8.3421	0.46154	0.4176	0.95559 (max o	3	7097; 6979; 6894	-0.8172219	-0.1751087		2.0
	ZINC12494166	-7.5	378.309	-5.9441	12	6	253.47	0.58121	198.07	0.5748	2560.287713445	0.29504	11.536	-20.146	0.42308	0.45401	0.95257 (max o	3	6509; 6508; 5854	0.17619659	-0.9501928		2.0
	ZINC12494166	-7.5	378.309	-5.7714	12	6	253.58	0.58139	201.56	1.738	2560.287713445	0.29504	11.363	-19.561	0.42308	0.43397	0.95257 (max o	3	6509; 6508; 5855	0.14662883	-0.9572907		2.0
	ZINC20113039	-7.5	361.353	-3.0669	9	2	265.37	0.38799	104.6	3.003	2560.287713445	0.29504	8.6586	-10.395	0.42308	0.34623	0.90377 (max o	2	6547; 5122	-0.3453531	0.52661544		2.0
	ZINC20462823	-7.5	365.448	-3.9841	7	1	291.07	0.30515	105.01	5.6861	2560.287713445	0.29504	9.5758	-13.503	0.53846	0.59309	0.91007 (max o	3	5124; 5125; 5908	-0.2091424	-0.2878923		2.0
	ZINC20463132	-7.5	365.472	-2.3059	7	2	292.06	0.30751	107.97	6.6209	2560.287713445	0.29504	7.8976	-7.8154	0.53846	0.57391	0.92702 (max o	6	6552; 5915; 4640; 47	-0.1717476	-0.3004340		2.0
	ZINC20464009	-7.5	361.440	-2.8835	7	3	282.7	0.2954	85.77	0.20333	2560.287713445	0.29504	8.4752	-9.7731	0.57692	0.48863	0.89688 (max o	2	7389; 6812	0.10434245	0.97997105		2.0
	ZINC22922447	-7.5	360.460	-5.5723	8	5	286.57	0.29358	95.81	4.8938	2560.287713445	0.29504	11.164	-18.886	0.61538	0.46232	0.9083 (max of	5	6848; 3022; 3427; 34	-0.0905537	0.44880185		2.0
	ZINC22922447	-7.5	359.452	-3.5945	8	4	297.54	0.31962	94.56	5.0524	2560.287713445	0.29504	9.1862	-12.183	0.61538	0.48164	0.9066 (max of	5	7068; 6849; 3023; 44	-0.0517303	0.44994864		2.0
	ZINC31156440	-7.5	359.350	-3.6225	9	4	244.43	0.44888	148.74	-3.4303	2560.287713445	0.30685	9.2142	-11.806	0.48	0.37141	0.94678 (max o	6	6291; 3035; 3069; 32	-0.6307459	0.09479927		2.0
	ZINC31160605	-7.5	403.359	-4.0657	11	4	283.96	0.46753	175.04	-7.5769	2560.287713445	0.27397	9.6574	-14.84	0.46429	0.49207	0.96519 (max o	3	7237; 4805; 5972	-0.4790679	0.86188626		2.0
	ZINC31160601	-7.5	403.359	-4.0657	11	4	283.96	0.46753	175.04	-7.5769	2560.287713445	0.27397	9.6574	-14.84	0.46429	0.49207	0.96519 (max o	3	7237; 4805; 5973	-0.4742516	0.8716627		2.0
	ZINC35442508	-7.5	353.485	-2.0884	7	2	285.52	0.25298	66.32	5.5175	2560.287713445	0.30685	7.6801	-6.806	0.56	0.5227	0.92342 (max o	8	6321; 3488; 3490; 38	-0.1075325	0.5628115		2.0
	ZINC36370069	-7.5	414.480	-4.6842	8	1	334.85	0.27669	102.1	-4.9417	2560.287713445	0.25571	10.276	-18.319	0.5	0.51152	0.95579 (max o	2	6892; 6656	0.7774965	-0.5086912		2.0
	ZINC49543607	-7.5	371.459	-0.9237	7	3	306.28	0.29591	87.99	-1.0938	2560.287713445	0.28412	6.5154	-3.2511	0.62963	0.51338	0.97658	1	6037	0.5024327	0.6859588		2.0
	ZINC49543607	-7.5	371.459	-0.9237	7	3	306.28	0.29591	87.99	-1.0938	2560.287713445	0.28412	6.5154	-3.2511	0.62963	0.51338	0.97658	1	6038	0.4894154	0.7041357		2.0
	ZINC67912201	-7.5	362.373	-1.8885	9	5	259.34	0.41852	145.91	-5.845	2560.287713445	0.30685	7.4802	-6.1545	0.48	0.59862	0.9594 (max of	3	6672; 6673; 6360	-0.924089	0.12070274		2.0
	ZINC68603333	-7.5	448.423	0.6883	10	1	317.9	0.37213	142.5	-6.1982	2560.287713445	0.23972	4.9034	2.8712	0.34375	0.47785	0.95949	1	7107	-0.5162055	-0.6566376		2.0
	ZINC02145188	-7.5	360.321	0.9481	9	2	255.86	0.38001	114.4	3.2797	2560.287713445	0.29504	4.6436	3.2134	0.5	0.39618	0.92702	1	3629	-0.5586844	0.49482188		1.0
	ZINC02145440	-7.5	364.377	-0.3499	7	1	232.66	0.51509	155.36	3.2314	2560.287713445	0.31963	5.9416	-1.0947	0.41667	0.25917	0.92619 (max o	3	559; 3195; 4070	0.5967942	-0.4788225		1.0
	ZINC03775143	-7.5	405.494	-1.2354	7	1	308.36	0.32086	131.91	7.4113	2560.287713445	0.27397	6.8271	-4.5093	0.57143	0.55716		0		-0.6941264	0.7198098		1.0
	ZINC03841828	-7.5	350.437	-0.9606	7	2	265.33	0.24796	76.64	-6.9079	2560.287713445	0.30685	6.5523	-3.1306	0.68	0.43407		0		-0.5802601	0.8072349		1.0
	ZINC04082322	-7.5	373.451	-1.0199	7	1	282.95	0.26499	108.39	4.7581	2560.287713445	0.28412	6.6116	-3.5897	0.59259	0.48669	0.87324	1	7469	0.95474553	0.09161635		1.0
	ZINC04097986	-7.5	398.838	0.3686	7	2	269.39	0.33383	105.59	-6.8258	2560.287713445	0.28412	5.2231	1.2974	0.44444	0.30515	0.87121 (max o	4	6162; 4142; 5417; 56	0.51177627	-0.2706838		1.0
	ZINC04098922	-7.5	421.377	-2.8041	10	6	288.26	0.43683	179.97	-1.4783	2560.287713445	0.25571	8.3958	-10.966	0.4	0.3734		0		-0.0524411	-0.9408005		1.0
	ZINC04214799	-7.5	440.583	3.0188	8	1	313.53	0.26699	115.42	1.3833	2560.287713445	0.25571	2.5729	11.806	0.56667	0.46623	0.96832	1	5510	0.5494687	-0.8315837		1.0
	ZINC04221721	-7.5	439.535	-0.8218	10	4	329.13	0.42029	182.96	9.5906	2560.287713445	0.25571	6.4135	-3.2139	0.43333	0.55381	0.86427 (max o	2	7676; 7144	-0.7530470	0.51621926		1.0
	ZINC04236341	-7.5	415.425	-3.5206	9	1	299.86	0.39899	161.15	2.9363	2560.287713445	0.26452	9.1123	-13.309	0.44828	0.55845		0		0.22308026	-0.9568045		1.0
	ZINC04237241	-7.5	354.433	-1.6869	7	1	278.46	0.26111	70.84	4.76	2560.287713445	0.29504	7.2786	-5.7174	0.57692	0.41554	0.96998 (max o	4	6742; 591; 4542; 498	-0.4711605	-0.3506106		1.0
	ZINC04270832	-7.5	403.479	0.2984	9	0	278.26	0.37975	133.08	2.8693	2560.287713445	0.29504	5.2933	1.0114	0.5	0.59553	0.83573	1	6148	0.2285655	0.3441501		1.0
	ZINC08624375	-7.5	356.417	-1.0918	8	3	260.3	0.33534	108.33	4.288	2560.287713445	0.30685	6.6835	-3.5581	0.6	0.52152	0.88798 (max o	2	3732; 5449	0.22597663	-0.4138702		1.0
	ZINC08627202	-7.5	380.463	-2.2201	8	3	284.59	0.28638	108.73	1.9743	2560.287713445	0.28412	7.8118	-7.814	0.62963	0.46856	0.87816 (max o	2	4175; 4176	0.01419273	-0.5326211		1.0
	ZINC08636325	-7.5	429.563	-0.6303	7	5	329.88	0.3374	121.36	3.7352	2560.287713445	0.25571	6.222	-2.4649	0.46667	0.38758	0.88589 (max o	2	6983; 1254	0.3957635	0.18879977		1.0
	ZINC08636502	-7.5	421.473	0.1887	9	3	289.54	0.39994	135.82	4.1653	2560.287713445	0.26452	5.403	0.71336	0.44828	0.37507	0.97415 (max o	8	7335; 6479; 6184; 61	0.47375298	0.05616250		1.0
	ZINC08880012	-7.5	383.468	0.0053	8	1	277.15	0.35436	121.32	3.0025	2560.287713445	0.29504	5.5864	0.017963	0.61538	0.54325	0.94008 (max o	2	6762; 6522	0.9146556	0.3866374		1.0
	ZINC12480755	-7.5	387.522	-0.703	6	1	323.81	0.2238	77.77	3.9855	2560.287713445	0.27397	6.2947	-2.566	0.57143	0.58747	0.90045 (max o	2	2144; 3322	-0.2348942	-0.1441292		1.0
	ZINC12481105	-7.5	435.587	-0.5666	7	1	350.3	0.28104	113.43	6.5687	2560.287713445	0.25571	6.1583	-2.2158	0.53333	0.61667	0.934 (max of 3	3	5909; 4207; 4208	-0.1103961	-0.3123868		1.0
	ZINC12481189	-7.5	368.452	-2.5503	8	0	284.57	0.24953	85.38	3.9125	2560.287713445	0.29504	8.142	-8.6438	0.53846	0.63055	0.93361	1	6498	-0.2421582	-0.1652058		1.0

Structure	Molecule Name	affinity	Total Molweight	cLogP	H-Acceptors	H-Donors	Total Surface Area	Relative PSA	Polar Surface Area	Druglikeness	Kd (nM) @ 20°C	LE from K...	LLE from Kd ...	LELP from Kd (n...	Shape Index	Molecular...	Neighbor Si...	Neig...	Neighbor	Neighbor ...	Neighbor ...	Ring Systems	frequency
	ZINC12481244	-7.5	379.499	-1.2063	6	0	292.19	0.26633	101.15	5.9453	2560.287713445	0.29504	6.798	-4.0885	0.53846	0.61498	0.87695	1	7171	0.90857816	-0.1968549		1.0
	ZINC12481688	-7.5	360.476	-2.1501	6	2	283.72	0.24404	81.49	3.1101	2560.287713445	0.29504	7.7418	-7.2874	0.53846	0.54796	0.83638 (max o	2	1539; 2519	0.27332124	0.81371236		1.0
	ZINC12603988	-7.5	440.607	-0.3328	8	3	325.96	0.31648	134.59	-0.11466	2560.287713445	0.25571	5.9245	-1.3015	0.46667	0.53154		0		0.9162933	0.1315623		1.0
	ZINC12901069	-7.5	432.404	-2.1948	10	1	314.98	0.36806	128.52	1.222	2560.287713445	0.24746	7.7865	-8.8694	0.45161	0.35526	0.96739 (max o	3	3746; 4628; 5493	-0.9385968	-0.2075748		1.0
	ZINC13349056	-7.5	416.405	-1.5687	9	3	306.73	0.35217	137.38	-12.421	2560.287713445	0.25571	7.1604	-6.1348	0.53333	0.43785	0.96092 (max o	3	7005; 968; 1503	0.87874025	0.15144426		1.0
	ZINC15675312	-7.5	355.841	-1.0209	6	4	252.63	0.27091	95.4	0.773	2560.287713445	0.31963	6.6126	-3.194	0.625	0.45195	0.9115 (max of	5	7015; 6748; 6223; 21	0.1275235	-0.4580787		1.0
	ZINC15676122	-7.5	362.428	-1.8015	8	2	262.51	0.28376	110.68	4.0599	2560.287713445	0.29504	7.3932	-6.1059	0.46154	0.57592	0.87087 (max o	2	3811; 5888	0.83161503	-0.3596839		1.0
	ZINC15676122	-7.5	361.420	-1.6126	8	1	273.48	0.31249	109.43	4.6035	2560.287713445	0.29504	7.2043	-5.4656	0.46154	0.57592	0.87695 (max o	2	3812; 5887	0.84944284	-0.3661978		1.0
	ZINC15676232	-7.5	361.444	-0.5581	8	1	272.35	0.26323	81.77	6.5249	2560.287713445	0.29504	6.1498	-1.8916	0.5	0.49572	0.87452	1	5891	0.9196326	-0.0083468		1.0
	ZINC15676232	-7.5	363.460	-4.6456	8	3	275.42	0.27144	84.22	4.4828	2560.287713445	0.29504	10.237	-15.745	0.5	0.45194	0.88178	1	5892	-0.276235	0.94236946		1.0
	ZINC15676232	-7.5	362.452	-2.5359	8	2	261.38	0.23231	83.02	6.001	2560.287713445	0.29504	8.1276	-8.595	0.5	0.49572	0.87452	1	5889	0.91186714	0.02850225		1.0
	ZINC15676232	-7.5	362.452	-2.6678	8	2	286.39	0.29935	82.97	4.842	2560.287713445	0.29504	8.2595	-9.042	0.5	0.45194	0.88178	1	5890	-0.2533981	0.94915974		1.0
	ZINC15676294	-7.5	380.403	-3.2032	8	3	278.66	0.29954	115.54	5.5395	2560.287713445	0.27397	8.7949	-11.692	0.53571	0.48243	0.8703 (max of	4	1522; 3814; 4265; 43	-0.2771789	0.46440345		1.0
	ZINC15676294	-7.5	381.411	-3.0716	8	4	292.7	0.33314	116.74	3.3961	2560.287713445	0.27397	8.6633	-11.211	0.53571	0.4379	0.8703 (max of	3	6539; 1523; 4266	0.15997589	-0.7435405		1.0
	ZINC15708400	-7.5	359.405	-2.7274	8	4	291.79	0.36053	104.88	3.3396	2560.287713445	0.29504	8.3191	-9.244	0.61538	0.4372	0.87344 (max o	3	6244; 4267; 4348	-0.2169198	0.10569261		1.0
	ZINC20463007	-7.5	365.472	-2.2505	7	2	292.06	0.30751	107.97	5.0496	2560.287713445	0.29504	7.8422	-7.6277	0.5	0.52512	0.92721 (max o	4	6803; 6552; 6252; 62	-0.0942033	-0.3442341		1.0
	ZINC20463218	-7.5	389.494	-1.5456	7	1	302.49	0.21554	89.52	4.2327	2560.287713445	0.27397	7.1373	-5.6415	0.57143	0.59789	0.93091 (max o	3	7379; 4778; 5846	-0.1305291	-0.0813275		1.0
	ZINC20463239	-7.5	365.472	-1.899	7	1	293.6	0.26597	85.19	5.5966	2560.287713445	0.29504	7.4907	-6.4363	0.53846	0.58572	0.93367 (max o	5	6499; 4640; 5132; 55	-0.1422204	-0.2723861		1.0
	ZINC20463239	-7.5	364.464	-2.0306	7	0	279.56	0.22911	83.99	10.123	2560.287713445	0.29504	7.6223	-6.8824	0.53846	0.59059	0.89688 (max o	8	2583; 5918; 4720; 51	-0.2263988	-0.2194468		1.0
	ZINC20463998	-7.5	372.467	-3.0191	7	3	291.77	0.27546	85.52	-0.27172	2560.287713445	0.28412	8.6108	-10.626	0.59259	0.48817	0.93373 (max o	4	7849; 7847; 7629; 73	0.4191165	0.42602012		1.0
	ZINC20463998	-7.5	371.459	-1.6528	7	2	277.73	0.23883	84.32	4.3573	2560.287713445	0.28412	7.2445	-5.8173	0.59259	0.48248	0.97149 (max o	8	7850; 7848; 7630; 73	0.6805411	0.57008255		1.0
	ZINC20465110	-7.5	358.437	-2.0887	9	3	265.67	0.30621	97.74	7.3305	2560.287713445	0.30685	7.6804	-6.807	0.56	0.585	0.89839 (max o	5	2233; 2997; 3397; 51	-0.3651781	0.08289812		1.0
	ZINC20465110	-7.5	359.445	-4.1984	9	4	279.71	0.34103	98.94	3.6512	2560.287713445	0.30685	9.7901	-13.682	0.56	0.585	0.89805 (max o	2	7396; 5930	-0.3291876	0.09432455		1.0
	ZINC20465268	-7.5	374.456	-3.6599	9	4	291.91	0.34887	104.93	2.6434	2560.287713445	0.29504	9.2516	-12.405	0.53846	0.55641	0.86577	1	7522	-0.7989239	-0.5745262		1.0
	ZINC20465921	-7.5	365.516	-2.3223	6	4	298.4	0.24296	83.63	-5.813	2560.287713445	0.29504	7.914	-7.871	0.57692	0.5019	0.96998 (max o	3	6827; 6588; 5570	0.5365966	0.7767976		1.0
	ZINC20465921	-7.5	364.508	-0.956	6	3	284.36	0.20558	82.43	-1.2889	2560.287713445	0.29504	6.5477	-3.2402	0.57692	0.49556	0.96998 (max o	3	6829; 6590; 5571	-0.4078692	0.74338865		1.0
	ZINC20504951	-7.5	433.527	-0.1867	9	3	332.57	0.28063	113.58	4.4643	2560.287713445	0.24746	5.7784	-0.75447	0.54839	0.53793	0.89334 (max o	3	7230; 7229; 7069	0.06819755	0.465113		1.0
	ZINC22921165	-7.5	370.475	-2.5911	7	3	307.97	0.26337	78.77	4.844	2560.287713445	0.28412	8.1828	-9.1198	0.62963	0.47815	0.88355 (max o	2	1560; 2655	-0.0013722	0.5094569		1.0
	ZINC22926985	-7.5	426.671	-4.2456	7	4	364.33	0.28326	80.74	4.7689	2560.287713445	0.26452	9.8373	-16.05	0.68966	0.66968	0.91204 (max o	4	7778; 7637; 7178; 59	0.4623641	-0.6190811		1.0
	ZINC22926985	-7.5	426.671	-4.2456	7	4	364.33	0.28326	80.74	2.9931	2560.287713445	0.26452	9.8373	-16.05	0.68966	0.68037	0.90299 (max o	6	7637; 7178; 7060; 70	0.479117	-0.6043527		1.0
	ZINC22926985	-7.5	427.679	-6.3553	7	5	378.37	0.30986	81.94	0.79433	2560.287713445	0.26452	11.947	-24.026	0.68966	0.68245	0.90299 (max o	2	7059; 5958	0.49517	-0.5872471		1.0
	ZINC32123972	-7.5	387.395	0.1367	10	2	292.42	0.37638	126.51	0.40949	2560.287713445	0.27397	5.455	0.49896	0.46429	0.53047	0.9543 (max of	4	5988; 5987; 5618; 56	-0.6129841	0.21489553		1.0
	ZINC32123972	-7.5	388.403	-1.8411	10	3	281.45	0.35207	127.76	-0.11909	2560.287713445	0.27397	7.4328	-6.7201	0.46429	0.53047	0.92774 (max o	8	7424; 5988; 5172; 56	-0.6444179	0.20243193		1.0
	ZINC32123972	-7.5	388.403	-1.8411	10	3	281.45	0.35207	127.76	-0.11909	2560.287713445	0.27397	7.4328	-6.7201	0.46429	0.53047	0.93686 (max o	5	5618; 5619; 5620; 59	-0.6271413	0.19649032		1.0
	ZINC35456930	-7.5	404.367	-3.1441	11	6	270.25	0.4871	175.37	-5.0325	2560.287713445	0.27397	8.7358	-11.476	0.46429	0.37662	0.95148 (max o	2	6881; 6004	0.779598	-0.0296053		1.0
	ZINC35456932	-7.5	404.367	-3.1441	11	6	270.25	0.4871	175.37	-5.0325	2560.287713445	0.27397	8.7358	-11.476	0.46429	0.38571	0.95148 (max o	2	6643; 6003	0.7922834	-0.0477439		1.0
	ZINC40309631	-7.5	395.435	-0.2179	9	2	292.03	0.39763	139.34	1.2185	2560.287713445	0.28412	5.8096	-0.76694	0.55556	0.45875		0		0.33834863	0.90494895		1.0
	ZINC67913819	-7.5	381.467	-0.7857	7	3	289.97	0.29072	123.5	-3.2548	2560.287713445	0.28412	6.3774	-2.7654	0.44444	0.45552	0.94302	1	3109	-0.5029197	-0.5985127		1.0
	ZINC68590699	-7.5	362.330	-3.6103	10	6	234.68	0.53797	161.6	-4.3947	2560.287713445	0.30685	9.202	-11.766	0.44	0.34878	0.94351 (max o	4	6907; 6677; 4438; 54	0.5342607	0.5919664		1.0
	ZINC70666354	-7.5	368.324	-1.406	9	2	276.85	0.40083	137.61	2.2987	2560.287713445	0.28412	6.9977	-4.9487	0.55556	0.39106		0		-0.7620947	0.48981434		1.0
	ZINC35464302	-7.5	367.373	-0.8653	8	2	290.28	0.34312	133.19	-7.5278	2560.287713445	0.29504	6.457	-2.9328	0.57692	0.3816	0.96998 (max o	5	2266; 2267; 4378; 43	-0.0154037	0.83053607		0.0
	ZINC04533885	-7.5	373.408	-5.0956	11	5	287.15	0.48685	184.17	1.0103	2560.287713445	0.29504	10.687	-17.271	0.61538	0.61457	0.95401	1	4582	0.9060285	-0.3066798		0.0
	ZINC31163094	-7.5	447.463	-4.3629	13	5	344.88	0.43145	199.64	-0.0014118	2560.287713445	0.24746	9.9546	-17.631	0.67742	0.7663	1	1	5198	0.30815825	0.93981844		0.0

Structure	Molecule Name	affinity	Total Molweight	cLogP	H-Acceptors	H-Donors	Total Surface Area	Relative PSA	Polar Surface Area	Druglikeness	Kd (nM) @ 20°C	LE from K...	LLE from Kd ...	LELP from Kd (n...	Shape Index	Molecular...	Neighbor Si...	Neig...	Neighbor	Neighbor ...	Neighbor ...	Ring Systems	frequency
	ZINC49600148	-7.5	369.476	0.8703	6	4	297.04	0.26838	121.05	1.0099	2560.287713445	0.29504	4.7214	2.9497	0.57692	0.69138	0.98284 (max o	3	6356; 6357; 4853	0.33053806	-0.7443535		0.0
	ZINC35457506	-7.5	436.540	0.256	9	8	340.73	0.33692	171.07	-3.3718	2560.287713445	0.25571	5.3357	1.0012	0.66667	0.73168	0.99612 (max o	3	7263; 7088; 5638	-0.9324243	-0.2959505		0.0
	ZINC02111254	-7.4	403.410	-1.574	9	3	303.54	0.37049	144.86	3.7186	3039.798880273	0.261	7.0912	-6.0307	0.58621	0.58714	0.96473 (max o	6	2355; 2721; 2732; 44	0.04348528	0.02044668		172.0
	ZINC02124930	-7.4	447.462	-1.3683	10	2	341.71	0.3493	143.09	5.6987	3039.798880273	0.23653	6.8855	-5.785	0.5625	0.60557	0.95983 (max o	9	6385; 2740; 3566; 40	0.05382340	0.0550604		172.0
	ZINC02124197	-7.4	350.370	-1.472	7	1	262.03	0.3906	130.06	1.467	3039.798880273	0.31537	6.9892	-4.6675	0.625	0.52912	0.96029 (max o	2	5294; 5308	0.20896456	0.19880024		172.0
	ZINC08764727	-7.4	351.354	-0.0802	8	3	262.9	0.35656	114.32	4.149	3039.798880273	0.30276	5.5974	-0.2649	0.6	0.53136	0.97118 (max o	3	6760; 5700; 5749	0.00428853	0.06052756		172.0
	ZINC04537048	-7.4	378.404	-2.0163	9	3	300.73	0.3685	136.66	-9.1308	3039.798880273	0.28033	7.5335	-7.1926	0.66667	0.63515	0.92721 (max o	7	6737; 2176; 2514; 41	-0.0142580	-0.4608853		134.0
	ZINC08762004	-7.4	427.524	-1.7887	10	5	306.89	0.38268	187.42	-6.0696	3039.798880273	0.261	7.3059	-6.8534	0.58621	0.60378	0.95642 (max o	4	6515; 3996; 4234; 48	0.582669	-0.3006899		134.0
	ZINC03874915	-7.4	441.440	-6.0726	12	3	324.93	0.51097	237.22	-4.4889	3039.798880273	0.2523	11.59	-24.069	0.56667	0.69796	0.97304 (max o	3	4056; 5347; 5737	-0.6283671	0.651336		134.0
	ZINC13522965	-7.4	379.348	-3.1158	11	3	286.88	0.45747	173.25	-3.5051	3039.798880273	0.28033	8.633	-11.115	0.66667	0.58949	0.8325	1	5504	-0.9353983	0.07514342		134.0
	ZINC02530804	-7.4	350.350	-2.6331	9	3	271.77	0.42179	150.65	-10.6	3039.798880273	0.30276	8.1503	-8.6971	0.64	0.55305	0.91964 (max o	9	7323; 6738; 6415; 64	-0.0526987	-0.4805567		134.0
	ZINC04027281	-7.4	421.496	-1.1444	10	5	317.73	0.35219	171.2	-51.722	3039.798880273	0.2523	6.6616	-4.5359	0.5	0.60305	0.97533 (max o	4	7467; 6945; 4578; 53	-0.1431274	-0.4216392		134.0
	ZINC08764481	-7.4	399.873	-1.6651	8	4	302.26	0.34374	146.2	4.3744	3039.798880273	0.28033	7.1823	-5.9398	0.48148	0.57079	0.93361 (max o	8	7448; 7166; 6485; 52	0.6474803	0.33508876		134.0
	ZINC31166575	-7.4	366.277	-4.9637	10	2	267.45	0.47456	173.32	-1.6285	3039.798880273	0.29111	10.481	-17.051	0.53846	0.5543	0.96438 (max o	3	6862; 6861; 5976	-0.6060983	0.7078345		134.0
	ZINC34302723	-7.4	363.456	-1.1943	7	3	289.99	0.31311	125.97	3.2028	3039.798880273	0.29111	6.7115	-4.1026	0.53846	0.58185	0.94951 (max o	5	7430; 6872; 6443; 63	0.01734156	0.5983096		134.0
	ZINC32124119	-7.4	436.555	-3.4532	10	6	332.52	0.33712	175.78	0.43345	3039.798880273	0.24416	8.9704	-14.143	0.48387	0.58003	0.96018	1	6871	0.43716443	-0.8084292		134.0
	ZINC49180800	-7.4	352.362	-1.4927	8	3	270.67	0.37337	135.99	2.2083	3039.798880273	0.30276	7.0099	-4.9304	0.56	0.62155	0.98434 (max o	5	7446; 7419; 7099; 68	0.39619672	0.42421722		134.0
	ZINC31167856	-7.4	352.362	-1.4927	8	3	270.67	0.37337	135.99	2.2083	3039.798880273	0.30276	7.0099	-4.9304	0.64	0.66553	0.98434 (max o	8	7800; 7419; 7265; 72	0.3923668	0.38018164		134.0
	ZINC49180791	-7.4	398.410	-3.2289	10	5	296.1	0.43482	187.35	-0.71607	3039.798880273	0.27032	8.7461	-11.945	0.53571	0.63324	0.95148 (max o	4	7655; 7270; 7239; 66	-0.3912783	0.73266906		134.0
	ZINC49181820	-7.4	414.409	-3.3527	11	6	302.34	0.46881	204.09	0.26746	3039.798880273	0.261	8.8699	-12.846	0.51724	0.61417	0.98727 (max o	2	7656; 6899	-0.3095047	0.7711088		134.0
	ZINC03872257	-7.4	392.454	-2.6882	9	4	310.96	0.37079	155.07	3.953	3039.798880273	0.27032	8.2054	-9.9446	0.5	0.60894		0		0.76545125	-0.5440120		134.0
	ZINC04090385	-7.4	369.504	-0.9277	7	3	298.03	0.30467	125.97	2.7954	3039.798880273	0.29111	6.4449	-3.1868	0.61538	0.5902	0.96721 (max o	7	6776; 3208; 5754; 57	0.4947165	0.01416564		127.0
	ZINC04089906	-7.4	355.477	-1.3821	7	3	284.27	0.31941	125.97	2.3079	3039.798880273	0.30276	6.8993	-4.5651	0.6	0.59369	0.95 (max of 1	10	7138; 6521; 6127; 32	0.48374552	0.03464055		127.0
	ZINC04088877	-7.4	369.504	-0.9277	7	3	298.03	0.30467	125.97	2.7954	3039.798880273	0.29111	6.4449	-3.1868	0.61538	0.59232	0.96721 (max o	9	7313; 6953; 6128; 61	0.5171412	0.04845379		127.0
	ZINC04090090	-7.4	369.504	-0.9277	7	3	298.03	0.30467	125.97	3.0831	3039.798880273	0.29111	6.4449	-3.1868	0.61538	0.60594	0.96721 (max o	9	7138; 7135; 6521; 49	0.5307437	0.06379612		127.0
	ZINC08481030	-7.4	383.531	-0.4733	7	3	311.79	0.29122	125.97	3.4553	3039.798880273	0.28033	5.9905	-1.6884	0.62963	0.60923	0.94927 (max o	10	7501; 7331; 6776; 67	0.5197985	-0.0243073		127.0
	ZINC04082419	-7.4	383.531	-0.4733	7	3	311.79	0.29122	125.97	3.4553	3039.798880273	0.28033	5.9905	-1.6884	0.62963	0.60923	0.94927 (max o	11	7501; 7331; 7311; 71	0.5552903	0.01129194		127.0
	ZINC04085591	-7.4	369.504	-0.9277	7	3	298.03	0.30467	125.97	2.3672	3039.798880273	0.29111	6.4449	-3.1868	0.61538	0.61002	0.93361 (max o	8	7138; 7136; 6954; 65	0.53852654	0.04597222		127.0
	ZINC12887301	-7.4	383.531	-0.4733	7	3	311.79	0.29122	125.97	3.1439	3039.798880273	0.28033	5.9905	-1.6884	0.62963	0.613	0.96885 (max o	5	7331; 6954; 5867; 61	0.53537273	0.01155057		127.0
	ZINC31170287	-7.4	421.464	0.5332	8	2	326.7	0.30487	133.19	-4.2966	3039.798880273	0.2523	4.984	2.1134	0.5	0.60057	0.96343 (max o	7	7555; 7433; 7259; 72	0.08505466	0.7463958		127.0
	ZINC35271426	-7.4	421.464	0.5332	8	2	326.7	0.30487	133.19	-4.2966	3039.798880273	0.2523	4.984	2.1134	0.5	0.60182	0.96343 (max o	9	7555; 7437; 7433; 74	0.08786075	0.77881104		127.0
	ZINC31170264	-7.4	395.426	-0.9065	8	4	297.67	0.34521	147.35	-8.9294	3039.798880273	0.27032	6.4237	-3.3535	0.46429	0.60852	0.95983 (max o	4	7434; 7248; 7079; 68	0.4374981	-0.5928479		127.0
	ZINC03838739	-7.4	362.449	-2.4337	9	7	270.13	0.52949	189.03	2.1562	3039.798880273	0.31537	7.9509	-7.717	0.58333	0.67381		0		0.95998293	-0.0485735		127.0
	ZINC72319910	-7.4	412.477	1.4598	8	4	318.09	0.3096	133.52	-15.489	3039.798880273	0.261	4.0574	5.5932	0.55172	0.67479	0.97649 (max o	3	5684; 5685; 6374	0.8653252	-0.3976988		120.0
	ZINC72319911	-7.4	412.477	1.4598	8	4	318.09	0.3096	133.52	-15.489	3039.798880273	0.261	4.0574	5.5932	0.55172	0.67479	0.97649 (max o	2	5686; 6373	0.8324845	-0.4251134		120.0
	ZINC39205607	-7.4	357.287	-7.4085	12	8	236.1	0.6443	220.43	-5.5171	3039.798880273	0.31537	12.926	-23.491	0.54167	0.62085	0.94378 (max o	3	6895; 4846; 6349	0.45107132	-0.7684603		120.0
	ZINC39205606	-7.4	357.287	-7.4085	12	8	236.1	0.6443	220.43	-5.5171	3039.798880273	0.31537	12.926	-23.491	0.54167	0.62032	0.94378 (max o	3	6895; 6350; 4846	0.46479747	-0.7821663		120.0
	ZINC35466050	-7.4	418.525	1.5486	8	6	321.01	0.30716	139.84	-10.55	3039.798880273	0.261	3.9686	5.9334	0.58621	0.68707	0.99279 (max o	3	6888; 6310; 6340	0.4680484	0.7058981		120.0
	ZINC35466048	-7.4	418.525	1.5486	8	6	321.01	0.30716	139.84	-10.55	3039.798880273	0.261	3.9686	5.9334	0.58621	0.69528	0.99279 (max o	3	6888; 6341; 6310	0.45910233	0.6888466		120.0
	ZINC35050063	-7.4	418.525	1.5486	8	6	321.01	0.30716	139.84	-10.55	3039.798880273	0.261	3.9686	5.9334	0.58621	0.68707	0.99279 (max o	3	6888; 6341; 6340	0.44623914	0.7065837		120.0
	ZINC05835358	-7.4	396.434	0.3826	8	6	325.5	0.30292	139.84	-21.493	3039.798880273	0.27032	5.1346	1.4154	0.53571	0.45377	0.96253 (max o	3	7159; 6463; 6462	0.79226506	0.3198181		120.0
	ZINC12880479	-7.4	351.351	-1.2752	10	4	255.48	0.45734	143.78	-54.673	3039.798880273	0.31537	6.7924	-4.0435	0.58333	0.57517	0.9665 (max of	3	6774; 6773; 6775	0.87575126	-0.2852188		120.0
	ZINC31169927	-7.4	350.406	-0.369	8	6	260.04	0.37917	139.84	-3.8701	3039.798880273	0.31537	5.8862	-1.1701	0.58333	0.6335	0.95053	1	6867	0.7055868	-0.3882485		120.0

Structure	Molecule Name	affinity	Total Molweight	cLogP	H-Acceptors	H-Donors	Total Surface Area	Relative PSA	Polar Surface Area	Druglikeness	Kd (nM) @ 20°C	LE from K...	LLE from Kd ...	LELP from Kd (n...	Shape Index	Molecular...	Neighbor Si...	Neig...	Neighbor	Neighbor ...	Neighbor ...	Ring Systems	frequency
	ZINC49601686	-7.4	365.354	-3.573	10	4	261.9	0.46873	165.81	1.5639	3039.798880273	0.30276	9.0902	-11.802	0.56	0.66021	0.94678 (max o	5	7748; 7658; 7567; 72	-0.62821	-0.363661		120.0
	ZINC03869250	-7.4	423.170	-13.388	15	2	266.13	0.71134	263.54	-22.848	3039.798880273	0.28033	18.905	-47.758	0.44444	0.42253	0.9578 (max of	9	6403; 6098; 6095; 36	0.6696775	0.4796632		67.0
	ZINC03869250	-7.4	424.178	-12.14	15	3	264.95	0.71006	260.71	-32.8	3039.798880273	0.28033	17.658	-43.308	0.44444	0.40459	0.9578 (max of	9	6404; 6099; 3658; 40	0.6464291	0.51964897		67.0
	ZINC08616444	-7.4	393.355	-0.2858	12	1	282.15	0.47553	157.75	1.9052	3039.798880273	0.27032	5.803	-1.0573	0.42857	0.30182	0.9428 (max of	5	5015; 5302; 5456; 57	-0.6272692	0.37456006		67.0
	ZINC03927870	-7.4	363.197	-7.1787	12	3	230	0.63839	201.54	-29.747	3039.798880273	0.31537	12.696	-22.763	0.5	0.40689	0.91522 (max o	3	7786; 3316; 6108	-0.3400554	-0.8978681		67.0
	ZINC03927870	-7.4	364.205	-5.9311	12	4	228.82	0.63653	198.71	-33.738	3039.798880273	0.31537	11.448	-18.807	0.5	0.38512	0.89637	1	6107	-0.3179242	-0.9017983		67.0
	ZINC31167909	-7.4	444.431	-0.9145	11	5	319.48	0.41186	172.21	-7.1849	3039.798880273	0.24416	6.4317	-3.7455	0.6129	0.60414	1 (max of 3)	3	3914; 4380; 5610	0.30519524	-0.7137153		49.0
	ZINC04042950	-7.4	355.342	-0.8017	9	4	258.34	0.41356	134.55	-5.0153	3039.798880273	0.30276	6.3189	-2.648	0.48	0.31523	0.94658 (max o	3	4859; 4999; 5000	-0.8205952	-0.3668772		49.0
	ZINC72320194	-7.4	416.421	-1.0879	10	6	296.82	0.40981	166.14	-5.3918	3039.798880273	0.261	6.6051	-4.1683	0.58621	0.54079	0.99279 (max o	7	3985; 4445; 4455; 60	0.09019764	0.49489626		49.0
	ZINC31166230	-7.4	355.318	-3.0071	9	4	255.46	0.4295	148.74	-3.2919	3039.798880273	0.30276	8.5243	-9.9325	0.48	0.37483	0.95631 (max o	4	7071; 6607; 3356; 56	0.21505027	-0.6440103		49.0
	ZINC35456635	-7.4	374.384	-1.0158	9	7	261.66	0.42689	160.07	-3.6927	3039.798880273	0.29111	6.533	-3.4894	0.46154	0.43745	0.9464 (max of	3	5635; 5636; 6002	-0.2167263	0.90079385		49.0
	ZINC04044005	-7.4	366.196	0.547	7	4	247.38	0.33875	108.25	-2.9168	3039.798880273	0.32908	4.9702	1.6622	0.47826	0.30695		0		0.407487	-0.9089299		49.0
	ZINC13544224	-7.4	387.300	-2.5082	13	4	263.88	0.54813	199.89	-7.9155	3039.798880273	0.28033	8.0254	-8.9474	0.44444	0.36261		0		-0.3312954	0.91474646		49.0
	ZINC05531765	-7.4	357.433	-3.525	10	5	259.89	0.41737	180.82	4.7798	3039.798880273	0.30276	9.0422	-11.643	0.64	0.56335	0.88896	1	4119	-0.0902957	0.80726045		47.0
	ZINC16321744	-7.4	394.339	-0.8122	12	1	281.98	0.47156	147.41	4.1075	3039.798880273	0.27032	6.3294	-3.0046	0.42857	0.34882	0.90883 (max o	2	4634; 5039	0.07529195	-0.6937493		44.0
	ZINC03872751	-7.4	361.206	-8.4657	13	4	230.21	0.69893	217.22	-20.626	3039.798880273	0.31537	13.983	-26.844	0.41667	0.39574	0.92257 (max o	5	6997; 4055; 4933; 57	-0.3504557	-0.4282626		44.0
	ZINC06090961	-7.4	385.062	-6.8798	10	2	217.3	0.55117	161.1	-29.415	3039.798880273	0.36042	12.397	-19.088	0.57143	0.57984	0.82791	1	5041	-0.0049601	-0.8976103		41.0
	ZINC03870128	-7.4	366.174	-9.7178	13	4	227.86	0.69784	218.63	-30.796	3039.798880273	0.31537	15.235	-30.814	0.5	0.57412	0.92619 (max o	5	6717; 6097; 4934; 57	-0.2490619	-0.7421162		41.0
	ZINC03870128	-7.4	365.166	-10.965	13	3	229.04	0.6994	221.46	-26.867	3039.798880273	0.31537	16.483	-34.77	0.5	0.59279	0.92619 (max o	5	6718; 4935; 5730; 57	-0.2731177	-0.7045302		41.0
	ZINC03190127	-7.4	438.169	0.0808	9	1	249.79	0.38901	111.24	3.9348	3039.798880273	0.32908	5.4364	0.24553	0.52174	0.47871	0.92193 (max o	3	7759; 6544; 3939	0.72668165	0.5403852		41.0
	ZINC20610528	-7.4	350.374	-2.1345	8	2	255.04	0.43287	143.94	5.8909	3039.798880273	0.31537	7.6517	-6.7682	0.58333	0.58625	0.96029 (max o	4	2734; 4019; 5905; 59	0.64504665	0.14855996		36.0
	ZINC03871401	-7.4	423.170	-13.388	15	2	266.13	0.71134	263.54	-22.95	3039.798880273	0.28033	18.905	-47.758	0.44444	0.4085	0.95493 (max o	9	6403; 3657; 4048; 60	0.6510048	0.4827215		33.0
	ZINC03871401	-7.4	424.178	-12.14	15	3	264.95	0.71006	260.71	-32.906	3039.798880273	0.28033	17.658	-43.308	0.44444	0.39014	0.95493 (max o	9	6404; 3658; 4049; 49	0.62745464	0.5301896		33.0
	ZINC04200821	-7.4	407.466	-5.627	11	6	287.98	0.48555	200.88	2.53	3039.798880273	0.28033	11.144	-20.073	0.55556	0.55476	0.93373 (max o	10	6431; 3650; 3653; 45	-0.603157	-0.1132438		29.0
	ZINC03838839	-7.4	413.537	-2.1444	9	6	319.92	0.34774	158.36	5.0486	3039.798880273	0.261	7.6616	-8.2162	0.51724	0.56254	0.9339 (max of	8	6698; 6396; 6352; 27	-0.5653857	-0.2091204		29.0
	ZINC04151704	-7.4	401.390	-4.5066	11	4	299.42	0.45274	174.32	0.3193	3039.798880273	0.27032	10.024	-16.672	0.57143	0.58241	0.86774 (max o	3	5003; 5717; 6030	-0.5320101	-0.2332308		29.0
	ZINC39560948	-7.4	371.456	-3.2715	9	6	281.4	0.39534	158.36	1.437	3039.798880273	0.29111	8.7887	-11.238	0.53846	0.53755	0.93069 (max o	8	6703; 6698; 6659; 63	-0.6030867	-0.2012277		29.0
	ZINC04221650	-7.4	416.429	-1.8695	12	5	309.21	0.45309	180.52	-3.0399	3039.798880273	0.261	7.3867	-7.163	0.48276	0.58109	0.934	1	6432	0.31490606	-0.6949988		29.0
	ZINC04221594	-7.4	438.507	-1.4394	11	6	344.93	0.47804	210.69	-14.989	3039.798880273	0.2523	6.9566	-5.7052	0.5	0.58427		0		0.5315596	-0.8419588		29.0
	ZINC39560948	-7.4	370.448	-2.0468	9	6	285.12	0.40323	153.78	2.8256	3039.798880273	0.29111	7.564	-7.031	0.53846	0.58539		0		0.7238855	-0.6590636		29.0
	ZINC35271341	-7.4	388.455	-0.2632	8	5	281.14	0.3505	136.68	-5.3631	3039.798880273	0.28033	5.7804	-0.9389	0.51852	0.43395	0.9578 (max of	3	3057; 4396; 4821	-0.0656135	0.59021467		23.0
	ZINC72319914	-7.4	406.470	-1.0841	9	7	287.33	0.38875	160.07	-4.0127	3039.798880273	0.27032	6.6013	-4.0105	0.46429	0.44224	0.94862 (max o	7	6916; 6678; 6680; 39	0.08427545	-0.7510681		23.0
	ZINC19323154	-7.4	357.469	-2.4049	7	5	264.45	0.3274	105.67	3.1491	3039.798880273	0.30276	7.9221	-7.9434	0.44	0.62071	0.97118 (max o	6	7368; 7367; 7200; 70	-0.2294774	0.69145244		23.0
	ZINC12882601	-7.4	446.478	-1.5067	10	2	333.59	0.33847	137.1	-0.24237	3039.798880273	0.23653	7.0239	-6.3701	0.53125	0.46794	0.97948 (max o	11	773; 1217; 1785; 229	0.52608943	0.17910641		22.0
	ZINC20464873	-7.4	350.481	-1.0738	6	3	266.88	0.19125	72.78	3.948	3039.798880273	0.30276	6.591	-3.5468	0.56	0.46863	0.95631 (max o	7	6818; 6587; 6585; 18	-0.4705135	0.1380898		21.0
	ZINC08964842	-7.4	401.382	1.4655	12	6	283.5	0.49531	184.16	1.3234	3039.798880273	0.261	4.0517	5.615	0.48276	0.31394	0.97649 (max o	12	6759; 6484; 2906; 37	-0.2065750	-0.5941765		20.0
	ZINC08740540	-7.4	385.383	1.8112	11	5	277.15	0.45939	163.93	1.3234	3039.798880273	0.27032	3.706	6.7003	0.5	0.33184	0.97288 (max o	11	6759; 6484; 6205; 33	-0.2171130	-0.6113861		20.0
	ZINC08254710	-7.4	359.449	0.2129	6	4	268.73	0.34871	116.92	3.9691	3039.798880273	0.30276	5.3043	0.70321	0.52	0.37211	0.9302 (max of	5	7164; 6743; 6478; 28	0.48419288	0.11618023		18.0
	ZINC08636379	-7.4	391.490	1.0128	7	3	288.01	0.32377	119.69	4.1629	3039.798880273	0.28033	4.5044	3.6129	0.59259	0.45737	0.9083 (max of	4	281; 3736; 4150; 582	-0.5275135	0.2788511		17.0
	ZINC14825926	-7.4	370.313	-1.0894	11	1	264.05	0.45525	137.54	0.69325	3039.798880273	0.29111	6.6066	-3.7422	0.46154	0.44588	0.97269 (max o	8	7129; 7128; 7115; 70	0.39532277	-0.5053858		17.0
	ZINC04099012	-7.4	412.442	-3.6694	9	1	267.33	0.48165	180.42	5.6501	3039.798880273	0.28033	9.1866	-13.09	0.48148	0.39445	0.94302 (max o	3	2773; 4498; 4939	0.09579354	-0.0295131		17.0
	ZINC02100717	-7.4	355.389	0.3809	6	2	263.68	0.27002	103.27	0.93543	3039.798880273	0.29111	5.1363	1.3084	0.5	0.45418	0.96438 (max o	5	4005; 4462; 4463; 48	0.7184788	0.62379307		16.0
	ZINC72320313	-7.4	353.433	-0.2864	6	2	262.54	0.29161	106.89	-1.0973	3039.798880273	0.30276	5.8036	-0.94598	0.44	0.46819	0.9402 (max of	3	7573; 6915; 5683	0.36576158	-0.7579371		16.0

Structure	Molecule Name	affinity	Total Molweight	cLogP	H-Acceptors	H-Donors	Total Surface Area	Relative PSA	Polar Surface Area	Druglikeness	Kd (nM) @ 20°C	LE from K...	LLE from Kd ...	LELP from Kd (n...	Shape Index	Molecular...	Neighbor Si...	Neig...	Neighbor	Neighbor ...	Neighbor ...	Ring Systems	frequency
	ZINC20762287	-7.4	406.437	-0.6471	10	3	312.66	0.35246	129.56	6.6075	3039.798880273	0.261	6.1643	-2.4794	0.58621	0.56628	0.95872 (max o	6	7426; 7063; 6843; 62	-0.0172992	-0.3284067		15.0
	ZINC20763273	-7.4	362.385	-0.3931	9	2	280.05	0.34672	109.33	5.4347	3039.798880273	0.29111	5.9103	-1.3503	0.61538	0.55293	0.92032 (max o	5	7253; 6843; 6592; 51	-0.0630851	-0.3877515		15.0
	ZINC20763248	-7.4	420.464	-0.1927	10	3	326.42	0.3376	129.56	7.6454	3039.798880273	0.2523	5.7099	-0.76379	0.56667	0.57827	0.97975 (max o	6	7426; 7063; 6843; 51	-8.455809E	-0.3591563		15.0
	ZINC20761038	-7.4	364.357	-1.6791	10	3	276.9	0.39798	129.56	4.3139	3039.798880273	0.29111	7.1963	-5.7679	0.65385	0.57337	0.92702 (max o	9	7426; 7253; 7063; 68	-0.0376165	-0.3383395		15.0
	ZINC13431053	-7.4	445.237	-10.296	15	4	286.85	0.65069	263.59	-43.957	3039.798880273	0.27032	15.813	-38.089	0.60714	0.65134	0.9308 (max of	4	6526; 4674; 5086; 58	-0.6106379	-0.3765459		14.0
	ZINC01891583	-7.4	359.421	0.0341	6	2	271.14	0.26259	103.27	-9.0811	3039.798880273	0.29111	5.4831	0.11714	0.53846	0.49053	0.95257 (max o	3	3192; 4585; 4895	-0.0186439	0.7159403		14.0
	ZINC22922143	-7.4	350.437	-1.8745	7	3	251.12	0.24307	82.01	6.8344	3039.798880273	0.30276	7.3917	-6.1915	0.56	0.45498	0.94338 (max o	4	7067; 6847; 4646; 58	0.41256097	-0.1176005		14.0
	ZINC20463674	-7.4	392.474	-1.5421	8	2	284.78	0.24925	88.08	6.279	3039.798880273	0.27032	7.0593	-5.7048	0.53571	0.46951	0.94883 (max o	9	7384; 7347; 7067; 67	0.41614038	-0.1698136		14.0
	ZINC22920767	-7.4	377.507	-1.6324	7	2	279.59	0.18416	65.02	7.1006	3039.798880273	0.28033	7.1496	-5.8232	0.59259	0.49316	0.93686 (max o	6	7346; 7221; 7065; 68	0.45375732	-0.2183150		14.0
	ZINC08299044	-7.4	351.453	-7.6842	9	4	283.31	0.38784	120.99	0.70912	3039.798880273	0.30276	13.201	-25.381	0.68	0.55685	0.9654 (max of	7	6175; 4158; 4600; 50	0.13576412	-0.2028945		14.0
	ZINC08299050	-7.4	379.507	-5.2617	9	4	324.44	0.34826	97.79	1.9232	3039.798880273	0.28033	10.779	-18.77	0.66667	0.58029	0.96885 (max o	7	7293; 4158; 4600; 50	0.11669306	-0.1952373		14.0
	ZINC12483803	-7.4	367.400	-0.7352	9	4	273.04	0.36815	110.31	1.4376	3039.798880273	0.29111	6.2524	-2.5255	0.65385	0.497	0.95257 (max o	4	7498; 3762; 5051; 50	-0.6771197	-0.3175806		14.0
	ZINC04252537	-7.4	420.413	0.4007	9	6	299.15	0.37319	156.91	-4.8731	3039.798880273	0.2523	5.1165	1.5882	0.5	0.4209	0.95579 (max o	5	2058; 3365; 4101; 41	0.7551557	-0.3011410		13.0
	ZINC08644013	-7.4	428.488	-3.9261	11	4	323.83	0.46373	199.06	4.5702	3039.798880273	0.261	9.4433	-15.043	0.55172	0.61976		0		0.60963744	0.6657195		13.0
	ZINC04082097	-7.4	389.494	-0.5094	7	2	307.11	0.2699	117.18	6.1636	3039.798880273	0.27032	6.0266	-1.8845	0.60714	0.51663	0.97288 (max o	8	6946; 3637; 3638; 45	0.28520367	0.32815826		12.0
	ZINC08643757	-7.4	402.490	-2.8294	9	5	305.61	0.38719	137.78	-5.1183	3039.798880273	0.28033	8.3466	-10.093	0.62963	0.58661	0.94291 (max o	5	6750; 4223; 4655; 50	0.25474867	-0.315308		12.0
	ZINC20464955	-7.4	422.952	-2.0617	8	4	314.38	0.30975	111.92	5.5915	3039.798880273	0.28033	7.5789	-7.3546	0.55556	0.60588	0.97658 (max o	4	7045; 6817; 3847; 59	-0.4141436	-0.1593023		12.0
	ZINC35456543	-7.4	448.419	-2.3095	12	5	302.17	0.46854	181.44	-5.7219	3039.798880273	0.24416	7.8267	-9.4591	0.41935	0.38595	0.95521 (max o	3	3928; 5232; 5233	-0.6234372	-0.6851834		12.0
	ZINC08624495	-7.4	352.386	-0.9452	8	4	266.68	0.35698	117.12	2.1139	3039.798880273	0.30276	6.4624	-3.122	0.68	0.57824	0.9464 (max of	4	6481; 6241; 2568; 46	-0.4040619	-0.2287969		12.0
	ZINC15675169	-7.4	440.447	-1.8861	11	4	331.95	0.38632	152.65	-2.823	3039.798880273	0.24416	7.4033	-7.7249	0.70968	0.58492	0.95267 (max o	4	4613; 5480; 5519; 61	-0.3846433	-0.2248648		12.0
	ZINC35271231	-7.4	434.396	-1.4258	10	8	285	0.44856	188.14	-2.6646	3039.798880273	0.24416	6.943	-5.8397	0.35484	0.35923	0.98656 (max o	8	1044; 1296; 2822; 38	-0.5165202	0.5633765		11.0
	ZINC14438558	-7.4	396.431	-1.2041	10	6	285.68	0.41515	158.3	-17.323	3039.798880273	0.28033	6.7213	-4.2953	0.62963	0.53908	0.95493 (max o	3	4680; 5511; 5880	-0.4132780	0.69921327		11.0
	ZINC03838740	-7.4	402.353	-1.9991	10	7	276.85	0.4626	174.01	1.4918	3039.798880273	0.27032	7.5163	-7.3954	0.57143	0.49162	0.92486 (max o	2	703; 886	-0.3979176	-0.3518535		10.0
	ZINC35454690	-7.4	413.397	-2.9205	10	4	298.4	0.41139	165.81	1.5959	3039.798880273	0.261	8.4377	-11.19	0.58621	0.60027	0.99848 (max o	7	6878; 6641; 1948; 26	-0.1886831	0.7180768		10.0
	ZINC04531089	-7.4	352.386	-0.754	8	4	265.42	0.35868	117.12	-0.49157	3039.798880273	0.30276	6.2712	-2.4905	0.48	0.47801	0.94351 (max o	3	7147; 6966; 6152	-0.1119849	0.8105662		10.0
	ZINC04531086	-7.4	352.386	-0.754	8	4	265.42	0.35868	117.12	-0.49157	3039.798880273	0.30276	6.2712	-2.4905	0.48	0.47801	0.95 (max of 3)	3	7147; 6966; 6153	-0.0953826	0.78781027		10.0
	ZINC04277715	-7.4	377.463	-1.9127	8	2	300.17	0.28407	83.39	5.6681	3039.798880273	0.28033	7.4299	-6.8231	0.62963	0.45371	0.91157 (max o	4	918; 4124; 4555; 613	-0.8058026	-0.2934985		10.0
	ZINC04236191	-7.4	377.439	0.1157	8	1	287.33	0.27035	88.18	5.9964	3039.798880273	0.28033	5.4015	0.41273	0.66667	0.47975	0.88737	1	6149	-0.7864827	-0.2996996		10.0
	ZINC35465974	-7.4	441.451	-2.6414	10	5	318.58	0.39507	176.81	-2.3234	3039.798880273	0.24416	8.1586	-10.818	0.48387	0.44414	0.97642 (max o	3	4840; 4841; 5242	0.21755353	-0.7394030		10.0
	ZINC04097194	-7.4	358.394	-4.462	9	2	245.75	0.51963	182.61	4.8789	3039.798880273	0.31537	9.9792	-14.148	0.58333	0.44564	0.93334	1	6719	-0.3022076	-0.8571721		9.0
	ZINC31169446	-7.4	360.358	-1.2596	9	6	256.4	0.42356	149.07	-4.4264	3039.798880273	0.30276	6.7768	-4.1605	0.52	0.5112	0.98671 (max o	10	6887; 6337; 3882; 38	0.1295502	0.7421609		9.0
	ZINC12285473	-7.4	443.383	-5.0358	10	1	333	0.38468	176.15	-6.2561	3039.798880273	0.23653	10.553	-21.291	0.625	0.62661	0.96877 (max o	8	7170; 7169; 7168; 58	-0.6518001	0.58816487		9.0
	ZINC12285473	-7.4	443.383	-5.0358	10	1	333	0.38468	176.15	-7.6209	3039.798880273	0.23653	10.553	-21.291	0.625	0.62618	0.971 (max of 8	8	7170; 7169; 7168; 62	-0.6318269	0.5964267		9.0
	ZINC12285473	-7.4	442.375	-7.1118	10	0	334.18	0.38686	178.98	-6.6469	3039.798880273	0.23653	12.629	-30.068	0.625	0.62829	0.97743 (max o	8	7170; 7169; 7168; 62	-0.6118901	0.6046016		9.0
	ZINC12419754	-7.4	384.336	-0.9776	10	4	266.69	0.43286	144.14	-5.8493	3039.798880273	0.28033	6.4948	-3.4873	0.44444	0.432	0.94605 (max o	5	6630; 3321; 3704; 49	-0.4565557	0.51432955		8.0
	ZINC35465907	-7.4	364.346	-3.2093	10	7	234.55	0.51887	169.3	-2.3427	3039.798880273	0.30276	8.7265	-10.6	0.44	0.36432	0.98188 (max o	4	1611; 1921; 3078; 31	0.8271375	-0.0021980		8.0
	ZINC05224227	-7.4	382.400	-4.6527	12	6	276.17	0.50534	162.9	2.8692	3039.798880273	0.28033	10.17	-16.597	0.44444	0.411	0.93996 (max o	4	3250; 4237; 4987; 57	-0.6503739	-0.4993174		8.0
	ZINC12529667	-7.4	379.455	-4.3344	8	5	281.37	0.35484	123.06	2.3408	3039.798880273	0.28033	9.8516	-15.462	0.55556	0.46006	0.94883 (max o	4	7043; 3771; 5540; 55	0.5895603	-0.1398410		8.0
	ZINC35455019	-7.4	396.431	-1.2041	10	6	285.68	0.41515	158.3	-17.323	3039.798880273	0.28033	6.7213	-4.2953	0.62963	0.53653	0.9578 (max of	5	1331; 4409; 4680; 48	-0.4601275	0.66350746		8.0
	ZINC13660226	-7.4	355.362	-2.4289	8	4	260.48	0.38283	139.51	-4.6528	3039.798880273	0.30276	7.9461	-8.0227	0.52	0.53909	0.95 (max of 7)	7	7086; 6532; 6237; 62	0.6652554	-0.3577944		8.0
	ZINC15675334	-7.4	397.790	-2.9171	9	4	280.87	0.41418	162.01	6.4594	3039.798880273	0.28033	8.4343	-10.406	0.55556	0.51519	0.93069 (max o	3	6397; 4928; 5113	0.7859078	-0.1960289		8.0
	ZINC03830749	-7.4	376.451	-0.8691	7	1	293.16	0.25478	103.35	1.9037	3039.798880273	0.28033	6.3863	-3.1003	0.51852	0.50029	0.95201	1	5714	-0.8475546	-0.0033390		8.0
	ZINC03873144	-7.4	405.493	-3.2679	8	2	315.18	0.2965	144.82	2.4951	3039.798880273	0.261	8.7851	-12.521	0.48276	0.53089	0.95908 (max o	4	7125; 6941; 6706; 53	-0.8291753	-0.4092653		8.0

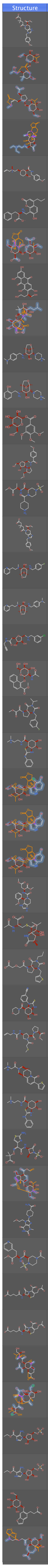

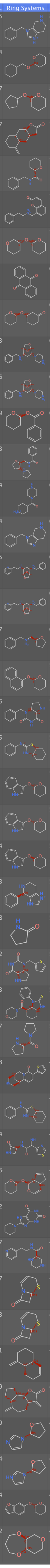

Structure	Molecule Name	affinity	Total Molweight	cLogP	H-Acceptors	H-Donors	Total Surface Area	Relative PSA	Polar Surface Area	Druglikeness	Kd (nM) @ 20°C	LE from K...	LLE from Kd ...	LELP from Kd (n...	Shape Index	Molecular...	Neighbor Si...	Neig...	Neighbor	Neighbor ...	Neighbor ...	Ring Systems	frequency
	ZINC35442245	-7.4	443.498	-0.9645	9	1	328.34	0.25528	115.04	0.64229	3039.798880273	0.23653	6.4817	-4.0778	0.53125	0.58415	0.95243 (max o	7	7613; 7196; 7016; 67	-0.2815196	0.39962575		8.0
	ZINC35455110	-7.4	392.487	-0.0831	8	6	286.85	0.34373	139.84	-10.396	3039.798880273	0.28033	5.6003	-0.29644	0.48148	0.56154	0.99202 (max o	7	7261; 6879; 6642; 66	-0.7121551	0.25133044		8.0
	ZINC31168974	-7.4	373.420	-1.7731	8	4	278.5	0.35806	139.51	-9.7968	3039.798880273	0.29111	7.2903	-6.0908	0.5	0.51929	0.95857 (max o	5	7851; 7247; 6865; 66	0.43718934	0.62093717		8.0
	ZINC22920501	-7.4	350.545	-0.3137	4	2	290.37	0.16658	47.35	-6.4065	3039.798880273	0.30276	5.8309	-1.0362	0.56	0.50037	0.93348	1	6844	-0.3062617	0.87861687		7.0
	ZINC30879307	-7.4	363.476	-0.2324	6	3	308.78	0.24351	72.23	0.11758	3039.798880273	0.29111	5.7496	-0.79832	0.69231	0.60326	0.94326 (max o	2	7641; 4786	-0.3231092	0.4646853		7.0
	ZINC02121312	-7.4	381.427	0.1906	6	2	291.15	0.24455	103.27	-3.3893	3039.798880273	0.27032	5.3266	0.7051	0.53571	0.52092	0.96567 (max o	7	7287; 7119; 6379; 31	-0.0809154	0.6739429		7.0
	ZINC35051186	-7.4	396.300	-8.4533	13	4	261.98	0.59944	207.33	-4.475	3039.798880273	0.28033	13.97	-30.155	0.48148	0.45254	0.9578 (max of	3	7646; 7257; 6873	-0.6205389	0.7399122		7.0
	ZINC06091284	-7.4	360.317	1.4386	8	6	246.9	0.42398	155.52	-5.8339	3039.798880273	0.29111	4.0786	4.9418	0.5	0.3451	0.98284	1	3366	-0.6716535	0.579128		6.0
	ZINC35464790	-7.4	434.524	0.0313	9	6	308.42	0.36197	156.91	-5.985	3039.798880273	0.2523	5.4859	0.12406	0.46667	0.49711	0.97304 (max o	7	6618; 3073; 3505; 35	-0.1549866	0.7808432		6.0
	ZINC22920767	-7.4	378.515	-3.7421	7	3	293.63	0.22317	66.22	3.3482	3039.798880273	0.28033	9.2593	-13.349	0.59259	0.49316	0.91109 (max o	6	7346; 7221; 7064; 68	0.48154116	-0.2426303		6.0
	ZINC22922143	-7.4	351.445	-3.9842	7	4	265.16	0.28315	83.21	2.5103	3039.798880273	0.30276	9.5014	-13.16	0.56	0.45498	0.94338 (max o	4	7404; 7064; 7066; 68	0.5142611	-0.1750866		6.0
	ZINC36374594	-7.4	374.341	-0.9024	10	4	265.95	0.43407	144.14	-4.135	3039.798880273	0.29111	6.4196	-3.0999	0.5	0.41835	0.94975 (max o	5	6788; 5634; 5882; 60	-0.6788624	-0.1382509		6.0
	ZINC20465288	-7.4	395.474	-2.2506	8	4	312.09	0.31494	101.69	1.2568	3039.798880273	0.27032	7.7678	-8.3258	0.57143	0.50487	0.91768 (max o	5	6582; 6583; 4735; 55	0.70259154	0.39617133		6.0
	ZINC05398643	-7.4	405.494	-1.6871	10	2	288.73	0.3602	138.08	2.8203	3039.798880273	0.28033	7.2043	-6.0183	0.48148	0.5535	0.90776 (max o	2	7157; 5420	-0.0929600	0.34817544		6.0
	ZINC35442245	-7.4	442.490	-0.7756	9	0	339.31	0.27936	113.79	1.1757	3039.798880273	0.23653	6.2928	-3.2791	0.53125	0.58415	0.9313 (max of	6	7614; 7195; 7017; 67	-0.2825669	0.35746604		6.0
	ZINC12489936	-7.4	388.461	-0.6051	6	2	285.32	0.19403	71.43	3.0173	3039.798880273	0.27032	6.1223	-2.2385	0.60714	0.4955	0.9399 (max of	8	7850; 7848; 7630; 73	0.664498	0.60511845		6.0
	ZINC12489919	-7.4	413.540	-0.8095	7	2	313.79	0.18774	74.67	4.6755	3039.798880273	0.2523	6.3267	-3.2085	0.6	0.51435	0.92206 (max o	8	7850; 7848; 7630; 73	0.62728703	0.5915727		6.0
	ZINC12603838	-7.4	353.825	-1.5234	6	4	263.68	0.25956	95.4	0.47258	3039.798880273	0.31537	7.0406	-4.8305	0.66667	0.47589	0.92641 (max o	8	7015; 6748; 2154; 32	0.1151248	-0.4420077		6.0
	ZINC70666200	-7.4	405.402	0.4424	9	4	291.15	0.36696	134.55	-4.9901	3039.798880273	0.261	5.0748	1.695	0.37931	0.40962	0.88044	1	5404	0.39666554	0.6113896		5.0
	ZINC04236342	-7.4	391.398	0.8685	8	0	280.07	0.25997	87.23	-3.3563	3039.798880273	0.27032	4.6487	3.2129	0.46429	0.4678	0.89657 (max o	4	2439; 2440; 3663; 45	-0.3573351	0.82320476		5.0
	ZINC20504119	-7.4	379.503	-1.6537	7	5	292.73	0.3715	123.69	4.6034	3039.798880273	0.29111	7.1709	-5.6807	0.57692	0.41484	0.93686 (max o	6	1254; 3010; 5162; 55	0.46723497	0.24841285		5.0
	ZINC02597047	-7.4	408.631	0.6952	7	5	239.64	0.3605	115.17	-5.48	3039.798880273	0.32908	4.822	2.1125	0.47826	0.23946	0.92947 (max o	5	6155; 3613; 5291; 54	-0.4640561	0.6436381		5.0
	ZINC04534184	-7.4	374.186	0.0892	7	5	224.22	0.38529	115.17	-5.5938	3039.798880273	0.34404	5.428	0.25927	0.5	0.24235	0.92913 (max o	5	4685; 4981; 5406; 60	-0.4300874	0.6260766		5.0
	ZINC04534183	-7.4	374.186	0.0892	7	5	224.22	0.38529	115.17	-5.5938	3039.798880273	0.34404	5.428	0.25927	0.5	0.24832	0.92913 (max o	5	4685; 4981; 5291; 54	-0.4656648	0.62264514		5.0
	ZINC32124269	-7.4	417.441	-0.9642	10	3	304.95	0.32176	124.1	-0.11909	3039.798880273	0.2523	6.4814	-3.8217	0.43333	0.51088	0.95318 (max o	7	7424; 4766; 5614; 56	-0.6102457	0.15713713		5.0
	ZINC03873056	-7.4	375.421	-4.4053	9	4	267.35	0.49766	181.16	-2.7923	3039.798880273	0.30276	9.9225	-14.551	0.44	0.65461	0.91255 (max o	4	7678; 7188; 5344; 57	-0.8695771	0.02979288		5.0
	ZINC04260476	-7.4	408.454	-1.867	9	0	295.21	0.36398	138.53	5.8702	3039.798880273	0.27032	7.3842	-6.9067	0.5	0.50259	0.9678 (max of	4	7477; 6963; 5782; 57	-0.1338545	-0.7965812		5.0
	ZINC08636499	-7.4	402.474	-0.0871	8	3	290.99	0.37582	141.15	-0.11054	3039.798880273	0.27032	5.6043	-0.32221	0.46429	0.39948	0.90502 (max o	7	7335; 6479; 6471; 58	0.4551938	0.06832513		5.0
	ZINC03838877	-7.4	412.509	-0.0403	9	2	311.39	0.35219	133.35	2.4046	3039.798880273	0.27032	5.5575	-0.14908	0.53571	0.5698	0.92774 (max o	3	7294; 6700; 4035	-0.0167800	-0.0572327		5.0
	ZINC04237148	-7.4	358.485	-0.2571	5	1	276.88	0.2569	73.3	6.2727	3039.798880273	0.30276	5.7743	-0.8492	0.56	0.4022	0.85824 (max o	2	3660; 4100	-0.8768767	-0.4676198		4.0
	ZINC20504119	-7.4	380.511	-1.5844	7	6	281.22	0.34578	125.3	4.3656	3039.798880273	0.29111	7.1016	-5.4426	0.57692	0.46955	0.92418 (max o	3	1253; 3009; 5574	-0.6860692	0.7051026		4.0
	ZINC19795769	-7.4	433.421	-5.9852	13	2	286.79	0.62548	243.86	5.3236	3039.798880273	0.27032	11.502	-22.141	0.46429	0.48641	0.97533 (max o	3	3768; 3821; 5320	-0.5407226	-0.6367964		4.0
	ZINC35456920	-7.4	404.367	-3.0019	11	6	270.55	0.48656	175.37	-5.2776	3039.798880273	0.27032	8.5191	-11.105	0.46429	0.3832	0.96519 (max o	3	4832; 5637; 6331	-0.1692636	-0.8679656		4.0
	ZINC01838637	-7.4	350.378	-0.8764	10	2	257.63	0.39122	122.79	7.4476	3039.798880273	0.30276	6.3936	-2.8947	0.48	0.45851	0.92342 (max o	2	6493; 5696	-0.0026177	-0.8210782		4.0
	ZINC04147981	-7.4	412.466	-1.0216	10	1	299.23	0.35835	134.36	6.8682	3039.798880273	0.27032	6.5388	-3.7793	0.46429	0.56103	0.88922 (max o	6	7155; 6150; 3219; 32	0.00536360	0.42177227		4.0
	ZINC08927232	-7.4	384.364	-4.7824	10	1	268.54	0.50741	181.27	4.9262	3039.798880273	0.29111	10.3	-16.428	0.61538	0.49233	0.93361 (max o	6	6204; 4497; 4910; 49	0.43341476	0.54485047		4.0
	ZINC08927236	-7.4	384.364	-4.7824	10	1	268.54	0.50741	181.27	4.9262	3039.798880273	0.29111	10.3	-16.428	0.61538	0.49233	0.93361 (max o	4	4911; 5467; 5839; 62	0.46563566	0.53337973		4.0
	ZINC35271218	-7.4	377.411	-0.7314	7	3	282.84	0.317	127.12	-10.534	3039.798880273	0.28033	6.2486	-2.6091	0.48148	0.52687	0.96617 (max o	3	4819; 4820; 5623	0.00639596	-0.9629411		4.0
	ZINC05395465	-7.4	435.299	0.8783	7	3	287.01	0.29748	113.29	-5.5588	3039.798880273	0.27032	4.6389	3.2491	0.42857	0.35175	0.9984 (max of	4	4873; 4874; 5417; 57	0.48870888	-0.2756879		4.0
	ZINC08791848	-7.4	365.234	-7.4348	12	3	245.3	0.60306	202.06	-30.618	3039.798880273	0.31537	12.952	-23.575	0.58333	0.48729	0.93334 (max o	3	6519; 6517; 5070	-0.4581841	-0.4087349		4.0
	ZINC08791848	-7.4	366.242	-7.6237	12	4	234.33	0.58447	203.31	-31.599	3039.798880273	0.31537	13.141	-24.174	0.58333	0.51178	0.93686 (max o	3	6520; 6518; 5071	-0.4649624	-0.4501324		4.0
	ZINC31158287	-7.4	367.329	-2.3622	9	4	258.51	0.44033	152.65	-4.8188	3039.798880273	0.29111	7.8794	-8.1144	0.46154	0.35744	0.90311 (max o	5	7547; 7071; 6855; 51	0.17592223	-0.6375404		4.0
	ZINC31160843	-7.4	389.419	-1.9705	9	2	290.57	0.36673	134.58	-13.144	3039.798880273	0.28033	7.4877	-7.0292	0.55556	0.51898	0.94905 (max o	7	6882; 6860; 6644; 66	-0.6934604	-0.0565592		4.0

Structure	Molecule Name	affinity	Total Molweight	cLogP	H-Acceptors	H-Donors	Total Surface Area	Relative PSA	Polar Surface Area	Druglikeness	Kd (nM) @ 20°C	LE from K...	LLE from Kd ...	LELP from Kd (n...	Shape Index	Molecular...	Neighbor Si...	Neig...	Neighbor	Neighbor ...	Neighbor ...	Ring Systems	frequency
	ZINC35459004	-7.4	410.414	-2.1178	11	6	284.15	0.46328	175.37	-2.7727	3039.798880273	0.27032	7.635	-7.8345	0.5	0.44389	0.9678 (max of	3	6884; 5237; 6008	0.69067353	-0.4827964		4.0
	ZINC03840031	-7.4	353.398	-1.611	8	5	256.58	0.48277	169.99	6.7463	3039.798880273	0.31537	7.1282	-5.1083	0.58333	0.56839	0.95983 (max o	4	4034; 5331; 5333; 57	-0.5324713	-0.2542431		4.0
	ZINC05273537	-7.4	353.354	-4.454	10	3	258.34	0.46923	161.73	9.1102	3039.798880273	0.30276	9.9712	-14.712	0.48	0.5356	0.97118 (max o	7	7152; 6449; 4252; 42	0.03570150	0.8080419		4.0
	ZINC12529667	-7.4	377.439	-1	8	4	271.05	0.33027	117.28	6.4079	3039.798880273	0.28033	6.5172	-3.5672	0.55556	0.48826	0.95461 (max o	5	7217; 7042; 3770; 55	-0.8014586	0.36185855		4.0
	ZINC35780714	-7.4	406.382	-2.7247	11	6	268.21	0.49081	175.37	-4.1099	3039.798880273	0.27032	8.2419	-10.08	0.42857	0.37997	0.95148 (max o	3	5645; 6011; 6342	0.38711232	-0.7188231		4.0
	ZINC35493987	-7.4	406.382	-2.7247	11	6	268.21	0.49081	175.37	-4.1099	3039.798880273	0.27032	8.2419	-10.08	0.42857	0.37997	0.95148 (max o	3	6346; 5645; 6011	0.3752389	-0.7021805		4.0
	ZINC04293780	-7.4	410.338	-0.3298	13	2	285.6	0.51106	164.15	4.6806	3039.798880273	0.261	5.847	-1.2636	0.41379	0.4311	0.93686 (max o	3	7146; 6441; 5787	-0.8841497	-0.0066384		4.0
	ZINC20465616	-7.4	387.479	-3.3053	9	4	262.21	0.31784	124.86	6.1136	3039.798880273	0.29111	8.8225	-11.354	0.53846	0.50041	0.88683 (max o	2	7052; 6267	-0.4358309	0.03086266		4.0
	ZINC13660232	-7.4	355.362	-2.4289	8	4	260.48	0.38283	139.51	-4.6528	3039.798880273	0.30276	7.9461	-8.0227	0.52	0.53225	0.95 (max of 7)	7	7086; 6532; 5632; 56	0.70413256	-0.3452713		4.0
	ZINC13660229	-7.4	355.362	-2.4289	8	4	260.48	0.38283	139.51	-4.6528	3039.798880273	0.30276	7.9461	-8.0227	0.52	0.53277	0.95 (max of 7)	7	7086; 6532; 6237; 56	0.68543977	-0.3357618		4.0
	ZINC35457081	-7.4	389.419	-1.9705	9	2	290.57	0.36673	134.58	-13.144	3039.798880273	0.28033	7.4877	-7.0292	0.55556	0.51898	0.94905 (max o	9	7435; 7262; 6882; 68	-0.7105331	-0.0675729		4.0
	ZINC00803230	-7.4	362.448	-1.6501	7	4	289.84	0.27722	90.37	7.2815	3039.798880273	0.29111	7.1673	-5.6683	0.46154	0.52963	0.97533 (max o	2	7281; 6922	-0.1604030	0.8433433		4.0
	ZINC05273793	-7.4	420.259	-10.349	14	7	261.47	0.67801	251.53	-34.627	3039.798880273	0.28033	15.866	-36.918	0.48148	0.56702	0.95201 (max o	3	7154; 6450; 6160	-0.5390208	-0.7607333		4.0
	ZINC05273791	-7.4	420.259	-10.349	14	7	261.47	0.67801	251.53	-34.627	3039.798880273	0.28033	15.866	-36.918	0.48148	0.56702	0.95201 (max o	3	7154; 6450; 6161	-0.5169340	-0.764078		4.0
	ZINC35270754	-7.4	351.417	0.013601	6	3	277.6	0.30357	113.35	-14.742	3039.798880273	0.30276	5.5036	0.044923	0.6	0.43755	0.93007 (max o	3	7258; 7078; 6623	0.67382383	-0.5859419		4.0
	ZINC08817088	-7.4	353.334	-2.2275	11	4	242.84	0.48056	141.67	2.2599	3039.798880273	0.30276	7.7447	-7.3574	0.44	0.41366	0.92342 (max o	2	3989; 4236	-0.522412	-0.5868111		3.0
	ZINC04029690	-7.4	355.460	-0.1535	6	2	280.62	0.23077	60.85	5.7616	3039.798880273	0.29111	5.6707	-0.52729	0.5	0.39617	0.94302 (max o	5	2216; 2357; 2397; 45	-0.3727665	-0.6648204		3.0
	ZINC04029620	-7.4	383.514	0.6591	6	2	308.14	0.21016	60.85	4.657	3039.798880273	0.27032	4.8581	2.4382	0.5	0.44962	0.94302 (max o	4	2216; 2397; 4516; 61	-0.3645050	-0.6840485		3.0
	ZINC70691919	-7.4	447.435	-2.8655	12	8	306.95	0.4664	190.56	-6.2956	3039.798880273	0.24416	8.3827	-11.736	0.54839	0.44763	0.93405 (max o	5	3744; 4186; 4648; 50	-0.1893135	-0.9462827		3.0
	ZINC04277834	-7.4	441.503	-0.0907	10	1	322.73	0.32925	130.7	6.7268	3039.798880273	0.2523	5.6079	-0.3595	0.5	0.56917	0.89799 (max o	5	7061; 6438; 6439; 54	0.02381792	0.43369022		3.0
	ZINC02130229	-7.4	373.475	0.2876	7	2	309.39	0.24936	67.6	2.44	3039.798880273	0.28033	5.2296	1.0259	0.55556	0.49012	0.90377 (max o	2	7456; 5435	0.76420504	-0.1732256		3.0
	ZINC03830749	-7.4	375.443	-2.1005	7	1	296.88	0.26411	98.77	3.6019	3039.798880273	0.28033	7.6177	-7.493	0.51852	0.61509	0.96343 (max o	2	6696; 5715	-0.5936513	0.64274204		3.0
	ZINC04222473	-7.4	411.497	-0.3532	8	3	295.81	0.40347	160.05	0.30875	3039.798880273	0.27032	5.8704	-1.3066	0.46429	0.41386	0.93686	1	5774	0.83879906	-0.0205581		3.0
	ZINC08643762	-7.4	387.499	-2.6751	9	6	314.28	0.36353	124.36	-2.9551	3039.798880273	0.28033	8.1923	-9.5427	0.66667	0.6202	0.91693 (max o	3	6749; 4227; 5829	0.14528015	-0.4203088		3.0
	ZINC15672912	-7.4	364.488	0.7777	7	1	289.28	0.20115	65.12	2.5968	3039.798880273	0.29111	4.7395	2.6715	0.65385	0.60751	0.95287 (max o	2	7194; 5885	-0.7039749	-0.3649160		3.0
	ZINC20465616	-7.4	387.479	-3.4372	9	4	287.22	0.37724	124.81	5.7354	3039.798880273	0.29111	8.9544	-11.807	0.53846	0.52874	0.95857 (max o	5	7053; 7050; 3392; 47	-0.3433838	-0.5626928		3.0
	ZINC19794585	-7.4	395.366	-3.1593	8	1	289.68	0.32564	130.03	4.1467	3039.798880273	0.261	8.6765	-12.105	0.41379	0.48321	0.91903 (max o	3	7027; 4282; 5946	0.04910931	0.8704944		3.0
	ZINC08298390	-7.4	377.515	-3.4177	8	3	309.91	0.38663	109.82	0.86856	3039.798880273	0.29111	8.9349	-11.74	0.61538	0.47487	0.96721 (max o	4	6984; 6472; 6473; 45	0.0673102	-0.132283		3.0
	ZINC62001272	-7.4	398.890	-3.3514	7	3	292.63	0.35974	135.66	4.9054	3039.798880273	0.29111	8.8686	-11.512	0.5	0.51609	0.93367 (max o	5	7448; 6904; 6667; 30	0.61385334	0.32675856		3.0
	ZINC67902535	-7.4	368.381	0.2665	8	2	256.64	0.35957	111.52	3.4604	3039.798880273	0.29111	5.2507	0.91546	0.38462	0.28721	0.97269 (max o	3	7361; 6669; 1892	0.8062454	0.17359622		3.0
	ZINC68603922	-7.4	371.344	1.0126	8	0	266.16	0.35963	119.76	-10.58	3039.798880273	0.28033	4.5046	3.6122	0.33333	0.3902	0.94605 (max o	2	7661; 7108	-0.9398622	0.00462939		3.0
	ZINC08829785	-7.4	418.397	0.5121	9	5	288.04	0.37682	145.91	-3.7146	3039.798880273	0.2523	5.0051	2.0298	0.53333	0.42714	0.97304 (max o	7	312; 345; 945; 978; 1	0.01546751	-0.3912713		2.0
	ZINC03197509	-7.4	387.319	-0.8425	9	2	262.94	0.40526	134.58	0.9819	3039.798880273	0.27032	6.3597	-3.1167	0.46429	0.2766	0.9543 (max of	3	1098; 1657; 5313	-0.2116896	0.76350075		2.0
	ZINC20503817	-7.4	403.613	-2.3351	6	3	353.63	0.18884	44.46	5.3717	3039.798880273	0.261	7.8523	-8.9469	0.65517	0.63976	0.90502 (max o	2	1888; 5587	-0.7996313	-0.2551383		2.0
	ZINC61992893	-7.4	353.413	0.5349	7	2	251.11	0.30198	96.3	6.0667	3039.798880273	0.30276	4.9823	1.7668	0.36	0.22509	0.99111 (max o	3	6537; 2677; 3092	-0.5557731	0.5176278		2.0
	ZINC08636386	-7.4	405.474	0.8949	8	3	288.56	0.36665	126.59	3.0496	3039.798880273	0.27032	4.6223	3.3106	0.46429	0.40364	0.90617 (max o	6	6988; 6753; 6471; 28	0.46754035	0.10124356		2.0
	ZINC35455098	-7.4	388.368	-1.9969	10	5	262.24	0.45203	155.14	-7.5663	3039.798880273	0.28033	7.5141	-7.1234	0.48148	0.37688	0.94605 (max o	3	3034; 3068; 5631	-0.5822193	0.25515085		2.0
	ZINC04084592	-7.4	359.425	-0.2233	6	0	245.34	0.31789	106.05	8.0296	3039.798880273	0.30276	5.7405	-0.73756	0.44	0.2481	0.92124 (max o	3	949; 3201; 3203	0.38286883	-0.6153747		2.0
	ZINC35455677	-7.4	396.478	0.2159	7	4	273.46	0.31244	116.45	-7.2753	3039.798880273	0.27032	5.3013	0.79869	0.39286	0.29698	0.96519 (max o	3	2631; 3455; 3926	0.72174656	0.30593723		2.0
	ZINC71404494	-7.4	389.332	-5.648	11	6	255.52	0.53193	189.2	-2.7509	3039.798880273	0.28033	11.165	-20.148	0.44444	0.3782	0.94302 (max o	3	6747; 4442; 6371	0.1456025	0.87652904		2.0
	ZINC04026724	-7.4	379.411	-0.2292	6	2	277.36	0.25671	103.27	-2.455	3039.798880273	0.27032	5.7464	-0.84789	0.5	0.46732	0.94573 (max o	5	7287; 7119; 7117; 45	-0.0244373	0.6939863		2.0
	ZINC12484175	-7.4	397.497	-0.9805	7	2	291.03	0.20242	74.67	4.5421	3039.798880273	0.261	6.4977	-3.7568	0.58621	0.43622	0.97415 (max o	6	7213; 6557; 6559; 14	0.39983895	-0.0412183		2.0
	ZINC08636320	-7.4	431.515	-1.0819	9	4	316.4	0.38417	143.97	5.039	3039.798880273	0.2523	6.5991	-4.2882	0.53333	0.42124	0.94251 (max o	6	7333; 7164; 7160; 67	0.48007783	0.13770844		2.0

Structure	Molecule Name	affinity	Total Molweight	cLogP	H-Acceptors	H-Donors	Total Surface Area	Relative PSA	Polar Surface Area	Druglikeness	Kd (nM) @ 20°C	LE from K...	LLE from Kd ...	LELP from Kd (n...	Shape Index	Molecular...	Neighbor Si...	Neig...	Neighbor	Neighbor ...	Neighbor ...	Ring Systems	frequency
	ZINC20462817	-7.4	395.518	-2.6093	7	1	325.5	0.27287	105.01	1.8917	3039.798880273	0.27032	8.1265	-9.6527	0.53571	0.66438	0.93686 (max o	2	4713; 5124	-0.2604364	-0.2883295		2.0
	ZINC12654789	-7.4	413.421	-0.3992	10	5	301.66	0.3976	154.78	-17.14	3039.798880273	0.261	5.9164	-1.5295	0.58621	0.54981	0.95908 (max o	3	3778; 4822; 5222	-0.6914331	0.56923604		2.0
	ZINC08643768	-7.4	411.501	-3.0901	9	6	300.08	0.43858	186.6	6.6976	3039.798880273	0.27032	8.6073	-11.431	0.53571	0.52457	0.93975 (max o	6	3603; 3604; 4540; 49	-0.5315867	-0.1168701		2.0
	ZINC03881686	-7.4	369.476	-0.9482	6	3	267.09	0.28687	110.05	-6.0776	3039.798880273	0.29111	6.4654	-3.2572	0.42308	0.46464	0.93686 (max o	3	6407; 4961; 5368	-0.8448515	-0.3391831		2.0
	ZINC13523533	-7.4	362.389	-2.3544	10	4	267.39	0.44452	150.28	9.5534	3039.798880273	0.29111	7.8716	-8.0876	0.46154	0.49258	0.96998 (max o	8	6527; 6233; 6232; 42	0.04435366	0.78129804		2.0
	ZINC13523533	-7.4	362.389	-2.3544	10	4	267.39	0.44452	150.28	9.5534	3039.798880273	0.29111	7.8716	-8.0876	0.46154	0.49258	0.96998 (max o	8	6528; 6233; 6232; 42	0.05498763	0.79832846		2.0
	ZINC13523533	-7.4	363.397	-4.3322	10	5	256.42	0.42076	151.53	8.9317	3039.798880273	0.29111	9.8494	-14.882	0.46154	0.48582	0.9615 (max of	8	6529; 4254; 4255; 62	0.02048380	0.81960946		2.0
	ZINC13523533	-7.4	363.397	-4.3322	10	5	256.42	0.42076	151.53	9.0745	3039.798880273	0.29111	9.8494	-14.882	0.46154	0.49258	0.96998 (max o	8	6530; 4254; 4255; 53	0.01201250	0.80301046		2.0
	ZINC04270804	-7.4	362.385	-0.0601	9	0	270.25	0.29876	92.28	1.7426	3039.798880273	0.29111	5.5773	-0.20645	0.53846	0.59124	0.93686 (max o	2	4554; 5398	-0.2896672	0.92977566		2.0
	ZINC09008170	-7.4	421.381	-2.168	11	2	319.56	0.41767	156.23	-3.591	3039.798880273	0.2523	7.6852	-8.5931	0.63333	0.56293	0.90725	1	5470	0.61296487	-0.6289313		2.0
	ZINC04064574	-7.4	429.559	-1.5591	7	3	352.78	0.22629	77.84	1.5415	3039.798880273	0.24416	7.0763	-6.3856	0.48387	0.41902	0.85572	1	5491	-0.7165437	-0.6912061		2.0
	ZINC05397915	-7.4	406.437	-0.0856	10	2	311.1	0.32845	117.28	1.6343	3039.798880273	0.261	5.6028	-0.32797	0.51724	0.57346	0.93686 (max o	3	3683; 3684; 5801	0.08349042	0.40829688		2.0
	ZINC31156445	-7.4	359.350	-3.6225	9	4	244.43	0.44888	148.74	-3.4303	3039.798880273	0.30276	9.1397	-11.965	0.48	0.37141	0.94678 (max o	6	1700; 3035; 3069; 34	-0.6574423	0.12514095		2.0
	ZINC35442507	-7.4	381.539	-1.2092	7	2	309.45	0.23341	66.32	1.7425	3039.798880273	0.28033	6.7264	-4.3135	0.55556	0.53418	0.92442 (max o	7	4340; 4341; 4342; 47	-0.0715031	0.5458893		2.0
	ZINC59585888	-7.4	362.373	-1.8885	9	5	259.34	0.41852	145.91	-5.845	3039.798880273	0.30276	7.4057	-6.2377	0.48	0.59862	0.9594 (max of	3	6672; 6673; 6044	-0.9426835	0.1175012		2.0
	ZINC00901160	-7.4	359.309	-0.6258	8	4	264.21	0.38893	147.35	-1.3192	3039.798880273	0.29111	6.143	-2.1497	0.61538	0.42876	0.9615 (max of	7	1894; 2743; 2952; 37	0.03526541	-0.8828351		2.0
	ZINC00899870	-7.4	359.309	-0.6258	8	4	264.21	0.38893	147.35	-1.3192	3039.798880273	0.29111	6.143	-2.1497	0.61538	0.44489	0.9615 (max of	7	1894; 2743; 2952; 37	0.0365708	-0.8643508		2.0
	ZINC08480999	-7.4	368.496	-2.1877	7	1	288.7	0.24489	94.56	1.9217	3039.798880273	0.29111	7.7049	-7.515	0.65385	0.56033	0.96998	1	6079	0.76196766	-0.4342396		2.0
	ZINC02138958	-7.4	368.496	-2.1877	7	1	288.7	0.24489	94.56	1.9217	3039.798880273	0.29111	7.7049	-7.515	0.65385	0.56033	0.96998	1	6176	0.7796688	-0.4428296		2.0
	ZINC03839626	-7.4	351.402	-1.1705	8	1	249.65	0.31116	88.18	7.0652	3039.798880273	0.30276	6.6877	-3.8662	0.6	0.30203	0.97118 (max o	4	6702; 6701; 2378; 49	-0.3642622	0.6933293		2.0
	ZINC03998978	-7.4	369.373	-1.6083	10	7	265.82	0.46219	160.38	-0.55001	3039.798880273	0.29111	7.1255	-5.5247	0.5	0.41595	0.94951 (max o	3	7351; 7180; 7127	0.54716104	-0.5569004		2.0
	ZINC04268202	-7.4	368.468	0.3765	6	5	245.81	0.31952	118.22	-1.3382	3039.798880273	0.29111	5.1407	1.2933	0.34615	0.3622	0.84495	1	6728	0.91292834	-0.1543970		2.0
	ZINC08551995	-7.4	425.996	-1.1969	7	5	318.59	0.34176	128.76	0.91283	3039.798880273	0.28033	6.7141	-4.2696	0.51852	0.46665	0.91768 (max o	3	7163; 6985; 5817	0.7264997	-0.2086353		2.0
	ZINC08740368	-7.4	352.350	-1.7011	11	4	243.01	0.48517	152.01	0.0425	3039.798880273	0.30276	7.2183	-5.6187	0.44	0.37483	0.92662 (max o	4	6990; 6989; 6758; 37	-0.8698888	0.10702185		2.0
	ZINC08740485	-7.4	386.371	0.8103	12	5	275.91	0.50121	176.82	1.3234	3039.798880273	0.27032	4.7069	2.9976	0.5	0.33184	0.98451 (max o	12	6759; 6484; 3313; 41	-0.1872250	-0.6458182		2.0
	ZINC08740524	-7.4	375.344	0.9999	12	5	266.84	0.53002	177.07	1.5916	3039.798880273	0.28033	4.5173	3.5669	0.48148	0.30941	0.96885 (max o	5	7338; 6483; 6482; 50	-0.3383079	0.2776202		2.0
	ZINC12481267	-7.4	404.485	-1.1471	8	1	316.21	0.24958	94.17	4.2362	3039.798880273	0.261	6.6643	-4.3951	0.58621	0.59426	0.95642 (max o	4	4209; 4210; 5846; 62	-0.1673363	-0.1400521		2.0
	ZINC12481264	-7.4	374.459	-1.0771	7	1	293.95	0.23446	84.94	4.1794	3039.798880273	0.28033	6.5943	-3.8423	0.55556	0.58418	0.95201 (max o	4	6764; 4209; 4643; 62	-0.1900016	-0.1423107		2.0
	ZINC20463032	-7.4	368.476	-2.416	8	3	300.26	0.33727	120	5.8352	3039.798880273	0.29111	7.9332	-8.2992	0.5	0.603	0.88178 (max o	4	6803; 6552; 5907; 62	-0.1168134	-0.3501253		2.0
	ZINC20463821	-7.4	391.510	-2.907	7	3	295.33	0.24373	72.21	2.9523	3039.798880273	0.27032	8.4242	-10.754	0.53571	0.46544	0.90776 (max o	6	7212; 7040; 4726; 47	-0.6141915	-0.6288261		2.0
	ZINC20463821	-7.4	390.502	-0.7973	7	2	281.29	0.20598	71.01	5.1707	3039.798880273	0.27032	6.3145	-2.9495	0.53571	0.46031	0.90776 (max o	10	7213; 7041; 4725; 47	0.36030984	-0.0130128		2.0
	ZINC20465192	-7.4	371.907	-3.4202	6	4	285.34	0.22808	73.98	1.9951	3039.798880273	0.30276	8.9374	-11.297	0.56	0.45789	0.91964 (max o	4	7631; 6575; 6568; 62	-0.4141850	0.27863857		2.0
	ZINC20465192	-7.4	370.899	-1.3105	6	3	271.3	0.18813	72.78	6.2625	3039.798880273	0.30276	6.8277	-4.3286	0.56	0.45789	0.91964 (max o	7	7631; 6576; 6568; 18	-0.4148571	0.25661084		2.0
	ZINC20465794	-7.4	366.480	-1.1551	7	5	301.79	0.29739	95.26	3.2503	3039.798880273	0.29111	6.6723	-3.9679	0.57692	0.60896	0.93355 (max o	5	7400; 7054; 6831; 38	0.30249846	-0.2015168		2.0
	ZINC31157982	-7.4	364.393	1.4512	7	2	267.37	0.31911	110.13	-16.562	3039.798880273	0.29111	4.066	4.985	0.42308	0.31382	0.97791 (max o	3	6611; 3896; 5601	-0.7766052	0.20519279		2.0
	ZINC31168492	-7.4	374.472	0.91	7	5	276.92	0.30875	119.61	-10.488	3039.798880273	0.29111	4.6072	3.126	0.53846	0.49076	0.99159 (max o	7	6620; 3526; 4843; 52	0.72548187	0.37113667		2.0
	ZINC35465762	-7.4	416.421	-1.0892	10	6	306.66	0.38675	158.3	-4.1006	3039.798880273	0.261	6.6064	-4.1732	0.58621	0.54786	0.98727 (max o	3	6887; 5982; 6301	0.1424428	0.75812596		2.0
	ZINC53281361	-7.4	433.504	-3.1318	8	4	317.17	0.36268	161.4	-7.2057	3039.798880273	0.2523	8.649	-12.413	0.43333	0.57251	0.95837 (max o	4	7096; 3513; 6343; 63	-0.6628594	-0.1536565		2.0
	ZINC35614467	-7.4	434.511	-4.3565	8	4	313.45	0.35511	165.98	2.3821	3039.798880273	0.2523	9.8737	-17.267	0.43333	0.59693	0.95837 (max o	3	7094; 7095; 3512	-0.5063247	-0.8161275		2.0
	ZINC70672630	-7.4	449.590	-15.151	11	8	314.94	0.43103	213.93	1.7104	3039.798880273	0.24416	20.668	-62.053	0.48387	0.434	0.94488	1	6909	-0.8595069	0.15138613		2.0
	ZINC71404492	-7.4	389.332	-5.648	11	6	255.52	0.53193	189.2	-2.7509	3039.798880273	0.28033	11.165	-20.148	0.44444	0.3782	0.94302 (max o	3	6747; 6372; 4442	0.14415798	0.8950827		2.0
	ZINC02099586	-7.4	357.413	1.47	8	2	274.71	0.27513	90.7	3.2531	3039.798880273	0.29111	4.0472	5.0496	0.5	0.48199	0.84869 (max o	2	7575; 7284	-0.0052183	-0.8598263		1.0
	ZINC02130345	-7.4	425.396	-2.744	10	3	319.2	0.39032	155.64	3.2084	3039.798880273	0.24416	8.2612	-11.239	0.51613	0.48317	0.85405	1	1265	0.04519569	-0.3008422		1.0

Structure	Molecule Name	affinity	Total Molweight	cLogP	H-Acceptors	H-Donors	Total Surface Area	Relative PSA	Polar Surface Area	Druglikeness	Kd (nM) @ 20°C	LE from K...	LLE from Kd ...	LELP from Kd (n...	Shape Index	Molecular...	Neighbor Si...	Neig...	Neighbor	Neighbor ...	Neighbor ...	Ring Systems	frequency
	ZINC03151496	-7.4	359.454	-1.6454	8	2	234.92	0.6263	204.73	6.1742	3039.798880273	0.34404	7.1626	-4.7826	0.59091	0.50467		0		-0.9895748	-0.0347619		1.0
	ZINC03831069	-7.4	379.412	-0.6766	8	1	263.24	0.40229	133.3	10.411	3039.798880273	0.29111	6.1938	-2.3242	0.46154	0.35201	0.91351	1	6932	-0.5164894	-0.7292827		1.0
	ZINC03927825	-7.4	382.435	-2.6731	9	2	297.74	0.37133	131.17	8.9795	3039.798880273	0.28033	8.1903	-9.5356	0.55556	0.56352	0.93686	1	6461	-0.9439227	-0.1845529		1.0
	ZINC03978483	-7.4	412.521	0.8159	7	5	276.15	0.32062	127.45	0.39745	3039.798880273	0.261	4.7013	3.1261	0.37931	0.2647	0.88773	1	6110	0.22781417	-0.8306926		1.0
	ZINC03978484	-7.4	412.521	0.8159	7	5	276.15	0.32062	127.45	0.39745	3039.798880273	0.261	4.7013	3.1261	0.37931	0.2647	0.88773	1	6109	0.20936257	-0.8209160		1.0
	ZINC04029811	-7.4	389.407	1.247	9	1	281	0.29491	99.62	4.6354	3039.798880273	0.27032	4.2702	4.6131	0.42857	0.38093	0.93385	1	4509	0.5797549	-0.6736185		1.0
	ZINC04045928	-7.4	378.380	-0.297	9	1	278.09	0.33849	103.4	2.4045	3039.798880273	0.28033	5.8142	-1.0595	0.55556	0.45082	0.9144	1	7306	-0.0749875	0.98910993		1.0
	ZINC04221686	-7.4	427.463	-1.8449	11	3	322.28	0.38076	152.31	5.7973	3039.798880273	0.24416	7.3621	-7.5562	0.45161	0.40544	0.90825 (max o	2	5388; 5772	0.71501654	0.4123614		1.0
	ZINC04228300	-7.4	420.259	-10.197	14	7	262.3	0.67587	251.53	-27.673	3039.798880273	0.28033	15.714	-36.375	0.48148	0.51759		0		0.92634815	-0.1289555		1.0
	ZINC04236319	-7.4	386.427	-1.8037	8	1	280.85	0.30265	109.85	-2.169	3039.798880273	0.27032	7.3209	-6.6725	0.57143	0.41017	0.90617 (max o	2	139; 574	-0.4467437	-0.5430155		1.0
	ZINC04236334	-7.4	358.366	-2.8328	9	1	264.73	0.3961	123.22	-0.89286	3039.798880273	0.30276	8.35	-9.3567	0.64	0.52871		0		0.8721858	-0.2413522		1.0
	ZINC04270838	-7.4	397.451	0.3093	9	0	282.32	0.30217	104.84	-4.6515	3039.798880273	0.28033	5.2079	1.1033	0.51852	0.57679	0.86201 (max o	2	7692; 5786	0.23211917	0.3638733		1.0
	ZINC04899895	-7.4	391.490	-2.0469	8	4	306.51	0.32149	142.58	0.063191	3039.798880273	0.27032	7.5641	-7.5722	0.5	0.50243		0		-0.9015149	-0.3165658		1.0
	ZINC06092746	-7.4	408.358	-1.0069	10	8	274.33	0.46601	188.14	-3.0467	3039.798880273	0.261	6.5241	-3.8579	0.48276	0.48334		0		-0.6893489	-0.6905125		1.0
	ZINC08076268	-7.4	365.389	-2.974	10	5	275.23	0.4215	175.11	2.431	3039.798880273	0.29111	8.4912	-10.216	0.65385	0.52693		0		0.5391714	0.8398587		1.0
	ZINC08254768	-7.4	443.527	-0.7539	9	3	317	0.35849	135.18	6.2103	3039.798880273	0.24416	6.2711	-3.0878	0.48387	0.40687	0.9112 (max of	5	6479; 6184; 6181; 50	0.4865863	0.09448663		1.0
	ZINC08643718	-7.4	356.401	-4.3095	10	3	260.05	0.382	126.55	-1.9602	3039.798880273	0.30276	9.8267	-14.234	0.64	0.55584	0.91999 (max o	3	6271; 2888; 6186	-0.1289794	0.91774917		1.0
	ZINC08643721	-7.4	368.456	-2.8762	9	3	274.81	0.3251	117.32	-1.3595	3039.798880273	0.29111	8.3934	-9.8801	0.65385	0.55708	0.91999 (max o	2	2888; 6185	-0.1065390	0.9189278		1.0
	ZINC08765353	-7.4	375.400	-1.2181	8	1	280.29	0.31542	108	1.5168	3039.798880273	0.28033	6.7353	-4.3453	0.55556	0.58862		0		0.5518579	0.8057355		1.0
	ZINC08964842	-7.4	402.390	0.849	12	7	271.99	0.47395	185.77	-0.63587	3039.798880273	0.261	4.6682	3.2529	0.48276	0.31912	0.93101	1	2907	0.03199810	0.90246356		1.0
	ZINC12480799	-7.4	424.543	0.0746	7	2	354.75	0.23659	89.8	4.3377	3039.798880273	0.24416	5.4426	0.30554	0.58065	0.59358	0.96018 (max o	2	2144; 5046	-0.1982587	-0.1601828		1.0
	ZINC12489936	-7.4	388.461	-0.7467	6	3	303.08	0.24126	68.05	-0.25606	3039.798880273	0.27032	6.2639	-2.7623	0.60714	0.52451	0.91292	1	2524	0.3522442	0.04384665		1.0
	ZINC12529667	-7.4	378.447	-3.1097	8	5	285.09	0.36325	118.48	3.4355	3039.798880273	0.28033	8.6269	-11.093	0.55556	0.49181	0.87919 (max o	2	7387; 3772	0.5483675	-0.2766581		1.0
	ZINC15708259	-7.4	396.534	-2.4962	8	4	308.09	0.3882	119.23	3.7757	3039.798880273	0.28033	8.0134	-8.9045	0.59259	0.53886	0.91482 (max o	6	7364; 7363; 7020; 46	-0.2012081	0.0462479		1.0
	ZINC15708458	-7.4	364.492	-2.7045	7	5	295.28	0.39796	118.79	2.3567	3039.798880273	0.30276	8.2217	-8.933	0.64	0.53788	0.90599 (max o	4	7364; 7365; 5116; 58	-0.2381228	0.07257454		1.0
	ZINC20462913	-7.4	381.902	-0.3055	6	1	311.21	0.23287	77.77	3.0217	3039.798880273	0.29111	5.8227	-1.0494	0.5	0.63748	0.91007 (max o	5	6602; 6603; 6496; 42	0.00170624	-0.1573925		1.0
	ZINC20463010	-7.4	379.499	-1.9085	7	2	305.82	0.29367	107.97	3.2751	3039.798880273	0.28033	7.4257	-6.8081	0.48148	0.50932	0.92721 (max o	4	6803; 6552; 6252; 59	-0.0918311	-0.3224297		1.0
	ZINC20464681	-7.4	449.516	-0.2813	6	3	346.14	0.19455	66.6	-0.054125	3039.798880273	0.23653	5.7985	-1.1893	0.4375	0.48542	0.93948	1	4733	0.513829	-0.6764502		1.0
	ZINC20464818	-7.4	357.856	-1.4199	6	3	258.74	0.22219	78.77	1.3672	3039.798880273	0.31537	6.9371	-4.5023	0.54167	0.45795	0.90399	1	6568	-0.3774918	0.25690407		1.0
	ZINC20465158	-7.4	370.472	-0.8311	8	4	270.99	0.36762	125.54	8.3458	3039.798880273	0.30276	6.3483	-2.7451	0.56	0.52698	0.89839 (max o	3	7633; 2999; 6261	-0.4157209	0.19280462		1.0
	ZINC20465158	-7.4	371.480	-2.9408	8	5	285.03	0.39877	126.74	4.5692	3039.798880273	0.30276	8.458	-9.7135	0.56	0.52698	0.89805 (max o	3	7634; 7046; 6260	-0.4358907	0.20454586		1.0
	ZINC20465622	-7.4	449.550	-2.0549	9	4	309.71	0.26909	124.86	-1.6476	3039.798880273	0.24416	7.5721	-8.4163	0.54839	0.47756	0.89979 (max o	5	2236; 3404; 3844; 55	-0.4170358	-0.0027760		1.0
	ZINC20465821	-7.4	379.543	-3.315	6	5	326.2	0.25395	70.6	1.3058	3039.798880273	0.28033	8.8322	-11.825	0.59259	0.55788	0.91768 (max o	3	6828; 5146; 5158	0.31141064	0.00808367		1.0
	ZINC20504101	-7.4	399.470	-4.8762	11	4	299.03	0.37053	138.58	-1.0163	3039.798880273	0.27032	10.393	-18.039	0.67857	0.58807	0.89688	1	6185	-0.1510027	0.9137008		1.0
	ZINC22923418	-7.4	377.531	-4.6709	7	4	326.33	0.26884	70.32	4.5725	3039.798880273	0.28033	10.188	-16.662	0.62963	0.49431	0.89169 (max o	4	6640; 2615; 3021; 38	-0.1715315	0.608617		1.0
	ZINC30690463	-7.4	388.467	-2.3099	7	1	262.95	0.34018	118.08	9.6591	3039.798880273	0.28033	7.8271	-8.24	0.48148	0.38375	0.93373 (max o	2	6305; 3829	0.45332202	0.84398454		1.0
	ZINC30727127	-7.4	381.423	0.089	8	1	281.42	0.33196	105.67	8.9299	3039.798880273	0.28033	5.4282	0.31748	0.40741	0.31867	0.9144 (max of	2	818; 3858	-0.7618509	0.45794162		1.0
	ZINC30878899	-7.4	357.449	-0.8468	8	2	280.03	0.27761	91.34	8.1589	3039.798880273	0.30276	6.364	-2.797	0.68	0.61406	0.89162 (max o	3	7931; 2620; 6288	-0.3151267	0.33101264		1.0
	ZINC30878899	-7.4	358.457	-2.9565	8	3	294.07	0.3121	92.54	3.9071	3039.798880273	0.30276	8.4737	-9.7653	0.68	0.60898	0.89524 (max o	3	7932; 2621; 6287	-0.3380894	0.3647082		1.0
	ZINC31156306	-7.4	390.383	-1.942	10	5	263.42	0.45	155.14	-4.365	3039.798880273	0.28033	7.4592	-6.9276	0.48148	0.37688	0.93686 (max o	2	2627; 5966	-0.6686033	0.01536915		1.0
	ZINC31539258	-7.4	388.467	-2.3099	7	1	262.95	0.34018	118.08	9.6591	3039.798880273	0.28033	7.8271	-8.24	0.48148	0.38514	0.93373 (max o	2	3829; 6285	0.46137992	0.85667723		1.0
	ZINC32109690	-7.4	358.434	-0.9102	7	2	244.47	0.32487	107.05	-14.377	3039.798880273	0.31537	6.4274	-2.8861	0.54167	0.45793	0.95053	1	5217	-0.2650752	0.85131085		1.0
	ZINC35442506	-7.4	366.504	-4.2393	7	3	302.82	0.28489	67.52	4.1791	3039.798880273	0.29111	9.7565	-14.563	0.57692	0.50371	0.85165	1	2282	0.7163337	0.19647123		1.0

Structure	Molecule Name	affinity	Total Molweight	cLogP	H-Acceptors	H-Donors	Total Surface Area	Relative PSA	Polar Surface Area	Druglikeness	Kd (nM) @ 20°C	LE from K...	LLE from Kd ...	LELP from Kd (n...	Shape Index	Molecular...	Neighbor Si...	Neig...	Neighbor	Neighbor ...	Neighbor ...	Ring Systems	frequency
	ZINC35442506	-7.4	365.496	-2.1296	7	2	288.78	0.25012	66.32	5.0823	3039.798880273	0.29111	7.6468	-7.3154	0.57692	0.48735	0.89253 (max c	2	2281; 3486	0.7706426	0.1791886		1.0
	ZINC35447004	-7.4	370.497	-0.7021	7	3	274.24	0.43287	158.07	9.8975	3039.798880273	0.31537	6.2193	-2.2263	0.58333	0.59049		0		-0.8641208	-0.0154058		1.0
	ZINC35454635	-7.4	437.463	-0.941	9	3	323.23	0.34867	153.42	-0.45976	3039.798880273	0.24416	6.4582	-3.8541	0.48387	0.48961	0.95521 (max c	3	6877; 4405; 5627	-0.9036199	-0.1195291		1.0
	ZINC35456913	-7.4	404.367	-3.0019	11	6	270.55	0.48656	175.37	-5.2776	3039.798880273	0.27032	8.5191	-11.105	0.46429	0.3832	0.96519 (max c	3	6332; 4832; 5637	-0.1918026	-0.8670158		1.0
	ZINC35459014	-7.4	388.368	-1.5759	10	4	277.62	0.42677	151.98	-7.1253	3039.798880273	0.28033	7.0931	-5.6216	0.44444	0.45564	0.96617 (max c	3	7439; 4415; 4837	0.840596	-0.317411		1.0
	ZINC35614467	-7.4	434.511	-1.1599	8	4	316.97	0.35899	155.41	-7.2548	3039.798880273	0.2523	6.6771	-4.5974	0.43333	0.57119	0.94785 (max c	4	7093; 6676; 6359; 35	-0.658428	-0.1095068		1.0
	ZINC35614467	-7.4	433.504	-3.1318	8	4	317.17	0.36268	161.4	-7.2057	3039.798880273	0.2523	8.649	-12.413	0.43333	0.57251	0.95837 (max c	3	7096; 6359; 3513	-0.6811536	-0.1604139		1.0
	ZINC38987088	-7.4	360.358	-2.5739	9	5	226.29	0.47965	145.91	-2.3592	3039.798880273	0.30276	8.0911	-8.5016	0.44	0.45853		0		-0.4550017	0.8823591		1.0
	ZINC68604226	-7.4	354.413	-0.9002	8	4	270.5	0.26226	111.92	0.13595	3039.798880273	0.29111	6.4174	-3.0923	0.76923	0.55838	0.87452	1	6366	-0.7934226	-0.3988860		1.0
	ZINC68604226	-7.4	353.405	-0.6763	8	3	282.01	0.29237	110.31	2.2577	3039.798880273	0.29111	6.1935	-2.3232	0.76923	0.51393	0.87452	1	6365	-0.76256	-0.4019640		1.0
	ZINC49600147	-7.4	369.476	0.8703	6	4	297.04	0.26838	121.05	1.0099	3039.798880273	0.29111	4.6469	2.9896	0.57692	0.68415	0.97791 (max c	3	4853; 6039; 6356	0.297751	-0.7661712		0.0
	ZINC49600144	-7.4	369.476	0.8703	6	4	297.04	0.26838	121.05	1.0099	3039.798880273	0.29111	4.6469	2.9896	0.57692	0.69138	0.98284 (max c	3	4853; 6039; 6357	0.30806467	-0.7483657		0.0
	ZINC68604309	-7.4	418.672	-8.8747	9	8	345.98	0.25088	123.79	0.38518	3039.798880273	0.261	14.392	-34.003	0.58621	0.4187		0		-0.6120736	0.78362644		0.0
	ZINC02118861	-7.3	433.436	-1.8227	10	2	327.95	0.36396	143.09	4.8467	3609.116734805	0.24086	7.2653	-7.5675	0.54839	0.59479	0.95983 (max c	7	2720; 2726; 3566; 44	0.10187951	0.03592437		172.0
	ZINC02154311	-7.3	375.356	-2.3303	9	2	283.69	0.38549	133.86	0.18211	3609.116734805	0.27654	7.7729	-8.4266	0.66667	0.57987	0.96106 (max c	6	7457; 1646; 2362; 27	0.12012393	0.09068496		172.0
	ZINC02121164	-7.3	362.357	-1.0296	8	1	276.74	0.34278	113.99	1.0104	3609.116734805	0.28718	6.4722	-3.5852	0.61538	0.53048	0.91768 (max c	7	7120; 4467; 4474; 53	0.07243651	0.10448066		172.0
	ZINC02120917	-7.3	378.424	-1.016	7	1	281.47	0.36363	130.06	3.977	3609.116734805	0.28718	6.4586	-3.5379	0.57692	0.53026	0.92064 (max c	3	6925; 6690; 6689	0.04264016	0.1494426		172.0
	ZINC61991208	-7.3	434.539	-4.0587	10	6	332.34	0.35256	187.99	1.0827	3609.116734805	0.24086	9.5013	-16.851	0.58065	0.62325	0.93686 (max c	3	1587; 5746; 6041	-0.0861457	-0.4169766		134.0
	ZINC12658274	-7.3	427.524	-1.7887	10	5	306.89	0.38268	187.42	-6.0696	3609.116734805	0.25747	7.2313	-6.9473	0.58621	0.59918	0.95642 (max c	4	3996; 4234; 4894; 61	0.5824327	-0.3213967		134.0
	ZINC02561182	-7.3	350.350	-2.7841	9	3	273.21	0.40562	136.66	-10.368	3609.116734805	0.29866	8.2267	-9.3219	0.68	0.59429	0.88178 (max c	2	4560; 6080	-0.0322542	-0.4709701		134.0
	ZINC04025663	-7.3	378.404	-1.9604	9	3	296.53	0.38657	150.65	-9.7358	3609.116734805	0.27654	7.403	-7.089	0.51852	0.55389	0.96617 (max c	5	4502; 4503; 5744; 60	-0.0148371	-0.5032019		134.0
	ZINC04025661	-7.3	378.404	-1.9604	9	3	296.53	0.38657	150.65	-9.7358	3609.116734805	0.27654	7.403	-7.089	0.51852	0.55421	0.96617 (max c	5	4502; 4503; 5744; 60	-0.0355681	-0.5100542		134.0
	ZINC04545849	-7.3	392.430	-4.3105	9	3	305.77	0.3863	166.1	2.1659	3609.116734805	0.26666	9.7531	-16.165	0.57143	0.59158	0.96253 (max c	4	6730; 6448; 5409; 64	-0.0049773	0.6052794		134.0
	ZINC04545848	-7.3	392.430	-4.3105	9	3	305.77	0.3863	166.1	2.1659	3609.116734805	0.26666	9.7531	-16.165	0.57143	0.59797	0.96253 (max c	6	7430; 6730; 6448; 54	0.01603000	0.6183499		134.0
	ZINC02504773	-7.3	363.456	-1.1943	7	3	289.99	0.31311	125.97	3.2028	3609.116734805	0.28718	6.6369	-4.1588	0.53846	0.57663	0.94951 (max c	5	6872; 6730; 6448; 60	-0.0023970	0.62417734		134.0
	ZINC04899500	-7.3	392.430	-4.3105	9	3	305.77	0.3863	166.1	2.1659	3609.116734805	0.26666	9.7531	-16.165	0.57143	0.59156	0.94862 (max c	5	6872; 6730; 6391; 64	0.00153574	0.58516794		134.0
	ZINC08764405	-7.3	355.821	-1.4111	7	3	269.65	0.33673	125.97	1.9311	3609.116734805	0.31111	6.8537	-4.5357	0.5	0.56008	0.93686 (max c	4	7653; 7166; 6024; 61	0.65550107	0.37219417		134.0
	ZINC49181835	-7.3	368.361	-2.7364	9	4	275.87	0.41422	159.71	0.18103	3609.116734805	0.28718	8.179	-9.5286	0.53846	0.63489	0.98517 (max c	4	7655; 7270; 7239; 63	-0.3715902	0.7425961		134.0
	ZINC02516170	-7.3	379.455	-1.54	8	4	296.34	0.35061	146.2	3.2312	3609.116734805	0.27654	6.9826	-5.5688	0.44444	0.58067		0		0.6117516	-0.5537834		134.0
	ZINC12888018	-7.3	369.504	-0.9277	7	3	298.03	0.30467	125.97	3.0831	3609.116734805	0.28718	6.3703	-3.2304	0.61538	0.60594	0.96721 (max c	9	7138; 6776; 6746; 49	0.50451165	0.03197153		127.0
	ZINC04090197	-7.3	387.543	-1.4512	7	3	304.52	0.35561	151.27	2.4375	3609.116734805	0.28718	6.8938	-5.0533	0.65385	0.59236	0.96721 (max c	7	7608; 7312; 7136; 71	0.56581825	0.0761462		127.0
	ZINC31170290	-7.3	421.464	0.5332	8	2	326.7	0.30487	133.19	-4.2966	3609.116734805	0.24889	4.9094	2.1423	0.5	0.60057	0.96343 (max c	7	7555; 7433; 7259; 72	0.1077156	0.7452244		127.0
	ZINC31170281	-7.3	421.464	0.5332	8	2	326.7	0.30487	133.19	-4.2966	3609.116734805	0.24889	4.9094	2.1423	0.5	0.60057	0.96343 (max c	7	7555; 7433; 7259; 72	0.09807142	0.76227194		127.0
	ZINC26819877	-7.3	414.044	-20.278	15	3	240.54	0.85657	307.38	-20.593	3609.116734805	0.31111	25.72	-65.179	0.41667	0.58345	0.93686 (max c	13	7928; 7882; 7833; 76	0.3648882	0.841669		127.0
	ZINC26819877	-7.3	415.052	-19.03	15	4	239.36	0.85587	304.55	-31.825	3609.116734805	0.31111	24.473	-61.168	0.41667	0.55758	0.97812 (max c	23	7929; 7884; 7883; 78	0.314444	0.8230419		127.0
	ZINC26819877	-7.3	415.052	-19.03	15	4	239.36	0.85587	304.55	-31.825	3609.116734805	0.31111	24.473	-61.168	0.41667	0.55758	0.97812 (max c	23	7929; 7884; 7883; 78	0.3352066	0.82477885		127.0
	ZINC26819877	-7.3	415.052	-19.03	15	4	239.36	0.85587	304.55	-31.825	3609.116734805	0.31111	24.473	-61.168	0.41667	0.54974	0.96029 (max c	21	7930; 7929; 7885; 78	0.38968435	0.80901116		127.0
	ZINC05835354	-7.3	396.434	0.3826	8	6	325.5	0.30292	139.84	-21.493	3609.116734805	0.26666	5.06	1.4348	0.53571	0.45483	0.96253 (max c	3	7159; 6166; 6462	0.8104658	0.32815516		120.0
	ZINC05835346	-7.3	396.434	0.3826	8	6	325.5	0.30292	139.84	-21.493	3609.116734805	0.26666	5.06	1.4348	0.53571	0.47492	0.96253 (max c	3	7159; 6463; 6166	0.775511	0.33328137		120.0
	ZINC03869248	-7.3	423.170	-13.388	15	2	266.13	0.71134	263.54	-22.848	3609.116734805	0.27654	18.831	-48.412	0.44444	0.42253	0.93996 (max c	9	3657; 4048; 6404; 49	0.66714585	0.44414574		67.0
	ZINC03869248	-7.3	424.178	-12.14	15	3	264.95	0.71006	260.71	-32.8	3609.116734805	0.27654	17.583	-43.901	0.44444	0.40459	0.93996 (max c	9	3658; 4049; 4937; 53	0.6307687	0.48988417		67.0
	ZINC14414160	-7.3	351.318	-0.7704	11	2	248.49	0.49994	151.68	1.3398	3609.116734805	0.29866	6.213	-2.5795	0.44	0.27264	0.9402 (max of	6	7478; 6965; 5105; 51	-0.6067784	0.3365787		67.0
	ZINC31155500	-7.3	355.318	-3.0071	9	4	255.46	0.4295	148.74	-3.2919	3609.116734805	0.29866	8.4497	-10.069	0.48	0.3375	0.95631 (max c	7	7071; 3356; 5205; 52	0.21896046	-0.6241694		49.0

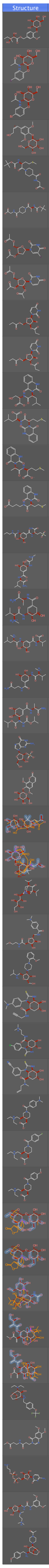

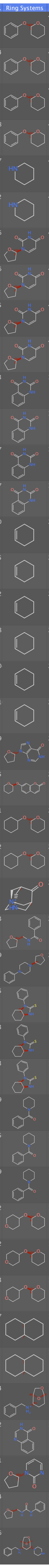

Structure	Molecule Name	affinity	Total Molweight	cLogP	H-Acceptors	H-Donors	Total Surface Area	Relative PSA	Polar Surface Area	Druglikeness	Kd (nM) @ 20°C	LE from K...	LLE from Kd ...	LELP from Kd (n...	Shape Index	Molecular...	Neighbor Si...	Neig...	Neighbor	Neighbor ...	Neighbor ...	Ring Systems	frequency
	ZINC77257565	-7.3	446.447	-1.1579	11	6	319.08	0.41256	175.37	-5.239	3609.116734805	0.24086	6.6005	-4.8074	0.54839	0.46022	0.99343 (max o	5	3985; 6059; 6060; 60	0.11003122	0.5032967		49.0
	ZINC08834020	-7.3	423.198	-0.2279	7	4	242.82	0.34511	108.25	-2.5394	3609.116734805	0.33939	5.6705	-0.6715	0.5	0.40616	0.96898 (max o	5	4512; 5745; 5794; 64	0.47591624	0.58167344		49.0
	ZINC04043472	-7.3	376.202	0.0602	7	4	235.17	0.35634	108.25	-4.8033	3609.116734805	0.33939	5.3824	0.17738	0.5	0.40616	0.96898 (max o	6	6487; 4512; 5745; 57	0.48488155	0.5643554		49.0
	ZINC36046230	-7.3	372.369	-0.6515	9	5	272.55	0.38708	138.07	-4.1548	3609.116734805	0.28718	6.0941	-2.2686	0.53846	0.47937	0.94326 (max o	3	7443; 7190; 6784	0.59798235	-0.2249828		49.0
	ZINC04064692	-7.3	416.517	-1.5492	9	2	320.01	0.37624	153.17	-51.63	3609.116734805	0.26666	6.9918	-5.8096	0.57143	0.6243	0.92774 (max o	5	3310; 4514; 5361; 53	0.44700804	-0.0084547		47.0
	ZINC02112131	-7.3	356.397	-2.1528	9	2	275	0.37422	127.87	-55.886	3609.116734805	0.29866	7.5954	-7.2081	0.64	0.59866	0.94905 (max o	7	6422; 4514; 5036; 50	0.46500483	-0.0622722		47.0
	ZINC17578983	-7.3	438.169	0.0808	9	1	249.79	0.38901	111.24	3.9348	3609.116734805	0.32463	5.3618	0.24889	0.52174	0.48074	0.92193 (max o	3	7759; 3939; 6083	0.7107407	0.55660826		41.0
	ZINC04011870	-7.3	370.313	-1.0894	11	1	264.05	0.45525	137.54	0.69325	3609.116734805	0.28718	6.532	-3.7935	0.46154	0.44535	0.94326 (max o	6	7129; 7128; 7115; 70	0.38665527	-0.5391196		41.0
	ZINC33904022	-7.3	419.227	0.6029	9	1	270.19	0.35964	111.24	-2.4553	3609.116734805	0.29866	4.8397	2.0187	0.52	0.50238	0.94678 (max o	3	7645; 7500; 6514	-0.6204079	0.77350295		41.0
	ZINC12657750	-7.3	419.227	0.6029	9	1	270.19	0.35964	111.24	-2.4553	3609.116734805	0.29866	4.8397	2.0187	0.52	0.50035	0.93348 (max o	3	7645; 7500; 6631	-0.6176913	0.7569433		41.0
	ZINC03985274	-7.3	362.361	-2.1784	9	3	267.4	0.39645	138.87	6.4588	3609.116734805	0.28718	7.621	-7.5856	0.46154	0.55143	0.93373 (max o	11	7062; 6691; 6591; 42	0.30294204	0.70853573		36.0
	ZINC20611718	-7.3	406.482	-0.6481	8	2	304.56	0.36249	143.94	5.7	3609.116734805	0.26666	6.0907	-2.4304	0.46429	0.56187	0.96519 (max o	5	7062; 6691; 4021; 63	0.33351597	0.6753544		36.0
	ZINC02133434	-7.3	364.401	-1.7752	8	2	266.04	0.41498	143.94	5.9859	3609.116734805	0.29866	7.2178	-5.9438	0.56	0.55626	0.91731 (max o	2	6691; 6591	0.34898096	0.65902627		36.0
	ZINC02128802	-7.3	378.428	-1.2257	8	2	282.56	0.39071	143.94	2.9044	3609.116734805	0.28718	6.6683	-4.2681	0.53846	0.57936	0.97599	1	7523	0.11247054	-0.7975367		36.0
	ZINC04200809	-7.3	420.485	-2.8396	11	6	305.46	0.46995	196.3	3.7196	3609.116734805	0.26666	8.2822	-10.649	0.57143	0.59643	0.91059 (max o	9	3650; 3653; 4532; 45	-0.6226683	-0.1188230		29.0
	ZINC03838847	-7.3	357.429	-3.6308	9	6	270.4	0.41143	158.36	5.0486	3609.116734805	0.29866	9.0734	-12.157	0.56	0.55921	0.92662 (max o	8	6699; 2759; 3166; 40	-0.5605869	-0.1743925		29.0
	ZINC39560947	-7.3	357.429	-3.7259	9	6	267.64	0.41567	158.36	1.515	3609.116734805	0.29866	9.1685	-12.475	0.52	0.52008	0.92721 (max o	7	4537; 4919; 5332; 57	-0.6143085	-0.1828832		29.0
	ZINC03838496	-7.3	399.486	-3.212	9	5	305.17	0.36131	155.4	4.0774	3609.116734805	0.26666	8.6546	-12.045	0.5	0.56297	0.89424 (max o	4	6698; 4927; 6090; 63	-0.5871155	-0.2337388		29.0
	ZINC03838846	-7.3	357.429	-3.6308	9	6	270.4	0.41143	158.36	4.0918	3609.116734805	0.29866	9.0734	-12.157	0.56	0.55135	0.92342 (max o	4	6703; 6659; 5326; 58	-0.6525196	-0.1919290		29.0
	ZINC04221651	-7.3	430.456	-1.5102	12	5	320.21	0.43753	180.52	-6.1699	3609.116734805	0.24889	6.9528	-6.0678	0.46667	0.56532	0.934	1	6135	0.32799873	-0.7099731		29.0
	ZINC03872748	-7.3	361.206	-8.4657	13	4	230.21	0.69893	217.22	-20.626	3609.116734805	0.31111	13.908	-27.211	0.41667	0.37572	0.9115 (max of	3	3175; 4055; 5735	-0.3930837	-0.3906239		26.0
	ZINC33831993	-7.3	384.336	-0.9776	10	4	266.69	0.43286	144.14	-5.8493	3609.116734805	0.27654	6.4202	-3.5351	0.44444	0.42813	0.98371 (max o	6	3046; 3893; 4952; 54	-0.4067419	0.53996575		24.0
	ZINC72320397	-7.3	406.470	-1.0841	9	7	287.33	0.38875	160.07	-4.0503	3609.116734805	0.26666	6.5267	-4.0654	0.5	0.44224	0.95148 (max o	7	6916; 3967; 3968; 48	0.08370426	-0.7707331		23.0
	ZINC72319915	-7.3	406.470	-1.0841	9	7	287.33	0.38875	160.07	-4.0127	3609.116734805	0.26666	6.5267	-4.0654	0.46429	0.44224	0.95148 (max o	7	6916; 3967; 3968; 48	0.04918605	-0.7451052		23.0
	ZINC19323203	-7.3	387.495	-2.9723	8	6	283.3	0.35185	125.9	3.5199	3609.116734805	0.27654	8.4149	-10.748	0.44444	0.63767	0.92032 (max o	5	7372; 7201; 7025; 67	-0.2746644	0.7483607		23.0
	ZINC08624586	-7.3	395.454	-1.0456	9	4	303	0.32591	120.36	0.61107	3609.116734805	0.26666	6.4882	-3.921	0.67857	0.59309	0.93686 (max o	8	7332; 2111; 2570; 32	0.34411672	-0.3489783		22.0
	ZINC20465586	-7.3	380.507	-1.1438	7	3	289.14	0.21111	82.01	4.0419	3609.116734805	0.27654	6.5864	-4.1361	0.51852	0.47578	0.91248 (max o	8	7051; 6587; 3402; 43	-0.4704991	0.18251309		21.0
	ZINC08636395	-7.3	388.491	-0.2346	7	4	291.29	0.33389	120.16	4.7361	3609.116734805	0.27654	5.6772	-0.84834	0.51852	0.42251	0.92418 (max o	4	6754; 6753; 5023; 61	0.5000278	0.13137275		18.0
	ZINC08636322	-7.3	413.948	-1.2187	7	5	310.7	0.35822	121.36	3.8099	3609.116734805	0.27654	6.6613	-4.4069	0.51852	0.43973	0.97658 (max o	5	7492; 6987; 6983; 67	0.4300023	0.18359633		18.0
	ZINC08254702	-7.3	386.475	0.6191	7	3	290.01	0.34261	131.92	-1.2354	3609.116734805	0.27654	4.8235	2.2387	0.48148	0.43665	0.90171 (max o	4	6988; 6753; 6183; 61	0.44905123	0.11054273		18.0
	ZINC12480683	-7.3	389.538	-0.1116	6	1	331.85	0.19602	68.12	3.7226	3609.116734805	0.26666	5.5542	-0.4185	0.53571	0.65975	0.91599 (max o	8	6802; 6801; 6800; 66	-0.0162189	-0.1466859		18.0
	ZINC30879449	-7.3	364.459	0.0928	5	1	303.38	0.20272	64.88	1.625	3609.116734805	0.28718	5.3498	0.32315	0.53846	0.63748	0.92721 (max o	5	7036; 7035; 6250; 64	-0.0022896	-0.1347405		18.0
	ZINC30879444	-7.3	376.495	-0.078	6	1	319.29	0.22393	74.11	3.0179	3609.116734805	0.27654	5.5206	-0.28205	0.55556	0.64646	0.92757 (max o	6	7381; 7380; 6992; 62	-0.0346989	-0.1518949		18.0
	ZINC04556849	-7.3	356.323	-4.03	11	7	235.73	0.55869	178.53	-7.2031	3609.116734805	0.31111	9.4726	-12.954	0.45833	0.45941	0.94718 (max o	7	6731; 6732; 2827; 54	0.554793	-0.4801922		17.0
	ZINC04556848	-7.3	356.323	-4.03	11	7	235.73	0.55869	178.53	-7.2031	3609.116734805	0.31111	9.4726	-12.954	0.45833	0.45757	0.94718 (max o	8	7255; 6731; 6732; 64	0.53431195	-0.4804152		17.0
	ZINC13348049	-7.3	383.349	-4.4475	12	8	254.94	0.57347	198.4	-3.0597	3609.116734805	0.28718	9.8901	-15.487	0.42308	0.46124	0.95559 (max o	5	7830; 7502; 7003; 67	0.5038534	-0.4743163		17.0
	ZINC67913291	-7.3	444.475	-0.5622	10	6	314.74	0.39614	173.98	-11.767	3609.116734805	0.24086	6.0048	-2.3342	0.41935	0.54324	0.96739 (max o	5	7449; 7106; 6874; 68	0.36240128	0.32881397		16.0
	ZINC31170372	-7.3	426.460	0.0489	9	4	309.91	0.35985	150.59	-12.343	3609.116734805	0.24889	5.3937	0.19647	0.43333	0.53144	0.96092 (max o	6	7423; 7422; 7251; 70	0.43412367	0.2767248		16.0
	ZINC12483562	-7.3	391.408	0.3044	6	2	272.17	0.19984	67.77	-5.5483	3609.116734805	0.27654	5.1382	1.1007	0.55556	0.56908	0.93686 (max o	7	7346; 7174; 2521; 38	0.42368102	-0.2453514		16.0
	ZINC36369926	-7.3	363.369	0.2243	9	1	281.35	0.33993	106.53	-1.2333	3609.116734805	0.28718	5.2183	0.78105	0.65385	0.55768	0.92368 (max o	3	7425; 7253; 6275	-0.0751748	-0.3346412		15.0
	ZINC13431050	-7.3	445.237	-10.296	15	4	286.85	0.65069	263.59	-43.957	3609.116734805	0.26666	15.739	-38.611	0.60714	0.65134	0.9308 (max of	5	621; 622; 4674; 5873	-0.62665	-0.4076261		14.0
	ZINC20464980	-7.3	427.520	-2.5004	10	5	344.11	0.32926	116.96	4.5399	3609.116734805	0.24889	7.943	-10.046	0.53333	0.6328	0.92507 (max o	5	4301; 4302; 4307; 43	-0.3350548	-0.0274994		14.0
	ZINC12483089	-7.3	392.474	-1.5421	8	2	284.78	0.24925	88.08	6.279	3609.116734805	0.26666	6.9847	-5.7829	0.60714	0.48638	0.93686 (max o	11	7384; 7065; 6995; 67	0.42942107	-0.1882956		14.0

Structure	Molecule Name	affinity	Total Molweight	cLogP	H-Acceptors	H-Donors	Total Surface Area	Relative PSA	Polar Surface Area	Druglikeness	Kd (nM) @ 20°C	LE from K...	LLE from Kd ...	LELP from Kd (n...	Shape Index	Molecular...	Neighbor Si...	Neig...	Neighbor	Neighbor ...	Neighbor ...	Ring Systems	frequency
	ZINC04083908	-7.3	407.533	-1.0329	7	2	313.6	0.32009	142.48	5.0647	3609.116734805	0.26666	6.4755	-3.8734	0.64286	0.52824	0.93686 (max o	11	6946; 1802; 3200; 36	0.34071013	0.29945028		14.0
	ZINC04083908	-7.3	406.525	-3.2324	7	2	314.39	0.3218	140.86	2.8132	3609.116734805	0.26666	8.675	-12.122	0.64286	0.57352	0.93686 (max o	2	3199; 6423	0.35902143	0.2643255		14.0
	ZINC20463969	-7.3	383.442	-1.8719	8	4	294.83	0.30594	87.5	1.9454	3609.116734805	0.27654	7.3145	-6.769	0.59259	0.52756	0.93686 (max o	6	6809; 6810; 3871; 47	-0.7060588	-0.2656403		14.0
	ZINC05434230	-7.3	376.433	-2.1966	10	2	267.69	0.40241	133.5	6.3132	3609.116734805	0.29866	7.6392	-7.3548	0.52	0.54855	0.91396 (max o	4	6741; 3374; 4126; 45	-0.0720490	0.39937377		13.0
	ZINC04270698	-7.3	354.401	1.2263	6	0	259.51	0.28404	92.22	1.0999	3609.116734805	0.31111	4.2163	3.9417	0.54167	0.60133	0.86859 (max o	2	7156; 1526	-0.0543231	0.3495337		13.0
	ZINC05396051	-7.3	434.491	-0.1831	10	1	334.46	0.28186	108.49	2.0107	3609.116734805	0.24086	5.6257	-0.7602	0.51613	0.60536	0.94241 (max o	5	7582; 6936; 3683; 64	0.08307927	0.4292419		13.0
	ZINC05396052	-7.3	440.519	-0.1224	10	1	321.53	0.31042	124.71	2.2534	3609.116734805	0.24889	5.565	-0.49179	0.53333	0.60491	0.92442 (max o	4	7155; 6704; 5798; 64	0.0843387	0.44987246		13.0
	ZINC04084384	-7.3	376.388	-2.6826	9	2	283.31	0.36324	127.87	6.0104	3609.116734805	0.27654	8.1252	-9.7006	0.62963	0.46807	0.96617 (max o	7	6714; 6658; 2301; 35	0.54597455	0.30189472		12.0
	ZINC38570555	-7.3	404.441	-2.0099	9	2	308.07	0.33405	127.87	5.8064	3609.116734805	0.25747	7.4525	-7.8064	0.58621	0.47459	0.96682 (max o	7	2301; 5032; 5250; 57	0.5270845	0.27150896		12.0
	ZINC72319952	-7.3	364.349	0.171	8	2	252.33	0.37776	119.36	-2.1253	3609.116734805	0.28718	5.2716	0.59545	0.46154	0.40419	0.95857 (max o	9	3548; 3976; 3977; 48	0.75842434	-0.0927594		12.0
	ZINC12603427	-7.3	370.376	-1.2732	8	4	274.53	0.34677	117.12	-0.30707	3609.116734805	0.28718	6.7158	-4.4335	0.69231	0.57286	0.94008 (max o	3	4182; 4612; 5451	-0.3677747	-0.2361518		12.0
	ZINC08643431	-7.3	388.440	-1.0652	9	4	277.51	0.39905	142.57	3.8324	3609.116734805	0.28718	6.5078	-3.7092	0.65385	0.58775	0.9302 (max of	4	4608; 4613; 4653; 61	-0.4045919	-0.204779		12.0
	ZINC31170360	-7.3	412.433	0.2102	9	4	295.62	0.37724	150.59	-8.6504	3609.116734805	0.25747	5.2324	0.81641	0.41379	0.52639	0.96682 (max o	5	7423; 7422; 7250; 52	0.41399673	0.2693572		12.0
	ZINC13532414	-7.3	383.349	-4.4475	12	8	254.94	0.57347	198.4	-4.7796	3609.116734805	0.28718	9.8901	-15.487	0.46154	0.46983	0.94008 (max o	4	6958; 6716; 4541; 55	0.18577847	0.7510167		12.0
	ZINC04095695	-7.3	424.401	-4.4371	13	8	290.06	0.54334	207.27	-3.1385	3609.116734805	0.25747	9.8797	-17.234	0.41379	0.47071	0.94823 (max o	2	6716; 5506	0.16415797	0.75463235		12.0
	ZINC03985310	-7.3	365.453	-0.9071	6	1	248.34	0.43694	153.64	2.7368	3609.116734805	0.31111	6.3497	-2.9157	0.45833	0.255	0.92315 (max o	7	1219; 1692; 2786; 37	0.33207542	-0.6573430		10.0
	ZINC06624143	-7.3	372.404	-0.2357	9	2	286.4	0.34117	119.39	-6.6208	3609.116734805	0.27654	5.6783	-0.85231	0.66667	0.512	0.85572 (max o	3	3711; 4241; 5001	0.60004866	-0.2457101		10.0
	ZINC35454681	-7.3	413.397	-2.9205	10	4	298.4	0.41139	165.81	1.5959	3609.116734805	0.25747	8.3631	-11.343	0.58621	0.59812	0.99848 (max o	7	6878; 1948; 2642; 35	-0.1698261	0.728959		10.0
	ZINC02108026	-7.3	380.512	-1.1635	6	1	281.82	0.37048	133.1	4.2173	3609.116734805	0.29866	6.6061	-3.8957	0.56	0.60336	0.9594 (max of	2	4943; 6382	0.13548294	0.8077442		10.0
	ZINC02108024	-7.3	380.512	-1.1635	6	1	281.82	0.37048	133.1	4.2173	3609.116734805	0.29866	6.6061	-3.8957	0.56	0.60536	0.9594 (max of	2	4943; 6383	0.15801941	0.8065074		10.0
	ZINC06640079	-7.3	393.463	0.3428	8	1	293.05	0.36062	120.27	-1.2311	3609.116734805	0.27654	5.0998	1.2396	0.51852	0.61148	0.97407	1	7330	0.47568372	0.7239902		10.0
	ZINC04236392	-7.3	360.345	-2.4262	9	1	259.15	0.36658	119.08	4.2901	3609.116734805	0.28718	7.8688	-8.4485	0.57692	0.46358	0.97533 (max o	4	3231; 3232; 3665; 45	0.11806184	-0.6056424		8.0
	ZINC12318136	-7.3	367.385	-4.2278	11	5	269.44	0.46129	136.88	2.9437	3609.116734805	0.28718	9.6704	-14.722	0.46154	0.41265	0.94008 (max o	5	4191; 4237; 4904; 54	-0.6199002	-0.5226614		8.0
	ZINC48056985	-7.3	382.273	-8.8812	13	5	246.07	0.65079	218.33	-2.9931	3609.116734805	0.28718	14.324	-30.926	0.46154	0.43405	0.94951 (max o	3	3950; 3951; 6033	0.1816085	0.931855		8.0
	ZINC04042898	-7.3	395.455	-1.4997	8	2	294.7	0.36987	144.3	-7.167	3609.116734805	0.27654	6.9423	-5.4231	0.51852	0.52885	0.95201 (max o	4	7133; 5102; 5359; 64	-0.75278	0.34421209		8.0
	ZINC04042886	-7.3	395.455	-1.4997	8	2	294.7	0.36987	144.3	-7.167	3609.116734805	0.27654	6.9423	-5.4231	0.51852	0.52877	0.96064 (max o	3	7133; 5359; 6418	-0.7447063	0.32536042		8.0
	ZINC04097981	-7.3	370.309	-1.2533	10	5	250.78	0.47269	155.14	-5.8493	3609.116734805	0.28718	6.6959	-4.3642	0.42308	0.41647	0.9464 (max of	6	3351; 3793; 4106; 49	-0.4048397	0.46297944		8.0
	ZINC13660224	-7.3	355.362	-2.4289	8	4	260.48	0.38283	139.51	-4.6528	3609.116734805	0.29866	7.8715	-8.1326	0.52	0.5396	0.95 (max of 7)	7	7086; 5632; 5633; 60	0.70434225	-0.3241339		8.0
	ZINC03838500	-7.3	412.801	-2.0914	9	3	294.61	0.37697	145.22	3.2382	3609.116734805	0.26666	7.534	-7.8428	0.53571	0.51912	0.93069 (max o	2	5718; 6242	0.8018551	-0.1804924		8.0
	ZINC35455113	-7.3	392.487	-0.0831	8	6	286.85	0.34373	139.84	-10.396	3609.116734805	0.27654	5.5257	-0.3005	0.48148	0.56222	0.99202 (max o	7	7261; 6879; 5597; 63	-0.7343079	0.21090005		8.0
	ZINC31156471	-7.3	392.487	-0.0831	8	6	286.85	0.34373	139.84	-10.396	3609.116734805	0.27654	5.5257	-0.3005	0.48148	0.55806	0.99202 (max o	7	7261; 6879; 6642; 55	-0.7137199	0.21350376		8.0
	ZINC31156466	-7.3	392.487	-0.0831	8	6	286.85	0.34373	139.84	-10.396	3609.116734805	0.27654	5.5257	-0.3005	0.48148	0.55874	0.99202 (max o	7	7261; 6879; 6642; 66	-0.7319990	0.2499349		8.0
	ZINC31156461	-7.3	392.487	-0.0831	8	6	286.85	0.34373	139.84	-10.396	3609.116734805	0.27654	5.5257	-0.3005	0.48148	0.56154	0.99202 (max o	7	7261; 6879; 6642; 66	-0.7451613	0.22860622		8.0
	ZINC34361066	-7.3	356.370	0.1552	8	1	277.9	0.33185	108.36	-3.5573	3609.116734805	0.29866	5.2874	0.51965	0.52	0.56552	0.96243 (max o	7	7360; 7012; 6786; 67	0.00614694	-0.762252		8.0
	ZINC34361064	-7.3	356.370	0.1552	8	1	277.9	0.33185	108.36	-3.5573	3609.116734805	0.29866	5.2874	0.51965	0.52	0.58596	0.96243 (max o	7	7360; 7012; 6786; 67	-0.0326074	-0.7590346		8.0
	ZINC31168990	-7.3	388.455	0.7308	8	4	293.23	0.32548	125.68	-15.27	3609.116734805	0.27654	4.7118	2.6427	0.48148	0.51734	0.9578 (max of	3	7077; 6866; 6621	0.9387228	0.05278352		8.0
	ZINC31168986	-7.3	388.455	0.7308	8	4	293.23	0.32548	125.68	-15.27	3609.116734805	0.27654	4.7118	2.6427	0.48148	0.51734	0.9578 (max of	3	7077; 6866; 6622	0.9198696	0.05247864		8.0
	ZINC04475165	-7.3	357.409	-3.6337	10	5	268.21	0.44573	174.5	3.4874	3609.116734805	0.29866	9.0763	-12.167	0.64	0.642	0.93348 (max o	3	7914; 7588; 7542	0.12394412	-0.7661897		8.0
	ZINC04090282	-7.3	403.521	-0.6129	7	3	320.77	0.28307	125.97	2.4898	3609.116734805	0.25747	6.0555	-2.3805	0.62069	0.54201	0.9561 (max of	5	6991; 6475; 2789; 32	0.5708449	-0.0795316		7.0
	ZINC08481027	-7.3	417.548	-0.1585	7	3	334.53	0.27143	125.97	3.606	3609.116734805	0.24889	5.6011	-0.63684	0.63333	0.55127	0.9561 (max of	6	7782; 6991; 2789; 32	0.5762914	-0.0396886		7.0
	ZINC01518799	-7.3	353.373	-0.7182	6	2	263.63	0.27008	103.27	-5.8017	3609.116734805	0.28718	6.1608	-2.5009	0.5	0.51726	0.94622 (max o	7	7119; 7117; 3192; 45	-0.0669844	0.6606166		7.0
	ZINC05433909	-7.3	409.486	-2.2151	9	2	299.45	0.40978	150.8	-0.5149	3609.116734805	0.26666	7.6577	-8.3067	0.60714	0.47735	0.96519 (max o	7	6976; 3255; 3262; 41	-0.6487745	-0.5410255		6.0
	ZINC05433645	-7.3	351.402	0.4664	8	1	263.72	0.32777	91.8	-4.72	3609.116734805	0.29866	4.9762	1.5616	0.6	0.38316	0.87324 (max o	2	6976; 4572	-0.6272404	-0.5944634		6.0

Structure	Molecule Name	affinity	Total Molweight	cLogP	H-Acceptors	H-Donors	Total Surface Area	Relative PSA	Polar Surface Area	Druglikeness	Kd (nM) @ 20°C	LE from K...	LLE from Kd ...	LELP from Kd (n...	Shape Index	Molecular...	Neighbor Si...	Neig...	Neighbor	Neighbor ...	Neighbor ...	Ring Systems	frequency
	ZINC20463857	-7.3	426.535	-0.1036	7	2	314.53	0.18421	71.01	4.6027	3609.116734805	0.24086	5.5462	-0.43013	0.54839	0.44598	0.95549 (max o	7	7067; 3840; 4646; 47	0.38588318	-0.0832041		6.0
	ZINC31167834	-7.3	434.524	0.0313	9	6	308.42	0.36197	156.91	-5.985	3609.116734805	0.24889	5.4113	0.12576	0.46667	0.49711	0.97304 (max o	7	3073; 3505; 3506; 39	-0.1474236	0.827206		6.0
	ZINC34235761	-7.3	388.324	-6.5696	11	4	269.23	0.50901	188.87	-5.5881	3609.116734805	0.27654	12.012	-23.756	0.44444	0.48967	0.96343 (max o	3	4389; 5219; 5621	-0.7734156	0.5474203		6.0
	ZINC06379177	-7.3	388.368	-1.8524	10	4	278.22	0.42585	151.98	-10.162	3609.116734805	0.27654	7.295	-6.6985	0.48148	0.46782	0.9578 (max of	3	7490; 5810; 6468	0.9075905	-0.1339941		6.0
	ZINC31158090	-7.3	388.324	-6.5696	11	4	269.23	0.50901	188.87	-5.5881	3609.116734805	0.27654	12.012	-23.756	0.44444	0.50651	0.95493 (max o	3	4367; 5602; 5968	0.75302887	-0.2149770		6.0
	ZINC20465276	-7.3	365.448	-2.1806	7	4	289.83	0.30463	92.46	1.2568	3609.116734805	0.28718	7.6232	-7.5932	0.53846	0.48482	0.91768 (max o	3	5148; 6264; 6583	0.6953786	0.41805932		6.0
	ZINC20465016	-7.3	364.464	-2.1101	7	4	289.89	0.28231	86.47	1.9445	3609.116734805	0.28718	7.5527	-7.3477	0.53846	0.52739	0.89885 (max o	2	6583; 5555	0.68217766	0.40249857		6.0
	ZINC20465292	-7.3	408.517	-2.2842	8	4	324.65	0.28289	95.7	2.0715	3609.116734805	0.25747	7.7268	-8.8718	0.55172	0.52138	0.91248 (max o	3	6264; 6572; 6582	0.66908026	0.3876174		6.0
	ZINC31167400	-7.3	417.456	-1.3541	8	0	322.92	0.28252	119.03	4.6442	3609.116734805	0.24889	6.7967	-5.4406	0.43333	0.52279	0.97975 (max o	5	7245; 6863; 5206; 56	-0.0604117	0.861927		6.0
	ZINC31167396	-7.3	417.456	-1.3541	8	0	322.92	0.28252	119.03	4.6442	3609.116734805	0.24889	6.7967	-5.4406	0.43333	0.52279	0.97975 (max o	5	7245; 6863; 5206; 56	-0.0381247	0.8615355		6.0
	ZINC39564138	-7.3	412.509	-0.7544	10	3	314.12	0.31809	124.66	2.4253	3609.116734805	0.25747	6.197	-2.9301	0.48276	0.57408	0.94823 (max o	5	7697; 2465; 3686; 56	-0.1515512	0.31628343		6.0
	ZINC12489893	-7.3	400.497	-0.7759	7	2	301.23	0.21698	80.66	4.4331	3609.116734805	0.25747	6.2185	-3.0136	0.62069	0.50339	0.9339 (max of	8	7850; 7848; 7630; 73	0.6445078	0.5578596		6.0
	ZINC04270695	-7.3	390.458	0.6818	8	1	293.69	0.25183	92.76	2.6677	3609.116734805	0.26666	4.7608	2.5568	0.57143	0.54034	0.91838 (max o	3	7592; 2065; 5400	0.14211282	0.41587466		5.0
	ZINC12659241	-7.3	427.409	0.2644	12	0	314.69	0.39343	141.11	4.2655	3609.116734805	0.24889	5.1782	1.0623	0.46667	0.49641	0.94241 (max o	3	6999; 5067; 6486	0.43414113	-0.4209044		5.0
	ZINC08770561	-7.3	427.409	0.2644	12	0	314.69	0.39343	141.11	4.2655	3609.116734805	0.24889	5.1782	1.0623	0.46667	0.49641	0.95053 (max o	5	6999; 6757; 6723; 65	0.45169017	-0.4331005		5.0
	ZINC06093098	-7.3	399.179	0.061301	5	2	234.92	0.27626	97.23	-0.74102	3609.116734805	0.35555	5.3813	0.17241	0.66667	0.55963		0		-0.4337051	-0.8958802		5.0
	ZINC08636492	-7.3	411.909	0.6833	7	3	284.7	0.33649	117.36	4.2307	3609.116734805	0.27654	4.7593	2.4709	0.48148	0.39785	0.97658 (max o	5	5825; 5826; 5827; 61	0.4701894	0.07917331		5.0
	ZINC22922217	-7.3	381.471	-3.1623	8	3	289.57	0.28311	81.44	3.4105	3609.116734805	0.27654	8.6049	-11.435	0.55556	0.50269	0.91109 (max o	7	7628; 7520; 7518; 74	0.4874386	-0.2053569		5.0
	ZINC22922217	-7.3	381.471	-6.6046	8	3	304.79	0.31891	85.47	-0.82854	3609.116734805	0.27654	12.047	-23.883	0.55556	0.49243	0.89688 (max o	4	7519; 7517; 7220; 59	0.5873387	0.01810693		5.0
	ZINC67903051	-7.3	367.460	-0.4817	6	4	276.3	0.28853	121.05	-4.8501	3609.116734805	0.28718	5.9243	-1.6774	0.46154	0.57414	0.96438 (max o	3	176; 6042; 6670	0.53161883	0.8101916		4.0
	ZINC67903048	-7.3	367.460	-0.4817	6	4	276.3	0.28853	121.05	-4.8501	3609.116734805	0.28718	5.9243	-1.6774	0.46154	0.56771	0.96438 (max o	3	176; 6042; 6671	0.5230778	0.7952786		4.0
	ZINC27642629	-7.3	424.444	0.1377	9	1	297.77	0.36898	120.89	0.65007	3609.116734805	0.24889	5.3049	0.55326	0.36667	0.29425	0.98593 (max o	5	7797; 7731; 7548; 74	-0.0981775	0.89802533		4.0
	ZINC15676322	-7.3	415.452	-2.5563	8	3	310.68	0.2669	126.19	1.2935	3609.116734805	0.24086	7.9989	-10.613	0.58065	0.49068	0.93686 (max o	2	1522; 4265	-0.2579793	0.4750745		4.0
	ZINC03871268	-7.3	409.112	-4.2064	11	3	220.98	0.56806	160.83	-13.945	3609.116734805	0.32463	9.649	-12.957	0.47826	0.3478	0.92193 (max o	3	1111; 1384; 4053	-0.5589485	-0.721467		4.0
	ZINC35458397	-7.3	432.511	-1.9041	7	1	310.36	0.30229	134.63	0.74206	3609.116734805	0.24086	7.3467	-7.9055	0.3871	0.4634	0.93956 (max o	4	1917; 4374; 5236; 56	-0.4552731	0.43553805		4.0
	ZINC31165589	-7.3	426.416	-1.3949	10	6	299.06	0.40674	166.14	-8.9951	3609.116734805	0.24889	6.8375	-5.6046	0.5	0.455	0.96092 (max o	5	2260; 3043; 4375; 43	-0.8278229	-0.0108878		4.0
	ZINC39737956	-7.3	368.381	-3.3848	8	0	280.16	0.35951	132.86	-11.135	3609.116734805	0.28718	8.8274	-11.786	0.53846	0.62243	0.96438 (max o	3	3169; 3611; 4046	-0.7603345	-0.4489259		4.0
	ZINC49601637	-7.3	361.365	-3.7858	9	5	255.01	0.44241	159.74	-3.3297	3609.116734805	0.29866	9.2284	-12.676	0.48	0.58091	0.97396 (max o	3	6900; 3529; 4430	0.81507576	-0.0841539		4.0
	ZINC05397666	-7.3	362.385	-0.5816	9	1	274.64	0.33386	108.91	1.5519	3609.116734805	0.28718	6.0242	-2.0252	0.53846	0.56806	0.93373 (max o	7	7155; 6973; 2011; 36	0.04507649	0.3833173		4.0
	ZINC06379183	-7.3	388.368	-1.8524	10	4	278.22	0.42585	151.98	-10.162	3609.116734805	0.27654	7.295	-6.6985	0.48148	0.47692	0.96064 (max o	3	7490; 5810; 6467	0.8946549	-0.1180406		4.0
	ZINC12377657	-7.3	364.405	-0.6235	10	1	268.73	0.34563	114	7.04	3609.116734805	0.28718	6.0661	-2.1711	0.46154	0.45301	0.92032 (max o	2	4458; 6069	0.01767080	-0.8134845		4.0
	ZINC12484201	-7.3	376.455	-0.035	8	3	277.7	0.27425	86.3	4.9711	3609.116734805	0.27654	5.4776	-0.12656	0.59259	0.47907	0.95744 (max o	3	5054; 5180; 6505	-0.6143754	-0.2813723		4.0
	ZINC12489800	-7.3	388.466	0.2339	8	3	289.98	0.26264	86.3	2.1925	3609.116734805	0.26666	5.2087	0.87713	0.60714	0.49316	0.95744 (max o	3	7385; 5475; 6504	-0.5985636	-0.2966305		4.0
	ZINC12663473	-7.3	365.234	-7.4348	12	3	245.3	0.60306	202.06	-30.618	3609.116734805	0.31111	12.877	-23.898	0.58333	0.48729	0.93334 (max o	4	5070; 6199; 6520; 65	-0.4611346	-0.4291818		4.0
	ZINC12663470	-7.3	365.234	-7.4348	12	3	245.3	0.60306	202.06	-30.618	3609.116734805	0.31111	12.877	-23.898	0.58333	0.48729	0.92619 (max o	4	6519; 6518; 5070; 61	-0.4410769	-0.4228812		4.0
	ZINC12663473	-7.3	366.242	-7.6237	12	4	234.33	0.58447	203.31	-31.599	3609.116734805	0.31111	13.066	-24.505	0.58333	0.51178	0.93686 (max o	4	5071; 6200; 6518; 65	-0.4276458	-0.4593100		4.0
	ZINC12663470	-7.3	366.242	-7.6237	12	4	234.33	0.58447	203.31	-31.599	3609.116734805	0.31111	13.066	-24.505	0.58333	0.51178	0.92978 (max o	4	6520; 5071; 6200; 65	-0.4446835	-0.4442426		4.0
	ZINC31155114	-7.3	386.439	-0.5583	8	5	279.37	0.35272	136.68	-6.1261	3609.116734805	0.27654	6.0009	-2.0189	0.44444	0.51342	0.97407 (max o	7	1906; 3508; 3509; 35	0.20485045	0.8868377		4.0
	ZINC31160839	-7.3	389.419	-1.9705	9	2	290.57	0.36673	134.58	-13.144	3609.116734805	0.27654	7.4131	-7.1255	0.55556	0.52531	0.95493 (max o	7	6882; 6860; 6644; 51	-0.6922646	-0.0789237		4.0
	ZINC35465107	-7.3	373.420	-1.7731	8	4	278.5	0.35806	139.51	-9.7968	3609.116734805	0.28718	7.2157	-6.1742	0.5	0.51159	0.95857 (max o	4	7092; 5241; 5642; 63	0.46776924	0.59938496		4.0
	ZINC72320476	-7.3	354.354	-0.5368	8	0	240.95	0.35725	89.52	-3.7722	3609.116734805	0.29866	5.9794	-1.7973	0.48	0.30982	0.96243 (max o	3	6918; 5285; 5688	0.79206824	0.4107873		4.0
	ZINC04268252	-7.3	419.448	-1.2708	8	2	284.75	0.36046	141.03	-0.21816	3609.116734805	0.24889	6.7134	-5.1059	0.4	0.42818	0.95579 (max o	3	5395; 5501; 5871	0.7940634	0.3859813		4.0
	ZINC01704128	-7.3	358.394	-4.462	9	2	245.75	0.51963	182.61	4.8789	3609.116734805	0.31111	9.9046	-14.342	0.58333	0.44402	0.90399 (max o	3	7324; 6719; 5415	-0.3231440	-0.8224774		4.0

Structure	Molecule Name	affinity	Total Molweight	cLogP	H-Acceptors	H-Donors	Total Surface Area	Relative PSA	Polar Surface Area	Druglikeness	Kd (nM) @ 20°C	LE from K...	LLE from Kd ...	LELP from Kd (n...	Shape Index	Molecular...	Neighbor Si...	Neig...	Neighbor	Neighbor ...	Neighbor ...	Ring Systems	frequency
	ZINC05273538	-7.3	353.354	-4.454	10	3	258.34	0.46923	161.73	9.1102	3609.116734805	0.29866	9.8966	-14.913	0.48	0.53466	0.97118 (max o	7	7152; 6530; 6527; 65	0.06577056	0.78090686		4.0
	ZINC12481431	-7.3	368.476	-2.0818	8	2	304.56	0.29403	97.22	5.2497	3609.116734805	0.28718	7.5244	-7.2492	0.57692	0.65391	0.93686 (max o	5	1796; 3761; 5911; 59	-0.0847917	-0.2770324		4.0
	ZINC40313291	-7.3	374.372	-6.1305	9	2	287.03	0.40905	141.95	-7.2883	3609.116734805	0.27654	11.573	-22.169	0.40741	0.40528	0.95174 (max o	3	6891; 5646; 5656	0.29560986	-0.5698107		4.0
	ZINC04293779	-7.3	410.338	-0.3298	13	2	285.6	0.51106	164.15	4.6806	3609.116734805	0.25747	5.7724	-1.2809	0.41379	0.43305	0.93975 (max o	3	7146; 5787; 6151	-0.9032063	-0.0075535		4.0
	ZINC20465663	-7.3	352.477	-3.7011	7	4	254.67	0.20042	86.92	3.948	3609.116734805	0.29866	9.1437	-12.392	0.56	0.44113	0.90377 (max o	3	5150; 5153; 5155	-0.5458368	0.19104454		4.0
	ZINC36370068	-7.3	414.480	-4.6842	8	1	334.85	0.27669	102.1	-5.4264	3609.116734805	0.24889	10.127	-18.821	0.5	0.51885	0.94241 (max o	3	7269; 5246; 6012	0.33013958	-0.5862093		4.0
	ZINC05273792	-7.3	420.259	-10.349	14	7	261.47	0.67801	251.53	-34.627	3609.116734805	0.27654	15.792	-37.424	0.48148	0.56756	0.94605 (max o	3	7154; 6160; 6161	-0.5496968	-0.7755098		4.0
	ZINC31169331	-7.3	351.417	0.013601	6	3	277.6	0.30357	113.35	-14.742	3609.116734805	0.29866	5.429	0.045538	0.6	0.45193	0.93007 (max o	3	7258; 7078; 6312	0.6762722	-0.6058934		4.0
	ZINC04277777	-7.3	441.503	-0.0907	10	1	322.73	0.32925	130.7	6.7268	3609.116734805	0.24889	5.5333	-0.36442	0.5	0.56636	0.8956 (max of	5	7069; 7061; 3219; 54	0.00567909	0.44425124		4.0
	ZINC04293459	-7.3	441.388	-0.0395	12	1	324.96	0.43319	156.67	2.0065	3609.116734805	0.24086	5.4821	-0.164	0.41935	0.40801	0.95521 (max o	5	7781; 7703; 7593; 73	0.32207817	0.41552806		4.0
	ZINC12480668	-7.3	352.517	-0.1077	5	1	305.07	0.20159	64.88	-1.4459	3609.116734805	0.29866	5.5503	-0.36061	0.52	0.61691	0.93007 (max o	4	7375; 7236; 7033; 70	-0.3334985	-0.1809703		4.0
	ZINC12654041	-7.3	383.372	-5.5025	9	1	279.03	0.42325	166.92	-4.6692	3609.116734805	0.27654	10.945	-19.898	0.40741	0.58806	0.98371 (max o	3	7761; 7584; 7181	0.01561428	-0.7037048		4.0
	ZINC35618517	-7.3	396.355	-2.075	12	1	279.99	0.46498	145.19	4.6603	3609.116734805	0.26666	7.5176	-7.7813	0.42857	0.41668	0.9428 (max of	4	7533; 7441; 6650; 65	-0.8014265	-0.0564353		4.0
	ZINC24718520	-7.3	396.355	-2.075	12	1	279.99	0.46498	145.19	4.6603	3609.116734805	0.26666	7.5176	-7.7813	0.42857	0.41611	0.93385 (max o	3	7533; 7441; 6649	-0.8022516	-0.0973406		4.0
	ZINC35457092	-7.3	389.419	-1.9705	9	2	290.57	0.36673	134.58	-13.144	3609.116734805	0.27654	7.4131	-7.1255	0.55556	0.52531	0.94605 (max o	8	7087; 6882; 6860; 51	-0.7123683	-0.0456010		4.0
	ZINC35464284	-7.3	357.334	-5.9476	9	1	269.19	0.43872	166.92	-6.2936	3609.116734805	0.29866	11.39	-19.914	0.64	0.50595	0.90896 (max o	3	7802; 7090; 6646	0.82569	0.13692284		4.0
	ZINC35464278	-7.3	357.334	-5.9476	9	1	269.19	0.43872	166.92	-6.2936	3609.116734805	0.29866	11.39	-19.914	0.64	0.50595	0.90896 (max o	3	7802; 7090; 6647	0.84267783	0.16947955		4.0
	ZINC15676322	-7.3	416.460	-2.4247	8	4	324.72	0.2986	127.39	-0.95455	3609.116734805	0.24086	7.8673	-10.067	0.58065	0.44159	0.93686 (max o	3	1523; 4266; 5894	0.17848457	-0.7547704		3.0
	ZINC08643313	-7.3	390.455	-1.438	9	3	284.04	0.4148	150.7	1.2761	3609.116734805	0.28718	6.8806	-5.0074	0.65385	0.62764		0		-0.2437861	0.95899016		3.0
	ZINC04222718	-7.3	411.405	-0.394	10	1	303.54	0.35429	110.78	-0.3106	3609.116734805	0.25747	5.8366	-1.5303	0.58621	0.39721	0.94558	1	5391	-0.8890760	-0.3031629		3.0
	ZINC03841696	-7.3	364.397	-0.2876	8	1	271.58	0.29859	86.33	0.56964	3609.116734805	0.28718	5.7302	-1.0015	0.65385	0.40883		0		-0.2614991	0.93542266		3.0
	ZINC04277822	-7.3	435.475	-0.1514	10	1	335.66	0.30007	114.48	5.0031	3609.116734805	0.24086	5.594	-0.62859	0.51613	0.57419	0.94241 (max o	4	7229; 5339; 5403; 61	0.02495160	0.41328895		3.0
	ZINC13118895	-7.3	363.412	-2.4958	8	3	270.69	0.27866	108.06	6.8934	3609.116734805	0.28718	7.9384	-8.6908	0.57692	0.48181	0.88286 (max o	4	6799; 3378; 3379; 54	-0.0442902	-0.8570275		3.0
	ZINC08298377	-7.3	363.465	-1.8415	8	3	281.16	0.41019	125.61	-0.75333	3609.116734805	0.29866	7.2841	-6.1658	0.64	0.46752	0.9654 (max of	4	3719; 6173; 6472; 64	0.04408745	-0.1716004		3.0
	ZINC08297739	-7.3	363.489	-3.5426	8	3	281.05	0.39128	121.99	0.94395	3609.116734805	0.29866	8.9852	-11.862	0.64	0.45703	0.9654 (max of	4	7162; 4592; 6173; 64	0.04874456	-0.1365830		3.0
	ZINC62001270	-7.3	384.863	-3.7107	7	3	281.63	0.37379	135.66	2.818	3609.116734805	0.29866	9.1533	-12.424	0.52	0.52089	0.91304 (max o	5	7448; 6904; 3093; 52	0.62157094	0.36357158		3.0
	ZINC67902530	-7.3	368.381	0.2665	8	2	256.64	0.35957	111.52	3.4604	3609.116734805	0.28718	5.1761	0.928	0.38462	0.28721	0.95257 (max o	2	7361; 6363	0.7875773	0.16526696		3.0
	ZINC00819933	-7.3	424.492	1.3414	9	0	317.79	0.29044	110.29	0.56195	3609.116734805	0.24889	4.1012	5.3896	0.43333	0.60286	0.85189	1	7617	0.8299844	0.35754478		3.0
	ZINC08298382	-7.3	363.465	-1.8415	8	3	281.16	0.41019	125.61	-0.75333	3609.116734805	0.29866	7.2841	-6.1658	0.64	0.46752	0.9654 (max of	4	7162; 6984; 5443; 64	0.01716666	-0.1435668		3.0
	ZINC12483124	-7.3	382.435	-2.8267	9	3	272.32	0.32385	112.22	7.0396	3609.116734805	0.27654	8.2693	-10.222	0.59259	0.46562	0.93033 (max o	2	7402; 7211	0.3977729	0.10043278		3.0
	ZINC12483562	-7.3	391.408	0.1628	6	3	289.93	0.24885	64.39	-8.0301	3609.116734805	0.27654	5.2798	0.5887	0.55556	0.61144	0.85614	1	7173	0.5402981	-0.1472075		3.0
	ZINC20465230	-7.3	363.456	-0.1231	7	4	275.75	0.27456	94.06	5.2639	3609.116734805	0.28718	5.5657	-0.42866	0.53846	0.50214	0.89885 (max o	3	7635; 3001; 4309	-0.6416334	0.554563		3.0
	ZINC35882820	-7.3	371.301	-1.2117	12	1	262.31	0.50215	149.9	5.0025	3609.116734805	0.28718	6.6543	-4.2194	0.46154	0.45362	0.95857 (max o	5	7744; 7700; 7493; 68	0.40014789	-0.3384260		3.0
	ZINC20463857	-7.3	427.543	-2.2133	7	3	328.57	0.21907	72.21	2.2729	3609.116734805	0.24086	7.6559	-9.1892	0.54839	0.44957	0.93148 (max o	5	7212; 3839; 4728; 55	-0.5790051	-0.5901612		2.0
	ZINC13523524	-7.3	362.389	-2.3544	10	4	267.39	0.44452	150.28	9.5534	3609.116734805	0.28718	7.797	-8.1984	0.46154	0.49291	0.96998 (max o	8	6530; 4252; 5375; 53	0.0410313	0.7453476		2.0
	ZINC13523524	-7.3	362.389	-2.3544	10	4	267.39	0.44452	150.28	9.5534	3609.116734805	0.28718	7.797	-8.1984	0.46154	0.49291	0.96998 (max o	9	7152; 6530; 6529; 42	0.05571038	0.7637599		2.0
	ZINC13523524	-7.3	363.397	-4.3322	10	5	256.42	0.42076	151.53	8.9317	3609.116734805	0.28718	9.7748	-15.085	0.46154	0.48615	0.9615 (max of	8	6530; 4254; 5379; 53	0.02216891	0.7744647		2.0
	ZINC13523524	-7.3	363.397	-4.3322	10	5	256.42	0.42076	151.53	9.0745	3609.116734805	0.28718	9.7748	-15.085	0.46154	0.49291	0.96998 (max o	8	4255; 5379; 5380; 62	0.00318224	0.76521176		2.0
	ZINC12377657	-7.3	365.412	-2.6013	10	2	257.76	0.31778	115.25	6.3932	3609.116734805	0.28718	8.0439	-9.0582	0.46154	0.45301	0.92032	1	4459	0.5558341	0.7448592		2.0
	ZINC05398514	-7.3	382.459	0.2602	9	2	295.38	0.31207	108.05	-2.5235	3609.116734805	0.27654	5.1824	0.94091	0.51852	0.59651	0.92721 (max o	3	4917; 6455; 6456	-0.0551353	0.32879457		2.0
	ZINC03881687	-7.3	369.476	-0.9482	6	3	267.09	0.28687	110.05	-6.0776	3609.116734805	0.28718	6.3908	-3.3018	0.42308	0.46464	0.93686 (max o	3	4961; 5368; 6105	-0.8770829	-0.3463440		2.0
	ZINC01531508	-7.3	445.466	-0.3709	9	1	353.23	0.30561	107.79	-0.53287	3609.116734805	0.23333	5.8135	-1.5896	0.4375	0.47434	0.90873	1	4977	0.25499427	-0.9106037		2.0
	ZINC08740536	-7.3	386.371	0.8103	12	5	275.91	0.50121	176.82	1.3234	3609.116734805	0.26666	4.6323	3.0387	0.5	0.33184	0.98451 (max o	12	6759; 3745; 3753; 46	-0.2269209	-0.5916857		2.0

Structure	Molecule Name	affinity	Total Molweight	cLogP	H-Acceptors	H-Donors	Total Surface Area	Relative PSA	Polar Surface Area	Druglikeness	Kd (nM) @ 20°C	LE from K...	LLE from Kd ...	LELP from Kd (n...	Shape Index	Molecular...	Neighbor Si...	Neig...	Neighbor	Neighbor ...	Neighbor ...	Ring Systems	frequency
	ZINC35456926	-7.3	404.367	-3.1441	11	6	270.25	0.4871	175.37	-5.0325	3609.116734805	0.26666	8.5867	-11.79	0.46429	0.38571	0.95148 (max o	2	6881; 6004	0.79938763	-0.0265946		2.0
	ZINC20111611	-7.3	375.380	-2.814	9	1	276.47	0.3438	95.81	2.1248	3609.116734805	0.27654	8.2566	-10.176	0.40741	0.34903	0.90377 (max o	3	2214; 5122; 6547	-0.3248655	0.52233356		2.0
	ZINC20761881	-7.3	360.369	-0.629	9	2	269.55	0.36023	109.33	5.2698	3609.116734805	0.28718	6.0716	-2.1903	0.61538	0.52626	0.92702 (max o	4	7253; 5170; 6275; 62	-0.0414396	-0.3786523		2.0
	ZINC31167966	-7.3	398.494	0.9599	7	4	288.68	0.29597	116.45	-3.8942	3609.116734805	0.26666	4.4827	3.5997	0.46429	0.43645	0.96519 (max o	6	1919; 1920; 2271; 39	-0.6876625	0.02444444		2.0
	ZINC13660239	-7.3	404.367	-3.1441	11	6	270.25	0.4871	175.37	-5.0325	3609.116734805	0.26666	8.5867	-11.79	0.46429	0.39392	0.95148 (max o	3	6535; 6534; 5104	0.39090896	0.6667883		2.0
	ZINC31160874	-7.3	351.717	-0.1115	7	1	248.22	0.34848	112.96	-3.798	3609.116734805	0.31111	5.5541	-0.3584	0.45833	0.48588	0.97246	1	5500	-0.1555814	0.9775031		2.0
	ZINC20463854	-7.3	398.505	-3.1442	7	3	305.07	0.23913	75.87	2.1693	3609.116734805	0.25747	8.5868	-12.212	0.58621	0.4512	0.91964 (max o	6	7212; 6558; 3839; 47	-0.5588869	-0.6242234		2.0
	ZINC20463854	-7.3	397.497	-1.0345	7	2	291.03	0.20242	74.67	4.5421	3609.116734805	0.25747	6.4771	-4.018	0.58621	0.44726	0.97415 (max o	7	7213; 2986; 4646; 47	0.38857606	-0.0290412		2.0
	ZINC05398196	-7.3	370.404	-1.06	10	1	277.96	0.33915	108.49	0.89201	3609.116734805	0.28718	6.5026	-3.6911	0.5	0.62244	0.93361 (max o	3	6457; 4966; 6455	-0.0352158	0.34043595		2.0
	ZINC20111609	-7.3	375.380	-2.814	9	1	276.47	0.3438	95.81	2.1248	3609.116734805	0.27654	8.2566	-10.176	0.40741	0.34903	0.90377 (max o	3	6548; 2212; 5903	-0.3526972	0.5476365		2.0
	ZINC36370068	-7.3	414.480	-4.6842	8	1	334.85	0.27669	102.1	-4.9417	3609.116734805	0.24889	10.127	-18.821	0.5	0.50324	0.95579 (max o	2	7268; 6013	0.79648024	-0.4987304		2.0
	ZINC20465189	-7.3	355.452	-3.9254	6	4	276.27	0.23557	73.98	0.56601	3609.116734805	0.29866	9.368	-13.143	0.6	0.45789	0.91964 (max o	2	7047; 6262	-0.3948168	0.28930575		2.0
	ZINC20465189	-7.3	354.444	-1.8157	6	3	262.23	0.19464	72.78	4.8714	3609.116734805	0.29866	7.2583	-6.0794	0.6	0.45789	0.91964 (max o	6	2228; 2229; 2230; 25	-0.4512335	0.27731752		2.0
	ZINC31157972	-7.3	364.393	1.4512	7	2	267.37	0.31911	110.13	-16.562	3609.116734805	0.28718	3.9914	5.0533	0.42308	0.31559	0.97791 (max o	3	3896; 5601; 6292	-0.783883	0.18301095		2.0
	ZINC31168488	-7.3	374.472	0.91	7	5	276.92	0.30875	119.61	-10.488	3609.116734805	0.28718	4.5326	3.1688	0.53846	0.49164	0.99159 (max o	7	3526; 4843; 5211; 52	0.71987635	0.35071877		2.0
	ZINC03844450	-7.3	388.371	-2.3522	8	0	306.14	0.30914	117.18	-12.899	3609.116734805	0.26666	7.7948	-8.8208	0.64286	0.5522	0.80974	1	6938	-0.9533872	-0.0112541		2.0
	ZINC04025190	-7.3	364.349	1.4871	8	1	258.07	0.35734	108.36	-3.926	3609.116734805	0.28718	3.9555	5.1783	0.38462	0.42992	0.95559 (max o	4	7762; 7585; 7468; 74	-0.5444227	0.2640874		2.0
	ZINC04043963	-7.3	379.185	-1.1144	6	0	247.41	0.2706	86.74	-6.4948	3609.116734805	0.32463	6.557	-3.4328	0.52174	0.56409	0.94762	1	6420	0.68862504	-0.4028378		2.0
	ZINC04043962	-7.3	379.185	-1.1144	6	0	247.41	0.2706	86.74	-6.4948	3609.116734805	0.32463	6.557	-3.4328	0.52174	0.56671	0.94762	1	6421	0.7072747	-0.4101187		2.0
	ZINC04096827	-7.3	372.488	-1.5166	8	4	280.72	0.42658	163.3	-12.912	3609.116734805	0.29866	6.9592	-5.078	0.72	0.50898	0.94351 (max o	3	7316; 7141; 7140	-0.6676451	-0.6912323		2.0
	ZINC06142300	-7.3	398.391	-0.9735	10	3	267.32	0.43345	150.85	-15.529	3609.116734805	0.27654	6.4161	-3.5203	0.51852	0.48357	0.94302 (max o	3	3994; 4000; 6465	-0.7724398	-0.5659275		2.0
	ZINC06142299	-7.3	398.391	-0.9735	10	3	267.32	0.43345	150.85	-15.529	3609.116734805	0.27654	6.4161	-3.5203	0.51852	0.48357	0.93996 (max o	3	3994; 4000; 6466	-0.7410195	-0.5844797		2.0
	ZINC08740475	-7.3	391.411	1.6778	11	5	273.09	0.54077	192.17	3.1748	3609.116734805	0.27654	3.7648	6.0671	0.48148	0.29326	0.96885 (max o	4	5027; 5833; 6193; 64	-0.3170884	0.24627355		2.0
	ZINC08740472	-7.3	391.411	1.6778	11	5	273.09	0.54077	192.17	3.1748	3609.116734805	0.27654	3.7648	6.0671	0.48148	0.29326	0.96885 (max o	5	7338; 6483; 5027; 58	-0.3010852	0.26013976		2.0
	ZINC12494159	-7.3	378.309	-5.9441	12	6	253.47	0.58121	198.07	0.5748	3609.116734805	0.28718	11.387	-20.698	0.42308	0.46296	0.95257 (max o	3	5854; 5855; 6508	0.16545191	-0.9630710		2.0
	ZINC12494159	-7.3	378.309	-5.7714	12	6	253.58	0.58139	201.56	1.738	3609.116734805	0.28718	11.214	-20.097	0.42308	0.44321	0.95257 (max o	3	6509; 5854; 5855	0.15737942	-0.9420991		2.0
	ZINC20410608	-7.3	351.358	-4.3613	9	4	262.28	0.39698	136.56	3.4248	3609.116734805	0.29866	9.8039	-14.603	0.64	0.5273	0.88798	1	6524	0.6756291	0.7094175		2.0
	ZINC13118895	-7.3	363.412	-2.9034	8	3	297.05	0.33957	104.16	2.2617	3609.116734805	0.28718	8.346	-10.11	0.57692	0.56332	0.88798	1	6549	0.6624584	0.70449173		2.0
	ZINC13660246	-7.3	404.367	-3.1441	11	6	270.25	0.4871	175.37	-5.0325	3609.116734805	0.26666	8.5867	-11.79	0.46429	0.37662	0.95148 (max o	3	5104; 6533; 6534	0.38536796	0.6475549		2.0
	ZINC20463944	-7.3	376.499	-3.2343	7	4	293.48	0.25886	78.77	3.7681	3609.116734805	0.27654	8.6769	-11.696	0.55556	0.47977	0.91109 (max o	2	3867; 3869	-0.0849439	-0.964011		2.0
	ZINC20463944	-7.3	376.499	-6.6766	7	4	308.7	0.2954	82.8	0.14578	3609.116734805	0.27654	12.119	-24.143	0.55556	0.46914	0.86499 (max o	2	7627; 7403	0.61193585	0.09884657		2.0
	ZINC20463944	-7.3	375.491	-2.0096	7	4	297.2	0.26814	74.19	4.2676	3609.116734805	0.27654	7.4522	-7.2669	0.55556	0.50645	0.90776	1	3868	0.44773617	0.6369345		2.0
	ZINC20463944	-7.3	376.499	-3.3759	7	5	311.24	0.30115	75.39	1.77	3609.116734805	0.27654	8.8185	-12.208	0.55556	0.49625		0		0.51716703	0.8258641		2.0
	ZINC20465334	-7.3	386.918	-2.8817	6	4	297.54	0.2404	79.97	-1.455	3609.116734805	0.28718	8.3243	-10.035	0.53846	0.42675	0.92368 (max o	2	6824; 5593	0.29574177	0.887081		2.0
	ZINC31501431	-7.3	384.364	-3.7143	11	3	279.49	0.44971	164.84	1.8978	3609.116734805	0.27654	9.1569	-13.431	0.48148	0.51589	0.96343 (max o	2	2075; 6628	-0.6500054	0.2988106		2.0
	ZINC31501427	-7.3	384.364	-3.7143	11	3	279.49	0.44971	164.84	1.8978	3609.116734805	0.27654	9.1569	-13.431	0.48148	0.51866	0.96343 (max o	2	2075; 6629	-0.6397072	0.279921		2.0
	ZINC34302823	-7.3	362.377	-0.1664	7	1	251.77	0.37526	97.89	-7.4006	3609.116734805	0.28718	5.609	-0.57943	0.42308	0.22723	0.98517 (max o	3	4142; 5622; 6633	0.54333544	-0.2215461		2.0
	ZINC34302822	-7.3	362.377	-0.1664	7	1	251.77	0.37526	97.89	-7.4006	3609.116734805	0.28718	5.609	-0.57943	0.42308	0.22201	0.98517 (max o	3	6634; 4142; 5622	0.5226229	-0.2303551		2.0
	ZINC35618517	-7.3	395.347	-4.2446	12	0	281.28	0.46743	151.51	2.1322	3609.116734805	0.26666	9.6872	-15.917	0.42857	0.40389	0.9428 (max of	2	7442; 6649	-0.766885	-0.0530431		2.0
	ZINC35882820	-7.3	370.293	-3.3813	12	0	263.6	0.50459	156.22	3.7857	3609.116734805	0.28718	8.8239	-11.774	0.46154	0.44595	0.95559 (max o	5	7745; 7432; 6890; 67	0.43569142	-0.3563849		2.0
	ZINC38144766	-7.3	350.350	-1.2389	9	2	227.36	0.42083	124.77	5.3027	3609.116734805	0.29866	6.6815	-4.1481	0.4	0.3266	0.81741	1	7098	0.8768272	-0.4272698		2.0
	ZINC67870731	-7.3	406.617	-0.3309	6	3	344.91	0.26972	85.95	3.3081	3609.116734805	0.26666	5.7735	-1.2409	0.71429	0.64469	0.85045 (max o	2	6905; 5573	0.73386157	-0.5747446		2.0
	ZINC67912206	-7.3	362.373	-1.8885	9	5	259.34	0.41852	145.91	-5.845	3609.116734805	0.29866	7.3311	-6.3232	0.48	0.59862	0.96243 (max o	3	6044; 6360; 6672	-0.9306999	0.09897511		2.0

Structure	Molecule Name	affinity	Total Molweight	cLogP	H-Acceptors	H-Donors	Total Surface Area	Relative PSA	Polar Surface Area	Druglikeness	Kd (nM) @ 20°C	LE from K...	LLE from Kd ...	LELP from Kd (n...	Shape Index	Molecular...	Neighbor Si...	Neig...	Neighbor	Neighbor ...	Neighbor ...	Ring Systems	frequency
	ZINC67912203	-7.3	362.373	-1.8885	9	5	259.34	0.41852	145.91	-5.845	3609.116734805	0.29866	7.3311	-6.3232	0.48	0.59862	0.96243 (max o	3	6044; 6360; 6673	-0.9480761	0.09540572		2.0
	ZINC03830449	-7.3	426.449	-2.0903	10	2	287.87	0.53694	204.63	9.467	3609.116734805	0.26666	7.5329	-7.8387	0.53571	0.53033		0		-0.8132282	0.34267956		1.0
	ZINC03830486	-7.3	423.381	-2.8488	12	2	292.92	0.54564	201.89	13.42	3609.116734805	0.25747	8.2914	-11.065	0.51724	0.38037	0.94823 (max o	5	3591; 3619; 4029; 40	-0.2953389	0.8483084		1.0
	ZINC03838656	-7.3	362.520	-3.538	7	3	309.49	0.28592	68.69	-1.8964	3609.116734805	0.28718	8.9806	-12.32	0.61538	0.48198	0.96721 (max o	3	7292; 4916; 5444	0.05070441	-0.1160495		1.0
	ZINC03999089	-7.3	364.405	-0.2678	10	4	257.77	0.41859	142.78	2.2674	3609.116734805	0.28718	5.7104	-0.93253	0.46154	0.37603	0.91999 (max o	2	3743; 3752	-0.8461995	0.13613245		1.0
	ZINC03999641	-7.3	375.218	-0.2228	7	5	236.63	0.36031	111.05	-5.1145	3609.116734805	0.33939	5.6654	-0.65647	0.5	0.4523	0.91723 (max o	2	6419; 6412	0.4532545	0.54805493		1.0
	ZINC03999642	-7.3	375.218	-0.2228	7	5	236.63	0.36031	111.05	-5.1145	3609.116734805	0.33939	5.6654	-0.65647	0.5	0.4491	0.93686 (max o	3	6487; 6419; 6411	0.46453843	0.563331		1.0
	ZINC05397673	-7.3	367.444	0.3196	8	1	283.92	0.28431	96.02	-3.3367	3609.116734805	0.28718	5.123	1.1129	0.5	0.55116	0.93361 (max o	4	7158; 6457; 3691; 64	-0.0359050	0.30001897		1.0
	ZINC05834616	-7.3	382.435	-2.6731	9	2	297.74	0.37133	131.17	8.9795	3609.116734805	0.27654	8.1157	-9.6662	0.55556	0.56122	0.93686	1	6106	-0.9320143	-0.1701190		1.0
	ZINC09058949	-7.3	389.363	0.5584	10	2	283.25	0.38351	123.27	7.9404	3609.116734805	0.26666	4.8842	2.094	0.46429	0.48212	0.88589 (max o	2	6490; 6489	0.1617656	0.9408254		1.0
	ZINC09058949	-7.3	388.355	-1.6112	10	1	284.54	0.38631	129.59	7.2831	3609.116734805	0.26666	7.0538	-6.0421	0.46429	0.47654	0.8991 (max of	2	6490; 6488	0.14239725	0.9321088		1.0
	ZINC09058949	-7.3	388.355	-1.6112	10	1	284.54	0.38631	129.59	7.6253	3609.116734805	0.26666	7.0538	-6.0421	0.46429	0.47654	0.8991 (max of	2	6488; 6489	0.14206378	0.94960386		1.0
	ZINC11616739	-7.3	382.416	-3.6789	8	1	252.44	0.52916	183.21	11.228	3609.116734805	0.29866	9.1215	-12.318	0.48	0.32631	0.91611 (max o	4	6978; 3594; 4889; 53	-0.4063682	-0.4533171		1.0
	ZINC12481172	-7.3	351.465	-1.5127	6	0	276.77	0.20761	72.91	1.9947	3609.116734805	0.29866	6.9553	-5.0649	0.52	0.54524	0.90599 (max o	3	6845; 6498; 4720	-0.2710313	-0.2122539		1.0
	ZINC12481179	-7.3	365.492	-1.1707	6	0	290.53	0.19778	72.91	-0.44856	3609.116734805	0.28718	6.6133	-4.0766	0.53846	0.57288	0.93361 (max o	4	6804; 4722; 5845; 64	-0.2485522	-0.1840944		1.0
	ZINC12489910	-7.3	376.519	-0.3736	6	2	287.01	0.19289	71.43	-0.51296	3609.116734805	0.27654	5.8162	-1.351	0.59259	0.50574		0		0.26421192	-0.9234423		1.0
	ZINC12530551	-7.3	367.468	-2.4864	8	3	294.93	0.31594	92.18	4.0137	3609.116734805	0.28718	7.929	-8.6581	0.61538	0.51413	0.96998 (max o	4	7499; 7363; 7362; 70	-0.2202744	0.06195267		1.0
	ZINC12603854	-7.3	356.397	-3.3273	9	5	284.47	0.41094	125.47	1.3132	3609.116734805	0.29866	8.7699	-11.141	0.64	0.57104		0		-0.8967166	0.14525509		1.0
	ZINC12891874	-7.3	383.468	0.0053	8	1	277.15	0.35436	121.32	3.0025	3609.116734805	0.28718	5.4373	0.018456	0.61538	0.54507	0.91693 (max o	2	6762; 5836	0.9045531	0.40542847		1.0
	ZINC13660242	-7.3	404.367	-3.1441	11	6	270.25	0.4871	175.37	-5.0325	3609.116734805	0.26666	8.5867	-11.79	0.46429	0.38495	0.95148 (max o	3	6535; 5104; 6533	0.40688524	0.6507675		1.0
	ZINC14770073	-7.3	353.413	0.5349	7	2	251.11	0.30198	96.3	6.0667	3609.116734805	0.29866	4.9077	1.791	0.36	0.22509	0.91964 (max o	3	2677; 3092; 6361	-0.5359283	0.53278667		1.0
	ZINC15707111	-7.3	357.409	-2.4926	7	1	265.66	0.22879	88.74	1.8862	3609.116734805	0.28718	7.9352	-8.6797	0.53846	0.50082	0.87803 (max o	2	1492; 6541	-0.1863004	0.42238602		1.0
	ZINC15707111	-7.3	356.401	-2.3037	7	0	276.63	0.25937	87.49	2.4642	3609.116734805	0.28718	7.7463	-8.0219	0.53846	0.50082	0.85984	1	6540	-0.1496554	0.4000214		1.0
	ZINC15707111	-7.3	358.417	-2.361	7	2	279.7	0.2675	89.94	1.0546	3609.116734805	0.28718	7.8036	-8.2214	0.53846	0.48125	0.87803	1	1493	0.12428512	-0.9700804		1.0
	ZINC17124164	-7.3	411.476	-0.0184	7	2	325.32	0.25624	88.35	0.07	3609.116734805	0.24889	5.461	-0.07393	0.46667	0.43971	0.85434 (max o	2	5117; 5118	-0.1616613	-0.9188974		1.0
	ZINC19796104	-7.3	358.504	0.2434	4	4	277.91	0.2205	84.17	-0.67707	3609.116734805	0.29866	5.1992	0.81496	0.52	0.43006		0		0.96977836	0.09243356		1.0
	ZINC20462732	-7.3	386.490	-2.7109	6	4	331.93	0.26994	75.62	0.71746	3609.116734805	0.26666	8.1535	-10.166	0.5	0.54375	0.83096	1	6551	-0.1139955	0.97365344		1.0
	ZINC20462732	-7.3	385.482	-0.6012	6	3	317.89	0.23769	74.42	4.1537	3609.116734805	0.26666	6.0438	-2.2545	0.5	0.54375	0.83096	1	6550	-0.1412984	0.97302216		1.0
	ZINC20463004	-7.3	351.445	-2.5925	7	2	278.3	0.32271	107.97	5.2786	3609.116734805	0.29866	8.0351	-8.6803	0.48	0.53367	0.89162 (max o	5	6803; 5907; 5908; 62	-0.1268926	-0.3307529		1.0
	ZINC20463665	-7.3	363.436	-2.419	8	2	261.28	0.27538	91.74	6.2154	3609.116734805	0.28718	7.8616	-8.4234	0.57692	0.45523	0.96998 (max o	5	7384; 6765; 5850; 58	0.37743056	-0.1886573		1.0
	ZINC20463850	-7.3	400.521	-3.234	7	3	307.77	0.22371	67.91	4.2272	3609.116734805	0.25747	8.6766	-12.561	0.55172	0.44521	0.8886 (max of	3	7212; 6556; 4730	-0.5950776	-0.621558		1.0
	ZINC20463850	-7.3	399.513	-1.1243	7	2	293.73	0.1866	66.71	6.1176	3609.116734805	0.25747	6.5669	-4.3667	0.55172	0.44113	0.90934 (max o	5	7626; 7213; 6768; 47	0.37126526	0.03893025		1.0
	ZINC20463966	-7.3	371.500	-2.1257	8	4	296.52	0.3042	87.5	-0.83502	3609.116734805	0.28718	7.5683	-7.4021	0.57692	0.55228	0.85446	1	5055	-0.5894725	-0.2548842		1.0
	ZINC20463988	-7.3	421.519	-0.3899	7	2	312.33	0.21237	84.32	4.3573	3609.116734805	0.24086	5.8325	-1.6188	0.58065	0.46034	0.89093 (max o	3	7850; 7848; 5921	0.66706944	0.54299736		1.0
	ZINC20463988	-7.3	421.519	-0.5315	7	3	330.09	0.25475	80.94	1.0839	3609.116734805	0.24086	5.9741	-2.2067	0.58065	0.49104	0.84666	1	5050	0.6342938	-0.0354048		1.0
	ZINC20464820	-7.3	357.856	-1.4199	6	3	258.74	0.22219	78.77	1.3672	3609.116734805	0.31111	6.8625	-4.564	0.54167	0.45795	0.91204 (max o	3	6257; 6262; 6263	-0.3972691	0.24629657		1.0
	ZINC20464864	-7.3	414.504	-0.2049	8	3	312.55	0.23891	90.2	7.5713	3609.116734805	0.24889	5.6475	-0.82327	0.53333	0.45762	0.92835 (max o	2	6814; 1864	-0.3977120	0.18227863		1.0
	ZINC20464864	-7.3	415.512	-2.3146	8	4	326.59	0.27162	91.4	5.7493	3609.116734805	0.24889	7.7572	-9.2998	0.53333	0.46161	0.93405 (max o	3	6815; 1865; 5927	-0.3115242	-0.5844717		1.0
	ZINC20465137	-7.3	391.512	-0.9337	8	3	270.36	0.38356	138.96	8.026	3609.116734805	0.29866	6.3763	-3.1263	0.52	0.53718	0.91204 (max o	2	7888; 6574	0.18733887	-0.9603544		1.0
	ZINC20465137	-7.3	392.519	-3.0434	8	4	284.4	0.41399	140.16	4.141	3609.116734805	0.29866	8.486	-10.19	0.52	0.53718	0.87823 (max o	2	7888; 6573	0.20376648	-0.9644735		1.0
	ZINC20465248	-7.3	364.484	-3.6171	6	4	283.88	0.25197	79.97	-4.2363	3609.116734805	0.28718	9.0597	-12.595	0.53846	0.39826	0.89253 (max o	2	6827; 6588	0.51997465	0.74243283		1.0
	ZINC20465248	-7.3	363.476	-2.3924	6	4	287.6	0.26165	75.39	-2.1711	3609.116734805	0.28718	7.835	-8.3308	0.53846	0.46409	0.89424 (max o	5	7636; 6828; 6589; 30	0.3168076	-0.0504090		1.0
	ZINC20465248	-7.3	363.476	-1.5074	6	3	269.84	0.21305	78.77	-1.1033	3609.116734805	0.28718	6.95	-5.249	0.53846	0.39355	0.88538	1	6829	-0.4024716	0.7850393		1.0

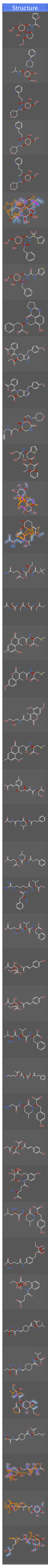

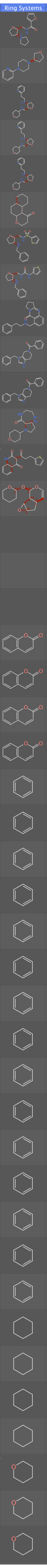

Structure	Molecule Name	affinity	Total Molweight	cLogP	H-Acceptors	H-Donors	Total Surface Area	Relative PSA	Polar Surface Area	Druglikeness	Kd (nM) @ 20°C	LE from K...	LLE from Kd ...	LELP from Kd (n...	Shape Index	Molecular...	Neighbor Si...	Neig...	Neighbor	Neighbor ...	Neighbor ...	Ring Systems	frequency
	ZINC20465271	-7.3	355.409	-2.9919	8	4	279.52	0.36634	105.6	1.9506	3609.116734805	0.29866	8.4345	-10.018	0.52	0.46559	0.9594 (max of	2	7522; 6823	-0.8042228	-0.5564203		1.0
	ZINC20465663	-7.3	351.469	-1.7233	7	3	265.64	0.23344	85.67	3.948	3609.116734805	0.29866	7.1659	-5.77	0.56	0.46782	0.95631 (max o	6	6818; 5149; 5156; 55	-0.5020957	0.16177154		1.0
	ZINC20465915	-7.3	367.488	-3.4863	7	4	294.64	0.28	92.86	-3.9931	3609.116734805	0.28718	8.9289	-12.14	0.57692	0.5019	0.96998 (max o	4	6827; 5570; 5942; 65	0.5099716	0.76107323		1.0
	ZINC20465915	-7.3	366.480	-2.2616	7	4	298.36	0.28898	88.28	-2.1304	3609.116734805	0.28718	7.7042	-7.8753	0.57692	0.55821	0.91351 (max o	4	6828; 4757; 5158; 65	0.32785243	-0.0322102		1.0
	ZINC20465915	-7.3	366.480	-2.12	7	3	280.6	0.24398	91.66	0.47107	3609.116734805	0.28718	7.5626	-7.3822	0.57692	0.49556	0.96998 (max o	3	6829; 5571; 5943	-0.4250612	0.75383157		1.0
	ZINC29477570	-7.3	368.468	0.836	6	4	255.32	0.29547	107.22	-5.1021	3609.116734805	0.28718	4.6066	2.9111	0.38462	0.43107	0.93686	1	3877	0.08676947	0.9632655		1.0
	ZINC30879688	-7.3	413.538	-0.9603	7	4	306.08	0.37307	136.92	2.4394	3609.116734805	0.27654	6.4029	-3.4725	0.51852	0.55401	0.93686 (max o	3	7399; 5569; 5572	0.2479666	-0.0998392		1.0
	ZINC30879721	-7.3	392.498	-0.8577	7	5	306.71	0.359	123.5	3.07	3609.116734805	0.27654	6.3003	-3.1015	0.55556	0.54489	0.9399 (max of	3	7400; 3853; 4322	0.32262695	-0.2067706		1.0
	ZINC35365966	-7.3	378.407	-2.2783	7	2	281.43	0.28419	105.06	4.7894	3609.116734805	0.26666	7.7209	-8.5437	0.5	0.50321		0		0.796449	0.5168455		1.0
	ZINC35442244	-7.3	449.462	-0.9066	9	1	325.65	0.25739	115.04	1.9801	3609.116734805	0.22626	6.3492	-4.0069	0.48485	0.51099	0.89672 (max o	6	7196; 7016; 6769; 14	-0.2443697	0.39594343		1.0
	ZINC35442244	-7.3	448.454	-0.7177	9	0	336.62	0.28159	113.79	2.5645	3609.116734805	0.22626	6.1603	-3.172	0.48485	0.51099	0.89672 (max o	5	7195; 7017; 6770; 63	-0.2453095	0.37527886		1.0
	ZINC35442532	-7.3	363.480	-2.6706	7	3	299.73	0.26737	75.11	0.95039	3609.116734805	0.28718	8.1132	-9.2995	0.61538	0.49345	0.88991 (max o	3	4401; 4825; 6284	-0.1243388	0.615537		1.0
	ZINC68565264	-7.3	434.511	-1.1599	8	4	316.97	0.35899	155.41	-7.2548	3609.116734805	0.24889	6.6025	-4.6604	0.43333	0.57119	0.96589 (max o	5	7096; 7093; 3511; 35	-0.6436317	-0.1475521		1.0
	ZINC68590692	-7.3	362.330	-3.6103	10	6	234.68	0.53797	161.6	-4.3947	3609.116734805	0.29866	9.0529	-12.088	0.44	0.33846	0.9402 (max of	3	6907; 4438; 6050	0.55345786	0.580437		1.0
	ZINC67913523	-7.3	429.576	0.7542	7	2	335.06	0.31466	148.83	-1.2855	3609.116734805	0.25747	4.6884	2.9293	0.44828	0.56223	0.97176 (max o	3	7571; 4864; 5673	0.7712661	0.26708966		0.0
	ZINC05317701	-7.3	358.413	-1.7779	9	3	283.96	0.40368	150.65	-57.945	3609.116734805	0.29866	7.2205	-5.9528	0.52	0.64764	0.97118 (max o	5	7763; 7681; 7325; 69	-0.2611337	0.8294501		0.0
	ZINC04761071	-7.3	358.393	-2.0992	10	4	281.65	0.42975	142.7	-4.5887	3609.116734805	0.29866	7.5418	-7.0286	0.64	0.61193		0		-0.7609655	0.6480705		0.0
	ZINC20610198	-7.2	364.397	-1.2917	7	2	265.56	0.39709	141.06	3.9109	4285.064499754	0.29457	6.6597	-4.385	0.56	0.54145	0.94338 (max o	3	2601; 4016; 6689	0.00314268	0.16958617		172.0
	ZINC02120903	-7.2	364.397	-1.3599	7	1	269.21	0.38019	130.06	3.977	4285.064499754	0.29457	6.7279	-4.6165	0.6	0.55154	0.94364 (max o	7	7120; 6924; 3139; 40	0.00667430	0.14869338		172.0
	ZINC08764726	-7.2	351.354	-0.0802	8	3	262.9	0.35656	114.32	4.149	4285.064499754	0.29457	5.4482	-0.27226	0.6	0.52427	0.97118 (max o	4	5700; 5701; 5749; 61	0.00306788	0.08317269		172.0
	ZINC02118124	-7.2	378.424	-1.016	7	1	281.47	0.36363	130.06	3.977	4285.064499754	0.28324	6.384	-3.587	0.53846	0.53026	0.94338 (max o	4	7120; 6924; 6835; 63	-6.486456E	0.13052404		172.0
	ZINC35458103	-7.2	401.457	-1.5171	7	2	324.65	0.28412	129.59	5.3725	4285.064499754	0.25394	6.8851	-5.9742	0.55172	0.53691	0.97878 (max o	3	2661; 4413; 4414	0.8404321	0.21414508		134.0
	ZINC04792352	-7.2	378.404	-2.0163	9	3	300.73	0.3685	136.66	-13.036	4285.064499754	0.27275	7.3843	-7.3924	0.62963	0.59232	0.96064 (max o	2	5414; 6156	0.00227798	-0.4269471		134.0
	ZINC13541192	-7.2	392.407	-3.1842	9	2	309.3	0.36741	147.69	-12.211	4285.064499754	0.26301	8.5522	-12.107	0.57143	0.57069	0.92721 (max o	3	7189; 4893; 5797	-0.0689177	-0.4286957		134.0
	ZINC32124120	-7.2	436.555	-3.4532	10	6	332.52	0.33712	175.78	0.43345	4285.064499754	0.23756	8.8212	-14.536	0.48387	0.58045	0.96018	1	6307	0.45199725	-0.8252623		134.0
	ZINC04545847	-7.2	392.430	-4.3105	9	3	305.77	0.3863	166.1	2.1659	4285.064499754	0.26301	9.6785	-16.389	0.57143	0.5932	0.96253 (max o	6	6872; 5409; 6391; 64	-0.0293382	0.6084982		134.0
	ZINC31166582	-7.2	366.277	-4.9637	10	2	267.45	0.47456	173.32	-1.6285	4285.064499754	0.28324	10.332	-17.525	0.53846	0.54688	0.96438 (max o	3	5976; 6296; 6861	-0.5883682	0.7230711		134.0
	ZINC31166579	-7.2	366.277	-4.9637	10	2	267.45	0.47456	173.32	-1.6285	4285.064499754	0.28324	10.332	-17.525	0.53846	0.54688	0.96438 (max o	3	6862; 5976; 6296	-0.6037497	0.7337026		134.0
	ZINC34302722	-7.2	363.456	-1.1943	7	3	289.99	0.31311	125.97	3.2028	4285.064499754	0.28324	6.5623	-4.2165	0.53846	0.57355	0.94951 (max o	5	7430; 6309; 6391; 64	-0.0197023	0.5901494		134.0
	ZINC04899812	-7.2	422.480	-1.9134	10	4	334.72	0.37434	164.3	-14.455	4285.064499754	0.24548	7.2814	-7.7946	0.66667	0.55583	0.95174 (max o	2	7323; 6080	-0.0574047	-0.5158615		134.0
	ZINC01575534	-7.2	351.402	-2.1176	8	4	274.34	0.37873	146.2	3.2057	4285.064499754	0.29457	7.4856	-7.1887	0.56	0.59428	0.89765	1	6722	-0.2347073	0.74878323		134.0
	ZINC31168612	-7.2	350.278	-4.1042	9	1	263.19	0.43246	153.09	-6.0092	4285.064499754	0.29457	9.4722	-13.933	0.6	0.53405	0.96832 (max o	7	7897; 7798; 7644; 72	0.8375661	0.0781608		134.0
	ZINC49181823	-7.2	414.409	-3.3527	11	6	302.34	0.46881	204.09	0.26746	4285.064499754	0.25394	8.7207	-13.203	0.51724	0.61279	0.98727 (max o	2	7656; 6355	-0.3129603	0.75050086		134.0
	ZINC04099087	-7.2	407.509	-0.6312	8	4	323.86	0.32082	146.2	3.2057	4285.064499754	0.25394	5.9992	-2.4856	0.55172	0.59323	0.89765 (max o	2	7595; 6683	-0.2323374	0.76900184		134.0
	ZINC35464845	-7.2	382.411	-1.9852	9	4	290.9	0.39718	163.63	1.315	4285.064499754	0.27275	7.3532	-7.2784	0.62963	0.65945	0.98593 (max o	6	7562; 7419; 7265; 72	0.39300248	0.35872948		134.0
	ZINC49180797	-7.2	352.362	-1.4927	8	3	270.67	0.37337	135.99	2.2083	4285.064499754	0.29457	6.8607	-5.0673	0.56	0.61986	0.98434 (max o	5	7800; 7446; 7099; 70	0.3882519	0.402776		134.0
	ZINC12882480	-7.2	383.531	-0.4733	7	3	311.79	0.29122	125.97	3.7029	4285.064499754	0.27275	5.8413	-1.7353	0.62963	0.60187	0.94302 (max o	7	7782; 7501; 5754; 61	0.540425	-0.0237575		127.0
	ZINC08481023	-7.2	369.504	-0.9277	7	3	298.03	0.30467	125.97	3.0831	4285.064499754	0.28324	6.2957	-3.2753	0.61538	0.60405	0.94927 (max o	8	7782; 7501; 7331; 67	0.5277144	-0.0061019		127.0
	ZINC31170267	-7.2	395.426	-0.9065	8	4	297.67	0.34521	147.35	-8.9294	4285.064499754	0.26301	6.2745	-3.4466	0.46429	0.60852	0.95983 (max o	4	7260; 7248; 7079; 63	0.4533704	-0.5777834		127.0
	ZINC04084454	-7.2	359.489	-2.1239	7	3	279.76	0.38708	151.27	2.7818	4285.064499754	0.30685	7.4919	-6.9217	0.66667	0.58118	0.93007 (max o	6	7764; 7608; 7312; 71	0.59295744	0.10917295		127.0
	ZINC39205605	-7.2	357.287	-7.4085	12	8	236.1	0.6443	220.43	-5.5171	4285.064499754	0.30685	12.777	-24.144	0.54167	0.62085	0.94034 (max o	3	4846; 6349; 6350	0.47291666	-0.7618767		120.0
	ZINC31169924	-7.2	350.406	-0.369	8	6	260.04	0.37917	139.84	-3.8701	4285.064499754	0.30685	5.737	-1.2026	0.58333	0.62846	0.95053	1	6302	0.6908769	-0.4256042		120.0
	ZINC35466047	-7.2	418.525	1.5486	8	6	321.01	0.30716	139.84	-10.55	4285.064499754	0.25394	3.8194	6.0983	0.58621	0.69528	0.99279 (max o	3	6310; 6340; 6341	0.45435354	0.72338146		120.0

Structure	Molecule Name	affinity	Total Molweight	cLogP	H-Acceptors	H-Donors	Total Surface Area	Relative PSA	Polar Surface Area	Druglikeness	Kd (nM) @ 20°C	LE from K...	LLE from Kd ...	LELP from Kd (n...	Shape Index	Molecular...	Neighbor Si...	Neig...	Neighbor	Neighbor ...	Neighbor ...	Ring Systems	frequency
	ZINC12880496	-7.2	351.351	-1.2752	10	4	255.48	0.45734	143.78	-54.673	4285.064499754	0.30685	6.6432	-4.1558	0.58333	0.57517	0.9665 (max of	3	6225; 6773; 6774	0.8588445	-0.2993038		120.0
	ZINC12880487	-7.2	351.351	-1.2752	10	4	255.48	0.45734	143.78	-54.673	4285.064499754	0.30685	6.6432	-4.1558	0.58333	0.57805	0.94034 (max o	3	6225; 6773; 6775	0.85639083	-0.2782821		120.0
	ZINC12880471	-7.2	351.351	-1.2752	10	4	255.48	0.45734	143.78	-54.673	4285.064499754	0.30685	6.6432	-4.1558	0.58333	0.57805	0.94034 (max o	3	6774; 6225; 6775	0.8708732	-0.2636768		120.0
	ZINC31160515	-7.2	350.406	-0.6232	8	6	256.52	0.38438	139.84	-11.141	4285.064499754	0.30685	5.9912	-2.031	0.5	0.67588	0.9665 (max of	3	7240; 7073; 7074	-0.6814026	-0.3391298		120.0
	ZINC77257663	-7.2	363.381	-2.8728	9	5	272.09	0.41464	159.74	-12.771	4285.064499754	0.29457	8.2408	-9.7524	0.52	0.57825	0.98897 (max o	3	7279; 7112; 7111	-0.8361003	0.07707955		120.0
	ZINC16346988	-7.2	441.253	-8.694	14	3	273.28	0.7189	276.45	-38.679	4285.064499754	0.27275	14.062	-31.875	0.51852	0.49068	0.98593 (max o	8	721; 1490; 1792; 179	-0.5590202	0.4365252		67.0
	ZINC14709424	-7.2	372.369	-0.6515	9	5	272.55	0.38708	138.07	-4.1548	4285.064499754	0.28324	6.0195	-2.3001	0.53846	0.47937	0.9464 (max of	3	7443; 7190; 6653	0.61756235	-0.2329715		49.0
	ZINC04073474	-7.2	412.505	-0.7592	9	1	324.62	0.29265	119.08	-59.32	4285.064499754	0.25394	6.1272	-2.9897	0.55172	0.64817	0.93975 (max o	5	4514; 5362; 5364; 54	0.45887056	-0.0261427		47.0
	ZINC02136455	-7.2	355.477	-0.8242	7	2	284.37	0.29149	117.18	5.2815	4285.064499754	0.29457	6.1922	-2.798	0.6	0.56841	0.91611	1	6949	0.7788962	-0.3990417		47.0
	ZINC04095542	-7.2	366.174	-9.7178	13	4	227.86	0.69784	218.63	-30.796	4285.064499754	0.30685	15.086	-31.67	0.5	0.57212	0.91522 (max o	5	6718; 4934; 5729; 57	-0.2577098	-0.7587391		41.0
	ZINC04095542	-7.2	365.166	-10.965	13	3	229.04	0.6994	221.46	-26.867	4285.064499754	0.30685	16.333	-35.736	0.5	0.59085	0.91522 (max o	5	4935; 5730; 5732; 60	-0.2616025	-0.7238701		41.0
	ZINC02130193	-7.2	406.482	-0.6481	8	2	304.56	0.36249	143.94	5.6406	4285.064499754	0.26301	6.0161	-2.4642	0.5	0.56187	0.96519 (max o	8	7062; 4244; 4760; 51	0.31285477	0.6735037		36.0
	ZINC03838849	-7.2	357.429	-5.474	9	6	271.89	0.43396	169.39	3.8756	4285.064499754	0.29457	10.842	-18.583	0.56	0.58189	0.92662 (max o	7	2759; 3166; 4040; 45	-0.5513566	-0.1953973		29.0
	ZINC03839694	-7.2	371.456	-3.1764	9	6	284.16	0.3915	158.36	4.0017	4285.064499754	0.28324	8.5444	-11.214	0.57692	0.56476	0.96196 (max o	5	4927; 5326; 6352; 63	-0.6384747	-0.2085325		29.0
	ZINC03838831	-7.2	357.429	-3.6308	9	6	270.4	0.41143	158.36	5.0262	4285.064499754	0.29457	8.9988	-12.326	0.56	0.55582	0.96196 (max o	5	6703; 4927; 6090; 63	-0.6029109	-0.2200979		29.0
	ZINC40309220	-7.2	381.205	-1.9175	7	2	254.43	0.32697	103.26	-0.15442	4285.064499754	0.32019	7.2855	-5.9887	0.65217	0.59384	0.93686 (max o	3	4848; 5580; 6032	-0.2915168	0.914439		28.0
	ZINC04073480	-7.2	366.365	0.0247	8	4	255.37	0.37373	125.68	-5.4909	4285.064499754	0.28324	5.3433	0.087204	0.5	0.28536	0.9464 (max of	4	52; 103; 564; 1122	0.3327045	0.4687745		24.0
	ZINC72320404	-7.2	390.471	-0.2607	8	6	283.07	0.34832	139.84	-5.146	4285.064499754	0.27275	5.6287	-0.95581	0.48148	0.45742	0.95493 (max o	3	4446; 4447; 6062	-0.3944283	0.82499063		23.0
	ZINC72320399	-7.2	406.470	-1.0841	9	7	287.33	0.38875	160.07	-4.0503	4285.064499754	0.26301	6.4521	-4.1219	0.5	0.44224	0.95148 (max o	7	3967; 3968; 4883; 56	0.06007407	-0.7763980		23.0
	ZINC19331345	-7.2	357.469	-2.4049	7	5	264.45	0.3274	105.67	3.1491	4285.064499754	0.29457	7.7729	-8.164	0.44	0.6265	0.97118 (max o	4	7372; 7201; 3826; 65	-0.2958023	0.73983866		23.0
	ZINC19323209	-7.2	387.495	-2.9723	8	6	283.3	0.35185	125.9	3.5199	4285.064499754	0.27275	8.3403	-10.897	0.44444	0.63378	0.96617 (max o	6	7368; 7367; 7200; 70	-0.2438415	0.7237775		23.0
	ZINC19323234	-7.2	387.495	-2.5001	8	6	288.82	0.34513	125.9	2.1725	4285.064499754	0.27275	7.8681	-9.1662	0.48148	0.64226	0.96064 (max o	5	7370; 7369; 7026; 70	-0.6154455	0.25347525		23.0
	ZINC19323231	-7.2	387.495	-2.5001	8	6	288.82	0.34513	125.9	2.1725	4285.064499754	0.27275	7.8681	-9.1662	0.48148	0.64517	0.90311 (max o	4	7616; 7370; 7369; 67	-0.6188311	0.23415574		23.0
	ZINC20464935	-7.2	370.443	-2.2535	7	4	294.48	0.27791	86.47	3.0599	4285.064499754	0.28324	7.6215	-7.9561	0.53846	0.60016	0.9302 (max of	6	7521; 7397; 7222; 70	-0.3079802	0.06660889		22.0
	ZINC20465495	-7.2	379.455	-0.628	8	3	285.85	0.27217	94.5	6.1423	4285.064499754	0.27275	5.996	-2.3025	0.51852	0.51553	0.94862 (max o	5	7049; 3400; 4311; 47	-0.3336436	0.11589783		21.0
	ZINC20464876	-7.2	379.455	-1.0568	8	3	287.35	0.27075	94.5	5.6093	4285.064499754	0.27275	6.4248	-3.8746	0.59259	0.52451	0.9399 (max of	5	7729; 3003; 5554; 59	-0.3648652	0.16108145		21.0
	ZINC08254655	-7.2	379.479	0.0932	7	4	280.94	0.36915	126.15	2.8382	4285.064499754	0.28324	5.2748	0.32905	0.53846	0.414	0.97533 (max o	7	7333; 7164; 7160; 22	0.46054855	0.1439905		18.0
	ZINC08636382	-7.2	404.533	0.6799	7	3	303.12	0.29477	111.37	3.338	4285.064499754	0.26301	4.6881	2.5851	0.5	0.47438	0.9083 (max of	5	6988; 6754; 6183; 64	0.46569505	0.12285226		18.0
	ZINC08254663	-7.2	397.493	-1.7239	7	5	301.63	0.369	121.36	2.3952	4285.064499754	0.27275	7.0919	-6.3204	0.51852	0.43973	0.97658 (max o	5	7492; 7164; 6987; 69	0.39439085	0.16768385		18.0
	ZINC20462951	-7.2	418.580	-2.3382	7	1	354.41	0.19356	71.36	5.6653	4285.064499754	0.24548	7.7062	-9.5251	0.56667	0.67798	0.92206 (max o	8	7380; 7207; 7035; 68	-0.0471596	-0.1362251		18.0
	ZINC20462951	-7.2	418.580	-2.3382	7	1	354.41	0.19356	71.36	6.8079	4285.064499754	0.24548	7.7062	-9.5251	0.56667	0.68016	0.92206 (max o	7	7379; 7206; 7034; 68	-0.0489405	-0.0988638		18.0
	ZINC20462951	-7.2	419.588	-1.9315	7	2	368.45	0.22429	72.56	4.8633	4285.064499754	0.24548	7.2995	-7.8684	0.56667	0.66684	0.92206 (max o	7	7381; 7208; 7036; 51	-0.0074225	-0.1150541		18.0
	ZINC04556851	-7.2	356.323	-4.03	11	7	235.73	0.55869	178.53	-7.2031	4285.064499754	0.30685	9.398	-13.134	0.45833	0.45941	0.94718 (max o	7	2827; 5411; 5412; 57	0.5570008	-0.4440456		17.0
	ZINC13348367	-7.2	383.349	-4.4475	12	8	254.94	0.57347	198.4	-4.7202	4285.064499754	0.28324	9.8155	-15.702	0.42308	0.46223	0.94951 (max o	10	7594; 7479; 7480; 73	0.51193756	-0.5153309		17.0
	ZINC13348060	-7.2	383.349	-4.4475	12	8	254.94	0.57347	198.4	-3.0597	4285.064499754	0.28324	9.8155	-15.702	0.42308	0.46124	0.95559 (max o	5	7912; 7878; 7502; 70	0.4897416	-0.4399608		17.0
	ZINC35271200	-7.2	426.460	0.0489	9	4	309.91	0.35985	150.59	-12.343	4285.064499754	0.24548	5.3191	0.1992	0.43333	0.53443	0.96589 (max o	5	7083; 4818; 5220; 66	0.36004668	0.307559		16.0
	ZINC72320310	-7.2	353.433	-0.2864	6	2	262.54	0.29161	106.89	-1.0973	4285.064499754	0.29457	5.6544	-0.97226	0.44	0.46819	0.93686 (max o	3	7573; 5683; 6377	0.37676552	-0.7385486		16.0
	ZINC31170363	-7.2	426.460	0.0489	9	4	309.91	0.35985	150.59	-12.343	4285.064499754	0.24548	5.3191	0.1992	0.43333	0.53144	0.96343 (max o	5	7423; 7422; 7251; 70	0.4476604	0.24088383		16.0
	ZINC40447743	-7.2	425.996	-1.1969	7	5	318.59	0.34176	128.76	0.91283	4285.064499754	0.27275	6.5649	-4.3882	0.51852	0.45528	0.97149 (max o	12	7679; 7464; 7460; 74	0.70761293	-0.2502527		15.0
	ZINC20763452	-7.2	378.384	-1.3198	10	3	287.9	0.38277	129.56	5.185	4285.064499754	0.27275	6.6878	-4.8388	0.62963	0.54776	0.93985 (max o	7	7426; 7063; 5170; 62	-0.0393047	-0.3587440		15.0
	ZINC12483080	-7.2	362.448	-1.4721	7	2	262.52	0.23229	78.85	6.2154	4285.064499754	0.28324	6.8401	-5.1973	0.57692	0.46969	0.96998 (max o	8	7384; 641; 5533; 585	0.41525716	-0.2059774		14.0
	ZINC22922164	-7.2	352.428	-1.428	6	2	251.12	0.1909	61.78	5.4944	4285.064499754	0.29457	6.796	-4.8477	0.6	0.46111	0.93033 (max o	7	7065; 3842; 4649; 51	0.4334549	-0.1646416		14.0
	ZINC08635291	-7.2	352.457	-2.8249	8	3	283.89	0.33111	93.71	-1.6975	4285.064499754	0.29457	8.1929	-9.5898	0.64	0.57252	0.9654 (max of	2	1760; 5452	0.24800591	0.22335728		14.0

Structure	Molecule Name	affinity	Total Molweight	cLogP	H-Acceptors	H-Donors	Total Surface Area	Relative PSA	Polar Surface Area	Druglikeness	Kd (nM) @ 20°C	LE from K...	LLE from Kd ...	LELP from Kd (n...	Shape Index	Molecular...	Neighbor Si...	Neig...	Neighbor	Neighbor ...	Neighbor ...	Ring Systems	frequency
	ZINC20463961	-7.2	365.452	-1.9727	8	4	288.48	0.31267	87.5	3.2854	4285.064499754	0.28324	7.3407	-6.9647	0.57692	0.52601	0.93686 (max o	6	4732; 5181; 5543; 59	-0.7170791	-0.2299659		14.0
	ZINC20463963	-7.2	433.449	-1.1244	8	4	317.94	0.2837	87.5	-3.9046	4285.064499754	0.24548	6.4924	-4.5805	0.53333	0.55439	0.92206 (max o	5	4732; 5543; 5919; 65	-0.7215766	-0.2500298		14.0
	ZINC12483670	-7.2	366.436	-0.9299	8	4	288.18	0.33538	93.49	-0.39401	4285.064499754	0.28324	6.2979	-3.283	0.57692	0.50478	0.95257 (max o	3	6994; 6993; 5474	0.89453274	0.26868215		14.0
	ZINC05413946	-7.2	435.501	-4.4039	11	2	301.96	0.47202	189.7	6.7233	4285.064499754	0.26301	9.7719	-16.744	0.53571	0.62236	0.91396 (max o	3	3689; 4573; 6460	-0.0517403	0.4298015		13.0
	ZINC03842123	-7.2	369.441	0.4346	8	0	267.56	0.28147	95.61	3.4279	4285.064499754	0.29457	4.9334	1.4754	0.52	0.62207	0.92442 (max o	3	7582; 7145; 6453	0.10315692	0.43831155		13.0
	ZINC04085155	-7.2	363.412	-0.257	8	3	278.14	0.31887	107.97	4.8383	4285.064499754	0.28324	5.625	-0.90735	0.65385	0.46209	0.96438 (max o	4	5370; 5756; 6022; 64.	0.5813736	0.30153587		12.0
	ZINC08624588	-7.2	388.440	-1.2554	9	4	279.01	0.3969	142.57	-6.0427	4285.064499754	0.28324	6.6234	-4.4322	0.65385	0.57165	0.94002 (max o	8	1800; 2115; 3287; 33	0.22376457	-0.3374478		12.0
	ZINC20464949	-7.2	388.507	-2.6677	8	4	298.96	0.32573	111.92	-2.6462	4285.064499754	0.28324	8.0357	-9.4184	0.53846	0.60337	0.94002 (max o	4	7045; 5564; 5928; 62	-0.3799175	-0.1507468		12.0
	ZINC35271311	-7.2	412.433	0.2102	9	4	295.62	0.37724	150.59	-8.6504	4285.064499754	0.25394	5.1578	0.82775	0.41379	0.52952	0.96682 (max o	7	7105; 7084; 4395; 52	0.39917263	0.3224186		12.0
	ZINC31170275	-7.2	438.471	1.6499	9	2	324.65	0.33377	136.43	-5.8488	4285.064499754	0.23756	3.7181	6.9452	0.45161	0.53187	0.96971 (max o	8	7250; 6876; 5216; 56.	0.35873333	0.24154164		12.0
	ZINC04095478	-7.2	424.401	-4.4371	13	8	290.06	0.54334	207.27	-3.1385	4285.064499754	0.25394	9.8051	-17.473	0.41379	0.47071	0.94823 (max o	2	6428; 6531	0.17229569	0.73537135		12.0
	ZINC04085101	-7.2	365.453	-1.4662	6	1	254.82	0.40574	140.14	6.1016	4285.064499754	0.30685	6.8342	-4.7783	0.54167	0.41355	0.95709 (max o	3	7309; 7308; 6950	-0.7178069	-0.6562369		10.0
	ZINC35454685	-7.2	413.397	-2.9205	10	4	298.4	0.41139	165.81	1.5959	4285.064499754	0.25394	8.2885	-11.501	0.58621	0.59812	0.99848 (max o	7	1948; 2642; 3546; 39.	-0.1517587	0.6986702		10.0
	ZINC20465495	-7.2	380.463	-2.7377	8	4	299.89	0.30625	95.7	3.9936	4285.064499754	0.27275	8.1057	-10.037	0.51852	0.52114	0.94862 (max o	6	7050; 3401; 4313; 47	-0.3549242	-0.5794598		10.0
	ZINC20464876	-7.2	380.463	-3.1665	8	4	301.39	0.30472	95.7	3.6337	4285.064499754	0.27275	8.5345	-11.609	0.59259	0.52938	0.96064 (max o	7	7730; 7053; 3004; 55	-0.3449915	-0.5988129		10.0
	ZINC04096831	-7.2	358.394	-4.462	9	2	245.75	0.51963	182.61	4.8789	4285.064499754	0.30685	9.83	-14.541	0.58333	0.44402	0.93334 (max o	4	7324; 5415; 6129; 63	-0.3010435	-0.8241847		9.0
	ZINC01578628	-7.2	374.483	-5.3374	7	4	296.3	0.29686	122.71	-0.69735	4285.064499754	0.27275	10.705	-19.569	0.62963	0.47782	0.88922	1	4559	0.66008466	0.72700024		8.0
	ZINC04556959	-7.2	356.323	-4.03	11	7	235.73	0.55869	178.53	-8.3431	4285.064499754	0.30685	9.398	-13.134	0.5	0.45941	0.94718 (max o	8	2827; 5411; 5412; 57	0.5660906	-0.4622529		8.0
	ZINC35455843	-7.2	352.425	1.0294	6	4	262.95	0.2869	107.22	4.8399	4285.064499754	0.29457	4.3386	3.4946	0.56	0.45151	0.97118 (max o	6	3898; 4798; 4799; 48	-0.6999585	-0.167611		8.0
	ZINC70666302	-7.2	359.357	-0.8644	8	2	260.62	0.37929	120.55	0.018054	4285.064499754	0.28324	6.2324	-3.0518	0.5	0.32755	0.94008 (max o	4	7110; 7109; 4440; 52	0.47729668	0.42029		8.0
	ZINC04042334	-7.2	384.136	0.9648	5	3	207.7	0.28551	79.15	-3.1395	4285.064499754	0.3876	4.4032	2.4892	0.52632	0.26722	0.91405 (max o	3	7682; 7458; 7305	-0.4513624	-0.8439996		8.0
	ZINC30879455	-7.2	389.538	-2.2365	6	1	333.35	0.19514	68.12	6.6855	4285.064499754	0.26301	7.6045	-8.5034	0.57143	0.6785	0.93101 (max o	5	7376; 7206; 7028; 47	-0.1210145	-0.1193718		8.0
	ZINC30879455	-7.2	390.546	-1.8298	6	2	347.39	0.22767	69.32	4.2834	4285.064499754	0.26301	7.1978	-6.9571	0.57143	0.66449	0.93101 (max o	5	7378; 7208; 7030; 50	-0.0714928	-0.1424365		8.0
	ZINC30879455	-7.2	389.538	-2.2365	6	1	333.35	0.19514	68.12	5.8202	4285.064499754	0.26301	7.6045	-8.5034	0.57143	0.67621	0.93101 (max o	5	7377; 7207; 7029; 68	-0.1045200	-0.1312282		8.0
	ZINC03791297	-7.2	376.451	-0.8691	7	1	293.16	0.25478	103.35	1.9037	4285.064499754	0.27275	6.2371	-3.1864	0.51852	0.50492	0.90299 (max o	4	3588; 3995; 5317; 57	-0.8132036	0.00452349		8.0
	ZINC03873145	-7.2	405.493	-3.2679	8	2	315.18	0.2965	144.82	2.4951	4285.064499754	0.25394	8.6359	-12.869	0.48276	0.53277	0.95908 (max o	4	7125; 6941; 5319; 61	-0.8114396	-0.4274828		8.0
	ZINC15676490	-7.2	421.836	-2.5283	9	1	296.57	0.28263	115.04	0.53246	4285.064499754	0.25394	7.8963	-9.9562	0.55172	0.51073	0.95675 (max o	6	7196; 7016; 6790; 55.	-0.3013650	0.34630257		8.0
	ZINC12530288	-7.2	401.418	-2.203	9	1	293.41	0.28568	115.04	0.87393	4285.064499754	0.25394	7.571	-8.6752	0.55172	0.53089	0.9561 (max of	7	7196; 7016; 6789; 67	-0.2633537	0.38762468		8.0
	ZINC35455117	-7.2	392.487	-0.0831	8	6	286.85	0.34373	139.84	-10.396	4285.064499754	0.27275	5.4511	-0.30467	0.48148	0.55806	0.99202 (max o	7	7261; 5597; 6327; 66	-0.7506016	0.24894105		8.0
	ZINC14719127	-7.2	356.370	0.1552	8	1	277.9	0.33185	108.36	-3.5573	4285.064499754	0.29457	5.2128	0.52686	0.52	0.57413	0.96243 (max o	7	7360; 7012; 5992; 59	-0.0077428	-0.7314027		8.0
	ZINC14719124	-7.2	356.370	0.1552	8	1	277.9	0.33185	108.36	-3.5573	4285.064499754	0.29457	5.2128	0.52686	0.52	0.5943	0.96243 (max o	6	7360; 7012; 6786; 59	-0.0113482	-0.7513068		8.0
	ZINC31168970	-7.2	373.420	-1.7731	8	4	278.5	0.35806	139.51	-9.7968	4285.064499754	0.28324	7.1411	-6.26	0.5	0.51929	0.95857 (max o	4	7851; 7247; 5642; 63	0.42676106	0.6041455		8.0
	ZINC31168993	-7.2	388.455	0.7308	8	4	293.23	0.32548	125.68	-15.27	4285.064499754	0.27275	4.6372	2.6794	0.48148	0.51734	0.94605 (max o	3	7077; 6621; 6622	0.94617295	0.07309800		8.0
	ZINC35051188	-7.2	396.300	-8.4533	13	4	261.98	0.59944	207.33	-4.475	4285.064499754	0.27275	13.821	-30.993	0.48148	0.45254	0.95493 (max o	3	7646; 7257; 6311	-0.6514002	0.73019105		7.0
	ZINC20465119	-7.2	350.437	-2.7191	7	4	277.63	0.29478	86.47	3.5744	4285.064499754	0.29457	8.0871	-9.2307	0.56	0.51564	0.9302 (max of	7	7397; 2591; 2593; 47	-0.3356953	0.00877532		6.0
	ZINC22922164	-7.2	353.436	-3.5377	6	3	265.16	0.23375	62.98	1.1703	4285.064499754	0.29457	8.9057	-12.01	0.6	0.46111	0.92342 (max o	8	7520; 7064; 7066; 10	0.50257903	-0.1918143		6.0
	ZINC15251354	-7.2	374.341	-0.9024	10	4	265.95	0.43407	144.14	-4.135	4285.064499754	0.28324	6.2704	-3.186	0.5	0.41835	0.94975 (max o	6	4802; 5193; 5194; 58	-0.6957767	-0.1238269		6.0
	ZINC31167388	-7.2	417.456	-1.3541	8	0	322.92	0.28252	119.03	4.6442	4285.064499754	0.24548	6.7221	-5.5162	0.43333	0.52584	0.97975 (max o	5	7245; 5206; 5609; 66	-0.0553544	0.8945214		6.0
	ZINC14858003	-7.2	388.368	-2.1091	10	5	265.99	0.44566	155.14	-3.1573	4285.064499754	0.27275	7.4771	-7.7327	0.48148	0.40236	0.94302 (max o	3	3791; 4377; 5238	-0.8929919	0.10683932		6.0
	ZINC12530288	-7.2	400.410	-2.0141	9	0	304.38	0.31142	113.79	1.4339	4285.064499754	0.25394	7.3821	-7.9313	0.55172	0.53089	0.9561 (max of	7	7195; 7017; 6790; 58	-0.2465706	0.3533581		6.0
	ZINC15676490	-7.2	420.828	-2.3394	9	0	307.54	0.30822	113.79	1.0868	4285.064499754	0.25394	7.7074	-9.2123	0.55172	0.51073	0.95675 (max o	5	7195; 7017; 5896; 67	-0.2717470	0.3231851		6.0
	ZINC08643629	-7.2	357.400	-2.1483	7	4	266.82	0.29398	104.63	-2.0423	4285.064499754	0.29457	7.5163	-7.2929	0.72	0.50901	0.91964 (max o	8	7015; 2528; 2876; 28	0.10807659	-0.4948455		6.0
	ZINC08624286	-7.2	357.856	-0.785	6	4	263.13	0.2601	95.4	1.2754	4285.064499754	0.30685	6.153	-2.5583	0.625	0.47108	0.92257 (max o	5	7015; 2154; 3731; 58	0.13800745	-0.439685		6.0

Structure	Molecule Name	affinity	Total Molweight	cLogP	H-Acceptors	H-Donors	Total Surface Area	Relative PSA	Polar Surface Area	Druglikeness	Kd (nM) @ 20°C	LE from K...	LLE from Kd ...	LELP from Kd (n...	Shape Index	Molecular...	Neighbor Si...	Neig...	Neighbor	Neighbor ...	Neighbor ...	Ring Systems	frequency
	ZINC08643633	-7.2	419.927	0.134	6	3	310.73	0.1948	86.61	2.6135	4285.064499754	0.25394	5.234	0.52768	0.62069	0.5273	0.93395 (max o	5	1828; 2154; 4172; 41	0.15074542	-0.4951630		5.0
	ZINC04097097	-7.2	383.349	-4.4475	12	8	254.94	0.57347	198.4	-4.7202	4285.064499754	0.28324	9.8155	-15.702	0.42308	0.46983	0.95257 (max o	6	7010; 6958; 4541; 49	0.2518235	0.73721576		5.0
	ZINC04214908	-7.2	371.301	-1.3036	12	1	262.31	0.50215	149.9	5.3179	4285.064499754	0.28324	6.6716	-4.6024	0.46154	0.43912	0.88492 (max o	4	6923; 6757; 6724; 64	0.4143372	-0.4295089		5.0
	ZINC22920496	-7.2	350.545	-0.3137	4	2	290.37	0.16658	47.35	-6.4065	4285.064499754	0.29457	5.6817	-1.0649	0.56	0.50037	0.93348	1	6279	-0.3111943	0.85955924		5.0
	ZINC61997002	-7.2	395.562	-0.3464	6	3	305.79	0.19795	86.61	-4.4229	4285.064499754	0.26301	5.7144	-1.3171	0.57143	0.55438	0.94573 (max o	3	7272; 7102; 5668	-0.6729122	0.21965902		5.0
	ZINC61997002	-7.2	395.562	-0.488	6	4	323.55	0.24197	83.23	-3.5843	4285.064499754	0.26301	5.856	-1.8554	0.57143	0.58716	0.94573 (max o	3	7273; 7103; 5669	-0.3796859	0.8983175		5.0
	ZINC03838873	-7.2	414.525	0.0501	9	2	310.11	0.35365	133.35	5.789	4285.064499754	0.26301	5.3179	0.19049	0.5	0.55004	0.92774 (max o	4	7754; 7461; 4268; 60	-0.0526472	-0.0583652		5.0
	ZINC20463806	-7.2	354.401	-3.0795	8	3	248.15	0.31666	99.6	-0.36826	4285.064499754	0.29457	8.4475	-10.454	0.6	0.45197	0.86809	1	5058	0.59194857	-0.1607637		5.0
	ZINC20463806	-7.2	353.394	-1.8548	8	3	251.87	0.32676	95.02	1.4333	4285.064499754	0.29457	7.2228	-6.2966	0.6	0.46174	0.89162 (max o	2	7172; 5136	0.46565494	-0.1418539		5.0
	ZINC04684272	-7.2	426.424	-2.8694	9	2	332.04	0.34225	147.69	-10.52	4285.064499754	0.23756	8.2374	-12.079	0.54839	0.53242	0.96971 (max o	4	7189; 2962; 4112; 49	-0.0331088	-0.4505942		4.0
	ZINC20760284	-7.2	401.461	-0.0253	8	3	287.81	0.30815	107.97	4.3334	4285.064499754	0.25394	5.3933	-0.09963	0.48276	0.28196	0.95908 (max o	3	3018; 3420; 4332	0.83269155	0.37921825		4.0
	ZINC49601641	-7.2	361.365	-3.7858	9	5	255.01	0.44241	159.74	-3.3297	4285.064499754	0.29457	9.1538	-12.852	0.48	0.58091	0.97396 (max o	3	3529; 4430; 6665	0.82346046	-0.0628671		4.0
	ZINC03882100	-7.2	388.368	-2.1498	10	5	265.46	0.44655	155.14	-4.9044	4285.064499754	0.27275	7.5178	-7.8819	0.48148	0.38564	0.94905	1	5738	-0.1515701	0.9599075		4.0
	ZINC35464612	-7.2	375.443	-1.0588	7	1	289.31	0.2544	100.98	7.6353	4285.064499754	0.27275	6.4268	-3.8819	0.51852	0.64015	0.96617 (max o	3	3932; 5240; 5640	-0.1590675	0.8947883		4.0
	ZINC12484189	-7.2	386.470	-0.8449	7	2	281.96	0.22007	74.92	4.6209	4285.064499754	0.26301	6.2129	-3.2124	0.57143	0.4338	0.93101 (max o	5	7213; 4731; 5135; 54	0.3837659	0.02375834		4.0
	ZINC31160847	-7.2	389.419	-1.9705	9	2	290.57	0.36673	134.58	-13.144	4285.064499754	0.27275	7.3385	-7.2245	0.55556	0.52479	0.95493 (max o	7	6882; 5197; 6005; 62	-0.7107969	-0.0890813		4.0
	ZINC35459000	-7.2	410.414	-2.1178	11	6	284.15	0.46328	175.37	-2.7727	4285.064499754	0.26301	7.4858	-8.0521	0.5	0.44389	0.9678 (max of	3	5237; 6008; 6334	0.70651394	-0.4665836		4.0
	ZINC72320478	-7.2	354.354	-0.5368	8	0	240.95	0.35725	89.52	-3.7722	4285.064499754	0.29457	5.9048	-1.8223	0.48	0.30982	0.96243 (max o	3	5285; 5688; 6681	0.77653486	0.39942124		4.0
	ZINC35271765	-7.2	438.471	1.6499	9	2	324.65	0.33377	136.43	-5.8488	4285.064499754	0.23756	3.7181	6.9452	0.45161	0.53187	0.96971 (max o	7	5216; 5624; 5983; 59	0.3868163	0.22585328		4.0
	ZINC36369863	-7.2	388.399	-5.6761	9	2	300.79	0.39034	141.95	3.1709	4285.064499754	0.26301	11.044	-21.581	0.42857	0.43729	0.95174 (max o	5	7269; 5246; 5646; 56	0.2737683	-0.5720665		4.0
	ZINC36370112	-7.2	388.399	-5.3534	9	1	305.62	0.34582	119.17	2.4683	4285.064499754	0.26301	10.721	-20.354	0.5	0.49282	0.93385 (max o	4	7269; 5247; 5646; 60	0.34130716	-0.602994		4.0
	ZINC35457089	-7.2	389.419	-1.9705	9	2	290.57	0.36673	134.58	-13.144	4285.064499754	0.27275	7.3385	-7.2245	0.55556	0.51898	0.94605 (max o	8	7649; 5197; 6005; 62	-0.7474729	-0.0670962		4.0
	ZINC01764552	-7.2	355.437	-3.8574	9	3	276.14	0.29239	93.59	-0.69321	4285.064499754	0.29457	9.2254	-13.095	0.56	0.56546	0.97118	1	6685	0.8150933	-0.4608366		4.0
	ZINC01764550	-7.2	355.437	-3.8574	9	3	276.14	0.29239	93.59	-0.69321	4285.064499754	0.29457	9.2254	-13.095	0.56	0.56335	0.97118	1	6687	0.81466985	-0.4384252		4.0
	ZINC01764552	-7.2	354.429	-1.8796	9	2	287.11	0.31942	92.34	-0.44817	4285.064499754	0.29457	7.2476	-6.3808	0.56	0.56838	0.97396	1	6686	0.92121494	0.2161192		4.0
	ZINC01764550	-7.2	354.429	-1.8796	9	2	287.11	0.31942	92.34	-0.44817	4285.064499754	0.29457	7.2476	-6.3808	0.56	0.56628	0.97396	1	6688	0.93555033	0.22757982		4.0
	ZINC14694900	-7.2	362.333	1.3714	8	5	253.8	0.38826	136.68	-0.38093	4285.064499754	0.28324	3.9966	4.8418	0.42308	0.33749	0.9615 (max of	3	7359; 7011; 6782	0.76082075	0.44418466		4.0
	ZINC14694894	-7.2	362.333	1.3714	8	5	253.8	0.38826	136.68	-0.38093	4285.064499754	0.28324	3.9966	4.8418	0.42308	0.34203	0.9615 (max of	3	7359; 7011; 6783	0.7535298	0.46591637		4.0
	ZINC72320585	-7.2	356.374	-3.0116	9	3	270.04	0.41638	152.63	1.1243	4285.064499754	0.29457	8.3796	-10.224	0.52	0.60592	0.96832 (max o	3	7453; 7452; 7278	0.9439475	-0.1324303		4.0
	ZINC02575127	-7.2	351.405	-0.5014	6	3	271.93	0.29526	112.66	2.9212	4285.064499754	0.28324	5.8694	-1.7702	0.57692	0.47677	0.93686 (max o	7	1639; 2349; 2741; 28	0.28962505	0.76087815		3.0
	ZINC62001274	-7.2	424.928	-2.7348	7	2	306.99	0.31714	126.87	7.7607	4285.064499754	0.26301	8.1028	-10.398	0.46429	0.49637	0.9398 (max of	5	7448; 3093; 5262; 63	0.593995	0.33415672		3.0
	ZINC04654843	-7.2	408.518	-14.16	11	6	292.02	0.45031	197.4	2.1351	4285.064499754	0.26301	19.528	-53.837	0.46429	0.48505	0.89582	1	6734	0.39250723	-0.6870673		3.0
	ZINC04654843	-7.2	409.526	-17.331	11	6	291.23	0.44882	199.02	1.7025	4285.064499754	0.26301	22.699	-65.893	0.46429	0.48505	0.89582	1	6735	0.4030329	-0.7033074		3.0
	ZINC04270701	-7.2	366.461	0.6184	7	1	267.37	0.31537	111.77	4.5709	4285.064499754	0.29457	4.7496	2.0993	0.52	0.49868	0.95318 (max o	2	3685; 5784	-0.1718680	0.23904778		3.0
	ZINC08740369	-7.2	352.350	-1.7011	11	4	243.01	0.48517	152.01	0.0425	4285.064499754	0.29457	7.0691	-5.7748	0.44	0.37685	0.93348 (max o	4	6990; 6989; 3752; 61	-0.8821157	0.08877115		3.0
	ZINC05412670	-7.2	355.501	-0.5231	7	2	301.54	0.23954	66.32	4.3195	4285.064499754	0.29457	5.8911	-1.7758	0.64	0.67652	0.86222 (max o	2	4590; 4993	0.8051903	0.21780117		3.0
	ZINC03872807	-7.2	422.520	-1.4272	8	8	321.68	0.29246	154.6	-7.0465	4285.064499754	0.24548	6.7952	-5.814	0.73333	0.49156	0.97533 (max o	2	6940; 5343	0.8544708	-0.2532307		3.0
	ZINC20410483	-7.2	377.439	-1.9933	8	3	284.45	0.26518	108.06	4.2964	4285.064499754	0.27275	7.3613	-7.3081	0.62963	0.5023	0.89688 (max o	4	3378; 3379; 5468; 65	-0.0415261	-0.8177453		3.0
	ZINC12481197	-7.2	359.444	-1.071	6	0	282.49	0.20341	72.91	4.0521	4285.064499754	0.28324	6.439	-3.7812	0.53846	0.58645	0.93686 (max o	7	2983; 4209; 4643; 50	-0.1472870	-0.1385016		3.0
	ZINC03791297	-7.2	375.443	-2.1005	7	1	296.88	0.26411	98.77	3.6019	4285.064499754	0.27275	7.4685	-7.7011	0.51852	0.61106	0.88044 (max o	2	5715; 6086	-0.5780371	0.62945616		3.0
	ZINC08624488	-7.2	373.448	-1.1011	9	5	287.68	0.37076	129.15	-3.8853	4285.064499754	0.28324	6.4691	-3.8875	0.69231	0.60762	0.9302 (max of	6	2924; 3293; 4227; 46	0.16528226	-0.4164650		3.0
	ZINC20465221	-7.2	356.442	-0.7377	7	3	250.36	0.3187	107.48	4.7813	4285.064499754	0.30685	6.1057	-2.4041	0.5	0.47367	0.86661	1	7635	-0.6051757	0.55097204		3.0
	ZINC12649667	-7.2	443.364	-1.3291	14	1	314.82	0.49158	176.2	5.3179	4285.064499754	0.23756	6.6971	-5.5948	0.3871	0.47836		0		0.16144721	0.95647055		3.0

Structure	Molecule Name	affinity	Total Molweight	cLogP	H-Acceptors	H-Donors	Total Surface Area	Relative PSA	Polar Surface Area	Druglikeness	Kd (nM) @ 20°C	LE from K...	LLE from Kd ...	LELP from Kd (n...	Shape Index	Molecular...	Neighbor Si...	Neig...	Neighbor	Neighbor ...	Neighbor ...	Ring Systems	frequency
	ZINC20463806	-7.2	353.394	-1.8548	8	3	251.87	0.32676	95.02	1.1445	4285.064499754	0.29457	7.2228	-6.2966	0.6	0.49092	0.86427 (max o	2	7497; 7173	0.5552517	-0.1388044		3.0
	ZINC20463806	-7.2	352.386	-0.6301	8	3	255.59	0.33655	90.44	1.5835	4285.064499754	0.29457	5.9981	-2.139	0.6	0.50011	0.84495	1	7495	0.5796181	-0.0187325		3.0
	ZINC27642623	-7.2	424.444	0.1377	9	1	297.77	0.36898	120.89	0.65007	4285.064499754	0.24548	5.2303	0.56095	0.36667	0.29425	0.98593 (max o	5	7797; 7731; 7548; 74(	-0.0914189	0.8807123		3.0
	ZINC08636298	-7.2	415.445	-0.4483	9	4	312.94	0.33927	130.01	0.98145	4285.064499754	0.24548	5.8163	-1.8262	0.66667	0.53452	0.94785 (max o	2	7599; 1516	0.43245855	-0.4424002		2.0
	ZINC31157178	-7.2	352.382	0.2866	7	2	246.67	0.34589	110.13	-8.7646	4285.064499754	0.29457	5.0814	0.97294	0.44	0.39461	0.97118 (max o	3	1576; 3452; 5598	-0.497686	0.54981464		2.0
	ZINC20465253	-7.2	403.928	0.125	5	3	294.74	0.16113	69.54	2.231	4285.064499754	0.26301	5.243	0.47526	0.53571	0.40645	0.9308 (max of	2	3396; 4748	-0.5652932	0.19292717		2.0
	ZINC72320121	-7.2	448.507	-1.5165	10	7	305.77	0.40795	177.14	-8.1275	4285.064499754	0.23756	6.8845	-6.3837	0.41935	0.52422	0.98656 (max o	3	2325; 3972; 3973	-0.4957926	-0.8022113		2.0
	ZINC18181653	-7.2	411.476	-0.0866	7	1	328.97	0.24397	77.35	0.091138	4285.064499754	0.24548	5.4546	-0.35278	0.43333	0.40964	0.82597	1	5117	-0.1175460	-0.9246309		2.0
	ZINC31157109	-7.2	397.355	-2.4322	10	4	280.77	0.44104	161.88	-4.6331	4285.064499754	0.26301	7.8002	-9.2475	0.42857	0.4504	0.97037 (max o	3	7547; 5192; 6293	0.15516828	-0.6458123		2.0
	ZINC22923368	-7.2	378.519	-4.5204	8	4	299.88	0.25417	87.02	6.6962	4285.064499754	0.27275	9.8884	-16.573	0.55556	0.50788	0.92774 (max o	11	1561; 2992; 3022; 34	-0.1074443	0.49636814		2.0
	ZINC22923368	-7.2	377.511	-2.5426	8	3	310.85	0.28049	85.77	6.8425	4285.064499754	0.27275	7.9106	-9.322	0.55556	0.52497	0.92774 (max o	10	1562; 2993; 3023; 34	-0.0711894	0.458581		2.0
	ZINC03839672	-7.2	367.468	-0.5225	7	1	257.14	0.33122	104.25	7.1158	4285.064499754	0.29457	5.8905	-1.7738	0.6	0.32926	0.97118 (max o	4	2378; 4923; 6092; 67(	-0.3580727	0.71231526		2.0
	ZINC04268225	-7.2	368.468	0.3019	6	5	248.57	0.31597	118.22	-8.2419	4285.064499754	0.28324	5.0661	1.0659	0.38462	0.38693	0.84495	1	6146	0.92459434	-0.1720786		2.0
	ZINC08740483	-7.2	386.371	0.8103	12	5	275.91	0.50121	176.82	1.3234	4285.064499754	0.26301	4.5577	3.0809	0.5	0.33184	0.98451 (max o	12	3753; 4188; 4618; 46	-0.2388684	-0.6064175		2.0
	ZINC20462988	-7.2	367.488	-1.6721	7	2	298.97	0.3004	107.97	0.57356	4285.064499754	0.28324	7.0401	-5.9034	0.46154	0.60533	0.89162 (max o	4	5907; 6251; 6252; 65	-0.0984582	-0.3631887		2.0
	ZINC35465759	-7.2	416.421	-1.0892	10	6	306.66	0.38675	158.3	-4.1006	4285.064499754	0.25394	6.4572	-4.2892	0.58621	0.546	0.98727 (max o	3	5982; 6301; 6337	0.12040195	0.7604615		2.0
	ZINC70672626	-7.2	449.590	-15.151	11	8	314.94	0.43103	213.93	1.7104	4285.064499754	0.23756	20.519	-63.776	0.48387	0.434	0.94488	1	6369	-0.8780933	0.14899571		2.0
	ZINC08618616	-7.2	389.332	-5.648	11	6	255.52	0.53193	189.2	-2.7509	4285.064499754	0.27275	11.016	-20.707	0.44444	0.3782	0.94302 (max o	3	4442; 6371; 6372	0.16549402	0.88604766		2.0
	ZINC35456923	-7.2	404.367	-3.1441	11	6	270.25	0.4871	175.37	-5.0325	4285.064499754	0.26301	8.5121	-11.954	0.46429	0.37662	0.95148 (max o	2	6003; 6643	0.7871956	-0.0089950		2.0
	ZINC20465331	-7.2	370.463	-3.3869	6	4	288.47	0.24796	79.97	-2.9155	4285.064499754	0.28324	8.7549	-11.958	0.57692	0.42675	0.92368	1	6584	0.31657955	0.9047578		2.0
	ZINC67870723	-7.2	367.536	-0.4841	6	2	291.8	0.24726	75.12	5.1489	4285.064499754	0.29457	5.8521	-1.6434	0.64	0.59194	0.85045	1	6668	0.74852574	-0.5575303		2.0
	ZINC08740070	-7.2	412.354	-1.3868	13	1	297.54	0.48138	162.26	3.8545	4285.064499754	0.25394	6.7548	-5.4611	0.48276	0.45273	0.88492 (max o	3	6486; 6723; 6724	0.4529092	-0.4117023		2.0
	ZINC04214908	-7.2	370.293	-3.4732	12	0	263.6	0.50459	156.22	4.1228	4285.064499754	0.28324	8.8412	-12.262	0.46154	0.43134	0.87816 (max o	4	7745; 6757; 6652; 67	0.43497592	-0.3774730		2.0
	ZINC04221573	-7.2	370.389	-2.2205	11	2	263.11	0.54179	188.8	-0.74433	4285.064499754	0.29457	7.5885	-7.538	0.48	0.42855	0.91255 (max o	5	3222; 3652; 4535; 45	-0.7270108	-0.4906514		2.0
	ZINC04221564	-7.2	370.389	-2.2205	11	2	263.11	0.54179	188.8	-0.7212	4285.064499754	0.29457	7.5885	-7.538	0.48	0.42855	0.91255 (max o	2	2427; 6726	-0.7020021	-0.5274693		2.0
	ZINC08636524	-7.2	390.506	0.2679	7	4	280.24	0.34706	120.16	5.1881	4285.064499754	0.27275	5.1001	0.98221	0.48148	0.39778	0.92418 (max o	3	7161; 6478; 6753	0.47384712	0.15860917		2.0
	ZINC61997632	-7.2	411.493	1.2902	8	2	311.34	0.27568	105.53	1.9981	4285.064499754	0.25394	4.0778	5.0807	0.51724	0.48287	0.9617	1	6791	-0.6754339	-0.7331344		2.0
	ZINC16343450	-7.2	411.493	1.2902	8	2	311.34	0.27568	105.53	1.9981	4285.064499754	0.25394	4.0778	5.0807	0.51724	0.49252	0.9617	1	6903	-0.6869345	-0.7227152		2.0
	ZINC18144456	-7.2	360.385	-0.6307	7	0	276.91	0.28887	103.81	-8.7411	4285.064499754	0.28324	5.9987	-2.2267	0.57692	0.53561	0.88538	1	7197	0.6641169	0.6855797		2.0
	ZINC20463993	-7.2	372.467	-4.0142	7	2	299.37	0.27114	75.81	3.02	4285.064499754	0.27275	9.3822	-14.717	0.55556	0.51223	0.89688 (max o	2	6813; 5922	0.399593	0.07914554		2.0
	ZINC20463993	-7.2	373.475	-1.9382	7	3	298.19	0.26825	72.98	3.3571	4285.064499754	0.27275	7.3062	-7.1061	0.55556	0.51223	0.89688 (max o	3	6996; 5926; 6811	0.35981923	0.07914553		2.0
	ZINC20465221	-7.2	357.450	-2.8474	7	4	264.4	0.35488	108.68	2.6515	4285.064499754	0.30685	8.2154	-9.2796	0.5	0.47938	0.86661	1	7636	0.32592708	-0.0683204		2.0
	ZINC20465884	-7.2	367.468	-2.102	8	5	300.55	0.33512	108.15	3.2503	4285.064499754	0.28324	7.47	-7.4212	0.57692	0.60784	0.91693 (max o	5	7400; 3855; 4322; 62(	0.28791547	-0.2331015		2.0
	ZINC20465848	-7.2	354.425	-3.0954	8	4	288.09	0.34455	105.35	2.1642	4285.064499754	0.29457	8.4634	-10.508	0.6	0.59511	0.91255 (max o	3	7054; 6831; 4319	0.28269306	-0.1883455		2.0
	ZINC20504786	-7.2	354.469	-0.2704	6	4	243.33	0.35072	135	-1.0018	4285.064499754	0.30685	5.6384	-0.88122	0.41667	0.41707	0.93686	1	7228	-0.9652251	-0.0274633		2.0
	ZINC20611195	-7.2	440.519	-6.8334	10	4	313.77	0.40718	199.91	5.6596	4285.064499754	0.24548	12.201	-27.837	0.56667	0.62271	0.88322	1	6840	-0.8294372	0.5282243		2.0
	ZINC20611195	-7.2	440.519	-7.4549	10	5	313.38	0.40644	195.46	5.2805	4285.064499754	0.24548	12.823	-30.369	0.56667	0.66309		0		-0.5961484	0.7944745		2.0
	ZINC30879728	-7.2	376.470	-1.8423	5	4	297.95	0.20651	70.74	-2.4056	4285.064499754	0.27275	7.2103	-6.7545	0.59259	0.48756	0.92739	1	7412	-0.3766473	-0.6992875		2.0
	ZINC35882819	-7.2	371.301	-1.2117	12	1	262.31	0.50215	149.9	5.0025	4285.064499754	0.28324	6.5797	-4.278	0.46154	0.45362	0.97269 (max o	5	7744; 7700; 7493; 68	0.3904444	-0.3187626		2.0
	ZINC36370112	-7.2	387.391	-5.485	9	0	291.58	0.31432	117.97	5.1997	4285.064499754	0.26301	10.853	-20.855	0.5	0.4888	0.9543 (max of	2	7268; 6013	0.80016726	-0.5194273		2.0
	ZINC38191133	-7.2	375.373	-2.4613	10	5	269.21	0.44226	161.86	-7.4413	4285.064499754	0.28324	7.8293	-8.6897	0.46154	0.4176	0.95559 (max o	3	7097; 6979; 5811	-0.8178676	-0.1520429		2.0
	ZINC72319928	-7.2	364.349	0.4791	8	2	252.33	0.37776	119.36	-4.1449	4285.064499754	0.28324	4.8889	1.6915	0.42308	0.26686	0.9464 (max of	3	7277; 7276; 6912	-0.5672565	0.67239994		2.0
	ZINC72319930	-7.2	364.349	0.4791	8	2	252.33	0.37776	119.36	-4.1449	4285.064499754	0.28324	4.8889	1.6915	0.42308	0.26686	0.9464 (max of	3	7277; 7276; 6911	-0.5577748	0.63894105		2.0

Structure	Molecule Name	affinity	Total Molweight	cLogP	H-Acceptors	H-Donors	Total Surface Area	Relative PSA	Polar Surface Area	Druglikeness	Kd (nM) @ 20°C	LE from K...	LLE from Kd ...	LELP from Kd (n...	Shape Index	Molecular...	Neighbor Si...	Neig...	Neighbor	Neighbor ...	Neighbor ...	Ring Systems	frequency
	ZINC03775000	-7.2	397.466	-3.3556	8	1	311.56	0.31821	127.82	-8.1512	4285.064499754	0.26301	8.7236	-12.758	0.60714	0.63381		0		-0.7964682	0.6003821		1.0
	ZINC03830513	-7.2	422.461	-2.1376	9	1	291.75	0.47095	179.33	10.36	4285.064499754	0.26301	7.5056	-8.1274	0.60714	0.49807	0.9428 (max of	3	2180; 2368; 4258	0.05350410	-0.8937965		1.0
	ZINC03839671	-7.2	370.429	-2.5147	9	2	256.89	0.44175	147.34	7.155	4285.064499754	0.29457	7.8827	-8.5368	0.6	0.36333	0.92315 (max o	2	6702; 6092	-0.3207965	0.71071446		1.0
	ZINC04084252	-7.2	385.527	-2.1425	7	3	295.29	0.3439	140.24	3.5615	4285.064499754	0.28324	7.5105	-7.5642	0.65385	0.5245	0.89688	1	7782	0.5960899	-0.0240929		1.0
	ZINC04085637	-7.2	354.465	-0.9097	6	1	262.82	0.26037	73.9	-11.215	4285.064499754	0.29457	6.2777	-3.0882	0.52	0.43218	0.98434 (max o	4	6951; 3641; 3831; 40	0.1127186	0.52409536		1.0
	ZINC04098206	-7.2	392.403	1.1199	8	2	289.19	0.32961	119.36	-11.485	4285.064499754	0.26301	4.2481	4.258	0.42857	0.53096	0.92774	1	5517	0.88202876	-0.0551477		1.0
	ZINC04260900	-7.2	353.378	-1.7531	10	1	257.57	0.37605	99.97	-0.20981	4285.064499754	0.29457	7.1211	-5.9513	0.64	0.44703	0.9083	1	449	-0.2206260	-0.1620932		1.0
	ZINC05439329	-7.2	354.433	-1.6869	7	1	278.46	0.26111	70.84	2.6546	4285.064499754	0.28324	7.0549	-5.9557	0.5	0.42658	0.89424 (max o	4	3264; 4566; 5776; 57	-0.4269879	-0.3169300		1.0
	ZINC08296074	-7.2	378.515	0.8733	6	4	265.28	0.28479	126.06	5.2911	4285.064499754	0.28324	4.4947	3.0832	0.42308	0.43836		0		0.08373791	0.9425801		1.0
	ZINC08770003	-7.2	403.453	-0.2132	8	2	301.42	0.27706	109.75	-1.5143	4285.064499754	0.25394	5.5812	-0.83956	0.58621	0.52439		0		0.2070568	0.95165235		1.0
	ZINC08880009	-7.2	383.468	0.0053	8	1	277.15	0.35436	121.32	3.0025	4285.064499754	0.28324	5.3627	0.018712	0.61538	0.54169	0.94008 (max o	2	5836; 6522	0.9098683	0.40167573		1.0
	ZINC12481197	-7.2	360.452	-0.9394	6	1	296.53	0.24112	74.11	2.3917	4285.064499754	0.28324	6.3074	-3.3166	0.53846	0.58302	0.89169 (max o	2	2982; 4291	-0.0048172	-0.2430548		1.0
	ZINC12483115	-7.2	368.496	-1.1398	7	2	270.56	0.22538	78.85	1.2611	4285.064499754	0.28324	6.5078	-4.0241	0.57692	0.48194	0.86774	1	5476	0.4203986	0.04537997		1.0
	ZINC12530578	-7.2	351.469	-1.9899	7	3	282.17	0.29479	82.95	5.7936	4285.064499754	0.29457	7.3579	-6.7552	0.56	0.4963	0.91999 (max o	5	7499; 7021; 632; 465	-0.2350940	0.09392637		1.0
	ZINC14649459	-7.2	379.439	-8.2916	10	4	292.29	0.42006	180.13	-1.8498	4285.064499754	0.27275	13.66	-30.4	0.66667	0.53331		0		-0.9325663	-0.2595228		1.0
	ZINC14689570	-7.2	390.387	0.6557	8	4	282.69	0.33761	125.68	-1.4102	4285.064499754	0.26301	4.7123	2.4931	0.46429	0.31552	0.92152 (max o	3	7193; 2046; 5207	-0.7302303	-0.0994310		1.0
	ZINC20463215	-7.2	365.516	-1.5753	6	1	288.27	0.19149	80.29	-0.21797	4285.064499754	0.28324	6.9433	-5.5617	0.53846	0.57288	0.90599 (max o	3	7031; 6845; 6498	-0.2643948	-0.1692189		1.0
	ZINC20463993	-7.2	374.483	-3.1629	7	3	294.47	0.25901	77.56	2.7053	4285.064499754	0.27275	8.5309	-11.596	0.55556	0.49416	0.89688	1	5924	0.08515779	0.9762787		1.0
	ZINC20465099	-7.2	352.457	-2.2675	8	3	264.4	0.27602	98.56	3.948	4285.064499754	0.29457	7.6355	-7.6976	0.56	0.44178	0.95631 (max o	5	5143; 5559; 5565; 62	-0.4899121	0.12762912		1.0
	ZINC20465274	-7.2	371.476	-2.314	7	4	285.77	0.3802	120.7	3.5007	4285.064499754	0.29457	7.682	-7.8555	0.52	0.46181	0.9594 (max of	2	7522; 6581	-0.7893948	-0.5510472		1.0
	ZINC20465824	-7.2	366.500	-2.5394	6	4	295.88	0.24175	79.97	-4.1764	4285.064499754	0.28324	7.9074	-8.9655	0.57692	0.50304	0.92368 (max o	4	5570; 5942; 6578; 65	0.53107876	0.7579466		1.0
	ZINC20465824	-7.2	365.492	-1.3147	6	4	299.6	0.25117	75.39	-2.1304	4285.064499754	0.28324	6.6827	-4.6416	0.57692	0.55925	0.91351 (max o	4	5157; 6269; 6579; 65	0.30795777	-0.0309161		1.0
	ZINC20465824	-7.2	365.492	-1.1731	6	3	281.84	0.20398	78.77	0.47107	4285.064499754	0.28324	6.5411	-4.1417	0.57692	0.49671	0.96998 (max o	4	5571; 5943; 6580; 65	-0.4221304	0.774404		1.0
	ZINC20465937	-7.2	363.476	-1.0777	6	4	293.59	0.26666	83.23	0.60121	4285.064499754	0.28324	6.4457	-3.8049	0.53846	0.50943	0.91396 (max o	5	7399; 7223; 5568; 55	0.2592483	-0.1171811		1.0
	ZINC20504963	-7.2	354.449	-0.8601	8	3	278.85	0.32584	113.76	2.8826	4285.064499754	0.29457	6.2281	-2.9198	0.56	0.58457	0.87576 (max o	2	2819; 3410	-0.2911898	0.16500884		1.0
	ZINC20610299	-7.2	377.464	0.7218	7	0	267.23	0.27774	87.62	10.123	4285.064499754	0.28324	4.6462	2.5483	0.53846	0.35816	0.92368	1	4325	-0.7633195	0.6194402		1.0
	ZINC20611195	-7.2	439.511	-4.8556	10	3	324.74	0.4272	198.66	5.6596	4285.064499754	0.24548	10.224	-19.78	0.56667	0.62271	0.88322	1	6838	-0.8217436	0.5470039		1.0
	ZINC20761552	-7.2	362.341	0.4563	10	3	258.65	0.41388	118.23	3.2492	4285.064499754	0.28324	4.9117	1.611	0.46154	0.44636	0.92702	1	4765	-0.1734406	0.95580614		1.0
	ZINC22920605	-7.2	351.489	-1.9173	6	1	274.51	0.20109	80.29	2.237	4285.064499754	0.29457	7.2853	-6.5088	0.52	0.54524	0.90599 (max o	3	7727; 6497; 6804	-0.2845110	-0.1780365		1.0
	ZINC31159670	-7.2	362.377	0.7609	7	1	261.09	0.36187	97.89	-19.088	4285.064499754	0.28324	4.6071	2.6864	0.38462	0.26938		0		0.70715785	0.47397596		1.0
	ZINC35454632	-7.2	437.463	-0.941	9	3	323.23	0.34867	153.42	-0.45976	4285.064499754	0.23756	6.309	-3.9611	0.48387	0.48961	0.95771 (max o	3	4405; 5627; 6323	-0.8881241	-0.1076919		1.0
	ZINC35882819	-7.2	370.293	-3.3813	12	0	263.6	0.50459	156.22	3.7857	4285.064499754	0.28324	8.7493	-11.938	0.46154	0.44595	0.93686 (max o	5	7745; 7700; 7647; 66	0.4210407	-0.3412793		1.0
	ZINC67902318	-7.2	376.436	0.3197	8	1	261.92	0.33873	107.49	6.2161	4285.064499754	0.28324	5.0483	1.1287	0.53846	0.40268	0.86499 (max o	5	2489; 3270; 4151; 50	0.5375197	0.52918017		1.0
	ZINC68590695	-7.2	362.330	-3.6103	10	6	234.68	0.53797	161.6	-4.3947	4285.064499754	0.29457	8.9783	-12.256	0.44	0.34878	0.9402 (max of	3	4438; 6050; 6677	0.55438924	0.5591601		1.0
	ZINC71405305	-7.2	380.492	0.6093	7	1	263.12	0.37656	126.5	7.7992	4285.064499754	0.29457	4.7587	2.0684	0.56	0.44066	0.88478	1	6975	0.4968658	0.47048858		1.0
	ZINC20610209	-7.2	441.475	-0.0128	10	2	323.63	0.3458	139.67	4.6336	4285.064499754	0.23756	5.3808	-0.053881	0.3871	0.42654	0.93139	1	3012	-0.2174298	0.94690067		0.0
	ZINC05317304	-7.2	358.413	-1.7779	9	3	283.96	0.40368	150.65	-57.945	4285.064499754	0.29457	7.1459	-6.0355	0.52	0.64764	0.97118 (max o	5	7763; 7681; 7325; 69	-0.2446973	0.84403926		0.0
	ZINC31160671	-7.2	356.413	-1.9376	7	3	287.28	0.327	140.95	-4.9219	4285.064499754	0.29457	7.3056	-6.5777	0.64	0.75283	0.99666 (max o	5	7957; 7799; 7415; 72	0.00978518	0.68438953		0.0
	ZINC72320203	-7.2	441.547	-1.2298	10	3	371.62	0.31594	147.74	2.5658	4285.064499754	0.23756	6.5978	-5.1768	0.80645	0.68952		0		-0.9089097	0.3641873		0.0
	ZINC04632132	-7.2	364.436	-0.6006	6	2	317.84	0.25434	120.72	-11.872	4285.064499754	0.28324	5.9686	-2.1204	0.84615	0.55385		0		-0.8378595	0.39782357		0.0
	ZINC02115609	-7.1	394.423	-1.4299	8	1	291.47	0.38546	139.29	3.977	5087.605662383	0.26896	6.7234	-5.3163	0.55556	0.53674	0.94927 (max o	8	7120; 3320; 3572; 35	0.05808826	0.11847842		172.0
	ZINC02112459	-7.1	394.423	-1.4299	8	1	291.47	0.38546	139.29	3.977	5087.605662383	0.26896	6.7234	-5.3163	0.55556	0.53399	0.94927 (max o	8	7120; 3586; 4900; 57	0.01892477	0.11961336		172.0
	ZINC49180795	-7.1	352.362	-1.4927	8	3	270.67	0.37337	135.99	2.2083	5087.605662383	0.29048	6.7862	-5.1387	0.56	0.61986	0.98434 (max o	5	7446; 7419; 6297; 63	0.4073453	0.4088187		134.0

Structure	Molecule Name	affinity	Total Molweight	cLogP	H-Acceptors	H-Donors	Total Surface Area	Relative PSA	Polar Surface Area	Druglikeness	Kd (nM) @ 20°C	LE from K...	LLE from Kd ...	LELP from Kd (n...	Shape Index	Molecular...	Neighbor Si...	Neig...	Neighbor	Neighbor ...	Neighbor ...	Ring Systems	frequency
	ZINC31167853	-7.1	352.362	-1.4927	8	3	270.67	0.37337	135.99	2.2083	5087.605662383	0.29048	6.7862	-5.1387	0.64	0.66395	0.98434 (max o	8	7800; 7562; 7446; 74	0.35107458	0.3934045		134.0
	ZINC04654927	-7.1	438.634	-4.7516	8	6	364.88	0.2523	139.29	0.031661	5087.605662383	0.23426	10.045	-20.284	0.70968	0.62442	0.96018	1	7329	0.02844027	-0.9848465		134.0
	ZINC03874235	-7.1	409.549	-0.809	7	3	324	0.33423	151.27	3.1065	5087.605662383	0.25936	6.1025	-3.1192	0.57143	0.59698	0.96519 (max o	4	7760; 7489; 7297; 71	0.47009444	0.66956687		134.0
	ZINC03844950	-7.1	373.984	-1.8615	4	0	210.5	0.25957	80.26	-8.2415	5087.605662383	0.40345	7.155	-4.614	0.61111	0.55406		0		0.83207756	-0.4473485		134.0
	ZINC35457657	-7.1	367.416	-0.5417	7	3	280.83	0.31927	127.12	-7.3952	5087.605662383	0.27931	5.8352	-1.9394	0.46154	0.62833	0.97269 (max o	3	7437; 7436; 1608	0.11318683	0.8093204		127.0
	ZINC04089657	-7.1	383.531	-0.4733	7	3	311.79	0.29122	125.97	3.1439	5087.605662383	0.26896	5.7668	-1.7597	0.62963	0.613	0.96885 (max o	6	7501; 7136; 3208; 61	0.56922305	0.02368033		127.0
	ZINC04089700	-7.1	402.514	-3.3436	9	4	307.53	0.44418	194.36	3.1139	5087.605662383	0.26896	8.6371	-12.431	0.66667	0.60896	0.92094 (max o	11	7764; 7587; 7470; 74	0.5353355	0.13926406		127.0
	ZINC31170261	-7.1	395.426	-0.9065	8	4	297.67	0.34521	147.35	-8.9294	5087.605662383	0.25936	6.2	-3.4952	0.46429	0.60852	0.95983 (max o	4	7556; 7248; 6303; 68	0.4688871	-0.5627189		127.0
	ZINC04089584	-7.1	416.517	-4.5674	9	3	320.3	0.42338	191.4	2.3362	5087.605662383	0.25936	9.8609	-17.61	0.67857	0.61367	0.96023 (max o	6	7764; 7587; 7314; 69	0.5552396	0.13146529		127.0
	ZINC04089614	-7.1	401.570	-0.9968	7	3	318.28	0.34024	151.27	2.5304	5087.605662383	0.26896	6.2903	-3.7061	0.66667	0.61214	0.92721 (max o	13	7764; 7587; 7470; 74	0.53955406	0.0987046		127.0
	ZINC12887151	-7.1	389.515	-2.6913	8	4	298.61	0.40652	171.5	0.74603	5087.605662383	0.27931	7.9848	-9.6356	0.65385	0.60667	0.98742 (max o	8	7764; 7470; 7471; 73	0.50268066	0.11001133		127.0
	ZINC31160523	-7.1	350.406	-0.6232	8	6	256.52	0.38438	139.84	-11.141	5087.605662383	0.30258	5.9167	-2.0596	0.5	0.67588	0.9665 (max of	3	7240; 6858; 7074	-0.6850325	-0.3609275		120.0
	ZINC77257666	-7.1	363.381	-2.8728	9	5	272.09	0.41464	159.74	-12.771	5087.605662383	0.29048	8.1663	-9.8898	0.52	0.57085	0.98897 (max o	3	7279; 6920; 7111	-0.8514445	0.06431305		120.0
	ZINC77257664	-7.1	363.381	-2.8728	9	5	272.09	0.41464	159.74	-12.771	5087.605662383	0.29048	8.1663	-9.8898	0.52	0.57904	0.98897 (max o	3	7279; 7112; 6920	-0.8170304	0.06531589		120.0
	ZINC31160527	-7.1	350.406	-0.6232	8	6	256.52	0.38438	139.84	-11.141	5087.605662383	0.30258	5.9167	-2.0596	0.5	0.67073	0.9665 (max of	5	7545; 7414; 7240; 68	-0.6676895	-0.374665		120.0
	ZINC49601688	-7.1	365.354	-3.573	10	4	261.9	0.46873	165.81	1.5639	5087.605662383	0.29048	8.8665	-12.3	0.56	0.66255	0.94678 (max o	6	7748; 7658; 7567; 74	-0.5871008	-0.3576537		120.0
	ZINC34943430	-7.1	436.502	-3.8395	11	4	288.25	0.57929	238.9	-8.9899	5087.605662383	0.27931	9.133	-13.746	0.5	0.59806	0.98956 (max o	5	7752; 7688; 7689; 75	-0.3575714	0.6230418		120.0
	ZINC02169845	-7.1	377.137	-0.2002	8	3	201.1	0.45375	119.31	-1.9084	5087.605662383	0.38221	5.4937	-0.52379	0.52632	0.26456	0.86791	1	4257	-0.5348689	0.4034903		67.0
	ZINC31155496	-7.1	357.334	-2.8838	9	4	256.48	0.42779	148.74	-4.8025	5087.605662383	0.29048	8.1773	-9.9277	0.48	0.4148	0.90171 (max o	3	6293; 6295; 6607	0.21059456	-0.6644424		49.0
	ZINC13515708	-7.1	370.353	-0.8638	9	4	271.98	0.38768	134.91	-7.8087	5087.605662383	0.27931	6.1573	-3.0926	0.53846	0.44162	0.95257 (max o	2	7139; 6928	0.6121183	-0.1681346		49.0
	ZINC03779261	-7.1	372.369	-0.6515	9	5	272.55	0.38708	138.07	-4.1548	5087.605662383	0.27931	5.945	-2.3325	0.53846	0.46185	0.95257 (max o	2	7139; 7008	0.6329298	-0.1963507		49.0
	ZINC04083637	-7.1	373.516	-1.3477	7	2	290.86	0.34511	142.48	5.1691	5087.605662383	0.29048	6.6412	-4.6395	0.64	0.57501	0.9654 (max of	6	7815; 7586; 7134; 71	0.7797097	-0.4210847		47.0
	ZINC12875748	-7.1	373.516	-1.3477	7	2	290.86	0.34511	142.48	5.6742	5087.605662383	0.29048	6.6412	-4.6395	0.64	0.58155	0.9654 (max of	5	7815; 7586; 7134; 71	0.7607265	-0.3905263		47.0
	ZINC02137766	-7.1	359.489	-1.8021	7	2	277.1	0.36225	142.48	5.0763	5087.605662383	0.30258	7.0956	-5.9557	0.625	0.55621		0		-0.3681206	0.7633625		47.0
	ZINC12501360	-7.1	361.206	-8.4657	13	4	230.21	0.69893	217.22	-20.626	5087.605662383	0.30258	13.759	-27.978	0.41667	0.39494	0.92257 (max o	4	2148; 4055; 5796; 61	-0.3380094	-0.4117177		44.0
	ZINC01319228	-7.1	370.313	-1.0894	11	1	264.05	0.45525	137.54	0.69325	5087.605662383	0.27931	6.3829	-3.9003	0.46154	0.44351	0.94008 (max o	9	7129; 7128; 7115; 70	0.38683626	-0.4847352		41.0
	ZINC20758139	-7.1	406.482	-0.6481	8	2	304.56	0.36249	143.94	5.6406	5087.605662383	0.25936	5.9416	-2.4989	0.5	0.55991	0.96519 (max o	9	4244; 4760; 5507; 55	0.30912173	0.7260834		36.0
	ZINC04517519	-7.1	351.318	-0.7704	11	2	248.49	0.49994	151.68	1.3398	5087.605662383	0.29048	6.0639	-2.6522	0.44	0.25022	0.9402 (max of	7	7478; 4979; 4980; 51	-0.6181000	0.31767187		33.0
	ZINC19323200	-7.1	387.495	-2.9723	8	6	283.3	0.35185	125.9	3.5199	5087.605662383	0.26896	8.2658	-11.051	0.44444	0.62195	0.96998 (max o	7	7368; 7367; 7200; 59	-0.2631009	0.7158686		23.0
	ZINC19322200	-7.1	359.441	-3.4089	8	6	261.3	0.38148	125.9	2.5969	5087.605662383	0.29048	8.7024	-11.735	0.52	0.61942	0.91693 (max o	3	7371; 7369; 6797	-0.6511268	0.2595367		23.0
	ZINC19323243	-7.1	359.441	-3.4089	8	6	261.3	0.38148	125.9	3.8635	5087.605662383	0.29048	8.7024	-11.735	0.48	0.63719	0.95631 (max o	4	7616; 7512; 7371; 67	-0.6616427	0.2398925		23.0
	ZINC20464938	-7.1	404.888	-1.6475	7	4	309.9	0.26409	86.47	3.1332	5087.605662383	0.26896	6.941	-6.1254	0.55556	0.60198	0.92064 (max o	7	7521; 7397; 7222; 43	-0.3225464	0.05115004		22.0
	ZINC20465530	-7.1	415.509	-0.748	9	3	296.68	0.31462	119.95	7.585	5087.605662383	0.25936	6.0415	-2.884	0.5	0.53031	0.91157 (max o	4	4751; 5552; 5937; 68	-0.3257271	0.15376835		21.0
	ZINC20465589	-7.1	366.480	-1.7845	7	3	293.24	0.24175	69.84	3.9558	5087.605662383	0.27931	7.078	-6.389	0.5	0.48412	0.90719 (max o	2	5566; 6585	-0.4345213	0.1828125		21.0
	ZINC08636378	-7.1	391.490	0.7135	7	3	290.56	0.32971	117.36	3.1197	5087.605662383	0.26896	4.58	2.6528	0.51852	0.45337	0.9083 (max of	7	7775; 7160; 7161; 28	0.43284377	0.14498687		18.0
	ZINC08254659	-7.1	379.503	-1.8247	7	5	295.28	0.37693	121.36	3.7352	5087.605662383	0.27931	7.1182	-6.5329	0.5	0.43591	0.93686 (max o	6	7492; 7333; 6987; 58	0.41278172	0.15494247		18.0
	ZINC08636323	-7.1	409.529	-1.8947	8	5	317.54	0.382	130.59	3.8539	5087.605662383	0.25936	7.1882	-7.3053	0.53571	0.45447	0.93069 (max o	8	7775; 7492; 7160; 71	0.41532168	0.19622411		18.0
	ZINC20462958	-7.1	394.509	-1.7271	6	2	339.98	0.23263	69.32	2.7697	5087.605662383	0.25936	7.0206	-6.6591	0.57143	0.64903	0.9399 (max of	6	7381; 7208; 5130; 66	-0.0083290	-0.0947605		18.0
	ZINC20462958	-7.1	393.501	-2.1338	6	1	325.94	0.19958	68.12	5.1994	5087.605662383	0.25936	7.4273	-8.2272	0.57143	0.66407	0.9399 (max of	5	7379; 7206; 5128; 68	-0.0510328	-0.0787080		18.0
	ZINC20462958	-7.1	393.501	-2.1338	6	1	325.94	0.19958	68.12	4.3253	5087.605662383	0.25936	7.4273	-8.2272	0.57143	0.66161	0.9399 (max of	7	7380; 7207; 7036; 51	-0.0270013	-0.1075406		18.0
	ZINC13348057	-7.1	383.349	-4.4475	12	8	254.94	0.57347	198.4	-3.0597	5087.605662383	0.27931	9.741	-15.923	0.42308	0.46124	0.95559 (max o	5	7911; 7830; 7502; 65	0.46998653	-0.4465643		17.0
	ZINC33851453	-7.1	397.375	-4.0196	12	7	270.85	0.52834	187.4	-6.2954	5087.605662383	0.26896	9.3131	-14.945	0.40741	0.4601	0.94622 (max o	7	7594; 7552; 7480; 73	0.5319655	-0.5175653		17.0
	ZINC13348374	-7.1	383.349	-4.4475	12	8	254.94	0.57347	198.4	-4.7202	5087.605662383	0.27931	9.741	-15.923	0.42308	0.46223	0.94951 (max o	9	7594; 7552; 7479; 74	0.4764156	-0.5259138		17.0

Structure	Molecule Name	affinity	Total Molweight	cLogP	H-Acceptors	H-Donors	Total Surface Area	Relative PSA	Polar Surface Area	Druglikeness	Kd (nM) @ 20°C	LE from K...	LLE from Kd ...	LELP from Kd (n...	Shape Index	Molecular...	Neighbor Si...	Neig...	Neighbor	Neighbor ...	Neighbor ...	Ring Systems	frequency
	ZINC67913289	-7.1	444.475	-0.5622	10	6	314.74	0.39614	173.98	-11.767	5087.605662383	0.23426	5.8557	-2.3999	0.41935	0.54324	0.96739 (max o	5	7449; 4395; 4818; 60	0.39772823	0.30269626		16.0
	ZINC35271191	-7.1	426.460	0.0489	9	4	309.91	0.35985	150.59	-12.343	5087.605662383	0.24207	5.2446	0.20201	0.43333	0.53443	0.96589 (max o	5	7449; 7084; 4818; 52	0.3772536	0.27370855		16.0
	ZINC31170366	-7.1	426.460	0.0489	9	4	309.91	0.35985	150.59	-12.343	5087.605662383	0.24207	5.2446	0.20201	0.43333	0.53144	0.96343 (max o	5	7423; 7422; 7251; 66	0.45118862	0.2628352		16.0
	ZINC03978029	-7.1	425.996	-1.1969	7	5	318.59	0.34176	128.76	0.91283	5087.605662383	0.26896	6.4904	-4.45	0.51852	0.45426	0.97658 (max o	9	7679; 7464; 7298; 69	0.7013365	-0.2724251		15.0
	ZINC04081954	-7.1	407.550	-2.4	8	6	309.52	0.39409	148.99	0.28877	5087.605662383	0.26896	7.6935	-8.9231	0.51852	0.44445	0.98806 (max o	8	7679; 7464; 7460; 72	0.71495426	-0.2885503		15.0
	ZINC03830582	-7.1	425.996	-1.1969	7	5	318.59	0.34176	128.76	0.91283	5087.605662383	0.26896	6.4904	-4.45	0.51852	0.47411	0.97658 (max o	16	7679; 7460; 7428; 74	0.695175	-0.2355548		15.0
	ZINC03830583	-7.1	425.996	-1.1969	7	5	318.59	0.34176	128.76	0.91283	5087.605662383	0.26896	6.4904	-4.45	0.51852	0.47411	0.97658 (max o	5	7291; 7122; 7104; 69	0.67148846	-0.2023067		15.0
	ZINC65739439	-7.1	411.969	-1.6513	7	5	304.83	0.35718	128.76	2.9342	5087.605662383	0.27931	6.9448	-5.9121	0.5	0.47254	0.97533 (max o	6	7460; 7428; 7429; 68	0.6832298	-0.2190602		15.0
	ZINC79188403	-7.1	363.436	-1.2265	8	2	300.67	0.29996	84.67	4.0902	5087.605662383	0.27931	6.52	-4.3912	0.61538	0.5434	0.8934 (max of	4	7425; 4452; 5690; 56	-0.1174745	-0.3722012		15.0
	ZINC20763253	-7.1	420.464	-0.1927	10	3	326.42	0.3376	129.56	7.6454	5087.605662383	0.24207	5.4862	-0.79606	0.56667	0.57827	0.97975 (max o	6	7426; 3863; 6275; 62	0.00108200	-0.3380309		15.0
	ZINC22922134	-7.1	364.464	-1.5988	7	2	267.03	0.21698	71.01	6.8687	5087.605662383	0.27931	6.8923	-5.7241	0.61538	0.47566	0.93373 (max o	7	7067; 3842; 5179; 62	0.44513837	-0.1776788		14.0
	ZINC22922155	-7.1	364.464	-1.5988	7	2	267.03	0.21698	71.01	6.8687	5087.605662383	0.27931	6.8923	-5.7241	0.53846	0.4568	0.94338 (max o	5	7347; 6253; 6283; 65	0.427954	-0.1303559		14.0
	ZINC12483730	-7.1	366.436	-0.9299	8	4	288.18	0.33538	93.49	-0.39401	5087.605662383	0.27931	6.2234	-3.3293	0.53846	0.50478	0.95257 (max o	3	5474; 6767; 6993	0.8786721	0.25954506		14.0
	ZINC12483705	-7.1	354.400	-0.7591	7	4	272.27	0.31825	84.26	-1.8725	5087.605662383	0.29048	6.0526	-2.6133	0.56	0.4916	0.9302 (max of	3	6994; 5474; 6767	0.86863315	0.28158164		14.0
	ZINC05412659	-7.1	415.441	0.082	9	1	293.06	0.32847	121.47	-1.0337	5087.605662383	0.25936	5.2115	0.31617	0.5	0.57368	0.89979 (max o	3	7156; 3256; 4992	0.02358526	0.35311946		13.0
	ZINC03842118	-7.1	363.412	0.3739	8	0	280.49	0.24874	79.39	2.9669	5087.605662383	0.27931	4.9196	1.3387	0.57692	0.62131	0.97791 (max o	3	7582; 5338; 6452	0.10248294	0.39599088		13.0
	ZINC04073853	-7.1	407.533	-1.0329	7	2	313.6	0.32009	142.48	5.0647	5087.605662383	0.25936	6.3264	-3.9825	0.64286	0.52621	0.96519 (max o	5	4515; 5366; 5490; 61	0.32501414	0.33537462		12.0
	ZINC20464958	-7.1	402.534	-2.3238	8	4	311.22	0.3129	111.92	-1.5855	5087.605662383	0.26896	7.6173	-8.6398	0.55556	0.60588	0.97658 (max o	4	4306; 5928; 6259; 68	-0.3996973	-0.1445978		12.0
	ZINC35271303	-7.1	412.433	0.2102	9	4	295.62	0.37724	150.59	-8.6504	5087.605662383	0.25041	5.0833	0.83941	0.41379	0.52952	0.96682 (max o	7	7900; 7449; 4395; 52	0.41592476	0.2897131		12.0
	ZINC13545957	-7.1	383.349	-4.4475	12	8	254.94	0.57347	198.4	-4.7796	5087.605662383	0.27931	9.741	-15.923	0.46154	0.46983	0.95257 (max o	7	4541; 4964; 5097; 53	0.20775299	0.74707717		12.0
	ZINC04085044	-7.1	365.453	-1.4662	6	1	254.82	0.40574	140.14	6.1016	5087.605662383	0.30258	6.7597	-4.8456	0.54167	0.41641	0.95709 (max o	3	7309; 7308; 6713	-0.7025624	-0.6727178		10.0
	ZINC04531088	-7.1	352.386	-0.754	8	4	265.42	0.35868	117.12	-0.49157	5087.605662383	0.29048	6.0475	-2.5957	0.48	0.47801	0.95 (max of 3)	3	7147; 6152; 6153	-0.1179094	0.7923524		10.0
	ZINC20465530	-7.1	416.517	-2.8577	9	4	310.72	0.34558	121.15	4.5621	5087.605662383	0.25936	8.1512	-11.018	0.5	0.53585	0.91157 (max o	7	3401; 4313; 4752; 55	-0.3729313	-0.542296		10.0
	ZINC08215630	-7.1	385.027	-17.294	14	2	225.42	0.83693	273.32	-45.594	5087.605662383	0.33009	22.587	-52.391	0.54545	0.6848	0.88407 (max o	2	6980; 6981	-0.9201963	-0.1332461		10.0
	ZINC08215630	-7.1	386.034	-16.046	14	3	224.24	0.83607	270.49	-53.943	5087.605662383	0.33009	21.34	-48.612	0.54545	0.68507	0.87552 (max o	2	6982; 6980	-0.9354722	-0.1474244		10.0
	ZINC08215630	-7.1	386.034	-16.046	14	3	224.24	0.83607	270.49	-53.185	5087.605662383	0.33009	21.34	-48.612	0.54545	0.66548	0.88407 (max o	2	6982; 6981	-0.9385583	-0.1248041		10.0
	ZINC12661729	-7.1	390.340	-0.2551	11	0	286.31	0.43729	140.73	-0.85962	5087.605662383	0.26896	5.5486	-0.94845	0.40741	0.42906	0.96885	1	7167	0.82574886	0.29227626		10.0
	ZINC05664943	-7.1	390.340	-0.2551	11	0	286.31	0.43729	140.73	-0.85962	5087.605662383	0.26896	5.5486	-0.94845	0.40741	0.39737	0.93373 (max o	4	7788; 7698; 7506; 71	0.7732176	0.2894815		10.0
	ZINC04073864	-7.1	428.531	-0.47	8	3	331.28	0.29244	132.97	5.9299	5087.605662383	0.23426	5.7635	-2.0063	0.58065	0.4936	0.97423 (max o	10	438; 475; 487; 566; 9	0.3039475	0.27735257		8.0
	ZINC04654875	-7.1	373.563	-3.5633	5	4	311.64	0.17475	89.96	-0.92296	5087.605662383	0.26896	8.8568	-13.248	0.77778	0.59488		0		-0.2548707	0.90816784		8.0
	ZINC04095838	-7.1	368.246	-9.3091	13	6	230.16	0.70925	229.33	-0.5377	5087.605662383	0.29048	14.603	-32.047	0.44	0.44121	0.94351	1	6956	-0.7250915	0.4187817		8.0
	ZINC04095837	-7.1	368.246	-9.3091	13	6	230.16	0.70925	229.33	-0.5377	5087.605662383	0.29048	14.603	-32.047	0.44	0.44121	0.94351	1	6957	-0.7332868	0.43790266		8.0
	ZINC20463905	-7.1	393.482	-4.0587	8	4	297.28	0.32542	112.06	2.4571	5087.605662383	0.25936	9.3522	-15.649	0.53571	0.4614	0.94883 (max o	6	7177; 3389; 3771; 55	0.57192355	-0.1522844		8.0
	ZINC70666305	-7.1	359.357	-0.8644	8	2	260.62	0.37929	120.55	0.018054	5087.605662383	0.27931	6.1579	-3.0948	0.5	0.32755	0.94008 (max o	4	7274; 4440; 5268; 69	0.47967705	0.39860877		8.0
	ZINC70666363	-7.1	359.357	-0.8644	8	2	260.62	0.37929	120.55	0.018054	5087.605662383	0.27931	6.1579	-3.0948	0.42308	0.30714	0.94008 (max o	4	7274; 4872; 6053; 69	0.465033	0.38569325		8.0
	ZINC12480649	-7.1	390.522	-0.0799	6	1	333.05	0.21468	74.11	3.1926	5087.605662383	0.25936	5.3734	-0.30807	0.57143	0.6711	0.92757 (max o	7	7377; 7378; 3836; 47	-0.0190755	-0.1674247		8.0
	ZINC03873146	-7.1	405.493	-3.2679	8	2	315.18	0.2965	144.82	2.4951	5087.605662383	0.25041	8.5614	-13.05	0.48276	0.53252	0.95642 (max o	4	7125; 5319; 6103; 67	-0.8317162	-0.4507972		8.0
	ZINC15676432	-7.1	433.484	-2.4656	9	1	310.9	0.32586	140.34	2.4759	5087.605662383	0.24207	7.7591	-10.186	0.56667	0.57305	0.9561 (max of	7	7196; 7017; 5895; 63	-0.2820223	0.37840694		8.0
	ZINC14719125	-7.1	356.370	0.1552	8	1	277.9	0.33185	108.36	-3.5573	5087.605662383	0.29048	5.1383	0.53429	0.52	0.56574	0.96243 (max o	7	7360; 5992; 5993; 66	-0.0149509	-0.7714893		8.0
	ZINC31168997	-7.1	388.455	0.7308	8	4	293.23	0.32548	125.68	-15.27	5087.605662383	0.26896	4.5627	2.7171	0.48148	0.51734	0.94905 (max o	3	6621; 6622; 6866	0.9269218	0.07386841		8.0
	ZINC35464703	-7.1	356.413	-2.5089	7	2	285.37	0.34513	133.25	-4.0636	5087.605662383	0.29048	7.8024	-8.637	0.64	0.61937	0.98434 (max o	3	7652; 7651; 7440	-0.6037752	0.6878491		8.0
	ZINC09012616	-7.1	435.587	-0.682	7	3	341.02	0.31755	151.27	2.3408	5087.605662383	0.24207	5.9755	-2.8174	0.66667	0.55851	0.9167 (max of	5	3205; 4960; 5759; 64	0.5537204	-0.0757475		7.0
	ZINC61997001	-7.1	395.562	-0.3464	6	3	305.79	0.19795	86.61	-4.4229	5087.605662383	0.25936	5.6399	-1.3356	0.57143	0.55438	0.94573 (max o	3	7272; 5668; 6901	-0.6931163	0.21647464		7.0

Structure	Molecule Name	affinity	Total Molweight	cLogP	H-Acceptors	H-Donors	Total Surface Area	Relative PSA	Polar Surface Area	Druglikeness	Kd (nM) @ 20°C	LE from K...	LLE from Kd ...	LELP from Kd (n...	Shape Index	Molecular...	Neighbor Si...	Neig...	Neighbor	Neighbor ...	Neighbor ...	Ring Systems	frequency
	ZINC61997001	-7.1	395.562	-0.488	6	4	323.55	0.24197	83.23	-3.5843	5087.605662383	0.25936	5.7815	-1.8816	0.57143	0.58716	0.9428 (max of	3	7273; 5669; 6902	-0.4005811	0.8897579		7.0
	ZINC04201049	-7.1	418.452	-0.7606	11	3	327.32	0.37282	142.62	-6.2096	5087.605662383	0.24207	6.0541	-3.1421	0.53333	0.52593	0.89329 (max o	3	7319; 7318; 5523	-0.0301962	0.01655018		7.0
	ZINC03830580	-7.1	425.996	-1.1969	7	5	318.59	0.34176	128.76	0.91283	5087.605662383	0.26896	6.4904	-4.45	0.51852	0.47411	0.97658 (max o	7	7427; 7290; 7289; 71	0.6753874	-0.2406103		7.0
	ZINC04097283	-7.1	407.550	-2.4	8	6	309.52	0.39409	148.99	0.28877	5087.605662383	0.26896	7.6935	-8.9231	0.51852	0.4647	0.92094 (max o	3	7298; 7289; 6929	0.6603142	-0.2817792		7.0
	ZINC05433889	-7.1	350.417	-0.0077999	8	2	265.18	0.33147	94.6	-1.098	5087.605662383	0.29048	5.3013	-0.026852	0.6	0.39123	0.92032 (max o	6	3262; 4131; 5424; 54	-0.6205879	-0.5765040		6.0
	ZINC22922134	-7.1	365.472	-3.7085	7	3	281.07	0.25609	72.21	2.6139	5087.605662383	0.27931	9.002	-13.277	0.61538	0.47566	0.93069 (max o	8	7518; 3841; 5178; 62	0.50784266	-0.2129048		6.0
	ZINC22922155	-7.1	365.472	-3.7085	7	3	281.07	0.25609	72.21	2.6139	5087.605662383	0.27931	9.002	-13.277	0.53846	0.4568	0.94338 (max o	6	7628; 7347; 5178; 62	0.482803	-0.1852367		6.0
	ZINC05765543	-7.1	382.416	-3.6789	8	1	252.44	0.52916	183.21	11.228	5087.605662383	0.29048	8.9724	-12.665	0.48	0.32631	0.98188 (max o	6	3594; 4889; 4913; 53	-0.3889424	-0.4281173		6.0
	ZINC04097358	-7.1	382.416	-3.6789	8	1	252.44	0.52916	183.21	11.228	5087.605662383	0.29048	8.9724	-12.665	0.48	0.32631	0.98188 (max o	7	7494; 6978; 4481; 48	-0.3621365	-0.4475460		6.0
	ZINC15676432	-7.1	432.476	-2.2767	9	0	321.87	0.34884	139.09	3.0248	5087.605662383	0.24207	7.5702	-9.4052	0.56667	0.57305	0.9561 (max of	7	7195; 5896; 6318; 66	-0.2664769	0.34535077		6.0
	ZINC15675194	-7.1	373.855	-1.6431	7	4	275.89	0.28432	104.63	-0.61616	5087.605662383	0.29048	6.9366	-5.6565	0.68	0.50901	0.92641 (max o	9	3286; 3731; 3810; 41	0.1052361	-0.4614129		6.0
	ZINC20464989	-7.1	357.468	-3.5606	6	4	286.77	0.22694	73.98	1.4915	5087.605662383	0.29048	8.8541	-12.258	0.6	0.5408	0.91964 (max o	2	7631; 6575	-0.3762816	0.27842593		6.0
	ZINC20462969	-7.1	411.565	-2.8661	7	1	330.42	0.2439	93.57	-2.2463	5087.605662383	0.25936	8.1596	-11.051	0.53571	0.64669	0.90561 (max o	3	2981; 7037; 7039	0.8540761	-0.0783466		5.0
	ZINC20462969	-7.1	412.573	-2.4594	7	2	344.46	0.27472	94.77	-3.8308	5087.605662383	0.25936	7.7529	-9.4826	0.53571	0.63369	0.8991 (max of	2	7037; 7038	0.8341352	-0.0812553		5.0
	ZINC20462969	-7.1	411.565	-2.8661	7	1	330.42	0.2439	93.57	-1.3538	5087.605662383	0.25936	8.1596	-11.051	0.53571	0.64924	0.90561 (max o	2	7038; 7039	0.84516597	-0.0996025		5.0
	ZINC31155594	-7.1	378.376	0.3884	8	5	264.64	0.37235	136.68	-4.2659	5087.605662383	0.26896	4.9051	1.4441	0.40741	0.40595	0.82679 (max o	3	1570; 1572; 3987	-0.8625703	-0.4111451		5.0
	ZINC30879674	-7.1	414.524	-0.7396	7	4	334.13	0.24493	86.47	2.793	5087.605662383	0.24207	6.0331	-3.0553	0.56667	0.55736	0.92566 (max o	4	7411; 1252; 3406; 51	0.37946787	-0.3016027		5.0
	ZINC04096473	-7.1	383.349	-4.4475	12	8	254.94	0.57347	198.4	-4.7202	5087.605662383	0.27931	9.741	-15.923	0.42308	0.46983	0.95559 (max o	8	7831; 7010; 4541; 49	0.21881622	0.762308		5.0
	ZINC12659244	-7.1	427.409	0.2644	12	0	314.69	0.39343	141.11	4.2655	5087.605662383	0.24207	5.0291	1.0923	0.46667	0.49641	0.94241 (max o	3	5067; 6486; 6516	0.45326388	-0.3902918		5.0
	ZINC04260478	-7.1	424.521	-1.1283	8	0	302.7	0.37972	154.6	7.5733	5087.605662383	0.25936	6.4218	-4.3504	0.5	0.56162	0.96519 (max o	4	7477; 5782; 5783; 61	-0.1144311	-0.789075		5.0
	ZINC03844370	-7.1	383.400	-0.0995	10	1	292.57	0.35465	122.32	-0.45661	5087.605662383	0.26896	5.393	-0.36994	0.51852	0.64858	0.96885 (max o	3	7583; 7124; 7123	0.5545898	-0.5758982		5.0
	ZINC05398507	-7.1	405.453	-0.087	10	2	310.86	0.33182	120.94	2.3603	5087.605662383	0.25041	5.3805	-0.34742	0.48276	0.58566	0.90934 (max o	4	3683; 4567; 4930; 64	0.00614946	0.38266173		4.0
	ZINC15708262	-7.1	414.549	-3.1184	9	4	331.35	0.39113	128.46	3.5807	5087.605662383	0.25936	8.4119	-12.024	0.64286	0.58973	0.93373 (max o	7	7364; 7363; 4690; 70	-0.2123100	0.02585161		4.0
	ZINC15707529	-7.1	398.550	-2.2964	8	4	321.35	0.37218	119.23	5.4855	5087.605662383	0.26896	7.5899	-8.5379	0.62963	0.55995	0.93033 (max o	6	7365; 7364; 7362; 70	-0.2352754	0.02955669		4.0
	ZINC20462926	-7.1	405.537	-2.6597	7	1	343.35	0.21858	77.35	5.6335	5087.605662383	0.25041	7.9532	-10.621	0.58621	0.63143	0.93091 (max o	6	7207; 5046; 7030; 51	-0.1124659	-0.1516095		4.0
	ZINC20462926	-7.1	406.545	-2.253	7	2	357.39	0.24928	78.55	4.1152	5087.605662383	0.25041	7.5465	-8.9971	0.58621	0.61985	0.93091 (max o	6	7378; 7208; 5183; 68	-0.0911482	-0.1460651		4.0
	ZINC20462926	-7.1	405.537	-2.6597	7	1	343.35	0.21858	77.35	6.4924	5087.605662383	0.25041	7.9532	-10.621	0.58621	0.6337	0.93091 (max o	4	7206; 7030; 7029; 68	-0.1039528	-0.1099861		4.0
	ZINC35465098	-7.1	373.420	-1.7731	8	4	278.5	0.35806	139.51	-9.7968	5087.605662383	0.27931	7.0666	-6.3482	0.5	0.51159	0.9464 (max of	4	7851; 5241; 5642; 66	0.4464389	0.6011769		4.0
	ZINC67913284	-7.1	410.417	0.3539	9	3	294.52	0.37845	147.43	-9.4267	5087.605662383	0.25041	4.9396	1.4133	0.41379	0.5305	0.95908 (max o	5	7900; 5221; 5264; 60	0.4027292	0.3421663		4.0
	ZINC20463905	-7.1	391.466	-0.7243	8	3	286.96	0.30116	106.28	6.4734	5087.605662383	0.25936	6.0178	-2.7927	0.53571	0.49141	0.95461 (max o	5	7217; 3770; 5539; 55	-0.7810988	0.36257386		4.0
	ZINC20465595	-7.1	381.451	-3.6841	9	4	275.14	0.28277	108.64	5.5288	5087.605662383	0.26896	8.9776	-13.697	0.59259	0.50288	0.9083 (max of	7	2233; 2235; 2236; 34	-0.4335549	0.05056476		4.0
	ZINC35456138	-7.1	355.362	-2.4289	8	4	260.48	0.38283	139.51	-4.6528	5087.605662383	0.29048	7.7224	-8.3616	0.52	0.53277	0.98434 (max o	7	5632; 5633; 6000; 62	0.68630666	-0.3586728		4.0
	ZINC00803229	-7.1	362.448	-1.6501	7	4	289.84	0.27722	90.37	7.2815	5087.605662383	0.27931	6.9436	-5.9078	0.46154	0.51711	0.97533 (max o	2	7280; 6066	-0.1383298	0.84591687		4.0
	ZINC31169327	-7.1	351.417	0.013601	6	3	277.6	0.30357	113.35	-14.742	5087.605662383	0.29048	5.2799	0.046821	0.6	0.45193	0.93007 (max o	3	7258; 6312; 6623	0.69383055	-0.5792765		4.0
	ZINC25425825	-7.1	363.412	0.0378	8	2	283.05	0.30238	96.97	4.9464	5087.605662383	0.27931	5.2557	0.13533	0.53846	0.56517	0.93373 (max o	6	7230; 7229; 5403; 59	0.02480013	0.45416093		4.0
	ZINC20504953	-7.1	399.467	-0.4003	9	2	293.88	0.34412	122.42	5.4312	5087.605662383	0.26896	5.6938	-1.4883	0.51852	0.56938	0.87932 (max o	4	7229; 7069; 6150; 64	0.04874394	0.4593381		4.0
	ZINC04293458	-7.1	441.388	-0.0395	12	1	324.96	0.43319	156.67	2.0065	5087.605662383	0.23426	5.333	-0.16862	0.41935	0.40654	0.95521 (max o	5	7781; 7703; 7593; 73	0.31393644	0.3959147		4.0
	ZINC20462940	-7.1	381.559	-2.3343	6	1	327.63	0.19855	68.12	1.1619	5087.605662383	0.26896	7.6278	-8.6789	0.55556	0.64898	0.91351 (max o	6	7726; 7619; 7033; 33	-0.3178800	-0.1664231		4.0
	ZINC20462940	-7.1	382.567	-1.9276	6	2	341.67	0.23148	69.32	-0.30729	5087.605662383	0.26896	7.2211	-7.1668	0.55556	0.63562	0.91351 (max o	4	7728; 7621; 6495; 70	-0.3434417	-0.1401920		4.0
	ZINC20462940	-7.1	381.559	-2.3343	6	1	327.63	0.19855	68.12	1.9952	5087.605662383	0.26896	7.6278	-8.6789	0.55556	0.6516	0.91351 (max o	4	7727; 7620; 6804; 70	-0.2978427	-0.1663659		4.0
	ZINC35457076	-7.1	375.392	-2.1888	9	2	279.57	0.38116	134.58	-21.096	5087.605662383	0.27931	7.4823	-7.8365	0.57692	0.51135	0.94326 (max o	4	7649; 7435; 7262; 66	-0.7505276	-0.0259788		4.0
	ZINC35464275	-7.1	357.334	-5.9476	9	1	269.19	0.43872	166.92	-6.2936	5087.605662383	0.29048	11.241	-20.475	0.64	0.51518	0.90535 (max o	3	7802; 6646; 6647	0.82566375	0.1593933		4.0
	ZINC14694898	-7.1	362.333	1.3714	8	5	253.8	0.38826	136.68	-0.38093	5087.605662383	0.27931	3.9221	4.91	0.42308	0.37172	0.9615 (max of	3	7359; 6782; 6783	0.7797348	0.4491509		4.0

Structure	Molecule Name	affinity	Total Molweight	cLogP	H-Acceptors	H-Donors	Total Surface Area	Relative PSA	Polar Surface Area	Druglikeness	Kd (nM) @ 20°C	LE from K...	LLE from Kd ...	LELP from Kd (n...	Shape Index	Molecular...	Neighbor Si...	Neig...	Neighbor	Neighbor ...	Neighbor ...	Ring Systems	frequency
	ZINC03984368	-7.1	417.308	2.4972	6	2	292.32	0.19205	78.96	-11.758	5087.605662383	0.26896	2.7963	9.2845	0.55556	0.42641	0.96343 (max o	3	7465; 7300; 7299	0.7518886	0.21432261		4.0
	ZINC15707433	-7.1	386.539	-2.5883	8	5	317.99	0.40099	128.02	3.5155	5087.605662383	0.27931	7.8818	-9.2668	0.65385	0.60046	0.93361 (max o	5	7365; 7364; 7021; 70	-0.2511734	0.01594955		4.0
	ZINC15708655	-7.1	400.566	-2.2748	8	4	331.85	0.3604	119.23	4.9067	5087.605662383	0.26896	7.5683	-8.4576	0.62963	0.63836	0.93033 (max o	7	7499; 7365; 7364; 67	-0.2392376	0.05111807		4.0
	ZINC35442565	-7.1	437.587	-1.8032	9	2	340.24	0.33662	120.34	2.3474	5087.605662383	0.24207	7.0967	-7.4492	0.56667	0.48768	0.92257 (max o	10	7405; 1887; 3489; 43	-0.0636809	0.5267447		3.0
	ZINC20465860	-7.1	386.470	-1.1528	7	4	311.83	0.28625	96.12	1.9306	5087.605662383	0.25936	6.4463	-4.4448	0.57143	0.54307	0.92464 (max o	4	7411; 3405; 3406; 43	0.3925601	-0.2876261		3.0
	ZINC08740367	-7.1	352.350	-1.7011	11	4	243.01	0.48517	152.01	0.0425	5087.605662383	0.29048	6.9946	-5.8561	0.44	0.37685	0.93348 (max o	4	3752; 6191; 6758; 69	-0.866987	0.07589397		3.0
	ZINC12483972	-7.1	351.421	-1.7082	7	2	254.47	0.25304	77	2.0741	5087.605662383	0.29048	7.0017	-5.8806	0.6	0.47648	0.92721 (max o	6	7347; 7065; 7064; 42	0.4674367	-0.2013749		3.0
	ZINC03872806	-7.1	422.520	-1.4272	8	8	321.68	0.29246	154.6	-7.0465	5087.605662383	0.24207	6.7207	-5.8959	0.73333	0.49316	0.97533 (max o	2	5343; 6705	0.8354142	-0.2462373		3.0
	ZINC08551994	-7.1	425.996	-1.1969	7	5	318.59	0.34176	128.76	0.91283	5087.605662383	0.26896	6.4904	-4.45	0.51852	0.46665	0.96343 (max o	3	7163; 5817; 6178	0.73660624	-0.2257397		3.0
	ZINC20465595	-7.1	381.451	-3.816	9	4	300.15	0.34253	108.59	3.6337	5087.605662383	0.26896	9.1095	-14.188	0.59259	0.52874	0.96064 (max o	9	7730; 2232; 3403; 47	-0.3568991	-0.6147581		3.0
	ZINC20113182	-7.1	395.366	-3.338	8	0	291.83	0.31261	119.03	-6.2595	5087.605662383	0.25041	8.6315	-13.33	0.44828	0.42916	0.91292 (max o	3	5904; 5946; 6247	0.06307194	0.85890734		3.0
	ZINC22922460	-7.1	377.492	-2.6594	8	3	302.67	0.37149	119.9	0.85443	5087.605662383	0.27931	7.9529	-9.5214	0.61538	0.48164	0.9066 (max of	5	7405; 4402; 4404; 59	-0.0325048	0.46104285		3.0
	ZINC68603930	-7.1	371.344	1.0126	8	0	266.16	0.35963	119.76	-10.58	5087.605662383	0.26896	4.2809	3.7648	0.33333	0.3902	0.94605 (max o	2	7661; 6364	-0.9214991	-0.0018111		3.0
	ZINC08298385	-7.1	377.515	-3.4177	8	3	309.91	0.38663	109.82	0.86856	5087.605662383	0.27931	8.7112	-12.236	0.61538	0.47487	0.96721 (max o	4	7162; 4593; 6173; 64	0.03201548	-0.1299447		3.0
	ZINC18042631	-7.1	362.357	-1.5054	8	1	270.67	0.33269	116.2	3.3251	5087.605662383	0.27931	6.7989	-5.3897	0.5	0.49393	0.88178 (max o	3	7199; 7198; 7022	0.9050098	0.11356435		3.0
	ZINC18042631	-7.1	361.349	-3.5814	8	0	271.85	0.33559	119.03	3.0964	5087.605662383	0.27931	8.8749	-12.822	0.5	0.49393	0.91351 (max o	3	7205; 7198; 7023	0.89546067	0.08731252		3.0
	ZINC05433727	-7.1	353.466	1.1279	6	0	251.16	0.34886	114.47	7.434	5087.605662383	0.31574	4.1656	3.5722	0.6087	0.51068	0.88478 (max o	2	3695; 6910	0.49802658	0.4902036		2.0
	ZINC03839096	-7.1	431.491	0.1636	10	2	329.1	0.30097	112.98	3.5187	5087.605662383	0.23426	5.1299	0.69837	0.45161	0.48243	0.93414 (max o	3	4035; 5327; 6935	0.02190508	-0.0182057		2.0
	ZINC13543753	-7.1	393.446	-1.0702	11	5	284.48	0.40962	188.88	0.28599	5087.605662383	0.25936	6.3637	-4.1263	0.64286	0.52864	0.86499	1	5503	0.90721846	-0.3749289		2.0
	ZINC06403195	-7.1	375.373	-2.4613	10	5	269.21	0.44226	161.86	-7.4413	5087.605662383	0.27931	7.7548	-8.8121	0.46154	0.4176	0.95559 (max o	3	7097; 5811; 6894	-0.8006994	-0.1641367		2.0
	ZINC68603327	-7.1	448.423	0.6883	10	1	317.9	0.37213	142.5	-6.1982	5087.605662383	0.22694	4.6052	3.033	0.34375	0.47785	0.95949	1	6051	-0.5051496	-0.6778195		2.0
	ZINC08740366	-7.1	352.350	-1.7011	11	4	243.01	0.48517	152.01	0.0425	5087.605662383	0.29048	6.9946	-5.8561	0.44	0.37483	0.92662 (max o	4	6990; 3752; 6191; 67	-0.8530969	0.091154		2.0
	ZINC20465743	-7.1	353.437	-2.1485	7	4	289.33	0.30515	92.46	2.1642	5087.605662383	0.29048	7.442	-7.3963	0.6	0.59633	0.91255 (max o	4	7636; 5568; 6268; 68	0.28532693	-0.1474663		2.0
	ZINC03844451	-7.1	388.371	-2.3522	8	0	306.14	0.30914	117.18	-12.899	5087.605662383	0.25936	7.6457	-9.0693	0.71429	0.6076	0.97817 (max o	2	7756; 6402	-0.9360519	-0.0174531		2.0
	ZINC43845871	-7.1	350.350	-3.2625	9	3	240.31	0.45204	123.6	5.7262	5087.605662383	0.29048	8.556	-11.231	0.4	0.29651	0.81741	1	6657	0.8619077	-0.4418941		2.0
	ZINC38191132	-7.1	375.373	-2.4613	10	5	269.21	0.44226	161.86	-7.4413	5087.605662383	0.27931	7.7548	-8.8121	0.46154	0.4176	0.95559 (max o	3	5811; 6894; 6979	-0.8347025	-0.1627864		2.0
	ZINC05397923	-7.1	367.425	0.2859	8	1	271.82	0.37186	124.26	3.23	5087.605662383	0.29048	5.0076	0.98423	0.48	0.54842	0.87244	1	3610	0.02568093	0.3934397		2.0
	ZINC12490002	-7.1	363.436	-1.8047	8	4	290.72	0.33579	97.15	-1.2647	5087.605662383	0.27931	7.0982	-6.4613	0.61538	0.55503	0.91304 (max o	5	7910; 2524; 3763; 59	0.34529832	0.06535527		2.0
	ZINC13425091	-7.1	386.380	-0.7289	10	1	279.01	0.48446	152.56	1.149	5087.605662383	0.27931	6.0224	-2.6097	0.46154	0.44404	0.97269 (max o	5	7115; 7014; 7013; 62	0.36067104	-0.4902023		2.0
	ZINC14766422	-7.1	386.380	-0.7289	10	1	279.01	0.48446	152.56	1.149	5087.605662383	0.27931	6.0224	-2.6097	0.46154	0.44588	0.97269 (max o	6	7129; 7115; 6239; 69	0.37442562	-0.5027708		2.0
	ZINC35614465	-7.1	433.504	-3.1318	8	4	317.17	0.36268	161.4	-7.2057	5087.605662383	0.24207	8.4253	-12.938	0.43333	0.57251	0.95837 (max o	6	3511; 3513; 6345; 63	-0.647793	-0.1695704		2.0
	ZINC35614465	-7.1	434.511	-4.3565	8	4	313.45	0.35511	165.98	2.3821	5087.605662383	0.24207	9.65	-17.997	0.43333	0.59693	0.95837 (max o	3	3512; 6344; 7095	-0.4943950	-0.8338773		2.0
	ZINC00608191	-7.1	386.474	-0.4039	8	2	313.61	0.26055	83.11	3.0303	5087.605662383	0.25936	5.6974	-1.5573	0.46429	0.56138		0		0.22685692	-0.6773721		1.0
	ZINC03831070	-7.1	379.412	-0.6766	8	1	263.24	0.40229	133.3	10.411	5087.605662383	0.27931	5.9701	-2.4224	0.46154	0.35201	0.91351	1	6087	-0.5272857	-0.74467		1.0
	ZINC03838450	-7.1	434.491	-1.2497	10	3	337.08	0.3379	140.06	7.4231	5087.605662383	0.23426	6.5432	-5.3347	0.51613	0.55113	0.89174 (max o	2	7676; 7475	-0.7348617	0.52731735		1.0
	ZINC03839120	-7.1	390.442	-1.3715	10	3	301.1	0.34646	129.77	6.6619	5087.605662383	0.25936	6.665	-5.2881	0.46429	0.55658	0.88391 (max o	4	7816; 7581; 5327; 69	0.00259549	-0.0235567		1.0
	ZINC04085640	-7.1	354.465	-0.9097	6	1	262.82	0.26037	73.9	-11.215	5087.605662383	0.29048	6.2032	-3.1317	0.52	0.43218	0.98434 (max o	3	3641; 4088; 6715	0.07654998	0.54908246		1.0
	ZINC04962900	-7.1	362.333	0.3171	8	0	262.5	0.39779	103.96	-0.15641	5087.605662383	0.27931	4.9764	1.1353	0.5	0.35721		0		0.10499442	-0.9487738		1.0
	ZINC08636197	-7.1	362.516	-2.9726	6	3	318.77	0.253	60.1	2.2067	5087.605662383	0.27931	8.2661	-10.643	0.69231	0.60517		0		-0.6852205	-0.7276069		1.0
	ZINC12603867	-7.1	358.457	-2.9577	8	5	289.06	0.3556	112.33	-0.87654	5087.605662383	0.29048	8.2512	-10.182	0.64	0.55497	0.86353	1	2502	0.42689398	-0.4857435		1.0
	ZINC13349043	-7.1	416.405	-1.5687	9	3	306.73	0.35217	137.38	-12.421	5087.605662383	0.24207	6.8622	-6.4804	0.53333	0.43395	0.96343 (max o	3	968; 1503; 5870	0.8596791	0.15809262		1.0
	ZINC13377821	-7.1	356.466	0.3411	6	2	238.11	0.39629	131.68	6.4707	5087.605662383	0.31574	4.9524	1.0803	0.3913	0.33877	0.83219	1	7771	0.19570954	-0.9398818		1.0
	ZINC14766420	-7.1	386.380	-0.7289	10	1	279.01	0.48446	152.56	1.149	5087.605662383	0.27931	6.0224	-2.6097	0.46154	0.44535	0.97269 (max o	6	7129; 7014; 6239; 64	0.38079208	-0.5211142		1.0

Structure	Molecule Name	affinity	Total Molweight	cLogP	H-Acceptors	H-Donors	Total Surface Area	Relative PSA	Polar Surface Area	Druglikeness	Kd (nM) @ 20°C	LE from K...	LLE from Kd ...	LELP from Kd (n...	Shape Index	Molecular...	Neighbor Si...	Neig...	Neighbor	Neighbor ...	Neighbor ...	Ring Systems	frequency
	ZINC20463632	-7.1	445.602	-2.0577	7	3	352.21	0.23233	60.04	2.9523	5087.605662383	0.22694	7.3512	-9.0672	0.46875	0.48962	0.88009	1	6254	-0.6139676	-0.6488715		1.0
	ZINC20463632	-7.1	444.594	0.052	7	2	338.17	0.20046	58.84	5.1707	5087.605662383	0.22694	5.2415	0.22914	0.46875	0.48579	0.88322	1	6255	0.3800396	-0.0128652		1.0
	ZINC20464964	-7.1	373.496	-2.576	8	5	295.53	0.3846	126.74	5.3438	5087.605662383	0.29048	7.8695	-8.868	0.56	0.60857	0.89805 (max o	2	7792; 6261	-0.4735236	0.20356657		1.0
	ZINC20465325	-7.1	359.509	-5.2897	7	5	302.96	0.30644	79.83	0.46032	5087.605662383	0.29048	10.583	-18.21	0.56	0.49283		0		0.9615015	0.21065147		1.0
	ZINC20465918	-7.1	391.485	-2.3591	6	4	310.47	0.23352	83.63	-2.2151	5087.605662383	0.25936	7.6526	-9.0959	0.60714	0.5002	0.92464 (max o	3	7224; 3006; 3395	-0.3693766	-0.6307313		1.0
	ZINC20465918	-7.1	390.477	-0.9928	6	3	296.43	0.19721	82.43	2.4256	5087.605662383	0.25936	6.2863	-3.8279	0.60714	0.49467	0.96519 (max o	4	7225; 3007; 3396; 43	-0.5137877	0.21878505		1.0
	ZINC20465961	-7.1	365.516	-3.7451	6	5	312.44	0.26514	70.6	1.3058	5087.605662383	0.27931	9.0386	-13.408	0.57692	0.54781	0.88798	1	7226	0.26558128	0.9431802		1.0
	ZINC20503811	-7.1	413.629	-4.355	7	4	351.77	0.31171	86.73	2.1878	5087.605662383	0.25936	9.6485	-16.791	0.67857	0.68467	0.90299 (max o	3	7060; 5958; 5959	0.47432476	-0.5835069		1.0
	ZINC20503811	-7.1	412.621	-2.2453	7	3	337.73	0.2831	85.53	3.0067	5087.605662383	0.25936	7.5388	-8.6571	0.67857	0.68253	0.90299 (max o	3	7637; 5958; 7059	0.48970413	-0.5673709		1.0
	ZINC35614465	-7.1	434.511	-1.1599	8	4	316.97	0.35899	155.41	-7.2548	5087.605662383	0.24207	6.4534	-4.7916	0.43333	0.57119	0.95053 (max o	4	3511; 7096; 6343; 66	-0.6422908	-0.1250308		1.0
	ZINC35614465	-7.1	435.519	-1.7249	8	5	312.27	0.35268	163.15	1.7384	5087.605662383	0.24207	7.0184	-7.1257	0.43333	0.59693	0.87063 (max o	3	3512; 6344; 7094	-0.4852729	-0.8211017		1.0
	ZINC56898742	-7.1	375.440	-3.0151	9	5	261.23	0.3887	134.09	-2.3056	5087.605662383	0.27931	8.3086	-10.795	0.5	0.45587		0		-0.8198943	0.5600063		1.0
	ZINC04023923	-7.1	387.479	-1.4592	10	5	294.99	0.37933	171.2	-56.425	5087.605662383	0.26896	6.7527	-5.4253	0.55556	0.65953	0.96343 (max o	3	7467; 4954; 6113	-0.1627857	-0.4288038		0.0
	ZINC04556707	-7.1	441.595	0.4292	10	6	350.57	0.31982	168.01	1.4649	5087.605662383	0.23426	4.8643	1.8322	0.54839	0.67085	0.97423 (max o	5	7686; 7483; 7472; 71	-0.3463238	0.6814476		0.0
	ZINC35457514	-7.1	436.540	0.256	9	8	340.73	0.33692	171.07	-3.3718	5087.605662383	0.24207	5.0375	1.0576	0.66667	0.73675	0.99612 (max o	3	7263; 5638; 6006	-0.9184580	-0.3087701		0.0
	ZINC05317337	-7.1	358.413	-1.7779	9	3	283.96	0.40368	150.65	-57.945	5087.605662383	0.29048	7.0714	-6.1205	0.52	0.65451	0.97118 (max o	5	7763; 7681; 7325; 64	-0.2609685	0.80955094		0.0
	ZINC31160668	-7.1	356.413	-1.9376	7	3	287.28	0.327	140.95	-4.9219	5087.605662383	0.29048	7.2311	-6.6703	0.64	0.75283	0.99666 (max o	5	7957; 7799; 7415; 72	0.02636240	0.67170656		0.0
	ZINC04096970	-7.1	402.335	-11.312	13	3	300.83	0.57428	246.36	1.0342	5087.605662383	0.25936	16.606	-43.616	0.60714	0.6661	0.92152	1	7610	-0.9221158	-0.1105496		0.0
	ZINC02160015	-7.0	394.423	-1.4299	8	1	291.47	0.38546	139.29	3.977	6040.453154764	0.26518	6.6488	-5.3923	0.55556	0.51974	0.94927 (max o	10	3585; 4010; 4016; 44	0.02240709	0.13795334		172.0
	ZINC32116816	-7.0	435.547	-1.8592	10	6	331.55	0.35102	189.61	3.0367	6040.453154764	0.23096	7.0781	-8.0499	0.58065	0.5884	0.90529 (max o	2	5797; 6041	-0.1232688	-0.4134768		134.0
	ZINC08880038	-7.0	371.820	-2.3378	8	4	277.5	0.37441	146.2	4.0838	6040.453154764	0.28639	7.5567	-8.163	0.48	0.57348	0.93686 (max o	4	7448; 6024; 6196; 64	0.66108835	0.35026434		134.0
	ZINC31165089	-7.0	350.278	-4.1042	9	1	263.19	0.43246	153.09	-6.0092	6040.453154764	0.28639	9.3231	-14.331	0.6	0.54738	0.97396 (max o	7	7897; 7798; 7644; 52	0.8025509	0.08439587		134.0
	ZINC49181838	-7.0	368.361	-2.7364	9	4	275.87	0.41422	159.71	0.18103	6040.453154764	0.27537	7.9553	-9.937	0.53846	0.63333	0.98517 (max o	4	7655; 6353; 6664; 72	-0.3777285	0.71851945		134.0
	ZINC31160157	-7.0	368.361	-2.7364	9	4	275.87	0.41422	159.71	0.18103	6040.453154764	0.27537	7.9553	-9.937	0.53846	0.63643	0.98517 (max o	4	7655; 7270; 6353; 66	-0.3547109	0.7318644		134.0
	ZINC35464854	-7.0	382.411	-1.9852	9	4	290.9	0.39718	163.63	1.315	6040.453154764	0.26518	7.2041	-7.4864	0.62963	0.66081	0.98593 (max o	6	7800; 7562; 6297; 68	0.3649783	0.35031334		134.0
	ZINC35464851	-7.0	382.411	-1.9852	9	4	290.9	0.39718	163.63	1.315	6040.453154764	0.26518	7.2041	-7.4864	0.62963	0.66081	0.98593 (max o	6	7800; 7562; 7419; 62	0.3572945	0.37549987		134.0
	ZINC20112413	-7.0	354.274	-6.3073	8	1	265.63	0.41577	159.3	-12.739	6040.453154764	0.29832	11.526	-21.143	0.625	0.65773	0.99646 (max o	3	7373; 7374; 7203	0.571386	0.58880883		134.0
	ZINC20112410	-7.0	354.274	-6.3073	8	1	265.63	0.41577	159.3	-12.739	6040.453154764	0.29832	11.526	-21.143	0.625	0.65773	0.99646 (max o	3	7373; 7374; 7204	0.57203716	0.56743914		134.0
	ZINC03874238	-7.0	409.549	-0.809	7	3	324	0.33423	151.27	3.1065	6040.453154764	0.25571	6.0279	-3.1638	0.57143	0.59694	0.9678 (max of	3	7760; 7297; 6942	0.51259494	0.6658639		134.0
	ZINC02271037	-7.0	375.113	-3.4947	6	1	219.33	0.36083	109.36	2.8127	6040.453154764	0.37683	8.7136	-9.274	0.63158	0.57696		0		-0.8231833	-0.5639709		134.0
	ZINC00537957	-7.0	386.221	-4.1854	7	2	274.06	0.35295	113.8	-1.2741	6040.453154764	0.31129	9.4043	-13.445	0.52174	0.56886		0		-0.5227127	0.84644806		134.0
	ZINC04088672	-7.0	387.543	-1.4512	7	3	304.52	0.35561	151.27	2.4375	6040.453154764	0.27537	6.6701	-5.2699	0.65385	0.59445	0.96721 (max o	4	7313; 7311; 5758; 64	0.552799	0.06030722		127.0
	ZINC04089991	-7.0	387.543	-1.4512	7	3	304.52	0.35561	151.27	2.1528	6040.453154764	0.27537	6.6701	-5.2699	0.65385	0.61484	0.97533 (max o	12	7764; 7470; 7471; 73	0.5045753	0.08844554		127.0
	ZINC04085306	-7.0	401.570	-0.9968	7	3	318.28	0.34024	151.27	3.5206	6040.453154764	0.26518	6.2157	-3.759	0.66667	0.60655	0.96885 (max o	11	7764; 7608; 7313; 73	0.57412744	0.05758303		127.0
	ZINC35271423	-7.0	421.464	0.5332	8	2	326.7	0.30487	133.19	-4.2966	6040.453154764	0.23866	4.6857	2.2342	0.5	0.60182	0.96343 (max o	7	7555; 7433; 6304; 63	0.09538511	0.72957325		127.0
	ZINC31170284	-7.0	421.464	0.5332	8	2	326.7	0.30487	133.19	-4.2966	6040.453154764	0.23866	4.6857	2.2342	0.5	0.60057	0.96343 (max o	7	7555; 7433; 7259; 63	0.06240431	0.74612486		127.0
	ZINC04087696	-7.0	401.570	-0.9968	7	3	318.28	0.34024	151.27	2.382	6040.453154764	0.26518	6.2157	-3.759	0.66667	0.61028	0.95493 (max o	9	7764; 7608; 7312; 61	0.58452123	0.0750098		127.0
	ZINC31170258	-7.0	395.426	-0.9065	8	4	297.67	0.34521	147.35	-8.9294	6040.453154764	0.25571	6.1254	-3.5451	0.46429	0.60852	0.95983 (max o	5	7557; 7434; 6303; 68	0.4327485	-0.571529		127.0
	ZINC26973597	-7.0	415.052	-19.03	15	4	239.36	0.85587	304.55	-31.825	6040.453154764	0.29832	24.249	-63.79	0.41667	0.55758	0.97812 (max o	23	7929; 7884; 7883; 78	0.32849243	0.80708814		127.0
	ZINC26973597	-7.0	415.052	-19.03	15	4	239.36	0.85587	304.55	-31.825	6040.453154764	0.29832	24.249	-63.79	0.41667	0.55758	0.97812 (max o	24	7929; 7884; 7883; 78	0.36281228	0.7919385		127.0
	ZINC26973597	-7.0	414.044	-20.278	15	3	240.54	0.85657	307.38	-20.593	6040.453154764	0.29832	25.497	-67.972	0.41667	0.58345	0.95709 (max o	10	7928; 7882; 7833; 76	0.36289096	0.8735162		127.0
	ZINC26973597	-7.0	415.052	-19.03	15	4	239.36	0.85587	304.55	-31.825	6040.453154764	0.29832	24.249	-63.79	0.41667	0.54974	0.95709 (max o	23	7929; 7930; 7885; 78	0.3827174	0.79241925		127.0
	ZINC35271742	-7.0	395.426	-0.9065	8	4	297.67	0.34521	147.35	-8.9294	6040.453154764	0.25571	6.1254	-3.5451	0.46429	0.61198	0.95983 (max o	5	7741; 7556; 7557; 74	0.42840838	-0.550272		127.0

Structure	Molecule Name	affinity	Total Molweight	cLogP	H-Acceptors	H-Donors	Total Surface Area	Relative PSA	Polar Surface Area	Druglikeness	Kd (nM) @ 20°C	LE from K...	LLE from Kd ...	LELP from Kd (n...	Shape Index	Molecular...	Neighbor Si...	Neig...	Neighbor	Neighbor ...	Neighbor ...	Ring Systems	frequency
	ZINC05835336	-7.0	396.434	0.3826	8	6	325.5	0.30292	139.84	-21.493	6040.453154764	0.25571	4.8363	1.4963	0.53571	0.45564	0.96253 (max o	3	6166; 6462; 6463	0.79434556	0.3421704		120.0
	ZINC77257667	-7.0	363.381	-2.8728	9	5	272.09	0.41464	159.74	-12.771	6040.453154764	0.28639	8.0917	-10.031	0.52	0.57165	0.98897 (max o	3	6920; 7111; 7112	-0.834985	0.05419716		120.0
	ZINC31160519	-7.0	350.406	-0.6232	8	6	256.52	0.38438	139.84	-11.141	6040.453154764	0.29832	5.8421	-2.089	0.5	0.67073	0.9665 (max of	5	7545; 7414; 6858; 70	-0.6655052	-0.354172		120.0
	ZINC49601681	-7.0	365.354	-3.573	10	4	261.9	0.46873	165.81	-8.2227	6040.453154764	0.28639	8.7919	-12.476	0.56	0.6044	0.95 (max of 6)	6	7748; 7658; 7657; 74	-0.6214514	-0.3224909		120.0
	ZINC14773875	-7.0	357.270	-6.1116	11	4	257.11	0.54685	203.43	-8.5455	6040.453154764	0.28639	11.331	-21.34	0.48	0.58602	0.94678 (max o	2	7508; 7444	-0.8492522	0.26437956		120.0
	ZINC31162647	-7.0	365.397	-2.6206	9	5	273.11	0.41309	159.74	-12.662	6040.453154764	0.28639	7.8395	-9.1505	0.56	0.594	0.99111 (max o	5	7738; 7560; 7559; 74	-0.1188974	-0.5292537		120.0
	ZINC31162651	-7.0	365.397	-2.6206	9	5	273.11	0.41309	159.74	-12.662	6040.453154764	0.28639	7.8395	-9.1505	0.56	0.59348	0.99111 (max o	5	7854; 7742; 7738; 74	-0.1384283	-0.5371811		120.0
	ZINC04038466	-7.0	354.314	-0.795	10	0	269.88	0.43071	144.94	-8.7321	6040.453154764	0.28639	6.0139	-2.7759	0.44	0.42603	0.89524	1	7776	-0.5155641	0.81752974		120.0
	ZINC04026061	-7.0	394.200	1.6235	5	3	227.37	0.26081	79.15	-1.8727	6040.453154764	0.35799	3.5954	4.5351	0.6	0.42499	0.95318 (max o	2	7304; 7302	0.12482228	0.88425714		49.0
	ZINC04221722	-7.0	445.515	-4.1997	11	4	332.7	0.4563	207.26	8.384	6040.453154764	0.23866	9.4186	-17.597	0.46667	0.66717	0.87477 (max o	2	7676; 5773	-0.7307880	0.5486426		47.0
	ZINC04082648	-7.0	373.516	-1.3477	7	2	290.86	0.34511	142.48	5.6742	6040.453154764	0.28639	6.5666	-4.7058	0.64	0.58155	0.9654 (max of	5	7815; 7586; 6949; 70	0.7789552	-0.3775477		47.0
	ZINC02097025	-7.0	359.489	-1.8021	7	2	277.1	0.36225	142.48	4.8244	6040.453154764	0.29832	7.021	-6.0408	0.625	0.57246	0.94718 (max o	5	7815; 7586; 7134; 69	0.7965409	-0.3857268		47.0
	ZINC01081238	-7.0	370.094	-1.7405	8	4	187.56	0.48193	119.33	1.7864	6040.453154764	0.39776	6.9594	-4.3757	0.55556	0.50454	0.87379 (max o	2	7822; 5502	0.5264197	0.84163034		41.0
	ZINC04011869	-7.0	370.313	-1.0894	11	1	264.05	0.45525	137.54	0.69325	6040.453154764	0.27537	6.3083	-3.9561	0.46154	0.44351	0.93686 (max o	5	7129; 6239; 6413; 69	0.40247738	-0.523851		41.0
	ZINC04011871	-7.0	370.313	-1.0894	11	1	264.05	0.45525	137.54	0.69325	6040.453154764	0.27537	6.3083	-3.9561	0.46154	0.44535	0.97269 (max o	7	6239; 6413; 6923; 70	0.36380422	-0.5327614		41.0
	ZINC32123910	-7.0	361.377	-2.5215	9	3	279.08	0.38863	138.51	3.8076	6040.453154764	0.27537	7.7404	-9.1566	0.65385	0.57157	0.91693	1	4850	-0.3852892	0.774511		28.0
	ZINC19331350	-7.0	357.469	-2.4049	7	5	264.45	0.3274	105.67	3.1491	6040.453154764	0.28639	7.6238	-8.3973	0.44	0.61234	0.97118 (max o	4	7372; 3826; 6545; 67	-0.2997412	0.7196742		23.0
	ZINC19323206	-7.0	387.495	-2.9723	8	6	283.3	0.35185	125.9	3.5199	6040.453154764	0.26518	8.1912	-11.209	0.44444	0.63463	0.96617 (max o	6	7368; 7367; 5901; 62	-0.2667016	0.6952345		23.0
	ZINC19331406	-7.0	359.441	-3.4089	8	6	261.3	0.38148	125.9	2.5969	6040.453154764	0.28639	8.6278	-11.903	0.52	0.61996		0		0.9503258	0.22167541		23.0
	ZINC20464946	-7.0	395.522	-2.4579	8	4	322.95	0.26441	89.71	4.7193	6040.453154764	0.25571	7.6768	-9.6122	0.57143	0.62109	0.91204 (max o	7	7521; 7397; 4300; 59	-0.3262239	0.07312874		22.0
	ZINC08636398	-7.0	405.474	-0.271	8	4	300.99	0.37779	135.38	4.0787	6040.453154764	0.25571	5.4899	-1.0598	0.46429	0.42018	0.88128 (max o	2	7335; 2882	0.46369	0.03130647		18.0
	ZINC08636310	-7.0	383.443	-0.1499	7	4	275.03	0.37709	126.15	1.5013	6040.453154764	0.27537	5.3688	-0.54435	0.53846	0.414	0.97533 (max o	8	7333; 4180; 5825; 61	0.4476192	0.13180654		18.0
	ZINC08254650	-7.0	395.478	-0.3207	8	4	290.94	0.39084	135.38	2.8413	6040.453154764	0.26518	5.5396	-1.2094	0.55556	0.4319	0.93385 (max o	9	7775; 7333; 7164; 50	0.44870636	0.18199839		18.0
	ZINC20462944	-7.0	375.511	-2.2346	6	1	319.59	0.20354	68.12	6.5394	6040.453154764	0.26518	7.4535	-8.4269	0.55556	0.66291	0.9399 (max of	7	7379; 7207; 5128; 68	-0.0876333	-0.1005297		18.0
	ZINC20462944	-7.0	376.519	-1.8279	6	2	333.63	0.23706	69.32	4.1097	6040.453154764	0.26518	7.0468	-6.8932	0.55556	0.64723	0.9399 (max of	7	7381; 5130; 6802; 68	-0.0444779	-0.1159141		18.0
	ZINC20462944	-7.0	375.511	-2.2346	6	1	319.59	0.20354	68.12	5.6653	6040.453154764	0.26518	7.4535	-8.4269	0.55556	0.66034	0.9399 (max of	8	7380; 7208; 5129; 68	-0.0640395	-0.1252268		18.0
	ZINC00643247	-7.0	370.313	-1.0894	11	1	264.05	0.45525	137.54	0.69325	6040.453154764	0.27537	6.3083	-3.9561	0.46154	0.44404	0.97269 (max o	7	7129; 7128; 6239; 64	0.35413897	-0.5125338		17.0
	ZINC33851452	-7.0	397.375	-4.0196	12	7	270.85	0.52834	187.4	-6.2954	6040.453154764	0.26518	9.2385	-15.158	0.40741	0.4601	0.94622 (max o	8	7594; 7552; 7479; 73	0.52194214	-0.4971012		17.0
	ZINC33851454	-7.0	397.375	-4.0196	12	7	270.85	0.52834	187.4	-6.2954	6040.453154764	0.26518	9.2385	-15.158	0.40741	0.4601	0.94622 (max o	6	7552; 7321; 7004; 70	0.5045029	-0.5515444		17.0
	ZINC13348378	-7.0	383.349	-4.4475	12	8	254.94	0.57347	198.4	-4.7202	6040.453154764	0.27537	9.6664	-16.151	0.42308	0.46223	0.94951 (max o	9	7594; 7552; 7479; 74	0.46366104	-0.5418193		17.0
	ZINC31170369	-7.0	426.460	0.0489	9	4	309.91	0.35985	150.59	-12.343	6040.453154764	0.23866	5.17	0.20489	0.43333	0.53144	0.96092 (max o	4	6627; 6870; 7080; 72	0.41290674	0.24919198		16.0
	ZINC20463949	-7.0	380.531	-6.8196	7	3	319.35	0.25286	70.25	0.69593	6040.453154764	0.26518	12.039	-25.717	0.59259	0.55457	0.91007 (max o	5	7519; 7517; 2611; 59	0.62094885	0.03008817		16.0
	ZINC12483750	-7.0	393.381	-0.8672	7	3	271.07	0.26672	85.79	-6.5799	6040.453154764	0.26518	6.0861	-3.2703	0.62963	0.56417	0.90171 (max o	3	7496; 4213; 6503	0.45019287	-0.2712922		16.0
	ZINC20463949	-7.0	380.531	-3.3773	7	3	304.13	0.21547	66.22	3.756	6040.453154764	0.26518	8.5962	-12.736	0.59259	0.56493	0.91109 (max o	6	7518; 7520; 7346; 59	0.47108772	-0.2613971		16.0
	ZINC32123877	-7.0	363.369	0.2243	9	1	281.35	0.33993	106.53	-5.8362	6040.453154764	0.27537	4.9946	0.81453	0.65385	0.55886	0.92368 (max o	4	6275; 6278; 6592; 66	-0.0595412	-0.3669945		15.0
	ZINC13508072	-7.0	353.111	-1.3798	7	3	184.75	0.43069	108.38	2.1634	6040.453154764	0.42116	6.5987	-3.2762	0.58824	0.49161	0.90064 (max o	5	7887; 7840; 7812; 76	-0.4762327	-0.0132055		14.0
	ZINC12605318	-7.0	429.472	-4.4646	11	2	314.89	0.43504	173.48	5.4283	6040.453154764	0.24689	9.6835	-18.084	0.48276	0.62313	0.9892 (max of	3	3689; 3690; 3777	0.01795419	0.48168105		13.0
	ZINC05398137	-7.0	409.413	0.0213	9	1	305.99	0.29648	105.25	0.040477	6040.453154764	0.24689	5.1976	0.086274	0.48276	0.57627	0.93686 (max o	4	3687; 4992; 6437; 69	-0.0151813	0.35216564		13.0
	ZINC04270826	-7.0	356.398	0.4682	8	0	255	0.32063	101.6	4.1094	6040.453154764	0.29832	4.7507	1.5694	0.54167	0.60089	0.89347 (max o	2	5399; 6704	0.12280683	0.44725084		13.0
	ZINC31170357	-7.0	412.433	0.2102	9	4	295.62	0.37724	150.59	-8.6504	6040.453154764	0.24689	5.0087	0.8514	0.41379	0.52639	0.96682 (max o	6	7423; 7422; 4395; 72	0.39450675	0.26213622		12.0
	ZINC04531087	-7.0	352.386	-0.754	8	4	265.42	0.35868	117.12	-0.49157	6040.453154764	0.28639	5.9729	-2.6328	0.48	0.47801	0.94678 (max o	3	6152; 6153; 6966	-0.1338175	0.77426046		10.0
	ZINC09033930	-7.0	390.340	-0.2551	11	0	286.31	0.43729	140.73	-0.85962	6040.453154764	0.26518	5.474	-0.962	0.40741	0.39737	0.96885 (max o	3	7506; 6977; 7000	0.80778337	0.2842949		10.0
	ZINC12285470	-7.0	443.383	-5.0358	10	1	333	0.38468	176.15	-6.2561	6040.453154764	0.22374	10.255	-22.507	0.625	0.63166	0.97743 (max o	8	5860; 5861; 5862; 62	-0.6251122	0.6173648		9.0

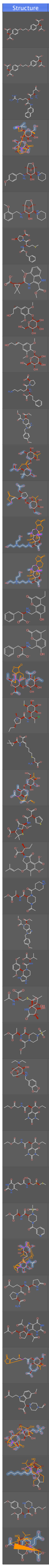

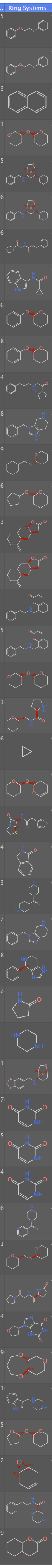

Structure	Molecule Name	affinity	Total Molweight	cLogP	H-Acceptors	H-Donors	Total Surface Area	Relative PSA	Polar Surface Area	Druglikeness	Kd (nM) @ 20°C	LE from K...	LLE from Kd ...	LELP from Kd (n...	Shape Index	Molecular...	Neighbor Si...	Neig...	Neighbor	Neighbor ...	Neighbor ...	Ring Systems	frequency
	ZINC12285470	-7.0	443.383	-5.0358	10	1	333	0.38468	176.15	-7.6209	6040.453154764	0.22374	10.255	-22.507	0.625	0.62894	0.97743 (max o	8	7170; 5860; 5861; 58	-0.6452544	0.60912085		9.0
	ZINC12285470	-7.0	442.375	-7.1118	10	0	334.18	0.38686	178.98	-6.6469	6040.453154764	0.22374	12.331	-31.786	0.625	0.63333	0.97743 (max o	8	7170; 7169; 5860; 58	-0.6175630	0.5842468		9.0
	ZINC04899556	-7.0	415.472	-1.8363	10	6	302.87	0.38426	189.61	2.7713	6040.453154764	0.23866	7.0552	-7.6943	0.5	0.53242	0.9218 (max of	3	1991; 3156; 5312	0.6798373	0.28581983		8.0
	ZINC71404531	-7.0	409.386	-3.6382	12	7	280.11	0.51858	202.32	-14.737	6040.453154764	0.25571	8.8571	-14.228	0.42857	0.47846	0.94862 (max o	4	2827; 3545; 3966; 60	0.5527999	-0.5188811		8.0
	ZINC12529597	-7.0	350.457	-2.6539	6	3	272.27	0.25133	68.97	-3.2537	6040.453154764	0.28639	7.8728	-9.2667	0.6	0.47907	0.90896 (max o	2	7177; 5542	0.5390036	-0.1238095		8.0
	ZINC12529614	-7.0	350.457	-2.6539	6	3	272.27	0.25133	68.97	-3.2537	6040.453154764	0.28639	7.8728	-9.2667	0.52	0.45954	0.90896 (max o	2	7043; 7176	0.5538676	-0.1633036		8.0
	ZINC04042890	-7.0	395.455	-1.4997	8	2	294.7	0.36987	144.3	-7.167	6040.453154764	0.26518	6.7186	-5.6555	0.51852	0.52877	0.96064 (max o	3	5359; 6417; 6418	-0.7651707	0.32486886		8.0
	ZINC70666366	-7.0	359.357	-0.8644	8	2	260.62	0.37929	120.55	0.018054	6040.453154764	0.27537	6.0833	-3.139	0.42308	0.30714	0.94008 (max o	4	4872; 6053; 7109; 71	0.44528311	0.3946573		8.0
	ZINC14709426	-7.0	372.369	-0.6515	9	5	272.55	0.38708	138.07	-4.1548	6040.453154764	0.27537	5.8704	-2.3659	0.53846	0.48289	0.9464 (max of	4	7443; 6653; 6784; 71	0.6151821	-0.2109006		8.0
	ZINC04095667	-7.0	372.369	-0.6515	9	5	272.55	0.38708	138.07	-4.1548	6040.453154764	0.27537	5.8704	-2.3659	0.53846	0.46546	0.9464 (max of	4	7443; 7190; 6928; 70	0.6132378	-0.1891008		8.0
	ZINC03873147	-7.0	405.493	-3.2679	8	2	315.18	0.2965	144.82	2.4951	6040.453154764	0.24689	8.4868	-13.236	0.48276	0.53439	0.95642 (max o	3	6103; 6706; 6941	-0.8452710	-0.4189204		8.0
	ZINC15676426	-7.0	374.416	-1.8768	8	1	276.63	0.24039	84.14	1.8385	6040.453154764	0.26518	7.0957	-7.0776	0.55556	0.50791	0.93385 (max o	7	7511; 6317; 6638; 67	-0.2638677	0.36640888		8.0
	ZINC35455122	-7.0	392.487	-0.0831	8	6	286.85	0.34373	139.84	-10.396	6040.453154764	0.26518	5.302	-0.31338	0.48148	0.55874	0.99202 (max o	7	5597; 6327; 6608; 66	-0.7242567	0.23107979		8.0
	ZINC31168982	-7.0	373.420	-1.7731	8	4	278.5	0.35806	139.51	-9.7968	6040.453154764	0.27537	6.992	-6.4389	0.5	0.51929	0.94951 (max o	4	7851; 5241; 6300; 68	0.45867157	0.61860186		8.0
	ZINC61996999	-7.0	395.562	-0.3464	6	3	305.79	0.19795	86.61	-4.4229	6040.453154764	0.25571	5.5653	-1.3547	0.57143	0.55438	0.94573 (max o	3	5668; 6901; 7102	-0.6824394	0.19863583		7.0
	ZINC61996999	-7.0	395.562	-0.488	6	4	323.55	0.24197	83.23	-3.5843	6040.453154764	0.25571	5.7069	-1.9084	0.57143	0.58716	0.9428 (max of	3	5669; 6902; 7103	-0.4023936	0.8738584		7.0
	ZINC02139622	-7.0	367.400	-0.2638	6	2	277.39	0.25668	103.27	-1.2836	6040.453154764	0.26518	5.4827	-0.99481	0.51852	0.52214	0.96567 (max o	7	7287; 4955; 5703; 60	-0.0613084	0.6800584		7.0
	ZINC01518796	-7.0	353.373	-0.7182	6	2	263.63	0.27008	103.27	-5.8017	6040.453154764	0.27537	5.9371	-2.6081	0.5	0.51754	0.94622 (max o	5	7287; 7119; 4955; 61	-0.0289892	0.67354685		7.0
	ZINC35051190	-7.0	396.300	-8.4533	13	4	261.98	0.59944	207.33	-4.475	6040.453154764	0.26518	13.672	-31.878	0.48148	0.45254	0.9578 (max of	3	7646; 6311; 6873	-0.6376856	0.7252727		7.0
	ZINC03830581	-7.0	425.996	-1.1969	7	5	318.59	0.34176	128.76	0.91283	6040.453154764	0.26518	6.4158	-4.5136	0.51852	0.47411	0.97658 (max o	7	7427; 7290; 7289; 71	0.6508879	-0.2054473		7.0
	ZINC04097341	-7.0	357.450	-2.7913	7	2	272.19	0.40821	162.3	-1.413	6040.453154764	0.29832	8.0102	-9.3566	0.66667	0.65036	0.94034 (max o	4	7916; 7716; 7589; 15	-0.1832006	0.46623346		6.0
	ZINC31157290	-7.0	403.359	-4.0657	11	4	283.96	0.46753	175.04	-7.5769	6040.453154764	0.25571	9.2846	-15.9	0.46429	0.50044	0.9678 (max of	4	7315; 4805; 5972; 59	-0.4975661	0.8560198		6.0
	ZINC04214391	-7.0	412.442	-3.6619	9	1	272.61	0.52669	192.44	9.2125	6040.453154764	0.26518	8.8808	-13.809	0.44444	0.43458	0.97903 (max o	2	7494; 4913	-0.3500448	-0.465588		6.0
	ZINC31167391	-7.0	417.456	-1.3541	8	0	322.92	0.28252	119.03	4.6442	6040.453154764	0.23866	6.573	-5.6738	0.43333	0.52584	0.97975 (max o	5	5206; 5609; 6616; 66	-0.0475372	0.87793404		6.0
	ZINC05398703	-7.0	369.440	-1.249	9	2	277.9	0.31832	112.63	1.6017	6040.453154764	0.27537	6.4679	-4.5356	0.5	0.55116	0.8996 (max of	2	4567; 6455	-0.0160576	0.33111903		6.0
	ZINC15676426	-7.0	373.408	0.101	8	0	287.6	0.26937	82.89	2.4228	6040.453154764	0.26518	5.1179	0.38088	0.55556	0.50791	0.93385 (max o	7	7196; 5896; 6318; 66	-0.2509096	0.33256057		6.0
	ZINC36369912	-7.0	402.449	-0.8529	9	3	295.73	0.30217	120.86	5.0527	6040.453154764	0.24689	6.0718	-3.4546	0.41379	0.48555	0.95372	1	7746	-4.0998845	-0.9753258		5.0
	ZINC13545968	-7.0	375.421	-4.4053	9	4	267.35	0.49766	181.16	-2.7923	6040.453154764	0.28639	9.6242	-15.382	0.44	0.65074	0.92315 (max o	4	7678; 5344; 5736; 61	-0.8683974	0.05303872		5.0
	ZINC05398325	-7.0	365.406	-1.9614	10	1	262.97	0.40408	130.7	2.7526	6040.453154764	0.29832	7.1803	-6.5748	0.5	0.61286	0.88969 (max o	5	7770; 7488; 4094; 41	-0.0727176	0.378717		5.0
	ZINC12483634	-7.0	351.421	-0.6832	7	2	252.97	0.25454	77	2.4527	6040.453154764	0.28639	5.9021	-2.3856	0.52	0.46356	0.9302 (max of	6	4212; 5136; 5178; 51	0.47471008	-0.1634311		5.0
	ZINC03844368	-7.0	369.373	-0.7686	10	1	282.1	0.36781	122.32	-1.4617	6040.453154764	0.27537	5.9875	-2.7911	0.53846	0.66057	0.97533 (max o	3	7583; 6937; 7123	0.56655246	-0.5928297		5.0
	ZINC03844367	-7.0	369.373	-0.7686	10	1	282.1	0.36781	122.32	-1.4617	6040.453154764	0.27537	5.9875	-2.7911	0.53846	0.66057	0.97533 (max o	3	7583; 7124; 6937	0.568123	-0.5565295		5.0
	ZINC04742519	-7.0	361.442	-1.8138	8	4	261.91	0.48417	169.11	1.1577	6040.453154764	0.31129	7.0327	-5.8267	0.65217	0.46525		0		-0.2719138	-0.9377874		5.0
	ZINC05397965	-7.0	398.439	-1.0197	10	1	285.47	0.37563	134.36	3.7301	6040.453154764	0.26518	6.2386	-3.8454	0.51852	0.57057	0.89657 (max o	3	6131; 6453; 6454	0.04416559	0.42422166		4.0
	ZINC13348397	-7.0	383.349	-4.4475	12	8	254.94	0.57347	198.4	-3.1137	6040.453154764	0.27537	9.6664	-16.151	0.46154	0.52717	0.94951 (max o	3	4250; 4251; 5082	-0.7602230	0.14907451		4.0
	ZINC04899524	-7.0	353.354	-4.454	10	3	258.34	0.46923	161.73	9.1102	6040.453154764	0.28639	9.6729	-15.552	0.48	0.53429	0.97118 (max o	8	5375; 5376; 5379; 53	0.0372442	0.763331		4.0
	ZINC04293776	-7.0	410.338	-0.3298	13	2	285.6	0.51106	164.15	4.6806	6040.453154764	0.24689	5.5487	-1.3358	0.41379	0.43163	0.93975 (max o	3	5787; 6151; 6441	-0.8873368	0.01626104		4.0
	ZINC35457066	-7.0	375.392	-2.1888	9	2	279.57	0.38116	134.58	-21.096	6040.453154764	0.27537	7.4077	-7.9484	0.57692	0.50497	0.9464 (max of	5	7649; 7435; 6005; 63	-0.7156303	-0.0232759		4.0
	ZINC36370111	-7.0	388.399	-5.3534	9	1	305.62	0.34582	119.17	2.4683	6040.453154764	0.25571	10.572	-20.936	0.5	0.49365	0.93385 (max o	4	5246; 6655; 6891; 68	0.31735224	-0.5689581		4.0
	ZINC05273794	-7.0	420.259	-10.349	14	7	261.47	0.67801	251.53	-34.627	6040.453154764	0.26518	15.568	-39.028	0.48148	0.56756	0.94905 (max o	3	6160; 6161; 6450	-0.5279919	-0.7792355		4.0
	ZINC35270752	-7.0	351.417	0.013601	6	3	277.6	0.30357	113.35	-14.742	6040.453154764	0.28639	5.2053	0.04749	0.6	0.43755	0.93007 (max o	3	6312; 6623; 7078	0.6978081	-0.5998802		4.0
	ZINC20504945	-7.0	393.438	-0.461	9	2	306.81	0.31156	106.2	4.6191	6040.453154764	0.25571	5.6799	-1.8029	0.57143	0.57362	0.93373 (max o	5	7230; 5945; 6439; 70	0.04255374	0.4421038		4.0
	ZINC12654044	-7.0	383.372	-5.5025	9	1	279.03	0.42325	166.92	-4.6692	6040.453154764	0.26518	10.721	-20.75	0.40741	0.58806	0.98371 (max o	3	7761; 7584; 6513	-0.0032157	-0.6914369		4.0

Structure	Molecule Name	affinity	Total Molweight	cLogP	H-Acceptors	H-Donors	Total Surface Area	Relative PSA	Polar Surface Area	Druglikeness	Kd (nM) @ 20°C	LE from K...	LLE from Kd ...	LELP from Kd (n...	Shape Index	Molecular...	Neighbor Si...	Neig...	Neighbor	Neighbor ...	Neighbor ...	Ring Systems	frequency
	ZINC72320584	-7.0	356.374	-3.0116	9	3	270.04	0.41638	152.63	1.1243	6040.453154764	0.28639	8.2305	-10.516	0.52	0.60811	0.96832 (max o	3	7453; 7452; 6919	0.94239575	-0.1664454		4.0
	ZINC35458436	-7.0	418.485	-2.7648	9	3	299.04	0.40362	164.89	-4.0506	6040.453154764	0.25571	7.9837	-10.812	0.53571	0.54102	0.99414 (max o	3	7855; 7801; 7438	-0.5470035	0.8148802		4.0
	ZINC30879438	-7.0	355.477	-1.4873	7	1	299.11	0.25091	77.35	2.0987	6040.453154764	0.28639	6.7062	-5.1933	0.52	0.6846	0.93007 (max o	2	7375; 6495	-0.3078556	-0.2078592		3.0
	ZINC15672924	-7.0	353.461	-0.1253	7	0	275.54	0.20589	62.32	4.5282	6040.453154764	0.28639	5.3442	-0.43751	0.64	0.57802	0.95631 (max o	3	5518; 5885; 6240	-0.719586	-0.3482811		3.0
	ZINC08297736	-7.0	363.489	-3.5426	8	3	281.05	0.39128	121.99	0.94395	6040.453154764	0.28639	8.7615	-12.37	0.64	0.45703	0.9654 (max of	4	5438; 6472; 6474; 69	0.01394672	-0.1218418		3.0
	ZINC20463684	-7.0	352.409	-2.2834	8	2	252.21	0.29773	91.99	6.3222	6040.453154764	0.28639	7.5023	-7.973	0.56	0.45022	0.92032 (max o	2	7626; 6501	0.4184486	0.06759265		3.0
	ZINC12483634	-7.0	351.421	-0.8248	7	3	270.73	0.30344	73.62	-0.01335	6040.453154764	0.28639	6.0437	-2.88	0.52	0.50789	0.87695 (max o	3	3772; 6502; 6808	0.55778456	-0.1855277		3.0
	ZINC20113203	-7.0	361.349	-3.5814	8	0	271.85	0.33559	119.03	3.0964	6040.453154764	0.27537	8.8003	-13.006	0.5	0.5177	0.91351 (max o	2	7718; 7022	0.8894045	0.06579579		3.0
	ZINC12602216	-7.0	397.630	-2.9054	6	4	350.79	0.28407	77.5	0.30309	6040.453154764	0.26518	8.1243	-10.957	0.7037	0.71896	0.91109 (max o	3	7778; 5957; 5958	0.44168928	-0.6140967		3.0
	ZINC20463635	-7.0	358.413	-3.1644	9	4	274.19	0.40005	110.56	-0.12605	6040.453154764	0.28639	8.3833	-11.049	0.6	0.52857	0.95287 (max o	2	7219; 7209	0.550298	-0.3687159		3.0
	ZINC20463626	-7.0	370.424	-2.8955	9	4	286.47	0.3829	110.56	-2.6438	6040.453154764	0.27537	8.1144	-10.515	0.61538	0.54458	0.95287 (max o	2	7219; 7210	0.5501144	-0.3475651		3.0
	ZINC20463929	-7.0	355.413	-3.7212	9	5	272.73	0.42152	127.35	0.80237	6040.453154764	0.28639	8.9401	-12.993	0.6	0.51602	0.92342 (max o	4	7387; 7209; 7210; 72	0.53370506	-0.3378209		3.0
	ZINC12659991	-7.0	354.465	-0.927	6	1	260.06	0.26313	73.9	-9.96	6040.453154764	0.28639	6.1459	-3.2368	0.52	0.43249	0.92662 (max o	2	3380; 4507	-0.6130194	0.508864		2.0
	ZINC12652500	-7.0	369.373	-1.6083	10	7	265.82	0.46219	160.38	-0.55001	6040.453154764	0.27537	6.8272	-5.8404	0.5	0.41725	0.94951 (max o	3	7351; 6111; 7127	0.52586347	-0.5548905		2.0
	ZINC08551996	-7.0	425.996	-1.1969	7	5	318.59	0.34176	128.76	0.91283	6040.453154764	0.26518	6.4158	-4.5136	0.51852	0.46665	0.91768 (max o	5	7290; 5817; 6178; 69	0.70126843	-0.2144258		2.0
	ZINC20463852	-7.0	361.484	-2.837	6	3	273.07	0.22697	62.98	2.855	6040.453154764	0.27537	8.0559	-10.302	0.57692	0.45507	0.90776 (max o	6	3839; 4730; 6254; 65	-0.5776726	-0.6124081		2.0
	ZINC20463852	-7.0	360.476	-0.7273	6	2	259.03	0.18508	61.78	5.109	6040.453154764	0.27537	5.9462	-2.6411	0.57692	0.45011	0.90776 (max o	8	4646; 4731; 5476; 62	0.39332262	0.00166094		2.0
	ZINC04096828	-7.0	372.488	-1.5166	8	4	280.72	0.42658	163.3	-12.912	6040.453154764	0.28639	6.7355	-5.2956	0.72	0.50898	0.94351 (max o	3	7316; 6429; 7140	-0.6776931	-0.6591312		2.0
	ZINC08254772	-7.0	377.464	0.3015	7	4	267.68	0.38744	126.15	4.5407	6040.453154764	0.27537	4.9174	1.0949	0.5	0.37227	0.91059 (max o	6	7775; 5022; 6754; 69	0.43219614	0.16491818		2.0
	ZINC18206569	-7.0	362.357	-1.6095	8	0	271.65	0.33127	113.04	-1.4111	6040.453154764	0.27537	6.8284	-5.8448	0.5	0.4779	0.82609	1	7023	0.89825964	0.13991944		2.0
	ZINC20504813	-7.0	380.507	0.4048	6	4	269.37	0.31681	135	-3.4432	6040.453154764	0.27537	4.8141	1.47	0.46154	0.45883	0.93686	1	6833	-0.9485394	-0.0333062		2.0
	ZINC36370111	-7.0	387.391	-5.485	9	0	291.58	0.31432	117.97	5.1997	6040.453154764	0.25571	10.704	-21.45	0.5	0.48061	0.9543 (max of	2	6656; 6892	0.78093725	-0.5281961		2.0
	ZINC72319929	-7.0	364.349	0.4791	8	2	252.33	0.37776	119.36	-4.1449	6040.453154764	0.27537	4.7398	1.7398	0.42308	0.26686	0.94326 (max o	3	7277; 6911; 6912	-0.5526852	0.6597276		2.0
	ZINC72319931	-7.0	364.349	0.4791	8	2	252.33	0.37776	119.36	-4.1449	6040.453154764	0.27537	4.7398	1.7398	0.42308	0.26686	0.94326 (max o	3	6911; 6912; 7276	-0.5730223	0.6511178		2.0
	ZINC03998979	-7.0	369.373	-1.6083	10	7	265.82	0.46219	160.38	-0.55001	6040.453154764	0.27537	6.8272	-5.8404	0.5	0.41245	0.94951 (max o	3	7351; 7180; 6111	0.5111105	-0.5714647		2.0
	ZINC12496087	-7.0	367.460	0.7901	6	3	302.68	0.26318	117.89	-18.726	6040.453154764	0.27537	4.4288	2.8692	0.73077	0.63962	0.93033 (max o	2	7350; 7348	0.09236158	-0.7921192		2.0
	ZINC12660577	-7.0	382.385	-0.5685	10	0	258.55	0.44347	132.04	-0.75239	6040.453154764	0.28639	5.7874	-1.9851	0.44	0.42146	0.96832	1	7337	-0.7865739	0.2238544		2.0
	ZINC15657775	-7.0	390.387	0.6557	8	4	282.69	0.33761	125.68	-1.4102	6040.453154764	0.25571	4.5632	2.5643	0.46429	0.31218	0.9543 (max of	4	7420; 2046; 5207; 67	-0.7489376	-0.1097515		2.0
	ZINC18206569	-7.0	361.349	-3.5814	8	0	271.85	0.33559	119.03	3.0964	6040.453154764	0.27537	8.8003	-13.006	0.5	0.48042	0.91351 (max o	3	7718; 7022; 7023	0.9142712	0.09235269		2.0
	ZINC04026446	-7.0	357.314	-0.7564	10	0	257.17	0.43815	130.38	-6.135	6040.453154764	0.28639	5.9753	-2.6412	0.44	0.36168		0		-0.7784818	-0.6254260		1.0
	ZINC04096826	-7.0	372.488	-1.5166	8	4	280.72	0.42658	163.3	-12.912	6040.453154764	0.28639	6.7355	-5.2956	0.72	0.50898	0.94351 (max o	3	7316; 6429; 7141	-0.6840964	-0.6757866		1.0
	ZINC12481236	-7.0	350.394	-2.6067	8	0	270.94	0.30464	98.94	5.1873	6040.453154764	0.28639	7.8256	-9.1019	0.52	0.57674	0.87695	1	5847	0.8882421	-0.184672		1.0
	ZINC13547499	-7.0	426.424	-2.8694	9	2	332.04	0.34225	147.69	-10.52	6040.453154764	0.23096	8.0883	-12.424	0.51613	0.52813	0.96971 (max o	5	2962; 4893; 4984; 67	-0.0514503	-0.4397400		1.0
	ZINC14711904	-7.0	415.397	-4.0082	9	2	308.07	0.3742	155.89	-0.74118	6040.453154764	0.23866	9.2271	-16.795	0.5	0.42048	0.8956	1	5349	-0.4072104	-0.7839461		1.0
	ZINC18153625	-7.0	360.385	-0.6307	7	0	276.91	0.28887	103.81	-8.7411	6040.453154764	0.27537	5.8496	-2.2903	0.57692	0.53727	0.88538	1	6793	0.6643852	0.66535974		1.0
	ZINC20463893	-7.0	366.460	-5.0794	8	4	276.48	0.33858	107.76	4.8183	6040.453154764	0.27537	10.298	-18.445	0.53846	0.45165	0.89162	1	7386	0.5967726	-0.1030768		1.0
	ZINC20463893	-7.0	365.452	-3.8547	8	4	280.2	0.34736	103.18	5.3872	6040.453154764	0.27537	9.0736	-13.998	0.53846	0.47437	0.89162 (max o	2	7387; 7219	0.56517285	-0.3337228		1.0
	ZINC20463899	-7.0	363.436	-7.0116	8	3	274.96	0.35965	118.55	2.0725	6040.453154764	0.27537	12.231	-25.462	0.57692	0.44589	0.96998 (max o	4	7388; 7218; 2222; 33	0.59801865	-0.0449633		1.0
	ZINC20463902	-7.0	362.428	-1.6552	8	3	263.46	0.3317	109.94	6.4079	6040.453154764	0.27537	6.8741	-6.0107	0.57692	0.48409	0.96998 (max o	5	3770; 5539; 5541; 62	-0.7896328	0.38097176		1.0
	ZINC20463902	-7.0	363.436	-7.0656	8	3	274.96	0.35965	118.55	2.0725	6040.453154764	0.27537	12.285	-25.658	0.57692	0.46057	0.96998 (max o	4	7388; 2222; 3390; 72	0.61769843	-0.0505967		1.0
	ZINC20465851	-7.0	378.491	-1.5945	7	4	306.11	0.29159	96.12	0.60121	6040.453154764	0.26518	6.8134	-6.013	0.55556	0.52129	0.96343 (max o	2	5568; 6832	0.2744001	-0.1289543		1.0
	ZINC20465909	-7.0	374.483	-3.4068	7	4	302.88	0.27559	96.52	-0.87513	6040.453154764	0.26518	8.6257	-12.847	0.59259	0.4883	0.92094 (max o	3	4317; 4747; 7056	-0.3474596	-0.6347628		1.0
	ZINC20465909	-7.0	373.475	-2.0405	7	3	288.84	0.24038	95.32	3.7656	6040.453154764	0.26518	7.2594	-7.6949	0.59259	0.48236	0.96343 (max o	3	4318; 4748; 7057	-0.5094034	0.1990527		1.0

Structure	Molecule Name	affinity	Total Molweight	cLogP	H-Acceptors	H-Donors	Total Surface Area	Relative PSA	Polar Surface Area	Druglikeness	Kd (nM) @ 20°C	LE from K...	LLE from Kd ...	LELP from Kd (n...	Shape Index	Molecular...	Neighbor Si...	Neig...	Neighbor	Neighbor ...	Neighbor ...	Ring Systems	frequency
	ZINC20465964	-7.0	351.465	-1.7448	6	4	285.84	0.26326	75.39	-2.1304	6040.453154764	0.28639	6.9637	-6.0924	0.56	0.5488	0.88798	1	7058	0.24296717	0.9427924		1.0
	ZINC20465964	-7.0	351.465	-1.6032	6	3	268.08	0.21445	78.77	-0.44862	6040.453154764	0.28639	6.8221	-5.598	0.56	0.48052		0		-0.7102204	0.6761708		1.0
	ZINC20504949	-7.0	434.491	0.0023003	10	2	332.63	0.29805	109.44	4.24	6040.453154764	0.23096	5.2166	0.0099597	0.54839	0.58011	0.88804 (max o	3	5945; 7069; 7229	0.06420133	0.44231328		1.0
	ZINC30879005	-7.0	381.491	-0.732	7	3	321.76	0.26476	81.46	2.2043	6040.453154764	0.26518	5.9509	-2.7604	0.59259	0.6095	0.89582 (max o	4	7932; 7641; 7640; 76	-0.3177909	0.4216376		1.0
	ZINC04556664	-7.0	427.568	-0.0252	10	6	336.81	0.33289	168.01	-3.5735	6040.453154764	0.23866	5.2441	-0.10559	0.53333	0.67149	0.98786 (max o	6	7686; 7483; 7472; 15	-0.351012	0.660975		0.0
	ZINC35457510	-7.0	436.540	0.256	9	8	340.73	0.33692	171.07	-3.3718	6040.453154764	0.23866	4.9629	1.0727	0.66667	0.73675	0.99612 (max o	3	5638; 6006; 7088	-0.9091271	-0.2933539		0.0
	ZINC31160660	-7.0	356.413	-1.9376	7	3	287.28	0.327	140.95	-4.9219	6040.453154764	0.28639	7.1565	-6.7656	0.64	0.74789	0.99666 (max o	5	7933; 7550; 7415; 68	0.00732134	0.6612322		0.0
	ZINC31157899	-7.0	436.456	-2.3414	12	6	333.18	0.4433	198.7	-1.3926	6040.453154764	0.23866	7.5603	-9.8107	0.6	0.66787	0.97757 (max o	3	7643; 7642; 7544	0.60862327	0.74456567		0.0
	ZINC31167440	-7.0	356.413	-2.0268	7	3	297.45	0.31582	140.95	-11.414	6040.453154764	0.28639	7.2457	-7.0771	0.8	0.69996	0.97118 (max o	3	7739; 7650; 7561	-0.6175039	0.4872866		0.0
	ZINC04629874	-7.0	438.607	0.5269	6	2	375.23	0.26573	153.43	-16.001	6040.453154764	0.23866	4.692	2.2078	0.7	0.64667	0.95053 (max o	5	7715; 7714; 7712; 77	0.14012767	-0.7339715		0.0
	ZINC04534251	-7.0	438.520	-2.731	10	2	324.36	0.48902	207.52	-53.944	6040.453154764	0.25571	7.9499	-10.68	0.57143	0.77944	0.97288 (max o	2	7908; 7907	0.45053172	-0.6922322		0.0
	ZINC13507607	-7.0	393.850	-4.248	11	6	282.96	0.45738	206.68	3.3478	6040.453154764	0.27537	9.4669	-15.426	0.65385	0.67133		0		0.6204665	0.76371235		0.0
	ZINC09033381	-6.9	435.523	-0.4017	10	5	340.6	0.32854	171.2	-18.893	7171.757548877	0.22766	5.5461	-1.7645	0.74194	0.58073	0.90299	1	5357	-0.1124313	-0.4884106		134.0
	ZINC04899816	-6.9	351.402	-1.3564	8	3	280.75	0.35904	135.2	-14.455	7171.757548877	0.2823	6.5008	-4.8048	0.68	0.54672	0.95174 (max o	4	4114; 4893; 6080; 67	-0.0729337	-0.4926926		134.0
	ZINC34302724	-6.9	363.456	-1.1943	7	3	289.99	0.31311	125.97	3.2028	7171.757548877	0.27144	6.3387	-4.3999	0.53846	0.5788	0.94951 (max o	3	6309; 6443; 6872	-0.0222399	0.62707835		134.0
	ZINC05819598	-6.9	438.634	-4.7516	8	6	364.88	0.2523	139.29	-1.2082	7171.757548877	0.22766	9.896	-20.871	0.70968	0.62155	0.96018	1	6969	0.04271795	-0.9777785		134.0
	ZINC31167849	-6.9	352.362	-1.4927	8	3	270.67	0.37337	135.99	2.2083	7171.757548877	0.2823	6.6371	-5.2877	0.64	0.66395	0.98434 (max o	8	7800; 7562; 6297; 63	0.36505345	0.40755302		134.0
	ZINC49180803	-6.9	352.362	-1.4927	8	3	270.67	0.37337	135.99	2.2083	7171.757548877	0.2823	6.6371	-5.2877	0.56	0.62155	0.98434 (max o	5	7800; 6354; 6898; 70	0.37582716	0.42096764		134.0
	ZINC20112418	-6.9	354.274	-6.3073	8	1	265.63	0.41577	159.3	-12.739	7171.757548877	0.29406	11.452	-21.449	0.625	0.6623	0.99646 (max o	3	7203; 7204; 7373	0.5900014	0.59647304		134.0
	ZINC20112416	-6.9	354.274	-6.3073	8	1	265.63	0.41577	159.3	-12.739	7171.757548877	0.29406	11.452	-21.449	0.625	0.6623	0.99646 (max o	3	7203; 7204; 7374	0.5905352	0.5761377		134.0
	ZINC03874237	-6.9	409.549	-0.809	7	3	324	0.33423	151.27	3.1065	7171.757548877	0.25205	5.9534	-3.2096	0.57143	0.59533	0.9678 (max of	4	7760; 7489; 6942; 71	0.48010668	0.68657285		134.0
	ZINC13541272	-6.9	424.544	-4.8177	9	5	329.44	0.40608	183.33	4.965	7171.757548877	0.24336	9.9621	-19.797	0.51724	0.60448	0.80862	1	7699	-0.4621904	0.8626302		134.0
	ZINC01836587	-6.9	371.365	-3.6539	9	1	276.16	0.41527	153.27	3.4151	7171.757548877	0.2823	8.7983	-12.943	0.56	0.57462		0		0.59391254	-0.7044905		134.0
	ZINC35457662	-6.9	367.416	-0.5417	7	3	280.83	0.31927	127.12	-7.3952	7171.757548877	0.27144	5.6861	-1.9956	0.46154	0.62833	0.97269 (max o	4	1608; 6316; 7089; 74	0.11038412	0.77714455		127.0
	ZINC35457659	-6.9	367.416	-0.5417	7	3	280.83	0.31927	127.12	-7.3952	7171.757548877	0.27144	5.6861	-1.9956	0.46154	0.62833	0.97269 (max o	4	7437; 1608; 6316; 70	0.1002481	0.79423016		127.0
	ZINC08479565	-6.9	383.531	-0.4733	7	3	311.79	0.29122	125.97	3.4553	7171.757548877	0.26139	5.6177	-1.8107	0.62963	0.60923	0.94302 (max o	8	7501; 5751; 5754; 58	0.54972667	-0.0058913		127.0
	ZINC04089444	-6.9	401.570	-0.9968	7	3	318.28	0.34024	151.27	2.382	7171.757548877	0.26139	6.1412	-3.8135	0.66667	0.61172	0.95493 (max o	8	7764; 7608; 5372; 53	0.5964663	0.08871274		127.0
	ZINC04090327	-6.9	387.543	-1.4512	7	3	304.52	0.35561	151.27	2.4375	7171.757548877	0.27144	6.5956	-5.3463	0.65385	0.59759	0.94927 (max o	8	7782; 7608; 7587; 32	0.56062365	0.04230268		127.0
	ZINC35271433	-6.9	421.464	0.5332	8	2	326.7	0.30487	133.19	-4.2966	7171.757548877	0.23525	4.6112	2.2665	0.5	0.60182	0.96343 (max o	7	7555; 6304; 6316; 66	0.07600263	0.76282674		127.0
	ZINC04087741	-6.9	387.543	-1.4512	7	3	304.52	0.35561	151.27	2.1528	7171.757548877	0.27144	6.5956	-5.3463	0.65385	0.61303	0.95257 (max o	9	7587; 7470; 7471; 73	0.5243215	0.08352645		127.0
	ZINC04089208	-6.9	401.570	-0.9968	7	3	318.28	0.34024	151.27	3.5206	7171.757548877	0.26139	6.1412	-3.8135	0.66667	0.60844	0.96885 (max o	8	7782; 7608; 7587; 73	0.58275527	0.03964920		127.0
	ZINC04090445	-6.9	402.490	-5.0218	9	3	306.54	0.44239	191.4	2.379	7171.757548877	0.26139	10.166	-19.212	0.66667	0.62068	0.96023 (max o	8	7764; 7587; 7471; 57	0.5572013	0.11115289		127.0
	ZINC35271744	-6.9	395.426	-0.9065	8	4	297.67	0.34521	147.35	-8.9294	7171.757548877	0.25205	6.0509	-3.5965	0.46429	0.61198	0.95983 (max o	6	7935; 7556; 7557; 63	0.4168933	-0.586503		127.0
	ZINC38212398	-6.9	357.270	-6.1116	11	4	257.11	0.54685	203.43	-8.5455	7171.757548877	0.2823	11.256	-21.649	0.48	0.58602	0.94351 (max o	2	7508; 7192	-0.8565376	0.29882488		120.0
	ZINC31159582	-6.9	350.406	-0.6232	8	6	256.52	0.38438	139.84	-9.7913	7171.757548877	0.29406	5.7676	-2.1193	0.5	0.56944	0.99271 (max o	5	7896; 7545; 7546; 70	-0.6450367	-0.347936		120.0
	ZINC31162641	-6.9	365.397	-2.6206	9	5	273.11	0.41309	159.74	-12.662	7171.757548877	0.2823	7.765	-9.2831	0.56	0.59504	0.99111 (max o	5	7738; 7559; 7560; 72	-0.1332405	-0.4960229		120.0
	ZINC49601684	-6.9	365.354	-3.573	10	4	261.9	0.46873	165.81	-8.2227	7171.757548877	0.2823	8.7174	-12.657	0.56	0.6044	0.95318 (max o	6	7748; 7658; 7657; 75	-0.5853021	-0.3349280		120.0
	ZINC34943434	-6.9	436.502	-3.8395	11	4	288.25	0.57929	238.9	-8.9899	7171.757548877	0.27144	8.9839	-14.145	0.5	0.59806	0.98956 (max o	5	7752; 7688; 7689; 75	-0.3914298	0.6138405		120.0
	ZINC13549482	-6.9	438.474	-3.8535	12	4	280.35	0.61652	245.14	-8.1983	7171.757548877	0.27144	8.9979	-14.196	0.46154	0.58491	0.9066 (max of	4	7688; 7689; 7504; 74	-0.3872671	0.5932611		120.0
	ZINC04026733	-6.9	396.172	0.794	6	3	223.61	0.30991	88.38	-1.631	7171.757548877	0.35287	4.3504	2.2501	0.6	0.4116	0.95318 (max o	2	7130; 7302	0.10536512	0.8953305		49.0
	ZINC04023530	-6.9	400.591	1.47	5	3	216.77	0.27356	79.15	-1.6883	7171.757548877	0.37145	3.6744	3.9575	0.57895	0.38466	0.91465 (max o	2	7304; 7130	0.12404369	0.9034259		49.0
	ZINC04042341	-6.9	384.136	0.9648	5	3	207.7	0.28551	79.15	-3.1395	7171.757548877	0.37145	4.1796	2.5974	0.52632	0.26113	0.91405 (max o	3	7682; 7458; 6708	-0.4633756	-0.8547967		49.0
	ZINC04012916	-6.9	362.139	-1.7236	10	5	206.36	0.57385	155.22	-0.33304	7171.757548877	0.33607	6.868	-5.1287	0.47619	0.39758	0.90776 (max o	3	7466; 5710; 5840	-0.2228905	-0.4540084		44.0

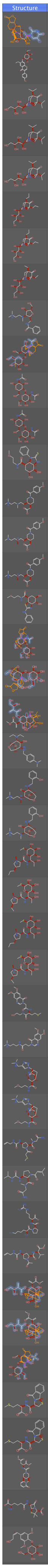

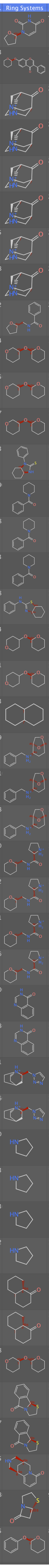

Structure	Molecule Name	affinity	Total Molweight	cLogP	H-Acceptors	H-Donors	Total Surface Area	Relative PSA	Polar Surface Area	Druglikeness	Kd (nM) @ 20°C	LE from K...	LLE from Kd ...	LELP from Kd (n...	Shape Index	Molecular...	Neighbor Si...	Neig...	Neighbor	Neighbor ...	Neighbor ...	Ring Systems	frequency
	ZINC17779640	−6.9	354.095	−0.8884	7	3	182.47	0.42358	99.1	3.3731	7171.757548877	0.41515	6.0328	−2.14	0.58824	0.50105	0.90064 (max o	4	7673; 7577; 7505; 57	0.41000497	0.3896838		41.0
	ZINC12495605	−6.9	446.407	−0.0864	10	5	308.75	0.38394	155.14	−3.4398	7171.757548877	0.22055	5.2308	−0.39176	0.53125	0.30874	0.95949 (max o	7	611; 659; 743; 955; 1	0.17380574	0.56674683		30.0
	ZINC19331341	−6.9	357.469	−2.4049	7	5	264.45	0.3274	105.67	3.1491	7171.757548877	0.2823	7.5493	−8.519	0.44	0.6126	0.97118 (max o	4	3826; 6545; 6798; 72	−0.2919640	0.7593265		23.0
	ZINC19323149	−6.9	357.469	−2.4049	7	5	264.45	0.3274	105.67	3.1491	7171.757548877	0.2823	7.5493	−8.519	0.44	0.62541	0.97118 (max o	6	5901; 6246; 6795; 70	−0.2499563	0.6832206		23.0
	ZINC19323147	−6.9	357.469	−2.4049	7	5	264.45	0.3274	105.67	3.1491	7171.757548877	0.2823	7.5493	−8.519	0.44	0.61122	0.97118 (max o	6	7368; 5901; 6246; 67	−0.2267917	0.7118046		23.0
	ZINC19323229	−6.9	387.495	−2.5001	8	6	288.82	0.34513	125.9	2.1725	7171.757548877	0.26139	7.6445	−9.5647	0.48148	0.63013	0.96064 (max o	5	7371; 6796; 6797; 70	−0.61787	0.2772386		23.0
	ZINC19323236	−6.9	387.495	−2.5001	8	6	288.82	0.34513	125.9	2.1725	7171.757548877	0.26139	7.6445	−9.5647	0.48148	0.64145	0.96064 (max o	4	7512; 6796; 6797; 73	−0.6006931	0.26496935		23.0
	ZINC19323239	−6.9	359.441	−3.4089	8	6	261.3	0.38148	125.9	3.8635	7171.757548877	0.2823	8.5533	−12.076	0.48	0.62355	0.95631 (max o	5	7616; 7512; 7024; 70	−0.6395215	0.24005158		23.0
	ZINC20464932	−6.9	352.453	−2.3543	7	4	288.13	0.28404	86.47	4.3999	7171.757548877	0.2823	7.4987	−8.3398	0.56	0.59811	0.9302 (max of	7	7521; 4300; 5929; 68	−0.3052043	0.04353442		22.0
	ZINC04533907	−6.9	383.349	−4.4475	12	8	254.94	0.57347	198.4	−4.7796	7171.757548877	0.27144	9.5919	−16.385	0.46154	0.46223	0.94951 (max o	10	7830; 7706; 7594; 74	0.49256933	−0.5134964		19.0
	ZINC13348005	−6.9	424.401	−4.4371	13	8	290.06	0.54334	207.27	−3.1385	7171.757548877	0.24336	9.5815	−18.233	0.41379	0.46239	0.95372 (max o	4	7707; 7706; 7594; 73	0.4627698	−0.4901275		19.0
	ZINC13348001	−6.9	424.401	−4.4371	13	8	290.06	0.54334	207.27	−3.1385	7171.757548877	0.24336	9.5815	−18.233	0.41379	0.46239	0.95372 (max o	5	7830; 7707; 7706; 74	0.46659625	−0.4681827		19.0
	ZINC08636308	−6.9	365.453	−0.2507	7	4	268.68	0.386	126.15	2.8413	7171.757548877	0.2823	5.3951	−0.88807	0.52	0.40853	0.93686 (max o	5	6181; 6743; 6983; 71	0.45175493	0.16127573		18.0
	ZINC20462948	−6.9	405.537	−2.3046	7	1	341.85	0.21954	77.35	6.5467	7171.757548877	0.24336	7.449	−9.4699	0.58621	0.67016	0.934 (max of 7	7	5128; 7380; 5910; 68	−0.0908137	−0.0793319		18.0
	ZINC20462948	−6.9	405.537	−2.3046	7	1	341.85	0.21954	77.35	5.683	7171.757548877	0.24336	7.449	−9.4699	0.58621	0.66783	0.934 (max of 9	9	7381; 5129; 5472; 66	−0.0668117	−0.1070038		18.0
	ZINC20462948	−6.9	406.545	−1.8979	7	2	355.89	0.25033	78.55	4.1526	7171.757548877	0.24336	7.0423	−7.7987	0.58621	0.65599	0.934 (max of 7	7	5130; 6602; 6802; 70	−0.0257740	−0.1289273		18.0
	ZINC08636309	−6.9	365.453	0.0486	7	4	266.13	0.38011	128.48	3.6911	7171.757548877	0.2823	5.0958	0.17216	0.6	0.41555	0.94678 (max o	7	3716; 3735; 4229; 46	−0.4869615	0.40445018		17.0
	ZINC13348371	−6.9	383.349	−4.4475	12	8	254.94	0.57347	198.4	−4.7202	7171.757548877	0.27144	9.5919	−16.385	0.42308	0.46223	0.95257 (max o	9	7594; 7479; 7480; 67	0.4978748	−0.5323404		17.0
	ZINC13508664	−6.9	356.323	−4.03	11	7	235.73	0.55869	178.53	−7.2031	7171.757548877	0.29406	9.1744	−13.705	0.45833	0.45604		0		−0.8357706	0.24931711		17.0
	ZINC67913287	−6.9	444.475	−0.5622	10	6	314.74	0.39614	173.98	−11.767	7171.757548877	0.22766	5.7066	−2.4695	0.41935	0.54324	0.96739 (max o	5	6046; 6674; 7083; 70	0.37756795	0.29459453		16.0
	ZINC12483644	−6.9	350.481	−0.8982	6	2	267.53	0.17919	61.78	3.2903	7171.757548877	0.2823	6.0426	−3.1817	0.56	0.53062	0.92032 (max o	6	7496; 5533; 6280; 62	0.4443866	−0.2521239		16.0
	ZINC22922214	−6.9	353.461	−7.0617	7	4	290.88	0.31044	87.24	−0.77955	7171.757548877	0.2823	12.206	−25.015	0.56	0.52271	0.94338 (max o	2	7627; 6560	0.60983676	0.12023771		16.0
	ZINC22922214	−6.9	353.461	−3.6194	7	4	275.66	0.27236	83.21	3.4155	7171.757548877	0.2823	8.7638	−12.821	0.56	0.53505	0.94338 (max o	5	7628; 7518; 7520; 62	0.52654564	−0.2117288		16.0
	ZINC03982483	−6.9	407.550	−2.4	8	6	309.52	0.39409	148.99	0.28877	7171.757548877	0.26139	7.5444	−9.1818	0.51852	0.4647	0.97658 (max o	9	7679; 7464; 7460; 24	0.6947519	−0.2935200		15.0
	ZINC33902779	−6.9	393.523	−2.8544	8	6	295.76	0.41243	148.99	2.3085	7171.757548877	0.27144	7.9988	−10.516	0.5	0.46343	0.97533 (max o	8	7553; 7460; 6897; 69	0.66691154	−0.2617662		15.0
	ZINC03830998	−6.9	407.550	−2.4	8	6	309.52	0.39409	148.99	0.28877	7171.757548877	0.26139	7.5444	−9.1818	0.51852	0.4657	0.97658 (max o	8	7460; 7428; 7427; 74	0.6637747	−0.2232821		15.0
	ZINC33902780	−6.9	393.523	−2.8544	8	6	295.76	0.41243	148.99	2.3085	7171.757548877	0.27144	7.9988	−10.516	0.5	0.46343	0.95257 (max o	7	7553; 7460; 6930; 71	0.65537316	−0.2455886		15.0
	ZINC32124417	−6.9	438.503	−0.7162	10	3	332.91	0.38356	154.86	6.8664	7171.757548877	0.23525	5.8606	−3.0444	0.56667	0.57819	0.9218 (max of	5	6275; 6276; 6277; 68	−0.0209563	−0.3695170		15.0
	ZINC32124286	−6.9	363.436	−1.0001	8	2	303.96	0.29672	84.67	3.9208	7171.757548877	0.27144	6.1445	−3.6844	0.65385	0.55738	0.89253 (max o	2	6654; 7113	−0.1083056	−0.3320758		15.0
	ZINC03838646	−6.9	352.481	−4.8442	8	3	305.23	0.32267	77.92	−0.94891	7171.757548877	0.2823	9.9886	−17.16	0.68	0.57217	0.9654 (max of	4	4593; 5008; 6398; 72	0.08018156	−0.1519511		14.0
	ZINC03838647	−6.9	352.481	−4.8442	8	3	305.23	0.32267	77.92	−0.94891	7171.757548877	0.2823	9.9886	−17.16	0.68	0.57217	0.9654 (max of	3	4594; 6175; 7292	0.08658371	−0.1753806		14.0
	ZINC03838883	−6.9	371.461	−1.5754	9	3	283.39	0.40559	150.14	6.2602	7171.757548877	0.2823	6.7198	−5.5806	0.56	0.67074	0.91611 (max o	5	7816; 7461; 5723; 57	−0.0325987	−0.0446680		13.0
	ZINC03839105	−6.9	367.448	−0.3712	9	3	287.83	0.33857	124.84	6.0847	7171.757548877	0.27144	5.5156	−1.3675	0.46154	0.61612	0.92032 (max o	4	7755; 7581; 7317; 57	−0.0323280	−0.0039753		13.0
	ZINC13541698	−6.9	401.510	−3.3002	11	6	296.64	0.41448	208.46	4.3065	7171.757548877	0.25205	8.4446	−13.093	0.64286	0.6033	0.81947	1	7484	−0.1020022	−0.9125613		13.0
	ZINC03838909	−6.9	368.432	−0.1334	9	3	290.54	0.3445	116.84	2.4852	7171.757548877	0.27144	5.2778	−0.49145	0.57692	0.58901		0		0.7964574	−0.4728172		13.0
	ZINC31170351	−6.9	412.433	0.2102	9	4	295.62	0.37724	150.59	−8.6504	7171.757548877	0.24336	4.9342	0.86374	0.41379	0.52639	0.96931 (max o	7	7423; 5983; 6626; 66	0.4283793	0.2344056		12.0
	ZINC31170354	−6.9	412.433	0.2102	9	4	295.62	0.37724	150.59	−8.6504	7171.757548877	0.24336	4.9342	0.86374	0.41379	0.52639	0.96931 (max o	7	5984; 6626; 6627; 68	0.4321947	0.2559624		12.0
	ZINC04556805	−6.9	420.259	−10.197	14	7	262.3	0.67587	251.53	−31.289	7171.757548877	0.26139	15.341	−39.011	0.48148	0.52622	0.95201 (max o	4	7943; 7696; 5791; 60	0.02109613	−0.5112238		12.0
	ZINC04085102	−6.9	365.453	−1.4662	6	1	254.82	0.40574	140.14	6.1016	7171.757548877	0.29406	6.6106	−4.986	0.54167	0.41355	0.95709 (max o	3	6713; 6950; 7308	−0.6972066	−0.657524		10.0
	ZINC04085045	−6.9	365.453	−1.4662	6	1	254.82	0.40574	140.14	6.1016	7171.757548877	0.29406	6.6106	−4.986	0.54167	0.41641	0.95709 (max o	3	7309; 6713; 6950	−0.7127195	−0.6408707		10.0
	ZINC06640061	−6.9	393.463	0.3428	8	1	293.05	0.36062	120.27	−1.2311	7171.757548877	0.26139	4.8016	1.3115	0.51852	0.60924	0.97407	1	6470	0.5005304	0.74066395		10.0
	ZINC04995221	−6.9	358.394	−4.462	9	2	245.75	0.51963	182.61	4.8789	7171.757548877	0.29406	9.6064	−15.174	0.58333	0.44402	0.90399 (max o	3	5415; 6381; 6719	−0.3126573	−0.8395558		9.0
	ZINC36046229	−6.9	372.369	−0.6515	9	5	272.55	0.38708	138.07	−4.1548	7171.757548877	0.27144	5.7959	−2.4002	0.53846	0.48289	0.94326 (max o	4	6653; 6784; 7139; 71	0.5950872	−0.2030165		8.0

Structure	Molecule Name	affinity	Total Molweight	cLogP	H-Acceptors	H-Donors	Total Surface Area	Relative PSA	Polar Surface Area	Druglikeness	Kd (nM) @ 20°C	LE from K...	LLE from Kd ...	LELP from Kd (n...	Shape Index	Molecular...	Neighbor Si...	Neig...	Neighbor	Neighbor ...	Neighbor ...	Ring Systems	frequency
	ZINC20462930	-6.9	419.564	-2.3065	7	1	355.61	0.21105	77.35	6.6944	7171.757548877	0.23525	7.4509	-9.8045	0.6	0.68871	0.934 (max of	5	7377; 7378; 4778; 68	-0.0851256	-0.1226079		8.0
	ZINC20462930	-6.9	419.564	-2.3065	7	1	355.61	0.21105	77.35	5.8391	7171.757548877	0.23525	7.4509	-9.8045	0.6	0.68666	0.934 (max of	6	7380; 5913; 6852; 69	-0.0771402	-0.1622143		8.0
	ZINC20462930	-6.9	420.572	-1.8998	7	2	369.65	0.24101	78.55	4.3264	7171.757548877	0.23525	7.0442	-8.0757	0.6	0.67621	0.934 (max of	6	7381; 6853; 6992; 70	-0.0560722	-0.1553302		8.0
	ZINC14719122	-6.9	356.370	0.1552	8	1	277.9	0.33185	108.36	-3.5573	7171.757548877	0.2823	4.9892	0.54977	0.52	0.58617	0.96243 (max o	7	5992; 5993; 6635; 66	0.00999924	-0.7430573		8.0
	ZINC35464700	-6.9	356.413	-2.5089	7	2	285.37	0.34513	133.25	-4.0636	7171.757548877	0.2823	7.6533	-8.8874	0.64	0.61937	0.98434 (max o	3	7652; 7651; 7091	-0.6231274	0.6918087		8.0
	ZINC30878645	-6.9	378.535	-0.8713	6	2	321.66	0.18911	57.45	5.931	7171.757548877	0.26139	6.0157	-3.3334	0.62963	0.66346	0.89424 (max o	4	7410; 2253; 4356; 74	-0.3747083	0.4052151		7.0
	ZINC30878645	-6.9	378.535	-0.8713	6	2	321.66	0.18911	57.45	5.1804	7171.757548877	0.26139	6.0157	-3.3334	0.62963	0.66114	0.89424 (max o	4	7639; 4355; 7408; 74	-0.3559886	0.43715885		7.0
	ZINC30878645	-6.9	379.543	-2.2376	6	3	335.7	0.22303	58.65	4.5021	7171.757548877	0.26139	7.382	-8.5605	0.62963	0.64932	0.89424 (max o	2	7410; 7409	-0.3781519	0.44122797		7.0
	ZINC02121311	-6.9	381.427	0.1906	6	2	291.15	0.24455	103.27	-3.3893	7171.757548877	0.25205	4.9538	0.75619	0.53571	0.52118	0.96567 (max o	6	4955; 5703; 6074; 61	-0.0590215	0.70219946		7.0
	ZINC04200941	-6.9	391.430	-2.1382	11	3	295.88	0.40351	150.62	9.4789	7171.757548877	0.25205	7.2826	-8.4832	0.46429	0.51969	0.92774 (max o	5	7755; 5523; 5765; 73	-0.0513099	0.00712453		7.0
	ZINC04200942	-6.9	389.415	-2.2286	11	3	297.16	0.40177	150.62	6.1099	7171.757548877	0.25205	7.373	-8.8418	0.5	0.54014	0.92774 (max o	6	7816; 7581; 7319; 57	-0.0122745	0.00604598		7.0
	ZINC04201101	-6.9	432.479	-0.6374	11	3	335.56	0.36366	142.62	2.0973	7171.757548877	0.22766	5.7818	-2.7998	0.45161	0.50469	0.89329 (max o	2	6962; 7318	0.00742349	-0.0027015		7.0
	ZINC31169032	-6.9	354.350	-2.5673	10	6	244.4	0.48527	158.3	-6.1681	7171.757548877	0.29406	7.7117	-8.7305	0.5	0.46608	0.90719 (max o	3	3903; 4382; 5613	-0.4288644	0.40894508		7.0
	ZINC03830995	-6.9	407.550	-2.4	8	6	309.52	0.39409	148.99	0.28877	7171.757548877	0.26139	7.5444	-9.1818	0.51852	0.4657	0.97658 (max o	8	7553; 7460; 7427; 72	0.6445576	-0.2657610		7.0
	ZINC03830996	-6.9	407.550	-2.4	8	6	309.52	0.39409	148.99	0.28877	7171.757548877	0.26139	7.5444	-9.1818	0.51852	0.4657	0.97658 (max o	8	7553; 7460; 7427; 72	0.6464471	-0.2279557		7.0
	ZINC33902778	-6.9	393.523	-2.8544	8	6	295.76	0.41243	148.99	2.3085	7171.757548877	0.27144	7.9988	-10.516	0.5	0.46343	0.96389 (max o	8	7553; 7428; 6929; 71	0.63526905	-0.2455604		7.0
	ZINC03794794	-6.9	446.502	-2.9876	10	8	328.9	0.35537	172.34	-0.035	7171.757548877	0.22055	8.132	-13.546	0.5625	0.49359	0.87898	1	7870	0.736092	0.5839793		6.0
	ZINC01529258	-6.9	357.450	-2.7913	7	2	272.19	0.40821	162.3	-1.413	7171.757548877	0.29406	7.9357	-9.4922	0.66667	0.61517	0.96029 (max o	4	7916; 7716; 7589; 15	-0.1706739	0.48980033		6.0
	ZINC04095728	-6.9	388.368	-1.8524	10	4	278.22	0.42585	151.98	-10.162	7171.757548877	0.26139	6.9968	-7.0868	0.48148	0.48454	0.94589 (max o	2	5991; 7237	-0.5109921	0.8428329		6.0
	ZINC20464003	-6.9	386.470	-1.0516	7	3	285.32	0.23994	91.66	4.3573	7171.757548877	0.25205	6.196	-4.1721	0.57143	0.491	0.95123 (max o	8	7850; 7848; 7630; 25	0.64566845	0.5985499		6.0
	ZINC05320607	-6.9	410.425	1.9926	8	2	316.4	0.28129	95.12	2.9999	7171.757548877	0.23525	3.1518	8.4702	0.6	0.36732	0.83798 (max o	2	4141; 4564	0.21203582	-0.7804628		5.0
	ZINC32124268	-6.9	417.441	-0.9642	10	3	304.95	0.32176	124.1	-0.11909	7171.757548877	0.23525	6.1086	-4.0986	0.43333	0.51536	0.95318 (max o	6	5614; 5985; 5987; 59	-0.6194656	0.17664227		5.0
	ZINC30879646	-6.9	371.455	-0.636	6	4	299.31	0.26157	83.23	1.9458	7171.757548877	0.26139	5.7804	-2.4332	0.55556	0.53485	0.92152 (max o	7	3405; 5159; 5184; 55	0.40521908	-0.3016243		5.0
	ZINC05398450	-6.9	372.420	-1.0287	10	2	288.36	0.35435	117.28	2.3537	7171.757548877	0.27144	6.1731	-3.7898	0.5	0.63531	0.90033 (max o	5	2202; 3220; 5655; 58	-0.0932358	0.32759818		5.0
	ZINC08636497	-6.9	437.515	-0.0627	9	3	313.8	0.36902	135.82	4.2492	7171.757548877	0.23525	5.2071	-0.26653	0.43333	0.43047	0.89412 (max o	4	5826; 5827; 6184; 71	0.44043395	0.03221674		5.0
	ZINC04025089	-6.9	354.266	-5.9772	10	0	239.68	0.52904	167	-5.683	7171.757548877	0.2823	11.122	-21.173	0.44	0.5901	0.94678	1	7939	0.94930327	-0.1107501		5.0
	ZINC27642641	-6.9	382.407	-0.3469	8	2	264.11	0.37837	114.82	1.115	7171.757548877	0.26139	5.4913	-1.3271	0.37037	0.29503	0.98371 (max o	5	7797; 7731; 7548; 66	-0.0695562	0.87959176		4.0
	ZINC15708201	-6.9	369.440	-3.6504	9	3	291.17	0.35436	101.41	4.2979	7171.757548877	0.27144	8.7948	-13.448	0.61538	0.51413	0.96998 (max o	9	7499; 4221; 4267; 46	-0.2035438	0.07202407		4.0
	ZINC15707479	-6.9	353.441	-2.8284	8	3	281.17	0.3314	92.18	6.0102	7171.757548877	0.2823	7.9728	-10.019	0.6	0.46905	0.93033 (max o	5	7499; 7363; 5897; 65	-0.2192935	0.08375682		4.0
	ZINC20463991	-6.9	389.450	-4.2781	8	3	307.71	0.32989	104.25	2.0178	7171.757548877	0.25205	9.4225	-16.973	0.60714	0.53331	0.88589 (max o	2	5922; 7392	0.36115006	0.09961226		4.0
	ZINC20463991	-6.9	390.458	-2.2021	8	4	306.53	0.32731	101.42	2.3515	7171.757548877	0.25205	7.3465	-8.7367	0.60714	0.53331	0.89424 (max o	2	5926; 7391	0.33844918	0.08707152		4.0
	ZINC20463991	-6.9	390.458	-2.0605	8	3	288.77	0.28594	104.8	5.2847	7171.757548877	0.25205	7.2049	-8.1749	0.60714	0.48914	0.92418 (max o	2	5925; 7390	0.3435854	-0.7959329		4.0
	ZINC20463991	-6.9	389.450	-4.1365	8	2	289.95	0.28884	107.63	5.0311	7171.757548877	0.25205	9.2809	-16.411	0.60714	0.48914	0.92418 (max o	2	7393; 5923	0.33230224	-0.7800221		4.0
	ZINC35457070	-6.9	375.392	-2.1888	9	2	279.57	0.38116	134.58	-21.096	7171.757548877	0.27144	7.3332	-8.0636	0.57692	0.51135	0.9464 (max of	5	7649; 6005; 6333; 70	-0.7482059	-0.0464478		4.0
	ZINC01106025	-6.9	354.472	0.7411	5	2	298.7	0.18129	49.91	4.6605	7171.757548877	0.27144	4.4033	2.7302	0.46154	0.51984	0.97791 (max o	2	6066; 7280	-0.1517642	0.8615435		4.0
	ZINC01106024	-6.9	354.472	0.7411	5	2	298.7	0.18129	49.91	4.6605	7171.757548877	0.27144	4.4033	2.7302	0.46154	0.50713	0.97791 (max o	2	6922; 7281	-0.1289903	0.8644525		4.0
	ZINC04293461	-6.9	441.388	-0.0395	12	1	324.96	0.43319	156.67	2.0065	7171.757548877	0.22766	5.1839	-0.1735	0.41935	0.40801	0.95771 (max o	6	7781; 7703; 7593; 75	0.29448688	0.39000863		4.0
	ZINC35618515	-6.9	396.355	-2.075	12	1	279.99	0.46498	145.19	4.6603	7171.757548877	0.25205	7.2194	-8.2324	0.42857	0.41517	0.9428 (max of	4	7533; 7442; 6595; 66	-0.7838428	-0.0871484		4.0
	ZINC14694896	-6.9	362.333	1.3714	8	5	253.8	0.38826	136.68	-0.38093	7171.757548877	0.27144	3.773	5.0523	0.42308	0.37494	0.9615 (max of	3	6782; 6783; 7011	0.77240205	0.47035182		4.0
	ZINC72320583	-6.9	356.374	-3.0116	9	3	270.04	0.41638	152.63	1.1243	7171.757548877	0.2823	8.156	-10.668	0.52	0.59713	0.96832 (max o	3	6919; 7278; 7452	0.954709	-0.1485527		4.0
	ZINC72320582	-6.9	356.374	-3.0116	9	3	270.04	0.41638	152.63	1.1243	7171.757548877	0.2823	8.156	-10.668	0.52	0.59935	0.96832 (max o	3	7453; 6919; 7278	0.93161905	-0.1502090		4.0
	ZINC03984370	-6.9	417.308	2.4972	6	2	292.32	0.19205	78.96	-11.758	7171.757548877	0.26139	2.6472	9.5536	0.55556	0.42641	0.96343 (max o	3	7465; 6944; 7299	0.7342768	0.20561606		4.0
	ZINC03984364	-6.9	417.308	2.4972	6	2	292.32	0.19205	78.96	-11.758	7171.757548877	0.26139	2.6472	9.5536	0.55556	0.42417	0.96343 (max o	3	7465; 7300; 6944	0.7701212	0.20099892		4.0

Structure	Molecule Name	affinity	Total Molweight	cLogP	H-Acceptors	H-Donors	Total Surface Area	Relative PSA	Polar Surface Area	Druglikeness	Kd (nM) @ 20°C	LE from K...	LLE from Kd ...	LELP from Kd (n...	Shape Index	Molecular...	Neighbor Si...	Neig...	Neighbor	Neighbor ...	Neighbor ...	Ring Systems	frequency
	ZINC15708461	-6.9	382.507	-3.3267	8	5	318.54	0.4003	128.02	2.3218	7171.757548877	0.27144	8.4711	-12.256	0.69231	0.60263	0.93361 (max c	6	5116; 6244; 7018; 70	-0.2564643	0.06069895		4.0
	ZINC35458429	-6.9	418.485	-2.7648	9	3	299.04	0.40362	164.89	-4.0506	7171.757548877	0.25205	7.9092	-10.969	0.53571	0.54102	0.99414 (max c	3	7855; 7801; 7264	-0.5492449	0.8233609		4.0
	ZINC04062078	-6.9	378.376	1.2887	8	1	277.82	0.34288	116.2	-3.9534	7171.757548877	0.26139	3.8557	4.9302	0.44444	0.50718	0.98806 (max c	4	7684; 7683; 7585; 74	-0.5648769	0.27021		4.0
	ZINC12480704	-6.9	366.524	-0.1433	5	1	306.73	0.26688	93.12	5.0639	7171.757548877	0.2823	5.2877	-0.50762	0.52	0.6671	0.91567 (max c	3	7618; 7515; 7516	-0.2285326	-0.0670839		4.0
	ZINC20465779	-6.9	421.536	-0.5193	7	4	323.9	0.28969	108.68	-5.0737	7171.757548877	0.24336	5.6637	-2.1339	0.55172	0.54785	0.96682 (max c	6	1874; 1875; 4321; 47	0.4544805	-0.3691148		3.0
	ZINC12484055	-6.9	351.421	-1.7082	7	2	254.47	0.25304	77	2.0741	7171.757548877	0.2823	6.8526	-6.051	0.52	0.45687	0.91652 (max c	5	5136; 6253; 6995; 70	0.45547625	-0.1600913		3.0
	ZINC22922540	-6.9	354.454	-2.2124	8	2	275.9	0.40225	117.1	-7.1364	7171.757548877	0.29406	7.3568	-7.5236	0.58333	0.47707	0.90535 (max c	6	1887; 4342; 4402; 59	-0.0175555	0.49549025		3.0
	ZINC62001269	-6.9	414.889	-4.2781	8	4	300.48	0.39394	155.89	6.5869	7171.757548877	0.26139	9.4225	-16.367	0.48148	0.52738	0.93367 (max c	7	3093; 5262; 6196; 63	0.6279209	0.34152082		3.0
	ZINC15195939	-6.9	368.381	0.2665	8	2	256.64	0.35957	111.52	3.4604	7171.757548877	0.27144	4.8779	0.9818	0.38462	0.28721	0.97269 (max c	3	1892; 6363; 6669	0.78853935	0.18750419		3.0
	ZINC22922139	-6.9	354.425	-2.8834	8	3	254.57	0.2952	95.15	7.5412	7171.757548877	0.2823	8.0278	-10.214	0.6	0.45038	0.93033 (max c	3	4731; 6501; 7401	0.37955552	0.08956554		3.0
	ZINC20464003	-6.9	387.478	-2.4179	7	4	299.36	0.27559	92.86	-0.27172	7171.757548877	0.25205	7.5623	-9.5929	0.57143	0.49636	0.95123 (max c	5	7849; 7847; 7629; 80	0.4225519	0.44729128		3.0
	ZINC70691758	-6.9	438.583	-1.8787	7	4	307.48	0.28259	92.82	-0.30899	7171.757548877	0.22766	7.0231	-8.2522	0.3871	0.34735	0.79631	1	3980	0.53718895	0.7180941		2.0
	ZINC01532361	-6.9	370.428	-0.3474	7	4	295.68	0.27733	119.19	-2.5311	7171.757548877	0.26139	5.4918	-1.3291	0.7037	0.56642	0.9578	1	4460	0.89032876	-0.2299104		2.0
	ZINC27644079	-6.9	353.302	-2.8445	9	5	246.47	0.47008	167.58	0.33063	7171.757548877	0.2823	7.9889	-10.076	0.56	0.43889	0.96243 (max c	3	1112; 2204; 5090	-0.2363427	-0.8305637		2.0
	ZINC38549934	-6.9	374.499	0.8471	5	3	312.02	0.20444	63.36	-6.941	7171.757548877	0.26139	4.2973	3.2408	0.51852	0.47399	0.97407 (max c	3	7563; 3520; 5648	0.02321658	-0.7742728		2.0
	ZINC20463991	-6.9	391.466	-3.4268	8	4	302.81	0.31904	106	1.1744	7171.757548877	0.25205	8.5712	-13.596	0.60714	0.49461	0.88044	1	5924	0.12432633	0.9739274		2.0
	ZINC08740521	-6.9	375.344	0.9999	12	5	266.84	0.53002	177.07	1.5916	7171.757548877	0.26139	4.1445	3.8254	0.48148	0.30941	0.96885 (max c	4	5028; 5834; 6193; 64	-0.3191357	0.28928635		2.0
	ZINC35618515	-6.9	395.347	-4.2446	12	0	281.28	0.46743	151.51	2.1322	7171.757548877	0.25205	9.389	-16.84	0.42857	0.40235	0.9428 (max of	2	6650; 7441	-0.7848414	-0.0436990		2.0
	ZINC30879733	-6.9	392.925	-1.3371	5	4	307.02	0.20041	70.74	-0.93633	7171.757548877	0.26139	6.4815	-5.1154	0.55556	0.48756	0.92739 (max c	2	2599; 6854	-0.3854482	-0.6797619		2.0
	ZINC12652494	-6.9	369.373	-1.6083	10	7	265.82	0.46219	160.38	-0.55001	7171.757548877	0.27144	6.7527	-5.925	0.5	0.41376	0.94951 (max c	3	6111; 7127; 7180	0.5395339	-0.5370952		2.0
	ZINC12495318	-6.9	367.460	0.695	6	3	299.92	0.2656	117.89	-11.4	7171.757548877	0.27144	4.4494	2.5604	0.69231	0.64185	0.93033 (max c	2	7350; 7175	0.10645383	-0.7783343		2.0
	ZINC08722207	-6.9	382.385	-0.5685	10	0	258.55	0.44347	132.04	-0.75239	7171.757548877	0.2823	5.7129	-2.0138	0.44	0.41717	0.96832	1	7183	-0.7660785	0.2260624		2.0
	ZINC31167887	-6.9	406.386	0.31	9	5	289.04	0.37552	145.91	-1.4102	7171.757548877	0.24336	4.8344	1.2738	0.48276	0.30757	0.93686 (max c	3	2308; 5207; 7193	-0.7664038	-0.1200416		2.0
	ZINC20463908	-6.9	352.409	-6.876	8	3	265.89	0.38373	118.8	2.3636	7171.757548877	0.2823	12.02	-24.357	0.56	0.43993	0.86809 (max c	3	7216; 7218; 7386	0.6107031	-0.0875116		2.0
	ZINC20463908	-6.9	353.417	-4.8	8	4	264.71	0.38098	115.97	2.6415	7171.757548877	0.2823	9.9444	-17.003	0.56	0.43993	0.89162 (max c	2	7388; 7214	0.61640334	-0.1090717		2.0
	ZINC35242176	-6.9	386.360	-3.1619	11	0	278.56	0.5312	171.24	3.2541	7171.757548877	0.27144	8.3063	-11.649	0.46154	0.44595	0.94951 (max c	4	7648; 7647; 7554; 66	0.46196944	-0.3267896		2.0
	ZINC01822455	-6.9	373.412	1.0331	9	1	285.36	0.28904	88.93	3.9669	7171.757548877	0.26139	4.1113	3.9524	0.51852	0.47757	0.82831 (max c	2	7575; 6071	-0.0042060	-0.8406751		1.0
	ZINC01822455	-6.9	374.420	-0.9447	9	2	274.39	0.26061	90.18	3.2085	7171.757548877	0.26139	6.0891	-3.6142	0.51852	0.51129	0.84842	1	5355	0.52363956	0.6462981		1.0
	ZINC04045930	-6.9	378.380	-0.297	9	1	278.09	0.33849	103.4	2.4045	7171.757548877	0.26139	5.4414	-1.1362	0.55556	0.45082	0.9144	1	6119	-0.1133433	0.98240715		1.0
	ZINC04096829	-6.9	372.488	-1.5166	8	4	280.72	0.42658	163.3	-12.912	7171.757548877	0.2823	6.661	-5.3723	0.72	0.50898	0.94351 (max c	3	6429; 7140; 7141	-0.6612531	-0.6752533		1.0
	ZINC05327851	-6.9	354.354	-1.5399	8	3	234.44	0.43947	125.82	0.30799	7171.757548877	0.2823	6.6843	-5.4549	0.4	0.26875	0.97932	1	7450	0.62601894	-0.6439743		1.0
	ZINC08636282	-6.9	426.536	-0.655	9	5	329.42	0.37997	147.41	0.62927	7171.757548877	0.24336	5.7994	-2.6915	0.68966	0.65271	0.89979 (max c	2	3739; 6476	0.3217564	-0.3834507		1.0
	ZINC08643695	-6.9	358.441	-6.2115	10	5	286.05	0.40147	130.55	-2.1887	7171.757548877	0.2823	11.356	-22.003	0.68	0.60587	0.87695 (max c	2	5450; 5478	0.20303369	-0.7062434		1.0
	ZINC09232763	-6.9	440.498	-3.0337	9	2	351.27	0.31173	119.86	2.6607	7171.757548877	0.22055	8.1781	-13.755	0.46875	0.52982	0.93157 (max c	3	7342; 7341; 7343	-0.8040816	0.23787718		1.0
	ZINC09232763	-6.9	441.506	-0.8426	9	3	350.09	0.30941	117.03	-0.94338	7171.757548877	0.22055	5.987	-3.8205	0.46875	0.41714	0.93157 (max c	3	7342; 7340; 7343	-0.8158501	0.254667		1.0
	ZINC09232766	-6.9	440.498	-3.0337	9	2	351.27	0.31173	119.86	2.6607	7171.757548877	0.22055	8.1781	-13.755	0.46875	0.52897	0.93157 (max c	3	7340; 7341; 7343	-0.797829	0.26404938		1.0
	ZINC09232766	-6.9	441.506	-0.8426	9	3	350.09	0.30941	117.03	-0.94338	7171.757548877	0.22055	5.987	-3.8205	0.46875	0.41612	0.93157 (max c	3	7340; 7341; 7342	-0.787668	0.24646488		1.0
	ZINC12481132	-6.9	353.481	-0.8219	6	0	283.68	0.20255	72.91	1.0466	7171.757548877	0.2823	5.9663	-2.9115	0.52	0.65455	0.89438 (max c	2	7524; 5912	-0.2130636	-0.1438480		1.0
	ZINC12496233	-6.9	369.476	0.6464	6	4	303.78	0.26243	121.05	-19.923	7171.757548877	0.27144	4.498	2.3814	0.73077	0.63962	0.90311 (max c	2	7175; 7348	0.07453838	-0.7879194		1.0
	ZINC15708337	-6.9	384.523	-2.8242	8	5	307.49	0.41468	128.02	3.0533	7171.757548877	0.27144	7.9686	-10.404	0.65385	0.57062	0.92032 (max c	8	5115; 6243; 6244; 73	-0.2214183	0.04283838		1.0
	ZINC20462916	-6.9	367.532	-0.1671	6	2	316.53	0.2305	76.91	-0.80464	7171.757548877	0.27144	5.3115	-0.6156	0.53846	0.65421	0.88798 (max c	3	4714; 6495; 7236	-0.3293773	-0.2161371		1.0
	ZINC20463645	-6.9	388.530	-0.5503	6	2	302.61	0.21549	59.26	4.9837	7171.757548877	0.25205	5.6947	-2.1833	0.5	0.53034	0.82174	1	7789	-0.8749658	-0.441652		1.0
	ZINC20463645	-6.9	388.530	-3.9926	6	2	317.83	0.25306	63.29	0.26907	7171.757548877	0.25205	9.137	-15.84	0.5	0.53542	0.81091	1	7790	0.9235399	-0.2421656		1.0

Structure	Molecule Name	affinity	Total Molweight	cLogP	H-Acceptors	H-Donors	Total Surface Area	Relative PSA	Polar Surface Area	Druglikeness	Kd (nM) @ 20°C	LE from K...	LLE from Kd ...	LELP from Kd (n...	Shape Index	Molecular...	Neighbor Si...	Neig...	Neighbor	Neighbor ...	Neighbor ...	Ring Systems	frequency
	ZINC20463667	-6.9	363.436	-2.473	8	2	261.28	0.27538	91.74	6.2154	7171.757548877	0.27144	7.6174	-9.1106	0.57692	0.46969	0.96998 (max o	7	5533; 5850; 5851; 62	0.39462635	-0.1991594		1.0
	ZINC20463702	-6.9	354.405	-1.2046	9	3	260.23	0.34277	103.37	3.6145	7171.757548877	0.2823	6.349	-4.2671	0.6	0.5266	0.87932 (max o	4	7625; 3324; 3838; 65	-0.5828082	-0.3122359		1.0
	ZINC20463908	-6.9	352.409	-3.5753	8	4	268.43	0.38956	111.39	3.7026	7171.757548877	0.2823	8.7197	-12.665	0.56	0.48134	0.89162 (max o	3	6222; 7215; 7219	0.54098946	-0.3186715		1.0
	ZINC20464923	-6.9	361.461	-3.8336	9	4	290.21	0.32869	98.94	5.1508	7171.757548877	0.2823	8.978	-13.58	0.56	0.66605	0.89805	1	5931	-0.3457590	0.07873724		1.0
	ZINC20465940	-6.9	377.483	-0.7694	6	4	295.25	0.33412	111.47	4.2472	7171.757548877	0.27144	5.9138	-2.8345	0.53846	0.52154	0.9615 (max of	4	4316; 5591; 6604; 68	0.22061744	-0.1295849		1.0
	ZINC20465942	-6.9	352.453	-1.5852	7	5	288.03	0.3116	95.26	3.264	7171.757548877	0.2823	6.7296	-5.6153	0.56	0.60156	0.93355 (max o	3	6268; 6605; 6831	0.3064797	-0.2421855		1.0
	ZINC22922139	-6.9	355.433	-4.9931	8	4	268.61	0.33204	96.35	3.604	7171.757548877	0.2823	10.137	-17.687	0.6	0.45038	0.86809	1	7402	0.39736676	0.12309577		1.0
	ZINC30879746	-6.9	358.457	-2.8745	8	4	281.91	0.32578	95.7	3.8125	7171.757548877	0.2823	8.0189	-10.182	0.56	0.58774		0		0.6824206	-0.7237957		1.0
	ZINC35459011	-6.9	388.368	-1.5759	10	4	277.62	0.42677	151.98	-7.1253	7171.757548877	0.26139	6.7203	-6.029	0.44444	0.45564	0.96617 (max o	3	4415; 4837; 6335	0.8153288	-0.3282799		1.0
	ZINC70647132	-6.9	354.354	-1.5399	8	3	234.44	0.43947	125.82	0.30799	7171.757548877	0.2823	6.6843	-5.4549	0.4	0.26875	0.97932	1	7327	0.64804256	-0.6360947		1.0
	ZINC05317725	-6.9	358.413	-1.7779	9	3	283.96	0.40368	150.65	-57.945	7171.757548877	0.2823	6.9223	-6.2979	0.52	0.65451	0.97118 (max o	5	7763; 7681; 6451; 67	-0.2203091	0.82101613		0.0
	ZINC31160664	-6.9	356.413	-1.9376	7	3	287.28	0.327	140.95	-4.9219	7171.757548877	0.2823	7.082	-6.8637	0.64	0.74789	0.99666 (max o	5	7933; 7550; 6859; 70	-0.0086532	0.6788198		0.0
	ZINC31160730	-6.9	354.441	-0.6502	6	1	298.93	0.26006	109.72	-4.9275	7171.757548877	0.2823	5.7946	-2.3032	0.64	0.70603	0.97932 (max o	7	7957; 7933; 7799; 77	0.02218839	0.7344286		0.0
	ZINC31160722	-6.9	354.441	-0.6502	6	1	298.93	0.26006	109.72	-4.9275	7171.757548877	0.2823	5.7946	-2.3032	0.64	0.70603	0.97932 (max o	7	7957; 7933; 7799; 77	0.04506982	0.7265407		0.0
	ZINC02110828	-6.8	418.381	-3.5223	11	4	310.75	0.44074	173.96	0.1125	8514.941681041	0.23184	8.5921	-15.193	0.66667	0.61099	0.96519 (max o	3	1647; 2362; 6390	0.1376081	0.08269130		172.0
	ZINC35464848	-6.8	382.411	-1.9852	9	4	290.9	0.39718	163.63	1.315	8514.941681041	0.2576	7.055	-7.7065	0.62963	0.65945	0.98593 (max o	6	7800; 6886; 7076; 72	0.37517765	0.3671048		134.0
	ZINC05759681	-6.8	409.549	-0.809	7	3	324	0.33423	151.27	3.1065	8514.941681041	0.2484	5.8788	-3.2569	0.57143	0.59565	0.9428 (max of	3	7760; 6942; 7297	0.5020989	0.6488063		134.0
	ZINC12874093	-6.8	383.531	-0.4733	7	3	311.79	0.29122	125.97	3.4553	8514.941681041	0.2576	5.5431	-1.8373	0.62963	0.60923	0.94927 (max o	8	7782; 3208; 6122; 61	0.561035	-0.0213170		127.0
	ZINC35271429	-6.8	421.464	0.5332	8	2	326.7	0.30487	133.19	-4.2966	8514.941681041	0.23184	4.5366	2.2999	0.5	0.60182	0.96343 (max o	7	6304; 6316; 6624; 66	0.07220789	0.7306093		127.0
	ZINC04089541	-6.8	389.515	-2.6913	8	4	298.61	0.40652	171.5	0.74603	8514.941681041	0.26751	7.7611	-10.061	0.65385	0.60922	0.98742 (max o	8	7764; 5755; 6953; 69	0.5187356	0.12519477		127.0
	ZINC04089629	-6.8	359.489	-2.1239	7	3	279.76	0.38708	151.27	1.071	8514.941681041	0.2898	7.1937	-7.3289	0.70833	0.61366	0.95384 (max o	10	7764; 7587; 5755; 69	0.5206965	0.10435336		127.0
	ZINC24714660	-6.8	415.052	-19.03	15	4	239.36	0.85587	304.55	-31.825	8514.941681041	0.2898	24.1	-65.666	0.41667	0.55758	0.97246 (max o	23	7929; 7884; 7883; 78	0.2974636	0.77736014		127.0
	ZINC24433941	-6.8	415.052	-19.03	15	4	239.36	0.85587	304.55	-31.825	8514.941681041	0.2898	24.1	-65.666	0.41667	0.55758	0.97246 (max o	26	7929; 7885; 7884; 78	0.3286946	0.7583684		127.0
	ZINC26819872	-6.8	415.052	-19.03	15	4	239.36	0.85587	304.55	-31.825	8514.941681041	0.2898	24.1	-65.666	0.41667	0.55758	0.97246 (max o	25	7929; 7885; 7884; 78	0.35623512	0.8259512		127.0
	ZINC26973607	-6.8	414.044	-20.278	15	3	240.54	0.85657	307.38	-20.593	8514.941681041	0.2898	25.347	-69.971	0.41667	0.58345	0.94034 (max o	10	7928; 7882; 7833; 76	0.38454694	0.87089133		127.0
	ZINC26819872	-6.8	414.044	-20.278	15	3	240.54	0.85657	307.38	-20.593	8514.941681041	0.2898	25.347	-69.971	0.41667	0.58345	0.94718 (max o	10	7928; 7882; 7833; 76	0.39249203	0.88429826		127.0
	ZINC24714660	-6.8	414.044	-20.278	15	3	240.54	0.85657	307.38	-20.593	8514.941681041	0.2898	25.347	-69.971	0.41667	0.58345	0.93686 (max o	10	7928; 7882; 7833; 76	0.39665306	0.8535368		127.0
	ZINC24433941	-6.8	414.044	-20.278	15	3	240.54	0.85657	307.38	-20.593	8514.941681041	0.2898	25.347	-69.971	0.41667	0.58345	0.95709 (max o	10	7928; 7882; 7833; 76	0.40802976	0.8357791		127.0
	ZINC24714660	-6.8	415.052	-19.03	15	4	239.36	0.85587	304.55	-31.825	8514.941681041	0.2898	24.1	-65.666	0.41667	0.55758	0.97246 (max o	23	7929; 7884; 7883; 78	0.32577896	0.7902276		127.0
	ZINC26973607	-6.8	415.052	-19.03	15	4	239.36	0.85587	304.55	-31.825	8514.941681041	0.2898	24.1	-65.666	0.41667	0.55758	0.97246 (max o	24	7929; 7930; 7884; 78	0.36899483	0.80876136		127.0
	ZINC24433941	-6.8	415.052	-19.03	15	4	239.36	0.85587	304.55	-31.825	8514.941681041	0.2898	24.1	-65.666	0.41667	0.55758	0.97246 (max o	28	7929; 7930; 7885; 78	0.34824786	0.758035		127.0
	ZINC24433941	-6.8	415.052	-19.03	15	4	239.36	0.85587	304.55	-31.825	8514.941681041	0.2898	24.1	-65.666	0.41667	0.54974	0.96029 (max o	26	7930; 7929; 7885; 78	0.338758	0.7419957		127.0
	ZINC26819872	-6.8	415.052	-19.03	15	4	239.36	0.85587	304.55	-31.825	8514.941681041	0.2898	24.1	-65.666	0.41667	0.55758	0.97246 (max o	24	7929; 7885; 7884; 78	0.3442085	0.7920475		127.0
	ZINC26973607	-6.8	415.052	-19.03	15	4	239.36	0.85587	304.55	-31.825	8514.941681041	0.2898	24.1	-65.666	0.41667	0.55758	0.97246 (max o	24	7930; 7929; 7884; 78	0.3759324	0.77604574		127.0
	ZINC24714660	-6.8	415.052	-19.03	15	4	239.36	0.85587	304.55	-31.825	8514.941681041	0.2898	24.1	-65.666	0.41667	0.54974	0.95709 (max o	16	7930; 7885; 7862; 78	0.40044862	0.7417127		127.0
	ZINC35271750	-6.8	395.426	-0.9065	8	4	297.67	0.34521	147.35	-8.9294	8514.941681041	0.2484	5.9763	-3.6494	0.46429	0.61198	0.95983 (max o	5	7558; 7248; 7260; 74	0.41231593	-0.5651980		127.0
	ZINC26973607	-6.8	415.052	-19.03	15	4	239.36	0.85587	304.55	-31.825	8514.941681041	0.2898	24.1	-65.666	0.41667	0.54974	0.95709 (max o	18	7930; 7929; 7885; 78	0.40300742	0.79338497		127.0
	ZINC35271747	-6.8	395.426	-0.9065	8	4	297.67	0.34521	147.35	-8.9294	8514.941681041	0.2484	5.9763	-3.6494	0.46429	0.61198	0.95983 (max o	5	7936; 7079; 7260; 74	0.44823378	-0.5564469		127.0
	ZINC26819872	-6.8	415.052	-19.03	15	4	239.36	0.85587	304.55	-31.825	8514.941681041	0.2898	24.1	-65.666	0.41667	0.54974	0.96029 (max o	23	7930; 7885; 7884; 78	0.39566487	0.77580553		127.0
	ZINC14781596	-6.8	410.461	1.5974	8	4	312.4	0.31524	133.52	-11.395	8514.941681041	0.23983	3.4724	6.6604	0.44828	0.60755	0.96428 (max o	4	7886; 7741; 7717; 76	0.44338346	-0.4975513		127.0
	ZINC35271762	-6.8	410.461	1.5974	8	4	312.4	0.31524	133.52	-11.395	8514.941681041	0.23983	3.4724	6.6604	0.44828	0.60936	0.96428 (max o	4	7936; 7935; 7741; 75	0.42296818	-0.5300666		127.0
	ZINC04097583	-6.8	358.367	-2.7155	10	4	236.53	0.6099	202.62	-7.7206	8514.941681041	0.31614	7.7853	-8.5894	0.5	0.58961	0.96898 (max o	3	7504; 5374; 7358	-0.4032443	0.5784339		120.0
	ZINC13527691	-6.8	358.367	-2.7155	10	4	236.53	0.6099	202.62	-7.7206	8514.941681041	0.31614	7.7853	-8.5894	0.5	0.58961	0.96898 (max o	5	7688; 7689; 5374; 73	-0.4080288	0.59930086		120.0

Structure	Molecule Name	affinity	Total Molweight	cLogP	H-Acceptors	H-Donors	Total Surface Area	Relative PSA	Polar Surface Area	Druglikeness	Kd (nM) @ 20°C	LE from K...	LLE from Kd ...	LELP from Kd (n...	Shape Index	Molecular...	Neighbor Si...	Neig...	Neighbor	Neighbor ...	Neighbor ...	Ring Systems	frequency
	ZINC14773871	−6.8	357.270	−6.1116	11	4	257.11	0.54685	203.43	−8.5455	8514.941681041	0.27821	11.181	−21.968	0.48	0.58661	0.94678 (max o	2	7192; 7444	−0.8641046	0.27730894		120.0
	ZINC49601690	−6.8	365.354	−3.573	10	4	261.9	0.46873	165.81	1.5639	8514.941681041	0.27821	8.6428	−12.843	0.56	0.66021	0.94678 (max o	6	7748; 7658; 7657; 63	−0.6017371	−0.3178525		120.0
	ZINC31159574	−6.8	350.406	−0.6232	8	6	256.52	0.38438	139.84	−9.7913	8514.941681041	0.2898	5.693	−2.1505	0.5	0.56944	0.99271 (max o	5	7896; 7074; 7240; 74	−0.6477242	−0.3675037		120.0
	ZINC71404434	−6.8	436.502	−3.8395	11	4	288.25	0.57929	238.9	−8.9899	8514.941681041	0.26751	8.9093	−14.353	0.5	0.60303	0.98956 (max o	5	7752; 7688; 7689; 70	−0.3656919	0.5881008		120.0
	ZINC35457923	−6.8	365.397	−2.6206	9	5	273.11	0.41309	159.74	−12.662	8514.941681041	0.27821	7.6904	−9.4196	0.56	0.58662	0.99111 (max o	5	7854; 7742; 7242; 74	−0.1539277	−0.5044319		120.0
	ZINC35457917	−6.8	365.397	−2.6206	9	5	273.11	0.41309	159.74	−12.662	8514.941681041	0.27821	7.6904	−9.4196	0.56	0.58767	0.99111 (max o	6	7854; 7742; 7738; 72	−0.1364530	−0.5169760		120.0
	ZINC31159578	−6.8	350.406	−0.6232	8	6	256.52	0.38438	139.84	−9.7913	8514.941681041	0.2898	5.693	−2.1505	0.5	0.57812	0.99271 (max o	3	7896; 7414; 7545	−0.6614709	−0.3330856		120.0
	ZINC13425156	−6.8	389.356	−0.672	11	1	287.77	0.44014	143.53	−0.087423	8514.941681041	0.2576	5.7418	−2.6087	0.40741	0.45182	0.95493 (max o	7	7949; 7838; 7694; 75	0.2769267	0.4014254		120.0
	ZINC05249464	−6.8	389.356	−0.672	11	1	287.77	0.44014	143.53	−0.087423	8514.941681041	0.2576	5.7418	−2.6087	0.40741	0.44763	0.96343 (max o	6	7828; 7820; 7783; 75	0.24941427	0.43843287		120.0
	ZINC05249465	−6.8	389.356	−0.672	11	1	287.77	0.44014	143.53	−0.087423	8514.941681041	0.2576	5.7418	−2.6087	0.40741	0.44763	0.95493 (max o	6	7874; 7828; 7765; 75	0.28032425	0.45571527		120.0
	ZINC05249466	−6.8	389.356	−0.672	11	1	287.77	0.44014	143.53	−0.087423	8514.941681041	0.2576	5.7418	−2.6087	0.40741	0.44763	0.96343 (max o	9	7918; 7874; 7828; 78	0.26937494	0.436869		120.0
	ZINC04096286	−6.8	350.299	−5.4965	11	5	242.59	0.56581	185.68	−1.0924	8514.941681041	0.2898	10.566	−18.967	0.45833	0.6115		0		−0.7721395	0.5059247		120.0
	ZINC04151729	−6.8	444.527	−2.4899	10	2	338.59	0.3941	170.24	3.3443	8514.941681041	0.23184	7.5597	−10.74	0.46667	0.63778	0.89488	1	7856	−0.1842424	−0.9166139		47.0
	ZINC02083416	−6.8	359.465	−0.6329	7	1	277.04	0.34616	124.07	−30.148	8514.941681041	0.2898	5.7027	−2.1839	0.625	0.56278		0		0.5268142	−0.7469278		47.0
	ZINC04012915	−6.8	362.139	−1.7236	10	5	206.36	0.57385	155.22	−0.33304	8514.941681041	0.3312	6.7934	−5.2041	0.47619	0.39655	0.91628 (max o	4	4059; 5710; 5840; 73	−0.2400542	−0.4406628		44.0
	ZINC12657747	−6.8	419.227	0.6029	9	1	270.19	0.35964	111.24	−2.4553	8514.941681041	0.27821	4.4669	2.1671	0.52	0.50238	0.94678 (max o	3	7645; 6514; 6631	−0.6289522	0.76035917		41.0
	ZINC13547843	−6.8	354.095	−0.8884	7	3	182.47	0.42358	99.1	3.3731	8514.941681041	0.40913	5.9582	−2.1714	0.58824	0.49973	0.90064 (max o	4	7673; 7577; 5711; 73	0.42687297	0.4039788		41.0
	ZINC20611520	−6.8	364.401	−1.6801	8	2	268.8	0.41071	143.94	5.2396	8514.941681041	0.27821	6.7499	−6.039	0.52	0.58028	0.97599	1	6388	0.07520076	−0.8077253		36.0
	ZINC04517514	−6.8	351.318	−0.7704	11	2	248.49	0.49994	151.68	1.3398	8514.941681041	0.27821	5.8402	−2.7692	0.44	0.2512	0.93348 (max o	7	4979; 4980; 5105; 51	−0.6297695	0.2989128		33.0
	ZINC19323246	−6.8	359.441	−3.4089	8	6	261.3	0.38148	125.9	3.8635	8514.941681041	0.27821	8.4787	−12.253	0.48	0.63628	0.95631 (max o	4	7616; 7026; 7370; 73	−0.6320989	0.26064768		23.0
	ZINC20464943	−6.8	382.479	−2.4243	8	4	310.39	0.29589	95.7	4.503	8514.941681041	0.2576	7.4941	−9.4111	0.59259	0.60873	0.91351 (max o	7	4300; 4302; 5933; 68	−0.3021192	0.02288660		22.0
	ZINC04533908	−6.8	383.349	−4.4475	12	8	254.94	0.57347	198.4	−4.7796	8514.941681041	0.26751	9.5173	−16.626	0.46154	0.46223	0.94951 (max o	10	7707; 7594; 7552; 67	0.48350742	−0.5467942		19.0
	ZINC04533905	−6.8	383.349	−4.4475	12	8	254.94	0.57347	198.4	−4.7796	8514.941681041	0.26751	9.5173	−16.626	0.46154	0.46223	0.94951 (max o	10	7830; 7594; 6778; 70	0.4777202	−0.5031142		19.0
	ZINC08636327	−6.8	407.557	−1.1369	7	5	319.8	0.34803	121.36	3.7326	8514.941681041	0.2484	6.2067	−4.5769	0.46429	0.44325	0.89505 (max o	5	5161; 6477; 6744; 69	0.4144846	0.1340909		18.0
	ZINC13348053	−6.8	383.349	−4.4475	12	8	254.94	0.57347	198.4	−3.0597	8514.941681041	0.26751	9.5173	−16.626	0.42308	0.46124	0.95559 (max o	4	7878; 6525; 6777; 70	0.50986767	−0.4317729		17.0
	ZINC33851455	−6.8	397.375	−4.0196	12	7	270.85	0.52834	187.4	−6.2954	8514.941681041	0.2576	9.0894	−15.604	0.40741	0.4601	0.94622 (max o	7	7594; 7004; 7081; 71	0.5186886	−0.5350856		17.0
	ZINC72320312	−6.8	353.433	−0.2864	6	2	262.54	0.29161	106.89	−1.0973	8514.941681041	0.27821	5.3562	−1.0294	0.44	0.46819	0.9402 (max of	3	5683; 6377; 6915	0.3538092	−0.7411948		16.0
	ZINC20463955	−6.8	355.452	−6.6152	6	3	290.88	0.2654	67.01	−2.1961	8514.941681041	0.27821	11.685	−23.778	0.6	0.52882	0.92342 (max o	6	2611; 5581; 5951; 65	0.6064755	0.01406993		16.0
	ZINC20463934	−6.8	367.488	−6.786	7	3	306.79	0.28423	76.24	−0.6715	8514.941681041	0.26751	11.856	−25.368	0.61538	0.53977	0.93069 (max o	7	7627; 2611; 5581; 59	0.60227776	0.03574879		16.0
	ZINC20463955	−6.8	355.452	−3.1729	6	3	275.66	0.22484	62.98	2.0755	8514.941681041	0.27821	8.2427	−11.405	0.6	0.54105	0.92342 (max o	8	7628; 2612; 5952; 65	0.503522	−0.2365140		16.0
	ZINC20463934	−6.8	367.488	−3.3437	7	3	291.57	0.24687	72.21	3.5077	8514.941681041	0.26751	8.4135	−12.499	0.61538	0.55102	0.93069 (max o	7	7628; 5952; 6594; 70	0.49580425	−0.256463		16.0
	ZINC12483763	−6.8	352.453	−2.0698	7	3	266.43	0.24716	79.8	2.3154	8514.941681041	0.27821	7.1396	−7.4398	0.64	0.5262	0.86427 (max o	2	7174; 7346	0.46847537	−0.283097		16.0
	ZINC03977951	−6.8	407.550	−2.4	8	6	309.52	0.39409	148.99	0.28877	8514.941681041	0.2576	7.4698	−9.3168	0.51852	0.44769	0.98806 (max o	8	7679; 2403; 5779; 68	0.73160875	−0.2781945		15.0
	ZINC03830997	−6.8	407.550	−2.4	8	6	309.52	0.39409	148.99	0.28877	8514.941681041	0.2576	7.4698	−9.3168	0.51852	0.4657	0.97658 (max o	15	7679; 7464; 2403; 39	0.68774796	−0.2571604		15.0
	ZINC04556585	−6.8	353.111	−1.3798	7	3	184.75	0.43069	108.38	2.1634	8514.941681041	0.40913	6.4496	−3.3725	0.58824	0.49295	0.90064 (max o	5	7887; 7840; 7812; 76	−0.4448181	−0.0229510		14.0
	ZINC12483821	−6.8	355.365	−0.5644	8	4	257.13	0.35204	101.08	0.097552	8514.941681041	0.27821	5.6342	−2.0287	0.64	0.4836	0.9302 (max of	4	4645; 5051; 5052; 62	−0.6579293	−0.3122834		14.0
	ZINC03838881	−6.8	373.476	−1.485	9	3	282.11	0.40743	150.14	9.6273	8514.941681041	0.27821	6.5548	−5.3377	0.52	0.64912	0.91611 (max o	5	7755; 5722; 5765; 67	−0.0684014	−0.0460818		13.0
	ZINC14416994	−6.8	390.340	−0.2551	11	0	286.31	0.43729	140.73	−0.85962	8514.941681041	0.2576	5.3249	−0.9903	0.40741	0.39737	0.93058 (max o	3	7788; 6977; 7167	0.78979486	0.2758836		10.0
	ZINC02140640	−6.8	384.136	0.9648	5	3	207.7	0.28551	79.15	−3.1395	8514.941681041	0.36606	4.105	2.6356	0.52632	0.26722	0.91405 (max o	3	7682; 6708; 7305	−0.4394518	−0.8614909		8.0
	ZINC15676407	−6.8	358.417	−1.3524	7	1	268.13	0.21072	74.91	1.5766	8514.941681041	0.26751	6.4222	−5.0556	0.53846	0.50045	0.87576	1	7196	−0.2274957	0.3621585		8.0
	ZINC28244802	−6.8	357.409	−3.6337	10	5	268.21	0.44573	174.5	3.4874	8514.941681041	0.27821	8.7035	−13.061	0.64	0.64303	0.9654 (max of	3	7914; 7588; 6442	0.12783448	−0.7852683		8.0
	ZINC33902777	−6.8	393.523	−2.8544	8	6	295.76	0.41243	148.99	2.3085	8514.941681041	0.26751	7.9242	−10.67	0.5	0.46343	0.90311 (max o	5	7289; 7290; 7427; 74	0.63276887	−0.2176452		7.0
	ZINC49180885	−6.8	418.481	−0.2872	9	6	293.11	0.38088	156.91	−5.2779	8514.941681041	0.23983	5.357	−1.1975	0.37931	0.4602	0.95908 (max o	3	7747; 4427; 6034	−0.5760433	−0.6540048		6.0

Structure	Molecule Name	affinity	Total Molweight	cLogP	H-Acceptors	H-Donors	Total Surface Area	Relative PSA	Polar Surface Area	Druglikeness	Kd (nM) @ 20°C	LE from K...	LLE from Kd ...	LELP from Kd (n...	Shape Index	Molecular...	Neighbor Si...	Neig...	Neighbor	Neighbor ...	Neighbor ...	Ring Systems	frequency
	ZINC09111944	-6.8	412.442	-3.6619	9	1	272.61	0.52669	192.44	9.2125	8514.941681041	0.2576	8.7317	-14.215	0.44444	0.42685	0.97903 (max o	2	6961; 7143	-0.3546939	-0.4852450		6.0
	ZINC04236340	-6.8	364.401	-1.7745	8	0	257.69	0.37817	129.3	3.3816	8514.941681041	0.27821	6.8443	-6.3783	0.44	0.48134	0.91351 (max o	4	5782; 5783; 6145; 69	-0.1551593	-0.7836808		5.0
	ZINC04899557	-6.8	369.444	-3.195	10	4	260.49	0.38604	172.03	5.0569	8514.941681041	0.26751	8.2648	-11.944	0.57692	0.57433	0.84495 (max o	2	5100; 7357	-0.0824291	-0.8968285		5.0
	ZINC31161979	-6.8	382.407	-0.3469	8	2	264.11	0.37837	114.82	1.0254	8514.941681041	0.2576	5.4167	-1.3467	0.37037	0.29852	0.98371 (max o	6	7797; 7731; 815; 660	-0.1061797	0.86589366		4.0
	ZINC06379180	-6.8	388.368	-1.8524	10	4	278.22	0.42585	151.98	-10.162	8514.941681041	0.2576	6.9222	-7.191	0.48148	0.47692	0.96064 (max o	3	5810; 6467; 6468	0.9004423	-0.0957143		4.0
	ZINC49601554	-6.8	445.396	-4.3457	12	6	310.43	0.47985	206.27	-2.4636	8514.941681041	0.22436	9.4155	-19.369	0.3871	0.49772	0.98065 (max o	3	4429; 4854; 5261	-0.5061130	-0.7489569		4.0
	ZINC31160236	-6.8	397.355	-2.4322	10	4	280.77	0.44104	161.88	-4.6331	8514.941681041	0.2484	7.502	-9.7915	0.42857	0.47211	0.89582 (max o	3	5192; 6293; 6855	0.15897879	-0.6245320		4.0
	ZINC24718523	-6.8	396.355	-2.075	12	1	279.99	0.46498	145.19	4.6603	8514.941681041	0.2484	7.1448	-8.3535	0.42857	0.4146	0.93385 (max o	3	6595; 6649; 7441	-0.8008588	-0.0764317		4.0
	ZINC03984365	-6.8	417.308	2.4972	6	2	292.32	0.19205	78.96	-11.758	8514.941681041	0.2576	2.5726	9.6941	0.55556	0.42417	0.96343 (max o	3	6944; 7299; 7300	0.7525266	0.1926425		4.0
	ZINC12530576	-6.8	355.457	-2.8068	8	3	291.67	0.31947	92.18	5.419	8514.941681041	0.27821	7.8766	-10.089	0.6	0.55296	0.93033 (max o	5	6510; 6771; 7021; 73	-0.2546003	0.0829674		4.0
	ZINC20462966	-6.8	395.566	-2.3699	6	1	329.29	0.25938	96.36	7.55	8514.941681041	0.2576	7.4397	-9.1999	0.55556	0.68743	0.90719 (max o	4	7622; 7344; 7514; 75	-0.2679533	-0.0583157		4.0
	ZINC20462966	-6.8	396.574	-1.9632	6	2	343.33	0.28966	97.56	6.1912	8514.941681041	0.2576	7.033	-7.6211	0.55556	0.67497	0.90311 (max o	4	7624; 7344; 7514; 75	-0.2482389	-0.0629804		4.0
	ZINC20462966	-6.8	395.566	-2.3699	6	1	329.29	0.25938	96.36	8.3296	8514.941681041	0.2576	7.4397	-9.1999	0.55556	0.68987	0.92394 (max o	4	7724; 7623; 7515; 75	-0.2560825	-0.1024456		4.0
	ZINC62001867	-6.8	350.346	-1.0278	8	1	258.71	0.36667	113.99	-1.092	8514.941681041	0.27821	6.0976	-3.6944	0.48	0.34288	0.92662 (max o	3	7659; 7568; 7569	0.5920372	-0.6320044		4.0
	ZINC62001866	-6.8	350.346	-1.0278	8	1	258.71	0.36667	113.99	-1.092	8514.941681041	0.27821	6.0976	-3.6944	0.48	0.34288	0.92662 (max o	3	7659; 7570; 7568	0.57710737	-0.6166965		4.0
	ZINC62001865	-6.8	350.346	-1.0278	8	1	258.71	0.36667	113.99	-1.092	8514.941681041	0.27821	6.0976	-3.6944	0.48	0.34288	0.9402 (max of	3	7659; 7570; 7569	0.5838301	-0.6526329		4.0
	ZINC04082894	-6.8	357.473	-1.8582	7	1	266.7	0.34672	133.69	4.5333	8514.941681041	0.2898	6.928	-6.412	0.625	0.5306	0.87324 (max o	2	5371; 5750	0.9625621	0.11292854		3.0
	ZINC02110058	-6.8	361.397	0.3672	8	2	272.07	0.28614	94.83	4.084	8514.941681041	0.26751	4.7026	1.3727	0.5	0.43974	0.81848 (max o	2	5435; 6077	0.7654745	-0.1950647		3.0
	ZINC12483763	-6.8	352.453	-2.0698	7	3	266.43	0.24716	79.8	2.0439	8514.941681041	0.27821	7.1396	-7.4398	0.64	0.55556	0.84495 (max o	3	5536; 6808; 7495	0.5525813	-0.0961483		3.0
	ZINC12483763	-6.8	351.445	-0.8451	7	3	270.15	0.25752	75.22	2.4708	8514.941681041	0.27821	5.9149	-3.0377	0.64	0.5648	0.84495 (max o	2	6805; 7497	0.5839744	-0.0613939		3.0
	ZINC38549936	-6.8	374.499	0.8471	5	3	312.02	0.20444	63.36	-6.941	8514.941681041	0.2576	4.2227	3.2884	0.51852	0.47399	0.97407 (max o	3	3520; 5648; 7445	0.03999304	-0.7852774		2.0
	ZINC04025192	-6.8	364.349	1.4871	8	1	258.07	0.35734	108.36	-3.926	8514.941681041	0.26751	3.5827	5.5591	0.38462	0.42992	0.95559 (max o	4	7762; 6414; 7307; 74	-0.5396589	0.2437821		2.0
	ZINC09033111	-6.8	371.301	-1.2117	12	1	262.31	0.50215	149.9	5.0025	8514.941681041	0.26751	6.2815	-4.5296	0.46154	0.45362	0.95857 (max o	4	7744; 7700; 6651; 68	0.36726943	-0.3169348		2.0
	ZINC05398265	-6.8	403.479	-0.1522	9	1	282.65	0.4126	149.71	4.3554	8514.941681041	0.26751	5.222	-0.56896	0.46154	0.54963	0.8719 (max of	2	7770; 7157	-0.1116798	0.3787891		2.0
	ZINC00626769	-6.8	364.349	1.4871	8	1	258.07	0.35734	108.36	-3.926	8514.941681041	0.26751	3.5827	5.5591	0.38462	0.42992	0.95559 (max o	3	7762; 7468; 6414	-0.5181682	0.23864414		2.0
	ZINC35242174	-6.8	386.360	-3.1619	11	0	278.56	0.5312	171.24	3.2541	8514.941681041	0.26751	8.2317	-11.82	0.46154	0.44595	0.94951 (max o	3	7648; 7647; 7432	0.44579178	-0.3128694		2.0
	ZINC40313016	-6.8	386.491	-0.667	8	2	296.3	0.30773	89.92	4.5684	8514.941681041	0.26751	5.7368	-2.4934	0.5	0.49466	0.96488	1	7654	0.51393557	-0.6550899		2.0
	ZINC03830510	-6.8	395.435	-1.4848	8	1	271.44	0.47635	169.38	11.089	8514.941681041	0.26751	6.5546	-5.5505	0.57692	0.4758	0.94008 (max o	4	3592; 4910; 4911; 58	0.42294195	0.50265324		1.0
	ZINC04151665	-6.8	440.519	-1.3831	10	3	333.02	0.40316	168.3	9.0358	8514.941681041	0.23184	6.4529	-5.9658	0.5	0.55918	0.88128 (max o	2	7676; 6933	-0.7153812	0.5375557		1.0
	ZINC08627195	-6.8	354.449	-4.77	8	5	285.83	0.33936	112.73	0.30635	8514.941681041	0.27821	9.8398	-17.145	0.68	0.51542	0.86353	1	4173	0.19317873	-0.4944156		1.0
	ZINC15673452	-6.8	354.449	-1.6395	8	3	295.35	0.33164	102.48	1.5929	8514.941681041	0.27821	6.7093	-5.8931	0.64	0.63515	0.85045 (max o	2	4166; 5160	0.01855564	0.8502989		1.0
	ZINC19703402	-6.8	372.804	-3.136	8	1	283.76	0.33736	129.67	-4.8652	8514.941681041	0.27821	8.2058	-11.272	0.72	0.65826		0		0.89968604	0.3683881		1.0
	ZINC20465265	-6.8	357.469	-2.6223	7	4	284.11	0.31076	92.46	0.72453	8514.941681041	0.27821	7.6921	-9.4257	0.52	0.44652	0.86577 (max o	3	5936; 6581; 6823	-0.7880946	-0.5698033		1.0
	ZINC22920588	-6.8	353.505	-1.2265	6	1	281.42	0.19615	80.29	1.189	8514.941681041	0.27821	6.2963	-4.4086	0.52	0.65455	0.89438 (max o	3	7724; 4777; 7345	-0.2032194	-0.1257128		1.0
	ZINC04095673	-6.8	427.568	-0.0252	10	6	336.81	0.33289	168.01	-3.5735	8514.941681041	0.23184	5.095	-0.1087	0.53333	0.67233	0.98786 (max o	6	7686; 7483; 1567; 15	-0.3322865	0.653045		0.0
	ZINC67913526	-6.8	429.576	0.7542	7	2	335.06	0.31466	148.83	-1.2855	8514.941681041	0.23983	4.3156	3.1447	0.44828	0.56223	0.97176 (max o	3	4864; 5673; 6675	0.75337195	0.25868225		0.0
	ZINC04023944	-6.8	387.479	-1.4592	10	5	294.99	0.37933	171.2	-56.425	8514.941681041	0.2576	6.529	-5.6646	0.55556	0.66106	0.96343 (max o	3	5358; 6113; 6945	-0.1458179	-0.4429711		0.0
	ZINC04556708	-6.8	441.595	0.4292	10	6	350.57	0.31982	168.01	1.4649	8514.941681041	0.22436	4.6406	1.913	0.54839	0.67145	0.97423 (max o	4	7686; 6967; 7149; 74	-0.3743330	0.65466344		0.0
	ZINC31157904	-6.8	436.456	-2.3414	12	6	333.18	0.4433	198.7	-1.3926	8514.941681041	0.23184	7.4112	-10.099	0.6	0.67776	0.97757 (max o	3	7643; 7642; 7238	0.57363945	0.75193655		0.0
	ZINC35464455	-6.8	356.413	-2.0268	7	3	297.45	0.31582	140.95	-11.414	8514.941681041	0.27821	7.0966	-7.2852	0.8	0.70385	0.96832 (max o	3	7739; 7650; 7246	-0.5971123	0.49697992		0.0
	ZINC31166295	-6.8	354.441	-0.6237	6	2	296.78	0.27239	120.72	-4.8261	8514.941681041	0.27821	5.6935	-2.2419	0.68	0.68996	0.97932 (max o	9	7957; 7933; 7799; 77	-0.0061001	0.6989581		0.0
	ZINC03920510	-6.8	420.374	-11.982	13	3	304.56	0.62467	271.66	1.7888	8514.941681041	0.2484	17.052	-48.237	0.5	0.73054	0.98232 (max o	3	7785; 7600; 7601	-0.0916196	0.76796556		0.0
	ZINC04556666	-6.8	357.493	-0.6003	7	3	294.77	0.30804	125.97	3.1822	8514.941681041	0.27821	5.6701	-2.1577	0.52	0.65015	0.9594 (max of	3	7768; 7695; 7595	-0.1960026	0.7951506		0.0

Structure	Molecule Name	affinity	Total Molweight	cLogP	H-Acceptors	H-Donors	Total Surface Area	Relative PSA	Polar Surface Area	Druglikeness	Kd (nM) @ 20°C	LE from K...	LLE from Kd ...	LELP from Kd (n...	Shape Index	Molecular...	Neighbor Si...	Neig...	Neighbor	Neighbor ...	Neighbor ...	Ring Systems	frequency
	ZINC31167600	-6.8	400.494	-1.8098	9	4	325.36	0.35014	147.66	3.0018	8514.941681041	0.2484	6.8796	-7.2858	0.60714	0.66585	0.97533 (max o	3	7803; 7743; 7740	-0.8895744	0.24304175		0.0
	ZINC03874923	-6.8	390.479	-4.3206	9	3	308.8	0.43915	191.4	-8.4511	8514.941681041	0.26751	9.3904	-16.151	0.61538	0.71144	0.97668 (max o	4	7915; 7825; 7824; 77	0.31820205	-0.9383296		0.0
	ZINC30728756	-6.8	401.246	-4.9537	9	3	283.29	0.4787	191.4	0.6575	8514.941681041	0.2898	10.024	-17.094	0.54167	0.69268	0.89547 (max o	4	7915; 7869; 7818; 77	0.33933112	-0.9162341		0.0
	ZINC14613439	-6.8	425.462	-9.2176	12	4	304.5	0.64007	284.47	0.11401	8514.941681041	0.2576	14.287	-35.783	0.55556	0.75908	0.97407 (max o	3	7915; 7839; 7836	0.27538234	-0.9336384		0.0
	ZINC31163788	-6.8	417.541	-0.0595	8	5	341.45	0.31407	165.76	-22.567	8514.941681041	0.23983	5.1293	-0.24809	0.72414	0.64811	0.9279 (max of	6	7909; 7899; 7881; 78	0.7399917	0.5033868		0.0
	ZINC38573710	-6.7	383.874	-0.7384	7	3	294.41	0.30841	125.97	2.4098	10109.69635951	0.26357	5.7337	-2.8015	0.5	0.55558	0.91255	1	6485	0.6484886	0.39358845		134.0
	ZINC31165077	-6.7	350.278	-4.1042	9	1	263.19	0.43246	153.09	-6.0092	10109.69635951	0.27412	9.0995	-14.972	0.6	0.55283	0.97396 (max o	7	7897; 7798; 5212; 59	0.82360154	0.11491597		134.0
	ZINC49181826	-6.7	414.409	-3.3527	11	6	302.34	0.46881	204.09	0.26746	10109.69635951	0.23631	8.348	-14.188	0.51724	0.61554	0.98727 (max o	3	6355; 6899; 7655	-0.3304356	0.7618828		134.0
	ZINC49180788	-6.7	398.410	-3.2289	10	5	296.1	0.43482	187.35	-0.71607	10109.69635951	0.24475	8.2242	-13.193	0.53571	0.63234	0.95148 (max o	5	7656; 6353; 6664; 72	-0.3512251	0.752608		134.0
	ZINC04084430	-6.7	419.609	-1.5203	7	3	324.77	0.38729	176.57	2.3408	10109.69635951	0.25381	6.5156	-5.9899	0.7037	0.61651	0.96885 (max o	10	7764; 5755; 6952; 69	0.5764703	0.09673155		127.0
	ZINC12503563	-6.7	415.052	-19.03	15	4	239.36	0.85587	304.55	-31.825	10109.69635951	0.28554	24.025	-66.646	0.41667	0.55758	0.97246 (max o	26	7929; 7885; 7884; 78	0.35625267	0.77503556		127.0
	ZINC12883785	-6.7	401.570	-0.9968	7	3	318.28	0.34024	151.27	3.5206	10109.69635951	0.25381	5.9921	-3.9273	0.66667	0.60655	0.96885 (max o	9	7782; 7764; 6426; 67	0.596499	0.05777265		127.0
	ZINC12503563	-6.7	415.052	-19.03	15	4	239.36	0.85587	304.55	-31.825	10109.69635951	0.28554	24.025	-66.646	0.41667	0.55758	0.97246 (max o	25	7929; 7884; 7883; 78	0.30781552	0.79103184		127.0
	ZINC12503563	-6.7	414.044	-20.278	15	3	240.54	0.85657	307.38	-20.593	10109.69635951	0.28554	25.273	-71.016	0.41667	0.58345	0.94718 (max o	10	7928; 7882; 7833; 50	0.40611675	0.8678304		127.0
	ZINC12503563	-6.7	415.052	-19.03	15	4	239.36	0.85587	304.55	-31.825	10109.69635951	0.28554	24.025	-66.646	0.41667	0.54974	0.96029 (max o	27	7930; 7929; 7885; 78	0.3683983	0.7592114		127.0
	ZINC14781598	-6.7	410.461	1.5974	8	4	312.4	0.31524	133.52	-11.395	10109.69635951	0.23631	3.3979	6.7599	0.44828	0.60755	0.96428 (max o	3	7886; 7717; 7509	0.4497995	-0.4545651		127.0
	ZINC67913936	-6.7	424.441	-1.0613	11	5	315.59	0.41693	172.21	-18.63	10109.69635951	0.23631	6.0566	-4.4912	0.51724	0.56882	0.95642 (max o	2	7749; 6048	-0.6676034	0.3763141		120.0
	ZINC49601692	-6.7	365.354	-3.573	10	4	261.9	0.46873	165.81	1.5639	10109.69635951	0.27412	8.5683	-13.035	0.56	0.66255	0.94678 (max o	6	6358; 7100; 7271; 74	-0.6050212	-0.3378853		120.0
	ZINC49601683	-6.7	365.354	-3.573	10	4	261.9	0.46873	165.81	-8.2227	10109.69635951	0.27412	8.5683	-13.035	0.56	0.60151	0.95318 (max o	5	7748; 7658; 7271; 74	-0.6080793	-0.3574992		120.0
	ZINC05227086	-6.7	389.356	-0.672	11	1	287.77	0.44014	143.53	-0.087423	10109.69635951	0.25381	5.6673	-2.6476	0.40741	0.45182	0.95493 (max o	5	7874; 7828; 7486; 74	0.28062528	0.41988614		120.0
	ZINC14515456	-6.7	376.313	-0.6041	11	0	272.55	0.45937	140.73	-2.6337	10109.69635951	0.26357	5.5994	-2.292	0.38462	0.38639	0.94951 (max o	3	7953; 7832; 7693	0.18423432	0.43219107		120.0
	ZINC04204375	-6.7	375.329	-1.6813	11	1	272.57	0.47867	157.52	1.71	10109.69635951	0.26357	6.6766	-6.3789	0.42308	0.45974		0		-0.9076872	0.23016544		120.0
	ZINC08918128	-6.7	430.544	-1.1899	9	2	331.01	0.36374	153.17	-53.087	10109.69635951	0.23631	6.1852	-5.0354	0.62069	0.59499	0.92805 (max o	5	5074; 5466; 5697; 57	0.5033998	-0.1350691		47.0
	ZINC04081163	-6.7	373.516	-1.3477	7	2	290.86	0.34511	142.48	5.1691	10109.69635951	0.27412	6.343	-4.9165	0.64	0.57258	0.9654 (max of	5	7815; 6949; 7001; 71	0.7619936	-0.4126764		47.0
	ZINC33904021	-6.7	419.227	0.6029	9	1	270.19	0.35964	111.24	-2.4553	10109.69635951	0.27412	4.3924	2.1994	0.52	0.50035	0.93348 (max o	3	6514; 6631; 7500	-0.6107614	0.7722233		41.0
	ZINC02562618	-6.7	354.095	-0.8884	7	3	182.47	0.42358	99.1	3.3731	10109.69635951	0.40311	5.8837	-2.2039	0.58824	0.50105	0.90599 (max o	4	7673; 7669; 7366; 75	0.4296927	0.3816821		41.0
	ZINC19323242	-6.7	359.441	-3.4089	8	6	261.3	0.38148	125.9	3.8635	10109.69635951	0.27412	8.4042	-12.436	0.48	0.64047	0.90311 (max o	4	6796; 7026; 7371; 75	-0.6525457	0.22171846		23.0
	ZINC08643323	-6.7	396.462	-2.586	9	5	317.04	0.35576	121.56	0.83312	10109.69635951	0.24475	7.5813	-10.566	0.67857	0.59257	0.9428 (max of	4	4225; 5063; 5455; 67	0.39577356	-0.4406241		22.0
	ZINC12957391	-6.7	353.111	-1.3798	7	3	184.75	0.43069	108.38	2.1634	10109.69635951	0.40311	6.3751	-3.4229	0.58824	0.49063	0.90599 (max o	5	7841; 7840; 7826; 71	-0.4637324	-0.0285642		20.0
	ZINC17142919	-6.7	371.101	-1.5308	7	3	189.84	0.41914	108.38	1.2024	10109.69635951	0.38072	6.5261	-4.0208	0.55556	0.49566	0.84217	1	7841	-0.4543351	-0.0648163		20.0
	ZINC04533906	-6.7	383.349	-4.4475	12	8	254.94	0.57347	198.4	-4.7796	10109.69635951	0.26357	9.4428	-16.874	0.46154	0.46223	0.95257 (max o	12	7830; 6778; 7004; 70	0.50085986	-0.4951846		19.0
	ZINC12496137	-6.7	370.349	-3.6021	11	6	251.64	0.51105	167.53	-12.378	10109.69635951	0.27412	8.5974	-13.141	0.52	0.45537	0.82138	1	7777	-0.1306143	-0.9679599		19.0
	ZINC20463947	-6.7	367.488	-6.786	7	3	306.79	0.28423	76.24	-0.6715	10109.69635951	0.26357	11.781	-25.746	0.53846	0.52082	0.94338 (max o	3	6560; 7403; 7517	0.6120563	0.07502796		16.0
	ZINC20463947	-6.7	367.488	-3.3437	7	3	291.57	0.24687	72.21	3.5077	10109.69635951	0.26357	8.339	-12.686	0.53846	0.5324	0.94338 (max o	5	6594; 7066; 7404; 75	0.4909621	-0.2240688		16.0
	ZINC03839117	-6.7	365.432	-0.4616	9	3	289.11	0.33707	124.84	2.5978	10109.69635951	0.26357	5.4569	-1.7513	0.5	0.63834	0.92032 (max o	5	7816; 5723; 6935; 72	-0.0149272	-0.0146294		13.0
	ZINC03842120	-6.7	379.479	1.0219	7	0	287.98	0.26828	95.46	3.1652	10109.69635951	0.26357	3.9734	3.8771	0.57692	0.63868	0.97791 (max o	4	5338; 6452; 6704; 69	0.12100828	0.40530992		13.0
	ZINC04270694	-6.7	366.437	1.0555	7	0	275.42	0.30394	101.45	2.4399	10109.69635951	0.27412	3.9398	3.8506	0.6	0.61512	0.88639	1	6436	0.13872145	0.3724338		13.0
	ZINC04095645	-6.7	357.409	-3.6337	10	5	268.21	0.44573	174.5	3.4874	10109.69635951	0.27412	8.629	-13.256	0.64	0.6405	0.93348 (max o	3	7914; 6442; 7542	0.15265253	-0.7919337		8.0
	ZINC35464697	-6.7	356.413	-2.5089	7	2	285.37	0.34513	133.25	-4.0636	10109.69635951	0.27412	7.5042	-9.1527	0.64	0.62516	0.98434 (max o	3	7091; 7440; 7651	-0.6194640	0.67162085		8.0
	ZINC35464694	-6.7	356.413	-2.5089	7	2	285.37	0.34513	133.25	-4.0636	10109.69635951	0.27412	7.5042	-9.1527	0.64	0.62516	0.98434 (max o	3	7652; 7091; 7440	-0.6408086	0.6662545		8.0
	ZINC30879345	-6.7	425.544	-1.0926	8	3	357.78	0.26606	90.69	2.6434	10109.69635951	0.22843	6.0879	-4.7831	0.63333	0.63373	0.88151 (max o	4	7932; 4786; 6289; 72	-0.2998116	0.43417498		7.0
	ZINC30878622	-6.7	409.545	-0.7561	7	2	346.62	0.22295	72.67	4.4649	10109.69635951	0.23631	5.7514	-3.1997	0.58621	0.66469	0.89582 (max o	2	7235; 7410	-0.3393702	0.4505974		7.0
	ZINC35051184	-6.7	396.300	-8.4533	13	4	261.98	0.59944	207.33	-4.475	10109.69635951	0.25381	13.449	-33.305	0.48148	0.45254	0.95493 (max o	3	6311; 6873; 7257	-0.6355596	0.74510926		7.0

Structure	Molecule Name	affinity	Total Molweight	cLogP	H-Acceptors	H-Donors	Total Surface Area	Relative PSA	Polar Surface Area	Druglikeness	Kd (nM) @ 20°C	LE from K...	LLE from Kd ...	LELP from Kd (n...	Shape Index	Molecular...	Neighbor Si...	Neig...	Neighbor	Neighbor ...	Neighbor ...	Ring Systems	frequency
	ZINC04095696	-6.7	357.450	-2.7913	7	2	272.19	0.40821	162.3	-1.413	10109.69635951	0.28554	7.7866	-9.7756	0.66667	0.65098	0.96343 (max o	5	7916; 7716; 1569; 71	-0.1639104	0.47243598		6.0
	ZINC20464006	-6.7	400.497	-0.7759	7	2	301.23	0.21698	80.66	4.4331	10109.69635951	0.23631	5.7712	-3.2834	0.55172	0.48922	0.95123 (max o	8	7850; 7848; 2523; 59	0.6456485	0.5786389		6.0
	ZINC20464994	-6.7	373.923	-3.0554	6	4	295.84	0.21998	73.98	2.9126	10109.69635951	0.27412	8.0507	-11.146	0.56	0.5408	0.91964 (max o	3	6262; 6263; 7047	-0.3950032	0.26728958		6.0
	ZINC05397660	-6.7	371.434	-1.9007	10	1	250.04	0.44713	146.92	1.8271	10109.69635951	0.29795	6.896	-6.3792	0.47826	0.60722	0.85984	1	3221	0.00560729	0.46538278		5.0
	ZINC03839095	-6.7	397.498	-0.866	9	3	297.65	0.38616	150.14	5.4949	10109.69635951	0.25381	5.8613	-3.412	0.48148	0.56569	0.92418 (max o	2	7816; 7754	-0.0113454	-0.0753330		5.0
	ZINC03844369	-6.7	383.400	-0.0995	10	1	292.57	0.35465	122.32	-0.45661	10109.69635951	0.25381	5.0948	-0.39202	0.51852	0.64858	0.96885 (max o	3	6937; 7123; 7124	0.5779124	-0.5740944		5.0
	ZINC04293460	-6.7	441.388	-0.0395	12	1	324.96	0.43319	156.67	2.0065	10109.69635951	0.22106	5.0348	-0.17868	0.41935	0.40654	0.95771 (max o	5	7781; 7703; 6440; 69	0.3329686	0.39895412		4.0
	ZINC04024209	-6.7	383.372	-5.5025	9	1	279.03	0.42325	166.92	-4.6692	10109.69635951	0.25381	10.498	-21.68	0.40741	0.58806	0.98371 (max o	3	7761; 6513; 7181	-0.0044509	-0.7116235		4.0
	ZINC35457072	-6.7	375.392	-2.1888	9	2	279.57	0.38116	134.58	-21.096	10109.69635951	0.26357	7.1841	-8.3043	0.57692	0.50497	0.94326 (max o	4	6882; 7087; 7262; 74	-0.7317289	-0.0347175		4.0
	ZINC04062075	-6.7	378.376	1.2887	8	1	277.82	0.34288	116.2	-3.9534	10109.69635951	0.25381	3.7066	5.0774	0.44444	0.50718	0.98806 (max o	4	7684; 7683; 6414; 73	-0.5491108	0.28454545		4.0
	ZINC62001868	-6.7	350.346	-1.0278	8	1	258.71	0.36667	113.99	-1.092	10109.69635951	0.27412	6.0231	-3.7495	0.48	0.34288	0.9402 (max of	3	7568; 7569; 7570	0.57105577	-0.6382457		4.0
	ZINC20462911	-6.7	352.497	-0.1414	5	1	292.97	0.27941	93.12	4.9525	10109.69635951	0.28554	5.1367	-0.49521	0.5	0.62141	0.91567 (max o	3	7624; 7622; 7344	-0.2431467	-0.0849567		4.0
	ZINC20462962	-6.7	381.539	-2.368	6	1	315.53	0.27069	96.36	8.2254	10109.69635951	0.26357	7.3633	-8.9842	0.53846	0.65573	0.92394 (max o	2	7514; 7622	-0.2828746	-0.0752723		4.0
	ZINC20462962	-6.7	382.547	-1.9613	6	2	329.57	0.30176	97.56	6.0786	10109.69635951	0.26357	6.9566	-7.4412	0.53846	0.6386	0.89805 (max o	3	7516; 7618; 7622	-0.2624535	-0.0802661		4.0
	ZINC20462962	-6.7	381.539	-2.368	6	1	315.53	0.27069	96.36	7.4429	10109.69635951	0.26357	7.3633	-8.9842	0.53846	0.65293	0.90719 (max o	4	7624; 7515; 7623; 76	-0.277876	-0.0966571		4.0
	ZINC68603490	-6.7	388.371	1.1684	8	0	274.46	0.34686	113.04	-7.5418	10109.69635951	0.24475	3.8269	4.7739	0.32143	0.42832	0.84318 (max o	2	6364; 7108	-0.9264154	0.02010049		3.0
	ZINC19450366	-6.7	368.472	1.228	7	0	282.29	0.23458	76.15	0.67181	10109.69635951	0.26357	3.7673	4.659	0.5	0.59676	0.85189	1	6378	0.8121177	0.350327		3.0
	ZINC02101103	-6.7	396.438	0.6228	9	0	295.79	0.31205	110.29	-14.784	10109.69635951	0.24475	4.3725	2.5447	0.60714	0.63397		0		0.7457694	0.544964		3.0
	ZINC20465204	-6.7	350.413	-1.1165	7	3	263.29	0.28201	91.26	4.0754	10109.69635951	0.27412	6.1118	-4.0731	0.56	0.47947	0.89885 (max o	4	3001; 4309; 6577; 68	-0.6210923	0.5625864		3.0
	ZINC20503944	-6.7	394.606	-2.1871	6	3	336.02	0.25478	76.3	4.7994	10109.69635951	0.25381	7.1824	-8.617	0.7037	0.71986	0.89839 (max o	4	5957; 5958; 7060; 76	0.4580024	-0.5985977		3.0
	ZINC20464006	-6.7	401.505	-2.1422	7	3	315.27	0.25185	81.86	-0.10256	10109.69635951	0.23631	7.1375	-9.0653	0.55172	0.49431	0.95123 (max o	5	7849; 7847; 805; 592	0.43536082	0.4645325		3.0
	ZINC20462933	-6.7	354.513	-2.6116	6	2	314.15	0.25176	69.32	3.909	10109.69635951	0.27412	7.6069	-9.5274	0.56	0.63197	0.91351 (max o	3	7728; 7033; 7619	-0.3592965	-0.1568851		3.0
	ZINC20462933	-6.7	353.505	-3.0183	6	1	300.11	0.21675	68.12	5.3838	10109.69635951	0.27412	8.0136	-11.011	0.56	0.64762	0.91351 (max o	4	7726; 7621; 7032; 76	-0.3235109	-0.1456516		3.0
	ZINC20462933	-6.7	353.505	-3.0183	6	1	300.11	0.21675	68.12	6.2194	10109.69635951	0.27412	8.0136	-11.011	0.56	0.65067	0.92342 (max o	4	7727; 7721; 7031; 76	-0.287052	-0.1332794		3.0
	ZINC20465204	-6.7	351.421	-3.2262	7	4	277.33	0.31836	92.46	2.1281	10109.69635951	0.27412	8.2215	-11.769	0.56	0.48458	0.87569 (max o	4	3002; 6579; 6821; 70	0.30458406	-0.1072829		2.0
	ZINC35242178	-6.7	386.360	-3.1619	11	0	278.56	0.5312	171.24	3.2541	10109.69635951	0.26357	8.1572	-11.996	0.46154	0.44595	0.94951 (max o	4	7745; 7432; 7554; 76	0.42919996	-0.3221690		2.0
	ZINC35242172	-6.7	386.360	-3.1619	11	0	278.56	0.5312	171.24	3.2541	10109.69635951	0.26357	8.1572	-11.996	0.46154	0.44595	0.94951 (max o	5	7700; 7648; 6890; 74	0.44845676	-0.293076		2.0
	ZINC40312877	-6.7	372.464	-1.1214	8	2	282.54	0.32272	89.92	4.5684	10109.69635951	0.27412	6.1167	-4.091	0.48	0.49144	0.96488	1	7564	0.5343204	-0.6438277		2.0
	ZINC03812867	-6.7	368.472	-0.8718	7	1	284.68	0.26236	103.35	-6.9075	10109.69635951	0.26357	5.8671	-3.3076	0.5	0.48296	0.84666 (max o	2	3995; 7579	-0.8419453	-0.0250244		2.0
	ZINC03812867	-6.7	367.464	-2.1032	7	1	288.4	0.27188	98.77	-4.981	10109.69635951	0.26357	7.0985	-7.9796	0.5	0.6182	0.81466	1	7578	-0.8202249	-0.0572674		2.0
	ZINC02134184	-6.7	357.413	1.2628	8	0	274.2	0.23549	70.91	4.4146	10109.69635951	0.26357	3.7325	4.7911	0.46154	0.49917	0.84869 (max o	2	6071; 7284	0.01611427	-0.8326988		1.0
	ZINC02134184	-6.7	358.421	-0.715	8	1	263.23	0.20362	72.16	3.6615	10109.69635951	0.26357	5.7103	-2.7127	0.46154	0.49917		0		0.6459764	0.699048		1.0
	ZINC15707026	-6.7	365.428	-1.5416	8	1	272.34	0.27106	105.81	1.1757	10109.69635951	0.26357	6.5369	-5.8489	0.5	0.63463	0.88355 (max o	3	5520; 6317; 7614	-0.2993826	0.4111503		1.0
	ZINC15707026	-6.7	364.420	-1.3527	8	0	283.31	0.29928	104.56	1.6904	10109.69635951	0.26357	6.348	-5.1322	0.5	0.63463	0.87803 (max o	2	6318; 7613	-0.2630815	0.40910965		1.0
	ZINC20463658	-6.7	364.444	-1.3381	8	2	267.7	0.26735	79.2	8.0264	10109.69635951	0.26357	6.3334	-5.0768	0.53846	0.48358	0.85067	1	7385	-0.5676979	-0.349271		1.0
	ZINC20463658	-6.7	365.452	-2.5628	8	2	263.98	0.25703	83.78	7.6637	10109.69635951	0.26357	7.5581	-9.7233	0.53846	0.46119	0.89524 (max o	2	6555; 7211	0.40255457	0.05821638		1.0
	ZINC20464994	-6.7	373.923	-3.197	6	5	313.6	0.26416	70.6	0.68053	10109.69635951	0.27412	8.1923	-11.663	0.56	0.6026		0		0.8056402	-0.587911		1.0
	ZINC20465116	-6.7	355.458	-0.7428	7	3	259.53	0.33969	113.51	9.3057	10109.69635951	0.28554	5.7381	-2.6014	0.54167	0.49395	0.86661	1	6260	-0.4171108	0.2144786		1.0
	ZINC20465116	-6.7	356.465	-2.8525	7	4	273.57	0.37358	114.71	5.6676	10109.69635951	0.28554	7.8478	-9.9899	0.54167	0.49395	0.89253 (max o	2	7792; 6261	-0.4555979	0.21531875		1.0
	ZINC22926977	-6.7	370.584	-1.8972	6	3	310.71	0.27553	76.3	7.9649	10109.69635951	0.27412	6.8925	-6.9212	0.72	0.68166	0.85614	1	7637	0.42115915	-0.6072423		1.0
	ZINC30878870	-6.7	387.539	-0.885	7	3	329.8	0.25831	81.46	-0.95804	10109.69635951	0.25381	5.8803	-3.4868	0.59259	0.6244	0.88896 (max o	4	7932; 7895; 7733; 72	-0.3174082	0.3988105		1.0
	ZINC13514809	-6.7	403.410	-8.3572	12	4	310.8	0.51454	233.87	-0.92135	10109.69635951	0.24475	13.352	-34.146	0.64286	0.65145	0.92152	1	6959	-0.9252863	-0.0879590		0.0
	ZINC04556665	-6.7	357.493	-0.6003	7	3	294.77	0.30804	125.97	3.1822	10109.69635951	0.27412	5.5956	-2.1899	0.52	0.64828	0.9594 (max of	4	7768; 7695; 6722; 74	-0.2167550	0.8024453		0.0

Structure	Molecule Name	affinity	Total Molweight	cLogP	H-Acceptors	H-Donors	Total Surface Area	Relative PSA	Polar Surface Area	Druglikeness	Kd (nM) @ 20°C	LE from K...	LLE from Kd ...	LELP from Kd (n...	Shape Index	Molecular...	Neighbor Si...	Neig...	Neighbor	Neighbor ...	Neighbor ...	Ring Systems	frequency
	ZINC31157909	-6.7	436.456	-2.3414	12	6	333.18	0.4433	198.7	-1.3926	10109.69635951	0.22843	7.3367	-10.25	0.6	0.67052	0.97757 (max o	3	7238; 7544; 7642	0.59045964	0.75765157		0.0
	ZINC31157895	-6.7	436.456	-2.3414	12	6	333.18	0.4433	198.7	-1.3926	10109.69635951	0.22843	7.3367	-10.25	0.6	0.67514	0.97757 (max o	3	7643; 7238; 7544	0.5917389	0.73887646		0.0
	ZINC35464458	-6.7	356.413	-2.0268	7	3	297.45	0.31582	140.95	-11.414	10109.69635951	0.27412	7.0221	-7.3939	0.8	0.70354	0.96832 (max o	3	7739; 7246; 7561	-0.5804281	0.4872671		0.0
	ZINC04629873	-6.7	438.607	0.5269	6	2	375.23	0.26573	153.43	-16.001	10109.69635951	0.22843	4.4684	2.3066	0.7	0.64711	0.95053 (max o	5	7715; 7714; 7712; 77	0.09533175	-0.7213219		0.0
	ZINC08681562	-6.7	434.401	-11.528	13	3	318.32	0.59767	271.66	1.8032	10109.69635951	0.23631	16.523	-48.782	0.51724	0.72551	0.981 (max of 3	3	7785; 7463; 7600	-0.0925365	0.7471775		0.0
	ZINC08681561	-6.7	434.401	-11.528	13	3	318.32	0.59767	271.66	1.8032	10109.69635951	0.23631	16.523	-48.782	0.51724	0.72425	0.981 (max of 3	3	7785; 7463; 7601	-0.0713424	0.77455807		0.0
	ZINC68604154	-6.7	371.500	-9.3237	8	1	322.96	0.25663	94.42	5.3849	10109.69635951	0.26357	14.319	-35.374	0.5	0.61606	0.85242 (max o	2	7662; 7663	0.49879968	-0.6263383		0.0
	ZINC68604154	-6.7	373.516	-8.5103	8	3	351.04	0.31609	96.82	4.07	10109.69635951	0.26357	13.506	-32.288	0.5	0.60672	0.85614 (max o	2	7662; 7663	0.51806664	-0.6130482		0.0
	ZINC68604154	-6.7	372.508	-8.917	8	2	337	0.2876	95.62	4.6999	10109.69635951	0.26357	13.912	-33.831	0.5	0.61649	0.85242 (max o	2	7665; 7664	0.49430576	-0.6470933		0.0
	ZINC68604154	-6.7	372.508	-8.917	8	2	337	0.2876	95.62	4.4823	10109.69635951	0.26357	13.912	-33.831	0.5	0.60629	0.85614 (max o	2	7665; 7664	0.51616305	-0.6344366		0.0
	ZINC71766842	-6.7	353.399	-9.3329	10	4	253.75	0.66353	242.72	-2.3867	10109.69635951	0.3115	14.328	-29.962	0.72727	0.80026	0.92521 (max o	6	7811; 7711; 7709; 77	0.15154894	-0.6650358		0.0
	ZINC71766842	-6.7	352.391	-11.532	10	4	254.54	0.66457	241.1	-0.9741	10109.69635951	0.3115	16.528	-37.023	0.72727	0.81376	0.95174 (max o	4	7810; 7711; 7710; 76	0.18698421	-0.6928757		0.0
	ZINC04215557	-6.7	390.551	-5.57	10	8	308.66	0.35606	186.32	-9.9583	10109.69635951	0.25381	10.565	-21.945	0.85185	0.60183	0.9578	1	7753	0.9007929	-0.0528052		0.0
	ZINC68606635	-6.7	374.305	-10.744	13	1	271.25	0.50352	193.71	4.0623	10109.69635951	0.26357	15.739	-40.764	0.42308	0.59281	0.98517	1	7809	-0.0923352	0.98212886		0.0
	ZINC06019738	-6.6	410.493	-3.1558	9	4	313.25	0.43607	194.36	3.9817	12003.12117742	0.24109	8.0765	-13.09	0.57143	0.59941	0.80862	1	7356	-0.4418462	0.871795		134.0
	ZINC04266061	-6.6	411.497	1.2902	9	2	320.03	0.22004	110.68	-18.445	12003.12117742	0.23278	3.6305	5.5426	0.62069	0.5604	0.89169	1	7913	-0.9202406	0.18913175		134.0
	ZINC35271754	-6.6	410.461	1.5974	8	4	312.4	0.31524	133.52	-11.395	12003.12117742	0.23278	3.3233	6.8623	0.44828	0.60936	0.96428 (max o	5	7936; 7935; 7260; 75	0.43791848	-0.5164563		127.0
	ZINC14781594	-6.6	410.461	1.5974	8	4	312.4	0.31524	133.52	-11.395	12003.12117742	0.23278	3.3233	6.8623	0.44828	0.60755	0.96931 (max o	3	7886; 7509; 7612	0.4301877	-0.4637088		127.0
	ZINC67913938	-6.6	424.441	-1.0613	11	5	315.59	0.41693	172.21	-17.515	12003.12117742	0.23278	5.982	-4.5592	0.55172	0.56882	0.96682 (max o	2	6048; 7660	-0.6383968	0.3561397		120.0
	ZINC49601680	-6.6	365.354	-3.573	10	4	261.9	0.46873	165.81	-8.2227	12003.12117742	0.27002	8.4937	-13.232	0.56	0.60151	0.95 (max of 6)	6	6358; 7100; 7271; 74	-0.6250345	-0.3429751		120.0
	ZINC04097575	-6.6	436.502	-3.8395	11	4	288.25	0.57929	238.9	-8.9899	12003.12117742	0.25964	8.7602	-14.788	0.5	0.60251	0.98956 (max o	7	7752; 7082; 7358; 74	-0.3788412	0.6312518		120.0
	ZINC04097574	-6.6	436.502	-3.8395	11	4	288.25	0.57929	238.9	-8.9899	12003.12117742	0.25964	8.7602	-14.788	0.5	0.60251	0.98956 (max o	7	7752; 7082; 7358; 74	-0.3716623	0.6081059		120.0
	ZINC71404435	-6.6	436.502	-3.8395	11	4	288.25	0.57929	238.9	-8.9899	12003.12117742	0.25964	8.7602	-14.788	0.5	0.60303	0.98956 (max o	5	7082; 7431; 7572; 76	-0.3504198	0.60356265		120.0
	ZINC31162643	-6.6	365.397	-2.6206	9	5	273.11	0.41309	159.74	-12.662	12003.12117742	0.27002	7.5413	-9.705	0.56	0.59452	0.99111 (max o	6	7854; 7742; 7242; 72	-0.1569134	-0.5253473		120.0
	ZINC35457920	-6.6	365.397	-2.6206	9	5	273.11	0.41309	159.74	-12.662	12003.12117742	0.27002	7.5413	-9.705	0.56	0.5861	0.99111 (max o	5	7854; 7243; 7559; 75	-0.1582800	-0.5447726		120.0
	ZINC04353235	-6.6	390.340	-0.1978	11	0	286.31	0.43729	140.73	-0.85962	12003.12117742	0.25002	5.1185	-0.79113	0.40741	0.43144	0.95493 (max o	8	7992; 7955; 7875; 78	0.21983425	0.41821116		120.0
	ZINC04353236	-6.6	390.340	-0.1978	11	0	286.31	0.43729	140.73	-0.85962	12003.12117742	0.25002	5.1185	-0.79113	0.40741	0.43367	0.95493 (max o	10	7955; 7953; 7949; 78	0.23700292	0.3848191		120.0
	ZINC68603036	-6.6	376.341	-1.8443	12	2	247.29	0.44442	131.04	0.14627	12003.12117742	0.25964	6.765	-7.1033	0.42308	0.49087	0.94951 (max o	3	7857; 7858; 7807	-0.1508926	-0.7642046		120.0
	ZINC12655870	-6.6	444.439	-0.1088	13	1	343.44	0.4397	152.42	-2.9238	12003.12117742	0.21776	5.0295	-0.49963	0.48387	0.51545	0.94251 (max o	2	7981; 7965	0.12291067	0.9423909		120.0
	ZINC04215172	-6.6	357.287	-7.4085	12	8	236.1	0.6443	220.43	-3.1947	12003.12117742	0.28128	12.329	-26.339	0.45833	0.5608		0		-0.6633206	0.6740562		120.0
	ZINC04042335	-6.6	384.136	0.9648	5	3	207.7	0.28551	79.15	-3.1395	12003.12117742	0.3553	3.9559	2.7155	0.52632	0.26113	0.91405 (max o	3	6708; 7305; 7458	-0.4505217	-0.8700016		49.0
	ZINC03838447	-6.6	400.474	-1.5645	10	3	314.34	0.36235	140.06	7.5026	12003.12117742	0.24109	6.4852	-6.4892	0.5	0.59897	0.89174 (max o	5	5773; 6933; 7144; 74	-0.7197768	0.51617056		47.0
	ZINC20462923	-6.6	369.548	-1.9855	6	1	320.78	0.20279	68.12	3.485	12003.12117742	0.25964	6.9062	-7.6471	0.53846	0.7466	0.9302 (max of	4	7514; 7524; 7721; 77	-0.2487253	-0.1253270		47.0
	ZINC20462921	-6.6	355.521	-2.4399	6	1	307.02	0.21188	68.12	2.5156	12003.12117742	0.27002	7.3606	-9.0358	0.52	0.74485	0.9302 (max of	3	7724; 7620; 7720	-0.2558495	-0.1494885		47.0
	ZINC20462921	-6.6	355.521	-2.4399	6	1	307.02	0.21188	68.12	1.6882	12003.12117742	0.27002	7.3606	-9.0358	0.52	0.74179	0.9302 (max of	3	7723; 7721; 7722	-0.2673313	-0.1358734		47.0
	ZINC20462923	-6.6	370.556	-1.5788	6	2	334.82	0.23622	69.32	1.1494	12003.12117742	0.25964	6.4995	-6.0807	0.53846	0.72937	0.9302 (max of	2	7722; 7723	-0.2710761	-0.1170446		47.0
	ZINC20462921	-6.6	356.529	-2.0332	6	2	321.06	0.24634	69.32	0.23594	12003.12117742	0.27002	6.9539	-7.5297	0.52	0.72607	0.9302 (max of	2	7725; 7720	-0.2920848	-0.1128134		47.0
	ZINC20462923	-6.6	369.548	-1.9855	6	1	320.78	0.20279	68.12	2.6382	12003.12117742	0.25964	6.9062	-7.6471	0.53846	0.74378	0.9302 (max of	3	7725; 7720; 7724	-0.2364962	-0.1067990		47.0
	ZINC03834173	-6.6	354.095	-0.8884	7	3	182.47	0.42358	99.1	3.3731	12003.12117742	0.3971	5.8091	-2.2372	0.58824	0.49973	0.91652 (max o	4	7769; 7366; 7505; 75	0.41196555	0.36846122		41.0
	ZINC20465342	-6.6	397.445	-0.956	8	3	293.7	0.2649	94.5	4.2693	12003.12117742	0.24109	5.8767	-3.9653	0.60714	0.52898	0.94605 (max o	8	1870; 3003; 3397; 47	-0.3526159	0.12048338		21.0
	ZINC13348010	-6.6	424.401	-4.4371	13	8	290.06	0.54334	207.27	-3.1385	12003.12117742	0.23278	9.3578	-19.061	0.41379	0.46239	0.95642 (max o	4	7707; 7321; 7352; 73	0.48302397	-0.4819771		19.0
	ZINC13348014	-6.6	424.401	-4.4371	13	8	290.06	0.54334	207.27	-3.1385	12003.12117742	0.23278	9.3578	-19.061	0.41379	0.46239	0.95642 (max o	4	7352; 7353; 7480; 77	0.45879897	-0.5139106		19.0
	ZINC00537850	-6.6	354.095	-0.8884	7	3	182.47	0.42358	99.1	3.3731	12003.12117742	0.3971	5.8091	-2.2372	0.58824	0.50202	0.90599 (max o	3	7769; 5711; 7577	0.4544605	0.35004935		17.0

Structure	Molecule Name	affinity	Total Molweight	cLogP	H-Acceptors	H-Donors	Total Surface Area	Relative PSA	Polar Surface Area	Druglikeness	Kd (nM) @ 20°C	LE from K...	LLE from Kd ...	LELP from Kd (n...	Shape Index	Molecular...	Neighbor Si...	Neig...	Neighbor	Neighbor ...	Neighbor ...	Ring Systems	frequency
	ZINC03978028	-6.6	425.996	-1.1969	7	5	318.59	0.34176	128.76	0.91283	12003.12117742	0.25002	6.1176	-4.7872	0.51852	0.47313	0.99202 (max o	11	2403; 3982; 5779; 58	0.71946937	-0.2651309		15.0
	ZINC04556807	-6.6	420.259	-10.197	14	7	262.3	0.67587	251.53	-31.289	12003.12117742	0.25002	15.118	-40.784	0.48148	0.52622	0.95201 (max o	4	7943; 5651; 5791; 73	1.2405906E	-0.5177213		12.0
	ZINC20465342	-6.6	398.453	-3.0657	8	4	307.74	0.29843	95.7	2.2937	12003.12117742	0.24109	7.9864	-12.716	0.60714	0.53401	0.94605 (max o	8	3004; 3398; 3849; 47	-0.3228986	-0.6010206		10.0
	ZINC05664939	-6.6	390.340	-0.2551	11	0	286.31	0.43729	140.73	-0.85962	12003.12117742	0.25002	5.1758	-1.0203	0.40741	0.39925	0.93058 (max o	2	7788; 6977	0.773275	0.31115285		10.0
	ZINC08733250	-6.6	376.044	-1.8403	5	1	160.53	0.35514	80.67	-2.4417	12003.12117742	0.42191	6.761	-4.3618	0.4375	0.2085	0.86186 (max o	2	7962; 1215	-0.4290067	-0.8149347		9.0
	ZINC13549850	-6.6	357.450	-2.7913	7	2	272.19	0.40821	162.3	-1.413	12003.12117742	0.28128	7.712	-9.9237	0.66667	0.65036	0.94034 (max o	4	1569; 7142; 7282; 75	-0.1466708	0.4634875		6.0
	ZINC49180894	-6.6	418.481	-0.2872	9	6	293.11	0.38088	156.91	-5.2779	12003.12117742	0.23278	5.2079	-1.2338	0.37931	0.4602	0.95099 (max o	3	4427; 6034; 7565	-0.5585139	-0.6869853		6.0
	ZINC05398593	-6.6	399.466	-1.7478	10	2	301.66	0.32639	121.86	1.4377	12003.12117742	0.24109	6.6685	-7.2495	0.5	0.55937	0.90934 (max o	5	2465; 3686; 5420; 56	-0.1510485	0.33658978		6.0
	ZINC36369911	-6.6	402.449	-0.8529	9	3	295.73	0.30217	120.86	5.0527	12003.12117742	0.23278	5.7736	-3.664	0.41379	0.49219	0.95372	1	7267	-0.0220468	-0.9765476		5.0
	ZINC03873055	-6.6	375.421	-4.4053	9	4	267.35	0.49766	181.16	-2.7923	12003.12117742	0.27002	9.326	-16.314	0.44	0.65461	0.91611 (max o	4	5344; 5736; 6102; 71	-0.8516905	0.04147528		5.0
	ZINC04025758	-6.6	384.336	-0.9694	10	0	269.14	0.43933	139.34	-6.1644	12003.12117742	0.25002	5.8901	-3.8772	0.40741	0.58847	0.95201 (max o	2	7864; 5863	-0.3141049	-0.8722021		5.0
	ZINC04270837	-6.6	356.398	-0.154	8	0	256.5	0.31875	101.6	-3.9576	12003.12117742	0.28128	5.0747	-0.54751	0.54167	0.56148	0.86201 (max o	2	5418; 6148	0.19522978	0.37373942		4.0
	ZINC12660123	-6.6	351.493	-0.9736	7	2	288.57	0.19974	64.13	-7.5033	12003.12117742	0.27002	5.8943	-3.6056	0.6	0.53377	0.81344	1	7705	-0.4618422	0.7531391		4.0
	ZINC01646906	-6.6	358.215	-0.018	7	1	221.05	0.24017	76.76	-4.1386	12003.12117742	0.32146	4.9387	-0.055995	0.57143	0.48683		0		0.14312208	0.9137398		4.0
	ZINC12660123	-6.6	350.485	1.0042	7	1	299.54	0.22905	62.88	-7.3081	12003.12117742	0.27002	3.9165	3.7189	0.6	0.53377	0.81344	1	7704	-0.4446562	0.74328196		4.0
	ZINC01646906	-6.6	357.207	1.9598	7	0	232.02	0.2761	75.51	-3.8269	12003.12117742	0.32146	2.9609	6.0966	0.57143	0.48683		0		-0.9637269	0.03198712		4.0
	ZINC04062076	-6.6	378.376	1.2887	8	1	277.82	0.34288	116.2	-3.9534	12003.12117742	0.25002	3.632	5.1543	0.44444	0.50718	0.98806 (max o	3	7307; 7585; 7683	-0.5845391	0.27703583		4.0
	ZINC04062074	-6.6	378.376	1.2887	8	1	277.82	0.34288	116.2	-3.9534	12003.12117742	0.25002	3.632	5.1543	0.44444	0.50718	0.98806 (max o	3	7307; 7684; 7585	-0.5690740	0.29236668		4.0
	ZINC35882823	-6.6	371.301	-1.2117	12	1	262.31	0.50215	149.9	5.0025	12003.12117742	0.25964	6.1324	-4.6669	0.46154	0.45362	0.95857 (max o	5	7745; 6651; 6889; 74	0.37828016	-0.3351618		3.0
	ZINC27642636	-6.6	382.407	-0.3469	8	2	264.11	0.37837	114.82	1.115	12003.12117742	0.25002	5.2676	-1.3875	0.37037	0.29503	0.98371 (max o	5	7797; 6600; 6850; 74	-0.0771536	0.8972281		3.0
	ZINC04085357	-6.6	363.434	-2.185	8	1	241.08	0.47486	157.36	6.261	12003.12117742	0.29351	7.1057	-7.4445	0.47826	0.41212	0.83367	1	2159	-0.1871706	0.9233489		3.0
	ZINC20462936	-6.6	367.532	-2.6763	6	1	313.87	0.20725	68.12	4.4361	12003.12117742	0.25964	7.597	-10.308	0.53846	0.62929	0.92342 (max o	4	6845; 7031; 7620; 77	-0.3027402	-0.1487117		3.0
	ZINC20462936	-6.6	368.540	-2.2696	6	2	327.91	0.24119	69.32	2.1444	12003.12117742	0.25964	7.1903	-8.7414	0.53846	0.6114	0.89162 (max o	3	7033; 7621; 7726	-0.3647535	-0.1357024		3.0
	ZINC20462936	-6.6	367.532	-2.6763	6	1	313.87	0.20725	68.12	3.6065	12003.12117742	0.25964	7.597	-10.308	0.53846	0.62637	0.90243 (max o	4	7728; 7032; 7619; 77	-0.3388423	-0.1609729		3.0
	ZINC01189225	-6.6	402.398	-1.9901	8	0	313.9	0.3015	117.18	-1.2482	12003.12117742	0.23278	6.9108	-8.5493	0.44828	0.56037		0		0.6484105	0.75911546		2.0
	ZINC35882823	-6.6	370.293	-3.3813	12	0	263.6	0.50459	156.22	3.7857	12003.12117742	0.25964	8.302	-13.023	0.46154	0.44595	0.95559 (max o	5	6652; 6724; 6890; 76	0.4139549	-0.361422		2.0
	ZINC20113205	-6.6	361.349	-3.5814	8	0	271.85	0.33559	119.03	3.0964	12003.12117742	0.25964	8.5021	-13.794	0.5	0.51531	0.91351 (max o	2	7198; 7205	0.90800947	0.07044243		2.0
	ZINC12659904	-6.6	441.388	-0.0395	12	1	324.96	0.43319	156.67	2.0065	12003.12117742	0.21776	4.9602	-0.18139	0.41935	0.40654	0.96018 (max o	5	7781; 6440; 6964; 73	0.3125452	0.37486088		2.0
	ZINC30878801	-6.6	374.476	-0.8389	8	1	298.34	0.24549	80.7	3.7046	12003.12117742	0.25964	5.7596	-3.231	0.57692	0.65363	0.90599 (max o	3	7931; 7894; 2620	-0.316725	0.31036723		2.0
	ZINC30878801	-6.6	375.484	-2.2052	8	2	312.38	0.2794	81.9	3.3222	12003.12117742	0.25964	7.1259	-8.4933	0.57692	0.65499	0.90599 (max o	4	7932; 7895; 2621; 76	-0.3189841	0.37659168		2.0
	ZINC03838387	-6.6	365.452	-1.4738	8	3	283.92	0.30692	118.34	4.1822	12003.12117742	0.25964	6.3945	-5.6763	0.5	0.52208		0		0.56595343	0.8071874		1.0
	ZINC03838446	-6.6	399.442	-1.2493	10	1	303.92	0.3314	114.48	4.389	12003.12117742	0.24109	6.17	-5.1818	0.5	0.55445	0.82392	1	7676	-0.7426363	0.49997663		1.0
	ZINC06143106	-6.6	387.368	-0.8512	11	1	277.27	0.52902	164.92	4.1169	12003.12117742	0.25964	5.7719	-3.2784	0.46154	0.45362	0.97269 (max o	6	7744; 6651; 6889; 68	0.41062284	-0.3204207		1.0
	ZINC20462918	-6.6	361.484	-0.0141	6	2	308.49	0.23651	76.91	3.1437	12003.12117742	0.25964	4.9348	-0.054305	0.53846	0.62914	0.94951 (max o	2	5045; 5183	-0.1049630	-0.1870194		1.0
	ZINC30878813	-6.6	364.464	-1.603	7	2	297.36	0.26315	76.33	3.3416	12003.12117742	0.25964	6.5237	-6.174	0.61538	0.58069		0		-0.7476098	-0.6481116		1.0
	ZINC04095672	-6.6	427.568	-0.0252	10	6	336.81	0.33289	168.01	-3.5735	12003.12117742	0.22502	4.9459	-0.11199	0.53333	0.67107	0.97533 (max o	6	1567; 1568; 6967; 71	-0.3410169	0.6369748		0.0
	ZINC04027430	-6.6	358.413	-1.7779	9	3	283.96	0.40368	150.65	-57.945	12003.12117742	0.27002	6.6986	-6.5842	0.52	0.65159	0.97118 (max o	5	7763; 6451; 6739; 69	-0.2387333	0.8071593		0.0
	ZINC31167436	-6.6	356.413	-2.0268	7	3	297.45	0.31582	140.95	-11.414	12003.12117742	0.27002	6.9475	-7.506	0.8	0.69965	0.97118 (max o	3	7246; 7561; 7650	-0.6028977	0.47546548		0.0
	ZINC13549779	-6.6	438.607	0.5269	6	2	375.23	0.26573	153.43	-16.001	12003.12117742	0.22502	4.3938	2.3416	0.7	0.66582	0.9707 (max of	5	7150; 7596; 7712; 77	0.12155987	-0.7471955		0.0
	ZINC13549773	-6.6	438.607	0.5269	6	2	375.23	0.26573	153.43	-16.001	12003.12117742	0.22502	4.3938	2.3416	0.7	0.6994	0.9707 (max of	5	7715; 7150; 7596; 77	0.113415	-0.708204		0.0
	ZINC13540466	-6.6	438.607	0.5269	6	2	375.23	0.26573	153.43	-16.001	12003.12117742	0.22502	4.3938	2.3416	0.7	0.66539	0.9707 (max of	5	7715; 7714; 7150; 75	0.1006707	-0.7398254		0.0
	ZINC13540464	-6.6	438.607	0.5269	6	2	375.23	0.26573	153.43	-16.001	12003.12117742	0.22502	4.3938	2.3416	0.7	0.69899	0.9707 (max of	5	7715; 7714; 7150; 75	0.1331292	-0.7160959		0.0
	ZINC04556668	-6.6	357.493	-0.6003	7	3	294.77	0.30804	125.97	3.1822	12003.12117742	0.27002	5.521	-2.2231	0.52	0.65144	0.9594 (max of	3	7768; 7482; 7595	-0.2139393	0.7828184		0.0

Structure	Molecule Name	affinity	Total Molweight	cLogP	H-Acceptors	H-Donors	Total Surface Area	Relative PSA	Polar Surface Area	Druglikeness	Kd (nM) @ 20°C	LE from K...	LLE from Kd ...	LELP from Kd (n...	Shape Index	Molecular...	Neighbor Si...	Neig...	Neighbor	Neighbor ...	Neighbor ...	Ring Systems	frequency
	ZINC31160734	-6.6	354.441	-0.6502	6	1	298.93	0.26006	109.72	-4.9275	12003.12117742	0.27002	5.5709	-2.4079	0.64	0.70073	0.97932 (max o	7	7957; 7933; 7799; 77	0.00202993	0.72466934		0.0
	ZINC35464597	-6.6	400.494	-1.8098	9	4	325.36	0.35014	147.66	3.0018	12003.12117742	0.24109	6.7305	-7.5066	0.60714	0.66873	0.97288 (max o	3	7803; 7551; 7740	-0.8714705	0.25542048		0.0
	ZINC31167607	-6.6	400.494	-1.8098	9	4	325.36	0.35014	147.66	3.0018	12003.12117742	0.24109	6.7305	-7.5066	0.60714	0.6683	0.97288 (max o	3	7803; 7743; 7551	-0.8833544	0.2726545		0.0
	ZINC13508230	-6.6	353.399	-9.3329	10	4	253.75	0.66353	242.72	-2.3867	12003.12117742	0.30685	14.254	-30.416	0.72727	0.80048	0.95532 (max o	6	7811; 7667; 7668; 77	0.1503469	-0.6863619		0.0
	ZINC13508230	-6.6	354.407	-7.1334	10	4	252.96	0.66248	244.34	-0.91441	12003.12117742	0.30685	12.054	-23.247	0.72727	0.78657	1 (max of 4)	4	7811; 7711; 7668; 77	0.16773602	-0.6993042		0.0
	ZINC13508230	-6.6	354.407	-7.1334	10	4	252.96	0.66248	244.34	-0.91441	12003.12117742	0.30685	12.054	-23.247	0.72727	0.78657	1 (max of 4)	4	7811; 7711; 7709; 76	0.17267565	-0.6583508		0.0
	ZINC13508230	-6.6	352.391	-11.532	10	4	254.54	0.66457	241.1	-0.9741	12003.12117742	0.30685	16.453	-37.584	0.72727	0.82225	0.95174 (max o	5	7811; 7810; 7711; 76	0.1697943	-0.6781403		0.0
	ZINC31156846	-6.6	431.568	1.0024	8	4	358.19	0.29074	154.76	-19.845	12003.12117742	0.22502	3.9183	4.4547	0.7	0.62683	1	1	7735	0.3801276	0.62720305		0.0
	ZINC31156841	-6.6	431.568	1.0024	8	4	358.19	0.29074	154.76	-19.845	12003.12117742	0.22502	3.9183	4.4547	0.7	0.6271	1	1	7736	0.37447983	0.60740316		0.0
	ZINC04096078	-6.6	371.412	-3.5992	10	4	297.2	0.42167	156.53	-3.5653	12003.12117742	0.25964	8.5199	-13.862	0.61538	0.64335		0		0.08669537	-0.9355331		0.0
	ZINC03873037	-6.6	362.426	-5.3652	9	3	275.63	0.51058	204.9	-5.6215	12003.12117742	0.28128	10.286	-19.075	0.58333	0.65209		0		0.5096143	-0.7365646		0.0
	ZINC68606252	-6.6	354.541	-3.7317	7	3	334.42	0.25163	82.24	-5.0435	12003.12117742	0.27002	8.6524	-13.82	0.64	0.57074		0		-0.4211009	0.88044465		0.0
	ZINC31165085	-6.5	350.278	-4.1042	9	1	263.19	0.43246	153.09	-6.0092	14251.16174377	0.26593	8.9503	-15.433	0.6	0.54738	0.96832 (max o	7	7897; 5212; 5978; 59	0.8211727	0.09199847		134.0
	ZINC31167860	-6.5	352.362	-1.4927	8	3	270.67	0.37337	135.99	2.2083	14251.16174377	0.26593	6.3388	-5.6131	0.64	0.66553	0.98434 (max o	8	6297; 6898; 7076; 72	0.37316212	0.38875052		134.0
	ZINC03874236	-6.5	409.549	-0.809	7	3	324	0.33423	151.27	3.1065	14251.16174377	0.23744	5.6551	-3.4072	0.57143	0.59858	0.96519 (max o	4	6942; 7126; 7297; 74	0.48121208	0.6516427		134.0
	ZINC03861723	-6.5	432.974	-0.8972	4	2	191.44	0.28677	88	-0.74224	14251.16174377	0.44322	5.7433	-2.0243	0.6	0.57821	0.87063	1	7987	-0.7224699	0.5914322		134.0
	ZINC12886814	-6.5	401.570	-0.9968	7	3	318.28	0.34024	151.27	3.5206	14251.16174377	0.24623	5.8429	-4.0482	0.66667	0.60844	0.94302 (max o	8	6475; 6711; 6746; 67	0.5713558	5.8947527E-		127.0
	ZINC04089885	-6.5	419.609	-1.5203	7	3	324.77	0.38729	176.57	2.3408	14251.16174377	0.24623	6.3664	-6.1742	0.7037	0.61468	0.96885 (max o	15	5755; 6712; 6952; 69	0.55710256	0.09271345		127.0
	ZINC08740156	-6.5	372.305	-3.3929	11	0	273.45	0.47541	161.28	-5.5779	14251.16174377	0.25571	8.2391	-13.269	0.42308	0.47687	0.89524	1	7132	-0.505377	0.83350545		120.0
	ZINC13425098	-6.5	390.340	-0.1978	11	0	286.31	0.43729	140.73	-0.85962	14251.16174377	0.24623	5.0439	-0.8033	0.40741	0.42936	0.95493 (max o	7	7918; 7917; 7485; 74	0.25740403	0.39461714		120.0
	ZINC13425099	-6.5	390.340	-0.1978	11	0	286.31	0.43729	140.73	-0.85962	14251.16174377	0.24623	5.0439	-0.8033	0.40741	0.42711	0.95493 (max o	6	7837; 7832; 7823; 76	0.2206867	0.39833882		120.0
	ZINC04141140	-6.5	361.346	-0.7287	10	1	270.02	0.42078	126.46	-1.6283	14251.16174377	0.26593	5.5748	-2.7402	0.44	0.46449	0.94678 (max o	5	7940; 7877; 7820; 78	0.23573321	0.45521772		120.0
	ZINC68603028	-6.5	376.341	-1.8443	12	2	247.29	0.44442	131.04	0.14627	14251.16174377	0.25571	6.6904	-7.2126	0.42308	0.49087	0.94951 (max o	3	7857; 7858; 7750	-0.1301669	-0.7582751		120.0
	ZINC13473519	-6.5	379.652	-6.8009	12	3	239.07	0.61417	201.54	-28.319	14251.16174377	0.27701	11.647	-24.551	0.5	0.40689	0.87695 (max o	3	3316; 3917; 6107	-0.3623437	-0.8949838		67.0
	ZINC03861761	-6.5	438.169	0.0808	9	1	249.79	0.38901	111.24	3.9348	14251.16174377	0.28906	4.7653	0.27953	0.52174	0.47871	0.92193 (max o	3	3939; 6083; 6544	0.7076187	0.53575325		41.0
	ZINC08636303	-6.5	352.410	0.246	7	3	251.96	0.40582	123.35	0.047591	14251.16174377	0.27701	4.6001	0.88804	0.5	0.43685	0.90599 (max o	5	5022; 6987; 6988; 71	0.41273263	0.1754639		18.0
	ZINC05223557	-6.5	354.095	-0.8884	7	3	182.47	0.42358	99.1	3.3731	14251.16174377	0.39108	5.7345	-2.2717	0.58824	0.5007	0.91652 (max o	3	5711; 7669; 7673	0.43818203	0.3388934		17.0
	ZINC03839107	-6.5	373.476	-1.485	9	3	282.11	0.40743	150.14	9.6065	14251.16174377	0.26593	6.3311	-5.5841	0.48	0.64912	0.91611 (max o	5	7816; 7296; 7317; 74	-0.0315948	-0.0262056		13.0
	ZINC14416998	-6.5	390.340	-0.2551	11	0	286.31	0.43729	140.73	-0.85962	14251.16174377	0.24623	5.1012	-1.036	0.40741	0.39925	0.93058 (max o	3	6977; 7506; 7698	0.7911647	0.2979913		10.0
	ZINC12496256	-6.5	370.349	-3.6021	11	6	251.64	0.51105	167.53	-12.378	14251.16174377	0.26593	8.4482	-13.545	0.52	0.46743	0.8509 (max of	2	4648; 7603	-0.1519257	-0.9691685		7.0
	ZINC05398494	-6.5	378.448	-0.968	10	2	275.43	0.3911	133.5	3.6843	14251.16174377	0.26593	5.8141	-3.64	0.48	0.63259	0.88969 (max o	2	7157; 7488	-0.1109586	0.39905304		5.0
	ZINC03839088	-6.5	399.514	-0.7756	9	3	296.37	0.38783	150.14	8.8534	14251.16174377	0.24623	5.6217	-3.1498	0.44444	0.54438	0.92418 (max o	3	7755; 6700; 7580	-0.0329763	-0.0677609		5.0
	ZINC04024211	-6.5	383.372	-5.5025	9	1	279.03	0.42325	166.92	-4.6692	14251.16174377	0.24623	10.349	-22.347	0.40741	0.58806	0.98371 (max o	3	6513; 7181; 7584	-0.0216481	-0.6997204		4.0
	ZINC35464281	-6.5	357.334	-5.9476	9	1	269.19	0.43872	166.92	-6.2936	14251.16174377	0.26593	10.794	-22.365	0.64	0.51518	0.90535 (max o	3	6646; 6647; 7090	0.8433303	0.14622779		4.0
	ZINC35458433	-6.5	418.485	-2.7648	9	3	299.04	0.40362	164.89	-4.0506	14251.16174377	0.23744	7.611	-11.644	0.53571	0.54332	0.98866 (max o	3	7855; 7264; 7438	-0.5273520	0.82471204		4.0
	ZINC68606233	-6.5	354.561	-6.2724	6	4	312.6	0.21571	85.05	-0.87649	14251.16174377	0.26593	11.119	-23.586	0.6	0.62736	0.98897 (max o	3	7902; 7861; 7860	-0.0462754	-0.7271518		4.0
	ZINC31161975	-6.5	382.407	-0.3469	8	2	264.11	0.37837	114.82	1.0254	14251.16174377	0.24623	5.193	-1.4088	0.37037	0.29852	0.98371 (max o	5	6600; 6850; 7406; 75	-0.0828639	0.8632456		3.0
	ZINC12602282	-6.5	399.646	-2.768	6	4	352.27	0.28288	77.5	1.7859	14251.16174377	0.24623	7.6141	-11.241	0.66667	0.71839	0.91204 (max o	2	5957; 7178	0.43731052	-0.6422938		3.0
	ZINC04025194	-6.5	364.349	1.4871	8	1	258.07	0.35734	108.36	-3.926	14251.16174377	0.25571	3.359	5.8157	0.38462	0.42992	0.95559 (max o	3	6414; 7454; 7468	-0.5228874	0.25863257		2.0
	ZINC12659905	-6.5	441.388	-0.0395	12	1	324.96	0.43319	156.67	2.0065	14251.16174377	0.21446	4.8856	-0.18418	0.41935	0.40371	0.96018 (max o	6	7874; 6440; 6964; 73	0.30002433	0.41280416		2.0
	ZINC13537961	-6.5	384.333	-4.1256	12	7	252.65	0.57289	195.6	-1.7541	14251.16174377	0.25571	8.9717	-16.134	0.5	0.50906	0.89979	1	2961	0.93310994	-0.0627011		2.0
	ZINC03844452	-6.5	432.424	-2.4447	9	0	343.66	0.30449	126.41	-12.899	14251.16174377	0.21446	7.2908	-11.399	0.74194	0.62168	0.97817	1	6938	-0.9176675	-0.0236588		1.0
	ZINC04416789	-6.5	382.364	-7.2288	9	0	291.05	0.42027	177.59	-2.6692	14251.16174377	0.24623	12.075	-29.357	0.40741	0.76293		0		0.04697249	0.9784129		1.0

Structure	Molecule Name	affinity	Total Molweight	cLogP	H-Acceptors	H-Donors	Total Surface Area	Relative PSA	Polar Surface Area	Druglikeness	Kd (nM) @ 20°C	LE from K...	LLE from Kd ...	LELP from Kd (n...	Shape Index	Molecular...	Neighbor Si...	Neig...	Neighbor	Neighbor ...	Neighbor ...	Ring Systems	frequency
	ZINC05599818	-6.5	356.466	0.3411	6	2	238.11	0.39629	131.68	6.4707	14251.16174377	0.28906	4.505	1.18	0.3913	0.33877	0.83219	1	7006	0.20662525	-0.9512870		1.0
	ZINC05699864	-6.5	382.320	-4.9132	10	0	269.54	0.44787	151.32	-2.0093	14251.16174377	0.24623	9.7593	-19.953	0.40741	0.56124		0		-0.7180663	-0.6717462		1.0
	ZINC05732103	-6.5	390.395	0.3794	10	8	289.76	0.42904	163.18	3.211	14251.16174377	0.23744	4.4668	1.5979	0.64286	0.60974		0		-0.9121578	-0.3303314		1.0
	ZINC12602282	-6.5	399.646	-2.768	6	4	352.27	0.28288	77.5	0.22078	14251.16174377	0.24623	7.6141	-11.241	0.66667	0.69497	0.85214	1	7780	0.70324796	-0.6343574		1.0
	ZINC12602282	-6.5	398.638	-0.6583	6	3	338.23	0.25311	76.3	2.3112	14251.16174377	0.24623	5.5044	-2.6735	0.66667	0.69248	0.86162 (max o	2	5573; 7779	0.7105513	-0.6132763		1.0
	ZINC20463648	-6.5	380.507	-1.7234	7	3	284.62	0.29004	89.46	6.4149	14251.16174377	0.24623	6.5695	-6.999	0.48148	0.50414	0.82174	1	7382	-0.8595664	-0.4514355		1.0
	ZINC20463648	-6.5	380.507	-5.9091	7	3	299.84	0.32607	93.49	2.5793	14251.16174377	0.24623	10.755	-23.998	0.48148	0.50765	0.81091	1	7383	0.91106814	-0.2622863		1.0
	ZINC20463726	-6.5	358.413	0.048	6	2	262.39	0.23187	73.76	-0.20384	14251.16174377	0.25571	4.7981	0.18772	0.53846	0.40333	0.87218	1	2985	0.21185051	0.90437144		1.0
	ZINC20464929	-6.5	358.481	-2.4877	7	4	284.07	0.35977	114.71	6.4034	14251.16174377	0.27701	7.3338	-8.9804	0.54167	0.58413	0.89253 (max o	2	7046; 7634	-0.4968937	0.23255666		1.0
	ZINC04027431	-6.5	358.413	-1.7779	9	3	283.96	0.40368	150.65	-57.945	14251.16174377	0.26593	6.624	-6.6855	0.52	0.65159	0.97118 (max o	5	6451; 6739; 6971; 73	-0.2405236	0.82559174		0.0
	ZINC31166301	-6.5	354.441	-0.6237	6	2	296.78	0.27239	120.72	-4.8261	14251.16174377	0.26593	5.4698	-2.3453	0.68	0.69491	0.97932 (max o	9	7957; 7933; 6859; 70	0.02682594	0.7155932		0.0
	ZINC13451235	-6.5	420.374	-11.982	13	3	304.56	0.62467	271.66	1.7888	14251.16174377	0.23744	16.828	-50.463	0.5	0.73193	0.98232 (max o	3	7463; 7600; 7601	-0.0743996	0.7353284		0.0
	ZINC04556667	-6.5	357.493	-0.6003	7	3	294.77	0.30804	125.97	3.1822	14251.16174377	0.26593	5.4464	-2.2573	0.52	0.64957	0.9594 (max of	3	7482; 7595; 7695	-0.2336866	0.7883932		0.0
	ZINC03775146	-6.5	390.551	-5.57	10	8	308.66	0.35606	186.32	-9.9583	14251.16174377	0.24623	10.416	-22.621	0.85185	0.60183	0.9578	1	7591	0.9193764	-0.0478365		0.0
	ZINC68606631	-6.5	374.305	-10.744	13	1	271.25	0.50352	193.71	4.0623	14251.16174377	0.25571	15.59	-42.018	0.42308	0.58895	0.98517	1	7666	-0.0966737	0.9879214		0.0
	ZINC35464600	-6.5	400.494	-1.8098	9	4	325.36	0.35014	147.66	3.0018	14251.16174377	0.23744	6.6559	-7.6221	0.60714	0.66629	0.97533 (max o	3	7551; 7740; 7743	-0.9010052	0.26087794		0.0
	ZINC31160726	-6.5	354.441	-0.6502	6	1	298.93	0.26006	109.72	-4.9275	14251.16174377	0.26593	5.4963	-2.445	0.64	0.70073	0.97932 (max o	7	7957; 7933; 7799; 74	0.01078564	0.70600873		0.0
	ZINC71766843	-6.5	353.399	-9.3329	10	4	253.75	0.66353	242.72	-2.3867	14251.16174377	0.3022	14.179	-30.883	0.72727	0.80923	0.95532 (max o	6	7668; 7708; 7709; 77	0.19009219	-0.6718045		0.0
	ZINC71766843	-6.5	352.391	-11.532	10	4	254.54	0.66457	241.1	-0.9741	14251.16174377	0.3022	16.379	-38.162	0.72727	0.83057	0.95174 (max o	3	7811; 7667; 7710	0.20669031	-0.6847074		0.0
	ZINC04534315	-6.5	362.426	-5.2294	9	3	281.28	0.48212	191.4	-1.8111	14251.16174377	0.27701	10.076	-18.878	0.58333	0.70898	0.97668 (max o	9	7915; 7876; 7869; 78	0.29639164	-0.9268463		0.0
	ZINC60578435	-6.5	381.453	-8.6143	10	4	275.75	0.61059	242.72	-0.31497	14251.16174377	0.27701	13.46	-31.097	0.66667	0.78515	0.88866 (max o	2	7804; 7805	-0.2092151	0.9189446		0.0
	ZINC60578435	-6.5	382.461	-6.4148	10	4	274.96	0.60947	244.34	1.235	14251.16174377	0.27701	11.261	-23.157	0.66667	0.77009	0.88866	1	7806	-0.1948271	0.9054381		0.0
	ZINC60578435	-6.5	380.445	-10.814	10	4	276.54	0.6117	241.1	1.0895	14251.16174377	0.27701	15.66	-39.037	0.66667	0.79975	0.88866	1	7806	-0.1815593	0.9403977		0.0
	ZINC05782523	-6.5	401.542	0.9623	7	4	336.36	0.27988	145.53	-22.292	14251.16174377	0.23744	3.8838	4.0528	0.75	0.65339	0.9678 (max of	5	7909; 7881; 7880; 78	0.73867357	0.4830946		0.0
	ZINC03861663	-6.5	378.333	-13.819	12	2	320.9	0.5125	187.86	-13.133	14251.16174377	0.25571	18.665	-54.043	0.61538	0.80128	0.8541	1	7845	0.45858708	-0.8055102		0.0
	ZINC22016981	-6.5	403.454	-13.842	11	3	341.35	0.40234	155.98	-9.6447	14251.16174377	0.23744	18.688	-58.295	0.46429	0.61802	0.99059 (max o	5	7923; 7922; 7893; 78	0.17976584	0.80310696		0.0
	ZINC22016981	-6.5	403.454	-13.842	11	3	341.35	0.40234	155.98	-9.2655	14251.16174377	0.23744	18.688	-58.295	0.46429	0.64236	0.99059 (max o	5	7923; 7922; 7893; 78	0.2092171	0.8297995		0.0
	ZINC22016981	-6.5	404.462	-13.435	11	4	355.39	0.42595	157.18	-9.6447	14251.16174377	0.23744	18.281	-56.583	0.46429	0.60865	0.99059	1	7921	-0.8348375	0.47008705		0.0
	ZINC03843503	-6.4	369.008	-1.2565	5	3	211.12	0.33682	85.03	-3.5635	16920.23333308	0.38506	6.0281	-3.2631	0.52941	0.50195	0.82414	1	7865	0.36577833	-0.7928588		134.0
	ZINC71789369	-6.4	415.052	-19.03	15	4	239.36	0.85587	304.55	-31.704	16920.23333308	0.27275	23.802	-69.77	0.375	0.54974	0.94034 (max o	19	7930; 7885; 5092; 50	0.38008854	0.74190825		127.0
	ZINC71789369	-6.4	415.052	-19.03	15	4	239.36	0.85587	304.55	-31.704	16920.23333308	0.27275	23.802	-69.77	0.375	0.54974	0.95709 (max o	19	7930; 7885; 7884; 78	0.37175956	0.7267103		127.0
	ZINC14556041	-6.4	415.052	-19.03	15	4	239.36	0.85587	304.55	-31.778	16920.23333308	0.27275	23.802	-69.77	0.375	0.55758	0.95709 (max o	24	7929; 7884; 7883; 50	0.308108	0.806467		127.0
	ZINC14556041	-6.4	414.044	-20.278	15	3	240.54	0.85657	307.38	-20.56	16920.23333308	0.27275	25.049	-74.344	0.375	0.58345	0.95709 (max o	10	7928; 7882; 5091; 65	0.37497637	0.8566758		127.0
	ZINC14556041	-6.4	415.052	-19.03	15	4	239.36	0.85587	304.55	-31.778	16920.23333308	0.27275	23.802	-69.77	0.375	0.54974	0.92619 (max o	16	7930; 7885; 7884; 78	0.39119986	0.7235402		127.0
	ZINC35457914	-6.4	365.397	-2.6206	9	5	273.11	0.41309	159.74	-12.662	16920.23333308	0.26184	7.3922	-10.008	0.56	0.58714	0.99111 (max o	5	7243; 7559; 7560; 77	-0.1745466	-0.5133517		120.0
	ZINC05249463	-6.4	389.356	-0.672	11	1	287.77	0.44014	143.53	-0.087423	16920.23333308	0.24245	5.4436	-2.7717	0.40741	0.44763	0.95201 (max o	6	7940; 7874; 7485; 74	0.25846285	0.45631337		120.0
	ZINC04353233	-6.4	390.340	-0.1978	11	0	286.31	0.43729	140.73	-0.85962	16920.23333308	0.24245	4.9694	-0.81585	0.40741	0.43144	0.94905 (max o	4	7875; 7693; 7694; 77	0.19869828	0.41323858		120.0
	ZINC14515460	-6.4	376.313	-0.6041	11	0	272.55	0.45937	140.73	-2.6337	16920.23333308	0.25177	5.3757	-2.3994	0.38462	0.38122	0.94951 (max o	3	7917; 7611; 7784	0.17811646	0.41371208		120.0
	ZINC68603031	-6.4	376.341	-1.8443	12	2	247.29	0.44442	131.04	0.14627	16920.23333308	0.25177	6.6159	-7.3253	0.42308	0.48874	0.95559 (max o	3	7750; 7807; 7857	-0.1479703	-0.7453530		120.0
	ZINC68603023	-6.4	376.341	-1.8443	12	2	247.29	0.44442	131.04	0.14627	16920.23333308	0.25177	6.6159	-7.3253	0.42308	0.48874	0.95559 (max o	3	7750; 7807; 7858	-0.1683966	-0.7505448		120.0
	ZINC04141139	-6.4	361.346	-0.7287	10	1	270.02	0.42078	126.46	-1.6283	16920.23333308	0.26184	5.5003	-2.783	0.44	0.46449	0.96243 (max o	7	7984; 7952; 7940; 78	0.21325953	0.45437315		120.0
	ZINC04141141	-6.4	361.346	-0.7287	10	1	270.02	0.42078	126.46	-1.6283	16920.23333308	0.26184	5.5003	-2.783	0.44	0.46449	0.96243 (max o	7	7984; 7952; 7940; 78	0.24558663	0.47362724		120.0
	ZINC14766256	-6.4	406.407	0.0381	10	0	293.8	0.45163	156.8	1.9111	16920.23333308	0.24245	4.7335	0.15715	0.40741	0.42686	0.95493 (max o	3	7992; 7918; 7784	0.20543112	0.4344699		120.0

Structure	Molecule Name	affinity	Total Molweight	cLogP	H-Acceptors	H-Donors	Total Surface Area	Relative PSA	Polar Surface Area	Druglikeness	Kd (nM) @ 20°C	LE from K...	LLE from Kd ...	LELP from Kd (n...	Shape Index	Molecular...	Neighbor Si...	Neig...	Neighbor	Neighbor ...	Neighbor ...	Ring Systems	frequency
	ZINC39563706	-6.4	431.576	-1.8848	9	3	330.55	0.34446	146.72	4.3141	16920.23333308	0.22573	6.6564	-8.3499	0.48276	0.59889	0.89631 (max o	2	5653; 7476	-0.1930596	-0.8994559		47.0
	ZINC02123581	-6.4	359.489	-1.8021	7	2	277.1	0.36225	142.48	4.8244	16920.23333308	0.27275	6.5737	-6.6071	0.625	0.57489	0.94718 (max o	5	6949; 7001; 7118; 71	0.796585	-0.4075093		47.0
	ZINC04216113	-6.4	372.085	-1.0394	7	3	187.56	0.41208	99.1	2.4733	16920.23333308	0.36367	5.811	-2.8581	0.55556	0.50454	0.85572	1	7116	0.5083635	0.8475193		41.0
	ZINC19312955	-6.4	353.437	-0.8325	7	1	271.24	0.2959	77.35	-6.5494	16920.23333308	0.26184	5.6041	-3.1794	0.44	0.58452		0		0.7669604	0.64057505		23.0
	ZINC17174211	-6.4	353.111	-1.3798	7	3	184.75	0.43069	108.38	2.1634	16920.23333308	0.38506	6.1514	-3.5833	0.58824	0.49197	0.90064 (max o	5	7841; 7187; 7481; 76	-0.4492210	-0.0442420		20.0
	ZINC17174212	-6.4	353.111	-1.3798	7	3	184.75	0.43069	108.38	2.1634	16920.23333308	0.38506	6.1514	-3.5833	0.58824	0.49063	0.91652 (max o	5	7887; 7609; 7615; 78	-0.4294614	-0.0383599		20.0
	ZINC04556586	-6.4	353.111	-1.3798	7	3	184.75	0.43069	108.38	2.1634	16920.23333308	0.38506	6.1514	-3.5833	0.58824	0.49197	0.90599 (max o	4	7841; 7840; 7609; 78	-0.42713	-0.0186615		20.0
	ZINC13348082	-6.4	383.349	-4.4475	12	8	254.94	0.57347	198.4	-7.3097	16920.23333308	0.25177	9.2191	-17.665	0.42308	0.46124	0.95559 (max o	9	7912; 7911; 7878; 65	0.48690298	-0.4611814		19.0
	ZINC00537842	-6.4	353.111	-1.3798	7	3	184.75	0.43069	108.38	2.1634	16920.23333308	0.38506	6.1514	-3.5833	0.58824	0.49295	0.90599 (max o	4	7887; 7826; 7187; 74	-0.4568451	-0.0064734		14.0
	ZINC03839119	-6.4	371.461	-1.5754	9	3	283.39	0.40559	150.14	6.2388	16920.23333308	0.26184	6.347	-6.0166	0.52	0.67074	0.91611 (max o	6	6935; 7294; 7318; 75	-0.0109955	-0.0362552		13.0
	ZINC31168978	-6.4	373.420	-1.7731	8	4	278.5	0.35806	139.51	-9.7968	16920.23333308	0.25177	6.5447	-7.0425	0.5	0.51929	0.9464 (max of	4	6300; 6865; 7092; 72	0.41576776	0.6205745		8.0
	ZINC13549364	-6.4	383.349	-4.4475	12	8	254.94	0.57347	198.4	-7.3097	16920.23333308	0.25177	9.2191	-17.665	0.42308	0.46965	0.88538 (max o	2	5383; 6958	0.23078336	0.73597497		7.0
	ZINC20463981	-6.4	370.471	-0.7059	6	2	278.97	0.19844	71.43	4.3573	16920.23333308	0.24245	5.4775	-2.9116	0.59259	0.49429	0.97149 (max o	9	2523; 7850; 5921; 62	0.6272117	0.5713695		6.0
	ZINC35458441	-6.4	418.485	-2.7648	9	3	299.04	0.40362	164.89	-4.0506	16920.23333308	0.23379	7.5364	-11.826	0.53571	0.54332	0.98866 (max o	3	7264; 7438; 7801	-0.5301125	0.8335643		4.0
	ZINC68606245	-6.4	354.561	-6.2724	6	4	312.6	0.21571	85.05	-0.87649	16920.23333308	0.26184	11.044	-23.955	0.6	0.62726	0.98897 (max o	3	7902; 7808; 7860	-0.0682533	-0.7343963		4.0
	ZINC68606236	-6.4	354.561	-6.2724	6	4	312.6	0.21571	85.05	-0.87649	16920.23333308	0.26184	11.044	-23.955	0.6	0.63015	0.98897 (max o	3	7902; 7861; 7808	-0.0711462	-0.7546614		4.0
	ZINC20463981	-6.4	371.479	-2.0722	6	3	293.01	0.23685	72.63	-0.27172	16920.23333308	0.24245	6.8438	-8.547	0.59259	0.49986	0.97149 (max o	5	805; 7849; 5920; 739	0.40259367	0.44279635		3.0
	ZINC01323255	-6.4	390.028	-1.1205	6	0	176.01	0.41185	102.96	-2.6653	16920.23333308	0.38506	5.8921	-2.9099	0.41176	0.19985	0.86653 (max o	2	7964; 7813	-0.4421669	0.76412165		3.0
	ZINC04654665	-6.4	397.634	-1.1611	8	5	246.2	0.3879	128.84	-6.7168	16920.23333308	0.28461	5.9327	-4.0796	0.47826	0.50642		0		0.72634256	-0.6842845		2.0
	ZINC01323254	-6.4	390.028	-1.1205	6	0	176.01	0.41185	102.96	-2.6653	16920.23333308	0.38506	5.8921	-2.9099	0.41176	0.19985	0.86653 (max o	2	7963; 7814	-0.4593431	0.7735324		2.0
	ZINC14766370	-6.4	406.407	1.1941	10	0	293.8	0.45163	156.8	1.4965	16920.23333308	0.24245	3.5775	4.9252	0.40741	0.45775	0.9578 (max of	4	7955; 7949; 7503; 76	0.25360218	0.37308604		2.0
	ZINC05828137	-6.4	377.492	3.6341	7	2	255.21	0.31782	103.23	-0.069206	16920.23333308	0.26184	1.1375	13.879	0.48	0.45965		0		-0.7299290	-0.6832033		1.0
	ZINC19312955	-6.4	352.429	1.2772	7	0	257.2	0.25747	76.15	-6.2795	16920.23333308	0.26184	3.4944	4.8777	0.44	0.44383		0		0.9609275	0.14878711		1.0
	ZINC20464000	-6.4	372.467	-3.0731	7	3	291.77	0.27546	85.52	-0.27172	16920.23333308	0.24245	7.8447	-12.675	0.59259	0.49986	0.97149 (max o	5	805; 5920; 7394; 762	0.41023615	0.46321645		1.0
	ZINC20464000	-6.4	371.459	-1.7068	7	2	277.73	0.23883	84.32	4.3573	16920.23333308	0.24245	6.4784	-7.0399	0.59259	0.49429	0.97149 (max o	9	2523; 5921; 6217; 62	0.6635728	0.58464557		1.0
	ZINC14613441	-6.4	425.462	-9.2176	12	4	304.5	0.64007	284.47	0.11401	16920.23333308	0.24245	13.989	-38.019	0.55556	0.7676	0.97407 (max o	3	7839; 7507; 7824	0.28505486	-0.9161334		0.0
	ZINC16125229	-6.4	425.462	-9.2176	12	4	304.5	0.64007	284.47	0.11401	16920.23333308	0.24245	13.989	-38.019	0.55556	0.75963	0.93996 (max o	4	7915; 7507; 7767; 78	0.31766888	-0.9209915		0.0
	ZINC19419017	-6.4	390.324	-17.994	13	2	322.72	0.54329	212.77	-8.6009	16920.23333308	0.24245	22.766	-74.218	0.48148	0.86704	0.8541	1	7757	0.4303869	-0.8280388		0.0
	ZINC04534317	-6.4	362.426	-5.2294	9	3	281.28	0.48212	191.4	-1.8111	16920.23333308	0.27275	10.001	-19.173	0.58333	0.71011	0.96343 (max o	5	7915; 7876; 7462; 77	0.29730594	-0.9450261		0.0
	ZINC03870223	-6.4	378.381	-7.8293	11	4	276.6	0.58478	228.7	-2.1657	16920.23333308	0.26184	12.601	-29.901	0.52	0.6804	0.98671 (max o	4	7915; 7869; 7543; 77	0.3063534	-0.9097131		0.0
	ZINC04534316	-6.4	362.426	-5.2294	9	3	281.28	0.48212	191.4	-1.8111	16920.23333308	0.27275	10.001	-19.173	0.58333	0.71731	0.96343 (max o	6	7915; 7876; 7836; 78	0.28668466	-0.9409691		0.0
	ZINC35455715	-6.4	436.456	-2.6868	12	6	332.96	0.44294	191.72	-1.2191	16920.23333308	0.2182	7.4584	-12.313	0.6	0.72087	0.97757 (max o	2	7898; 7852	-0.4693161	0.84669894		0.0
	ZINC35455710	-6.4	436.456	-2.6868	12	6	332.96	0.44294	191.72	-1.2191	16920.23333308	0.2182	7.4584	-12.313	0.6	0.71416	0.96092 (max o	2	7898; 7853	-0.4673211	0.83009773		0.0
	ZINC19419017	-6.4	390.324	-16.325	13	2	307.5	0.52068	208.74	-14.253	16920.23333308	0.24245	21.096	-67.333	0.48148	0.85685	0.88831	1	7892	0.24748851	0.880657		0.0
	ZINC04098976	-6.4	358.596	1.9278	7	7	288.48	0.20417	143.89	-5.0195	16920.23333308	0.26184	2.8438	7.3625	0.92	0.60698		0		-0.5581189	-0.7931928		0.0
	ZINC20112509	-6.4	434.274	-11.254	13	4	291.14	0.58776	245.86	-34.379	16920.23333308	0.24245	16.026	-46.418	0.37037	0.77654		0		0.8912602	-0.2960485		0.0
	ZINC68604510	-6.4	364.444	-0.5206	8	2	305.38	0.30133	101.38	-0.75748	16920.23333308	0.25177	5.2922	-2.0677	0.61538	0.42785		0		0.1819348	0.9487162		0.0
	ZINC31165081	-6.3	350.278	-4.1042	9	1	263.19	0.43246	153.09	-6.0092	20089.19000383	0.25775	8.8012	-15.923	0.6	0.55283	0.96832 (max o	7	5212; 5978; 5979; 68	0.83083844	0.05683006		134.0
	ZINC03843466	-6.3	353.009	-0.9108	4	2	204.77	0.28329	64.8	-3.5635	20089.19000383	0.40274	5.6078	-2.2615	0.5625	0.52782	0.82414	1	7817	0.35454994	-0.8115912		134.0
	ZINC13541740	-6.3	415.052	-19.03	15	4	239.36	0.85587	304.55	-31.825	20089.19000383	0.26849	23.727	-70.878	0.41667	0.55758	0.97246 (max o	25	7929; 7885; 5092; 50	0.31805974	0.7740962		127.0
	ZINC13541740	-6.3	414.044	-20.278	15	3	240.54	0.85657	307.38	-20.593	20089.19000383	0.26849	24.975	-75.524	0.41667	0.58345	0.94718 (max o	10	7928; 5091; 6596; 72	0.41859543	0.8510969		127.0
	ZINC13541740	-6.3	415.052	-19.03	15	4	239.36	0.85587	304.55	-31.825	20089.19000383	0.26849	23.727	-70.878	0.41667	0.55758	0.97246 (max o	27	7929; 7885; 5092; 50	0.33755022	0.77454925		127.0
	ZINC13541740	-6.3	415.052	-19.03	15	4	239.36	0.85587	304.55	-31.825	20089.19000383	0.26849	23.727	-70.878	0.41667	0.54974	0.96029 (max o	20	7930; 5093; 5094; 65	0.3881703	0.7588126		127.0

Structure	Molecule Name	affinity	Total Molweight	cLogP	H-Acceptors	H-Donors	Total Surface Area	Relative PSA	Polar Surface Area	Druglikeness	Kd (nM) @ 20°C	LE from K...	LLE from Kd ...	LELP from Kd (n...	Shape Index	Molecular...	Neighbor Si...	Neig...	Neighbor	Neighbor ...	Neighbor ...	Ring Systems	frequency
	ZINC14781592	-6.3	410.461	1.5974	8	4	312.4	0.31524	133.52	-11.395	20089.19000383	0.2222	3.0996	7.189	0.44828	0.60755	0.96931 (max o	3	7509; 7612; 7717	0.44687277	-0.4762902		127.0
	ZINC31159585	-6.3	350.406	-0.6232	8	6	256.52	0.38438	139.84	-9.7913	20089.19000383	0.26849	5.3202	-2.3211	0.5	0.57812	0.99271 (max o	3	7414; 7545; 7546	-0.6420532	-0.3277585		120.0
	ZINC04204382	-6.3	389.356	-0.672	11	1	287.77	0.44014	143.53	-0.087423	20089.19000383	0.23866	5.369	-2.8157	0.40741	0.44545	0.95201 (max o	5	7486; 7487; 7597; 77	0.29132295	0.43535253		120.0
	ZINC04353234	-6.3	390.340	-0.1978	11	0	286.31	0.43729	140.73	-0.85962	20089.19000383	0.23866	4.8948	-0.8288	0.40741	0.43367	0.94905 (max o	3	7693; 7694; 7823	0.20290893	0.3939808		120.0
	ZINC12406313	-6.3	361.346	-0.7287	10	1	270.02	0.42078	126.46	-1.6283	20089.19000383	0.25775	5.4257	-2.8271	0.44	0.46677	0.95631 (max o	7	7984; 7954; 7952; 79	0.20824504	0.4901745		120.0
	ZINC04073235	-6.3	388.368	0.4207	10	0	294.6	0.38072	123.66	-6.1796	20089.19000383	0.23866	4.2763	1.7628	0.40741	0.46889	0.94905	1	7906	0.6720779	-0.4181017		120.0
	ZINC13348087	-6.3	383.349	-4.4475	12	8	254.94	0.57347	198.4	-7.3097	20089.19000383	0.24784	9.1445	-17.945	0.42308	0.46124	0.95559 (max o	5	7912; 7911; 6777; 75	0.522565	-0.4646287		19.0
	ZINC16952033	-6.3	353.111	-1.3798	7	3	184.75	0.43069	108.38	2.1634	20089.19000383	0.37905	6.0768	-3.6402	0.58824	0.49161	0.91652 (max o	4	7187; 7481; 7812; 78	-0.4382049	-9.4564864		14.0
	ZINC03871513	-6.3	414.504	-2.2962	8	6	316.2	0.28678	131.88	-0.035	20089.19000383	0.21479	6.9932	-10.69	0.6	0.50541	0.87898	1	7288	0.7489143	0.5653487		6.0
	ZINC00640221	-6.3	384.336	-0.9694	10	0	269.14	0.43933	139.34	-6.1644	20089.19000383	0.23866	5.6664	-4.0619	0.40741	0.58847	0.95201 (max o	2	5863; 7680	-0.2916957	-0.8746148		5.0
	ZINC67913280	-6.3	410.417	0.3539	9	3	294.52	0.37845	147.43	-9.4267	20089.19000383	0.2222	4.3431	1.5927	0.41379	0.5305	0.95908 (max o	5	4395; 5264; 6045; 70	0.42026863	0.32940716		4.0
	ZINC68606243	-6.3	354.561	-6.2724	6	4	312.6	0.21571	85.05	-0.87649	20089.19000383	0.25775	10.969	-24.335	0.6	0.62447	0.98897 (max o	3	7808; 7860; 7861	-0.0503312	-0.7461169		4.0
	ZINC03848633	-6.3	356.282	-1.7156	10	0	242.45	0.48769	139.34	-6.5621	20089.19000383	0.25775	6.4126	-6.656	0.36	0.37691	0.91611 (max o	2	7866; 7867	-0.5928978	0.51851153		3.0
	ZINC03848631	-6.3	356.282	-1.7156	10	0	242.45	0.48769	139.34	-6.5621	20089.19000383	0.25775	6.4126	-6.656	0.36	0.37691	0.93348 (max o	2	7868; 7866	-0.5756888	0.50745595		3.0
	ZINC03848629	-6.3	356.282	-1.7156	10	0	242.45	0.48769	139.34	-6.5621	20089.19000383	0.25775	6.4126	-6.656	0.36	0.37691	0.93348 (max o	2	7868; 7867	-0.5727412	0.5287896		3.0
	ZINC30879254	-6.3	359.485	-1.3832	7	2	302.38	0.25557	72.67	5.014	20089.19000383	0.25775	6.0802	-5.3664	0.56	0.63108	0.90599 (max o	3	7932; 7640; 7733	-0.3543408	0.37812337		2.0
	ZINC30879254	-6.3	358.477	-0.0169	7	1	288.34	0.21932	71.47	5.3955	20089.19000383	0.25775	4.7139	-0.065568	0.56	0.62956	0.90599 (max o	2	7931; 7732	-0.2973893	0.29787803		2.0
	ZINC20464788	-6.3	378.469	-1.0431	8	3	257.8	0.42727	144.95	3.5003	20089.19000383	0.26849	5.7401	-3.8851	0.5	0.53976	0.91204 (max o	2	6573; 6574	0.18521942	-0.9716165		1.0
	ZINC20464788	-6.3	379.477	-3.1528	8	4	271.84	0.45685	146.15	0.66535	20089.19000383	0.26849	7.8498	-11.743	0.5	0.54249		0		-0.546525	-0.81021		1.0
	ZINC35458484	-6.3	443.579	1.3518	8	3	367.26	0.2834	151.6	-21.203	20089.19000383	0.20786	3.3452	6.5033	0.67742	0.63036	0.85787	1	7549	0.74241966	0.5240581		0.0
	ZINC03870222	-6.3	378.381	-7.8293	11	4	276.6	0.58478	228.7	-2.1657	20089.19000383	0.25775	12.526	-30.375	0.52	0.6804	0.98671 (max o	4	7915; 7543; 7767; 78	0.3283009	-0.9058909		0.0
	ZINC13515786	-6.3	401.542	0.9623	7	4	336.36	0.27988	145.53	-22.292	20089.19000383	0.23013	3.7347	4.1815	0.75	0.65123	0.98006 (max o	5	7909; 7549; 7774; 78	0.758501	0.507803		0.0
	ZINC13515428	-6.3	401.542	0.9623	7	4	336.36	0.27988	145.53	-22.292	20089.19000383	0.23013	3.7347	4.1815	0.75	0.65362	0.9678 (max of	5	7909; 7881; 7549; 77	0.757394	0.48741665		0.0
	ZINC13515427	-6.3	401.542	0.9623	7	4	336.36	0.27988	145.53	-22.292	20089.19000383	0.23013	3.7347	4.1815	0.75	0.64937	0.9678 (max of	5	7909; 7881; 7880; 75	0.72357774	0.5190696		0.0
	ZINC04534318	-6.3	362.426	-5.2294	9	3	281.28	0.48212	191.4	-1.8111	20089.19000383	0.26849	9.9264	-19.477	0.58333	0.71844	0.96343 (max o	3	7767; 7824; 7825	0.3286475	-0.9283579		0.0
	ZINC35455708	-6.3	436.456	-2.6868	12	6	332.96	0.44294	191.72	-1.2191	20089.19000383	0.21479	7.3838	-12.509	0.6	0.70722	0.97757 (max o	2	7852; 7853	-0.4870111	0.8334326		0.0
	ZINC22059268	-6.3	401.395	-17.033	12	1	321.48	0.42777	174.68	-8.1276	20089.19000383	0.23013	21.73	-74.014	0.46429	0.63095	0.88831 (max o	2	7844; 7890	0.242241	0.84430414		0.0
	ZINC22059268	-6.3	403.410	-14.551	12	3	334.34	0.44978	173.05	-14.253	20089.19000383	0.23013	19.248	-63.226	0.46429	0.63451	0.9308 (max of	6	7926; 7923; 7922; 77	0.21513577	0.8121547		0.0
	ZINC22059268	-6.3	402.402	-16.627	12	2	335.52	0.45172	175.88	-8.6009	20089.19000383	0.23013	21.324	-72.247	0.46429	0.63916	0.92774 (max o	8	7925; 7923; 7924; 79	0.20202287	0.7981119		0.0
	ZINC22059268	-6.3	402.402	-16.627	12	2	335.52	0.45172	175.88	-8.1276	20089.19000383	0.23013	21.324	-72.247	0.46429	0.63053	0.90045 (max o	4	7925; 7924; 7892; 78	0.23252138	0.8274287		0.0
	ZINC04204370	-6.3	405.355	-1.4702	12	1	303.58	0.47273	174.59	1.71	20089.19000383	0.23013	6.1672	-6.3884	0.39286	0.59491	0.91804 (max o	2	7971; 7872	-0.8333701	-0.1853063		0.0
	ZINC04204368	-6.3	405.331	-3.1484	12	0	302.59	0.471	171.63	1.0021	20089.19000383	0.23013	7.8454	-13.681	0.39286	0.59009	0.91804 (max o	2	7971; 7873	-0.8326593	-0.2064874		0.0
	ZINC68604261	-6.3	373.428	-13.316	10	3	325.16	0.40642	144.54	-8.2995	20089.19000383	0.24784	18.013	-53.727	0.53846	0.58831	0.8991 (max of	2	7926; 7924	0.25358465	0.81035537		0.0
	ZINC13403665	-6.2	366.500	2.2118	6	2	296.36	0.13497	64.86	-13.625	23851.67375831	0.2439	2.4107	9.0683	0.65385	0.62573	0.89169	1	7691	-0.9246468	0.1636098		134.0
	ZINC26750109	-6.2	415.052	-19.03	15	4	239.36	0.85587	304.55	-31.778	23851.67375831	0.26423	23.652	-72.021	0.375	0.55758	0.93686 (max o	26	5092; 5093; 5094; 65	0.34897652	0.80908173		127.0
	ZINC26750109	-6.2	414.044	-20.278	15	3	240.54	0.85657	307.38	-20.56	23851.67375831	0.26423	24.9	-76.743	0.375	0.58345	0.93686 (max o	10	5091; 6596; 7231; 75	0.35252106	0.85783225		127.0
	ZINC26750109	-6.2	415.052	-19.03	15	4	239.36	0.85587	304.55	-31.778	23851.67375831	0.26423	23.652	-72.021	0.375	0.54974	0.92257 (max o	16	5094; 6599; 7234; 75	0.40842268	0.7592026		127.0
	ZINC35271755	-6.2	410.461	1.5974	8	4	312.4	0.31524	133.52	-11.395	23851.67375831	0.21867	3.0251	7.305	0.44828	0.60936	0.96428 (max o	4	7936; 7434; 7558; 77	0.40746525	-0.5448305		127.0
	ZINC35271758	-6.2	410.461	1.5974	8	4	312.4	0.31524	133.52	-11.395	23851.67375831	0.21867	3.0251	7.305	0.44828	0.60936	0.96428 (max o	4	7556; 7558; 7741; 79	0.44438586	-0.5345408		127.0
	ZINC30878926	-6.2	362.465	-1.1669	8	2	297.74	0.27255	89.49	3.4146	23851.67375831	0.25366	5.7894	-4.6003	0.64	0.69836	0.89162 (max o	3	6287; 7732; 7894	-0.2959127	0.3195132		120.0
	ZINC30878926	-6.2	363.473	-2.5332	8	3	311.78	0.30531	90.69	1.6172	23851.67375831	0.25366	7.1557	-9.9866	0.64	0.69987	0.89524 (max o	6	6288; 7235; 7640; 76	-0.3362681	0.4088898		120.0
	ZINC14766254	-6.2	406.407	0.0381	10	0	293.8	0.45163	156.8	1.9111	23851.67375831	0.23487	4.5844	0.16222	0.40741	0.4291	0.95493 (max o	4	7949; 7487; 7783; 78	0.23967299	0.42310125		120.0
	ZINC14515459	-6.2	376.313	-0.6041	11	0	272.55	0.45937	140.73	-2.6337	23851.67375831	0.2439	5.2266	-2.4768	0.38462	0.39612	0.94951 (max o	3	7953; 7783; 7832	0.21663599	0.37745497		120.0

Structure	Molecule Name	affinity	Total Molweight	cLogP	H-Acceptors	H-Donors	Total Surface Area	Relative PSA	Polar Surface Area	Druglikeness	Kd (nM) @ 20°C	LE from K...	LLE from Kd ...	LELP from Kd (n...	Shape Index	Molecular...	Neighbor Si...	Neig...	Neighbor	Neighbor ...	Neighbor ...	Ring Systems	frequency
	ZINC04027062	-6.2	388.368	0.4207	10	0	294.6	0.38072	123.66	-6.1796	23851.67375831	0.23487	4.2018	1.7912	0.40741	0.46889	0.94905	1	7871	0.67707103	-0.4441607		120.0
	ZINC13348095	-6.2	383.349	-4.4475	12	8	254.94	0.57347	198.4	-7.3097	23851.67375831	0.2439	9.07	-18.235	0.42308	0.46124	0.95559 (max o	4	6777; 7830; 7878; 79	0.5277428	-0.4439444		19.0
	ZINC13348091	-6.2	383.349	-4.4475	12	8	254.94	0.57347	198.4	-7.3097	23851.67375831	0.2439	9.07	-18.235	0.42308	0.46124	0.95559 (max o	4	7912; 7003; 7830; 78	0.5068908	-0.4526935		19.0
	ZINC01322846	-6.2	376.044	-1.8403	5	1	160.53	0.35514	80.67	-2.4417	23851.67375831	0.39634	6.4628	-4.6432	0.4375	0.2085	0.86186 (max o	2	7962; 1215	-0.4194896	-0.8340614		9.0
	ZINC00800351	-6.2	358.030	-0.0276	4	0	164.92	0.3723	85.74	0.13091	23851.67375831	0.42277	4.6501	-0.065284	0.46667	0.21812	0.8435	1	7986	-0.535143	0.8079454		9.0
	ZINC13493525	-6.2	357.409	-3.6337	10	5	268.21	0.44573	174.5	3.4874	23851.67375831	0.25366	8.2562	-14.325	0.64	0.642	0.9654 (max of	3	6442; 7542; 7588	0.14429812	-0.7744738		8.0
	ZINC13831255	-6.2	357.450	-2.7913	7	2	272.19	0.40821	162.3	-1.413	23851.67375831	0.26423	7.4138	-10.564	0.66667	0.61517	0.96343 (max o	4	1569; 7142; 7282; 75	-0.1902980	0.4872768		6.0
	ZINC68562268	-6.2	360.359	-1.658	10	0	235.63	0.51012	148.26	1.4331	23851.67375831	0.28825	6.2805	-5.752	0.45455	0.59648	0.9722 (max of	3	7973; 7959; 7937	-0.1597337	0.94454813		4.0
	ZINC68562263	-6.2	360.359	-1.658	10	0	235.63	0.51012	148.26	1.4331	23851.67375831	0.28825	6.2805	-5.752	0.45455	0.62564	0.9722 (max of	3	7973; 7959; 7938	-0.1651281	0.92747724		4.0
	ZINC12489950	-6.2	351.425	-2.0736	8	4	278.44	0.3506	97.15	1.241	23851.67375831	0.25366	6.6961	-8.1747	0.6	0.54394	0.91304 (max o	4	1489; 3326; 3764; 69	0.34184203	0.02559830		2.0
	ZINC03934758	-6.2	360.342	-0.3605	10	2	242.62	0.54229	170.76	4.3952	23851.67375831	0.26423	4.983	-1.3643	0.5	0.46341	0.92257 (max o	3	7946; 7944; 7945	-0.0618436	0.9730426		2.0
	ZINC33384378	-6.2	430.369	-1.344	14	1	322.92	0.50198	185.96	0.43495	23851.67375831	0.21138	5.9665	-6.3581	0.36667	0.51153		0		0.67013454	-0.4878028		1.0
	ZINC04534249	-6.2	438.520	-2.731	10	2	324.36	0.48902	207.52	-53.944	23851.67375831	0.22648	7.3535	-12.058	0.57143	0.77195	0.97288 (max o	2	7148; 7907	0.47202116	-0.6915251		0.0
	ZINC04534248	-6.2	438.520	-2.731	10	2	324.36	0.48902	207.52	-53.944	23851.67375831	0.22648	7.3535	-12.058	0.57143	0.76435	0.97288 (max o	2	7148; 7908	0.48580548	-0.6681369		0.0
	ZINC31166292	-6.2	354.441	-0.6237	6	2	296.78	0.27239	120.72	-4.8261	23851.67375831	0.25366	5.2462	-2.4588	0.68	0.68996	0.97932 (max o	9	7957; 7241; 7415; 74	0.02732721	0.6935187		0.0
	ZINC13673702	-6.2	350.371	-7.0649	10	4	261.61	0.56844	211.63	0.38619	23851.67375831	0.27572	11.687	-25.624	0.56522	0.7114	0.96395 (max o	9	7462; 7507; 7543; 77	0.30767244	-0.9343840		0.0
	ZINC05782527	-6.2	401.542	0.9623	7	4	336.36	0.27988	145.53	-22.292	23851.67375831	0.22648	3.6602	4.2489	0.75	0.65123	0.98006 (max o	5	7549; 7774; 7879; 78	0.7210894	0.49756035		0.0
	ZINC22016976	-6.2	404.462	-13.435	11	4	355.39	0.42595	157.18	-9.6447	23851.67375831	0.22648	18.057	-59.32	0.46429	0.60865	0.99059	1	7793	-0.8468158	0.4506497		0.0
	ZINC22016976	-6.2	403.454	-13.842	11	3	341.35	0.40234	155.98	-9.6447	23851.67375831	0.22648	18.464	-61.116	0.46429	0.61802	0.99059 (max o	5	7794; 7795; 7891; 78	0.18746226	0.83401096		0.0
	ZINC22016976	-6.2	403.454	-13.842	11	3	341.35	0.40234	155.98	-9.2655	23851.67375831	0.22648	18.464	-61.116	0.46429	0.63833	0.99059 (max o	5	7923; 7794; 7795; 78	0.19354762	0.81680465		0.0
	ZINC22942356	-6.2	430.456	-15.718	12	2	363.04	0.41747	175.88	-7.9817	23851.67375831	0.21138	20.34	-74.357	0.46667	0.60879	0.94241 (max o	3	7890; 7891; 7924	0.2240753	0.7929907		0.0
	ZINC22942356	-6.2	431.464	-13.642	12	3	361.86	0.41558	173.05	-13.995	23851.67375831	0.21138	18.264	-64.536	0.46667	0.60458	0.93686 (max o	3	7893; 7901; 7924	0.25633544	0.8288399		0.0
	ZINC22942356	-6.2	430.456	-15.718	12	2	363.04	0.41747	175.88	-8.3532	23851.67375831	0.21138	20.34	-74.357	0.46667	0.60879	0.94241 (max o	5	7926; 7925; 7890; 78	0.2347059	0.80880266		0.0
	ZINC21297783	-6.2	418.425	-13.935	13	4	355.1	0.47787	190.71	-6.8024	23851.67375831	0.21867	18.557	-63.724	0.51724	0.82746		0		-0.4527893	-0.8865285		0.0
	ZINC22942356	-6.2	431.464	-15.311	12	3	377.08	0.43916	177.08	-8.3532	23851.67375831	0.21138	19.934	-72.433	0.46667	0.60549		0		-0.7374156	-0.6721513		0.0
	ZINC15249305	-6.2	376.269	-6.0586	12	0	274.1	0.53557	185.46	0.62318	23851.67375831	0.2439	10.681	-24.84	0.38462	0.57236		0		-0.7793330	0.58129984		0.0
	ZINC19203111	-6.1	431.967	-2.4752	4	1	192.62	0.29114	90.83	-0.77054	28318.80604762	0.41595	7.0231	-5.9508	0.6	0.57821		0		-0.6560365	-0.7388938		134.0
	ZINC14515454	-6.1	376.313	-0.6041	11	0	272.55	0.45937	140.73	-2.6337	28318.80604762	0.23997	5.152	-2.5174	0.38462	0.40116	0.94951 (max o	3	7611; 7694; 7917	0.18380933	0.3960627		120.0
	ZINC04916031	-6.1	406.407	0.0381	10	0	293.8	0.45163	156.8	1.9111	28318.80604762	0.23108	4.5098	0.16488	0.40741	0.43224	0.95493 (max o	5	7992; 7503; 7694; 78	0.25958285	0.4167645		120.0
	ZINC04141138	-6.1	361.346	-0.7287	10	1	270.02	0.42078	126.46	-1.6283	28318.80604762	0.24957	5.2766	-2.9198	0.44	0.46449	0.94678 (max o	6	7984; 7765; 7820; 78	0.22236393	0.4725808		120.0
	ZINC12406315	-6.1	361.346	-0.7287	10	1	270.02	0.42078	126.46	-1.6283	28318.80604762	0.24957	5.2766	-2.9198	0.44	0.46441	0.95318 (max o	5	7982; 7984; 7820; 78	0.23211287	0.4905297		120.0
	ZINC14586682	-6.1	362.330	-0.2545	10	0	268.56	0.41763	123.66	-2.375	28318.80604762	0.24957	4.8024	-1.0198	0.44	0.44696	0.95 (max of 3)	3	7982; 7877; 7948	0.24097458	0.5078335		120.0
	ZINC04802843	-6.1	392.424	1.1618	9	0	289.81	0.41286	139.73	1.2024	28318.80604762	0.23997	3.3861	4.8414	0.42308	0.46663	0.9464	1	7948	0.27792016	0.49253744		120.0
	ZINC04802845	-6.1	392.424	1.1618	9	0	289.81	0.41286	139.73	1.2024	28318.80604762	0.23997	3.3861	4.8414	0.42308	0.46896	0.9464 (max of	2	7954; 7947	0.26819372	0.47375107		120.0
	ZINC04501340	-6.1	364.370	0.4484	9	0	262.16	0.47593	153.23	-3.4141	28318.80604762	0.25997	4.0995	1.7248	0.45833	0.44654	0.94718	1	7990	0.7593716	0.6488804		120.0
	ZINC05224446	-6.1	389.380	1.2663	10	0	291.58	0.48587	158.88	-3.7477	28318.80604762	0.23997	3.2816	5.2769	0.42308	0.46538		0		-0.4840952	-0.8502373		120.0
	ZINC43794417	-6.1	403.407	0.4074	10	0	299.26	0.44513	163.52	-2.8028	28318.80604762	0.23108	4.1405	1.763	0.40741	0.46819		0		0.5119973	-0.7650327		120.0
	ZINC09033321	-6.1	421.422	-0.0721	11	1	312.73	0.48483	167.78	1.315	28318.80604762	0.22283	4.62	-0.32357	0.39286	0.44696		0		-0.8944093	0.28942838		120.0
	ZINC04556804	-6.1	420.259	-10.197	14	7	262.3	0.67587	251.53	-31.289	28318.80604762	0.23108	14.745	-44.127	0.48148	0.52563	0.95201 (max o	4	3946; 5791; 7322; 76	0.04393079	-0.5079298		12.0
	ZINC02097702	-6.1	354.266	-5.9772	10	0	239.68	0.52904	167	-5.683	28318.80604762	0.24957	10.525	-23.95	0.44	0.5901	0.94678	1	7303	0.95428896	-0.0888381		5.0
	ZINC68562261	-6.1	360.359	-1.658	10	0	235.63	0.51012	148.26	1.4331	28318.80604762	0.2836	6.2059	-5.8463	0.45455	0.59404	0.9722 (max of	3	7973; 7937; 7938	-0.1380675	0.9487471		4.0
	ZINC14766372	-6.1	406.407	1.1941	10	0	293.8	0.45163	156.8	1.4965	28318.80604762	0.23108	3.3538	5.1674	0.40741	0.45775	0.9578 (max of	4	7992; 7693; 7694; 78	0.24015324	0.4051927		2.0
	ZINC04655092	-6.1	360.342	-0.3605	10	2	242.62	0.54229	170.76	4.3952	28318.80604762	0.25997	4.9084	-1.3867	0.5	0.46341	0.92257 (max o	3	7946; 7905; 7945	-0.0688244	0.98272324		2.0

Structure	Molecule Name	affinity	Total Molweight	cLogP	H-Acceptors	H-Donors	Total Surface Area	Relative PSA	Polar Surface Area	Druglikeness	Kd (nM) @ 20°C	LE from K...	LLE from Kd ...	LELP from Kd (n...	Shape Index	Molecular...	Neighbor Si...	Neig...	Neighbor	Neighbor ...	Neighbor ...	Ring Systems	frequency
	ZINC04655094	-6.1	360.342	-0.3605	10	2	242.62	0.54229	170.76	4.3952	28318.80604762	0.25997	4.9084	-1.3867	0.5	0.46341	0.92257 (max o	3	7905; 7944; 7945	-0.0834049	0.9741905		2.0
	ZINC04655093	-6.1	360.342	-0.3605	10	2	242.62	0.54229	170.76	4.3952	28318.80604762	0.25997	4.9084	-1.3867	0.5	0.46341	0.92257 (max o	3	7946; 7905; 7944	-0.0469095	0.982299		2.0
	ZINC31166297	-6.1	354.441	-0.6237	6	2	296.78	0.27239	120.72	-4.8261	28318.80604762	0.24957	5.1716	-2.4991	0.68	0.69491	0.97932 (max o	9	6859; 7075; 7416; 74	0.04384832	0.7051709		0.0
	ZINC71617544	-6.1	389.332	-2.1901	11	0	297.05	0.43589	154.56	0.22417	28318.80604762	0.23108	6.738	-9.4776	0.40741	0.59889	0.96064 (max o	2	7994; 7972	0.2872404	-0.8225975		0.0
	ZINC71617544	-6.1	390.340	-0.9351	11	0	296.85	0.432	148.57	-2.2745	28318.80604762	0.23108	5.483	-4.0466	0.40741	0.56399	0.97658 (max o	3	8002; 7993; 7971	-0.7967517	-0.2132577		0.0
	ZINC04556777	-6.1	405.562	-2.4474	10	6	330.74	0.40588	208.89	0.17186	28318.80604762	0.22283	6.9953	-10.983	0.60714	0.6059		0		0.7806267	-0.6204377		0.0
	ZINC04023762	-6.0	403.426	0.3503	10	1	308.54	0.36825	126.46	1.0944	33622.57861185	0.21918	4.1231	1.5983	0.39286	0.49318	0.93686 (max o	2	7981; 7702	0.10369503	0.9329995		120.0
	ZINC01322848	-6.0	376.044	-1.8403	5	1	160.53	0.35514	80.67	-2.4417	33622.57861185	0.38356	6.3137	-4.798	0.4375	0.2085	0.86186 (max o	2	7701; 7904	-0.4508695	-0.8107794		9.0
	ZINC68562267	-6.0	360.359	-1.658	10	0	235.63	0.51012	148.26	1.4331	33622.57861185	0.27895	6.1314	-5.9437	0.45455	0.59823	0.9722 (max of	3	7937; 7938; 7959	-0.1430323	0.93167675		4.0
	ZINC01323253	-6.0	390.028	-1.1205	6	0	176.01	0.41185	102.96	-2.6653	33622.57861185	0.361	5.5939	-3.1039	0.41176	0.19985	0.86653 (max o	2	7814; 7963	-0.419788	0.7958493		3.0
	ZINC01323252	-6.0	390.028	-1.1205	6	0	176.01	0.41185	102.96	-2.6653	33622.57861185	0.361	5.5939	-3.1039	0.41176	0.19985	0.86653 (max o	2	7964; 7813	-0.4387710	0.78355455		2.0
	ZINC68586317	-6.0	356.370	0.4474	8	0	261.16	0.35289	105.2	-2.5005	33622.57861185	0.24548	4.026	1.8226	0.44	0.59804		0		-0.3116738	0.9252659		1.0
	ZINC34525274	-6.0	390.340	-0.9351	11	0	296.85	0.432	148.57	-2.2745	33622.57861185	0.22729	5.4085	-4.1141	0.40741	0.59391	0.97407 (max o	5	8002; 7993; 7872; 78	-0.8136742	-0.2011045		0.0
	ZINC19855121	-6.0	431.083	-25.044	14	3	298.88	0.72377	298.05	-18.246	33622.57861185	0.25571	29.517	-97.941	0.41667	0.8634	0.88866	1	7968	-0.8714619	-0.0940172		0.0
	ZINC19855121	-6.0	430.075	-26.292	14	2	300.06	0.72486	300.88	-14.552	33622.57861185	0.25571	30.765	-102.82	0.41667	0.86802	0.88866	1	7969	-0.8902645	-0.0861193		0.0
	ZINC34525274	-6.0	389.332	-2.1901	11	0	297.05	0.43589	154.56	0.22417	33622.57861185	0.22729	6.6635	-9.6356	0.40741	0.62723	0.97407 (max o	2	7994; 7961	0.2658132	-0.8296941		0.0
	ZINC14416829	-6.0	434.392	-0.2382	12	0	331.08	0.41754	157.8	-0.052322	33622.57861185	0.20456	4.7116	-1.1644	0.4	0.59919	0.97757 (max o	5	8000; 7983; 7978; 79	0.43225956	-0.7536292		0.0
	ZINC04403108	-6.0	434.392	-0.2382	12	0	331.08	0.41754	157.8	-0.052322	33622.57861185	0.20456	4.7116	-1.1644	0.4	0.57923	0.97757 (max o	5	8000; 7983; 7978; 79	0.41506207	-0.7178994		0.0
	ZINC19855121	-6.0	431.083	-23.362	14	3	268.44	0.69245	289.99	-13.57	33622.57861185	0.25571	27.836	-91.364	0.41667	0.83164		0		-0.2829001	-0.923583		0.0
	ZINC04095597	-5.9	430.935	-1.0033	4	1	188.96	0.28292	77.43	-6.9251	39919.68413529	0.40231	5.4021	-2.4938	0.6	0.66227		0		-0.7326677	0.62078756		134.0
	ZINC04692329	-5.9	432.377	-0.153	12	0	315.06	0.43877	157.8	-0.49421	39919.68413529	0.20115	4.5518	-0.76061	0.33333	0.36963		0		0.33865568	0.9309419		127.0
	ZINC05420471	-5.9	403.426	0.5864	10	1	311.3	0.36499	126.46	-3.7623	39919.68413529	0.21552	3.8124	2.7208	0.42857	0.48893	0.94251 (max o	2	7702; 7965	0.10299423	0.950767		120.0
	ZINC12406317	-5.9	361.346	-0.7287	10	1	270.02	0.42078	126.46	-1.6283	39919.68413529	0.24139	5.1275	-3.0188	0.44	0.46213	0.95318 (max o	5	7820; 7821; 7877; 79	0.21868978	0.5066118		120.0
	ZINC05495580	-5.9	362.330	-0.2545	10	0	268.56	0.41763	123.66	-2.375	39919.68413529	0.24139	4.6533	-1.0543	0.44	0.44453	0.95 (max of 2)	2	7952; 7954	0.25499567	0.4913807		120.0
	ZINC00800348	-5.9	358.030	-0.0276	4	0	164.92	0.3723	85.74	0.13091	39919.68413529	0.40231	4.4264	-0.068604	0.46667	0.21812	0.8435	1	7995	0.7808229	0.5331859		4.0
	ZINC08602570	-5.9	434.392	-0.2382	12	0	331.08	0.41754	157.8	-0.052322	39919.68413529	0.20115	4.637	-1.1842	0.4	0.62146	0.96589 (max o	5	8000; 7966; 7967; 79	0.44464907	-0.7352667		0.0
	ZINC04403112	-5.9	434.392	-0.2382	12	0	331.08	0.41754	157.8	-0.052322	39919.68413529	0.20115	4.637	-1.1842	0.4	0.60958	0.97757 (max o	5	8000; 7983; 7966; 79	0.41009855	-0.7529303		0.0
	ZINC04403110	-5.9	434.392	-0.2382	12	0	331.08	0.41754	157.8	-0.052322	39919.68413529	0.20115	4.637	-1.1842	0.4	0.612	0.97757 (max o	5	8000; 7983; 7978; 79	0.4002001	-0.735171		0.0
	ZINC04403119	-5.9	362.330	-0.1554	10	0	278.57	0.41354	131.5	-0.052322	39919.68413529	0.24139	4.5542	-0.64378	0.44	0.60198		0		0.8259509	-0.5595754		0.0
	ZINC67903527	-5.9	358.429	-1.0915	7	1	296.95	0.30571	126.79	-23.322	39919.68413529	0.24139	5.4903	-4.5218	0.68	0.67438		0		0.20115827	0.9710164		0.0
	ZINC03875424	-5.8	432.974	-0.8972	4	2	191.44	0.28677	88	-0.74224	47396.16196183	0.39549	5.2215	-2.2686	0.6	0.57663	0.87063	1	7758	-0.7177637	0.6117953		134.0
	ZINC04916029	-5.8	406.407	0.0381	10	0	293.8	0.45163	156.8	1.9111	47396.16196183	0.21972	4.2862	0.1734	0.40741	0.43	0.95493 (max o	4	7693; 7837; 7949; 79	0.22613533	0.4375345		120.0
	ZINC04498304	-5.8	364.370	0.4484	9	0	262.16	0.47593	153.23	-3.4141	47396.16196183	0.24718	3.8759	1.814	0.45833	0.44399	0.94718	1	7941	0.7675017	0.63895047		120.0
	ZINC00800349	-5.8	358.030	-0.0276	4	0	164.92	0.3723	85.74	0.13091	47396.16196183	0.39549	4.3519	-0.069787	0.46667	0.21812	0.8435	1	7903	-0.5546777	0.7981873		9.0
	ZINC03984374	-5.8	367.248	2.1063	6	1	257.48	0.2178	75.8	-21.055	47396.16196183	0.25793	2.218	8.1662	0.56522	0.47812	0.91811	1	7988	0.32166207	0.887558		2.0
	ZINC03984373	-5.8	367.248	2.1063	6	1	257.48	0.2178	75.8	-21.055	47396.16196183	0.25793	2.218	8.1662	0.56522	0.47812	0.91811	1	7989	0.29560572	0.9044731		2.0
	ZINC71617549	-5.8	389.332	-2.1901	11	0	297.05	0.43589	154.56	0.22417	47396.16196183	0.21972	6.5144	-9.9678	0.40741	0.63008	0.97407 (max o	2	7961; 7972	0.2805269	-0.8433903		0.0
	ZINC71617549	-5.8	390.340	-0.9351	11	0	296.85	0.432	148.57	-2.2745	47396.16196183	0.21972	5.2594	-4.2559	0.40741	0.59692	0.97407 (max o	3	8002; 7960; 7971	-0.7832435	-0.1984005		0.0
	ZINC04899612	-5.8	351.490	-2.7744	7	3	269.23	0.46718	176.57	2.1637	47396.16196183	0.26965	7.0987	-10.289	0.59091	0.64498	0.95532 (max o	2	7998; 7999	-0.8652332	-0.1871054		0.0
	ZINC00800350	-5.7	358.030	-0.0276	4	0	164.92	0.3723	85.74	0.13091	56272.94043683	0.38867	4.2773	-0.071011	0.46667	0.21812	0.8435	1	7975	0.76458585	0.54910547		4.0
	ZINC14416831	-5.7	434.392	-0.2382	12	0	331.08	0.41754	157.8	-0.052322	56272.94043683	0.19434	4.4879	-1.2257	0.4	0.60164	0.97757 (max o	6	7966; 7967; 7977; 79	0.42302445	-0.735719		0.0
	ZINC13541721	-5.7	351.490	-2.7744	7	3	269.23	0.46718	176.57	2.1637	56272.94043683	0.265	7.0241	-10.469	0.59091	0.64536	0.95532 (max o	2	7991; 7998	-0.8798191	-0.2012682		0.0
	ZINC04899465	-5.7	411.610	-2.1708	7	3	314.24	0.45593	201.87	2.9145	56272.94043683	0.2332	6.4205	-9.3086	0.6	0.66263	0.89938 (max o	2	7991; 7999	-0.8837777	-0.1782701		0.0

Structure	Molecule Name	affinity	Total Molweight	cLogP	H-Acceptors	H-Donors	Total Surface Area	Relative PSA	Polar Surface Area	Druglikeness	Kd (nM) @ 20°C	LE from K...	LLE from Kd ...	LELP from Kd (n...	Shape Index	Molecular...	Neighbor Si...	Neig...	Neighbor	Neighbor ...	Neighbor ...	Ring Systems	frequency
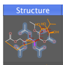	ZINC04204372	-5.7	436.408	-0.5963	12	1	332.18	0.41634	160.96	-2.0769	56272.94043683	0.19434	4.846	-3.0684	0.4	0.6128	0.9707 (max of	2	8004; 8000	0.43700007	-0.71705		0.0
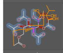	ZINC04705755	-5.7	436.408	-0.5747	12	0	334.33	0.40439	149.96	-7.5633	56272.94043683	0.19434	4.8244	-2.9573	0.36667	0.62024		0		-0.9478196	0.17938706		0.0
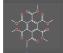	ZINC04802496	-5.6	426.329	1.1376	12	0	313.32	0.44121	157.8	-3.9278	66812.18693949	0.19093	3.0375	5.9583	0.33333	0.58841		0		0.8988533	0.3052025	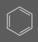	134.0
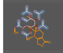	ZINC71617547	-5.6	390.340	-0.9351	11	0	296.85	0.432	148.57	-2.2745	66812.18693949	0.21214	5.1102	-4.4079	0.40741	0.58874	0.97658 (max o	3	7960; 7971; 7993	-0.7996973	-0.1868425		0.0
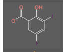	ZINC03860382	-5.5	388.898	-0.4028	3	1	157.45	0.25672	60.36	-0.54917	79325.30784753	0.46879	4.5034	-0.85923	0.5	0.38471		0		-0.1985734	0.8153678	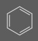	134.0
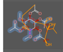	ZINC71325835	-5.4	376.357	-0.2664	10	0	279.56	0.4012	123.66	-0.0794	94181.98615178	0.21243	4.2924	-1.254	0.38462	0.38151		0		0.84133935	-0.2945652	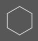	127.0
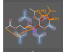	ZINC04204374	-5.4	436.408	-0.5963	12	1	332.18	0.41634	160.96	-2.0769	94181.98615178	0.18411	4.6223	-3.2389	0.4	0.61088	0.9707	1	7996	0.42834938	-0.6987108		0.0
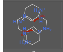	ZINC68604779	-5.4	377.643	-16.21	8	6	348.67	0.28964	124.3	0.2	94181.98615178	0.21243	20.236	-76.306	0.53846	0.61031	0.93686 (max o	3	8005; 8006; 8007	-0.6555732	0.44995886		0.0
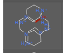	ZINC68604779	-5.4	377.643	-18.014	8	5	333.84	0.25809	124.72	1.3525	94181.98615178	0.21243	22.04	-84.8	0.53846	0.6075	0.93686 (max o	3	8008; 8005; 8006	-0.6723976	0.43529472		0.0
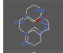	ZINC68604779	-5.4	376.635	-14.844	8	5	334.63	0.25984	123.1	1.0797	94181.98615178	0.21243	18.87	-69.874	0.53846	0.61835	0.93686 (max o	2	8008; 8007	-0.6664040	0.41206464		0.0
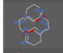	ZINC68604779	-5.4	378.651	-19.381	8	6	347.88	0.28803	125.92	0.46387	94181.98615178	0.21243	23.407	-91.232	0.53846	0.59932	0.93686 (max o	2	8008; 8007	-0.6753090	0.45623332		0.0
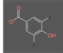	ZINC04099416	-5.3	388.898	-0.4028	3	1	157.45	0.25672	60.36	-0.54917	111821.1420312	0.45175	4.3543	-0.89165	0.58333	0.32097		0		-0.6674354	-0.7434515	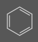	134.0
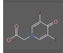	ZINC03830941	-5.2	403.913	-1.5866	4	0	169.07	0.25971	60.44	-7.4221	132764.0088278	0.40913	5.4635	-3.878	0.61538	0.53685		0		-0.4836971	-0.6817634	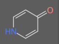	1.0
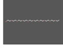	ZINC61389413	-4.9	442.543	-0.782	10	1	383.55	0.2688	103.3	-21.677	222202.3221785	0.16706	4.4353	-4.6809	1	0.72558		0		-0.8428797	-0.5240598		0.0
